# The landscape of human transposable element insertions in Chinese population

**DOI:** 10.1038/s41421-026-00886-5

**Published:** 2026-04-21

**Authors:** Jiali Wang, Chong Chu, Shengqing Wan, Jun Xiang, Zheng Gong, Shengnan Liu, Cheng Wang, Jiaojiao Pang, Feng Xu, Chang Pan, Juying Qian, Hongbing Shen, Guang Ning, Yanan Cao, Yuguo Chen

**Affiliations:** 1 Department of Emergency and Chest Pain Center, Shandong Provincial Clinical Research Center for Emergency and Critical Care Medicine, Qilu Hospital of Shandong University, Jinan, Shandong, China; 2 Key Laboratory of Emergency and Critical Care Medicine of Shandong Province, Key Laboratory of Cardiovascular Remodeling and Function Research, Chinese Ministry of Education and Chinese Ministry of Public Health, Qilu Hospital of Shandong University, Jinan, Shandong, China; 3 Ruijin Yangtze River Delta Health Institute, Wuxi Branch of Ruijin Hospital, Ruijin Hospital, Shanghai Jiao Tong University School of Medicine, Shanghai, China; 4 Department of Epidemiology, Center for Global Health, School of Public Health, Nanjing Medical University, Nanjing, Jiangsu, China; 5 Department of Cardiology, Zhongshan Hospital, Shanghai Institute of Cardiovascular Diseases, National Clinical Research Center for Interventional Medicine, Fudan University, Shanghai, China; 6 Jiangsu Key Lab of Cancer Biomarkers, Prevention and Treatment, Collaborative Innovation Center for Cancer Medicine, Nanjing Medical University, Nanjing, Jiangsu, China; 7 Department of Endocrine and Metabolic Diseases, Shanghai Institute of Endocrine and Metabolic Diseases, Ruijin Hospital, Shanghai Jiao Tong University School of Medicine, Shanghai, China; 8 National Research Center for Translational Medicine, National Key Scientific Infrastructure for Translational Medicine, Shanghai Jiao Tong University, Shanghai, China

**Keywords:** Molecular biology, DNA damage and repair

Dear Editor,

Transposable elements (TEs), taking ~50% of the human genome^1^, are one of the major drivers of genomic evolution and diversity^2^. Among these, long interspersed nuclear element 1 (LINE-1)^3^, Alu^4^, and SINE-VNTR-Alu (SVA)^5,6^ are the known active retrotransposons in the human genome. These three types of TEs (also referred to mobile elements) replicate through ribonucleic acid (RNA) intermediates by a “copy and paste” mechanism mediated by the LINE-1-encoded ORF2p protein. Newly retrotransposed copies, referred to as TE insertions, form polymorphic variations when compared to the human reference genome. Both germline and somatic TE insertions have been shown to play important regulation roles in evolution and diseases^7^. More than 100 TE insertions have been causally linked to Mendelian disorders and hereditary cancers^7^. Although large cohorts of whole genome sequencing (WGS) data in recent years have enabled genome-wide analysis of mutations^2,8,9^, including our studies from the China Metabolic Analytics Project (ChinaMAP) cohort, which is one of the largest cohorts of Chinese population^10^, limitations on TE insertions remain^11^. Here, we applied the xTea^12^ method to 10,013 WGS samples from diverse subpopulations in the ChinaMAP consortium to comprehensively characterize germline polymorphic TE insertions in the Chinese population.

We ran the xTea germline module on 10,013 samples from the ChinaMAP consortium which composed with diverse subpopulations, including 747 South Han, 612 Northwest Han, 922 Lingnan Han, 1098 North Han, 3168 East Han, 1040 Central Han, 953 Southeast Han, 202 Manchu, 216 Hui, 220 Mongolian, 214 Zhuang, 217 Miao, 206 Tibetan, and 198 Yi samples (Supplementary Fig. S1a). In total, we identified 51,045 Alu, 12,929 LINE-1, and 5118 SVA insertions from the ChinaMAP consortium (Fig. 1a and Supplementary Table S1). The number of TE insertions per sample varied by subpopulation, with more TE insertions identified from Tibetan and less in Yi and Miao populations (1008 vs 893, 912 for Alu, 149 vs 119, 119 for LINE-1, and 59 vs 46, 45 for SVA) (Fig. 1b and Supplementary Fig. S1b). When comparing the 69,092 TE insertions identified in this study to the 76,722 TE insertions cataloged in Genome Aggregation Database-Structural Variants (gnomAD-SV) (v2), we observed a surprisingly small overlap, with only 9,091 shared insertions (13% of those identified in this study) (Fig. 1c). Our examination revealed that the shared insertions showed higher population allele frequencies (AF) (for the population in which they occur) compared to those unique to either study. Some of the shared TE insertions showed higher population AF in our study (Supplementary Figs. S1c and S2a), indicating more commonly shared in the Chinese population (Supplementary Fig. S2b).

**Fig. 1:**
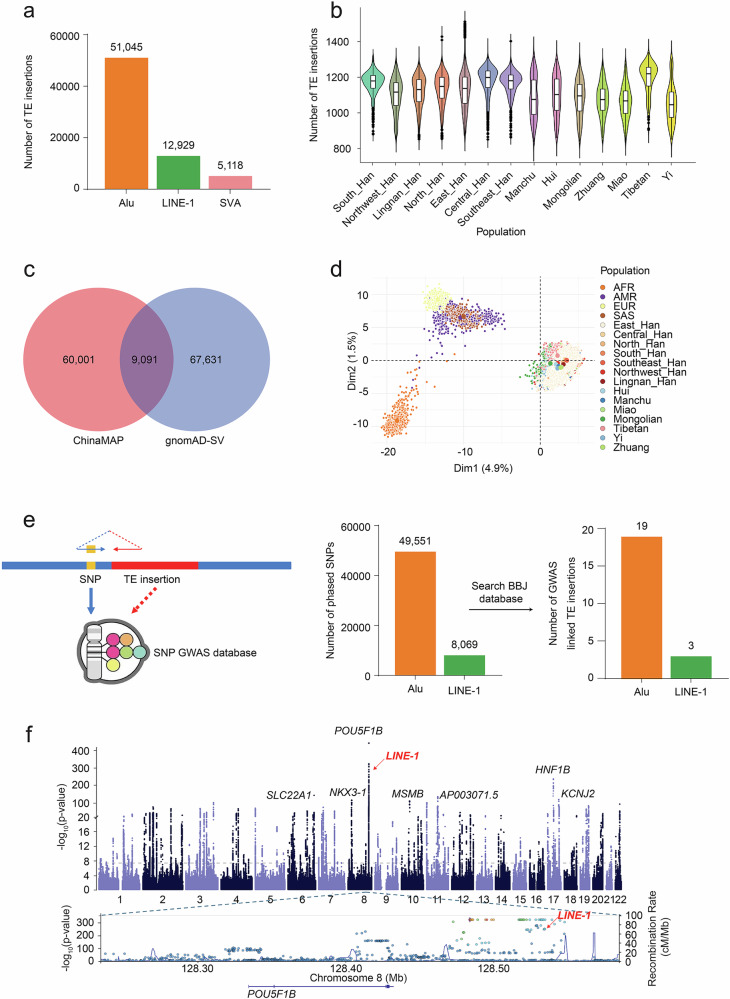
Landscape of TE insertions in the Chinese population. **a** Total number of identified TE insertions from the ChinaMAP cohort: 51,045 Alu, 12,929 LINE-1, and 5118 SVA insertions. **b** Number of TE insertions per sample. Tibetan samples showed the highest number of insertions, while Yi and Miao samples had fewer. **c** Comparison of the 69,092 polymorphic TE insertions identified in this study with the 76,722 insertions in the gnomAD-SV database. Only 9091 insertions were shared between the two datasets. **d** PCA analysis demonstrating the Chinese population specificity of TE insertions compared to other superpopulations: AFR, AMR, EUR, and SAS. **e** Schematic showing how TE insertions are locally phased with nearby SNPs using paired-end reads. A total of 49,551 and 8069 SNPs were locally phased with Alu and LINE-1 TE insertions, respectively, of which 59 and 34 SNPs were identified as genome-wide significant. **f** A LINE-1 insertion is locally phased with SNP rs4645527, which is strongly associated with prostate cancer.

The large size of the ChinaMAP cohort with diverse subpopulations provides the opportunity to characterize the specific TE insertions within the Chinese populations. First, to identify those Chinese specific TE insertions, we aggregated 2137 non-East Asia samples from the high-depth 1000 Genomes Project (1KGP), which include 737 Africa (AFR), 411 America (AMR), 493 South Asia (SAS), and 496 Europe (EUR) samples. Then, similarly, we ran xTea on these 2137 samples to identify TE insertions. In total, we identified 45,224 Alu, 7129 LINE-1, and 4131 SVA insertions (Supplementary Table S2). Then, we compared the TE insertions identified from ChinaMAP and the non-Eastern 1KG and found that 44,961 Alu (88%; out of 51,045), 11,943 (92%; out of 12,929) LINE-1, and 4585 SVA (90%; out of 5118) insertions are ChinaMAP-specific. When we compare the patterns of all the non-rare insertions (AF > 0.01) across populations in the two cohorts using principal component analysis (Fig. 1d), it shows distinct clusters of Africa, European, and South Asian samples, and a separate Chinese cluster with continuum among sub-populations. These population-specific clusters indicate ongoing TE insertion mobilization during population diversification.

With the availability of large-scale sequencing data, genome-wide association studies (GWAS) have identified thousands of small mutations, primarily single nucleotide polymorphisms (SNPs), that are associated with various phenotypes. Some TE insertions can be locally phased with nearby SNPs using short paired-end reads, and if these SNPs have been linked to a phenotype, the associated TE insertions may also be identified as phenotype-associated. Building on this concept, we developed a novel local phasing module (Fig. 1e) to phase each identified TE insertion with its nearby SNPs. From the identified 12,929 LINE-1 and 51,045 Alu insertions, we identified 8069 and 49,551 SNPs that can be locally phased with these TE insertions, respectively. We then searched these identified SNPs against the BioBank Japan (BBJ) database to identify the phenotype associated SNPs. The BBJ database was conducted from a GWAS with 212,453 Japanese individuals across 42 diseases. Here, we used *P*-value 9.58e-9 as a genome-wide significance and identified 19 and 3 significantly GWAS SNPs locally phased with Alu and LINE-1 insertions, respectively (Fig. 1e and Supplementary Tables S3 and S4). One notable example is a LINE-1 insertion that is found to be locally phased with SNP rs4645527 (Fig. 1f), which has been identified to be highly associated with prostate cancer.

Somatic LINE-1 insertions have been identified in several different types of cancers, especially in epithelial tumors, which contribute to the second largest somatic structural alterations in pan-cancer^13^. From 98 non-small cell lung cancer samples (with paired tumor-normal samples), we identified 4731 somatic LINE-1 insertions (Supplementary Table S5). More than half (55%; 54/98) of the samples have > 5 somatic LINE-1 insertions identified, out of which 38% (37/98) of the samples have > 15 somatic insertions, with some samples even having ~400 somatic LINE-1 insertions (Supplementary Fig. S3a), indicating LINE-1s are actively reverse-transcribed in a large portion of the non-small cell lung cancer (NSCLC) tumors. For a subset of LINE-1 insertions, a segment of DNA adjacent to the source element is also retrotransposed (either a 5′ or 3′ transduction)^14,15^. Here, we also characterized transduction events in both tumor samples and the two cohorts of normal samples (ChinaMAP and 1KGP; Supplementary Tables S6 and S7). From the 98 NSCLC samples, we identified 1400 somatic transduction events, which account for 30% of the 4731 somatic events (Supplementary Fig. S3b, c and Table S8). From the 10,013 ChinaMAP samples, we identified 9066 germline LINE-1 transduction events, which represent 13% of all 69,092 germline events (Supplementary Fig. S3d and Table S6). In total, we identified 2572 source LINE-1 elements, 34 of which are specific to ChinaMAP (Supplementary Fig. S3e). Our results indicate that a large number of both shared and population-specific LINE-1 source elements are actively mobilizing in the Chinese population.

Collectively, we systematically analyzed polymorphic TE insertions in the Chinese population using the ChinaMAP cohort and deep whole-genome sequencing data from the 1KGP. The limited overlap between ChinaMAP and gnomAD-SV highlights the importance of sample size and diversity in building comprehensive TE databases. In addition, differences in the sensitivity of the computational methods used likely also contributed to the small overlap. We developed a method to locally phase TE insertions with nearby SNPs, identifying candidate TEs linked to known GWAS loci, suggesting possible regulatory or causal roles, though the method’s resolution is limited by short-read sequencing. Future long-read sequencing datasets, particularly from large cohorts of the Chinese population, will help overcome these limitations and facilitate the discovery and validation of additional TE insertions associated with complex traits. Another limitation of our local-phasing approach is that the nominated TE insertions are only associated with the phenotype and do not establish causality; additional functional validation is required to confirm any causal relationships. We also uncovered 2572 LINE-1 source elements, including 34 specifically to the Chinese population, with some showing activity in Chinese NSCLC samples — providing direct evidence of germline TE contribution to tumor genome instability. The findings underscore the value of TE insertion datasets for functional genomics and the need for broader datasets, including long-read and RNA-seq data, to fully understand TE-mediated genomic variation and disease relevance.

## Supplementary information

Supplementary
