# Supplementary material for: The landscape of human transposable element insertions in Chinese population
Source: Cell Discov. 2026 Apr 21;12:29. doi: 10.1038/s41421-026-00886-5 (PMC13096231; doi:10.1038/s41421-026-00886-5)
Supplement: Supplementary file 1 — Supplementary [file 41421_2026_886_MOESM1_ESM.pdf]

Supplementary File: The landscape of human transposable element insertions in Chinese population

METHODS

Identification of Polymorphic Germline TE Insertions from the ChinaMAP Consortium and 1KGP WGS Datasets

The China Metabolic Analytics Project (ChinaMAP) cohort is a nationwide study that conducted deep whole-genome sequencing (WGS) on 10,588 individuals from diverse regions and ethnic groups across China. These samples represent 27 provinces and municipalities, encompassing 8 ethnic groups, including the Han majority and 7 minority groups. The sequencing was performed using a domestically produced high-throughput platform, with an average depth of 40X. The resulting database contains 136 million single nucleotide polymorphisms (SNPs) and 10 million insertions or deletions (INDELs), approximately half of which are newly identified and not recorded in widely used international databases such as Single-Nucleotide Polymorphism database (dbSNP)[1], 1000 Genomes[2], Genome Aggregation Database (gnomAD)[3], and Trans-Omics for Precision Medicine (TOPMed)[4]. Informed consent was obtained from all study participants. All the protocols were approved by the Ruijin Hospital Ethics Committee, Shanghai Jiao Tong University School of Medicine.

We first applied the xTea (v0.1.7) germline module to the 10,588 WGS samples from the ChinaMAP cohort. Due to lower data quality in some samples, which contained a high number of partially mapped results, many false positives were observed. Consequently, we excluded samples with more than 1,300 Alu insertions, resulting in a final dataset of 10,013 samples.

To construct the transposable element (TE) insertion catalog, we assigned each sample to one of 14 Chinese sub-populations: South Han, Northwest Han, Lingnan Han, North Han, East Han, Central Han, Southeast Han, Manchu, Hui, Mongolian, Zhuang, Miao, Tibetan, and Yi. The identified TE insertions for each sample were merged into a single VCF file using in-house scripts. We then calculated the allele frequency (AF) of each TE insertion for each sub-population. In addition to classic long interspersed nuclear element 1 (LINE-1) insertions, we also identified transduction events, separating them into 5’ and 3’ transductions, and calculated the total number of transductions for each sub-population. For each LINE-1 source element,

which may have multiple descendants across different populations, we calculated the sub-population AF based on the number of transductions.

In addition to the ChinaMAP cohort, we also ran xTea on the high-depth WGS samples from the 1000 Genomes Project (1KGP). We used the 1KGP samples primarily as a “control” to identify TE insertions specific to the Chinese population. To achieve this, we excluded the East Asian samples from the 1KGP dataset and ran xTea on the remaining 2,137 non-East Asian WGS samples.

Polymorphic TE insertion comparison with existing databases

All the germline polymorphic TE insertions identified from the diverged population in this study are based on the human reference genome hg38. To compare the TE insertions released in the gnomAD-SV (v2.0; labeled as LINE-1, Alu, and SINE-VNTR-Alu (SVA) insertion) database which is based on the human reference gnome hg19, we first mapped the positions of the identified TE insertions from hg38 to hg19 with LiftoverVcf (https://gatk.broadinstitute.org/hc/en-us/articles/360036884431-LiftoverVcf-Picard-), and then compared against gnomAD-SV TE insertions using script in xTea (https://github.com/parklab/xTea_paper/x_cmp.py; with option “--extnd 50”).

Local phasing of TE insertions with nearby SNPs

The key idea behind locally phasing TE insertions is to identify SNPs in the flanking regions that reside on the same haplotype as the identified TE insertion. Using paired-end reads, if one read from the flanking region contains an SNP and its mate read maps to the TE insertion, this read pair (referred to as a discordant pair) links the SNP and the TE insertion, a process known as local phasing. To achieve this, we developed a novel module to identify the relevant flanking region SNPs. Specifically, for each identified TE insertion, the associated discordant pairs and clipped reads have been extracted and saved in file by xTea, with which we developed in house scripts to extract the reads from the flanking regions whose read names are of the same as the discordant reads (indicating paired-end reads). We only kept those uniquely mapped ones. We also captured reads clipped at the TE insertion breakpoints. These curated flanking discordant and clipped reads were then realigned to the flanking regions using Burrows-Wheeler-Alignment (BWA)[5]. Next, we sorted and indexed the aligned BAM file using samtools[6]. Finally, SNP

calling was performed with bcftools[7] (v1.11) using the ‘mpileup’ (-Q 30) and ‘call’ commands. For each sample, the identified SNPs associated with all TE insertions were output to a variant call format (VCF) file.

Candidate genome-wide-association TE insertion identification

The BioBank Japan Project (BBJ) is a large biobank project focusing on the Japanese population, one of the largest non-European biobank projects. The latest research achievement of BBJ includes a large-scale genome-wide association study (GWAS) that identified novel susceptibility loci across different diseases. The study involved 212,453 Japanese individuals with 42 common diseases, with 32,793 samples from a prospective cohort study serving as controls. The GWAS summary statistics and genotype data from BBJ are publicly available.

For each TE insertion identified in the ChinaMAP cohort, we first obtained nearby SNPs that could be locally phased with the insertion (as described in the previous step). We then queried these SNPs in the BBJ database and retained those significantly associated with at least one phenotype ($P < 9.58 \times 10^{-9}$). For each retained SNP–TE pair, we extracted genotypes across all ChinaMAP samples and computed linkage disequilibrium (LD; r^2) using PLINK[8]. TE insertions whose paired SNPs showed $r^2 > 0.5$ were considered putatively associated with the corresponding phenotype(s).

Somatic TE insertion identification from Chinese NSCLC samples

The xTea (v0.1.7) short read module was run on the 98 non-small cell lung cancer (NSCLC) samples for somatic L1 insertion identification. Informed consent was obtained from study participants, and this study was approved by the Nanjing Medical University Nanjing Medical University Ethics Committee. xTea first calculated the average depth and then automatically adjusted the parameters based on the calculated depth. In addition, we set the tumor purity to 0.45 ("--purity 0.45"). When parsing transductions from the resulting calls, we further grouped the identified L1 transductions whose traced source elements are within 1000 bp, and we disregarded transductions whose source fell within sequence that could multimap to L1 sequence.

REFERENCES

1. Sherry S T, Ward M H, Kholodov M. *Nucleic Acids Res*, **29**, 308-11 (2001).

2. Genomes Project C, Auton A, Brooks L D. *Nature*, **526**, 68-74 (2015).

3. Karczewski K J, Francioli L C, Tiao G. *Nature*, **581**, 434-443 (2020).

4. Taliun D, Harris D N, Kessler M D. *Nature*, **590**, 290-299 (2021).

5. Li H, Durbin R. *Bioinformatics*, **25**, 1754-60 (2009).

6. Li H, Handsaker B, Wysoker A. *Bioinformatics*, **25**, 2078-9 (2009).

7. Li H. *Bioinformatics*, **27**, 2987-93 (2011).

8. Chang C C, Chow C C, Tellier L C. *Gigascience*, **4**, 7 (2015).

Supplementary Figures: The landscape of human transposable element insertions in Chinese population

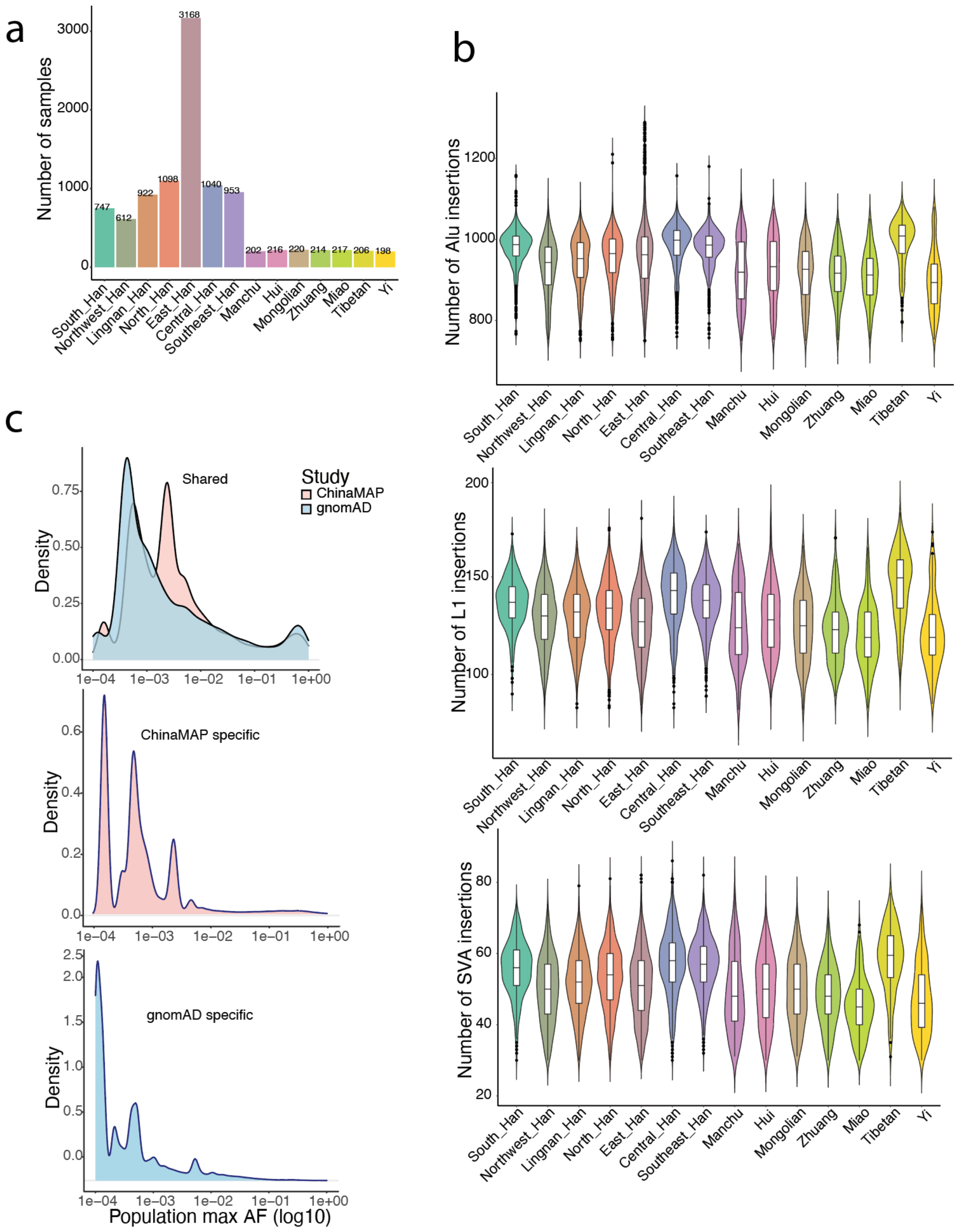

Fig. S1 Polymorphic TE insertions from the diverse Chinese population and comparison with gnomAD-SV database. (a) Distribution of samples across sub-populations in the 10,013 ChinaMAP cohort. **(b)** Number of Alu, LINE-1, and SVA insertions per sample. Tibetan samples showed the highest number of insertions, while Yi and Miao samples had fewer. **(c)** Comparison of the 69,092 polymorphic TE insertions identified in this study with the 76,722 insertions in the gnomAD-SV database. Only 9,091 insertions were shared between the two datasets. The majority of gnomAD-SV-specific insertions had a low population allele frequency (AF) (<0.01), while the ChinaMAP-specific insertions exhibited a higher AF distribution. Overlapping insertions showed similar densities across both datasets, with a small proportion showing higher AF in ChinaMAP.

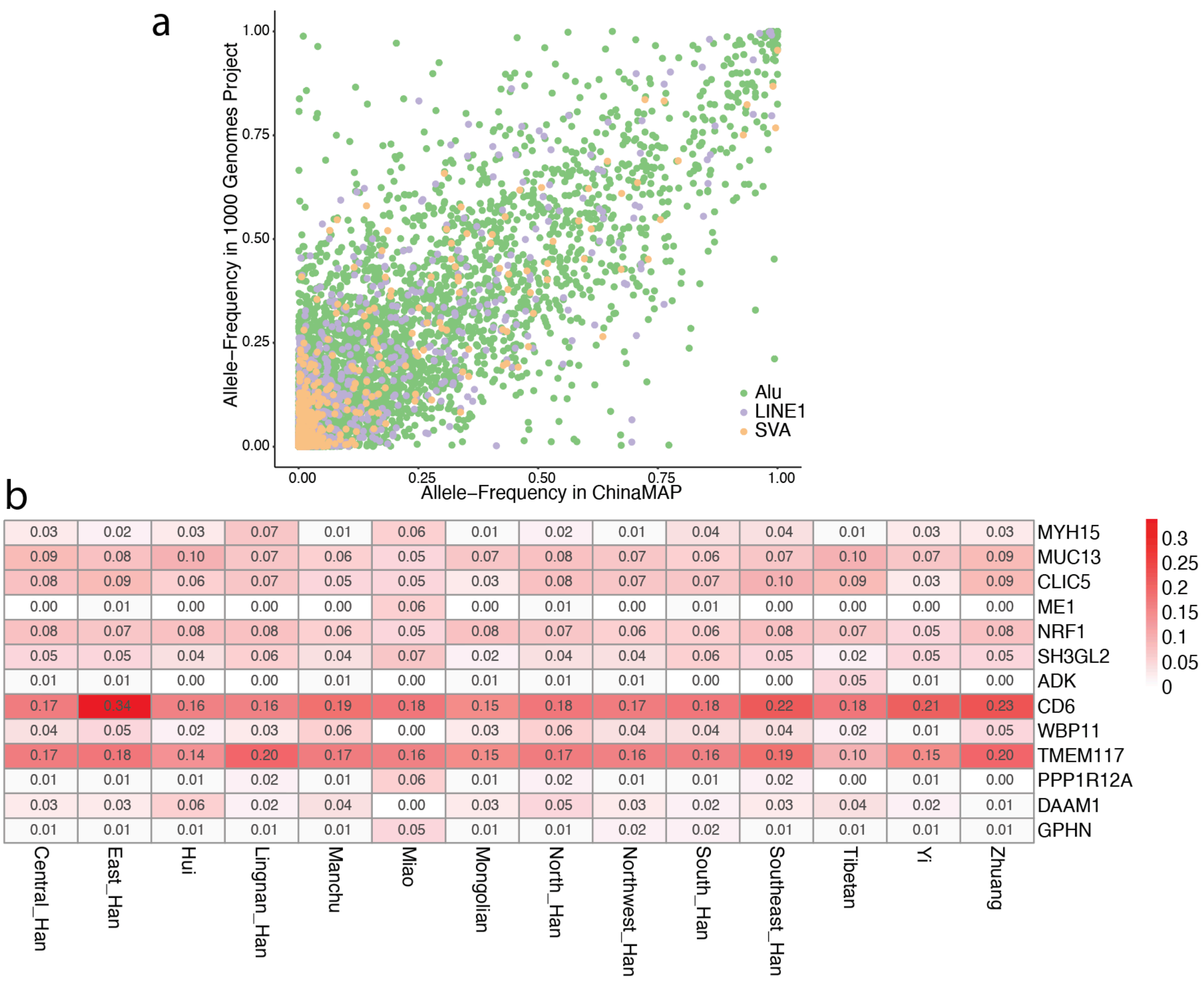

Fig. S2 Chinese population specific TE insertions. (a) Population allele frequencies of shared TE insertions between ChinaMAP and 1000G. Alu, LINE-1, and SVA insertions are shown in green, purple, and orange, respectively. **(b)** Population allele frequencies of intronic Chinese population-specific TE insertions across sub-populations within China.

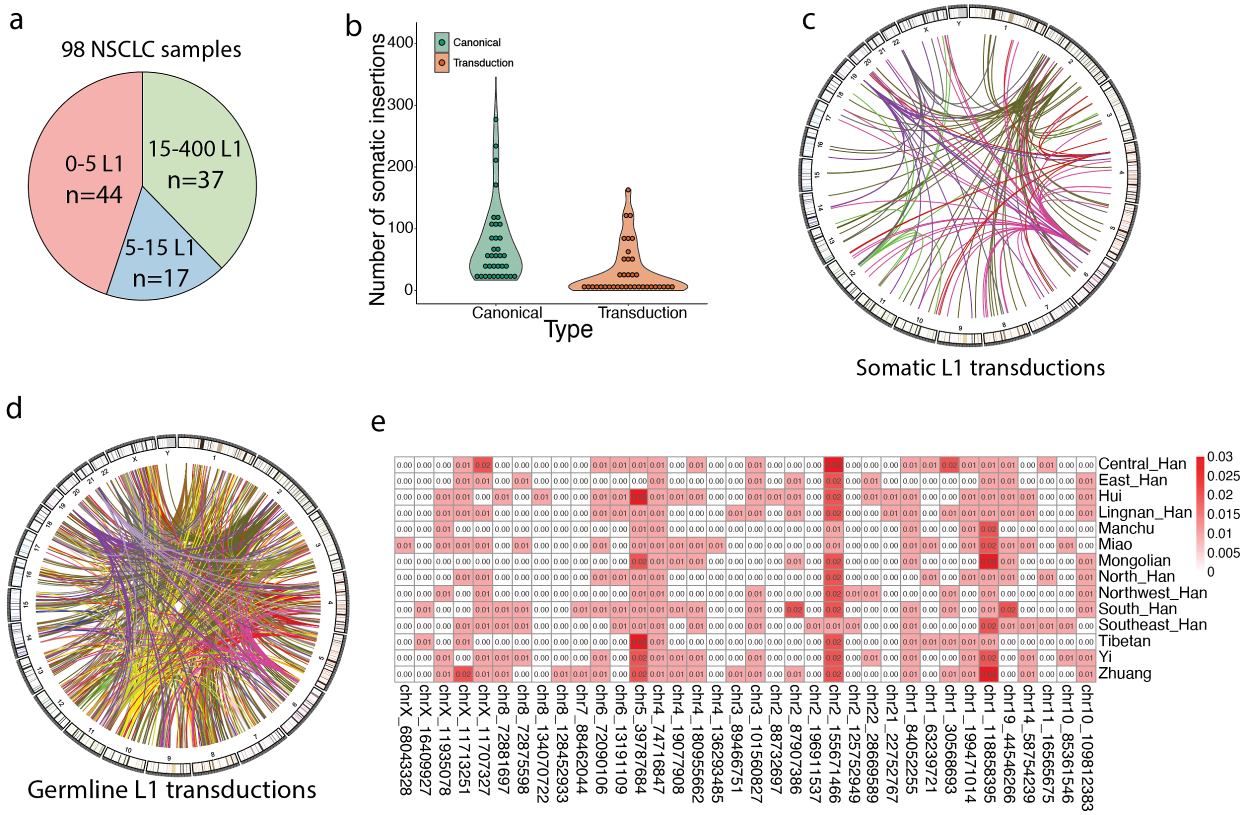

Fig. S3 Somatic LINE-1 insertions and Chinese population specific hot source elements in NSCLC samples. (a) Distribution of somatic LINE-1 insertions across 98 NSCLC samples: 44 samples had 0 to 5 insertions, 17 had 5 to 15, and 37 had more than 15 somatic LINE-1 insertions. **(b)** Number of somatic canonical and transduction LINE-1 insertions per sample. **(c)** Circos plot depicting somatic active LINE-1 source elements with more than 10 somatic offspring insertions. **(d)** Circos plot showing germline active LINE-1 source elements with more than 10 germline offspring insertions identified in ChinaMAP. **(e)** Source elements with sub-population-specific activity are shown, with the number in each cell representing the activity of each source element (column) within each sub-population (row).

TabS1. ChinaMAP TE insertion

CHROM	POS	ID	REF	ALT	QUAL	FILTER	INFO
chr1	2363785	.	A	<INS:ME:LINE1>	.	PASS	SVTYPE=INS:ME:LINE1;END=2363808;SVLEN=1353;AN=20618;AC=51;AF=0.0024735667863032304;N_HET=41;N_HOMALT=5;N_HOMREF=10263;FREQ_HET=0.003977107381899311;FREQ_HOMALT=0.00048501309535357455;FREQ_HOMREF=0.9955378795227471;N_BI_GENOS=10309;South_Han_AN=1496;South_
chr1	4181296	.	G	<INS:ME:LINE1>	.	PASS	SVTYPE=INS:ME:LINE1;END=4181296;SVLEN=0;AN=20618;AC=4;AF=0.00019400523814142983;N_HET=4;N_HOMALT=0;N_HOMREF=10305;FREQ_HET=0.00038801047628285966;FREQ_HOMALT=0.0;FREQ_HOMREF=0.9996119895237171;N_BI_GENOS=10309;North_Han_AN=2272;North_Han_AC=1;North_Han_AF
chr1	4417386	.	A	<INS:ME:LINE1>	.	PASS	SVTYPE=INS:ME:LINE1;END=4417401;SVLEN=455;AN=20618;AC=1;AF=4.850130953535746e-05;N_HET=1;N_HOMALT=0;N_HOMREF=10308;FREQ_HET=9.700261907071492e-05;FREQ_HOMALT=0.0;FREQ_HOMREF=0.9999029973809292;N_BI_GENOS=10309;North_Han_AN=2272;North_Han_AC=1;North_Han_AF
chr1	4881031	.	A	<INS:ME:LINE1>	.	PASS	SVTYPE=INS:ME:LINE1;END=4881052;SVLEN=1067;AN=20618;AC=8;AF=0.00038801047628285966;N_HET=6;N_HOMALT=1;N_HOMREF=10302;FREQ_HET=0.0005820157144242894;FREQ_HOMALT=9.700261907071492e-05;FREQ_HOMREF=0.999320981666505;N_BI_GENOS=10309;Lingnan_Han_AN=1888;Lingna
chr1	4926318	.	A	<INS:ME:LINE1>	.	PASS	SVTYPE=INS:ME:LINE1;END=4926319;SVLEN=3006;AN=20618;AC=6;AF=0.0002910078572121447;N_HET=6;N_HOMALT=0;N_HOMREF=10303;FREQ_HET=0.0005820157144242894;FREQ_HOMALT=0.0;FREQ_HOMREF=0.9994179842855757;N_BI_GENOS=10309;Lingnan_Han_AN=1888;Lingnan_Han_AC=1;Lingnan
chr1	4937720	.	A	<INS:ME:LINE1>	.	PASS	SVTYPE=INS:ME:LINE1;END=4937725;SVLEN=1038;AN=20618;AC=1;AF=4.850130953535746e-05;N_HET=1;N_HOMALT=0;N_HOMREF=10308;FREQ_HET=9.700261907071492e-05;FREQ_HOMALT=0.0;FREQ_HOMREF=0.9999029973809292;N_BI_GENOS=10309;North_Han_AN=2272;North_Han_AC=1;North_Han_A
chr1	5455598	.	A	<INS:ME:LINE1>	.	PASS	SVTYPE=INS:ME:LINE1;END=5455612;SVLEN=1043;AN=20618;AC=1;AF=4.850130953535746e-05;N_HET=1;N_HOMALT=0;N_HOMREF=10308;FREQ_HET=9.700261907071492e-05;FREQ_HOMALT=0.0;FREQ_HOMREF=0.9999029973809292;N_BI_GENOS=10309;Southeast_Han_AN=1908;Southeast_Han_AC=1;Sou
chr1	5807859	.	A	<INS:ME:LINE1>	.	PASS	SVTYPE=INS:ME:LINE1;END=5807873;SVLEN=4324;AN=20618;AC=1;AF=4.850130953535746e-05;N_HET=1;N_HOMALT=0;N_HOMREF=10308;FREQ_HET=9.700261907071492e-05;FREQ_HOMALT=0.0;FREQ_HOMREF=0.9999029973809292;N_BI_GENOS=10309;Southeast_Han_AN=1908;Southeast_Han_AC=1;Sou
chr1	6989644	.	A	<INS:ME:LINE1>	.	PASS	SVTYPE=INS:ME:LINE1;END=6989661;SVLEN=2284;AN=20618;AC=1;AF=4.850130953535746e-05;N_HET=1;N_HOMALT=0;N_HOMREF=10308;FREQ_HET=9.700261907071492e-05;FREQ_HOMALT=0.0;FREQ_HOMREF=0.9999029973809292;N_BI_GENOS=10309;East_Han_AN=6554;East_Han_AC=1;East_Han_AF=0
chr1	7074746	.	T	<INS:ME:LINE1>	.	PASS	SVTYPE=INS:ME:LINE1;END=7074746;SVLEN=105;AN=20618;AC=1;AF=4.850130953535746e-05;N_HET=1;N_HOMALT=0;N_HOMREF=10308;FREQ_HET=9.700261907071492e-05;FREQ_HOMALT=0.0;FREQ_HOMREF=0.9999029973809292;N_BI_GENOS=10309;Southeast_Han_AN=1908;Southeast_Han_AC=1;Sout
chr1	7868166	.	G	<INS:ME:LINE1>	.	PASS	SVTYPE=INS:ME:LINE1;END=7868166;SVLEN=5998;AN=20618;AC=1;AF=4.850130953535746e-05;N_HET=1;N_HOMALT=0;N_HOMREF=10308;FREQ_HET=9.700261907071492e-05;FREQ_HOMALT=0.0;FREQ_HOMREF=0.9999029973809292;N_BI_GENOS=10309;East_Han_AN=6554;East_Han_AC=1;East_Han_AF=0
chr1	8007498	.	T	<INS:ME:LINE1>	.	PASS	SVTYPE=INS:ME:LINE1;END=8007498;SVLEN=0;AN=20618;AC=1;AF=4.850130953535746e-05;N_HET=1;N_HOMALT=0;N_HOMREF=10308;FREQ_HET=9.700261907071492e-05;FREQ_HOMALT=0.0;FREQ_HOMREF=0.9999029973809292;N_BI_GENOS=10309;Central_Han_AN=2128;Central_Han_AC=1;Central_Ha
chr1	10052224	.	C	<INS:ME:LINE1>	.	PASS	SVTYPE=INS:ME:LINE1;END=10052252;SVLEN=1412;AN=20618;AC=1;AF=4.850130953535746e-05;N_HET=1;N_HOMALT=0;N_HOMREF=10308;FREQ_HET=9.700261907071492e-05;FREQ_HOMALT=0.0;FREQ_HOMREF=0.9999029973809292;N_BI_GENOS=10309;Southeast_Han_AN=1908;Southeast_Han_AC=1;So
chr1	10069272	.	A	<INS:ME:LINE1>	.	PASS	SVTYPE=INS:ME:LINE1;END=10069289;SVLEN=635;AN=20618;AC=1;AF=4.850130953535746e-05;N_HET=1;N_HOMALT=0;N_HOMREF=10308;FREQ_HET=9.700261907071492e-05;FREQ_HOMALT=0.0;FREQ_HOMREF=0.9999029973809292;N_BI_GENOS=10309;Yi_AN=422;Yi_AC=1;Yi_AF=0.002369668246445498
chr1	11431743	.	C	<INS:ME:LINE1>	.	PASS	SVTYPE=INS:ME:LINE1;END=11431743;SVLEN=0;AN=20618;AC=2;AF=9.700261907071492e-05;N_HET=0;N_HOMALT=1;N_HOMREF=10308;FREQ_HET=0.0;FREQ_HOMALT=9.700261907071492e-05;FREQ_HOMREF=0.9999029973809292;N_BI_GENOS=10309;East_Han_AN=6554;East_Han_AC=2;East_Han_AF=0.0
chr1	11606092	.	T	<INS:ME:LINE1>	.	PASS	SVTYPE=INS:ME:LINE1;END=11606109;SVLEN=989;AN=20618;AC=1;AF=4.850130953535746e-05;N_HET=1;N_HOMALT=0;N_HOMREF=10308;FREQ_HET=9.700261907071492e-05;FREQ_HOMALT=0.0;FREQ_HOMREF=0.9999029973809292;N_BI_GENOS=10309;North_Han_AN=2272;North_Han_AC=1;North_Han_A
chr1	11770151	.	T	<INS:ME:LINE1>	.	PASS	SVTYPE=INS:ME:LINE1;END=11770151;SVLEN=0;AN=20618;AC=1;AF=4.850130953535746e-05;N_HET=1;N_HOMALT=0;N_HOMREF=10308;FREQ_HET=9.700261907071492e-05;FREQ_HOMALT=0.0;FREQ_HOMREF=0.9999029973809292;N_BI_GENOS=10309;Lingnan_Han_AN=1888;Lingnan_Han_AC=1;Lingnan_H
chr1	12385449	.	T	<INS:ME:LINE1>	.	PASS	SVTYPE=INS:ME:LINE1;END=12385463;SVLEN=1082;AN=20618;AC=1;AF=4.850130953535746e-05;N_HET=1;N_HOMALT=0;N_HOMREF=10308;FREQ_HET=9.700261907071492e-05;FREQ_HOMALT=0.0;FREQ_HOMREF=0.9999029973809292;N_BI_GENOS=10309;South_Han_AN=1496;South_Han_AC=1;South_Han_
chr1	13860608	.	A	<INS:ME:LINE1>	.	PASS	SVTYPE=INS:ME:LINE1;END=13860623;SVLEN=1043;AN=20618;AC=1;AF=4.850130953535746e-05;N_HET=1;N_HOMALT=0;N_HOMREF=10308;FREQ_HET=9.700261907071492e-05;FREQ_HOMALT=0.0;FREQ_HOMREF=0.9999029973809292;N_BI_GENOS=10309;Tibetan_AN=414;Tibetan_AC=1;Tibetan_AF=0.00
chr1	14212697	.	G	<INS:ME:LINE1>	.	PASS	SVTYPE=INS:ME:LINE1;END=14212723;SVLEN=1010;AN=20618;AC=2;AF=9.700261907071492e-05;N_HET=2;N_HOMALT=0;N_HOMREF=10307;FREQ_HET=0.00019400523814142983;FREQ_HOMALT=0.0;FREQ_HOMREF=0.9998059947618586;N_BI_GENOS=10309;East_Han_AN=6554;East_Han_AC=1;East_Han_AF
chr1	14237196	.	G	<INS:ME:LINE1>	.	PASS	SVTYPE=INS:ME:LINE1;END=14237212;SVLEN=251;AN=20618;AC=2;AF=9.700261907071492e-05;N_HET=2;N_HOMALT=0;N_HOMREF=10307;FREQ_HET=0.00019400523814142983;FREQ_HOMALT=0.0;FREQ_HOMREF=0.9998059947618586;N_BI_GENOS=10309;South_Han_AN=1496;South_Han_AC=1;South_Han_
chr1	14253367	.	A	<INS:ME:LINE1>	.	PASS	SVTYPE=INS:ME:LINE1;END=14253383;SVLEN=6084;AN=20618;AC=1;AF=4.850130953535746e-05;N_HET=1;N_HOMALT=0;N_HOMREF=10308;FREQ_HET=9.700261907071492e-05;FREQ_HOMALT=0.0;FREQ_HOMREF=0.9999029973809292;N_BI_GENOS=10309;North_Han_AN=2272;North_Han_AC=1;North_Han_
chr1	14477338	.	T	<INS:ME:LINE1>	.	PASS	SVTYPE=INS:ME:LINE1;END=14477338;SVLEN=2928;AN=20618;AC=2;AF=9.700261907071492e-05;N_HET=2;N_HOMALT=0;N_HOMREF=10307;FREQ_HET=0.00019400523814142983;FREQ_HOMALT=0.0;FREQ_HOMREF=0.9998059947618586;N_BI_GENOS=10309;Central_Han_AN=2128;Central_Han_AC=1;Centr
chr1	14522552	.	T	<INS:ME:LINE1>	.	PASS	SVTYPE=INS:ME:LINE1;END=14522567;SVLEN=96;AN=20618;AC=1;AF=4.850130953535746e-05;N_HET=1;N_HOMALT=0;N_HOMREF=10308;FREQ_HET=9.700261907071492e-05;FREQ_HOMALT=0.0;FREQ_HOMREF=0.9999029973809292;N_BI_GENOS=10309;East_Han_AN=6554;East_Han_AC=1;East_Han_AF=0.
chr1	14528479	.	T	<INS:ME:LINE1>	.	PASS	SVTYPE=INS:ME:LINE1;END=14528490;SVLEN=89;AN=20618;AC=7;AF=0.0003395091667475022;N_HET=7;N_HOMALT=0;N_HOMREF=10302;FREQ_HET=0.0006790183334950044;FREQ_HOMALT=0.0;FREQ_HOMREF=0.999320981666505;N_BI_GENOS=10309;North_Han_AN=2272;North_Han_AC=1;North_Han_AF=
chr1	15396485	.	C	<INS:ME:LINE1>	.	PASS	SVTYPE=INS:ME:LINE1;END=15396498;SVLEN=6007;AN=20618;AC=2;AF=9.700261907071492e-05;N_HET=2;N_HOMALT=0;N_HOMREF=10307;FREQ_HET=0.00019400523814142983;FREQ_HOMALT=0.0;FREQ_HOMREF=0.9998059947618586;N_BI_GENOS=10309;East_Han_AN=6554;East_Han_AC=1;East_Han_AF
chr1	17793079	.	T	<INS:ME:LINE1>	.	PASS	SVTYPE=INS:ME:LINE1;END=17793089;SVLEN=472;AN=20618;AC=6;AF=0.0002910078572121447;N_HET=6;N_HOMALT=0;N_HOMREF=10303;FREQ_HET=0.0005820157144242894;FREQ_HOMALT=0.0;FREQ_HOMREF=0.9994179842855757;N_BI_GENOS=10309;Northwest_Han_AN=1296;Northwest_Han_AC=1;Nor
chr1	19201339	.	A	<INS:ME:LINE1>	.	PASS	SVTYPE=INS:ME:LINE1;END=19201352;SVLEN=6002;AN=20618;AC=1;AF=4.850130953535746e-05;N_HET=1;N_HOMALT=0;N_HOMREF=10308;FREQ_HET=9.700261907071492e-05;FREQ_HOMALT=0.0;FREQ_HOMREF=0.9999029973809292;N_BI_GENOS=10309;Northwest_Han_AN=1296;Northwest_Han_AC=1;No
chr1	19865130	.	G	<INS:ME:LINE1>	.	PASS	SVTYPE=INS:ME:LINE1;END=19865147;SVLEN=1431;AN=20618;AC=1;AF=4.850130953535746e-05;N_HET=1;N_HOMALT=0;N_HOMREF=10308;FREQ_HET=9.700261907071492e-05;FREQ_HOMALT=0.0;FREQ_HOMREF=0.9999029973809292;N_BI_GENOS=10309;Miao_AN=482;Miao_AC=1;Miao_AF=0.00207468879
chr1	20212940	.	C	<INS:ME:LINE1>	.	PASS	SVTYPE=INS:ME:LINE1;END=20212951;SVLEN=676;AN=20618;AC=1;AF=4.850130953535746e-05;N_HET=1;N_HOMALT=0;N_HOMREF=10308;FREQ_HET=9.700261907071492e-05;FREQ_HOMALT=0.0;FREQ_HOMREF=0.9999029973809292;N_BI_GENOS=10309;South_Han_AN=1496;South_Han_AC=1;South_Han_A
chr1	22830720	.	C	<INS:ME:LINE1>	.	PASS	SVTYPE=INS:ME:LINE1;END=22830736;SVLEN=6027;AN=20618;AC=2;AF=9.700261907071492e-05;N_HET=2;N_HOMALT=0;N_HOMREF=10307;FREQ_HET=0.00019400523814142983;FREQ_HOMALT=0.0;FREQ_HOMREF=0.9998059947618586;N_BI_GENOS=10309;Northwest_Han_AN=1296;Northwest_Han_AC=1;N
chr1	24195311	.	A	<INS:ME:LINE1>	.	PASS	SVTYPE=INS:ME:LINE1;END=24195327;SVLEN=256;AN=20618;AC=2;AF=9.700261907071492e-05;N_HET=0;N_HOMALT=1;N_HOMREF=10308;FREQ_HET=0.0;FREQ_HOMALT=9.700261907071492e-05;FREQ_HOMREF=0.9999029973809292;N_BI_GENOS=10309;Manchu_AN=416;Manchu_AC=2;Manchu_AF=0.004807
chr1	24448673	.	A	<INS:ME:LINE1>	.	PASS	SVTYPE=INS:ME:LINE1;END=24448673;SVLEN=2706;AN=20618;AC=1;AF=4.850130953535746e-05;N_HET=1;N_HOMALT=0;N_HOMREF=10308;FREQ_HET=9.700261907071492e-05;FREQ_HOMALT=0.0;FREQ_HOMREF=0.9999029973809292;N_BI_GENOS=10309;Manchu_AN=416;Manchu_AC=1;Manchu_AF=0.00240
chr1	24575214	.	A	<INS:ME:LINE1>	.	PASS	SVTYPE=INS:ME:LINE1;END=24575230;SVLEN=343;AN=20618;AC=2;AF=9.700261907071492e-05;N_HET=2;N_HOMALT=0;N_HOMREF=10307;FREQ_HET=0.00019400523814142983;FREQ_HOMALT=0.0;FREQ_HOMREF=0.9998059947618586;N_BI_GENOS=10309;East_Han_AN=6554;East_Han_AC=2;East_Han_AF=
chr1	25307524	.	A	<INS:ME:LINE1>	.	PASS	SVTYPE=INS:ME:LINE1;END=25307538;SVLEN=5398;AN=20618;AC=3;AF=0.00014550392860607236;N_HET=3;N_HOMALT=0;N_HOMREF=10306;FREQ_HET=0.0002910078572121447;FREQ_HOMALT=0.0;FREQ_HOMREF=0.9997089921427879;N_BI_GENOS=10309;North_Han_AN=2272;North_Han_AC=1;North_Han
chr1	25454489	.	T	<INS:ME:LINE1>	.	PASS	SVTYPE=INS:ME:LINE1;END=25454489;SVLEN=0;AN=20618;AC=57;AF=0.0027645746435153748;N_HET=1;N_HOMALT=28;N_HOMREF=10280;FREQ_HET=9.700261907071492e-05;FREQ_HOMALT=0.0027160733339800175;FREQ_HOMREF=0.9971869240469493;N_BI_GENOS=10309;South_Han_AN=1496;South_Ha
chr1	25677696	.	T	<INS:ME:LINE1>	.	PASS	SVTYPE=INS:ME:LINE1;END=25677705;SVLEN=1605;AN=20618;AC=1;AF=4.850130953535746e-05;N_HET=1;N_HOMALT=0;N_HOMREF=10308;FREQ_HET=9.700261907071492e-05;FREQ_HOMALT=0.0;FREQ_HOMREF=0.9999029973809292;N_BI_GENOS=10309;East_Han_AN=6554;East_Han_AC=1;East_Han_AF=
chr1	26113149	.	A	<INS:ME:LINE1>	.	PASS	SVTYPE=INS:ME:LINE1;END=26113165;SVLEN=318;AN=20618;AC=1;AF=4.850130953535746e-05;N_HET=1;N_HOMALT=0;N_HOMREF=10308;FREQ_HET=9.700261907071492e-05;FREQ_HOMALT=0.0;FREQ_HOMREF=0.9999029973809292;N_BI_GENOS=10309;Tibetan_AN=414;Tibetan_AC=1;Tibetan_AF=0.002
chr1	26206481	.	A	<INS:ME:LINE1>	.	PASS	SVTYPE=INS:ME:LINE1;END=26206481;SVLEN=0;AN=20618;AC=1;AF=4.850130953535746e-05;N_HET=1;N_HOMALT=0;N_HOMREF=10308;FREQ_HET=9.700261907071492e-05;FREQ_HOMALT=0.0;FREQ_HOMREF=0.9999029973809292;N_BI_GENOS=10309;East_Han_AN=6554;East_Han_AC=1;East_Han_AF=0.0
chr1	28353523	.	A	<INS:ME:LINE1>	.	PASS	SVTYPE=INS:ME:LINE1;END=28353523;SVLEN=0;AN=20618;AC=24;AF=0.0011640314288485789;N_HET=18;N_HOMALT=3;N_HOMREF=10288;FREQ_HET=0.0017460471432728683;FREQ_HOMALT=0.0002910078572121447;FREQ_HOMREF=0.997962944999515;N_BI_GENOS=10309;South_Han_AN=1496;South_Han
chr1	30169003	.	A	<INS:ME:LINE1>	.	PASS	SVTYPE=INS:ME:LINE1;END=30169018;SVLEN=1071;AN=20618;AC=6;AF=0.0002910078572121447;N_HET=6;N_HOMALT=0;N_HOMREF=10303;FREQ_HET=0.0005820157144242894;FREQ_HOMALT=0.0;FREQ_HOMREF=0.9994179842855757;N_BI_GENOS=10309;Northwest_Han_AN=1296;Northwest_Han_AC=1;No
chr1	30460770	.	A	<INS:ME:LINE1>	.	PASS	SVTYPE=INS:ME:LINE1;END=30460785;SVLEN=5997;AN=20618;AC=1;AF=4.850130953535746e-05;N_HET=1;N_HOMALT=0;N_HOMREF=10308;FREQ_HET=9.700261907071492e-05;FREQ_HOMALT=0.0;FREQ_HOMREF=0.9999029973809292;N_BI_GENOS=10309;North_Han_AN=2272;North_Han_AC=1;North_Han_
chr1	31538736	.	T	<INS:ME:LINE1>	.	PASS	SVTYPE=INS:ME:LINE1;END=31538742;SVLEN=6007;AN=20618;AC=1;AF=4.850130953535746e-05;N_HET=1;N_HOMALT=0;N_HOMREF=10308;FREQ_HET=9.700261907071492e-05;FREQ_HOMALT=0.0;FREQ_HOMREF=0.9999029973809292;N_BI_GENOS=10309;Manchu_AN=416;Manchu_AC=1;Manchu_AF=0.00240
chr1	33565637	.	A	<INS:ME:LINE1>	.	PASS	SVTYPE=INS:ME:LINE1;END=33565651;SVLEN=951;AN=20618;AC=1;AF=4.850130953535746e-05;N_HET=1;N_HOMALT=0;N_HOMREF=10308;FREQ_HET=9.700261907071492e-05;FREQ_HOMALT=0.0;FREQ_HOMREF=0.9999029973809292;N_BI_GENOS=10309;Hui_AN=444;Hui_AC=1;Hui_AF=0.002252252252252
chr1	33769239	.	A	<INS:ME:LINE1>	.	PASS	SVTYPE=INS:ME:LINE1;END=33769256;SVLEN=1148;AN=20618;AC=1;AF=4.850130953535746e-05;N_HET=1;N_HOMALT=0;N_HOMREF=10308;FREQ_HET=9.700261907071492e-05;FREQ_HOMALT=0.0;FREQ_HOMREF=0.9999029973809292;N_BI_GENOS=10309;Manchu_AN=416;Manchu_AC=1;Manchu_AF=0.00240
chr1	34311192	.	A	<INS:ME:LINE1>	.	PASS	SVTYPE=INS:ME:LINE1;END=34311192;SVLEN=838;AN=20618;AC=1;AF=4.850130953535746e-05;N_HET=1;N_HOMALT=0;N_HOMREF=10308;FREQ_HET=9.700261907071492e-05;FREQ_HOMALT=0.0;FREQ_HOMREF=0.9999029973809292;N_BI_GENOS=10309;East_Han_AN=6554;East_Han_AC=1;East_Han_AF=0
chr1	34352241	.	C	<INS:ME:LINE1>	.	PASS	SVTYPE=INS:ME:LINE1;END=34352253;SVLEN=751;AN=20618;AC=3;AF=0.00014550392860607236;N_HET=3;N_HOMALT=0;N_HOMREF=10306;FREQ_HET=0.0002910078572121447;FREQ_HOMALT=0.0;FREQ_HOMREF=0.9997089921427879;N_BI_GENOS=10309;Lingnan_Han_AN=1888;Lingnan_Han_AC=2;Lingna
chr1	34459066	.	C	<INS:ME:LINE1>	.	PASS	SVTYPE=INS:ME:LINE1;END=34459080;SVLEN=5999;AN=20618;AC=1;AF=4.850130953535746e-05;N_HET=1;N_HOMALT=0;N_HOMREF=10308;FREQ_HET=9.700261907071492e-05;FREQ_HOMALT=0.0;FREQ_HOMREF=0.9999029973809292;N_BI_GENOS=10309;South_Han_AN=1496;South_Han_AC=1;South_Han_
chr1	34539052	.	A	<INS:ME:LINE1>	.	PASS	SVTYPE=INS:ME:LINE1;END=34539060;SVLEN=749;AN=20618;AC=6;AF=0.0002910078572121447;N_HET=6;N_HOMALT=0;N_HOMREF=10303;FREQ_HET=0.0005820157144242894;FREQ_HOMALT=0.0;FREQ_HOMREF=0.9994179842855757;N_BI_GENOS=10309;Lingnan_Han_AN=1888;Lingnan_Han_AC=2;Lingnan
chr1	34541475	.	A	<INS:ME:LINE1>	.	PASS	SVTYPE=INS:ME:LINE1;END=34541475;SVLEN=845;AN=20618;AC=2;AF=9.700261907071492e-05;N_HET=0;N_HOMALT=1;N_HOMREF=10308;FREQ_HET=0.0;FREQ_HOMALT=9.700261907071492e-05;FREQ_HOMREF=0.9999029973809292;N_BI_GENOS=10309;East_Han_AN=6554;East_Han_AC=2;East_Han_AF=0
chr1	34562274	.	A	<INS:ME:LINE1>	.	PASS	SVTYPE=INS:ME:LINE1;END=34562289;SVLEN=847;AN=20618;AC=1;AF=4.850130953535746e-05;N_HET=1;N_HOMALT=0;N_HOMREF=10308;FREQ_HET=9.700261907071492e-05;FREQ_HOMALT=0.0;FREQ_HOMREF=0.9999029973809292;N_BI_GENOS=10309;Yi_AN=422;Yi_AC=1;Yi_AF=0.002369668246445498
chr1	34608586	.	A	<INS:ME:LINE1>	.	PASS	SVTYPE=INS:ME:LINE1;END=34608600;SVLEN=2283;AN=20618;AC=2;AF=9.700261907071492e-05;N_HET=2;N_HOMALT=0;N_HOMREF=10307;FREQ_HET=0.00019400523814142983;FREQ_HOMALT=0.0;FREQ_HOMREF=0.9998059947618586;N_BI_GENOS=10309;North_Han_AN=2272;North_Han_AC=1;North_Han
chr1	35211889	.	G	<INS:ME:LINE1>	.	PASS	SVTYPE=INS:ME:LINE1;END=35211889;SVLEN=0;AN=20618;AC=3;AF=0.00014550392860607236;N_HET=1;N_HOMALT=1;N_HOMREF=10307;FREQ_HET=9.700261907071492e-05;FREQ_HOMALT=9.700261907071492e-05;FREQ_HOMREF=0.9998059947618586;N_BI_GENOS=10309;Lingnan_Han_AN=1888;Lingnan
chr1	35998796	.	C	<INS:ME:LINE1>	.	PASS	SVTYPE=INS:ME:LINE1;END=35998806;SVLEN=873;AN=20618;AC=2;AF=9.700261907071492e-05;N_HET=2;N_HOMALT=0;N_HOMREF=10307;FREQ_HET=0.00019400523814142983;FREQ_HOMALT=0.0;FREQ_HOMREF=0.9998059947618586;N_BI_GENOS=10309;East_Han_AN=6554;East_Han_AC=2;East_Han_AF=
chr1	36312007	.	A	<INS:ME:LINE1>	.	PASS	SVTYPE=INS:ME:LINE1;END=36312023;SVLEN=221;AN=20618;AC=1;AF=4.850130953535746e-05;N_HET=1;N_HOMALT=0;N_HOMREF=10308;FREQ_HET=9.700261907071492e-05;FREQ_HOMALT=0.0;FREQ_HOMREF=0.9999029973809292;N_BI_GENOS=10309;Central_Han_AN=2128;Central_Han_AC=1;Central
chr1	38325787	.	C	<INS:ME:LINE1>	.	PASS	SVTYPE=INS:ME:LINE1;END=38325802;SVLEN=5998;AN=20618;AC=1;AF=4.850130953535746e-05;N_HET=1;N_HOMALT=0;N_HOMREF=10308;FREQ_HET=9.700261907071492e-05;FREQ_HOMALT=0.0;FREQ_HOMREF=0.9999029973809292;N_BI_GENOS=10309;East_Han_AN=6554;East_Han_AC=1;East_Han_AF=
chr1	38371224	.	A	<INS:ME:LINE1>	.	PASS	SVTYPE=INS:ME:LINE1;END=38371240;SVLEN=6000;AN=20618;AC=1;AF=4.850130953535746e-05;N_HET=1;N_HOMALT=0;N_HOMREF=10308;FREQ_HET=9.700261907071492e-05;FREQ_HOMALT=0.0;FREQ_HOMREF=0.9999029973809292;N_BI_GENOS=10309;North_Han_AN=2272;North_Han_AC=1;North_Han_
chr1	38577017	.	G	<INS:ME:LINE1>	.	PASS	SVTYPE=INS:ME:LINE1;END=38577032;SVLEN=6000;AN=20618;AC=2;AF=9.700261907071492e-05;N_HET=2;N_HOMALT=0;N_HOMREF=10307;FREQ_HET=0.00019400523814142983;FREQ_HOMALT=0.0;FREQ_HOMREF=0.9998059947618586;N_BI_GENOS=10309;East_Han_AN=6554;East_Han_AC=2;East_Han_AF
chr1	41128576	.	A	<INS:ME:LINE1>	.	PASS	SVTYPE=INS:ME:LINE1;END=41128588;SVLEN=5997;AN=20618;AC=1;AF=4.850130953535746e-05;N_HET=1;N_HOMALT=0;N_HOMREF=10308;FREQ_HET=9.700261907071492e-05;FREQ_HOMALT=0.0;FREQ_HOMREF=0.9999029973809292;N_BI_GENOS=10309;North_Han_AN=2272;North_Han_AC=1;North_Han_
chr1	41234469	.	T	<INS:ME:LINE1>	.	PASS	SVTYPE=INS:ME:LINE1;END=41234480;SVLEN=1577;AN=20618;AC=2;AF=9.700261907071492e-05;N_HET=0;N_HOMALT=1;N_HOMREF=10308;FREQ_HET=0.0;FREQ_HOMALT=9.700261907071492e-05;FREQ_HOMREF=0.9999029973809292;N_BI_GENOS=10309;South_Han_AN=1496;South_Han_AC=2;South_Han_
chr1	41319828	.	C	<INS:ME:LINE1>	.	PASS	SVTYPE=INS:ME:LINE1;END=41319848;SVLEN=6002;AN=20618;AC=3;AF=0.00014550392860607236;N_HET=3;N_HOMALT=0;N_HOMREF=10306;FREQ_HET=0.0002910078572121447;FREQ_HOMALT=0.0;FREQ_HOMREF=0.9997089921427879;N_BI_GENOS=10309;Central_Han_AN=2128;Central_Han_AC=1;Centr
chr1	42018289	.	A	<INS:ME:LINE1>	.	PASS	SVTYPE=INS:ME:LINE1;END=42018289;SVLEN=1410;AN=20618;AC=3496;AF=0.16956057813560965;N_HET=2238;N_HOMALT=629;N_HOMREF=7442;FREQ_HET=0.21709186148025997;FREQ_HOMALT=0.061014647395479675;FREQ_HOMREF=0.7218934911242604;N_BI_GENOS=10309;South_Han_AN=1496;South
chr1	42294799	.	A	<INS:ME:LINE1>	.	PASS	SVTYPE=INS:ME:LINE1;END=42294815;SVLEN=442;AN=20618;AC=1;AF=4.850130953535746e-05;N_HET=1;N_HOMALT=0;N_HOMREF=10308;FREQ_HET=9.700261907071492e-05;FREQ_HOMALT=0.0;FREQ_HOMREF=0.9999029973809292;N_BI_GENOS=10309;South_Han_AN=1496;South_Han_AC=1;South_Han_A
chr1	42308992	.	T	<INS:ME:LINE1>	.	PASS	SVTYPE=INS:ME:LINE1;END=42309001;SVLEN=6000;AN=20618;AC=35;AF=0.0016975458337375108;N_HET=15;N_HOMALT=10;N_HOMREF=10284;FREQ_HET=0.0014550392860607237;FREQ_HOMALT=0.0009700261907071491;FREQ_HOMREF=0.9975749345232321;N_BI_GENOS=10309;South_Han_AN=1496;Sout
chr1	42542002	.	A	<INS:ME:LINE1>	.	PASS	SVTYPE=INS:ME:LINE1;END=42542018;SVLEN=1384;AN=20618;AC=1;AF=4.850130953535746e-05;N_HET=1;N_HOMALT=0;N_HOMREF=10308;FREQ_HET=9.700261907071492e-05;FREQ_HOMALT=0.0;FREQ_HOMREF=0.9999029973809292;N_BI_GENOS=10309;South_Han_AN=1496;South_Han_AC=1;South_Han_
chr1	42593316	.	A	<INS:ME:LINE1>	.	PASS	SVTYPE=INS:ME:LINE1;END=42593331;SVLEN=6293;AN=20618;AC=3;AF=0.00014550392860607236;N_HET=3;N_HOMALT=0;N_HOMREF=10306;FREQ_HET=0.0002910078572121447;FREQ_HOMALT=0.0;FREQ_HOMREF=0.9997089921427879;N_BI_GENOS=10309;South_Han_AN=1496;South_Han_AC=2;South_Han
chr1	43642140	.	G	<INS:ME:LINE1>	.	PASS	SVTYPE=INS:ME:LINE1;END=43642156;SVLEN=334;AN=20618;AC=1;AF=4.850130953535746e-05;N_HET=1;N_HOMALT=0;N_HOMREF=10308;FREQ_HET=9.700261907071492e-05;FREQ_HOMALT=0.0;FREQ_HOMREF=0.9999029973809292;N_BI_GENOS=10309;Northwest_Han_AN=1296;Northwest_Han_AC=1;Nor
chr1	44270798	.	C	<INS:ME:LINE1>	.	PASS	SVTYPE=INS:ME:LINE1;END=44270801;SVLEN=521;AN=20618;AC=3;AF=0.00014550392860607236;N_HET=3;N_HOMALT=0;N_HOMREF=10306;FREQ_HET=0.0002910078572121447;FREQ_HOMALT=0.0;FREQ_HOMREF=0.9997089921427879;N_BI_GENOS=10309;Northwest_Han_AN=1296;Northwest_Han_AC=1;No
chr1	44742564	.	A	<INS:ME:LINE1>	.	PASS	SVTYPE=INS:ME:LINE1;END=44742582;SVLEN=3571;AN=20618;AC=1;AF=4.850130953535746e-05;N_HET=1;N_HOMALT=0;N_HOMREF=10308;FREQ_HET=9.700261907071492e-05;FREQ_HOMALT=0.0;FREQ_HOMREF=0.9999029973809292;N_BI_GENOS=10309;Mongolian_AN=454;Mongolian_AC=1;Mongolian_A
chr1	44960993	.	A	<INS:ME:LINE1>	.	PASS	SVTYPE=INS:ME:LINE1;END=44961005;SVLEN=2187;AN=20618;AC=1;AF=4.850130953535746e-05;N_HET=1;N_HOMALT=0;N_HOMREF=10308;FREQ_HET=9.700261907071492e-05;FREQ_HOMALT=0.0;FREQ_HOMREF=0.9999029973809292;N_BI_GENOS=10309;South_Han_AN=1496;South_Han_AC=1;South_Han_
chr1	45854677	.	A	<INS:ME:LINE1>	.	PASS	SVTYPE=INS:ME:LINE1;END=45854677;SVLEN=7;AN=20618;AC=1;AF=4.850130953535746e-05;N_HET=1;N_HOMALT=0;N_HOMREF=10308;FREQ_HET=9.700261907071492e-05;FREQ_HOMALT=0.0;FREQ_HOMREF=0.9999029973809292;N_BI_GENOS=10309;Southeast_Han_AN=1908;Southeast_Han_AC=1;South
chr1	46758744	.	G	<INS:ME:LINE1>	.	PASS	SVTYPE=INS:ME:LINE1;END=46758753;SVLEN=1026;AN=20618;AC=1;AF=4.850130953535746e-05;N_HET=1;N_HOMALT=0;N_HOMREF=10308;FREQ_HET=9.700261907071492e-05;FREQ_HOMALT=0.0;FREQ_HOMREF=0.9999029973809292;N_BI_GENOS=10309;East_Han_AN=6554;East_Han_AC=1;East_Han_AF=
chr1	46775830	.	T	<INS:ME:LINE1>	.	PASS	SVTYPE=INS:ME:LINE1;END=46775848;SVLEN=6000;AN=20618;AC=3;AF=0.00014550392860607236;N_HET=3;N_HOMALT=0;N_HOMREF=10306;FREQ_HET=0.0002910078572121447;FREQ_HOMALT=0.0;FREQ_HOMREF=0.9997089921427879;N_BI_GENOS=10309;East_Han_AN=6554;East_Han_AC=1;East_Han_AF
chr1	47229561	.	A	<INS:ME:LINE1>	.	PASS	SVTYPE=INS:ME:LINE1;END=47229603;SVLEN=3562;AN=20618;AC=1;AF=4.850130953535746e-05;N_HET=1;N_HOMALT=0;N_HOMREF=10308;FREQ_HET=9.700261907071492e-05;FREQ_HOMALT=0.0;FREQ_HOMREF=0.9999029973809292;N_BI_GENOS=10309;Central_Han_AN=2128;Central_Han_AC=1;Centra
chr1	47588699	.	T	<INS:ME:LINE1>	.	PASS	SVTYPE=INS:ME:LINE1;END=47588717;SVLEN=685;AN=20618;AC=1;AF=4.850130953535746e-05;N_HET=1;N_HOMALT=0;N_HOMREF=10308;FREQ_HET=9.700261907071492e-05;FREQ_HOMALT=0.0;FREQ_HOMREF=0.9999029973809292;N_BI_GENOS=10309;East_Han_AN=6554;East_Han_AC=1;East_Han_AF=0
chr1	47682969	.	A	<INS:ME:LINE1>	.	PASS	SVTYPE=INS:ME:LINE1;END=47682981;SVLEN=5994;AN=20618;AC=1;AF=4.850130953535746e-05;N_HET=1;N_HOMALT=0;N_HOMREF=10308;FREQ_HET=9.700261907071492e-05;FREQ_HOMALT=0.0;FREQ_HOMREF=0.9999029973809292;N_BI_GENOS=10309;East_Han_AN=6554;East_Han_AC=1;East_Han_AF=
chr1	47812139	.	T	<INS:ME:LINE1>	.	PASS	SVTYPE=INS:ME:LINE1;END=47812150;SVLEN=664;AN=20618;AC=1;AF=4.850130953535746e-05;N_HET=1;N_HOMALT=0;N_HOMREF=10308;FREQ_HET=9.700261907071492e-05;FREQ_HOMALT=0.0;FREQ_HOMREF=0.9999029973809292;N_BI_GENOS=10309;Southeast_Han_AN=1908;Southeast_Han_AC=1;Sou
chr1	48705641	.	C	<INS:ME:LINE1>	.	PASS	SVTYPE=INS:ME:LINE1;END=48705652;SVLEN=5963;AN=20618;AC=1;AF=4.850130953535746e-05;N_HET=1;N_HOMALT=0;N_HOMREF=10308;FREQ_HET=9.700261907071492e-05;FREQ_HOMALT=0.0;FREQ_HOMREF=0.9999029973809292;N_BI_GENOS=10309;Southeast_Han_AN=1908;Southeast_Han_AC=1;So
chr1	48778883	.	A	<INS:ME:LINE1>	.	PASS	SVTYPE=INS:ME:LINE1;END=48778884;SVLEN=327;AN=20618;AC=6;AF=0.0002910078572121447;N_HET=6;N_HOMALT=0;N_HOMREF=10303;FREQ_HET=0.0005820157144242894;FREQ_HOMALT=0.0;FREQ_HOMREF=0.9994179842855757;N_BI_GENOS=10309;Lingnan_Han_AN=1888;Lingnan_Han_AC=4;Lingnan
chr1	48963066	.	C	<INS:ME:LINE1>	.	PASS	SVTYPE=INS:ME:LINE1;END=48963084;SVLEN=5503;AN=20618;AC=2258;AF=0.10951595693083713;N_HET=1796;N_HOMALT=231;N_HOMREF=8282;FREQ_HET=0.174216703851004;FREQ_HOMALT=0.022407605005335143;FREQ_HOMREF=0.8033756911436609;N_BI_GENOS=10309;South_Han_AN=1496;South_H
chr1	48997577	.	G	<INS:ME:LINE1>	.	PASS	SVTYPE=INS:ME:LINE1;END=48997592;SVLEN=6000;AN=20618;AC=141;AF=0.006838684644485401;N_HET=139;N_HOMALT=1;N_HOMREF=10169;FREQ_HET=0.013483364050829373;FREQ_HOMALT=9.700261907071492e-05;FREQ_HOMREF=0.9864196333301;N_BI_GENOS=10309;South_Han_AN=1496;South_Ha
chr1	49090963	.	G	<INS:ME:LINE1>	.	PASS	SVTYPE=INS:ME:LINE1;END=49090963;SVLEN=573;AN=20618;AC=1;AF=4.850130953535746e-05;N_HET=1;N_HOMALT=0;N_HOMREF=10308;FREQ_HET=9.700261907071492e-05;FREQ_HOMALT=0.0;FREQ_HOMREF=0.9999029973809292;N_BI_GENOS=10309;East_Han_AN=6554;East_Han_AC=1;East_Han_AF=0
chr1	49092467	.	A	<INS:ME:LINE1>	.	PASS	SVTYPE=INS:ME:LINE1;END=49092476;SVLEN=2370;AN=20618;AC=1;AF=4.850130953535746e-05;N_HET=1;N_HOMALT=0;N_HOMREF=10308;FREQ_HET=9.700261907071492e-05;FREQ_HOMALT=0.0;FREQ_HOMREF=0.9999029973809292;N_BI_GENOS=10309;Central_Han_AN=2128;Central_Han_AC=1;Centra
chr1	49204024	.	A	<INS:ME:LINE1>	.	PASS	SVTYPE=INS:ME:LINE1;END=49204033;SVLEN=6002;AN=20618;AC=1;AF=4.850130953535746e-05;N_HET=1;N_HOMALT=0;N_HOMREF=10308;FREQ_HET=9.700261907071492e-05;FREQ_HOMALT=0.0;FREQ_HOMREF=0.9999029973809292;N_BI_GENOS=10309;Lingnan_Han_AN=1888;Lingnan_Han_AC=1;Lingna
chr1	49255347	.	C	<INS:ME:LINE1>	.	PASS	SVTYPE=INS:ME:LINE1;END=49255347;SVLEN=612;AN=20618;AC=1;AF=4.850130953535746e-05;N_HET=1;N_HOMALT=0;N_HOMREF=10308;FREQ_HET=9.700261907071492e-05;FREQ_HOMALT=0.0;FREQ_HOMREF=0.9999029973809292;N_BI_GENOS=10309;Northwest_Han_AN=1296;Northwest_Han_AC=1;Nor
chr1	49310708	.	C	<INS:ME:LINE1>	.	PASS	SVTYPE=INS:ME:LINE1;END=49310720;SVLEN=934;AN=20618;AC=2;AF=9.700261907071492e-05;N_HET=2;N_HOMALT=0;N_HOMREF=10307;FREQ_HET=0.00019400523814142983;FREQ_HOMALT=0.0;FREQ_HOMREF=0.9998059947618586;N_BI_GENOS=10309;Southeast_Han_AN=1908;Southeast_Han_AC=2;So
chr1	49325618	.	A	<INS:ME:LINE1>	.	PASS	SVTYPE=INS:ME:LINE1;END=49325634;SVLEN=965;AN=20618;AC=1;AF=4.850130953535746e-05;N_HET=1;N_HOMALT=0;N_HOMREF=10308;FREQ_HET=9.700261907071492e-05;FREQ_HOMALT=0.0;FREQ_HOMREF=0.9999029973809292;N_BI_GENOS=10309;Central_Han_AN=2128;Central_Han_AC=1;Central
chr1	49449616	.	A	<INS:ME:LINE1>	.	PASS	SVTYPE=INS:ME:LINE1;END=49449625;SVLEN=3039;AN=20618;AC=4;AF=0.00019400523814142983;N_HET=4;N_HOMALT=0;N_HOMREF=10305;FREQ_HET=0.00038801047628285966;FREQ_HOMALT=0.0;FREQ_HOMREF=0.9996119895237171;N_BI_GENOS=10309;North_Han_AN=2272;North_Han_AC=1;North_Ha
chr1	49472445	.	A	<INS:ME:LINE1>	.	PASS	SVTYPE=INS:ME:LINE1;END=49472463;SVLEN=2104;AN=20618;AC=1;AF=4.850130953535746e-05;N_HET=1;N_HOMALT=0;N_HOMREF=10308;FREQ_HET=9.700261907071492e-05;FREQ_HOMALT=0.0;FREQ_HOMREF=0.9999029973809292;N_BI_GENOS=10309;North_Han_AN=2272;North_Han_AC=1;North_Han_
chr1	49570876	.	T	<INS:ME:LINE1>	.	PASS	SVTYPE=INS:ME:LINE1;END=49570876;SVLEN=709;AN=20618;AC=1;AF=4.850130953535746e-05;N_HET=1;N_HOMALT=0;N_HOMREF=10308;FREQ_HET=9.700261907071492e-05;FREQ_HOMALT=0.0;FREQ_HOMREF=0.9999029973809292;N_BI_GENOS=10309;East_Han_AN=6554;East_Han_AC=1;East_Han_AF=0
chr1	49680381	.	A	<INS:ME:LINE1>	.	PASS	SVTYPE=INS:ME:LINE1;END=49680394;SVLEN=6000;AN=20618;AC=2;AF=9.700261907071492e-05;N_HET=0;N_HOMALT=1;N_HOMREF=10308;FREQ_HET=0.0;FREQ_HOMALT=9.700261907071492e-05;FREQ_HOMREF=0.9999029973809292;N_BI_GENOS=10309;East_Han_AN=6554;East_Han_AC=2;East_Han_AF=
chr1	49694042	.	A	<INS:ME:LINE1>	.	PASS	SVTYPE=INS:ME:LINE1;END=49694052;SVLEN=253;AN=20618;AC=1;AF=4.850130953535746e-05;N_HET=1;N_HOMALT=0;N_HOMREF=10308;FREQ_HET=9.700261907071492e-05;FREQ_HOMALT=0.0;FREQ_HOMREF=0.9999029973809292;N_BI_GENOS=10309;North_Han_AN=2272;North_Han_AC=1;North_Han_A
chr1	49851207	.	C	<INS:ME:LINE1>	.	PASS	SVTYPE=INS:ME:LINE1;END=49851212;SVLEN=44;AN=20618;AC=1;AF=4.850130953535746e-05;N_HET=1;N_HOMALT=0;N_HOMREF=10308;FREQ_HET=9.700261907071492e-05;FREQ_HOMALT=0.0;FREQ_HOMREF=0.9999029973809292;N_BI_GENOS=10309;Central_Han_AN=2128;Central_Han_AC=1;Central_
chr1	50143971	.	A	<INS:ME:LINE1>	.	PASS	SVTYPE=INS:ME:LINE1;END=50143982;SVLEN=6018;AN=20618;AC=2;AF=9.700261907071492e-05;N_HET=2;N_HOMALT=0;N_HOMREF=10307;FREQ_HET=0.00019400523814142983;FREQ_HOMALT=0.0;FREQ_HOMREF=0.9998059947618586;N_BI_GENOS=10309;North_Han_AN=2272;North_Han_AC=1;North_Han
chr1	50207191	.	A	<INS:ME:LINE1>	.	PASS	SVTYPE=INS:ME:LINE1;END=50207206;SVLEN=6000;AN=20618;AC=4;AF=0.00019400523814142983;N_HET=4;N_HOMALT=0;N_HOMREF=10305;FREQ_HET=0.00038801047628285966;FREQ_HOMALT=0.0;FREQ_HOMREF=0.9996119895237171;N_BI_GENOS=10309;Northwest_Han_AN=1296;Northwest_Han_AC=2;

chr1	50217047	.	A	<INS:ME:LINE1>	.	PASS	SVTYPE=INS:ME:LINE1;END=50217064;SVLEN=6000;AN=20618;AC=1438;AF=0.06974488311184401;N_HET=1220;N_HOMALT=109;N_HOMREF=8980;FREQ_HET=0.11834319526627218;FREQ_HOMALT=0.010573285478707926;FREQ_HOMREF=0.8710835192550199;N_BI_GENOS=10309;South_Han_AN=1496;South
chr1	52476656	.	A	<INS:ME:LINE1>	.	PASS	SVTYPE=INS:ME:LINE1;END=52476676;SVLEN=6025;AN=20618;AC=7;AF=0.0003395091667475022;N_HET=7;N_HOMALT=0;N_HOMREF=10302;FREQ_HET=0.0006790183334950044;FREQ_HOMALT=0.0;FREQ_HOMREF=0.999320981666505;N_BI_GENOS=10309;South_Han_AN=1496;South_Han_AC=2;South_Han_A
chr1	52976489	.	A	<INS:ME:LINE1>	.	PASS	SVTYPE=INS:ME:LINE1;END=52976507;SVLEN=6028;AN=20618;AC=1;AF=4.850130953535746e-05;N_HET=1;N_HOMALT=0;N_HOMREF=10308;FREQ_HET=9.700261907071492e-05;FREQ_HOMALT=0.0;FREQ_HOMREF=0.9999029973809292;N_BI_GENOS=10309;Southeast_Han_AN=1908;Southeast_Han_AC=1;So
chr1	53425973	.	T	<INS:ME:LINE1>	.	PASS	SVTYPE=INS:ME:LINE1;END=53425979;SVLEN=0;AN=20618;AC=29;AF=0.0014065379765253662;N_HET=5;N_HOMALT=12;N_HOMREF=10292;FREQ_HET=0.0004850130953535746;FREQ_HOMALT=0.0011640314288485789;FREQ_HOMREF=0.9983509554757979;N_BI_GENOS=10309;South_Han_AN=1496;South_H
chr1	53561261	.	A	<INS:ME:LINE1>	.	PASS	SVTYPE=INS:ME:LINE1;END=53561280;SVLEN=730;AN=20618;AC=3;AF=0.00014550392860607236;N_HET=1;N_HOMALT=1;N_HOMREF=10307;FREQ_HET=9.700261907071492e-05;FREQ_HOMALT=9.700261907071492e-05;FREQ_HOMREF=0.9998059947618586;N_BI_GENOS=10309;South_Han_AN=1496;South_H
chr1	55367392	.	T	<INS:ME:LINE1>	.	PASS	SVTYPE=INS:ME:LINE1;END=55367403;SVLEN=6000;AN=20618;AC=1;AF=4.850130953535746e-05;N_HET=1;N_HOMALT=0;N_HOMREF=10308;FREQ_HET=9.700261907071492e-05;FREQ_HOMALT=0.0;FREQ_HOMREF=0.9999029973809292;N_BI_GENOS=10309;East_Han_AN=6554;East_Han_AC=1;East_Han_AF=
chr1	55584626	.	A	<INS:ME:LINE1>	.	PASS	SVTYPE=INS:ME:LINE1;END=55584641;SVLEN=813;AN=20618;AC=1;AF=4.850130953535746e-05;N_HET=1;N_HOMALT=0;N_HOMREF=10308;FREQ_HET=9.700261907071492e-05;FREQ_HOMALT=0.0;FREQ_HOMREF=0.9999029973809292;N_BI_GENOS=10309;Central_Han_AN=2128;Central_Han_AC=1;Central
chr1	55709625	.	C	<INS:ME:LINE1>	.	PASS	SVTYPE=INS:ME:LINE1;END=55709636;SVLEN=830;AN=20618;AC=2;AF=9.700261907071492e-05;N_HET=2;N_HOMALT=0;N_HOMREF=10307;FREQ_HET=0.00019400523814142983;FREQ_HOMALT=0.0;FREQ_HOMREF=0.9998059947618586;N_BI_GENOS=10309;Northwest_Han_AN=1296;Northwest_Han_AC=1;No
chr1	55802976	.	T	<INS:ME:LINE1>	.	PASS	SVTYPE=INS:ME:LINE1;END=55802992;SVLEN=838;AN=20618;AC=584;AF=0.028324764768648755;N_HET=520;N_HOMALT=32;N_HOMREF=9757;FREQ_HET=0.05044136191677175;FREQ_HOMALT=0.0031040838102628773;FREQ_HOMREF=0.9464545542729653;N_BI_GENOS=10309;South_Han_AN=1496;South_H
chr1	55939713	.	T	<INS:ME:LINE1>	.	PASS	SVTYPE=INS:ME:LINE1;END=55939725;SVLEN=1530;AN=20618;AC=7016;AF=0.3402851877000679;N_HET=4504;N_HOMALT=1256;N_HOMREF=4549;FREQ_HET=0.43689979629449993;FREQ_HOMALT=0.12183528955281793;FREQ_HOMREF=0.4412649141526821;N_BI_GENOS=10309;South_Han_AN=1496;South_
chr1	56018127	.	G	<INS:ME:LINE1>	.	PASS	SVTYPE=INS:ME:LINE1;END=56018146;SVLEN=1308;AN=20618;AC=1;AF=4.850130953535746e-05;N_HET=1;N_HOMALT=0;N_HOMREF=10308;FREQ_HET=9.700261907071492e-05;FREQ_HOMALT=0.0;FREQ_HOMREF=0.9999029973809292;N_BI_GENOS=10309;North_Han_AN=2272;North_Han_AC=1;North_Han_
chr1	56070135	.	A	<INS:ME:LINE1>	.	PASS	SVTYPE=INS:ME:LINE1;END=56070144;SVLEN=435;AN=20618;AC=32;AF=0.0015520419051314386;N_HET=30;N_HOMALT=1;N_HOMREF=10278;FREQ_HET=0.0029100785721214474;FREQ_HOMALT=9.700261907071492e-05;FREQ_HOMREF=0.9969929188088078;N_BI_GENOS=10309;South_Han_AN=1496;South_
chr1	56667451	.	A	<INS:ME:LINE1>	.	PASS	SVTYPE=INS:ME:LINE1;END=56667464;SVLEN=6000;AN=20618;AC=4;AF=0.00019400523814142983;N_HET=4;N_HOMALT=0;N_HOMREF=10305;FREQ_HET=0.00038801047628285966;FREQ_HOMALT=0.0;FREQ_HOMREF=0.9996119895237171;N_BI_GENOS=10309;East_Han_AN=6554;East_Han_AC=3;East_Han_A
chr1	56716011	.	A	<INS:ME:LINE1>	.	PASS	SVTYPE=INS:ME:LINE1;END=56716011;SVLEN=6012;AN=20618;AC=1;AF=4.850130953535746e-05;N_HET=1;N_HOMALT=0;N_HOMREF=10308;FREQ_HET=9.700261907071492e-05;FREQ_HOMALT=0.0;FREQ_HOMREF=0.9999029973809292;N_BI_GENOS=10309;North_Han_AN=2272;North_Han_AC=1;North_Han_
chr1	56969564	.	G	<INS:ME:LINE1>	.	PASS	SVTYPE=INS:ME:LINE1;END=56969564;SVLEN=2540;AN=20618;AC=1;AF=4.850130953535746e-05;N_HET=1;N_HOMALT=0;N_HOMREF=10308;FREQ_HET=9.700261907071492e-05;FREQ_HOMALT=0.0;FREQ_HOMREF=0.9999029973809292;N_BI_GENOS=10309;Lingnan_Han_AN=1888;Lingnan_Han_AC=1;Lingna
chr1	57195378	.	A	<INS:ME:LINE1>	.	PASS	SVTYPE=INS:ME:LINE1;END=57195390;SVLEN=6056;AN=20618;AC=3;AF=0.00014550392860607236;N_HET=3;N_HOMALT=0;N_HOMREF=10306;FREQ_HET=0.0002910078572121447;FREQ_HOMALT=0.0;FREQ_HOMREF=0.9997089921427879;N_BI_GENOS=10309;South_Han_AN=1496;South_Han_AC=1;South_Han
chr1	58903207	.	G	<INS:ME:LINE1>	.	PASS	SVTYPE=INS:ME:LINE1;END=58903222;SVLEN=6014;AN=20618;AC=1;AF=4.850130953535746e-05;N_HET=1;N_HOMALT=0;N_HOMREF=10308;FREQ_HET=9.700261907071492e-05;FREQ_HOMALT=0.0;FREQ_HOMREF=0.9999029973809292;N_BI_GENOS=10309;South_Han_AN=1496;South_Han_AC=1;South_Han_
chr1	58992684	.	A	<INS:ME:LINE1>	.	PASS	SVTYPE=INS:ME:LINE1;END=58992701;SVLEN=4968;AN=20618;AC=2;AF=9.700261907071492e-05;N_HET=2;N_HOMALT=0;N_HOMREF=10307;FREQ_HET=0.00019400523814142983;FREQ_HOMALT=0.0;FREQ_HOMREF=0.9998059947618586;N_BI_GENOS=10309;North_Han_AN=2272;North_Han_AC=1;North_Han
chr1	59151087	.	A	<INS:ME:LINE1>	.	PASS	SVTYPE=INS:ME:LINE1;END=59151092;SVLEN=233;AN=20618;AC=1;AF=4.850130953535746e-05;N_HET=1;N_HOMALT=0;N_HOMREF=10308;FREQ_HET=9.700261907071492e-05;FREQ_HOMALT=0.0;FREQ_HOMREF=0.9999029973809292;N_BI_GENOS=10309;Southeast_Han_AN=1908;Southeast_Han_AC=1;Sou
chr1	59286303	.	A	<INS:ME:LINE1>	.	PASS	SVTYPE=INS:ME:LINE1;END=59286319;SVLEN=5969;AN=20618;AC=4;AF=0.00019400523814142983;N_HET=4;N_HOMALT=0;N_HOMREF=10305;FREQ_HET=0.00038801047628285966;FREQ_HOMALT=0.0;FREQ_HOMREF=0.9996119895237171;N_BI_GENOS=10309;North_Han_AN=2272;North_Han_AC=1;North_Ha
chr1	59439830	.	T	<INS:ME:LINE1>	.	PASS	SVTYPE=INS:ME:LINE1;END=59439847;SVLEN=3559;AN=20618;AC=2;AF=9.700261907071492e-05;N_HET=2;N_HOMALT=0;N_HOMREF=10307;FREQ_HET=0.00019400523814142983;FREQ_HOMALT=0.0;FREQ_HOMREF=0.9998059947618586;N_BI_GENOS=10309;South_Han_AN=1496;South_Han_AC=1;South_Han
chr1	59631510	.	A	<INS:ME:LINE1>	.	PASS	SVTYPE=INS:ME:LINE1;END=59631522;SVLEN=1517;AN=20618;AC=1;AF=4.850130953535746e-05;N_HET=1;N_HOMALT=0;N_HOMREF=10308;FREQ_HET=9.700261907071492e-05;FREQ_HOMALT=0.0;FREQ_HOMREF=0.9999029973809292;N_BI_GENOS=10309;Central_Han_AN=2128;Central_Han_AC=1;Centra
chr1	59758896	.	A	<INS:ME:LINE1>	.	PASS	SVTYPE=INS:ME:LINE1;END=59758912;SVLEN=1135;AN=20618;AC=1;AF=4.850130953535746e-05;N_HET=1;N_HOMALT=0;N_HOMREF=10308;FREQ_HET=9.700261907071492e-05;FREQ_HOMALT=0.0;FREQ_HOMREF=0.9999029973809292;N_BI_GENOS=10309;South_Han_AN=1496;South_Han_AC=1;South_Han_
chr1	60198150	.	A	<INS:ME:LINE1>	.	PASS	SVTYPE=INS:ME:LINE1;END=60198169;SVLEN=1229;AN=20618;AC=1;AF=4.850130953535746e-05;N_HET=1;N_HOMALT=0;N_HOMREF=10308;FREQ_HET=9.700261907071492e-05;FREQ_HOMALT=0.0;FREQ_HOMREF=0.9999029973809292;N_BI_GENOS=10309;South_Han_AN=1496;South_Han_AC=1;South_Han_
chr1	60250025	.	G	<INS:ME:LINE1>	.	PASS	SVTYPE=INS:ME:LINE1;END=60250042;SVLEN=6000;AN=20618;AC=969;AF=0.046997768939761375;N_HET=893;N_HOMALT=38;N_HOMREF=9378;FREQ_HET=0.08662333883014842;FREQ_HOMALT=0.0036860995246871665;FREQ_HOMREF=0.9096905616451644;N_BI_GENOS=10309;South_Han_AN=1496;South_
chr1	60446806	.	A	<INS:ME:LINE1>	.	PASS	SVTYPE=INS:ME:LINE1;END=60446819;SVLEN=783;AN=20618;AC=1;AF=4.850130953535746e-05;N_HET=1;N_HOMALT=0;N_HOMREF=10308;FREQ_HET=9.700261907071492e-05;FREQ_HOMALT=0.0;FREQ_HOMREF=0.9999029973809292;N_BI_GENOS=10309;Hui_AN=444;Hui_AC=1;Hui_AF=0.002252252252252
chr1	60448984	.	T	<INS:ME:LINE1>	.	PASS	SVTYPE=INS:ME:LINE1;END=60448984;SVLEN=5999;AN=20618;AC=1;AF=4.850130953535746e-05;N_HET=1;N_HOMALT=0;N_HOMREF=10308;FREQ_HET=9.700261907071492e-05;FREQ_HOMALT=0.0;FREQ_HOMREF=0.9999029973809292;N_BI_GENOS=10309;East_Han_AN=6554;East_Han_AC=1;East_Han_AF=
chr1	60941440	.	A	<INS:ME:LINE1>	.	PASS	SVTYPE=INS:ME:LINE1;END=60941454;SVLEN=1105;AN=20618;AC=1;AF=4.850130953535746e-05;N_HET=1;N_HOMALT=0;N_HOMREF=10308;FREQ_HET=9.700261907071492e-05;FREQ_HOMALT=0.0;FREQ_HOMREF=0.9999029973809292;N_BI_GENOS=10309;East_Han_AN=6554;East_Han_AC=1;East_Han_AF=
chr1	61110907	.	A	<INS:ME:LINE1>	.	PASS	SVTYPE=INS:ME:LINE1;END=61110923;SVLEN=5998;AN=20618;AC=1;AF=4.850130953535746e-05;N_HET=1;N_HOMALT=0;N_HOMREF=10308;FREQ_HET=9.700261907071492e-05;FREQ_HOMALT=0.0;FREQ_HOMREF=0.9999029973809292;N_BI_GENOS=10309;North_Han_AN=2272;North_Han_AC=1;North_Han_
chr1	61155679	.	A	<INS:ME:LINE1>	.	PASS	SVTYPE=INS:ME:LINE1;END=61155679;SVLEN=0;AN=20618;AC=2;AF=9.700261907071492e-05;N_HET=2;N_HOMALT=0;N_HOMREF=10307;FREQ_HET=0.00019400523814142983;FREQ_HOMALT=0.0;FREQ_HOMREF=0.9998059947618586;N_BI_GENOS=10309;Lingnan_Han_AN=1888;Lingnan_Han_AC=1;Lingnan_
chr1	61383472	.	G	<INS:ME:LINE1>	.	PASS	SVTYPE=INS:ME:LINE1;END=61383489;SVLEN=648;AN=20618;AC=1;AF=4.850130953535746e-05;N_HET=1;N_HOMALT=0;N_HOMREF=10308;FREQ_HET=9.700261907071492e-05;FREQ_HOMALT=0.0;FREQ_HOMREF=0.9999029973809292;N_BI_GENOS=10309;East_Han_AN=6554;East_Han_AC=1;East_Han_AF=0
chr1	61439290	.	A	<INS:ME:LINE1>	.	PASS	SVTYPE=INS:ME:LINE1;END=61439306;SVLEN=1004;AN=20618;AC=4;AF=0.00019400523814142983;N_HET=4;N_HOMALT=0;N_HOMREF=10305;FREQ_HET=0.00038801047628285966;FREQ_HOMALT=0.0;FREQ_HOMREF=0.9996119895237171;N_BI_GENOS=10309;South_Han_AN=1496;South_Han_AC=1;South_Ha
chr1	61495018	.	T	<INS:ME:LINE1>	.	PASS	SVTYPE=INS:ME:LINE1;END=61495033;SVLEN=5994;AN=20618;AC=3;AF=0.00014550392860607236;N_HET=3;N_HOMALT=0;N_HOMREF=10306;FREQ_HET=0.0002910078572121447;FREQ_HOMALT=0.0;FREQ_HOMREF=0.9997089921427879;N_BI_GENOS=10309;Lingnan_Han_AN=1888;Lingnan_Han_AC=2;Lingn
chr1	61520359	.	G	<INS:ME:LINE1>	.	PASS	SVTYPE=INS:ME:LINE1;END=61520372;SVLEN=5991;AN=20618;AC=1;AF=4.850130953535746e-05;N_HET=1;N_HOMALT=0;N_HOMREF=10308;FREQ_HET=9.700261907071492e-05;FREQ_HOMALT=0.0;FREQ_HOMREF=0.9999029973809292;N_BI_GENOS=10309;South_Han_AN=1496;South_Han_AC=1;South_Han_
chr1	61944171	.	A	<INS:ME:LINE1>	.	PASS	SVTYPE=INS:ME:LINE1;END=61944189;SVLEN=2954;AN=20618;AC=1;AF=4.850130953535746e-05;N_HET=1;N_HOMALT=0;N_HOMREF=10308;FREQ_HET=9.700261907071492e-05;FREQ_HOMALT=0.0;FREQ_HOMREF=0.9999029973809292;N_BI_GENOS=10309;Tibetan_AN=414;Tibetan_AC=1;Tibetan_AF=0.00
chr1	61985310	.	A	<INS:ME:LINE1>	.	PASS	SVTYPE=INS:ME:LINE1;END=61985329;SVLEN=817;AN=20618;AC=1;AF=4.850130953535746e-05;N_HET=1;N_HOMALT=0;N_HOMREF=10308;FREQ_HET=9.700261907071492e-05;FREQ_HOMALT=0.0;FREQ_HOMREF=0.9999029973809292;N_BI_GENOS=10309;South_Han_AN=1496;South_Han_AC=1;South_Han_A
chr1	62108130	.	A	<INS:ME:LINE1>	.	PASS	SVTYPE=INS:ME:LINE1;END=62108141;SVLEN=2386;AN=20618;AC=183;AF=0.008875739644970414;N_HET=179;N_HOMALT=2;N_HOMREF=10128;FREQ_HET=0.017363468813657967;FREQ_HOMALT=0.00019400523814142983;FREQ_HOMREF=0.9824425259482006;N_BI_GENOS=10309;South_Han_AN=1496;Sout
chr1	62160405	.	G	<INS:ME:LINE1>	.	PASS	SVTYPE=INS:ME:LINE1;END=62160417;SVLEN=5999;AN=20618;AC=4;AF=0.00019400523814142983;N_HET=4;N_HOMALT=0;N_HOMREF=10305;FREQ_HET=0.00038801047628285966;FREQ_HOMALT=0.0;FREQ_HOMREF=0.9996119895237171;N_BI_GENOS=10309;South_Han_AN=1496;South_Han_AC=1;South_Ha
chr1	62278323	.	C	<INS:ME:LINE1>	.	PASS	SVTYPE=INS:ME:LINE1;END=62278323;SVLEN=0;AN=20618;AC=2;AF=9.700261907071492e-05;N_HET=0;N_HOMALT=1;N_HOMREF=10308;FREQ_HET=0.0;FREQ_HOMALT=9.700261907071492e-05;FREQ_HOMREF=0.9999029973809292;N_BI_GENOS=10309;Southeast_Han_AN=1908;Southeast_Han_AC=2;South
chr1	62486930	.	T	<INS:ME:LINE1>	.	PASS	SVTYPE=INS:ME:LINE1;END=62486942;SVLEN=5998;AN=20618;AC=2;AF=9.700261907071492e-05;N_HET=2;N_HOMALT=0;N_HOMREF=10307;FREQ_HET=0.00019400523814142983;FREQ_HOMALT=0.0;FREQ_HOMREF=0.9998059947618586;N_BI_GENOS=10309;East_Han_AN=6554;East_Han_AC=2;East_Han_AF
chr1	63108282	.	C	<INS:ME:LINE1>	.	PASS	SVTYPE=INS:ME:LINE1;END=63108299;SVLEN=6003;AN=20618;AC=1;AF=4.850130953535746e-05;N_HET=1;N_HOMALT=0;N_HOMREF=10308;FREQ_HET=9.700261907071492e-05;FREQ_HOMALT=0.0;FREQ_HOMREF=0.9999029973809292;N_BI_GENOS=10309;North_Han_AN=2272;North_Han_AC=1;North_Han_
chr1	63646904	.	A	<INS:ME:LINE1>	.	PASS	SVTYPE=INS:ME:LINE1;END=63646917;SVLEN=6000;AN=20618;AC=1;AF=4.850130953535746e-05;N_HET=1;N_HOMALT=0;N_HOMREF=10308;FREQ_HET=9.700261907071492e-05;FREQ_HOMALT=0.0;FREQ_HOMREF=0.9999029973809292;N_BI_GENOS=10309;Southeast_Han_AN=1908;Southeast_Han_AC=1;So
chr1	63732411	.	C	<INS:ME:LINE1>	.	PASS	SVTYPE=INS:ME:LINE1;END=63732425;SVLEN=5997;AN=20618;AC=1;AF=4.850130953535746e-05;N_HET=1;N_HOMALT=0;N_HOMREF=10308;FREQ_HET=9.700261907071492e-05;FREQ_HOMALT=0.0;FREQ_HOMREF=0.9999029973809292;N_BI_GENOS=10309;Southeast_Han_AN=1908;Southeast_Han_AC=1;So
chr1	63938685	.	T	<INS:ME:LINE1>	.	PASS	SVTYPE=INS:ME:LINE1;END=63938698;SVLEN=1166;AN=20618;AC=1;AF=4.850130953535746e-05;N_HET=1;N_HOMALT=0;N_HOMREF=10308;FREQ_HET=9.700261907071492e-05;FREQ_HOMALT=0.0;FREQ_HOMREF=0.9999029973809292;N_BI_GENOS=10309;South_Han_AN=1496;South_Han_AC=1;South_Han_
chr1	64350010	.	C	<INS:ME:LINE1>	.	PASS	SVTYPE=INS:ME:LINE1;END=64350010;SVLEN=0;AN=20618;AC=27;AF=0.0013095353574546513;N_HET=13;N_HOMALT=7;N_HOMREF=10289;FREQ_HET=0.0012610304079192938;FREQ_HOMALT=0.0006790183334950044;FREQ_HOMREF=0.9980599476185857;N_BI_GENOS=10309;South_Han_AN=1496;South_Ha
chr1	64432301	.	A	<INS:ME:LINE1>	.	PASS	SVTYPE=INS:ME:LINE1;END=64432318;SVLEN=2046;AN=20618;AC=15;AF=0.0007275196430303619;N_HET=15;N_HOMALT=0;N_HOMREF=10294;FREQ_HET=0.0014550392860607237;FREQ_HOMALT=0.0;FREQ_HOMREF=0.9985449607139393;N_BI_GENOS=10309;South_Han_AN=1496;South_Han_AC=2;South_Ha
chr1	64771513	.	A	<INS:ME:LINE1>	.	PASS	SVTYPE=INS:ME:LINE1;END=64771527;SVLEN=2049;AN=20618;AC=1;AF=4.850130953535746e-05;N_HET=1;N_HOMALT=0;N_HOMREF=10308;FREQ_HET=9.700261907071492e-05;FREQ_HOMALT=0.0;FREQ_HOMREF=0.9999029973809292;N_BI_GENOS=10309;South_Han_AN=1496;South_Han_AC=1;South_Han_
chr1	64872809	.	C	<INS:ME:LINE1>	.	PASS	SVTYPE=INS:ME:LINE1;END=64872817;SVLEN=386;AN=20618;AC=1;AF=4.850130953535746e-05;N_HET=1;N_HOMALT=0;N_HOMREF=10308;FREQ_HET=9.700261907071492e-05;FREQ_HOMALT=0.0;FREQ_HOMREF=0.9999029973809292;N_BI_GENOS=10309;Northwest_Han_AN=1296;Northwest_Han_AC=1;Nor
chr1	64970916	.	A	<INS:ME:LINE1>	.	PASS	SVTYPE=INS:ME:LINE1;END=64970929;SVLEN=367;AN=20618;AC=1;AF=4.850130953535746e-05;N_HET=1;N_HOMALT=0;N_HOMREF=10308;FREQ_HET=9.700261907071492e-05;FREQ_HOMALT=0.0;FREQ_HOMREF=0.9999029973809292;N_BI_GENOS=10309;Northwest_Han_AN=1296;Northwest_Han_AC=1;Nor
chr1	65232739	.	G	<INS:ME:LINE1>	.	PASS	SVTYPE=INS:ME:LINE1;END=65232753;SVLEN=6004;AN=20618;AC=2;AF=9.700261907071492e-05;N_HET=2;N_HOMALT=0;N_HOMREF=10307;FREQ_HET=0.00019400523814142983;FREQ_HOMALT=0.0;FREQ_HOMREF=0.9998059947618586;N_BI_GENOS=10309;Southeast_Han_AN=1908;Southeast_Han_AC=2;S
chr1	65312133	.	G	<INS:ME:LINE1>	.	PASS	SVTYPE=INS:ME:LINE1;END=65312146;SVLEN=6066;AN=20618;AC=1;AF=4.850130953535746e-05;N_HET=1;N_HOMALT=0;N_HOMREF=10308;FREQ_HET=9.700261907071492e-05;FREQ_HOMALT=0.0;FREQ_HOMREF=0.9999029973809292;N_BI_GENOS=10309;Central_Han_AN=2128;Central_Han_AC=1;Centra
chr1	65492833	.	G	<INS:ME:LINE1>	.	PASS	SVTYPE=INS:ME:LINE1;END=65492847;SVLEN=6001;AN=20618;AC=1;AF=4.850130953535746e-05;N_HET=1;N_HOMALT=0;N_HOMREF=10308;FREQ_HET=9.700261907071492e-05;FREQ_HOMALT=0.0;FREQ_HOMREF=0.9999029973809292;N_BI_GENOS=10309;South_Han_AN=1496;South_Han_AC=1;South_Han_
chr1	65886235	.	A	<INS:ME:LINE1>	.	PASS	SVTYPE=INS:ME:LINE1;END=65886250;SVLEN=247;AN=20618;AC=1;AF=4.850130953535746e-05;N_HET=1;N_HOMALT=0;N_HOMREF=10308;FREQ_HET=9.700261907071492e-05;FREQ_HOMALT=0.0;FREQ_HOMREF=0.9999029973809292;N_BI_GENOS=10309;Northwest_Han_AN=1296;Northwest_Han_AC=1;Nor
chr1	65968886	.	G	<INS:ME:LINE1>	.	PASS	SVTYPE=INS:ME:LINE1;END=65968897;SVLEN=1528;AN=20618;AC=4;AF=0.00019400523814142983;N_HET=4;N_HOMALT=0;N_HOMREF=10305;FREQ_HET=0.00038801047628285966;FREQ_HOMALT=0.0;FREQ_HOMREF=0.9996119895237171;N_BI_GENOS=10309;Northwest_Han_AN=1296;Northwest_Han_AC=1;
chr1	66503639	.	A	<INS:ME:LINE1>	.	PASS	SVTYPE=INS:ME:LINE1;END=66503651;SVLEN=4699;AN=20618;AC=4;AF=0.00019400523814142983;N_HET=2;N_HOMALT=1;N_HOMREF=10306;FREQ_HET=0.00019400523814142983;FREQ_HOMALT=9.700261907071492e-05;FREQ_HOMREF=0.9997089921427879;N_BI_GENOS=10309;South_Han_AN=1496;South
chr1	66789400	.	T	<INS:ME:LINE1>	.	PASS	SVTYPE=INS:ME:LINE1;END=66789420;SVLEN=5999;AN=20618;AC=1;AF=4.850130953535746e-05;N_HET=1;N_HOMALT=0;N_HOMREF=10308;FREQ_HET=9.700261907071492e-05;FREQ_HOMALT=0.0;FREQ_HOMREF=0.9999029973809292;N_BI_GENOS=10309;North_Han_AN=2272;North_Han_AC=1;North_Han_
chr1	66851943	.	A	<INS:ME:LINE1>	.	PASS	SVTYPE=INS:ME:LINE1;END=66851960;SVLEN=101;AN=20618;AC=21;AF=0.0010185275002425065;N_HET=19;N_HOMALT=1;N_HOMREF=10289;FREQ_HET=0.0018430497623435833;FREQ_HOMALT=9.700261907071492e-05;FREQ_HOMREF=0.9980599476185857;N_BI_GENOS=10309;South_Han_AN=1496;South_
chr1	67197232	.	T	<INS:ME:LINE1>	.	PASS	SVTYPE=INS:ME:LINE1;END=67197245;SVLEN=1412;AN=20618;AC=2;AF=9.700261907071492e-05;N_HET=2;N_HOMALT=0;N_HOMREF=10307;FREQ_HET=0.00019400523814142983;FREQ_HOMALT=0.0;FREQ_HOMREF=0.9998059947618586;N_BI_GENOS=10309;East_Han_AN=6554;East_Han_AC=2;East_Han_AF
chr1	67223923	.	A	<INS:ME:LINE1>	.	PASS	SVTYPE=INS:ME:LINE1;END=67223941;SVLEN=5996;AN=20618;AC=1;AF=4.850130953535746e-05;N_HET=1;N_HOMALT=0;N_HOMREF=10308;FREQ_HET=9.700261907071492e-05;FREQ_HOMALT=0.0;FREQ_HOMREF=0.9999029973809292;N_BI_GENOS=10309;North_Han_AN=2272;North_Han_AC=1;North_Han_
chr1	67313527	.	A	<INS:ME:LINE1>	.	PASS	SVTYPE=INS:ME:LINE1;END=67313543;SVLEN=5999;AN=20618;AC=1;AF=4.850130953535746e-05;N_HET=1;N_HOMALT=0;N_HOMREF=10308;FREQ_HET=9.700261907071492e-05;FREQ_HOMALT=0.0;FREQ_HOMREF=0.9999029973809292;N_BI_GENOS=10309;East_Han_AN=6554;East_Han_AC=1;East_Han_AF=
chr1	67651935	.	C	<INS:ME:LINE1>	.	PASS	SVTYPE=INS:ME:LINE1;END=67651949;SVLEN=6007;AN=20618;AC=1;AF=4.850130953535746e-05;N_HET=1;N_HOMALT=0;N_HOMREF=10308;FREQ_HET=9.700261907071492e-05;FREQ_HOMALT=0.0;FREQ_HOMREF=0.9999029973809292;N_BI_GENOS=10309;Northwest_Han_AN=1296;Northwest_Han_AC=1;No
chr1	67864593	.	C	<INS:ME:LINE1>	.	PASS	SVTYPE=INS:ME:LINE1;END=67864611;SVLEN=6000;AN=20618;AC=3;AF=0.00014550392860607236;N_HET=3;N_HOMALT=0;N_HOMREF=10306;FREQ_HET=0.0002910078572121447;FREQ_HOMALT=0.0;FREQ_HOMREF=0.9997089921427879;N_BI_GENOS=10309;Central_Han_AN=2128;Central_Han_AC=1;Centr
chr1	67930035	.	T	<INS:ME:LINE1>	.	PASS	SVTYPE=INS:ME:LINE1;END=67930035;SVLEN=0;AN=20618;AC=5;AF=0.00024250654767678728;N_HET=1;N_HOMALT=2;N_HOMREF=10306;FREQ_HET=9.700261907071492e-05;FREQ_HOMALT=0.00019400523814142983;FREQ_HOMREF=0.9997089921427879;N_BI_GENOS=10309;Lingnan_Han_AN=1888;Lingna
chr1	68224894	.	A	<INS:ME:LINE1>	.	PASS	SVTYPE=INS:ME:LINE1;END=68224912;SVLEN=1593;AN=20618;AC=1;AF=4.850130953535746e-05;N_HET=1;N_HOMALT=0;N_HOMREF=10308;FREQ_HET=9.700261907071492e-05;FREQ_HOMALT=0.0;FREQ_HOMREF=0.9999029973809292;N_BI_GENOS=10309;East_Han_AN=6554;East_Han_AC=1;East_Han_AF=
chr1	68636191	.	A	<INS:ME:LINE1>	.	PASS	SVTYPE=INS:ME:LINE1;END=68636202;SVLEN=1192;AN=20618;AC=2;AF=9.700261907071492e-05;N_HET=2;N_HOMALT=0;N_HOMREF=10307;FREQ_HET=0.00019400523814142983;FREQ_HOMALT=0.0;FREQ_HOMREF=0.9998059947618586;N_BI_GENOS=10309;Central_Han_AN=2128;Central_Han_AC=2;Centr
chr1	68676089	.	A	<INS:ME:LINE1>	.	PASS	SVTYPE=INS:ME:LINE1;END=68676101;SVLEN=863;AN=20618;AC=1;AF=4.850130953535746e-05;N_HET=1;N_HOMALT=0;N_HOMREF=10308;FREQ_HET=9.700261907071492e-05;FREQ_HOMALT=0.0;FREQ_HOMREF=0.9999029973809292;N_BI_GENOS=10309;Southeast_Han_AN=1908;Southeast_Han_AC=1;Sou
chr1	68853244	.	T	<INS:ME:LINE1>	.	PASS	SVTYPE=INS:ME:LINE1;END=68853244;SVLEN=318;AN=20618;AC=1;AF=4.850130953535746e-05;N_HET=1;N_HOMALT=0;N_HOMREF=10308;FREQ_HET=9.700261907071492e-05;FREQ_HOMALT=0.0;FREQ_HOMREF=0.9999029973809292;N_BI_GENOS=10309;East_Han_AN=6554;East_Han_AC=1;East_Han_AF=0
chr1	68883105	.	A	<INS:ME:LINE1>	.	PASS	SVTYPE=INS:ME:LINE1;END=68883113;SVLEN=748;AN=20618;AC=1;AF=4.850130953535746e-05;N_HET=1;N_HOMALT=0;N_HOMREF=10308;FREQ_HET=9.700261907071492e-05;FREQ_HOMALT=0.0;FREQ_HOMREF=0.9999029973809292;N_BI_GENOS=10309;South_Han_AN=1496;South_Han_AC=1;South_Han_A
chr1	69001626	.	C	<INS:ME:LINE1>	.	PASS	SVTYPE=INS:ME:LINE1;END=69001639;SVLEN=175;AN=20618;AC=1;AF=4.850130953535746e-05;N_HET=1;N_HOMALT=0;N_HOMREF=10308;FREQ_HET=9.700261907071492e-05;FREQ_HOMALT=0.0;FREQ_HOMREF=0.9999029973809292;N_BI_GENOS=10309;Southeast_Han_AN=1908;Southeast_Han_AC=1;Sou
chr1	69152680	.	A	<INS:ME:LINE1>	.	PASS	SVTYPE=INS:ME:LINE1;END=69152680;SVLEN=269;AN=20618;AC=1;AF=4.850130953535746e-05;N_HET=1;N_HOMALT=0;N_HOMREF=10308;FREQ_HET=9.700261907071492e-05;FREQ_HOMALT=0.0;FREQ_HOMREF=0.9999029973809292;N_BI_GENOS=10309;East_Han_AN=6554;East_Han_AC=1;East_Han_AF=0
chr1	69771344	.	A	<INS:ME:LINE1>	.	PASS	SVTYPE=INS:ME:LINE1;END=69771344;SVLEN=6034;AN=20618;AC=2;AF=9.700261907071492e-05;N_HET=2;N_HOMALT=0;N_HOMREF=10307;FREQ_HET=0.00019400523814142983;FREQ_HOMALT=0.0;FREQ_HOMREF=0.9998059947618586;N_BI_GENOS=10309;Lingnan_Han_AN=1888;Lingnan_Han_AC=1;Lingn
chr1	69866273	.	G	<INS:ME:LINE1>	.	PASS	SVTYPE=INS:ME:LINE1;END=69866273;SVLEN=1359;AN=20618;AC=1;AF=4.850130953535746e-05;N_HET=1;N_HOMALT=0;N_HOMREF=10308;FREQ_HET=9.700261907071492e-05;FREQ_HOMALT=0.0;FREQ_HOMREF=0.9999029973809292;N_BI_GENOS=10309;Southeast_Han_AN=1908;Southeast_Han_AC=1;So
chr1	70003431	.	G	<INS:ME:LINE1>	.	PASS	SVTYPE=INS:ME:LINE1;END=70003445;SVLEN=1441;AN=20618;AC=2;AF=9.700261907071492e-05;N_HET=0;N_HOMALT=1;N_HOMREF=10308;FREQ_HET=0.0;FREQ_HOMALT=9.700261907071492e-05;FREQ_HOMREF=0.9999029973809292;N_BI_GENOS=10309;Hui_AN=444;Hui_AC=2;Hui_AF=0.00450450450450
chr1	70079788	.	A	<INS:ME:LINE1>	.	PASS	SVTYPE=INS:ME:LINE1;END=70079805;SVLEN=6004;AN=20618;AC=3;AF=0.00014550392860607236;N_HET=3;N_HOMALT=0;N_HOMREF=10306;FREQ_HET=0.0002910078572121447;FREQ_HOMALT=0.0;FREQ_HOMREF=0.9997089921427879;N_BI_GENOS=10309;East_Han_AN=6554;East_Han_AC=1;East_Han_AF
chr1	70295568	.	T	<INS:ME:LINE1>	.	PASS	SVTYPE=INS:ME:LINE1;END=70295579;SVLEN=748;AN=20618;AC=1;AF=4.850130953535746e-05;N_HET=1;N_HOMALT=0;N_HOMREF=10308;FREQ_HET=9.700261907071492e-05;FREQ_HOMALT=0.0;FREQ_HOMREF=0.9999029973809292;N_BI_GENOS=10309;East_Han_AN=6554;East_Han_AC=1;East_Han_AF=0
chr1	70323146	.	G	<INS:ME:LINE1>	.	PASS	SVTYPE=INS:ME:LINE1;END=70323163;SVLEN=5994;AN=20618;AC=2;AF=9.700261907071492e-05;N_HET=2;N_HOMALT=0;N_HOMREF=10307;FREQ_HET=0.00019400523814142983;FREQ_HOMALT=0.0;FREQ_HOMREF=0.9998059947618586;N_BI_GENOS=10309;North_Han_AN=2272;North_Han_AC=2;North_Han
chr1	70383467	.	A	<INS:ME:LINE1>	.	PASS	SVTYPE=INS:ME:LINE1;END=70383467;SVLEN=774;AN=20618;AC=1;AF=4.850130953535746e-05;N_HET=1;N_HOMALT=0;N_HOMREF=10308;FREQ_HET=9.700261907071492e-05;FREQ_HOMALT=0.0;FREQ_HOMREF=0.9999029973809292;N_BI_GENOS=10309;Southeast_Han_AN=1908;Southeast_Han_AC=1;Sou
chr1	70498531	.	G	<INS:ME:LINE1>	.	PASS	SVTYPE=INS:ME:LINE1;END=70498549;SVLEN=6002;AN=20618;AC=1;AF=4.850130953535746e-05;N_HET=1;N_HOMALT=0;N_HOMREF=10308;FREQ_HET=9.700261907071492e-05;FREQ_HOMALT=0.0;FREQ_HOMREF=0.9999029973809292;N_BI_GENOS=10309;Central_Han_AN=2128;Central_Han_AC=1;Centra
chr1	70576790	.	A	<INS:ME:LINE1>	.	PASS	SVTYPE=INS:ME:LINE1;END=70576804;SVLEN=6000;AN=20618;AC=1;AF=4.850130953535746e-05;N_HET=1;N_HOMALT=0;N_HOMREF=10308;FREQ_HET=9.700261907071492e-05;FREQ_HOMALT=0.0;FREQ_HOMREF=0.9999029973809292;N_BI_GENOS=10309;South_Han_AN=1496;South_Han_AC=1;South_Han_
chr1	70683271	.	T	<INS:ME:LINE1>	.	PASS	SVTYPE=INS:ME:LINE1;END=70683271;SVLEN=1669;AN=20618;AC=1;AF=4.850130953535746e-05;N_HET=1;N_HOMALT=0;N_HOMREF=10308;FREQ_HET=9.700261907071492e-05;FREQ_HOMALT=0.0;FREQ_HOMREF=0.9999029973809292;N_BI_GENOS=10309;East_Han_AN=6554;East_Han_AC=1;East_Han_AF=
chr1	70741350	.	T	<INS:ME:LINE1>	.	PASS	SVTYPE=INS:ME:LINE1;END=70741350;SVLEN=912;AN=20618;AC=1;AF=4.850130953535746e-05;N_HET=1;N_HOMALT=0;N_HOMREF=10308;FREQ_HET=9.700261907071492e-05;FREQ_HOMALT=0.0;FREQ_HOMREF=0.9999029973809292;N_BI_GENOS=10309;East_Han_AN=6554;East_Han_AC=1;East_Han_AF=0
chr1	70782908	.	A	<INS:ME:LINE1>	.	PASS	SVTYPE=INS:ME:LINE1;END=70782926;SVLEN=5997;AN=20618;AC=4637;AF=0.22490057231545252;N_HET=2859;N_HOMALT=889;N_HOMREF=6561;FREQ_HET=0.27733048792317394;FREQ_HOMALT=0.08623532835386555;FREQ_HOMREF=0.6364341837229606;N_BI_GENOS=10309;South_Han_AN=1496;South_
chr1	71072413	.	A	<INS:ME:LINE1>	.	PASS	SVTYPE=INS:ME:LINE1;END=71072427;SVLEN=5993;AN=20618;AC=1;AF=4.850130953535746e-05;N_HET=1;N_HOMALT=0;N_HOMREF=10308;FREQ_HET=9.700261907071492e-05;FREQ_HOMALT=0.0;FREQ_HOMREF=0.9999029973809292;N_BI_GENOS=10309;Northwest_Han_AN=1296;Northwest_Han_AC=1;No
chr1	71705795	.	A	<INS:ME:LINE1>	.	PASS	SVTYPE=INS:ME:LINE1;END=71705808;SVLEN=347;AN=20618;AC=1;AF=4.850130953535746e-05;N_HET=1;N_HOMALT=0;N_HOMREF=10308;FREQ_HET=9.700261907071492e-05;FREQ_HOMALT=0.0;FREQ_HOMREF=0.9999029973809292;N_BI_GENOS=10309;Tibetan_AN=414;Tibetan_AC=1;Tibetan_AF=0.002
chr1	71789212	.	A	<INS:ME:LINE1>	.	PASS	SVTYPE=INS:ME:LINE1;END=71789229;SVLEN=350;AN=20618;AC=2;AF=9.700261907071492e-05;N_HET=2;N_HOMALT=0;N_HOMREF=10307;FREQ_HET=0.00019400523814142983;FREQ_HOMALT=0.0;FREQ_HOMREF=0.9998059947618586;N_BI_GENOS=10309;South_Han_AN=1496;South_Han_AC=1;South_Han_
chr1	72049168	.	A	<INS:ME:LINE1>	.	PASS	SVTYPE=INS:ME:LINE1;END=72049168;SVLEN=695;AN=20618;AC=1;AF=4.850130953535746e-05;N_HET=1;N_HOMALT=0;N_HOMREF=10308;FREQ_HET=9.700261907071492e-05;FREQ_HOMALT=0.0;FREQ_HOMREF=0.9999029973809292;N_BI_GENOS=10309;Lingnan_Han_AN=1888;Lingnan_Han_AC=1;Lingnan
chr1	72105272	.	C	<INS:ME:LINE1>	.	PASS	SVTYPE=INS:ME:LINE1;END=72105291;SVLEN=1932;AN=20618;AC=1;AF=4.850130953535746e-05;N_HET=1;N_HOMALT=0;N_HOMREF=10308;FREQ_HET=9.700261907071492e-05;FREQ_HOMALT=0.0;FREQ_HOMREF=0.9999029973809292;N_BI_GENOS=10309;Southeast_Han_AN=1908;Southeast_Han_AC=1;So
chr1	72199835	.	A	<INS:ME:LINE1>	.	PASS	SVTYPE=INS:ME:LINE1;END=72199835;SVLEN=534;AN=20618;AC=1;AF=4.850130953535746e-05;N_HET=1;N_HOMALT=0;N_HOMREF=10308;FREQ_HET=9.700261907071492e-05;FREQ_HOMALT=0.0;FREQ_HOMREF=0.9999029973809292;N_BI_GENOS=10309;East_Han_AN=6554;East_Han_AC=1;East_Han_AF=0
chr1	72199992	.	C	<INS:ME:LINE1>	.	PASS	SVTYPE=INS:ME:LINE1;END=72199992;SVLEN=742;AN=20618;AC=2;AF=9.700261907071492e-05;N_HET=0;N_HOMALT=1;N_HOMREF=10308;FREQ_HET=0.0;FREQ_HOMALT=9.700261907071492e-05;FREQ_HOMREF=0.9999029973809292;N_BI_GENOS=10309;East_Han_AN=6554;East_Han_AC=2;East_Han_AF=0
chr1	72339812	.	A	<INS:ME:LINE1>	.	PASS	SVTYPE=INS:ME:LINE1;END=72339812;SVLEN=153;AN=20618;AC=4;AF=0.00019400523814142983;N_HET=0;N_HOMALT=2;N_HOMREF=10307;FREQ_HET=0.0;FREQ_HOMALT=0.00019400523814142983;FREQ_HOMREF=0.9998059947618586;N_BI_GENOS=10309;Northwest_Han_AN=1296;Northwest_Han_AC=2;N
chr1	72368971	.	C	<INS:ME:LINE1>	.	PASS	SVTYPE=INS:ME:LINE1;END=72368971;SVLEN=474;AN=20618;AC=1;AF=4.850130953535746e-05;N_HET=1;N_HOMALT=0;N_HOMREF=10308;FREQ_HET=9.700261907071492e-05;FREQ_HOMALT=0.0;FREQ_HOMREF=0.9999029973809292;N_BI_GENOS=10309;East_Han_AN=6554;East_Han_AC=1;East_Han_AF=0
chr1	72523726	.	A	<INS:ME:LINE1>	.	PASS	SVTYPE=INS:ME:LINE1;END=72523741;SVLEN=1495;AN=20618;AC=34;AF=0.0016490445242021534;N_HET=28;N_HOMALT=3;N_HOMREF=10278;FREQ_HET=0.0027160733339800175;FREQ_HOMALT=0.0002910078572121447;FREQ_HOMREF=0.9969929188088078;N_BI_GENOS=10309;South_Han_AN=1496;South
chr1	72561955	.	A	<INS:ME:LINE1>	.	PASS	SVTYPE=INS:ME:LINE1;END=72561955;SVLEN=1324;AN=20618;AC=1;AF=4.850130953535746e-05;N_HET=1;N_HOMALT=0;N_HOMREF=10308;FREQ_HET=9.700261907071492e-05;FREQ_HOMALT=0.0;FREQ_HOMREF=0.9999029973809292;N_BI_GENOS=10309;North_Han_AN=2272;North_Han_AC=1;North_Han_
chr1	72689445	.	G	<INS:ME:LINE1>	.	PASS	SVTYPE=INS:ME:LINE1;END=72689445;SVLEN=397;AN=20618;AC=1;AF=4.850130953535746e-05;N_HET=1;N_HOMALT=0;N_HOMREF=10308;FREQ_HET=9.700261907071492e-05;FREQ_HOMALT=0.0;FREQ_HOMREF=0.9999029973809292;N_BI_GENOS=10309;North_Han_AN=2272;North_Han_AC=1;North_Han_A
chr1	73245375	.	C	<INS:ME:LINE1>	.	PASS	SVTYPE=INS:ME:LINE1;END=73245375;SVLEN=128;AN=20618;AC=1;AF=4.850130953535746e-05;N_HET=1;N_HOMALT=0;N_HOMREF=10308;FREQ_HET=9.700261907071492e-05;FREQ_HOMALT=0.0;FREQ_HOMREF=0.9999029973809292;N_BI_GENOS=10309;East_Han_AN=6554;East_Han_AC=1;East_Han_AF=0
chr1	73322859	.	A	<INS:ME:LINE1>	.	PASS	SVTYPE=INS:ME:LINE1;END=73322882;SVLEN=1640;AN=20618;AC=1;AF=4.850130953535746e-05;N_HET=1;N_HOMALT=0;N_HOMREF=10308;FREQ_HET=9.700261907071492e-05;FREQ_HOMALT=0.0;FREQ_HOMREF=0.9999029973809292;N_BI_GENOS=10309;East_Han_AN=6554;East_Han_AC=1;East_Han_AF=

chr1	73410850	.	G	<INS:ME:LINE1>	.	PASS	SVTYPE=INS:ME:LINE1;END=73410864;SVLEN=411;AN=20618;AC=1;AF=4.850130953535746e-05;N_HET=1;N_HOMALT=0;N_HOMREF=10308;FREQ_HET=9.700261907071492e-05;FREQ_HOMALT=0.0;FREQ_HOMREF=0.9999029973809292;N_BI_GENOS=10309;North_Han_AN=2272;North_Han_AC=1;North_Han_A
chr1	73533471	.	G	<INS:ME:LINE1>	.	PASS	SVTYPE=INS:ME:LINE1;END=73533471;SVLEN=1144;AN=20618;AC=1;AF=4.850130953535746e-05;N_HET=1;N_HOMALT=0;N_HOMREF=10308;FREQ_HET=9.700261907071492e-05;FREQ_HOMALT=0.0;FREQ_HOMREF=0.9999029973809292;N_BI_GENOS=10309;East_Han_AN=6554;East_Han_AC=1;East_Han_AF=
chr1	73549544	.	A	<INS:ME:LINE1>	.	PASS	SVTYPE=INS:ME:LINE1;END=73549544;SVLEN=0;AN=20618;AC=4;AF=0.00019400523814142983;N_HET=0;N_HOMALT=2;N_HOMREF=10307;FREQ_HET=0.0;FREQ_HOMALT=0.00019400523814142983;FREQ_HOMREF=0.9998059947618586;N_BI_GENOS=10309;East_Han_AN=6554;East_Han_AC=4;East_Han_AF=0
chr1	73566710	.	G	<INS:ME:LINE1>	.	PASS	SVTYPE=INS:ME:LINE1;END=73566710;SVLEN=0;AN=20618;AC=6;AF=0.0002910078572121447;N_HET=0;N_HOMALT=3;N_HOMREF=10306;FREQ_HET=0.0;FREQ_HOMALT=0.0002910078572121447;FREQ_HOMREF=0.9997089921427879;N_BI_GENOS=10309;East_Han_AN=6554;East_Han_AC=4;East_Han_AF=0.0
chr1	73677896	.	C	<INS:ME:LINE1>	.	PASS	SVTYPE=INS:ME:LINE1;END=73677912;SVLEN=1225;AN=20618;AC=1;AF=4.850130953535746e-05;N_HET=1;N_HOMALT=0;N_HOMREF=10308;FREQ_HET=9.700261907071492e-05;FREQ_HOMALT=0.0;FREQ_HOMREF=0.9999029973809292;N_BI_GENOS=10309;Lingnan_Han_AN=1888;Lingnan_Han_AC=1;Lingna
chr1	74061547	.	T	<INS:ME:LINE1>	.	PASS	SVTYPE=INS:ME:LINE1;END=74061560;SVLEN=1445;AN=20618;AC=1;AF=4.850130953535746e-05;N_HET=1;N_HOMALT=0;N_HOMREF=10308;FREQ_HET=9.700261907071492e-05;FREQ_HOMALT=0.0;FREQ_HOMREF=0.9999029973809292;N_BI_GENOS=10309;East_Han_AN=6554;East_Han_AC=1;East_Han_AF=
chr1	74124387	.	T	<INS:ME:LINE1>	.	PASS	SVTYPE=INS:ME:LINE1;END=74124387;SVLEN=857;AN=20618;AC=1;AF=4.850130953535746e-05;N_HET=1;N_HOMALT=0;N_HOMREF=10308;FREQ_HET=9.700261907071492e-05;FREQ_HOMALT=0.0;FREQ_HOMREF=0.9999029973809292;N_BI_GENOS=10309;East_Han_AN=6554;East_Han_AC=1;East_Han_AF=0
chr1	74416702	.	A	<INS:ME:LINE1>	.	PASS	SVTYPE=INS:ME:LINE1;END=74416707;SVLEN=719;AN=20618;AC=3;AF=0.00014550392860607236;N_HET=3;N_HOMALT=0;N_HOMREF=10306;FREQ_HET=0.0002910078572121447;FREQ_HOMALT=0.0;FREQ_HOMREF=0.9997089921427879;N_BI_GENOS=10309;South_Han_AN=1496;South_Han_AC=1;South_Han_
chr1	74441903	.	T	<INS:ME:LINE1>	.	PASS	SVTYPE=INS:ME:LINE1;END=74441919;SVLEN=1016;AN=20618;AC=1;AF=4.850130953535746e-05;N_HET=1;N_HOMALT=0;N_HOMREF=10308;FREQ_HET=9.700261907071492e-05;FREQ_HOMALT=0.0;FREQ_HOMREF=0.9999029973809292;N_BI_GENOS=10309;Lingnan_Han_AN=1888;Lingnan_Han_AC=1;Lingna
chr1	74595750	.	A	<INS:ME:LINE1>	.	PASS	SVTYPE=INS:ME:LINE1;END=74595761;SVLEN=5337;AN=20618;AC=10;AF=0.0004850130953535455;N_HET=10;N_HOMALT=0;N_HOMREF=10299;FREQ_HET=0.0009700261907071491;FREQ_HOMALT=0.0;FREQ_HOMREF=0.9990299738092928;N_BI_GENOS=10309;North_Han_AN=2272;North_Han_AC=2;North_H
chr1	74682360	.	T	<INS:ME:LINE1>	.	PASS	SVTYPE=INS:ME:LINE1;END=74682360;SVLEN=15;AN=20618;AC=2;AF=9.700261907071492e-05;N_HET=2;N_HOMALT=0;N_HOMREF=10307;FREQ_HET=0.00019400523814142983;FREQ_HOMALT=0.0;FREQ_HOMREF=0.9998059947618586;N_BI_GENOS=10309;South_Han_AN=1496;South_Han_AC=1;South_Han_A
chr1	74727205	.	C	<INS:ME:LINE1>	.	PASS	SVTYPE=INS:ME:LINE1;END=74727223;SVLEN=3654;AN=20618;AC=363;AF=0.017605975361334757;N_HET=345;N_HOMALT=9;N_HOMREF=9955;FREQ_HET=0.03346590357939665;FREQ_HOMALT=0.0008730235716364342;FREQ_HOMREF=0.965661072848967;N_BI_GENOS=10309;South_Han_AN=1496;South_Ha
chr1	74965136	.	A	<INS:ME:LINE1>	.	PASS	SVTYPE=INS:ME:LINE1;END=74965153;SVLEN=6023;AN=20618;AC=1;AF=4.850130953535746e-05;N_HET=1;N_HOMALT=0;N_HOMREF=10308;FREQ_HET=9.700261907071492e-05;FREQ_HOMALT=0.0;FREQ_HOMREF=0.9999029973809292;N_BI_GENOS=10309;Central_Han_AN=2128;Central_Han_AC=1;Centra
chr1	74983678	.	A	<INS:ME:LINE1>	.	PASS	SVTYPE=INS:ME:LINE1;END=74983693;SVLEN=556;AN=20618;AC=1;AF=4.850130953535746e-05;N_HET=1;N_HOMALT=0;N_HOMREF=10308;FREQ_HET=9.700261907071492e-05;FREQ_HOMALT=0.0;FREQ_HOMREF=0.9999029973809292;N_BI_GENOS=10309;South_Han_AN=1496;South_Han_AC=1;South_Han_A
chr1	75395824	.	T	<INS:ME:LINE1>	.	PASS	SVTYPE=INS:ME:LINE1;END=75395838;SVLEN=5998;AN=20618;AC=16;AF=0.0007760209525657193;N_HET=16;N_HOMALT=0;N_HOMREF=10293;FREQ_HET=0.0015520419051314386;FREQ_HOMALT=0.0;FREQ_HOMREF=0.9984479580948685;N_BI_GENOS=10309;South_Han_AN=1496;South_Han_AC=2;South_Ha
chr1	75500822	.	A	<INS:ME:LINE1>	.	PASS	SVTYPE=INS:ME:LINE1;END=75500837;SVLEN=330;AN=20618;AC=1;AF=4.850130953535746e-05;N_HET=1;N_HOMALT=0;N_HOMREF=10308;FREQ_HET=9.700261907071492e-05;FREQ_HOMALT=0.0;FREQ_HOMREF=0.9999029973809292;N_BI_GENOS=10309;East_Han_AN=6554;East_Han_AC=1;East_Han_AF=0
chr1	75600320	.	A	<INS:ME:LINE1>	.	PASS	SVTYPE=INS:ME:LINE1;END=75600322;SVLEN=72;AN=20618;AC=1;AF=4.850130953535746e-05;N_HET=1;N_HOMALT=0;N_HOMREF=10308;FREQ_HET=9.700261907071492e-05;FREQ_HOMALT=0.0;FREQ_HOMREF=0.9999029973809292;N_BI_GENOS=10309;North_Han_AN=2272;North_Han_AC=1;North_Han_AF
chr1	76526500	.	C	<INS:ME:LINE1>	.	PASS	SVTYPE=INS:ME:LINE1;END=76526514;SVLEN=5998;AN=20618;AC=1;AF=4.850130953535746e-05;N_HET=1;N_HOMALT=0;N_HOMREF=10308;FREQ_HET=9.700261907071492e-05;FREQ_HOMALT=0.0;FREQ_HOMREF=0.9999029973809292;N_BI_GENOS=10309;East_Han_AN=6554;East_Han_AC=1;East_Han_AF=
chr1	76629389	.	A	<INS:ME:LINE1>	.	PASS	SVTYPE=INS:ME:LINE1;END=76629404;SVLEN=483;AN=20618;AC=1;AF=4.850130953535746e-05;N_HET=1;N_HOMALT=0;N_HOMREF=10308;FREQ_HET=9.700261907071492e-05;FREQ_HOMALT=0.0;FREQ_HOMREF=0.9999029973809292;N_BI_GENOS=10309;Tibetan_AN=414;Tibetan_AC=1;Tibetan_AF=0.002
chr1	76768904	.	A	<INS:ME:LINE1>	.	PASS	SVTYPE=INS:ME:LINE1;END=76768904;SVLEN=5640;AN=20618;AC=1;AF=4.850130953535746e-05;N_HET=1;N_HOMALT=0;N_HOMREF=10308;FREQ_HET=9.700261907071492e-05;FREQ_HOMALT=0.0;FREQ_HOMREF=0.9999029973809292;N_BI_GENOS=10309;East_Han_AN=6554;East_Han_AC=1;East_Han_AF=
chr1	77102675	.	T	<INS:ME:LINE1>	.	PASS	SVTYPE=INS:ME:LINE1;END=77102675;SVLEN=3204;AN=20618;AC=1;AF=4.850130953535746e-05;N_HET=1;N_HOMALT=0;N_HOMREF=10308;FREQ_HET=9.700261907071492e-05;FREQ_HOMALT=0.0;FREQ_HOMREF=0.9999029973809292;N_BI_GENOS=10309;North_Han_AN=2272;North_Han_AC=1;North_Han_
chr1	77452831	.	A	<INS:ME:LINE1>	.	PASS	SVTYPE=INS:ME:LINE1;END=77452846;SVLEN=4077;AN=20618;AC=100;AF=0.004850130953535745;N_HET=98;N_HOMALT=1;N_HOMREF=10210;FREQ_HET=0.009506256668930062;FREQ_HOMALT=9.700261907071492e-05;FREQ_HOMREF=0.9903967407119992;N_BI_GENOS=10309;South_Han_AN=1496;South_
chr1	77604354	.	A	<INS:ME:LINE1>	.	PASS	SVTYPE=INS:ME:LINE1;END=77604368;SVLEN=1573;AN=20618;AC=1;AF=4.850130953535746e-05;N_HET=1;N_HOMALT=0;N_HOMREF=10308;FREQ_HET=9.700261907071492e-05;FREQ_HOMALT=0.0;FREQ_HOMREF=0.9999029973809292;N_BI_GENOS=10309;Southeast_Han_AN=1908;Southeast_Han_AC=1;So
chr1	78141369	.	T	<INS:ME:LINE1>	.	PASS	SVTYPE=INS:ME:LINE1;END=78141369;SVLEN=321;AN=20618;AC=1;AF=4.850130953535746e-05;N_HET=1;N_HOMALT=0;N_HOMREF=10308;FREQ_HET=9.700261907071492e-05;FREQ_HOMALT=0.0;FREQ_HOMREF=0.9999029973809292;N_BI_GENOS=10309;Yi_AN=422;Yi_AC=1;Yi_AF=0.002369668246445498
chr1	78297825	.	G	<INS:ME:LINE1>	.	PASS	SVTYPE=INS:ME:LINE1;END=78297843;SVLEN=6001;AN=20618;AC=1;AF=4.850130953535746e-05;N_HET=1;N_HOMALT=0;N_HOMREF=10308;FREQ_HET=9.700261907071492e-05;FREQ_HOMALT=0.0;FREQ_HOMREF=0.9999029973809292;N_BI_GENOS=10309;North_Han_AN=2272;North_Han_AC=1;North_Han_
chr1	78365920	.	A	<INS:ME:LINE1>	.	PASS	SVTYPE=INS:ME:LINE1;END=78365932;SVLEN=848;AN=20618;AC=1;AF=4.850130953535746e-05;N_HET=1;N_HOMALT=0;N_HOMREF=10308;FREQ_HET=9.700261907071492e-05;FREQ_HOMALT=0.0;FREQ_HOMREF=0.9999029973809292;N_BI_GENOS=10309;Lingnan_Han_AN=1888;Lingnan_Han_AC=1;Lingnan
chr1	78401829	.	C	<INS:ME:LINE1>	.	PASS	SVTYPE=INS:ME:LINE1;END=78401842;SVLEN=5998;AN=20618;AC=1;AF=4.850130953535746e-05;N_HET=1;N_HOMALT=0;N_HOMREF=10308;FREQ_HET=9.700261907071492e-05;FREQ_HOMALT=0.0;FREQ_HOMREF=0.9999029973809292;N_BI_GENOS=10309;South_Han_AN=1496;South_Han_AC=1;South_Han_
chr1	78426733	.	A	<INS:ME:LINE1>	.	PASS	SVTYPE=INS:ME:LINE1;END=78426749;SVLEN=1880;AN=20618;AC=4;AF=0.00019400523814142983;N_HET=4;N_HOMALT=0;N_HOMREF=10305;FREQ_HET=0.00038801047628285966;FREQ_HOMALT=0.0;FREQ_HOMREF=0.9996119895237171;N_BI_GENOS=10309;East_Han_AN=6554;East_Han_AC=4;East_Han_A
chr1	78545797	.	A	<INS:ME:LINE1>	.	PASS	SVTYPE=INS:ME:LINE1;END=78545811;SVLEN=6014;AN=20618;AC=1;AF=4.850130953535746e-05;N_HET=1;N_HOMALT=0;N_HOMREF=10308;FREQ_HET=9.700261907071492e-05;FREQ_HOMALT=0.0;FREQ_HOMREF=0.9999029973809292;N_BI_GENOS=10309;Northwest_Han_AN=1296;Northwest_Han_AC=1;No
chr1	78558467	.	A	<INS:ME:LINE1>	.	PASS	SVTYPE=INS:ME:LINE1;END=78558484;SVLEN=1048;AN=20618;AC=3;AF=0.00014550392860607236;N_HET=1;N_HOMALT=1;N_HOMREF=10307;FREQ_HET=9.700261907071492e-05;FREQ_HOMALT=9.700261907071492e-05;FREQ_HOMREF=0.9998059947618586;N_BI_GENOS=10309;East_Han_AN=6554;East_Ha
chr1	78571068	.	C	<INS:ME:LINE1>	.	PASS	SVTYPE=INS:ME:LINE1;END=78571082;SVLEN=85;AN=20618;AC=17;AF=0.0008245222621010767;N_HET=17;N_HOMALT=0;N_HOMREF=10292;FREQ_HET=0.0016490445242021534;FREQ_HOMALT=0.0;FREQ_HOMREF=0.9983509554757979;N_BI_GENOS=10309;South_Han_AN=1496;South_Han_AC=1;South_Han_
chr1	78634142	.	A	<INS:ME:LINE1>	.	PASS	SVTYPE=INS:ME:LINE1;END=78634149;SVLEN=807;AN=20618;AC=25;AF=0.0012125327383839363;N_HET=23;N_HOMALT=1;N_HOMREF=10285;FREQ_HET=0.002231060238626443;FREQ_HOMALT=9.700261907071492e-05;FREQ_HOMREF=0.9976719371423028;N_BI_GENOS=10309;Northwest_Han_AN=1296;Nor
chr1	78634339	.	A	<INS:ME:LINE1>	.	PASS	SVTYPE=INS:ME:LINE1;END=78634347;SVLEN=158;AN=20618;AC=2;AF=9.700261907071492e-05;N_HET=2;N_HOMALT=0;N_HOMREF=10307;FREQ_HET=0.00019400523814142983;FREQ_HOMALT=0.0;FREQ_HOMREF=0.9998059947618586;N_BI_GENOS=10309;Lingnan_Han_AN=1888;Lingnan_Han_AC=1;Lingna
chr1	78743994	.	G	<INS:ME:LINE1>	.	PASS	SVTYPE=INS:ME:LINE1;END=78744011;SVLEN=1265;AN=20618;AC=1;AF=4.850130953535746e-05;N_HET=1;N_HOMALT=0;N_HOMREF=10308;FREQ_HET=9.700261907071492e-05;FREQ_HOMALT=0.0;FREQ_HOMREF=0.9999029973809292;N_BI_GENOS=10309;Lingnan_Han_AN=1888;Lingnan_Han_AC=1;Lingna
chr1	78780212	.	T	<INS:ME:LINE1>	.	PASS	SVTYPE=INS:ME:LINE1;END=78780226;SVLEN=6021;AN=20618;AC=1;AF=4.850130953535746e-05;N_HET=1;N_HOMALT=0;N_HOMREF=10308;FREQ_HET=9.700261907071492e-05;FREQ_HOMALT=0.0;FREQ_HOMREF=0.9999029973809292;N_BI_GENOS=10309;Southeast_Han_AN=1908;Southeast_Han_AC=1;So
chr1	78785159	.	A	<INS:ME:LINE1>	.	PASS	SVTYPE=INS:ME:LINE1;END=78785167;SVLEN=526;AN=20618;AC=1;AF=4.850130953535746e-05;N_HET=1;N_HOMALT=0;N_HOMREF=10308;FREQ_HET=9.700261907071492e-05;FREQ_HOMALT=0.0;FREQ_HOMREF=0.9999029973809292;N_BI_GENOS=10309;East_Han_AN=6554;East_Han_AC=1;East_Han_AF=0
chr1	78785563	.	G	<INS:ME:LINE1>	.	PASS	SVTYPE=INS:ME:LINE1;END=78785636;SVLEN=805;AN=20618;AC=1;AF=4.850130953535746e-05;N_HET=1;N_HOMALT=0;N_HOMREF=10308;FREQ_HET=9.700261907071492e-05;FREQ_HOMALT=0.0;FREQ_HOMREF=0.9999029973809292;N_BI_GENOS=10309;East_Han_AN=6554;East_Han_AC=1;East_Han_AF=0
chr1	78959728	.	A	<INS:ME:LINE1>	.	PASS	SVTYPE=INS:ME:LINE1;END=78959730;SVLEN=277;AN=20618;AC=1;AF=4.850130953535746e-05;N_HET=1;N_HOMALT=0;N_HOMREF=10308;FREQ_HET=9.700261907071492e-05;FREQ_HOMALT=0.0;FREQ_HOMREF=0.9999029973809292;N_BI_GENOS=10309;Zhuang_AN=444;Zhuang_AC=1;Zhuang_AF=0.002252
chr1	78990267	.	T	<INS:ME:LINE1>	.	PASS	SVTYPE=INS:ME:LINE1;END=78990282;SVLEN=840;AN=20618;AC=2;AF=9.700261907071492e-05;N_HET=2;N_HOMALT=0;N_HOMREF=10307;FREQ_HET=0.00019400523814142983;FREQ_HOMALT=0.0;FREQ_HOMREF=0.9998059947618586;N_BI_GENOS=10309;Lingnan_Han_AN=1888;Lingnan_Han_AC=1;Lingna
chr1	79060576	.	G	<INS:ME:LINE1>	.	PASS	SVTYPE=INS:ME:LINE1;END=79060599;SVLEN=1771;AN=20618;AC=1;AF=4.850130953535746e-05;N_HET=1;N_HOMALT=0;N_HOMREF=10308;FREQ_HET=9.700261907071492e-05;FREQ_HOMALT=0.0;FREQ_HOMREF=0.9999029973809292;N_BI_GENOS=10309;East_Han_AN=6554;East_Han_AC=1;East_Han_AF=
chr1	79531630	.	G	<INS:ME:LINE1>	.	PASS	SVTYPE=INS:ME:LINE1;END=79531640;SVLEN=6006;AN=20618;AC=3;AF=0.00014550392860607236;N_HET=3;N_HOMALT=0;N_HOMREF=10306;FREQ_HET=0.0002910078572121447;FREQ_HOMALT=0.0;FREQ_HOMREF=0.9997089921427879;N_BI_GENOS=10309;Lingnan_Han_AN=1888;Lingnan_Han_AC=1;Lingn
chr1	79779625	.	G	<INS:ME:LINE1>	.	PASS	SVTYPE=INS:ME:LINE1;END=79779634;SVLEN=6007;AN=20618;AC=1;AF=4.850130953535746e-05;N_HET=1;N_HOMALT=0;N_HOMREF=10308;FREQ_HET=9.700261907071492e-05;FREQ_HOMALT=0.0;FREQ_HOMREF=0.9999029973809292;N_BI_GENOS=10309;Lingnan_Han_AN=1888;Lingnan_Han_AC=1;Lingna
chr1	79928663	.	G	<INS:ME:LINE1>	.	PASS	SVTYPE=INS:ME:LINE1;END=79928663;SVLEN=240;AN=20618;AC=1;AF=4.850130953535746e-05;N_HET=1;N_HOMALT=0;N_HOMREF=10308;FREQ_HET=9.700261907071492e-05;FREQ_HOMALT=0.0;FREQ_HOMREF=0.9999029973809292;N_BI_GENOS=10309;North_Han_AN=2272;North_Han_AC=1;North_Han_A
chr1	79954146	.	T	<INS:ME:LINE1>	.	PASS	SVTYPE=INS:ME:LINE1;END=79954146;SVLEN=0;AN=20618;AC=1;AF=4.850130953535746e-05;N_HET=1;N_HOMALT=0;N_HOMREF=10308;FREQ_HET=9.700261907071492e-05;FREQ_HOMALT=0.0;FREQ_HOMREF=0.9999029973809292;N_BI_GENOS=10309;Northwest_Han_AN=1296;Northwest_Han_AC=1;North
chr1	80332809	.	A	<INS:ME:LINE1>	.	PASS	SVTYPE=INS:ME:LINE1;END=80332809;SVLEN=883;AN=20618;AC=2;AF=9.700261907071492e-05;N_HET=2;N_HOMALT=0;N_HOMREF=10307;FREQ_HET=0.00019400523814142983;FREQ_HOMALT=0.0;FREQ_HOMREF=0.9998059947618586;N_BI_GENOS=10309;Lingnan_Han_AN=1888;Lingnan_Han_AC=1;Lingna
chr1	81212983	.	A	<INS:ME:LINE1>	.	PASS	SVTYPE=INS:ME:LINE1;END=81212999;SVLEN=811;AN=20618;AC=9;AF=0.0004365117858182171;N_HET=9;N_HOMALT=0;N_HOMREF=10300;FREQ_HET=0.0008730235716364342;FREQ_HOMALT=0.0;FREQ_HOMREF=0.9991269764283636;N_BI_GENOS=10309;Northwest_Han_AN=1296;Northwest_Han_AC=2;Nor
chr1	81351232	.	T	<INS:ME:LINE1>	.	PASS	SVTYPE=INS:ME:LINE1;END=81351247;SVLEN=6017;AN=20618;AC=3;AF=0.00014550392860607236;N_HET=3;N_HOMALT=0;N_HOMREF=10306;FREQ_HET=0.0002910078572121447;FREQ_HOMALT=0.0;FREQ_HOMREF=0.9997089921427879;N_BI_GENOS=10309;North_Han_AN=2272;North_Han_AC=1;North_Han
chr1	81390097	.	A	<INS:ME:LINE1>	.	PASS	SVTYPE=INS:ME:LINE1;END=81390121;SVLEN=115;AN=20618;AC=51;AF=0.0024735667863032304;N_HET=51;N_HOMALT=0;N_HOMREF=10258;FREQ_HET=0.004947133572606461;FREQ_HOMALT=0.0;FREQ_HOMREF=0.9950528664273935;N_BI_GENOS=10309;South_Han_AN=1496;South_Han_AC=4;South_Han_
chr1	81416836	.	G	<INS:ME:LINE1>	.	PASS	SVTYPE=INS:ME:LINE1;END=81416853;SVLEN=798;AN=20618;AC=3;AF=0.00014550392860607236;N_HET=3;N_HOMALT=0;N_HOMREF=10306;FREQ_HET=0.0002910078572121447;FREQ_HOMALT=0.0;FREQ_HOMREF=0.9997089921427879;N_BI_GENOS=10309;South_Han_AN=1496;South_Han_AC=1;South_Han_
chr1	81593855	.	A	<INS:ME:LINE1>	.	PASS	SVTYPE=INS:ME:LINE1;END=81593873;SVLEN=2472;AN=20618;AC=1;AF=4.850130953535746e-05;N_HET=1;N_HOMALT=0;N_HOMREF=10308;FREQ_HET=9.700261907071492e-05;FREQ_HOMALT=0.0;FREQ_HOMREF=0.9999029973809292;N_BI_GENOS=10309;East_Han_AN=6554;East_Han_AC=1;East_Han_AF=
chr1	81710948	.	T	<INS:ME:LINE1>	.	PASS	SVTYPE=INS:ME:LINE1;END=81710961;SVLEN=6004;AN=20618;AC=1;AF=4.850130953535746e-05;N_HET=1;N_HOMALT=0;N_HOMREF=10308;FREQ_HET=9.700261907071492e-05;FREQ_HOMALT=0.0;FREQ_HOMREF=0.9999029973809292;N_BI_GENOS=10309;North_Han_AN=2272;North_Han_AC=1;North_Han_
chr1	82658863	.	A	<INS:ME:LINE1>	.	PASS	SVTYPE=INS:ME:LINE1;END=82658869;SVLEN=767;AN=20618;AC=1;AF=4.850130953535746e-05;N_HET=1;N_HOMALT=0;N_HOMREF=10308;FREQ_HET=9.700261907071492e-05;FREQ_HOMALT=0.0;FREQ_HOMREF=0.9999029973809292;N_BI_GENOS=10309;Lingnan_Han_AN=1888;Lingnan_Han_AC=1;Lingnan
chr1	83144051	.	A	<INS:ME:LINE1>	.	PASS	SVTYPE=INS:ME:LINE1;END=83144055;SVLEN=1707;AN=20618;AC=3071;AF=0.14894752158308275;N_HET=2009;N_HOMALT=531;N_HOMREF=7769;FREQ_HET=0.19487826171306624;FREQ_HOMALT=0.05150839072654962;FREQ_HOMREF=0.7536133475603841;N_BI_GENOS=10309;South_Han_AN=1496;South_
chr1	83238143	.	A	<INS:ME:LINE1>	.	PASS	SVTYPE=INS:ME:LINE1;END=83238144;SVLEN=273;AN=20618;AC=1;AF=4.850130953535746e-05;N_HET=1;N_HOMALT=0;N_HOMREF=10308;FREQ_HET=9.700261907071492e-05;FREQ_HOMALT=0.0;FREQ_HOMREF=0.9999029973809292;N_BI_GENOS=10309;Mongolian_AN=454;Mongolian_AC=1;Mongolian_AF
chr1	84145026	.	A	<INS:ME:LINE1>	.	PASS	SVTYPE=INS:ME:LINE1;END=84145032;SVLEN=187;AN=20618;AC=1;AF=4.850130953535746e-05;N_HET=1;N_HOMALT=0;N_HOMREF=10308;FREQ_HET=9.700261907071492e-05;FREQ_HOMALT=0.0;FREQ_HOMREF=0.9999029973809292;N_BI_GENOS=10309;Central_Han_AN=2128;Central_Han_AC=1;Central
chr1	84368306	.	T	<INS:ME:LINE1>	.	PASS	SVTYPE=INS:ME:LINE1;END=84368306;SVLEN=42;AN=20618;AC=2;AF=9.700261907071492e-05;N_HET=2;N_HOMALT=0;N_HOMREF=10307;FREQ_HET=0.00019400523814142983;FREQ_HOMALT=0.0;FREQ_HOMREF=0.9998059947618586;N_BI_GENOS=10309;Mongolian_AN=454;Mongolian_AC=1;Mongolian_AF
chr1	85133033	.	C	<INS:ME:LINE1>	.	PASS	SVTYPE=INS:ME:LINE1;END=85133049;SVLEN=6000;AN=20618;AC=1;AF=4.850130953535746e-05;N_HET=1;N_HOMALT=0;N_HOMREF=10308;FREQ_HET=9.700261907071492e-05;FREQ_HOMALT=0.0;FREQ_HOMREF=0.9999029973809292;N_BI_GENOS=10309;Southeast_Han_AN=1908;Southeast_Han_AC=1;So
chr1	85344821	.	G	<INS:ME:LINE1>	.	PASS	SVTYPE=INS:ME:LINE1;END=85344828;SVLEN=2764;AN=20618;AC=2;AF=9.700261907071492e-05;N_HET=2;N_HOMALT=0;N_HOMREF=10307;FREQ_HET=0.00019400523814142983;FREQ_HOMALT=0.0;FREQ_HOMREF=0.9998059947618586;N_BI_GENOS=10309;South_Han_AN=1496;South_Han_AC=2;South_Han
chr1	85743369	.	T	<INS:ME:LINE1>	.	PASS	SVTYPE=INS:ME:LINE1;END=85743386;SVLEN=1275;AN=20618;AC=1;AF=4.850130953535746e-05;N_HET=1;N_HOMALT=0;N_HOMREF=10308;FREQ_HET=9.700261907071492e-05;FREQ_HOMALT=0.0;FREQ_HOMREF=0.9999029973809292;N_BI_GENOS=10309;Zhuang_AN=444;Zhuang_AC=1;Zhuang_AF=0.00225
chr1	85761907	.	A	<INS:ME:LINE1>	.	PASS	SVTYPE=INS:ME:LINE1;END=85761922;SVLEN=5997;AN=20618;AC=1;AF=4.850130953535746e-05;N_HET=1;N_HOMALT=0;N_HOMREF=10308;FREQ_HET=9.700261907071492e-05;FREQ_HOMALT=0.0;FREQ_HOMREF=0.9999029973809292;N_BI_GENOS=10309;Central_Han_AN=2128;Central_Han_AC=1;Centra
chr1	86009592	.	A	<INS:ME:LINE1>	.	PASS	SVTYPE=INS:ME:LINE1;END=86009609;SVLEN=1181;AN=20618;AC=92;AF=0.004462120477252886;N_HET=90;N_HOMALT=1;N_HOMREF=10218;FREQ_HET=0.008730235716364342;FREQ_HOMALT=9.700261907071492e-05;FREQ_HOMREF=0.9911727616645649;N_BI_GENOS=10309;South_Han_AN=1496;South_H
chr1	86128561	.	A	<INS:ME:LINE1>	.	PASS	SVTYPE=INS:ME:LINE1;END=86128575;SVLEN=616;AN=20618;AC=1;AF=4.850130953535746e-05;N_HET=1;N_HOMALT=0;N_HOMREF=10308;FREQ_HET=9.700261907071492e-05;FREQ_HOMALT=0.0;FREQ_HOMREF=0.9999029973809292;N_BI_GENOS=10309;Central_Han_AN=2128;Central_Han_AC=1;Central
chr1	86198125	.	A	<INS:ME:LINE1>	.	PASS	SVTYPE=INS:ME:LINE1;END=86198137;SVLEN=6001;AN=20618;AC=1;AF=4.850130953535746e-05;N_HET=1;N_HOMALT=0;N_HOMREF=10308;FREQ_HET=9.700261907071492e-05;FREQ_HOMALT=0.0;FREQ_HOMREF=0.9999029973809292;N_BI_GENOS=10309;Tibetan_AN=414;Tibetan_AC=1;Tibetan_AF=0.00
chr1	86692916	.	G	<INS:ME:LINE1>	.	PASS	SVTYPE=INS:ME:LINE1;END=86692929;SVLEN=942;AN=20618;AC=1;AF=4.850130953535746e-05;N_HET=1;N_HOMALT=0;N_HOMREF=10308;FREQ_HET=9.700261907071492e-05;FREQ_HOMALT=0.0;FREQ_HOMREF=0.9999029973809292;N_BI_GENOS=10309;Lingnan_Han_AN=1888;Lingnan_Han_AC=1;Lingnan
chr1	86727360	.	G	<INS:ME:LINE1>	.	PASS	SVTYPE=INS:ME:LINE1;END=86727375;SVLEN=703;AN=20618;AC=1;AF=4.850130953535746e-05;N_HET=1;N_HOMALT=0;N_HOMREF=10308;FREQ_HET=9.700261907071492e-05;FREQ_HOMALT=0.0;FREQ_HOMREF=0.9999029973809292;N_BI_GENOS=10309;Manchu_AN=416;Manchu_AC=1;Manchu_AF=0.002403
chr1	86761536	.	A	<INS:ME:LINE1>	.	PASS	SVTYPE=INS:ME:LINE1;END=86761536;SVLEN=137;AN=20618;AC=1;AF=4.850130953535746e-05;N_HET=1;N_HOMALT=0;N_HOMREF=10308;FREQ_HET=9.700261907071492e-05;FREQ_HOMALT=0.0;FREQ_HOMREF=0.9999029973809292;N_BI_GENOS=10309;Lingnan_Han_AN=1888;Lingnan_Han_AC=1;Lingnan
chr1	87080074	.	A	<INS:ME:LINE1>	.	PASS	SVTYPE=INS:ME:LINE1;END=87080085;SVLEN=1792;AN=20618;AC=1;AF=4.850130953535746e-05;N_HET=1;N_HOMALT=0;N_HOMREF=10308;FREQ_HET=9.700261907071492e-05;FREQ_HOMALT=0.0;FREQ_HOMREF=0.9999029973809292;N_BI_GENOS=10309;Tibetan_AN=414;Tibetan_AC=1;Tibetan_AF=0.00
chr1	87101066	.	C	<INS:ME:LINE1>	.	PASS	SVTYPE=INS:ME:LINE1;END=87101079;SVLEN=847;AN=20618;AC=2;AF=9.700261907071492e-05;N_HET=0;N_HOMALT=1;N_HOMREF=10308;FREQ_HET=0.0;FREQ_HOMALT=9.700261907071492e-05;FREQ_HOMREF=0.9999029973809292;N_BI_GENOS=10309;East_Han_AN=6554;East_Han_AC=2;East_Han_AF=0
chr1	87131569	.	A	<INS:ME:LINE1>	.	PASS	SVTYPE=INS:ME:LINE1;END=87131579;SVLEN=3643;AN=20618;AC=1;AF=4.850130953535746e-05;N_HET=1;N_HOMALT=0;N_HOMREF=10308;FREQ_HET=9.700261907071492e-05;FREQ_HOMALT=0.0;FREQ_HOMREF=0.9999029973809292;N_BI_GENOS=10309;Central_Han_AN=2128;Central_Han_AC=1;Centra
chr1	87176386	.	G	<INS:ME:LINE1>	.	PASS	SVTYPE=INS:ME:LINE1;END=87176386;SVLEN=5997;AN=20618;AC=1;AF=4.850130953535746e-05;N_HET=1;N_HOMALT=0;N_HOMREF=10308;FREQ_HET=9.700261907071492e-05;FREQ_HOMALT=0.0;FREQ_HOMREF=0.9999029973809292;N_BI_GENOS=10309;Tibetan_AN=414;Tibetan_AC=1;Tibetan_AF=0.00
chr1	87380442	.	G	<INS:ME:LINE1>	.	PASS	SVTYPE=INS:ME:LINE1;END=87380457;SVLEN=6004;AN=20618;AC=1;AF=4.850130953535746e-05;N_HET=1;N_HOMALT=0;N_HOMREF=10308;FREQ_HET=9.700261907071492e-05;FREQ_HOMALT=0.0;FREQ_HOMREF=0.9999029973809292;N_BI_GENOS=10309;South_Han_AN=1496;South_Han_AC=1;South_Han_
chr1	87563222	.	T	<INS:ME:LINE1>	.	PASS	SVTYPE=INS:ME:LINE1;END=87563222;SVLEN=2292;AN=20618;AC=2;AF=9.700261907071492e-05;N_HET=2;N_HOMALT=0;N_HOMREF=10307;FREQ_HET=0.00019400523814142983;FREQ_HOMALT=0.0;FREQ_HOMREF=0.9998059947618586;N_BI_GENOS=10309;Central_Han_AN=2128;Central_Han_AC=2;Centr
chr1	87903646	.	G	<INS:ME:LINE1>	.	PASS	SVTYPE=INS:ME:LINE1;END=87903655;SVLEN=5164;AN=20618;AC=2;AF=9.700261907071492e-05;N_HET=2;N_HOMALT=0;N_HOMREF=10307;FREQ_HET=0.00019400523814142983;FREQ_HOMALT=0.0;FREQ_HOMREF=0.9998059947618586;N_BI_GENOS=10309;East_Han_AN=6554;East_Han_AC=2;East_Han_AF
chr1	87928098	.	A	<INS:ME:LINE1>	.	PASS	SVTYPE=INS:ME:LINE1;END=87928108;SVLEN=829;AN=20618;AC=1;AF=4.850130953535746e-05;N_HET=1;N_HOMALT=0;N_HOMREF=10308;FREQ_HET=9.700261907071492e-05;FREQ_HOMALT=0.0;FREQ_HOMREF=0.9999029973809292;N_BI_GENOS=10309;Yi_AN=422;Yi_AC=1;Yi_AF=0.002369668246445498
chr1	87979848	.	G	<INS:ME:LINE1>	.	PASS	SVTYPE=INS:ME:LINE1;END=87979862;SVLEN=1102;AN=20618;AC=1;AF=4.850130953535746e-05;N_HET=1;N_HOMALT=0;N_HOMREF=10308;FREQ_HET=9.700261907071492e-05;FREQ_HOMALT=0.0;FREQ_HOMREF=0.9999029973809292;N_BI_GENOS=10309;Lingnan_Han_AN=1888;Lingnan_Han_AC=1;Lingna
chr1	88064151	.	A	<INS:ME:LINE1>	.	PASS	SVTYPE=INS:ME:LINE1;END=88064168;SVLEN=1102;AN=20618;AC=2;AF=9.700261907071492e-05;N_HET=2;N_HOMALT=0;N_HOMREF=10307;FREQ_HET=0.00019400523814142983;FREQ_HOMALT=0.0;FREQ_HOMREF=0.9998059947618586;N_BI_GENOS=10309;Southeast_Han_AN=1908;Southeast_Han_AC=2;S
chr1	88237253	.	C	<INS:ME:LINE1>	.	PASS	SVTYPE=INS:ME:LINE1;END=88237266;SVLEN=608;AN=20618;AC=3;AF=0.00014550392860607236;N_HET=3;N_HOMALT=0;N_HOMREF=10306;FREQ_HET=0.0002910078572121447;FREQ_HOMALT=0.0;FREQ_HOMREF=0.9997089921427879;N_BI_GENOS=10309;Central_Han_AN=2128;Central_Han_AC=3;Centra
chr1	88372260	.	A	<INS:ME:LINE1>	.	PASS	SVTYPE=INS:ME:LINE1;END=88372275;SVLEN=277;AN=20618;AC=1;AF=4.850130953535746e-05;N_HET=1;N_HOMALT=0;N_HOMREF=10308;FREQ_HET=9.700261907071492e-05;FREQ_HOMALT=0.0;FREQ_HOMREF=0.9999029973809292;N_BI_GENOS=10309;Northwest_Han_AN=1296;Northwest_Han_AC=1;Nor
chr1	88434487	.	A	<INS:ME:LINE1>	.	PASS	SVTYPE=INS:ME:LINE1;END=88434501;SVLEN=6005;AN=20618;AC=2;AF=9.700261907071492e-05;N_HET=0;N_HOMALT=1;N_HOMREF=10308;FREQ_HET=0.0;FREQ_HOMALT=9.700261907071492e-05;FREQ_HOMREF=0.9999029973809292;N_BI_GENOS=10309;Central_Han_AN=2128;Central_Han_AC=2;Centra
chr1	88686823	.	A	<INS:ME:LINE1>	.	PASS	SVTYPE=INS:ME:LINE1;END=88686838;SVLEN=342;AN=20618;AC=1;AF=4.850130953535746e-05;N_HET=1;N_HOMALT=0;N_HOMREF=10308;FREQ_HET=9.700261907071492e-05;FREQ_HOMALT=0.0;FREQ_HOMREF=0.9999029973809292;N_BI_GENOS=10309;South_Han_AN=1496;South_Han_AC=1;South_Han_A
chr1	88815826	.	C	<INS:ME:LINE1>	.	PASS	SVTYPE=INS:ME:LINE1;END=88815841;SVLEN=6005;AN=20618;AC=1;AF=4.850130953535746e-05;N_HET=1;N_HOMALT=0;N_HOMREF=10308;FREQ_HET=9.700261907071492e-05;FREQ_HOMALT=0.0;FREQ_HOMREF=0.9999029973809292;N_BI_GENOS=10309;Central_Han_AN=2128;Central_Han_AC=1;Centra
chr1	88997890	.	A	<INS:ME:LINE1>	.	PASS	SVTYPE=INS:ME:LINE1;END=88997899;SVLEN=2924;AN=20618;AC=1;AF=4.850130953535746e-05;N_HET=1;N_HOMALT=0;N_HOMREF=10308;FREQ_HET=9.700261907071492e-05;FREQ_HOMALT=0.0;FREQ_HOMREF=0.9999029973809292;N_BI_GENOS=10309;North_Han_AN=2272;North_Han_AC=1;North_Han_
chr1	89060002	.	T	<INS:ME:LINE1>	.	PASS	SVTYPE=INS:ME:LINE1;END=89060018;SVLEN=2352;AN=20618;AC=2;AF=9.700261907071492e-05;N_HET=2;N_HOMALT=0;N_HOMREF=10307;FREQ_HET=0.00019400523814142983;FREQ_HOMALT=0.0;FREQ_HOMREF=0.9998059947618586;N_BI_GENOS=10309;Southeast_Han_AN=1908;Southeast_Han_AC=1;S
chr1	89087580	.	C	<INS:ME:LINE1>	.	PASS	SVTYPE=INS:ME:LINE1;END=89087596;SVLEN=5998;AN=20618;AC=2;AF=9.700261907071492e-05;N_HET=2;N_HOMALT=0;N_HOMREF=10307;FREQ_HET=0.00019400523814142983;FREQ_HOMALT=0.0;FREQ_HOMREF=0.9998059947618586;N_BI_GENOS=10309;East_Han_AN=6554;East_Han_AC=1;East_Han_AF
chr1	89137326	.	T	<INS:ME:LINE1>	.	PASS	SVTYPE=INS:ME:LINE1;END=89137326;SVLEN=2451;AN=20618;AC=1;AF=4.850130953535746e-05;N_HET=1;N_HOMALT=0;N_HOMREF=10308;FREQ_HET=9.700261907071492e-05;FREQ_HOMALT=0.0;FREQ_HOMREF=0.9999029973809292;N_BI_GENOS=10309;East_Han_AN=6554;East_Han_AC=1;East_Han_AF=
chr1	89138527	.	A	<INS:ME:LINE1>	.	PASS	SVTYPE=INS:ME:LINE1;END=89138527;SVLEN=660;AN=20618;AC=1;AF=4.850130953535746e-05;N_HET=1;N_HOMALT=0;N_HOMREF=10308;FREQ_HET=9.700261907071492e-05;FREQ_HOMALT=0.0;FREQ_HOMREF=0.9999029973809292;N_BI_GENOS=10309;East_Han_AN=6554;East_Han_AC=1;East_Han_AF=0
chr1	89188417	.	A	<INS:ME:LINE1>	.	PASS	SVTYPE=INS:ME:LINE1;END=89188429;SVLEN=123;AN=20618;AC=2;AF=9.700261907071492e-05;N_HET=2;N_HOMALT=0;N_HOMREF=10307;FREQ_HET=0.00019400523814142983;FREQ_HOMALT=0.0;FREQ_HOMREF=0.9998059947618586;N_BI_GENOS=10309;South_Han_AN=1496;South_Han_AC=1;South_Han_
chr1	89190989	.	A	<INS:ME:LINE1>	.	PASS	SVTYPE=INS:ME:LINE1;END=89190999;SVLEN=6003;AN=20618;AC=6;AF=0.0002910078572121447;N_HET=6;N_HOMALT=0;N_HOMREF=10303;FREQ_HET=0.0005820157144242894;FREQ_HOMALT=0.0;FREQ_HOMREF=0.9994179842855757;N_BI_GENOS=10309;South_Han_AN=1496;South_Han_AC=1;South_Han_
chr1	89198919	.	A	<INS:ME:LINE1>	.	PASS	SVTYPE=INS:ME:LINE1;END=89198925;SVLEN=207;AN=20618;AC=1;AF=4.850130953535746e-05;N_HET=1;N_HOMALT=0;N_HOMREF=10308;FREQ_HET=9.700261907071492e-05;FREQ_HOMALT=0.0;FREQ_HOMREF=0.9999029973809292;N_BI_GENOS=10309;East_Han_AN=6554;East_Han_AC=1;East_Han_AF=0
chr1	89265768	.	T	<INS:ME:LINE1>	.	PASS	SVTYPE=INS:ME:LINE1;END=89265785;SVLEN=4077;AN=20618;AC=4;AF=0.00019400523814142983;N_HET=4;N_HOMALT=0;N_HOMREF=10305;FREQ_HET=0.00038801047628285966;FREQ_HOMALT=0.0;FREQ_HOMREF=0.9996119895237171;N_BI_GENOS=10309;Manchu_AN=416;Manchu_AC=3;Manchu_AF=0.007
chr1	89405494	.	A	<INS:ME:LINE1>	.	PASS	SVTYPE=INS:ME:LINE1;END=89405510;SVLEN=426;AN=20618;AC=2;AF=9.700261907071492e-05;N_HET=0;N_HOMALT=1;N_HOMREF=10308;FREQ_HET=0.0;FREQ_HOMALT=9.700261907071492e-05;FREQ_HOMREF=0.9999029973809292;N_BI_GENOS=10309;North_Han_AN=2272;North_Han_AC=2;North_Han_A
chr1	89424914	.	A	<INS:ME:LINE1>	.	PASS	SVTYPE=INS:ME:LINE1;END=89424927;SVLEN=5998;AN=20618;AC=1;AF=4.850130953535746e-05;N_HET=1;N_HOMALT=0;N_HOMREF=10308;FREQ_HET=9.700261907071492e-05;FREQ_HOMALT=0.0;FREQ_HOMREF=0.9999029973809292;N_BI_GENOS=10309;Central_Han_AN=2128;Central_Han_AC=1;Centra
chr1	89536648	.	A	<INS:ME:LINE1>	.	PASS	SVTYPE=INS:ME:LINE1;END=89536660;SVLEN=5996;AN=20618;AC=1;AF=4.850130953535746e-05;N_HET=1;N_HOMALT=0;N_HOMREF=10308;FREQ_HET=9.700261907071492e-05;FREQ_HOMALT=0.0;FREQ_HOMREF=0.9999029973809292;N_BI_GENOS=10309;East_Han_AN=6554;East_Han_AC=1;East_Han_AF=
chr1	89626638	.	A	<INS:ME:LINE1>	.	PASS	SVTYPE=INS:ME:LINE1;END=89626653;SVLEN=188;AN=20618;AC=1;AF=4.850130953535746e-05;N_HET=1;N_HOMALT=0;N_HOMREF=10308;FREQ_HET=9.700261907071492e-05;FREQ_HOMALT=0.0;FREQ_HOMREF=0.9999029973809292;N_BI_GENOS=10309;North_Han_AN=2272;North_Han_AC=1;North_Han_A
chr1	89692111	.	A	<INS:ME:LINE1>	.	PASS	SVTYPE=INS:ME:LINE1;END=89692127;SVLEN=3672;AN=20618;AC=1;AF=4.850130953535746e-05;N_HET=1;N_HOMALT=0;N_HOMREF=10308;FREQ_HET=9.700261907071492e-05;FREQ_HOMALT=0.0;FREQ_HOMREF=0.9999029973809292;N_BI_GENOS=10309;North_Han_AN=2272;North_Han_AC=1;North_Han_
chr1	89742037	.	C	<INS:ME:LINE1>	.	PASS	SVTYPE=INS:ME:LINE1;END=89742056;SVLEN=3968;AN=20618;AC=1;AF=4.850130953535746e-05;N_HET=1;N_HOMALT=0;N_HOMREF=10308;FREQ_HET=9.700261907071492e-05;FREQ_HOMALT=0.0;FREQ_HOMREF=0.9999029973809292;N_BI_GENOS=10309;Lingnan_Han_AN=1888;Lingnan_Han_AC=1;Lingna

chr1	89843136	.	C	<INS:ME:LINE1>	.	PASS	SVTYPE=INS:ME:LINE1;END=89843147;SVLEN=157;AN=20618;AC=1;AF=4.850130953535746e-05;N_HET=1;N_HOMALT=0;N_HOMREF=10308;FREQ_HET=9.700261907071492e-05;FREQ_HOMALT=0.0;FREQ_HOMREF=0.9999029973809292;N_BI_GENOS=10309;Lingnan_Han_AN=1888;Lingnan_Han_AC=1;Lingnan
chr1	89901451	.	T	<INS:ME:LINE1>	.	PASS	SVTYPE=INS:ME:LINE1;END=89901464;SVLEN=502;AN=20618;AC=1;AF=4.850130953535746e-05;N_HET=1;N_HOMALT=0;N_HOMREF=10308;FREQ_HET=9.700261907071492e-05;FREQ_HOMALT=0.0;FREQ_HOMREF=0.9999029973809292;N_BI_GENOS=10309;North_Han_AN=2272;North_Han_AC=1;North_Han_A
chr1	89973008	.	A	<INS:ME:LINE1>	.	PASS	SVTYPE=INS:ME:LINE1;END=89973023;SVLEN=3167;AN=20618;AC=6;AF=0.0002910078572121447;N_HET=6;N_HOMALT=0;N_HOMREF=10303;FREQ_HET=0.0005820157144242894;FREQ_HOMALT=0.0;FREQ_HOMREF=0.9994179842855757;N_BI_GENOS=10309;Lingnan_Han_AN=1888;Lingnan_Han_AC=1;Lingna
chr1	90263946	.	A	<INS:ME:LINE1>	.	PASS	SVTYPE=INS:ME:LINE1;END=90263959;SVLEN=3128;AN=20618;AC=11;AF=0.0005335144048888932;N_HET=11;N_HOMALT=0;N_HOMREF=10298;FREQ_HET=0.0010670288097777864;FREQ_HOMALT=0.0;FREQ_HOMREF=0.9989329711902222;N_BI_GENOS=10309;Lingnan_Han_AN=1888;Lingnan_Han_AC=1;Lingna
chr1	90416276	.	A	<INS:ME:LINE1>	.	PASS	SVTYPE=INS:ME:LINE1;END=90416276;SVLEN=5976;AN=20618;AC=1;AF=4.850130953535746e-05;N_HET=1;N_HOMALT=0;N_HOMREF=10308;FREQ_HET=9.700261907071492e-05;FREQ_HOMALT=0.0;FREQ_HOMREF=0.9999029973809292;N_BI_GENOS=10309;Central_Han_AN=2128;Central_Han_AC=1;Centra
chr1	90448940	.	A	<INS:ME:LINE1>	.	PASS	SVTYPE=INS:ME:LINE1;END=90448955;SVLEN=321;AN=20618;AC=5346;AF=0.25928800077602093;N_HET=2820;N_HOMALT=1263;N_HOMREF=6226;FREQ_HET=0.27354738577941606;FREQ_HOMALT=0.12251430788631293;FREQ_HOMREF=0.603938306334271;N_BI_GENOS=10309;South_Han_AN=1496;South_H
chr1	90602417	.	A	<INS:ME:LINE1>	.	PASS	SVTYPE=INS:ME:LINE1;END=90602435;SVLEN=1123;AN=20618;AC=1;AF=4.850130953535746e-05;N_HET=1;N_HOMALT=0;N_HOMREF=10308;FREQ_HET=9.700261907071492e-05;FREQ_HOMALT=0.0;FREQ_HOMREF=0.9999029973809292;N_BI_GENOS=10309;Southeast_Han_AN=1908;Southeast_Han_AC=1;So
chr1	90683600	.	A	<INS:ME:LINE1>	.	PASS	SVTYPE=INS:ME:LINE1;END=90683601;SVLEN=362;AN=20618;AC=24;AF=0.0011640314288485789;N_HET=22;N_HOMALT=1;N_HOMREF=10286;FREQ_HET=0.002134057619555728;FREQ_HOMALT=9.700261907071492e-05;FREQ_HOMREF=0.9977689397613736;N_BI_GENOS=10309;South_Han_AN=1496;South_H
chr1	91208771	.	A	<INS:ME:LINE1>	.	PASS	SVTYPE=INS:ME:LINE1;END=91208786;SVLEN=1555;AN=20618;AC=1;AF=4.850130953535746e-05;N_HET=1;N_HOMALT=0;N_HOMREF=10308;FREQ_HET=9.700261907071492e-05;FREQ_HOMALT=0.0;FREQ_HOMREF=0.9999029973809292;N_BI_GENOS=10309;South_Han_AN=1496;South_Han_AC=1;South_Han_
chr1	91217492	.	G	<INS:ME:LINE1>	.	PASS	SVTYPE=INS:ME:LINE1;END=91217502;SVLEN=6032;AN=20618;AC=1;AF=4.850130953535746e-05;N_HET=1;N_HOMALT=0;N_HOMREF=10308;FREQ_HET=9.700261907071492e-05;FREQ_HOMALT=0.0;FREQ_HOMREF=0.9999029973809292;N_BI_GENOS=10309;Tibetan_AN=414;Tibetan_AC=1;Tibetan_AF=0.00
chr1	91235251	.	A	<INS:ME:LINE1>	.	PASS	SVTYPE=INS:ME:LINE1;END=91235265;SVLEN=2000;AN=20618;AC=1;AF=4.850130953535746e-05;N_HET=1;N_HOMALT=0;N_HOMREF=10308;FREQ_HET=9.700261907071492e-05;FREQ_HOMALT=0.0;FREQ_HOMREF=0.9999029973809292;N_BI_GENOS=10309;Central_Han_AN=2128;Central_Han_AC=1;Centra
chr1	91286202	.	T	<INS:ME:LINE1>	.	PASS	SVTYPE=INS:ME:LINE1;END=91286217;SVLEN=889;AN=20618;AC=1;AF=4.850130953535746e-05;N_HET=1;N_HOMALT=0;N_HOMREF=10308;FREQ_HET=9.700261907071492e-05;FREQ_HOMALT=0.0;FREQ_HOMREF=0.9999029973809292;N_BI_GENOS=10309;East_Han_AN=6554;East_Han_AC=1;East_Han_AF=0
chr1	91472146	.	G	<INS:ME:LINE1>	.	PASS	SVTYPE=INS:ME:LINE1;END=91472161;SVLEN=5993;AN=20618;AC=4;AF=0.00019400523814142983;N_HET=4;N_HOMALT=0;N_HOMREF=10305;FREQ_HET=0.0003880104762828566;FREQ_HOMALT=0.0;FREQ_HOMREF=0.9996119895237171;N_BI_GENOS=10309;Lingnan_Han_AN=1888;Lingnan_Han_AC=1;Ling
chr1	91528523	.	A	<INS:ME:LINE1>	.	PASS	SVTYPE=INS:ME:LINE1;END=91528536;SVLEN=6015;AN=20618;AC=1;AF=4.850130953535746e-05;N_HET=1;N_HOMALT=0;N_HOMREF=10308;FREQ_HET=9.700261907071492e-05;FREQ_HOMALT=0.0;FREQ_HOMREF=0.9999029973809292;N_BI_GENOS=10309;South_Han_AN=1496;South_Han_AC=1;South_Han_
chr1	91817156	.	T	<INS:ME:LINE1>	.	PASS	SVTYPE=INS:ME:LINE1;END=91817164;SVLEN=541;AN=20618;AC=1;AF=4.850130953535746e-05;N_HET=1;N_HOMALT=0;N_HOMREF=10308;FREQ_HET=9.700261907071492e-05;FREQ_HOMALT=0.0;FREQ_HOMREF=0.9999029973809292;N_BI_GENOS=10309;Yi_AN=422;Yi_AC=1;Yi_AF=0.002369668246445498
chr1	92191937	.	C	<INS:ME:LINE1>	.	PASS	SVTYPE=INS:ME:LINE1;END=92191939;SVLEN=940;AN=20618;AC=1;AF=4.850130953535746e-05;N_HET=1;N_HOMALT=0;N_HOMREF=10308;FREQ_HET=9.700261907071492e-05;FREQ_HOMALT=0.0;FREQ_HOMREF=0.9999029973809292;N_BI_GENOS=10309;Southeast_Han_AN=1908;Southeast_Han_AC=1;Sou
chr1	92239396	.	A	<INS:ME:LINE1>	.	PASS	SVTYPE=INS:ME:LINE1;END=92239413;SVLEN=1924;AN=20618;AC=1;AF=4.850130953535746e-05;N_HET=1;N_HOMALT=0;N_HOMREF=10308;FREQ_HET=9.700261907071492e-05;FREQ_HOMALT=0.0;FREQ_HOMREF=0.9999029973809292;N_BI_GENOS=10309;East_Han_AN=6554;East_Han_AC=1;East_Han_AF=
chr1	92239834	.	G	<INS:ME:LINE1>	.	PASS	SVTYPE=INS:ME:LINE1;END=92239849;SVLEN=775;AN=20618;AC=1;AF=4.850130953535746e-05;N_HET=1;N_HOMALT=0;N_HOMREF=10308;FREQ_HET=9.700261907071492e-05;FREQ_HOMALT=0.0;FREQ_HOMREF=0.9999029973809292;N_BI_GENOS=10309;East_Han_AN=6554;East_Han_AC=1;East_Han_AF=0
chr1	92508177	.	A	<INS:ME:LINE1>	.	PASS	SVTYPE=INS:ME:LINE1;END=92508178;SVLEN=388;AN=20618;AC=2;AF=9.700261907071492e-05;N_HET=0;N_HOMALT=1;N_HOMREF=10308;FREQ_HET=0.0;FREQ_HOMALT=9.700261907071492e-05;FREQ_HOMREF=0.9999029973809292;N_BI_GENOS=10309;Southeast_Han_AN=1908;Southeast_Han_AC=2;Sou
chr1	92846472	.	A	<INS:ME:LINE1>	.	PASS	SVTYPE=INS:ME:LINE1;END=92846489;SVLEN=395;AN=20618;AC=1;AF=4.850130953535746e-05;N_HET=1;N_HOMALT=0;N_HOMREF=10308;FREQ_HET=9.700261907071492e-05;FREQ_HOMALT=0.0;FREQ_HOMREF=0.9999029973809292;N_BI_GENOS=10309;Lingnan_Han_AN=1888;Lingnan_Han_AC=1;Lingnan
chr1	93116011	.	A	<INS:ME:LINE1>	.	PASS	SVTYPE=INS:ME:LINE1;END=93116019;SVLEN=33;AN=20618;AC=1;AF=4.850130953535746e-05;N_HET=1;N_HOMALT=0;N_HOMREF=10308;FREQ_HET=9.700261907071492e-05;FREQ_HOMALT=0.0;FREQ_HOMREF=0.9999029973809292;N_BI_GENOS=10309;East_Han_AN=6554;East_Han_AC=1;East_Han_AF=0.
chr1	94081644	.	A	<INS:ME:LINE1>	.	PASS	SVTYPE=INS:ME:LINE1;END=94081647;SVLEN=1263;AN=20618;AC=1;AF=4.850130953535746e-05;N_HET=1;N_HOMALT=0;N_HOMREF=10308;FREQ_HET=9.700261907071492e-05;FREQ_HOMALT=0.0;FREQ_HOMREF=0.9999029973809292;N_BI_GENOS=10309;North_Han_AN=2272;North_Han_AC=1;North_Han_
chr1	94085374	.	A	<INS:ME:LINE1>	.	PASS	SVTYPE=INS:ME:LINE1;END=94085386;SVLEN=237;AN=20618;AC=1;AF=4.850130953535746e-05;N_HET=1;N_HOMALT=0;N_HOMREF=10308;FREQ_HET=9.700261907071492e-05;FREQ_HOMALT=0.0;FREQ_HOMREF=0.9999029973809292;N_BI_GENOS=10309;South_Han_AN=1496;South_Han_AC=1;South_Han_A
chr1	94338158	.	A	<INS:ME:LINE1>	.	PASS	SVTYPE=INS:ME:LINE1;END=94338168;SVLEN=779;AN=20618;AC=1;AF=4.850130953535746e-05;N_HET=1;N_HOMALT=0;N_HOMREF=10308;FREQ_HET=9.700261907071492e-05;FREQ_HOMALT=0.0;FREQ_HOMREF=0.9999029973809292;N_BI_GENOS=10309;North_Han_AN=2272;North_Han_AC=1;North_Han_A
chr1	94726437	.	A	<INS:ME:LINE1>	.	PASS	SVTYPE=INS:ME:LINE1;END=94726454;SVLEN=2455;AN=20618;AC=104;AF=0.005044136191677175;N_HET=100;N_HOMALT=2;N_HOMREF=10207;FREQ_HET=0.00970026190707149;FREQ_HOMALT=0.00019400523814142983;FREQ_HOMREF=0.9901057328547871;N_BI_GENOS=10309;South_Han_AN=1496;South
chr1	94988297	.	A	<INS:ME:LINE1>	.	PASS	SVTYPE=INS:ME:LINE1;END=94988312;SVLEN=6003;AN=20618;AC=1;AF=4.850130953535746e-05;N_HET=1;N_HOMALT=0;N_HOMREF=10308;FREQ_HET=9.700261907071492e-05;FREQ_HOMALT=0.0;FREQ_HOMREF=0.9999029973809292;N_BI_GENOS=10309;North_Han_AN=2272;North_Han_AC=1;North_Han_
chr1	95067470	.	C	<INS:ME:LINE1>	.	PASS	SVTYPE=INS:ME:LINE1;END=95067485;SVLEN=6000;AN=20618;AC=1;AF=4.850130953535746e-05;N_HET=1;N_HOMALT=0;N_HOMREF=10308;FREQ_HET=9.700261907071492e-05;FREQ_HOMALT=0.0;FREQ_HOMREF=0.9999029973809292;N_BI_GENOS=10309;Lingnan_Han_AN=1888;Lingnan_Han_AC=1;Lingna
chr1	95263891	.	G	<INS:ME:LINE1>	.	PASS	SVTYPE=INS:ME:LINE1;END=95263905;SVLEN=5999;AN=20618;AC=1;AF=4.850130953535746e-05;N_HET=1;N_HOMALT=0;N_HOMREF=10308;FREQ_HET=9.700261907071492e-05;FREQ_HOMALT=0.0;FREQ_HOMREF=0.9999029973809292;N_BI_GENOS=10309;Mongolian_AN=454;Mongolian_AC=1;Mongolian_A
chr1	95336286	.	C	<INS:ME:LINE1>	.	PASS	SVTYPE=INS:ME:LINE1;END=95336301;SVLEN=5998;AN=20618;AC=1447;AF=0.07018139489766223;N_HET=1315;N_HOMALT=66;N_HOMREF=8928;FREQ_HET=0.12755844407779901;FREQ_HOMALT=0.006402172858667184;FREQ_HOMREF=0.8660393830633427;N_BI_GENOS=10309;South_Han_AN=1496;South_H
chr1	95735992	.	T	<INS:ME:LINE1>	.	PASS	SVTYPE=INS:ME:LINE1;END=95736008;SVLEN=6014;AN=20618;AC=5;AF=0.00024250654767678728;N_HET=5;N_HOMALT=0;N_HOMREF=10304;FREQ_HET=0.00048501309535357455;FREQ_HOMALT=0.0;FREQ_HOMREF=0.9995149869046465;N_BI_GENOS=10309;Northwest_Han_AN=1296;Northwest_Han_AC=1;
chr1	96106239	.	G	<INS:ME:LINE1>	.	PASS	SVTYPE=INS:ME:LINE1;END=96106254;SVLEN=5997;AN=20618;AC=1;AF=4.850130953535746e-05;N_HET=1;N_HOMALT=0;N_HOMREF=10308;FREQ_HET=9.700261907071492e-05;FREQ_HOMALT=0.0;FREQ_HOMREF=0.9999029973809292;N_BI_GENOS=10309;North_Han_AN=2272;North_Han_AC=1;North_Han_
chr1	96414805	.	A	<INS:ME:LINE1>	.	PASS	SVTYPE=INS:ME:LINE1;END=96414813;SVLEN=447;AN=20618;AC=6;AF=0.0002910078572121447;N_HET=6;N_HOMALT=0;N_HOMREF=10303;FREQ_HET=0.0005820157144242894;FREQ_HOMALT=0.0;FREQ_HOMREF=0.9994179842855757;N_BI_GENOS=10309;Northwest_Han_AN=1296;Northwest_Han_AC=1;Nor
chr1	96508893	.	G	<INS:ME:LINE1>	.	PASS	SVTYPE=INS:ME:LINE1;END=96508908;SVLEN=6009;AN=20618;AC=3;AF=0.00014550392860607236;N_HET=3;N_HOMALT=0;N_HOMREF=10306;FREQ_HET=0.0002910078572121447;FREQ_HOMALT=0.0;FREQ_HOMREF=0.9997089921427879;N_BI_GENOS=10309;East_Han_AN=6554;East_Han_AC=3;East_Han_AF
chr1	96746333	.	G	<INS:ME:LINE1>	.	PASS	SVTYPE=INS:ME:LINE1;END=96746348;SVLEN=5998;AN=20618;AC=1;AF=4.850130953535746e-05;N_HET=1;N_HOMALT=0;N_HOMREF=10308;FREQ_HET=9.700261907071492e-05;FREQ_HOMALT=0.0;FREQ_HOMREF=0.9999029973809292;N_BI_GENOS=10309;Miao_AN=482;Miao_AC=1;Miao_AF=0.00207468879
chr1	96869977	.	T	<INS:ME:LINE1>	.	PASS	SVTYPE=INS:ME:LINE1;END=96869977;SVLEN=208;AN=20618;AC=1;AF=4.850130953535746e-05;N_HET=1;N_HOMALT=0;N_HOMREF=10308;FREQ_HET=9.700261907071492e-05;FREQ_HOMALT=0.0;FREQ_HOMREF=0.9999029973809292;N_BI_GENOS=10309;East_Han_AN=6554;East_Han_AC=1;East_Han_AF=0
chr1	96887859	.	T	<INS:ME:LINE1>	.	PASS	SVTYPE=INS:ME:LINE1;END=96887871;SVLEN=5995;AN=20618;AC=1;AF=4.850130953535746e-05;N_HET=1;N_HOMALT=0;N_HOMREF=10308;FREQ_HET=9.700261907071492e-05;FREQ_HOMALT=0.0;FREQ_HOMREF=0.9999029973809292;N_BI_GENOS=10309;Central_Han_AN=2128;Central_Han_AC=1;Centra
chr1	97005419	.	G	<INS:ME:LINE1>	.	PASS	SVTYPE=INS:ME:LINE1;END=97005433;SVLEN=5991;AN=20618;AC=1;AF=4.850130953535746e-05;N_HET=1;N_HOMALT=0;N_HOMREF=10308;FREQ_HET=9.700261907071492e-05;FREQ_HOMALT=0.0;FREQ_HOMREF=0.9999029973809292;N_BI_GENOS=10309;Southeast_Han_AN=1908;Southeast_Han_AC=1;So
chr1	97035016	.	A	<INS:ME:LINE1>	.	PASS	SVTYPE=INS:ME:LINE1;END=97035025;SVLEN=2216;AN=20618;AC=1;AF=4.850130953535746e-05;N_HET=1;N_HOMALT=0;N_HOMREF=10308;FREQ_HET=9.700261907071492e-05;FREQ_HOMALT=0.0;FREQ_HOMREF=0.9999029973809292;N_BI_GENOS=10309;Central_Han_AN=2128;Central_Han_AC=1;Centra
chr1	97178817	.	A	<INS:ME:LINE1>	.	PASS	SVTYPE=INS:ME:LINE1;END=97178830;SVLEN=263;AN=20618;AC=1;AF=4.850130953535746e-05;N_HET=1;N_HOMALT=0;N_HOMREF=10308;FREQ_HET=9.700261907071492e-05;FREQ_HOMALT=0.0;FREQ_HOMREF=0.9999029973809292;N_BI_GENOS=10309;East_Han_AN=6554;East_Han_AC=1;East_Han_AF=0
chr1	97388991	.	G	<INS:ME:LINE1>	.	PASS	SVTYPE=INS:ME:LINE1;END=97389005;SVLEN=822;AN=20618;AC=1;AF=4.850130953535746e-05;N_HET=1;N_HOMALT=0;N_HOMREF=10308;FREQ_HET=9.700261907071492e-05;FREQ_HOMALT=0.0;FREQ_HOMREF=0.9999029973809292;N_BI_GENOS=10309;East_Han_AN=6554;East_Han_AC=1;East_Han_AF=0
chr1	97541542	.	A	<INS:ME:LINE1>	.	PASS	SVTYPE=INS:ME:LINE1;END=97541556;SVLEN=811;AN=20618;AC=1;AF=4.850130953535746e-05;N_HET=1;N_HOMALT=0;N_HOMREF=10308;FREQ_HET=9.700261907071492e-05;FREQ_HOMALT=0.0;FREQ_HOMREF=0.9999029973809292;N_BI_GENOS=10309;Mongolian_AN=454;Mongolian_AC=1;Mongolian_AF
chr1	97637752	.	T	<INS:ME:LINE1>	.	PASS	SVTYPE=INS:ME:LINE1;END=97637754;SVLEN=502;AN=20618;AC=1;AF=4.850130953535746e-05;N_HET=1;N_HOMALT=0;N_HOMREF=10308;FREQ_HET=9.700261907071492e-05;FREQ_HOMALT=0.0;FREQ_HOMREF=0.9999029973809292;N_BI_GENOS=10309;North_Han_AN=2272;North_Han_AC=1;North_Han_A
chr1	97714202	.	A	<INS:ME:LINE1>	.	PASS	SVTYPE=INS:ME:LINE1;END=97714202;SVLEN=645;AN=20618;AC=1;AF=4.850130953535746e-05;N_HET=1;N_HOMALT=0;N_HOMREF=10308;FREQ_HET=9.700261907071492e-05;FREQ_HOMALT=0.0;FREQ_HOMREF=0.9999029973809292;N_BI_GENOS=10309;Central_Han_AN=2128;Central_Han_AC=1;Central
chr1	97726861	.	A	<INS:ME:LINE1>	.	PASS	SVTYPE=INS:ME:LINE1;END=97726878;SVLEN=1075;AN=20618;AC=6;AF=0.0002910078572121447;N_HET=6;N_HOMALT=0;N_HOMREF=10303;FREQ_HET=0.0005820157144242894;FREQ_HOMALT=0.0;FREQ_HOMREF=0.9994179842855757;N_BI_GENOS=10309;South_Han_AN=1496;South_Han_AC=1;South_Han_
chr1	97732069	.	C	<INS:ME:LINE1>	.	PASS	SVTYPE=INS:ME:LINE1;END=97732084;SVLEN=2721;AN=20618;AC=45;AF=0.0021825589290910856;N_HET=43;N_HOMALT=1;N_HOMREF=10265;FREQ_HET=0.004171112620040741;FREQ_HOMALT=9.700261907071492e-05;FREQ_HOMREF=0.9957318847608886;N_BI_GENOS=10309;South_Han_AN=1496;South_
chr1	97748884	.	A	<INS:ME:LINE1>	.	PASS	SVTYPE=INS:ME:LINE1;END=97748887;SVLEN=784;AN=20618;AC=1;AF=4.850130953535746e-05;N_HET=1;N_HOMALT=0;N_HOMREF=10308;FREQ_HET=9.700261907071492e-05;FREQ_HOMALT=0.0;FREQ_HOMREF=0.9999029973809292;N_BI_GENOS=10309;Zhuang_AN=444;Zhuang_AC=1;Zhuang_AF=0.002252
chr1	97749862	.	T	<INS:ME:LINE1>	.	PASS	SVTYPE=INS:ME:LINE1;END=97749876;SVLEN=5989;AN=20618;AC=1;AF=4.850130953535746e-05;N_HET=1;N_HOMALT=0;N_HOMREF=10308;FREQ_HET=9.700261907071492e-05;FREQ_HOMALT=0.0;FREQ_HOMREF=0.9999029973809292;N_BI_GENOS=10309;North_Han_AN=2272;North_Han_AC=1;North_Han_
chr1	97883180	.	T	<INS:ME:LINE1>	.	PASS	SVTYPE=INS:ME:LINE1;END=97883184;SVLEN=243;AN=20618;AC=1;AF=4.850130953535746e-05;N_HET=1;N_HOMALT=0;N_HOMREF=10308;FREQ_HET=9.700261907071492e-05;FREQ_HOMALT=0.0;FREQ_HOMREF=0.9999029973809292;N_BI_GENOS=10309;East_Han_AN=6554;East_Han_AC=1;East_Han_AF=0
chr1	97898153	.	T	<INS:ME:LINE1>	.	PASS	SVTYPE=INS:ME:LINE1;END=97898170;SVLEN=1325;AN=20618;AC=840;AF=0.040741100009700264;N_HET=780;N_HOMALT=30;N_HOMREF=9499;FREQ_HET=0.07566204287515763;FREQ_HOMALT=0.0029100785721214474;FREQ_HOMREF=0.9214278785527209;N_BI_GENOS=10309;South_Han_AN=1496;South_
chr1	97926374	.	G	<INS:ME:LINE1>	.	PASS	SVTYPE=INS:ME:LINE1;END=97926385;SVLEN=1335;AN=20618;AC=2;AF=9.700261907071492e-05;N_HET=2;N_HOMALT=0;N_HOMREF=10307;FREQ_HET=0.00019400523814142983;FREQ_HOMALT=0.0;FREQ_HOMREF=0.9998059947618586;N_BI_GENOS=10309;Hui_AN=444;Hui_AC=2;Hui_AF=0.0045045045045
chr1	97966907	.	A	<INS:ME:LINE1>	.	PASS	SVTYPE=INS:ME:LINE1;END=97966921;SVLEN=6022;AN=20618;AC=1;AF=4.850130953535746e-05;N_HET=1;N_HOMALT=0;N_HOMREF=10308;FREQ_HET=9.700261907071492e-05;FREQ_HOMALT=0.0;FREQ_HOMREF=0.9999029973809292;N_BI_GENOS=10309;East_Han_AN=6554;East_Han_AC=1;East_Han_AF=
chr1	98024836	.	A	<INS:ME:LINE1>	.	PASS	SVTYPE=INS:ME:LINE1;END=98024848;SVLEN=2616;AN=20618;AC=1;AF=4.850130953535746e-05;N_HET=1;N_HOMALT=0;N_HOMREF=10308;FREQ_HET=9.700261907071492e-05;FREQ_HOMALT=0.0;FREQ_HOMREF=0.9999029973809292;N_BI_GENOS=10309;East_Han_AN=6554;East_Han_AC=1;East_Han_AF=
chr1	98226431	.	A	<INS:ME:LINE1>	.	PASS	SVTYPE=INS:ME:LINE1;END=98226445;SVLEN=625;AN=20618;AC=4;AF=0.00019400523814142983;N_HET=4;N_HOMALT=0;N_HOMREF=10305;FREQ_HET=0.0003880104762828566;FREQ_HOMALT=0.0;FREQ_HOMREF=0.9996119895237171;N_BI_GENOS=10309;Lingnan_Han_AN=1888;Lingnan_Han_AC=2;Lingn
chr1	98232096	.	A	<INS:ME:LINE1>	.	PASS	SVTYPE=INS:ME:LINE1;END=98232113;SVLEN=1019;AN=20618;AC=1;AF=4.850130953535746e-05;N_HET=1;N_HOMALT=0;N_HOMREF=10308;FREQ_HET=9.700261907071492e-05;FREQ_HOMALT=0.0;FREQ_HOMREF=0.9999029973809292;N_BI_GENOS=10309;South_Han_AN=1496;South_Han_AC=1;South_Han_
chr1	98299337	.	T	<INS:ME:LINE1>	.	PASS	SVTYPE=INS:ME:LINE1;END=98299337;SVLEN=1080;AN=20618;AC=1;AF=4.850130953535746e-05;N_HET=1;N_HOMALT=0;N_HOMREF=10308;FREQ_HET=9.700261907071492e-05;FREQ_HOMALT=0.0;FREQ_HOMREF=0.9999029973809292;N_BI_GENOS=10309;Tibetan_AN=414;Tibetan_AC=1;Tibetan_AF=0.00
chr1	98503333	.	A	<INS:ME:LINE1>	.	PASS	SVTYPE=INS:ME:LINE1;END=98503345;SVLEN=2269;AN=20618;AC=876;AF=0.04248714715297313;N_HET=784;N_HOMALT=46;N_HOMREF=9479;FREQ_HET=0.07605005335144405;FREQ_HOMALT=0.004462120477252886;FREQ_HOMREF=0.9194878261713066;N_BI_GENOS=10309;South_Han_AN=1496;South_Han
chr1	98582732	.	T	<INS:ME:LINE1>	.	PASS	SVTYPE=INS:ME:LINE1;END=98582748;SVLEN=1320;AN=20618;AC=1;AF=4.850130953535746e-05;N_HET=1;N_HOMALT=0;N_HOMREF=10308;FREQ_HET=9.700261907071492e-05;FREQ_HOMALT=0.0;FREQ_HOMREF=0.9999029973809292;N_BI_GENOS=10309;Central_Han_AN=2128;Central_Han_AC=1;Centra
chr1	98609909	.	A	<INS:ME:LINE1>	.	PASS	SVTYPE=INS:ME:LINE1;END=98609930;SVLEN=5998;AN=20618;AC=2;AF=9.700261907071492e-05;N_HET=2;N_HOMALT=0;N_HOMREF=10307;FREQ_HET=0.00019400523814142983;FREQ_HOMALT=0.0;FREQ_HOMREF=0.9998059947618586;N_BI_GENOS=10309;South_Han_AN=1496;South_Han_AC=1;South_Han
chr1	98756375	.	T	<INS:ME:LINE1>	.	PASS	SVTYPE=INS:ME:LINE1;END=98756392;SVLEN=5996;AN=20618;AC=395;AF=0.019158017266466196;N_HET=313;N_HOMALT=41;N_HOMREF=9955;FREQ_HET=0.030361819769133765;FREQ_HOMALT=0.003977107381899311;FREQ_HOMREF=0.965661072848967;N_BI_GENOS=10309;South_Han_AN=1496;South_H
chr1	98824487	.	G	<INS:ME:LINE1>	.	PASS	SVTYPE=INS:ME:LINE1;END=98824498;SVLEN=2136;AN=20618;AC=1;AF=4.850130953535746e-05;N_HET=1;N_HOMALT=0;N_HOMREF=10308;FREQ_HET=9.700261907071492e-05;FREQ_HOMALT=0.0;FREQ_HOMREF=0.9999029973809292;N_BI_GENOS=10309;South_Han_AN=1496;South_Han_AC=1;South_Han_
chr1	98946886	.	A	<INS:ME:LINE1>	.	PASS	SVTYPE=INS:ME:LINE1;END=98946907;SVLEN=361;AN=20618;AC=1;AF=4.850130953535746e-05;N_HET=1;N_HOMALT=0;N_HOMREF=10308;FREQ_HET=9.700261907071492e-05;FREQ_HOMALT=0.0;FREQ_HOMREF=0.9999029973809292;N_BI_GENOS=10309;Northwest_Han_AN=1296;Northwest_Han_AC=1;Nor
chr1	99267777	.	C	<INS:ME:LINE1>	.	PASS	SVTYPE=INS:ME:LINE1;END=99267790;SVLEN=801;AN=20618;AC=5;AF=0.00024250654767678728;N_HET=5;N_HOMALT=0;N_HOMREF=10304;FREQ_HET=0.00048501309535357455;FREQ_HOMALT=0.0;FREQ_HOMREF=0.9995149869046465;N_BI_GENOS=10309;North_Han_AN=2272;North_Han_AC=1;North_Han
chr1	99477205	.	A	<INS:ME:LINE1>	.	PASS	SVTYPE=INS:ME:LINE1;END=99477221;SVLEN=6000;AN=20618;AC=1;AF=4.850130953535746e-05;N_HET=1;N_HOMALT=0;N_HOMREF=10308;FREQ_HET=9.700261907071492e-05;FREQ_HOMALT=0.0;FREQ_HOMREF=0.9999029973809292;N_BI_GENOS=10309;Central_Han_AN=2128;Central_Han_AC=1;Centra
chr1	99492793	.	A	<INS:ME:LINE1>	.	PASS	SVTYPE=INS:ME:LINE1;END=99492808;SVLEN=6012;AN=20618;AC=2;AF=9.700261907071492e-05;N_HET=2;N_HOMALT=0;N_HOMREF=10307;FREQ_HET=0.00019400523814142983;FREQ_HOMALT=0.0;FREQ_HOMREF=0.9998059947618586;N_BI_GENOS=10309;North_Han_AN=2272;North_Han_AC=1;North_Han
chr1	99506258	.	A	<INS:ME:LINE1>	.	PASS	SVTYPE=INS:ME:LINE1;END=99506274;SVLEN=5999;AN=20618;AC=1;AF=4.850130953535746e-05;N_HET=1;N_HOMALT=0;N_HOMREF=10308;FREQ_HET=9.700261907071492e-05;FREQ_HOMALT=0.0;FREQ_HOMREF=0.9999029973809292;N_BI_GENOS=10309;North_Han_AN=2272;North_Han_AC=1;North_Han_
chr1	99608890	.	T	<INS:ME:LINE1>	.	PASS	SVTYPE=INS:ME:LINE1;END=99608890;SVLEN=554;AN=20618;AC=1;AF=4.850130953535746e-05;N_HET=1;N_HOMALT=0;N_HOMREF=10308;FREQ_HET=9.700261907071492e-05;FREQ_HOMALT=0.0;FREQ_HOMREF=0.9999029973809292;N_BI_GENOS=10309;East_Han_AN=6554;East_Han_AC=1;East_Han_AF=0
chr1	99881185	.	A	<INS:ME:LINE1>	.	PASS	SVTYPE=INS:ME:LINE1;END=99881199;SVLEN=6000;AN=20618;AC=1;AF=4.850130953535746e-05;N_HET=1;N_HOMALT=0;N_HOMREF=10308;FREQ_HET=9.700261907071492e-05;FREQ_HOMALT=0.0;FREQ_HOMREF=0.9999029973809292;N_BI_GENOS=10309;North_Han_AN=2272;North_Han_AC=1;North_Han_
chr1	100028266	.	T	<INS:ME:LINE1>	.	PASS	SVTYPE=INS:ME:LINE1;END=100028266;SVLEN=5992;AN=20618;AC=1;AF=4.850130953535746e-05;N_HET=1;N_HOMALT=0;N_HOMREF=10308;FREQ_HET=9.700261907071492e-05;FREQ_HOMALT=0.0;FREQ_HOMREF=0.9999029973809292;N_BI_GENOS=10309;East_Han_AN=6554;East_Han_AC=1;East_Han_AF
chr1	100091314	.	A	<INS:ME:LINE1>	.	PASS	SVTYPE=INS:ME:LINE1;END=100091329;SVLEN=1822;AN=20618;AC=1;AF=4.850130953535746e-05;N_HET=1;N_HOMALT=0;N_HOMREF=10308;FREQ_HET=9.700261907071492e-05;FREQ_HOMALT=0.0;FREQ_HOMREF=0.9999029973809292;N_BI_GENOS=10309;East_Han_AN=6554;East_Han_AC=1;East_Han_AF
chr1	100142762	.	A	<INS:ME:LINE1>	.	PASS	SVTYPE=INS:ME:LINE1;END=100142775;SVLEN=5998;AN=20618;AC=1;AF=4.850130953535746e-05;N_HET=1;N_HOMALT=0;N_HOMREF=10308;FREQ_HET=9.700261907071492e-05;FREQ_HOMALT=0.0;FREQ_HOMREF=0.9999029973809292;N_BI_GENOS=10309;Central_Han_AN=2128;Central_Han_AC=1;Centr
chr1	100396944	.	A	<INS:ME:LINE1>	.	PASS	SVTYPE=INS:ME:LINE1;END=100396960;SVLEN=990;AN=20618;AC=1;AF=4.850130953535746e-05;N_HET=1;N_HOMALT=0;N_HOMREF=10308;FREQ_HET=9.700261907071492e-05;FREQ_HOMALT=0.0;FREQ_HOMREF=0.9999029973809292;N_BI_GENOS=10309;North_Han_AN=2272;North_Han_AC=1;North_Han_
chr1	100509861	.	G	<INS:ME:LINE1>	.	PASS	SVTYPE=INS:ME:LINE1;END=100509871;SVLEN=975;AN=20618;AC=2;AF=9.700261907071492e-05;N_HET=2;N_HOMALT=0;N_HOMREF=10307;FREQ_HET=0.00019400523814142983;FREQ_HOMALT=0.0;FREQ_HOMREF=0.9998059947618586;N_BI_GENOS=10309;North_Han_AN=2272;North_Han_AC=1;North_Han
chr1	100573222	.	G	<INS:ME:LINE1>	.	PASS	SVTYPE=INS:ME:LINE1;END=100573222;SVLEN=647;AN=20618;AC=3;AF=0.00014550392860607236;N_HET=3;N_HOMALT=0;N_HOMREF=10306;FREQ_HET=0.0002910078572121447;FREQ_HOMALT=0.0;FREQ_HOMREF=0.9997089921427879;N_BI_GENOS=10309;East_Han_AN=6554;East_Han_AC=3;East_Han_AF
chr1	100651244	.	A	<INS:ME:LINE1>	.	PASS	SVTYPE=INS:ME:LINE1;END=100651257;SVLEN=271;AN=20618;AC=2;AF=9.700261907071492e-05;N_HET=2;N_HOMALT=0;N_HOMREF=10307;FREQ_HET=0.00019400523814142983;FREQ_HOMALT=0.0;FREQ_HOMREF=0.9998059947618586;N_BI_GENOS=10309;Northwest_Han_AN=1296;Northwest_Han_AC=1;N
chr1	100651604	.	A	<INS:ME:LINE1>	.	PASS	SVTYPE=INS:ME:LINE1;END=100651604;SVLEN=576;AN=20618;AC=1;AF=4.850130953535746e-05;N_HET=1;N_HOMALT=0;N_HOMREF=10308;FREQ_HET=9.700261907071492e-05;FREQ_HOMALT=0.0;FREQ_HOMREF=0.9999029973809292;N_BI_GENOS=10309;East_Han_AN=6554;East_Han_AC=1;East_Han_AF=
chr1	100763135	.	A	<INS:ME:LINE1>	.	PASS	SVTYPE=INS:ME:LINE1;END=100763153;SVLEN=6145;AN=20618;AC=1;AF=4.850130953535746e-05;N_HET=1;N_HOMALT=0;N_HOMREF=10308;FREQ_HET=9.700261907071492e-05;FREQ_HOMALT=0.0;FREQ_HOMREF=0.9999029973809292;N_BI_GENOS=10309;Lingnan_Han_AN=1888;Lingnan_Han_AC=1;Lingn
chr1	100776416	.	T	<INS:ME:LINE1>	.	PASS	SVTYPE=INS:ME:LINE1;END=100776430;SVLEN=534;AN=20618;AC=4;AF=0.00019400523814142983;N_HET=4;N_HOMALT=0;N_HOMREF=10305;FREQ_HET=0.0003880104762828566;FREQ_HOMALT=0.0;FREQ_HOMREF=0.9996119895237171;N_BI_GENOS=10309;Lingnan_Han_AN=1888;Lingnan_Han_AC=4;Ling
chr1	101198682	.	A	<INS:ME:LINE1>	.	PASS	SVTYPE=INS:ME:LINE1;END=101198696;SVLEN=74;AN=20618;AC=85;AF=0.0041226113105053835;N_HET=81;N_HOMALT=2;N_HOMREF=10226;FREQ_HET=0.007857212144727907;FREQ_HOMALT=0.00019400523814142983;FREQ_HOMREF=0.9919487826171307;N_BI_GENOS=10309;South_Han_AN=1496;South_Han_
chr1	101513424	.	T	<INS:ME:LINE1>	.	PASS	SVTYPE=INS:ME:LINE1;END=101513435;SVLEN=2317;AN=20618;AC=3559;AF=0.17261616063633717;N_HET=2749;N_HOMALT=405;N_HOMREF=7155;FREQ_HET=0.2666601998253953;FREQ_HOMALT=0.039286060723639535;FREQ_HOMREF=0.6940537394509652;N_BI_GENOS=10309;South_Han_AN=1496;South
chr1	101883806	.	A	<INS:ME:LINE1>	.	PASS	SVTYPE=INS:ME:LINE1;END=101883808;SVLEN=925;AN=20618;AC=1;AF=4.850130953535746e-05;N_HET=1;N_HOMALT=0;N_HOMREF=10308;FREQ_HET=9.700261907071492e-05;FREQ_HOMALT=0.0;FREQ_HOMREF=0.9999029973809292;N_BI_GENOS=10309;East_Han_AN=6554;East_Han_AC=1;East_Han_AF=
chr1	102067193	.	G	<INS:ME:LINE1>	.	PASS	SVTYPE=INS:ME:LINE1;END=102067193;SVLEN=1067;AN=20618;AC=1;AF=4.850130953535746e-05;N_HET=1;N_HOMALT=0;N_HOMREF=10308;FREQ_HET=9.700261907071492e-05;FREQ_HOMALT=0.0;FREQ_HOMREF=0.9999029973809292;N_BI_GENOS=10309;East_Han_AN=6554;East_Han_AC=1;East_Han_AF
chr1	102103352	.	A	<INS:ME:LINE1>	.	PASS	SVTYPE=INS:ME:LINE1;END=102103367;SVLEN=5997;AN=20618;AC=4445;AF=0.2155883208846639;N_HET=3191;N_HOMALT=627;N_HOMREF=6491;FREQ_HET=0.30953535745465127;FREQ_HOMALT=0.06082064215733825;FREQ_HOMREF=0.6296440003880105;N_BI_GENOS=10309;South_Han_AN=1496;South_
chr1	102181649	.	T	<INS:ME:LINE1>	.	PASS	SVTYPE=INS:ME:LINE1;END=102181664;SVLEN=5998;AN=20618;AC=7;AF=0.0003395091667475022;N_HET=7;N_HOMALT=0;N_HOMREF=10302;FREQ_HET=0.0006790183334950044;FREQ_HOMALT=0.0;FREQ_HOMREF=0.999320981666505;N_BI_GENOS=10309;North_Han_AN=2272;North_Han_AC=1;North_Han_
chr1	102353561	.	T	<INS:ME:LINE1>	.	PASS	SVTYPE=INS:ME:LINE1;END=102353561;SVLEN=825;AN=20618;AC=1;AF=4.850130953535746e-05;N_HET=1;N_HOMALT=0;N_HOMREF=10308;FREQ_HET=9.700261907071492e-05;FREQ_HOMALT=0.0;FREQ_HOMREF=0.9999029973809292;N_BI_GENOS=10309;East_Han_AN=6554;East_Han_AC=1;East_Han_AF=
chr1	102432172	.	G	<INS:ME:LINE1>	.	PASS	SVTYPE=INS:ME:LINE1;END=102432188;SVLEN=2818;AN=20618;AC=37;AF=0.0017945484528082258;N_HET=37;N_HOMALT=0;N_HOMREF=10272;FREQ_HET=0.0035890969056164516;FREQ_HOMALT=0.0;FREQ_HOMREF=0.9964109030943835;N_BI_GENOS=10309;South_Han_AN=1496;South_Han_AC=4;South_H
chr1	102580551	.	G	<INS:ME:LINE1>	.	PASS	SVTYPE=INS:ME:LINE1;END=102580559;SVLEN=729;AN=20618;AC=2;AF=9.700261907071492e-05;N_HET=2;N_HOMALT=0;N_HOMREF=10307;FREQ_HET=0.00019400523814142983;FREQ_HOMALT=0.0;FREQ_HOMREF=0.9998059947618586;N_BI_GENOS=10309;East_Han_AN=6554;East_Han_AC=1;East_Han_AF
chr1	102646803	.	T	<INS:ME:LINE1>	.	PASS	SVTYPE=INS:ME:LINE1;END=102646804;SVLEN=692;AN=20618;AC=8;AF=0.0003880104762828566;N_HET=8;N_HOMALT=0;N_HOMREF=10301;FREQ_HET=0.0007760209525657193;FREQ_HOMALT=0.0;FREQ_HOMREF=0.9992239790474343;N_BI_GENOS=10309;South_Han_AN=1496;South_Han_AC=1;South_Han
chr1	102753454	.	A	<INS:ME:LINE1>	.	PASS	SVTYPE=INS:ME:LINE1;END=102753454;SVLEN=0;AN=20618;AC=1;AF=4.850130953535746e-05;N_HET=1;N_HOMALT=0;N_HOMREF=10308;FREQ_HET=9.700261907071492e-05;FREQ_HOMALT=0.0;FREQ_HOMREF=0.9999029973809292;N_BI_GENOS=10309;South_Han_AN=1496;South_Han_AC=1;South_Han_AF
chr1	102902188	.	T	<INS:ME:LINE1>	.	PASS	SVTYPE=INS:ME:LINE1;END=102902190;SVLEN=2605;AN=20618;AC=1;AF=4.850130953535746e-05;N_HET=1;N_HOMALT=0;N_HOMREF=10308;FREQ_HET=9.700261907071492e-05;FREQ_HOMALT=0.0;FREQ_HOMREF=0.9999029973809292;N_BI_GENOS=10309;Northwest_Han_AN=1296;Northwest_Han_AC=1;N
chr1	103035840	.	A	<INS:ME:LINE1>	.	PASS	SVTYPE=INS:ME:LINE1;END=103035845;SVLEN=95;AN=20618;AC=1;AF=4.850130953535746e-05;N_HET=1;N_HOMALT=0;N_HOMREF=10308;FREQ_HET=9.700261907071492e-05;FREQ_HOMALT=0.0;FREQ_HOMREF=0.9999029973809292;N_BI_GENOS=10309;Southeast_Han_AN=1908;Southeast_Han_AC=1;Sou
chr1	103058104	.	T	<INS:ME:LINE1>	.	PASS	SVTYPE=INS:ME:LINE1;END=103058117;SVLEN=515;AN=20618;AC=1;AF=4.850130953535746e-05;N_HET=1;N_HOMALT=0;N_HOMREF=10308;FREQ_HET=9.700261907071492e-05;FREQ_HOMALT=0.0;FREQ_HOMREF=0.9999029973809292;N_BI_GENOS=10309;East_Han_AN=6554;East_Han_AC=1;East_Han_AF=
chr1	103181739	.	G	<INS:ME:LINE1>	.	PASS	SVTYPE=INS:ME:LINE1;END=103181755;SVLEN=488;AN=20618;AC=4;AF=0.00019400523814142983;N_HET=2;N_HOMALT=1;N_HOMREF=10306;FREQ_HET=0.00019400523814142983;FREQ_HOMALT=9.700261907071492e-05;FREQ_HOMREF=0.9997089921427879;N_BI_GENOS=10309;Lingnan_Han_AN=1888;Lin
chr1	103194653	.	A	<INS:ME:LINE1>	.	PASS	SVTYPE=INS:ME:LINE1;END=103194670;SVLEN=758;AN=20618;AC=1;AF=4.850130953535746e-05;N_HET=1;N_HOMALT=0;N_HOMREF=10308;FREQ_HET=9.700261907071492e-05;FREQ_HOMALT=0.0;FREQ_HOMREF=0.9999029973809292;N_BI_GENOS=10309;Southeast_Han_AN=1908;Southeast_Han_AC=1;So
chr1	103442373	.	C	<INS:ME:LINE1>	.	PASS	SVTYPE=INS:ME:LINE1;END=103442388;SVLEN=5997;AN=20618;AC=1;AF=4.850130953535746e-05;N_HET=1;N_HOMALT=0;N_HOMREF=10308;FREQ_HET=9.700261907071492e-05;FREQ_HOMALT=0.0;FREQ_HOMREF=0.9999029973809292;N_BI_GENOS=10309;East_Han_AN=6554;East_Han_AC=1;East_Han_AF
chr1	103462106	.	A	<INS:ME:LINE1>	.	PASS	SVTYPE=INS:ME:LINE1;END=103462123;SVLEN=893;AN=20618;AC=6;AF=0.0002910078572121447;N_HET=4;N_HOMALT=1;N_HOMREF=10304;FREQ_HET=0.0003880104762828566;FREQ_HOMALT=9.700261907071492e-05;FREQ_HOMREF=0.9995149869046465;N_BI_GENOS=10309;Lingnan_Han_AN=1888;Ling
chr1	103503371	.	A	<INS:ME:LINE1>	.	PASS	SVTYPE=INS:ME:LINE1;END=103503379;SVLEN=1042;AN=20618;AC=1;AF=4.850130953535746e-05;N_HET=1;N_HOMALT=0;N_HOMREF=10308;FREQ_HET=9.700261907071492e-05;FREQ_HOMALT=0.0;FREQ_HOMREF=0.9999029973809292;N_BI_GENOS=10309;Lingnan_Han_AN=1888;Lingnan_Han_AC=1;Lingn

chr1	103788630	.	A	<INS:ME:LINE1>	.	PASS	SVTYPE=INS:ME:LINE1;END=103788644;SVLEN=1276;AN=20618;AC=1;AF=4.850130953535746e-05;N_HET=1;N_HOMALT=0;N_HOMREF=10308;FREQ_HET=9.700261907071492e-05;FREQ_HOMALT=0.0;FREQ_HOMREF=0.9999029973809292;N_BI_GENOS=10309;East_Han_AN=6554;East_Han_AC=1;East_Han_AF
chr1	103935771	.	A	<INS:ME:LINE1>	.	PASS	SVTYPE=INS:ME:LINE1;END=103935785;SVLEN=6039;AN=20618;AC=1;AF=4.850130953535746e-05;N_HET=1;N_HOMALT=0;N_HOMREF=10308;FREQ_HET=9.700261907071492e-05;FREQ_HOMALT=0.0;FREQ_HOMREF=0.9999029973809292;N_BI_GENOS=10309;Northwest_Han_AN=1296;Northwest_Han_AC=1;N
chr1	103947536	.	A	<INS:ME:LINE1>	.	PASS	SVTYPE=INS:ME:LINE1;END=103947536;SVLEN=0;AN=20618;AC=1;AF=4.850130953535746e-05;N_HET=1;N_HOMALT=0;N_HOMREF=10308;FREQ_HET=9.700261907071492e-05;FREQ_HOMALT=0.0;FREQ_HOMREF=0.9999029973809292;N_BI_GENOS=10309;East_Han_AN=6554;East_Han_AC=1;East_Han_AF=0.
chr1	103948903	.	A	<INS:ME:LINE1>	.	PASS	SVTYPE=INS:ME:LINE1;END=103948904;SVLEN=254;AN=20618;AC=1;AF=4.850130953535746e-05;N_HET=1;N_HOMALT=0;N_HOMREF=10308;FREQ_HET=9.700261907071492e-05;FREQ_HOMALT=0.0;FREQ_HOMREF=0.9999029973809292;N_BI_GENOS=10309;East_Han_AN=6554;East_Han_AC=1;East_Han_AF=
chr1	104312345	.	T	<INS:ME:LINE1>	.	PASS	SVTYPE=INS:ME:LINE1;END=104312360;SVLEN=1305;AN=20618;AC=2;AF=9.700261907071492e-05;N_HET=0;N_HOMALT=1;N_HOMREF=10308;FREQ_HET=0.0;FREQ_HOMALT=9.700261907071492e-05;FREQ_HOMREF=0.9999029973809292;N_BI_GENOS=10309;Southeast_Han_AN=1908;Southeast_Han_AC=2;S
chr1	104322114	.	G	<INS:ME:LINE1>	.	PASS	SVTYPE=INS:ME:LINE1;END=104322114;SVLEN=847;AN=20618;AC=1;AF=4.850130953535746e-05;N_HET=1;N_HOMALT=0;N_HOMREF=10308;FREQ_HET=9.700261907071492e-05;FREQ_HOMALT=0.0;FREQ_HOMREF=0.9999029973809292;N_BI_GENOS=10309;Lingnan_Han_AN=1888;Lingnan_Han_AC=1;Lingna
chr1	104365661	.	A	<INS:ME:LINE1>	.	PASS	SVTYPE=INS:ME:LINE1;END=104365676;SVLEN=1986;AN=20618;AC=1;AF=4.850130953535746e-05;N_HET=1;N_HOMALT=0;N_HOMREF=10308;FREQ_HET=9.700261907071492e-05;FREQ_HOMALT=0.0;FREQ_HOMREF=0.9999029973809292;N_BI_GENOS=10309;Northwest_Han_AN=1296;Northwest_Han_AC=1;N
chr1	104370103	.	G	<INS:ME:LINE1>	.	PASS	SVTYPE=INS:ME:LINE1;END=104370110;SVLEN=2984;AN=20618;AC=1;AF=4.850130953535746e-05;N_HET=1;N_HOMALT=0;N_HOMREF=10308;FREQ_HET=9.700261907071492e-05;FREQ_HOMALT=0.0;FREQ_HOMREF=0.9999029973809292;N_BI_GENOS=10309;Central_Han_AN=2128;Central_Han_AC=1;Centr
chr1	104485593	.	A	<INS:ME:LINE1>	.	PASS	SVTYPE=INS:ME:LINE1;END=104485604;SVLEN=55;AN=20618;AC=441;AF=0.021389077505092637;N_HET=401;N_HOMALT=20;N_HOMREF=9888;FREQ_HET=0.03889805024735668;FREQ_HOMALT=0.0019400523814142982;FREQ_HOMREF=0.959161897371229;N_BI_GENOS=10309;South_Han_AN=1496;South_Ha
chr1	104517868	.	A	<INS:ME:LINE1>	.	PASS	SVTYPE=INS:ME:LINE1;END=104517868;SVLEN=575;AN=20618;AC=1;AF=4.850130953535746e-05;N_HET=1;N_HOMALT=0;N_HOMREF=10308;FREQ_HET=9.700261907071492e-05;FREQ_HOMALT=0.0;FREQ_HOMREF=0.9999029973809292;N_BI_GENOS=10309;East_Han_AN=6554;East_Han_AC=1;East_Han_AF=
chr1	104640939	.	C	<INS:ME:LINE1>	.	PASS	SVTYPE=INS:ME:LINE1;END=104640948;SVLEN=6035;AN=20618;AC=1;AF=4.850130953535746e-05;N_HET=1;N_HOMALT=0;N_HOMREF=10308;FREQ_HET=9.700261907071492e-05;FREQ_HOMALT=0.0;FREQ_HOMREF=0.9999029973809292;N_BI_GENOS=10309;Yi_AN=422;Yi_AC=1;Yi_AF=0.0023696682464454
chr1	104886654	.	A	<INS:ME:LINE1>	.	PASS	SVTYPE=INS:ME:LINE1;END=104886671;SVLEN=711;AN=20618;AC=1;AF=4.850130953535746e-05;N_HET=1;N_HOMALT=0;N_HOMREF=10308;FREQ_HET=9.700261907071492e-05;FREQ_HOMALT=0.0;FREQ_HOMREF=0.9999029973809292;N_BI_GENOS=10309;Hui_AN=444;Hui_AC=1;Hui_AF=0.00225225225225
chr1	104909536	.	C	<INS:ME:LINE1>	.	PASS	SVTYPE=INS:ME:LINE1;END=104909550;SVLEN=5996;AN=20618;AC=1;AF=4.850130953535746e-05;N_HET=1;N_HOMALT=0;N_HOMREF=10308;FREQ_HET=9.700261907071492e-05;FREQ_HOMALT=0.0;FREQ_HOMREF=0.9999029973809292;N_BI_GENOS=10309;North_Han_AN=2272;North_Han_AC=1;North_Han
chr1	105115658	.	G	<INS:ME:LINE1>	.	PASS	SVTYPE=INS:ME:LINE1;END=105115658;SVLEN=0;AN=20618;AC=2;AF=9.700261907071492e-05;N_HET=0;N_HOMALT=1;N_HOMREF=10308;FREQ_HET=0.0;FREQ_HOMALT=9.700261907071492e-05;FREQ_HOMREF=0.9999029973809292;N_BI_GENOS=10309;East_Han_AN=6554;East_Han_AC=2;East_Han_AF=0.
chr1	105146861	.	C	<INS:ME:LINE1>	.	PASS	SVTYPE=INS:ME:LINE1;END=105146879;SVLEN=2051;AN=20618;AC=1;AF=4.850130953535746e-05;N_HET=1;N_HOMALT=0;N_HOMREF=10308;FREQ_HET=9.700261907071492e-05;FREQ_HOMALT=0.0;FREQ_HOMREF=0.9999029973809292;N_BI_GENOS=10309;Tibetan_AN=414;Tibetan_AC=1;Tibetan_AF=0.0
chr1	105188945	.	T	<INS:ME:LINE1>	.	PASS	SVTYPE=INS:ME:LINE1;END=105188955;SVLEN=3031;AN=20618;AC=1;AF=4.850130953535746e-05;N_HET=1;N_HOMALT=0;N_HOMREF=10308;FREQ_HET=9.700261907071492e-05;FREQ_HOMALT=0.0;FREQ_HOMREF=0.9999029973809292;N_BI_GENOS=10309;Lingnan_Han_AN=1888;Lingnan_Han_AC=1;Lingn
chr1	105189509	.	T	<INS:ME:LINE1>	.	PASS	SVTYPE=INS:ME:LINE1;END=105189550;SVLEN=219;AN=20618;AC=1;AF=4.850130953535746e-05;N_HET=1;N_HOMALT=0;N_HOMREF=10308;FREQ_HET=9.700261907071492e-05;FREQ_HOMALT=0.0;FREQ_HOMREF=0.9999029973809292;N_BI_GENOS=10309;East_Han_AN=6554;East_Han_AC=1;East_Han_AF=
chr1	105257209	.	A	<INS:ME:LINE1>	.	PASS	SVTYPE=INS:ME:LINE1;END=105257226;SVLEN=1359;AN=20618;AC=12;AF=0.0005820157144242894;N_HET=8;N_HOMALT=2;N_HOMREF=10299;FREQ_HET=0.0007760209525657193;FREQ_HOMALT=0.00019400523814142983;FREQ_HOMREF=0.9990299738092928;N_BI_GENOS=10309;South_Han_AN=1496;Sout
chr1	105499717	.	T	<INS:ME:LINE1>	.	PASS	SVTYPE=INS:ME:LINE1;END=105499735;SVLEN=2328;AN=20618;AC=1;AF=4.850130953535746e-05;N_HET=1;N_HOMALT=0;N_HOMREF=10308;FREQ_HET=9.700261907071492e-05;FREQ_HOMALT=0.0;FREQ_HOMREF=0.9999029973809292;N_BI_GENOS=10309;East_Han_AN=6554;East_Han_AC=1;East_Han_AF
chr1	105564727	.	G	<INS:ME:LINE1>	.	PASS	SVTYPE=INS:ME:LINE1;END=105564742;SVLEN=218;AN=20618;AC=3;AF=0.00014550392860607236;N_HET=3;N_HOMALT=0;N_HOMREF=10306;FREQ_HET=0.0002910078572121447;FREQ_HOMALT=0.0;FREQ_HOMREF=0.9997089921427879;N_BI_GENOS=10309;South_Han_AN=1496;South_Han_AC=1;South_Han
chr1	105668948	.	T	<INS:ME:LINE1>	.	PASS	SVTYPE=INS:ME:LINE1;END=105668963;SVLEN=829;AN=20618;AC=1;AF=4.850130953535746e-05;N_HET=1;N_HOMALT=0;N_HOMREF=10308;FREQ_HET=9.700261907071492e-05;FREQ_HOMALT=0.0;FREQ_HOMREF=0.9999029973809292;N_BI_GENOS=10309;Northwest_Han_AN=1296;Northwest_Han_AC=1;No
chr1	105677246	.	A	<INS:ME:LINE1>	.	PASS	SVTYPE=INS:ME:LINE1;END=105677262;SVLEN=744;AN=20618;AC=1;AF=4.850130953535746e-05;N_HET=1;N_HOMALT=0;N_HOMREF=10308;FREQ_HET=9.700261907071492e-05;FREQ_HOMALT=0.0;FREQ_HOMREF=0.9999029973809292;N_BI_GENOS=10309;East_Han_AN=6554;East_Han_AC=1;East_Han_AF=
chr1	105716360	.	A	<INS:ME:LINE1>	.	PASS	SVTYPE=INS:ME:LINE1;END=105716373;SVLEN=2117;AN=20618;AC=1;AF=4.850130953535746e-05;N_HET=1;N_HOMALT=0;N_HOMREF=10308;FREQ_HET=9.700261907071492e-05;FREQ_HOMALT=0.0;FREQ_HOMREF=0.9999029973809292;N_BI_GENOS=10309;Northwest_Han_AN=1296;Northwest_Han_AC=1;N
chr1	105853790	.	A	<INS:ME:LINE1>	.	PASS	SVTYPE=INS:ME:LINE1;END=105853806;SVLEN=474;AN=20618;AC=8;AF=0.00038801047628285966;N_HET=8;N_HOMALT=0;N_HOMREF=10301;FREQ_HET=0.0007760209525657193;FREQ_HOMALT=0.0;FREQ_HOMREF=0.9992239790474343;N_BI_GENOS=10309;Northwest_Han_AN=1296;Northwest_Han_AC=2;N
chr1	106082510	.	T	<INS:ME:LINE1>	.	PASS	SVTYPE=INS:ME:LINE1;END=106082524;SVLEN=346;AN=20618;AC=1;AF=4.850130953535746e-05;N_HET=1;N_HOMALT=0;N_HOMREF=10308;FREQ_HET=9.700261907071492e-05;FREQ_HOMALT=0.0;FREQ_HOMREF=0.9999029973809292;N_BI_GENOS=10309;Central_Han_AN=2128;Central_Han_AC=1;Centra
chr1	106172135	.	A	<INS:ME:LINE1>	.	PASS	SVTYPE=INS:ME:LINE1;END=106172153;SVLEN=352;AN=20618;AC=1;AF=4.850130953535746e-05;N_HET=1;N_HOMALT=0;N_HOMREF=10308;FREQ_HET=9.700261907071492e-05;FREQ_HOMALT=0.0;FREQ_HOMREF=0.9999029973809292;N_BI_GENOS=10309;North_Han_AN=2272;North_Han_AC=1;North_Han_
chr1	106177548	.	T	<INS:ME:LINE1>	.	PASS	SVTYPE=INS:ME:LINE1;END=106177560;SVLEN=1088;AN=20618;AC=3;AF=0.00014550392860607236;N_HET=3;N_HOMALT=0;N_HOMREF=10306;FREQ_HET=0.0002910078572121447;FREQ_HOMALT=0.0;FREQ_HOMREF=0.9997089921427879;N_BI_GENOS=10309;Lingnan_Han_AN=1888;Lingnan_Han_AC=1;Ling
chr1	106477910	.	T	<INS:ME:LINE1>	.	PASS	SVTYPE=INS:ME:LINE1;END=106477925;SVLEN=6018;AN=20618;AC=1;AF=4.850130953535746e-05;N_HET=1;N_HOMALT=0;N_HOMREF=10308;FREQ_HET=9.700261907071492e-05;FREQ_HOMALT=0.0;FREQ_HOMREF=0.9999029973809292;N_BI_GENOS=10309;South_Han_AN=1496;South_Han_AC=1;South_Han
chr1	106749418	.	C	<INS:ME:LINE1>	.	PASS	SVTYPE=INS:ME:LINE1;END=106749434;SVLEN=6000;AN=20618;AC=2;AF=9.700261907071492e-05;N_HET=2;N_HOMALT=0;N_HOMREF=10307;FREQ_HET=0.00019400523814142983;FREQ_HOMALT=0.0;FREQ_HOMREF=0.9998059947618586;N_BI_GENOS=10309;Lingnan_Han_AN=1888;Lingnan_Han_AC=1;Ling
chr1	106769381	.	G	<INS:ME:LINE1>	.	PASS	SVTYPE=INS:ME:LINE1;END=106769381;SVLEN=5973;AN=20618;AC=1;AF=4.850130953535746e-05;N_HET=1;N_HOMALT=0;N_HOMREF=10308;FREQ_HET=9.700261907071492e-05;FREQ_HOMALT=0.0;FREQ_HOMREF=0.9999029973809292;N_BI_GENOS=10309;Hui_AN=444;Hui_AC=1;Hui_AF=0.0022522522522
chr1	106784102	.	G	<INS:ME:LINE1>	.	PASS	SVTYPE=INS:ME:LINE1;END=106784112;SVLEN=5993;AN=20618;AC=1;AF=4.850130953535746e-05;N_HET=1;N_HOMALT=0;N_HOMREF=10308;FREQ_HET=9.700261907071492e-05;FREQ_HOMALT=0.0;FREQ_HOMREF=0.9999029973809292;N_BI_GENOS=10309;Lingnan_Han_AN=1888;Lingnan_Han_AC=1;Lingn
chr1	106801619	.	A	<INS:ME:LINE1>	.	PASS	SVTYPE=INS:ME:LINE1;END=106801638;SVLEN=1123;AN=20618;AC=1;AF=4.850130953535746e-05;N_HET=1;N_HOMALT=0;N_HOMREF=10308;FREQ_HET=9.700261907071492e-05;FREQ_HOMALT=0.0;FREQ_HOMREF=0.9999029973809292;N_BI_GENOS=10309;North_Han_AN=2272;North_Han_AC=1;North_Han
chr1	106863824	.	A	<INS:ME:LINE1>	.	PASS	SVTYPE=INS:ME:LINE1;END=106863836;SVLEN=5999;AN=20618;AC=1;AF=4.850130953535746e-05;N_HET=1;N_HOMALT=0;N_HOMREF=10308;FREQ_HET=9.700261907071492e-05;FREQ_HOMALT=0.0;FREQ_HOMREF=0.9999029973809292;N_BI_GENOS=10309;Mongolian_AN=454;Mongolian_AC=1;Mongolian_
chr1	106870363	.	T	<INS:ME:LINE1>	.	PASS	SVTYPE=INS:ME:LINE1;END=106870375;SVLEN=6000;AN=20618;AC=1;AF=4.850130953535746e-05;N_HET=1;N_HOMALT=0;N_HOMREF=10308;FREQ_HET=9.700261907071492e-05;FREQ_HOMALT=0.0;FREQ_HOMREF=0.9999029973809292;N_BI_GENOS=10309;Southeast_Han_AN=1908;Southeast_Han_AC=1;S
chr1	106918420	.	A	<INS:ME:LINE1>	.	PASS	SVTYPE=INS:ME:LINE1;END=106918429;SVLEN=212;AN=20618;AC=1;AF=4.850130953535746e-05;N_HET=1;N_HOMALT=0;N_HOMREF=10308;FREQ_HET=9.700261907071492e-05;FREQ_HOMALT=0.0;FREQ_HOMREF=0.9999029973809292;N_BI_GENOS=10309;South_Han_AN=1496;South_Han_AC=1;South_Han_
chr1	106929389	.	A	<INS:ME:LINE1>	.	PASS	SVTYPE=INS:ME:LINE1;END=106929389;SVLEN=2966;AN=20618;AC=1;AF=4.850130953535746e-05;N_HET=1;N_HOMALT=0;N_HOMREF=10308;FREQ_HET=9.700261907071492e-05;FREQ_HOMALT=0.0;FREQ_HOMREF=0.9999029973809292;N_BI_GENOS=10309;Lingnan_Han_AN=1888;Lingnan_Han_AC=1;Lingn
chr1	106942224	.	T	<INS:ME:LINE1>	.	PASS	SVTYPE=INS:ME:LINE1;END=106942239;SVLEN=193;AN=20618;AC=1;AF=4.850130953535746e-05;N_HET=1;N_HOMALT=0;N_HOMREF=10308;FREQ_HET=9.700261907071492e-05;FREQ_HOMALT=0.0;FREQ_HOMREF=0.9999029973809292;N_BI_GENOS=10309;Northwest_Han_AN=1296;Northwest_Han_AC=1;No
chr1	106985293	.	A	<INS:ME:LINE1>	.	PASS	SVTYPE=INS:ME:LINE1;END=106985309;SVLEN=295;AN=20618;AC=1;AF=4.850130953535746e-05;N_HET=1;N_HOMALT=0;N_HOMREF=10308;FREQ_HET=9.700261907071492e-05;FREQ_HOMALT=0.0;FREQ_HOMREF=0.9999029973809292;N_BI_GENOS=10309;East_Han_AN=6554;East_Han_AC=1;East_Han_AF=
chr1	107234670	.	C	<INS:ME:LINE1>	.	PASS	SVTYPE=INS:ME:LINE1;END=107234687;SVLEN=5987;AN=20618;AC=1;AF=4.850130953535746e-05;N_HET=1;N_HOMALT=0;N_HOMREF=10308;FREQ_HET=9.700261907071492e-05;FREQ_HOMALT=0.0;FREQ_HOMREF=0.9999029973809292;N_BI_GENOS=10309;North_Han_AN=2272;North_Han_AC=1;North_Han
chr1	107389492	.	T	<INS:ME:LINE1>	.	PASS	SVTYPE=INS:ME:LINE1;END=107389506;SVLEN=483;AN=20618;AC=1;AF=4.850130953535746e-05;N_HET=1;N_HOMALT=0;N_HOMREF=10308;FREQ_HET=9.700261907071492e-05;FREQ_HOMALT=0.0;FREQ_HOMREF=0.9999029973809292;N_BI_GENOS=10309;East_Han_AN=6554;East_Han_AC=1;East_Han_AF=
chr1	107425971	.	A	<INS:ME:LINE1>	.	PASS	SVTYPE=INS:ME:LINE1;END=107425971;SVLEN=25;AN=20618;AC=1;AF=4.850130953535746e-05;N_HET=1;N_HOMALT=0;N_HOMREF=10308;FREQ_HET=9.700261907071492e-05;FREQ_HOMALT=0.0;FREQ_HOMREF=0.9999029973809292;N_BI_GENOS=10309;East_Han_AN=6554;East_Han_AC=1;East_Han_AF=0
chr1	107427049	.	T	<INS:ME:LINE1>	.	PASS	SVTYPE=INS:ME:LINE1;END=107427049;SVLEN=139;AN=20618;AC=1;AF=4.850130953535746e-05;N_HET=1;N_HOMALT=0;N_HOMREF=10308;FREQ_HET=9.700261907071492e-05;FREQ_HOMALT=0.0;FREQ_HOMREF=0.9999029973809292;N_BI_GENOS=10309;East_Han_AN=6554;East_Han_AC=1;East_Han_AF=
chr1	107445407	.	A	<INS:ME:LINE1>	.	PASS	SVTYPE=INS:ME:LINE1;END=107445425;SVLEN=843;AN=20618;AC=1;AF=4.850130953535746e-05;N_HET=1;N_HOMALT=0;N_HOMREF=10308;FREQ_HET=9.700261907071492e-05;FREQ_HOMALT=0.0;FREQ_HOMREF=0.9999029973809292;N_BI_GENOS=10309;Central_Han_AN=2128;Central_Han_AC=1;Centra
chr1	107608088	.	A	<INS:ME:LINE1>	.	PASS	SVTYPE=INS:ME:LINE1;END=107608103;SVLEN=1005;AN=20618;AC=1;AF=4.850130953535746e-05;N_HET=1;N_HOMALT=0;N_HOMREF=10308;FREQ_HET=9.700261907071492e-05;FREQ_HOMALT=0.0;FREQ_HOMREF=0.9999029973809292;N_BI_GENOS=10309;Southeast_Han_AN=1908;Southeast_Han_AC=1;S
chr1	107838621	.	A	<INS:ME:LINE1>	.	PASS	SVTYPE=INS:ME:LINE1;END=107838636;SVLEN=692;AN=20618;AC=1;AF=4.850130953535746e-05;N_HET=1;N_HOMALT=0;N_HOMREF=10308;FREQ_HET=9.700261907071492e-05;FREQ_HOMALT=0.0;FREQ_HOMREF=0.9999029973809292;N_BI_GENOS=10309;East_Han_AN=6554;East_Han_AC=1;East_Han_AF=
chr1	107921630	.	T	<INS:ME:LINE1>	.	PASS	SVTYPE=INS:ME:LINE1;END=107921646;SVLEN=572;AN=20618;AC=1;AF=4.850130953535746e-05;N_HET=1;N_HOMALT=0;N_HOMREF=10308;FREQ_HET=9.700261907071492e-05;FREQ_HOMALT=0.0;FREQ_HOMREF=0.9999029973809292;N_BI_GENOS=10309;Central_Han_AN=2128;Central_Han_AC=1;Centra
chr1	108135595	.	T	<INS:ME:LINE1>	.	PASS	SVTYPE=INS:ME:LINE1;END=108135610;SVLEN=1189;AN=20618;AC=1;AF=4.850130953535746e-05;N_HET=1;N_HOMALT=0;N_HOMREF=10308;FREQ_HET=9.700261907071492e-05;FREQ_HOMALT=0.0;FREQ_HOMREF=0.9999029973809292;N_BI_GENOS=10309;Central_Han_AN=2128;Central_Han_AC=1;Centr
chr1	108918463	.	A	<INS:ME:LINE1>	.	PASS	SVTYPE=INS:ME:LINE1;END=108918478;SVLEN=1348;AN=20618;AC=5;AF=0.00024250654767678728;N_HET=5;N_HOMALT=0;N_HOMREF=10304;FREQ_HET=0.0004850130953535455;FREQ_HOMALT=0.0;FREQ_HOMREF=0.9995149869046465;N_BI_GENOS=10309;Lingnan_Han_AN=1888;Lingnan_Han_AC=1;Lin
chr1	109664457	.	C	<INS:ME:LINE1>	.	PASS	SVTYPE=INS:ME:LINE1;END=109664471;SVLEN=383;AN=20618;AC=4;AF=0.00019400523814142983;N_HET=2;N_HOMALT=1;N_HOMREF=10306;FREQ_HET=0.00019400523814142983;FREQ_HOMALT=9.700261907071492e-05;FREQ_HOMREF=0.9997089921427879;N_BI_GENOS=10309;East_Han_AN=6554;East_H
chr1	109792832	.	A	<INS:ME:LINE1>	.	PASS	SVTYPE=INS:ME:LINE1;END=109792845;SVLEN=6030;AN=20618;AC=1;AF=4.850130953535746e-05;N_HET=1;N_HOMALT=0;N_HOMREF=10308;FREQ_HET=9.700261907071492e-05;FREQ_HOMALT=0.0;FREQ_HOMREF=0.9999029973809292;N_BI_GENOS=10309;Lingnan_Han_AN=1888;Lingnan_Han_AC=1;Lingn
chr1	110718148	.	T	<INS:ME:LINE1>	.	PASS	SVTYPE=INS:ME:LINE1;END=110718183;SVLEN=1554;AN=20618;AC=1;AF=4.850130953535746e-05;N_HET=1;N_HOMALT=0;N_HOMREF=10308;FREQ_HET=9.700261907071492e-05;FREQ_HOMALT=0.0;FREQ_HOMREF=0.9999029973809292;N_BI_GENOS=10309;Mongolian_AN=454;Mongolian_AC=1;Mongolian_
chr1	110940981	.	T	<INS:ME:LINE1>	.	PASS	SVTYPE=INS:ME:LINE1;END=110940991;SVLEN=340;AN=20618;AC=3;AF=0.00014550392860607236;N_HET=3;N_HOMALT=0;N_HOMREF=10306;FREQ_HET=0.0002910078572121447;FREQ_HOMALT=0.0;FREQ_HOMREF=0.9997089921427879;N_BI_GENOS=10309;Lingnan_Han_AN=1888;Lingnan_Han_AC=2;Lingn
chr1	110985163	.	A	<INS:ME:LINE1>	.	PASS	SVTYPE=INS:ME:LINE1;END=110985179;SVLEN=617;AN=20618;AC=1;AF=4.850130953535746e-05;N_HET=1;N_HOMALT=0;N_HOMREF=10308;FREQ_HET=9.700261907071492e-05;FREQ_HOMALT=0.0;FREQ_HOMREF=0.9999029973809292;N_BI_GENOS=10309;Southeast_Han_AN=1908;Southeast_Han_AC=1;So
chr1	111049428	.	A	<INS:ME:LINE1>	.	PASS	SVTYPE=INS:ME:LINE1;END=111049428;SVLEN=96;AN=20618;AC=1;AF=4.850130953535746e-05;N_HET=1;N_HOMALT=0;N_HOMREF=10308;FREQ_HET=9.700261907071492e-05;FREQ_HOMALT=0.0;FREQ_HOMREF=0.9999029973809292;N_BI_GENOS=10309;East_Han_AN=6554;East_Han_AC=1;East_Han_AF=0
chr1	111123794	.	A	<INS:ME:LINE1>	.	PASS	SVTYPE=INS:ME:LINE1;END=111123811;SVLEN=1752;AN=20618;AC=109;AF=0.005286642739353963;N_HET=105;N_HOMALT=2;N_HOMREF=10202;FREQ_HET=0.010185275002425066;FREQ_HOMALT=0.00019400523814142983;FREQ_HOMREF=0.9896207197594336;N_BI_GENOS=10309;South_Han_AN=1496;Sou
chr1	111578156	.	T	<INS:ME:LINE1>	.	PASS	SVTYPE=INS:ME:LINE1;END=111578171;SVLEN=383;AN=20618;AC=1;AF=4.850130953535746e-05;N_HET=1;N_HOMALT=0;N_HOMREF=10308;FREQ_HET=9.700261907071492e-05;FREQ_HOMALT=0.0;FREQ_HOMREF=0.9999029973809292;N_BI_GENOS=10309;Southeast_Han_AN=1908;Southeast_Han_AC=1;So
chr1	111612169	.	A	<INS:ME:LINE1>	.	PASS	SVTYPE=INS:ME:LINE1;END=111612185;SVLEN=6077;AN=20618;AC=1;AF=4.850130953535746e-05;N_HET=1;N_HOMALT=0;N_HOMREF=10308;FREQ_HET=9.700261907071492e-05;FREQ_HOMALT=0.0;FREQ_HOMREF=0.9999029973809292;N_BI_GENOS=10309;Southeast_Han_AN=1908;Southeast_Han_AC=1;S
chr1	111792832	.	G	<INS:ME:LINE1>	.	PASS	SVTYPE=INS:ME:LINE1;END=111792845;SVLEN=5985;AN=20618;AC=2;AF=9.700261907071492e-05;N_HET=2;N_HOMALT=0;N_HOMREF=10307;FREQ_HET=0.00019400523814142983;FREQ_HOMALT=0.0;FREQ_HOMREF=0.9998059947618586;N_BI_GENOS=10309;Lingnan_Han_AN=1888;Lingnan_Han_AC=1;Ling
chr1	111868692	.	A	<INS:ME:LINE1>	.	PASS	SVTYPE=INS:ME:LINE1;END=111868708;SVLEN=6005;AN=20618;AC=1;AF=4.850130953535746e-05;N_HET=1;N_HOMALT=0;N_HOMREF=10308;FREQ_HET=9.700261907071492e-05;FREQ_HOMALT=0.0;FREQ_HOMREF=0.9999029973809292;N_BI_GENOS=10309;East_Han_AN=6554;East_Han_AC=1;East_Han_AF
chr1	111970463	.	C	<INS:ME:LINE1>	.	PASS	SVTYPE=INS:ME:LINE1;END=111970478;SVLEN=6002;AN=20618;AC=1;AF=4.850130953535746e-05;N_HET=1;N_HOMALT=0;N_HOMREF=10308;FREQ_HET=9.700261907071492e-05;FREQ_HOMALT=0.0;FREQ_HOMREF=0.9999029973809292;N_BI_GENOS=10309;East_Han_AN=6554;East_Han_AC=1;East_Han_AF
chr1	112088024	.	T	<INS:ME:LINE1>	.	PASS	SVTYPE=INS:ME:LINE1;END=112088035;SVLEN=1051;AN=20618;AC=1;AF=4.850130953535746e-05;N_HET=1;N_HOMALT=0;N_HOMREF=10308;FREQ_HET=9.700261907071492e-05;FREQ_HOMALT=0.0;FREQ_HOMREF=0.9999029973809292;N_BI_GENOS=10309;East_Han_AN=6554;East_Han_AC=1;East_Han_AF
chr1	112190595	.	A	<INS:ME:LINE1>	.	PASS	SVTYPE=INS:ME:LINE1;END=112190608;SVLEN=5998;AN=20618;AC=1;AF=4.850130953535746e-05;N_HET=1;N_HOMALT=0;N_HOMREF=10308;FREQ_HET=9.700261907071492e-05;FREQ_HOMALT=0.0;FREQ_HOMREF=0.9999029973809292;N_BI_GENOS=10309;Mongolian_AN=454;Mongolian_AC=1;Mongolian_
chr1	112197946	.	A	<INS:ME:LINE1>	.	PASS	SVTYPE=INS:ME:LINE1;END=112197963;SVLEN=896;AN=20618;AC=1;AF=4.850130953535746e-05;N_HET=1;N_HOMALT=0;N_HOMREF=10308;FREQ_HET=9.700261907071492e-05;FREQ_HOMALT=0.0;FREQ_HOMREF=0.9999029973809292;N_BI_GENOS=10309;Central_Han_AN=2128;Central_Han_AC=1;Centra
chr1	112286417	.	A	<INS:ME:LINE1>	.	PASS	SVTYPE=INS:ME:LINE1;END=112286419;SVLEN=649;AN=20618;AC=4;AF=0.00019400523814142983;N_HET=4;N_HOMALT=0;N_HOMREF=10305;FREQ_HET=0.00038801047628285966;FREQ_HOMALT=0.0;FREQ_HOMREF=0.9996119895237171;N_BI_GENOS=10309;Northwest_Han_AN=1296;Northwest_Han_AC=1;
chr1	113247269	.	A	<INS:ME:LINE1>	.	PASS	SVTYPE=INS:ME:LINE1;END=113247285;SVLEN=1592;AN=20618;AC=4;AF=0.00019400523814142983;N_HET=4;N_HOMALT=0;N_HOMREF=10305;FREQ_HET=0.00038801047628285966;FREQ_HOMALT=0.0;FREQ_HOMREF=0.9996119895237171;N_BI_GENOS=10309;East_Han_AN=6554;East_Han_AC=1;East_Han_
chr1	113331298	.	A	<INS:ME:LINE1>	.	PASS	SVTYPE=INS:ME:LINE1;END=113331314;SVLEN=6005;AN=20618;AC=6;AF=0.0002910078572121447;N_HET=6;N_HOMALT=0;N_HOMREF=10303;FREQ_HET=0.0005820157144242894;FREQ_HOMALT=0.0;FREQ_HOMREF=0.9994179842855757;N_BI_GENOS=10309;Northwest_Han_AN=1296;Northwest_Han_AC=1;N
chr1	113352142	.	T	<INS:ME:LINE1>	.	PASS	SVTYPE=INS:ME:LINE1;END=113352159;SVLEN=170;AN=20618;AC=1;AF=4.850130953535746e-05;N_HET=1;N_HOMALT=0;N_HOMREF=10308;FREQ_HET=9.700261907071492e-05;FREQ_HOMALT=0.0;FREQ_HOMREF=0.9999029973809292;N_BI_GENOS=10309;North_Han_AN=2272;North_Han_AC=1;North_Han_
chr1	113434257	.	T	<INS:ME:LINE1>	.	PASS	SVTYPE=INS:ME:LINE1;END=113434272;SVLEN=802;AN=20618;AC=4;AF=0.00019400523814142983;N_HET=4;N_HOMALT=0;N_HOMREF=10305;FREQ_HET=0.00038801047628285966;FREQ_HOMALT=0.0;FREQ_HOMREF=0.9996119895237171;N_BI_GENOS=10309;South_Han_AN=1496;South_Han_AC=1;South_Ha
chr1	113465342	.	T	<INS:ME:LINE1>	.	PASS	SVTYPE=INS:ME:LINE1;END=113465342;SVLEN=222;AN=20618;AC=1;AF=4.850130953535746e-05;N_HET=1;N_HOMALT=0;N_HOMREF=10308;FREQ_HET=9.700261907071492e-05;FREQ_HOMALT=0.0;FREQ_HOMREF=0.9999029973809292;N_BI_GENOS=10309;East_Han_AN=6554;East_Han_AC=1;East_Han_AF=
chr1	113503724	.	T	<INS:ME:LINE1>	.	PASS	SVTYPE=INS:ME:LINE1;END=113503748;SVLEN=5064;AN=20618;AC=3;AF=0.00014550392860607236;N_HET=3;N_HOMALT=0;N_HOMREF=10306;FREQ_HET=0.0002910078572121447;FREQ_HOMALT=0.0;FREQ_HOMREF=0.9997089921427879;N_BI_GENOS=10309;South_Han_AN=1496;South_Han_AC=1;South_Ha
chr1	113519933	.	T	<INS:ME:LINE1>	.	PASS	SVTYPE=INS:ME:LINE1;END=113519950;SVLEN=1240;AN=20618;AC=3;AF=0.00014550392860607236;N_HET=3;N_HOMALT=0;N_HOMREF=10306;FREQ_HET=0.0002910078572121447;FREQ_HOMALT=0.0;FREQ_HOMREF=0.9997089921427879;N_BI_GENOS=10309;Lingnan_Han_AN=1888;Lingnan_Han_AC=1;Ling
chr1	113545882	.	T	<INS:ME:LINE1>	.	PASS	SVTYPE=INS:ME:LINE1;END=113545899;SVLEN=4779;AN=20618;AC=1;AF=4.850130953535746e-05;N_HET=1;N_HOMALT=0;N_HOMREF=10308;FREQ_HET=9.700261907071492e-05;FREQ_HOMALT=0.0;FREQ_HOMREF=0.9999029973809292;N_BI_GENOS=10309;North_Han_AN=2272;North_Han_AC=1;North_Han
chr1	113616727	.	T	<INS:ME:LINE1>	.	PASS	SVTYPE=INS:ME:LINE1;END=113616731;SVLEN=359;AN=20618;AC=1;AF=4.850130953535746e-05;N_HET=1;N_HOMALT=0;N_HOMREF=10308;FREQ_HET=9.700261907071492e-05;FREQ_HOMALT=0.0;FREQ_HOMREF=0.9999029973809292;N_BI_GENOS=10309;East_Han_AN=6554;East_Han_AC=1;East_Han_AF=
chr1	113660320	.	T	<INS:ME:LINE1>	.	PASS	SVTYPE=INS:ME:LINE1;END=113660337;SVLEN=436;AN=20618;AC=2;AF=9.700261907071492e-05;N_HET=2;N_HOMALT=0;N_HOMREF=10307;FREQ_HET=0.00019400523814142983;FREQ_HOMALT=0.0;FREQ_HOMREF=0.9998059947618586;N_BI_GENOS=10309;Miao_AN=482;Miao_AC=2;Miao_AF=0.0041493775
chr1	114861502	.	A	<INS:ME:LINE1>	.	PASS	SVTYPE=INS:ME:LINE1;END=114861511;SVLEN=1065;AN=20618;AC=2;AF=9.700261907071492e-05;N_HET=2;N_HOMALT=0;N_HOMREF=10307;FREQ_HET=0.00019400523814142983;FREQ_HOMALT=0.0;FREQ_HOMREF=0.9998059947618586;N_BI_GENOS=10309;East_Han_AN=6554;East_Han_AC=2;East_Han_A
chr1	115029634	.	A	<INS:ME:LINE1>	.	PASS	SVTYPE=INS:ME:LINE1;END=115029649;SVLEN=1332;AN=20618;AC=2;AF=9.700261907071492e-05;N_HET=2;N_HOMALT=0;N_HOMREF=10307;FREQ_HET=0.00019400523814142983;FREQ_HOMALT=0.0;FREQ_HOMREF=0.9998059947618586;N_BI_GENOS=10309;East_Han_AN=6554;East_Han_AC=1;East_Han_A
chr1	115056305	.	T	<INS:ME:LINE1>	.	PASS	SVTYPE=INS:ME:LINE1;END=115056317;SVLEN=519;AN=20618;AC=35;AF=0.0016975458337375108;N_HET=33;N_HOMALT=1;N_HOMREF=10275;FREQ_HET=0.003201086429333592;FREQ_HOMALT=9.700261907071492e-05;FREQ_HOMREF=0.9967019109515957;N_BI_GENOS=10309;South_Han_AN=1496;South_
chr1	115372716	.	G	<INS:ME:LINE1>	.	PASS	SVTYPE=INS:ME:LINE1;END=115372730;SVLEN=920;AN=20618;AC=1;AF=4.850130953535746e-05;N_HET=1;N_HOMALT=0;N_HOMREF=10308;FREQ_HET=9.700261907071492e-05;FREQ_HOMALT=0.0;FREQ_HOMREF=0.9999029973809292;N_BI_GENOS=10309;Lingnan_Han_AN=1888;Lingnan_Han_AC=1;Lingna
chr1	115541895	.	A	<INS:ME:LINE1>	.	PASS	SVTYPE=INS:ME:LINE1;END=115541911;SVLEN=6002;AN=20618;AC=1;AF=4.850130953535746e-05;N_HET=1;N_HOMALT=0;N_HOMREF=10308;FREQ_HET=9.700261907071492e-05;FREQ_HOMALT=0.0;FREQ_HOMREF=0.9999029973809292;N_BI_GENOS=10309;North_Han_AN=2272;North_Han_AC=1;North_Han
chr1	115793537	.	A	<INS:ME:LINE1>	.	PASS	SVTYPE=INS:ME:LINE1;END=115793543;SVLEN=83;AN=20618;AC=1;AF=4.850130953535746e-05;N_HET=1;N_HOMALT=0;N_HOMREF=10308;FREQ_HET=9.700261907071492e-05;FREQ_HOMALT=0.0;FREQ_HOMREF=0.9999029973809292;N_BI_GENOS=10309;North_Han_AN=2272;North_Han_AC=1;North_Han_A
chr1	115847237	.	G	<INS:ME:LINE1>	.	PASS	SVTYPE=INS:ME:LINE1;END=115847250;SVLEN=548;AN=20618;AC=4;AF=0.00019400523814142983;N_HET=2;N_HOMALT=1;N_HOMREF=10306;FREQ_HET=0.00019400523814142983;FREQ_HOMALT=9.700261907071492e-05;FREQ_HOMREF=0.9997089921427879;N_BI_GENOS=10309;East_Han_AN=6554;East_H
chr1	115941871	.	A	<INS:ME:LINE1>	.	PASS	SVTYPE=INS:ME:LINE1;END=115941874;SVLEN=792;AN=20618;AC=5;AF=0.00024250654767678728;N_HET=5;N_HOMALT=0;N_HOMREF=10304;FREQ_HET=0.0004850130953535455;FREQ_HOMALT=0.0;FREQ_HOMREF=0.9995149869046465;N_BI_GENOS=10309;North_Han_AN=2272;North_Han_AC=1;North_Ha
chr1	116261738	.	G	<INS:ME:LINE1>	.	PASS	SVTYPE=INS:ME:LINE1;END=116261747;SVLEN=5998;AN=20618;AC=1;AF=4.850130953535746e-05;N_HET=1;N_HOMALT=0;N_HOMREF=10308;FREQ_HET=9.700261907071492e-05;FREQ_HOMALT=0.0;FREQ_HOMREF=0.9999029973809292;N_BI_GENOS=10309;North_Han_AN=2272;North_Han_AC=1;North_Han
chr1	116313949	.	A	<INS:ME:LINE1>	.	PASS	SVTYPE=INS:ME:LINE1;END=116313967;SVLEN=902;AN=20618;AC=4;AF=0.00019400523814142983;N_HET=4;N_HOMALT=0;N_HOMREF=10305;FREQ_HET=0.00038801047628285966;FREQ_HOMALT=0.0;FREQ_HOMREF=0.9996119895237171;N_BI_GENOS=10309;North_Han_AN=2272;North_Han_AC=1;North_Ha
chr1	116438205	.	T	<INS:ME:LINE1>	.	PASS	SVTYPE=INS:ME:LINE1;END=116438205;SVLEN=5998;AN=20618;AC=2;AF=9.700261907071492e-05;N_HET=2;N_HOMALT=0;N_HOMREF=10307;FREQ_HET=0.00019400523814142983;FREQ_HOMALT=0.0;FREQ_HOMREF=0.9998059947618586;N_BI_GENOS=10309;South_Han_AN=1496;South_Han_AC=1;South_Ha
chr1	116821148	.	T	<INS:ME:LINE1>	.	PASS	SVTYPE=INS:ME:LINE1;END=116821166;SVLEN=6005;AN=20618;AC=3;AF=0.00014550392860607236;N_HET=3;N_HOMALT=0;N_HOMREF=10306;FREQ_HET=0.0002910078572121447;FREQ_HOMALT=0.0;FREQ_HOMREF=0.9997089921427879;N_BI_GENOS=10309;North_Han_AN=2272;North_Han_AC=1;North_Ha
chr1	117259468	.	A	<INS:ME:LINE1>	.	PASS	SVTYPE=INS:ME:LINE1;END=117259468;SVLEN=0;AN=20618;AC=3;AF=0.00014550392860607236;N_HET=1;N_HOMALT=1;N_HOMREF=10307;FREQ_HET=9.700261907071492e-05;FREQ_HOMALT=9.700261907071492e-05;FREQ_HOMREF=0.9998059947618586;N_BI_GENOS=10309;East_Han_AN=6554;East_Han_
chr1	117411906	.	T	<INS:ME:LINE1>	.	PASS	SVTYPE=INS:ME:LINE1;END=117411908;SVLEN=767;AN=20618;AC=2;AF=9.700261907071492e-05;N_HET=0;N_HOMALT=1;N_HOMREF=10308;FREQ_HET=0.0;FREQ_HOMALT=9.700261907071492e-05;FREQ_HOMREF=0.9999029973809292;N_BI_GENOS=10309;North_Han_AN=2272;North_Han_AC=2;North_Han_
chr1	117731872	.	T	<INS:ME:LINE1>	.	PASS	SVTYPE=INS:ME:LINE1;END=117731872;SVLEN=0;AN=20618;AC=1;AF=4.850130953535746e-05;N_HET=1;N_HOMALT=0;N_HOMREF=10308;FREQ_HET=9.700261907071492e-05;FREQ_HOMALT=0.0;FREQ_HOMREF=0.9999029973809292;N_BI_GENOS=10309;North_Han_AN=2272;North_Han_AC=1;North_Han_AF
chr1	117916206	.	A	<INS:ME:LINE1>	.	PASS	SVTYPE=INS:ME:LINE1;END=117916221;SVLEN=92;AN=20618;AC=5;AF=0.00024250654767678728;N_HET=5;N_HOMALT=0;N_HOMREF=10304;FREQ_HET=0.0004850130953535455;FREQ_HOMALT=0.0;FREQ_HOMREF=0.9995149869046465;N_BI_GENOS=10309;East_Han_AN=6554;East_Han_AC=3;East_Han_AF
chr1	118263060	.	A	<INS:ME:LINE1>	.	PASS	SVTYPE=INS:ME:LINE1;END=118263061;SVLEN=307;AN=20618;AC=14;AF=0.0006790183334950044;N_HET=14;N_HOMALT=0;N_HOMREF=10295;FREQ_HET=0.0013580366669900088;FREQ_HOMALT=0.0;FREQ_HOMREF=0.99864196333301;N_BI_GENOS=10309;Northwest_Han_AN=1296;Northwest_Han_AC=3;No
chr1	118322088	.	T	<INS:ME:LINE1>	.	PASS	SVTYPE=INS:ME:LINE1;END=118322105;SVLEN=572;AN=20618;AC=1;AF=4.850130953535746e-05;N_HET=1;N_HOMALT=0;N_HOMREF=10308;FREQ_HET=9.700261907071492e-05;FREQ_HOMALT=0.0;FREQ_HOMREF=0.9999029973809292;N_BI_GENOS=10309;East_Han_AN=6554;East_Han_AC=1;East_Han_AF=
chr1	118492991	.	A	<INS:ME:LINE1>	.	PASS	SVTYPE=INS:ME:LINE1;END=118492997;SVLEN=5989;AN=20618;AC=8;AF=0.00038801047628285966;N_HET=8;N_HOMALT=0;N_HOMREF=10301;FREQ_HET=0.0007760209525657193;FREQ_HOMALT=0.0;FREQ_HOMREF=0.9992239790474343;N_BI_GENOS=10309;North_Han_AN=2272;North_Han_AC=1;North_Ha
chr1	118672268	.	G	<INS:ME:LINE1>	.	PASS	SVTYPE=INS:ME:LINE1;END=118672278;SVLEN=75;AN=20618;AC=2;AF=9.700261907071492e-05;N_HET=2;N_HOMALT=0;N_HOMREF=10307;FREQ_HET=0.00019400523814142983;FREQ_HOMALT=0.0;FREQ_HOMREF=0.9998059947618586;N_BI_GENOS=10309;North_Han_AN=2272;North_Han_AC=1;North_Han_
chr1	118686173	.	A	<INS:ME:LINE1>	.	PASS	SVTYPE=INS:ME:LINE1;END=118686182;SVLEN=5548;AN=20618;AC=3;AF=0.00014550392860607236;N_HET=1;N_HOMALT=1;N_HOMREF=10307;FREQ_HET=9.700261907071492e-05;FREQ_HOMALT=9.700261907071492e-05;FREQ_HOMREF=0.9998059947618586;N_BI_GENOS=10309;Central_Han_AN=2128;Cen
chr1	118716749	.	A	<INS:ME:LINE1>	.	PASS	SVTYPE=INS:ME:LINE1;END=118716764;SVLEN=66;AN=20618;AC=3;AF=0.00014550392860607236;N_HET=3;N_HOMALT=0;N_HOMREF=10306;FREQ_HET=0.0002910078572121447;FREQ_HOMALT=0.0;FREQ_HOMREF=0.9997089921427879;N_BI_GENOS=10309;Lingnan_Han_AN=1888;Lingnan_Han_AC=2;Lingna

chr1	118732192	.	T	<INS:ME:LINE1>	.	PASS	SVTYPE=INS:ME:LINE1;END=118732210;SVLEN=476;AN=20618;AC=21;AF=0.0010185275002425065;N_HET=21;N_HOMALT=0;N_HOMREF=10288;FREQ_HET=0.002037055000485013;FREQ_HOMALT=0.0;FREQ_HOMREF=0.997962944999515;N_BI_GENOS=10309;South_Han_AN=1496;South_Han_AC=1;South_Han_
chr1	118945563	.	G	<INS:ME:LINE1>	.	PASS	SVTYPE=INS:ME:LINE1;END=118945578;SVLEN=4592;AN=20618;AC=1;AF=4.850130953535746e-05;N_HET=1;N_HOMALT=0;N_HOMREF=10308;FREQ_HET=9.700261907071492e-05;FREQ_HOMALT=0.0;FREQ_HOMREF=0.9999029973809292;N_BI_GENOS=10309;East_Han_AN=6554;East_Han_AC=1;East_Han_AF
chr1	119010728	.	G	<INS:ME:LINE1>	.	PASS	SVTYPE=INS:ME:LINE1;END=119010743;SVLEN=978;AN=20618;AC=9672;AF=0.4691046658259773;N_HET=4642;N_HOMALT=2515;N_HOMREF=3152;FREQ_HET=0.45028615772625863;FREQ_HOMALT=0.243961586962848;FREQ_HOMREF=0.3057522553108934;N_BI_GENOS=10309;South_Han_AN=1496;South_Ha
chr1	119053214	.	A	<INS:ME:LINE1>	.	PASS	SVTYPE=INS:ME:LINE1;END=119053228;SVLEN=757;AN=20618;AC=1;AF=4.850130953535746e-05;N_HET=1;N_HOMALT=0;N_HOMREF=10308;FREQ_HET=9.700261907071492e-05;FREQ_HOMALT=0.0;FREQ_HOMREF=0.9999029973809292;N_BI_GENOS=10309;Miao_AN=482;Miao_AC=1;Miao_AF=0.00207468879
chr1	119108237	.	G	<INS:ME:LINE1>	.	PASS	SVTYPE=INS:ME:LINE1;END=119108251;SVLEN=6000;AN=20618;AC=1;AF=4.850130953535746e-05;N_HET=1;N_HOMALT=0;N_HOMREF=10308;FREQ_HET=9.700261907071492e-05;FREQ_HOMALT=0.0;FREQ_HOMREF=0.9999029973809292;N_BI_GENOS=10309;East_Han_AN=6554;East_Han_AC=1;East_Han_AF
chr1	119836605	.	T	<INS:ME:LINE1>	.	PASS	SVTYPE=INS:ME:LINE1;END=119836619;SVLEN=6052;AN=20618;AC=1;AF=4.850130953535746e-05;N_HET=1;N_HOMALT=0;N_HOMREF=10308;FREQ_HET=9.700261907071492e-05;FREQ_HOMALT=0.0;FREQ_HOMREF=0.9999029973809292;N_BI_GENOS=10309;Lingnan_Han_AN=1888;Lingnan_Han_AC=1;Lingn
chr1	119954588	.	T	<INS:ME:LINE1>	.	PASS	SVTYPE=INS:ME:LINE1;END=119954604;SVLEN=135;AN=20618;AC=5;AF=0.00024250654767678728;N_HET=5;N_HOMALT=0;N_HOMREF=10304;FREQ_HET=0.00048501309535357455;FREQ_HOMALT=0.0;FREQ_HOMREF=0.9995149869046465;N_BI_GENOS=10309;Central_Han_AN=2128;Central_Han_AC=3;Cent
chr1	120146353	.	T	<INS:ME:LINE1>	.	PASS	SVTYPE=INS:ME:LINE1;END=120146354;SVLEN=1078;AN=20618;AC=1;AF=4.850130953535746e-05;N_HET=1;N_HOMALT=0;N_HOMREF=10308;FREQ_HET=9.700261907071492e-05;FREQ_HOMALT=0.0;FREQ_HOMREF=0.9999029973809292;N_BI_GENOS=10309;East_Han_AN=6554;East_Han_AC=1;East_Han_AF
chr1	120449345	.	A	<INS:ME:LINE1>	.	PASS	SVTYPE=INS:ME:LINE1;END=120449358;SVLEN=1033;AN=20618;AC=1;AF=4.850130953535746e-05;N_HET=1;N_HOMALT=0;N_HOMREF=10308;FREQ_HET=9.700261907071492e-05;FREQ_HOMALT=0.0;FREQ_HOMREF=0.9999029973809292;N_BI_GENOS=10309;Central_Han_AN=2128;Central_Han_AC=1;Centr
chr1	121430646	.	A	<INS:ME:LINE1>	.	PASS	SVTYPE=INS:ME:LINE1;END=121430656;SVLEN=1915;AN=20618;AC=1;AF=4.850130953535746e-05;N_HET=1;N_HOMALT=0;N_HOMREF=10308;FREQ_HET=9.700261907071492e-05;FREQ_HOMALT=0.0;FREQ_HOMREF=0.9999029973809292;N_BI_GENOS=10309;Central_Han_AN=2128;Central_Han_AC=1;Centr
chr1	121594838	.	G	<INS:ME:LINE1>	.	PASS	SVTYPE=INS:ME:LINE1;END=121594847;SVLEN=5997;AN=20618;AC=1;AF=4.850130953535746e-05;N_HET=1;N_HOMALT=0;N_HOMREF=10308;FREQ_HET=9.700261907071492e-05;FREQ_HOMALT=0.0;FREQ_HOMREF=0.9999029973809292;N_BI_GENOS=10309;Central_Han_AN=2128;Central_Han_AC=1;Centr
chr1	122068692	.	T	<INS:ME:LINE1>	.	PASS	SVTYPE=INS:ME:LINE1;END=122068707;SVLEN=6001;AN=20618;AC=1;AF=4.850130953535746e-05;N_HET=1;N_HOMALT=0;N_HOMREF=10308;FREQ_HET=9.700261907071492e-05;FREQ_HOMALT=0.0;FREQ_HOMREF=0.9999029973809292;N_BI_GENOS=10309;East_Han_AN=6554;East_Han_AC=1;East_Han_AF
chr1	124984105	.	A	<INS:ME:LINE1>	.	PASS	SVTYPE=INS:ME:LINE1;END=124984117;SVLEN=3143;AN=20618;AC=1;AF=4.850130953535746e-05;N_HET=1;N_HOMALT=0;N_HOMREF=10308;FREQ_HET=9.700261907071492e-05;FREQ_HOMALT=0.0;FREQ_HOMREF=0.9999029973809292;N_BI_GENOS=10309;Central_Han_AN=2128;Central_Han_AC=1;Centr
chr1	124991880	.	T	<INS:ME:LINE1>	.	PASS	SVTYPE=INS:ME:LINE1;END=124991892;SVLEN=974;AN=20618;AC=5;AF=0.00024250654767678728;N_HET=5;N_HOMALT=0;N_HOMREF=10304;FREQ_HET=0.00048501309535357455;FREQ_HOMALT=0.0;FREQ_HOMREF=0.9995149869046465;N_BI_GENOS=10309;East_Han_AN=6554;East_Han_AC=4;East_Han_A
chr1	125045950	.	A	<INS:ME:LINE1>	.	PASS	SVTYPE=INS:ME:LINE1;END=125045950;SVLEN=1591;AN=20618;AC=7;AF=0.0003395091667475022;N_HET=7;N_HOMALT=0;N_HOMREF=10302;FREQ_HET=0.0006790183334950044;FREQ_HOMALT=0.0;FREQ_HOMREF=0.999320981666505;N_BI_GENOS=10309;Lingnan_Han_AN=1888;Lingnan_Han_AC=1;Lingna
chr1	125132297	.	G	<INS:ME:LINE1>	.	PASS	SVTYPE=INS:ME:LINE1;END=125132297;SVLEN=0;AN=20618;AC=18;AF=0.0008730235716364342;N_HET=18;N_HOMALT=0;N_HOMREF=10291;FREQ_HET=0.0017460471432728683;FREQ_HOMALT=0.0;FREQ_HOMREF=0.9982539528567271;N_BI_GENOS=10309;Northwest_Han_AN=1296;Northwest_Han_AC=1;No
chr1	125132487	.	T	<INS:ME:LINE1>	.	PASS	SVTYPE=INS:ME:LINE1;END=125132487;SVLEN=0;AN=20618;AC=1;AF=4.850130953535746e-05;N_HET=1;N_HOMALT=0;N_HOMREF=10308;FREQ_HET=9.700261907071492e-05;FREQ_HOMALT=0.0;FREQ_HOMREF=0.9999029973809292;N_BI_GENOS=10309;Manchu_AN=416;Manchu_AC=1;Manchu_AF=0.0024038
chr1	143574023	.	G	<INS:ME:LINE1>	.	PASS	SVTYPE=INS:ME:LINE1;END=143574038;SVLEN=888;AN=20618;AC=10;AF=0.00048501309535357455;N_HET=10;N_HOMALT=0;N_HOMREF=10299;FREQ_HET=0.0009700261907071491;FREQ_HOMALT=0.0;FREQ_HOMREF=0.9990299738092928;N_BI_GENOS=10309;South_Han_AN=1496;South_Han_AC=1;South_H
chr1	145754694	.	A	<INS:ME:LINE1>	.	PASS	SVTYPE=INS:ME:LINE1;END=145754700;SVLEN=2190;AN=20618;AC=1;AF=4.850130953535746e-05;N_HET=1;N_HOMALT=0;N_HOMREF=10308;FREQ_HET=9.700261907071492e-05;FREQ_HOMALT=0.0;FREQ_HOMREF=0.9999029973809292;N_BI_GENOS=10309;Northwest_Han_AN=1296;Northwest_Han_AC=1;N
chr1	146906472	.	G	<INS:ME:LINE1>	.	PASS	SVTYPE=INS:ME:LINE1;END=146906488;SVLEN=5993;AN=20618;AC=1;AF=4.850130953535746e-05;N_HET=1;N_HOMALT=0;N_HOMREF=10308;FREQ_HET=9.700261907071492e-05;FREQ_HOMALT=0.0;FREQ_HOMREF=0.9999029973809292;N_BI_GENOS=10309;Northwest_Han_AN=1296;Northwest_Han_AC=1;N
chr1	147042080	.	G	<INS:ME:LINE1>	.	PASS	SVTYPE=INS:ME:LINE1;END=147042096;SVLEN=1247;AN=20618;AC=1;AF=4.850130953535746e-05;N_HET=1;N_HOMALT=0;N_HOMREF=10308;FREQ_HET=9.700261907071492e-05;FREQ_HOMALT=0.0;FREQ_HOMREF=0.9999029973809292;N_BI_GENOS=10309;Southeast_Han_AN=1908;Southeast_Han_AC=1;S
chr1	147136108	.	A	<INS:ME:LINE1>	.	PASS	SVTYPE=INS:ME:LINE1;END=147136121;SVLEN=5969;AN=20618;AC=1;AF=4.850130953535746e-05;N_HET=1;N_HOMALT=0;N_HOMREF=10308;FREQ_HET=9.700261907071492e-05;FREQ_HOMALT=0.0;FREQ_HOMREF=0.9999029973809292;N_BI_GENOS=10309;Yi_AN=422;Yi_AC=1;Yi_AF=0.0023696682464454
chr1	147210220	.	A	<INS:ME:LINE1>	.	PASS	SVTYPE=INS:ME:LINE1;END=147210235;SVLEN=1760;AN=20618;AC=1;AF=4.850130953535746e-05;N_HET=1;N_HOMALT=0;N_HOMREF=10308;FREQ_HET=9.700261907071492e-05;FREQ_HOMALT=0.0;FREQ_HOMREF=0.9999029973809292;N_BI_GENOS=10309;East_Han_AN=6554;East_Han_AC=1;East_Han_AF
chr1	147225890	.	A	<INS:ME:LINE1>	.	PASS	SVTYPE=INS:ME:LINE1;END=147225891;SVLEN=1067;AN=20618;AC=1;AF=4.850130953535746e-05;N_HET=1;N_HOMALT=0;N_HOMREF=10308;FREQ_HET=9.700261907071492e-05;FREQ_HOMALT=0.0;FREQ_HOMREF=0.9999029973809292;N_BI_GENOS=10309;Northwest_Han_AN=1296;Northwest_Han_AC=1;N
chr1	147727037	.	G	<INS:ME:LINE1>	.	PASS	SVTYPE=INS:ME:LINE1;END=147727050;SVLEN=6002;AN=20618;AC=1;AF=4.850130953535746e-05;N_HET=1;N_HOMALT=0;N_HOMREF=10308;FREQ_HET=9.700261907071492e-05;FREQ_HOMALT=0.0;FREQ_HOMREF=0.9999029973809292;N_BI_GENOS=10309;Southeast_Han_AN=1908;Southeast_Han_AC=1;S
chr1	147764980	.	C	<INS:ME:LINE1>	.	PASS	SVTYPE=INS:ME:LINE1;END=147764996;SVLEN=6000;AN=20618;AC=1;AF=4.850130953535746e-05;N_HET=1;N_HOMALT=0;N_HOMREF=10308;FREQ_HET=9.700261907071492e-05;FREQ_HOMALT=0.0;FREQ_HOMREF=0.9999029973809292;N_BI_GENOS=10309;Hui_AN=444;Hui_AC=1;Hui_AF=0.0022522522522
chr1	148404472	.	A	<INS:ME:LINE1>	.	PASS	SVTYPE=INS:ME:LINE1;END=148404472;SVLEN=0;AN=20618;AC=22;AF=0.001067028809777864;N_HET=22;N_HOMALT=0;N_HOMREF=10287;FREQ_HET=0.002134057619555728;FREQ_HOMALT=0.0;FREQ_HOMREF=0.9978659423804442;N_BI_GENOS=10309;South_Han_AN=1496;South_Han_AC=3;South_Han_AF
chr1	149977826	.	T	<INS:ME:LINE1>	.	PASS	SVTYPE=INS:ME:LINE1;END=149977842;SVLEN=6002;AN=20618;AC=1;AF=4.850130953535746e-05;N_HET=1;N_HOMALT=0;N_HOMREF=10308;FREQ_HET=9.700261907071492e-05;FREQ_HOMALT=0.0;FREQ_HOMREF=0.9999029973809292;N_BI_GENOS=10309;North_Han_AN=2272;North_Han_AC=1;North_Han
chr1	152090632	.	G	<INS:ME:LINE1>	.	PASS	SVTYPE=INS:ME:LINE1;END=152090647;SVLEN=274;AN=20618;AC=1;AF=4.850130953535746e-05;N_HET=1;N_HOMALT=0;N_HOMREF=10308;FREQ_HET=9.700261907071492e-05;FREQ_HOMALT=0.0;FREQ_HOMREF=0.9999029973809292;N_BI_GENOS=10309;South_Han_AN=1496;South_Han_AC=1;South_Han_
chr1	152374723	.	C	<INS:ME:LINE1>	.	PASS	SVTYPE=INS:ME:LINE1;END=152374723;SVLEN=565;AN=20618;AC=1;AF=4.850130953535746e-05;N_HET=1;N_HOMALT=0;N_HOMREF=10308;FREQ_HET=9.700261907071492e-05;FREQ_HOMALT=0.0;FREQ_HOMREF=0.9999029973809292;N_BI_GENOS=10309;North_Han_AN=2272;North_Han_AC=1;North_Han_
chr1	152471408	.	T	<INS:ME:LINE1>	.	PASS	SVTYPE=INS:ME:LINE1;END=152471419;SVLEN=6055;AN=20618;AC=499;AF=0.02420215345814337;N_HET=249;N_HOMALT=125;N_HOMREF=9935;FREQ_HET=0.024153652148608012;FREQ_HOMALT=0.012125327383839363;FREQ_HOMREF=0.9637210204675526;N_BI_GENOS=10309;South_Han_AN=1496;South
chr1	152583066	.	T	<INS:ME:LINE1>	.	PASS	SVTYPE=INS:ME:LINE1;END=152583066;SVLEN=2314;AN=20618;AC=7;AF=0.0003395091667475022;N_HET=5;N_HOMALT=1;N_HOMREF=10303;FREQ_HET=0.00048501309535357455;FREQ_HOMALT=9.700261907071492e-05;FREQ_HOMREF=0.9994179842855757;N_BI_GENOS=10309;Northwest_Han_AN=1296;N
chr1	152644888	.	A	<INS:ME:LINE1>	.	PASS	SVTYPE=INS:ME:LINE1;END=152644900;SVLEN=48;AN=20618;AC=1;AF=4.850130953535746e-05;N_HET=1;N_HOMALT=0;N_HOMREF=10308;FREQ_HET=9.700261907071492e-05;FREQ_HOMALT=0.0;FREQ_HOMREF=0.9999029973809292;N_BI_GENOS=10309;Southeast_Han_AN=1908;Southeast_Han_AC=1;Sou
chr1	152856125	.	C	<INS:ME:LINE1>	.	PASS	SVTYPE=INS:ME:LINE1;END=152856140;SVLEN=1500;AN=20618;AC=1;AF=4.850130953535746e-05;N_HET=1;N_HOMALT=0;N_HOMREF=10308;FREQ_HET=9.700261907071492e-05;FREQ_HOMALT=0.0;FREQ_HOMREF=0.9999029973809292;N_BI_GENOS=10309;Zhuang_AN=444;Zhuang_AC=1;Zhuang_AF=0.0022
chr1	153041660	.	A	<INS:ME:LINE1>	.	PASS	SVTYPE=INS:ME:LINE1;END=153041676;SVLEN=1326;AN=20618;AC=1;AF=4.850130953535746e-05;N_HET=1;N_HOMALT=0;N_HOMREF=10308;FREQ_HET=9.700261907071492e-05;FREQ_HOMALT=0.0;FREQ_HOMREF=0.9999029973809292;N_BI_GENOS=10309;North_Han_AN=2272;North_Han_AC=1;North_Han
chr1	153138175	.	A	<INS:ME:LINE1>	.	PASS	SVTYPE=INS:ME:LINE1;END=153138190;SVLEN=5997;AN=20618;AC=2;AF=9.700261907071492e-05;N_HET=2;N_HOMALT=0;N_HOMREF=10307;FREQ_HET=0.00019400523814142983;FREQ_HOMALT=0.0;FREQ_HOMREF=0.9998059947618586;N_BI_GENOS=10309;Northwest_Han_AN=1296;Northwest_Han_AC=2;
chr1	153466043	.	T	<INS:ME:LINE1>	.	PASS	SVTYPE=INS:ME:LINE1;END=153466059;SVLEN=5998;AN=20618;AC=3;AF=0.00014550392860607236;N_HET=3;N_HOMALT=0;N_HOMREF=10306;FREQ_HET=0.0002910078572121447;FREQ_HOMALT=0.0;FREQ_HOMREF=0.9997089921427879;N_BI_GENOS=10309;Northwest_Han_AN=1296;Northwest_Han_AC=1;
chr1	154259058	.	A	<INS:ME:LINE1>	.	PASS	SVTYPE=INS:ME:LINE1;END=154259071;SVLEN=560;AN=20618;AC=1;AF=4.850130953535746e-05;N_HET=1;N_HOMALT=0;N_HOMREF=10308;FREQ_HET=9.700261907071492e-05;FREQ_HOMALT=0.0;FREQ_HOMREF=0.9999029973809292;N_BI_GENOS=10309;Southeast_Han_AN=1908;Southeast_Han_AC=1;So
chr1	154680736	.	T	<INS:ME:LINE1>	.	PASS	SVTYPE=INS:ME:LINE1;END=154680751;SVLEN=991;AN=20618;AC=1;AF=4.850130953535746e-05;N_HET=1;N_HOMALT=0;N_HOMREF=10308;FREQ_HET=9.700261907071492e-05;FREQ_HOMALT=0.0;FREQ_HOMREF=0.9999029973809292;N_BI_GENOS=10309;North_Han_AN=2272;North_Han_AC=1;North_Han_
chr1	154888631	.	A	<INS:ME:LINE1>	.	PASS	SVTYPE=INS:ME:LINE1;END=154888647;SVLEN=1179;AN=20618;AC=1;AF=4.850130953535746e-05;N_HET=1;N_HOMALT=0;N_HOMREF=10308;FREQ_HET=9.700261907071492e-05;FREQ_HOMALT=0.0;FREQ_HOMREF=0.9999029973809292;N_BI_GENOS=10309;Southeast_Han_AN=1908;Southeast_Han_AC=1;S
chr1	155455906	.	G	<INS:ME:LINE1>	.	PASS	SVTYPE=INS:ME:LINE1;END=155455916;SVLEN=446;AN=20618;AC=1;AF=4.850130953535746e-05;N_HET=1;N_HOMALT=0;N_HOMREF=10308;FREQ_HET=9.700261907071492e-05;FREQ_HOMALT=0.0;FREQ_HOMREF=0.9999029973809292;N_BI_GENOS=10309;Northwest_Han_AN=1296;Northwest_Han_AC=1;No
chr1	155741130	.	A	<INS:ME:LINE1>	.	PASS	SVTYPE=INS:ME:LINE1;END=155741140;SVLEN=2091;AN=20618;AC=1;AF=4.850130953535746e-05;N_HET=1;N_HOMALT=0;N_HOMREF=10308;FREQ_HET=9.700261907071492e-05;FREQ_HOMALT=0.0;FREQ_HOMREF=0.9999029973809292;N_BI_GENOS=10309;Central_Han_AN=2128;Central_Han_AC=1;Centr
chr1	157442109	.	G	<INS:ME:LINE1>	.	PASS	SVTYPE=INS:ME:LINE1;END=157442124;SVLEN=890;AN=20618;AC=1;AF=4.850130953535746e-05;N_HET=1;N_HOMALT=0;N_HOMREF=10308;FREQ_HET=9.700261907071492e-05;FREQ_HOMALT=0.0;FREQ_HOMREF=0.9999029973809292;N_BI_GENOS=10309;East_Han_AN=6554;East_Han_AC=1;East_Han_AF=
chr1	157684139	.	T	<INS:ME:LINE1>	.	PASS	SVTYPE=INS:ME:LINE1;END=157684154;SVLEN=109;AN=20618;AC=2;AF=9.700261907071492e-05;N_HET=2;N_HOMALT=0;N_HOMREF=10307;FREQ_HET=0.00019400523814142983;FREQ_HOMALT=0.0;FREQ_HOMREF=0.9998059947618586;N_BI_GENOS=10309;Lingnan_Han_AN=1888;Lingnan_Han_AC=1;Lingn
chr1	157685019	.	G	<INS:ME:LINE1>	.	PASS	SVTYPE=INS:ME:LINE1;END=157685034;SVLEN=375;AN=20618;AC=2;AF=9.700261907071492e-05;N_HET=2;N_HOMALT=0;N_HOMREF=10307;FREQ_HET=0.00019400523814142983;FREQ_HOMALT=0.0;FREQ_HOMREF=0.9998059947618586;N_BI_GENOS=10309;South_Han_AN=1496;South_Han_AC=1;South_Han
chr1	157831508	.	C	<INS:ME:LINE1>	.	PASS	SVTYPE=INS:ME:LINE1;END=157831522;SVLEN=270;AN=20618;AC=1;AF=4.850130953535746e-05;N_HET=1;N_HOMALT=0;N_HOMREF=10308;FREQ_HET=9.700261907071492e-05;FREQ_HOMALT=0.0;FREQ_HOMREF=0.9999029973809292;N_BI_GENOS=10309;Northwest_Han_AN=1296;Northwest_Han_AC=1;No
chr1	157858860	.	A	<INS:ME:LINE1>	.	PASS	SVTYPE=INS:ME:LINE1;END=157858875;SVLEN=5989;AN=20618;AC=1;AF=4.850130953535746e-05;N_HET=1;N_HOMALT=0;N_HOMREF=10308;FREQ_HET=9.700261907071492e-05;FREQ_HOMALT=0.0;FREQ_HOMREF=0.9999029973809292;N_BI_GENOS=10309;East_Han_AN=6554;East_Han_AC=1;East_Han_AF
chr1	157865325	.	A	<INS:ME:LINE1>	.	PASS	SVTYPE=INS:ME:LINE1;END=157865341;SVLEN=6038;AN=20618;AC=1;AF=4.850130953535746e-05;N_HET=1;N_HOMALT=0;N_HOMREF=10308;FREQ_HET=9.700261907071492e-05;FREQ_HOMALT=0.0;FREQ_HOMREF=0.9999029973809292;N_BI_GENOS=10309;Lingnan_Han_AN=1888;Lingnan_Han_AC=1;Lingn
chr1	158593664	.	T	<INS:ME:LINE1>	.	PASS	SVTYPE=INS:ME:LINE1;END=158593674;SVLEN=1336;AN=20618;AC=2;AF=9.700261907071492e-05;N_HET=2;N_HOMALT=0;N_HOMREF=10307;FREQ_HET=0.00019400523814142983;FREQ_HOMALT=0.0;FREQ_HOMREF=0.9998059947618586;N_BI_GENOS=10309;Central_Han_AN=2128;Central_Han_AC=2;Cent
chr1	158665964	.	A	<INS:ME:LINE1>	.	PASS	SVTYPE=INS:ME:LINE1;END=158665973;SVLEN=693;AN=20618;AC=1;AF=4.850130953535746e-05;N_HET=1;N_HOMALT=0;N_HOMREF=10308;FREQ_HET=9.700261907071492e-05;FREQ_HOMALT=0.0;FREQ_HOMREF=0.9999029973809292;N_BI_GENOS=10309;Central_Han_AN=2128;Central_Han_AC=1;Centra
chr1	158719157	.	A	<INS:ME:LINE1>	.	PASS	SVTYPE=INS:ME:LINE1;END=158719173;SVLEN=1314;AN=20618;AC=1;AF=4.850130953535746e-05;N_HET=1;N_HOMALT=0;N_HOMREF=10308;FREQ_HET=9.700261907071492e-05;FREQ_HOMALT=0.0;FREQ_HOMREF=0.9999029973809292;N_BI_GENOS=10309;North_Han_AN=2272;North_Han_AC=1;North_Han
chr1	158767624	.	A	<INS:ME:LINE1>	.	PASS	SVTYPE=INS:ME:LINE1;END=158767637;SVLEN=450;AN=20618;AC=1;AF=4.850130953535746e-05;N_HET=1;N_HOMALT=0;N_HOMREF=10308;FREQ_HET=9.700261907071492e-05;FREQ_HOMALT=0.0;FREQ_HOMREF=0.9999029973809292;N_BI_GENOS=10309;Zhuang_AN=444;Zhuang_AC=1;Zhuang_AF=0.00225
chr1	158840031	.	G	<INS:ME:LINE1>	.	PASS	SVTYPE=INS:ME:LINE1;END=158840047;SVLEN=502;AN=20618;AC=1;AF=4.850130953535746e-05;N_HET=1;N_HOMALT=0;N_HOMREF=10308;FREQ_HET=9.700261907071492e-05;FREQ_HOMALT=0.0;FREQ_HOMREF=0.9999029973809292;N_BI_GENOS=10309;East_Han_AN=6554;East_Han_AC=1;East_Han_AF=
chr1	158851864	.	A	<INS:ME:LINE1>	.	PASS	SVTYPE=INS:ME:LINE1;END=158851864;SVLEN=945;AN=20618;AC=1;AF=4.850130953535746e-05;N_HET=1;N_HOMALT=0;N_HOMREF=10308;FREQ_HET=9.700261907071492e-05;FREQ_HOMALT=0.0;FREQ_HOMREF=0.9999029973809292;N_BI_GENOS=10309;East_Han_AN=6554;East_Han_AC=1;East_Han_AF=
chr1	158852887	.	G	<INS:ME:LINE1>	.	PASS	SVTYPE=INS:ME:LINE1;END=158852930;SVLEN=525;AN=20618;AC=1;AF=4.850130953535746e-05;N_HET=1;N_HOMALT=0;N_HOMREF=10308;FREQ_HET=9.700261907071492e-05;FREQ_HOMALT=0.0;FREQ_HOMREF=0.9999029973809292;N_BI_GENOS=10309;East_Han_AN=6554;East_Han_AC=1;East_Han_AF=
chr1	158917509	.	G	<INS:ME:LINE1>	.	PASS	SVTYPE=INS:ME:LINE1;END=158917524;SVLEN=5998;AN=20618;AC=1;AF=4.850130953535746e-05;N_HET=1;N_HOMALT=0;N_HOMREF=10308;FREQ_HET=9.700261907071492e-05;FREQ_HOMALT=0.0;FREQ_HOMREF=0.9999029973809292;N_BI_GENOS=10309;Northwest_Han_AN=1296;Northwest_Han_AC=1;N
chr1	158968345	.	C	<INS:ME:LINE1>	.	PASS	SVTYPE=INS:ME:LINE1;END=158968418;SVLEN=694;AN=20618;AC=1;AF=4.850130953535746e-05;N_HET=1;N_HOMALT=0;N_HOMREF=10308;FREQ_HET=9.700261907071492e-05;FREQ_HOMALT=0.0;FREQ_HOMREF=0.9999029973809292;N_BI_GENOS=10309;East_Han_AN=6554;East_Han_AC=1;East_Han_AF=
chr1	159010953	.	T	<INS:ME:LINE1>	.	PASS	SVTYPE=INS:ME:LINE1;END=159010957;SVLEN=1839;AN=20618;AC=1;AF=4.850130953535746e-05;N_HET=1;N_HOMALT=0;N_HOMREF=10308;FREQ_HET=9.700261907071492e-05;FREQ_HOMALT=0.0;FREQ_HOMREF=0.9999029973809292;N_BI_GENOS=10309;Manchu_AN=416;Manchu_AC=1;Manchu_AF=0.0024
chr1	159040094	.	C	<INS:ME:LINE1>	.	PASS	SVTYPE=INS:ME:LINE1;END=159040107;SVLEN=5993;AN=20618;AC=2;AF=9.700261907071492e-05;N_HET=2;N_HOMALT=0;N_HOMREF=10307;FREQ_HET=0.00019400523814142983;FREQ_HOMALT=0.0;FREQ_HOMREF=0.9998059947618586;N_BI_GENOS=10309;Lingnan_Han_AN=1888;Lingnan_Han_AC=1;Ling
chr1	159303310	.	A	<INS:ME:LINE1>	.	PASS	SVTYPE=INS:ME:LINE1;END=159303326;SVLEN=469;AN=20618;AC=1;AF=4.850130953535746e-05;N_HET=1;N_HOMALT=0;N_HOMREF=10308;FREQ_HET=9.700261907071492e-05;FREQ_HOMALT=0.0;FREQ_HOMREF=0.9999029973809292;N_BI_GENOS=10309;Southeast_Han_AN=1908;Southeast_Han_AC=1;So
chr1	159414764	.	G	<INS:ME:LINE1>	.	PASS	SVTYPE=INS:ME:LINE1;END=159414764;SVLEN=429;AN=20618;AC=1;AF=4.850130953535746e-05;N_HET=1;N_HOMALT=0;N_HOMREF=10308;FREQ_HET=9.700261907071492e-05;FREQ_HOMALT=0.0;FREQ_HOMREF=0.9999029973809292;N_BI_GENOS=10309;East_Han_AN=6554;East_Han_AC=1;East_Han_AF=
chr1	159414917	.	A	<INS:ME:LINE1>	.	PASS	SVTYPE=INS:ME:LINE1;END=159414917;SVLEN=256;AN=20618;AC=1;AF=4.850130953535746e-05;N_HET=1;N_HOMALT=0;N_HOMREF=10308;FREQ_HET=9.700261907071492e-05;FREQ_HOMALT=0.0;FREQ_HOMREF=0.9999029973809292;N_BI_GENOS=10309;East_Han_AN=6554;East_Han_AC=1;East_Han_AF=
chr1	159503478	.	A	<INS:ME:LINE1>	.	PASS	SVTYPE=INS:ME:LINE1;END=159503495;SVLEN=1844;AN=20618;AC=2;AF=9.700261907071492e-05;N_HET=2;N_HOMALT=0;N_HOMREF=10307;FREQ_HET=0.00019400523814142983;FREQ_HOMALT=0.0;FREQ_HOMREF=0.9998059947618586;N_BI_GENOS=10309;North_Han_AN=2272;North_Han_AC=1;North_Ha
chr1	159511590	.	T	<INS:ME:LINE1>	.	PASS	SVTYPE=INS:ME:LINE1;END=159511604;SVLEN=6025;AN=20618;AC=2;AF=9.700261907071492e-05;N_HET=2;N_HOMALT=0;N_HOMREF=10307;FREQ_HET=0.00019400523814142983;FREQ_HOMALT=0.0;FREQ_HOMREF=0.9998059947618586;N_BI_GENOS=10309;East_Han_AN=6554;East_Han_AC=2;East_Han_A
chr1	160315664	.	C	<INS:ME:LINE1>	.	PASS	SVTYPE=INS:ME:LINE1;END=160315664;SVLEN=8;AN=20618;AC=1;AF=4.850130953535746e-05;N_HET=1;N_HOMALT=0;N_HOMREF=10308;FREQ_HET=9.700261907071492e-05;FREQ_HOMALT=0.0;FREQ_HOMREF=0.9999029973809292;N_BI_GENOS=10309;Yi_AN=422;Yi_AC=1;Yi_AF=0.002369668246445498;
chr1	161618290	.	T	<INS:ME:LINE1>	.	PASS	SVTYPE=INS:ME:LINE1;END=161618290;SVLEN=0;AN=20618;AC=1;AF=4.850130953535746e-05;N_HET=1;N_HOMALT=0;N_HOMREF=10308;FREQ_HET=9.700261907071492e-05;FREQ_HOMALT=0.0;FREQ_HOMREF=0.9999029973809292;N_BI_GENOS=10309;Southeast_Han_AN=1908;Southeast_Han_AC=1;Sout
chr1	161785396	.	A	<INS:ME:LINE1>	.	PASS	SVTYPE=INS:ME:LINE1;END=161785415;SVLEN=1201;AN=20618;AC=4;AF=0.00019400523814142983;N_HET=2;N_HOMALT=1;N_HOMREF=10306;FREQ_HET=0.00019400523814142983;FREQ_HOMALT=9.700261907071492e-05;FREQ_HOMREF=0.9997089921427879;N_BI_GENOS=10309;East_Han_AN=6554;East_
chr1	161855748	.	A	<INS:ME:LINE1>	.	PASS	SVTYPE=INS:ME:LINE1;END=161855760;SVLEN=1498;AN=20618;AC=2;AF=9.700261907071492e-05;N_HET=2;N_HOMALT=0;N_HOMREF=10307;FREQ_HET=0.00019400523814142983;FREQ_HOMALT=0.0;FREQ_HOMREF=0.9998059947618586;N_BI_GENOS=10309;Central_Han_AN=2128;Central_Han_AC=2;Cent
chr1	161953206	.	T	<INS:ME:LINE1>	.	PASS	SVTYPE=INS:ME:LINE1;END=161953206;SVLEN=5972;AN=20618;AC=2;AF=9.700261907071492e-05;N_HET=2;N_HOMALT=0;N_HOMREF=10307;FREQ_HET=0.00019400523814142983;FREQ_HOMALT=0.0;FREQ_HOMREF=0.9998059947618586;N_BI_GENOS=10309;Northwest_Han_AN=1296;Northwest_Han_AC=1;
chr1	161990676	.	G	<INS:ME:LINE1>	.	PASS	SVTYPE=INS:ME:LINE1;END=161990687;SVLEN=6000;AN=20618;AC=1;AF=4.850130953535746e-05;N_HET=1;N_HOMALT=0;N_HOMREF=10308;FREQ_HET=9.700261907071492e-05;FREQ_HOMALT=0.0;FREQ_HOMREF=0.9999029973809292;N_BI_GENOS=10309;Central_Han_AN=2128;Central_Han_AC=1;Centr
chr1	162089893	.	A	<INS:ME:LINE1>	.	PASS	SVTYPE=INS:ME:LINE1;END=162089907;SVLEN=6018;AN=20618;AC=1;AF=4.850130953535746e-05;N_HET=1;N_HOMALT=0;N_HOMREF=10308;FREQ_HET=9.700261907071492e-05;FREQ_HOMALT=0.0;FREQ_HOMREF=0.9999029973809292;N_BI_GENOS=10309;Northwest_Han_AN=1296;Northwest_Han_AC=1;N
chr1	162132664	.	G	<INS:ME:LINE1>	.	PASS	SVTYPE=INS:ME:LINE1;END=162132678;SVLEN=1012;AN=20618;AC=1;AF=4.850130953535746e-05;N_HET=1;N_HOMALT=0;N_HOMREF=10308;FREQ_HET=9.700261907071492e-05;FREQ_HOMALT=0.0;FREQ_HOMREF=0.9999029973809292;N_BI_GENOS=10309;Lingnan_Han_AN=1888;Lingnan_Han_AC=1;Lingn
chr1	162487921	.	A	<INS:ME:LINE1>	.	PASS	SVTYPE=INS:ME:LINE1;END=162487978;SVLEN=11;AN=20618;AC=1;AF=4.850130953535746e-05;N_HET=1;N_HOMALT=0;N_HOMREF=10308;FREQ_HET=9.700261907071492e-05;FREQ_HOMALT=0.0;FREQ_HOMREF=0.9999029973809292;N_BI_GENOS=10309;East_Han_AN=6554;East_Han_AC=1;East_Han_AF=0
chr1	162640833	.	A	<INS:ME:LINE1>	.	PASS	SVTYPE=INS:ME:LINE1;END=162640850;SVLEN=5998;AN=20618;AC=1;AF=4.850130953535746e-05;N_HET=1;N_HOMALT=0;N_HOMREF=10308;FREQ_HET=9.700261907071492e-05;FREQ_HOMALT=0.0;FREQ_HOMREF=0.9999029973809292;N_BI_GENOS=10309;South_Han_AN=1496;South_Han_AC=1;South_Han
chr1	162949992	.	G	<INS:ME:LINE1>	.	PASS	SVTYPE=INS:ME:LINE1;END=162950008;SVLEN=1553;AN=20618;AC=11;AF=0.000533514404888932;N_HET=11;N_HOMALT=0;N_HOMREF=10298;FREQ_HET=0.001067028809777864;FREQ_HOMALT=0.0;FREQ_HOMREF=0.9989329711902222;N_BI_GENOS=10309;Lingnan_Han_AN=1888;Lingnan_Han_AC=1;Lingn
chr1	163062678	.	A	<INS:ME:LINE1>	.	PASS	SVTYPE=INS:ME:LINE1;END=163062694;SVLEN=181;AN=20618;AC=1;AF=4.850130953535746e-05;N_HET=1;N_HOMALT=0;N_HOMREF=10308;FREQ_HET=9.700261907071492e-05;FREQ_HOMALT=0.0;FREQ_HOMREF=0.9999029973809292;N_BI_GENOS=10309;South_Han_AN=1496;South_Han_AC=1;South_Han_
chr1	163122133	.	A	<INS:ME:LINE1>	.	PASS	SVTYPE=INS:ME:LINE1;END=163122150;SVLEN=5998;AN=20618;AC=12;AF=0.0005820157144242894;N_HET=12;N_HOMALT=0;N_HOMREF=10297;FREQ_HET=0.0011640314288485789;FREQ_HOMALT=0.0;FREQ_HOMREF=0.9988359685711514;N_BI_GENOS=10309;Lingnan_Han_AN=1888;Lingnan_Han_AC=6;Lin
chr1	163238498	.	T	<INS:ME:LINE1>	.	PASS	SVTYPE=INS:ME:LINE1;END=163238512;SVLEN=2105;AN=20618;AC=3;AF=0.00014550392860607236;N_HET=3;N_HOMALT=0;N_HOMREF=10306;FREQ_HET=0.0002910078572121447;FREQ_HOMALT=0.0;FREQ_HOMREF=0.9997089921427879;N_BI_GENOS=10309;East_Han_AN=6554;East_Han_AC=3;East_Han_A
chr1	163314278	.	A	<INS:ME:LINE1>	.	PASS	SVTYPE=INS:ME:LINE1;END=163314291;SVLEN=5996;AN=20618;AC=1;AF=4.850130953535746e-05;N_HET=1;N_HOMALT=0;N_HOMREF=10308;FREQ_HET=9.700261907071492e-05;FREQ_HOMALT=0.0;FREQ_HOMREF=0.9999029973809292;N_BI_GENOS=10309;Hui_AN=444;Hui_AC=1;Hui_AF=0.0022522522522
chr1	163390830	.	T	<INS:ME:LINE1>	.	PASS	SVTYPE=INS:ME:LINE1;END=163390841;SVLEN=1774;AN=20618;AC=3;AF=0.00014550392860607236;N_HET=3;N_HOMALT=0;N_HOMREF=10306;FREQ_HET=0.0002910078572121447;FREQ_HOMALT=0.0;FREQ_HOMREF=0.9997089921427879;N_BI_GENOS=10309;Northwest_Han_AN=1296;Northwest_Han_AC=1;
chr1	163683572	.	A	<INS:ME:LINE1>	.	PASS	SVTYPE=INS:ME:LINE1;END=163683572;SVLEN=778;AN=20618;AC=1;AF=4.850130953535746e-05;N_HET=1;N_HOMALT=0;N_HOMREF=10308;FREQ_HET=9.700261907071492e-05;FREQ_HOMALT=0.0;FREQ_HOMREF=0.9999029973809292;N_BI_GENOS=10309;East_Han_AN=6554;East_Han_AC=1;East_Han_AF=
chr1	163936497	.	A	<INS:ME:LINE1>	.	PASS	SVTYPE=INS:ME:LINE1;END=163936522;SVLEN=1137;AN=20618;AC=2;AF=9.700261907071492e-05;N_HET=2;N_HOMALT=0;N_HOMREF=10307;FREQ_HET=0.00019400523814142983;FREQ_HOMALT=0.0;FREQ_HOMREF=0.9998059947618586;N_BI_GENOS=10309;South_Han_AN=1496;South_Han_AC=1;South_Ha
chr1	163957308	.	C	<INS:ME:LINE1>	.	PASS	SVTYPE=INS:ME:LINE1;END=163957324;SVLEN=1835;AN=20618;AC=1;AF=4.850130953535746e-05;N_HET=1;N_HOMALT=0;N_HOMREF=10308;FREQ_HET=9.700261907071492e-05;FREQ_HOMALT=0.0;FREQ_HOMREF=0.9999029973809292;N_BI_GENOS=10309;South_Han_AN=1496;South_Han_AC=1;South_Han
chr1	164144638	.	T	<INS:ME:LINE1>	.	PASS	SVTYPE=INS:ME:LINE1;END=164144638;SVLEN=2965;AN=20618;AC=1;AF=4.850130953535746e-05;N_HET=1;N_HOMALT=0;N_HOMREF=10308;FREQ_HET=9.700261907071492e-05;FREQ_HOMALT=0.0;FREQ_HOMREF=0.9999029973809292;N_BI_GENOS=10309;Lingnan_Han_AN=1888;Lingnan_Han_AC=1;Lingn
chr1	164349804	.	A	<INS:ME:LINE1>	.	PASS	SVTYPE=INS:ME:LINE1;END=164349847;SVLEN=2602;AN=20618;AC=1;AF=4.850130953535746e-05;N_HET=1;N_HOMALT=0;N_HOMREF=10308;FREQ_HET=9.700261907071492e-05;FREQ_HOMALT=0.0;FREQ_HOMREF=0.9999029973809292;N_BI_GENOS=10309;East_Han_AN=6554;East_Han_AC=1;East_Han_AF
chr1	164350812	.	A	<INS:ME:LINE1>	.	PASS	SVTYPE=INS:ME:LINE1;END=164350812;SVLEN=464;AN=20618;AC=1;AF=4.850130953535746e-05;N_HET=1;N_HOMALT=0;N_HOMREF=10308;FREQ_HET=9.700261907071492e-05;FREQ_HOMALT=0.0;FREQ_HOMREF=0.9999029973809292;N_BI_GENOS=10309;East_Han_AN=6554;East_Han_AC=1;East_Han_AF=
chr1	164358018	.	T	<INS:ME:LINE1>	.	PASS	SVTYPE=INS:ME:LINE1;END=164358028;SVLEN=6000;AN=20618;AC=2;AF=9.700261907071492e-05;N_HET=2;N_HOMALT=0;N_HOMREF=10307;FREQ_HET=0.00019400523814142983;FREQ_HOMALT=0.0;FREQ_HOMREF=0.9998059947618586;N_BI_GENOS=10309;East_Han_AN=6554;East_Han_AC=2;East_Han_A
chr1	164419792	.	A	<INS:ME:LINE1>	.	PASS	SVTYPE=INS:ME:LINE1;END=164419807;SVLEN=697;AN=20618;AC=1;AF=4.850130953535746e-05;N_HET=1;N_HOMALT=0;N_HOMREF=10308;FREQ_HET=9.700261907071492e-05;FREQ_HOMALT=0.0;FREQ_HOMREF=0.9999029973809292;N_BI_GENOS=10309;North_Han_AN=2272;North_Han_AC=1;North_Han_
chr1	165379429	.	A	<INS:ME:LINE1>	.	PASS	SVTYPE=INS:ME:LINE1;END=165379433;SVLEN=260;AN=20618;AC=3;AF=0.00014550392860607236;N_HET=3;N_HOMALT=0;N_HOMREF=10306;FREQ_HET=0.0002910078572121447;FREQ_HOMALT=0.0;FREQ_HOMREF=0.9997089921427879;N_BI_GENOS=10309;Northwest_Han_AN=1296;Northwest_Han_AC=2;N
chr1	165477915	.	A	<INS:ME:LINE1>	.	PASS	SVTYPE=INS:ME:LINE1;END=165477931;SVLEN=6000;AN=20618;AC=40;AF=0.0019400523814142982;N_HET=18;N_HOMALT=11;N_HOMREF=10280;FREQ_HET=0.0017460471432728683;FREQ_HOMALT=0.001067028809777864;FREQ_HOMREF=0.9971869240469493;N_BI_GENOS=10309;South_Han_AN=1496;Sout
chr1	165562673	.	C	<INS:ME:LINE1>	.	PASS	SVTYPE=INS:ME:LINE1;END=165562692;SVLEN=2154;AN=20618;AC=1673;AF=0.08114269085265302;N_HET=1303;N_HOMALT=185;N_HOMREF=8821;FREQ_HET=0.12639441264914153;FREQ_HOMALT=0.01794548452808226;FREQ_HOMREF=0.8556601028227763;N_BI_GENOS=10309;South_Han_AN=1496;South
chr1	165583898	.	T	<INS:ME:LINE1>	.	PASS	SVTYPE=INS:ME:LINE1;END=165583912;SVLEN=6000;AN=20618;AC=2915;AF=0.141381317295567;N_HET=2425;N_HOMALT=245;N_HOMREF=7639;FREQ_HET=0.23523135124648364;FREQ_HOMALT=0.023765641672325154;FREQ_HOMREF=0.7410030070811912;N_BI_GENOS=10309;South_Han_AN=1496;South_
chr1	165928097	.	A	<INS:ME:LINE1>	.	PASS	SVTYPE=INS:ME:LINE1;END=165928113;SVLEN=6012;AN=20618;AC=3;AF=0.00014550392860607236;N_HET=3;N_HOMALT=0;N_HOMREF=10306;FREQ_HET=0.0002910078572121447;FREQ_HOMALT=0.0;FREQ_HOMREF=0.9997089921427879;N_BI_GENOS=10309;South_Han_AN=1496;South_Han_AC=1;South_Ha
chr1	166024836	.	A	<INS:ME:LINE1>	.	PASS	SVTYPE=INS:ME:LINE1;END=166024852;SVLEN=5998;AN=20618;AC=1;AF=4.850130953535746e-05;N_HET=1;N_HOMALT=0;N_HOMREF=10308;FREQ_HET=9.700261907071492e-05;FREQ_HOMALT=0.0;FREQ_HOMREF=0.9999029973809292;N_BI_GENOS=10309;Central_Han_AN=2128;Central_Han_AC=1;Centr
chr1	166222105	.	A	<INS:ME:LINE1>	.	PASS	SVTYPE=INS:ME:LINE1;END=166222105;SVLEN=124;AN=20618;AC=1;AF=4.850130953535746e-05;N_HET=1;N_HOMALT=0;N_HOMREF=10308;FREQ_HET=9.700261907071492e-05;FREQ_HOMALT=0.0;FREQ_HOMREF=0.9999029973809292;N_BI_GENOS=10309;East_Han_AN=6554;East_Han_AC=1;East_Han_AF=
chr1	166222675	.	G	<INS:ME:LINE1>	.	PASS	SVTYPE=INS:ME:LINE1;END=166222675;SVLEN=258;AN=20618;AC=1;AF=4.850130953535746e-05;N_HET=1;N_HOMALT=0;N_HOMREF=10308;FREQ_HET=9.700261907071492e-05;FREQ_HOMALT=0.0;FREQ_HOMREF=0.9999029973809292;N_BI_GENOS=10309;East_Han_AN=6554;East_Han_AC=1;East_Han_AF=

chr1	166278722	.	G	<INS:ME:LINE1>	.	PASS	SVTYPE=INS:ME:LINE1;END=166278736;SVLEN=893;AN=20618;AC=6;AF=0.0002910078572121447;N_HET=6;N_HOMALT=0;N_HOMREF=10303;FREQ_HET=0.0005820157144242894;FREQ_HOMALT=0.0;FREQ_HOMREF=0.9994179842855757;N_BI_GENOS=10309;South_Han_AN=1496;South_Han_AC=1;South_Han_
chr1	166507859	.	G	<INS:ME:LINE1>	.	PASS	SVTYPE=INS:ME:LINE1;END=166507872;SVLEN=1943;AN=20618;AC=1;AF=4.850130953535746e-05;N_HET=1;N_HOMALT=0;N_HOMREF=10308;FREQ_HET=9.700261907071492e-05;FREQ_HOMALT=0.0;FREQ_HOMREF=0.9999029973809292;N_BI_GENOS=10309;East_Han_AN=6554;East_Han_AC=1;East_Han_AF
chr1	166539703	.	A	<INS:ME:LINE1>	.	PASS	SVTYPE=INS:ME:LINE1;END=166539721;SVLEN=913;AN=20618;AC=1;AF=4.850130953535746e-05;N_HET=1;N_HOMALT=0;N_HOMREF=10308;FREQ_HET=9.700261907071492e-05;FREQ_HOMALT=0.0;FREQ_HOMREF=0.9999029973809292;N_BI_GENOS=10309;Manchu_AN=416;Manchu_AC=1;Manchu_AF=0.00240
chr1	166606537	.	A	<INS:ME:LINE1>	.	PASS	SVTYPE=INS:ME:LINE1;END=166606552;SVLEN=1238;AN=20618;AC=1;AF=4.850130953535746e-05;N_HET=1;N_HOMALT=0;N_HOMREF=10308;FREQ_HET=9.700261907071492e-05;FREQ_HOMALT=0.0;FREQ_HOMREF=0.9999029973809292;N_BI_GENOS=10309;East_Han_AN=6554;East_Han_AC=1;East_Han_AF
chr1	166866429	.	A	<INS:ME:LINE1>	.	PASS	SVTYPE=INS:ME:LINE1;END=166866442;SVLEN=12;AN=20618;AC=2;AF=9.700261907071492e-05;N_HET=2;N_HOMALT=0;N_HOMREF=10307;FREQ_HET=0.00019400523814142983;FREQ_HOMALT=0.0;FREQ_HOMREF=0.9998059947618586;N_BI_GENOS=10309;East_Han_AN=6554;East_Han_AC=1;East_Han_AF=
chr1	166916432	.	A	<INS:ME:LINE1>	.	PASS	SVTYPE=INS:ME:LINE1;END=166916447;SVLEN=859;AN=20618;AC=1;AF=4.850130953535746e-05;N_HET=1;N_HOMALT=0;N_HOMREF=10308;FREQ_HET=9.700261907071492e-05;FREQ_HOMALT=0.0;FREQ_HOMREF=0.9999029973809292;N_BI_GENOS=10309;Southeast_Han_AN=1908;Southeast_Han_AC=1;So
chr1	167609027	.	A	<INS:ME:LINE1>	.	PASS	SVTYPE=INS:ME:LINE1;END=167609042;SVLEN=758;AN=20618;AC=1;AF=4.850130953535746e-05;N_HET=1;N_HOMALT=0;N_HOMREF=10308;FREQ_HET=9.700261907071492e-05;FREQ_HOMALT=0.0;FREQ_HOMREF=0.9999029973809292;N_BI_GENOS=10309;Lingnan_Han_AN=1888;Lingnan_Han_AC=1;Lingna
chr1	168159268	.	T	<INS:ME:LINE1>	.	PASS	SVTYPE=INS:ME:LINE1;END=168159291;SVLEN=400;AN=20618;AC=1;AF=4.850130953535746e-05;N_HET=1;N_HOMALT=0;N_HOMREF=10308;FREQ_HET=9.700261907071492e-05;FREQ_HOMALT=0.0;FREQ_HOMREF=0.9999029973809292;N_BI_GENOS=10309;South_Han_AN=1496;South_Han_AC=1;South_Han_
chr1	168346036	.	T	<INS:ME:LINE1>	.	PASS	SVTYPE=INS:ME:LINE1;END=168346050;SVLEN=269;AN=20618;AC=2;AF=9.700261907071492e-05;N_HET=2;N_HOMALT=0;N_HOMREF=10307;FREQ_HET=0.00019400523814142983;FREQ_HOMALT=0.0;FREQ_HOMREF=0.9998059947618586;N_BI_GENOS=10309;Yi_AN=422;Yi_AC=2;Yi_AF=0.0047393364928909
chr1	168419620	.	G	<INS:ME:LINE1>	.	PASS	SVTYPE=INS:ME:LINE1;END=168419632;SVLEN=2543;AN=20618;AC=2;AF=9.700261907071492e-05;N_HET=2;N_HOMALT=0;N_HOMREF=10307;FREQ_HET=0.00019400523814142983;FREQ_HOMALT=0.0;FREQ_HOMREF=0.9998059947618586;N_BI_GENOS=10309;North_Han_AN=2272;North_Han_AC=2;North_Ha
chr1	168451690	.	C	<INS:ME:LINE1>	.	PASS	SVTYPE=INS:ME:LINE1;END=168451690;SVLEN=17;AN=20618;AC=2;AF=9.700261907071492e-05;N_HET=2;N_HOMALT=0;N_HOMREF=10307;FREQ_HET=0.00019400523814142983;FREQ_HOMALT=0.0;FREQ_HOMREF=0.9998059947618586;N_BI_GENOS=10309;Central_Han_AN=2128;Central_Han_AC=1;Centra
chr1	168559840	.	T	<INS:ME:LINE1>	.	PASS	SVTYPE=INS:ME:LINE1;END=168559854;SVLEN=6000;AN=20618;AC=4;AF=0.00019400523814142983;N_HET=4;N_HOMALT=0;N_HOMREF=10305;FREQ_HET=0.00038801047628285966;FREQ_HOMALT=0.0;FREQ_HOMREF=0.9996119895237171;N_BI_GENOS=10309;South_Han_AN=1496;South_Han_AC=1;South_H
chr1	168591799	.	T	<INS:ME:LINE1>	.	PASS	SVTYPE=INS:ME:LINE1;END=168591799;SVLEN=1118;AN=20618;AC=1;AF=4.850130953535746e-05;N_HET=1;N_HOMALT=0;N_HOMREF=10308;FREQ_HET=9.700261907071492e-05;FREQ_HOMALT=0.0;FREQ_HOMREF=0.9999029973809292;N_BI_GENOS=10309;North_Han_AN=2272;North_Han_AC=1;North_Han
chr1	168980092	.	C	<INS:ME:LINE1>	.	PASS	SVTYPE=INS:ME:LINE1;END=168980111;SVLEN=3882;AN=20618;AC=1;AF=4.850130953535746e-05;N_HET=1;N_HOMALT=0;N_HOMREF=10308;FREQ_HET=9.700261907071492e-05;FREQ_HOMALT=0.0;FREQ_HOMREF=0.9999029973809292;N_BI_GENOS=10309;East_Han_AN=6554;East_Han_AC=1;East_Han_AF
chr1	169292402	.	A	<INS:ME:LINE1>	.	PASS	SVTYPE=INS:ME:LINE1;END=169292418;SVLEN=763;AN=20618;AC=1;AF=4.850130953535746e-05;N_HET=1;N_HOMALT=0;N_HOMREF=10308;FREQ_HET=9.700261907071492e-05;FREQ_HOMALT=0.0;FREQ_HOMREF=0.9999029973809292;N_BI_GENOS=10309;Lingnan_Han_AN=1888;Lingnan_Han_AC=1;Lingna
chr1	169356669	.	A	<INS:ME:LINE1>	.	PASS	SVTYPE=INS:ME:LINE1;END=169356685;SVLEN=704;AN=20618;AC=4;AF=0.00019400523814142983;N_HET=4;N_HOMALT=0;N_HOMREF=10305;FREQ_HET=0.00038801047628285966;FREQ_HOMALT=0.0;FREQ_HOMREF=0.9996119895237171;N_BI_GENOS=10309;Northwest_Han_AN=1296;Northwest_Han_AC=1;
chr1	169395327	.	A	<INS:ME:LINE1>	.	PASS	SVTYPE=INS:ME:LINE1;END=169395337;SVLEN=2051;AN=20618;AC=1;AF=4.850130953535746e-05;N_HET=1;N_HOMALT=0;N_HOMREF=10308;FREQ_HET=9.700261907071492e-05;FREQ_HOMALT=0.0;FREQ_HOMREF=0.9999029973809292;N_BI_GENOS=10309;North_Han_AN=2272;North_Han_AC=1;North_Han
chr1	169555621	.	T	<INS:ME:LINE1>	.	PASS	SVTYPE=INS:ME:LINE1;END=169555633;SVLEN=588;AN=20618;AC=14060;AF=0.6819284120671258;N_HET=4216;N_HOMALT=4922;N_HOMREF=1171;FREQ_HET=0.40896304200213407;FREQ_HOMALT=0.4774468910660588;FREQ_HOMREF=0.11359006693180716;N_BI_GENOS=10309;South_Han_AN=1496;South
chr1	169887060	.	T	<INS:ME:LINE1>	.	PASS	SVTYPE=INS:ME:LINE1;END=169887060;SVLEN=1122;AN=20618;AC=2;AF=9.700261907071492e-05;N_HET=0;N_HOMALT=1;N_HOMREF=10308;FREQ_HET=0.0;FREQ_HOMALT=9.700261907071492e-05;FREQ_HOMREF=0.9999029973809292;N_BI_GENOS=10309;East_Han_AN=6554;East_Han_AC=2;East_Han_AF
chr1	170313457	.	T	<INS:ME:LINE1>	.	PASS	SVTYPE=INS:ME:LINE1;END=170313469;SVLEN=792;AN=20618;AC=2;AF=9.700261907071492e-05;N_HET=2;N_HOMALT=0;N_HOMREF=10307;FREQ_HET=0.00019400523814142983;FREQ_HOMALT=0.0;FREQ_HOMREF=0.9998059947618586;N_BI_GENOS=10309;East_Han_AN=6554;East_Han_AC=2;East_Han_AF
chr1	170600611	.	T	<INS:ME:LINE1>	.	PASS	SVTYPE=INS:ME:LINE1;END=170600619;SVLEN=466;AN=20618;AC=1;AF=4.850130953535746e-05;N_HET=1;N_HOMALT=0;N_HOMREF=10308;FREQ_HET=9.700261907071492e-05;FREQ_HOMALT=0.0;FREQ_HOMREF=0.9999029973809292;N_BI_GENOS=10309;South_Han_AN=1496;South_Han_AC=1;South_Han_
chr1	170732946	.	G	<INS:ME:LINE1>	.	PASS	SVTYPE=INS:ME:LINE1;END=170732958;SVLEN=456;AN=20618;AC=1;AF=4.850130953535746e-05;N_HET=1;N_HOMALT=0;N_HOMREF=10308;FREQ_HET=9.700261907071492e-05;FREQ_HOMALT=0.0;FREQ_HOMREF=0.9999029973809292;N_BI_GENOS=10309;Northwest_Han_AN=1296;Northwest_Han_AC=1;No
chr1	170735409	.	C	<INS:ME:LINE1>	.	PASS	SVTYPE=INS:ME:LINE1;END=170735425;SVLEN=5997;AN=20618;AC=1;AF=4.850130953535746e-05;N_HET=1;N_HOMALT=0;N_HOMREF=10308;FREQ_HET=9.700261907071492e-05;FREQ_HOMALT=0.0;FREQ_HOMREF=0.9999029973809292;N_BI_GENOS=10309;North_Han_AN=2272;North_Han_AC=1;North_Han
chr1	170933501	.	A	<INS:ME:LINE1>	.	PASS	SVTYPE=INS:ME:LINE1;END=170933501;SVLEN=0;AN=20618;AC=3;AF=0.00014550392860607236;N_HET=1;N_HOMALT=1;N_HOMREF=10307;FREQ_HET=9.700261907071492e-05;FREQ_HOMALT=9.700261907071492e-05;FREQ_HOMREF=0.9998059947618586;N_BI_GENOS=10309;Central_Han_AN=2128;Centra
chr1	170992908	.	T	<INS:ME:LINE1>	.	PASS	SVTYPE=INS:ME:LINE1;END=170992923;SVLEN=5985;AN=20618;AC=2;AF=9.700261907071492e-05;N_HET=2;N_HOMALT=0;N_HOMREF=10307;FREQ_HET=0.00019400523814142983;FREQ_HOMALT=0.0;FREQ_HOMREF=0.9998059947618586;N_BI_GENOS=10309;East_Han_AN=6554;East_Han_AC=2;East_Han_A
chr1	171235734	.	T	<INS:ME:LINE1>	.	PASS	SVTYPE=INS:ME:LINE1;END=171235749;SVLEN=5997;AN=20618;AC=1;AF=4.850130953535746e-05;N_HET=1;N_HOMALT=0;N_HOMREF=10308;FREQ_HET=9.700261907071492e-05;FREQ_HOMALT=0.0;FREQ_HOMREF=0.9999029973809292;N_BI_GENOS=10309;North_Han_AN=2272;North_Han_AC=1;North_Han
chr1	171327704	.	A	<INS:ME:LINE1>	.	PASS	SVTYPE=INS:ME:LINE1;END=171327706;SVLEN=1194;AN=20618;AC=2;AF=9.700261907071492e-05;N_HET=0;N_HOMALT=1;N_HOMREF=10308;FREQ_HET=0.0;FREQ_HOMALT=9.700261907071492e-05;FREQ_HOMREF=0.9999029973809292;N_BI_GENOS=10309;East_Han_AN=6554;East_Han_AC=2;East_Han_AF
chr1	171335766	.	A	<INS:ME:LINE1>	.	PASS	SVTYPE=INS:ME:LINE1;END=171335776;SVLEN=2229;AN=20618;AC=3;AF=0.00014550392860607236;N_HET=3;N_HOMALT=0;N_HOMREF=10306;FREQ_HET=0.0002910078572121447;FREQ_HOMALT=0.0;FREQ_HOMREF=0.9997089921427879;N_BI_GENOS=10309;East_Han_AN=6554;East_Han_AC=1;East_Han_A
chr1	171909536	.	G	<INS:ME:LINE1>	.	PASS	SVTYPE=INS:ME:LINE1;END=171909551;SVLEN=1156;AN=20618;AC=1;AF=4.850130953535746e-05;N_HET=1;N_HOMALT=0;N_HOMREF=10308;FREQ_HET=9.700261907071492e-05;FREQ_HOMALT=0.0;FREQ_HOMREF=0.9999029973809292;N_BI_GENOS=10309;Lingnan_Han_AN=1888;Lingnan_Han_AC=1;Lingn
chr1	172205604	.	A	<INS:ME:LINE1>	.	PASS	SVTYPE=INS:ME:LINE1;END=172205620;SVLEN=6009;AN=20618;AC=1;AF=4.850130953535746e-05;N_HET=1;N_HOMALT=0;N_HOMREF=10308;FREQ_HET=9.700261907071492e-05;FREQ_HOMALT=0.0;FREQ_HOMREF=0.9999029973809292;N_BI_GENOS=10309;South_Han_AN=1496;South_Han_AC=1;South_Han
chr1	172256077	.	A	<INS:ME:LINE1>	.	PASS	SVTYPE=INS:ME:LINE1;END=172256094;SVLEN=4305;AN=20618;AC=1;AF=4.850130953535746e-05;N_HET=1;N_HOMALT=0;N_HOMREF=10308;FREQ_HET=9.700261907071492e-05;FREQ_HOMALT=0.0;FREQ_HOMREF=0.9999029973809292;N_BI_GENOS=10309;Central_Han_AN=2128;Central_Han_AC=1;Centr
chr1	172675299	.	A	<INS:ME:LINE1>	.	PASS	SVTYPE=INS:ME:LINE1;END=172675314;SVLEN=870;AN=20618;AC=1;AF=4.850130953535746e-05;N_HET=1;N_HOMALT=0;N_HOMREF=10308;FREQ_HET=9.700261907071492e-05;FREQ_HOMALT=0.0;FREQ_HOMREF=0.9999029973809292;N_BI_GENOS=10309;Miao_AN=482;Miao_AC=1;Miao_AF=0.00207468879
chr1	173043409	.	G	<INS:ME:LINE1>	.	PASS	SVTYPE=INS:ME:LINE1;END=173043416;SVLEN=583;AN=20618;AC=1;AF=4.850130953535746e-05;N_HET=1;N_HOMALT=0;N_HOMREF=10308;FREQ_HET=9.700261907071492e-05;FREQ_HOMALT=0.0;FREQ_HOMREF=0.9999029973809292;N_BI_GENOS=10309;East_Han_AN=6554;East_Han_AC=1;East_Han_AF=
chr1	173178666	.	T	<INS:ME:LINE1>	.	PASS	SVTYPE=INS:ME:LINE1;END=173178683;SVLEN=2986;AN=20618;AC=1;AF=4.850130953535746e-05;N_HET=1;N_HOMALT=0;N_HOMREF=10308;FREQ_HET=9.700261907071492e-05;FREQ_HOMALT=0.0;FREQ_HOMREF=0.9999029973809292;N_BI_GENOS=10309;Northwest_Han_AN=1296;Northwest_Han_AC=1;N
chr1	173203002	.	C	<INS:ME:LINE1>	.	PASS	SVTYPE=INS:ME:LINE1;END=173203011;SVLEN=103;AN=20618;AC=4;AF=0.00019400523814142983;N_HET=4;N_HOMALT=0;N_HOMREF=10305;FREQ_HET=0.00038801047628285966;FREQ_HOMALT=0.0;FREQ_HOMREF=0.9996119895237171;N_BI_GENOS=10309;South_Han_AN=1496;South_Han_AC=1;South_Ha
chr1	173264818	.	A	<INS:ME:LINE1>	.	PASS	SVTYPE=INS:ME:LINE1;END=173264834;SVLEN=3163;AN=20618;AC=1;AF=4.850130953535746e-05;N_HET=1;N_HOMALT=0;N_HOMREF=10308;FREQ_HET=9.700261907071492e-05;FREQ_HOMALT=0.0;FREQ_HOMREF=0.9999029973809292;N_BI_GENOS=10309;North_Han_AN=2272;North_Han_AC=1;North_Han
chr1	173387586	.	A	<INS:ME:LINE1>	.	PASS	SVTYPE=INS:ME:LINE1;END=173387601;SVLEN=1725;AN=20618;AC=2;AF=9.700261907071492e-05;N_HET=2;N_HOMALT=0;N_HOMREF=10307;FREQ_HET=0.00019400523814142983;FREQ_HOMALT=0.0;FREQ_HOMREF=0.9998059947618586;N_BI_GENOS=10309;East_Han_AN=6554;East_Han_AC=2;East_Han_A
chr1	173464778	.	G	<INS:ME:LINE1>	.	PASS	SVTYPE=INS:ME:LINE1;END=173464778;SVLEN=1008;AN=20618;AC=1;AF=4.850130953535746e-05;N_HET=1;N_HOMALT=0;N_HOMREF=10308;FREQ_HET=9.700261907071492e-05;FREQ_HOMALT=0.0;FREQ_HOMREF=0.9999029973809292;N_BI_GENOS=10309;East_Han_AN=6554;East_Han_AC=1;East_Han_AF
chr1	173465512	.	G	<INS:ME:LINE1>	.	PASS	SVTYPE=INS:ME:LINE1;END=173465512;SVLEN=1145;AN=20618;AC=2;AF=9.700261907071492e-05;N_HET=2;N_HOMALT=0;N_HOMREF=10307;FREQ_HET=0.00019400523814142983;FREQ_HOMALT=0.0;FREQ_HOMREF=0.9998059947618586;N_BI_GENOS=10309;East_Han_AN=6554;East_Han_AC=2;East_Han_A
chr1	173466254	.	A	<INS:ME:LINE1>	.	PASS	SVTYPE=INS:ME:LINE1;END=173466271;SVLEN=1058;AN=20618;AC=1;AF=4.850130953535746e-05;N_HET=1;N_HOMALT=0;N_HOMREF=10308;FREQ_HET=9.700261907071492e-05;FREQ_HOMALT=0.0;FREQ_HOMREF=0.9999029973809292;N_BI_GENOS=10309;East_Han_AN=6554;East_Han_AC=1;East_Han_AF
chr1	173466396	.	C	<INS:ME:LINE1>	.	PASS	SVTYPE=INS:ME:LINE1;END=173466396;SVLEN=818;AN=20618;AC=1;AF=4.850130953535746e-05;N_HET=1;N_HOMALT=0;N_HOMREF=10308;FREQ_HET=9.700261907071492e-05;FREQ_HOMALT=0.0;FREQ_HOMREF=0.9999029973809292;N_BI_GENOS=10309;East_Han_AN=6554;East_Han_AC=1;East_Han_AF=
chr1	173789773	.	C	<INS:ME:LINE1>	.	PASS	SVTYPE=INS:ME:LINE1;END=173789786;SVLEN=75;AN=20618;AC=15;AF=0.0007275196430303619;N_HET=15;N_HOMALT=0;N_HOMREF=10294;FREQ_HET=0.0014550392860607237;FREQ_HOMALT=0.0;FREQ_HOMREF=0.9985449607139393;N_BI_GENOS=10309;South_Han_AN=1496;South_Han_AC=2;South_Han
chr1	174074894	.	C	<INS:ME:LINE1>	.	PASS	SVTYPE=INS:ME:LINE1;END=174074909;SVLEN=433;AN=20618;AC=1;AF=4.850130953535746e-05;N_HET=1;N_HOMALT=0;N_HOMREF=10308;FREQ_HET=9.700261907071492e-05;FREQ_HOMALT=0.0;FREQ_HOMREF=0.9999029973809292;N_BI_GENOS=10309;South_Han_AN=1496;South_Han_AC=1;South_Han_
chr1	174272382	.	A	<INS:ME:LINE1>	.	PASS	SVTYPE=INS:ME:LINE1;END=174272396;SVLEN=6022;AN=20618;AC=1;AF=4.850130953535746e-05;N_HET=1;N_HOMALT=0;N_HOMREF=10308;FREQ_HET=9.700261907071492e-05;FREQ_HOMALT=0.0;FREQ_HOMREF=0.9999029973809292;N_BI_GENOS=10309;Central_Han_AN=2128;Central_Han_AC=1;Centr
chr1	174320625	.	T	<INS:ME:LINE1>	.	PASS	SVTYPE=INS:ME:LINE1;END=174320642;SVLEN=568;AN=20618;AC=1;AF=4.850130953535746e-05;N_HET=1;N_HOMALT=0;N_HOMREF=10308;FREQ_HET=9.700261907071492e-05;FREQ_HOMALT=0.0;FREQ_HOMREF=0.9999029973809292;N_BI_GENOS=10309;Central_Han_AN=2128;Central_Han_AC=1;Centra
chr1	174431876	.	A	<INS:ME:LINE1>	.	PASS	SVTYPE=INS:ME:LINE1;END=174431891;SVLEN=5998;AN=20618;AC=2;AF=9.700261907071492e-05;N_HET=2;N_HOMALT=0;N_HOMREF=10307;FREQ_HET=0.00019400523814142983;FREQ_HOMALT=0.0;FREQ_HOMREF=0.9998059947618586;N_BI_GENOS=10309;Northwest_Han_AN=1296;Northwest_Han_AC=2;
chr1	174431940	.	T	<INS:ME:LINE1>	.	PASS	SVTYPE=INS:ME:LINE1;END=174431954;SVLEN=877;AN=20618;AC=1;AF=4.850130953535746e-05;N_HET=1;N_HOMALT=0;N_HOMREF=10308;FREQ_HET=9.700261907071492e-05;FREQ_HOMALT=0.0;FREQ_HOMREF=0.9999029973809292;N_BI_GENOS=10309;Lingnan_Han_AN=1888;Lingnan_Han_AC=1;Lingna
chr1	174701189	.	G	<INS:ME:LINE1>	.	PASS	SVTYPE=INS:ME:LINE1;END=174701189;SVLEN=208;AN=20618;AC=2;AF=9.700261907071492e-05;N_HET=2;N_HOMALT=0;N_HOMREF=10307;FREQ_HET=0.00019400523814142983;FREQ_HOMALT=0.0;FREQ_HOMREF=0.9998059947618586;N_BI_GENOS=10309;East_Han_AN=6554;East_Han_AC=2;East_Han_AF
chr1	174706438	.	A	<INS:ME:LINE1>	.	PASS	SVTYPE=INS:ME:LINE1;END=174706452;SVLEN=789;AN=20618;AC=1;AF=4.850130953535746e-05;N_HET=1;N_HOMALT=0;N_HOMREF=10308;FREQ_HET=9.700261907071492e-05;FREQ_HOMALT=0.0;FREQ_HOMREF=0.9999029973809292;N_BI_GENOS=10309;Southeast_Han_AN=1908;Southeast_Han_AC=1;So
chr1	174778117	.	G	<INS:ME:LINE1>	.	PASS	SVTYPE=INS:ME:LINE1;END=174778127;SVLEN=6053;AN=20618;AC=1;AF=4.850130953535746e-05;N_HET=1;N_HOMALT=0;N_HOMREF=10308;FREQ_HET=9.700261907071492e-05;FREQ_HOMALT=0.0;FREQ_HOMREF=0.9999029973809292;N_BI_GENOS=10309;Northwest_Han_AN=1296;Northwest_Han_AC=1;N
chr1	174957214	.	T	<INS:ME:LINE1>	.	PASS	SVTYPE=INS:ME:LINE1;END=174957215;SVLEN=372;AN=20618;AC=1;AF=4.850130953535746e-05;N_HET=1;N_HOMALT=0;N_HOMREF=10308;FREQ_HET=9.700261907071492e-05;FREQ_HOMALT=0.0;FREQ_HOMREF=0.9999029973809292;N_BI_GENOS=10309;East_Han_AN=6554;East_Han_AC=1;East_Han_AF=
chr1	175193587	.	C	<INS:ME:LINE1>	.	PASS	SVTYPE=INS:ME:LINE1;END=175193600;SVLEN=6051;AN=20618;AC=1;AF=4.850130953535746e-05;N_HET=1;N_HOMALT=0;N_HOMREF=10308;FREQ_HET=9.700261907071492e-05;FREQ_HOMALT=0.0;FREQ_HOMREF=0.9999029973809292;N_BI_GENOS=10309;South_Han_AN=1496;South_Han_AC=1;South_Han
chr1	175532703	.	A	<INS:ME:LINE1>	.	PASS	SVTYPE=INS:ME:LINE1;END=175532720;SVLEN=1135;AN=20618;AC=1;AF=4.850130953535746e-05;N_HET=1;N_HOMALT=0;N_HOMREF=10308;FREQ_HET=9.700261907071492e-05;FREQ_HOMALT=0.0;FREQ_HOMREF=0.9999029973809292;N_BI_GENOS=10309;East_Han_AN=6554;East_Han_AC=1;East_Han_AF
chr1	175611651	.	T	<INS:ME:LINE1>	.	PASS	SVTYPE=INS:ME:LINE1;END=175611669;SVLEN=6000;AN=20618;AC=1;AF=4.850130953535746e-05;N_HET=1;N_HOMALT=0;N_HOMREF=10308;FREQ_HET=9.700261907071492e-05;FREQ_HOMALT=0.0;FREQ_HOMREF=0.9999029973809292;N_BI_GENOS=10309;East_Han_AN=6554;East_Han_AC=1;East_Han_AF
chr1	175619059	.	G	<INS:ME:LINE1>	.	PASS	SVTYPE=INS:ME:LINE1;END=175619059;SVLEN=147;AN=20618;AC=9;AF=0.0004365117858182171;N_HET=7;N_HOMALT=1;N_HOMREF=10301;FREQ_HET=0.0006790183334950044;FREQ_HOMALT=9.700261907071492e-05;FREQ_HOMREF=0.9992239790474343;N_BI_GENOS=10309;North_Han_AN=2272;North_H
chr1	175759286	.	A	<INS:ME:LINE1>	.	PASS	SVTYPE=INS:ME:LINE1;END=175759298;SVLEN=5999;AN=20618;AC=1;AF=4.850130953535746e-05;N_HET=1;N_HOMALT=0;N_HOMREF=10308;FREQ_HET=9.700261907071492e-05;FREQ_HOMALT=0.0;FREQ_HOMREF=0.9999029973809292;N_BI_GENOS=10309;East_Han_AN=6554;East_Han_AC=1;East_Han_AF
chr1	176254486	.	C	<INS:ME:LINE1>	.	PASS	SVTYPE=INS:ME:LINE1;END=176254486;SVLEN=709;AN=20618;AC=1;AF=4.850130953535746e-05;N_HET=1;N_HOMALT=0;N_HOMREF=10308;FREQ_HET=9.700261907071492e-05;FREQ_HOMALT=0.0;FREQ_HOMREF=0.9999029973809292;N_BI_GENOS=10309;East_Han_AN=6554;East_Han_AC=1;East_Han_AF=
chr1	176580196	.	C	<INS:ME:LINE1>	.	PASS	SVTYPE=INS:ME:LINE1;END=176580212;SVLEN=1594;AN=20618;AC=6;AF=0.0002910078572121447;N_HET=6;N_HOMALT=0;N_HOMREF=10303;FREQ_HET=0.0005820157144242894;FREQ_HOMALT=0.0;FREQ_HOMREF=0.9994179842855757;N_BI_GENOS=10309;South_Han_AN=1496;South_Han_AC=2;South_Han
chr1	176675567	.	A	<INS:ME:LINE1>	.	PASS	SVTYPE=INS:ME:LINE1;END=176675579;SVLEN=1848;AN=20618;AC=1;AF=4.850130953535746e-05;N_HET=1;N_HOMALT=0;N_HOMREF=10308;FREQ_HET=9.700261907071492e-05;FREQ_HOMALT=0.0;FREQ_HOMREF=0.9999029973809292;N_BI_GENOS=10309;Lingnan_Han_AN=1888;Lingnan_Han_AC=1;Lingn
chr1	176802563	.	T	<INS:ME:LINE1>	.	PASS	SVTYPE=INS:ME:LINE1;END=176802578;SVLEN=1082;AN=20618;AC=2;AF=9.700261907071492e-05;N_HET=2;N_HOMALT=0;N_HOMREF=10307;FREQ_HET=0.00019400523814142983;FREQ_HOMALT=0.0;FREQ_HOMREF=0.9998059947618586;N_BI_GENOS=10309;East_Han_AN=6554;East_Han_AC=2;East_Han_A
chr1	176989499	.	A	<INS:ME:LINE1>	.	PASS	SVTYPE=INS:ME:LINE1;END=176989516;SVLEN=5993;AN=20618;AC=7;AF=0.0003395091667475022;N_HET=7;N_HOMALT=0;N_HOMREF=10302;FREQ_HET=0.0006790183334950044;FREQ_HOMALT=0.0;FREQ_HOMREF=0.999320981666505;N_BI_GENOS=10309;Northwest_Han_AN=1296;Northwest_Han_AC=1;No
chr1	177303374	.	A	<INS:ME:LINE1>	.	PASS	SVTYPE=INS:ME:LINE1;END=177303389;SVLEN=1347;AN=20618;AC=1;AF=4.850130953535746e-05;N_HET=1;N_HOMALT=0;N_HOMREF=10308;FREQ_HET=9.700261907071492e-05;FREQ_HOMALT=0.0;FREQ_HOMREF=0.9999029973809292;N_BI_GENOS=10309;South_Han_AN=1496;South_Han_AC=1;South_Han
chr1	177611804	.	A	<INS:ME:LINE1>	.	PASS	SVTYPE=INS:ME:LINE1;END=177611820;SVLEN=1210;AN=20618;AC=1;AF=4.850130953535746e-05;N_HET=1;N_HOMALT=0;N_HOMREF=10308;FREQ_HET=9.700261907071492e-05;FREQ_HOMALT=0.0;FREQ_HOMREF=0.9999029973809292;N_BI_GENOS=10309;Central_Han_AN=2128;Central_Han_AC=1;Centr
chr1	177683547	.	A	<INS:ME:LINE1>	.	PASS	SVTYPE=INS:ME:LINE1;END=177683564;SVLEN=6007;AN=20618;AC=1;AF=4.850130953535746e-05;N_HET=1;N_HOMALT=0;N_HOMREF=10308;FREQ_HET=9.700261907071492e-05;FREQ_HOMALT=0.0;FREQ_HOMREF=0.9999029973809292;N_BI_GENOS=10309;South_Han_AN=1496;South_Han_AC=1;South_Han
chr1	177749890	.	C	<INS:ME:LINE1>	.	PASS	SVTYPE=INS:ME:LINE1;END=177749906;SVLEN=917;AN=20618;AC=1;AF=4.850130953535746e-05;N_HET=1;N_HOMALT=0;N_HOMREF=10308;FREQ_HET=9.700261907071492e-05;FREQ_HOMALT=0.0;FREQ_HOMREF=0.9999029973809292;N_BI_GENOS=10309;Central_Han_AN=2128;Central_Han_AC=1;Centra
chr1	177819882	.	A	<INS:ME:LINE1>	.	PASS	SVTYPE=INS:ME:LINE1;END=177819897;SVLEN=6000;AN=20618;AC=1;AF=4.850130953535746e-05;N_HET=1;N_HOMALT=0;N_HOMREF=10308;FREQ_HET=9.700261907071492e-05;FREQ_HOMALT=0.0;FREQ_HOMREF=0.9999029973809292;N_BI_GENOS=10309;East_Han_AN=6554;East_Han_AC=1;East_Han_AF
chr1	177879738	.	T	<INS:ME:LINE1>	.	PASS	SVTYPE=INS:ME:LINE1;END=177879738;SVLEN=0;AN=20618;AC=1;AF=4.850130953535746e-05;N_HET=1;N_HOMALT=0;N_HOMREF=10308;FREQ_HET=9.700261907071492e-05;FREQ_HOMALT=0.0;FREQ_HOMREF=0.9999029973809292;N_BI_GENOS=10309;Northwest_Han_AN=1296;Northwest_Han_AC=1;Nort
chr1	177912695	.	A	<INS:ME:LINE1>	.	PASS	SVTYPE=INS:ME:LINE1;END=177912712;SVLEN=1872;AN=20618;AC=1;AF=4.850130953535746e-05;N_HET=1;N_HOMALT=0;N_HOMREF=10308;FREQ_HET=9.700261907071492e-05;FREQ_HOMALT=0.0;FREQ_HOMREF=0.9999029973809292;N_BI_GENOS=10309;South_Han_AN=1496;South_Han_AC=1;South_Han
chr1	177967016	.	A	<INS:ME:LINE1>	.	PASS	SVTYPE=INS:ME:LINE1;END=177967031;SVLEN=6000;AN=20618;AC=2;AF=9.700261907071492e-05;N_HET=2;N_HOMALT=0;N_HOMREF=10307;FREQ_HET=0.00019400523814142983;FREQ_HOMALT=0.0;FREQ_HOMREF=0.9998059947618586;N_BI_GENOS=10309;Lingnan_Han_AN=1888;Lingnan_Han_AC=1;Ling
chr1	178408121	.	T	<INS:ME:LINE1>	.	PASS	SVTYPE=INS:ME:LINE1;END=178408123;SVLEN=4301;AN=20618;AC=1;AF=4.850130953535746e-05;N_HET=1;N_HOMALT=0;N_HOMREF=10308;FREQ_HET=9.700261907071492e-05;FREQ_HOMALT=0.0;FREQ_HOMREF=0.9999029973809292;N_BI_GENOS=10309;East_Han_AN=6554;East_Han_AC=1;East_Han_AF
chr1	178771665	.	A	<INS:ME:LINE1>	.	PASS	SVTYPE=INS:ME:LINE1;END=178771672;SVLEN=5990;AN=20618;AC=1;AF=4.850130953535746e-05;N_HET=1;N_HOMALT=0;N_HOMREF=10308;FREQ_HET=9.700261907071492e-05;FREQ_HOMALT=0.0;FREQ_HOMREF=0.9999029973809292;N_BI_GENOS=10309;Southeast_Han_AN=1908;Southeast_Han_AC=1;S
chr1	179494865	.	T	<INS:ME:LINE1>	.	PASS	SVTYPE=INS:ME:LINE1;END=179494878;SVLEN=5998;AN=20618;AC=2;AF=9.700261907071492e-05;N_HET=2;N_HOMALT=0;N_HOMREF=10307;FREQ_HET=0.00019400523814142983;FREQ_HOMALT=0.0;FREQ_HOMREF=0.9998059947618586;N_BI_GENOS=10309;Southeast_Han_AN=1908;Southeast_Han_AC=2;
chr1	179606226	.	C	<INS:ME:LINE1>	.	PASS	SVTYPE=INS:ME:LINE1;END=179606242;SVLEN=5999;AN=20618;AC=1514;AF=0.07343098263653118;N_HET=576;N_HOMALT=469;N_HOMREF=9264;FREQ_HET=0.055873508584731786;FREQ_HOMALT=0.04549422834416529;FREQ_HOMREF=0.8986322630711029;N_BI_GENOS=10309;South_Han_AN=1496;South
chr1	179780745	.	G	<INS:ME:LINE1>	.	PASS	SVTYPE=INS:ME:LINE1;END=179780755;SVLEN=6006;AN=20618;AC=1;AF=4.850130953535746e-05;N_HET=1;N_HOMALT=0;N_HOMREF=10308;FREQ_HET=9.700261907071492e-05;FREQ_HOMALT=0.0;FREQ_HOMREF=0.9999029973809292;N_BI_GENOS=10309;South_Han_AN=1496;South_Han_AC=1;South_Han
chr1	180039329	.	A	<INS:ME:LINE1>	.	PASS	SVTYPE=INS:ME:LINE1;END=180039342;SVLEN=1431;AN=20618;AC=1;AF=4.850130953535746e-05;N_HET=1;N_HOMALT=0;N_HOMREF=10308;FREQ_HET=9.700261907071492e-05;FREQ_HOMALT=0.0;FREQ_HOMREF=0.9999029973809292;N_BI_GENOS=10309;South_Han_AN=1496;South_Han_AC=1;South_Han
chr1	180672801	.	T	<INS:ME:LINE1>	.	PASS	SVTYPE=INS:ME:LINE1;END=180672803;SVLEN=764;AN=20618;AC=414;AF=0.020079542147637987;N_HET=400;N_HOMALT=7;N_HOMREF=9902;FREQ_HET=0.03880104762828596;FREQ_HOMALT=0.0006790183334950044;FREQ_HOMREF=0.960519934038219;N_BI_GENOS=10309;South_Han_AN=1496;South_Ha
chr1	180696874	.	G	<INS:ME:LINE1>	.	PASS	SVTYPE=INS:ME:LINE1;END=180696874;SVLEN=978;AN=20618;AC=1;AF=4.850130953535746e-05;N_HET=1;N_HOMALT=0;N_HOMREF=10308;FREQ_HET=9.700261907071492e-05;FREQ_HOMALT=0.0;FREQ_HOMREF=0.9999029973809292;N_BI_GENOS=10309;North_Han_AN=2272;North_Han_AC=1;North_Han_
chr1	180776849	.	A	<INS:ME:LINE1>	.	PASS	SVTYPE=INS:ME:LINE1;END=180776865;SVLEN=304;AN=20618;AC=2;AF=9.700261907071492e-05;N_HET=2;N_HOMALT=0;N_HOMREF=10307;FREQ_HET=0.00019400523814142983;FREQ_HOMALT=0.0;FREQ_HOMREF=0.9998059947618586;N_BI_GENOS=10309;Lingnan_Han_AN=1888;Lingnan_Han_AC=1;Lingn
chr1	181189295	.	C	<INS:ME:LINE1>	.	PASS	SVTYPE=INS:ME:LINE1;END=181189309;SVLEN=421;AN=20618;AC=17;AF=0.0008245222621010767;N_HET=17;N_HOMALT=0;N_HOMREF=10292;FREQ_HET=0.0016490445242021534;FREQ_HOMALT=0.0;FREQ_HOMREF=0.9983509554757979;N_BI_GENOS=10309;South_Han_AN=1496;South_Han_AC=1;South_Ha
chr1	182101697	.	A	<INS:ME:LINE1>	.	PASS	SVTYPE=INS:ME:LINE1;END=182101711;SVLEN=417;AN=20618;AC=1;AF=4.850130953535746e-05;N_HET=1;N_HOMALT=0;N_HOMREF=10308;FREQ_HET=9.700261907071492e-05;FREQ_HOMALT=0.0;FREQ_HOMREF=0.9999029973809292;N_BI_GENOS=10309;East_Han_AN=6554;East_Han_AC=1;East_Han_AF=
chr1	182810613	.	A	<INS:ME:LINE1>	.	PASS	SVTYPE=INS:ME:LINE1;END=182810623;SVLEN=5998;AN=20618;AC=1;AF=4.850130953535746e-05;N_HET=1;N_HOMALT=0;N_HOMREF=10308;FREQ_HET=9.700261907071492e-05;FREQ_HOMALT=0.0;FREQ_HOMREF=0.9999029973809292;N_BI_GENOS=10309;East_Han_AN=6554;East_Han_AC=1;East_Han_AF
chr1	183614015	.	A	<INS:ME:LINE1>	.	PASS	SVTYPE=INS:ME:LINE1;END=183614015;SVLEN=0;AN=20618;AC=2;AF=9.700261907071492e-05;N_HET=2;N_HOMALT=0;N_HOMREF=10307;FREQ_HET=0.00019400523814142983;FREQ_HOMALT=0.0;FREQ_HOMREF=0.9998059947618586;N_BI_GENOS=10309;South_Han_AN=1496;South_Han_AC=1;South_Han_A
chr1	183778004	.	C	<INS:ME:LINE1>	.	PASS	SVTYPE=INS:ME:LINE1;END=183778017;SVLEN=91;AN=20618;AC=13;AF=0.0006305170239596469;N_HET=13;N_HOMALT=0;N_HOMREF=10296;FREQ_HET=0.0012610340479192938;FREQ_HOMALT=0.0;FREQ_HOMREF=0.9987389659520807;N_BI_GENOS=10309;South_Han_AN=1496;South_Han_AC=4;South_Han
chr1	184077235	.	A	<INS:ME:LINE1>	.	PASS	SVTYPE=INS:ME:LINE1;END=184077252;SVLEN=2161;AN=20618;AC=1;AF=4.850130953535746e-05;N_HET=1;N_HOMALT=0;N_HOMREF=10308;FREQ_HET=9.700261907071492e-05;FREQ_HOMALT=0.0;FREQ_HOMREF=0.9999029973809292;N_BI_GENOS=10309;Central_Han_AN=2128;Central_Han_AC=1;Centr
chr1	184159478	.	A	<INS:ME:LINE1>	.	PASS	SVTYPE=INS:ME:LINE1;END=184159494;SVLEN=874;AN=20618;AC=1;AF=4.850130953535746e-05;N_HET=1;N_HOMALT=0;N_HOMREF=10308;FREQ_HET=9.700261907071492e-05;FREQ_HOMALT=0.0;FREQ_HOMREF=0.9999029973809292;N_BI_GENOS=10309;South_Han_AN=1496;South_Han_AC=1;South_Han_
chr1	184313138	.	C	<INS:ME:LINE1>	.	PASS	SVTYPE=INS:ME:LINE1;END=184313138;SVLEN=1023;AN=20618;AC=1;AF=4.850130953535746e-05;N_HET=1;N_HOMALT=0;N_HOMREF=10308;FREQ_HET=9.700261907071492e-05;FREQ_HOMALT=0.0;FREQ_HOMREF=0.9999029973809292;N_BI_GENOS=10309;East_Han_AN=6554;East_Han_AC=1;East_Han_AF
chr1	184473722	.	T	<INS:ME:LINE1>	.	PASS	SVTYPE=INS:ME:LINE1;END=184473728;SVLEN=602;AN=20618;AC=1;AF=4.850130953535746e-05;N_HET=1;N_HOMALT=0;N_HOMREF=10308;FREQ_HET=9.700261907071492e-05;FREQ_HOMALT=0.0;FREQ_HOMREF=0.9999029973809292;N_BI_GENOS=10309;Lingnan_Han_AN=1888;Lingnan_Han_AC=1;Lingna
chr1	184676263	.	A	<INS:ME:LINE1>	.	PASS	SVTYPE=INS:ME:LINE1;END=184676278;SVLEN=5997;AN=20618;AC=41;AF=0.0019885536909496557;N_HET=25;N_HOMALT=8;N_HOMREF=10276;FREQ_HET=0.0024250654767678727;FREQ_HOMALT=0.0007760209525657193;FREQ_HOMREF=0.9967989135706664;N_BI_GENOS=10309;South_Han_AN=1496;Sout
chr1	184695325	.	C	<INS:ME:LINE1>	.	PASS	SVTYPE=INS:ME:LINE1;END=184695339;SVLEN=876;AN=20618;AC=1;AF=4.850130953535746e-05;N_HET=1;N_HOMALT=0;N_HOMREF=10308;FREQ_HET=9.700261907071492e-05;FREQ_HOMALT=0.0;FREQ_HOMREF=0.9999029973809292;N_BI_GENOS=10309;East_Han_AN=6554;East_Han_AC=1;East_Han_AF=
chr1	185791159	.	G	<INS:ME:LINE1>	.	PASS	SVTYPE=INS:ME:LINE1;END=185791175;SVLEN=1363;AN=20618;AC=1;AF=4.850130953535746e-05;N_HET=1;N_HOMALT=0;N_HOMREF=10308;FREQ_HET=9.700261907071492e-05;FREQ_HOMALT=0.0;FREQ_HOMREF=0.9999029973809292;N_BI_GENOS=10309;Mongolian_AN=454;Mongolian_AC=1;Mongolian_
chr1	187769533	.	T	<INS:ME:LINE1>	.	PASS	SVTYPE=INS:ME:LINE1;END=187769533;SVLEN=0;AN=20618;AC=1;AF=4.850130953535746e-05;N_HET=1;N_HOMALT=0;N_HOMREF=10308;FREQ_HET=9.700261907071492e-05;FREQ_HOMALT=0.0;FREQ_HOMREF=0.9999029973809292;N_BI_GENOS=10309;North_Han_AN=2272;North_Han_AC=1;North_Han_AF
chr1	188188463	.	A	<INS:ME:LINE1>	.	PASS	SVTYPE=INS:ME:LINE1;END=188188478;SVLEN=1189;AN=20618;AC=11;AF=0.000533514404888932;N_HET=9;N_HOMALT=1;N_HOMREF=10299;FREQ_HET=0.0008730235716364342;FREQ_HOMALT=9.700261907071492e-05;FREQ_HOMREF=0.9990299738092928;N_BI_GENOS=10309;East_Han_AN=6554;East_Ha
chr1	188229833	.	T	<INS:ME:LINE1>	.	PASS	SVTYPE=INS:ME:LINE1;END=188229836;SVLEN=1223;AN=20618;AC=1;AF=4.850130953535746e-05;N_HET=1;N_HOMALT=0;N_HOMREF=10308;FREQ_HET=9.700261907071492e-05;FREQ_HOMALT=0.0;FREQ_HOMREF=0.9999029973809292;N_BI_GENOS=10309;Northwest_Han_AN=1296;Northwest_Han_AC=1;N
chr1	188791661	.	A	<INS:ME:LINE1>	.	PASS	SVTYPE=INS:ME:LINE1;END=188791674;SVLEN=5997;AN=20618;AC=1;AF=4.850130953535746e-05;N_HET=1;N_HOMALT=0;N_HOMREF=10308;FREQ_HET=9.700261907071492e-05;FREQ_HOMALT=0.0;FREQ_HOMREF=0.9999029973809292;N_BI_GENOS=10309;Southeast_Han_AN=1908;Southeast_Han_AC=1;S
chr1	188903791	.	G	<INS:ME:LINE1>	.	PASS	SVTYPE=INS:ME:LINE1;END=188903808;SVLEN=531;AN=20618;AC=33;AF=0.001600543214666796;N_HET=29;N_HOMALT=2;N_HOMREF=10278;FREQ_HET=0.0028130759530507325;FREQ_HOMALT=0.00019400523814142983;FREQ_HOMREF=0.9969929188088078;N_BI_GENOS=10309;South_Han_AN=1496;South
chr1	189169369	.	T	<INS:ME:LINE1>	.	PASS	SVTYPE=INS:ME:LINE1;END=189169371;SVLEN=1995;AN=20618;AC=2;AF=9.700261907071492e-05;N_HET=2;N_HOMALT=0;N_HOMREF=10307;FREQ_HET=0.00019400523814142983;FREQ_HOMALT=0.0;FREQ_HOMREF=0.9998059947618586;N_BI_GENOS=10309;Northwest_Han_AN=1296;Northwest_Han_AC=2;

chr1	189454497	.	A	<INS:ME:LINE1>	.	PASS	SVTYPE=INS:ME:LINE1;END=189454497;SVLEN=2556;AN=20618;AC=1;AF=4.850130953535746e-05;N_HET=1;N_HOMALT=0;N_HOMREF=10308;FREQ_HET=9.700261907071492e-05;FREQ_HOMALT=0.0;FREQ_HOMREF=0.9999029973809292;N_BI_GENOS=10309;East_Han_AN=6554;East_Han_AC=1;East_Han_AF
chr1	189659707	.	A	<INS:ME:LINE1>	.	PASS	SVTYPE=INS:ME:LINE1;END=189659707;SVLEN=16;AN=20618;AC=1;AF=4.850130953535746e-05;N_HET=1;N_HOMALT=0;N_HOMREF=10308;FREQ_HET=9.700261907071492e-05;FREQ_HOMALT=0.0;FREQ_HOMREF=0.9999029973809292;N_BI_GENOS=10309;Tibetan_AN=414;Tibetan_AC=1;Tibetan_AF=0.002
chr1	189772402	.	G	<INS:ME:LINE1>	.	PASS	SVTYPE=INS:ME:LINE1;END=189772420;SVLEN=5997;AN=20618;AC=1;AF=4.850130953535746e-05;N_HET=1;N_HOMALT=0;N_HOMREF=10308;FREQ_HET=9.700261907071492e-05;FREQ_HOMALT=0.0;FREQ_HOMREF=0.9999029973809292;N_BI_GENOS=10309;East_Han_AN=6554;East_Han_AC=1;East_Han_AF
chr1	189794684	.	G	<INS:ME:LINE1>	.	PASS	SVTYPE=INS:ME:LINE1;END=189794702;SVLEN=1002;AN=20618;AC=2;AF=9.700261907071492e-05;N_HET=0;N_HOMALT=1;N_HOMREF=10308;FREQ_HET=0.0;FREQ_HOMALT=9.700261907071492e-05;FREQ_HOMREF=0.9999029973809292;N_BI_GENOS=10309;East_Han_AN=6554;East_Han_AC=2;East_Han_AF
chr1	189930441	.	T	<INS:ME:LINE1>	.	PASS	SVTYPE=INS:ME:LINE1;END=189930460;SVLEN=1886;AN=20618;AC=2;AF=9.700261907071492e-05;N_HET=2;N_HOMALT=0;N_HOMREF=10307;FREQ_HET=0.0001940052381414298З;FREQ_HOMALT=0.0;FREQ_HOMREF=0.9998059947618586;N_BI_GENOS=10309;Lingnan_Han_AN=1888;Lingnan_Han_AC=1;Ling
chr1	190318626	.	T	<INS:ME:LINE1>	.	PASS	SVTYPE=INS:ME:LINE1;END=190318642;SVLEN=1092;AN=20618;AC=7;AF=0.0003395091667475022;N_HET=7;N_HOMALT=0;N_HOMREF=10302;FREQ_HET=0.0006790183334950044;FREQ_HOMALT=0.0;FREQ_HOMREF=0.999320981666505;N_BI_GENOS=10309;South_Han_AN=1496;South_Han_AC=1;South_Han_
chr1	190340821	.	A	<INS:ME:LINE1>	.	PASS	SVTYPE=INS:ME:LINE1;END=190340837;SVLEN=491;AN=20618;AC=1;AF=4.850130953535746e-05;N_HET=1;N_HOMALT=0;N_HOMREF=10308;FREQ_HET=9.700261907071492e-05;FREQ_HOMALT=0.0;FREQ_HOMREF=0.9999029973809292;N_BI_GENOS=10309;South_Han_AN=1496;South_Han_AC=1;South_Han_
chr1	190468775	.	A	<INS:ME:LINE1>	.	PASS	SVTYPE=INS:ME:LINE1;END=190468793;SVLEN=840;AN=20618;AC=1;AF=4.850130953535746e-05;N_HET=1;N_HOMALT=0;N_HOMREF=10308;FREQ_HET=9.700261907071492e-05;FREQ_HOMALT=0.0;FREQ_HOMREF=0.9999029973809292;N_BI_GENOS=10309;Manchu_AN=416;Manchu_AC=1;Manchu_AF=0.00240
chr1	190668550	.	G	<INS:ME:LINE1>	.	PASS	SVTYPE=INS:ME:LINE1;END=190668564;SVLEN=798;AN=20618;AC=1;AF=4.850130953535746e-05;N_HET=1;N_HOMALT=0;N_HOMREF=10308;FREQ_HET=9.700261907071492e-05;FREQ_HOMALT=0.0;FREQ_HOMREF=0.9999029973809292;N_BI_GENOS=10309;East_Han_AN=6554;East_Han_AC=1;East_Han_AF=
chr1	190824566	.	T	<INS:ME:LINE1>	.	PASS	SVTYPE=INS:ME:LINE1;END=190824576;SVLEN=2438;AN=20618;AC=1;AF=4.850130953535746e-05;N_HET=1;N_HOMALT=0;N_HOMREF=10308;FREQ_HET=9.700261907071492e-05;FREQ_HOMALT=0.0;FREQ_HOMREF=0.9999029973809292;N_BI_GENOS=10309;Central_Han_AN=2128;Central_Han_AC=1;Centr
chr1	190853344	.	C	<INS:ME:LINE1>	.	PASS	SVTYPE=INS:ME:LINE1;END=190853344;SVLEN=284;AN=20618;AC=1;AF=4.850130953535746e-05;N_HET=1;N_HOMALT=0;N_HOMREF=10308;FREQ_HET=9.700261907071492e-05;FREQ_HOMALT=0.0;FREQ_HOMREF=0.9999029973809292;N_BI_GENOS=10309;East_Han_AN=6554;East_Han_AC=1;East_Han_AF=
chr1	190886270	.	G	<INS:ME:LINE1>	.	PASS	SVTYPE=INS:ME:LINE1;END=190886283;SVLEN=1501;AN=20618;AC=1;AF=4.850130953535746e-05;N_HET=1;N_HOMALT=0;N_HOMREF=10308;FREQ_HET=9.700261907071492e-05;FREQ_HOMALT=0.0;FREQ_HOMREF=0.9999029973809292;N_BI_GENOS=10309;North_Han_AN=2272;North_Han_AC=1;North_Han
chr1	190888315	.	T	<INS:ME:LINE1>	.	PASS	SVTYPE=INS:ME:LINE1;END=190888329;SVLEN=691;AN=20618;AC=6;AF=0.0002910078572121447;N_HET=6;N_HOMALT=0;N_HOMREF=10303;FREQ_HET=0.0005820157144242894;FREQ_HOMALT=0.0;FREQ_HOMREF=0.9994179842855757;N_BI_GENOS=10309;South_Han_AN=1496;South_Han_AC=1;South_Han_
chr1	190995165	.	A	<INS:ME:LINE1>	.	PASS	SVTYPE=INS:ME:LINE1;END=190995175;SVLEN=822;AN=20618;AC=2;AF=9.700261907071492e-05;N_HET=2;N_HOMALT=0;N_HOMREF=10307;FREQ_HET=0.00019400523814142983;FREQ_HOMALT=0.0;FREQ_HOMREF=0.9998059947618586;N_BI_GENOS=10309;East_Han_AN=6554;East_Han_AC=1;East_Han_AF
chr1	190999082	.	T	<INS:ME:LINE1>	.	PASS	SVTYPE=INS:ME:LINE1;END=190999096;SVLEN=653;AN=20618;AC=1;AF=4.850130953535746e-05;N_HET=1;N_HOMALT=0;N_HOMREF=10308;FREQ_HET=9.700261907071492e-05;FREQ_HOMALT=0.0;FREQ_HOMREF=0.9999029973809292;N_BI_GENOS=10309;Central_Han_AN=2128;Central_Han_AC=1;Centra
chr1	191003872	.	C	<INS:ME:LINE1>	.	PASS	SVTYPE=INS:ME:LINE1;END=191003888;SVLEN=906;AN=20618;AC=848;AF=0.04112911048598312;N_HET=626;N_HOMALT=111;N_HOMREF=9572;FREQ_HET=0.06072363953826753;FREQ_HOMALT=0.010767290716849355;FREQ_HOMREF=0.9285090697448831;N_BI_GENOS=10309;South_Han_AN=1496;South_H
chr1	191019645	.	T	<INS:ME:LINE1>	.	PASS	SVTYPE=INS:ME:LINE1;END=191019659;SVLEN=5997;AN=20618;AC=1;AF=4.850130953535746e-05;N_HET=1;N_HOMALT=0;N_HOMREF=10308;FREQ_HET=9.700261907071492e-05;FREQ_HOMALT=0.0;FREQ_HOMREF=0.9999029973809292;N_BI_GENOS=10309;Northwest_Han_AN=1296;Northwest_Han_AC=1;N
chr1	191073557	.	C	<INS:ME:LINE1>	.	PASS	SVTYPE=INS:ME:LINE1;END=191073575;SVLEN=1469;AN=20618;AC=3;AF=0.0001455039286060 7236;N_HET=3;N_HOMALT=0;N_HOMREF=10306;FREQ_HET=0.0002910078572121447;FREQ_HOMALT=0.0;FREQ_HOMREF=0.9997089921427879;N_BI_GENOS=10309;Lingnan_Han_AN=1888;Lingnan_Han_AC=2;Ling
chr1	191138190	.	C	<INS:ME:LINE1>	.	PASS	SVTYPE=INS:ME:LINE1;END=191138206;SVLEN=3635;AN=20618;AC=14;AF=0.0006790183334950044;N_HET=12;N_HOMALT=1;N_HOMREF=10296;FREQ_HET=0.0011640314288485789;FREQ_HOMALT=9.700261907071492e-05;FREQ_HOMREF=0.9987389659520807;N_BI_GENOS=10309;Lingnan_Han_AN=1888;Li
chr1	191324608	.	C	<INS:ME:LINE1>	.	PASS	SVTYPE=INS:ME:LINE1;END=191324619;SVLEN=660;AN=20618;AC=27;AF=0.0013095353574546513;N_HET=27;N_HOMALT=0;N_HOMREF=10282;FREQ_HET=0.0026190707149093026;FREQ_HOMALT=0.0;FREQ_HOMREF=0.9973809292850907;N_BI_GENOS=10309;South_Han_AN=1496;South_Han_AC=2;South_Ha
chr1	191497808	.	G	<INS:ME:LINE1>	.	PASS	SVTYPE=INS:ME:LINE1;END=191497824;SVLEN=1045;AN=20618;AC=594;AF=0.02880977786400233;N_HET=514;N_HOMALT=40;N_HOMREF=9755;FREQ_HET=0.049859346202347464;FREQ_HOMALT=0.0038801047628285964;FREQ_HOMREF=0.9462605490348239;N_BI_GENOS=10309;South_Han_AN=1496;South
chr1	191611638	.	T	<INS:ME:LINE1>	.	PASS	SVTYPE=INS:ME:LINE1;END=191611638;SVLEN=0;AN=20618;AC=2;AF=9.700261907071492e-05;N_HET=2;N_HOMALT=0;N_HOMREF=10307;FREQ_HET=0.00019400523814142983;FREQ_HOMALT=0.0;FREQ_HOMREF=0.9998059947618586;N_BI_GENOS=10309;East_Han_AN=6554;East_Han_AC=1;East_Han_AF=0
chr1	191626383	.	A	<INS:ME:LINE1>	.	PASS	SVTYPE=INS:ME:LINE1;END=191626399;SVLEN=868;AN=20618;AC=1;AF=4.850130953535746e-05;N_HET=1;N_HOMALT=0;N_HOMREF=10308;FREQ_HET=9.700261907071492e-05;FREQ_HOMALT=0.0;FREQ_HOMREF=0.9999029973809292;N_BI_GENOS=10309;East_Han_AN=6554;East_Han_AC=1;East_Han_AF=
chr1	191767753	.	G	<INS:ME:LINE1>	.	PASS	SVTYPE=INS:ME:LINE1;END=191767769;SVLEN=303;AN=20618;AC=1;AF=4.850130953535746e-05;N_HET=1;N_HOMALT=0;N_HOMREF=10308;FREQ_HET=9.700261907071492e-05;FREQ_HOMALT=0.0;FREQ_HOMREF=0.9999029973809292;N_BI_GENOS=10309;Central_Han_AN=2128;Central_Han_AC=1;Centra
chr1	192039548	.	T	<INS:ME:LINE1>	.	PASS	SVTYPE=INS:ME:LINE1;END=192039549;SVLEN=587;AN=20618;AC=3;AF=0.00014550392860607236;N_HET=3;N_HOMALT=0;N_HOMREF=10306;FREQ_HET=0.0002910078572121447;FREQ_HOMALT=0.0;FREQ_HOMREF=0.9997089921427879;N_BI_GENOS=10309;South_Han_AN=1496;South_Han_AC=1;South_Han
chr1	192172287	.	G	<INS:ME:LINE1>	.	PASS	SVTYPE=INS:ME:LINE1;END=192172302;SVLEN=1916;AN=20618;AC=1;AF=4.850130953535746e-05;N_HET=1;N_HOMALT=0;N_HOMREF=10308;FREQ_HET=9.700261907071492e-05;FREQ_HOMALT=0.0;FREQ_HOMREF=0.9999029973809292;N_BI_GENOS=10309;East_Han_AN=6554;East_Han_AC=1;East_Han_AF
chr1	192293123	.	G	<INS:ME:LINE1>	.	PASS	SVTYPE=INS:ME:LINE1;END=192293138;SVLEN=517;AN=20618;AC=163;AF=0.007905713454263266;N_HET=103;N_HOMALT=30;N_HOMREF=10176;FREQ_HET=0.009991269764283635;FREQ_HOMALT=0.002910078572121447;FREQ_HOMREF=0.9870986516635949;N_BI_GENOS=10309;South_Han_AN=1496;Sout
chr1	192905791	.	T	<INS:ME:LINE1>	.	PASS	SVTYPE=INS:ME:LINE1;END=192905808;SVLEN=234;AN=20618;AC=28;AF=0.0013580366669900088;N_HET=26;N_HOMALT=1;N_HOMREF=10282;FREQ_HET=0.0025220680958385876;FREQ_HOMALT=9.700261907071492e-05;FREQ_HOMREF=0.9973809292850907;N_BI_GENOS=10309;Northwest_Han_AN=1296;N
chr1	192921788	.	A	<INS:ME:LINE1>	.	PASS	SVTYPE=INS:ME:LINE1;END=192921806;SVLEN=5963;AN=20618;AC=1;AF=4.850130953535746e-05;N_HET=1;N_HOMALT=0;N_HOMREF=10308;FREQ_HET=9.700261907071492e-05;FREQ_HOMALT=0.0;FREQ_HOMREF=0.9999029973809292;N_BI_GENOS=10309;East_Han_AN=6554;East_Han_AC=1;East_Han_AF
chr1	193024243	.	A	<INS:ME:LINE1>	.	PASS	SVTYPE=INS:ME:LINE1;END=193024254;SVLEN=335;AN=20618;AC=1;AF=4.850130953535746e-05;N_HET=1;N_HOMALT=0;N_HOMREF=10308;FREQ_HET=9.700261907071492e-05;FREQ_HOMALT=0.0;FREQ_HOMREF=0.9999029973809292;N_BI_GENOS=10309;East_Han_AN=6554;East_Han_AC=1;East_Han_AF=
chr1	193435513	.	A	<INS:ME:LINE1>	.	PASS	SVTYPE=INS:ME:LINE1;END=193435522;SVLEN=80;AN=20618;AC=1;AF=4.850130953535746e-05;N_HET=1;N_HOMALT=0;N_HOMREF=10308;FREQ_HET=9.700261907071492e-05;FREQ_HOMALT=0.0;FREQ_HOMREF=0.9999029973809292;N_BI_GENOS=10309;South_Han_AN=1496;South_Han_AC=1;South_Han_A
chr1	193479289	.	G	<INS:ME:LINE1>	.	PASS	SVTYPE=INS:ME:LINE1;END=193479305;SVLEN=1207;AN=20618;AC=1;AF=4.850130953535746e-05;N_HET=1;N_HOMALT=0;N_HOMREF=10308;FREQ_HET=9.700261907071492e-05;FREQ_HOMALT=0.0;FREQ_HOMREF=0.9999029973809292;N_BI_GENOS=10309;South_Han_AN=1496;South_Han_AC=1;South_Han
chr1	193580510	.	A	<INS:ME:LINE1>	.	PASS	SVTYPE=INS:ME:LINE1;END=193580525;SVLEN=6000;AN=20618;AC=68;AF=0.0032980890484043067;N_HET=68;N_HOMALT=0;N_HOMREF=10241;FREQ_HET=0.006596178096808613 5;FREQ_HOMALT=0.0;FREQ_HOMREF=0.9934038219031914;N_BI_GENOS=10309;South_Han_AN=1496;South_Han_AC=6;South_H
chr1	193891099	.	C	<INS:ME:LINE1>	.	PASS	SVTYPE=INS:ME:LINE1;END=193891115;SVLEN=928;AN=20618;AC=1;AF=4.850130953535746e-05;N_HET=1;N_HOMALT=0;N_HOMREF=10308;FREQ_HET=9.700261907071492e-05;FREQ_HOMALT=0.0;FREQ_HOMREF=0.9999029973809292;N_BI_GENOS=10309;Mongolian_AN=454;Mongolian_AC=1;Mongolian_A
chr1	194080852	.	T	<INS:ME:LINE1>	.	PASS	SVTYPE=INS:ME:LINE1;END=194080861;SVLEN=1219;AN=20618;AC=2;AF=9.700261907071492e-05;N_HET=2;N_HOMALT=0;N_HOMREF=10307;FREQ_HET=0.00019400523814142983;FREQ_HOMALT=0.0;FREQ_HOMREF=0.9998059947618586;N_BI_GENOS=10309;East_Han_AN=6554;East_Han_AC=2;East_Han_A
chr1	194222109	.	A	<INS:ME:LINE1>	.	PASS	SVTYPE=INS:ME:LINE1;END=194222125;SVLEN=2198;AN=20618;AC=1;AF=4.850130953535746e-05;N_HET=1;N_HOMALT=0;N_HOMREF=10308;FREQ_HET=9.700261907071492e-05;FREQ_HOMALT=0.0;FREQ_HOMREF=0.9999029973809292;N_BI_GENOS=10309;South_Han_AN=1496;South_Han_AC=1;South_Han
chr1	194223661	.	A	<INS:ME:LINE1>	.	PASS	SVTYPE=INS:ME:LINE1;END=194223661;SVLEN=489;AN=20618;AC=1;AF=4.850130953535746e-05;N_HET=1;N_HOMALT=0;N_HOMREF=10308;FREQ_HET=9.700261907071492e-05;FREQ_HOMALT=0.0;FREQ_HOMREF=0.9999029973809292;N_BI_GENOS=10309;East_Han_AN=6554;East_Han_AC=1;East_Han_AF=
chr1	194236280	.	A	<INS:ME:LINE1>	.	PASS	SVTYPE=INS:ME:LINE1;END=194236292;SVLEN=885;AN=20618;AC=2;AF=9.700261907071492e-05;N_HET=2;N_HOMALT=0;N_HOMREF=10307;FREQ_HET=0.00019400523814142983;FREQ_HOMALT=0.0;FREQ_HOMREF=0.9998059947618586;N_BI_GENOS=10309;East_Han_AN=6554;East_Han_AC=2;East_Han_AF
chr1	194321878	.	A	<INS:ME:LINE1>	.	PASS	SVTYPE=INS:ME:LINE1;END=194321897;SVLEN=1644;AN=20618;AC=16;AF=0.0007760209525657193;N_HET=16;N_HOMALT=0;N_HOMREF=10293;FREQ_HET=0.0015520419051314386;FREQ_HOMALT=0.0;FREQ_HOMREF=0.9984479580948685;N_BI_GENOS=10309;South_Han_AN=1496;South_Han_AC=1;South_H
chr1	194377684	.	C	<INS:ME:LINE1>	.	PASS	SVTYPE=INS:ME:LINE1;END=194377702;SVLEN=2489;AN=20618;AC=2;AF=9.700261907071492e-05;N_HET=2;N_HOMALT=0;N_HOMREF=10307;FREQ_HET=0.00019400523814142983;FREQ_HOMALT=0.0;FREQ_HOMREF=0.9998059947618586;N_BI_GENOS=10309;East_Han_AN=6554;East_Han_AC=1;East_Han_A
chr1	194624091	.	A	<INS:ME:LINE1>	.	PASS	SVTYPE=INS:ME:LINE1;END=194624107;SVLEN=2947;AN=20618;AC=1;AF=4.850130953535746e-05;N_HET=1;N_HOMALT=0;N_HOMREF=10308;FREQ_HET=9.700261907071492e-05;FREQ_HOMALT=0.0;FREQ_HOMREF=0.9999029973809292;N_BI_GENOS=10309;North_Han_AN=2272;North_Han_AC=1;North_Han
chr1	194706010	.	A	<INS:ME:LINE1>	.	PASS	SVTYPE=INS:ME:LINE1;END=194706010;SVLEN=6;AN=20618;AC=6;AF=0.0002910078572121447;N_HET=6;N_HOMALT=0;N_HOMREF=10303;FREQ_HET=0.0005820157144242894;FREQ_HOMALT=0.0;FREQ_HOMREF=0.9994179842855757;N_BI_GENOS=10309;Lingnan_Han_AN=1888;Lingnan_Han_AC=2;Lingnan_
chr1	194941716	.	C	<INS:ME:LINE1>	.	PASS	SVTYPE=INS:ME:LINE1;END=194941726;SVLEN=1216;AN=20618;AC=2;AF=9.700261907071492e-05;N_HET=2;N_HOMALT=0;N_HOMREF=10307;FREQ_HET=0.00019400523814142983;FREQ_HOMALT=0.0;FREQ_HOMREF=0.9998059947618586;N_BI_GENOS=10309;East_Han_AN=6554;East_Han_AC=1;East_Han_A
chr1	194965511	.	T	<INS:ME:LINE1>	.	PASS	SVTYPE=INS:ME:LINE1;END=194965514;SVLEN=851;AN=20618;AC=15;AF=0.0007275196430303619;N_HET=15;N_HOMALT=0;N_HOMREF=10294;FREQ_HET=0.0014550392860607237;FREQ_HOMALT=0.0;FREQ_HOMREF=0.9985449607139393;N_BI_GENOS=10309;Northwest_Han_AN=1296;Northwest_Han_AC=6;
chr1	194998973	.	A	<INS:ME:LINE1>	.	PASS	SVTYPE=INS:ME:LINE1;END=194998990;SVLEN=5998;AN=20618;AC=5;AF=0.00024250654767678728;N_HET=5;N_HOMALT=0;N_HOMREF=10304;FREQ_HET=0.00048501309535357455;FREQ_HOMALT=0.0;FREQ_HOMREF=0.9995149869046465;N_BI_GENOS=10309;South_Han_AN=1496;South_Han_AC=4;South_H
chr1	195025780	.	A	<INS:ME:LINE1>	.	PASS	SVTYPE=INS:ME:LINE1;END=195025782;SVLEN=368;AN=20618;AC=33;AF=0.0016005432146667 96;N_HET=27;N_HOMALT=3;N_HOMREF=10279;FREQ_HET=0.0026190707149093026;FREQ_HOMALT=0.0002910078572121447;FREQ_HOMREF=0.9970899214278786;N_BI_GENOS=10309;South_Han_AN=1496;South_
chr1	195117197	.	T	<INS:ME:LINE1>	.	PASS	SVTYPE=INS:ME:LINE1;END=195117212;SVLEN=868;AN=20618;AC=2;AF=9.700261907071492e-05;N_HET=2;N_HOMALT=0;N_HOMREF=10307;FREQ_HET=0.00019400523814142983;FREQ_HOMALT=0.0;FREQ_HOMREF=0.9998059947618586;N_BI_GENOS=10309;Southeast_Han_AN=1908;Southeast_Han_AC=2;S
chr1	195192857	.	A	<INS:ME:LINE1>	.	PASS	SVTYPE=INS:ME:LINE1;END=195192857;SVLEN=419;AN=20618;AC=1;AF=4.850130953535746e-05;N_HET=1;N_HOMALT=0;N_HOMREF=10308;FREQ_HET=9.700261907071492e-05;FREQ_HOMALT=0.0;FREQ_HOMREF=0.9999029973809292;N_BI_GENOS=10309;East_Han_AN=6554;East_Han_AC=1;East_Han_AF=
chr1	195391062	.	A	<INS:ME:LINE1>	.	PASS	SVTYPE=INS:ME:LINE1;END=195391069;SVLEN=370;AN=20618;AC=5;AF=0.00024250654767678728;N_HET=5;N_HOMALT=0;N_HOMREF=10304;FREQ_HET=0.00048501309535357455;FREQ_HOMALT=0.0;FREQ_HOMREF=0.9995149869046465;N_BI_GENOS=10309;South_Han_AN=1496;South_Han_AC=1;South_Ha
chr1	195403231	.	A	<INS:ME:LINE1>	.	PASS	SVTYPE=INS:ME:LINE1;END=195403241;SVLEN=3367;AN=20618;AC=2;AF=9.700261907071492e-05;N_HET=2;N_HOMALT=0;N_HOMREF=10307;FREQ_HET=0.00019400523814142983;FREQ_HOMALT=0.0;FREQ_HOMREF=0.9998059947618586;N_BI_GENOS=10309;East_Han_AN=6554;East_Han_AC=2;East_Han_A
chr1	195534476	.	A	<INS:ME:LINE1>	.	PASS	SVTYPE=INS:ME:LINE1;END=195534486;SVLEN=1386;AN=20618;AC=1;AF=4.850130953535746e-05;N_HET=1;N_HOMALT=0;N_HOMREF=10308;FREQ_HET=9.700261907071492e-05;FREQ_HOMALT=0.0;FREQ_HOMREF=0.9999029973809292;N_BI_GENOS=10309;East_Han_AN=6554;East_Han_AC=1;East_Han_AF
chr1	195554582	.	A	<INS:ME:LINE1>	.	PASS	SVTYPE=INS:ME:LINE1;END=195554621;SVLEN=2489;AN=20618;AC=1;AF=4.850130953535746e-05;N_HET=1;N_HOMALT=0;N_HOMREF=10308;FREQ_HET=9.700261907071492e-05;FREQ_HOMALT=0.0;FREQ_HOMREF=0.9999029973809292;N_BI_GENOS=10309;East_Han_AN=6554;East_Han_AC=1;East_Han_AF
chr1	195710547	.	A	<INS:ME:LINE1>	.	PASS	SVTYPE=INS:ME:LINE1;END=195710560;SVLEN=5994;AN=20618;AC=2;AF=9.700261907071492e-05;N_HET=2;N_HOMALT=0;N_HOMREF=10307;FREQ_HET=0.00019400523814142983;FREQ_HOMALT=0.0;FREQ_HOMREF=0.9998059947618586;N_BI_GENOS=10309;Northwest_Han_AN=1296;Northwest_Han_AC=1;
chr1	196002297	.	A	<INS:ME:LINE1>	.	PASS	SVTYPE=INS:ME:LINE1;END=196002299;SVLEN=1510;AN=20618;AC=3;AF=0.00014550392860607236;N_HET=3;N_HOMALT=0;N_HOMREF=10306;FREQ_HET=0.0002910078572121447;FREQ_HOMALT=0.0;FREQ_HOMREF=0.9997089921427879;N_BI_GENOS=10309;Northwest_Han_AN=1296;Northwest_Han_AC=2;
chr1	196288437	.	C	<INS:ME:LINE1>	.	PASS	SVTYPE=INS:ME:LINE1;END=196288450;SVLEN=272;AN=20618;AC=1;AF=4.850130953535746e-05;N_HET=1;N_HOMALT=0;N_HOMREF=10308;FREQ_HET=9.700261907071492e-05;FREQ_HOMALT=0.0;FREQ_HOMREF=0.9999029973809292;N_BI_GENOS=10309;Central_Han_AN=2128;Central_Han_AC=1;Centra
chr1	196321192	.	A	<INS:ME:LINE1>	.	PASS	SVTYPE=INS:ME:LINE1;END=196321208;SVLEN=6009;AN=20618;AC=1;AF=4.850130953535746e-05;N_HET=1;N_HOMALT=0;N_HOMREF=10308;FREQ_HET=9.700261907071492e-05;FREQ_HOMALT=0.0;FREQ_HOMREF=0.9999029973809292;N_BI_GENOS=10309;East_Han_AN=6554;East_Han_AC=1;East_Han_AF
chr1	196495323	.	G	<INS:ME:LINE1>	.	PASS	SVTYPE=INS:ME:LINE1;END=196495335;SVLEN=538;AN=20618;AC=22;AF=0.0010670280897778 64;N_HET=22;N_HOMALT=0;N_HOMREF=10287;FREQ_HET=0.002134057619555728;FREQ_HOMALT=0.0;FREQ_HOMREF=0.9978659423804442;N_BI_GENOS=10309;South_Han_AN=1496;South_Han_AC=2;South_Han_
chr1	196590003	.	C	<INS:ME:LINE1>	.	PASS	SVTYPE=INS:ME:LINE1;END=196590018;SVLEN=1805;AN=20618;AC=1;AF=4.850130953535746e-05;N_HET=1;N_HOMALT=0;N_HOMREF=10308;FREQ_HET=9.700261907071492e-05;FREQ_HOMALT=0.0;FREQ_HOMREF=0.9999029973809292;N_BI_GENOS=10309;East_Han_AN=6554;East_Han_AC=1;East_Han_AF
chr1	196654738	.	A	<INS:ME:LINE1>	.	PASS	SVTYPE=INS:ME:LINE1;END=196654747;SVLEN=3474;AN=20618;AC=2;AF=9.700261907071492e-05;N_HET=2;N_HOMALT=0;N_HOMREF=10307;FREQ_HET=0.00019400523814142983;FREQ_HOMALT=0.0;FREQ_HOMREF=0.9998059947618586;N_BI_GENOS=10309;North_Han_AN=2272;North_Han_AC=1;North_Ha
chr1	197008768	.	C	<INS:ME:LINE1>	.	PASS	SVTYPE=INS:ME:LINE1;END=197008768;SVLEN=44;AN=20618;AC=1;AF=4.850130953535746e-05;N_HET=1;N_HOMALT=0;N_HOMREF=10308;FREQ_HET=9.700261907071492e-05;FREQ_HOMALT=0.0;FREQ_HOMREF=0.9999029973809292;N_BI_GENOS=10309;Central_Han_AN=2128;Central_Han_AC=1;Central
chr1	197099146	.	A	<INS:ME:LINE1>	.	PASS	SVTYPE=INS:ME:LINE1;END=197099159;SVLEN=5980;AN=20618;AC=1;AF=4.850130953535746e-05;N_HET=1;N_HOMALT=0;N_HOMREF=10308;FREQ_HET=9.700261907071492e-05;FREQ_HOMALT=0.0;FREQ_HOMREF=0.9999029973809292;N_BI_GENOS=10309;East_Han_AN=6554;East_Han_AC=1;East_Han_AF
chr1	198132431	.	C	<INS:ME:LINE1>	.	PASS	SVTYPE=INS:ME:LINE1;END=198132446;SVLEN=279;AN=20618;AC=1;AF=4.850130953535746e-05;N_HET=1;N_HOMALT=0;N_HOMREF=10308;FREQ_HET=9.700261907071492e-05;FREQ_HOMALT=0.0;FREQ_HOMREF=0.9999029973809292;N_BI_GENOS=10309;Manchu_AN=416;Manchu_AC=1;Manchu_AF=0.00240
chr1	198150812	.	A	<INS:ME:LINE1>	.	PASS	SVTYPE=INS:ME:LINE1;END=198150825;SVLEN=5998;AN=20618;AC=1;AF=4.850130953535746e-05;N_HET=1;N_HOMALT=0;N_HOMREF=10308;FREQ_HET=9.700261907071492e-05;FREQ_HOMALT=0.0;FREQ_HOMREF=0.9999029973809292;N_BI_GENOS=10309;Lingnan_Han_AN=1888;Lingnan_Han_AC=1;Lingn
chr1	199050416	.	A	<INS:ME:LINE1>	.	PASS	SVTYPE=INS:ME:LINE1;END=199050416;SVLEN=1499;AN=20618;AC=1;AF=4.850130953535746e-05;N_HET=1;N_HOMALT=0;N_HOMREF=10308;FREQ_HET=9.700261907071492e-05;FREQ_HOMALT=0.0;FREQ_HOMREF=0.9999029973809292;N_BI_GENOS=10309;East_Han_AN=6554;East_Han_AC=1;East_Han_AF
chr1	199051245	.	G	<INS:ME:LINE1>	.	PASS	SVTYPE=INS:ME:LINE1;END=199051245;SVLEN=680;AN=20618;AC=1;AF=4.850130953535746e-05;N_HET=1;N_HOMALT=0;N_HOMREF=10308;FREQ_HET=9.700261907071492e-05;FREQ_HOMALT=0.0;FREQ_HOMREF=0.9999029973809292;N_BI_GENOS=10309;East_Han_AN=6554;East_Han_AC=1;East_Han_AF=
chr1	199344022	.	G	<INS:ME:LINE1>	.	PASS	SVTYPE=INS:ME:LINE1;END=199344039;SVLEN=3800;AN=20618;AC=1;AF=4.850130953535746e-05;N_HET=1;N_HOMALT=0;N_HOMREF=10308;FREQ_HET=9.700261907071492e-05;FREQ_HOMALT=0.0;FREQ_HOMREF=0.9999029973809292;N_BI_GENOS=10309;Hui_AN=444;Hui_AC=1;Hui_AF=0.0022522522522
chr1	199428825	.	T	<INS:ME:LINE1>	.	PASS	SVTYPE=INS:ME:LINE1;END=199428837;SVLEN=1797;AN=20618;AC=12;AF=0.0005820157144242894;N_HET=12;N_HOMALT=0;N_HOMREF=10297;FREQ_HET=0.0011640314288485789;FREQ_HOMALT=0.0;FREQ_HOMREF=0.9988359685711514;N_BI_GENOS=10309;Northwest_Han_AN=1296;Northwest_Han_AC=2
chr1	199754654	.	G	<INS:ME:LINE1>	.	PASS	SVTYPE=INS:ME:LINE1;END=199754669;SVLEN=5996;AN=20618;AC=1;AF=4.850130953535746e-05;N_HET=1;N_HOMALT=0;N_HOMREF=10308;FREQ_HET=9.700261907071492e-05;FREQ_HOMALT=0.0;FREQ_HOMREF=0.9999029973809292;N_BI_GENOS=10309;Lingnan_Han_AN=1888;Lingnan_Han_AC=1;Lingn
chr1	199926654	.	A	<INS:ME:LINE1>	.	PASS	SVTYPE=INS:ME:LINE1;END=199926670;SVLEN=215;AN=20618;AC=1;AF=4.850130953535746e-05;N_HET=1;N_HOMALT=0;N_HOMREF=10308;FREQ_HET=9.700261907071492e-05;FREQ_HOMALT=0.0;FREQ_HOMREF=0.9999029973809292;N_BI_GENOS=10309;East_Han_AN=6554;East_Han_AC=1;East_Han_AF=
chr1	200083316	.	T	<INS:ME:LINE1>	.	PASS	SVTYPE=INS:ME:LINE1;END=200083333;SVLEN=3423;AN=20618;AC=2;AF=9.700261907071492e-05;N_HET=2;N_HOMALT=0;N_HOMREF=10307;FREQ_HET=0.00019400523814142983;FREQ_HOMALT=0.0;FREQ_HOMREF=0.9998059947618586;N_BI_GENOS=10309;Central_Han_AN=2128;Central_Han_AC=1;Cent
chr1	200197927	.	A	<INS:ME:LINE1>	.	PASS	SVTYPE=INS:ME:LINE1;END=200197935;SVLEN=881;AN=20618;AC=1;AF=4.850130953535746e-05;N_HET=1;N_HOMALT=0;N_HOMREF=10308;FREQ_HET=9.700261907071492e-05;FREQ_HOMALT=0.0;FREQ_HOMREF=0.9999029973809292;N_BI_GENOS=10309;Central_Han_AN=2128;Central_Han_AC=1;Centra
chr1	200808383	.	A	<INS:ME:LINE1>	.	PASS	SVTYPE=INS:ME:LINE1;END=200808392;SVLEN=419;AN=20618;AC=1;AF=4.850130953535746e-05;N_HET=1;N_HOMALT=0;N_HOMREF=10308;FREQ_HET=9.700261907071492e-05;FREQ_HOMALT=0.0;FREQ_HOMREF=0.9999029973809292;N_BI_GENOS=10309;Northwest_Han_AN=1296;Northwest_Han_AC=1;No
chr1	201948342	.	A	<INS:ME:LINE1>	.	PASS	SVTYPE=INS:ME:LINE1;END=201948342;SVLEN=0;AN=20618;AC=2;AF=9.700261907071492e-05;N_HET=0;N_HOMALT=1;N_HOMREF=10308;FREQ_HET=0.0;FREQ_HOMALT=9.700261907071492e-05;FREQ_HOMREF=0.9999029973809292;N_BI_GENOS=10309;North_Han_AN=2272;North_Han_AC=2;North_Han_AF
chr1	203459081	.	C	<INS:ME:LINE1>	.	PASS	SVTYPE=INS:ME:LINE1;END=203459081;SVLEN=0;AN=20618;AC=3;AF=0.00014550392860607236;N_HET=1;N_HOMALT=1;N_HOMREF=10307;FREQ_HET=9.700261907071492e-05;FREQ_HOMALT=9.700261907071492e-05;FREQ_HOMREF=0.9998059947618586;N_BI_GENOS=10309;Central_Han_AN=2128;Centra
chr1	204234344	.	G	<INS:ME:LINE1>	.	PASS	SVTYPE=INS:ME:LINE1;END=204234358;SVLEN=3530;AN=20618;AC=1;AF=4.850130953535746e-05;N_HET=1;N_HOMALT=0;N_HOMREF=10308;FREQ_HET=9.700261907071492e-05;FREQ_HOMALT=0.0;FREQ_HOMREF=0.9999029973809292;N_BI_GENOS=10309;North_Han_AN=2272;North_Han_AC=1;North_Han
chr1	204642767	.	G	<INS:ME:LINE1>	.	PASS	SVTYPE=INS:ME:LINE1;END=204642767;SVLEN=413;AN=20618;AC=1;AF=4.850130953535746e-05;N_HET=1;N_HOMALT=0;N_HOMREF=10308;FREQ_HET=9.700261907071492e-05;FREQ_HOMALT=0.0;FREQ_HOMREF=0.9999029973809292;N_BI_GENOS=10309;Central_Han_AN=2128;Central_Han_AC=1;Centra
chr1	205737960	.	T	<INS:ME:LINE1>	.	PASS	SVTYPE=INS:ME:LINE1;END=205737963;SVLEN=2030;AN=20618;AC=1;AF=4.850130953535746e-05;N_HET=1;N_HOMALT=0;N_HOMREF=10308;FREQ_HET=9.700261907071492e-05;FREQ_HOMALT=0.0;FREQ_HOMREF=0.9999029973809292;N_BI_GENOS=10309;East_Han_AN=6554;East_Han_AC=1;East_Han_AF
chr1	205911937	.	C	<INS:ME:LINE1>	.	PASS	SVTYPE=INS:ME:LINE1;END=205911953;SVLEN=6006;AN=20618;AC=1;AF=4.850130953535746e-05;N_HET=1;N_HOMALT=0;N_HOMREF=10308;FREQ_HET=9.700261907071492e-05;FREQ_HOMALT=0.0;FREQ_HOMREF=0.9999029973809292;N_BI_GENOS=10309;Central_Han_AN=2128;Central_Han_AC=1;Centr
chr1	206748644	.	T	<INS:ME:LINE1>	.	PASS	SVTYPE=INS:ME:LINE1;END=206748661;SVLEN=6031;AN=20618;AC=2;AF=9.700261907071492e-05;N_HET=2;N_HOMALT=0;N_HOMREF=10307;FREQ_HET=0.00019400523814142983;FREQ_HOMALT=0.0;FREQ_HOMREF=0.9998059947618586;N_BI_GENOS=10309;North_Han_AN=2272;North_Han_AC=1;North_Ha
chr1	207078195	.	T	<INS:ME:LINE1>	.	PASS	SVTYPE=INS:ME:LINE1;END=207078195;SVLEN=1434;AN=20618;AC=1;AF=4.850130953535746e-05;N_HET=1;N_HOMALT=0;N_HOMREF=10308;FREQ_HET=9.700261907071492e-05;FREQ_HOMALT=0.0;FREQ_HOMREF=0.9999029973809292;N_BI_GENOS=10309;Lingnan_Han_AN=1888;Lingnan_Han_AC=1;Lingn
chr1	207242453	.	T	<INS:ME:LINE1>	.	PASS	SVTYPE=INS:ME:LINE1;END=207242470;SVLEN=388;AN=20618;AC=1;AF=4.850130953535746e-05;N_HET=1;N_HOMALT=0;N_HOMREF=10308;FREQ_HET=9.700261907071492e-05;FREQ_HOMALT=0.0;FREQ_HOMREF=0.9999029973809292;N_BI_GENOS=10309;Tibetan_AN=414;Tibetan_AC=1;Tibetan_AF=0.00
chr1	207377388	.	A	<INS:ME:LINE1>	.	PASS	SVTYPE=INS:ME:LINE1;END=207377403;SVLEN=696;AN=20618;AC=1;AF=4.850130953535746e-05;N_HET=1;N_HOMALT=0;N_HOMREF=10308;FREQ_HET=9.700261907071492e-05;FREQ_HOMALT=0.0;FREQ_HOMREF=0.9999029973809292;N_BI_GENOS=10309;Northwest_Han_AN=1296;Northwest_Han_AC=1;No
chr1	207388185	.	C	<INS:ME:LINE1>	.	PASS	SVTYPE=INS:ME:LINE1;END=207388185;SVLEN=915;AN=20618;AC=1;AF=4.850130953535746e-05;N_HET=1;N_HOMALT=0;N_HOMREF=10308;FREQ_HET=9.700261907071492e-05;FREQ_HOMALT=0.0;FREQ_HOMREF=0.9999029973809292;N_BI_GENOS=10309;East_Han_AN=6554;East_Han_AC=1;East_Han_AF=
chr1	207403178	.	T	<INS:ME:LINE1>	.	PASS	SVTYPE=INS:ME:LINE1;END=207403186;SVLEN=4346;AN=20618;AC=1;AF=4.850130953535746e-05;N_HET=1;N_HOMALT=0;N_HOMREF=10308;FREQ_HET=9.700261907071492e-05;FREQ_HOMALT=0.0;FREQ_HOMREF=0.9999029973809292;N_BI_GENOS=10309;East_Han_AN=6554;East_Han_AC=1;East_Han_AF
chr1	208272726	.	G	<INS:ME:LINE1>	.	PASS	SVTYPE=INS:ME:LINE1;END=208272726;SVLEN=0;AN=20618;AC=2;AF=9.700261907071492e-05;N_HET=0;N_HOMALT=1;N_HOMREF=10308;FREQ_HET=0.0;FREQ_HOMALT=9.700261907071492e-05;FREQ_HOMREF=0.9999029973809292;N_BI_GENOS=10309;Northwest_Han_AN=1296;Northwest_Han_AC=2;Nort
chr1	208307630	.	T	<INS:ME:LINE1>	.	PASS	SVTYPE=INS:ME:LINE1;END=208307645;SVLEN=5998;AN=20618;AC=6;AF=0.0002910078572121447;N_HET=6;N_HOMALT=0;N_HOMREF=10303;FREQ_HET=0.0005820157144242894;FREQ_HOMALT=0.0;FREQ_HOMREF=0.9994179842855757;N_BI_GENOS=10309;Lingnan_Han_AN=1888;Lingnan_Han_AC=2;Lin
chr1	208514566	.	A	<INS:ME:LINE1>	.	PASS	SVTYPE=INS:ME:LINE1;END=208514581;SVLEN=530;AN=20618;AC=1;AF=4.850130953535746e-05;N_HET=1;N_HOMALT=0;N_HOMREF=10308;FREQ_HET=9.700261907071492e-05;FREQ_HOMALT=0.0;FREQ_HOMREF=0.9999029973809292;N_BI_GENOS=10309;Yi_AN=422;Yi_AC=1;Yi_AF=0.002369668246445 49
chr1	209453870	.	G	<INS:ME:LINE1>	.	PASS	SVTYPE=INS:ME:LINE1;END=209453870;SVLEN=0;AN=20618;AC=3;AF=0.00014550392860607236;N_HET=1;N_HOMALT=1;N_HOMREF=10307;FREQ_HET=9.700261907071492e-05;FREQ_HOMALT=9.700261907071492e-05;FREQ_HOMREF=0.9998059947618586;N_BI_GENOS=10309;South_Han_AN=1496;South_Ha
chr1	209577307	.	T	<INS:ME:LINE1>	.	PASS	SVTYPE=INS:ME:LINE1;END=209577317;SVLEN=5999;AN=20618;AC=18;AF=0.0008730235716364342;N_HET=18;N_HOMALT=0;N_HOMREF=10291;FREQ_HET=0.0017460471432728683;FREQ_HOMALT=0.0;FREQ_HOMREF=0.9982539528567271;N_BI_GENOS=10309;South_Han_AN=1496;South_Han_AC=2;South_H
chr1	209940312	.	T	<INS:ME:LINE1>	.	PASS	SVTYPE=INS:ME:LINE1;END=209940325;SVLEN=5997;AN=20618;AC=36;AF=0.0017460471432728683;N_HET=36;N_HOMALT=0;N_HOMREF=10273;FREQ_HET=0.0034920942865457366;FREQ_HOMALT=0.0;FREQ_HOMREF=0.9965079057134543;N_BI_GENOS=10309;South_Han_AN=1496;South_Han_AC=1;South_H
chr1	209949068	.	A	<INS:ME:LINE1>	.	PASS	SVTYPE=INS:ME:LINE1;END=209949086;SVLEN=1483;AN=20618;AC=1;AF=4.850130953535746e-05;N_HET=1;N_HOMALT=0;N_HOMREF=10308;FREQ_HET=9.700261907071492e-05;FREQ_HOMALT=0.0;FREQ_HOMREF=0.9999029973809292;N_BI_GENOS=10309;Lingnan_Han_AN=1888;Lingnan_Han_AC=1;Lingn
chr1	210320965	.	A	<INS:ME:LINE1>	.	PASS	SVTYPE=INS:ME:LINE1;END=210320975;SVLEN=5562;AN=20618;AC=1;AF=4.850130953535746e-05;N_HET=1;N_HOMALT=0;N_HOMREF=10308;FREQ_HET=9.700261907071492e-05;FREQ_HOMALT=0.0;FREQ_HOMREF=0.9999029973809292;N_BI_GENOS=10309;Central_Han_AN=2128;Central_Han_AC=1;Centr
chr1	210461812	.	A	<INS:ME:LINE1>	.	PASS	SVTYPE=INS:ME:LINE1;END=210461825;SVLEN=5964;AN=20618;AC=1;AF=4.850130953535746e-05;N_HET=1;N_HOMALT=0;N_HOMREF=10308;FREQ_HET=9.700261907071492e-05;FREQ_HOMALT=0.0;FREQ_HOMREF=0.9999029973809292;N_BI_GENOS=10309;Southeast_Han_AN=1908;Southeast_Han_AC=1;S
chr1	210663871	.	T	<INS:ME:LINE1>	.	PASS	SVTYPE=INS:ME:LINE1;END=210663871;SVLEN=761;AN=20618;AC=1;AF=4.850130953535746e-05;N_HET=1;N_HOMALT=0;N_HOMREF=10308;FREQ_HET=9.700261907071492e-05;FREQ_HOMALT=0.0;FREQ_HOMREF=0.9999029973809292;N_BI_GENOS=10309;Miao_AN=482;Miao_AC=1;Miao_AF=0.00207468879
chr1	210874070	.	G	<INS:ME:LINE1>	.	PASS	SVTYPE=INS:ME:LINE1;END=210874070;SVLEN=1941;AN=20618;AC=1;AF=4.850130953535746e-05;N_HET=1;N_HOMALT=0;N_HOMREF=10308;FREQ_HET=9.700261907071492e-05;FREQ_HOMALT=0.0;FREQ_HOMREF=0.9999029973809292;N_BI_GENOS=10309;Central_Han_AN=2128;Central_Han_AC=1;Centr
chr1	210911395	.	A	<INS:ME:LINE1>	.	PASS	SVTYPE=INS:ME:LINE1;END=210911410;SVLEN=1931;AN=20618;AC=1;AF=4.850130953535746e-05;N_HET=1;N_HOMALT=0;N_HOMREF=10308;FREQ_HET=9.700261907071492e-05;FREQ_HOMALT=0.0;FREQ_HOMREF=0.9999029973809292;N_BI_GENOS=10309;South_Han_AN=1496;South_Han_AC=1;South_Han

chr1	210944280	.	A	<INS:ME:LINE1>	.	PASS	SVTYPE=INS:ME:LINE1;END=210944294;SVLEN=6023;AN=20618;AC=1;AF=4.850130953535746e-05;N_HET=1;N_HOMALT=0;N_HOMREF=10308;FREQ_HET=9.700261907071492e-05;FREQ_HOMALT=0.0;FREQ_HOMREF=0.9999029973809292;N_BI_GENOS=10309;East_Han_AN=6554;East_Han_AC=1;East_Han_AF
chr1	212340300	.	T	<INS:ME:LINE1>	.	PASS	SVTYPE=INS:ME:LINE1;END=212340317;SVLEN=5998;AN=20618;AC=157;AF=0.007614705597051121;N_HET=153;N_HOMALT=2;N_HOMREF=10154;FREQ_HET=0.014841400717819381;FREQ_HOMALT=0.00019400523814142983;FREQ_HOMREF=0.9849645940440392;N_BI_GENOS=10309;South_Han_AN=1496;Sou
chr1	212908546	.	G	<INS:ME:LINE1>	.	PASS	SVTYPE=INS:ME:LINE1;END=212908546;SVLEN=0;AN=20618;AC=1;AF=4.850130953535746e-05;N_HET=1;N_HOMALT=0;N_HOMREF=10308;FREQ_HET=9.700261907071492e-05;FREQ_HOMALT=0.0;FREQ_HOMREF=0.9999029973809292;N_BI_GENOS=10309;Lingnan_Han_AN=1888;Lingnan_Han_AC=1;Lingnan_
chr1	212914257	.	C	<INS:ME:LINE1>	.	PASS	SVTYPE=INS:ME:LINE1;END=212914257;SVLEN=0;AN=20618;AC=1;AF=4.850130953535746e-05;N_HET=1;N_HOMALT=0;N_HOMREF=10308;FREQ_HET=9.700261907071492e-05;FREQ_HOMALT=0.0;FREQ_HOMREF=0.9999029973809292;N_BI_GENOS=10309;Zhuang_AN=444;Zhuang_AC=1;Zhuang_AF=0.0022522
chr1	212914491	.	C	<INS:ME:LINE1>	.	PASS	SVTYPE=INS:ME:LINE1;END=212914491;SVLEN=0;AN=20618;AC=6;AF=0.0002910078572121447;N_HET=2;N_HOMALT=2;N_HOMREF=10305;FREQ_HET=0.00019400523814142983;FREQ_HOMALT=0.00019400523814142983;FREQ_HOMREF=0.9996119895237171;N_BI_GENOS=10309;North_Han_AN=2272;North_H
chr1	213007509	.	T	<INS:ME:LINE1>	.	PASS	SVTYPE=INS:ME:LINE1;END=213007522;SVLEN=501;AN=20618;AC=1;AF=4.850130953535746e-05;N_HET=1;N_HOMALT=0;N_HOMREF=10308;FREQ_HET=9.700261907071492e-05;FREQ_HOMALT=0.0;FREQ_HOMREF=0.9999029973809292;N_BI_GENOS=10309;East_Han_AN=6554;East_Han_AC=1;East_Han_AF=
chr1	213057344	.	T	<INS:ME:LINE1>	.	PASS	SVTYPE=INS:ME:LINE1;END=213057355;SVLEN=980;AN=20618;AC=1;AF=4.850130953535746e-05;N_HET=1;N_HOMALT=0;N_HOMREF=10308;FREQ_HET=9.700261907071492e-05;FREQ_HOMALT=0.0;FREQ_HOMREF=0.9999029973809292;N_BI_GENOS=10309;Central_Han_AN=2128;Central_Han_AC=1;Centra
chr1	213221141	.	C	<INS:ME:LINE1>	.	PASS	SVTYPE=INS:ME:LINE1;END=213221158;SVLEN=1321;AN=20618;AC=1;AF=4.850130953535746e-05;N_HET=1;N_HOMALT=0;N_HOMREF=10308;FREQ_HET=9.700261907071492e-05;FREQ_HOMALT=0.0;FREQ_HOMREF=0.9999029973809292;N_BI_GENOS=10309;East_Han_AN=6554;East_Han_AC=1;East_Han_AF
chr1	213230939	.	A	<INS:ME:LINE1>	.	PASS	SVTYPE=INS:ME:LINE1;END=213230956;SVLEN=5995;AN=20618;AC=3;AF=0.00014550392860607236;N_HET=3;N_HOMALT=0;N_HOMREF=10306;FREQ_HET=0.0002910078572121447;FREQ_HOMALT=0.0;FREQ_HOMREF=0.9997089921427879;N_BI_GENOS=10309;East_Han_AN=6554;East_Han_AC=3;East_Han_A
chr1	213453968	.	G	<INS:ME:LINE1>	.	PASS	SVTYPE=INS:ME:LINE1;END=213453985;SVLEN=874;AN=20618;AC=96;AF=0.004656125715394315 5;N_HET=94;N_HOMALT=1;N_HOMREF=10214;FREQ_HET=0.009118246192647202;FREQ_HOMALT=9.700261907071492e-05;FREQ_HOMREF=0.990784751188 2821;N_BI_GENOS=10309;South_Han_AN=1496;South_
chr1	213471205	.	A	<INS:ME:LINE1>	.	PASS	SVTYPE=INS:ME:LINE1;END=213471222;SVLEN=1228;AN=20618;AC=3;AF=0.00014550392860607236;N_HET=3;N_HOMALT=0;N_HOMREF=10306;FREQ_HET=0.0002910078572121447;FREQ_HOMALT=0.0;FREQ_HOMREF=0.9997089921427879;N_BI_GENOS=10309;Lingnan_Han_AN=1888;Lingnan_Han_AC=2;Ling
chr1	213680628	.	A	<INS:ME:LINE1>	.	PASS	SVTYPE=INS:ME:LINE1;END=213680642;SVLEN=874;AN=20618;AC=1;AF=4.850130953535746e-05;N_HET=1;N_HOMALT=0;N_HOMREF=10308;FREQ_HET=9.700261907071492e-05;FREQ_HOMALT=0.0;FREQ_HOMREF=0.9999029973809292;N_BI_GENOS=10309;Southeast_Han_AN=1908;Southeast_Han_AC=1;So
chr1	214141531	.	A	<INS:ME:LINE1>	.	PASS	SVTYPE=INS:ME:LINE1;END=214141556;SVLEN=4611;AN=20618;AC=2;AF=9.700261907071492e-05;N_HET=2;N_HOMALT=0;N_HOMREF=10307;FREQ_HET=0.00019400523814142983;FREQ_HOMALT=0.0;FREQ_HOMREF=0.9998059947618586;N_BI_GENOS=10309;East_Han_AN=6554;East_Han_AC=1;East_Han_A
chr1	215221482	.	A	<INS:ME:LINE1>	.	PASS	SVTYPE=INS:ME:LINE1;END=215221500;SVLEN=599;AN=20618;AC=1;AF=4.850130953535746e-05;N_HET=1;N_HOMALT=0;N_HOMREF=10308;FREQ_HET=9.700261907071492e-05;FREQ_HOMALT=0.0;FREQ_HOMREF=0.9999029973809292;N_BI_GENOS=10309;East_Han_AN=6554;East_Han_AC=1;East_Han_AF=
chr1	215315155	.	A	<INS:ME:LINE1>	.	PASS	SVTYPE=INS:ME:LINE1;END=215315170;SVLEN=1013;AN=20618;AC=1;AF=4.850130953535746e-05;N_HET=1;N_HOMALT=0;N_HOMREF=10308;FREQ_HET=9.700261907071492e-05;FREQ_HOMALT=0.0;FREQ_HOMREF=0.9999029973809292;N_BI_GENOS=10309;East_Han_AN=6554;East_Han_AC=1;East_Han_AF
chr1	215324349	.	G	<INS:ME:LINE1>	.	PASS	SVTYPE=INS:ME:LINE1;END=215324349;SVLEN=2846;AN=20618;AC=1;AF=4.850130953535746e-05;N_HET=1;N_HOMALT=0;N_HOMREF=10308;FREQ_HET=9.700261907071492e-05;FREQ_HOMALT=0.0;FREQ_HOMREF=0.9999029973809292;N_BI_GENOS=10309;Tibetan_AN=414;Tibetan_AC=1;Tibetan_AF=0.0
chr1	215426620	.	A	<INS:ME:LINE1>	.	PASS	SVTYPE=INS:ME:LINE1;END=215426637;SVLEN=6009;AN=20618;AC=6;AF=0.0002910078572121447;N_HET=6;N_HOMALT=0;N_HOMREF=10303;FREQ_HET=0.0005820157144242894;FREQ_HOMALT=0.0;FREQ_HOMREF=0.9994179842855757;N_BI_GENOS=10309;Northwest_Han_AN=1296;Northwest_Han_AC=1;N
chr1	215506067	.	A	<INS:ME:LINE1>	.	PASS	SVTYPE=INS:ME:LINE1;END=215506076;SVLEN=1072;AN=20618;AC=1;AF=4.850130953535746e-05;N_HET=1;N_HOMALT=0;N_HOMREF=10308;FREQ_HET=9.700261907071492e-05;FREQ_HOMALT=0.0;FREQ_HOMREF=0.9999029973809292;N_BI_GENOS=10309;South_Han_AN=1496;South_Han_AC=1;South_Han
chr1	215625421	.	C	<INS:ME:LINE1>	.	PASS	SVTYPE=INS:ME:LINE1;END=215625437;SVLEN=1297;AN=20618;AC=8;AF=0.00038801047628285966;N_HET=8;N_HOMALT=0;N_HOMREF=10301;FREQ_HET=0.0007760209525657193;FREQ_HOMALT=0.0;FREQ_HOMREF=0.9992239790474343;N_BI_GENOS=10309;East_Han_AN=6554;East_Han_AC=5;East_Han_A
chr1	215828908	.	A	<INS:ME:LINE1>	.	PASS	SVTYPE=INS:ME:LINE1;END=215828926;SVLEN=858;AN=20618;AC=1;AF=4.850130953535746e-05;N_HET=1;N_HOMALT=0;N_HOMREF=10308;FREQ_HET=9.700261907071492e-05;FREQ_HOMALT=0.0;FREQ_HOMREF=0.9999029973809292;N_BI_GENOS=10309;North_Han_AN=2272;North_Han_AC=1;North_Han_
chr1	215924055	.	A	<INS:ME:LINE1>	.	PASS	SVTYPE=INS:ME:LINE1;END=215924067;SVLEN=1438;AN=20618;AC=57;AF=0.0027645746435153748;N_HET=51;N_HOMALT=3;N_HOMREF=10255;FREQ_HET=0.00494713357260646 1;FREQ_HOMALT=0.0002910078572121447;FREQ_HOMREF=0.9947618585701814;N_BI_GENOS=10309;South_Han_AN=1496;South
chr1	216169385	.	A	<INS:ME:LINE1>	.	PASS	SVTYPE=INS:ME:LINE1;END=216169399;SVLEN=5997;AN=20618;AC=1;AF=4.850130953535746e-05;N_HET=1;N_HOMALT=0;N_HOMREF=10308;FREQ_HET=9.700261907071492e-05;FREQ_HOMALT=0.0;FREQ_HOMREF=0.9999029973809292;N_BI_GENOS=10309;East_Han_AN=6554;East_Han_AC=1;East_Han_AF
chr1	216249463	.	T	<INS:ME:LINE1>	.	PASS	SVTYPE=INS:ME:LINE1;END=216249481;SVLEN=479;AN=20618;AC=6;AF=0.0002910078572121447;N_HET=6;N_HOMALT=0;N_HOMREF=10303;FREQ_HET=0.0005820157144242894;FREQ_HOMALT=0.0;FREQ_HOMREF=0.9994179842855757;N_BI_GENOS=10309;Lingnan_Han_AN=1888;Lingnan_Han_AC=3;Lingna
chr1	216652906	.	A	<INS:ME:LINE1>	.	PASS	SVTYPE=INS:ME:LINE1;END=216652921;SVLEN=5999;AN=20618;AC=1;AF=4.850130953535746e-05;N_HET=1;N_HOMALT=0;N_HOMREF=10308;FREQ_HET=9.700261907071492e-05;FREQ_HOMALT=0.0;FREQ_HOMREF=0.9999029973809292;N_BI_GENOS=10309;East_Han_AN=6554;East_Han_AC=1;East_Han_AF
chr1	216653492	.	T	<INS:ME:LINE1>	.	PASS	SVTYPE=INS:ME:LINE1;END=216653511;SVLEN=274;AN=20618;AC=4;AF=0.00019400523814142983;N_HET=4;N_HOMALT=0;N_HOMREF=10305;FREQ_HET=0.00038801047628285966;FREQ_HOMALT=0.0;FREQ_HOMREF=0.9996119895237171;N_BI_GENOS=10309;Northwest_Han_AN=1296;Northwest_Han_AC=1;
chr1	216997071	.	A	<INS:ME:LINE1>	.	PASS	SVTYPE=INS:ME:LINE1;END=216997088;SVLEN=1529;AN=20618;AC=1;AF=4.850130953535746e-05;N_HET=1;N_HOMALT=0;N_HOMREF=10308;FREQ_HET=9.700261907071492e-05;FREQ_HOMALT=0.0;FREQ_HOMREF=0.9999029973809292;N_BI_GENOS=10309;Northwest_Han_AN=1296;Northwest_Han_AC=1;N
chr1	217375648	.	T	<INS:ME:LINE1>	.	PASS	SVTYPE=INS:ME:LINE1;END=217375650;SVLEN=234;AN=20618;AC=4;AF=0.00019400523814142983;N_HET=4;N_HOMALT=0;N_HOMREF=10305;FREQ_HET=0.00038801047628285966;FREQ_HOMALT=0.0;FREQ_HOMREF=0.9996119895237171;N_BI_GENOS=10309;North_Han_AN=2272;North_Han_AC=1;North_Ha
chr1	217432776	.	A	<INS:ME:LINE1>	.	PASS	SVTYPE=INS:ME:LINE1;END=217432791;SVLEN=1174;AN=20618;AC=1;AF=4.850130953535746e-05;N_HET=1;N_HOMALT=0;N_HOMREF=10308;FREQ_HET=9.700261907071492e-05;FREQ_HOMALT=0.0;FREQ_HOMREF=0.9999029973809292;N_BI_GENOS=10309;Southeast_Han_AN=1908;Southeast_Han_AC=1;S
chr1	217649116	.	A	<INS:ME:LINE1>	.	PASS	SVTYPE=INS:ME:LINE1;END=217649135;SVLEN=2946;AN=20618;AC=1;AF=4.850130953535746e-05;N_HET=1;N_HOMALT=0;N_HOMREF=10308;FREQ_HET=9.700261907071492e-05;FREQ_HOMALT=0.0;FREQ_HOMREF=0.9999029973809292;N_BI_GENOS=10309;Zhuang_AN=444;Zhuang_AC=1;Zhuang_AF=0.0022
chr1	217924101	.	A	<INS:ME:LINE1>	.	PASS	SVTYPE=INS:ME:LINE1;END=217924118;SVLEN=1109;AN=20618;AC=1;AF=4.850130953535746e-05;N_HET=1;N_HOMALT=0;N_HOMREF=10308;FREQ_HET=9.700261907071492e-05;FREQ_HOMALT=0.0;FREQ_HOMREF=0.9999029973809292;N_BI_GENOS=10309;East_Han_AN=6554;East_Han_AC=1;East_Han_AF
chr1	217994037	.	T	<INS:ME:LINE1>	.	PASS	SVTYPE=INS:ME:LINE1;END=217994043;SVLEN=849;AN=20618;AC=8;AF=0.00038801047628285966;N_HET=8;N_HOMALT=0;N_HOMREF=10301;FREQ_HET=0.0007760209525657193;FREQ_HOMALT=0.0;FREQ_HOMREF=0.9992239790474343;N_BI_GENOS=10309;South_Han_AN=1496;South_Han_AC=1;South_Han
chr1	218545693	.	T	<INS:ME:LINE1>	.	PASS	SVTYPE=INS:ME:LINE1;END=218545701;SVLEN=612;AN=20618;AC=1;AF=4.850130953535746e-05;N_HET=1;N_HOMALT=0;N_HOMREF=10308;FREQ_HET=9.700261907071492e-05;FREQ_HOMALT=0.0;FREQ_HOMREF=0.9999029973809292;N_BI_GENOS=10309;Southeast_Han_AN=1908;Southeast_Han_AC=1;So
chr1	218674673	.	T	<INS:ME:LINE1>	.	PASS	SVTYPE=INS:ME:LINE1;END=218674687;SVLEN=544;AN=20618;AC=1;AF=4.850130953535746e-05;N_HET=1;N_HOMALT=0;N_HOMREF=10308;FREQ_HET=9.700261907071492e-05;FREQ_HOMALT=0.0;FREQ_HOMREF=0.9999029973809292;N_BI_GENOS=10309;North_Han_AN=2272;North_Han_AC=1;North_Han_
chr1	218787289	.	T	<INS:ME:LINE1>	.	PASS	SVTYPE=INS:ME:LINE1;END=218787307;SVLEN=941;AN=20618;AC=1;AF=4.850130953535746e-05;N_HET=1;N_HOMALT=0;N_HOMREF=10308;FREQ_HET=9.700261907071492e-05;FREQ_HOMALT=0.0;FREQ_HOMREF=0.9999029973809292;N_BI_GENOS=10309;East_Han_AN=6554;East_Han_AC=1;East_Han_AF=
chr1	218838196	.	A	<INS:ME:LINE1>	.	PASS	SVTYPE=INS:ME:LINE1;END=218838210;SVLEN=5999;AN=20618;AC=1;AF=4.850130953535746e-05;N_HET=1;N_HOMALT=0;N_HOMREF=10308;FREQ_HET=9.700261907071492e-05;FREQ_HOMALT=0.0;FREQ_HOMREF=0.9999029973809292;N_BI_GENOS=10309;East_Han_AN=6554;East_Han_AC=1;East_Han_AF
chr1	218869701	.	C	<INS:ME:LINE1>	.	PASS	SVTYPE=INS:ME:LINE1;END=218869713;SVLEN=265;AN=20618;AC=1;AF=4.850130953535746e-05;N_HET=1;N_HOMALT=0;N_HOMREF=10308;FREQ_HET=9.700261907071492e-05;FREQ_HOMALT=0.0;FREQ_HOMREF=0.9999029973809292;N_BI_GENOS=10309;East_Han_AN=6554;East_Han_AC=1;East_Han_AF=
chr1	219163705	.	T	<INS:ME:LINE1>	.	PASS	SVTYPE=INS:ME:LINE1;END=219163717;SVLEN=672;AN=20618;AC=3;AF=0.00014550392860607236;N_HET=3;N_HOMALT=0;N_HOMREF=10306;FREQ_HET=0.0002910078572121447;FREQ_HOMALT=0.0;FREQ_HOMREF=0.9997089921427879;N_BI_GENOS=10309;South_Han_AN=1496;South_Han_AC=1;South_Han
chr1	219377521	.	A	<INS:ME:LINE1>	.	PASS	SVTYPE=INS:ME:LINE1;END=219377537;SVLEN=290;AN=20618;AC=3;AF=0.00014550392860607236;N_HET=3;N_HOMALT=0;N_HOMREF=10306;FREQ_HET=0.0002910078572121447;FREQ_HOMALT=0.0;FREQ_HOMREF=0.9997089921427879;N_BI_GENOS=10309;East_Han_AN=6554;East_Han_AC=2;East_Han_AF
chr1	219670121	.	A	<INS:ME:LINE1>	.	PASS	SVTYPE=INS:ME:LINE1;END=219670133;SVLEN=4204;AN=20618;AC=11;AF=0.000533514404888932;N_HET=11;N_HOMALT=0;N_HOMREF=10298;FREQ_HET=0.001067028809777864;FREQ_HOMALT=0.0;FREQ_HOMREF=0.9989329711902222;N_BI_GENOS=10309;North_Han_AN=2272;North_Han_AC=1;North_Han
chr1	219736255	.	A	<INS:ME:LINE1>	.	PASS	SVTYPE=INS:ME:LINE1;END=219736271;SVLEN=885;AN=20618;AC=1;AF=4.850130953535746e-05;N_HET=1;N_HOMALT=0;N_HOMREF=10308;FREQ_HET=9.700261907071492e-05;FREQ_HOMALT=0.0;FREQ_HOMREF=0.9999029973809292;N_BI_GENOS=10309;Yi_AN=422;Yi_AC=1;Yi_AF=0.002369668246445 49
chr1	220000088	.	A	<INS:ME:LINE1>	.	PASS	SVTYPE=INS:ME:LINE1;END=220000088;SVLEN=1613;AN=20618;AC=28;AF=0.0013580366669900088;N_HET=26;N_HOMALT=1;N_HOMREF=10282;FREQ_HET=0.0025220680958385876;FREQ_HOMALT=9.700261907071492e-05;FREQ_HOMREF=0.9973809292850907;N_BI_GENOS=10309;South_Han_AN=1496;Sout
chr1	221224009	.	C	<INS:ME:LINE1>	.	PASS	SVTYPE=INS:ME:LINE1;END=221224025;SVLEN=3013;AN=20618;AC=1;AF=4.850130953535746e-05;N_HET=1;N_HOMALT=0;N_HOMREF=10308;FREQ_HET=9.700261907071492e-05;FREQ_HOMALT=0.0;FREQ_HOMREF=0.9999029973809292;N_BI_GENOS=10309;Southeast_Han_AN=1908;Southeast_Han_AC=1;S
chr1	221275862	.	A	<INS:ME:LINE1>	.	PASS	SVTYPE=INS:ME:LINE1;END=221275869;SVLEN=1894;AN=20618;AC=1;AF=4.850130953535746e-05;N_HET=1;N_HOMALT=0;N_HOMREF=10308;FREQ_HET=9.700261907071492e-05;FREQ_HOMALT=0.0;FREQ_HOMREF=0.9999029973809292;N_BI_GENOS=10309;North_Han_AN=2272;North_Han_AC=1;North_Han
chr1	221514926	.	A	<INS:ME:LINE1>	.	PASS	SVTYPE=INS:ME:LINE1;END=221514944;SVLEN=1198;AN=20618;AC=1;AF=4.850130953535746e-05;N_HET=1;N_HOMALT=0;N_HOMREF=10308;FREQ_HET=9.700261907071492e-05;FREQ_HOMALT=0.0;FREQ_HOMREF=0.9999029973809292;N_BI_GENOS=10309;Central_Han_AN=2128;Central_Han_AC=1;Centr
chr1	221653912	.	A	<INS:ME:LINE1>	.	PASS	SVTYPE=INS:ME:LINE1;END=221653912;SVLEN=3949;AN=20618;AC=4;AF=0.00019400523814142983;N_HET=4;N_HOMALT=0;N_HOMREF=10305;FREQ_HET=0.00038801047628285966;FREQ_HOMALT=0.0;FREQ_HOMREF=0.9996119895237171;N_BI_GENOS=10309;Lingnan_Han_AN=1888;Lingnan_Han_AC=1;Lin
chr1	221654479	.	A	<INS:ME:LINE1>	.	PASS	SVTYPE=INS:ME:LINE1;END=221654479;SVLEN=364;AN=20618;AC=406;AF=0.019691531671355126;N_HET=126;N_HOMALT=140;N_HOMREF=10043;FREQ_HET=0.012222330002910078;FREQ_HOMALT=0.013580366669900087;FREQ_HOMREF=0.9741973033271898;N_BI_GENOS=10309;South_Han_AN=1496;Sout
chr1	221967440	.	G	<INS:ME:LINE1>	.	PASS	SVTYPE=INS:ME:LINE1;END=221967440;SVLEN=0;AN=20618;AC=6;AF=0.0002910078572121447;N_HET=6;N_HOMALT=0;N_HOMREF=10303;FREQ_HET=0.0005820157144242894;FREQ_HOMALT=0.0;FREQ_HOMREF=0.9994179842855757;N_BI_GENOS=10309;Lingnan_Han_AN=1888;Lingnan_Han_AC=1;Lingnan_
chr1	222314036	.	T	<INS:ME:LINE1>	.	PASS	SVTYPE=INS:ME:LINE1;END=222314053;SVLEN=6085;AN=20618;AC=1;AF=4.850130953535746e-05;N_HET=1;N_HOMALT=0;N_HOMREF=10308;FREQ_HET=9.700261907071492e-05;FREQ_HOMALT=0.0;FREQ_HOMREF=0.9999029973809292;N_BI_GENOS=10309;East_Han_AN=6554;East_Han_AC=1;East_Han_AF
chr1	222390114	.	T	<INS:ME:LINE1>	.	PASS	SVTYPE=INS:ME:LINE1;END=222390124;SVLEN=412;AN=20618;AC=8;AF=0.00038801047628285966;N_HET=8;N_HOMALT=0;N_HOMREF=10301;FREQ_HET=0.0007760209525657193;FREQ_HOMALT=0.0;FREQ_HOMREF=0.9992239790474343;N_BI_GENOS=10309;South_Han_AN=1496;South_Han_AC=1;South_Han
chr1	222427714	.	A	<INS:ME:LINE1>	.	PASS	SVTYPE=INS:ME:LINE1;END=222427735;SVLEN=1312;AN=20618;AC=1;AF=4.850130953535746e-05;N_HET=1;N_HOMALT=0;N_HOMREF=10308;FREQ_HET=9.700261907071492e-05;FREQ_HOMALT=0.0;FREQ_HOMREF=0.9999029973809292;N_BI_GENOS=10309;South_Han_AN=1496;South_Han_AC=1;South_Han
chr1	222710631	.	A	<INS:ME:LINE1>	.	PASS	SVTYPE=INS:ME:LINE1;END=222710643;SVLEN=6000;AN=20618;AC=1;AF=4.850130953535746e-05;N_HET=1;N_HOMALT=0;N_HOMREF=10308;FREQ_HET=9.700261907071492e-05;FREQ_HOMALT=0.0;FREQ_HOMREF=0.9999029973809292;N_BI_GENOS=10309;East_Han_AN=6554;East_Han_AC=1;East_Han_AF
chr1	222816932	.	T	<INS:ME:LINE1>	.	PASS	SVTYPE=INS:ME:LINE1;END=222816946;SVLEN=5375;AN=20618;AC=1;AF=4.850130953535746e-05;N_HET=1;N_HOMALT=0;N_HOMREF=10308;FREQ_HET=9.700261907071492e-05;FREQ_HOMALT=0.0;FREQ_HOMREF=0.9999029973809292;N_BI_GENOS=10309;East_Han_AN=6554;East_Han_AC=1;East_Han_AF
chr1	223024582	.	T	<INS:ME:LINE1>	.	PASS	SVTYPE=INS:ME:LINE1;END=223024582;SVLEN=0;AN=20618;AC=10;AF=0.00048501309535357455;N_HET=0;N_HOMALT=5;N_HOMREF=10304;FREQ_HET=0.0;FREQ_HOMALT=0.00048501309535357455;FREQ_HOMREF=0.9995149869046465;N_BI_GENOS=10309;Lingnan_Han_AN=1888;Lingnan_Han_AC=2;Lingn
chr1	223415307	.	A	<INS:ME:LINE1>	.	PASS	SVTYPE=INS:ME:LINE1;END=223415321;SVLEN=2500;AN=20618;AC=1;AF=4.850130953535746e-05;N_HET=1;N_HOMALT=0;N_HOMREF=10308;FREQ_HET=9.700261907071492e-05;FREQ_HOMALT=0.0;FREQ_HOMREF=0.9999029973809292;N_BI_GENOS=10309;East_Han_AN=6554;East_Han_AC=1;East_Han_AF
chr1	223539282	.	A	<INS:ME:LINE1>	.	PASS	SVTYPE=INS:ME:LINE1;END=223539297;SVLEN=3797;AN=20618;AC=1;AF=4.850130953535746e-05;N_HET=1;N_HOMALT=0;N_HOMREF=10308;FREQ_HET=9.700261907071492e-05;FREQ_HOMALT=0.0;FREQ_HOMREF=0.9999029973809292;N_BI_GENOS=10309;East_Han_AN=6554;East_Han_AC=1;East_Han_AF
chr1	224569471	.	A	<INS:ME:LINE1>	.	PASS	SVTYPE=INS:ME:LINE1;END=224569484;SVLEN=134;AN=20618;AC=2;AF=9.700261907071492e-05;N_HET=2;N_HOMALT=0;N_HOMREF=10307;FREQ_HET=0.00019400523814142983;FREQ_HOMALT=0.0;FREQ_HOMREF=0.9998059947618586;N_BI_GENOS=10309;East_Han_AN=6554;East_Han_AC=2;East_Han_AF
chr1	224648157	.	G	<INS:ME:LINE1>	.	PASS	SVTYPE=INS:ME:LINE1;END=224648166;SVLEN=5998;AN=20618;AC=1;AF=4.850130953535746e-05;N_HET=1;N_HOMALT=0;N_HOMREF=10308;FREQ_HET=9.700261907071492e-05;FREQ_HOMALT=0.0;FREQ_HOMREF=0.9999029973809292;N_BI_GENOS=10309;East_Han_AN=6554;East_Han_AC=1;East_Han_AF
chr1	224680729	.	A	<INS:ME:LINE1>	.	PASS	SVTYPE=INS:ME:LINE1;END=224680746;SVLEN=6025;AN=20618;AC=1;AF=4.850130953535746e-05;N_HET=1;N_HOMALT=0;N_HOMREF=10308;FREQ_HET=9.700261907071492e-05;FREQ_HOMALT=0.0;FREQ_HOMREF=0.9999029973809292;N_BI_GENOS=10309;North_Han_AN=2272;North_Han_AC=1;North_Han
chr1	224841182	.	G	<INS:ME:LINE1>	.	PASS	SVTYPE=INS:ME:LINE1;END=224841182;SVLEN=0;AN=20618;AC=2;AF=9.700261907071492e-05;N_HET=2;N_HOMALT=0;N_HOMREF=10307;FREQ_HET=0.00019400523814142983;FREQ_HOMALT=0.0;FREQ_HOMREF=0.9998059947618586;N_BI_GENOS=10309;Southeast_Han_AN=1908;Southeast_Han_AC=1;Sou
chr1	224995023	.	T	<INS:ME:LINE1>	.	PASS	SVTYPE=INS:ME:LINE1;END=224995041;SVLEN=888;AN=20618;AC=2;AF=9.700261907071492e-05;N_HET=2;N_HOMALT=0;N_HOMREF=10307;FREQ_HET=0.00019400523814142983;FREQ_HOMALT=0.0;FREQ_HOMREF=0.9998059947618586;N_BI_GENOS=10309;East_Han_AN=6554;East_Han_AC=2;East_Han_AF
chr1	225046003	.	A	<INS:ME:LINE1>	.	PASS	SVTYPE=INS:ME:LINE1;END=225046006;SVLEN=2726;AN=20618;AC=2;AF=9.700261907071492e-05;N_HET=2;N_HOMALT=0;N_HOMREF=10307;FREQ_HET=0.00019400523814142983;FREQ_HOMALT=0.0;FREQ_HOMREF=0.9998059947618586;N_BI_GENOS=10309;Mongolian_AN=454;Mongolian_AC=1;Mongolian
chr1	225051834	.	A	<INS:ME:LINE1>	.	PASS	SVTYPE=INS:ME:LINE1;END=225051850;SVLEN=1307;AN=20618;AC=1;AF=4.850130953535746e-05;N_HET=1;N_HOMALT=0;N_HOMREF=10308;FREQ_HET=9.700261907071492e-05;FREQ_HOMALT=0.0;FREQ_HOMREF=0.9999029973809292;N_BI_GENOS=10309;Central_Han_AN=2128;Central_Han_AC=1;Centr
chr1	225196691	.	T	<INS:ME:LINE1>	.	PASS	SVTYPE=INS:ME:LINE1;END=225196708;SVLEN=799;AN=20618;AC=1;AF=4.850130953535746e-05;N_HET=1;N_HOMALT=0;N_HOMREF=10308;FREQ_HET=9.700261907071492e-05;FREQ_HOMALT=0.0;FREQ_HOMREF=0.9999029973809292;N_BI_GENOS=10309;East_Han_AN=6554;East_Han_AC=1;East_Han_AF=
chr1	225281061	.	C	<INS:ME:LINE1>	.	PASS	SVTYPE=INS:ME:LINE1;END=225281078;SVLEN=1736;AN=20618;AC=1;AF=4.850130953535746e-05;N_HET=1;N_HOMALT=0;N_HOMREF=10308;FREQ_HET=9.700261907071492e-05;FREQ_HOMALT=0.0;FREQ_HOMREF=0.9999029973809292;N_BI_GENOS=10309;Central_Han_AN=2128;Central_Han_AC=1;Centr
chr1	225362816	.	A	<INS:ME:LINE1>	.	PASS	SVTYPE=INS:ME:LINE1;END=225362829;SVLEN=6004;AN=20618;AC=2;AF=9.700261907071492e-05;N_HET=0;N_HOMALT=1;N_HOMREF=10308;FREQ_HET=0.0;FREQ_HOMALT=9.700261907071492e-05;FREQ_HOMREF=0.9999029973809292;N_BI_GENOS=10309;Lingnan_Han_AN=1888;Lingnan_Han_AC=2;Lingn
chr1	225408966	.	A	<INS:ME:LINE1>	.	PASS	SVTYPE=INS:ME:LINE1;END=225408980;SVLEN=537;AN=20618;AC=2;AF=9.700261907071492e-05;N_HET=2;N_HOMALT=0;N_HOMREF=10307;FREQ_HET=0.00019400523814142983;FREQ_HOMALT=0.0;FREQ_HOMREF=0.9998059947618586;N_BI_GENOS=10309;South_Han_AN=1496;South_Han_AC=1;South_Han
chr1	225966420	.	A	<INS:ME:LINE1>	.	PASS	SVTYPE=INS:ME:LINE1;END=225966420;SVLEN=0;AN=20618;AC=1;AF=4.850130953535746e-05;N_HET=1;N_HOMALT=0;N_HOMREF=10308;FREQ_HET=9.700261907071492e-05;FREQ_HOMALT=0.0;FREQ_HOMREF=0.9999029973809292;N_BI_GENOS=10309;Northwest_Han_AN=1296;Northwest_Han_AC=1;Nort
chr1	226683876	.	A	<INS:ME:LINE1>	.	PASS	SVTYPE=INS:ME:LINE1;END=226683893;SVLEN=6008;AN=20618;AC=1;AF=4.850130953535746e-05;N_HET=1;N_HOMALT=0;N_HOMREF=10308;FREQ_HET=9.700261907071492e-05;FREQ_HOMALT=0.0;FREQ_HOMREF=0.9999029973809292;N_BI_GENOS=10309;East_Han_AN=6554;East_Han_AC=1;East_Han_AF
chr1	226730954	.	T	<INS:ME:LINE1>	.	PASS	SVTYPE=INS:ME:LINE1;END=226730961;SVLEN=971;AN=20618;AC=1;AF=4.850130953535746e-05;N_HET=1;N_HOMALT=0;N_HOMREF=10308;FREQ_HET=9.700261907071492e-05;FREQ_HOMALT=0.0;FREQ_HOMREF=0.9999029973809292;N_BI_GENOS=10309;Central_Han_AN=2128;Central_Han_AC=1;Centra
chr1	226855621	.	A	<INS:ME:LINE1>	.	PASS	SVTYPE=INS:ME:LINE1;END=226855632;SVLEN=6019;AN=20618;AC=1;AF=4.850130953535746e-05;N_HET=1;N_HOMALT=0;N_HOMREF=10308;FREQ_HET=9.700261907071492e-05;FREQ_HOMALT=0.0;FREQ_HOMREF=0.9999029973809292;N_BI_GENOS=10309;Central_Han_AN=2128;Central_Han_AC=1;Centr
chr1	227044541	.	T	<INS:ME:LINE1>	.	PASS	SVTYPE=INS:ME:LINE1;END=227044557;SVLEN=247;AN=20618;AC=1;AF=4.850130953535746e-05;N_HET=1;N_HOMALT=0;N_HOMREF=10308;FREQ_HET=9.700261907071492e-05;FREQ_HOMALT=0.0;FREQ_HOMREF=0.9999029973809292;N_BI_GENOS=10309;Lingnan_Han_AN=1888;Lingnan_Han_AC=1;Lingna
chr1	227088600	.	T	<INS:ME:LINE1>	.	PASS	SVTYPE=INS:ME:LINE1;END=227088601;SVLEN=211;AN=20618;AC=7;AF=0.0003395091667475022;N_HET=7;N_HOMALT=0;N_HOMREF=10302;FREQ_HET=0.0006790183334950044;FREQ_HOMALT=0.0;FREQ_HOMREF=0.999320981666505;N_BI_GENOS=10309;South_Han_AN=1496;South_Han_AC=1;South_Han_A
chr1	227102732	.	A	<INS:ME:LINE1>	.	PASS	SVTYPE=INS:ME:LINE1;END=227102749;SVLEN=5999;AN=20618;AC=4;AF=0.00019400523814142983;N_HET=4;N_HOMALT=0;N_HOMREF=10305;FREQ_HET=0.00038801047628285966;FREQ_HOMALT=0.0;FREQ_HOMREF=0.9996119895237171;N_BI_GENOS=10309;Lingnan_Han_AN=1888;Lingnan_Han_AC=1;Lin
chr1	227116010	.	C	<INS:ME:LINE1>	.	PASS	SVTYPE=INS:ME:LINE1;END=227116027;SVLEN=938;AN=20618;AC=1;AF=4.850130953535746e-05;N_HET=1;N_HOMALT=0;N_HOMREF=10308;FREQ_HET=9.700261907071492e-05;FREQ_HOMALT=0.0;FREQ_HOMREF=0.9999029973809292;N_BI_GENOS=10309;Hui_AN=444;Hui_AC=1;Hui_AF=0.0022522522522 5
chr1	227138759	.	T	<INS:ME:LINE1>	.	PASS	SVTYPE=INS:ME:LINE1;END=227138760;SVLEN=439;AN=20618;AC=3;AF=0.00014550392860607236;N_HET=1;N_HOMALT=1;N_HOMREF=10307;FREQ_HET=9.700261907071492e-05;FREQ_HOMALT=9.700261907071492e-05;FREQ_HOMREF=0.9998059947618586;N_BI_GENOS=10309;Central_Han_AN=2128;Cent
chr1	227163504	.	G	<INS:ME:LINE1>	.	PASS	SVTYPE=INS:ME:LINE1;END=227163510;SVLEN=411;AN=20618;AC=2;AF=9.700261907071492e-05;N_HET=2;N_HOMALT=0;N_HOMREF=10307;FREQ_HET=0.00019400523814142983;FREQ_HOMALT=0.0;FREQ_HOMREF=0.9998059947618586;N_BI_GENOS=10309;Central_Han_AN=2128;Central_Han_AC=2;Centr
chr1	227213696	.	G	<INS:ME:LINE1>	.	PASS	SVTYPE=INS:ME:LINE1;END=227213715;SVLEN=5684;AN=20618;AC=1;AF=4.850130953535746e-05;N_HET=1;N_HOMALT=0;N_HOMREF=10308;FREQ_HET=9.700261907071492e-05;FREQ_HOMALT=0.0;FREQ_HOMREF=0.9999029973809292;N_BI_GENOS=10309;East_Han_AN=6554;East_Han_AC=1;East_Han_AF
chr1	227248824	.	A	<INS:ME:LINE1>	.	PASS	SVTYPE=INS:ME:LINE1;END=227248840;SVLEN=5998;AN=20618;AC=2;AF=9.700261907071492e-05;N_HET=2;N_HOMALT=0;N_HOMREF=10307;FREQ_HET=0.00019400523814142983;FREQ_HOMALT=0.0;FREQ_HOMREF=0.9998059947618586;N_BI_GENOS=10309;Lingnan_Han_AN=1888;Lingnan_Han_AC=1;Ling
chr1	227594408	.	A	<INS:ME:LINE1>	.	PASS	SVTYPE=INS:ME:LINE1;END=227594423;SVLEN=853;AN=20618;AC=1;AF=4.850130953535746e-05;N_HET=1;N_HOMALT=0;N_HOMREF=10308;FREQ_HET=9.700261907071492e-05;FREQ_HOMALT=0.0;FREQ_HOMREF=0.9999029973809292;N_BI_GENOS=10309;East_Han_AN=6554;East_Han_AC=1;East_Han_AF=
chr1	229094222	.	C	<INS:ME:LINE1>	.	PASS	SVTYPE=INS:ME:LINE1;END=229094239;SVLEN=6000;AN=20618;AC=1;AF=4.850130953535746e-05;N_HET=1;N_HOMALT=0;N_HOMREF=10308;FREQ_HET=9.700261907071492e-05;FREQ_HOMALT=0.0;FREQ_HOMREF=0.9999029973809292;N_BI_GENOS=10309;North_Han_AN=2272;North_Han_AC=1;North_Han
chr1	229303785	.	T	<INS:ME:LINE1>	.	PASS	SVTYPE=INS:ME:LINE1;END=229303803;SVLEN=1417;AN=20618;AC=2;AF=9.700261907071492e-05;N_HET=2;N_HOMALT=0;N_HOMREF=10307;FREQ_HET=0.00019400523814142983;FREQ_HOMALT=0.0;FREQ_HOMREF=0.9998059947618586;N_BI_GENOS=10309;North_Han_AN=2272;North_Han_AC=1;North_Ha
chr1	229418657	.	G	<INS:ME:LINE1>	.	PASS	SVTYPE=INS:ME:LINE1;END=229418657;SVLEN=0;AN=20618;AC=1;AF=4.850130953535746e-05;N_HET=1;N_HOMALT=0;N_HOMREF=10308;FREQ_HET=9.700261907071492e-05;FREQ_HOMALT=0.0;FREQ_HOMREF=0.9999029973809292;N_BI_GENOS=10309;East_Han_AN=6554;East_Han_AC=1;East_Han_AF=0.
chr1	229964776	.	G	<INS:ME:LINE1>	.	PASS	SVTYPE=INS:ME:LINE1;END=229964794;SVLEN=5995;AN=20618;AC=1;AF=4.850130953535746e-05;N_HET=1;N_HOMALT=0;N_HOMREF=10308;FREQ_HET=9.700261907071492e-05;FREQ_HOMALT=0.0;FREQ_HOMREF=0.9999029973809292;N_BI_GENOS=10309;Hui_AN=444;Hui_AC=1;Hui_AF=0.0022522522522
chr1	230105831	.	A	<INS:ME:LINE1>	.	PASS	SVTYPE=INS:ME:LINE1;END=230105847;SVLEN=6000;AN=20618;AC=287;AF=0.01391987583664759;N_HET=261;N_HOMALT=13;N_HOMREF=10035;FREQ_HET=0.02531768357745659;FREQ_HOMALT=0.0012610340479192938;FREQ_HOMREF=0.9734212823746241;N_BI_GENOS=10309;South_Han_AN=1496;South
chr1	230527974	.	T	<INS:ME:LINE1>	.	PASS	SVTYPE=INS:ME:LINE1;END=230527991;SVLEN=899;AN=20618;AC=1;AF=4.850130953535746e-05;N_HET=1;N_HOMALT=0;N_HOMREF=10308;FREQ_HET=9.700261907071492e-05;FREQ_HOMALT=0.0;FREQ_HOMREF=0.9999029973809292;N_BI_GENOS=10309;Southeast_Han_AN=1908;Southeast_Han_AC=1;So
chr1	231046336	.	C	<INS:ME:LINE1>	.	PASS	SVTYPE=INS:ME:LINE1;END=231046336;SVLEN=0;AN=20618;AC=2;AF=9.700261907071492e-05;N_HET=2;N_HOMALT=0;N_HOMREF=10307;FREQ_HET=0.00019400523814142983;FREQ_HOMALT=0.0;FREQ_HOMREF=0.9998059947618586;N_BI_GENOS=10309;South_Han_AN=1496;South_Han_AC=1;South_Han_A
chr1	231363723	.	A	<INS:ME:LINE1>	.	PASS	SVTYPE=INS:ME:LINE1;END=231363739;SVLEN=539;AN=20618;AC=1;AF=4.850130953535746e-05;N_HET=1;N_HOMALT=0;N_HOMREF=10308;FREQ_HET=9.700261907071492e-05;FREQ_HOMALT=0.0;FREQ_HOMREF=0.9999029973809292;N_BI_GENOS=10309;Lingnan_Han_AN=1888;Lingnan_Han_AC=1;Lingna
chr1	231517840	.	A	<INS:ME:LINE1>	.	PASS	SVTYPE=INS:ME:LINE1;END=231517855;SVLEN=1590;AN=20618;AC=7;AF=0.0003395091667475022;N_HET=7;N_HOMALT=0;N_HOMREF=10302;FREQ_HET=0.0006790183334950044;FREQ_HOMALT=0.0;FREQ_HOMREF=0.999320981666505;N_BI_GENOS=10309;South_Han_AN=1496;South_Han_AC=2;South_Han_
chr1	232119888	.	A	<INS:ME:LINE1>	.	PASS	SVTYPE=INS:ME:LINE1;END=232119888;SVLEN=0;AN=20618;AC=2;AF=9.700261907071492e-05;N_HET=2;N_HOMALT=0;N_HOMREF=10307;FREQ_HET=0.00019400523814142983;FREQ_HOMALT=0.0;FREQ_HOMREF=0.9998059947618586;N_BI_GENOS=10309;South_Han_AN=1496;South_Han_AC=1;South_Han_A
chr1	233010819	.	A	<INS:ME:LINE1>	.	PASS	SVTYPE=INS:ME:LINE1;END=233010830;SVLEN=6059;AN=20618;AC=1;AF=4.850130953535746e-05;N_HET=1;N_HOMALT=0;N_HOMREF=10308;FREQ_HET=9.700261907071492e-05;FREQ_HOMALT=0.0;FREQ_HOMREF=0.9999029973809292;N_BI_GENOS=10309;Southeast_Han_AN=1908;Southeast_Han_AC=1;S
chr1	233171288	.	A	<INS:ME:LINE1>	.	PASS	SVTYPE=INS:ME:LINE1;END=233171304;SVLEN=5999;AN=20618;AC=1;AF=4.850130953535746e-05;N_HET=1;N_HOMALT=0;N_HOMREF=10308;FREQ_HET=9.700261907071492e-05;FREQ_HOMALT=0.0;FREQ_HOMREF=0.9999029973809292;N_BI_GENOS=10309;Tibetan_AN=414;Tibetan_AC=1;Tibetan_AF=0.0
chr1	233221137	.	A	<INS:ME:LINE1>	.	PASS	SVTYPE=INS:ME:LINE1;END=233221138;SVLEN=563;AN=20618;AC=1;AF=4.850130953535746e-05;N_HET=1;N_HOMALT=0;N_HOMREF=10308;FREQ_HET=9.700261907071492e-05;FREQ_HOMALT=0.0;FREQ_HOMREF=0.9999029973809292;N_BI_GENOS=10309;Southeast_Han_AN=1908;Southeast_Han_AC=1;So
chr1	233442705	.	C	<INS:ME:LINE1>	.	PASS	SVTYPE=INS:ME:LINE1;END=233442716;SVLEN=6002;AN=20618;AC=6;AF=0.0002910078572121447;N_HET=6;N_HOMALT=0;N_HOMREF=10303;FREQ_HET=0.0005820157144242894;FREQ_HOMALT=0.0;FREQ_HOMREF=0.9994179842855757;N_BI_GENOS=10309;Lingnan_Han_AN=1888;Lingnan_Han_AC=1;Lingn
chr1	233565932	.	T	<INS:ME:LINE1>	.	PASS	SVTYPE=INS:ME:LINE1;END=233565932;SVLEN=382;AN=20618;AC=1;AF=4.850130953535746e-05;N_HET=1;N_HOMALT=0;N_HOMREF=10308;FREQ_HET=9.700261907071492e-05;FREQ_HOMALT=0.0;FREQ_HOMREF=0.9999029973809292;N_BI_GENOS=10309;East_Han_AN=6554;East_Han_AC=1;East_Han_AF=
chr1	233874774	.	G	<INS:ME:LINE1>	.	PASS	SVTYPE=INS:ME:LINE1;END=233874788;SVLEN=5989;AN=20618;AC=2;AF=9.700261907071492e-05;N_HET=2;N_HOMALT=0;N_HOMREF=10307;FREQ_HET=0.00019400523814142983;FREQ_HOMALT=0.0;FREQ_HOMREF=0.9998059947618586;N_BI_GENOS=10309;Northwest_Han_AN=1296;Northwest_Han_AC=1;
chr1	234563622	.	C	<INS:ME:LINE1>	.	PASS	SVTYPE=INS:ME:LINE1;END=234563641;SVLEN=780;AN=20618;AC=6;AF=0.0002910078572121447;N_HET=6;N_HOMALT=0;N_HOMREF=10303;FREQ_HET=0.0005820157144242894;FREQ_HOMALT=0.0;FREQ_HOMREF=0.9994179842855757;N_BI_GENOS=10309;Central_Han_AN=2128;Central_Han_AC=2;Centra

chr1	235477829	.	A	<INS:ME:LINE1>	.	PASS	SVTYPE=INS:ME:LINE1;END=235477829;SVLEN=337;AN=20618;AC=2;AF=9.700261907071492e-05;N_HET=2;N_HOMALT=0;N_HOMREF=10307;FREQ_HET=0.00019400523814142983;FREQ_HOMALT=0.0;FREQ_HOMREF=0.9998059947618586;N_BI_GENOS=10309;South_Han_AN=1496;South_Han_AC=1;South_Han
chr1	235725959	.	A	<INS:ME:LINE1>	.	PASS	SVTYPE=INS:ME:LINE1;END=235725974;SVLEN=1024;AN=20618;AC=1;AF=4.850130953535746e-05;N_HET=1;N_HOMALT=0;N_HOMREF=10308;FREQ_HET=9.700261907071492e-05;FREQ_HOMALT=0.0;FREQ_HOMREF=0.9999029973809292;N_BI_GENOS=10309;Miao_AN=482;Miao_AC=1;Miao_AF=0.0020746887
chr1	235796313	.	A	<INS:ME:LINE1>	.	PASS	SVTYPE=INS:ME:LINE1;END=235796326;SVLEN=6008;AN=20618;AC=1;AF=4.850130953535746e-05;N_HET=1;N_HOMALT=0;N_HOMREF=10308;FREQ_HET=9.700261907071492e-05;FREQ_HOMALT=0.0;FREQ_HOMREF=0.9999029973809292;N_BI_GENOS=10309;Lingnan_Han_AN=1888;Lingnan_Han_AC=1;Lingn
chr1	235950327	.	T	<INS:ME:LINE1>	.	PASS	SVTYPE=INS:ME:LINE1;END=235950344;SVLEN=3402;AN=20618;AC=3;AF=0.00014550392860607236;N_HET=3;N_HOMALT=0;N_HOMREF=10306;FREQ_HET=0.0002910078572121447;FREQ_HOMALT=0.0;FREQ_HOMREF=0.9997089921427879;N_BI_GENOS=10309;Lingnan_Han_AN=1888;Lingnan_Han_AC=2;Ling
chr1	236090321	.	C	<INS:ME:LINE1>	.	PASS	SVTYPE=INS:ME:LINE1;END=236090331;SVLEN=5988;AN=20618;AC=1;AF=4.850130953535746e-05;N_HET=1;N_HOMALT=0;N_HOMREF=10308;FREQ_HET=9.700261907071492e-05;FREQ_HOMALT=0.0;FREQ_HOMREF=0.9999029973809292;N_BI_GENOS=10309;South_Han_AN=1496;South_Han_AC=1;South_Han
chr1	236206944	.	A	<INS:ME:LINE1>	.	PASS	SVTYPE=INS:ME:LINE1;END=236206959;SVLEN=5377;AN=20618;AC=1;AF=4.850130953535746e-05;N_HET=1;N_HOMALT=0;N_HOMREF=10308;FREQ_HET=9.700261907071492e-05;FREQ_HOMALT=0.0;FREQ_HOMREF=0.9999029973809292;N_BI_GENOS=10309;South_Han_AN=1496;South_Han_AC=1;South_Han
chr1	236220802	.	A	<INS:ME:LINE1>	.	PASS	SVTYPE=INS:ME:LINE1;END=236220813;SVLEN=444;AN=20618;AC=1;AF=4.850130953535746e-05;N_HET=1;N_HOMALT=0;N_HOMREF=10308;FREQ_HET=9.700261907071492e-05;FREQ_HOMALT=0.0;FREQ_HOMREF=0.9999029973809292;N_BI_GENOS=10309;East_Han_AN=6554;East_Han_AC=1;East_Han_AF=
chr1	236543619	.	A	<INS:ME:LINE1>	.	PASS	SVTYPE=INS:ME:LINE1;END=236543632;SVLEN=5999;AN=20618;AC=1;AF=4.850130953535746e-05;N_HET=1;N_HOMALT=0;N_HOMREF=10308;FREQ_HET=9.700261907071492e-05;FREQ_HOMALT=0.0;FREQ_HOMREF=0.9999029973809292;N_BI_GENOS=10309;East_Han_AN=6554;East_Han_AC=1;East_Han_AF
chr1	236814529	.	A	<INS:ME:LINE1>	.	PASS	SVTYPE=INS:ME:LINE1;END=236814543;SVLEN=806;AN=20618;AC=1;AF=4.850130953535746e-05;N_HET=1;N_HOMALT=0;N_HOMREF=10308;FREQ_HET=9.700261907071492e-05;FREQ_HOMALT=0.0;FREQ_HOMREF=0.9999029973809292;N_BI_GENOS=10309;Southeast_Han_AN=1908;Southeast_Han_AC=1;So
chr1	236838655	.	A	<INS:ME:LINE1>	.	PASS	SVTYPE=INS:ME:LINE1;END=236838671;SVLEN=6000;AN=20618;AC=1;AF=4.850130953535746e-05;N_HET=1;N_HOMALT=0;N_HOMREF=10308;FREQ_HET=9.700261907071492e-05;FREQ_HOMALT=0.0;FREQ_HOMREF=0.9999029973809292;N_BI_GENOS=10309;North_Han_AN=2272;North_Han_AC=1;North_Han
chr1	237450698	.	C	<INS:ME:LINE1>	.	PASS	SVTYPE=INS:ME:LINE1;END=237450714;SVLEN=140;AN=20618;AC=4;AF=0.00019400523814142983;N_HET=4;N_HOMALT=0;N_HOMREF=10305;FREQ_HET=0.00038801047628285966;FREQ_HOMALT=0.0;FREQ_HOMREF=0.9996119895237171;N_BI_GENOS=10309;South_Han_AN=1496;South_Han_AC=1;South_Ha
chr1	237473462	.	A	<INS:ME:LINE1>	.	PASS	SVTYPE=INS:ME:LINE1;END=237473462;SVLEN=0;AN=20618;AC=6;AF=0.0002910078572121447;N_HET=0;N_HOMALT=3;N_HOMREF=10306;FREQ_HET=0.0;FREQ_HOMALT=0.0002910078572121447;FREQ_HOMREF=0.9997089921427879;N_BI_GENOS=10309;East_Han_AN=6554;East_Han_AC=2;East_Han_AF=0.
chr1	237525954	.	G	<INS:ME:LINE1>	.	PASS	SVTYPE=INS:ME:LINE1;END=237525987;SVLEN=2106;AN=20618;AC=1;AF=4.850130953535746e-05;N_HET=1;N_HOMALT=0;N_HOMREF=10308;FREQ_HET=9.700261907071492e-05;FREQ_HOMALT=0.0;FREQ_HOMREF=0.9999029973809292;N_BI_GENOS=10309;East_Han_AN=6554;East_Han_AC=1;East_Han_AF
chr1	237749659	.	A	<INS:ME:LINE1>	.	PASS	SVTYPE=INS:ME:LINE1;END=237749671;SVLEN=2937;AN=20618;AC=1;AF=4.850130953535746e-05;N_HET=1;N_HOMALT=0;N_HOMREF=10308;FREQ_HET=9.700261907071492e-05;FREQ_HOMALT=0.0;FREQ_HOMREF=0.9999029973809292;N_BI_GENOS=10309;North_Han_AN=2272;North_Han_AC=1;North_Han
chr1	238304075	.	A	<INS:ME:LINE1>	.	PASS	SVTYPE=INS:ME:LINE1;END=238304089;SVLEN=1173;AN=20618;AC=1;AF=4.850130953535746e-05;N_HET=1;N_HOMALT=0;N_HOMREF=10308;FREQ_HET=9.700261907071492e-05;FREQ_HOMALT=0.0;FREQ_HOMREF=0.9999029973809292;N_BI_GENOS=10309;Lingnan_Han_AN=1888;Lingnan_Han_AC=1;Lingn
chr1	238353268	.	A	<INS:ME:LINE1>	.	PASS	SVTYPE=INS:ME:LINE1;END=238353268;SVLEN=383;AN=20618;AC=1;AF=4.850130953535746e-05;N_HET=1;N_HOMALT=0;N_HOMREF=10308;FREQ_HET=9.700261907071492e-05;FREQ_HOMALT=0.0;FREQ_HOMREF=0.9999029973809292;N_BI_GENOS=10309;East_Han_AN=6554;East_Han_AC=1;East_Han_AF=
chr1	238358947	.	A	<INS:ME:LINE1>	.	PASS	SVTYPE=INS:ME:LINE1;END=238358958;SVLEN=5986;AN=20618;AC=2;AF=9.700261907071492e-05;N_HET=2;N_HOMALT=0;N_HOMREF=10307;FREQ_HET=0.00019400523814142983;FREQ_HOMALT=0.0;FREQ_HOMREF=0.9998059947618586;N_BI_GENOS=10309;East_Han_AN=6554;East_Han_AC=1;East_Han_A
chr1	238527875	.	A	<INS:ME:LINE1>	.	PASS	SVTYPE=INS:ME:LINE1;END=238527891;SVLEN=2249;AN=20618;AC=1;AF=4.850130953535746e-05;N_HET=1;N_HOMALT=0;N_HOMREF=10308;FREQ_HET=9.700261907071492e-05;FREQ_HOMALT=0.0;FREQ_HOMREF=0.9999029973809292;N_BI_GENOS=10309;Lingnan_Han_AN=1888;Lingnan_Han_AC=1;Lingn
chr1	238584901	.	A	<INS:ME:LINE1>	.	PASS	SVTYPE=INS:ME:LINE1;END=238584919;SVLEN=2632;AN=20618;AC=1;AF=4.850130953535746e-05;N_HET=1;N_HOMALT=0;N_HOMREF=10308;FREQ_HET=9.700261907071492e-05;FREQ_HOMALT=0.0;FREQ_HOMREF=0.9999029973809292;N_BI_GENOS=10309;East_Han_AN=6554;East_Han_AC=1;East_Han_AF
chr1	238982495	.	A	<INS:ME:LINE1>	.	PASS	SVTYPE=INS:ME:LINE1;END=238982509;SVLEN=3526;AN=20618;AC=1;AF=4.850130953535746e-05;N_HET=1;N_HOMALT=0;N_HOMREF=10308;FREQ_HET=9.700261907071492e-05;FREQ_HOMALT=0.0;FREQ_HOMREF=0.9999029973809292;N_BI_GENOS=10309;Mongolian_AN=454;Mongolian_AC=1;Mongolian_
chr1	239105844	.	C	<INS:ME:LINE1>	.	PASS	SVTYPE=INS:ME:LINE1;END=239105844;SVLEN=0;AN=20618;AC=6;AF=0.0002910078572121447;N_HET=4;N_HOMALT=1;N_HOMREF=10304;FREQ_HET=0.00038801047628285966;FREQ_HOMALT=9.700261907071492e-05;FREQ_HOMREF=0.9995149869046465;N_BI_GENOS=10309;South_Han_AN=1496;South_Ha
chr1	239281090	.	C	<INS:ME:LINE1>	.	PASS	SVTYPE=INS:ME:LINE1;END=239281098;SVLEN=548;AN=20618;AC=1;AF=4.850130953535746e-05;N_HET=1;N_HOMALT=0;N_HOMREF=10308;FREQ_HET=9.700261907071492e-05;FREQ_HOMALT=0.0;FREQ_HOMREF=0.9999029973809292;N_BI_GENOS=10309;Southeast_Han_AN=1908;Southeast_Han_AC=1;So
chr1	239473153	.	A	<INS:ME:LINE1>	.	PASS	SVTYPE=INS:ME:LINE1;END=239473156;SVLEN=475;AN=20618;AC=1;AF=4.850130953535746e-05;N_HET=1;N_HOMALT=0;N_HOMREF=10308;FREQ_HET=9.700261907071492e-05;FREQ_HOMALT=0.0;FREQ_HOMREF=0.9999029973809292;N_BI_GENOS=10309;Southeast_Han_AN=1908;Southeast_Han_AC=1;So
chr1	239946977	.	A	<INS:ME:LINE1>	.	PASS	SVTYPE=INS:ME:LINE1;END=239946993;SVLEN=5997;AN=20618;AC=3;AF=0.00014550392860607236;N_HET=3;N_HOMALT=0;N_HOMREF=10306;FREQ_HET=0.0002910078572121447;FREQ_HOMALT=0.0;FREQ_HOMREF=0.9997089921427879;N_BI_GENOS=10309;Southeast_Han_AN=1908;Southeast_Han_AC=3;
chr1	240219519	.	A	<INS:ME:LINE1>	.	PASS	SVTYPE=INS:ME:LINE1;END=240219530;SVLEN=359;AN=20618;AC=122;AF=0.005917159763313609;N_HET=112;N_HOMALT=5;N_HOMREF=10192;FREQ_HET=0.01086429333592007;FREQ_HOMALT=0.00048501309535357455;FREQ_HOMREF=0.9886506935687264;N_BI_GENOS=10309;South_Han_AN=1496;South
chr1	240237128	.	A	<INS:ME:LINE1>	.	PASS	SVTYPE=INS:ME:LINE1;END=240237143;SVLEN=2042;AN=20618;AC=1;AF=4.850130953535746e-05;N_HET=1;N_HOMALT=0;N_HOMREF=10308;FREQ_HET=9.700261907071492e-05;FREQ_HOMALT=0.0;FREQ_HOMREF=0.9999029973809292;N_BI_GENOS=10309;North_Han_AN=2272;North_Han_AC=1;North_Han
chr1	240268872	.	T	<INS:ME:LINE1>	.	PASS	SVTYPE=INS:ME:LINE1;END=240268915;SVLEN=1138;AN=20618;AC=1;AF=4.850130953535746e-05;N_HET=1;N_HOMALT=0;N_HOMREF=10308;FREQ_HET=9.700261907071492e-05;FREQ_HOMALT=0.0;FREQ_HOMREF=0.9999029973809292;N_BI_GENOS=10309;East_Han_AN=6554;East_Han_AC=1;East_Han_AF
chr1	240291145	.	T	<INS:ME:LINE1>	.	PASS	SVTYPE=INS:ME:LINE1;END=240291151;SVLEN=3492;AN=20618;AC=4;AF=0.00019400523814142983;N_HET=4;N_HOMALT=0;N_HOMREF=10305;FREQ_HET=0.00038801047628285966;FREQ_HOMALT=0.0;FREQ_HOMREF=0.9996119895237171;N_BI_GENOS=10309;South_Han_AN=1496;South_Han_AC=1;South_H
chr1	240384114	.	A	<INS:ME:LINE1>	.	PASS	SVTYPE=INS:ME:LINE1;END=240384131;SVLEN=5988;AN=20618;AC=18;AF=0.0008730235716364342;N_HET=18;N_HOMALT=0;N_HOMREF=10291;FREQ_HET=0.0017460471432728683;FREQ_HOMALT=0.0;FREQ_HOMREF=0.9982539528567271;N_BI_GENOS=10309;South_Han_AN=1496;South_Han_AC=5;South_H
chr1	240394967	.	A	<INS:ME:LINE1>	.	PASS	SVTYPE=INS:ME:LINE1;END=240394974;SVLEN=1607;AN=20618;AC=1;AF=4.850130953535746e-05;N_HET=1;N_HOMALT=0;N_HOMREF=10308;FREQ_HET=9.700261907071492e-05;FREQ_HOMALT=0.0;FREQ_HOMREF=0.9999029973809292;N_BI_GENOS=10309;South_Han_AN=1496;South_Han_AC=1;South_Han
chr1	241736416	.	A	<INS:ME:LINE1>	.	PASS	SVTYPE=INS:ME:LINE1;END=241736418;SVLEN=3308;AN=20618;AC=1;AF=4.850130953535746e-05;N_HET=1;N_HOMALT=0;N_HOMREF=10308;FREQ_HET=9.700261907071492e-05;FREQ_HOMALT=0.0;FREQ_HOMREF=0.9999029973809292;N_BI_GENOS=10309;Lingnan_Han_AN=1888;Lingnan_Han_AC=1;Lingn
chr1	241761206	.	G	<INS:ME:LINE1>	.	PASS	SVTYPE=INS:ME:LINE1;END=241761216;SVLEN=5998;AN=20618;AC=1;AF=4.850130953535746e-05;N_HET=1;N_HOMALT=0;N_HOMREF=10308;FREQ_HET=9.700261907071492e-05;FREQ_HOMALT=0.0;FREQ_HOMREF=0.9999029973809292;N_BI_GENOS=10309;Northwest_Han_AN=1296;Northwest_Han_AC=1;N
chr1	242019904	.	T	<INS:ME:LINE1>	.	PASS	SVTYPE=INS:ME:LINE1;END=242019918;SVLEN=6000;AN=20618;AC=1;AF=4.850130953535746e-05;N_HET=1;N_HOMALT=0;N_HOMREF=10308;FREQ_HET=9.700261907071492e-05;FREQ_HOMALT=0.0;FREQ_HOMREF=0.9999029973809292;N_BI_GENOS=10309;North_Han_AN=2272;North_Han_AC=1;North_Han
chr1	242148259	.	G	<INS:ME:LINE1>	.	PASS	SVTYPE=INS:ME:LINE1;END=242148275;SVLEN=6000;AN=20618;AC=1416;AF=0.06867785430206616;N_HET=1182;N_HOMALT=117;N_HOMREF=9010;FREQ_HET=0.11465709574158502;FREQ_HOMALT=0.011349306431273645;FREQ_HOMREF=0.8739935978271414;N_BI_GENOS=10309;South_Han_AN=1496;Sout
chr1	242171687	.	G	<INS:ME:LINE1>	.	PASS	SVTYPE=INS:ME:LINE1;END=242171688;SVLEN=3879;AN=20618;AC=1;AF=4.850130953535746e-05;N_HET=1;N_HOMALT=0;N_HOMREF=10308;FREQ_HET=9.700261907071492e-05;FREQ_HOMALT=0.0;FREQ_HOMREF=0.9999029973809292;N_BI_GENOS=10309;East_Han_AN=6554;East_Han_AC=1;East_Han_AF
chr1	242210475	.	G	<INS:ME:LINE1>	.	PASS	SVTYPE=INS:ME:LINE1;END=242210475;SVLEN=0;AN=20618;AC=2;AF=9.700261907071492e-05;N_HET=2;N_HOMALT=0;N_HOMREF=10307;FREQ_HET=0.00019400523814142983;FREQ_HOMALT=0.0;FREQ_HOMREF=0.9998059947618586;N_BI_GENOS=10309;Northwest_Han_AN=1296;Northwest_Han_AC=1;Nor
chr1	242210551	.	A	<INS:ME:LINE1>	.	PASS	SVTYPE=INS:ME:LINE1;END=242210551;SVLEN=0;AN=20618;AC=2;AF=9.700261907071492e-05;N_HET=0;N_HOMALT=1;N_HOMREF=10308;FREQ_HET=0.0;FREQ_HOMALT=9.700261907071492e-05;FREQ_HOMREF=0.9999029973809292;N_BI_GENOS=10309;South_Han_AN=1496;South_Han_AC=2;South_Han_AF
chr1	242239607	.	A	<INS:ME:LINE1>	.	PASS	SVTYPE=INS:ME:LINE1;END=242239620;SVLEN=140;AN=20618;AC=1;AF=4.850130953535746e-05;N_HET=1;N_HOMALT=0;N_HOMREF=10308;FREQ_HET=9.700261907071492e-05;FREQ_HOMALT=0.0;FREQ_HOMREF=0.9999029973809292;N_BI_GENOS=10309;South_Han_AN=1496;South_Han_AC=1;South_Han_
chr1	242266575	.	A	<INS:ME:LINE1>	.	PASS	SVTYPE=INS:ME:LINE1;END=242266590;SVLEN=1001;AN=20618;AC=1;AF=4.850130953535746e-05;N_HET=1;N_HOMALT=0;N_HOMREF=10308;FREQ_HET=9.700261907071492e-05;FREQ_HOMALT=0.0;FREQ_HOMREF=0.9999029973809292;N_BI_GENOS=10309;Central_Han_AN=2128;Central_Han_AC=1;Centr
chr1	242580396	.	T	<INS:ME:LINE1>	.	PASS	SVTYPE=INS:ME:LINE1;END=242580410;SVLEN=896;AN=20618;AC=1;AF=4.850130953535746e-05;N_HET=1;N_HOMALT=0;N_HOMREF=10308;FREQ_HET=9.700261907071492e-05;FREQ_HOMALT=0.0;FREQ_HOMREF=0.9999029973809292;N_BI_GENOS=10309;North_Han_AN=2272;North_Han_AC=1;North_Han_
chr1	242637084	.	A	<INS:ME:LINE1>	.	PASS	SVTYPE=INS:ME:LINE1;END=242637095;SVLEN=415;AN=20618;AC=1;AF=4.850130953535746e-05;N_HET=1;N_HOMALT=0;N_HOMREF=10308;FREQ_HET=9.700261907071492e-05;FREQ_HOMALT=0.0;FREQ_HOMREF=0.9999029973809292;N_BI_GENOS=10309;Hui_AN=444;Hui_AC=1;Hui_AF=0.00225225225225
chr1	242644057	.	G	<INS:ME:LINE1>	.	PASS	SVTYPE=INS:ME:LINE1;END=242644072;SVLEN=934;AN=20618;AC=1;AF=4.850130953535746e-05;N_HET=1;N_HOMALT=0;N_HOMREF=10308;FREQ_HET=9.700261907071492e-05;FREQ_HOMALT=0.0;FREQ_HOMREF=0.9999029973809292;N_BI_GENOS=10309;East_Han_AN=6554;East_Han_AC=1;East_Han_AF=
chr1	242727536	.	A	<INS:ME:LINE1>	.	PASS	SVTYPE=INS:ME:LINE1;END=242727550;SVLEN=345;AN=20618;AC=5;AF=0.00024250654767678728;N_HET=5;N_HOMALT=0;N_HOMREF=10304;FREQ_HET=0.00048501309535357455;FREQ_HOMALT=0.0;FREQ_HOMREF=0.9995149869046465;N_BI_GENOS=10309;East_Han_AN=6554;East_Han_AC=2;East_Han_A
chr1	242857941	.	A	<INS:ME:LINE1>	.	PASS	SVTYPE=INS:ME:LINE1;END=242857956;SVLEN=5492;AN=20618;AC=1;AF=4.850130953535746e-05;N_HET=1;N_HOMALT=0;N_HOMREF=10308;FREQ_HET=9.700261907071492e-05;FREQ_HOMALT=0.0;FREQ_HOMREF=0.9999029973809292;N_BI_GENOS=10309;North_Han_AN=2272;North_Han_AC=1;North_Han
chr1	244142647	.	T	<INS:ME:LINE1>	.	PASS	SVTYPE=INS:ME:LINE1;END=244142657;SVLEN=6000;AN=20618;AC=1;AF=4.850130953535746e-05;N_HET=1;N_HOMALT=0;N_HOMREF=10308;FREQ_HET=9.700261907071492e-05;FREQ_HOMALT=0.0;FREQ_HOMREF=0.9999029973809292;N_BI_GENOS=10309;East_Han_AN=6554;East_Han_AC=1;East_Han_AF
chr1	244838363	.	A	<INS:ME:LINE1>	.	PASS	SVTYPE=INS:ME:LINE1;END=244838380;SVLEN=356;AN=20618;AC=1;AF=4.850130953535746e-05;N_HET=1;N_HOMALT=0;N_HOMREF=10308;FREQ_HET=9.700261907071492e-05;FREQ_HOMALT=0.0;FREQ_HOMREF=0.9999029973809292;N_BI_GENOS=10309;East_Han_AN=6554;East_Han_AC=1;East_Han_AF=
chr1	246070643	.	A	<INS:ME:LINE1>	.	PASS	SVTYPE=INS:ME:LINE1;END=246070659;SVLEN=6033;AN=20618;AC=1;AF=4.850130953535746e-05;N_HET=1;N_HOMALT=0;N_HOMREF=10308;FREQ_HET=9.700261907071492e-05;FREQ_HOMALT=0.0;FREQ_HOMREF=0.9999029973809292;N_BI_GENOS=10309;Hui_AN=444;Hui_AC=1;Hui_AF=0.0022522522522
chr1	246117321	.	A	<INS:ME:LINE1>	.	PASS	SVTYPE=INS:ME:LINE1;END=246117337;SVLEN=658;AN=20618;AC=2;AF=9.700261907071492e-05;N_HET=0;N_HOMALT=1;N_HOMREF=10308;FREQ_HET=0.0;FREQ_HOMALT=9.700261907071492e-05;FREQ_HOMREF=0.9999029973809292;N_BI_GENOS=10309;Central_Han_AN=2128;Central_Han_AC=2;Centra
chr1	246608850	.	A	<INS:ME:LINE1>	.	PASS	SVTYPE=INS:ME:LINE1;END=246608850;SVLEN=87;AN=20618;AC=1;AF=4.850130953535746e-05;N_HET=1;N_HOMALT=0;N_HOMREF=10308;FREQ_HET=9.700261907071492e-05;FREQ_HOMALT=0.0;FREQ_HOMREF=0.9999029973809292;N_BI_GENOS=10309;Lingnan_Han_AN=1888;Lingnan_Han_AC=1;Lingnan
chr1	246688880	.	G	<INS:ME:LINE1>	.	PASS	SVTYPE=INS:ME:LINE1;END=246688896;SVLEN=212;AN=20618;AC=1;AF=4.850130953535746e-05;N_HET=1;N_HOMALT=0;N_HOMREF=10308;FREQ_HET=9.700261907071492e-05;FREQ_HOMALT=0.0;FREQ_HOMREF=0.9999029973809292;N_BI_GENOS=10309;Northwest_Han_AN=1296;Northwest_Han_AC=1;No
chr1	247505821	.	A	<INS:ME:LINE1>	.	PASS	SVTYPE=INS:ME:LINE1;END=247505835;SVLEN=171;AN=20618;AC=1;AF=4.850130953535746e-05;N_HET=1;N_HOMALT=0;N_HOMREF=10308;FREQ_HET=9.700261907071492e-05;FREQ_HOMALT=0.0;FREQ_HOMREF=0.9999029973809292;N_BI_GENOS=10309;South_Han_AN=1496;South_Han_AC=1;South_Han_
chr1	247596582	.	T	<INS:ME:LINE1>	.	PASS	SVTYPE=INS:ME:LINE1;END=247596582;SVLEN=629;AN=20618;AC=1;AF=4.850130953535746e-05;N_HET=1;N_HOMALT=0;N_HOMREF=10308;FREQ_HET=9.700261907071492e-05;FREQ_HOMALT=0.0;FREQ_HOMREF=0.9999029973809292;N_BI_GENOS=10309;East_Han_AN=6554;East_Han_AC=1;East_Han_AF=
chr1	247596739	.	A	<INS:ME:LINE1>	.	PASS	SVTYPE=INS:ME:LINE1;END=247596739;SVLEN=104;AN=20618;AC=23;AF=0.0011155301193132214;N_HET=11;N_HOMALT=6;N_HOMREF=10292;FREQ_HET=0.001067028809777864;FREQ_HOMALT=0.0005820157144242894;FREQ_HOMREF=0.9983509554757979;N_BI_GENOS=10309;Lingnan_Han_AN=1888;Ling
chr1	247681345	.	A	<INS:ME:LINE1>	.	PASS	SVTYPE=INS:ME:LINE1;END=247681356;SVLEN=5964;AN=20618;AC=3;AF=0.00014550392860607236;N_HET=3;N_HOMALT=0;N_HOMREF=10306;FREQ_HET=0.0002910078572121447;FREQ_HOMALT=0.0;FREQ_HOMREF=0.9997089921427879;N_BI_GENOS=10309;North_Han_AN=2272;North_Han_AC=1;North_Ha
chr1	247748075	.	A	<INS:ME:LINE1>	.	PASS	SVTYPE=INS:ME:LINE1;END=247748090;SVLEN=404;AN=20618;AC=2;AF=9.700261907071492e-05;N_HET=2;N_HOMALT=0;N_HOMREF=10307;FREQ_HET=0.00019400523814142983;FREQ_HOMALT=0.0;FREQ_HOMREF=0.9998059947618586;N_BI_GENOS=10309;Lingnan_Han_AN=1888;Lingnan_Han_AC=1;Lingn
chr1	247913214	.	A	<INS:ME:LINE1>	.	PASS	SVTYPE=INS:ME:LINE1;END=247913214;SVLEN=987;AN=20618;AC=1;AF=4.850130953535746e-05;N_HET=1;N_HOMALT=0;N_HOMREF=10308;FREQ_HET=9.700261907071492e-05;FREQ_HOMALT=0.0;FREQ_HOMREF=0.9999029973809292;N_BI_GENOS=10309;East_Han_AN=6554;East_Han_AC=1;East_Han_AF=
chr1	248265243	.	C	<INS:ME:LINE1>	.	PASS	SVTYPE=INS:ME:LINE1;END=248265243;SVLEN=229;AN=20618;AC=3;AF=0.00014550392860607236;N_HET=1;N_HOMALT=1;N_HOMREF=10307;FREQ_HET=9.700261907071492e-05;FREQ_HOMALT=9.700261907071492e-05;FREQ_HOMREF=0.9998059947618586;N_BI_GENOS=10309;East_Han_AN=6554;East_Ha
chr1	248338734	.	A	<INS:ME:LINE1>	.	PASS	SVTYPE=INS:ME:LINE1;END=248338734;SVLEN=0;AN=20618;AC=3;AF=0.00014550392860607236;N_HET=1;N_HOMALT=1;N_HOMREF=10307;FREQ_HET=9.700261907071492e-05;FREQ_HOMALT=9.700261907071492e-05;FREQ_HOMREF=0.9998059947618586;N_BI_GENOS=10309;East_Han_AN=6554;East_Han_
chr1	248614476	.	G	<INS:ME:LINE1>	.	PASS	SVTYPE=INS:ME:LINE1;END=248614493;SVLEN=465;AN=20618;AC=15;AF=0.0007275196430303619;N_HET=11;N_HOMALT=2;N_HOMREF=10296;FREQ_HET=0.001067028809777864;FREQ_HOMALT=0.00019400523814142983;FREQ_HOMREF=0.9987389659520807;N_BI_GENOS=10309;South_Han_AN=1496;South
chr1	248698912	.	A	<INS:ME:LINE1>	.	PASS	SVTYPE=INS:ME:LINE1;END=248698930;SVLEN=419;AN=20618;AC=1;AF=4.850130953535746e-05;N_HET=1;N_HOMALT=0;N_HOMREF=10308;FREQ_HET=9.700261907071492e-05;FREQ_HOMALT=0.0;FREQ_HOMREF=0.9999029973809292;N_BI_GENOS=10309;Southeast_Han_AN=1908;Southeast_Han_AC=1;So
chr1	248897262	.	A	<INS:ME:LINE1>	.	PASS	SVTYPE=INS:ME:LINE1;END=248897273;SVLEN=554;AN=20618;AC=2896;AF=0.14045979241439518;N_HET=2344;N_HOMALT=276;N_HOMREF=7689;FREQ_HET=0.22737413910175575;FREQ_HOMALT=0.026772722863517315;FREQ_HOMREF=0.745853138034727;N_BI_GENOS=10309;South_Han_AN=1496;South_
chr2	102005	.	A	<INS:ME:LINE1>	.	PASS	SVTYPE=INS:ME:LINE1;END=102018;SVLEN=2221;AN=20618;AC=2;AF=9.700261907071492e-05;N_HET=2;N_HOMALT=0;N_HOMREF=10307;FREQ_HET=0.00019400523814142983;FREQ_HOMALT=0.0;FREQ_HOMREF=0.9998059947618586;N_BI_GENOS=10309;Northwest_Han_AN=1296;Northwest_Han_AC=1;Nor
chr2	514138	.	G	<INS:ME:LINE1>	.	PASS	SVTYPE=INS:ME:LINE1;END=514150;SVLEN=1137;AN=20618;AC=1;AF=4.850130953535746e-05;N_HET=1;N_HOMALT=0;N_HOMREF=10308;FREQ_HET=9.700261907071492e-05;FREQ_HOMALT=0.0;FREQ_HOMREF=0.9999029973809292;N_BI_GENOS=10309;Yi_AN=422;Yi_AC=1;Yi_AF=0.002369668246445498;
chr2	517240	.	C	<INS:ME:LINE1>	.	PASS	SVTYPE=INS:ME:LINE1;END=517240;SVLEN=1010;AN=20618;AC=1;AF=4.850130953535746e-05;N_HET=1;N_HOMALT=0;N_HOMREF=10308;FREQ_HET=9.700261907071492e-05;FREQ_HOMALT=0.0;FREQ_HOMREF=0.9999029973809292;N_BI_GENOS=10309;East_Han_AN=6554;East_Han_AC=1;East_Han_AF=0.
chr2	703176	.	C	<INS:ME:LINE1>	.	PASS	SVTYPE=INS:ME:LINE1;END=703176;SVLEN=0;AN=20618;AC=1;AF=4.850130953535746e-05;N_HET=1;N_HOMALT=0;N_HOMREF=10308;FREQ_HET=9.700261907071492e-05;FREQ_HOMALT=0.0;FREQ_HOMREF=0.9999029973809292;N_BI_GENOS=10309;East_Han_AN=6554;East_Han_AC=1;East_Han_AF=0.000
chr2	969520	.	G	<INS:ME:LINE1>	.	PASS	SVTYPE=INS:ME:LINE1;END=969520;SVLEN=881;AN=20618;AC=1;AF=4.850130953535746e-05;N_HET=1;N_HOMALT=0;N_HOMREF=10308;FREQ_HET=9.700261907071492e-05;FREQ_HOMALT=0.0;FREQ_HOMREF=0.9999029973809292;N_BI_GENOS=10309;East_Han_AN=6554;East_Han_AC=1;East_Han_AF=0.0
chr2	1641462	.	T	<INS:ME:LINE1>	.	PASS	SVTYPE=INS:ME:LINE1;END=1641476;SVLEN=773;AN=20618;AC=1;AF=4.850130953535746e-05;N_HET=1;N_HOMALT=0;N_HOMREF=10308;FREQ_HET=9.700261907071492e-05;FREQ_HOMALT=0.0;FREQ_HOMREF=0.9999029973809292;N_BI_GENOS=10309;North_Han_AN=2272;North_Han_AC=1;North_Han_AF
chr2	2139922	.	A	<INS:ME:LINE1>	.	PASS	SVTYPE=INS:ME:LINE1;END=2139937;SVLEN=721;AN=20618;AC=1;AF=4.850130953535746e-05;N_HET=1;N_HOMALT=0;N_HOMREF=10308;FREQ_HET=9.700261907071492e-05;FREQ_HOMALT=0.0;FREQ_HOMREF=0.9999029973809292;N_BI_GENOS=10309;East_Han_AN=6554;East_Han_AC=1;East_Han_AF=0.
chr2	2157016	.	G	<INS:ME:LINE1>	.	PASS	SVTYPE=INS:ME:LINE1;END=2157032;SVLEN=6056;AN=20618;AC=3;AF=0.00014550392860607236;N_HET=3;N_HOMALT=0;N_HOMREF=10306;FREQ_HET=0.0002910078572121447;FREQ_HOMALT=0.0;FREQ_HOMREF=0.9997089921427879;N_BI_GENOS=10309;Miao_AN=482;Miao_AC=3;Miao_AF=0.00622406639
chr2	2647544	.	C	<INS:ME:LINE1>	.	PASS	SVTYPE=INS:ME:LINE1;END=2647556;SVLEN=980;AN=20618;AC=2;AF=9.700261907071492e-05;N_HET=2;N_HOMALT=0;N_HOMREF=10307;FREQ_HET=0.00019400523814142983;FREQ_HOMALT=0.0;FREQ_HOMREF=0.9998059947618586;N_BI_GENOS=10309;East_Han_AN=6554;East_Han_AC=2;East_Han_AF=0
chr2	3240010	.	A	<INS:ME:LINE1>	.	PASS	SVTYPE=INS:ME:LINE1;END=3240026;SVLEN=283;AN=20618;AC=2;AF=9.700261907071492e-05;N_HET=2;N_HOMALT=0;N_HOMREF=10307;FREQ_HET=0.00019400523814142983;FREQ_HOMALT=0.0;FREQ_HOMREF=0.9998059947618586;N_BI_GENOS=10309;East_Han_AN=6554;East_Han_AC=2;East_Han_AF=0
chr2	4035026	.	T	<INS:ME:LINE1>	.	PASS	SVTYPE=INS:ME:LINE1;END=4035045;SVLEN=5997;AN=20618;AC=1;AF=4.850130953535746e-05;N_HET=1;N_HOMALT=0;N_HOMREF=10308;FREQ_HET=9.700261907071492e-05;FREQ_HOMALT=0.0;FREQ_HOMREF=0.9999029973809292;N_BI_GENOS=10309;Tibetan_AN=414;Tibetan_AC=1;Tibetan_AF=0.002
chr2	4153326	.	T	<INS:ME:LINE1>	.	PASS	SVTYPE=INS:ME:LINE1;END=4153342;SVLEN=1491;AN=20618;AC=1;AF=4.850130953535746e-05;N_HET=1;N_HOMALT=0;N_HOMREF=10308;FREQ_HET=9.700261907071492e-05;FREQ_HOMALT=0.0;FREQ_HOMREF=0.9999029973809292;N_BI_GENOS=10309;Central_Han_AN=2128;Central_Han_AC=1;Central
chr2	4232895	.	A	<INS:ME:LINE1>	.	PASS	SVTYPE=INS:ME:LINE1;END=4232912;SVLEN=3863;AN=20618;AC=1;AF=4.850130953535746e-05;N_HET=1;N_HOMALT=0;N_HOMREF=10308;FREQ_HET=9.700261907071492e-05;FREQ_HOMALT=0.0;FREQ_HOMREF=0.9999029973809292;N_BI_GENOS=10309;Central_Han_AN=2128;Central_Han_AC=1;Central
chr2	4259138	.	C	<INS:ME:LINE1>	.	PASS	SVTYPE=INS:ME:LINE1;END=4259150;SVLEN=6038;AN=20618;AC=1;AF=4.850130953535746e-05;N_HET=1;N_HOMALT=0;N_HOMREF=10308;FREQ_HET=9.700261907071492e-05;FREQ_HOMALT=0.0;FREQ_HOMREF=0.9999029973809292;N_BI_GENOS=10309;East_Han_AN=6554;East_Han_AC=1;East_Han_AF=0
chr2	4393967	.	T	<INS:ME:LINE1>	.	PASS	SVTYPE=INS:ME:LINE1;END=4393981;SVLEN=488;AN=20618;AC=61;AF=0.0029585798816568047;N_HET=59;N_HOMALT=1;N_HOMREF=10249;FREQ_HET=0.0057231545251721794;FREQ_HOMALT=9.700261907071492e-05;FREQ_HOMREF=0.9941798428557571;N_BI_GENOS=10309;South_Han_AN=1496;South_H
chr2	4412278	.	T	<INS:ME:LINE1>	.	PASS	SVTYPE=INS:ME:LINE1;END=4412294;SVLEN=429;AN=20618;AC=3;AF=0.00014550392860607236;N_HET=3;N_HOMALT=0;N_HOMREF=10306;FREQ_HET=0.0002910078572121447;FREQ_HOMALT=0.0;FREQ_HOMREF=0.9997089921427879;N_BI_GENOS=10309;South_Han_AN=1496;South_Han_AC=1;South_Han_A
chr2	4773817	.	A	<INS:ME:LINE1>	.	PASS	SVTYPE=INS:ME:LINE1;END=4773817;SVLEN=5977;AN=20618;AC=1;AF=4.850130953535746e-05;N_HET=1;N_HOMALT=0;N_HOMREF=10308;FREQ_HET=9.700261907071492e-05;FREQ_HOMALT=0.0;FREQ_HOMREF=0.9999029973809292;N_BI_GENOS=10309;Southeast_Han_AN=1908;Southeast_Han_AC=1;Sou
chr2	4934987	.	T	<INS:ME:LINE1>	.	PASS	SVTYPE=INS:ME:LINE1;END=4934987;SVLEN=0;AN=20618;AC=1;AF=4.850130953535746e-05;N_HET=1;N_HOMALT=0;N_HOMREF=10308;FREQ_HET=9.700261907071492e-05;FREQ_HOMALT=0.0;FREQ_HOMREF=0.9999029973809292;N_BI_GENOS=10309;North_Han_AN=2272;North_Han_AC=1;North_Han_AF=0
chr2	5314359	.	T	<INS:ME:LINE1>	.	PASS	SVTYPE=INS:ME:LINE1;END=5314364;SVLEN=1239;AN=20618;AC=1;AF=4.850130953535746e-05;N_HET=1;N_HOMALT=0;N_HOMREF=10308;FREQ_HET=9.700261907071492e-05;FREQ_HOMALT=0.0;FREQ_HOMREF=0.9999029973809292;N_BI_GENOS=10309;Southeast_Han_AN=1908;Southeast_Han_AC=1;Sou
chr2	5529480	.	C	<INS:ME:LINE1>	.	PASS	SVTYPE=INS:ME:LINE1;END=5529493;SVLEN=656;AN=20618;AC=1;AF=4.850130953535746e-05;N_HET=1;N_HOMALT=0;N_HOMREF=10308;FREQ_HET=9.700261907071492e-05;FREQ_HOMALT=0.0;FREQ_HOMREF=0.9999029973809292;N_BI_GENOS=10309;South_Han_AN=1496;South_Han_AC=1;South_Han_AF
chr2	5575786	.	G	<INS:ME:LINE1>	.	PASS	SVTYPE=INS:ME:LINE1;END=5575786;SVLEN=1392;AN=20618;AC=1;AF=4.850130953535746e-05;N_HET=1;N_HOMALT=0;N_HOMREF=10308;FREQ_HET=9.700261907071492e-05;FREQ_HOMALT=0.0;FREQ_HOMREF=0.9999029973809292;N_BI_GENOS=10309;East_Han_AN=6554;East_Han_AC=1;East_Han_AF=0
chr2	5774257	.	T	<INS:ME:LINE1>	.	PASS	SVTYPE=INS:ME:LINE1;END=5774263;SVLEN=1088;AN=20618;AC=1;AF=4.850130953535746e-05;N_HET=1;N_HOMALT=0;N_HOMREF=10308;FREQ_HET=9.700261907071492e-05;FREQ_HOMALT=0.0;FREQ_HOMREF=0.9999029973809292;N_BI_GENOS=10309;Central_Han_AN=2128;Central_Han_AC=1;Central
chr2	5879423	.	A	<INS:ME:LINE1>	.	PASS	SVTYPE=INS:ME:LINE1;END=5879437;SVLEN=5970;AN=20618;AC=1;AF=4.850130953535746e-05;N_HET=1;N_HOMALT=0;N_HOMREF=10308;FREQ_HET=9.700261907071492e-05;FREQ_HOMALT=0.0;FREQ_HOMREF=0.9999029973809292;N_BI_GENOS=10309;Yi_AN=422;Yi_AC=1;Yi_AF=0.002369668246445498
chr2	6049945	.	C	<INS:ME:LINE1>	.	PASS	SVTYPE=INS:ME:LINE1;END=6049945;SVLEN=506;AN=20618;AC=1;AF=4.850130953535746e-05;N_HET=1;N_HOMALT=0;N_HOMREF=10308;FREQ_HET=9.700261907071492e-05;FREQ_HOMALT=0.0;FREQ_HOMREF=0.9999029973809292;N_BI_GENOS=10309;East_Han_AN=6554;East_Han_AC=1;East_Han_AF=0.
chr2	6050972	.	A	<INS:ME:LINE1>	.	PASS	SVTYPE=INS:ME:LINE1;END=6050972;SVLEN=296;AN=20618;AC=1;AF=4.850130953535746e-05;N_HET=1;N_HOMALT=0;N_HOMREF=10308;FREQ_HET=9.700261907071492e-05;FREQ_HOMALT=0.0;FREQ_HOMREF=0.9999029973809292;N_BI_GENOS=10309;East_Han_AN=6554;East_Han_AC=1;East_Han_AF=0.
chr2	6452392	.	A	<INS:ME:LINE1>	.	PASS	SVTYPE=INS:ME:LINE1;END=6452403;SVLEN=6001;AN=20618;AC=1;AF=4.850130953535746e-05;N_HET=1;N_HOMALT=0;N_HOMREF=10308;FREQ_HET=9.700261907071492e-05;FREQ_HOMALT=0.0;FREQ_HOMREF=0.9999029973809292;N_BI_GENOS=10309;Southeast_Han_AN=1908;Southeast_Han_AC=1;Sou
chr2	6658145	.	G	<INS:ME:LINE1>	.	PASS	SVTYPE=INS:ME:LINE1;END=6658156;SVLEN=2110;AN=20618;AC=1022;AF=0.04956833834513532;N_HET=942;N_HOMALT=40;N_HOMREF=9327;FREQ_HET=0.09137646716461345;FREQ_HOMALT=0.0038801047628285964;FREQ_HOMREF=0.9047434280725579;N_BI_GENOS=10309;South_Han_AN=1496;South_H
chr2	6910465	.	A	<INS:ME:LINE1>	.	PASS	SVTYPE=INS:ME:LINE1;END=6910472;SVLEN=72;AN=20618;AC=523;AF=0.02536618488699195;N_HET=467;N_HOMALT=28;N_HOMREF=9814;FREQ_HET=0.045300223106023864;FREQ_HOMALT=0.0027160733339800175;FREQ_HOMREF=0.9519837035599962;N_BI_GENOS=10309;South_Han_AN=1496;South_Han
chr2	6997540	.	G	<INS:ME:LINE1>	.	PASS	SVTYPE=INS:ME:LINE1;END=6997554;SVLEN=6005;AN=20618;AC=1;AF=4.850130953535746e-05;N_HET=1;N_HOMALT=0;N_HOMREF=10308;FREQ_HET=9.700261907071492e-05;FREQ_HOMALT=0.0;FREQ_HOMREF=0.9999029973809292;N_BI_GENOS=10309;Northwest_Han_AN=1296;Northwest_Han_AC=1;Nor
chr2	7201633	.	C	<INS:ME:LINE1>	.	PASS	SVTYPE=INS:ME:LINE1;END=7201633;SVLEN=6040;AN=20618;AC=1;AF=4.850130953535746e-05;N_HET=1;N_HOMALT=0;N_HOMREF=10308;FREQ_HET=9.700261907071492e-05;FREQ_HOMALT=0.0;FREQ_HOMREF=0.9999029973809292;N_BI_GENOS=10309;Southeast_Han_AN=1908;Southeast_Han_AC=1;Sou
chr2	7523475	.	A	<INS:ME:LINE1>	.	PASS	SVTYPE=INS:ME:LINE1;END=7523492;SVLEN=5980;AN=20618;AC=1;AF=4.850130953535746e-05;N_HET=1;N_HOMALT=0;N_HOMREF=10308;FREQ_HET=9.700261907071492e-05;FREQ_HOMALT=0.0;FREQ_HOMREF=0.9999029973809292;N_BI_GENOS=10309;North_Han_AN=2272;North_Han_AC=1;North_Han_A
chr2	7827617	.	A	<INS:ME:LINE1>	.	PASS	SVTYPE=INS:ME:LINE1;END=7827633;SVLEN=2278;AN=20618;AC=1;AF=4.850130953535746e-05;N_HET=1;N_HOMALT=0;N_HOMREF=10308;FREQ_HET=9.700261907071492e-05;FREQ_HOMALT=0.0;FREQ_HOMREF=0.9999029973809292;N_BI_GENOS=10309;Southeast_Han_AN=1908;Southeast_Han_AC=1;Sou
chr2	8177554	.	A	<INS:ME:LINE1>	.	PASS	SVTYPE=INS:ME:LINE1;END=8177568;SVLEN=5996;AN=20618;AC=1;AF=4.850130953535746e-05;N_HET=1;N_HOMALT=0;N_HOMREF=10308;FREQ_HET=9.700261907071492e-05;FREQ_HOMALT=0.0;FREQ_HOMREF=0.9999029973809292;N_BI_GENOS=10309;Lingnan_Han_AN=1888;Lingnan_Han_AC=1;Lingnan
chr2	8229144	.	C	<INS:ME:LINE1>	.	PASS	SVTYPE=INS:ME:LINE1;END=8229164;SVLEN=1317;AN=20618;AC=1;AF=4.850130953535746e-05;N_HET=1;N_HOMALT=0;N_HOMREF=10308;FREQ_HET=9.700261907071492e-05;FREQ_HOMALT=0.0;FREQ_HOMREF=0.9999029973809292;N_BI_GENOS=10309;Lingnan_Han_AN=1888;Lingnan_Han_AC=1;Lingnan
chr2	8699180	.	A	<INS:ME:LINE1>	.	PASS	SVTYPE=INS:ME:LINE1;END=8699198;SVLEN=6004;AN=20618;AC=1;AF=4.850130953535746e-05;N_HET=1;N_HOMALT=0;N_HOMREF=10308;FREQ_HET=9.700261907071492e-05;FREQ_HOMALT=0.0;FREQ_HOMREF=0.9999029973809292;N_BI_GENOS=10309;Zhuang_AN=444;Zhuang_AC=1;Zhuang_AF=0.002252

chr2	8722966	.	A	<INS:ME:LINE1>	.	PASS	SVTYPE=INS:ME:LINE1;END=8722975;SVLEN=459;AN=20618;AC=1;AF=4.850130953535746e-05;N_HET=1;N_HOMALT=0;N_HOMREF=10308;FREQ_HET=9.700261907071492e-05;FREQ_HOMALT=0.0;FREQ_HOMREF=0.9999029973809292;N_BI_GENOS=10309;Yi_AN=422;Yi_AC=1;Yi_AF=0.002369668246445498;
chr2	8801041	.	A	<INS:ME:LINE1>	.	PASS	SVTYPE=INS:ME:LINE1;END=8801054;SVLEN=5993;AN=20618;AC=15;AF=0.0007275196430303619;N_HET=15;N_HOMALT=0;N_HOMREF=10294;FREQ_HET=0.0014550392860607237;FREQ_HOMALT=0.0;FREQ_HOMREF=0.9985449607139393;N_BI_GENOS=10309;South_Han_AN=1496;South_Han_AC=3;South_Han
chr2	8863345	.	A	<INS:ME:LINE1>	.	PASS	SVTYPE=INS:ME:LINE1;END=8863358;SVLEN=364;AN=20618;AC=7;AF=0.0003395091667475022;N_HET=7;N_HOMALT=0;N_HOMREF=10302;FREQ_HET=0.0006790183334950044;FREQ_HOMALT=0.0;FREQ_HOMREF=0.999320981666505;N_BI_GENOS=10309;Lingnan_Han_AN=1888;Lingnan_Han_AC=1;Lingnan_H
chr2	8967036	.	A	<INS:ME:LINE1>	.	PASS	SVTYPE=INS:ME:LINE1;END=8967036;SVLEN=6027;AN=20618;AC=1;AF=4.850130953535746e-05;N_HET=1;N_HOMALT=0;N_HOMREF=10308;FREQ_HET=9.700261907071492e-05;FREQ_HOMALT=0.0;FREQ_HOMREF=0.9999029973809292;N_BI_GENOS=10309;East_Han_AN=6554;East_Han_AC=1;East_Han_AF=0
chr2	9792966	.	A	<INS:ME:LINE1>	.	PASS	SVTYPE=INS:ME:LINE1;END=9792984;SVLEN=5998;AN=20618;AC=17;AF=0.0008245222621010767;N_HET=15;N_HOMALT=1;N_HOMREF=10293;FREQ_HET=0.0014550392860607237;FREQ_HOMALT=9.700261907071492e-05;FREQ_HOMREF=0.9984479580948685;N_BI_GENOS=10309;South_Han_AN=1496;South_
chr2	10806477	.	T	<INS:ME:LINE1>	.	PASS	SVTYPE=INS:ME:LINE1;END=10806493;SVLEN=5433;AN=20618;AC=12;AF=0.0005820157144242894;N_HET=2;N_HOMALT=5;N_HOMREF=10302;FREQ_HET=0.00019400523814142983;FREQ_HOMALT=0.00048501309535357455;FREQ_HOMREF=0.999320981666505;N_BI_GENOS=10309;South_Han_AN=1496;South
chr2	10806628	.	A	<INS:ME:LINE1>	.	PASS	SVTYPE=INS:ME:LINE1;END=10806649;SVLEN=15;AN=20618;AC=66;AF=0.003201086429333592;N_HET=4;N_HOMALT=31;N_HOMREF=10274;FREQ_HET=0.00038801047628285966;FREQ_HOMALT=0.0030070811911921624;FREQ_HOMREF=0.996604908332525;N_BI_GENOS=10309;South_Han_AN=1496;South_Ha
chr2	11035758	.	A	<INS:ME:LINE1>	.	PASS	SVTYPE=INS:ME:LINE1;END=11035758;SVLEN=118;AN=20618;AC=1;AF=4.850130953535746e-05;N_HET=1;N_HOMALT=0;N_HOMREF=10308;FREQ_HET=9.700261907071492e-05;FREQ_HOMALT=0.0;FREQ_HOMREF=0.9999029973809292;N_BI_GENOS=10309;East_Han_AN=6554;East_Han_AC=1;East_Han_AF=0
chr2	11381050	.	G	<INS:ME:LINE1>	.	PASS	SVTYPE=INS:ME:LINE1;END=11381063;SVLEN=6008;AN=20618;AC=1;AF=4.850130953535746e-05;N_HET=1;N_HOMALT=0;N_HOMREF=10308;FREQ_HET=9.700261907071492e-05;FREQ_HOMALT=0.0;FREQ_HOMREF=0.9999029973809292;N_BI_GENOS=10309;Central_Han_AN=2128;Central_Han_AC=1;Centra
chr2	11789899	.	C	<INS:ME:LINE1>	.	PASS	SVTYPE=INS:ME:LINE1;END=11789925;SVLEN=685;AN=20618;AC=1;AF=4.850130953535746e-05;N_HET=1;N_HOMALT=0;N_HOMREF=10308;FREQ_HET=9.700261907071492e-05;FREQ_HOMALT=0.0;FREQ_HOMREF=0.9999029973809292;N_BI_GENOS=10309;East_Han_AN=6554;East_Han_AC=1;East_Han_AF=0
chr2	12025765	.	A	<INS:ME:LINE1>	.	PASS	SVTYPE=INS:ME:LINE1;END=12025776;SVLEN=2067;AN=20618;AC=2;AF=9.700261907071492e-05;N_HET=2;N_HOMALT=0;N_HOMREF=10307;FREQ_HET=0.00019400523814142983;FREQ_HOMALT=0.0;FREQ_HOMREF=0.9998059947618586;N_BI_GENOS=10309;East_Han_AN=6554;East_Han_AC=2;East_Han_AF
chr2	12104493	.	A	<INS:ME:LINE1>	.	PASS	SVTYPE=INS:ME:LINE1;END=12104504;SVLEN=6004;AN=20618;AC=1;AF=4.850130953535746e-05;N_HET=1;N_HOMALT=0;N_HOMREF=10308;FREQ_HET=9.700261907071492e-05;FREQ_HOMALT=0.0;FREQ_HOMREF=0.9999029973809292;N_BI_GENOS=10309;South_Han_AN=1496;South_Han_AC=1;South_Han_
chr2	12172700	.	T	<INS:ME:LINE1>	.	PASS	SVTYPE=INS:ME:LINE1;END=12172712;SVLEN=335;AN=20618;AC=1;AF=4.850130953535746e-05;N_HET=1;N_HOMALT=0;N_HOMREF=10308;FREQ_HET=9.700261907071492e-05;FREQ_HOMALT=0.0;FREQ_HOMREF=0.9999029973809292;N_BI_GENOS=10309;Central_Han_AN=2128;Central_Han_AC=1;Central
chr2	12236416	.	A	<INS:ME:LINE1>	.	PASS	SVTYPE=INS:ME:LINE1;END=12236434;SVLEN=1005;AN=20618;AC=1;AF=4.850130953535746e-05;N_HET=1;N_HOMALT=0;N_HOMREF=10308;FREQ_HET=9.700261907071492e-05;FREQ_HOMALT=0.0;FREQ_HOMREF=0.9999029973809292;N_BI_GENOS=10309;Central_Han_AN=2128;Central_Han_AC=1;Centra
chr2	12315371	.	A	<INS:ME:LINE1>	.	PASS	SVTYPE=INS:ME:LINE1;END=12315387;SVLEN=5993;AN=20618;AC=1;AF=4.850130953535746e-05;N_HET=1;N_HOMALT=0;N_HOMREF=10308;FREQ_HET=9.700261907071492e-05;FREQ_HOMALT=0.0;FREQ_HOMREF=0.9999029973809292;N_BI_GENOS=10309;Central_Han_AN=2128;Central_Han_AC=1;Centra
chr2	12350270	.	T	<INS:ME:LINE1>	.	PASS	SVTYPE=INS:ME:LINE1;END=12350284;SVLEN=1143;AN=20618;AC=6;AF=0.0002910078572121447;N_HET=6;N_HOMALT=0;N_HOMREF=10303;FREQ_HET=0.0005820157144242894;FREQ_HOMALT=0.0;FREQ_HOMREF=0.9994179842855757;N_BI_GENOS=10309;North_Han_AN=2272;North_Han_AC=1;North_Han_
chr2	12791859	.	T	<INS:ME:LINE1>	.	PASS	SVTYPE=INS:ME:LINE1;END=12791859;SVLEN=1101;AN=20618;AC=1;AF=4.850130953535746e-05;N_HET=1;N_HOMALT=0;N_HOMREF=10308;FREQ_HET=9.700261907071492e-05;FREQ_HOMALT=0.0;FREQ_HOMREF=0.9999029973809292;N_BI_GENOS=10309;Northwest_Han_AN=1296;Northwest_Han_AC=1;No
chr2	12884610	.	A	<INS:ME:LINE1>	.	PASS	SVTYPE=INS:ME:LINE1;END=12884623;SVLEN=6021;AN=20618;AC=1;AF=4.850130953535746e-05;N_HET=1;N_HOMALT=0;N_HOMREF=10308;FREQ_HET=9.700261907071492e-05;FREQ_HOMALT=0.0;FREQ_HOMREF=0.9999029973809292;N_BI_GENOS=10309;North_Han_AN=2272;North_Han_AC=1;North_Han_
chr2	13043072	.	A	<INS:ME:LINE1>	.	PASS	SVTYPE=INS:ME:LINE1;END=13043073;SVLEN=2360;AN=20618;AC=9;AF=0.0004365117858182171;N_HET=1;N_HOMALT=4;N_HOMREF=10304;FREQ_HET=9.700261907071492e-05;FREQ_HOMALT=0.00038801047628285966;FREQ_HOMREF=0.9995149869046465;N_BI_GENOS=10309;Northwest_Han_AN=1296;No
chr2	13064339	.	T	<INS:ME:LINE1>	.	PASS	SVTYPE=INS:ME:LINE1;END=13064352;SVLEN=108;AN=20618;AC=1;AF=4.850130953535746e-05;N_HET=1;N_HOMALT=0;N_HOMREF=10308;FREQ_HET=9.700261907071492e-05;FREQ_HOMALT=0.0;FREQ_HOMREF=0.9999029973809292;N_BI_GENOS=10309;Northwest_Han_AN=1296;Northwest_Han_AC=1;Nor
chr2	13167821	.	A	<INS:ME:LINE1>	.	PASS	SVTYPE=INS:ME:LINE1;END=13167821;SVLEN=0;AN=20618;AC=1;AF=4.850130953535746e-05;N_HET=1;N_HOMALT=0;N_HOMREF=10308;FREQ_HET=9.700261907071492e-05;FREQ_HOMALT=0.0;FREQ_HOMREF=0.9999029973809292;N_BI_GENOS=10309;North_Han_AN=2272;North_Han_AC=1;North_Han_AF=
chr2	13168634	.	A	<INS:ME:LINE1>	.	PASS	SVTYPE=INS:ME:LINE1;END=13168634;SVLEN=0;AN=20618;AC=1;AF=4.850130953535746e-05;N_HET=1;N_HOMALT=0;N_HOMREF=10308;FREQ_HET=9.700261907071492e-05;FREQ_HOMALT=0.0;FREQ_HOMREF=0.9999029973809292;N_BI_GENOS=10309;Southeast_Han_AN=1908;Southeast_Han_AC=1;South
chr2	13235590	.	G	<INS:ME:LINE1>	.	PASS	SVTYPE=INS:ME:LINE1;END=13235592;SVLEN=860;AN=20618;AC=15;AF=0.0007275196430303619;N_HET=15;N_HOMALT=0;N_HOMREF=10294;FREQ_HET=0.0014550392860607237;FREQ_HOMALT=0.0;FREQ_HOMREF=0.9985449607139393;N_BI_GENOS=10309;South_Han_AN=1496;South_Han_AC=1;South_Han
chr2	13360094	.	T	<INS:ME:LINE1>	.	PASS	SVTYPE=INS:ME:LINE1;END=13360098;SVLEN=694;AN=20618;AC=1;AF=4.850130953535746e-05;N_HET=1;N_HOMALT=0;N_HOMREF=10308;FREQ_HET=9.700261907071492e-05;FREQ_HOMALT=0.0;FREQ_HOMREF=0.9999029973809292;N_BI_GENOS=10309;North_Han_AN=2272;North_Han_AC=1;North_Han_A
chr2	13479311	.	A	<INS:ME:LINE1>	.	PASS	SVTYPE=INS:ME:LINE1;END=13479326;SVLEN=1556;AN=20618;AC=1;AF=4.850130953535746e-05;N_HET=1;N_HOMALT=0;N_HOMREF=10308;FREQ_HET=9.700261907071492e-05;FREQ_HOMALT=0.0;FREQ_HOMREF=0.9999029973809292;N_BI_GENOS=10309;Northwest_Han_AN=1296;Northwest_Han_AC=1;No
chr2	13480850	.	T	<INS:ME:LINE1>	.	PASS	SVTYPE=INS:ME:LINE1;END=13480867;SVLEN=432;AN=20618;AC=1;AF=4.850130953535746e-05;N_HET=1;N_HOMALT=0;N_HOMREF=10308;FREQ_HET=9.700261907071492e-05;FREQ_HOMALT=0.0;FREQ_HOMREF=0.9999029973809292;N_BI_GENOS=10309;Central_Han_AN=2128;Central_Han_AC=1;Central
chr2	13489043	.	T	<INS:ME:LINE1>	.	PASS	SVTYPE=INS:ME:LINE1;END=13489047;SVLEN=1027;AN=20618;AC=2;AF=9.700261907071492e-05;N_HET=2;N_HOMALT=0;N_HOMREF=10307;FREQ_HET=0.00019400523814142983;FREQ_HOMALT=0.0;FREQ_HOMREF=0.9998059947618586;N_BI_GENOS=10309;East_Han_AN=6554;East_Han_AC=2;East_Han_AF
chr2	13630220	.	A	<INS:ME:LINE1>	.	PASS	SVTYPE=INS:ME:LINE1;END=13630229;SVLEN=565;AN=20618;AC=1;AF=4.850130953535746e-05;N_HET=1;N_HOMALT=0;N_HOMREF=10308;FREQ_HET=9.700261907071492e-05;FREQ_HOMALT=0.0;FREQ_HOMREF=0.9999029973809292;N_BI_GENOS=10309;East_Han_AN=6554;East_Han_AC=1;East_Han_AF=0
chr2	13650383	.	A	<INS:ME:LINE1>	.	PASS	SVTYPE=INS:ME:LINE1;END=13650398;SVLEN=595;AN=20618;AC=1;AF=4.850130953535746e-05;N_HET=1;N_HOMALT=0;N_HOMREF=10308;FREQ_HET=9.700261907071492e-05;FREQ_HOMALT=0.0;FREQ_HOMREF=0.9999029973809292;N_BI_GENOS=10309;Lingnan_Han_AN=1888;Lingnan_Han_AC=1;Lingnan
chr2	13658462	.	G	<INS:ME:LINE1>	.	PASS	SVTYPE=INS:ME:LINE1;END=13658462;SVLEN=32;AN=20618;AC=1;AF=4.850130953535746e-05;N_HET=1;N_HOMALT=0;N_HOMREF=10308;FREQ_HET=9.700261907071492e-05;FREQ_HOMALT=0.0;FREQ_HOMREF=0.9999029973809292;N_BI_GENOS=10309;East_Han_AN=6554;East_Han_AC=1;East_Han_AF=0.
chr2	13668432	.	A	<INS:ME:LINE1>	.	PASS	SVTYPE=INS:ME:LINE1;END=13668448;SVLEN=2172;AN=20618;AC=1;AF=4.850130953535746e-05;N_HET=1;N_HOMALT=0;N_HOMREF=10308;FREQ_HET=9.700261907071492e-05;FREQ_HOMALT=0.0;FREQ_HOMREF=0.9999029973809292;N_BI_GENOS=10309;Southeast_Han_AN=1908;Southeast_Han_AC=1;So
chr2	13694567	.	A	<INS:ME:LINE1>	.	PASS	SVTYPE=INS:ME:LINE1;END=13694567;SVLEN=1879;AN=20618;AC=2;AF=9.700261907071492e-05;N_HET=2;N_HOMALT=0;N_HOMREF=10307;FREQ_HET=0.00019400523814142983;FREQ_HOMALT=0.0;FREQ_HOMREF=0.9998059947618586;N_BI_GENOS=10309;East_Han_AN=6554;East_Han_AC=1;East_Han_AF
chr2	13755957	.	G	<INS:ME:LINE1>	.	PASS	SVTYPE=INS:ME:LINE1;END=13755967;SVLEN=577;AN=20618;AC=418;AF=0.020273547385779415;N_HET=370;N_HOMALT=24;N_HOMREF=9915;FREQ_HET=0.03589096905616452;FREQ_HOMALT=0.0023280628576971578;FREQ_HOMREF=0.9617809680861383;N_BI_GENOS=10309;South_Han_AN=1496;South_H
chr2	13782128	.	A	<INS:ME:LINE1>	.	PASS	SVTYPE=INS:ME:LINE1;END=13782145;SVLEN=1700;AN=20618;AC=10;AF=0.00048501309535357455;N_HET=10;N_HOMALT=0;N_HOMREF=10299;FREQ_HET=0.0009700261907071491;FREQ_HOMALT=0.0;FREQ_HOMREF=0.9990299738092928;N_BI_GENOS=10309;South_Han_AN=1496;South_Han_AC=1;South_H
chr2	13847117	.	T	<INS:ME:LINE1>	.	PASS	SVTYPE=INS:ME:LINE1;END=13847131;SVLEN=962;AN=20618;AC=1;AF=4.850130953535746e-05;N_HET=1;N_HOMALT=0;N_HOMREF=10308;FREQ_HET=9.700261907071492e-05;FREQ_HOMALT=0.0;FREQ_HOMREF=0.9999029973809292;N_BI_GENOS=10309;East_Han_AN=6554;East_Han_AC=1;East_Han_AF=0
chr2	13852081	.	T	<INS:ME:LINE1>	.	PASS	SVTYPE=INS:ME:LINE1;END=13852095;SVLEN=1537;AN=20618;AC=6;AF=0.0002910078572121447;N_HET=6;N_HOMALT=0;N_HOMREF=10303;FREQ_HET=0.0005820157144242894;FREQ_HOMALT=0.0;FREQ_HOMREF=0.9994179842855757;N_BI_GENOS=10309;South_Han_AN=1496;South_Han_AC=1;South_Han_
chr2	13894616	.	C	<INS:ME:LINE1>	.	PASS	SVTYPE=INS:ME:LINE1;END=13894634;SVLEN=438;AN=20618;AC=1;AF=4.850130953535746e-05;N_HET=1;N_HOMALT=0;N_HOMREF=10308;FREQ_HET=9.700261907071492e-05;FREQ_HOMALT=0.0;FREQ_HOMREF=0.9999029973809292;N_BI_GENOS=10309;North_Han_AN=2272;North_Han_AC=1;North_Han_A
chr2	13962763	.	C	<INS:ME:LINE1>	.	PASS	SVTYPE=INS:ME:LINE1;END=13962764;SVLEN=943;AN=20618;AC=1;AF=4.850130953535746e-05;N_HET=1;N_HOMALT=0;N_HOMREF=10308;FREQ_HET=9.700261907071492e-05;FREQ_HOMALT=0.0;FREQ_HOMREF=0.9999029973809292;N_BI_GENOS=10309;East_Han_AN=6554;East_Han_AC=1;East_Han_AF=0
chr2	14120337	.	A	<INS:ME:LINE1>	.	PASS	SVTYPE=INS:ME:LINE1;END=14120351;SVLEN=409;AN=20618;AC=1;AF=4.850130953535746e-05;N_HET=1;N_HOMALT=0;N_HOMREF=10308;FREQ_HET=9.700261907071492e-05;FREQ_HOMALT=0.0;FREQ_HOMREF=0.9999029973809292;N_BI_GENOS=10309;South_Han_AN=1496;South_Han_AC=1;South_Han_A
chr2	14146900	.	A	<INS:ME:LINE1>	.	PASS	SVTYPE=INS:ME:LINE1;END=14146913;SVLEN=1758;AN=20618;AC=1;AF=4.850130953535746e-05;N_HET=1;N_HOMALT=0;N_HOMREF=10308;FREQ_HET=9.700261907071492e-05;FREQ_HOMALT=0.0;FREQ_HOMREF=0.9999029973809292;N_BI_GENOS=10309;Mongolian_AN=454;Mongolian_AC=1;Mongolian_A
chr2	14425683	.	G	<INS:ME:LINE1>	.	PASS	SVTYPE=INS:ME:LINE1;END=14425699;SVLEN=2307;AN=20618;AC=8;AF=0.00038801047628285966;N_HET=8;N_HOMALT=0;N_HOMREF=10301;FREQ_HET=0.0007760209525657193;FREQ_HOMALT=0.0;FREQ_HOMREF=0.9992239790474343;N_BI_GENOS=10309;Northwest_Han_AN=1296;Northwest_Han_AC=2;N
chr2	14512124	.	C	<INS:ME:LINE1>	.	PASS	SVTYPE=INS:ME:LINE1;END=14512139;SVLEN=1856;AN=20618;AC=2;AF=9.700261907071492e-05;N_HET=2;N_HOMALT=0;N_HOMREF=10307;FREQ_HET=0.00019400523814142983;FREQ_HOMALT=0.0;FREQ_HOMREF=0.9998059947618586;N_BI_GENOS=10309;South_Han_AN=1496;South_Han_AC=2;South_Han
chr2	14605014	.	C	<INS:ME:LINE1>	.	PASS	SVTYPE=INS:ME:LINE1;END=14605031;SVLEN=1588;AN=20618;AC=1;AF=4.850130953535746e-05;N_HET=1;N_HOMALT=0;N_HOMREF=10308;FREQ_HET=9.700261907071492e-05;FREQ_HOMALT=0.0;FREQ_HOMREF=0.9999029973809292;N_BI_GENOS=10309;Central_Han_AN=2128;Central_Han_AC=1;Centra
chr2	14670306	.	G	<INS:ME:LINE1>	.	PASS	SVTYPE=INS:ME:LINE1;END=14670318;SVLEN=1288;AN=20618;AC=1;AF=4.850130953535746e-05;N_HET=1;N_HOMALT=0;N_HOMREF=10308;FREQ_HET=9.700261907071492e-05;FREQ_HOMALT=0.0;FREQ_HOMREF=0.9999029973809292;N_BI_GENOS=10309;Manchu_AN=416;Manchu_AC=1;Manchu_AF=0.00240
chr2	14706952	.	C	<INS:ME:LINE1>	.	PASS	SVTYPE=INS:ME:LINE1;END=14706952;SVLEN=959;AN=20618;AC=3;AF=0.00014550392860607236;N_HET=3;N_HOMALT=0;N_HOMREF=10306;FREQ_HET=0.0002910078572121447;FREQ_HOMALT=0.0;FREQ_HOMREF=0.9997089921427879;N_BI_GENOS=10309;South_Han_AN=1496;South_Han_AC=1;South_Han_
chr2	14768681	.	G	<INS:ME:LINE1>	.	PASS	SVTYPE=INS:ME:LINE1;END=14768697;SVLEN=1528;AN=20618;AC=3;AF=0.00014550392860607236;N_HET=3;N_HOMALT=0;N_HOMREF=10306;FREQ_HET=0.0002910078572121447;FREQ_HOMALT=0.0;FREQ_HOMREF=0.9997089921427879;N_BI_GENOS=10309;South_Han_AN=1496;South_Han_AC=2;South_Han
chr2	14889373	.	A	<INS:ME:LINE1>	.	PASS	SVTYPE=INS:ME:LINE1;END=14889389;SVLEN=941;AN=20618;AC=3;AF=0.00014550392860607236;N_HET=3;N_HOMALT=0;N_HOMREF=10306;FREQ_HET=0.0002910078572121447;FREQ_HOMALT=0.0;FREQ_HOMREF=0.9997089921427879;N_BI_GENOS=10309;East_Han_AN=6554;East_Han_AC=3;East_Han_AF=
chr2	14923584	.	A	<INS:ME:LINE1>	.	PASS	SVTYPE=INS:ME:LINE1;END=14923602;SVLEN=6000;AN=20618;AC=15;AF=0.0007275196430303619;N_HET=15;N_HOMALT=0;N_HOMREF=10294;FREQ_HET=0.0014550392860607237;FREQ_HOMALT=0.0;FREQ_HOMREF=0.9985449607139393;N_BI_GENOS=10309;South_Han_AN=1496;South_Han_AC=1;South_Ha
chr2	15305875	.	A	<INS:ME:LINE1>	.	PASS	SVTYPE=INS:ME:LINE1;END=15305888;SVLEN=3121;AN=20618;AC=1;AF=4.850130953535746e-05;N_HET=1;N_HOMALT=0;N_HOMREF=10308;FREQ_HET=9.700261907071492e-05;FREQ_HOMALT=0.0;FREQ_HOMREF=0.9999029973809292;N_BI_GENOS=10309;East_Han_AN=6554;East_Han_AC=1;East_Han_AF=
chr2	15475904	.	A	<INS:ME:LINE1>	.	PASS	SVTYPE=INS:ME:LINE1;END=15475904;SVLEN=15;AN=20618;AC=1;AF=4.850130953535746e-05;N_HET=1;N_HOMALT=0;N_HOMREF=10308;FREQ_HET=9.700261907071492e-05;FREQ_HOMALT=0.0;FREQ_HOMREF=0.9999029973809292;N_BI_GENOS=10309;East_Han_AN=6554;East_Han_AC=1;East_Han_AF=0.
chr2	16374674	.	A	<INS:ME:LINE1>	.	PASS	SVTYPE=INS:ME:LINE1;END=16374691;SVLEN=1984;AN=20618;AC=1;AF=4.850130953535746e-05;N_HET=1;N_HOMALT=0;N_HOMREF=10308;FREQ_HET=9.700261907071492e-05;FREQ_HOMALT=0.0;FREQ_HOMREF=0.9999029973809292;N_BI_GENOS=10309;East_Han_AN=6554;East_Han_AC=1;East_Han_AF=
chr2	16484777	.	G	<INS:ME:LINE1>	.	PASS	SVTYPE=INS:ME:LINE1;END=16484791;SVLEN=5995;AN=20618;AC=1;AF=4.850130953535746e-05;N_HET=1;N_HOMALT=0;N_HOMREF=10308;FREQ_HET=9.700261907071492e-05;FREQ_HOMALT=0.0;FREQ_HOMREF=0.9999029973809292;N_BI_GENOS=10309;Zhuang_AN=444;Zhuang_AC=1;Zhuang_AF=0.00225
chr2	16493896	.	A	<INS:ME:LINE1>	.	PASS	SVTYPE=INS:ME:LINE1;END=16493915;SVLEN=2321;AN=20618;AC=6;AF=0.0002910078572121447;N_HET=6;N_HOMALT=0;N_HOMREF=10303;FREQ_HET=0.0005820157144242894;FREQ_HOMALT=0.0;FREQ_HOMREF=0.9994179842855757;N_BI_GENOS=10309;Lingnan_Han_AN=1888;Lingnan_Han_AC=1;Lingna
chr2	17018422	.	G	<INS:ME:LINE1>	.	PASS	SVTYPE=INS:ME:LINE1;END=17018437;SVLEN=3471;AN=20618;AC=1;AF=4.850130953535746e-05;N_HET=1;N_HOMALT=0;N_HOMREF=10308;FREQ_HET=9.700261907071492e-05;FREQ_HOMALT=0.0;FREQ_HOMREF=0.9999029973809292;N_BI_GENOS=10309;East_Han_AN=6554;East_Han_AC=1;East_Han_AF=
chr2	17042149	.	G	<INS:ME:LINE1>	.	PASS	SVTYPE=INS:ME:LINE1;END=17042164;SVLEN=1259;AN=20618;AC=1;AF=4.850130953535746e-05;N_HET=1;N_HOMALT=0;N_HOMREF=10308;FREQ_HET=9.700261907071492e-05;FREQ_HOMALT=0.0;FREQ_HOMREF=0.9999029973809292;N_BI_GENOS=10309;South_Han_AN=1496;South_Han_AC=1;South_Han_
chr2	17473233	.	T	<INS:ME:LINE1>	.	PASS	SVTYPE=INS:ME:LINE1;END=17473250;SVLEN=6000;AN=20618;AC=2;AF=9.700261907071492e-05;N_HET=2;N_HOMALT=0;N_HOMREF=10307;FREQ_HET=0.00019400523814142983;FREQ_HOMALT=0.0;FREQ_HOMREF=0.9998059947618586;N_BI_GENOS=10309;Lingnan_Han_AN=1888;Lingnan_Han_AC=1;Lingn
chr2	17728672	.	A	<INS:ME:LINE1>	.	PASS	SVTYPE=INS:ME:LINE1;END=17728684;SVLEN=5994;AN=20618;AC=1;AF=4.850130953535746e-05;N_HET=1;N_HOMALT=0;N_HOMREF=10308;FREQ_HET=9.700261907071492e-05;FREQ_HOMALT=0.0;FREQ_HOMREF=0.9999029973809292;N_BI_GENOS=10309;Central_Han_AN=2128;Central_Han_AC=1;Centra
chr2	18170138	.	A	<INS:ME:LINE1>	.	PASS	SVTYPE=INS:ME:LINE1;END=18170154;SVLEN=557;AN=20618;AC=1;AF=4.850130953535746e-05;N_HET=1;N_HOMALT=0;N_HOMREF=10308;FREQ_HET=9.700261907071492e-05;FREQ_HOMALT=0.0;FREQ_HOMREF=0.9999029973809292;N_BI_GENOS=10309;East_Han_AN=6554;East_Han_AC=1;East_Han_AF=0
chr2	18229250	.	A	<INS:ME:LINE1>	.	PASS	SVTYPE=INS:ME:LINE1;END=18229250;SVLEN=1047;AN=20618;AC=1;AF=4.850130953535746e-05;N_HET=1;N_HOMALT=0;N_HOMREF=10308;FREQ_HET=9.700261907071492e-05;FREQ_HOMALT=0.0;FREQ_HOMREF=0.9999029973809292;N_BI_GENOS=10309;East_Han_AN=6554;East_Han_AC=1;East_Han_AF=
chr2	18231361	.	A	<INS:ME:LINE1>	.	PASS	SVTYPE=INS:ME:LINE1;END=18231376;SVLEN=6000;AN=20618;AC=1;AF=4.850130953535746e-05;N_HET=1;N_HOMALT=0;N_HOMREF=10308;FREQ_HET=9.700261907071492e-05;FREQ_HOMALT=0.0;FREQ_HOMREF=0.9999029973809292;N_BI_GENOS=10309;Lingnan_Han_AN=1888;Lingnan_Han_AC=1;Lingna
chr2	18445961	.	A	<INS:ME:LINE1>	.	PASS	SVTYPE=INS:ME:LINE1;END=18445977;SVLEN=1506;AN=20618;AC=1;AF=4.850130953535746e-05;N_HET=1;N_HOMALT=0;N_HOMREF=10308;FREQ_HET=9.700261907071492e-05;FREQ_HOMALT=0.0;FREQ_HOMREF=0.9999029973809292;N_BI_GENOS=10309;Northwest_Han_AN=1296;Northwest_Han_AC=1;No
chr2	18574974	.	C	<INS:ME:LINE1>	.	PASS	SVTYPE=INS:ME:LINE1;END=18574974;SVLEN=101;AN=20618;AC=1;AF=4.850130953535746e-05;N_HET=1;N_HOMALT=0;N_HOMREF=10308;FREQ_HET=9.700261907071492e-05;FREQ_HOMALT=0.0;FREQ_HOMREF=0.9999029973809292;N_BI_GENOS=10309;North_Han_AN=2272;North_Han_AC=1;North_Han_A
chr2	18633885	.	C	<INS:ME:LINE1>	.	PASS	SVTYPE=INS:ME:LINE1;END=18633885;SVLEN=151;AN=20618;AC=1;AF=4.850130953535746e-05;N_HET=1;N_HOMALT=0;N_HOMREF=10308;FREQ_HET=9.700261907071492e-05;FREQ_HOMALT=0.0;FREQ_HOMREF=0.9999029973809292;N_BI_GENOS=10309;East_Han_AN=6554;East_Han_AC=1;East_Han_AF=0
chr2	18699870	.	A	<INS:ME:LINE1>	.	PASS	SVTYPE=INS:ME:LINE1;END=18699885;SVLEN=1381;AN=20618;AC=1;AF=4.850130953535746e-05;N_HET=1;N_HOMALT=0;N_HOMREF=10308;FREQ_HET=9.700261907071492e-05;FREQ_HOMALT=0.0;FREQ_HOMREF=0.9999029973809292;N_BI_GENOS=10309;East_Han_AN=6554;East_Han_AC=1;East_Han_AF=
chr2	18783725	.	T	<INS:ME:LINE1>	.	PASS	SVTYPE=INS:ME:LINE1;END=18783741;SVLEN=391;AN=20618;AC=1;AF=4.850130953535746e-05;N_HET=1;N_HOMALT=0;N_HOMREF=10308;FREQ_HET=9.700261907071492e-05;FREQ_HOMALT=0.0;FREQ_HOMREF=0.9999029973809292;N_BI_GENOS=10309;South_Han_AN=1496;South_Han_AC=1;South_Han_A
chr2	19189355	.	G	<INS:ME:LINE1>	.	PASS	SVTYPE=INS:ME:LINE1;END=19189401;SVLEN=5997;AN=20618;AC=1;AF=4.850130953535746e-05;N_HET=1;N_HOMALT=0;N_HOMREF=10308;FREQ_HET=9.700261907071492e-05;FREQ_HOMALT=0.0;FREQ_HOMREF=0.9999029973809292;N_BI_GENOS=10309;East_Han_AN=6554;East_Han_AC=1;East_Han_AF=
chr2	19287840	.	T	<INS:ME:LINE1>	.	PASS	SVTYPE=INS:ME:LINE1;END=19287842;SVLEN=2898;AN=20618;AC=2;AF=9.700261907071492e-05;N_HET=2;N_HOMALT=0;N_HOMREF=10307;FREQ_HET=0.00019400523814142983;FREQ_HOMALT=0.0;FREQ_HOMREF=0.9998059947618586;N_BI_GENOS=10309;Lingnan_Han_AN=1888;Lingnan_Han_AC=1;Lingn
chr2	19429270	.	G	<INS:ME:LINE1>	.	PASS	SVTYPE=INS:ME:LINE1;END=19429285;SVLEN=2460;AN=20618;AC=2;AF=9.700261907071492e-05;N_HET=2;N_HOMALT=0;N_HOMREF=10307;FREQ_HET=0.00019400523814142983;FREQ_HOMALT=0.0;FREQ_HOMREF=0.9998059947618586;N_BI_GENOS=10309;Southeast_Han_AN=1908;Southeast_Han_AC=2;S
chr2	19472540	.	T	<INS:ME:LINE1>	.	PASS	SVTYPE=INS:ME:LINE1;END=19472541;SVLEN=1050;AN=20618;AC=1;AF=4.850130953535746e-05;N_HET=1;N_HOMALT=0;N_HOMREF=10308;FREQ_HET=9.700261907071492e-05;FREQ_HOMALT=0.0;FREQ_HOMREF=0.9999029973809292;N_BI_GENOS=10309;Southeast_Han_AN=1908;Southeast_Han_AC=1;So
chr2	19818804	.	C	<INS:ME:LINE1>	.	PASS	SVTYPE=INS:ME:LINE1;END=19818819;SVLEN=5998;AN=20618;AC=1;AF=4.850130953535746e-05;N_HET=1;N_HOMALT=0;N_HOMREF=10308;FREQ_HET=9.700261907071492e-05;FREQ_HOMALT=0.0;FREQ_HOMREF=0.9999029973809292;N_BI_GENOS=10309;North_Han_AN=2272;North_Han_AC=1;North_Han_
chr2	19877081	.	A	<INS:ME:LINE1>	.	PASS	SVTYPE=INS:ME:LINE1;END=19877098;SVLEN=495;AN=20618;AC=19;AF=0.0009215248811717916;N_HET=15;N_HOMALT=2;N_HOMREF=10292;FREQ_HET=0.0014550392860607237;FREQ_HOMALT=0.00019400523814142983;FREQ_HOMREF=0.9983509554757979;N_BI_GENOS=10309;South_Han_AN=1496;South
chr2	19969730	.	A	<INS:ME:LINE1>	.	PASS	SVTYPE=INS:ME:LINE1;END=19969731;SVLEN=523;AN=20618;AC=1;AF=4.850130953535746e-05;N_HET=1;N_HOMALT=0;N_HOMREF=10308;FREQ_HET=9.700261907071492e-05;FREQ_HOMALT=0.0;FREQ_HOMREF=0.9999029973809292;N_BI_GENOS=10309;East_Han_AN=6554;East_Han_AC=1;East_Han_AF=0
chr2	20400345	.	A	<INS:ME:LINE1>	.	PASS	SVTYPE=INS:ME:LINE1;END=20400358;SVLEN=1546;AN=20618;AC=1;AF=4.850130953535746e-05;N_HET=1;N_HOMALT=0;N_HOMREF=10308;FREQ_HET=9.700261907071492e-05;FREQ_HOMALT=0.0;FREQ_HOMREF=0.9999029973809292;N_BI_GENOS=10309;East_Han_AN=6554;East_Han_AC=1;East_Han_AF=
chr2	20455210	.	C	<INS:ME:LINE1>	.	PASS	SVTYPE=INS:ME:LINE1;END=20455224;SVLEN=5998;AN=20618;AC=1;AF=4.850130953535746e-05;N_HET=1;N_HOMALT=0;N_HOMREF=10308;FREQ_HET=9.700261907071492e-05;FREQ_HOMALT=0.0;FREQ_HOMREF=0.9999029973809292;N_BI_GENOS=10309;Southeast_Han_AN=1908;Southeast_Han_AC=1;So
chr2	20501360	.	T	<INS:ME:LINE1>	.	PASS	SVTYPE=INS:ME:LINE1;END=20501360;SVLEN=0;AN=20618;AC=16;AF=0.0007760209525657193;N_HET=16;N_HOMALT=0;N_HOMREF=10293;FREQ_HET=0.0015520419051314386;FREQ_HOMALT=0.0;FREQ_HOMREF=0.9984479580948685;N_BI_GENOS=10309;Northwest_Han_AN=1296;Northwest_Han_AC=1;Nor
chr2	20501536	.	G	<INS:ME:LINE1>	.	PASS	SVTYPE=INS:ME:LINE1;END=20501536;SVLEN=0;AN=20618;AC=4;AF=0.00019400523814142983;N_HET=4;N_HOMALT=0;N_HOMREF=10305;FREQ_HET=0.00038801047628285966;FREQ_HOMALT=0.0;FREQ_HOMREF=0.9996119895237171;N_BI_GENOS=10309;North_Han_AN=2272;North_Han_AC=1;North_Han_A
chr2	20676804	.	G	<INS:ME:LINE1>	.	PASS	SVTYPE=INS:ME:LINE1;END=20676818;SVLEN=328;AN=20618;AC=1;AF=4.850130953535746e-05;N_HET=1;N_HOMALT=0;N_HOMREF=10308;FREQ_HET=9.700261907071492e-05;FREQ_HOMALT=0.0;FREQ_HOMREF=0.9999029973809292;N_BI_GENOS=10309;Southeast_Han_AN=1908;Southeast_Han_AC=1;Sou
chr2	20862764	.	G	<INS:ME:LINE1>	.	PASS	SVTYPE=INS:ME:LINE1;END=20862780;SVLEN=3143;AN=20618;AC=1;AF=4.850130953535746e-05;N_HET=1;N_HOMALT=0;N_HOMREF=10308;FREQ_HET=9.700261907071492e-05;FREQ_HOMALT=0.0;FREQ_HOMREF=0.9999029973809292;N_BI_GENOS=10309;East_Han_AN=6554;East_Han_AC=1;East_Han_AF=
chr2	21049356	.	T	<INS:ME:LINE1>	.	PASS	SVTYPE=INS:ME:LINE1;END=21049367;SVLEN=6007;AN=20618;AC=3;AF=0.00014550392860607236;N_HET=3;N_HOMALT=0;N_HOMREF=10306;FREQ_HET=0.0002910078572121447;FREQ_HOMALT=0.0;FREQ_HOMREF=0.9997089921427879;N_BI_GENOS=10309;North_Han_AN=2272;North_Han_AC=1;North_Han
chr2	21873463	.	T	<INS:ME:LINE1>	.	PASS	SVTYPE=INS:ME:LINE1;END=21873521;SVLEN=1649;AN=20618;AC=1;AF=4.850130953535746e-05;N_HET=1;N_HOMALT=0;N_HOMREF=10308;FREQ_HET=9.700261907071492e-05;FREQ_HOMALT=0.0;FREQ_HOMREF=0.9999029973809292;N_BI_GENOS=10309;East_Han_AN=6554;East_Han_AC=1;East_Han_AF=
chr2	21885432	.	A	<INS:ME:LINE1>	.	PASS	SVTYPE=INS:ME:LINE1;END=21885446;SVLEN=6014;AN=20618;AC=1;AF=4.850130953535746e-05;N_HET=1;N_HOMALT=0;N_HOMREF=10308;FREQ_HET=9.700261907071492e-05;FREQ_HOMALT=0.0;FREQ_HOMREF=0.9999029973809292;N_BI_GENOS=10309;East_Han_AN=6554;East_Han_AC=1;East_Han_AF=
chr2	22090755	.	G	<INS:ME:LINE1>	.	PASS	SVTYPE=INS:ME:LINE1;END=22090755;SVLEN=19;AN=20618;AC=1;AF=4.850130953535746e-05;N_HET=1;N_HOMALT=0;N_HOMREF=10308;FREQ_HET=9.700261907071492e-05;FREQ_HOMALT=0.0;FREQ_HOMREF=0.9999029973809292;N_BI_GENOS=10309;South_Han_AN=1496;South_Han_AC=1;South_Han_AF
chr2	22353592	.	T	<INS:ME:LINE1>	.	PASS	SVTYPE=INS:ME:LINE1;END=22353592;SVLEN=95;AN=20618;AC=6;AF=0.0002910078572121447;N_HET=6;N_HOMALT=0;N_HOMREF=10303;FREQ_HET=0.0005820157144242894;FREQ_HOMALT=0.0;FREQ_HOMREF=0.9994179842855757;N_BI_GENOS=10309;Lingnan_Han_AN=1888;Lingnan_Han_AC=1;Lingnan_
chr2	22353745	.	A	<INS:ME:LINE1>	.	PASS	SVTYPE=INS:ME:LINE1;END=22353762;SVLEN=95;AN=20618;AC=8;AF=0.00038801047628285966;N_HET=8;N_HOMALT=0;N_HOMREF=10301;FREQ_HET=0.0007760209525657193;FREQ_HOMALT=0.0;FREQ_HOMREF=0.9992239790474343;N_BI_GENOS=10309;South_Han_AN=1496;South_Han_AC=3;South_Han_A
chr2	22435708	.	A	<INS:ME:LINE1>	.	PASS	SVTYPE=INS:ME:LINE1;END=22435722;SVLEN=749;AN=20618;AC=17;AF=0.0008245222621010767;N_HET=15;N_HOMALT=1;N_HOMREF=10293;FREQ_HET=0.0014550392860607237;FREQ_HOMALT=9.700261907071492e-05;FREQ_HOMREF=0.9984479580948685;N_BI_GENOS=10309;South_Han_AN=1496;South_
chr2	22483450	.	A	<INS:ME:LINE1>	.	PASS	SVTYPE=INS:ME:LINE1;END=22483467;SVLEN=816;AN=20618;AC=7;AF=0.0003395091667475022;N_HET=7;N_HOMALT=0;N_HOMREF=10302;FREQ_HET=0.0006790183334950044;FREQ_HOMALT=0.0;FREQ_HOMREF=0.999320981666505;N_BI_GENOS=10309;Northwest_Han_AN=1296;Northwest_Han_AC=2;Nort
chr2	22542230	.	T	<INS:ME:LINE1>	.	PASS	SVTYPE=INS:ME:LINE1;END=22542243;SVLEN=6011;AN=20618;AC=1;AF=4.850130953535746e-05;N_HET=1;N_HOMALT=0;N_HOMREF=10308;FREQ_HET=9.700261907071492e-05;FREQ_HOMALT=0.0;FREQ_HOMREF=0.9999029973809292;N_BI_GENOS=10309;Lingnan_Han_AN=1888;Lingnan_Han_AC=1;Lingna
chr2	22838154	.	A	<INS:ME:LINE1>	.	PASS	SVTYPE=INS:ME:LINE1;END=22838162;SVLEN=107;AN=20618;AC=7;AF=0.0003395091667475022;N_HET=7;N_HOMALT=0;N_HOMREF=10302;FREQ_HET=0.0006790183334950044;FREQ_HOMALT=0.0;FREQ_HOMREF=0.999320981666505;N_BI_GENOS=10309;North_Han_AN=2272;North_Han_AC=1;North_Han_AF
chr2	22967690	.	C	<INS:ME:LINE1>	.	PASS	SVTYPE=INS:ME:LINE1;END=22967708;SVLEN=808;AN=20618;AC=1;AF=4.850130953535746e-05;N_HET=1;N_HOMALT=0;N_HOMREF=10308;FREQ_HET=9.700261907071492e-05;FREQ_HOMALT=0.0;FREQ_HOMREF=0.9999029973809292;N_BI_GENOS=10309;North_Han_AN=2272;North_Han_AC=1;North_Han_A
chr2	22968127	.	G	<INS:ME:LINE1>	.	PASS	SVTYPE=INS:ME:LINE1;END=22968144;SVLEN=5998;AN=20618;AC=12158;AF=0.589678921330876;N_HET=4824;N_HOMALT=3667;N_HOMREF=1818;FREQ_HET=0.46794063439712874;FREQ_HOMALT=0.3557086041323116;FREQ_HOMREF=0.1763507614705597;N_BI_GENOS=10309;South_Han_AN=1496;South_H
chr2	23241467	.	A	<INS:ME:LINE1>	.	PASS	SVTYPE=INS:ME:LINE1;END=23241481;SVLEN=2778;AN=20618;AC=18;AF=0.0008730235716364342;N_HET=18;N_HOMALT=0;N_HOMREF=10291;FREQ_HET=0.0017460471432728683;FREQ_HOMALT=0.0;FREQ_HOMREF=0.9982539528567271;N_BI_GENOS=10309;North_Han_AN=2272;North_Han_AC=1;North_Ha
chr2	23268930	.	A	<INS:ME:LINE1>	.	PASS	SVTYPE=INS:ME:LINE1;END=23268941;SVLEN=5993;AN=20618;AC=1;AF=4.850130953535746e-05;N_HET=1;N_HOMALT=0;N_HOMREF=10308;FREQ_HET=9.700261907071492e-05;FREQ_HOMALT=0.0;FREQ_HOMREF=0.9999029973809292;N_BI_GENOS=10309;East_Han_AN=6554;East_Han_AC=1;East_Han_AF=
chr2	23408361	.	A	<INS:ME:LINE1>	.	PASS	SVTYPE=INS:ME:LINE1;END=23408378;SVLEN=5998;AN=20618;AC=1;AF=4.850130953535746e-05;N_HET=1;N_HOMALT=0;N_HOMREF=10308;FREQ_HET=9.700261907071492e-05;FREQ_HOMALT=0.0;FREQ_HOMREF=0.9999029973809292;N_BI_GENOS=10309;Southeast_Han_AN=1908;Southeast_Han_AC=1;So
chr2	23627240	.	A	<INS:ME:LINE1>	.	PASS	SVTYPE=INS:ME:LINE1;END=23627256;SVLEN=473;AN=20618;AC=1;AF=4.850130953535746e-05;N_HET=1;N_HOMALT=0;N_HOMREF=10308;FREQ_HET=9.700261907071492e-05;FREQ_HOMALT=0.0;FREQ_HOMREF=0.9999029973809292;N_BI_GENOS=10309;North_Han_AN=2272;North_Han_AC=1;North_Han_A
chr2	23830298	.	A	<INS:ME:LINE1>	.	PASS	SVTYPE=INS:ME:LINE1;END=23830316;SVLEN=679;AN=20618;AC=20;AF=0.0009700261907071491;N_HET=14;N_HOMALT=3;N_HOMREF=10292;FREQ_HET=0.0013580366669900088;FREQ_HOMALT=0.0002910078572121447;FREQ_HOMREF=0.9983509554757979;N_BI_GENOS=10309;South_Han_AN=1496;South_
chr2	23946759	.	T	<INS:ME:LINE1>	.	PASS	SVTYPE=INS:ME:LINE1;END=23946767;SVLEN=197;AN=20618;AC=1;AF=4.850130953535746e-05;N_HET=1;N_HOMALT=0;N_HOMREF=10308;FREQ_HET=9.700261907071492e-05;FREQ_HOMALT=0.0;FREQ_HOMREF=0.9999029973809292;N_BI_GENOS=10309;East_Han_AN=6554;East_Han_AC=1;East_Han_AF=0

chr2	24573887	.	A	<INS:ME:LINE1>	.	PASS	SVTYPE=INS:ME:LINE1;END=24573902;SVLEN=1873;AN=20618;AC=2753;AF=0.13352410515083907;N_HET=1829;N_HOMALT=462;N_HOMREF=8018;FREQ_HET=0.17741779028033758;FREQ_HOMALT=0.044815210010670285;FREQ_HOMREF=0.7777669997089921;N_BI_GENOS=10309;South_Han_AN=1496;South
chr2	25184712	.	A	<INS:ME:LINE1>	.	PASS	SVTYPE=INS:ME:LINE1;END=25184727;SVLEN=924;AN=20618;AC=7;AF=0.0003395091667475022;N_HET=7;N_HOMALT=0;N_HOMREF=10302;FREQ_HET=0.0006790183334950044;FREQ_HOMALT=0.0;FREQ_HOMREF=0.999320981666505;N_BI_GENOS=10309;South_Han_AN=1496;South_Han_AF
chr2	25441811	.	T	<INS:ME:LINE1>	.	PASS	SVTYPE=INS:ME:LINE1;END=25441819;SVLEN=780;AN=20618;AC=1;AF=4.850130953535746e-05;N_HET=1;N_HOMALT=0;N_HOMREF=10308;FREQ_HET=9.700261907071492e-05;FREQ_HOMALT=0.0;FREQ_HOMREF=0.9999029973809292;N_BI_GENOS=10309;East_Han_AN=6554;East_Han_AC=1;East_Han_AF=0
chr2	25455808	.	T	<INS:ME:LINE1>	.	PASS	SVTYPE=INS:ME:LINE1;END=25455824;SVLEN=2064;AN=20618;AC=1;AF=4.850130953535746e-05;N_HET=1;N_HOMALT=0;N_HOMREF=10308;FREQ_HET=9.700261907071492e-05;FREQ_HOMALT=0.0;FREQ_HOMREF=0.9999029973809292;N_BI_GENOS=10309;Central_Han_AN=2128;Central_Han_AC=1;Centra
chr2	25606741	.	G	<INS:ME:LINE1>	.	PASS	SVTYPE=INS:ME:LINE1;END=25606759;SVLEN=230;AN=20618;AC=1;AF=4.850130953535746e-05;N_HET=1;N_HOMALT=0;N_HOMREF=10308;FREQ_HET=9.700261907071492e-05;FREQ_HOMALT=0.0;FREQ_HOMREF=0.9999029973809292;N_BI_GENOS=10309;Central_Han_AN=2128;Central_Han_AC=1;Central
chr2	26688019	.	T	<INS:ME:LINE1>	.	PASS	SVTYPE=INS:ME:LINE1;END=26688020;SVLEN=1064;AN=20618;AC=1;AF=4.850130953535746e-05;N_HET=1;N_HOMALT=0;N_HOMREF=10308;FREQ_HET=9.700261907071492e-05;FREQ_HOMALT=0.0;FREQ_HOMREF=0.9999029973809292;N_BI_GENOS=10309;Mongolian_AN=454;Mongolian_AC=1;Mongolian_A
chr2	27550129	.	T	<INS:ME:LINE1>	.	PASS	SVTYPE=INS:ME:LINE1;END=27550143;SVLEN=5998;AN=20618;AC=2;AF=9.700261907071492e-05;N_HET=2;N_HOMALT=0;N_HOMREF=10307;FREQ_HET=0.00019400523814142983;FREQ_HOMALT=0.0;FREQ_HOMREF=0.9998059947618586;N_BI_GENOS=10309;Central_Han_AN=2128;Central_Han_AC=2;Centr
chr2	27856126	.	G	<INS:ME:LINE1>	.	PASS	SVTYPE=INS:ME:LINE1;END=27856127;SVLEN=1289;AN=20618;AC=2;AF=9.700261907071492e-05;N_HET=2;N_HOMALT=0;N_HOMREF=10307;FREQ_HET=0.00019400523814142983;FREQ_HOMALT=0.0;FREQ_HOMREF=0.9998059947618586;N_BI_GENOS=10309;Central_Han_AN=2128;Central_Han_AC=2;Centr
chr2	28152747	.	A	<INS:ME:LINE1>	.	PASS	SVTYPE=INS:ME:LINE1;END=28152764;SVLEN=5998;AN=20618;AC=6;AF=0.0002910078572121447;N_HET=6;N_HOMALT=0;N_HOMREF=10303;FREQ_HET=0.0005820157144242894;FREQ_HOMALT=0.0;FREQ_HOMREF=0.9994179842855757;N_BI_GENOS=10309;North_Han_AN=2272;North_Han_AC=1;North_Han_
chr2	28283776	.	T	<INS:ME:LINE1>	.	PASS	SVTYPE=INS:ME:LINE1;END=28283793;SVLEN=5997;AN=20618;AC=20;AF=0.0009700261907071491;N_HET=20;N_HOMALT=0;N_HOMREF=10289;FREQ_HET=0.0019400523814142982;FREQ_HOMALT=0.0;FREQ_HOMREF=0.9980599476185857;N_BI_GENOS=10309;South_Han_AN=1496;South_Han_AC=1;South_Ha
chr2	28409729	.	A	<INS:ME:LINE1>	.	PASS	SVTYPE=INS:ME:LINE1;END=28409740;SVLEN=405;AN=20618;AC=5;AF=0.00024250654767678728;N_HET=3;N_HOMALT=1;N_HOMREF=10305;FREQ_HET=0.0002910078572121447;FREQ_HOMALT=9.700261907071492e-05;FREQ_HOMREF=0.9996119895237171;N_BI_GENOS=10309;South_Han_AN=1496;South_H
chr2	29031653	.	A	<INS:ME:LINE1>	.	PASS	SVTYPE=INS:ME:LINE1;END=29031653;SVLEN=248;AN=20618;AC=1;AF=4.850130953535746e-05;N_HET=1;N_HOMALT=0;N_HOMREF=10308;FREQ_HET=9.700261907071492e-05;FREQ_HOMALT=0.0;FREQ_HOMREF=0.9999029973809292;N_BI_GENOS=10309;Lingnan_Han_AN=1888;Lingnan_Han_AC=1;Lingnan
chr2	29356392	.	G	<INS:ME:LINE1>	.	PASS	SVTYPE=INS:ME:LINE1;END=29356407;SVLEN=6017;AN=20618;AC=3;AF=0.00014550392860607236;N_HET=3;N_HOMALT=0;N_HOMREF=10306;FREQ_HET=0.0002910078572121447;FREQ_HOMALT=0.0;FREQ_HOMREF=0.9997089921427879;N_BI_GENOS=10309;Central_Han_AN=2128;Central_Han_AC=3;Centr
chr2	29506973	.	A	<INS:ME:LINE1>	.	PASS	SVTYPE=INS:ME:LINE1;END=29506989;SVLEN=6150;AN=20618;AC=1;AF=4.850130953535746e-05;N_HET=1;N_HOMALT=0;N_HOMREF=10308;FREQ_HET=9.700261907071492e-05;FREQ_HOMALT=0.0;FREQ_HOMREF=0.9999029973809292;N_BI_GENOS=10309;East_Han_AN=6554;East_Han_AC=1;East_Han_AF=
chr2	29656004	.	G	<INS:ME:LINE1>	.	PASS	SVTYPE=INS:ME:LINE1;END=29656004;SVLEN=2399;AN=20618;AC=2;AF=9.700261907071492e-05;N_HET=0;N_HOMALT=1;N_HOMREF=10308;FREQ_HET=0.0;FREQ_HOMALT=9.700261907071492e-05;FREQ_HOMREF=0.9999029973809292;N_BI_GENOS=10309;Central_Han_AN=2128;Central_Han_AC=2;Centra
chr2	29860171	.	C	<INS:ME:LINE1>	.	PASS	SVTYPE=INS:ME:LINE1;END=29860188;SVLEN=1380;AN=20618;AC=1;AF=4.850130953535746e-05;N_HET=1;N_HOMALT=0;N_HOMREF=10308;FREQ_HET=9.700261907071492e-05;FREQ_HOMALT=0.0;FREQ_HOMREF=0.9999029973809292;N_BI_GENOS=10309;Central_Han_AN=2128;Central_Han_AC=1;Centra
chr2	29883975	.	A	<INS:ME:LINE1>	.	PASS	SVTYPE=INS:ME:LINE1;END=29883988;SVLEN=803;AN=20618;AC=2;AF=9.700261907071492e-05;N_HET=2;N_HOMALT=0;N_HOMREF=10307;FREQ_HET=0.00019400523814142983;FREQ_HOMALT=0.0;FREQ_HOMREF=0.9998059947618586;N_BI_GENOS=10309;Lingnan_Han_AN=1888;Lingnan_Han_AC=2;Lingna
chr2	30947895	.	A	<INS:ME:LINE1>	.	PASS	SVTYPE=INS:ME:LINE1;END=30947911;SVLEN=699;AN=20618;AC=1;AF=4.850130953535746e-05;N_HET=1;N_HOMALT=0;N_HOMREF=10308;FREQ_HET=9.700261907071492e-05;FREQ_HOMALT=0.0;FREQ_HOMREF=0.9999029973809292;N_BI_GENOS=10309;Central_Han_AN=2128;Central_Han_AC=1;Central
chr2	31112830	.	G	<INS:ME:LINE1>	.	PASS	SVTYPE=INS:ME:LINE1;END=31112838;SVLEN=297;AN=20618;AC=48;AF=0.0023280628576971578;N_HET=48;N_HOMALT=0;N_HOMREF=10261;FREQ_HET=0.0046561257153943155;FREQ_HOMALT=0.0;FREQ_HOMREF=0.9953438742846057;N_BI_GENOS=10309;South_Han_AN=1496;South_Han_AC=7;South_Han
chr2	31421579	.	T	<INS:ME:LINE1>	.	PASS	SVTYPE=INS:ME:LINE1;END=31421590;SVLEN=6006;AN=20618;AC=2;AF=9.700261907071492e-05;N_HET=2;N_HOMALT=0;N_HOMREF=10307;FREQ_HET=0.00019400523814142983;FREQ_HOMALT=0.0;FREQ_HOMREF=0.9998059947618586;N_BI_GENOS=10309;East_Han_AN=6554;East_Han_AC=2;East_Han_AF
chr2	31550662	.	A	<INS:ME:LINE1>	.	PASS	SVTYPE=INS:ME:LINE1;END=31550672;SVLEN=5999;AN=20618;AC=1;AF=4.850130953535746e-05;N_HET=1;N_HOMALT=0;N_HOMREF=10308;FREQ_HET=9.700261907071492e-05;FREQ_HOMALT=0.0;FREQ_HOMREF=0.9999029973809292;N_BI_GENOS=10309;Northwest_Han_AN=1296;Northwest_Han_AC=1;No
chr2	31579536	.	T	<INS:ME:LINE1>	.	PASS	SVTYPE=INS:ME:LINE1;END=31579547;SVLEN=1051;AN=20618;AC=2;AF=9.700261907071492e-05;N_HET=2;N_HOMALT=0;N_HOMREF=10307;FREQ_HET=0.00019400523814142983;FREQ_HOMALT=0.0;FREQ_HOMREF=0.9998059947618586;N_BI_GENOS=10309;Northwest_Han_AN=1296;Northwest_Han_AC=1;N
chr2	31723454	.	A	<INS:ME:LINE1>	.	PASS	SVTYPE=INS:ME:LINE1;END=31723467;SVLEN=6003;AN=20618;AC=1;AF=4.850130953535746e-05;N_HET=1;N_HOMALT=0;N_HOMREF=10308;FREQ_HET=9.700261907071492e-05;FREQ_HOMALT=0.0;FREQ_HOMREF=0.9999029973809292;N_BI_GENOS=10309;Manchu_AN=416;Manchu_AC=1;Manchu_AF=0.00240
chr2	31770345	.	A	<INS:ME:LINE1>	.	PASS	SVTYPE=INS:ME:LINE1;END=31770360;SVLEN=1862;AN=20618;AC=2;AF=9.700261907071492e-05;N_HET=2;N_HOMALT=0;N_HOMREF=10307;FREQ_HET=0.00019400523814142983;FREQ_HOMALT=0.0;FREQ_HOMREF=0.9998059947618586;N_BI_GENOS=10309;Southeast_Han_AN=1908;Southeast_Han_AC=2;S
chr2	32715957	.	T	<INS:ME:LINE1>	.	PASS	SVTYPE=INS:ME:LINE1;END=32715960;SVLEN=946;AN=20618;AC=2;AF=9.700261907071492e-05;N_HET=0;N_HOMALT=1;N_HOMREF=10308;FREQ_HET=0.0;FREQ_HOMALT=9.700261907071492e-05;FREQ_HOMREF=0.9999029973809292;N_BI_GENOS=10309;East_Han_AN=6554;East_Han_AC=2;East_Han_AF=0
chr2	32858686	.	T	<INS:ME:LINE1>	.	PASS	SVTYPE=INS:ME:LINE1;END=32858705;SVLEN=5999;AN=20618;AC=5;AF=0.00024250654767678728;N_HET=5;N_HOMALT=0;N_HOMREF=10304;FREQ_HET=0.00048501309535357455;FREQ_HOMALT=0.0;FREQ_HOMREF=0.9995149869046465;N_BI_GENOS=10309;South_Han_AN=1496;South_Han_AC=1;South_Ha
chr2	33112992	.	A	<INS:ME:LINE1>	.	PASS	SVTYPE=INS:ME:LINE1;END=33113006;SVLEN=5998;AN=20618;AC=1;AF=4.850130953535746e-05;N_HET=1;N_HOMALT=0;N_HOMREF=10308;FREQ_HET=9.700261907071492e-05;FREQ_HOMALT=0.0;FREQ_HOMREF=0.9999029973809292;N_BI_GENOS=10309;Northwest_Han_AN=1296;Northwest_Han_AC=1;No
chr2	33202205	.	T	<INS:ME:LINE1>	.	PASS	SVTYPE=INS:ME:LINE1;END=33202217;SVLEN=5961;AN=20618;AC=1;AF=4.850130953535746e-05;N_HET=1;N_HOMALT=0;N_HOMREF=10308;FREQ_HET=9.700261907071492e-05;FREQ_HOMALT=0.0;FREQ_HOMREF=0.9999029973809292;N_BI_GENOS=10309;North_Han_AN=2272;North_Han_AC=1;North_Han_
chr2	33251961	.	A	<INS:ME:LINE1>	.	PASS	SVTYPE=INS:ME:LINE1;END=33251968;SVLEN=2100;AN=20618;AC=1;AF=4.850130953535746e-05;N_HET=1;N_HOMALT=0;N_HOMREF=10308;FREQ_HET=9.700261907071492e-05;FREQ_HOMALT=0.0;FREQ_HOMREF=0.9999029973809292;N_BI_GENOS=10309;Southeast_Han_AN=1908;Southeast_Han_AC=1;So
chr2	33283152	.	A	<INS:ME:LINE1>	.	PASS	SVTYPE=INS:ME:LINE1;END=33283168;SVLEN=3778;AN=20618;AC=1;AF=4.850130953535746e-05;N_HET=1;N_HOMALT=0;N_HOMREF=10308;FREQ_HET=9.700261907071492e-05;FREQ_HOMALT=0.0;FREQ_HOMREF=0.9999029973809292;N_BI_GENOS=10309;Lingnan_Han_AN=1888;Lingnan_Han_AC=1;Lingna
chr2	33436885	.	A	<INS:ME:LINE1>	.	PASS	SVTYPE=INS:ME:LINE1;END=33436901;SVLEN=6004;AN=20618;AC=2;AF=9.700261907071492e-05;N_HET=2;N_HOMALT=0;N_HOMREF=10307;FREQ_HET=0.00019400523814142983;FREQ_HOMALT=0.0;FREQ_HOMREF=0.9998059947618586;N_BI_GENOS=10309;East_Han_AN=6554;East_Han_AC=2;East_Han_AF
chr2	33842910	.	C	<INS:ME:LINE1>	.	PASS	SVTYPE=INS:ME:LINE1;END=33842924;SVLEN=787;AN=20618;AC=2;AF=9.700261907071492e-05;N_HET=2;N_HOMALT=0;N_HOMREF=10307;FREQ_HET=0.00019400523814142983;FREQ_HOMALT=0.0;FREQ_HOMREF=0.9998059947618586;N_BI_GENOS=10309;East_Han_AN=6554;East_Han_AC=2;East_Han_AF=
chr2	33887628	.	C	<INS:ME:LINE1>	.	PASS	SVTYPE=INS:ME:LINE1;END=33887639;SVLEN=6004;AN=20618;AC=1;AF=4.850130953535746e-05;N_HET=1;N_HOMALT=0;N_HOMREF=10308;FREQ_HET=9.700261907071492e-05;FREQ_HOMALT=0.0;FREQ_HOMREF=0.9999029973809292;N_BI_GENOS=10309;East_Han_AN=6554;East_Han_AC=1;East_Han_AF=
chr2	34008772	.	G	<INS:ME:LINE1>	.	PASS	SVTYPE=INS:ME:LINE1;END=34008772;SVLEN=0;AN=20618;AC=1;AF=4.850130953535746e-05;N_HET=1;N_HOMALT=0;N_HOMREF=10308;FREQ_HET=9.700261907071492e-05;FREQ_HOMALT=0.0;FREQ_HOMREF=0.9999029973809292;N_BI_GENOS=10309;East_Han_AN=6554;East_Han_AC=1;East_Han_AF=0.0
chr2	34096838	.	A	<INS:ME:LINE1>	.	PASS	SVTYPE=INS:ME:LINE1;END=34096851;SVLEN=979;AN=20618;AC=1;AF=4.850130953535746e-05;N_HET=1;N_HOMALT=0;N_HOMREF=10308;FREQ_HET=9.700261907071492e-05;FREQ_HOMALT=0.0;FREQ_HOMREF=0.9999029973809292;N_BI_GENOS=10309;Southeast_Han_AN=1908;Southeast_Han_AC=1;Sou
chr2	34268149	.	A	<INS:ME:LINE1>	.	PASS	SVTYPE=INS:ME:LINE1;END=34268165;SVLEN=759;AN=20618;AC=24;AF=0.0011640314288485789;N_HET=18;N_HOMALT=3;N_HOMREF=10288;FREQ_HET=0.0017460471432728683;FREQ_HOMALT=0.0002910078572121447;FREQ_HOMREF=0.997962944999515;N_BI_GENOS=10309;South_Han_AN=1496;South_H
chr2	34805115	.	C	<INS:ME:LINE1>	.	PASS	SVTYPE=INS:ME:LINE1;END=34805115;SVLEN=0;AN=20618;AC=1;AF=4.850130953535746e-05;N_HET=1;N_HOMALT=0;N_HOMREF=10308;FREQ_HET=9.700261907071492e-05;FREQ_HOMALT=0.0;FREQ_HOMREF=0.9999029973809292;N_BI_GENOS=10309;Southeast_Han_AN=1908;Southeast_Han_AC=1;South
chr2	35153857	.	G	<INS:ME:LINE1>	.	PASS	SVTYPE=INS:ME:LINE1;END=35153857;SVLEN=670;AN=20618;AC=1;AF=4.850130953535746e-05;N_HET=1;N_HOMALT=0;N_HOMREF=10308;FREQ_HET=9.700261907071492e-05;FREQ_HOMALT=0.0;FREQ_HOMREF=0.9999029973809292;N_BI_GENOS=10309;East_Han_AN=6554;East_Han_AC=1;East_Han_AF=0
chr2	35178344	.	C	<INS:ME:LINE1>	.	PASS	SVTYPE=INS:ME:LINE1;END=35178344;SVLEN=3637;AN=20618;AC=1;AF=4.850130953535746e-05;N_HET=1;N_HOMALT=0;N_HOMREF=10308;FREQ_HET=9.700261907071492e-05;FREQ_HOMALT=0.0;FREQ_HOMREF=0.9999029973809292;N_BI_GENOS=10309;East_Han_AN=6554;East_Han_AC=1;East_Han_AF=
chr2	35260656	.	C	<INS:ME:LINE1>	.	PASS	SVTYPE=INS:ME:LINE1;END=35260668;SVLEN=108;AN=20618;AC=4;AF=0.00019400523814142983;N_HET=4;N_HOMALT=0;N_HOMREF=10305;FREQ_HET=0.00038801047628285966;FREQ_HOMALT=0.0;FREQ_HOMREF=0.9996119895237171;N_BI_GENOS=10309;North_Han_AN=2272;North_Han_AC=1;North_Han
chr2	35268178	.	T	<INS:ME:LINE1>	.	PASS	SVTYPE=INS:ME:LINE1;END=35268178;SVLEN=879;AN=20618;AC=1;AF=4.850130953535746e-05;N_HET=1;N_HOMALT=0;N_HOMREF=10308;FREQ_HET=9.700261907071492e-05;FREQ_HOMALT=0.0;FREQ_HOMREF=0.9999029973809292;N_BI_GENOS=10309;Central_Han_AN=2128;Central_Han_AC=1;Central
chr2	35395065	.	A	<INS:ME:LINE1>	.	PASS	SVTYPE=INS:ME:LINE1;END=35395078;SVLEN=1361;AN=20618;AC=1;AF=4.850130953535746e-05;N_HET=1;N_HOMALT=0;N_HOMREF=10308;FREQ_HET=9.700261907071492e-05;FREQ_HOMALT=0.0;FREQ_HOMREF=0.9999029973809292;N_BI_GENOS=10309;East_Han_AN=6554;East_Han_AC=1;East_Han_AF=
chr2	35589912	.	A	<INS:ME:LINE1>	.	PASS	SVTYPE=INS:ME:LINE1;END=35589926;SVLEN=1643;AN=20618;AC=1;AF=4.850130953535746e-05;N_HET=1;N_HOMALT=0;N_HOMREF=10308;FREQ_HET=9.700261907071492e-05;FREQ_HOMALT=0.0;FREQ_HOMREF=0.9999029973809292;N_BI_GENOS=10309;Lingnan_Han_AN=1888;Lingnan_Han_AC=1;Lingna
chr2	35602476	.	A	<INS:ME:LINE1>	.	PASS	SVTYPE=INS:ME:LINE1;END=35602489;SVLEN=788;AN=20618;AC=1;AF=4.850130953535746e-05;N_HET=1;N_HOMALT=0;N_HOMREF=10308;FREQ_HET=9.700261907071492e-05;FREQ_HOMALT=0.0;FREQ_HOMREF=0.9999029973809292;N_BI_GENOS=10309;East_Han_AN=6554;East_Han_AC=1;East_Han_AF=0
chr2	35654275	.	A	<INS:ME:LINE1>	.	PASS	SVTYPE=INS:ME:LINE1;END=35654288;SVLEN=5994;AN=20618;AC=1928;AF=0.09351052478416917;N_HET=984;N_HOMALT=472;N_HOMREF=8853;FREQ_HET=0.09545057716558347;FREQ_HOMALT=0.045785236201377436;FREQ_HOMREF=0.8587641866330391;N_BI_GENOS=10309;South_Han_AN=1496;South_
chr2	35702218	.	C	<INS:ME:LINE1>	.	PASS	SVTYPE=INS:ME:LINE1;END=35702229;SVLEN=6013;AN=20618;AC=1;AF=4.850130953535746e-05;N_HET=1;N_HOMALT=0;N_HOMREF=10308;FREQ_HET=9.700261907071492e-05;FREQ_HOMALT=0.0;FREQ_HOMREF=0.9999029973809292;N_BI_GENOS=10309;Northwest_Han_AN=1296;Northwest_Han_AC=1;No
chr2	35704395	.	T	<INS:ME:LINE1>	.	PASS	SVTYPE=INS:ME:LINE1;END=35704411;SVLEN=6000;AN=20618;AC=1;AF=4.850130953535746e-05;N_HET=1;N_HOMALT=0;N_HOMREF=10308;FREQ_HET=9.700261907071492e-05;FREQ_HOMALT=0.0;FREQ_HOMREF=0.9999029973809292;N_BI_GENOS=10309;East_Han_AN=6554;East_Han_AC=1;East_Han_AF=
chr2	36000454	.	C	<INS:ME:LINE1>	.	PASS	SVTYPE=INS:ME:LINE1;END=36000459;SVLEN=1346;AN=20618;AC=1;AF=4.850130953535746e-05;N_HET=1;N_HOMALT=0;N_HOMREF=10308;FREQ_HET=9.700261907071492e-05;FREQ_HOMALT=0.0;FREQ_HOMREF=0.9999029973809292;N_BI_GENOS=10309;Central_Han_AN=2128;Central_Han_AC=1;Centra
chr2	36044020	.	A	<INS:ME:LINE1>	.	PASS	SVTYPE=INS:ME:LINE1;END=36044033;SVLEN=6018;AN=20618;AC=1;AF=4.850130953535746e-05;N_HET=1;N_HOMALT=0;N_HOMREF=10308;FREQ_HET=9.700261907071492e-05;FREQ_HOMALT=0.0;FREQ_HOMREF=0.9999029973809292;N_BI_GENOS=10309;Lingnan_Han_AN=1888;Lingnan_Han_AC=1;Lingna
chr2	36154094	.	G	<INS:ME:LINE1>	.	PASS	SVTYPE=INS:ME:LINE1;END=36154109;SVLEN=1453;AN=20618;AC=3228;AF=0.15656222718013385;N_HET=2284;N_HOMALT=472;N_HOMREF=7553;FREQ_HET=0.22155398195751286;FREQ_HOMALT=0.045785236201377436;FREQ_HOMREF=0.7326607818411097;N_BI_GENOS=10309;South_Han_AN=1496;South
chr2	36225025	.	G	<INS:ME:LINE1>	.	PASS	SVTYPE=INS:ME:LINE1;END=36225040;SVLEN=2610;AN=20618;AC=7;AF=0.0003395091667475022;N_HET=7;N_HOMALT=0;N_HOMREF=10302;FREQ_HET=0.0006790183334950044;FREQ_HOMALT=0.0;FREQ_HOMREF=0.999320981666505;N_BI_GENOS=10309;Southeast_Han_AN=1908;Southeast_Han_AC=7;Sou
chr2	36601256	.	C	<INS:ME:LINE1>	.	PASS	SVTYPE=INS:ME:LINE1;END=36601256;SVLEN=0;AN=20618;AC=1;AF=4.850130953535746e-05;N_HET=1;N_HOMALT=0;N_HOMREF=10308;FREQ_HET=9.700261907071492e-05;FREQ_HOMALT=0.0;FREQ_HOMREF=0.9999029973809292;N_BI_GENOS=10309;Southeast_Han_AN=1908;Southeast_Han_AC=1;South
chr2	36677500	.	A	<INS:ME:LINE1>	.	PASS	SVTYPE=INS:ME:LINE1;END=36677514;SVLEN=220;AN=20618;AC=7;AF=0.0003395091667475022;N_HET=5;N_HOMALT=1;N_HOMREF=10303;FREQ_HET=0.00048501309535357455;FREQ_HOMALT=9.700261907071492e-05;FREQ_HOMREF=0.9994179842855757;N_BI_GENOS=10309;Northwest_Han_AN=1296;Nor
chr2	37382212	.	C	<INS:ME:LINE1>	.	PASS	SVTYPE=INS:ME:LINE1;END=37382212;SVLEN=0;AN=20618;AC=2;AF=9.700261907071492e-05;N_HET=0;N_HOMALT=1;N_HOMREF=10308;FREQ_HET=0.0;FREQ_HOMALT=9.700261907071492e-05;FREQ_HOMREF=0.9999029973809292;N_BI_GENOS=10309;Lingnan_Han_AN=1888;Lingnan_Han_AC=2;Lingnan_H
chr2	37439123	.	T	<INS:ME:LINE1>	.	PASS	SVTYPE=INS:ME:LINE1;END=37439136;SVLEN=5996;AN=20618;AC=1;AF=4.850130953535746e-05;N_HET=1;N_HOMALT=0;N_HOMREF=10308;FREQ_HET=9.700261907071492e-05;FREQ_HOMALT=0.0;FREQ_HOMREF=0.9999029973809292;N_BI_GENOS=10309;Southeast_Han_AN=1908;Southeast_Han_AC=1;So
chr2	37480483	.	A	<INS:ME:LINE1>	.	PASS	SVTYPE=INS:ME:LINE1;END=37480501;SVLEN=6002;AN=20618;AC=5;AF=0.00024250654767678728;N_HET=5;N_HOMALT=0;N_HOMREF=10304;FREQ_HET=0.00048501309535357455;FREQ_HOMALT=0.0;FREQ_HOMREF=0.9995149869046465;N_BI_GENOS=10309;North_Han_AN=2272;North_Han_AC=1;North_Ha
chr2	38682169	.	A	<INS:ME:LINE1>	.	PASS	SVTYPE=INS:ME:LINE1;END=38682187;SVLEN=516;AN=20618;AC=1;AF=4.850130953535746e-05;N_HET=1;N_HOMALT=0;N_HOMREF=10308;FREQ_HET=9.700261907071492e-05;FREQ_HOMALT=0.0;FREQ_HOMREF=0.9999029973809292;N_BI_GENOS=10309;Southeast_Han_AN=1908;Southeast_Han_AC=1;Sou
chr2	38921739	.	G	<INS:ME:LINE1>	.	PASS	SVTYPE=INS:ME:LINE1;END=38921754;SVLEN=6000;AN=20618;AC=1;AF=4.850130953535746e-05;N_HET=1;N_HOMALT=0;N_HOMREF=10308;FREQ_HET=9.700261907071492e-05;FREQ_HOMALT=0.0;FREQ_HOMREF=0.9999029973809292;N_BI_GENOS=10309;Lingnan_Han_AN=1888;Lingnan_Han_AC=1;Lingna
chr2	39024749	.	T	<INS:ME:LINE1>	.	PASS	SVTYPE=INS:ME:LINE1;END=39024765;SVLEN=6033;AN=20618;AC=1;AF=4.850130953535746e-05;N_HET=1;N_HOMALT=0;N_HOMREF=10308;FREQ_HET=9.700261907071492e-05;FREQ_HOMALT=0.0;FREQ_HOMREF=0.9999029973809292;N_BI_GENOS=10309;Central_Han_AN=2128;Central_Han_AC=1;Centra
chr2	39271747	.	T	<INS:ME:LINE1>	.	PASS	SVTYPE=INS:ME:LINE1;END=39271763;SVLEN=345;AN=20618;AC=2;AF=9.700261907071492e-05;N_HET=2;N_HOMALT=0;N_HOMREF=10307;FREQ_HET=0.00019400523814142983;FREQ_HOMALT=0.0;FREQ_HOMREF=0.9998059947618586;N_BI_GENOS=10309;East_Han_AN=6554;East_Han_AC=1;East_Han_AF=
chr2	39414143	.	A	<INS:ME:LINE1>	.	PASS	SVTYPE=INS:ME:LINE1;END=39414160;SVLEN=162;AN=20618;AC=1;AF=4.850130953535746e-05;N_HET=1;N_HOMALT=0;N_HOMREF=10308;FREQ_HET=9.700261907071492e-05;FREQ_HOMALT=0.0;FREQ_HOMREF=0.9999029973809292;N_BI_GENOS=10309;North_Han_AN=2272;North_Han_AC=1;North_Han_A
chr2	39719645	.	G	<INS:ME:LINE1>	.	PASS	SVTYPE=INS:ME:LINE1;END=39719656;SVLEN=6016;AN=20618;AC=1;AF=4.850130953535746e-05;N_HET=1;N_HOMALT=0;N_HOMREF=10308;FREQ_HET=9.700261907071492e-05;FREQ_HOMALT=0.0;FREQ_HOMREF=0.9999029973809292;N_BI_GENOS=10309;Central_Han_AN=2128;Central_Han_AC=1;Centra
chr2	40332706	.	A	<INS:ME:LINE1>	.	PASS	SVTYPE=INS:ME:LINE1;END=40332710;SVLEN=358;AN=20618;AC=1;AF=4.850130953535746e-05;N_HET=1;N_HOMALT=0;N_HOMREF=10308;FREQ_HET=9.700261907071492e-05;FREQ_HOMALT=0.0;FREQ_HOMREF=0.9999029973809292;N_BI_GENOS=10309;Lingnan_Han_AN=1888;Lingnan_Han_AC=1;Lingnan
chr2	40471197	.	G	<INS:ME:LINE1>	.	PASS	SVTYPE=INS:ME:LINE1;END=40471211;SVLEN=1222;AN=20618;AC=4;AF=0.00019400523814142983;N_HET=4;N_HOMALT=0;N_HOMREF=10305;FREQ_HET=0.00038801047628285966;FREQ_HOMALT=0.0;FREQ_HOMREF=0.9996119895237171;N_BI_GENOS=10309;South_Han_AN=1496;South_Han_AC=1;South_Ha
chr2	40597781	.	T	<INS:ME:LINE1>	.	PASS	SVTYPE=INS:ME:LINE1;END=40597785;SVLEN=439;AN=20618;AC=2;AF=9.700261907071492e-05;N_HET=2;N_HOMALT=0;N_HOMREF=10307;FREQ_HET=0.00019400523814142983;FREQ_HOMALT=0.0;FREQ_HOMREF=0.9998059947618586;N_BI_GENOS=10309;North_Han_AN=2272;North_Han_AC=1;North_
chr2	40749711	.	G	<INS:ME:LINE1>	.	PASS	SVTYPE=INS:ME:LINE1;END=40749727;SVLEN=45;AN=20618;AC=4;AF=0.00019400523814142983;N_HET=2;N_HOMALT=1;N_HOMREF=10306;FREQ_HET=0.00019400523814142983;FREQ_HOMALT=9.700261907071492e-05;FREQ_HOMREF=0.9997089921427879;N_BI_GENOS=10309;East_Han_AN=6554;East_Han
chr2	40891783	.	A	<INS:ME:LINE1>	.	PASS	SVTYPE=INS:ME:LINE1;END=40891798;SVLEN=6008;AN=20618;AC=1;AF=4.850130953535746e-05;N_HET=1;N_HOMALT=0;N_HOMREF=10308;FREQ_HET=9.700261907071492e-05;FREQ_HOMALT=0.0;FREQ_HOMREF=0.9999029973809292;N_BI_GENOS=10309;Central_Han_AN=2128;Central_Han_AC=1;Centra
chr2	40911856	.	T	<INS:ME:LINE1>	.	PASS	SVTYPE=INS:ME:LINE1;END=40911856;SVLEN=157;AN=20618;AC=1;AF=4.850130953535746e-05;N_HET=1;N_HOMALT=0;N_HOMREF=10308;FREQ_HET=9.700261907071492e-05;FREQ_HOMALT=0.0;FREQ_HOMREF=0.9999029973809292;N_BI_GENOS=10309;East_Han_AN=6554;East_Han_AC=1;East_Han_AF=0
chr2	40926707	.	G	<INS:ME:LINE1>	.	PASS	SVTYPE=INS:ME:LINE1;END=40926722;SVLEN=962;AN=20618;AC=1;AF=4.850130953535746e-05;N_HET=1;N_HOMALT=0;N_HOMREF=10308;FREQ_HET=9.700261907071492e-05;FREQ_HOMALT=0.0;FREQ_HOMREF=0.9999029973809292;N_BI_GENOS=10309;Lingnan_Han_AN=1888;Lingnan_Han_AC=1;Lingnan
chr2	41006977	.	T	<INS:ME:LINE1>	.	PASS	SVTYPE=INS:ME:LINE1;END=41006990;SVLEN=6006;AN=20618;AC=1;AF=4.850130953535746e-05;N_HET=1;N_HOMALT=0;N_HOMREF=10308;FREQ_HET=9.700261907071492e-05;FREQ_HOMALT=0.0;FREQ_HOMREF=0.9999029973809292;N_BI_GENOS=10309;North_Han_AN=2272;North_Han_AC=1;North_Han_
chr2	41127959	.	A	<INS:ME:LINE1>	.	PASS	SVTYPE=INS:ME:LINE1;END=41127974;SVLEN=935;AN=20618;AC=3;AF=0.00014550392860607236;N_HET=3;N_HOMALT=0;N_HOMREF=10306;FREQ_HET=0.0002910078572121447;FREQ_HOMALT=0.0;FREQ_HOMREF=0.9997089921427879;N_BI_GENOS=10309;East_Han_AN=6554;East_Han_AC=3;East_Han_AF=
chr2	41579226	.	A	<INS:ME:LINE1>	.	PASS	SVTYPE=INS:ME:LINE1;END=41579241;SVLEN=5999;AN=20618;AC=1;AF=4.850130953535746e-05;N_HET=1;N_HOMALT=0;N_HOMREF=10308;FREQ_HET=9.700261907071492e-05;FREQ_HOMALT=0.0;FREQ_HOMREF=0.9999029973809292;N_BI_GENOS=10309;Southeast_Han_AN=1908;Southeast_Han_AC=1;So
chr2	41601043	.	G	<INS:ME:LINE1>	.	PASS	SVTYPE=INS:ME:LINE1;END=41601043;SVLEN=1585;AN=20618;AC=1;AF=4.850130953535746e-05;N_HET=1;N_HOMALT=0;N_HOMREF=10308;FREQ_HET=9.700261907071492e-05;FREQ_HOMALT=0.0;FREQ_HOMREF=0.9999029973809292;N_BI_GENOS=10309;Central_Han_AN=2128;Central_Han_AC=1;Centra
chr2	41667694	.	T	<INS:ME:LINE1>	.	PASS	SVTYPE=INS:ME:LINE1;END=41667710;SVLEN=1535;AN=20618;AC=1;AF=4.850130953535746e-05;N_HET=1;N_HOMALT=0;N_HOMREF=10308;FREQ_HET=9.700261907071492e-05;FREQ_HOMALT=0.0;FREQ_HOMREF=0.9999029973809292;N_BI_GENOS=10309;Northwest_Han_AN=1296;Northwest_Han_AC=1;No
chr2	41686511	.	G	<INS:ME:LINE1>	.	PASS	SVTYPE=INS:ME:LINE1;END=41686521;SVLEN=5999;AN=20618;AC=994;AF=0.04821030167814531;N_HET=694;N_HOMALT=150;N_HOMREF=9465;FREQ_HET=0.06731981763507615;FREQ_HOMALT=0.014550392860607237;FREQ_HOMREF=0.9181297895043166;N_BI_GENOS=10309;South_Han_AN=1496;South_H
chr2	41714713	.	A	<INS:ME:LINE1>	.	PASS	SVTYPE=INS:ME:LINE1;END=41714713;SVLEN=6010;AN=20618;AC=3;AF=0.00014550392860607236;N_HET=3;N_HOMALT=0;N_HOMREF=10306;FREQ_HET=0.0002910078572121447;FREQ_HOMALT=0.0;FREQ_HOMREF=0.9997089921427879;N_BI_GENOS=10309;Lingnan_Han_AN=1888;Lingnan_Han_AC=2;Lingn
chr2	41822563	.	A	<INS:ME:LINE1>	.	PASS	SVTYPE=INS:ME:LINE1;END=41822577;SVLEN=273;AN=20618;AC=1;AF=4.850130953535746e-05;N_HET=1;N_HOMALT=0;N_HOMREF=10308;FREQ_HET=9.700261907071492e-05;FREQ_HOMALT=0.0;FREQ_HOMREF=0.9999029973809292;N_BI_GENOS=10309;Lingnan_Han_AN=1888;Lingnan_Han_AC=1;Lingnan
chr2	41824263	.	A	<INS:ME:LINE1>	.	PASS	SVTYPE=INS:ME:LINE1;END=41824276;SVLEN=5999;AN=20618;AC=563;AF=0.027306237268406246;N_HET=515;N_HOMALT=24;N_HOMREF=9770;FREQ_HET=0.04995634882141818;FREQ_HOMALT=0.0023280628576971578;FREQ_HOMREF=0.9477155883208846;N_BI_GENOS=10309;South_Han_AN=1496;South_
chr2	42746296	.	T	<INS:ME:LINE1>	.	PASS	SVTYPE=INS:ME:LINE1;END=42746310;SVLEN=345;AN=20618;AC=3;AF=0.00014550392860607236;N_HET=3;N_HOMALT=0;N_HOMREF=10306;FREQ_HET=0.0002910078572121447;FREQ_HOMALT=0.0;FREQ_HOMREF=0.9997089921427879;N_BI_GENOS=10309;Northwest_Han_AN=1296;Northwest_Han_AC=1;No
chr2	42772689	.	A	<INS:ME:LINE1>	.	PASS	SVTYPE=INS:ME:LINE1;END=42772705;SVLEN=245;AN=20618;AC=1;AF=4.850130953535746e-05;N_HET=1;N_HOMALT=0;N_HOMREF=10308;FREQ_HET=9.700261907071492e-05;FREQ_HOMALT=0.0;FREQ_HOMREF=0.9999029973809292;N_BI_GENOS=10309;North_Han_AN=2272;North_Han_AC=1;North_Han_A
chr2	42894163	.	A	<INS:ME:LINE1>	.	PASS	SVTYPE=INS:ME:LINE1;END=42894163;SVLEN=1052;AN=20618;AC=1;AF=4.850130953535746e-05;N_HET=1;N_HOMALT=0;N_HOMREF=10308;FREQ_HET=9.700261907071492e-05;FREQ_HOMALT=0.0;FREQ_HOMREF=0.9999029973809292;N_BI_GENOS=10309;Northwest_Han_AN=1296;Northwest_Han_AC=1;No
chr2	44662558	.	T	<INS:ME:LINE1>	.	PASS	SVTYPE=INS:ME:LINE1;END=44662558;SVLEN=17;AN=20618;AC=1;AF=4.850130953535746e-05;N_HET=1;N_HOMALT=0;N_HOMREF=10308;FREQ_HET=9.700261907071492e-05;FREQ_HOMALT=0.0;FREQ_HOMREF=0.9999029973809292;N_BI_GENOS=10309;Northwest_Han_AN=1296;Northwest_Han_AC=1;Nort
chr2	44825053	.	A	<INS:ME:LINE1>	.	PASS	SVTYPE=INS:ME:LINE1;END=44825069;SVLEN=306;AN=20618;AC=1;AF=4.850130953535746e-05;N_HET=1;N_HOMALT=0;N_HOMREF=10308;FREQ_HET=9.700261907071492e-05;FREQ_HOMALT=0.0;FREQ_HOMREF=0.9999029973809292;N_BI_GENOS=10309;Central_Han_AN=2128;Central_Han_AC=1;Central
chr2	45068523	.	G	<INS:ME:LINE1>	.	PASS	SVTYPE=INS:ME:LINE1;END=45068523;SVLEN=4131;AN=20618;AC=1;AF=4.850130953535746e-05;N_HET=1;N_HOMALT=0;N_HOMREF=10308;FREQ_HET=9.700261907071492e-05;FREQ_HOMALT=0.0;FREQ_HOMREF=0.9999029973809292;N_BI_GENOS=10309;North_Han_AN=2272;North_Han_AC=1;North_Han_
chr2	45561799	.	A	<INS:ME:LINE1>	.	PASS	SVTYPE=INS:ME:LINE1;END=45561811;SVLEN=746;AN=20618;AC=4;AF=0.00019400523814142983;N_HET=4;N_HOMALT=0;N_HOMREF=10305;FREQ_HET=0.00038801047628285966;FREQ_HOMALT=0.0;FREQ_HOMREF=0.9996119895237171;N_BI_GENOS=10309;Southeast_Han_AN=1908;Southeast_Han_AC=4;S
chr2	45774927	.	G	<INS:ME:LINE1>	.	PASS	SVTYPE=INS:ME:LINE1;END=45774942;SVLEN=892;AN=20618;AC=834;AF=0.04045009215248812;N_HET=766;N_HOMALT=34;N_HOMREF=9509;FREQ_HET=0.07430400620816761;FREQ_HOMALT=0.0032980890484043067;FREQ_HOMREF=0.9223979047434281;N_BI_GENOS=10309;South_Han_AN=1496;South_Ha
chr2	45830801	.	T	<INS:ME:LINE1>	.	PASS	SVTYPE=INS:ME:LINE1;END=45830801;SVLEN=6000;AN=20618;AC=1;AF=4.850130953535746e-05;N_HET=1;N_HOMALT=0;N_HOMREF=10308;FREQ_HET=9.700261907071492e-05;FREQ_HOMALT=0.0;FREQ_HOMREF=0.9999029973809292;N_BI_GENOS=10309;Lingnan_Han_AN=1888;Lingnan_Han_AC=1;Lingna
chr2	46075019	.	C	<INS:ME:LINE1>	.	PASS	SVTYPE=INS:ME:LINE1;END=46075020;SVLEN=4190;AN=20618;AC=2;AF=9.700261907071492e-05;N_HET=2;N_HOMALT=0;N_HOMREF=10307;FREQ_HET=0.00019400523814142983;FREQ_HOMALT=0.0;FREQ_HOMREF=0.9998059947618586;N_BI_GENOS=10309;East_Han_AN=6554;East_Han_AC=2;East_Han_AF
chr2	46287170	.	T	<INS:ME:LINE1>	.	PASS	SVTYPE=INS:ME:LINE1;END=46287181;SVLEN=1422;AN=20618;AC=1;AF=4.850130953535746e-05;N_HET=1;N_HOMALT=0;N_HOMREF=10308;FREQ_HET=9.700261907071492e-05;FREQ_HOMALT=0.0;FREQ_HOMREF=0.9999029973809292;N_BI_GENOS=10309;East_Han_AN=6554;East_Han_AC=1;East_Han_AF=
chr2	46517248	.	A	<INS:ME:LINE1>	.	PASS	SVTYPE=INS:ME:LINE1;END=46517266;SVLEN=484;AN=20618;AC=1;AF=4.850130953535746e-05;N_HET=1;N_HOMALT=0;N_HOMREF=10308;FREQ_HET=9.700261907071492e-05;FREQ_HOMALT=0.0;FREQ_HOMREF=0.9999029973809292;N_BI_GENOS=10309;South_Han_AN=1496;South_Han_AC=1;South_Han_A
chr2	47085025	.	G	<INS:ME:LINE1>	.	PASS	SVTYPE=INS:ME:LINE1;END=47085025;SVLEN=0;AN=20618;AC=2;AF=9.700261907071492e-05;N_HET=0;N_HOMALT=1;N_HOMREF=10308;FREQ_HET=0.0;FREQ_HOMALT=9.700261907071492e-05;FREQ_HOMREF=0.9999029973809292;N_BI_GENOS=10309;East_Han_AN=6554;East_Han_AC=2;East_Han_AF=0.0
chr2	47872920	.	T	<INS:ME:LINE1>	.	PASS	SVTYPE=INS:ME:LINE1;END=47872929;SVLEN=514;AN=20618;AC=5;AF=0.00024250654767678728;N_HET=5;N_HOMALT=0;N_HOMREF=10304;FREQ_HET=0.00048501309535357455;FREQ_HOMALT=0.0;FREQ_HOMREF=0.9995149869046465;N_BI_GENOS=10309;Northwest_Han_AN=1296;Northwest_Han_AC=1;N
chr2	48417452	.	A	<INS:ME:LINE1>	.	PASS	SVTYPE=INS:ME:LINE1;END=48417452;SVLEN=19;AN=20618;AC=2;AF=9.700261907071492e-05;N_HET=2;N_HOMALT=0;N_HOMREF=10307;FREQ_HET=0.00019400523814142983;FREQ_HOMALT=0.0;FREQ_HOMREF=0.9998059947618586;N_BI_GENOS=10309;Southeast_Han_AN=1908;Southeast_Han_AC=1;Sou
chr2	48596062	.	A	<INS:ME:LINE1>	.	PASS	SVTYPE=INS:ME:LINE1;END=48596075;SVLEN=5997;AN=20618;AC=1;AF=4.850130953535746e-05;N_HET=1;N_HOMALT=0;N_HOMREF=10308;FREQ_HET=9.700261907071492e-05;FREQ_HOMALT=0.0;FREQ_HOMREF=0.9999029973809292;N_BI_GENOS=10309;East_Han_AN=6554;East_Han_AC=1;East_Han_AF=
chr2	48604395	.	A	<INS:ME:LINE1>	.	PASS	SVTYPE=INS:ME:LINE1;END=48604409;SVLEN=5983;AN=20618;AC=1;AF=4.850130953535746e-05;N_HET=1;N_HOMALT=0;N_HOMREF=10308;FREQ_HET=9.700261907071492e-05;FREQ_HOMALT=0.0;FREQ_HOMREF=0.9999029973809292;N_BI_GENOS=10309;North_Han_AN=2272;North_Han_AC=1;North_Han_
chr2	48768481	.	T	<INS:ME:LINE1>	.	PASS	SVTYPE=INS:ME:LINE1;END=48768496;SVLEN=1724;AN=20618;AC=2;AF=9.700261907071492e-05;N_HET=2;N_HOMALT=0;N_HOMREF=10307;FREQ_HET=0.00019400523814142983;FREQ_HOMALT=0.0;FREQ_HOMREF=0.9998059947618586;N_BI_GENOS=10309;South_Han_AN=1496;South_Han_AC=1;South_Han

chr2	49452137	.	G	<INS:ME:LINE1>	.	PASS	SVTYPE=INS:ME:LINE1;END=49452150;SVLEN=5998;AN=20618;AC=19;AF=0.000921524881171916;N_HET=19;N_HOMALT=0;N_HOMREF=10290;FREQ_HET=0.0018430497623435833;FREQ_HOMALT=0.0;FREQ_HOMREF=0.9981569502376564;N_BI_GENOS=10309;South_Han_AN=1496;South_Han_AC=5;South_Ha
chr2	49504750	.	T	<INS:ME:LINE1>	.	PASS	SVTYPE=INS:ME:LINE1;END=49504761;SVLEN=538;AN=20618;AC=1;AF=4.850130953535746e-05;N_HET=1;N_HOMALT=0;N_HOMREF=10308;FREQ_HET=9.700261907071492e-05;FREQ_HOMALT=0.0;FREQ_HOMREF=0.9999029973809292;N_BI_GENOS=10309;Northwest_Han_AN=1296;Northwest_Han_AC=1;Nor
chr2	49708305	.	T	<INS:ME:LINE1>	.	PASS	SVTYPE=INS:ME:LINE1;END=49708313;SVLEN=3453;AN=20618;AC=1;AF=4.850130953535746e-05;N_HET=1;N_HOMALT=0;N_HOMREF=10308;FREQ_HET=9.700261907071492e-05;FREQ_HOMALT=0.0;FREQ_HOMREF=0.9999029973809292;N_BI_GENOS=10309;East_Han_AN=6554;East_Han_AC=1;East_Han_AF=
chr2	49952434	.	C	<INS:ME:LINE1>	.	PASS	SVTYPE=INS:ME:LINE1;END=49952442;SVLEN=5999;AN=20618;AC=5;AF=0.00024250654767678728;N_HET=5;N_HOMALT=0;N_HOMREF=10304;FREQ_HET=0.00048501309535357455;FREQ_HOMALT=0.0;FREQ_HOMREF=0.9995149869046465;N_BI_GENOS=10309;South_Han_AN=1496;South_Han_AC=1;South_Ha
chr2	49952541	.	T	<INS:ME:LINE1>	.	PASS	SVTYPE=INS:ME:LINE1;END=49952556;SVLEN=584;AN=20618;AC=10;AF=0.00048501309535357455;N_HET=10;N_HOMALT=0;N_HOMREF=10299;FREQ_HET=0.0009700261907071491;FREQ_HOMALT=0.0;FREQ_HOMREF=0.9990299738092928;N_BI_GENOS=10309;South_Han_AN=1496;South_Han_AC=4;South_Ha
chr2	49984445	.	A	<INS:ME:LINE1>	.	PASS	SVTYPE=INS:ME:LINE1;END=49984445;SVLEN=3712;AN=20618;AC=1;AF=4.850130953535746e-05;N_HET=1;N_HOMALT=0;N_HOMREF=10308;FREQ_HET=9.700261907071492e-05;FREQ_HOMALT=0.0;FREQ_HOMREF=0.9999029973809292;N_BI_GENOS=10309;South_Han_AN=1496;South_Han_AC=1;South_Han_
chr2	50442855	.	T	<INS:ME:LINE1>	.	PASS	SVTYPE=INS:ME:LINE1;END=50442870;SVLEN=1504;AN=20618;AC=1;AF=4.850130953535746e-05;N_HET=1;N_HOMALT=0;N_HOMREF=10308;FREQ_HET=9.700261907071492e-05;FREQ_HOMALT=0.0;FREQ_HOMREF=0.9999029973809292;N_BI_GENOS=10309;Lingnan_Han_AN=1888;Lingnan_Han_AC=1;Lingna
chr2	50444165	.	A	<INS:ME:LINE1>	.	PASS	SVTYPE=INS:ME:LINE1;END=50444181;SVLEN=706;AN=20618;AC=1;AF=4.850130953535746e-05;N_HET=1;N_HOMALT=0;N_HOMREF=10308;FREQ_HET=9.700261907071492e-05;FREQ_HOMALT=0.0;FREQ_HOMREF=0.9999029973809292;N_BI_GENOS=10309;East_Han_AN=6554;East_Han_AC=1;East_Han_AF=0
chr2	50454637	.	A	<INS:ME:LINE1>	.	PASS	SVTYPE=INS:ME:LINE1;END=50454641;SVLEN=3890;AN=20618;AC=1;AF=4.850130953535746e-05;N_HET=1;N_HOMALT=0;N_HOMREF=10308;FREQ_HET=9.700261907071492e-05;FREQ_HOMALT=0.0;FREQ_HOMREF=0.9999029973809292;N_BI_GENOS=10309;East_Han_AN=6554;East_Han_AC=1;East_Han_AF=
chr2	50679544	.	A	<INS:ME:LINE1>	.	PASS	SVTYPE=INS:ME:LINE1;END=50679556;SVLEN=2206;AN=20618;AC=1;AF=4.850130953535746e-05;N_HET=1;N_HOMALT=0;N_HOMREF=10308;FREQ_HET=9.700261907071492e-05;FREQ_HOMALT=0.0;FREQ_HOMREF=0.9999029973809292;N_BI_GENOS=10309;Hui_AN=444;Hui_AC=1;Hui_AF=0.00225225225225
chr2	50906451	.	T	<INS:ME:LINE1>	.	PASS	SVTYPE=INS:ME:LINE1;END=50906466;SVLEN=6000;AN=20618;AC=2;AF=9.700261907071492e-05;N_HET=2;N_HOMALT=0;N_HOMREF=10307;FREQ_HET=0.00019400523814142983;FREQ_HOMALT=0.0;FREQ_HOMREF=0.9998059947618586;N_BI_GENOS=10309;East_Han_AN=6554;East_Han_AC=1;East_Han_AF
chr2	50944539	.	A	<INS:ME:LINE1>	.	PASS	SVTYPE=INS:ME:LINE1;END=50944545;SVLEN=761;AN=20618;AC=1;AF=4.850130953535746e-05;N_HET=1;N_HOMALT=0;N_HOMREF=10308;FREQ_HET=9.700261907071492e-05;FREQ_HOMALT=0.0;FREQ_HOMREF=0.9999029973809292;N_BI_GENOS=10309;Southeast_Han_AN=1908;Southeast_Han_AC=1;Sou
chr2	51096984	.	T	<INS:ME:LINE1>	.	PASS	SVTYPE=INS:ME:LINE1;END=51097001;SVLEN=186;AN=20618;AC=1;AF=4.850130953535746e-05;N_HET=1;N_HOMALT=0;N_HOMREF=10308;FREQ_HET=9.700261907071492e-05;FREQ_HOMALT=0.0;FREQ_HOMREF=0.9999029973809292;N_BI_GENOS=10309;Northwest_Han_AN=1296;Northwest_Han_AC=1;Nor
chr2	51525441	.	G	<INS:ME:LINE1>	.	PASS	SVTYPE=INS:ME:LINE1;END=51525453;SVLEN=599;AN=20618;AC=1;AF=4.850130953535746e-05;N_HET=1;N_HOMALT=0;N_HOMREF=10308;FREQ_HET=9.700261907071492e-05;FREQ_HOMALT=0.0;FREQ_HOMREF=0.9999029973809292;N_BI_GENOS=10309;Yi_AN=422;Yi_AC=1;Yi_AF=0.002369668246445498
chr2	51752922	.	A	<INS:ME:LINE1>	.	PASS	SVTYPE=INS:ME:LINE1;END=51752937;SVLEN=1347;AN=20618;AC=1;AF=4.850130953535746e-05;N_HET=1;N_HOMALT=0;N_HOMREF=10308;FREQ_HET=9.700261907071492e-05;FREQ_HOMALT=0.0;FREQ_HOMREF=0.9999029973809292;N_BI_GENOS=10309;East_Han_AN=6554;East_Han_AC=1;East_Han_AF=
chr2	51760301	.	G	<INS:ME:LINE1>	.	PASS	SVTYPE=INS:ME:LINE1;END=51760320;SVLEN=5445;AN=20618;AC=41;AF=0.0019885536909496557;N_HET=39;N_HOMALT=1;N_HOMREF=10269;FREQ_HET=0.0037831021437578815;FREQ_HOMALT=9.700261907071492e-05;FREQ_HOMREF=0.9961198952371714;N_BI_GENOS=10309;South_Han_AN=1496;South
chr2	51952000	.	A	<INS:ME:LINE1>	.	PASS	SVTYPE=INS:ME:LINE1;END=51952016;SVLEN=4199;AN=20618;AC=1;AF=4.850130953535746e-05;N_HET=1;N_HOMALT=0;N_HOMREF=10308;FREQ_HET=9.700261907071492e-05;FREQ_HOMALT=0.0;FREQ_HOMREF=0.9999029973809292;N_BI_GENOS=10309;East_Han_AN=6554;East_Han_AC=1;East_Han_AF=
chr2	51979120	.	C	<INS:ME:LINE1>	.	PASS	SVTYPE=INS:ME:LINE1;END=51979139;SVLEN=6000;AN=20618;AC=2;AF=9.700261907071492e-05;N_HET=2;N_HOMALT=0;N_HOMREF=10307;FREQ_HET=0.00019400523814142983;FREQ_HOMALT=0.0;FREQ_HOMREF=0.9998059947618586;N_BI_GENOS=10309;North_Han_AN=2272;North_Han_AC=1;North_Han
chr2	52141009	.	G	<INS:ME:LINE1>	.	PASS	SVTYPE=INS:ME:LINE1;END=52141009;SVLEN=6046;AN=20618;AC=1;AF=4.850130953535746e-05;N_HET=1;N_HOMALT=0;N_HOMREF=10308;FREQ_HET=9.700261907071492e-05;FREQ_HOMALT=0.0;FREQ_HOMREF=0.9999029973809292;N_BI_GENOS=10309;Central_Han_AN=2128;Central_Han_AC=1;Centra
chr2	52863827	.	A	<INS:ME:LINE1>	.	PASS	SVTYPE=INS:ME:LINE1;END=52863829;SVLEN=810;AN=20618;AC=1;AF=4.850130953535746e-05;N_HET=1;N_HOMALT=0;N_HOMREF=10308;FREQ_HET=9.700261907071492e-05;FREQ_HOMALT=0.0;FREQ_HOMREF=0.9999029973809292;N_BI_GENOS=10309;North_Han_AN=2272;North_Han_AC=1;North_Han_A
chr2	52890918	.	G	<INS:ME:LINE1>	.	PASS	SVTYPE=INS:ME:LINE1;END=52890926;SVLEN=5995;AN=20618;AC=1;AF=4.850130953535746e-05;N_HET=1;N_HOMALT=0;N_HOMREF=10308;FREQ_HET=9.700261907071492e-05;FREQ_HOMALT=0.0;FREQ_HOMREF=0.9999029973809292;N_BI_GENOS=10309;South_Han_AN=1496;South_Han_AC=1;South_Han_
chr2	53232913	.	A	<INS:ME:LINE1>	.	PASS	SVTYPE=INS:ME:LINE1;END=53232913;SVLEN=161;AN=20618;AC=1;AF=4.850130953535746e-05;N_HET=1;N_HOMALT=0;N_HOMREF=10308;FREQ_HET=9.700261907071492e-05;FREQ_HOMALT=0.0;FREQ_HOMREF=0.9999029973809292;N_BI_GENOS=10309;Tibetan_AN=414;Tibetan_AC=1;Tibetan_AF=0.002
chr2	53424061	.	A	<INS:ME:LINE1>	.	PASS	SVTYPE=INS:ME:LINE1;END=53424077;SVLEN=259;AN=20618;AC=1;AF=4.850130953535746e-05;N_HET=1;N_HOMALT=0;N_HOMREF=10308;FREQ_HET=9.700261907071492e-05;FREQ_HOMALT=0.0;FREQ_HOMREF=0.9999029973809292;N_BI_GENOS=10309;Mongolian_AN=454;Mongolian_AC=1;Mongolian_AF
chr2	53503714	.	T	<INS:ME:LINE1>	.	PASS	SVTYPE=INS:ME:LINE1;END=53503726;SVLEN=6004;AN=20618;AC=1;AF=4.850130953535746e-05;N_HET=1;N_HOMALT=0;N_HOMREF=10308;FREQ_HET=9.700261907071492e-05;FREQ_HOMALT=0.0;FREQ_HOMREF=0.9999029973809292;N_BI_GENOS=10309;East_Han_AN=6554;East_Han_AC=1;East_Han_AF=
chr2	53691604	.	C	<INS:ME:LINE1>	.	PASS	SVTYPE=INS:ME:LINE1;END=53691604;SVLEN=0;AN=20618;AC=24;AF=0.0011640314288485789;N_HET=22;N_HOMALT=1;N_HOMREF=10286;FREQ_HET=0.002134057619555728;FREQ_HOMALT=9.700261907071492e-05;FREQ_HOMREF=0.9977689397613736;N_BI_GENOS=10309;South_Han_AN=1496;South_Han
chr2	54080138	.	C	<INS:ME:LINE1>	.	PASS	SVTYPE=INS:ME:LINE1;END=54080152;SVLEN=5998;AN=20618;AC=1;AF=4.850130953535746e-05;N_HET=1;N_HOMALT=0;N_HOMREF=10308;FREQ_HET=9.700261907071492e-05;FREQ_HOMALT=0.0;FREQ_HOMREF=0.9999029973809292;N_BI_GENOS=10309;North_Han_AN=2272;North_Han_AC=1;North_Han_
chr2	54252435	.	G	<INS:ME:LINE1>	.	PASS	SVTYPE=INS:ME:LINE1;END=54252435;SVLEN=114;AN=20618;AC=1;AF=4.850130953535746e-05;N_HET=1;N_HOMALT=0;N_HOMREF=10308;FREQ_HET=9.700261907071492e-05;FREQ_HOMALT=0.0;FREQ_HOMREF=0.9999029973809292;N_BI_GENOS=10309;East_Han_AN=6554;East_Han_AC=1;East_Han_AF=0
chr2	54745279	.	A	<INS:ME:LINE1>	.	PASS	SVTYPE=INS:ME:LINE1;END=54745293;SVLEN=2640;AN=20618;AC=1;AF=4.850130953535746e-05;N_HET=1;N_HOMALT=0;N_HOMREF=10308;FREQ_HET=9.700261907071492e-05;FREQ_HOMALT=0.0;FREQ_HOMREF=0.9999029973809292;N_BI_GENOS=10309;East_Han_AN=6554;East_Han_AC=1;East_Han_AF=
chr2	54812526	.	T	<INS:ME:LINE1>	.	PASS	SVTYPE=INS:ME:LINE1;END=54812538;SVLEN=5997;AN=20618;AC=1;AF=4.850130953535746e-05;N_HET=1;N_HOMALT=0;N_HOMREF=10308;FREQ_HET=9.700261907071492e-05;FREQ_HOMALT=0.0;FREQ_HOMREF=0.9999029973809292;N_BI_GENOS=10309;East_Han_AN=6554;East_Han_AC=1;East_Han_AF=
chr2	55091867	.	G	<INS:ME:LINE1>	.	PASS	SVTYPE=INS:ME:LINE1;END=55091867;SVLEN=0;AN=20618;AC=6;AF=0.0002910078572121447;N_HET=4;N_HOMALT=1;N_HOMREF=10304;FREQ_HET=0.00038801047628285966;FREQ_HOMALT=9.700261907071492e-05;FREQ_HOMREF=0.9995149869046465;N_BI_GENOS=10309;Northwest_Han_AN=1296;North
chr2	55401463	.	A	<INS:ME:LINE1>	.	PASS	SVTYPE=INS:ME:LINE1;END=55401463;SVLEN=0;AN=20618;AC=4;AF=0.00019400523814142983;N_HET=0;N_HOMALT=2;N_HOMREF=10307;FREQ_HET=0.0;FREQ_HOMALT=0.00019400523814142983;FREQ_HOMREF=0.9998059947618586;N_BI_GENOS=10309;South_Han_AN=1496;South_Han_AC=2;South_Han_A
chr2	55401571	.	C	<INS:ME:LINE1>	.	PASS	SVTYPE=INS:ME:LINE1;END=55401571;SVLEN=0;AN=20618;AC=1;AF=4.850130953535746e-05;N_HET=1;N_HOMALT=0;N_HOMREF=10308;FREQ_HET=9.700261907071492e-05;FREQ_HOMALT=0.0;FREQ_HOMREF=0.9999029973809292;N_BI_GENOS=10309;Lingnan_Han_AN=1888;Lingnan_Han_AC=1;Lingnan_H
chr2	55831521	.	C	<INS:ME:LINE1>	.	PASS	SVTYPE=INS:ME:LINE1;END=55831542;SVLEN=738;AN=20618;AC=1;AF=4.850130953535746e-05;N_HET=1;N_HOMALT=0;N_HOMREF=10308;FREQ_HET=9.700261907071492e-05;FREQ_HOMALT=0.0;FREQ_HOMREF=0.9999029973809292;N_BI_GENOS=10309;East_Han_AN=6554;East_Han_AC=1;East_Han_AF=0
chr2	55860620	.	A	<INS:ME:LINE1>	.	PASS	SVTYPE=INS:ME:LINE1;END=55860628;SVLEN=1241;AN=20618;AC=12;AF=0.0005820157144242894;N_HET=12;N_HOMALT=0;N_HOMREF=10297;FREQ_HET=0.0011640314288485789;FREQ_HOMALT=0.0;FREQ_HOMREF=0.9988359685711514;N_BI_GENOS=10309;Northwest_Han_AN=1296;Northwest_Han_AC=3;
chr2	56130233	.	C	<INS:ME:LINE1>	.	PASS	SVTYPE=INS:ME:LINE1;END=56130247;SVLEN=6002;AN=20618;AC=11;AF=0.000533514404888932;N_HET=11;N_HOMALT=0;N_HOMREF=10298;FREQ_HET=0.001067028809777864;FREQ_HOMALT=0.0;FREQ_HOMREF=0.9989329711902222;N_BI_GENOS=10309;South_Han_AN=1496;South_Han_AC=2;South_Han_
chr2	56206340	.	T	<INS:ME:LINE1>	.	PASS	SVTYPE=INS:ME:LINE1;END=56206341;SVLEN=206;AN=20618;AC=1;AF=4.850130953535746e-05;N_HET=1;N_HOMALT=0;N_HOMREF=10308;FREQ_HET=9.700261907071492e-05;FREQ_HOMALT=0.0;FREQ_HOMREF=0.9999029973809292;N_BI_GENOS=10309;Lingnan_Han_AN=1888;Lingnan_Han_AC=1;Lingnan
chr2	56324104	.	T	<INS:ME:LINE1>	.	PASS	SVTYPE=INS:ME:LINE1;END=56324112;SVLEN=706;AN=20618;AC=5;AF=0.00024250654767678728;N_HET=5;N_HOMALT=0;N_HOMREF=10304;FREQ_HET=0.00048501309535357455;FREQ_HOMALT=0.0;FREQ_HOMREF=0.9995149869046465;N_BI_GENOS=10309;South_Han_AN=1496;South_Han_AC=1;South_Han
chr2	56386933	.	T	<INS:ME:LINE1>	.	PASS	SVTYPE=INS:ME:LINE1;END=56386944;SVLEN=3882;AN=20618;AC=1;AF=4.850130953535746e-05;N_HET=1;N_HOMALT=0;N_HOMREF=10308;FREQ_HET=9.700261907071492e-05;FREQ_HOMALT=0.0;FREQ_HOMREF=0.9999029973809292;N_BI_GENOS=10309;Southeast_Han_AN=1908;Southeast_Han_AC=1;So
chr2	56413983	.	A	<INS:ME:LINE1>	.	PASS	SVTYPE=INS:ME:LINE1;END=56413998;SVLEN=434;AN=20618;AC=1;AF=4.850130953535746e-05;N_HET=1;N_HOMALT=0;N_HOMREF=10308;FREQ_HET=9.700261907071492e-05;FREQ_HOMALT=0.0;FREQ_HOMREF=0.9999029973809292;N_BI_GENOS=10309;North_Han_AN=2272;North_Han_AC=1;North_Han_A
chr2	56482920	.	A	<INS:ME:LINE1>	.	PASS	SVTYPE=INS:ME:LINE1;END=56482937;SVLEN=1265;AN=20618;AC=9;AF=0.0004365117858182171;N_HET=9;N_HOMALT=0;N_HOMREF=10300;FREQ_HET=0.0008730235716364342;FREQ_HOMALT=0.0;FREQ_HOMREF=0.9991269764283636;N_BI_GENOS=10309;Northwest_Han_AN=1296;Northwest_Han_AC=2;No
chr2	56580469	.	C	<INS:ME:LINE1>	.	PASS	SVTYPE=INS:ME:LINE1;END=56580513;SVLEN=675;AN=20618;AC=1;AF=4.850130953535746e-05;N_HET=1;N_HOMALT=0;N_HOMREF=10308;FREQ_HET=9.700261907071492e-05;FREQ_HOMALT=0.0;FREQ_HOMREF=0.9999029973809292;N_BI_GENOS=10309;East_Han_AN=6554;East_Han_AC=1;East_Han_AF=0
chr2	56822810	.	A	<INS:ME:LINE1>	.	PASS	SVTYPE=INS:ME:LINE1;END=56822819;SVLEN=6007;AN=20618;AC=3;AF=0.00014550392860607236;N_HET=3;N_HOMALT=0;N_HOMREF=10306;FREQ_HET=0.0002910078572121447;FREQ_HOMALT=0.0;FREQ_HOMREF=0.9997089921427879;N_BI_GENOS=10309;South_Han_AN=1496;South_Han_AC=1;South_Han
chr2	56851630	.	G	<INS:ME:LINE1>	.	PASS	SVTYPE=INS:ME:LINE1;END=56851649;SVLEN=5996;AN=20618;AC=2;AF=9.700261907071492e-05;N_HET=2;N_HOMALT=0;N_HOMREF=10307;FREQ_HET=0.00019400523814142983;FREQ_HOMALT=0.0;FREQ_HOMREF=0.9998059947618586;N_BI_GENOS=10309;Lingnan_Han_AN=1888;Lingnan_Han_AC=1;Lingn
chr2	57008485	.	T	<INS:ME:LINE1>	.	PASS	SVTYPE=INS:ME:LINE1;END=57008487;SVLEN=2679;AN=20618;AC=2;AF=9.700261907071492e-05;N_HET=2;N_HOMALT=0;N_HOMREF=10307;FREQ_HET=0.00019400523814142983;FREQ_HOMALT=0.0;FREQ_HOMREF=0.9998059947618586;N_BI_GENOS=10309;North_Han_AN=2272;North_Han_AC=1;North_Han
chr2	57034398	.	T	<INS:ME:LINE1>	.	PASS	SVTYPE=INS:ME:LINE1;END=57034414;SVLEN=6000;AN=20618;AC=1;AF=4.850130953535746e-05;N_HET=1;N_HOMALT=0;N_HOMREF=10308;FREQ_HET=9.700261907071492e-05;FREQ_HOMALT=0.0;FREQ_HOMREF=0.9999029973809292;N_BI_GENOS=10309;North_Han_AN=2272;North_Han_AC=1;North_Han_
chr2	57073809	.	T	<INS:ME:LINE1>	.	PASS	SVTYPE=INS:ME:LINE1;END=57073810;SVLEN=654;AN=20618;AC=1;AF=4.850130953535746e-05;N_HET=1;N_HOMALT=0;N_HOMREF=10308;FREQ_HET=9.700261907071492e-05;FREQ_HOMALT=0.0;FREQ_HOMREF=0.9999029973809292;N_BI_GENOS=10309;South_Han_AN=1496;South_Han_AC=1;South_Han_A
chr2	57124773	.	T	<INS:ME:LINE1>	.	PASS	SVTYPE=INS:ME:LINE1;END=57124785;SVLEN=541;AN=20618;AC=1;AF=4.850130953535746e-05;N_HET=1;N_HOMALT=0;N_HOMREF=10308;FREQ_HET=9.700261907071492e-05;FREQ_HOMALT=0.0;FREQ_HOMREF=0.9999029973809292;N_BI_GENOS=10309;Manchu_AN=416;Manchu_AC=1;Manchu_AF=0.002403
chr2	57187107	.	A	<INS:ME:LINE1>	.	PASS	SVTYPE=INS:ME:LINE1;END=57187128;SVLEN=493;AN=20618;AC=1;AF=4.850130953535746e-05;N_HET=1;N_HOMALT=0;N_HOMREF=10308;FREQ_HET=9.700261907071492e-05;FREQ_HOMALT=0.0;FREQ_HOMREF=0.9999029973809292;N_BI_GENOS=10309;East_Han_AN=6554;East_Han_AC=1;East_Han_AF=0
chr2	57194091	.	C	<INS:ME:LINE1>	.	PASS	SVTYPE=INS:ME:LINE1;END=57194091;SVLEN=0;AN=20618;AC=1;AF=4.850130953535746e-05;N_HET=1;N_HOMALT=0;N_HOMREF=10308;FREQ_HET=9.700261907071492e-05;FREQ_HOMALT=0.0;FREQ_HOMREF=0.9999029973809292;N_BI_GENOS=10309;Southeast_Han_AN=1908;Southeast_Han_AC=1;South
chr2	57209821	.	C	<INS:ME:LINE1>	.	PASS	SVTYPE=INS:ME:LINE1;END=57209838;SVLEN=719;AN=20618;AC=1;AF=4.850130953535746e-05;N_HET=1;N_HOMALT=0;N_HOMREF=10308;FREQ_HET=9.700261907071492e-05;FREQ_HOMALT=0.0;FREQ_HOMREF=0.9999029973809292;N_BI_GENOS=10309;Southeast_Han_AN=1908;Southeast_Han_AC=1;Sou
chr2	57281374	.	A	<INS:ME:LINE1>	.	PASS	SVTYPE=INS:ME:LINE1;END=57281391;SVLEN=102;AN=20618;AC=204;AF=0.009894267145212922;N_HET=196;N_HOMALT=4;N_HOMREF=10109;FREQ_HET=0.019012513337860124;FREQ_HOMALT=0.00038801047628285966;FREQ_HOMREF=0.980599476185857;N_BI_GENOS=10309;South_Han_AN=1496;South_
chr2	57312523	.	G	<INS:ME:LINE1>	.	PASS	SVTYPE=INS:ME:LINE1;END=57312539;SVLEN=5997;AN=20618;AC=1;AF=4.850130953535746e-05;N_HET=1;N_HOMALT=0;N_HOMREF=10308;FREQ_HET=9.700261907071492e-05;FREQ_HOMALT=0.0;FREQ_HOMREF=0.9999029973809292;N_BI_GENOS=10309;Northwest_Han_AN=1296;Northwest_Han_AC=1;No
chr2	57393552	.	T	<INS:ME:LINE1>	.	PASS	SVTYPE=INS:ME:LINE1;END=57393557;SVLEN=1661;AN=20618;AC=1;AF=4.850130953535746e-05;N_HET=1;N_HOMALT=0;N_HOMREF=10308;FREQ_HET=9.700261907071492e-05;FREQ_HOMALT=0.0;FREQ_HOMREF=0.9999029973809292;N_BI_GENOS=10309;Northwest_Han_AN=1296;Northwest_Han_AC=1;No
chr2	58290458	.	A	<INS:ME:LINE1>	.	PASS	SVTYPE=INS:ME:LINE1;END=58290474;SVLEN=5985;AN=20618;AC=1;AF=4.850130953535746e-05;N_HET=1;N_HOMALT=0;N_HOMREF=10308;FREQ_HET=9.700261907071492e-05;FREQ_HOMALT=0.0;FREQ_HOMREF=0.9999029973809292;N_BI_GENOS=10309;Miao_AN=482;Miao_AC=1;Miao_AF=0.00207468879
chr2	58350758	.	A	<INS:ME:LINE1>	.	PASS	SVTYPE=INS:ME:LINE1;END=58350774;SVLEN=836;AN=20618;AC=1;AF=4.850130953535746e-05;N_HET=1;N_HOMALT=0;N_HOMREF=10308;FREQ_HET=9.700261907071492e-05;FREQ_HOMALT=0.0;FREQ_HOMREF=0.9999029973809292;N_BI_GENOS=10309;Southeast_Han_AN=1908;Southeast_Han_AC=1;Sou
chr2	58411532	.	G	<INS:ME:LINE1>	.	PASS	SVTYPE=INS:ME:LINE1;END=58411547;SVLEN=1734;AN=20618;AC=2;AF=9.700261907071492e-05;N_HET=2;N_HOMALT=0;N_HOMREF=10307;FREQ_HET=0.00019400523814142983;FREQ_HOMALT=0.0;FREQ_HOMREF=0.9998059947618586;N_BI_GENOS=10309;East_Han_AN=6554;East_Han_AC=2;East_Han_AF
chr2	58841822	.	A	<INS:ME:LINE1>	.	PASS	SVTYPE=INS:ME:LINE1;END=58841835;SVLEN=1149;AN=20618;AC=1;AF=4.850130953535746e-05;N_HET=1;N_HOMALT=0;N_HOMREF=10308;FREQ_HET=9.700261907071492e-05;FREQ_HOMALT=0.0;FREQ_HOMREF=0.9999029973809292;N_BI_GENOS=10309;Southeast_Han_AN=1908;Southeast_Han_AC=1;So
chr2	58882076	.	G	<INS:ME:LINE1>	.	PASS	SVTYPE=INS:ME:LINE1;END=58882104;SVLEN=4538;AN=20618;AC=1;AF=4.850130953535746e-05;N_HET=1;N_HOMALT=0;N_HOMREF=10308;FREQ_HET=9.700261907071492e-05;FREQ_HOMALT=0.0;FREQ_HOMREF=0.9999029973809292;N_BI_GENOS=10309;East_Han_AN=6554;East_Han_AC=1;East_Han_AF=
chr2	59134682	.	A	<INS:ME:LINE1>	.	PASS	SVTYPE=INS:ME:LINE1;END=59134694;SVLEN=1049;AN=20618;AC=3;AF=0.00014550392860607236;N_HET=3;N_HOMALT=0;N_HOMREF=10306;FREQ_HET=0.0002910078572121447;FREQ_HOMALT=0.0;FREQ_HOMREF=0.9997089921427879;N_BI_GENOS=10309;East_Han_AN=6554;East_Han_AC=3;East_Han_AF
chr2	59621062	.	G	<INS:ME:LINE1>	.	PASS	SVTYPE=INS:ME:LINE1;END=59621078;SVLEN=6000;AN=20618;AC=8;AF=0.00038801047628285966;N_HET=8;N_HOMALT=0;N_HOMREF=10301;FREQ_HET=0.0007760209525657193;FREQ_HOMALT=0.0;FREQ_HOMREF=0.9992239790474343;N_BI_GENOS=10309;South_Han_AN=1496;South_Han_AC=3;South_Han
chr2	59994284	.	T	<INS:ME:LINE1>	.	PASS	SVTYPE=INS:ME:LINE1;END=59994284;SVLEN=324;AN=20618;AC=1;AF=4.850130953535746e-05;N_HET=1;N_HOMALT=0;N_HOMREF=10308;FREQ_HET=9.700261907071492e-05;FREQ_HOMALT=0.0;FREQ_HOMREF=0.9999029973809292;N_BI_GENOS=10309;Northwest_Han_AN=1296;Northwest_Han_AC=1;Nor
chr2	60944792	.	A	<INS:ME:LINE1>	.	PASS	SVTYPE=INS:ME:LINE1;END=60944811;SVLEN=52;AN=20618;AC=1;AF=4.850130953535746e-05;N_HET=1;N_HOMALT=0;N_HOMREF=10308;FREQ_HET=9.700261907071492e-05;FREQ_HOMALT=0.0;FREQ_HOMREF=0.9999029973809292;N_BI_GENOS=10309;Lingnan_Han_AN=1888;Lingnan_Han_AC=1;Lingnan_
chr2	61111051	.	G	<INS:ME:LINE1>	.	PASS	SVTYPE=INS:ME:LINE1;END=61111052;SVLEN=2047;AN=20618;AC=1;AF=4.850130953535746e-05;N_HET=1;N_HOMALT=0;N_HOMREF=10308;FREQ_HET=9.700261907071492e-05;FREQ_HOMALT=0.0;FREQ_HOMREF=0.9999029973809292;N_BI_GENOS=10309;Lingnan_Han_AN=1888;Lingnan_Han_AC=1;Lingna
chr2	61603209	.	G	<INS:ME:LINE1>	.	PASS	SVTYPE=INS:ME:LINE1;END=61603219;SVLEN=6000;AN=20618;AC=4;AF=0.00019400523814142983;N_HET=4;N_HOMALT=0;N_HOMREF=10305;FREQ_HET=0.00038801047628285966;FREQ_HOMALT=0.0;FREQ_HOMREF=0.9996119895237171;N_BI_GENOS=10309;South_Han_AN=1496;South_Han_AC=1;South_Ha
chr2	61696332	.	T	<INS:ME:LINE1>	.	PASS	SVTYPE=INS:ME:LINE1;END=61696339;SVLEN=1326;AN=20618;AC=1;AF=4.850130953535746e-05;N_HET=1;N_HOMALT=0;N_HOMREF=10308;FREQ_HET=9.700261907071492e-05;FREQ_HOMALT=0.0;FREQ_HOMREF=0.9999029973809292;N_BI_GENOS=10309;Lingnan_Han_AN=1888;Lingnan_Han_AC=1;Lingna
chr2	62886597	.	A	<INS:ME:LINE1>	.	PASS	SVTYPE=INS:ME:LINE1;END=62886610;SVLEN=1613;AN=20618;AC=1;AF=4.850130953535746e-05;N_HET=1;N_HOMALT=0;N_HOMREF=10308;FREQ_HET=9.700261907071492e-05;FREQ_HOMALT=0.0;FREQ_HOMREF=0.9999029973809292;N_BI_GENOS=10309;East_Han_AN=6554;East_Han_AC=1;East_Han_AF=
chr2	62938088	.	A	<INS:ME:LINE1>	.	PASS	SVTYPE=INS:ME:LINE1;END=62938103;SVLEN=5998;AN=20618;AC=135;AF=0.006547676787273257;N_HET=133;N_HOMALT=1;N_HOMREF=10175;FREQ_HET=0.012901348336405083;FREQ_HOMALT=9.700261907071492e-05;FREQ_HOMREF=0.9870016490445243;N_BI_GENOS=10309;South_Han_AN=1496;South
chr2	62965969	.	G	<INS:ME:LINE1>	.	PASS	SVTYPE=INS:ME:LINE1;END=62965984;SVLEN=1029;AN=20618;AC=22;AF=0.001067028809777864;N_HET=20;N_HOMALT=1;N_HOMREF=10288;FREQ_HET=0.0019400523814142982;FREQ_HOMALT=9.700261907071492e-05;FREQ_HOMREF=0.997962944999515;N_BI_GENOS=10309;South_Han_AN=1496;South_H
chr2	63131637	.	A	<INS:ME:LINE1>	.	PASS	SVTYPE=INS:ME:LINE1;END=63131649;SVLEN=6000;AN=20618;AC=3;AF=0.00014550392860607236;N_HET=3;N_HOMALT=0;N_HOMREF=10306;FREQ_HET=0.0002910078572121447;FREQ_HOMALT=0.0;FREQ_HOMREF=0.9997089921427879;N_BI_GENOS=10309;South_Han_AN=1496;South_Han_AC=1;South_Han
chr2	63218512	.	T	<INS:ME:LINE1>	.	PASS	SVTYPE=INS:ME:LINE1;END=63218518;SVLEN=459;AN=20618;AC=1;AF=4.850130953535746e-05;N_HET=1;N_HOMALT=0;N_HOMREF=10308;FREQ_HET=9.700261907071492e-05;FREQ_HOMALT=0.0;FREQ_HOMREF=0.9999029973809292;N_BI_GENOS=10309;East_Han_AN=6554;East_Han_AC=1;East_Han_AF=0
chr2	63372578	.	T	<INS:ME:LINE1>	.	PASS	SVTYPE=INS:ME:LINE1;END=63372592;SVLEN=1053;AN=20618;AC=1;AF=4.850130953535746e-05;N_HET=1;N_HOMALT=0;N_HOMREF=10308;FREQ_HET=9.700261907071492e-05;FREQ_HOMALT=0.0;FREQ_HOMREF=0.9999029973809292;N_BI_GENOS=10309;East_Han_AN=6554;East_Han_AC=1;East_Han_AF=
chr2	63441654	.	G	<INS:ME:LINE1>	.	PASS	SVTYPE=INS:ME:LINE1;END=63441672;SVLEN=362;AN=20618;AC=9;AF=0.0004365117858182171;N_HET=9;N_HOMALT=0;N_HOMREF=10300;FREQ_HET=0.0008730235716364342;FREQ_HOMALT=0.0;FREQ_HOMREF=0.9991269764283636;N_BI_GENOS=10309;South_Han_AN=1496;South_Han_AC=1;South_Han_A
chr2	63455951	.	G	<INS:ME:LINE1>	.	PASS	SVTYPE=INS:ME:LINE1;END=63455966;SVLEN=5998;AN=20618;AC=1;AF=4.850130953535746e-05;N_HET=1;N_HOMALT=0;N_HOMREF=10308;FREQ_HET=9.700261907071492e-05;FREQ_HOMALT=0.0;FREQ_HOMREF=0.9999029973809292;N_BI_GENOS=10309;South_Han_AN=1496;South_Han_AC=1;South_Han_
chr2	63492479	.	T	<INS:ME:LINE1>	.	PASS	SVTYPE=INS:ME:LINE1;END=63492479;SVLEN=554;AN=20618;AC=1;AF=4.850130953535746e-05;N_HET=1;N_HOMALT=0;N_HOMREF=10308;FREQ_HET=9.700261907071492e-05;FREQ_HOMALT=0.0;FREQ_HOMREF=0.9999029973809292;N_BI_GENOS=10309;Lingnan_Han_AN=1888;Lingnan_Han_AC=1;Lingnan
chr2	63694127	.	T	<INS:ME:LINE1>	.	PASS	SVTYPE=INS:ME:LINE1;END=63694144;SVLEN=835;AN=20618;AC=1;AF=4.850130953535746e-05;N_HET=1;N_HOMALT=0;N_HOMREF=10308;FREQ_HET=9.700261907071492e-05;FREQ_HOMALT=0.0;FREQ_HOMREF=0.9999029973809292;N_BI_GENOS=10309;South_Han_AN=1496;South_Han_AC=1;South_Han_A
chr2	64476689	.	T	<INS:ME:LINE1>	.	PASS	SVTYPE=INS:ME:LINE1;END=64476706;SVLEN=6006;AN=20618;AC=1;AF=4.850130953535746e-05;N_HET=1;N_HOMALT=0;N_HOMREF=10308;FREQ_HET=9.700261907071492e-05;FREQ_HOMALT=0.0;FREQ_HOMREF=0.9999029973809292;N_BI_GENOS=10309;Northwest_Han_AN=1296;Northwest_Han_AC=1;No
chr2	64720545	.	A	<INS:ME:LINE1>	.	PASS	SVTYPE=INS:ME:LINE1;END=64720558;SVLEN=5997;AN=20618;AC=7;AF=0.0003395091667475022;N_HET=3;N_HOMALT=2;N_HOMREF=10304;FREQ_HET=0.0002910078572121447;FREQ_HOMALT=0.00019400523814142983;FREQ_HOMREF=0.9995149869046465;N_BI_GENOS=10309;North_Han_AN=2272;North_
chr2	64970184	.	C	<INS:ME:LINE1>	.	PASS	SVTYPE=INS:ME:LINE1;END=64970200;SVLEN=4956;AN=20618;AC=1;AF=4.850130953535746e-05;N_HET=1;N_HOMALT=0;N_HOMREF=10308;FREQ_HET=9.700261907071492e-05;FREQ_HOMALT=0.0;FREQ_HOMREF=0.9999029973809292;N_BI_GENOS=10309;South_Han_AN=1496;South_Han_AC=1;South_Han_
chr2	64997137	.	A	<INS:ME:LINE1>	.	PASS	SVTYPE=INS:ME:LINE1;END=64997154;SVLEN=1063;AN=20618;AC=16;AF=0.0007760209525657193;N_HET=16;N_HOMALT=0;N_HOMREF=10293;FREQ_HET=0.0015520419051314386;FREQ_HOMALT=0.0;FREQ_HOMREF=0.9984479580948685;N_BI_GENOS=10309;South_Han_AN=1496;South_Han_AC=3;South_Ha
chr2	65283300	.	A	<INS:ME:LINE1>	.	PASS	SVTYPE=INS:ME:LINE1;END=65283302;SVLEN=697;AN=20618;AC=1;AF=4.850130953535746e-05;N_HET=1;N_HOMALT=0;N_HOMREF=10308;FREQ_HET=9.700261907071492e-05;FREQ_HOMALT=0.0;FREQ_HOMREF=0.9999029973809292;N_BI_GENOS=10309;East_Han_AN=6554;East_Han_AC=1;East_Han_AF=0
chr2	65357999	.	A	<INS:ME:LINE1>	.	PASS	SVTYPE=INS:ME:LINE1;END=65357999;SVLEN=0;AN=20618;AC=1;AF=4.850130953535746e-05;N_HET=1;N_HOMALT=0;N_HOMREF=10308;FREQ_HET=9.700261907071492e-05;FREQ_HOMALT=0.0;FREQ_HOMREF=0.9999029973809292;N_BI_GENOS=10309;Tibetan_AN=414;Tibetan_AC=1;Tibetan_AF=0.00241
chr2	65358892	.	T	<INS:ME:LINE1>	.	PASS	SVTYPE=INS:ME:LINE1;END=65358907;SVLEN=302;AN=20618;AC=70;AF=0.0033950916674750217;N_HET=70;N_HOMALT=0;N_HOMREF=10239;FREQ_HET=0.006790183334950043;FREQ_HOMALT=0.0;FREQ_HOMREF=0.9932098166650499;N_BI_GENOS=10309;South_Han_AN=1496;South_Han_AC=7;South_Han_
chr2	65528540	.	A	<INS:ME:LINE1>	.	PASS	SVTYPE=INS:ME:LINE1;END=65528556;SVLEN=794;AN=20618;AC=1;AF=4.850130953535746e-05;N_HET=1;N_HOMALT=0;N_HOMREF=10308;FREQ_HET=9.700261907071492e-05;FREQ_HOMALT=0.0;FREQ_HOMREF=0.9999029973809292;N_BI_GENOS=10309;Central_Han_AN=2128;Central_Han_AC=1;Central
chr2	65976155	.	T	<INS:ME:LINE1>	.	PASS	SVTYPE=INS:ME:LINE1;END=65976201;SVLEN=5383;AN=20618;AC=1;AF=4.850130953535746e-05;N_HET=1;N_HOMALT=0;N_HOMREF=10308;FREQ_HET=9.700261907071492e-05;FREQ_HOMALT=0.0;FREQ_HOMREF=0.9999029973809292;N_BI_GENOS=10309;Lingnan_Han_AN=1888;Lingnan_Han_AC=1;Lingna
chr2	66070842	.	A	<INS:ME:LINE1>	.	PASS	SVTYPE=INS:ME:LINE1;END=66070861;SVLEN=498;AN=20618;AC=1;AF=4.850130953535746e-05;N_HET=1;N_HOMALT=0;N_HOMREF=10308;FREQ_HET=9.700261907071492e-05;FREQ_HOMALT=0.0;FREQ_HOMREF=0.9999029973809292;N_BI_GENOS=10309;Manchu_AN=416;Manchu_AC=1;Manchu_AF=0.002403
chr2	66115208	.	A	<INS:ME:LINE1>	.	PASS	SVTYPE=INS:ME:LINE1;END=66115223;SVLEN=6019;AN=20618;AC=1;AF=4.850130953535746e-05;N_HET=1;N_HOMALT=0;N_HOMREF=10308;FREQ_HET=9.700261907071492e-05;FREQ_HOMALT=0.0;FREQ_HOMREF=0.9999029973809292;N_BI_GENOS=10309;Lingnan_Han_AN=1888;Lingnan_Han_AC=1;Lingna
chr2	66124028	.	A	<INS:ME:LINE1>	.	PASS	SVTYPE=INS:ME:LINE1;END=66124040;SVLEN=6000;AN=20618;AC=13;AF=0.0006305170239596469;N_HET=11;N_HOMALT=1;N_HOMREF=10297;FREQ_HET=0.001067028809777864;FREQ_HOMALT=9.700261907071492e-05;FREQ_HOMREF=0.9988359685711514;N_BI_GENOS=10309;South_Han_AN=1496;South_
chr2	66245667	.	G	<INS:ME:LINE1>	.	PASS	SVTYPE=INS:ME:LINE1;END=66245667;SVLEN=0;AN=20618;AC=5;AF=0.00024250654767678728;N_HET=3;N_HOMALT=1;N_HOMREF=10305;FREQ_HET=0.0002910078572121447;FREQ_HOMALT=9.700261907071492e-05;FREQ_HOMREF=0.9996119895237171;N_BI_GENOS=10309;North_Han_AN=2272;North_Han
chr2	66408740	.	A	<INS:ME:LINE1>	.	PASS	SVTYPE=INS:ME:LINE1;END=66408757;SVLEN=1143;AN=20618;AC=15;AF=0.0007275196430303619;N_HET=15;N_HOMALT=0;N_HOMREF=10294;FREQ_HET=0.0014550392860607237;FREQ_HOMALT=0.0;FREQ_HOMREF=0.9985449607139393;N_BI_GENOS=10309;Northwest_Han_AN=1296;Northwest_Han_AC=4;
chr2	66431859	.	A	<INS:ME:LINE1>	.	PASS	SVTYPE=INS:ME:LINE1;END=66431859;SVLEN=13;AN=20618;AC=1;AF=4.850130953535746e-05;N_HET=1;N_HOMALT=0;N_HOMREF=10308;FREQ_HET=9.700261907071492e-05;FREQ_HOMALT=0.0;FREQ_HOMREF=0.9999029973809292;N_BI_GENOS=10309;Northwest_Han_AN=1296;Northwest_Han_AC=1;Nort
chr2	66641002	.	T	<INS:ME:LINE1>	.	PASS	SVTYPE=INS:ME:LINE1;END=66641002;SVLEN=261;AN=20618;AC=2;AF=9.700261907071492e-05;N_HET=0;N_HOMALT=1;N_HOMREF=10308;FREQ_HET=0.0;FREQ_HOMALT=9.700261907071492e-05;FREQ_HOMREF=0.9999029973809292;N_BI_GENOS=10309;North_Han_AN=2272;North_Han_AC=2;North_Han_A
chr2	66714947	.	A	<INS:ME:LINE1>	.	PASS	SVTYPE=INS:ME:LINE1;END=66714962;SVLEN=6032;AN=20618;AC=2;AF=9.700261907071492e-05;N_HET=2;N_HOMALT=0;N_HOMREF=10307;FREQ_HET=0.00019400523814142983;FREQ_HOMALT=0.0;FREQ_HOMREF=0.9998059947618586;N_BI_GENOS=10309;North_Han_AN=2272;North_Han_AC=1;North_Han
chr2	66738827	.	G	<INS:ME:LINE1>	.	PASS	SVTYPE=INS:ME:LINE1;END=66738862;SVLEN=758;AN=20618;AC=1;AF=4.850130953535746e-05;N_HET=1;N_HOMALT=0;N_HOMREF=10308;FREQ_HET=9.700261907071492e-05;FREQ_HOMALT=0.0;FREQ_HOMREF=0.9999029973809292;N_BI_GENOS=10309;East_Han_AN=6554;East_Han_AC=1;East_Han_AF=0
chr2	66753964	.	A	<INS:ME:LINE1>	.	PASS	SVTYPE=INS:ME:LINE1;END=66753984;SVLEN=1328;AN=20618;AC=9;AF=0.0004365117858182171;N_HET=7;N_HOMALT=1;N_HOMREF=10301;FREQ_HET=0.0006790183334950044;FREQ_HOMALT=9.700261907071492e-05;FREQ_HOMREF=0.9992239790474343;N_BI_GENOS=10309;North_Han_AN=2272;North_H
chr2	66865107	.	T	<INS:ME:LINE1>	.	PASS	SVTYPE=INS:ME:LINE1;END=66865112;SVLEN=1057;AN=20618;AC=1;AF=4.850130953535746e-05;N_HET=1;N_HOMALT=0;N_HOMREF=10308;FREQ_HET=9.700261907071492e-05;FREQ_HOMALT=0.0;FREQ_HOMREF=0.9999029973809292;N_BI_GENOS=10309;Lingnan_Han_AN=1888;Lingnan_Han_AC=1;Lingna
chr2	67183886	.	A	<INS:ME:LINE1>	.	PASS	SVTYPE=INS:ME:LINE1;END=67183902;SVLEN=1267;AN=20618;AC=6;AF=0.0002910078572121447;N_HET=4;N_HOMALT=1;N_HOMREF=10304;FREQ_HET=0.00038801047628285966;FREQ_HOMALT=9.700261907071492e-05;FREQ_HOMREF=0.9995149869046465;N_BI_GENOS=10309;Northwest_Han_AN=1296;No

chr2	67979590	.	A	<INS:ME:LINE1>	.	PASS	SVTYPE=INS:ME:LINE1;END=67979606;SVLEN=5997;AN=20618;AC=2;AF=9.700261907071492e-05;N_HET=2;N_HOMALT=0;N_HOMREF=10307;FREQ_HET=0.0001940052381414283;FREQ_HOMALT=0.0;FREQ_HOMREF=0.9998059947618586;N_BI_GENOS=10309;North_Han_AN=2272;North_Han_AC=1;North_Han
chr2	68341490	.	C	<INS:ME:LINE1>	.	PASS	SVTYPE=INS:ME:LINE1;END=68341504;SVLEN=938;AN=20618;AC=1;AF=4.850130953535746e-05;N_HET=1;N_HOMALT=0;N_HOMREF=10308;FREQ_HET=9.700261907071492e-05;FREQ_HOMALT=0.0;FREQ_HOMREF=0.9999029973809292;N_BI_GENOS=10309;Northwest_Han_AN=1296;Northwest_Han_AC=1;Nor
chr2	68544454	.	A	<INS:ME:LINE1>	.	PASS	SVTYPE=INS:ME:LINE1;END=68544466;SVLEN=2929;AN=20618;AC=1;AF=4.850130953535746e-05;N_HET=1;N_HOMALT=0;N_HOMREF=10308;FREQ_HET=9.700261907071492e-05;FREQ_HOMALT=0.0;FREQ_HOMREF=0.9999029973809292;N_BI_GENOS=10309;Southeast_Han_AN=1908;Southeast_Han_AC=1;So
chr2	68557613	.	A	<INS:ME:LINE1>	.	PASS	SVTYPE=INS:ME:LINE1;END=68557630;SVLEN=6015;AN=20618;AC=1;AF=4.850130953535746e-05;N_HET=1;N_HOMALT=0;N_HOMREF=10308;FREQ_HET=9.700261907071492e-05;FREQ_HOMALT=0.0;FREQ_HOMREF=0.9999029973809292;N_BI_GENOS=10309;South_Han_AN=1496;South_Han_AC=1;South_Han_
chr2	68793634	.	T	<INS:ME:LINE1>	.	PASS	SVTYPE=INS:ME:LINE1;END=68793634;SVLEN=1590;AN=20618;AC=1;AF=4.850130953535746e-05;N_HET=1;N_HOMALT=0;N_HOMREF=10308;FREQ_HET=9.700261907071492e-05;FREQ_HOMALT=0.0;FREQ_HOMREF=0.9999029973809292;N_BI_GENOS=10309;East_Han_AN=6554;East_Han_AC=1;East_Han_AF=
chr2	69817476	.	A	<INS:ME:LINE1>	.	PASS	SVTYPE=INS:ME:LINE1;END=69817492;SVLEN=2418;AN=20618;AC=1;AF=4.850130953535746e-05;N_HET=1;N_HOMALT=0;N_HOMREF=10308;FREQ_HET=9.700261907071492e-05;FREQ_HOMALT=0.0;FREQ_HOMREF=0.9999029973809292;N_BI_GENOS=10309;Lingnan_Han_AN=1888;Lingnan_Han_AC=1;Lingna
chr2	69833248	.	A	<INS:ME:LINE1>	.	PASS	SVTYPE=INS:ME:LINE1;END=69833265;SVLEN=3861;AN=20618;AC=61;AF=0.0029585798816568047;N_HET=59;N_HOMALT=1;N_HOMREF=10249;FREQ_HET=0.0057231545251721794;FREQ_HOMALT=9.700261907071492e-05;FREQ_HOMREF=0.9941798428557571;N_BI_GENOS=10309;South_Han_AN=1496;South
chr2	70623207	.	T	<INS:ME:LINE1>	.	PASS	SVTYPE=INS:ME:LINE1;END=70623207;SVLEN=0;AN=20618;AC=1;AF=4.850130953535746e-05;N_HET=1;N_HOMALT=0;N_HOMREF=10308;FREQ_HET=9.700261907071492e-05;FREQ_HOMALT=0.0;FREQ_HOMREF=0.9999029973809292;N_BI_GENOS=10309;Central_Han_AN=2128;Central_Han_AC=1;Central_H
chr2	71086528	.	G	<INS:ME:LINE1>	.	PASS	SVTYPE=INS:ME:LINE1;END=71086528;SVLEN=0;AN=20618;AC=47;AF=0.0022795615481618005;N_HET=1;N_HOMALT=23;N_HOMREF=10285;FREQ_HET=9.700261907071492e-05;FREQ_HOMALT=0.002231060238626443;FREQ_HOMREF=0.9976719371423028;N_BI_GENOS=10309;Northwest_Han_AN=1296;North
chr2	71086597	.	T	<INS:ME:LINE1>	.	PASS	SVTYPE=INS:ME:LINE1;END=71086597;SVLEN=0;AN=20618;AC=2;AF=9.700261907071492e-05;N_HET=0;N_HOMALT=1;N_HOMREF=10308;FREQ_HET=0.0;FREQ_HOMALT=9.700261907071492e-05;FREQ_HOMREF=0.9999029973809292;N_BI_GENOS=10309;North_Han_AN=2272;North_Han_AC=2;North_Han_AF=
chr2	71956475	.	A	<INS:ME:LINE1>	.	PASS	SVTYPE=INS:ME:LINE1;END=71956493;SVLEN=5999;AN=20618;AC=2;AF=9.700261907071492e-05;N_HET=2;N_HOMALT=0;N_HOMREF=10307;FREQ_HET=0.0001940052381414283;FREQ_HOMALT=0.0;FREQ_HOMREF=0.9998059947618586;N_BI_GENOS=10309;Central_Han_AN=2128;Central_Han_AC=2;Centr
chr2	71962993	.	G	<INS:ME:LINE1>	.	PASS	SVTYPE=INS:ME:LINE1;END=71962999;SVLEN=271;AN=20618;AC=12;AF=0.0005820157144242894;N_HET=10;N_HOMALT=1;N_HOMREF=10298;FREQ_HET=0.0009700261907071491;FREQ_HOMALT=9.700261907071492e-05;FREQ_HOMREF=0.9989329711902222;N_BI_GENOS=10309;Lingnan_Han_AN=1888;Ling
chr2	72038984	.	A	<INS:ME:LINE1>	.	PASS	SVTYPE=INS:ME:LINE1;END=72038997;SVLEN=3667;AN=20618;AC=1;AF=4.850130953535746e-05;N_HET=1;N_HOMALT=0;N_HOMREF=10308;FREQ_HET=9.700261907071492e-05;FREQ_HOMALT=0.0;FREQ_HOMREF=0.9999029973809292;N_BI_GENOS=10309;Lingnan_Han_AN=1888;Lingnan_Han_AC=1;Lingna
chr2	72049974	.	A	<INS:ME:LINE1>	.	PASS	SVTYPE=INS:ME:LINE1;END=72049974;SVLEN=65;AN=20618;AC=5;AF=0.00024250654767678728;N_HET=5;N_HOMALT=0;N_HOMREF=10304;FREQ_HET=0.0004850130953535357455;FREQ_HOMALT=0.0;FREQ_HOMREF=0.9995149869046465;N_BI_GENOS=10309;South_Han_AN=1496;South_Han_AC=1;South_Han_
chr2	72308025	.	T	<INS:ME:LINE1>	.	PASS	SVTYPE=INS:ME:LINE1;END=72308039;SVLEN=6000;AN=20618;AC=9;AF=0.0004365117858182171;N_HET=9;N_HOMALT=0;N_HOMREF=10300;FREQ_HET=0.0008730235716364342;FREQ_HOMALT=0.0;FREQ_HOMREF=0.9991269764283636;N_BI_GENOS=10309;South_Han_AN=1496;South_Han_AC=1;South_Han_
chr2	72351397	.	T	<INS:ME:LINE1>	.	PASS	SVTYPE=INS:ME:LINE1;END=72351412;SVLEN=1471;AN=20618;AC=2;AF=9.700261907071492e-05;N_HET=2;N_HOMALT=0;N_HOMREF=10307;FREQ_HET=0.0001940052381414283;FREQ_HOMALT=0.0;FREQ_HOMREF=0.9998059947618586;N_BI_GENOS=10309;East_Han_AN=6554;East_Han_AC=2;East_Han_AF
chr2	72400645	.	T	<INS:ME:LINE1>	.	PASS	SVTYPE=INS:ME:LINE1;END=72400676;SVLEN=824;AN=20618;AC=1;AF=4.850130953535746e-05;N_HET=1;N_HOMALT=0;N_HOMREF=10308;FREQ_HET=9.700261907071492e-05;FREQ_HOMALT=0.0;FREQ_HOMREF=0.9999029973809292;N_BI_GENOS=10309;East_Han_AN=6554;East_Han_AC=1;East_Han_AF=0
chr2	72622205	.	A	<INS:ME:LINE1>	.	PASS	SVTYPE=INS:ME:LINE1;END=72622214;SVLEN=62;AN=20618;AC=3;AF=0.00014550392860607236;N_HET=3;N_HOMALT=0;N_HOMREF=10306;FREQ_HET=0.0002910078572121447;FREQ_HOMALT=0.0;FREQ_HOMREF=0.9997089921427879;N_BI_GENOS=10309;South_Han_AN=1496;South_Han_AC=1;South_Han_A
chr2	73416528	.	A	<INS:ME:LINE1>	.	PASS	SVTYPE=INS:ME:LINE1;END=73416537;SVLEN=1708;AN=20618;AC=1;AF=4.850130953535746e-05;N_HET=1;N_HOMALT=0;N_HOMREF=10308;FREQ_HET=9.700261907071492e-05;FREQ_HOMALT=0.0;FREQ_HOMREF=0.9999029973809292;N_BI_GENOS=10309;South_Han_AN=1496;South_Han_AC=1;South_Han_
chr2	73663623	.	A	<INS:ME:LINE1>	.	PASS	SVTYPE=INS:ME:LINE1;END=73663623;SVLEN=16;AN=20618;AC=1;AF=4.850130953535746e-05;N_HET=1;N_HOMALT=0;N_HOMREF=10308;FREQ_HET=9.700261907071492e-05;FREQ_HOMALT=0.0;FREQ_HOMREF=0.9999029973809292;N_BI_GENOS=10309;South_Han_AN=1496;South_Han_AC=1;South_Han_AF
chr2	73860038	.	T	<INS:ME:LINE1>	.	PASS	SVTYPE=INS:ME:LINE1;END=73860042;SVLEN=1121;AN=20618;AC=3;AF=0.00014550392860607236;N_HET=3;N_HOMALT=0;N_HOMREF=10306;FREQ_HET=0.0002910078572121447;FREQ_HOMALT=0.0;FREQ_HOMREF=0.9997089921427879;N_BI_GENOS=10309;Northwest_Han_AN=1296;Northwest_Han_AC=1;N
chr2	73937356	.	A	<INS:ME:LINE1>	.	PASS	SVTYPE=INS:ME:LINE1;END=73937370;SVLEN=1032;AN=20618;AC=1;AF=4.850130953535746e-05;N_HET=1;N_HOMALT=0;N_HOMREF=10308;FREQ_HET=9.700261907071492e-05;FREQ_HOMALT=0.0;FREQ_HOMREF=0.9999029973809292;N_BI_GENOS=10309;East_Han_AN=6554;East_Han_AC=1;East_Han_AF=
chr2	74692835	.	A	<INS:ME:LINE1>	.	PASS	SVTYPE=INS:ME:LINE1;END=74692840;SVLEN=565;AN=20618;AC=1;AF=4.850130953535746e-05;N_HET=1;N_HOMALT=0;N_HOMREF=10308;FREQ_HET=9.700261907071492e-05;FREQ_HOMALT=0.0;FREQ_HOMREF=0.9999029973809292;N_BI_GENOS=10309;Zhuang_AN=444;Zhuang_AC=1;Zhuang_AF=0.002252
chr2	75325663	.	A	<INS:ME:LINE1>	.	PASS	SVTYPE=INS:ME:LINE1;END=75325680;SVLEN=3066;AN=20618;AC=1;AF=4.850130953535746e-05;N_HET=1;N_HOMALT=0;N_HOMREF=10308;FREQ_HET=9.700261907071492e-05;FREQ_HOMALT=0.0;FREQ_HOMREF=0.9999029973809292;N_BI_GENOS=10309;Lingnan_Han_AN=1888;Lingnan_Han_AC=1;Lingna
chr2	75691813	.	C	<INS:ME:LINE1>	.	PASS	SVTYPE=INS:ME:LINE1;END=75691829;SVLEN=1056;AN=20618;AC=2;AF=9.700261907071492e-05;N_HET=2;N_HOMALT=0;N_HOMREF=10307;FREQ_HET=0.0001940052381414283;FREQ_HOMALT=0.0;FREQ_HOMREF=0.9998059947618586;N_BI_GENOS=10309;North_Han_AN=2272;North_Han_AC=2;North_Han
chr2	75720666	.	A	<INS:ME:LINE1>	.	PASS	SVTYPE=INS:ME:LINE1;END=75720681;SVLEN=6075;AN=20618;AC=1;AF=4.850130953535746e-05;N_HET=1;N_HOMALT=0;N_HOMREF=10308;FREQ_HET=9.700261907071492e-05;FREQ_HOMALT=0.0;FREQ_HOMREF=0.9999029973809292;N_BI_GENOS=10309;Tibetan_AN=414;Tibetan_AC=1;Tibetan_AF=0.00
chr2	75747937	.	A	<INS:ME:LINE1>	.	PASS	SVTYPE=INS:ME:LINE1;END=75747950;SVLEN=574;AN=20618;AC=1;AF=4.850130953535746e-05;N_HET=1;N_HOMALT=0;N_HOMREF=10308;FREQ_HET=9.700261907071492e-05;FREQ_HOMALT=0.0;FREQ_HOMREF=0.9999029973809292;N_BI_GENOS=10309;Central_Han_AN=2128;Central_Han_AC=1;Central
chr2	75939813	.	A	<INS:ME:LINE1>	.	PASS	SVTYPE=INS:ME:LINE1;END=75939826;SVLEN=6018;AN=20618;AC=2;AF=9.700261907071492e-05;N_HET=2;N_HOMALT=0;N_HOMREF=10307;FREQ_HET=0.0001940052381414283;FREQ_HOMALT=0.0;FREQ_HOMREF=0.9998059947618586;N_BI_GENOS=10309;Central_Han_AN=2128;Central_Han_AC=1;Centr
chr2	76179190	.	T	<INS:ME:LINE1>	.	PASS	SVTYPE=INS:ME:LINE1;END=76179190;SVLEN=1611;AN=20618;AC=1;AF=4.850130953535746e-05;N_HET=1;N_HOMALT=0;N_HOMREF=10308;FREQ_HET=9.700261907071492e-05;FREQ_HOMALT=0.0;FREQ_HOMREF=0.9999029973809292;N_BI_GENOS=10309;East_Han_AN=6554;East_Han_AC=1;East_Han_AF=
chr2	76523451	.	A	<INS:ME:LINE1>	.	PASS	SVTYPE=INS:ME:LINE1;END=76523453;SVLEN=353;AN=20618;AC=1;AF=4.850130953535746e-05;N_HET=1;N_HOMALT=0;N_HOMREF=10308;FREQ_HET=9.700261907071492e-05;FREQ_HOMALT=0.0;FREQ_HOMREF=0.9999029973809292;N_BI_GENOS=10309;Southeast_Han_AN=1908;Southeast_Han_AC=1;Sou
chr2	76577895	.	A	<INS:ME:LINE1>	.	PASS	SVTYPE=INS:ME:LINE1;END=76577895;SVLEN=0;AN=20618;AC=2;AF=9.700261907071492e-05;N_HET=0;N_HOMALT=1;N_HOMREF=10308;FREQ_HET=0.0;FREQ_HOMALT=9.700261907071492e-05;FREQ_HOMREF=0.9999029973809292;N_BI_GENOS=10309;East_Han_AN=6554;East_Han_AC=2;East_Han_AF=0.0
chr2	76583423	.	G	<INS:ME:LINE1>	.	PASS	SVTYPE=INS:ME:LINE1;END=76583423;SVLEN=186;AN=20618;AC=1;AF=4.850130953535746e-05;N_HET=1;N_HOMALT=0;N_HOMREF=10308;FREQ_HET=9.700261907071492e-05;FREQ_HOMALT=0.0;FREQ_HOMREF=0.9999029973809292;N_BI_GENOS=10309;East_Han_AN=6554;East_Han_AC=1;East_Han_AF=0
chr2	76745333	.	G	<INS:ME:LINE1>	.	PASS	SVTYPE=INS:ME:LINE1;END=76745344;SVLEN=5938;AN=20618;AC=1;AF=4.850130953535746e-05;N_HET=1;N_HOMALT=0;N_HOMREF=10308;FREQ_HET=9.700261907071492e-05;FREQ_HOMALT=0.0;FREQ_HOMREF=0.9999029973809292;N_BI_GENOS=10309;North_Han_AN=2272;North_Han_AC=1;North_Han_
chr2	76887926	.	A	<INS:ME:LINE1>	.	PASS	SVTYPE=INS:ME:LINE1;END=76887938;SVLEN=630;AN=20618;AC=1;AF=4.850130953535746e-05;N_HET=1;N_HOMALT=0;N_HOMREF=10308;FREQ_HET=9.700261907071492e-05;FREQ_HOMALT=0.0;FREQ_HOMREF=0.9999029973809292;N_BI_GENOS=10309;East_Han_AN=6554;East_Han_AC=1;East_Han_AF=0
chr2	77093892	.	C	<INS:ME:LINE1>	.	PASS	SVTYPE=INS:ME:LINE1;END=77093892;SVLEN=0;AN=20618;AC=1;AF=4.850130953535746e-05;N_HET=1;N_HOMALT=0;N_HOMREF=10308;FREQ_HET=9.700261907071492e-05;FREQ_HOMALT=0.0;FREQ_HOMREF=0.9999029973809292;N_BI_GENOS=10309;Manchu_AN=416;Manchu_AC=1;Manchu_AF=0.00240384
chr2	77130252	.	T	<INS:ME:LINE1>	.	PASS	SVTYPE=INS:ME:LINE1;END=77130253;SVLEN=474;AN=20618;AC=1;AF=4.850130953535746e-05;N_HET=1;N_HOMALT=0;N_HOMREF=10308;FREQ_HET=9.700261907071492e-05;FREQ_HOMALT=0.0;FREQ_HOMREF=0.9999029973809292;N_BI_GENOS=10309;North_Han_AN=2272;North_Han_AC=1;North_Han_A
chr2	77389435	.	A	<INS:ME:LINE1>	.	PASS	SVTYPE=INS:ME:LINE1;END=77389444;SVLEN=114;AN=20618;AC=1;AF=4.850130953535746e-05;N_HET=1;N_HOMALT=0;N_HOMREF=10308;FREQ_HET=9.700261907071492e-05;FREQ_HOMALT=0.0;FREQ_HOMREF=0.9999029973809292;N_BI_GENOS=10309;Southeast_Han_AN=1908;Southeast_Han_AC=1;Sou
chr2	77429265	.	A	<INS:ME:LINE1>	.	PASS	SVTYPE=INS:ME:LINE1;END=77429281;SVLEN=1881;AN=20618;AC=1;AF=4.850130953535746e-05;N_HET=1;N_HOMALT=0;N_HOMREF=10308;FREQ_HET=9.700261907071492e-05;FREQ_HOMALT=0.0;FREQ_HOMREF=0.9999029973809292;N_BI_GENOS=10309;Lingnan_Han_AN=1888;Lingnan_Han_AC=1;Lingna
chr2	77454881	.	A	<INS:ME:LINE1>	.	PASS	SVTYPE=INS:ME:LINE1;END=77454881;SVLEN=5927;AN=20618;AC=1;AF=4.850130953535746e-05;N_HET=1;N_HOMALT=0;N_HOMREF=10308;FREQ_HET=9.700261907071492e-05;FREQ_HOMALT=0.0;FREQ_HOMREF=0.9999029973809292;N_BI_GENOS=10309;Southeast_Han_AN=1908;Southeast_Han_AC=1;So
chr2	77460630	.	A	<INS:ME:LINE1>	.	PASS	SVTYPE=INS:ME:LINE1;END=77460646;SVLEN=5398;AN=20618;AC=1;AF=4.850130953535746e-05;N_HET=1;N_HOMALT=0;N_HOMREF=10308;FREQ_HET=9.700261907071492e-05;FREQ_HOMALT=0.0;FREQ_HOMREF=0.9999029973809292;N_BI_GENOS=10309;Central_Han_AN=2128;Central_Han_AC=1;Centra
chr2	77641016	.	A	<INS:ME:LINE1>	.	PASS	SVTYPE=INS:ME:LINE1;END=77641023;SVLEN=490;AN=20618;AC=3;AF=0.00014550392860607236;N_HET=3;N_HOMALT=0;N_HOMREF=10306;FREQ_HET=0.0002910078572121447;FREQ_HOMALT=0.0;FREQ_HOMREF=0.9997089921427879;N_BI_GENOS=10309;East_Han_AN=6554;East_Han_AC=1;East_Han_AF=
chr2	77983357	.	A	<INS:ME:LINE1>	.	PASS	SVTYPE=INS:ME:LINE1;END=77983384;SVLEN=967;AN=20618;AC=2;AF=9.700261907071492e-05;N_HET=0;N_HOMALT=1;N_HOMREF=10308;FREQ_HET=0.0;FREQ_HOMALT=9.700261907071492e-05;FREQ_HOMREF=0.9999029973809292;N_BI_GENOS=10309;East_Han_AN=6554;East_Han_AC=2;East_Han_AF=0
chr2	78022149	.	A	<INS:ME:LINE1>	.	PASS	SVTYPE=INS:ME:LINE1;END=78022149;SVLEN=880;AN=20618;AC=1;AF=4.850130953535746e-05;N_HET=1;N_HOMALT=0;N_HOMREF=10308;FREQ_HET=9.700261907071492e-05;FREQ_HOMALT=0.0;FREQ_HOMREF=0.9999029973809292;N_BI_GENOS=10309;East_Han_AN=6554;East_Han_AC=1;East_Han_AF=0
chr2	78207904	.	A	<INS:ME:LINE1>	.	PASS	SVTYPE=INS:ME:LINE1;END=78207904;SVLEN=709;AN=20618;AC=1;AF=4.850130953535746e-05;N_HET=1;N_HOMALT=0;N_HOMREF=10308;FREQ_HET=9.700261907071492e-05;FREQ_HOMALT=0.0;FREQ_HOMREF=0.9999029973809292;N_BI_GENOS=10309;East_Han_AN=6554;East_Han_AC=1;East_Han_AF=0
chr2	78464437	.	A	<INS:ME:LINE1>	.	PASS	SVTYPE=INS:ME:LINE1;END=78464453;SVLEN=563;AN=20618;AC=4;AF=0.0001940052381414283;N_HET=4;N_HOMALT=0;N_HOMREF=10305;FREQ_HET=0.0003880104762828566;FREQ_HOMALT=0.0;FREQ_HOMREF=0.9996119895237171;N_BI_GENOS=10309;East_Han_AN=6554;East_Han_AC=2;East_Han_AF
chr2	78569371	.	G	<INS:ME:LINE1>	.	PASS	SVTYPE=INS:ME:LINE1;END=78569384;SVLEN=109;AN=20618;AC=2;AF=9.700261907071492e-05;N_HET=2;N_HOMALT=0;N_HOMREF=10307;FREQ_HET=0.0001940052381414283;FREQ_HOMALT=0.0;FREQ_HOMREF=0.9998059947618586;N_BI_GENOS=10309;Miao_AN=482;Miao_AC=2;Miao_AF=0.00414937759
chr2	78749326	.	A	<INS:ME:LINE1>	.	PASS	SVTYPE=INS:ME:LINE1;END=78749341;SVLEN=1304;AN=20618;AC=2;AF=9.700261907071492e-05;N_HET=2;N_HOMALT=0;N_HOMREF=10307;FREQ_HET=0.0001940052381414283;FREQ_HOMALT=0.0;FREQ_HOMREF=0.9998059947618586;N_BI_GENOS=10309;East_Han_AN=6554;East_Han_AC=1;East_Han_AF
chr2	78764903	.	A	<INS:ME:LINE1>	.	PASS	SVTYPE=INS:ME:LINE1;END=78764917;SVLEN=1991;AN=20618;AC=74;AF=0.0035890969056164516;N_HET=64;N_HOMALT=5;N_HOMREF=10240;FREQ_HET=0.006208167620525755;FREQ_HOMALT=0.0004850130953535357455;FREQ_HOMREF=0.9933068192841207;N_BI_GENOS=10309;South_Han_AN=1496;South
chr2	78826623	.	A	<INS:ME:LINE1>	.	PASS	SVTYPE=INS:ME:LINE1;END=78826640;SVLEN=6035;AN=20618;AC=1;AF=4.850130953535746e-05;N_HET=1;N_HOMALT=0;N_HOMREF=10308;FREQ_HET=9.700261907071492e-05;FREQ_HOMALT=0.0;FREQ_HOMREF=0.9999029973809292;N_BI_GENOS=10309;Northwest_Han_AN=1296;Northwest_Han_AC=1;No
chr2	79011979	.	A	<INS:ME:LINE1>	.	PASS	SVTYPE=INS:ME:LINE1;END=79011992;SVLEN=5997;AN=20618;AC=1;AF=4.850130953535746e-05;N_HET=1;N_HOMALT=0;N_HOMREF=10308;FREQ_HET=9.700261907071492e-05;FREQ_HOMALT=0.0;FREQ_HOMREF=0.9999029973809292;N_BI_GENOS=10309;Lingnan_Han_AN=1888;Lingnan_Han_AC=1;Lingna
chr2	79296834	.	G	<INS:ME:LINE1>	.	PASS	SVTYPE=INS:ME:LINE1;END=79296841;SVLEN=722;AN=20618;AC=1;AF=4.850130953535746e-05;N_HET=1;N_HOMALT=0;N_HOMREF=10308;FREQ_HET=9.700261907071492e-05;FREQ_HOMALT=0.0;FREQ_HOMREF=0.9999029973809292;N_BI_GENOS=10309;North_Han_AN=2272;North_Han_AC=1;North_Han_A
chr2	79437232	.	A	<INS:ME:LINE1>	.	PASS	SVTYPE=INS:ME:LINE1;END=79437246;SVLEN=504;AN=20618;AC=3;AF=0.00014550392860607236;N_HET=1;N_HOMALT=1;N_HOMREF=10307;FREQ_HET=9.700261907071492e-05;FREQ_HOMALT=9.700261907071492e-05;FREQ_HOMREF=0.9998059947618586;N_BI_GENOS=10309;South_Han_AN=1496;South_H
chr2	79461660	.	A	<INS:ME:LINE1>	.	PASS	SVTYPE=INS:ME:LINE1;END=79461673;SVLEN=87;AN=20618;AC=1;AF=4.850130953535746e-05;N_HET=1;N_HOMALT=0;N_HOMREF=10308;FREQ_HET=9.700261907071492e-05;FREQ_HOMALT=0.0;FREQ_HOMREF=0.9999029973809292;N_BI_GENOS=10309;Central_Han_AN=2128;Central_Han_AC=1;Central_
chr2	79487259	.	A	<INS:ME:LINE1>	.	PASS	SVTYPE=INS:ME:LINE1;END=79487269;SVLEN=1953;AN=20618;AC=1;AF=4.850130953535746e-05;N_HET=1;N_HOMALT=0;N_HOMREF=10308;FREQ_HET=9.700261907071492e-05;FREQ_HOMALT=0.0;FREQ_HOMREF=0.9999029973809292;N_BI_GENOS=10309;Southeast_Han_AN=1908;Southeast_Han_AC=1;So
chr2	79730720	.	T	<INS:ME:LINE1>	.	PASS	SVTYPE=INS:ME:LINE1;END=79730735;SVLEN=1038;AN=20618;AC=5;AF=0.00024250654767678728;N_HET=3;N_HOMALT=1;N_HOMREF=10305;FREQ_HET=0.0002910078572121447;FREQ_HOMALT=9.700261907071492e-05;FREQ_HOMREF=0.9996119895237171;N_BI_GENOS=10309;East_Han_AN=6554;East_Ha
chr2	79887472	.	T	<INS:ME:LINE1>	.	PASS	SVTYPE=INS:ME:LINE1;END=79887490;SVLEN=357;AN=20618;AC=3;AF=0.00014550392860607236;N_HET=3;N_HOMALT=0;N_HOMREF=10306;FREQ_HET=0.0002910078572121447;FREQ_HOMALT=0.0;FREQ_HOMREF=0.9997089921427879;N_BI_GENOS=10309;Lingnan_Han_AN=1888;Lingnan_Han_AC=1;Lingna
chr2	80249892	.	C	<INS:ME:LINE1>	.	PASS	SVTYPE=INS:ME:LINE1;END=80249909;SVLEN=623;AN=20618;AC=1;AF=4.850130953535746e-05;N_HET=1;N_HOMALT=0;N_HOMREF=10308;FREQ_HET=9.700261907071492e-05;FREQ_HOMALT=0.0;FREQ_HOMREF=0.9999029973809292;N_BI_GENOS=10309;Mongolian_AN=454;Mongolian_AC=1;Mongolian_AF
chr2	80411808	.	A	<INS:ME:LINE1>	.	PASS	SVTYPE=INS:ME:LINE1;END=80411822;SVLEN=2440;AN=20618;AC=2;AF=9.700261907071492e-05;N_HET=2;N_HOMALT=0;N_HOMREF=10307;FREQ_HET=0.0001940052381414283;FREQ_HOMALT=0.0;FREQ_HOMREF=0.9998059947618586;N_BI_GENOS=10309;North_Han_AN=2272;North_Han_AC=1;North_Han
chr2	80446956	.	G	<INS:ME:LINE1>	.	PASS	SVTYPE=INS:ME:LINE1;END=80446971;SVLEN=5984;AN=20618;AC=1;AF=4.850130953535746e-05;N_HET=1;N_HOMALT=0;N_HOMREF=10308;FREQ_HET=9.700261907071492e-05;FREQ_HOMALT=0.0;FREQ_HOMREF=0.9999029973809292;N_BI_GENOS=10309;Lingnan_Han_AN=1888;Lingnan_Han_AC=1;Lingna
chr2	80637393	.	T	<INS:ME:LINE1>	.	PASS	SVTYPE=INS:ME:LINE1;END=80637407;SVLEN=694;AN=20618;AC=1;AF=4.850130953535746e-05;N_HET=1;N_HOMALT=0;N_HOMREF=10308;FREQ_HET=9.700261907071492e-05;FREQ_HOMALT=0.0;FREQ_HOMREF=0.9999029973809292;N_BI_GENOS=10309;North_Han_AN=2272;North_Han_AC=1;North_Han_A
chr2	80939710	.	T	<INS:ME:LINE1>	.	PASS	SVTYPE=INS:ME:LINE1;END=80939716;SVLEN=1151;AN=20618;AC=1;AF=4.850130953535746e-05;N_HET=1;N_HOMALT=0;N_HOMREF=10308;FREQ_HET=9.700261907071492e-05;FREQ_HOMALT=0.0;FREQ_HOMREF=0.9999029973809292;N_BI_GENOS=10309;Northwest_Han_AN=1296;Northwest_Han_AC=1;No
chr2	80982121	.	T	<INS:ME:LINE1>	.	PASS	SVTYPE=INS:ME:LINE1;END=80982124;SVLEN=1258;AN=20618;AC=1;AF=4.850130953535746e-05;N_HET=1;N_HOMALT=0;N_HOMREF=10308;FREQ_HET=9.700261907071492e-05;FREQ_HOMALT=0.0;FREQ_HOMREF=0.9999029973809292;N_BI_GENOS=10309;East_Han_AN=6554;East_Han_AC=1;East_Han_AF=
chr2	81067652	.	T	<INS:ME:LINE1>	.	PASS	SVTYPE=INS:ME:LINE1;END=81067673;SVLEN=712;AN=20618;AC=1;AF=4.850130953535746e-05;N_HET=1;N_HOMALT=0;N_HOMREF=10308;FREQ_HET=9.700261907071492e-05;FREQ_HOMALT=0.0;FREQ_HOMREF=0.9999029973809292;N_BI_GENOS=10309;Northwest_Han_AN=1296;Northwest_Han_AC=1;Nor
chr2	81317572	.	A	<INS:ME:LINE1>	.	PASS	SVTYPE=INS:ME:LINE1;END=81317585;SVLEN=6008;AN=20618;AC=1;AF=4.850130953535746e-05;N_HET=1;N_HOMALT=0;N_HOMREF=10308;FREQ_HET=9.700261907071492e-05;FREQ_HOMALT=0.0;FREQ_HOMREF=0.9999029973809292;N_BI_GENOS=10309;Mongolian_AN=454;Mongolian_AC=1;Mongolian_A
chr2	81599683	.	A	<INS:ME:LINE1>	.	PASS	SVTYPE=INS:ME:LINE1;END=81599699;SVLEN=1061;AN=20618;AC=4;AF=0.0001940052381414283;N_HET=4;N_HOMALT=0;N_HOMREF=10305;FREQ_HET=0.0003880104762828566;FREQ_HOMALT=0.0;FREQ_HOMREF=0.9996119895237171;N_BI_GENOS=10309;Lingnan_Han_AN=1888;Lingnan_Han_AC=2;Ling
chr2	81716757	.	A	<INS:ME:LINE1>	.	PASS	SVTYPE=INS:ME:LINE1;END=81716762;SVLEN=541;AN=20618;AC=1;AF=4.850130953535746e-05;N_HET=1;N_HOMALT=0;N_HOMREF=10308;FREQ_HET=9.700261907071492e-05;FREQ_HOMALT=0.0;FREQ_HOMREF=0.9999029973809292;N_BI_GENOS=10309;North_Han_AN=2272;North_Han_AC=1;North_Han_A
chr2	81870903	.	A	<INS:ME:LINE1>	.	PASS	SVTYPE=INS:ME:LINE1;END=81870919;SVLEN=5998;AN=20618;AC=12077;AF=0.585750315258512;N_HET=4759;N_HOMALT=3659;N_HOMREF=1891;FREQ_HET=0.46163546415753226;FREQ_HOMALT=0.35493258317974585;FREQ_HOMREF=0.1834319526627219;N_BI_GENOS=10309;South_Han_AN=1496;South_
chr2	82052577	.	T	<INS:ME:LINE1>	.	PASS	SVTYPE=INS:ME:LINE1;END=82052577;SVLEN=0;AN=20618;AC=2;AF=9.700261907071492e-05;N_HET=2;N_HOMALT=0;N_HOMREF=10307;FREQ_HET=0.0001940052381414283;FREQ_HOMALT=0.0;FREQ_HOMREF=0.9998059947618586;N_BI_GENOS=10309;Northwest_Han_AN=1296;Northwest_Han_AC=1;Nort
chr2	82070745	.	A	<INS:ME:LINE1>	.	PASS	SVTYPE=INS:ME:LINE1;END=82070763;SVLEN=3850;AN=20618;AC=1;AF=4.850130953535746e-05;N_HET=1;N_HOMALT=0;N_HOMREF=10308;FREQ_HET=9.700261907071492e-05;FREQ_HOMALT=0.0;FREQ_HOMREF=0.9999029973809292;N_BI_GENOS=10309;Lingnan_Han_AN=1888;Lingnan_Han_AC=1;Lingna
chr2	82188899	.	A	<INS:ME:LINE1>	.	PASS	SVTYPE=INS:ME:LINE1;END=82188914;SVLEN=618;AN=20618;AC=1;AF=4.850130953535746e-05;N_HET=1;N_HOMALT=0;N_HOMREF=10308;FREQ_HET=9.700261907071492e-05;FREQ_HOMALT=0.0;FREQ_HOMREF=0.9999029973809292;N_BI_GENOS=10309;Miao_AN=482;Miao_AC=1;Miao_AF=0.002074688796
chr2	82337428	.	A	<INS:ME:LINE1>	.	PASS	SVTYPE=INS:ME:LINE1;END=82337446;SVLEN=1036;AN=20618;AC=1;AF=4.850130953535746e-05;N_HET=1;N_HOMALT=0;N_HOMREF=10308;FREQ_HET=9.700261907071492e-05;FREQ_HOMALT=0.0;FREQ_HOMREF=0.9999029973809292;N_BI_GENOS=10309;North_Han_AN=2272;North_Han_AC=1;North_Han_
chr2	82392064	.	C	<INS:ME:LINE1>	.	PASS	SVTYPE=INS:ME:LINE1;END=82392078;SVLEN=2782;AN=20618;AC=1;AF=4.850130953535746e-05;N_HET=1;N_HOMALT=0;N_HOMREF=10308;FREQ_HET=9.700261907071492e-05;FREQ_HOMALT=0.0;FREQ_HOMREF=0.9999029973809292;N_BI_GENOS=10309;Lingnan_Han_AN=1888;Lingnan_Han_AC=1;Lingna
chr2	82561597	.	T	<INS:ME:LINE1>	.	PASS	SVTYPE=INS:ME:LINE1;END=82561612;SVLEN=743;AN=20618;AC=1;AF=4.850130953535746e-05;N_HET=1;N_HOMALT=0;N_HOMREF=10308;FREQ_HET=9.700261907071492e-05;FREQ_HOMALT=0.0;FREQ_HOMREF=0.9999029973809292;N_BI_GENOS=10309;Southeast_Han_AN=1908;Southeast_Han_AC=1;Sou
chr2	82651839	.	A	<INS:ME:LINE1>	.	PASS	SVTYPE=INS:ME:LINE1;END=82651853;SVLEN=1492;AN=20618;AC=2;AF=9.700261907071492e-05;N_HET=2;N_HOMALT=0;N_HOMREF=10307;FREQ_HET=0.0001940052381414283;FREQ_HOMALT=0.0;FREQ_HOMREF=0.9998059947618586;N_BI_GENOS=10309;South_Han_AN=1496;South_Han_AC=1;South_Han
chr2	82695466	.	A	<INS:ME:LINE1>	.	PASS	SVTYPE=INS:ME:LINE1;END=82695469;SVLEN=1264;AN=20618;AC=1;AF=4.850130953535746e-05;N_HET=1;N_HOMALT=0;N_HOMREF=10308;FREQ_HET=9.700261907071492e-05;FREQ_HOMALT=0.0;FREQ_HOMREF=0.9999029973809292;N_BI_GENOS=10309;Central_Han_AN=2128;Central_Han_AC=1;Centra
chr2	82961200	.	A	<INS:ME:LINE1>	.	PASS	SVTYPE=INS:ME:LINE1;END=82961215;SVLEN=3621;AN=20618;AC=4485;AF=0.21752837326607818;N_HET=3259;N_HOMALT=613;N_HOMREF=6437;FREQ_HET=0.3161313555514599;FREQ_HOMALT=0.059462605490348236;FREQ_HOMREF=0.6244058589581919;N_BI_GENOS=10309;South_Han_AN=1496;South_
chr2	83150918	.	G	<INS:ME:LINE1>	.	PASS	SVTYPE=INS:ME:LINE1;END=83150933;SVLEN=1417;AN=20618;AC=1;AF=4.850130953535746e-05;N_HET=1;N_HOMALT=0;N_HOMREF=10308;FREQ_HET=9.700261907071492e-05;FREQ_HOMALT=0.0;FREQ_HOMREF=0.9999029973809292;N_BI_GENOS=10309;Central_Han_AN=2128;Central_Han_AC=1;Centra
chr2	83231689	.	C	<INS:ME:LINE1>	.	PASS	SVTYPE=INS:ME:LINE1;END=83231689;SVLEN=1054;AN=20618;AC=1;AF=4.850130953535746e-05;N_HET=1;N_HOMALT=0;N_HOMREF=10308;FREQ_HET=9.700261907071492e-05;FREQ_HOMALT=0.0;FREQ_HOMREF=0.9999029973809292;N_BI_GENOS=10309;East_Han_AN=6554;East_Han_AC=1;East_Han_AF=
chr2	83327088	.	A	<INS:ME:LINE1>	.	PASS	SVTYPE=INS:ME:LINE1;END=83327088;SVLEN=174;AN=20618;AC=29;AF=0.0014065379765253662;N_HET=29;N_HOMALT=0;N_HOMREF=10280;FREQ_HET=0.0028130759530507325;FREQ_HOMALT=0.0;FREQ_HOMREF=0.9971869240469493;N_BI_GENOS=10309;South_Han_AN=1496;South_Han_AC=1;South_Han
chr2	83442163	.	A	<INS:ME:LINE1>	.	PASS	SVTYPE=INS:ME:LINE1;END=83442178;SVLEN=6027;AN=20618;AC=1;AF=4.850130953535746e-05;N_HET=1;N_HOMALT=0;N_HOMREF=10308;FREQ_HET=9.700261907071492e-05;FREQ_HOMALT=0.0;FREQ_HOMREF=0.9999029973809292;N_BI_GENOS=10309;North_Han_AN=2272;North_Han_AC=1;North_Han_
chr2	83603719	.	G	<INS:ME:LINE1>	.	PASS	SVTYPE=INS:ME:LINE1;END=83603733;SVLEN=5999;AN=20618;AC=1;AF=4.850130953535746e-05;N_HET=1;N_HOMALT=0;N_HOMREF=10308;FREQ_HET=9.700261907071492e-05;FREQ_HOMALT=0.0;FREQ_HOMREF=0.9999029973809292;N_BI_GENOS=10309;Southeast_Han_AN=1908;Southeast_Han_AC=1;So
chr2	83707370	.	C	<INS:ME:LINE1>	.	PASS	SVTYPE=INS:ME:LINE1;END=83707418;SVLEN=1265;AN=20618;AC=1;AF=4.850130953535746e-05;N_HET=1;N_HOMALT=0;N_HOMREF=10308;FREQ_HET=9.700261907071492e-05;FREQ_HOMALT=0.0;FREQ_HOMREF=0.9999029973809292;N_BI_GENOS=10309;East_Han_AN=6554;East_Han_AC=1;East_Han_AF=
chr2	84000701	.	G	<INS:ME:LINE1>	.	PASS	SVTYPE=INS:ME:LINE1;END=84000717;SVLEN=348;AN=20618;AC=1;AF=4.850130953535746e-05;N_HET=1;N_HOMALT=0;N_HOMREF=10308;FREQ_HET=9.700261907071492e-05;FREQ_HOMALT=0.0;FREQ_HOMREF=0.9999029973809292;N_BI_GENOS=10309;South_Han_AN=1496;South_Han_AC=1;South_Han_A
chr2	84414343	.	T	<INS:ME:LINE1>	.	PASS	SVTYPE=INS:ME:LINE1;END=84414353;SVLEN=97;AN=20618;AC=1;AF=4.850130953535746e-05;N_HET=1;N_HOMALT=0;N_HOMREF=10308;FREQ_HET=9.700261907071492e-05;FREQ_HOMALT=0.0;FREQ_HOMREF=0.9999029973809292;N_BI_GENOS=10309;North_Han_AN=2272;North_Han_AC=1;North_Han_AF
chr2	84550825	.	A	<INS:ME:LINE1>	.	PASS	SVTYPE=INS:ME:LINE1;END=84550834;SVLEN=170;AN=20618;AC=1;AF=4.850130953535746e-05;N_HET=1;N_HOMALT=0;N_HOMREF=10308;FREQ_HET=9.700261907071492e-05;FREQ_HOMALT=0.0;FREQ_HOMREF=0.9999029973809292;N_BI_GENOS=10309;Northwest_Han_AN=1296;Northwest_Han_AC=1;Nor
chr2	84559073	.	T	<INS:ME:LINE1>	.	PASS	SVTYPE=INS:ME:LINE1;END=84559088;SVLEN=5993;AN=20618;AC=1;AF=4.850130953535746e-05;N_HET=1;N_HOMALT=0;N_HOMREF=10308;FREQ_HET=9.700261907071492e-05;FREQ_HOMALT=0.0;FREQ_HOMREF=0.9999029973809292;N_BI_GENOS=10309;Tibetan_AN=414;Tibetan_AC=1;Tibetan_AF=0.00
chr2	84653207	.	A	<INS:ME:LINE1>	.	PASS	SVTYPE=INS:ME:LINE1;END=84653218;SVLEN=5999;AN=20618;AC=1;AF=4.850130953535746e-05;N_HET=1;N_HOMALT=0;N_HOMREF=10308;FREQ_HET=9.700261907071492e-05;FREQ_HOMALT=0.0;FREQ_HOMREF=0.9999029973809292;N_BI_GENOS=10309;North_Han_AN=2272;North_Han_AC=1;North_Han_
chr2	84705989	.	T	<INS:ME:LINE1>	.	PASS	SVTYPE=INS:ME:LINE1;END=84705989;SVLEN=5935;AN=20618;AC=1;AF=4.850130953535746e-05;N_HET=1;N_HOMALT=0;N_HOMREF=10308;FREQ_HET=9.700261907071492e-05;FREQ_HOMALT=0.0;FREQ_HOMREF=0.9999029973809292;N_BI_GENOS=10309;Central_Han_AN=2128;Central_Han_AC=1;Centra
chr2	84762615	.	A	<INS:ME:LINE1>	.	PASS	SVTYPE=INS:ME:LINE1;END=84762632;SVLEN=6031;AN=20618;AC=1;AF=4.850130953535746e-05;N_HET=1;N_HOMALT=0;N_HOMREF=10308;FREQ_HET=9.700261907071492e-05;FREQ_HOMALT=0.0;FREQ_HOMREF=0.9999029973809292;N_BI_GENOS=10309;East_Han_AN=6554;East_Han_AC=1;East_Han_AF=
chr2	84767102	.	A	<INS:ME:LINE1>	.	PASS	SVTYPE=INS:ME:LINE1;END=84767119;SVLEN=1104;AN=20618;AC=1;AF=4.850130953535746e-05;N_HET=1;N_HOMALT=0;N_HOMREF=10308;FREQ_HET=9.700261907071492e-05;FREQ_HOMALT=0.0;FREQ_HOMREF=0.9999029973809292;N_BI_GENOS=10309;Southeast_Han_AN=1908;Southeast_Han_AC=1;So
chr2	85636187	.	T	<INS:ME:LINE1>	.	PASS	SVTYPE=INS:ME:LINE1;END=85636187;SVLEN=6018;AN=20618;AC=1;AF=4.850130953535746e-05;N_HET=1;N_HOMALT=0;N_HOMREF=10308;FREQ_HET=9.700261907071492e-05;FREQ_HOMALT=0.0;FREQ_HOMREF=0.9999029973809292;N_BI_GENOS=10309;East_Han_AN=6554;East_Han_AC=1;East_Han_AF=
chr2	85932911	.	T	<INS:ME:LINE1>	.	PASS	SVTYPE=INS:ME:LINE1;END=85932927;SVLEN=719;AN=20618;AC=1;AF=4.850130953535746e-05;N_HET=1;N_HOMALT=0;N_HOMREF=10308;FREQ_HET=9.700261907071492e-05;FREQ_HOMALT=0.0;FREQ_HOMREF=0.9999029973809292;N_BI_GENOS=10309;Southeast_Han_AN=1908;Southeast_Han_AC=1;Sou
chr2	86419558	.	A	<INS:ME:LINE1>	.	PASS	SVTYPE=INS:ME:LINE1;END=86419559;SVLEN=1043;AN=20618;AC=2;AF=9.700261907071492e-05;N_HET=0;N_HOMALT=1;N_HOMREF=10308;FREQ_HET=0.0;FREQ_HOMALT=9.700261907071492e-05;FREQ_HOMREF=0.9999029973809292;N_BI_GENOS=10309;Northwest_Han_AN=1296;Northwest_Han_AC=2;No
chr2	86642883	.	A	<INS:ME:LINE1>	.	PASS	SVTYPE=INS:ME:LINE1;END=86642897;SVLEN=1268;AN=20618;AC=2;AF=9.700261907071492e-05;N_HET=0;N_HOMALT=1;N_HOMREF=10308;FREQ_HET=0.0;FREQ_HOMALT=9.700261907071492e-05;FREQ_HOMREF=0.9999029973809292;N_BI_GENOS=10309;East_Han_AN=6554;East_Han_AC=2;East_Han_AF=
chr2	86673140	.	A	<INS:ME:LINE1>	.	PASS	SVTYPE=INS:ME:LINE1;END=86673157;SVLEN=871;AN=20618;AC=1;AF=4.850130953535746e-05;N_HET=1;N_HOMALT=0;N_HOMREF=10308;FREQ_HET=9.700261907071492e-05;FREQ_HOMALT=0.0;FREQ_HOMREF=0.9999029973809292;N_BI_GENOS=10309;South_Han_AN=1496;South_Han_AC=1;South_Han_A
chr2	88146473	.	C	<INS:ME:LINE1>	.	PASS	SVTYPE=INS:ME:LINE1;END=88146489;SVLEN=47;AN=20618;AC=2;AF=9.700261907071492e-05;N_HET=2;N_HOMALT=0;N_HOMREF=10307;FREQ_HET=0.0001940052381414283;FREQ_HOMALT=0.0;FREQ_HOMREF=0.9998059947618586;N_BI_GENOS=10309;South_Han_AN=1496;South_Han_AC=1;South_Han_A

chr2	88795749	.	A	<INS:ME:LINE1>	.	PASS	SVTYPE=INS:ME:LINE1;END=88795749;SVLEN=1492;AN=20618;AC=1;AF=4.850130953535746e-05;N_HET=1;N_HOMALT=0;N_HOMREF=10308;FREQ_HET=9.700261907071492e-05;FREQ_HOMALT=0.0;FREQ_HOMREF=0.9999029973809292;N_BI_GENOS=10309;Northwest_Han_AN=1296;Northwest_Han_AC=1;No
chr2	88833514	.	G	<INS:ME:LINE1>	.	PASS	SVTYPE=INS:ME:LINE1;END=88833528;SVLEN=5998;AN=20618;AC=1;AF=4.850130953535746e-05;N_HET=1;N_HOMALT=0;N_HOMREF=10308;FREQ_HET=9.700261907071492e-05;FREQ_HOMALT=0.0;FREQ_HOMREF=0.9999029973809292;N_BI_GENOS=10309;Mongolian_AN=454;Mongolian_AC=1;Mongolian_A
chr2	88871166	.	A	<INS:ME:LINE1>	.	PASS	SVTYPE=INS:ME:LINE1;END=88871185;SVLEN=5475;AN=20618;AC=1;AF=4.850130953535746e-05;N_HET=1;N_HOMALT=0;N_HOMREF=10308;FREQ_HET=9.700261907071492e-05;FREQ_HOMALT=0.0;FREQ_HOMREF=0.9999029973809292;N_BI_GENOS=10309;Southeast_Han_AN=1908;Southeast_Han_AC=1;So
chr2	89675832	.	G	<INS:ME:LINE1>	.	PASS	SVTYPE=INS:ME:LINE1;END=89675832;SVLEN=165;AN=20618;AC=1;AF=4.850130953535746e-05;N_HET=1;N_HOMALT=0;N_HOMREF=10308;FREQ_HET=9.700261907071492e-05;FREQ_HOMALT=0.0;FREQ_HOMREF=0.9999029973809292;N_BI_GENOS=10309;East_Han_AN=6554;East_Han_AC=1;East_Han_AF=0
chr2	89676873	.	A	<INS:ME:LINE1>	.	PASS	SVTYPE=INS:ME:LINE1;END=89676903;SVLEN=3633;AN=20618;AC=1;AF=4.850130953535746e-05;N_HET=1;N_HOMALT=0;N_HOMREF=10308;FREQ_HET=9.700261907071492e-05;FREQ_HOMALT=0.0;FREQ_HOMREF=0.9999029973809292;N_BI_GENOS=10309;East_Han_AN=6554;East_Han_AC=1;East_Han_AF=
chr2	90134909	.	A	<INS:ME:LINE1>	.	PASS	SVTYPE=INS:ME:LINE1;END=90134928;SVLEN=1175;AN=20618;AC=1;AF=4.850130953535746e-05;N_HET=1;N_HOMALT=0;N_HOMREF=10308;FREQ_HET=9.700261907071492e-05;FREQ_HOMALT=0.0;FREQ_HOMREF=0.9999029973809292;N_BI_GENOS=10309;Zhuang_AN=444;Zhuang_AC=1;Zhuang_AF=0.00225
chr2	91415373	.	A	<INS:ME:LINE1>	.	PASS	SVTYPE=INS:ME:LINE1;END=91415373;SVLEN=0;AN=20618;AC=1;AF=4.850130953535746e-05;N_HET=1;N_HOMALT=0;N_HOMREF=10308;FREQ_HET=9.700261907071492e-05;FREQ_HOMALT=0.0;FREQ_HOMREF=0.9999029973809292;N_BI_GENOS=10309;South_Han_AN=1496;South_Han_AC=1;South_Han_AF=
chr2	91705318	.	G	<INS:ME:LINE1>	.	PASS	SVTYPE=INS:ME:LINE1;END=91705327;SVLEN=1330;AN=20618;AC=1;AF=4.850130953535746e-05;N_HET=1;N_HOMALT=0;N_HOMREF=10308;FREQ_HET=9.700261907071492e-05;FREQ_HOMALT=0.0;FREQ_HOMREF=0.9999029973809292;N_BI_GENOS=10309;Manchu_AN=416;Manchu_AC=1;Manchu_AF=0.00240
chr2	94506369	.	A	<INS:ME:LINE1>	.	PASS	SVTYPE=INS:ME:LINE1;END=94506416;SVLEN=6152;AN=20618;AC=21;AF=0.0010185275002425065;N_HET=13;N_HOMALT=4;N_HOMREF=10292;FREQ_HET=0.0012610340479192938;FREQ_HOMALT=0.00038801047628285966;FREQ_HOMREF=0.9983509554757979;N_BI_GENOS=10309;East_Han_AN=6554;East_
chr2	94529556	.	G	<INS:ME:LINE1>	.	PASS	SVTYPE=INS:ME:LINE1;END=94529572;SVLEN=5998;AN=20618;AC=3;AF=0.00014550392860607236;N_HET=3;N_HOMALT=0;N_HOMREF=10306;FREQ_HET=0.0002910078572121447;FREQ_HOMALT=0.0;FREQ_HOMREF=0.9997089921427879;N_BI_GENOS=10309;Central_Han_AN=2128;Central_Han_AC=1;Centr
chr2	94537235	.	C	<INS:ME:LINE1>	.	PASS	SVTYPE=INS:ME:LINE1;END=94537250;SVLEN=758;AN=20618;AC=6;AF=0.0002910078572121447;N_HET=6;N_HOMALT=0;N_HOMREF=10303;FREQ_HET=0.0005820157144242894;FREQ_HOMALT=0.0;FREQ_HOMREF=0.9994179842855757;N_BI_GENOS=10309;South_Han_AN=1496;South_Han_AC=1;South_Han_A
chr2	94542835	.	C	<INS:ME:LINE1>	.	PASS	SVTYPE=INS:ME:LINE1;END=94542835;SVLEN=0;AN=20618;AC=1;AF=4.850130953535746e-05;N_HET=1;N_HOMALT=0;N_HOMREF=10308;FREQ_HET=9.700261907071492e-05;FREQ_HOMALT=0.0;FREQ_HOMREF=0.9999029973809292;N_BI_GENOS=10309;Central_Han_AN=2128;Central_Han_AC=1;Central_H
chr2	94543002	.	T	<INS:ME:LINE1>	.	PASS	SVTYPE=INS:ME:LINE1;END=94543002;SVLEN=0;AN=20618;AC=2;AF=9.700261907071492e-05;N_HET=2;N_HOMALT=0;N_HOMREF=10307;FREQ_HET=0.0001940052381414298;FREQ_HOMALT=0.0;FREQ_HOMREF=0.9998059947618586;N_BI_GENOS=10309;Central_Han_AN=2128;Central_Han_AC=2;Central_
chr2	94669284	.	A	<INS:ME:LINE1>	.	PASS	SVTYPE=INS:ME:LINE1;END=94669284;SVLEN=610;AN=20618;AC=1;AF=4.850130953535746e-05;N_HET=1;N_HOMALT=0;N_HOMREF=10308;FREQ_HET=9.700261907071492e-05;FREQ_HOMALT=0.0;FREQ_HOMREF=0.9999029973809292;N_BI_GENOS=10309;North_Han_AN=2272;North_Han_AC=1;North_Han_A
chr2	94821198	.	G	<INS:ME:LINE1>	.	PASS	SVTYPE=INS:ME:LINE1;END=94821213;SVLEN=1829;AN=20618;AC=1;AF=4.850130953535746e-05;N_HET=1;N_HOMALT=0;N_HOMREF=10308;FREQ_HET=9.700261907071492e-05;FREQ_HOMALT=0.0;FREQ_HOMREF=0.9999029973809292;N_BI_GENOS=10309;Central_Han_AN=2128;Central_Han_AC=1;Centra
chr2	94935690	.	A	<INS:ME:LINE1>	.	PASS	SVTYPE=INS:ME:LINE1;END=94935690;SVLEN=2912;AN=20618;AC=2;AF=9.700261907071492e-05;N_HET=2;N_HOMALT=0;N_HOMREF=10307;FREQ_HET=0.0001940052381414298;FREQ_HOMALT=0.0;FREQ_HOMREF=0.9998059947618586;N_BI_GENOS=10309;East_Han_AN=6554;East_Han_AC=2;East_Han_AF
chr2	95854667	.	A	<INS:ME:LINE1>	.	PASS	SVTYPE=INS:ME:LINE1;END=95854669;SVLEN=944;AN=20618;AC=9;AF=0.0004365117858182171;N_HET=9;N_HOMALT=0;N_HOMREF=10300;FREQ_HET=0.0008730235716364342;FREQ_HOMALT=0.0;FREQ_HOMREF=0.9991269764283636;N_BI_GENOS=10309;South_Han_AN=1496;South_Han_AC=1;South_Han_A
chr2	95936281	.	T	<INS:ME:LINE1>	.	PASS	SVTYPE=INS:ME:LINE1;END=95936291;SVLEN=229;AN=20618;AC=1;AF=4.850130953535746e-05;N_HET=1;N_HOMALT=0;N_HOMREF=10308;FREQ_HET=9.700261907071492e-05;FREQ_HOMALT=0.0;FREQ_HOMREF=0.9999029973809292;N_BI_GENOS=10309;East_Han_AN=6554;East_Han_AC=1;East_Han_AF=0
chr2	96076959	.	A	<INS:ME:LINE1>	.	PASS	SVTYPE=INS:ME:LINE1;END=96076975;SVLEN=6012;AN=20618;AC=2;AF=9.700261907071492e-05;N_HET=2;N_HOMALT=0;N_HOMREF=10307;FREQ_HET=0.0001940052381414298;FREQ_HOMALT=0.0;FREQ_HOMREF=0.9998059947618586;N_BI_GENOS=10309;Central_Han_AN=2128;Central_Han_AC=2;Centr
chr2	96120054	.	A	<INS:ME:LINE1>	.	PASS	SVTYPE=INS:ME:LINE1;END=96120065;SVLEN=3521;AN=20618;AC=2;AF=9.700261907071492e-05;N_HET=2;N_HOMALT=0;N_HOMREF=10307;FREQ_HET=0.0001940052381414298;FREQ_HOMALT=0.0;FREQ_HOMREF=0.9998059947618586;N_BI_GENOS=10309;North_Han_AN=2272;North_Han_AC=1;North_Han
chr2	96197856	.	C	<INS:ME:LINE1>	.	PASS	SVTYPE=INS:ME:LINE1;END=96197872;SVLEN=974;AN=20618;AC=1;AF=4.850130953535746e-05;N_HET=1;N_HOMALT=0;N_HOMREF=10308;FREQ_HET=9.700261907071492e-05;FREQ_HOMALT=0.0;FREQ_HOMREF=0.9999029973809292;N_BI_GENOS=10309;Central_Han_AN=2128;Central_Han_AC=1;Central
chr2	96338818	.	T	<INS:ME:LINE1>	.	PASS	SVTYPE=INS:ME:LINE1;END=96338824;SVLEN=226;AN=20618;AC=1;AF=4.850130953535746e-05;N_HET=1;N_HOMALT=0;N_HOMREF=10308;FREQ_HET=9.700261907071492e-05;FREQ_HOMALT=0.0;FREQ_HOMREF=0.9999029973809292;N_BI_GENOS=10309;East_Han_AN=6554;East_Han_AC=1;East_Han_AF=0
chr2	96426771	.	A	<INS:ME:LINE1>	.	PASS	SVTYPE=INS:ME:LINE1;END=96426790;SVLEN=338;AN=20618;AC=1;AF=4.850130953535746e-05;N_HET=1;N_HOMALT=0;N_HOMREF=10308;FREQ_HET=9.700261907071492e-05;FREQ_HOMALT=0.0;FREQ_HOMREF=0.9999029973809292;N_BI_GENOS=10309;South_Han_AN=1496;South_Han_AC=1;South_Han_A
chr2	97149249	.	A	<INS:ME:LINE1>	.	PASS	SVTYPE=INS:ME:LINE1;END=97149266;SVLEN=3372;AN=20618;AC=8;AF=0.00038801047628285966;N_HET=8;N_HOMALT=0;N_HOMREF=10301;FREQ_HET=0.0007760209525657193;FREQ_HOMALT=0.0;FREQ_HOMREF=0.9992239790474343;N_BI_GENOS=10309;South_Han_AN=1496;South_Han_AC=3;South_Han
chr2	97568661	.	A	<INS:ME:LINE1>	.	PASS	SVTYPE=INS:ME:LINE1;END=97568675;SVLEN=2610;AN=20618;AC=1;AF=4.850130953535746e-05;N_HET=1;N_HOMALT=0;N_HOMREF=10308;FREQ_HET=9.700261907071492e-05;FREQ_HOMALT=0.0;FREQ_HOMREF=0.9999029973809292;N_BI_GENOS=10309;South_Han_AN=1496;South_Han_AC=1;South_Han_
chr2	97866573	.	C	<INS:ME:LINE1>	.	PASS	SVTYPE=INS:ME:LINE1;END=97866590;SVLEN=5990;AN=20618;AC=1;AF=4.850130953535746e-05;N_HET=1;N_HOMALT=0;N_HOMREF=10308;FREQ_HET=9.700261907071492e-05;FREQ_HOMALT=0.0;FREQ_HOMREF=0.9999029973809292;N_BI_GENOS=10309;Mongolian_AN=454;Mongolian_AC=1;Mongolian_A
chr2	98270232	.	A	<INS:ME:LINE1>	.	PASS	SVTYPE=INS:ME:LINE1;END=98270245;SVLEN=5989;AN=20618;AC=1;AF=4.850130953535746e-05;N_HET=1;N_HOMALT=0;N_HOMREF=10308;FREQ_HET=9.700261907071492e-05;FREQ_HOMALT=0.0;FREQ_HOMREF=0.9999029973809292;N_BI_GENOS=10309;Southeast_Han_AN=1908;Southeast_Han_AC=1;So
chr2	98413935	.	T	<INS:ME:LINE1>	.	PASS	SVTYPE=INS:ME:LINE1;END=98413935;SVLEN=0;AN=20618;AC=22;AF=0.001067028809777864;N_HET=18;N_HOMALT=2;N_HOMREF=10289;FREQ_HET=0.0017460471432728683;FREQ_HOMALT=0.0001940052381414298;FREQ_HOMREF=0.9980599476185857;N_BI_GENOS=10309;South_Han_AN=1496;South_Ha
chr2	98847508	.	C	<INS:ME:LINE1>	.	PASS	SVTYPE=INS:ME:LINE1;END=98847525;SVLEN=2505;AN=20618;AC=1;AF=4.850130953535746e-05;N_HET=1;N_HOMALT=0;N_HOMREF=10308;FREQ_HET=9.700261907071492e-05;FREQ_HOMALT=0.0;FREQ_HOMREF=0.9999029973809292;N_BI_GENOS=10309;East_Han_AN=6554;East_Han_AC=1;East_Han_AF=
chr2	98969309	.	T	<INS:ME:LINE1>	.	PASS	SVTYPE=INS:ME:LINE1;END=98969309;SVLEN=1315;AN=20618;AC=1;AF=4.850130953535746e-05;N_HET=1;N_HOMALT=0;N_HOMREF=10308;FREQ_HET=9.700261907071492e-05;FREQ_HOMALT=0.0;FREQ_HOMREF=0.9999029973809292;N_BI_GENOS=10309;East_Han_AN=6554;East_Han_AC=1;East_Han_AF=
chr2	99088282	.	A	<INS:ME:LINE1>	.	PASS	SVTYPE=INS:ME:LINE1;END=99088298;SVLEN=1651;AN=20618;AC=1;AF=4.850130953535746e-05;N_HET=1;N_HOMALT=0;N_HOMREF=10308;FREQ_HET=9.700261907071492e-05;FREQ_HOMALT=0.0;FREQ_HOMREF=0.9999029973809292;N_BI_GENOS=10309;Central_Han_AN=2128;Central_Han_AC=1;Centra
chr2	99591132	.	A	<INS:ME:LINE1>	.	PASS	SVTYPE=INS:ME:LINE1;END=99591147;SVLEN=6002;AN=20618;AC=2;AF=9.700261907071492e-05;N_HET=2;N_HOMALT=0;N_HOMREF=10307;FREQ_HET=0.0001940052381414298;FREQ_HOMALT=0.0;FREQ_HOMREF=0.9998059947618586;N_BI_GENOS=10309;Northwest_Han_AN=1296;Northwest_Han_AC=1;N
chr2	99750204	.	A	<INS:ME:LINE1>	.	PASS	SVTYPE=INS:ME:LINE1;END=99750218;SVLEN=6000;AN=20618;AC=4;AF=0.0001940052381414298;N_HET=4;N_HOMALT=0;N_HOMREF=10305;FREQ_HET=0.00038801047628285966;FREQ_HOMALT=0.0;FREQ_HOMREF=0.9996119895237171;N_BI_GENOS=10309;East_Han_AN=6554;East_Han_AC=4;East_Han_A
chr2	99760308	.	T	<INS:ME:LINE1>	.	PASS	SVTYPE=INS:ME:LINE1;END=99760325;SVLEN=180;AN=20618;AC=1;AF=4.850130953535746e-05;N_HET=1;N_HOMALT=0;N_HOMREF=10308;FREQ_HET=9.700261907071492e-05;FREQ_HOMALT=0.0;FREQ_HOMREF=0.9999029973809292;N_BI_GENOS=10309;Central_Han_AN=2128;Central_Han_AC=1;Central
chr2	99785170	.	T	<INS:ME:LINE1>	.	PASS	SVTYPE=INS:ME:LINE1;END=99785186;SVLEN=237;AN=20618;AC=1;AF=4.850130953535746e-05;N_HET=1;N_HOMALT=0;N_HOMREF=10308;FREQ_HET=9.700261907071492e-05;FREQ_HOMALT=0.0;FREQ_HOMREF=0.9999029973809292;N_BI_GENOS=10309;East_Han_AN=6554;East_Han_AC=1;East_Han_AF=0
chr2	99851403	.	A	<INS:ME:LINE1>	.	PASS	SVTYPE=INS:ME:LINE1;END=99851413;SVLEN=1095;AN=20618;AC=1;AF=4.850130953535746e-05;N_HET=1;N_HOMALT=0;N_HOMREF=10308;FREQ_HET=9.700261907071492e-05;FREQ_HOMALT=0.0;FREQ_HOMREF=0.9999029973809292;N_BI_GENOS=10309;Mongolian_AN=454;Mongolian_AC=1;Mongolian_A
chr2	99918213	.	A	<INS:ME:LINE1>	.	PASS	SVTYPE=INS:ME:LINE1;END=99918227;SVLEN=2393;AN=20618;AC=1;AF=4.850130953535746e-05;N_HET=1;N_HOMALT=0;N_HOMREF=10308;FREQ_HET=9.700261907071492e-05;FREQ_HOMALT=0.0;FREQ_HOMREF=0.9999029973809292;N_BI_GENOS=10309;East_Han_AN=6554;East_Han_AC=1;East_Han_AF=
chr2	99994103	.	C	<INS:ME:LINE1>	.	PASS	SVTYPE=INS:ME:LINE1;END=99994121;SVLEN=6000;AN=20618;AC=1;AF=4.850130953535746e-05;N_HET=1;N_HOMALT=0;N_HOMREF=10308;FREQ_HET=9.700261907071492e-05;FREQ_HOMALT=0.0;FREQ_HOMREF=0.9999029973809292;N_BI_GENOS=10309;Central_Han_AN=2128;Central_Han_AC=1;Centra
chr2	100196621	.	T	<INS:ME:LINE1>	.	PASS	SVTYPE=INS:ME:LINE1;END=100196635;SVLEN=395;AN=20618;AC=1;AF=4.850130953535746e-05;N_HET=1;N_HOMALT=0;N_HOMREF=10308;FREQ_HET=9.700261907071492e-05;FREQ_HOMALT=0.0;FREQ_HOMREF=0.9999029973809292;N_BI_GENOS=10309;East_Han_AN=6554;East_Han_AC=1;East_Han_AF=
chr2	100335320	.	G	<INS:ME:LINE1>	.	PASS	SVTYPE=INS:ME:LINE1;END=100335333;SVLEN=5998;AN=20618;AC=2;AF=9.700261907071492e-05;N_HET=2;N_HOMALT=0;N_HOMREF=10307;FREQ_HET=0.0001940052381414298;FREQ_HOMALT=0.0;FREQ_HOMREF=0.9998059947618586;N_BI_GENOS=10309;South_Han_AN=1496;South_Han_AC=2;South_Ha
chr2	100472405	.	T	<INS:ME:LINE1>	.	PASS	SVTYPE=INS:ME:LINE1;END=100472420;SVLEN=6026;AN=20618;AC=3;AF=0.00014550392860607236;N_HET=3;N_HOMALT=0;N_HOMREF=10306;FREQ_HET=0.0002910078572121447;FREQ_HOMALT=0.0;FREQ_HOMREF=0.9997089921427879;N_BI_GENOS=10309;Tibetan_AN=414;Tibetan_AC=3;Tibetan_AF=0.
chr2	100575626	.	A	<INS:ME:LINE1>	.	PASS	SVTYPE=INS:ME:LINE1;END=100575637;SVLEN=5996;AN=20618;AC=2;AF=9.700261907071492e-05;N_HET=2;N_HOMALT=0;N_HOMREF=10307;FREQ_HET=0.0001940052381414298;FREQ_HOMALT=0.0;FREQ_HOMREF=0.9998059947618586;N_BI_GENOS=10309;Northwest_Han_AN=1296;Northwest_Han_AC=1;
chr2	100754260	.	G	<INS:ME:LINE1>	.	PASS	SVTYPE=INS:ME:LINE1;END=100754273;SVLEN=6009;AN=20618;AC=2;AF=9.700261907071492e-05;N_HET=2;N_HOMALT=0;N_HOMREF=10307;FREQ_HET=0.0001940052381414298;FREQ_HOMALT=0.0;FREQ_HOMREF=0.9998059947618586;N_BI_GENOS=10309;South_Han_AN=1496;South_Han_AC=1;South_Ha
chr2	100867570	.	C	<INS:ME:LINE1>	.	PASS	SVTYPE=INS:ME:LINE1;END=100867585;SVLEN=6006;AN=20618;AC=1;AF=4.850130953535746e-05;N_HET=1;N_HOMALT=0;N_HOMREF=10308;FREQ_HET=9.700261907071492e-05;FREQ_HOMALT=0.0;FREQ_HOMREF=0.9999029973809292;N_BI_GENOS=10309;Southeast_Han_AN=1908;Southeast_Han_AC=1;S
chr2	101368896	.	A	<INS:ME:LINE1>	.	PASS	SVTYPE=INS:ME:LINE1;END=101368913;SVLEN=1097;AN=20618;AC=2;AF=9.700261907071492e-05;N_HET=2;N_HOMALT=0;N_HOMREF=10307;FREQ_HET=0.0001940052381414298;FREQ_HOMALT=0.0;FREQ_HOMREF=0.9998059947618586;N_BI_GENOS=10309;Lingnan_Han_AN=1888;Lingnan_Han_AC=1;Ling
chr2	101780161	.	A	<INS:ME:LINE1>	.	PASS	SVTYPE=INS:ME:LINE1;END=101780175;SVLEN=5999;AN=20618;AC=1;AF=4.850130953535746e-05;N_HET=1;N_HOMALT=0;N_HOMREF=10308;FREQ_HET=9.700261907071492e-05;FREQ_HOMALT=0.0;FREQ_HOMREF=0.9999029973809292;N_BI_GENOS=10309;Southeast_Han_AN=1908;Southeast_Han_AC=1;S
chr2	101899518	.	A	<INS:ME:LINE1>	.	PASS	SVTYPE=INS:ME:LINE1;END=101899535;SVLEN=5958;AN=20618;AC=23;AF=0.0011155301193132214;N_HET=19;N_HOMALT=2;N_HOMREF=10288;FREQ_HET=0.0018430497623435833;FREQ_HOMALT=0.0001940052381414298;FREQ_HOMREF=0.997962944999515;N_BI_GENOS=10309;South_Han_AN=1496;Sout
chr2	102031124	.	C	<INS:ME:LINE1>	.	PASS	SVTYPE=INS:ME:LINE1;END=102031140;SVLEN=341;AN=20618;AC=1;AF=4.850130953535746e-05;N_HET=1;N_HOMALT=0;N_HOMREF=10308;FREQ_HET=9.700261907071492e-05;FREQ_HOMALT=0.0;FREQ_HOMREF=0.9999029973809292;N_BI_GENOS=10309;Hui_AN=444;Hui_AC=1;Hui_AF=0.00225225225225
chr2	102295972	.	A	<INS:ME:LINE1>	.	PASS	SVTYPE=INS:ME:LINE1;END=102295990;SVLEN=5997;AN=20618;AC=13;AF=0.0006305170239596469;N_HET=13;N_HOMALT=0;N_HOMREF=10296;FREQ_HET=0.0012610340479192938;FREQ_HOMALT=0.0;FREQ_HOMREF=0.9987389659520807;N_BI_GENOS=10309;South_Han_AN=1496;South_Han_AC=1;South_H
chr2	102811126	.	A	<INS:ME:LINE1>	.	PASS	SVTYPE=INS:ME:LINE1;END=102811139;SVLEN=848;AN=20618;AC=1;AF=4.850130953535746e-05;N_HET=1;N_HOMALT=0;N_HOMREF=10308;FREQ_HET=9.700261907071492e-05;FREQ_HOMALT=0.0;FREQ_HOMREF=0.9999029973809292;N_BI_GENOS=10309;Central_Han_AN=2128;Central_Han_AC=1;Centra
chr2	103611112	.	A	<INS:ME:LINE1>	.	PASS	SVTYPE=INS:ME:LINE1;END=103611129;SVLEN=504;AN=20618;AC=1;AF=4.850130953535746e-05;N_HET=1;N_HOMALT=0;N_HOMREF=10308;FREQ_HET=9.700261907071492e-05;FREQ_HOMALT=0.0;FREQ_HOMREF=0.9999029973809292;N_BI_GENOS=10309;East_Han_AN=6554;East_Han_AC=1;East_Han_AF=
chr2	103721426	.	T	<INS:ME:LINE1>	.	PASS	SVTYPE=INS:ME:LINE1;END=103721426;SVLEN=200;AN=20618;AC=1;AF=4.850130953535746e-05;N_HET=1;N_HOMALT=0;N_HOMREF=10308;FREQ_HET=9.700261907071492e-05;FREQ_HOMALT=0.0;FREQ_HOMREF=0.9999029973809292;N_BI_GENOS=10309;East_Han_AN=6554;East_Han_AC=1;East_Han_AF=
chr2	104344766	.	G	<INS:ME:LINE1>	.	PASS	SVTYPE=INS:ME:LINE1;END=104344784;SVLEN=1114;AN=20618;AC=1;AF=4.850130953535746e-05;N_HET=1;N_HOMALT=0;N_HOMREF=10308;FREQ_HET=9.700261907071492e-05;FREQ_HOMALT=0.0;FREQ_HOMREF=0.9999029973809292;N_BI_GENOS=10309;Southeast_Han_AN=1908;Southeast_Han_AC=1;S
chr2	104706294	.	A	<INS:ME:LINE1>	.	PASS	SVTYPE=INS:ME:LINE1;END=104706309;SVLEN=3020;AN=20618;AC=1;AF=4.850130953535746e-05;N_HET=1;N_HOMALT=0;N_HOMREF=10308;FREQ_HET=9.700261907071492e-05;FREQ_HOMALT=0.0;FREQ_HOMREF=0.9999029973809292;N_BI_GENOS=10309;East_Han_AN=6554;East_Han_AC=1;East_Han_AF
chr2	105469091	.	A	<INS:ME:LINE1>	.	PASS	SVTYPE=INS:ME:LINE1;END=105469105;SVLEN=5953;AN=20618;AC=2;AF=9.700261907071492e-05;N_HET=2;N_HOMALT=0;N_HOMREF=10307;FREQ_HET=0.0001940052381414298;FREQ_HOMALT=0.0;FREQ_HOMREF=0.9998059947618586;N_BI_GENOS=10309;North_Han_AN=2272;North_Han_AC=1;North_Ha
chr2	105546701	.	A	<INS:ME:LINE1>	.	PASS	SVTYPE=INS:ME:LINE1;END=105546707;SVLEN=424;AN=20618;AC=1478;AF=0.07168493549325831;N_HET=1294;N_HOMALT=92;N_HOMREF=8923;FREQ_HET=0.1255213890775051;FREQ_HOMALT=0.008924240954505771;FREQ_HOMREF=0.8655543699679892;N_BI_GENOS=10309;South_Han_AN=1496;South_H
chr2	105945025	.	T	<INS:ME:LINE1>	.	PASS	SVTYPE=INS:ME:LINE1;END=105945026;SVLEN=363;AN=20618;AC=8;AF=0.00038801047628285966;N_HET=8;N_HOMALT=0;N_HOMREF=10301;FREQ_HET=0.0007760209525657193;FREQ_HOMALT=0.0;FREQ_HOMREF=0.9992239790474343;N_BI_GENOS=10309;South_Han_AN=1496;South_Han_AC=3;South_Han
chr2	106249007	.	G	<INS:ME:LINE1>	.	PASS	SVTYPE=INS:ME:LINE1;END=106249020;SVLEN=632;AN=20618;AC=13;AF=0.0006305170239596469;N_HET=9;N_HOMALT=2;N_HOMREF=10298;FREQ_HET=0.0008730235716364342;FREQ_HOMALT=0.0001940052381414298;FREQ_HOMREF=0.9989329711902222;N_BI_GENOS=10309;South_Han_AN=1496;South
chr2	106665654	.	G	<INS:ME:LINE1>	.	PASS	SVTYPE=INS:ME:LINE1;END=106665665;SVLEN=6003;AN=20618;AC=1;AF=4.850130953535746e-05;N_HET=1;N_HOMALT=0;N_HOMREF=10308;FREQ_HET=9.700261907071492e-05;FREQ_HOMALT=0.0;FREQ_HOMREF=0.9999029973809292;N_BI_GENOS=10309;Lingnan_Han_AN=1888;Lingnan_Han_AC=1;Lingn
chr2	106881306	.	A	<INS:ME:LINE1>	.	PASS	SVTYPE=INS:ME:LINE1;END=106881322;SVLEN=710;AN=20618;AC=1;AF=4.850130953535746e-05;N_HET=1;N_HOMALT=0;N_HOMREF=10308;FREQ_HET=9.700261907071492e-05;FREQ_HOMALT=0.0;FREQ_HOMREF=0.9999029973809292;N_BI_GENOS=10309;East_Han_AN=6554;East_Han_AC=1;East_Han_AF=
chr2	107247253	.	A	<INS:ME:LINE1>	.	PASS	SVTYPE=INS:ME:LINE1;END=107247268;SVLEN=1242;AN=20618;AC=2;AF=9.700261907071492e-05;N_HET=2;N_HOMALT=0;N_HOMREF=10307;FREQ_HET=0.0001940052381414298;FREQ_HOMALT=0.0;FREQ_HOMREF=0.9998059947618586;N_BI_GENOS=10309;East_Han_AN=6554;East_Han_AC=2;East_Han_A
chr2	107354359	.	T	<INS:ME:LINE1>	.	PASS	SVTYPE=INS:ME:LINE1;END=107354359;SVLEN=1144;AN=20618;AC=1;AF=4.850130953535746e-05;N_HET=1;N_HOMALT=0;N_HOMREF=10308;FREQ_HET=9.700261907071492e-05;FREQ_HOMALT=0.0;FREQ_HOMREF=0.9999029973809292;N_BI_GENOS=10309;East_Han_AN=6554;East_Han_AC=1;East_Han_AF
chr2	107557053	.	A	<INS:ME:LINE1>	.	PASS	SVTYPE=INS:ME:LINE1;END=107557070;SVLEN=191;AN=20618;AC=1;AF=4.850130953535746e-05;N_HET=1;N_HOMALT=0;N_HOMREF=10308;FREQ_HET=9.700261907071492e-05;FREQ_HOMALT=0.0;FREQ_HOMREF=0.9999029973809292;N_BI_GENOS=10309;South_Han_AN=1496;South_Han_AC=1;South_Han_
chr2	108048154	.	G	<INS:ME:LINE1>	.	PASS	SVTYPE=INS:ME:LINE1;END=108048168;SVLEN=6050;AN=20618;AC=1;AF=4.850130953535746e-05;N_HET=1;N_HOMALT=0;N_HOMREF=10308;FREQ_HET=9.700261907071492e-05;FREQ_HOMALT=0.0;FREQ_HOMREF=0.9999029973809292;N_BI_GENOS=10309;Hui_AN=444;Hui_AC=1;Hui_AF=0.0022522522522
chr2	108156984	.	A	<INS:ME:LINE1>	.	PASS	SVTYPE=INS:ME:LINE1;END=108157001;SVLEN=5998;AN=20618;AC=2;AF=9.700261907071492e-05;N_HET=0;N_HOMALT=1;N_HOMREF=10308;FREQ_HET=0.0;FREQ_HOMALT=9.700261907071492e-05;FREQ_HOMREF=0.9999029973809292;N_BI_GENOS=10309;Central_Han_AN=2128;Central_Han_AC=2;Centr
chr2	108252953	.	A	<INS:ME:LINE1>	.	PASS	SVTYPE=INS:ME:LINE1;END=108252970;SVLEN=5993;AN=20618;AC=1;AF=4.850130953535746e-05;N_HET=1;N_HOMALT=0;N_HOMREF=10308;FREQ_HET=9.700261907071492e-05;FREQ_HOMALT=0.0;FREQ_HOMREF=0.9999029973809292;N_BI_GENOS=10309;East_Han_AN=6554;East_Han_AC=1;East_Han_AF
chr2	108458275	.	G	<INS:ME:LINE1>	.	PASS	SVTYPE=INS:ME:LINE1;END=108458291;SVLEN=6082;AN=20618;AC=2;AF=9.700261907071492e-05;N_HET=2;N_HOMALT=0;N_HOMREF=10307;FREQ_HET=0.0001940052381414298;FREQ_HOMALT=0.0;FREQ_HOMREF=0.9998059947618586;N_BI_GENOS=10309;South_Han_AN=1496;South_Han_AC=1;South_Ha
chr2	108851855	.	A	<INS:ME:LINE1>	.	PASS	SVTYPE=INS:ME:LINE1;END=108851863;SVLEN=1498;AN=20618;AC=2;AF=9.700261907071492e-05;N_HET=2;N_HOMALT=0;N_HOMREF=10307;FREQ_HET=0.0001940052381414298;FREQ_HOMALT=0.0;FREQ_HOMREF=0.9998059947618586;N_BI_GENOS=10309;Miao_AN=482;Miao_AC=2;Miao_AF=0.004149377
chr2	109096368	.	A	<INS:ME:LINE1>	.	PASS	SVTYPE=INS:ME:LINE1;END=109096384;SVLEN=6000;AN=20618;AC=1;AF=4.850130953535746e-05;N_HET=1;N_HOMALT=0;N_HOMREF=10308;FREQ_HET=9.700261907071492e-05;FREQ_HOMALT=0.0;FREQ_HOMREF=0.9999029973809292;N_BI_GENOS=10309;Southeast_Han_AN=1908;Southeast_Han_AC=1;S
chr2	109248223	.	A	<INS:ME:LINE1>	.	PASS	SVTYPE=INS:ME:LINE1;END=109248239;SVLEN=6056;AN=20618;AC=2;AF=9.700261907071492e-05;N_HET=2;N_HOMALT=0;N_HOMREF=10307;FREQ_HET=0.0001940052381414298;FREQ_HOMALT=0.0;FREQ_HOMREF=0.9998059947618586;N_BI_GENOS=10309;South_Han_AN=1496;South_Han_AC=1;South_Ha
chr2	109322222	.	A	<INS:ME:LINE1>	.	PASS	SVTYPE=INS:ME:LINE1;END=109322239;SVLEN=1171;AN=20618;AC=1;AF=4.850130953535746e-05;N_HET=1;N_HOMALT=0;N_HOMREF=10308;FREQ_HET=9.700261907071492e-05;FREQ_HOMALT=0.0;FREQ_HOMREF=0.9999029973809292;N_BI_GENOS=10309;South_Han_AN=1496;South_Han_AC=1;South_Han
chr2	110658934	.	G	<INS:ME:LINE1>	.	PASS	SVTYPE=INS:ME:LINE1;END=110658934;SVLEN=103;AN=20618;AC=1;AF=4.850130953535746e-05;N_HET=1;N_HOMALT=0;N_HOMREF=10308;FREQ_HET=9.700261907071492e-05;FREQ_HOMALT=0.0;FREQ_HOMREF=0.9999029973809292;N_BI_GENOS=10309;East_Han_AN=6554;East_Han_AC=1;East_Han_AF=
chr2	111480979	.	A	<INS:ME:LINE1>	.	PASS	SVTYPE=INS:ME:LINE1;END=111480980;SVLEN=335;AN=20618;AC=2;AF=9.700261907071492e-05;N_HET=0;N_HOMALT=1;N_HOMREF=10308;FREQ_HET=0.0;FREQ_HOMALT=9.700261907071492e-05;FREQ_HOMREF=0.9999029973809292;N_BI_GENOS=10309;Southeast_Han_AN=1908;Southeast_Han_AC=2;So
chr2	111744039	.	C	<INS:ME:LINE1>	.	PASS	SVTYPE=INS:ME:LINE1;END=111744055;SVLEN=6006;AN=20618;AC=1;AF=4.850130953535746e-05;N_HET=1;N_HOMALT=0;N_HOMREF=10308;FREQ_HET=9.700261907071492e-05;FREQ_HOMALT=0.0;FREQ_HOMREF=0.9999029973809292;N_BI_GENOS=10309;East_Han_AN=6554;East_Han_AC=1;East_Han_AF
chr2	112197275	.	G	<INS:ME:LINE1>	.	PASS	SVTYPE=INS:ME:LINE1;END=112197275;SVLEN=0;AN=20618;AC=1;AF=4.850130953535746e-05;N_HET=1;N_HOMALT=0;N_HOMREF=10308;FREQ_HET=9.700261907071492e-05;FREQ_HOMALT=0.0;FREQ_HOMREF=0.9999029973809292;N_BI_GENOS=10309;South_Han_AN=1496;South_Han_AC=1;South_Han_AF
chr2	112323760	.	A	<INS:ME:LINE1>	.	PASS	SVTYPE=INS:ME:LINE1;END=112323775;SVLEN=342;AN=20618;AC=8;AF=0.00038801047628285966;N_HET=8;N_HOMALT=0;N_HOMREF=10301;FREQ_HET=0.0007760209525657193;FREQ_HOMALT=0.0;FREQ_HOMREF=0.9992239790474343;N_BI_GENOS=10309;Northwest_Han_AN=1296;Northwest_Han_AC=1;N
chr2	112672510	.	A	<INS:ME:LINE1>	.	PASS	SVTYPE=INS:ME:LINE1;END=112672514;SVLEN=387;AN=20618;AC=20;AF=0.0009700261907071491;N_HET=18;N_HOMALT=1;N_HOMREF=10290;FREQ_HET=0.0017460471432728683;FREQ_HOMALT=9.700261907071492e-05;FREQ_HOMREF=0.9981569502376564;N_BI_GENOS=10309;South_Han_AN=1496;South
chr2	112980378	.	A	<INS:ME:LINE1>	.	PASS	SVTYPE=INS:ME:LINE1;END=112980392;SVLEN=914;AN=20618;AC=1;AF=4.850130953535746e-05;N_HET=1;N_HOMALT=0;N_HOMREF=10308;FREQ_HET=9.700261907071492e-05;FREQ_HOMALT=0.0;FREQ_HOMREF=0.9999029973809292;N_BI_GENOS=10309;Northwest_Han_AN=1296;Northwest_Han_AC=1;No
chr2	113042359	.	A	<INS:ME:LINE1>	.	PASS	SVTYPE=INS:ME:LINE1;END=113042374;SVLEN=6040;AN=20618;AC=2;AF=9.700261907071492e-05;N_HET=2;N_HOMALT=0;N_HOMREF=10307;FREQ_HET=0.0001940052381414298;FREQ_HOMALT=0.0;FREQ_HOMREF=0.9998059947618586;N_BI_GENOS=10309;South_Han_AN=1496;South_Han_AC=1;South_Ha
chr2	114094385	.	A	<INS:ME:LINE1>	.	PASS	SVTYPE=INS:ME:LINE1;END=114094398;SVLEN=1577;AN=20618;AC=2;AF=9.700261907071492e-05;N_HET=2;N_HOMALT=0;N_HOMREF=10307;FREQ_HET=0.0001940052381414298;FREQ_HOMALT=0.0;FREQ_HOMREF=0.9998059947618586;N_BI_GENOS=10309;East_Han_AN=6554;East_Han_AC=2;East_Han_A
chr2	114106263	.	C	<INS:ME:LINE1>	.	PASS	SVTYPE=INS:ME:LINE1;END=114106278;SVLEN=1889;AN=20618;AC=1;AF=4.850130953535746e-05;N_HET=1;N_HOMALT=0;N_HOMREF=10308;FREQ_HET=9.700261907071492e-05;FREQ_HOMALT=0.0;FREQ_HOMREF=0.9999029973809292;N_BI_GENOS=10309;Lingnan_Han_AN=1888;Lingnan_Han_AC=1;Lingn
chr2	114163639	.	C	<INS:ME:LINE1>	.	PASS	SVTYPE=INS:ME:LINE1;END=114163654;SVLEN=239;AN=20618;AC=3;AF=0.00014550392860607236;N_HET=3;N_HOMALT=0;N_HOMREF=10306;FREQ_HET=0.0002910078572121447;FREQ_HOMALT=0.0;FREQ_HOMREF=0.9997089921427879;N_BI_GENOS=10309;South_Han_AN=1496;South_Han_AC=1;South_Han
chr2	114216243	.	A	<INS:ME:LINE1>	.	PASS	SVTYPE=INS:ME:LINE1;END=114216258;SVLEN=2523;AN=20618;AC=1;AF=4.850130953535746e-05;N_HET=1;N_HOMALT=0;N_HOMREF=10308;FREQ_HET=9.700261907071492e-05;FREQ_HOMALT=0.0;FREQ_HOMREF=0.9999029973809292;N_BI_GENOS=10309;Central_Han_AN=2128;Central_Han_AC=1;Centr
chr2	114220432	.	C	<INS:ME:LINE1>	.	PASS	SVTYPE=INS:ME:LINE1;END=114220449;SVLEN=6002;AN=20618;AC=1;AF=4.850130953535746e-05;N_HET=1;N_HOMALT=0;N_HOMREF=10308;FREQ_HET=9.700261907071492e-05;FREQ_HOMALT=0.0;FREQ_HOMREF=0.9999029973809292;N_BI_GENOS=10309;Northwest_Han_AN=1296;Northwest_Han_AC=1;N
chr2	114240346	.	A	<INS:ME:LINE1>	.	PASS	SVTYPE=INS:ME:LINE1;END=114240364;SVLEN=78;AN=20618;AC=13;AF=0.0006305170239596469;N_HET=9;N_HOMALT=2;N_HOMREF=10298;FREQ_HET=0.0008730235716364342;FREQ_HOMALT=0.0001940052381414298;FREQ_HOMREF=0.9989329711902222;N_BI_GENOS=10309;Northwest_Han_AN=1296;No
chr2	114469427	.	A	<INS:ME:LINE1>	.	PASS	SVTYPE=INS:ME:LINE1;END=114469430;SVLEN=707;AN=20618;AC=1;AF=4.850130953535746e-05;N_HET=1;N_HOMALT=0;N_HOMREF=10308;FREQ_HET=9.700261907071492e-05;FREQ_HOMALT=0.0;FREQ_HOMREF=0.9999029973809292;N_BI_GENOS=10309;East_Han_AN=6554;East_Han_AC=1;East_Han_AF=
chr2	114685281	.	A	<INS:ME:LINE1>	.	PASS	SVTYPE=INS:ME:LINE1;END=114685292;SVLEN=452;AN=20618;AC=1;AF=4.850130953535746e-05;N_HET=1;N_HOMALT=0;N_HOMREF=10308;FREQ_HET=9.700261907071492e-05;FREQ_HOMALT=0.0;FREQ_HOMREF=0.9999029973809292;N_BI_GENOS=10309;North_Han_AN=2272;North_Han_AC=1;North_Han_
chr2	114709295	.	T	<INS:ME:LINE1>	.	PASS	SVTYPE=INS:ME:LINE1;END=114709297;SVLEN=95;AN=20618;AC=1;AF=4.850130953535746e-05;N_HET=1;N_HOMALT=0;N_HOMREF=10308;FREQ_HET=9.700261907071492e-05;FREQ_HOMALT=0.0;FREQ_HOMREF=0.9999029973809292;N_BI_GENOS=10309;Southeast_Han_AN=1908;Southeast_Han_AC=1;Sou
chr2	115289493	.	A	<INS:ME:LINE1>	.	PASS	SVTYPE=INS:ME:LINE1;END=115289519;SVLEN=1741;AN=20618;AC=2;AF=9.700261907071492e-05;N_HET=0;N_HOMALT=1;N_HOMREF=10308;FREQ_HET=0.0;FREQ_HOMALT=9.700261907071492e-05;FREQ_HOMREF=0.9999029973809292;N_BI_GENOS=10309;East_Han_AN=6554;East_Han_AC=2;East_Han_AF
chr2	115753435	.	A	<INS:ME:LINE1>	.	PASS	SVTYPE=INS:ME:LINE1;END=115753451;SVLEN=1245;AN=20618;AC=1;AF=4.850130953535746e-05;N_HET=1;N_HOMALT=0;N_HOMREF=10308;FREQ_HET=9.700261907071492e-05;FREQ_HOMALT=0.0;FREQ_HOMREF=0.9999029973809292;N_BI_GENOS=10309;Central_Han_AN=2128;Central_Han_AC=1;Centr
chr2	115770540	.	T	<INS:ME:LINE1>	.	PASS	SVTYPE=INS:ME:LINE1;END=115770542;SVLEN=4094;AN=20618;AC=1;AF=4.850130953535746e-05;N_HET=1;N_HOMALT=0;N_HOMREF=10308;FREQ_HET=9.700261907071492e-05;FREQ_HOMALT=0.0;FREQ_HOMREF=0.9999029973809292;N_BI_GENOS=10309;Northwest_Han_AN=1296;Northwest_Han_AC=1;N
chr2	116244204	.	A	<INS:ME:LINE1>	.	PASS	SVTYPE=INS:ME:LINE1;END=116244221;SVLEN=1488;AN=20618;AC=2;AF=9.700261907071492e-05;N_HET=2;N_HOMALT=0;N_HOMREF=10307;FREQ_HET=0.0001940052381414298;FREQ_HOMALT=0.0;FREQ_HOMREF=0.9998059947618586;N_BI_GENOS=10309;Yi_AN=422;Yi_AC=2;Yi_AF=0.004739336492890
chr2	116253847	.	A	<INS:ME:LINE1>	.	PASS	SVTYPE=INS:ME:LINE1;END=116253854;SVLEN=1658;AN=20618;AC=2;AF=9.700261907071492e-05;N_HET=2;N_HOMALT=0;N_HOMREF=10307;FREQ_HET=0.0001940052381414298;FREQ_HOMALT=0.0;FREQ_HOMREF=0.9998059947618586;N_BI_GENOS=10309;Central_Han_AN=2128;Central_Han_AC=2;Cent
chr2	116395315	.	A	<INS:ME:LINE1>	.	PASS	SVTYPE=INS:ME:LINE1;END=116395331;SVLEN=216;AN=20618;AC=3;AF=0.00014550392860607236;N_HET=3;N_HOMALT=0;N_HOMREF=10306;FREQ_HET=0.0002910078572121447;FREQ_HOMALT=0.0;FREQ_HOMREF=0.9997089921427879;N_BI_GENOS=10309;East_Han_AN=6554;East_Han_AC=3;East_Han_AF
chr2	116468315	.	G	<INS:ME:LINE1>	.	PASS	SVTYPE=INS:ME:LINE1;END=116468329;SVLEN=5988;AN=20618;AC=21;AF=0.0010185275002425065;N_HET=19;N_HOMALT=1;N_HOMREF=10289;FREQ_HET=0.0018430497623435833;FREQ_HOMALT=9.700261907071492e-05;FREQ_HOMREF=0.9980599476185857;N_BI_GENOS=10309;South_Han_AN=1496;Sout
chr2	116503395	.	A	<INS:ME:LINE1>	.	PASS	SVTYPE=INS:ME:LINE1;END=116503406;SVLEN=663;AN=20618;AC=1;AF=4.850130953535746e-05;N_HET=1;N_HOMALT=0;N_HOMREF=10308;FREQ_HET=9.700261907071492e-05;FREQ_HOMALT=0.0;FREQ_HOMREF=0.9999029973809292;N_BI_GENOS=10309;North_Han_AN=2272;North_Han_AC=1;North_Han_

chr2	116617423 .	A	<INS:ME:LINE1>	.	PASS	SVTYPE=INS:ME:LINE1;END=116617425;SVLEN=1131;AN=20618;AC=2;AF=9.700261907071492e-05;N_HET=2;N_HOMALT=0;N_HOMREF=10307;FREQ_HET=0.00019400523814142983;FREQ_HOMALT=0.0;FREQ_HOMREF=0.9998059947618586;N_BI_GENOS=10309;East_Han_AN=6554;East_Han_AC=1;East_Han_A
chr2	116804500 .	G	<INS:ME:LINE1>	.	PASS	SVTYPE=INS:ME:LINE1;END=116804514;SVLEN=611;AN=20618;AC=1;AF=4.850130953535746e-05;N_HET=1;N_HOMALT=0;N_HOMREF=10308;FREQ_HET=9.700261907071492e-05;FREQ_HOMALT=0.0;FREQ_HOMREF=0.9999029973809292;N_BI_GENOS=10309;North_Han_AN=2272;North_Han_AC=1;North_Han_
chr2	117340606 .	A	<INS:ME:LINE1>	.	PASS	SVTYPE=INS:ME:LINE1;END=117340622;SVLEN=1849;AN=20618;AC=1;AF=4.850130953535746e-05;N_HET=1;N_HOMALT=0;N_HOMREF=10308;FREQ_HET=9.700261907071492e-05;FREQ_HOMALT=0.0;FREQ_HOMREF=0.9999029973809292;N_BI_GENOS=10309;South_Han_AN=1496;South_Han_AC=1;South_Han
chr2	117348683 .	C	<INS:ME:LINE1>	.	PASS	SVTYPE=INS:ME:LINE1;END=117348687;SVLEN=134;AN=20618;AC=1;AF=4.850130953535746e-05;N_HET=1;N_HOMALT=0;N_HOMREF=10308;FREQ_HET=9.700261907071492e-05;FREQ_HOMALT=0.0;FREQ_HOMREF=0.9999029973809292;N_BI_GENOS=10309;East_Han_AN=6554;East_Han_AC=1;East_Han_AF=
chr2	117363999 .	C	<INS:ME:LINE1>	.	PASS	SVTYPE=INS:ME:LINE1;END=117363999;SVLEN=716;AN=20618;AC=1;AF=4.850130953535746e-05;N_HET=1;N_HOMALT=0;N_HOMREF=10308;FREQ_HET=9.700261907071492e-05;FREQ_HOMALT=0.0;FREQ_HOMREF=0.9999029973809292;N_BI_GENOS=10309;East_Han_AN=6554;East_Han_AC=1;East_Han_AF=
chr2	117371412 .	A	<INS:ME:LINE1>	.	PASS	SVTYPE=INS:ME:LINE1;END=117371432;SVLEN=1952;AN=20618;AC=1;AF=4.850130953535746e-05;N_HET=1;N_HOMALT=0;N_HOMREF=10308;FREQ_HET=9.700261907071492e-05;FREQ_HOMALT=0.0;FREQ_HOMREF=0.9999029973809292;N_BI_GENOS=10309;Southeast_Han_AN=1908;Southeast_Han_AC=1;S
chr2	117717176 .	T	<INS:ME:LINE1>	.	PASS	SVTYPE=INS:ME:LINE1;END=117717193;SVLEN=5989;AN=20618;AC=2;AF=9.700261907071492e-05;N_HET=2;N_HOMALT=0;N_HOMREF=10307;FREQ_HET=0.00019400523814142983;FREQ_HOMALT=0.0;FREQ_HOMREF=0.9998059947618586;N_BI_GENOS=10309;Lingnan_Han_AN=1888;Lingnan_Han_AC=1;Ling
chr2	117955610 .	C	<INS:ME:LINE1>	.	PASS	SVTYPE=INS:ME:LINE1;END=117955610;SVLEN=68;AN=20618;AC=1;AF=4.850130953535746e-05;N_HET=1;N_HOMALT=0;N_HOMREF=10308;FREQ_HET=9.700261907071492e-05;FREQ_HOMALT=0.0;FREQ_HOMREF=0.9999029973809292;N_BI_GENOS=10309;East_Han_AN=6554;East_Han_AC=1;East_Han_AF=0
chr2	118557693 .	A	<INS:ME:LINE1>	.	PASS	SVTYPE=INS:ME:LINE1;END=118557711;SVLEN=958;AN=20618;AC=4;AF=0.00019400523814142983;N_HET=4;N_HOMALT=0;N_HOMREF=10305;FREQ_HET=0.0003880104762828596 6;FREQ_HOMALT=0.0;FREQ_HOMREF=0.9996119895237171;N_BI_GENOS=10309;South_Han_AN=1496;South_Han_AC=1;South_Ha
chr2	118606177 .	A	<INS:ME:LINE1>	.	PASS	SVTYPE=INS:ME:LINE1;END=118606192;SVLEN=630;AN=20618;AC=2;AF=9.700261907071492e-05;N_HET=2;N_HOMALT=0;N_HOMREF=10307;FREQ_HET=0.00019400523814142983;FREQ_HOMALT=0.0;FREQ_HOMREF=0.9998059947618586;N_BI_GENOS=10309;Northwest_Han_AN=1296;Northwest_Han_AC=1;N
chr2	118902896 .	A	<INS:ME:LINE1>	.	PASS	SVTYPE=INS:ME:LINE1;END=118902910;SVLEN=5997;AN=20618;AC=1;AF=4.850130953535746e-05;N_HET=1;N_HOMALT=0;N_HOMREF=10308;FREQ_HET=9.700261907071492e-05;FREQ_HOMALT=0.0;FREQ_HOMREF=0.9999029973809292;N_BI_GENOS=10309;Southeast_Han_AN=1908;Southeast_Han_AC=1;S
chr2	119056060 .	T	<INS:ME:LINE1>	.	PASS	SVTYPE=INS:ME:LINE1;END=119056073;SVLEN=6006;AN=20618;AC=1;AF=4.850130953535746e-05;N_HET=1;N_HOMALT=0;N_HOMREF=10308;FREQ_HET=9.700261907071492e-05;FREQ_HOMALT=0.0;FREQ_HOMREF=0.9999029973809292;N_BI_GENOS=10309;Central_Han_AN=2128;Central_Han_AC=1;Centr
chr2	119108858 .	G	<INS:ME:LINE1>	.	PASS	SVTYPE=INS:ME:LINE1;END=119108874;SVLEN=5998;AN=20618;AC=1;AF=4.850130953535746e-05;N_HET=1;N_HOMALT=0;N_HOMREF=10308;FREQ_HET=9.700261907071492e-05;FREQ_HOMALT=0.0;FREQ_HOMREF=0.9999029973809292;N_BI_GENOS=10309;North_Han_AN=2272;North_Han_AC=1;North_Han
chr2	120150231 .	A	<INS:ME:LINE1>	.	PASS	SVTYPE=INS:ME:LINE1;END=120150248;SVLEN=6020;AN=20618;AC=5;AF=0.0002425065476767872 8;N_HET=5;N_HOMALT=0;N_HOMREF=10304;FREQ_HET=0.00048501309535357455;FREQ_HOMALT=0.0;FREQ_HOMREF=0.9995149869046465;N_BI_GENOS=10309;South_Han_AN=1496;South_Han_AC=1;South_H
chr2	121036641 .	C	<INS:ME:LINE1>	.	PASS	SVTYPE=INS:ME:LINE1;END=121036655;SVLEN=274;AN=20618;AC=6;AF=0.0002910078572121447;N_HET=6;N_HOMALT=0;N_HOMREF=10303;FREQ_HET=0.0005820157144242894;FREQ_HOMALT=0.0;FREQ_HOMREF=0.9994179842855757;N_BI_GENOS=10309;South_Han_AN=1496;South_Han_AC=2;South_Han_
chr2	121097137 .	A	<INS:ME:LINE1>	.	PASS	SVTYPE=INS:ME:LINE1;END=121097151;SVLEN=6014;AN=20618;AC=2;AF=9.700261907071492e-05;N_HET=0;N_HOMALT=1;N_HOMREF=10308;FREQ_HET=0.0;FREQ_HOMALT=9.700261907071492e-05;FREQ_HOMREF=0.9999029973809292;N_BI_GENOS=10309;East_Han_AN=6554;East_Han_AC=2;East_Han_AF
chr2	121389599 .	A	<INS:ME:LINE1>	.	PASS	SVTYPE=INS:ME:LINE1;END=121389615;SVLEN=691;AN=20618;AC=1;AF=4.850130953535746e-05;N_HET=1;N_HOMALT=0;N_HOMREF=10308;FREQ_HET=9.700261907071492e-05;FREQ_HOMALT=0.0;FREQ_HOMREF=0.9999029973809292;N_BI_GENOS=10309;North_Han_AN=2272;North_Han_AC=1;North_Han_
chr2	121852262 .	A	<INS:ME:LINE1>	.	PASS	SVTYPE=INS:ME:LINE1;END=121852273;SVLEN=271;AN=20618;AC=1;AF=4.850130953535746e-05;N_HET=1;N_HOMALT=0;N_HOMREF=10308;FREQ_HET=9.700261907071492e-05;FREQ_HOMALT=0.0;FREQ_HOMREF=0.9999029973809292;N_BI_GENOS=10309;North_Han_AN=2272;North_Han_AC=1;North_Han_
chr2	121937161 .	A	<INS:ME:LINE1>	.	PASS	SVTYPE=INS:ME:LINE1;END=121937178;SVLEN=703;AN=20618;AC=3;AF=0.00014550392860607236;N_HET=3;N_HOMALT=0;N_HOMREF=10306;FREQ_HET=0.0002910078572121447;FREQ_HOMALT=0.0;FREQ_HOMREF=0.9997089921427879;N_BI_GENOS=10309;Lingnan_Han_AN=1888;Lingnan_Han_AC=1;Lingn
chr2	121951814 .	A	<INS:ME:LINE1>	.	PASS	SVTYPE=INS:ME:LINE1;END=121951831;SVLEN=5993;AN=20618;AC=1;AF=4.850130953535746e-05;N_HET=1;N_HOMALT=0;N_HOMREF=10308;FREQ_HET=9.700261907071492e-05;FREQ_HOMALT=0.0;FREQ_HOMREF=0.9999029973809292;N_BI_GENOS=10309;Lingnan_Han_AN=1888;Lingnan_Han_AC=1;Lingn
chr2	122213321 .	T	<INS:ME:LINE1>	.	PASS	SVTYPE=INS:ME:LINE1;END=122213337;SVLEN=1623;AN=20618;AC=1;AF=4.850130953535746e-05;N_HET=1;N_HOMALT=0;N_HOMREF=10308;FREQ_HET=9.700261907071492e-05;FREQ_HOMALT=0.0;FREQ_HOMREF=0.9999029973809292;N_BI_GENOS=10309;East_Han_AN=6554;East_Han_AC=1;East_Han_AF
chr2	122483465 .	C	<INS:ME:LINE1>	.	PASS	SVTYPE=INS:ME:LINE1;END=122483484;SVLEN=4458;AN=20618;AC=1;AF=4.850130953535746e-05;N_HET=1;N_HOMALT=0;N_HOMREF=10308;FREQ_HET=9.700261907071492e-05;FREQ_HOMALT=0.0;FREQ_HOMREF=0.9999029973809292;N_BI_GENOS=10309;Lingnan_Han_AN=1888;Lingnan_Han_AC=1;Lingn
chr2	122665069 .	G	<INS:ME:LINE1>	.	PASS	SVTYPE=INS:ME:LINE1;END=122665080;SVLEN=5984;AN=20618;AC=1;AF=4.850130953535746e-05;N_HET=1;N_HOMALT=0;N_HOMREF=10308;FREQ_HET=9.700261907071492e-05;FREQ_HOMALT=0.0;FREQ_HOMREF=0.9999029973809292;N_BI_GENOS=10309;East_Han_AN=6554;East_Han_AC=1;East_Han_AF
chr2	122767041 .	A	<INS:ME:LINE1>	.	PASS	SVTYPE=INS:ME:LINE1;END=122767041;SVLEN=860;AN=20618;AC=1;AF=4.850130953535746e-05;N_HET=1;N_HOMALT=0;N_HOMREF=10308;FREQ_HET=9.700261907071492e-05;FREQ_HOMALT=0.0;FREQ_HOMREF=0.9999029973809292;N_BI_GENOS=10309;East_Han_AN=6554;East_Han_AC=1;East_Han_AF=
chr2	122856697 .	G	<INS:ME:LINE1>	.	PASS	SVTYPE=INS:ME:LINE1;END=122856708;SVLEN=352;AN=20618;AC=1;AF=4.850130953535746e-05;N_HET=1;N_HOMALT=0;N_HOMREF=10308;FREQ_HET=9.700261907071492e-05;FREQ_HOMALT=0.0;FREQ_HOMREF=0.9999029973809292;N_BI_GENOS=10309;East_Han_AN=6554;East_Han_AC=1;East_Han_AF=
chr2	123035157 .	G	<INS:ME:LINE1>	.	PASS	SVTYPE=INS:ME:LINE1;END=123035172;SVLEN=6004;AN=20618;AC=3;AF=0.00014550392860607236;N_HET=3;N_HOMALT=0;N_HOMREF=10306;FREQ_HET=0.0002910078572121447;FREQ_HOMALT=0.0;FREQ_HOMREF=0.9997089921427879;N_BI_GENOS=10309;Central_Han_AN=2128;Central_Han_AC=3;Cent
chr2	123040362 .	G	<INS:ME:LINE1>	.	PASS	SVTYPE=INS:ME:LINE1;END=123040378;SVLEN=5993;AN=20618;AC=1;AF=4.850130953535746e-05;N_HET=1;N_HOMALT=0;N_HOMREF=10308;FREQ_HET=9.700261907071492e-05;FREQ_HOMALT=0.0;FREQ_HOMREF=0.9999029973809292;N_BI_GENOS=10309;Tibetan_AN=414;Tibetan_AC=1;Tibetan_AF=0.0
chr2	123045057 .	A	<INS:ME:LINE1>	.	PASS	SVTYPE=INS:ME:LINE1;END=123045073;SVLEN=846;AN=20618;AC=1;AF=4.850130953535746e-05;N_HET=1;N_HOMALT=0;N_HOMREF=10308;FREQ_HET=9.700261907071492e-05;FREQ_HOMALT=0.0;FREQ_HOMREF=0.9999029973809292;N_BI_GENOS=10309;Central_Han_AN=2128;Central_Han_AC=1;Centra
chr2	123080033 .	C	<INS:ME:LINE1>	.	PASS	SVTYPE=INS:ME:LINE1;END=123080033;SVLEN=832;AN=20618;AC=1;AF=4.850130953535746e-05;N_HET=1;N_HOMALT=0;N_HOMREF=10308;FREQ_HET=9.700261907071492e-05;FREQ_HOMALT=0.0;FREQ_HOMREF=0.9999029973809292;N_BI_GENOS=10309;East_Han_AN=6554;East_Han_AC=1;East_Han_AF=
chr2	123117250 .	A	<INS:ME:LINE1>	.	PASS	SVTYPE=INS:ME:LINE1;END=123117250;SVLEN=217;AN=20618;AC=1;AF=4.850130953535746e-05;N_HET=1;N_HOMALT=0;N_HOMREF=10308;FREQ_HET=9.700261907071492e-05;FREQ_HOMALT=0.0;FREQ_HOMREF=0.9999029973809292;N_BI_GENOS=10309;East_Han_AN=6554;East_Han_AC=1;East_Han_AF=
chr2	123338278 .	G	<INS:ME:LINE1>	.	PASS	SVTYPE=INS:ME:LINE1;END=123338304;SVLEN=1306;AN=20618;AC=1;AF=4.850130953535746e-05;N_HET=1;N_HOMALT=0;N_HOMREF=10308;FREQ_HET=9.700261907071492e-05;FREQ_HOMALT=0.0;FREQ_HOMREF=0.9999029973809292;N_BI_GENOS=10309;East_Han_AN=6554;East_Han_AC=1;East_Han_AF
chr2	123533118 .	C	<INS:ME:LINE1>	.	PASS	SVTYPE=INS:ME:LINE1;END=123533134;SVLEN=2413;AN=20618;AC=1;AF=4.850130953535746e-05;N_HET=1;N_HOMALT=0;N_HOMREF=10308;FREQ_HET=9.700261907071492e-05;FREQ_HOMALT=0.0;FREQ_HOMREF=0.9999029973809292;N_BI_GENOS=10309;Southeast_Han_AN=1908;Southeast_Han_AC=1;S
chr2	123540027 .	C	<INS:ME:LINE1>	.	PASS	SVTYPE=INS:ME:LINE1;END=123540027;SVLEN=990;AN=20618;AC=1;AF=4.850130953535746e-05;N_HET=1;N_HOMALT=0;N_HOMREF=10308;FREQ_HET=9.700261907071492e-05;FREQ_HOMALT=0.0;FREQ_HOMREF=0.9999029973809292;N_BI_GENOS=10309;East_Han_AN=6554;East_Han_AC=1;East_Han_AF=
chr2	123576851 .	T	<INS:ME:LINE1>	.	PASS	SVTYPE=INS:ME:LINE1;END=123576851;SVLEN=1446;AN=20618;AC=2;AF=9.700261907071492e-05;N_HET=2;N_HOMALT=0;N_HOMREF=10307;FREQ_HET=0.00019400523814142983;FREQ_HOMALT=0.0;FREQ_HOMREF=0.9998059947618586;N_BI_GENOS=10309;Lingnan_Han_AN=1888;Lingnan_Han_AC=1;Ling
chr2	123680727 .	A	<INS:ME:LINE1>	.	PASS	SVTYPE=INS:ME:LINE1;END=123680738;SVLEN=847;AN=20618;AC=1;AF=4.850130953535746e-05;N_HET=1;N_HOMALT=0;N_HOMREF=10308;FREQ_HET=9.700261907071492e-05;FREQ_HOMALT=0.0;FREQ_HOMREF=0.9999029973809292;N_BI_GENOS=10309;Yi_AN=422;Yi_AC=1;Yi_AF=0.00236966824644549
chr2	123863900 .	A	<INS:ME:LINE1>	.	PASS	SVTYPE=INS:ME:LINE1;END=123863902;SVLEN=140;AN=20618;AC=1;AF=4.850130953535746e-05;N_HET=1;N_HOMALT=0;N_HOMREF=10308;FREQ_HET=9.700261907071492e-05;FREQ_HOMALT=0.0;FREQ_HOMREF=0.9999029973809292;N_BI_GENOS=10309;Lingnan_Han_AN=1888;Lingnan_Han_AC=1;Lingna
chr2	124149559 .	C	<INS:ME:LINE1>	.	PASS	SVTYPE=INS:ME:LINE1;END=124149576;SVLEN=6039;AN=20618;AC=1;AF=4.850130953535746e-05;N_HET=1;N_HOMALT=0;N_HOMREF=10308;FREQ_HET=9.700261907071492e-05;FREQ_HOMALT=0.0;FREQ_HOMREF=0.9999029973809292;N_BI_GENOS=10309;South_Han_AN=1496;South_Han_AC=1;South_Han
chr2	124603891 .	G	<INS:ME:LINE1>	.	PASS	SVTYPE=INS:ME:LINE1;END=124603905;SVLEN=5999;AN=20618;AC=1;AF=4.850130953535746e-05;N_HET=1;N_HOMALT=0;N_HOMREF=10308;FREQ_HET=9.700261907071492e-05;FREQ_HOMALT=0.0;FREQ_HOMREF=0.9999029973809292;N_BI_GENOS=10309;North_Han_AN=2272;North_Han_AC=1;North_Han
chr2	124798313 .	A	<INS:ME:LINE1>	.	PASS	SVTYPE=INS:ME:LINE1;END=124798329;SVLEN=536;AN=20618;AC=1;AF=4.850130953535746e-05;N_HET=1;N_HOMALT=0;N_HOMREF=10308;FREQ_HET=9.700261907071492e-05;FREQ_HOMALT=0.0;FREQ_HOMREF=0.9999029973809292;N_BI_GENOS=10309;Central_Han_AN=2128;Central_Han_AC=1;Centra
chr2	124977411 .	C	<INS:ME:LINE1>	.	PASS	SVTYPE=INS:ME:LINE1;END=124977423;SVLEN=105;AN=20618;AC=1;AF=4.850130953535746e-05;N_HET=1;N_HOMALT=0;N_HOMREF=10308;FREQ_HET=9.700261907071492e-05;FREQ_HOMALT=0.0;FREQ_HOMREF=0.9999029973809292;N_BI_GENOS=10309;Mongolian_AN=454;Mongolian_AC=1;Mongolian_A
chr2	125306971 .	A	<INS:ME:LINE1>	.	PASS	SVTYPE=INS:ME:LINE1;END=125306986;SVLEN=344;AN=20618;AC=2;AF=9.700261907071492e-05;N_HET=2;N_HOMALT=0;N_HOMREF=10307;FREQ_HET=0.00019400523814142983;FREQ_HOMALT=0.0;FREQ_HOMREF=0.9998059947618586;N_BI_GENOS=10309;Northwest_Han_AN=1296;Northwest_Han_AC=1;N
chr2	125453295 .	T	<INS:ME:LINE1>	.	PASS	SVTYPE=INS:ME:LINE1;END=125453314;SVLEN=5090;AN=20618;AC=1;AF=4.850130953535746e-05;N_HET=1;N_HOMALT=0;N_HOMREF=10308;FREQ_HET=9.700261907071492e-05;FREQ_HOMALT=0.0;FREQ_HOMREF=0.9999029973809292;N_BI_GENOS=10309;East_Han_AN=6554;East_Han_AC=1;East_Han_AF
chr2	125930139 .	A	<INS:ME:LINE1>	.	PASS	SVTYPE=INS:ME:LINE1;END=125930153;SVLEN=535;AN=20618;AC=1;AF=4.850130953535746e-05;N_HET=1;N_HOMALT=0;N_HOMREF=10308;FREQ_HET=9.700261907071492e-05;FREQ_HOMALT=0.0;FREQ_HOMREF=0.9999029973809292;N_BI_GENOS=10309;Northwest_Han_AN=1296;Northwest_Han_AC=1;No
chr2	125954069 .	A	<INS:ME:LINE1>	.	PASS	SVTYPE=INS:ME:LINE1;END=125954084;SVLEN=745;AN=20618;AC=1;AF=4.850130953535746e-05;N_HET=1;N_HOMALT=0;N_HOMREF=10308;FREQ_HET=9.700261907071492e-05;FREQ_HOMALT=0.0;FREQ_HOMREF=0.9999029973809292;N_BI_GENOS=10309;Central_Han_AN=2128;Central_Han_AC=1;Centra
chr2	125972598 .	G	<INS:ME:LINE1>	.	PASS	SVTYPE=INS:ME:LINE1;END=125972616;SVLEN=1194;AN=20618;AC=1;AF=4.850130953535746e-05;N_HET=1;N_HOMALT=0;N_HOMREF=10308;FREQ_HET=9.700261907071492e-05;FREQ_HOMALT=0.0;FREQ_HOMREF=0.9999029973809292;N_BI_GENOS=10309;Lingnan_Han_AN=1888;Lingnan_Han_AC=1;Lingn
chr2	125974536 .	A	<INS:ME:LINE1>	.	PASS	SVTYPE=INS:ME:LINE1;END=125974536;SVLEN=6000;AN=20618;AC=1;AF=4.850130953535746e-05;N_HET=1;N_HOMALT=0;N_HOMREF=10308;FREQ_HET=9.700261907071492e-05;FREQ_HOMALT=0.0;FREQ_HOMREF=0.9999029973809292;N_BI_GENOS=10309;Southeast_Han_AN=1908;Southeast_Han_AC=1;S
chr2	125976314 .	A	<INS:ME:LINE1>	.	PASS	SVTYPE=INS:ME:LINE1;END=125976320;SVLEN=1448;AN=20618;AC=1;AF=4.850130953535746e-05;N_HET=1;N_HOMALT=0;N_HOMREF=10308;FREQ_HET=9.700261907071492e-05;FREQ_HOMALT=0.0;FREQ_HOMREF=0.9999029973809292;N_BI_GENOS=10309;Central_Han_AN=2128;Central_Han_AC=1;Centr
chr2	126105534 .	A	<INS:ME:LINE1>	.	PASS	SVTYPE=INS:ME:LINE1;END=126105552;SVLEN=885;AN=20618;AC=1;AF=4.850130953535746e-05;N_HET=1;N_HOMALT=0;N_HOMREF=10308;FREQ_HET=9.700261907071492e-05;FREQ_HOMALT=0.0;FREQ_HOMREF=0.9999029973809292;N_BI_GENOS=10309;Central_Han_AN=2128;Central_Han_AC=1;Centra
chr2	126124109 .	T	<INS:ME:LINE1>	.	PASS	SVTYPE=INS:ME:LINE1;END=126124152;SVLEN=862;AN=20618;AC=1;AF=4.850130953535746e-05;N_HET=1;N_HOMALT=0;N_HOMREF=10308;FREQ_HET=9.700261907071492e-05;FREQ_HOMALT=0.0;FREQ_HOMREF=0.9999029973809292;N_BI_GENOS=10309;East_Han_AN=6554;East_Han_AC=1;East_Han_AF=
chr2	126134079 .	T	<INS:ME:LINE1>	.	PASS	SVTYPE=INS:ME:LINE1;END=126134097;SVLEN=3730;AN=20618;AC=1;AF=4.850130953535746e-05;N_HET=1;N_HOMALT=0;N_HOMREF=10308;FREQ_HET=9.700261907071492e-05;FREQ_HOMALT=0.0;FREQ_HOMREF=0.9999029973809292;N_BI_GENOS=10309;Central_Han_AN=2128;Central_Han_AC=1;Centr
chr2	126264185 .	G	<INS:ME:LINE1>	.	PASS	SVTYPE=INS:ME:LINE1;END=126264200;SVLEN=5996;AN=20618;AC=1;AF=4.850130953535746e-05;N_HET=1;N_HOMALT=0;N_HOMREF=10308;FREQ_HET=9.700261907071492e-05;FREQ_HOMALT=0.0;FREQ_HOMREF=0.9999029973809292;N_BI_GENOS=10309;North_Han_AN=2272;North_Han_AC=1;North_Han
chr2	126346949 .	T	<INS:ME:LINE1>	.	PASS	SVTYPE=INS:ME:LINE1;END=126346959;SVLEN=3081;AN=20618;AC=1;AF=4.850130953535746e-05;N_HET=1;N_HOMALT=0;N_HOMREF=10308;FREQ_HET=9.700261907071492e-05;FREQ_HOMALT=0.0;FREQ_HOMREF=0.9999029973809292;N_BI_GENOS=10309;South_Han_AN=1496;South_Han_AC=1;South_Han
chr2	126347567 .	A	<INS:ME:LINE1>	.	PASS	SVTYPE=INS:ME:LINE1;END=126347582;SVLEN=6008;AN=20618;AC=2;AF=9.700261907071492e-05;N_HET=2;N_HOMALT=0;N_HOMREF=10307;FREQ_HET=0.00019400523814142983;FREQ_HOMALT=0.0;FREQ_HOMREF=0.9998059947618586;N_BI_GENOS=10309;North_Han_AN=2272;North_Han_AC=1;North_Ha
chr2	126367012 .	G	<INS:ME:LINE1>	.	PASS	SVTYPE=INS:ME:LINE1;END=126367027;SVLEN=6038;AN=20618;AC=2;AF=9.700261907071492e-05;N_HET=0;N_HOMALT=1;N_HOMREF=10308;FREQ_HET=0.0;FREQ_HOMALT=9.700261907071492e-05;FREQ_HOMREF=0.9999029973809292;N_BI_GENOS=10309;East_Han_AN=6554;East_Han_AC=2;East_Han_AF
chr2	126965363 .	A	<INS:ME:LINE1>	.	PASS	SVTYPE=INS:ME:LINE1;END=126965363;SVLEN=1752;AN=20618;AC=4;AF=0.00019400523814142983;N_HET=4;N_HOMALT=0;N_HOMREF=10305;FREQ_HET=0.0003880104762828596 6;FREQ_HOMALT=0.0;FREQ_HOMREF=0.9996119895237171;N_BI_GENOS=10309;East_Han_AN=6554;East_Han_AC=3;East_Han_
chr2	128121406 .	T	<INS:ME:LINE1>	.	PASS	SVTYPE=INS:ME:LINE1;END=128121422;SVLEN=2563;AN=20618;AC=1;AF=4.850130953535746e-05;N_HET=1;N_HOMALT=0;N_HOMREF=10308;FREQ_HET=9.700261907071492e-05;FREQ_HOMALT=0.0;FREQ_HOMREF=0.9999029973809292;N_BI_GENOS=10309;East_Han_AN=6554;East_Han_AC=1;East_Han_AF
chr2	128691674 .	C	<INS:ME:LINE1>	.	PASS	SVTYPE=INS:ME:LINE1;END=128691711;SVLEN=743;AN=20618;AC=1;AF=4.850130953535746e-05;N_HET=1;N_HOMALT=0;N_HOMREF=10308;FREQ_HET=9.700261907071492e-05;FREQ_HOMALT=0.0;FREQ_HOMREF=0.9999029973809292;N_BI_GENOS=10309;East_Han_AN=6554;East_Han_AC=1;East_Han_AF=
chr2	128816400 .	A	<INS:ME:LINE1>	.	PASS	SVTYPE=INS:ME:LINE1;END=128816410;SVLEN=6000;AN=20618;AC=2680;AF=0.129983509554758;N_HET=1210;N_HOMALT=735;N_HOMREF=8364;FREQ_HET=0.11737316907556504;FREQ_HOMALT=0.07129692501697546;FREQ_HOMREF=0.8113299059074595;N_BI_GENOS=10309;South_Han_AN=1496;South_H
chr2	128914461 .	A	<INS:ME:LINE1>	.	PASS	SVTYPE=INS:ME:LINE1;END=128914466;SVLEN=234;AN=20618;AC=1;AF=4.850130953535746e-05;N_HET=1;N_HOMALT=0;N_HOMREF=10308;FREQ_HET=9.700261907071492e-05;FREQ_HOMALT=0.0;FREQ_HOMREF=0.9999029973809292;N_BI_GENOS=10309;Southeast_Han_AN=1908;Southeast_Han_AC=1;So
chr2	129032481 .	A	<INS:ME:LINE1>	.	PASS	SVTYPE=INS:ME:LINE1;END=129032496;SVLEN=1404;AN=20618;AC=1;AF=4.850130953535746e-05;N_HET=1;N_HOMALT=0;N_HOMREF=10308;FREQ_HET=9.700261907071492e-05;FREQ_HOMALT=0.0;FREQ_HOMREF=0.9999029973809292;N_BI_GENOS=10309;North_Han_AN=2272;North_Han_AC=1;North_Han
chr2	129080722 .	A	<INS:ME:LINE1>	.	PASS	SVTYPE=INS:ME:LINE1;END=129080731;SVLEN=1776;AN=20618;AC=2;AF=9.700261907071492e-05;N_HET=2;N_HOMALT=0;N_HOMREF=10307;FREQ_HET=0.00019400523814142983;FREQ_HOMALT=0.0;FREQ_HOMREF=0.9998059947618586;N_BI_GENOS=10309;North_Han_AN=2272;North_Han_AC=1;North_Ha
chr2	129085277 .	T	<INS:ME:LINE1>	.	PASS	SVTYPE=INS:ME:LINE1;END=129085296;SVLEN=1656;AN=20618;AC=1;AF=4.850130953535746e-05;N_HET=1;N_HOMALT=0;N_HOMREF=10308;FREQ_HET=9.700261907071492e-05;FREQ_HOMALT=0.0;FREQ_HOMREF=0.9999029973809292;N_BI_GENOS=10309;South_Han_AN=1496;South_Han_AC=1;South_Han
chr2	129101916 .	C	<INS:ME:LINE1>	.	PASS	SVTYPE=INS:ME:LINE1;END=129101931;SVLEN=621;AN=20618;AC=2;AF=9.700261907071492e-05;N_HET=2;N_HOMALT=0;N_HOMREF=10307;FREQ_HET=0.00019400523814142983;FREQ_HOMALT=0.0;FREQ_HOMREF=0.9998059947618586;N_BI_GENOS=10309;Lingnan_Han_AN=1888;Lingnan_Han_AC=1;Lingn
chr2	129161950 .	A	<INS:ME:LINE1>	.	PASS	SVTYPE=INS:ME:LINE1;END=129161966;SVLEN=6029;AN=20618;AC=5;AF=0.0002425065476767872 8;N_HET=5;N_HOMALT=0;N_HOMREF=10304;FREQ_HET=0.00048501309535357455;FREQ_HOMALT=0.0;FREQ_HOMREF=0.9995149869046465;N_BI_GENOS=10309;South_Han_AN=1496;South_Han_AC=1;South_H
chr2	129517953 .	A	<INS:ME:LINE1>	.	PASS	SVTYPE=INS:ME:LINE1;END=129517953;SVLEN=550;AN=20618;AC=1;AF=4.850130953535746e-05;N_HET=1;N_HOMALT=0;N_HOMREF=10308;FREQ_HET=9.700261907071492e-05;FREQ_HOMALT=0.0;FREQ_HOMREF=0.9999029973809292;N_BI_GENOS=10309;East_Han_AN=6554;East_Han_AC=1;East_Han_AF=
chr2	129775188 .	C	<INS:ME:LINE1>	.	PASS	SVTYPE=INS:ME:LINE1;END=129775188;SVLEN=6000;AN=20618;AC=2;AF=9.700261907071492e-05;N_HET=2;N_HOMALT=0;N_HOMREF=10307;FREQ_HET=0.00019400523814142983;FREQ_HOMALT=0.0;FREQ_HOMREF=0.9998059947618586;N_BI_GENOS=10309;Central_Han_AN=2128;Central_Han_AC=2;Cent
chr2	129861612 .	A	<INS:ME:LINE1>	.	PASS	SVTYPE=INS:ME:LINE1;END=129861627;SVLEN=76;AN=20618;AC=4;AF=0.00019400523814142983;N_HET=2;N_HOMALT=1;N_HOMREF=10306;FREQ_HET=0.00019400523814142983;FREQ_HOMALT=9.700261907071492e-05;FREQ_HOMREF=0.9997089921427879;N_BI_GENOS=10309;Southeast_Han_AN=1908;So
chr2	130910817 .	A	<INS:ME:LINE1>	.	PASS	SVTYPE=INS:ME:LINE1;END=130910832;SVLEN=6000;AN=20618;AC=1;AF=4.850130953535746e-05;N_HET=1;N_HOMALT=0;N_HOMREF=10308;FREQ_HET=9.700261907071492e-05;FREQ_HOMALT=0.0;FREQ_HOMREF=0.9999029973809292;N_BI_GENOS=10309;Yi_AN=422;Yi_AC=1;Yi_AF=0.0023696682464454
chr2	131243583 .	A	<INS:ME:LINE1>	.	PASS	SVTYPE=INS:ME:LINE1;END=131243600;SVLEN=5996;AN=20618;AC=10;AF=0.00048501309535357455;N_HET=10;N_HOMALT=0;N_HOMREF=10299;FREQ_HET=0.0009700261907071491;FREQ_HOMALT=0.0;FREQ_HOMREF=0.9990299738092928;N_BI_GENOS=10309;Lingnan_Han_AN=1888;Lingnan_Han_AC=1;Li
chr2	131610486 .	A	<INS:ME:LINE1>	.	PASS	SVTYPE=INS:ME:LINE1;END=131610488;SVLEN=291;AN=20618;AC=1;AF=4.850130953535746e-05;N_HET=1;N_HOMALT=0;N_HOMREF=10308;FREQ_HET=9.700261907071492e-05;FREQ_HOMALT=0.0;FREQ_HOMREF=0.9999029973809292;N_BI_GENOS=10309;Southeast_Han_AN=1908;Southeast_Han_AC=1;So
chr2	131879699 .	G	<INS:ME:LINE1>	.	PASS	SVTYPE=INS:ME:LINE1;END=131879711;SVLEN=4643;AN=20618;AC=10;AF=0.00048501309535357455;N_HET=10;N_HOMALT=0;N_HOMREF=10299;FREQ_HET=0.0009700261907071491;FREQ_HOMALT=0.0;FREQ_HOMREF=0.9990299738092928;N_BI_GENOS=10309;South_Han_AN=1496;South_Han_AC=1;South_
chr2	132036695 .	T	<INS:ME:LINE1>	.	PASS	SVTYPE=INS:ME:LINE1;END=132036713;SVLEN=1183;AN=20618;AC=13;AF=0.0006305170239596469;N_HET=13;N_HOMALT=0;N_HOMREF=10296;FREQ_HET=0.0012610340479192938;FREQ_HOMALT=0.0;FREQ_HOMREF=0.9987389659520807;N_BI_GENOS=10309;South_Han_AN=1496;South_Han_AC=2;South_H
chr2	132054866 .	T	<INS:ME:LINE1>	.	PASS	SVTYPE=INS:ME:LINE1;END=132054866;SVLEN=0;AN=20618;AC=8;AF=0.0003880104762828596 6;N_HET=8;N_HOMALT=0;N_HOMREF=10301;FREQ_HET=0.0007760209525657193;FREQ_HOMALT=0.0;FREQ_HOMREF=0.9992239790474343;N_BI_GENOS=10309;South_Han_AN=1496;South_Han_AC=1;South_Han_A
chr2	132055038 .	A	<INS:ME:LINE1>	.	PASS	SVTYPE=INS:ME:LINE1;END=132055038;SVLEN=0;AN=20618;AC=1;AF=4.850130953535746e-05;N_HET=1;N_HOMALT=0;N_HOMREF=10308;FREQ_HET=9.700261907071492e-05;FREQ_HOMALT=0.0;FREQ_HOMREF=0.9999029973809292;N_BI_GENOS=10309;East_Han_AN=6554;East_Han_AC=1;East_Han_AF=0.
chr2	132444673 .	A	<INS:ME:LINE1>	.	PASS	SVTYPE=INS:ME:LINE1;END=132444688;SVLEN=6063;AN=20618;AC=1;AF=4.850130953535746e-05;N_HET=1;N_HOMALT=0;N_HOMREF=10308;FREQ_HET=9.700261907071492e-05;FREQ_HOMALT=0.0;FREQ_HOMREF=0.9999029973809292;N_BI_GENOS=10309;East_Han_AN=6554;East_Han_AC=1;East_Han_AF
chr2	133042903 .	T	<INS:ME:LINE1>	.	PASS	SVTYPE=INS:ME:LINE1;END=133042903;SVLEN=72;AN=20618;AC=1;AF=4.850130953535746e-05;N_HET=1;N_HOMALT=0;N_HOMREF=10308;FREQ_HET=9.700261907071492e-05;FREQ_HOMALT=0.0;FREQ_HOMREF=0.9999029973809292;N_BI_GENOS=10309;South_Han_AN=1496;South_Han_AC=1;South_Han_A
chr2	133158923 .	G	<INS:ME:LINE1>	.	PASS	SVTYPE=INS:ME:LINE1;END=133158923;SVLEN=14;AN=20618;AC=2;AF=9.700261907071492e-05;N_HET=0;N_HOMALT=1;N_HOMREF=10308;FREQ_HET=0.0;FREQ_HOMALT=9.700261907071492e-05;FREQ_HOMREF=0.9999029973809292;N_BI_GENOS=10309;East_Han_AN=6554;East_Han_AC=2;East_Han_AF=0
chr2	133186256 .	T	<INS:ME:LINE1>	.	PASS	SVTYPE=INS:ME:LINE1;END=133186256;SVLEN=167;AN=20618;AC=1;AF=4.850130953535746e-05;N_HET=1;N_HOMALT=0;N_HOMREF=10308;FREQ_HET=9.700261907071492e-05;FREQ_HOMALT=0.0;FREQ_HOMREF=0.9999029973809292;N_BI_GENOS=10309;East_Han_AN=6554;East_Han_AC=1;East_Han_AF=
chr2	133275126 .	A	<INS:ME:LINE1>	.	PASS	SVTYPE=INS:ME:LINE1;END=133275141;SVLEN=6013;AN=20618;AC=2;AF=9.700261907071492e-05;N_HET=0;N_HOMALT=1;N_HOMREF=10308;FREQ_HET=0.0;FREQ_HOMALT=9.700261907071492e-05;FREQ_HOMREF=0.9999029973809292;N_BI_GENOS=10309;East_Han_AN=6554;East_Han_AC=2;East_Han_AF
chr2	133867468 .	T	<INS:ME:LINE1>	.	PASS	SVTYPE=INS:ME:LINE1;END=133867485;SVLEN=5960;AN=20618;AC=1;AF=4.850130953535746e-05;N_HET=1;N_HOMALT=0;N_HOMREF=10308;FREQ_HET=9.700261907071492e-05;FREQ_HOMALT=0.0;FREQ_HOMREF=0.9999029973809292;N_BI_GENOS=10309;Central_Han_AN=2128;Central_Han_AC=1;Centr
chr2	134501568 .	T	<INS:ME:LINE1>	.	PASS	SVTYPE=INS:ME:LINE1;END=134501568;SVLEN=5974;AN=20618;AC=1;AF=4.850130953535746e-05;N_HET=1;N_HOMALT=0;N_HOMREF=10308;FREQ_HET=9.700261907071492e-05;FREQ_HOMALT=0.0;FREQ_HOMREF=0.9999029973809292;N_BI_GENOS=10309;Northwest_Han_AN=1296;Northwest_Han_AC=1;N
chr2	134505336 .	A	<INS:ME:LINE1>	.	PASS	SVTYPE=INS:ME:LINE1;END=134505351;SVLEN=6024;AN=20618;AC=1;AF=4.850130953535746e-05;N_HET=1;N_HOMALT=0;N_HOMREF=10308;FREQ_HET=9.700261907071492e-05;FREQ_HOMALT=0.0;FREQ_HOMREF=0.9999029973809292;N_BI_GENOS=10309;North_Han_AN=2272;North_Han_AC=1;North_Han
chr2	134642894 .	C	<INS:ME:LINE1>	.	PASS	SVTYPE=INS:ME:LINE1;END=134642894;SVLEN=79;AN=20618;AC=2;AF=9.700261907071492e-05;N_HET=2;N_HOMALT=0;N_HOMREF=10307;FREQ_HET=0.00019400523814142983;FREQ_HOMALT=0.0;FREQ_HOMREF=0.9998059947618586;N_BI_GENOS=10309;South_Han_AN=1496;South_Han_AC=1;South_Han_
chr2	134772886 .	A	<INS:ME:LINE1>	.	PASS	SVTYPE=INS:ME:LINE1;END=134772896;SVLEN=436;AN=20618;AC=1;AF=4.850130953535746e-05;N_HET=1;N_HOMALT=0;N_HOMREF=10308;FREQ_HET=9.700261907071492e-05;FREQ_HOMALT=0.0;FREQ_HOMREF=0.9999029973809292;N_BI_GENOS=10309;Northwest_Han_AN=1296;Northwest_Han_AC=1;No
chr2	135245926 .	A	<INS:ME:LINE1>	.	PASS	SVTYPE=INS:ME:LINE1;END=135245942;SVLEN=2800;AN=20618;AC=6;AF=0.0002910078572121447;N_HET=0;N_HOMALT=3;N_HOMREF=10306;FREQ_HET=0.0;FREQ_HOMALT=0.0002910078572121447;FREQ_HOMREF=0.9997089921427879;N_BI_GENOS=10309;Northwest_Han_AN=1296;Northwest_Han_AC=2;N
chr2	135267541 .	C	<INS:ME:LINE1>	.	PASS	SVTYPE=INS:ME:LINE1;END=135267556;SVLEN=641;AN=20618;AC=10;AF=0.00048501309535357455;N_HET=10;N_HOMALT=0;N_HOMREF=10299;FREQ_HET=0.0009700261907071491;FREQ_HOMALT=0.0;FREQ_HOMREF=0.9990299738092928;N_BI_GENOS=10309;North_Han_AN=2272;North_Han_AC=1;North_H
chr2	135571210 .	A	<INS:ME:LINE1>	.	PASS	SVTYPE=INS:ME:LINE1;END=135571226;SVLEN=1388;AN=20618;AC=2;AF=9.700261907071492e-05;N_HET=2;N_HOMALT=0;N_HOMREF=10307;FREQ_HET=0.00019400523814142983;FREQ_HOMALT=0.0;FREQ_HOMREF=0.9998059947618586;N_BI_GENOS=10309;South_Han_AN=1496;South_Han_AC=2;South_Ha
chr2	135643449 .	A	<INS:ME:LINE1>	.	PASS	SVTYPE=INS:ME:LINE1;END=135643465;SVLEN=269;AN=20618;AC=1;AF=4.850130953535746e-05;N_HET=1;N_HOMALT=0;N_HOMREF=10308;FREQ_HET=9.700261907071492e-05;FREQ_HOMALT=0.0;FREQ_HOMREF=0.9999029973809292;N_BI_GENOS=10309;Central_Han_AN=2128;Central_Han_AC=1;Centra
chr2	135668059 .	A	<INS:ME:LINE1>	.	PASS	SVTYPE=INS:ME:LINE1;END=135668074;SVLEN=6012;AN=20618;AC=1;AF=4.850130953535746e-05;N_HET=1;N_HOMALT=0;N_HOMREF=10308;FREQ_HET=9.700261907071492e-05;FREQ_HOMALT=0.0;FREQ_HOMREF=0.9999029973809292;N_BI_GENOS=10309;East_Han_AN=6554;East_Han_AC=1;East_Han_AF
chr2	135688189 .	G	<INS:ME:LINE1>	.	PASS	SVTYPE=INS:ME:LINE1;END=135688193;SVLEN=1819;AN=20618;AC=1;AF=4.850130953535746e-05;N_HET=1;N_HOMALT=0;N_HOMREF=10308;FREQ_HET=9.700261907071492e-05;FREQ_HOMALT=0.0;FREQ_HOMREF=0.9999029973809292;N_BI_GENOS=10309;Yi_AN=422;Yi_AC=1;Yi_AF=0.0023696682464454
chr2	135716002 .	T	<INS:ME:LINE1>	.	PASS	SVTYPE=INS:ME:LINE1;END=135716005;SVLEN=52;AN=20618;AC=1;AF=4.850130953535746e-05;N_HET=1;N_HOMALT=0;N_HOMREF=10308;FREQ_HET=9.700261907071492e-05;FREQ_HOMALT=0.0;FREQ_HOMREF=0.9999029973809292;N_BI_GENOS=10309;Southeast_Han_AN=1908;Southeast_Han_AC=1;Sou
chr2	135825226 .	C	<INS:ME:LINE1>	.	PASS	SVTYPE=INS:ME:LINE1;END=135825244;SVLEN=6002;AN=20618;AC=3;AF=0.00014550392860607236;N_HET=3;N_HOMALT=0;N_HOMREF=10306;FREQ_HET=0.0002910078572121447;FREQ_HOMALT=0.0;FREQ_HOMREF=0.9997089921427879;N_BI_GENOS=10309;Lingnan_Han_AN=1888;Lingnan_Han_AC=1;Ling
chr2	136121495 .	A	<INS:ME:LINE1>	.	PASS	SVTYPE=INS:ME:LINE1;END=136121495;SVLEN=0;AN=20618;AC=4;AF=0.00019400523814142983;N_HET=2;N_HOMALT=1;N_HOMREF=10306;FREQ_HET=0.00019400523814142983;FREQ_HOMALT=9.700261907071492e-05;FREQ_HOMREF=0.9997089921427879;N_BI_GENOS=10309;South_Han_AN=1496;South_H
chr2	136248767 .	A	<INS:ME:LINE1>	.	PASS	SVTYPE=INS:ME:LINE1;END=136248783;SVLEN=995;AN=20618;AC=1;AF=4.850130953535746e-05;N_HET=1;N_HOMALT=0;N_HOMREF=10308;FREQ_HET=9.700261907071492e-05;FREQ_HOMALT=0.0;FREQ_HOMREF=0.9999029973809292;N_BI_GENOS=10309;Southeast_Han_AN=1908;Southeast_Han_AC=1;So
chr2	136453560 .	G	<INS:ME:LINE1>	.	PASS	SVTYPE=INS:ME:LINE1;END=136453560;SVLEN=6000;AN=20618;AC=1;AF=4.850130953535746e-05;N_HET=1;N_HOMALT=0;N_HOMREF=10308;FREQ_HET=9.700261907071492e-05;FREQ_HOMALT=0.0;FREQ_HOMREF=0.9999029973809292;N_BI_GENOS=10309;East_Han_AN=6554;East_Han_AC=1;East_Han_AF
chr2	136637019 .	A	<INS:ME:LINE1>	.	PASS	SVTYPE=INS:ME:LINE1;END=136637035;SVLEN=5993;AN=20618;AC=2;AF=9.700261907071492e-05;N_HET=2;N_HOMALT=0;N_HOMREF=10307;FREQ_HET=0.00019400523814142983;FREQ_HOMALT=0.0;FREQ_HOMREF=0.9998059947618586;N_BI_GENOS=10309;East_Han_AN=6554;East_Han_AC=1;East_Han_A

chr2	136668562	.	G	<INS:ME:LINE1>	.	PASS	SVTYPE=INS:ME:LINE1;END=136668572;SVLEN=445;AN=20618;AC=2;AF=9.700261907071492e-05;N_HET=2;N_HOMALT=0;N_HOMREF=10307;FREQ_HET=0.00019400523814142983;FREQ_HOMALT=0.0;FREQ_HOMREF=0.9998059947618586;N_BI_GENOS=10309;Lingnan_Han_AN=1888;Lingnan_Han_AC=1;Lingn
chr2	136753222	.	A	<INS:ME:LINE1>	.	PASS	SVTYPE=INS:ME:LINE1;END=136753231;SVLEN=1410;AN=20618;AC=1;AF=4.850130953535746e-05;N_HET=1;N_HOMALT=0;N_HOMREF=10308;FREQ_HET=9.700261907071492e-05;FREQ_HOMALT=0.0;FREQ_HOMREF=0.9999029973809292;N_BI_GENOS=10309;Southeast_Han_AN=1908;Southeast_Han_AC=1;S
chr2	136890038	.	T	<INS:ME:LINE1>	.	PASS	SVTYPE=INS:ME:LINE1;END=136890051;SVLEN=387;AN=20618;AC=2;AF=9.700261907071492e-05;N_HET=2;N_HOMALT=0;N_HOMREF=10307;FREQ_HET=0.00019400523814142983;FREQ_HOMALT=0.0;FREQ_HOMREF=0.9998059947618586;N_BI_GENOS=10309;East_Han_AN=6554;East_Han_AC=2;East_Han_AF
chr2	136896688	.	A	<INS:ME:LINE1>	.	PASS	SVTYPE=INS:ME:LINE1;END=136896702;SVLEN=5999;AN=20618;AC=11;AF=0.0005335144404888932;N_HET=11;N_HOMALT=0;N_HOMREF=10298;FREQ_HET=0.0010670288809777864;FREQ_HOMALT=0.0;FREQ_HOMREF=0.9989329711902222;N_BI_GENOS=10309;South_Han_AN=1496;South_Han_AC=1;South_Han
chr2	136899382	.	T	<INS:ME:LINE1>	.	PASS	SVTYPE=INS:ME:LINE1;END=136899383;SVLEN=2120;AN=20618;AC=3;AF=0.00014550392860607236;N_HET=3;N_HOMALT=0;N_HOMREF=10306;FREQ_HET=0.0002910078572121447;FREQ_HOMALT=0.0;FREQ_HOMREF=0.9997089921427879;N_BI_GENOS=10309;North_Han_AN=2272;North_Han_AC=1;North_Ha
chr2	137006935	.	A	<INS:ME:LINE1>	.	PASS	SVTYPE=INS:ME:LINE1;END=137006945;SVLEN=6074;AN=20618;AC=1;AF=4.850130953535746e-05;N_HET=1;N_HOMALT=0;N_HOMREF=10308;FREQ_HET=9.700261907071492e-05;FREQ_HOMALT=0.0;FREQ_HOMREF=0.9999029973809292;N_BI_GENOS=10309;Northwest_Han_AN=1296;Northwest_Han_AC=1;N
chr2	137489420	.	A	<INS:ME:LINE1>	.	PASS	SVTYPE=INS:ME:LINE1;END=137489439;SVLEN=974;AN=20618;AC=1;AF=4.850130953535746e-05;N_HET=1;N_HOMALT=0;N_HOMREF=10308;FREQ_HET=9.700261907071492e-05;FREQ_HOMALT=0.0;FREQ_HOMREF=0.9999029973809292;N_BI_GENOS=10309;South_Han_AN=1496;South_Han_AC=1;South_Han_
chr2	137521267	.	A	<INS:ME:LINE1>	.	PASS	SVTYPE=INS:ME:LINE1;END=137521284;SVLEN=1057;AN=20618;AC=1;AF=4.850130953535746e-05;N_HET=1;N_HOMALT=0;N_HOMREF=10308;FREQ_HET=9.700261907071492e-05;FREQ_HOMALT=0.0;FREQ_HOMREF=0.9999029973809292;N_BI_GENOS=10309;Lingnan_Han_AN=1888;Lingnan_Han_AC=1;Lingn
chr2	137607475	.	A	<INS:ME:LINE1>	.	PASS	SVTYPE=INS:ME:LINE1;END=137607481;SVLEN=321;AN=20618;AC=1;AF=4.850130953535746e-05;N_HET=1;N_HOMALT=0;N_HOMREF=10308;FREQ_HET=9.700261907071492e-05;FREQ_HOMALT=0.0;FREQ_HOMREF=0.9999029973809292;N_BI_GENOS=10309;South_Han_AN=1496;South_Han_AC=1;South_Han_
chr2	137793046	.	A	<INS:ME:LINE1>	.	PASS	SVTYPE=INS:ME:LINE1;END=137793048;SVLEN=251;AN=20618;AC=1;AF=4.850130953535746e-05;N_HET=1;N_HOMALT=0;N_HOMREF=10308;FREQ_HET=9.700261907071492e-05;FREQ_HOMALT=0.0;FREQ_HOMREF=0.9999029973809292;N_BI_GENOS=10309;Central_Han_AN=2128;Central_Han_AC=1;Centra
chr2	138330993	.	A	<INS:ME:LINE1>	.	PASS	SVTYPE=INS:ME:LINE1;END=138331001;SVLEN=6025;AN=20618;AC=2;AF=9.700261907071492e-05;N_HET=2;N_HOMALT=0;N_HOMREF=10307;FREQ_HET=0.00019400523814142983;FREQ_HOMALT=0.0;FREQ_HOMREF=0.9998059947618586;N_BI_GENOS=10309;Tibetan_AN=414;Tibetan_AC=2;Tibetan_AF=0.
chr2	138699395	.	A	<INS:ME:LINE1>	.	PASS	SVTYPE=INS:ME:LINE1;END=138699405;SVLEN=384;AN=20618;AC=1;AF=4.850130953535746e-05;N_HET=1;N_HOMALT=0;N_HOMREF=10308;FREQ_HET=9.700261907071492e-05;FREQ_HOMALT=0.0;FREQ_HOMREF=0.9999029973809292;N_BI_GENOS=10309;South_Han_AN=1496;South_Han_AC=1;South_Han_
chr2	138877275	.	A	<INS:ME:LINE1>	.	PASS	SVTYPE=INS:ME:LINE1;END=138877275;SVLEN=20;AN=20618;AC=1;AF=4.850130953535746e-05;N_HET=1;N_HOMALT=0;N_HOMREF=10308;FREQ_HET=9.700261907071492e-05;FREQ_HOMALT=0.0;FREQ_HOMREF=0.9999029973809292;N_BI_GENOS=10309;Northwest_Han_AN=1296;Northwest_Han_AC=1;Nor
chr2	138974270	.	A	<INS:ME:LINE1>	.	PASS	SVTYPE=INS:ME:LINE1;END=138974281;SVLEN=1412;AN=20618;AC=6;AF=0.0002910078572121447;N_HET=6;N_HOMALT=0;N_HOMREF=10303;FREQ_HET=0.0005820157144242894;FREQ_HOMALT=0.0;FREQ_HOMREF=0.9994179842855757;N_BI_GENOS=10309;Lingnan_Han_AN=1888;Lingnan_Han_AC=1;Lingn
chr2	139012780	.	T	<INS:ME:LINE1>	.	PASS	SVTYPE=INS:ME:LINE1;END=139012780;SVLEN=116;AN=20618;AC=1;AF=4.850130953535746e-05;N_HET=1;N_HOMALT=0;N_HOMREF=10308;FREQ_HET=9.700261907071492e-05;FREQ_HOMALT=0.0;FREQ_HOMREF=0.9999029973809292;N_BI_GENOS=10309;East_Han_AN=6554;East_Han_AC=1;East_Han_AF=
chr2	139014260	.	A	<INS:ME:LINE1>	.	PASS	SVTYPE=INS:ME:LINE1;END=139014305;SVLEN=2527;AN=20618;AC=1;AF=4.850130953535746e-05;N_HET=1;N_HOMALT=0;N_HOMREF=10308;FREQ_HET=9.700261907071492e-05;FREQ_HOMALT=0.0;FREQ_HOMREF=0.9999029973809292;N_BI_GENOS=10309;East_Han_AN=6554;East_Han_AC=1;East_Han_AF
chr2	139606592	.	A	<INS:ME:LINE1>	.	PASS	SVTYPE=INS:ME:LINE1;END=139606612;SVLEN=295;AN=20618;AC=1;AF=4.850130953535746e-05;N_HET=1;N_HOMALT=0;N_HOMREF=10308;FREQ_HET=9.700261907071492e-05;FREQ_HOMALT=0.0;FREQ_HOMREF=0.9999029973809292;N_BI_GENOS=10309;East_Han_AN=6554;East_Han_AC=1;East_Han_AF=
chr2	139692143	.	A	<INS:ME:LINE1>	.	PASS	SVTYPE=INS:ME:LINE1;END=139692158;SVLEN=5998;AN=20618;AC=13;AF=0.0006305170239596469;N_HET=13;N_HOMALT=0;N_HOMREF=10296;FREQ_HET=0.0012610340479192938;FREQ_HOMALT=0.0;FREQ_HOMREF=0.9987389659520807;N_BI_GENOS=10309;South_Han_AN=1496;South_Han_AC=5;South_H
chr2	139790921	.	T	<INS:ME:LINE1>	.	PASS	SVTYPE=INS:ME:LINE1;END=139790935;SVLEN=758;AN=20618;AC=2;AF=9.700261907071492e-05;N_HET=2;N_HOMALT=0;N_HOMREF=10307;FREQ_HET=0.00019400523814142983;FREQ_HOMALT=0.0;FREQ_HOMREF=0.9998059947618586;N_BI_GENOS=10309;East_Han_AN=6554;East_Han_AC=1;East_Han_AF
chr2	139925891	.	G	<INS:ME:LINE1>	.	PASS	SVTYPE=INS:ME:LINE1;END=139925897;SVLEN=548;AN=20618;AC=1;AF=4.850130953535746e-05;N_HET=1;N_HOMALT=0;N_HOMREF=10308;FREQ_HET=9.700261907071492e-05;FREQ_HOMALT=0.0;FREQ_HOMREF=0.9999029973809292;N_BI_GENOS=10309;North_Han_AN=2272;North_Han_AC=1;North_Han_
chr2	140052063	.	T	<INS:ME:LINE1>	.	PASS	SVTYPE=INS:ME:LINE1;END=140052081;SVLEN=423;AN=20618;AC=29;AF=0.0014065379765253662;N_HET=27;N_HOMALT=1;N_HOMREF=10281;FREQ_HET=0.0026190707149093026;FREQ_HOMALT=9.700261907071492e-05;FREQ_HOMREF=0.99728392666602;N_BI_GENOS=10309;South_Han_AN=1496;South_H
chr2	140463648	.	G	<INS:ME:LINE1>	.	PASS	SVTYPE=INS:ME:LINE1;END=140463661;SVLEN=2617;AN=20618;AC=1;AF=4.850130953535746e-05;N_HET=1;N_HOMALT=0;N_HOMREF=10308;FREQ_HET=9.700261907071492e-05;FREQ_HOMALT=0.0;FREQ_HOMREF=0.9999029973809292;N_BI_GENOS=10309;East_Han_AN=6554;East_Han_AC=1;East_Han_AF
chr2	140524170	.	A	<INS:ME:LINE1>	.	PASS	SVTYPE=INS:ME:LINE1;END=140524170;SVLEN=257;AN=20618;AC=1;AF=4.850130953535746e-05;N_HET=1;N_HOMALT=0;N_HOMREF=10308;FREQ_HET=9.700261907071492e-05;FREQ_HOMALT=0.0;FREQ_HOMREF=0.9999029973809292;N_BI_GENOS=10309;East_Han_AN=6554;East_Han_AC=1;East_Han_AF=
chr2	140636356	.	A	<INS:ME:LINE1>	.	PASS	SVTYPE=INS:ME:LINE1;END=140636371;SVLEN=920;AN=20618;AC=16;AF=0.0007760209525657193;N_HET=16;N_HOMALT=0;N_HOMREF=10293;FREQ_HET=0.0015520419051314386;FREQ_HOMALT=0.0;FREQ_HOMREF=0.9984479580948685;N_BI_GENOS=10309;South_Han_AN=1496;South_Han_AC=4;South_Ha
chr2	140944726	.	C	<INS:ME:LINE1>	.	PASS	SVTYPE=INS:ME:LINE1;END=140944726;SVLEN=43;AN=20618;AC=1;AF=4.850130953535746e-05;N_HET=1;N_HOMALT=0;N_HOMREF=10308;FREQ_HET=9.700261907071492e-05;FREQ_HOMALT=0.0;FREQ_HOMREF=0.9999029973809292;N_BI_GENOS=10309;East_Han_AN=6554;East_Han_AC=1;East_Han_AF=0
chr2	141017416	.	T	<INS:ME:LINE1>	.	PASS	SVTYPE=INS:ME:LINE1;END=141017428;SVLEN=555;AN=20618;AC=1352;AF=0.06557377049180328;N_HET=1050;N_HOMALT=151;N_HOMREF=9108;FREQ_HET=0.10185275002425065;FREQ_HOMALT=0.01464739547967795;FREQ_HOMREF=0.8834989854496071 4;N_BI_GENOS=10309;South_Han_AN=1496;South_
chr2	141095338	.	G	<INS:ME:LINE1>	.	PASS	SVTYPE=INS:ME:LINE1;END=141095353;SVLEN=197;AN=20618;AC=3886;AF=0.18847608885439907;N_HET=2702;N_HOMALT=592;N_HOMREF=7015;FREQ_HET=0.26210107672907117;FREQ_HOMALT=0.057425550489863225;FREQ_HOMREF=0.6804733727810651;N_BI_GENOS=10309;South_Han_AN=1496;South_
chr2	141189937	.	A	<INS:ME:LINE1>	.	PASS	SVTYPE=INS:ME:LINE1;END=141189969;SVLEN=16;AN=20618;AC=1;AF=4.850130953535746e-05;N_HET=1;N_HOMALT=0;N_HOMREF=10308;FREQ_HET=9.700261907071492e-05;FREQ_HOMALT=0.0;FREQ_HOMREF=0.9999029973809292;N_BI_GENOS=10309;East_Han_AN=6554;East_Han_AC=1;East_Han_AF=0
chr2	141190100	.	A	<INS:ME:LINE1>	.	PASS	SVTYPE=INS:ME:LINE1;END=141190114;SVLEN=565;AN=20618;AC=3;AF=0.00014550392860607236;N_HET=3;N_HOMALT=0;N_HOMREF=10306;FREQ_HET=0.0002910078572121447;FREQ_HOMALT=0.0;FREQ_HOMREF=0.9997089921427879;N_BI_GENOS=10309;East_Han_AN=6554;East_Han_AC=3;East_Han_AF
chr2	141639330	.	T	<INS:ME:LINE1>	.	PASS	SVTYPE=INS:ME:LINE1;END=141639330;SVLEN=0;AN=20618;AC=6;AF=0.0002910078572121447;N_HET=0;N_HOMALT=3;N_HOMREF=10306;FREQ_HET=0.0;FREQ_HOMALT=0.0002910078572121447;FREQ_HOMREF=0.9997089921427879;N_BI_GENOS=10309;East_Han_AN=6554;East_Han_AC=4;East_Han_AF=0.
chr2	141822130	.	A	<INS:ME:LINE1>	.	PASS	SVTYPE=INS:ME:LINE1;END=141822130;SVLEN=0;AN=20618;AC=1;AF=4.850130953535746e-05;N_HET=1;N_HOMALT=0;N_HOMREF=10308;FREQ_HET=9.700261907071492e-05;FREQ_HOMALT=0.0;FREQ_HOMREF=0.9999029973809292;N_BI_GENOS=10309;Southeast_Han_AN=1908;Southeast_Han_AC=1;Sout
chr2	141994148	.	C	<INS:ME:LINE1>	.	PASS	SVTYPE=INS:ME:LINE1;END=141994162;SVLEN=232;AN=20618;AC=1;AF=4.850130953535746e-05;N_HET=1;N_HOMALT=0;N_HOMREF=10308;FREQ_HET=9.700261907071492e-05;FREQ_HOMALT=0.0;FREQ_HOMREF=0.9999029973809292;N_BI_GENOS=10309;South_Han_AN=1496;South_Han_AC=1;South_Han_
chr2	142007614	.	A	<INS:ME:LINE1>	.	PASS	SVTYPE=INS:ME:LINE1;END=142007626;SVLEN=1006;AN=20618;AC=1;AF=4.850130953535746e-05;N_HET=1;N_HOMALT=0;N_HOMREF=10308;FREQ_HET=9.700261907071492e-05;FREQ_HOMALT=0.0;FREQ_HOMREF=0.9999029973809292;N_BI_GENOS=10309;Central_Han_AN=2128;Central_Han_AC=1;Centr
chr2	142024912	.	A	<INS:ME:LINE1>	.	PASS	SVTYPE=INS:ME:LINE1;END=142024925;SVLEN=325;AN=20618;AC=1;AF=4.850130953535746e-05;N_HET=1;N_HOMALT=0;N_HOMREF=10308;FREQ_HET=9.700261907071492e-05;FREQ_HOMALT=0.0;FREQ_HOMREF=0.9999029973809292;N_BI_GENOS=10309;East_Han_AN=6554;East_Han_AC=1;East_Han_AF=
chr2	142027732	.	G	<INS:ME:LINE1>	.	PASS	SVTYPE=INS:ME:LINE1;END=142027737;SVLEN=975;AN=20618;AC=3570;AF=0.1731496750412261;N_HET=2564;N_HOMALT=503;N_HOMREF=7242;FREQ_HET=0.24871471529731304;FREQ_HOMALT=0.0487923173925696;FREQ_HOMREF=0.7024929673101173;N_BI_GENOS=10309;South_Han_AN=1496;South_Ha
chr2	142173843	.	A	<INS:ME:LINE1>	.	PASS	SVTYPE=INS:ME:LINE1;END=142173857;SVLEN=1389;AN=20618;AC=1;AF=4.850130953535746e-05;N_HET=1;N_HOMALT=0;N_HOMREF=10308;FREQ_HET=9.700261907071492e-05;FREQ_HOMALT=0.0;FREQ_HOMREF=0.9999029973809292;N_BI_GENOS=10309;Manchu_AN=416;Manchu_AC=1;Manchu_AF=0.0024
chr2	142345589	.	G	<INS:ME:LINE1>	.	PASS	SVTYPE=INS:ME:LINE1;END=142345608;SVLEN=6000;AN=20618;AC=1;AF=4.850130953535746e-05;N_HET=1;N_HOMALT=0;N_HOMREF=10308;FREQ_HET=9.700261907071492e-05;FREQ_HOMALT=0.0;FREQ_HOMREF=0.9999029973809292;N_BI_GENOS=10309;North_Han_AN=2272;North_Han_AC=1;North_Han
chr2	142470147	.	A	<INS:ME:LINE1>	.	PASS	SVTYPE=INS:ME:LINE1;END=142470166;SVLEN=5997;AN=20618;AC=1;AF=4.850130953535746e-05;N_HET=1;N_HOMALT=0;N_HOMREF=10308;FREQ_HET=9.700261907071492e-05;FREQ_HOMALT=0.0;FREQ_HOMREF=0.9999029973809292;N_BI_GENOS=10309;Northwest_Han_AN=1296;Northwest_Han_AC=1;N
chr2	143253209	.	A	<INS:ME:LINE1>	.	PASS	SVTYPE=INS:ME:LINE1;END=143253224;SVLEN=5999;AN=20618;AC=1639;AF=0.07949364632845086;N_HET=1387;N_HOMALT=126;N_HOMREF=8796;FREQ_HET=0.13454263265108157;FREQ_HOMALT=0.012222330002910078;FREQ_HOMREF=0.8532350373460084;N_BI_GENOS=10309;South_Han_AN=1496;Sout
chr2	143313882	.	A	<INS:ME:LINE1>	.	PASS	SVTYPE=INS:ME:LINE1;END=143313893;SVLEN=292;AN=20618;AC=2;AF=9.700261907071492e-05;N_HET=2;N_HOMALT=0;N_HOMREF=10307;FREQ_HET=0.00019400523814142983;FREQ_HOMALT=0.0;FREQ_HOMREF=0.9998059947618586;N_BI_GENOS=10309;East_Han_AN=6554;East_Han_AC=2;East_Han_AF
chr2	143489057	.	C	<INS:ME:LINE1>	.	PASS	SVTYPE=INS:ME:LINE1;END=143489067;SVLEN=2490;AN=20618;AC=1;AF=4.850130953535746e-05;N_HET=1;N_HOMALT=0;N_HOMREF=10308;FREQ_HET=9.700261907071492e-05;FREQ_HOMALT=0.0;FREQ_HOMREF=0.9999029973809292;N_BI_GENOS=10309;East_Han_AN=6554;East_Han_AC=1;East_Han_AF
chr2	143622085	.	T	<INS:ME:LINE1>	.	PASS	SVTYPE=INS:ME:LINE1;END=143622099;SVLEN=6006;AN=20618;AC=1;AF=4.850130953535746e-05;N_HET=1;N_HOMALT=0;N_HOMREF=10308;FREQ_HET=9.700261907071492e-05;FREQ_HOMALT=0.0;FREQ_HOMREF=0.9999029973809292;N_BI_GENOS=10309;Lingnan_Han_AN=1888;Lingnan_Han_AC=1;Lingn
chr2	143826031	.	A	<INS:ME:LINE1>	.	PASS	SVTYPE=INS:ME:LINE1;END=143826050;SVLEN=6031;AN=20618;AC=2;AF=9.700261907071492e-05;N_HET=2;N_HOMALT=0;N_HOMREF=10307;FREQ_HET=0.00019400523814142983;FREQ_HOMALT=0.0;FREQ_HOMREF=0.9998059947618586;N_BI_GENOS=10309;South_Han_AN=1496;South_Han_AC=1;South_Ha
chr2	143961414	.	T	<INS:ME:LINE1>	.	PASS	SVTYPE=INS:ME:LINE1;END=143961429;SVLEN=6062;AN=20618;AC=1;AF=4.850130953535746e-05;N_HET=1;N_HOMALT=0;N_HOMREF=10308;FREQ_HET=9.700261907071492e-05;FREQ_HOMALT=0.0;FREQ_HOMREF=0.9999029973809292;N_BI_GENOS=10309;East_Han_AN=6554;East_Han_AC=1;East_Han_AF
chr2	144361747	.	G	<INS:ME:LINE1>	.	PASS	SVTYPE=INS:ME:LINE1;END=144361761;SVLEN=355;AN=20618;AC=4;AF=0.00019400523814142983;N_HET=4;N_HOMALT=0;N_HOMREF=10305;FREQ_HET=0.00038801047628285966;FREQ_HOMALT=0.0;FREQ_HOMREF=0.9996119895237171;N_BI_GENOS=10309;South_Han_AN=1496;South_Han_AC=2;South_Ha
chr2	144546810	.	T	<INS:ME:LINE1>	.	PASS	SVTYPE=INS:ME:LINE1;END=144546827;SVLEN=405;AN=20618;AC=1;AF=4.850130953535746e-05;N_HET=1;N_HOMALT=0;N_HOMREF=10308;FREQ_HET=9.700261907071492e-05;FREQ_HOMALT=0.0;FREQ_HOMREF=0.9999029973809292;N_BI_GENOS=10309;East_Han_AN=6554;East_Han_AC=1;East_Han_AF=
chr2	144654041	.	G	<INS:ME:LINE1>	.	PASS	SVTYPE=INS:ME:LINE1;END=144654054;SVLEN=6015;AN=20618;AC=3;AF=0.00014550392860607236;N_HET=3;N_HOMALT=0;N_HOMREF=10306;FREQ_HET=0.0002910078572121447;FREQ_HOMALT=0.0;FREQ_HOMREF=0.9997089921427879;N_BI_GENOS=10309;East_Han_AN=6554;East_Han_AC=3;East_Han_A
chr2	144925822	.	G	<INS:ME:LINE1>	.	PASS	SVTYPE=INS:ME:LINE1;END=144925840;SVLEN=1994;AN=20618;AC=1;AF=4.850130953535746e-05;N_HET=1;N_HOMALT=0;N_HOMREF=10308;FREQ_HET=9.700261907071492e-05;FREQ_HOMALT=0.0;FREQ_HOMREF=0.9999029973809292;N_BI_GENOS=10309;Yi_AN=422;Yi_AC=1;Yi_AF=0.0023696682464454
chr2	144944976	.	T	<INS:ME:LINE1>	.	PASS	SVTYPE=INS:ME:LINE1;END=144944993;SVLEN=824;AN=20618;AC=1;AF=4.850130953535746e-05;N_HET=1;N_HOMALT=0;N_HOMREF=10308;FREQ_HET=9.700261907071492e-05;FREQ_HOMALT=0.0;FREQ_HOMREF=0.9999029973809292;N_BI_GENOS=10309;East_Han_AN=6554;East_Han_AC=1;East_Han_AF=
chr2	145323200	.	A	<INS:ME:LINE1>	.	PASS	SVTYPE=INS:ME:LINE1;END=145323214;SVLEN=964;AN=20618;AC=1;AF=4.850130953535746e-05;N_HET=1;N_HOMALT=0;N_HOMREF=10308;FREQ_HET=9.700261907071492e-05;FREQ_HOMALT=0.0;FREQ_HOMREF=0.9999029973809292;N_BI_GENOS=10309;South_Han_AN=1496;South_Han_AC=1;South_Han_
chr2	145617441	.	A	<INS:ME:LINE1>	.	PASS	SVTYPE=INS:ME:LINE1;END=145617441;SVLEN=0;AN=20618;AC=1;AF=4.850130953535746e-05;N_HET=1;N_HOMALT=0;N_HOMREF=10308;FREQ_HET=9.700261907071492e-05;FREQ_HOMALT=0.0;FREQ_HOMREF=0.9999029973809292;N_BI_GENOS=10309;Southeast_Han_AN=1908;Southeast_Han_AC=1;Sout
chr2	145662769	.	G	<INS:ME:LINE1>	.	PASS	SVTYPE=INS:ME:LINE1;END=145662778;SVLEN=1937;AN=20618;AC=1;AF=4.850130953535746e-05;N_HET=1;N_HOMALT=0;N_HOMREF=10308;FREQ_HET=9.700261907071492e-05;FREQ_HOMALT=0.0;FREQ_HOMREF=0.9999029973809292;N_BI_GENOS=10309;Hui_AN=444;Hui_AC=1;Hui_AF=0.0022522522522522
chr2	146183162	.	T	<INS:ME:LINE1>	.	PASS	SVTYPE=INS:ME:LINE1;END=146183172;SVLEN=1088;AN=20618;AC=4;AF=0.00019400523814142983;N_HET=4;N_HOMALT=0;N_HOMREF=10305;FREQ_HET=0.00038801047628285966;FREQ_HOMALT=0.0;FREQ_HOMREF=0.9996119895237171;N_BI_GENOS=10309;South_Han_AN=1496;South_Han_AC=1;South_H
chr2	146216819	.	A	<INS:ME:LINE1>	.	PASS	SVTYPE=INS:ME:LINE1;END=146216837;SVLEN=2220;AN=20618;AC=1;AF=4.850130953535746e-05;N_HET=1;N_HOMALT=0;N_HOMREF=10308;FREQ_HET=9.700261907071492e-05;FREQ_HOMALT=0.0;FREQ_HOMREF=0.9999029973809292;N_BI_GENOS=10309;Northwest_Han_AN=1296;Northwest_Han_AC=1;Northwest_H
chr2	146229839	.	A	<INS:ME:LINE1>	.	PASS	SVTYPE=INS:ME:LINE1;END=146229855;SVLEN=732;AN=20618;AC=2;AF=9.700261907071492e-05;N_HET=2;N_HOMALT=0;N_HOMREF=10307;FREQ_HET=0.00019400523814142983;FREQ_HOMALT=0.0;FREQ_HOMREF=0.9998059947618586;N_BI_GENOS=10309;South_Han_AN=1496;South_Han_AC=1;South_Han
chr2	146263356	.	A	<INS:ME:LINE1>	.	PASS	SVTYPE=INS:ME:LINE1;END=146263358;SVLEN=1436;AN=20618;AC=16494;AF=0.7999805994761858;N_HET=1028;N_HOMALT=7733;N_HOMREF=1548;FREQ_HET=0.09971869240469493;FREQ_HOMALT=0.7501212532738384;FREQ_HOMREF=0.15016005432146667;N_BI_GENOS=10309;South_Han_AN=1496;Sout
chr2	146743750	.	A	<INS:ME:LINE1>	.	PASS	SVTYPE=INS:ME:LINE1;END=146743764;SVLEN=49;AN=20618;AC=1;AF=4.850130953535746e-05;N_HET=1;N_HOMALT=0;N_HOMREF=10308;FREQ_HET=9.700261907071492e-05;FREQ_HOMALT=0.0;FREQ_HOMREF=0.9999029973809292;N_BI_GENOS=10309;Central_Han_AN=2128;Central_Han_AC=1;Central
chr2	146923344	.	G	<INS:ME:LINE1>	.	PASS	SVTYPE=INS:ME:LINE1;END=146923353;SVLEN=605;AN=20618;AC=14;AF=0.0006790183334950044;N_HET=12;N_HOMALT=1;N_HOMREF=10296;FREQ_HET=0.0011640314288485789;FREQ_HOMALT=9.700261907071492e-05;FREQ_HOMREF=0.9987389659520807;N_BI_GENOS=10309;South_Han_AN=1496;South
chr2	146962191	.	C	<INS:ME:LINE1>	.	PASS	SVTYPE=INS:ME:LINE1;END=146962205;SVLEN=5980;AN=20618;AC=1;AF=4.850130953535746e-05;N_HET=1;N_HOMALT=0;N_HOMREF=10308;FREQ_HET=9.700261907071492e-05;FREQ_HOMALT=0.0;FREQ_HOMREF=0.9999029973809292;N_BI_GENOS=10309;Lingnan_Han_AN=1888;Lingnan_Han_AC=1;Lingn
chr2	147134905	.	T	<INS:ME:LINE1>	.	PASS	SVTYPE=INS:ME:LINE1;END=147134918;SVLEN=6006;AN=20618;AC=8;AF=0.00038801047628285966;N_HET=8;N_HOMALT=0;N_HOMREF=10301;FREQ_HET=0.0007760209525657193;FREQ_HOMALT=0.0;FREQ_HOMREF=0.9992239790474343;N_BI_GENOS=10309;Northwest_Han_AN=1296;Northwest_Han_AC=2;
chr2	147476963	.	A	<INS:ME:LINE1>	.	PASS	SVTYPE=INS:ME:LINE1;END=147476964;SVLEN=179;AN=20618;AC=1;AF=4.850130953535746e-05;N_HET=1;N_HOMALT=0;N_HOMREF=10308;FREQ_HET=9.700261907071492e-05;FREQ_HOMALT=0.0;FREQ_HOMREF=0.9999029973809292;N_BI_GENOS=10309;Central_Han_AN=2128;Central_Han_AC=1;Centra
chr2	147612415	.	A	<INS:ME:LINE1>	.	PASS	SVTYPE=INS:ME:LINE1;END=147612430;SVLEN=293;AN=20618;AC=1;AF=4.850130953535746e-05;N_HET=1;N_HOMALT=0;N_HOMREF=10308;FREQ_HET=9.700261907071492e-05;FREQ_HOMALT=0.0;FREQ_HOMREF=0.9999029973809292;N_BI_GENOS=10309;Central_Han_AN=2128;Central_Han_AC=1;Centra
chr2	147818758	.	G	<INS:ME:LINE1>	.	PASS	SVTYPE=INS:ME:LINE1;END=147818771;SVLEN=5998;AN=20618;AC=1;AF=4.850130953535746e-05;N_HET=1;N_HOMALT=0;N_HOMREF=10308;FREQ_HET=9.700261907071492e-05;FREQ_HOMALT=0.0;FREQ_HOMREF=0.9999029973809292;N_BI_GENOS=10309;East_Han_AN=6554;East_Han_AC=1;East_Han_AF
chr2	147891873	.	T	<INS:ME:LINE1>	.	PASS	SVTYPE=INS:ME:LINE1;END=147891883;SVLEN=3304;AN=20618;AC=1;AF=4.850130953535746e-05;N_HET=1;N_HOMALT=0;N_HOMREF=10308;FREQ_HET=9.700261907071492e-05;FREQ_HOMALT=0.0;FREQ_HOMREF=0.9999029973809292;N_BI_GENOS=10309;Central_Han_AN=2128;Central_Han_AC=1;Centr
chr2	148140626	.	T	<INS:ME:LINE1>	.	PASS	SVTYPE=INS:ME:LINE1;END=148140639;SVLEN=6016;AN=20618;AC=1;AF=4.850130953535746e-05;N_HET=1;N_HOMALT=0;N_HOMREF=10308;FREQ_HET=9.700261907071492e-05;FREQ_HOMALT=0.0;FREQ_HOMREF=0.9999029973809292;N_BI_GENOS=10309;East_Han_AN=6554;East_Han_AC=1;East_Han_AF
chr2	148419063	.	G	<INS:ME:LINE1>	.	PASS	SVTYPE=INS:ME:LINE1;END=148419063;SVLEN=581;AN=20618;AC=1;AF=4.850130953535746e-05;N_HET=1;N_HOMALT=0;N_HOMREF=10308;FREQ_HET=9.700261907071492e-05;FREQ_HOMALT=0.0;FREQ_HOMREF=0.9999029973809292;N_BI_GENOS=10309;East_Han_AN=6554;East_Han_AC=1;East_Han_AF=
chr2	148554526	.	C	<INS:ME:LINE1>	.	PASS	SVTYPE=INS:ME:LINE1;END=148554529;SVLEN=895;AN=20618;AC=1;AF=4.850130953535746e-05;N_HET=1;N_HOMALT=0;N_HOMREF=10308;FREQ_HET=9.700261907071492e-05;FREQ_HOMALT=0.0;FREQ_HOMREF=0.9999029973809292;N_BI_GENOS=10309;East_Han_AN=6554;East_Han_AC=1;East_Han_AF=
chr2	149127011	.	G	<INS:ME:LINE1>	.	PASS	SVTYPE=INS:ME:LINE1;END=149127011;SVLEN=549;AN=20618;AC=1;AF=4.850130953535746e-05;N_HET=1;N_HOMALT=0;N_HOMREF=10308;FREQ_HET=9.700261907071492e-05;FREQ_HOMALT=0.0;FREQ_HOMREF=0.9999029973809292;N_BI_GENOS=10309;Southeast_Han_AN=1908;Southeast_Han_AC=1;So
chr2	149486429	.	T	<INS:ME:LINE1>	.	PASS	SVTYPE=INS:ME:LINE1;END=149486442;SVLEN=6007;AN=20618;AC=1;AF=4.850130953535746e-05;N_HET=1;N_HOMALT=0;N_HOMREF=10308;FREQ_HET=9.700261907071492e-05;FREQ_HOMALT=0.0;FREQ_HOMREF=0.9999029973809292;N_BI_GENOS=10309;Central_Han_AN=2128;Central_Han_AC=1;Centr
chr2	149569622	.	G	<INS:ME:LINE1>	.	PASS	SVTYPE=INS:ME:LINE1;END=149569638;SVLEN=3585;AN=20618;AC=1;AF=4.850130953535746e-05;N_HET=1;N_HOMALT=0;N_HOMREF=10308;FREQ_HET=9.700261907071492e-05;FREQ_HOMALT=0.0;FREQ_HOMREF=0.9999029973809292;N_BI_GENOS=10309;Hui_AN=444;Hui_AC=1;Hui_AF=0.0022522522522522
chr2	150138166	.	A	<INS:ME:LINE1>	.	PASS	SVTYPE=INS:ME:LINE1;END=150138183;SVLEN=6020;AN=20618;AC=2;AF=9.700261907071492e-05;N_HET=0;N_HOMALT=1;N_HOMREF=10308;FREQ_HET=0.0;FREQ_HOMALT=9.700261907071492e-05;FREQ_HOMREF=0.9999029973809292;N_BI_GENOS=10309;East_Han_AN=6554;East_Han_AC=2;East_Han_AF
chr2	150640160	.	A	<INS:ME:LINE1>	.	PASS	SVTYPE=INS:ME:LINE1;END=150640174;SVLEN=6004;AN=20618;AC=1;AF=4.850130953535746e-05;N_HET=1;N_HOMALT=0;N_HOMREF=10308;FREQ_HET=9.700261907071492e-05;FREQ_HOMALT=0.0;FREQ_HOMREF=0.9999029973809292;N_BI_GENOS=10309;Central_Han_AN=2128;Central_Han_AC=1;Centr
chr2	150687016	.	G	<INS:ME:LINE1>	.	PASS	SVTYPE=INS:ME:LINE1;END=150687025;SVLEN=3991;AN=20618;AC=1;AF=4.850130953535746e-05;N_HET=1;N_HOMALT=0;N_HOMREF=10308;FREQ_HET=9.700261907071492e-05;FREQ_HOMALT=0.0;FREQ_HOMREF=0.9999029973809292;N_BI_GENOS=10309;Central_Han_AN=2128;Central_Han_AC=1;Centr
chr2	150745956	.	A	<INS:ME:LINE1>	.	PASS	SVTYPE=INS:ME:LINE1;END=150745972;SVLEN=5967;AN=20618;AC=4;AF=0.00019400523814142983;N_HET=4;N_HOMALT=0;N_HOMREF=10305;FREQ_HET=0.00038801047628285966;FREQ_HOMALT=0.0;FREQ_HOMREF=0.9996119895237171;N_BI_GENOS=10309;North_Han_AN=2272;North_Han_AC=1;North_H
chr2	150976371	.	T	<INS:ME:LINE1>	.	PASS	SVTYPE=INS:ME:LINE1;END=150976386;SVLEN=4337;AN=20618;AC=4;AF=0.00019400523814142983;N_HET=4;N_HOMALT=0;N_HOMREF=10305;FREQ_HET=0.00038801047628285966;FREQ_HOMALT=0.0;FREQ_HOMREF=0.9996119895237171;N_BI_GENOS=10309;East_Han_AN=6554;East_Han_AC=3;East_Han_
chr2	151242240	.	T	<INS:ME:LINE1>	.	PASS	SVTYPE=INS:ME:LINE1;END=151242259;SVLEN=573;AN=20618;AC=1;AF=4.850130953535746e-05;N_HET=1;N_HOMALT=0;N_HOMREF=10308;FREQ_HET=9.700261907071492e-05;FREQ_HOMALT=0.0;FREQ_HOMREF=0.9999029973809292;N_BI_GENOS=10309;Central_Han_AN=2128;Central_Han_AC=1;Centra
chr2	151456042	.	C	<INS:ME:LINE1>	.	PASS	SVTYPE=INS:ME:LINE1;END=151456058;SVLEN=214;AN=20618;AC=5;AF=0.00024250654767678728;N_HET=5;N_HOMALT=0;N_HOMREF=10304;FREQ_HET=0.00048501309535357455;FREQ_HOMALT=0.0;FREQ_HOMREF=0.9995149869046465;N_BI_GENOS=10309;East_Han_AN=6554;East_Han_AC=5;East_Han_A
chr2	152466395	.	A	<INS:ME:LINE1>	.	PASS	SVTYPE=INS:ME:LINE1;END=152466412;SVLEN=6000;AN=20618;AC=1;AF=4.850130953535746e-05;N_HET=1;N_HOMALT=0;N_HOMREF=10308;FREQ_HET=9.700261907071492e-05;FREQ_HOMALT=0.0;FREQ_HOMREF=0.9999029973809292;N_BI_GENOS=10309;South_Han_AN=1496;South_Han_AC=1;South_Han
chr2	152653177	.	A	<INS:ME:LINE1>	.	PASS	SVTYPE=INS:ME:LINE1;END=152653194;SVLEN=5998;AN=20618;AC=1;AF=4.850130953535746e-05;N_HET=1;N_HOMALT=0;N_HOMREF=10308;FREQ_HET=9.700261907071492e-05;FREQ_HOMALT=0.0;FREQ_HOMREF=0.9999029973809292;N_BI_GENOS=10309;North_Han_AN=2272;North_Han_AC=1;North_Han
chr2	152839049	.	A	<INS:ME:LINE1>	.	PASS	SVTYPE=INS:ME:LINE1;END=152839063;SVLEN=5999;AN=20618;AC=2;AF=9.700261907071492e-05;N_HET=2;N_HOMALT=0;N_HOMREF=10307;FREQ_HET=0.00019400523814142983;FREQ_HOMALT=0.0;FREQ_HOMREF=0.9998059947618586;N_BI_GENOS=10309;Tibetan_AN=414;Tibetan_AC=2;Tibetan_AF=0.
chr2	152907444	.	A	<INS:ME:LINE1>	.	PASS	SVTYPE=INS:ME:LINE1;END=152907459;SVLEN=1049;AN=20618;AC=2;AF=9.700261907071492e-05;N_HET=2;N_HOMALT=0;N_HOMREF=10307;FREQ_HET=0.00019400523814142983;FREQ_HOMALT=0.0;FREQ_HOMREF=0.9998059947618586;N_BI_GENOS=10309;Northwest_Han_AN=1296;Northwest_Han_AC=1;
chr2	153244074	.	G	<INS:ME:LINE1>	.	PASS	SVTYPE=INS:ME:LINE1;END=153244085;SVLEN=5998;AN=20618;AC=17;AF=0.0008245222621010767;N_HET=17;N_HOMALT=0;N_HOMREF=10292;FREQ_HET=0.0016490445242021534;FREQ_HOMALT=0.0;FREQ_HOMREF=0.9983509554757979;N_BI_GENOS=10309;South_Han_AN=1496;South_Han_AC=5;South_H
chr2	153667548	.	T	<INS:ME:LINE1>	.	PASS	SVTYPE=INS:ME:LINE1;END=153667561;SVLEN=2248;AN=20618;AC=1;AF=4.850130953535746e-05;N_HET=1;N_HOMALT=0;N_HOMREF=10308;FREQ_HET=9.700261907071492e-05;FREQ_HOMALT=0.0;FREQ_HOMREF=0.9999029973809292;N_BI_GENOS=10309;Southeast_Han_AN=1908;Southeast_Han_AC=1;S
chr2	153677695	.	C	<INS:ME:LINE1>	.	PASS	SVTYPE=INS:ME:LINE1;END=153677695;SVLEN=515;AN=20618;AC=2;AF=9.700261907071492e-05;N_HET=0;N_HOMALT=1;N_HOMREF=10308;FREQ_HET=0.0;FREQ_HOMALT=9.700261907071492e-05;FREQ_HOMREF=0.9999029973809292;N_BI_GENOS=10309;East_Han_AN=6554;East_Han_AC=2;East_Han_AF=
chr2	153775113	.	A	<INS:ME:LINE1>	.	PASS	SVTYPE=INS:ME:LINE1;END=153775126;SVLEN=6029;AN=20618;AC=2;AF=9.700261907071492e-05;N_HET=0;N_HOMALT=1;N_HOMREF=10308;FREQ_HET=0.0;FREQ_HOMALT=9.700261907071492e-05;FREQ_HOMREF=0.9999029973809292;N_BI_GENOS=10309;Lingnan_Han_AN=1888;Lingnan_Han_AC=2;Lingn
chr2	153954210	.	T	<INS:ME:LINE1>	.	PASS	SVTYPE=INS:ME:LINE1;END=153954228;SVLEN=913;AN=20618;AC=1;AF=4.850130953535746e-05;N_HET=1;N_HOMALT=0;N_HOMREF=10308;FREQ_HET=9.700261907071492e-05;FREQ_HOMALT=0.0;FREQ_HOMREF=0.9999029973809292;N_BI_GENOS=10309;Central_Han_AN=2128;Central_Han_AC=1;Centra
chr2	154040916	.	T	<INS:ME:LINE1>	.	PASS	SVTYPE=INS:ME:LINE1;END=154040934;SVLEN=1462;AN=20618;AC=1;AF=4.850130953535746e-05;N_HET=1;N_HOMALT=0;N_HOMREF=10308;FREQ_HET=9.700261907071492e-05;FREQ_HOMALT=0.0;FREQ_HOMREF=0.9999029973809292;N_BI_GENOS=10309;Tibetan_AN=414;Tibetan_AC=1;Tibetan_AF=0.0
chr2	154084166	.	T	<INS:ME:LINE1>	.	PASS	SVTYPE=INS:ME:LINE1;END=154084181;SVLEN=532;AN=20618;AC=1;AF=4.850130953535746e-05;N_HET=1;N_HOMALT=0;N_HOMREF=10308;FREQ_HET=9.700261907071492e-05;FREQ_HOMALT=0.0;FREQ_HOMREF=0.9999029973809292;N_BI_GENOS=10309;East_Han_AN=6554;East_Han_AC=1;East_Han_AF=
chr2	154135849	.	A	<INS:ME:LINE1>	.	PASS	SVTYPE=INS:ME:LINE1;END=154135849;SVLEN=506;AN=20618;AC=2;AF=9.700261907071492e-05;N_HET=2;N_HOMALT=0;N_HOMREF=10307;FREQ_HET=0.00019400523814142983;FREQ_HOMALT=0.0;FREQ_HOMREF=0.9998059947618586;N_BI_GENOS=10309;Northwest_Han_AN=1296;Northwest_Han_AC=1;N
chr2	154375720	.	A	<INS:ME:LINE1>	.	PASS	SVTYPE=INS:ME:LINE1;END=154375736;SVLEN=2242;AN=20618;AC=1;AF=4.850130953535746e-05;N_HET=1;N_HOMALT=0;N_HOMREF=10308;FREQ_HET=9.700261907071492e-05;FREQ_HOMALT=0.0;FREQ_HOMREF=0.9999029973809292;N_BI_GENOS=10309;East_Han_AN=6554;East_Han_AC=1;East_Han_AF
chr2	154475949	.	A	<INS:ME:LINE1>	.	PASS	SVTYPE=INS:ME:LINE1;END=154475964;SVLEN=6027;AN=20618;AC=1;AF=4.850130953535746e-05;N_HET=1;N_HOMALT=0;N_HOMREF=10308;FREQ_HET=9.700261907071492e-05;FREQ_HOMALT=0.0;FREQ_HOMREF=0.9999029973809292;N_BI_GENOS=10309;East_Han_AN=6554;East_Han_AC=1;East_Han_AF
chr2	155001269	.	A	<INS:ME:LINE1>	.	PASS	SVTYPE=INS:ME:LINE1;END=155001284;SVLEN=6000;AN=20618;AC=2;AF=9.700261907071492e-05;N_HET=2;N_HOMALT=0;N_HOMREF=10307;FREQ_HET=0.00019400523814142983;FREQ_HOMALT=0.0;FREQ_HOMREF=0.9998059947618586;N_BI_GENOS=10309;North_Han_AN=2272;North_Han_AC=1;North_Ha
chr2	155506001	.	A	<INS:ME:LINE1>	.	PASS	SVTYPE=INS:ME:LINE1;END=155506001;SVLEN=728;AN=20618;AC=1;AF=4.850130953535746e-05;N_HET=1;N_HOMALT=0;N_HOMREF=10308;FREQ_HET=9.700261907071492e-05;FREQ_HOMALT=0.0;FREQ_HOMREF=0.9999029973809292;N_BI_GENOS=10309;East_Han_AN=6554;East_Han_AC=1;East_Han_AF=
chr2	155671309	.	A	<INS:ME:LINE1>	.	PASS	SVTYPE=INS:ME:LINE1;END=155671336;SVLEN=6000;AN=20618;AC=2180;AF=0.10573285478707925;N_HET=592;N_HOMALT=794;N_HOMREF=8923;FREQ_HET=0.057425550489863225;FREQ_HOMALT=0.07702007954214764;FREQ_HOMREF=0.8655543699679892;N_BI_GENOS=10309;South_Han_AN=1496;South
chr2	155671418	.	G	<INS:ME:LINE1>	.	PASS	SVTYPE=INS:ME:LINE1;END=155671445;SVLEN=5999;AN=20618;AC=3;AF=0.00014550392860607236;N_HET=3;N_HOMALT=0;N_HOMREF=10306;FREQ_HET=0.0002910078572121447;FREQ_HOMALT=0.0;FREQ_HOMREF=0.9997089921427879;N_BI_GENOS=10309;East_Han_AN=6554;East_Han_AC=1;East_Han_A
chr2	155671570	.	T	<INS:ME:LINE1>	.	PASS	SVTYPE=INS:ME:LINE1;END=155671570;SVLEN=230;AN=20618;AC=6;AF=0.0002910078572121447;N_HET=6;N_HOMALT=0;N_HOMREF=10303;FREQ_HET=0.0005820157144242894;FREQ_HOMALT=0.0;FREQ_HOMREF=0.9994179842855757;N_BI_GENOS=10309;South_Han_AN=1496;South_Han_AC=1;South_Han_

chr2	155674942	.	C	<INS:ME:LINE1>	.	PASS	SVTYPE=INS:ME:LINE1;END=155674967;SVLEN=1148;AN=20618;AC=1;AF=4.850130953535746e-05;N_HET=1;N_HOMALT=0;N_HOMREF=10308;FREQ_HET=9.700261907071492e-05;FREQ_HOMALT=0.0;FREQ_HOMREF=0.9999029973809292;N_BI_GENOS=10309;East_Han_AN=6554;East_Han_AC=1;East_Han_AF
chr2	156061381	.	A	<INS:ME:LINE1>	.	PASS	SVTYPE=INS:ME:LINE1;END=156061399;SVLEN=4088;AN=20618;AC=4;AF=0.00019400523814142983;N_HET=4;N_HOMALT=0;N_HOMREF=10305;FREQ_HET=0.0003880104762828966;FREQ_HOMALT=0.0;FREQ_HOMREF=0.9996119895237171;N_BI_GENOS=10309;South_Han_AN=1496;South_Han_AC=2;South_H
chr2	156631836	.	T	<INS:ME:LINE1>	.	PASS	SVTYPE=INS:ME:LINE1;END=156631845;SVLEN=6000;AN=20618;AC=1;AF=4.850130953535746e-05;N_HET=1;N_HOMALT=0;N_HOMREF=10308;FREQ_HET=9.700261907071492e-05;FREQ_HOMALT=0.0;FREQ_HOMREF=0.9999029973809292;N_BI_GENOS=10309;Lingnan_Han_AN=1888;Lingnan_Han_AC=1;Lingn
chr2	156682076	.	A	<INS:ME:LINE1>	.	PASS	SVTYPE=INS:ME:LINE1;END=156682093;SVLEN=4999;AN=20618;AC=1;AF=4.850130953535746e-05;N_HET=1;N_HOMALT=0;N_HOMREF=10308;FREQ_HET=9.700261907071492e-05;FREQ_HOMALT=0.0;FREQ_HOMREF=0.9999029973809292;N_BI_GENOS=10309;Southeast_Han_AN=1908;Southeast_Han_AC=1;S
chr2	156789100	.	T	<INS:ME:LINE1>	.	PASS	SVTYPE=INS:ME:LINE1;END=156789115;SVLEN=5985;AN=20618;AC=2;AF=9.700261907071492e-05;N_HET=0;N_HOMALT=1;N_HOMREF=10308;FREQ_HET=0.0;FREQ_HOMALT=9.700261907071492e-05;FREQ_HOMREF=0.9999029973809292;N_BI_GENOS=10309;South_Han_AN=1496;South_Han_AC=2;South_Han
chr2	157449702	.	T	<INS:ME:LINE1>	.	PASS	SVTYPE=INS:ME:LINE1;END=157449720;SVLEN=6017;AN=20618;AC=2;AF=9.700261907071492e-05;N_HET=0;N_HOMALT=1;N_HOMREF=10308;FREQ_HET=0.0;FREQ_HOMALT=9.700261907071492e-05;FREQ_HOMREF=0.9999029973809292;N_BI_GENOS=10309;Yi_AN=422;Yi_AC=2;Yi_AF=0.0047393364928909
chr2	157452107	.	A	<INS:ME:LINE1>	.	PASS	SVTYPE=INS:ME:LINE1;END=157452120;SVLEN=5865;AN=20618;AC=1;AF=4.850130953535746e-05;N_HET=1;N_HOMALT=0;N_HOMREF=10308;FREQ_HET=9.700261907071492e-05;FREQ_HOMALT=0.0;FREQ_HOMREF=0.9999029973809292;N_BI_GENOS=10309;North_Han_AN=2272;North_Han_AC=1;North_Han
chr2	158071694	.	C	<INS:ME:LINE1>	.	PASS	SVTYPE=INS:ME:LINE1;END=158071711;SVLEN=395;AN=20618;AC=3;AF=0.00014550392860607236;N_HET=3;N_HOMALT=0;N_HOMREF=10306;FREQ_HET=0.0002910078572121447;FREQ_HOMALT=0.0;FREQ_HOMREF=0.9997089921427879;N_BI_GENOS=10309;North_Han_AN=2272;North_Han_AC=2;North_Han
chr2	158320261	.	A	<INS:ME:LINE1>	.	PASS	SVTYPE=INS:ME:LINE1;END=158320274;SVLEN=2000;AN=20618;AC=1;AF=4.850130953535746e-05;N_HET=1;N_HOMALT=0;N_HOMREF=10308;FREQ_HET=9.700261907071492e-05;FREQ_HOMALT=0.0;FREQ_HOMREF=0.9999029973809292;N_BI_GENOS=10309;Lingnan_Han_AN=1888;Lingnan_Han_AC=1;Lingn
chr2	158540433	.	A	<INS:ME:LINE1>	.	PASS	SVTYPE=INS:ME:LINE1;END=158540449;SVLEN=120;AN=20618;AC=1;AF=4.850130953535746e-05;N_HET=1;N_HOMALT=0;N_HOMREF=10308;FREQ_HET=9.700261907071492e-05;FREQ_HOMALT=0.0;FREQ_HOMREF=0.9999029973809292;N_BI_GENOS=10309;East_Han_AN=6554;East_Han_AC=1;East_Han_AF=
chr2	158653174	.	A	<INS:ME:LINE1>	.	PASS	SVTYPE=INS:ME:LINE1;END=158653192;SVLEN=431;AN=20618;AC=7;AF=0.0003395091667475022;N_HET=7;N_HOMALT=0;N_HOMREF=10302;FREQ_HET=0.0006790183334950044;FREQ_HOMALT=0.0;FREQ_HOMREF=0.999320981666505;N_BI_GENOS=10309;Northwest_Han_AN=1296;Northwest_Han_AC=1;Nor
chr2	158682210	.	A	<INS:ME:LINE1>	.	PASS	SVTYPE=INS:ME:LINE1;END=158682225;SVLEN=6037;AN=20618;AC=1;AF=4.850130953535746e-05;N_HET=1;N_HOMALT=0;N_HOMREF=10308;FREQ_HET=9.700261907071492e-05;FREQ_HOMALT=0.0;FREQ_HOMREF=0.9999029973809292;N_BI_GENOS=10309;East_Han_AN=6554;East_Han_AC=1;East_Han_AF
chr2	159052547	.	A	<INS:ME:LINE1>	.	PASS	SVTYPE=INS:ME:LINE1;END=159052562;SVLEN=5997;AN=20618;AC=1;AF=4.850130953535746e-05;N_HET=1;N_HOMALT=0;N_HOMREF=10308;FREQ_HET=9.700261907071492e-05;FREQ_HOMALT=0.0;FREQ_HOMREF=0.9999029973809292;N_BI_GENOS=10309;East_Han_AN=6554;East_Han_AC=1;East_Han_AF
chr2	159162115	.	C	<INS:ME:LINE1>	.	PASS	SVTYPE=INS:ME:LINE1;END=159162125;SVLEN=5960;AN=20618;AC=8;AF=0.0003880104762828966;N_HET=8;N_HOMALT=0;N_HOMREF=10301;FREQ_HET=0.0007760209525657193;FREQ_HOMALT=0.0;FREQ_HOMREF=0.9992239790474343;N_BI_GENOS=10309;North_Han_AN=2272;North_Han_AC=2;North_Ha
chr2	159194806	.	T	<INS:ME:LINE1>	.	PASS	SVTYPE=INS:ME:LINE1;END=159194818;SVLEN=227;AN=20618;AC=1;AF=4.850130953535746e-05;N_HET=1;N_HOMALT=0;N_HOMREF=10308;FREQ_HET=9.700261907071492e-05;FREQ_HOMALT=0.0;FREQ_HOMREF=0.9999029973809292;N_BI_GENOS=10309;Southeast_Han_AN=1908;Southeast_Han_AC=1;So
chr2	159391099	.	A	<INS:ME:LINE1>	.	PASS	SVTYPE=INS:ME:LINE1;END=159391104;SVLEN=485;AN=20618;AC=2;AF=9.700261907071492e-05;N_HET=2;N_HOMALT=0;N_HOMREF=10307;FREQ_HET=0.00019400523814142983;FREQ_HOMALT=0.0;FREQ_HOMREF=0.9998059947618586;N_BI_GENOS=10309;South_Han_AN=1496;South_Han_AC=1;South_Han
chr2	160013721	.	T	<INS:ME:LINE1>	.	PASS	SVTYPE=INS:ME:LINE1;END=160013721;SVLEN=0;AN=20618;AC=32;AF=0.0015520419051314386;N_HET=2;N_HOMALT=15;N_HOMREF=10292;FREQ_HET=0.00019400523814142983;FREQ_HOMALT=0.0014550392860607237;FREQ_HOMREF=0.9983509554757979;N_BI_GENOS=10309;North_Han_AN=2272;North_
chr2	160169600	.	C	<INS:ME:LINE1>	.	PASS	SVTYPE=INS:ME:LINE1;END=160169617;SVLEN=6000;AN=20618;AC=4;AF=0.00019400523814142983;N_HET=4;N_HOMALT=0;N_HOMREF=10305;FREQ_HET=0.0003880104762828966;FREQ_HOMALT=0.0;FREQ_HOMREF=0.9996119895237171;N_BI_GENOS=10309;South_Han_AN=1496;South_Han_AC=1;South_H
chr2	160274955	.	A	<INS:ME:LINE1>	.	PASS	SVTYPE=INS:ME:LINE1;END=160274972;SVLEN=810;AN=20618;AC=20;AF=0.0009700261907071491;N_HET=20;N_HOMALT=0;N_HOMREF=10289;FREQ_HET=0.0019400523814142982;FREQ_HOMALT=0.0;FREQ_HOMREF=0.9980599476185857;N_BI_GENOS=10309;South_Han_AN=1496;South_Han_AC=6;South_Ha
chr2	160489320	.	C	<INS:ME:LINE1>	.	PASS	SVTYPE=INS:ME:LINE1;END=160489336;SVLEN=5994;AN=20618;AC=1;AF=4.850130953535746e-05;N_HET=1;N_HOMALT=0;N_HOMREF=10308;FREQ_HET=9.700261907071492e-05;FREQ_HOMALT=0.0;FREQ_HOMREF=0.9999029973809292;N_BI_GENOS=10309;Zhuang_AN=444;Zhuang_AC=1;Zhuang_AF=0.0022
chr2	160614987	.	A	<INS:ME:LINE1>	.	PASS	SVTYPE=INS:ME:LINE1;END=160615008;SVLEN=5999;AN=20618;AC=4;AF=0.00019400523814142983;N_HET=4;N_HOMALT=0;N_HOMREF=10305;FREQ_HET=0.0003880104762828966;FREQ_HOMALT=0.0;FREQ_HOMREF=0.9996119895237171;N_BI_GENOS=10309;South_Han_AN=1496;South_Han_AC=3;South_H
chr2	160780362	.	T	<INS:ME:LINE1>	.	PASS	SVTYPE=INS:ME:LINE1;END=160780362;SVLEN=564;AN=20618;AC=1;AF=4.850130953535746e-05;N_HET=1;N_HOMALT=0;N_HOMREF=10308;FREQ_HET=9.700261907071492e-05;FREQ_HOMALT=0.0;FREQ_HOMREF=0.9999029973809292;N_BI_GENOS=10309;East_Han_AN=6554;East_Han_AC=1;East_Han_AF=
chr2	160781073	.	T	<INS:ME:LINE1>	.	PASS	SVTYPE=INS:ME:LINE1;END=160781073;SVLEN=822;AN=20618;AC=1;AF=4.850130953535746e-05;N_HET=1;N_HOMALT=0;N_HOMREF=10308;FREQ_HET=9.700261907071492e-05;FREQ_HOMALT=0.0;FREQ_HOMREF=0.9999029973809292;N_BI_GENOS=10309;East_Han_AN=6554;East_Han_AC=1;East_Han_AF=
chr2	161171716	.	C	<INS:ME:LINE1>	.	PASS	SVTYPE=INS:ME:LINE1;END=161171716;SVLEN=58;AN=20618;AC=1;AF=4.850130953535746e-05;N_HET=1;N_HOMALT=0;N_HOMREF=10308;FREQ_HET=9.700261907071492e-05;FREQ_HOMALT=0.0;FREQ_HOMREF=0.9999029973809292;N_BI_GENOS=10309;Northwest_Han_AN=1296;Northwest_Han_AC=1;Nor
chr2	161176241	.	T	<INS:ME:LINE1>	.	PASS	SVTYPE=INS:ME:LINE1;END=161176257;SVLEN=323;AN=20618;AC=1;AF=4.850130953535746e-05;N_HET=1;N_HOMALT=0;N_HOMREF=10308;FREQ_HET=9.700261907071492e-05;FREQ_HOMALT=0.0;FREQ_HOMREF=0.9999029973809292;N_BI_GENOS=10309;East_Han_AN=6554;East_Han_AC=1;East_Han_AF=
chr2	161180971	.	A	<INS:ME:LINE1>	.	PASS	SVTYPE=INS:ME:LINE1;END=161180986;SVLEN=1080;AN=20618;AC=8;AF=0.0003880104762828966;N_HET=8;N_HOMALT=0;N_HOMREF=10301;FREQ_HET=0.0007760209525657193;FREQ_HOMALT=0.0;FREQ_HOMREF=0.9992239790474343;N_BI_GENOS=10309;South_Han_AN=1496;South_Han_AC=4;South_Ha
chr2	161256353	.	C	<INS:ME:LINE1>	.	PASS	SVTYPE=INS:ME:LINE1;END=161256353;SVLEN=5994;AN=20618;AC=1;AF=4.850130953535746e-05;N_HET=1;N_HOMALT=0;N_HOMREF=10308;FREQ_HET=9.700261907071492e-05;FREQ_HOMALT=0.0;FREQ_HOMREF=0.9999029973809292;N_BI_GENOS=10309;North_Han_AN=2272;North_Han_AC=1;North_Han
chr2	161256738	.	T	<INS:ME:LINE1>	.	PASS	SVTYPE=INS:ME:LINE1;END=161256751;SVLEN=5579;AN=20618;AC=2;AF=9.700261907071492e-05;N_HET=2;N_HOMALT=0;N_HOMREF=10307;FREQ_HET=0.00019400523814142983;FREQ_HOMALT=0.0;FREQ_HOMREF=0.9998059947618586;N_BI_GENOS=10309;North_Han_AN=2272;North_Han_AC=2;North_Ha
chr2	161330490	.	C	<INS:ME:LINE1>	.	PASS	SVTYPE=INS:ME:LINE1;END=161330505;SVLEN=2016;AN=20618;AC=1;AF=4.850130953535746e-05;N_HET=1;N_HOMALT=0;N_HOMREF=10308;FREQ_HET=9.700261907071492e-05;FREQ_HOMALT=0.0;FREQ_HOMREF=0.9999029973809292;N_BI_GENOS=10309;East_Han_AN=6554;East_Han_AC=1;East_Han_AF
chr2	161362634	.	T	<INS:ME:LINE1>	.	PASS	SVTYPE=INS:ME:LINE1;END=161362634;SVLEN=1568;AN=20618;AC=1;AF=4.850130953535746e-05;N_HET=1;N_HOMALT=0;N_HOMREF=10308;FREQ_HET=9.700261907071492e-05;FREQ_HOMALT=0.0;FREQ_HOMREF=0.9999029973809292;N_BI_GENOS=10309;Northwest_Han_AN=1296;Northwest_Han_AC=1;N
chr2	161574351	.	G	<INS:ME:LINE1>	.	PASS	SVTYPE=INS:ME:LINE1;END=161574364;SVLEN=6184;AN=20618;AC=1;AF=4.850130953535746e-05;N_HET=1;N_HOMALT=0;N_HOMREF=10308;FREQ_HET=9.700261907071492e-05;FREQ_HOMALT=0.0;FREQ_HOMREF=0.9999029973809292;N_BI_GENOS=10309;East_Han_AN=6554;East_Han_AC=1;East_Han_AF
chr2	161603114	.	A	<INS:ME:LINE1>	.	PASS	SVTYPE=INS:ME:LINE1;END=161603132;SVLEN=987;AN=20618;AC=1;AF=4.850130953535746e-05;N_HET=1;N_HOMALT=0;N_HOMREF=10308;FREQ_HET=9.700261907071492e-05;FREQ_HOMALT=0.0;FREQ_HOMREF=0.9999029973809292;N_BI_GENOS=10309;Northwest_Han_AN=1296;Northwest_Han_AC=1;No
chr2	161713768	.	T	<INS:ME:LINE1>	.	PASS	SVTYPE=INS:ME:LINE1;END=161713784;SVLEN=711;AN=20618;AC=1;AF=4.850130953535746e-05;N_HET=1;N_HOMALT=0;N_HOMREF=10308;FREQ_HET=9.700261907071492e-05;FREQ_HOMALT=0.0;FREQ_HOMREF=0.9999029973809292;N_BI_GENOS=10309;Tibetan_AN=414;Tibetan_AC=1;Tibetan_AF=0.00
chr2	161756073	.	A	<INS:ME:LINE1>	.	PASS	SVTYPE=INS:ME:LINE1;END=161756083;SVLEN=170;AN=20618;AC=1;AF=4.850130953535746e-05;N_HET=1;N_HOMALT=0;N_HOMREF=10308;FREQ_HET=9.700261907071492e-05;FREQ_HOMALT=0.0;FREQ_HOMREF=0.9999029973809292;N_BI_GENOS=10309;North_Han_AN=2272;North_Han_AC=1;North_Han_
chr2	161798341	.	A	<INS:ME:LINE1>	.	PASS	SVTYPE=INS:ME:LINE1;END=161798359;SVLEN=702;AN=20618;AC=1;AF=4.850130953535746e-05;N_HET=1;N_HOMALT=0;N_HOMREF=10308;FREQ_HET=9.700261907071492e-05;FREQ_HOMALT=0.0;FREQ_HOMREF=0.9999029973809292;N_BI_GENOS=10309;Southeast_Han_AN=1908;Southeast_Han_AC=1;So
chr2	161805624	.	G	<INS:ME:LINE1>	.	PASS	SVTYPE=INS:ME:LINE1;END=161805638;SVLEN=6010;AN=20618;AC=1;AF=4.850130953535746e-05;N_HET=1;N_HOMALT=0;N_HOMREF=10308;FREQ_HET=9.700261907071492e-05;FREQ_HOMALT=0.0;FREQ_HOMREF=0.9999029973809292;N_BI_GENOS=10309;North_Han_AN=2272;North_Han_AC=1;North_Han
chr2	161830219	.	T	<INS:ME:LINE1>	.	PASS	SVTYPE=INS:ME:LINE1;END=161830236;SVLEN=5999;AN=20618;AC=2;AF=9.700261907071492e-05;N_HET=2;N_HOMALT=0;N_HOMREF=10307;FREQ_HET=0.00019400523814142983;FREQ_HOMALT=0.0;FREQ_HOMREF=0.9998059947618586;N_BI_GENOS=10309;Northwest_Han_AN=1296;Northwest_Han_AC=1;
chr2	161859276	.	T	<INS:ME:LINE1>	.	PASS	SVTYPE=INS:ME:LINE1;END=161859300;SVLEN=390;AN=20618;AC=1;AF=4.850130953535746e-05;N_HET=1;N_HOMALT=0;N_HOMREF=10308;FREQ_HET=9.700261907071492e-05;FREQ_HOMALT=0.0;FREQ_HOMREF=0.9999029973809292;N_BI_GENOS=10309;East_Han_AN=6554;East_Han_AC=1;East_Han_AF=
chr2	161935960	.	T	<INS:ME:LINE1>	.	PASS	SVTYPE=INS:ME:LINE1;END=161935976;SVLEN=1535;AN=20618;AC=1;AF=4.850130953535746e-05;N_HET=1;N_HOMALT=0;N_HOMREF=10308;FREQ_HET=9.700261907071492e-05;FREQ_HOMALT=0.0;FREQ_HOMREF=0.9999029973809292;N_BI_GENOS=10309;North_Han_AN=2272;North_Han_AC=1;North_Han
chr2	162214832	.	A	<INS:ME:LINE1>	.	PASS	SVTYPE=INS:ME:LINE1;END=162214848;SVLEN=1382;AN=20618;AC=1;AF=4.850130953535746e-05;N_HET=1;N_HOMALT=0;N_HOMREF=10308;FREQ_HET=9.700261907071492e-05;FREQ_HOMALT=0.0;FREQ_HOMREF=0.9999029973809292;N_BI_GENOS=10309;Central_Han_AN=2128;Central_Han_AC=1;Centr
chr2	162217308	.	T	<INS:ME:LINE1>	.	PASS	SVTYPE=INS:ME:LINE1;END=162217312;SVLEN=5999;AN=20618;AC=1;AF=4.850130953535746e-05;N_HET=1;N_HOMALT=0;N_HOMREF=10308;FREQ_HET=9.700261907071492e-05;FREQ_HOMALT=0.0;FREQ_HOMREF=0.9999029973809292;N_BI_GENOS=10309;East_Han_AN=6554;East_Han_AC=1;East_Han_AF
chr2	162642857	.	A	<INS:ME:LINE1>	.	PASS	SVTYPE=INS:ME:LINE1;END=162642873;SVLEN=1105;AN=20618;AC=25;AF=0.0012125327383839363;N_HET=23;N_HOMALT=1;N_HOMREF=10285;FREQ_HET=0.002231060238626443;FREQ_HOMALT=9.700261907071492e-05;FREQ_HOMREF=0.9976719371423028;N_BI_GENOS=10309;South_Han_AN=1496;South
chr2	163092670	.	G	<INS:ME:LINE1>	.	PASS	SVTYPE=INS:ME:LINE1;END=163092684;SVLEN=1085;AN=20618;AC=1;AF=4.850130953535746e-05;N_HET=1;N_HOMALT=0;N_HOMREF=10308;FREQ_HET=9.700261907071492e-05;FREQ_HOMALT=0.0;FREQ_HOMREF=0.9999029973809292;N_BI_GENOS=10309;Southeast_Han_AN=1908;Southeast_Han_AC=1;S
chr2	163326258	.	C	<INS:ME:LINE1>	.	PASS	SVTYPE=INS:ME:LINE1;END=163326274;SVLEN=1448;AN=20618;AC=66;AF=0.0032010864293333592;N_HET=62;N_HOMALT=2;N_HOMREF=10245;FREQ_HET=0.006014162382384325;FREQ_HOMALT=0.00019400523814142983;FREQ_HOMREF=0.9937918323794742;N_BI_GENOS=10309;South_Han_AN=1496;South
chr2	163431990	.	T	<INS:ME:LINE1>	.	PASS	SVTYPE=INS:ME:LINE1;END=163432003;SVLEN=5999;AN=20618;AC=1378;AF=0.06683480453972257;N_HET=1202;N_HOMALT=88;N_HOMREF=9019;FREQ_HET=0.11659714812299932;FREQ_HOMALT=0.008536230478222911;FREQ_HOMREF=0.8748666213987778;N_BI_GENOS=10309;South_Han_AN=1496;South
chr2	163763416	.	A	<INS:ME:LINE1>	.	PASS	SVTYPE=INS:ME:LINE1;END=163763425;SVLEN=760;AN=20618;AC=1;AF=4.850130953535746e-05;N_HET=1;N_HOMALT=0;N_HOMREF=10308;FREQ_HET=9.700261907071492e-05;FREQ_HOMALT=0.0;FREQ_HOMREF=0.9999029973809292;N_BI_GENOS=10309;North_Han_AN=2272;North_Han_AC=1;North_Han_
chr2	163811560	.	A	<INS:ME:LINE1>	.	PASS	SVTYPE=INS:ME:LINE1;END=163811574;SVLEN=160;AN=20618;AC=1;AF=4.850130953535746e-05;N_HET=1;N_HOMALT=0;N_HOMREF=10308;FREQ_HET=9.700261907071492e-05;FREQ_HOMALT=0.0;FREQ_HOMREF=0.9999029973809292;N_BI_GENOS=10309;East_Han_AN=6554;East_Han_AC=1;East_Han_AF=
chr2	164195445	.	A	<INS:ME:LINE1>	.	PASS	SVTYPE=INS:ME:LINE1;END=164195450;SVLEN=356;AN=20618;AC=1;AF=4.850130953535746e-05;N_HET=1;N_HOMALT=0;N_HOMREF=10308;FREQ_HET=9.700261907071492e-05;FREQ_HOMALT=0.0;FREQ_HOMREF=0.9999029973809292;N_BI_GENOS=10309;North_Han_AN=2272;North_Han_AC=1;North_Han_
chr2	164276267	.	C	<INS:ME:LINE1>	.	PASS	SVTYPE=INS:ME:LINE1;END=164276274;SVLEN=1045;AN=20618;AC=1;AF=4.850130953535746e-05;N_HET=1;N_HOMALT=0;N_HOMREF=10308;FREQ_HET=9.700261907071492e-05;FREQ_HOMALT=0.0;FREQ_HOMREF=0.9999029973809292;N_BI_GENOS=10309;Mongolian_AN=454;Mongolian_AC=1;Mongolian_
chr2	164377516	.	T	<INS:ME:LINE1>	.	PASS	SVTYPE=INS:ME:LINE1;END=164377532;SVLEN=756;AN=20618;AC=3;AF=0.00014550392860607236;N_HET=3;N_HOMALT=0;N_HOMREF=10306;FREQ_HET=0.0002910078572121447;FREQ_HOMALT=0.0;FREQ_HOMREF=0.9997089921427879;N_BI_GENOS=10309;North_Han_AN=2272;North_Han_AC=1;North_Han
chr2	164475201	.	A	<INS:ME:LINE1>	.	PASS	SVTYPE=INS:ME:LINE1;END=164475206;SVLEN=364;AN=20618;AC=1;AF=4.850130953535746e-05;N_HET=1;N_HOMALT=0;N_HOMREF=10308;FREQ_HET=9.700261907071492e-05;FREQ_HOMALT=0.0;FREQ_HOMREF=0.9999029973809292;N_BI_GENOS=10309;South_Han_AN=1496;South_Han_AC=1;South_Han_
chr2	165463999	.	T	<INS:ME:LINE1>	.	PASS	SVTYPE=INS:ME:LINE1;END=165464015;SVLEN=5979;AN=20618;AC=1;AF=4.850130953535746e-05;N_HET=1;N_HOMALT=0;N_HOMREF=10308;FREQ_HET=9.700261907071492e-05;FREQ_HOMALT=0.0;FREQ_HOMREF=0.9999029973809292;N_BI_GENOS=10309;East_Han_AN=6554;East_Han_AC=1;East_Han_AF
chr2	165559220	.	G	<INS:ME:LINE1>	.	PASS	SVTYPE=INS:ME:LINE1;END=165559232;SVLEN=6018;AN=20618;AC=1;AF=4.850130953535746e-05;N_HET=1;N_HOMALT=0;N_HOMREF=10308;FREQ_HET=9.700261907071492e-05;FREQ_HOMALT=0.0;FREQ_HOMREF=0.9999029973809292;N_BI_GENOS=10309;North_Han_AN=2272;North_Han_AC=1;North_Han
chr2	166069239	.	T	<INS:ME:LINE1>	.	PASS	SVTYPE=INS:ME:LINE1;END=166069253;SVLEN=5989;AN=20618;AC=1;AF=4.850130953535746e-05;N_HET=1;N_HOMALT=0;N_HOMREF=10308;FREQ_HET=9.700261907071492e-05;FREQ_HOMALT=0.0;FREQ_HOMREF=0.9999029973809292;N_BI_GENOS=10309;East_Han_AN=6554;East_Han_AC=1;East_Han_AF
chr2	166334482	.	A	<INS:ME:LINE1>	.	PASS	SVTYPE=INS:ME:LINE1;END=166334497;SVLEN=824;AN=20618;AC=1;AF=4.850130953535746e-05;N_HET=1;N_HOMALT=0;N_HOMREF=10308;FREQ_HET=9.700261907071492e-05;FREQ_HOMALT=0.0;FREQ_HOMREF=0.9999029973809292;N_BI_GENOS=10309;North_Han_AN=2272;North_Han_AC=1;North_Han_
chr2	166336684	.	T	<INS:ME:LINE1>	.	PASS	SVTYPE=INS:ME:LINE1;END=166336701;SVLEN=1032;AN=20618;AC=9;AF=0.0004365117858182171;N_HET=9;N_HOMALT=0;N_HOMREF=10300;FREQ_HET=0.0008730235716364342;FREQ_HOMALT=0.0;FREQ_HOMREF=0.9991269764283636;N_BI_GENOS=10309;North_Han_AN=2272;North_Han_AC=2;North_Han
chr2	166403044	.	A	<INS:ME:LINE1>	.	PASS	SVTYPE=INS:ME:LINE1;END=166403061;SVLEN=6000;AN=20618;AC=5;AF=0.00024250654767678728;N_HET=3;N_HOMALT=1;N_HOMREF=10305;FREQ_HET=0.0002910078572121447;FREQ_HOMALT=9.700261907071492e-05;FREQ_HOMREF=0.9996119895237171;N_BI_GENOS=10309;North_Han_AN=2272;North
chr2	166562064	.	C	<INS:ME:LINE1>	.	PASS	SVTYPE=INS:ME:LINE1;END=166562081;SVLEN=1845;AN=20618;AC=3;AF=0.00014550392860607236;N_HET=3;N_HOMALT=0;N_HOMREF=10306;FREQ_HET=0.0002910078572121447;FREQ_HOMALT=0.0;FREQ_HOMREF=0.9997089921427879;N_BI_GENOS=10309;East_Han_AN=6554;East_Han_AC=3;East_Han_A
chr2	166930622	.	T	<INS:ME:LINE1>	.	PASS	SVTYPE=INS:ME:LINE1;END=166930632;SVLEN=6007;AN=20618;AC=1;AF=4.850130953535746e-05;N_HET=1;N_HOMALT=0;N_HOMREF=10308;FREQ_HET=9.700261907071492e-05;FREQ_HOMALT=0.0;FREQ_HOMREF=0.9999029973809292;N_BI_GENOS=10309;North_Han_AN=2272;North_Han_AC=1;North_Han
chr2	167045542	.	A	<INS:ME:LINE1>	.	PASS	SVTYPE=INS:ME:LINE1;END=167045555;SVLEN=5989;AN=20618;AC=2;AF=9.700261907071492e-05;N_HET=2;N_HOMALT=0;N_HOMREF=10307;FREQ_HET=0.00019400523814142983;FREQ_HOMALT=0.0;FREQ_HOMREF=0.9998059947618586;N_BI_GENOS=10309;East_Han_AN=6554;East_Han_AC=2;East_Han_A
chr2	167109688	.	G	<INS:ME:LINE1>	.	PASS	SVTYPE=INS:ME:LINE1;END=167109688;SVLEN=762;AN=20618;AC=1;AF=4.850130953535746e-05;N_HET=1;N_HOMALT=0;N_HOMREF=10308;FREQ_HET=9.700261907071492e-05;FREQ_HOMALT=0.0;FREQ_HOMREF=0.9999029973809292;N_BI_GENOS=10309;East_Han_AN=6554;East_Han_AC=1;East_Han_AF=
chr2	167110375	.	C	<INS:ME:LINE1>	.	PASS	SVTYPE=INS:ME:LINE1;END=167110385;SVLEN=1238;AN=20618;AC=1;AF=4.850130953535746e-05;N_HET=1;N_HOMALT=0;N_HOMREF=10308;FREQ_HET=9.700261907071492e-05;FREQ_HOMALT=0.0;FREQ_HOMREF=0.9999029973809292;N_BI_GENOS=10309;East_Han_AN=6554;East_Han_AC=1;East_Han_AF
chr2	167125232	.	A	<INS:ME:LINE1>	.	PASS	SVTYPE=INS:ME:LINE1;END=167125247;SVLEN=190;AN=20618;AC=21;AF=0.0010185275002425065;N_HET=21;N_HOMALT=0;N_HOMREF=10288;FREQ_HET=0.002037055000485013;FREQ_HOMALT=0.0;FREQ_HOMREF=0.997962944999515;N_BI_GENOS=10309;South_Han_AN=1496;South_Han_AC=2;South_Han_
chr2	167179190	.	T	<INS:ME:LINE1>	.	PASS	SVTYPE=INS:ME:LINE1;END=167179208;SVLEN=708;AN=20618;AC=1;AF=4.850130953535746e-05;N_HET=1;N_HOMALT=0;N_HOMREF=10308;FREQ_HET=9.700261907071492e-05;FREQ_HOMALT=0.0;FREQ_HOMREF=0.9999029973809292;N_BI_GENOS=10309;Mongolian_AN=454;Mongolian_AC=1;Mongolian_A
chr2	167659411	.	C	<INS:ME:LINE1>	.	PASS	SVTYPE=INS:ME:LINE1;END=167659426;SVLEN=6000;AN=20618;AC=1;AF=4.850130953535746e-05;N_HET=1;N_HOMALT=0;N_HOMREF=10308;FREQ_HET=9.700261907071492e-05;FREQ_HOMALT=0.0;FREQ_HOMREF=0.9999029973809292;N_BI_GENOS=10309;Southeast_Han_AN=1908;Southeast_Han_AC=1;S
chr2	167796658	.	A	<INS:ME:LINE1>	.	PASS	SVTYPE=INS:ME:LINE1;END=167796670;SVLEN=6006;AN=20618;AC=1;AF=4.850130953535746e-05;N_HET=1;N_HOMALT=0;N_HOMREF=10308;FREQ_HET=9.700261907071492e-05;FREQ_HOMALT=0.0;FREQ_HOMREF=0.9999029973809292;N_BI_GENOS=10309;East_Han_AN=6554;East_Han_AC=1;East_Han_AF
chr2	167879010	.	T	<INS:ME:LINE1>	.	PASS	SVTYPE=INS:ME:LINE1;END=167879017;SVLEN=511;AN=20618;AC=27;AF=0.0013095353574546513;N_HET=27;N_HOMALT=0;N_HOMREF=10282;FREQ_HET=0.0026190707149093026;FREQ_HOMALT=0.0;FREQ_HOMREF=0.9973809292850907;N_BI_GENOS=10309;South_Han_AN=1496;South_Han_AC=1;South_Ha
chr2	168404374	.	A	<INS:ME:LINE1>	.	PASS	SVTYPE=INS:ME:LINE1;END=168404390;SVLEN=401;AN=20618;AC=1;AF=4.850130953535746e-05;N_HET=1;N_HOMALT=0;N_HOMREF=10308;FREQ_HET=9.700261907071492e-05;FREQ_HOMALT=0.0;FREQ_HOMREF=0.9999029973809292;N_BI_GENOS=10309;East_Han_AN=6554;East_Han_AC=1;East_Han_AF=
chr2	168517589	.	T	<INS:ME:LINE1>	.	PASS	SVTYPE=INS:ME:LINE1;END=168517605;SVLEN=1073;AN=20618;AC=1;AF=4.850130953535746e-05;N_HET=1;N_HOMALT=0;N_HOMREF=10308;FREQ_HET=9.700261907071492e-05;FREQ_HOMALT=0.0;FREQ_HOMREF=0.9999029973809292;N_BI_GENOS=10309;East_Han_AN=6554;East_Han_AC=1;East_Han_AF
chr2	168576261	.	T	<INS:ME:LINE1>	.	PASS	SVTYPE=INS:ME:LINE1;END=168576277;SVLEN=2286;AN=20618;AC=1;AF=4.850130953535746e-05;N_HET=1;N_HOMALT=0;N_HOMREF=10308;FREQ_HET=9.700261907071492e-05;FREQ_HOMALT=0.0;FREQ_HOMREF=0.9999029973809292;N_BI_GENOS=10309;East_Han_AN=6554;East_Han_AC=1;East_Han_AF
chr2	168604868	.	C	<INS:ME:LINE1>	.	PASS	SVTYPE=INS:ME:LINE1;END=168604868;SVLEN=5949;AN=20618;AC=1;AF=4.850130953535746e-05;N_HET=1;N_HOMALT=0;N_HOMREF=10308;FREQ_HET=9.700261907071492e-05;FREQ_HOMALT=0.0;FREQ_HOMREF=0.9999029973809292;N_BI_GENOS=10309;North_Han_AN=2272;North_Han_AC=1;North_Han
chr2	168612766	.	A	<INS:ME:LINE1>	.	PASS	SVTYPE=INS:ME:LINE1;END=168612779;SVLEN=949;AN=20618;AC=1;AF=4.850130953535746e-05;N_HET=1;N_HOMALT=0;N_HOMREF=10308;FREQ_HET=9.700261907071492e-05;FREQ_HOMALT=0.0;FREQ_HOMREF=0.9999029973809292;N_BI_GENOS=10309;South_Han_AN=1496;South_Han_AC=1;South_Han_
chr2	168637991	.	T	<INS:ME:LINE1>	.	PASS	SVTYPE=INS:ME:LINE1;END=168637994;SVLEN=4891;AN=20618;AC=1;AF=4.850130953535746e-05;N_HET=1;N_HOMALT=0;N_HOMREF=10308;FREQ_HET=9.700261907071492e-05;FREQ_HOMALT=0.0;FREQ_HOMREF=0.9999029973809292;N_BI_GENOS=10309;Tibetan_AN=414;Tibetan_AC=1;Tibetan_AF=0.0
chr2	168759053	.	A	<INS:ME:LINE1>	.	PASS	SVTYPE=INS:ME:LINE1;END=168759065;SVLEN=6080;AN=20618;AC=1;AF=4.850130953535746e-05;N_HET=1;N_HOMALT=0;N_HOMREF=10308;FREQ_HET=9.700261907071492e-05;FREQ_HOMALT=0.0;FREQ_HOMREF=0.9999029973809292;N_BI_GENOS=10309;South_Han_AN=1496;South_Han_AC=1;South_Han
chr2	168825898	.	G	<INS:ME:LINE1>	.	PASS	SVTYPE=INS:ME:LINE1;END=168825913;SVLEN=322;AN=20618;AC=2;AF=9.700261907071492e-05;N_HET=2;N_HOMALT=0;N_HOMREF=10307;FREQ_HET=0.00019400523814142983;FREQ_HOMALT=0.0;FREQ_HOMREF=0.9998059947618586;N_BI_GENOS=10309;North_Han_AN=2272;North_Han_AC=1;North_Han
chr2	168951651	.	G	<INS:ME:LINE1>	.	PASS	SVTYPE=INS:ME:LINE1;END=168951651;SVLEN=213;AN=20618;AC=1;AF=4.850130953535746e-05;N_HET=1;N_HOMALT=0;N_HOMREF=10308;FREQ_HET=9.700261907071492e-05;FREQ_HOMALT=0.0;FREQ_HOMREF=0.9999029973809292;N_BI_GENOS=10309;Central_Han_AN=2128;Central_Han_AC=1;Centra
chr2	169047954	.	A	<INS:ME:LINE1>	.	PASS	SVTYPE=INS:ME:LINE1;END=169047970;SVLEN=1122;AN=20618;AC=1;AF=4.850130953535746e-05;N_HET=1;N_HOMALT=0;N_HOMREF=10308;FREQ_HET=9.700261907071492e-05;FREQ_HOMALT=0.0;FREQ_HOMREF=0.9999029973809292;N_BI_GENOS=10309;Southeast_Han_AN=1908;Southeast_Han_AC=1;S
chr2	169422830	.	A	<INS:ME:LINE1>	.	PASS	SVTYPE=INS:ME:LINE1;END=169422846;SVLEN=6000;AN=20618;AC=1;AF=4.850130953535746e-05;N_HET=1;N_HOMALT=0;N_HOMREF=10308;FREQ_HET=9.700261907071492e-05;FREQ_HOMALT=0.0;FREQ_HOMREF=0.9999029973809292;N_BI_GENOS=10309;Lingnan_Han_AN=1888;Lingnan_Han_AC=1;Lingn
chr2	169540497	.	A	<INS:ME:LINE1>	.	PASS	SVTYPE=INS:ME:LINE1;END=169540509;SVLEN=1132;AN=20618;AC=1;AF=4.850130953535746e-05;N_HET=1;N_HOMALT=0;N_HOMREF=10308;FREQ_HET=9.700261907071492e-05;FREQ_HOMALT=0.0;FREQ_HOMREF=0.9999029973809292;N_BI_GENOS=10309;East_Han_AN=6554;East_Han_AC=1;East_Han_AF
chr2	169928982	.	T	<INS:ME:LINE1>	.	PASS	SVTYPE=INS:ME:LINE1;END=169928995;SVLEN=6008;AN=20618;AC=5;AF=0.00024250654767678728;N_HET=5;N_HOMALT=0;N_HOMREF=10304;FREQ_HET=0.00048501309535357455;FREQ_HOMALT=0.0;FREQ_HOMREF=0.9995149869046465;N_BI_GENOS=10309;Northwest_Han_AN=1296;Northwest_Han_AC=2
chr2	170321294	.	A	<INS:ME:LINE1>	.	PASS	SVTYPE=INS:ME:LINE1;END=170321308;SVLEN=6009;AN=20618;AC=1;AF=4.850130953535746e-05;N_HET=1;N_HOMALT=0;N_HOMREF=10308;FREQ_HET=9.700261907071492e-05;FREQ_HOMALT=0.0;FREQ_HOMREF=0.9999029973809292;N_BI_GENOS=10309;East_Han_AN=6554;East_Han_AC=1;East_Han_AF
chr2	170350507	.	A	<INS:ME:LINE1>	.	PASS	SVTYPE=INS:ME:LINE1;END=170350522;SVLEN=1386;AN=20618;AC=1;AF=4.850130953535746e-05;N_HET=1;N_HOMALT=0;N_HOMREF=10308;FREQ_HET=9.700261907071492e-05;FREQ_HOMALT=0.0;FREQ_HOMREF=0.9999029973809292;N_BI_GENOS=10309;Southeast_Han_AN=1908;Southeast_Han_AC=1;S
chr2	170380696	.	T	<INS:ME:LINE1>	.	PASS	SVTYPE=INS:ME:LINE1;END=170380697;SVLEN=640;AN=20618;AC=1;AF=4.850130953535746e-05;N_HET=1;N_HOMALT=0;N_HOMREF=10308;FREQ_HET=9.700261907071492e-05;FREQ_HOMALT=0.0;FREQ_HOMREF=0.9999029973809292;N_BI_GENOS=10309;Lingnan_Han_AN=1888;Lingnan_Han_AC=1;Lingna
chr2	170781223	.	A	<INS:ME:LINE1>	.	PASS	SVTYPE=INS:ME:LINE1;END=170781223;SVLEN=2451;AN=20618;AC=1;AF=4.850130953535746e-05;N_HET=1;N_HOMALT=0;N_HOMREF=10308;FREQ_HET=9.700261907071492e-05;FREQ_HOMALT=0.0;FREQ_HOMREF=0.9999029973809292;N_BI_GENOS=10309;Lingnan_Han_AN=1888;Lingnan_Han_AC=1;Lingn
chr2	170892125	.	A	<INS:ME:LINE1>	.	PASS	SVTYPE=INS:ME:LINE1;END=170892141;SVLEN=881;AN=20618;AC=1;AF=4.850130953535746e-05;N_HET=1;N_HOMALT=0;N_HOMREF=10308;FREQ_HET=9.700261907071492e-05;FREQ_HOMALT=0.0;FREQ_HOMREF=0.9999029973809292;N_BI_GENOS=10309;Lingnan_Han_AN=1888;Lingnan_Han_AC=1;Lingna
chr2	170959118	.	C	<INS:ME:LINE1>	.	PASS	SVTYPE=INS:ME:LINE1;END=170959133;SVLEN=6009;AN=20618;AC=1;AF=4.850130953535746e-05;N_HET=1;N_HOMALT=0;N_HOMREF=10308;FREQ_HET=9.700261907071492e-05;FREQ_HOMALT=0.0;FREQ_HOMREF=0.9999029973809292;N_BI_GENOS=10309;Lingnan_Han_AN=1888;Lingnan_Han_AC=1;Lingn
chr2	171193712	.	T	<INS:ME:LINE1>	.	PASS	SVTYPE=INS:ME:LINE1;END=171193732;SVLEN=6012;AN=20618;AC=2;AF=9.700261907071492e-05;N_HET=0;N_HOMALT=1;N_HOMREF=10308;FREQ_HET=0.0;FREQ_HOMALT=9.700261907071492e-05;FREQ_HOMREF=0.9999029973809292;N_BI_GENOS=10309;South_Han_AN=1496;South_Han_AC=2;South_Han
chr2	171906328	.	A	<INS:ME:LINE1>	.	PASS	SVTYPE=INS:ME:LINE1;END=171906343;SVLEN=658;AN=20618;AC=2;AF=9.700261907071492e-05;N_HET=2;N_HOMALT=0;N_HOMREF=10307;FREQ_HET=0.00019400523814142983;FREQ_HOMALT=0.0;FREQ_HOMREF=0.9998059947618586;N_BI_GENOS=10309;East_Han_AN=6554;East_Han_AC=2;East_Han_AF
chr2	172388932	.	G	<INS:ME:LINE1>	.	PASS	SVTYPE=INS:ME:LINE1;END=172388942;SVLEN=6001;AN=20618;AC=2;AF=9.700261907071492e-05;N_HET=2;N_HOMALT=0;N_HOMREF=10307;FREQ_HET=0.00019400523814142983;FREQ_HOMALT=0.0;FREQ_HOMREF=0.9998059947618586;N_BI_GENOS=10309;Lingnan_Han_AN=1888;Lingnan_Han_AC=1;Ling
chr2	172989367	.	C	<INS:ME:LINE1>	.	PASS	SVTYPE=INS:ME:LINE1;END=172989382;SVLEN=733;AN=20618;AC=3;AF=0.00014550392860607236;N_HET=3;N_HOMALT=0;N_HOMREF=10306;FREQ_HET=0.0002910078572121447;FREQ_HOMALT=0.0;FREQ_HOMREF=0.9997089921427879;N_BI_GENOS=10309;Northwest_Han_AN=1296;Northwest_Han_AC=1;N
chr2	173727829	.	A	<INS:ME:LINE1>	.	PASS	SVTYPE=INS:ME:LINE1;END=173727845;SVLEN=704;AN=20618;AC=1;AF=4.850130953535746e-05;N_HET=1;N_HOMALT=0;N_HOMREF=10308;FREQ_HET=9.700261907071492e-05;FREQ_HOMALT=0.0;FREQ_HOMREF=0.9999029973809292;N_BI_GENOS=10309;South_Han_AN=1496;South_Han_AC=1;South_Han
chr2	173922200	.	A	<INS:ME:LINE1>	.	PASS	SVTYPE=INS:ME:LINE1;END=173922212;SVLEN=5998;AN=20618;AC=3;AF=0.00014550392860607236;N_HET=3;N_HOMALT=0;N_HOMREF=10306;FREQ_HET=0.0002910078572121447;FREQ_HOMALT=0.0;FREQ_HOMREF=0.9997089921427879;N_BI_GENOS=10309;Central_Han_AN=2128;Central_Han_AC=3;Cent
chr2	173976484	.	A	<INS:ME:LINE1>	.	PASS	SVTYPE=INS:ME:LINE1;END=173976504;SVLEN=6002;AN=20618;AC=1;AF=4.850130953535746e-05;N_HET=1;N_HOMALT=0;N_HOMREF=10308;FREQ_HET=9.700261907071492e-05;FREQ_HOMALT=0.0;FREQ_HOMREF=0.9999029973809292;N_BI_GENOS=10309;South_Han_AN=1496;South_Han_AC=1;South_Han
chr2	174114639	.	A	<INS:ME:LINE1>	.	PASS	SVTYPE=INS:ME:LINE1;END=174114653;SVLEN=387;AN=20618;AC=7;AF=0.0003395091667475022;N_HET=5;N_HOMALT=1;N_HOMREF=10303;FREQ_HET=0.00048501309535357455;FREQ_HOMALT=9.700261907071492e-05;FREQ_HOMREF=0.9994179842855757;N_BI_GENOS=10309;Lingnan_Han_AN=1888;Ling
chr2	174176645	.	G	<INS:ME:LINE1>	.	PASS	SVTYPE=INS:ME:LINE1;END=174176661;SVLEN=5988;AN=20618;AC=4;AF=0.00019400523814142983;N_HET=4;N_HOMALT=0;N_HOMREF=10305;FREQ_HET=0.0003880104762828966;FREQ_HOMALT=0.0;FREQ_HOMREF=0.9996119895237171;N_BI_GENOS=10309;South_Han_AN=1496;South_Han_AC=1;South_H
chr2	174302477	.	C	<INS:ME:LINE1>	.	PASS	SVTYPE=INS:ME:LINE1;END=174302492;SVLEN=1575;AN=20618;AC=4;AF=0.00019400523814142983;N_HET=4;N_HOMALT=0;N_HOMREF=10305;FREQ_HET=0.0003880104762828966;FREQ_HOMALT=0.0;FREQ_HOMREF=0.9996119895237171;N_BI_GENOS=10309;South_Han_AN=1496;South_Han_AC=2;South_H

chr2	174697711	.	C	<INS:ME:LINE1>	.	PASS	SVTYPE=INS:ME:LINE1;END=174697726;SVLEN=6004;AN=20618;AC=1;AF=4.850130953535746e-05;N_HET=1;N_HOMALT=0;N_HOMREF=10308;FREQ_HET=9.700261907071492e-05;FREQ_HOMALT=0.0;FREQ_HOMREF=0.9999029973809292;N_BI_GENOS=10309;Lingnan_Han_AN=1888;Lingnan_Han_AC=1;Lingn
chr2	174865186	.	T	<INS:ME:LINE1>	.	PASS	SVTYPE=INS:ME:LINE1;END=174865201;SVLEN=151;AN=20618;AC=2;AF=9.700261907071492e-05;N_HET=2;N_HOMALT=0;N_HOMREF=10307;FREQ_HET=0.00019400523814142983;FREQ_HOMALT=0.0;FREQ_HOMREF=0.9998059947618586;N_BI_GENOS=10309;South_Han_AN=1496;South_Han_AC=1;South_Han
chr2	174882931	.	T	<INS:ME:LINE1>	.	PASS	SVTYPE=INS:ME:LINE1;END=174882945;SVLEN=5979;AN=20618;AC=21;AF=0.0010185275002425065;N_HET=21;N_HOMALT=0;N_HOMREF=10288;FREQ_HET=0.002037055000485013;FREQ_HOMALT=0.0;FREQ_HOMREF=0.997962944999515;N_BI_GENOS=10309;South_Han_AN=1496;South_Han_AC=3;South_Han
chr2	175155836	.	A	<INS:ME:LINE1>	.	PASS	SVTYPE=INS:ME:LINE1;END=175155850;SVLEN=1792;AN=20618;AC=1;AF=4.850130953535746e-05;N_HET=1;N_HOMALT=0;N_HOMREF=10308;FREQ_HET=9.700261907071492e-05;FREQ_HOMALT=0.0;FREQ_HOMREF=0.9999029973809292;N_BI_GENOS=10309;East_Han_AN=6554;East_Han_AC=1;East_Han_AF
chr2	175354151	.	T	<INS:ME:LINE1>	.	PASS	SVTYPE=INS:ME:LINE1;END=175354169;SVLEN=1200;AN=20618;AC=3;AF=0.00014550392860607236;N_HET=3;N_HOMALT=0;N_HOMREF=10306;FREQ_HET=0.0002910078572121447;FREQ_HOMALT=0.0;FREQ_HOMREF=0.9997089921427879;N_BI_GENOS=10309;Lingnan_Han_AN=1888;Lingnan_Han_AC=1;Ling
chr2	175614426	.	T	<INS:ME:LINE1>	.	PASS	SVTYPE=INS:ME:LINE1;END=175614427;SVLEN=56;AN=20618;AC=1;AF=4.850130953535746e-05;N_HET=1;N_HOMALT=0;N_HOMREF=10308;FREQ_HET=9.700261907071492e-05;FREQ_HOMALT=0.0;FREQ_HOMREF=0.9999029973809292;N_BI_GENOS=10309;East_Han_AN=6554;East_Han_AC=1;East_Han_AF=0
chr2	175631946	.	T	<INS:ME:LINE1>	.	PASS	SVTYPE=INS:ME:LINE1;END=175631960;SVLEN=6079;AN=20618;AC=1;AF=4.850130953535746e-05;N_HET=1;N_HOMALT=0;N_HOMREF=10308;FREQ_HET=9.700261907071492e-05;FREQ_HOMALT=0.0;FREQ_HOMREF=0.9999029973809292;N_BI_GENOS=10309;Central_Han_AN=2128;Central_Han_AC=1;Centr
chr2	175730936	.	T	<INS:ME:LINE1>	.	PASS	SVTYPE=INS:ME:LINE1;END=175730936;SVLEN=15;AN=20618;AC=1;AF=4.850130953535746e-05;N_HET=1;N_HOMALT=0;N_HOMREF=10308;FREQ_HET=9.700261907071492e-05;FREQ_HOMALT=0.0;FREQ_HOMREF=0.9999029973809292;N_BI_GENOS=10309;Southeast_Han_AN=1908;Southeast_Han_AC=1;Sou
chr2	175998557	.	A	<INS:ME:LINE1>	.	PASS	SVTYPE=INS:ME:LINE1;END=175998559;SVLEN=297;AN=20618;AC=1;AF=4.850130953535746e-05;N_HET=1;N_HOMALT=0;N_HOMREF=10308;FREQ_HET=9.700261907071492e-05;FREQ_HOMALT=0.0;FREQ_HOMREF=0.9999029973809292;N_BI_GENOS=10309;East_Han_AN=6554;East_Han_AC=1;East_Han_AF=
chr2	176752872	.	G	<INS:ME:LINE1>	.	PASS	SVTYPE=INS:ME:LINE1;END=176752888;SVLEN=748;AN=20618;AC=1;AF=4.850130953535746e-05;N_HET=1;N_HOMALT=0;N_HOMREF=10308;FREQ_HET=9.700261907071492e-05;FREQ_HOMALT=0.0;FREQ_HOMREF=0.9999029973809292;N_BI_GENOS=10309;Northwest_Han_AN=1296;Northwest_Han_AC=1;No
chr2	176810529	.	A	<INS:ME:LINE1>	.	PASS	SVTYPE=INS:ME:LINE1;END=176810540;SVLEN=872;AN=20618;AC=2;AF=9.700261907071492e-05;N_HET=0;N_HOMALT=1;N_HOMREF=10308;FREQ_HET=0.0;FREQ_HOMALT=9.700261907071492e-05;FREQ_HOMREF=0.9999029973809292;N_BI_GENOS=10309;North_Han_AN=2272;North_Han_AC=2;North_Han_
chr2	176839761	.	A	<INS:ME:LINE1>	.	PASS	SVTYPE=INS:ME:LINE1;END=176839762;SVLEN=720;AN=20618;AC=356;AF=0.017266466194587254;N_HET=346;N_HOMALT=5;N_HOMREF=9958;FREQ_HET=0.03356290619846736;FREQ_HOMALT=0.00048501309535357455;FREQ_HOMREF=0.9659520807061791;N_BI_GENOS=10309;South_Han_AN=1496;South_
chr2	177030021	.	A	<INS:ME:LINE1>	.	PASS	SVTYPE=INS:ME:LINE1;END=177030034;SVLEN=1941;AN=20618;AC=2;AF=9.700261907071492e-05;N_HET=0;N_HOMALT=1;N_HOMREF=10308;FREQ_HET=0.0;FREQ_HOMALT=9.700261907071492e-05;FREQ_HOMREF=0.9999029973809292;N_BI_GENOS=10309;Mongolian_AN=454;Mongolian_AC=2;Mongolian_
chr2	177368293	.	A	<INS:ME:LINE1>	.	PASS	SVTYPE=INS:ME:LINE1;END=177368306;SVLEN=1945;AN=20618;AC=1;AF=4.850130953535746e-05;N_HET=1;N_HOMALT=0;N_HOMREF=10308;FREQ_HET=9.700261907071492e-05;FREQ_HOMALT=0.0;FREQ_HOMREF=0.9999029973809292;N_BI_GENOS=10309;East_Han_AN=6554;East_Han_AC=1;East_Han_AF
chr2	177811517	.	A	<INS:ME:LINE1>	.	PASS	SVTYPE=INS:ME:LINE1;END=177811533;SVLEN=322;AN=20618;AC=5919;AF=0.2870792511397808;N_HET=3671;N_HOMALT=1124;N_HOMREF=5514;FREQ_HET=0.35609661460859443;FREQ_HOMALT=0.10903094383548356;FREQ_HOMREF=0.5348724415559221;N_BI_GENOS=10309;South_Han_AN=1496;South_
chr2	177922401	.	A	<INS:ME:LINE1>	.	PASS	SVTYPE=INS:ME:LINE1;END=177922412;SVLEN=65;AN=20618;AC=324;AF=0.015714424289455815;N_HET=256;N_HOMALT=34;N_HOMREF=10019;FREQ_HET=0.02483267048210302;FREQ_HOMALT=0.0032980890484043067;FREQ_HOMREF=0.9718692404694926;N_BI_GENOS=10309;South_Han_AN=1496;South_
chr2	178162194	.	T	<INS:ME:LINE1>	.	PASS	SVTYPE=INS:ME:LINE1;END=178162210;SVLEN=6006;AN=20618;AC=1;AF=4.850130953535746e-05;N_HET=1;N_HOMALT=0;N_HOMREF=10308;FREQ_HET=9.700261907071492e-05;FREQ_HOMALT=0.0;FREQ_HOMREF=0.9999029973809292;N_BI_GENOS=10309;East_Han_AN=6554;East_Han_AC=1;East_Han_AF
chr2	178399456	.	A	<INS:ME:LINE1>	.	PASS	SVTYPE=INS:ME:LINE1;END=178399465;SVLEN=1924;AN=20618;AC=1;AF=4.850130953535746e-05;N_HET=1;N_HOMALT=0;N_HOMREF=10308;FREQ_HET=9.700261907071492e-05;FREQ_HOMALT=0.0;FREQ_HOMREF=0.9999029973809292;N_BI_GENOS=10309;Southeast_Han_AN=1908;Southeast_Han_AC=1;S
chr2	178627934	.	C	<INS:ME:LINE1>	.	PASS	SVTYPE=INS:ME:LINE1;END=178627947;SVLEN=6143;AN=20618;AC=1;AF=4.850130953535746e-05;N_HET=1;N_HOMALT=0;N_HOMREF=10308;FREQ_HET=9.700261907071492e-05;FREQ_HOMALT=0.0;FREQ_HOMREF=0.9999029973809292;N_BI_GENOS=10309;Central_Han_AN=2128;Central_Han_AC=1;Centr
chr2	178763830	.	A	<INS:ME:LINE1>	.	PASS	SVTYPE=INS:ME:LINE1;END=178763830;SVLEN=148;AN=20618;AC=1;AF=4.850130953535746e-05;N_HET=1;N_HOMALT=0;N_HOMREF=10308;FREQ_HET=9.700261907071492e-05;FREQ_HOMALT=0.0;FREQ_HOMREF=0.9999029973809292;N_BI_GENOS=10309;North_Han_AN=2272;North_Han_AC=1;North_Han_
chr2	178895761	.	T	<INS:ME:LINE1>	.	PASS	SVTYPE=INS:ME:LINE1;END=178895776;SVLEN=6157;AN=20618;AC=1;AF=4.850130953535746e-05;N_HET=1;N_HOMALT=0;N_HOMREF=10308;FREQ_HET=9.700261907071492e-05;FREQ_HOMALT=0.0;FREQ_HOMREF=0.9999029973809292;N_BI_GENOS=10309;East_Han_AN=6554;East_Han_AC=1;East_Han_AF
chr2	179018734	.	G	<INS:ME:LINE1>	.	PASS	SVTYPE=INS:ME:LINE1;END=179018749;SVLEN=836;AN=20618;AC=1;AF=4.850130953535746e-05;N_HET=1;N_HOMALT=0;N_HOMREF=10308;FREQ_HET=9.700261907071492e-05;FREQ_HOMALT=0.0;FREQ_HOMREF=0.9999029973809292;N_BI_GENOS=10309;East_Han_AN=6554;East_Han_AC=1;East_Han_AF=
chr2	179150899	.	C	<INS:ME:LINE1>	.	PASS	SVTYPE=INS:ME:LINE1;END=179150917;SVLEN=6008;AN=20618;AC=3;AF=0.00014550392860607236;N_HET=3;N_HOMALT=0;N_HOMREF=10306;FREQ_HET=0.0002910078572121447;FREQ_HOMALT=0.0;FREQ_HOMREF=0.9997089921427879;N_BI_GENOS=10309;Northwest_Han_AN=1296;Northwest_Han_AC=1;
chr2	179443713	.	T	<INS:ME:LINE1>	.	PASS	SVTYPE=INS:ME:LINE1;END=179443727;SVLEN=194;AN=20618;AC=1;AF=4.850130953535746e-05;N_HET=1;N_HOMALT=0;N_HOMREF=10308;FREQ_HET=9.700261907071492e-05;FREQ_HOMALT=0.0;FREQ_HOMREF=0.9999029973809292;N_BI_GENOS=10309;Central_Han_AN=2128;Central_Han_AC=1;Centra
chr2	179457330	.	C	<INS:ME:LINE1>	.	PASS	SVTYPE=INS:ME:LINE1;END=179457346;SVLEN=6002;AN=20618;AC=10;AF=0.00048501309535357455;N_HET=10;N_HOMALT=0;N_HOMREF=10299;FREQ_HET=0.0009700261907071491;FREQ_HOMALT=0.0;FREQ_HOMREF=0.9990299738092928;N_BI_GENOS=10309;North_Han_AN=2272;North_Han_AC=1;North_
chr2	179530559	.	G	<INS:ME:LINE1>	.	PASS	SVTYPE=INS:ME:LINE1;END=179530572;SVLEN=6033;AN=20618;AC=2;AF=9.700261907071492e-05;N_HET=0;N_HOMALT=1;N_HOMREF=10308;FREQ_HET=0.0;FREQ_HOMALT=9.700261907071492e-05;FREQ_HOMREF=0.9999029973809292;N_BI_GENOS=10309;North_Han_AN=2272;North_Han_AC=2;North_Han
chr2	179666248	.	A	<INS:ME:LINE1>	.	PASS	SVTYPE=INS:ME:LINE1;END=179666260;SVLEN=5994;AN=20618;AC=1;AF=4.850130953535746e-05;N_HET=1;N_HOMALT=0;N_HOMREF=10308;FREQ_HET=9.700261907071492e-05;FREQ_HOMALT=0.0;FREQ_HOMREF=0.9999029973809292;N_BI_GENOS=10309;East_Han_AN=6554;East_Han_AC=1;East_Han_AF
chr2	179866783	.	A	<INS:ME:LINE1>	.	PASS	SVTYPE=INS:ME:LINE1;END=179866783;SVLEN=0;AN=20618;AC=1;AF=4.850130953535746e-05;N_HET=1;N_HOMALT=0;N_HOMREF=10308;FREQ_HET=9.700261907071492e-05;FREQ_HOMALT=0.0;FREQ_HOMREF=0.9999029973809292;N_BI_GENOS=10309;Southeast_Han_AN=1908;Southeast_Han_AC=1;Sout
chr2	180135812	.	A	<INS:ME:LINE1>	.	PASS	SVTYPE=INS:ME:LINE1;END=180135828;SVLEN=1264;AN=20618;AC=1981;AF=0.09608109418954312;N_HET=1549;N_HOMALT=216;N_HOMREF=8544;FREQ_HET=0.1502570569405374;FREQ_HOMALT=0.02095256571927442;FREQ_HOMREF=0.8287903773401882;N_BI_GENOS=10309;South_Han_AN=1496;South_
chr2	180393638	.	T	<INS:ME:LINE1>	.	PASS	SVTYPE=INS:ME:LINE1;END=180393638;SVLEN=691;AN=20618;AC=1;AF=4.850130953535746e-05;N_HET=1;N_HOMALT=0;N_HOMREF=10308;FREQ_HET=9.700261907071492e-05;FREQ_HOMALT=0.0;FREQ_HOMREF=0.9999029973809292;N_BI_GENOS=10309;East_Han_AN=6554;East_Han_AC=1;East_Han_AF=
chr2	180393699	.	C	<INS:ME:LINE1>	.	PASS	SVTYPE=INS:ME:LINE1;END=180393713;SVLEN=188;AN=20618;AC=1;AF=4.850130953535746e-05;N_HET=1;N_HOMALT=0;N_HOMREF=10308;FREQ_HET=9.700261907071492e-05;FREQ_HOMALT=0.0;FREQ_HOMREF=0.9999029973809292;N_BI_GENOS=10309;East_Han_AN=6554;East_Han_AC=1;East_Han_AF=
chr2	180461551	.	G	<INS:ME:LINE1>	.	PASS	SVTYPE=INS:ME:LINE1;END=180461567;SVLEN=6050;AN=20618;AC=1;AF=4.850130953535746e-05;N_HET=1;N_HOMALT=0;N_HOMREF=10308;FREQ_HET=9.700261907071492e-05;FREQ_HOMALT=0.0;FREQ_HOMREF=0.9999029973809292;N_BI_GENOS=10309;North_Han_AN=2272;North_Han_AC=1;North_Han
chr2	180618042	.	A	<INS:ME:LINE1>	.	PASS	SVTYPE=INS:ME:LINE1;END=180618059;SVLEN=6018;AN=20618;AC=1;AF=4.850130953535746e-05;N_HET=1;N_HOMALT=0;N_HOMREF=10308;FREQ_HET=9.700261907071492e-05;FREQ_HOMALT=0.0;FREQ_HOMREF=0.9999029973809292;N_BI_GENOS=10309;East_Han_AN=6554;East_Han_AC=1;East_Han_AF
chr2	180886448	.	A	<INS:ME:LINE1>	.	PASS	SVTYPE=INS:ME:LINE1;END=180886448;SVLEN=372;AN=20618;AC=1;AF=4.850130953535746e-05;N_HET=1;N_HOMALT=0;N_HOMREF=10308;FREQ_HET=9.700261907071492e-05;FREQ_HOMALT=0.0;FREQ_HOMREF=0.9999029973809292;N_BI_GENOS=10309;East_Han_AN=6554;East_Han_AC=1;East_Han_AF=
chr2	180933207	.	C	<INS:ME:LINE1>	.	PASS	SVTYPE=INS:ME:LINE1;END=180933225;SVLEN=314;AN=20618;AC=1;AF=4.850130953535746e-05;N_HET=1;N_HOMALT=0;N_HOMREF=10308;FREQ_HET=9.700261907071492e-05;FREQ_HOMALT=0.0;FREQ_HOMREF=0.9999029973809292;N_BI_GENOS=10309;Northwest_Han_AN=1296;Northwest_Han_AC=1;No
chr2	181279009	.	A	<INS:ME:LINE1>	.	PASS	SVTYPE=INS:ME:LINE1;END=181279029;SVLEN=181;AN=20618;AC=2;AF=9.700261907071492e-05;N_HET=2;N_HOMALT=0;N_HOMREF=10307;FREQ_HET=0.00019400523814142983;FREQ_HOMALT=0.0;FREQ_HOMREF=0.9998059947618586;N_BI_GENOS=10309;Hui_AN=444;Hui_AC=1;Hui_AF=0.0022522522522
chr2	181435768	.	T	<INS:ME:LINE1>	.	PASS	SVTYPE=INS:ME:LINE1;END=181435814;SVLEN=985;AN=20618;AC=1;AF=4.850130953535746e-05;N_HET=1;N_HOMALT=0;N_HOMREF=10308;FREQ_HET=9.700261907071492e-05;FREQ_HOMALT=0.0;FREQ_HOMREF=0.9999029973809292;N_BI_GENOS=10309;East_Han_AN=6554;East_Han_AC=1;East_Han_AF=
chr2	181761031	.	G	<INS:ME:LINE1>	.	PASS	SVTYPE=INS:ME:LINE1;END=181761044;SVLEN=127;AN=20618;AC=3;AF=0.00014550392860607236;N_HET=3;N_HOMALT=0;N_HOMREF=10306;FREQ_HET=0.0002910078572121447;FREQ_HOMALT=0.0;FREQ_HOMREF=0.9997089921427879;N_BI_GENOS=10309;Northwest_Han_AN=1296;Northwest_Han_AC=1;N
chr2	182022082	.	A	<INS:ME:LINE1>	.	PASS	SVTYPE=INS:ME:LINE1;END=182022092;SVLEN=789;AN=20618;AC=276;AF=0.013386361431758658;N_HET=262;N_HOMALT=7;N_HOMREF=10040;FREQ_HET=0.025414686196527307;FREQ_HOMALT=0.0006790183334950044;FREQ_HOMREF=0.9739062954699776;N_BI_GENOS=10309;South_Han_AN=1496;South
chr2	182033996	.	T	<INS:ME:LINE1>	.	PASS	SVTYPE=INS:ME:LINE1;END=182034011;SVLEN=577;AN=20618;AC=4;AF=0.00019400523814142983;N_HET=4;N_HOMALT=0;N_HOMREF=10305;FREQ_HET=0.00038801047628285966;FREQ_HOMALT=0.0;FREQ_HOMREF=0.9996119895237171;N_BI_GENOS=10309;Yi_AN=422;Yi_AC=4;Yi_AF=0.009478672985781
chr2	182140946	.	T	<INS:ME:LINE1>	.	PASS	SVTYPE=INS:ME:LINE1;END=182140946;SVLEN=366;AN=20618;AC=1;AF=4.850130953535746e-05;N_HET=1;N_HOMALT=0;N_HOMREF=10308;FREQ_HET=9.700261907071492e-05;FREQ_HOMALT=0.0;FREQ_HOMREF=0.9999029973809292;N_BI_GENOS=10309;Mongolian_AN=454;Mongolian_AC=1;Mongolian_A
chr2	182157858	.	G	<INS:ME:LINE1>	.	PASS	SVTYPE=INS:ME:LINE1;END=182157875;SVLEN=915;AN=20618;AC=2;AF=9.700261907071492e-05;N_HET=2;N_HOMALT=0;N_HOMREF=10307;FREQ_HET=0.00019400523814142983;FREQ_HOMALT=0.0;FREQ_HOMREF=0.9998059947618586;N_BI_GENOS=10309;Central_Han_AN=2128;Central_Han_AC=2;Centr
chr2	182199834	.	A	<INS:ME:LINE1>	.	PASS	SVTYPE=INS:ME:LINE1;END=182199847;SVLEN=5462;AN=20618;AC=1;AF=4.850130953535746e-05;N_HET=1;N_HOMALT=0;N_HOMREF=10308;FREQ_HET=9.700261907071492e-05;FREQ_HOMALT=0.0;FREQ_HOMREF=0.9999029973809292;N_BI_GENOS=10309;North_Han_AN=2272;North_Han_AC=1;North_Han
chr2	182257410	.	C	<INS:ME:LINE1>	.	PASS	SVTYPE=INS:ME:LINE1;END=182257421;SVLEN=2525;AN=20618;AC=1;AF=4.850130953535746e-05;N_HET=1;N_HOMALT=0;N_HOMREF=10308;FREQ_HET=9.700261907071492e-05;FREQ_HOMALT=0.0;FREQ_HOMREF=0.9999029973809292;N_BI_GENOS=10309;Manchu_AN=416;Manchu_AC=1;Manchu_AF=0.0024
chr2	182457972	.	A	<INS:ME:LINE1>	.	PASS	SVTYPE=INS:ME:LINE1;END=182457986;SVLEN=6006;AN=20618;AC=1;AF=4.850130953535746e-05;N_HET=1;N_HOMALT=0;N_HOMREF=10308;FREQ_HET=9.700261907071492e-05;FREQ_HOMALT=0.0;FREQ_HOMREF=0.9999029973809292;N_BI_GENOS=10309;East_Han_AN=6554;East_Han_AC=1;East_Han_AF
chr2	182467981	.	A	<INS:ME:LINE1>	.	PASS	SVTYPE=INS:ME:LINE1;END=182467997;SVLEN=2025;AN=20618;AC=1;AF=4.850130953535746e-05;N_HET=1;N_HOMALT=0;N_HOMREF=10308;FREQ_HET=9.700261907071492e-05;FREQ_HOMALT=0.0;FREQ_HOMREF=0.9999029973809292;N_BI_GENOS=10309;Central_Han_AN=2128;Central_Han_AC=1;Centr
chr2	182468404	.	A	<INS:ME:LINE1>	.	PASS	SVTYPE=INS:ME:LINE1;END=182468419;SVLEN=5966;AN=20618;AC=3;AF=0.00014550392860607236;N_HET=3;N_HOMALT=0;N_HOMREF=10306;FREQ_HET=0.0002910078572121447;FREQ_HOMALT=0.0;FREQ_HOMREF=0.9997089921427879;N_BI_GENOS=10309;Northwest_Han_AN=1296;Northwest_Han_AC=1;
chr2	182481168	.	A	<INS:ME:LINE1>	.	PASS	SVTYPE=INS:ME:LINE1;END=182481183;SVLEN=1836;AN=20618;AC=2;AF=9.700261907071492e-05;N_HET=2;N_HOMALT=0;N_HOMREF=10307;FREQ_HET=0.00019400523814142983;FREQ_HOMALT=0.0;FREQ_HOMREF=0.9998059947618586;N_BI_GENOS=10309;East_Han_AN=6554;East_Han_AC=1;East_Han_A
chr2	182718839	.	A	<INS:ME:LINE1>	.	PASS	SVTYPE=INS:ME:LINE1;END=182718855;SVLEN=5997;AN=20618;AC=1;AF=4.850130953535746e-05;N_HET=1;N_HOMALT=0;N_HOMREF=10308;FREQ_HET=9.700261907071492e-05;FREQ_HOMALT=0.0;FREQ_HOMREF=0.9999029973809292;N_BI_GENOS=10309;Southeast_Han_AN=1908;Southeast_Han_AC=1;S
chr2	182735930	.	T	<INS:ME:LINE1>	.	PASS	SVTYPE=INS:ME:LINE1;END=182735947;SVLEN=914;AN=20618;AC=1;AF=4.850130953535746e-05;N_HET=1;N_HOMALT=0;N_HOMREF=10308;FREQ_HET=9.700261907071492e-05;FREQ_HOMALT=0.0;FREQ_HOMREF=0.9999029973809292;N_BI_GENOS=10309;Southeast_Han_AN=1908;Southeast_Han_AC=1;So
chr2	182779943	.	C	<INS:ME:LINE1>	.	PASS	SVTYPE=INS:ME:LINE1;END=182779963;SVLEN=5997;AN=20618;AC=2;AF=9.700261907071492e-05;N_HET=2;N_HOMALT=0;N_HOMREF=10307;FREQ_HET=0.00019400523814142983;FREQ_HOMALT=0.0;FREQ_HOMREF=0.9998059947618586;N_BI_GENOS=10309;Central_Han_AN=2128;Central_Han_AC=2;Cent
chr2	183192145	.	A	<INS:ME:LINE1>	.	PASS	SVTYPE=INS:ME:LINE1;END=183192163;SVLEN=342;AN=20618;AC=1;AF=4.850130953535746e-05;N_HET=1;N_HOMALT=0;N_HOMREF=10308;FREQ_HET=9.700261907071492e-05;FREQ_HOMALT=0.0;FREQ_HOMREF=0.9999029973809292;N_BI_GENOS=10309;Northwest_Han_AN=1296;Northwest_Han_AC=1;No
chr2	183383607	.	G	<INS:ME:LINE1>	.	PASS	SVTYPE=INS:ME:LINE1;END=183383622;SVLEN=6007;AN=20618;AC=1;AF=4.850130953535746e-05;N_HET=1;N_HOMALT=0;N_HOMREF=10308;FREQ_HET=9.700261907071492e-05;FREQ_HOMALT=0.0;FREQ_HOMREF=0.9999029973809292;N_BI_GENOS=10309;South_Han_AN=1496;South_Han_AC=1;South_Han
chr2	183631270	.	T	<INS:ME:LINE1>	.	PASS	SVTYPE=INS:ME:LINE1;END=183631285;SVLEN=1183;AN=20618;AC=1;AF=4.850130953535746e-05;N_HET=1;N_HOMALT=0;N_HOMREF=10308;FREQ_HET=9.700261907071492e-05;FREQ_HOMALT=0.0;FREQ_HOMREF=0.9999029973809292;N_BI_GENOS=10309;Southeast_Han_AN=1908;Southeast_Han_AC=1;S
chr2	183661602	.	A	<INS:ME:LINE1>	.	PASS	SVTYPE=INS:ME:LINE1;END=183661616;SVLEN=5993;AN=20618;AC=2;AF=9.700261907071492e-05;N_HET=2;N_HOMALT=0;N_HOMREF=10307;FREQ_HET=0.00019400523814142983;FREQ_HOMALT=0.0;FREQ_HOMREF=0.9998059947618586;N_BI_GENOS=10309;Central_Han_AN=2128;Central_Han_AC=1;Cent
chr2	183927239	.	G	<INS:ME:LINE1>	.	PASS	SVTYPE=INS:ME:LINE1;END=183927253;SVLEN=283;AN=20618;AC=1;AF=4.850130953535746e-05;N_HET=1;N_HOMALT=0;N_HOMREF=10308;FREQ_HET=9.700261907071492e-05;FREQ_HOMALT=0.0;FREQ_HOMREF=0.9999029973809292;N_BI_GENOS=10309;Southeast_Han_AN=1908;Southeast_Han_AC=1;So
chr2	183996744	.	A	<INS:ME:LINE1>	.	PASS	SVTYPE=INS:ME:LINE1;END=183996771;SVLEN=296;AN=20618;AC=2;AF=9.700261907071492e-05;N_HET=2;N_HOMALT=0;N_HOMREF=10307;FREQ_HET=0.00019400523814142983;FREQ_HOMALT=0.0;FREQ_HOMREF=0.9998059947618586;N_BI_GENOS=10309;Central_Han_AN=2128;Central_Han_AC=2;Centr
chr2	184028837	.	G	<INS:ME:LINE1>	.	PASS	SVTYPE=INS:ME:LINE1;END=184028851;SVLEN=1244;AN=20618;AC=1;AF=4.850130953535746e-05;N_HET=1;N_HOMALT=0;N_HOMREF=10308;FREQ_HET=9.700261907071492e-05;FREQ_HOMALT=0.0;FREQ_HOMREF=0.9999029973809292;N_BI_GENOS=10309;Northwest_Han_AN=1296;Northwest_Han_AC=1;N
chr2	184155914	.	A	<INS:ME:LINE1>	.	PASS	SVTYPE=INS:ME:LINE1;END=184155928;SVLEN=2017;AN=20618;AC=1;AF=4.850130953535746e-05;N_HET=1;N_HOMALT=0;N_HOMREF=10308;FREQ_HET=9.700261907071492e-05;FREQ_HOMALT=0.0;FREQ_HOMREF=0.9999029973809292;N_BI_GENOS=10309;Lingnan_Han_AN=1888;Lingnan_Han_AC=1;Lingn
chr2	184253405	.	G	<INS:ME:LINE1>	.	PASS	SVTYPE=INS:ME:LINE1;END=184253422;SVLEN=5999;AN=20618;AC=1;AF=4.850130953535746e-05;N_HET=1;N_HOMALT=0;N_HOMREF=10308;FREQ_HET=9.700261907071492e-05;FREQ_HOMALT=0.0;FREQ_HOMREF=0.9999029973809292;N_BI_GENOS=10309;Hui_AN=444;Hui_AC=1;Hui_AF=0.0022522522522
chr2	184751837	.	A	<INS:ME:LINE1>	.	PASS	SVTYPE=INS:ME:LINE1;END=184751837;SVLEN=528;AN=20618;AC=1;AF=4.850130953535746e-05;N_HET=1;N_HOMALT=0;N_HOMREF=10308;FREQ_HET=9.700261907071492e-05;FREQ_HOMALT=0.0;FREQ_HOMREF=0.9999029973809292;N_BI_GENOS=10309;East_Han_AN=6554;East_Han_AC=1;East_Han_AF=
chr2	184751893	.	A	<INS:ME:LINE1>	.	PASS	SVTYPE=INS:ME:LINE1;END=184751893;SVLEN=1037;AN=20618;AC=1;AF=4.850130953535746e-05;N_HET=1;N_HOMALT=0;N_HOMREF=10308;FREQ_HET=9.700261907071492e-05;FREQ_HOMALT=0.0;FREQ_HOMREF=0.9999029973809292;N_BI_GENOS=10309;South_Han_AN=1496;South_Han_AC=1;South_Han
chr2	185561152	.	A	<INS:ME:LINE1>	.	PASS	SVTYPE=INS:ME:LINE1;END=185561169;SVLEN=5994;AN=20618;AC=19;AF=0.0009215248811717916;N_HET=17;N_HOMALT=1;N_HOMREF=10291;FREQ_HET=0.0016490445242021534;FREQ_HOMALT=9.700261907071492e-05;FREQ_HOMREF=0.9982539528567271;N_BI_GENOS=10309;South_Han_AN=1496;Sout
chr2	185658892	.	G	<INS:ME:LINE1>	.	PASS	SVTYPE=INS:ME:LINE1;END=185658892;SVLEN=23;AN=20618;AC=1;AF=4.850130953535746e-05;N_HET=1;N_HOMALT=0;N_HOMREF=10308;FREQ_HET=9.700261907071492e-05;FREQ_HOMALT=0.0;FREQ_HOMREF=0.9999029973809292;N_BI_GENOS=10309;East_Han_AN=6554;East_Han_AC=1;East_Han_AF=0
chr2	185712769	.	T	<INS:ME:LINE1>	.	PASS	SVTYPE=INS:ME:LINE1;END=185712781;SVLEN=2570;AN=20618;AC=3;AF=0.00014550392860607236;N_HET=3;N_HOMALT=0;N_HOMREF=10306;FREQ_HET=0.0002910078572121447;FREQ_HOMALT=0.0;FREQ_HOMREF=0.9997089921427879;N_BI_GENOS=10309;South_Han_AN=1496;South_Han_AC=1;South_Ha
chr2	185752244	.	A	<INS:ME:LINE1>	.	PASS	SVTYPE=INS:ME:LINE1;END=185752258;SVLEN=167;AN=20618;AC=1;AF=4.850130953535746e-05;N_HET=1;N_HOMALT=0;N_HOMREF=10308;FREQ_HET=9.700261907071492e-05;FREQ_HOMALT=0.0;FREQ_HOMREF=0.9999029973809292;N_BI_GENOS=10309;Lingnan_Han_AN=1888;Lingnan_Han_AC=1;Lingna
chr2	185969742	.	A	<INS:ME:LINE1>	.	PASS	SVTYPE=INS:ME:LINE1;END=185969759;SVLEN=1781;AN=20618;AC=6;AF=0.0002910078572121447;N_HET=6;N_HOMALT=0;N_HOMREF=10303;FREQ_HET=0.0005820157144242894;FREQ_HOMALT=0.0;FREQ_HOMREF=0.9994179842855757;N_BI_GENOS=10309;Northwest_Han_AN=1296;Northwest_Han_AC=1;N
chr2	186062773	.	A	<INS:ME:LINE1>	.	PASS	SVTYPE=INS:ME:LINE1;END=186062786;SVLEN=1565;AN=20618;AC=2;AF=9.700261907071492e-05;N_HET=2;N_HOMALT=0;N_HOMREF=10307;FREQ_HET=0.00019400523814142983;FREQ_HOMALT=0.0;FREQ_HOMREF=0.9998059947618586;N_BI_GENOS=10309;Miao_AN=482;Miao_AC=2;Miao_AF=0.004149377
chr2	186610524	.	A	<INS:ME:LINE1>	.	PASS	SVTYPE=INS:ME:LINE1;END=186610540;SVLEN=1166;AN=20618;AC=2;AF=9.700261907071492e-05;N_HET=2;N_HOMALT=0;N_HOMREF=10307;FREQ_HET=0.00019400523814142983;FREQ_HOMALT=0.0;FREQ_HOMREF=0.9998059947618586;N_BI_GENOS=10309;Central_Han_AN=2128;Central_Han_AC=1;Cent
chr2	186770489	.	A	<INS:ME:LINE1>	.	PASS	SVTYPE=INS:ME:LINE1;END=186770503;SVLEN=6000;AN=20618;AC=2;AF=9.700261907071492e-05;N_HET=2;N_HOMALT=0;N_HOMREF=10307;FREQ_HET=0.00019400523814142983;FREQ_HOMALT=0.0;FREQ_HOMREF=0.9998059947618586;N_BI_GENOS=10309;South_Han_AN=1496;South_Han_AC=1;South_Ha
chr2	187081372	.	T	<INS:ME:LINE1>	.	PASS	SVTYPE=INS:ME:LINE1;END=187081372;SVLEN=62;AN=20618;AC=1;AF=4.850130953535746e-05;N_HET=1;N_HOMALT=0;N_HOMREF=10308;FREQ_HET=9.700261907071492e-05;FREQ_HOMALT=0.0;FREQ_HOMREF=0.9999029973809292;N_BI_GENOS=10309;East_Han_AN=6554;East_Han_AC=1;East_Han_AF=0
chr2	187274404	.	T	<INS:ME:LINE1>	.	PASS	SVTYPE=INS:ME:LINE1;END=187274404;SVLEN=135;AN=20618;AC=1;AF=4.850130953535746e-05;N_HET=1;N_HOMALT=0;N_HOMREF=10308;FREQ_HET=9.700261907071492e-05;FREQ_HOMALT=0.0;FREQ_HOMREF=0.9999029973809292;N_BI_GENOS=10309;East_Han_AN=6554;East_Han_AC=1;East_Han_AF=
chr2	187275332	.	T	<INS:ME:LINE1>	.	PASS	SVTYPE=INS:ME:LINE1;END=187275332;SVLEN=439;AN=20618;AC=1;AF=4.850130953535746e-05;N_HET=1;N_HOMALT=0;N_HOMREF=10308;FREQ_HET=9.700261907071492e-05;FREQ_HOMALT=0.0;FREQ_HOMREF=0.9999029973809292;N_BI_GENOS=10309;East_Han_AN=6554;East_Han_AC=1;East_Han_AF=
chr2	187721282	.	T	<INS:ME:LINE1>	.	PASS	SVTYPE=INS:ME:LINE1;END=187721282;SVLEN=260;AN=20618;AC=1;AF=4.850130953535746e-05;N_HET=1;N_HOMALT=0;N_HOMREF=10308;FREQ_HET=9.700261907071492e-05;FREQ_HOMALT=0.0;FREQ_HOMREF=0.9999029973809292;N_BI_GENOS=10309;East_Han_AN=6554;East_Han_AC=1;East_Han_AF=
chr2	188000437	.	G	<INS:ME:LINE1>	.	PASS	SVTYPE=INS:ME:LINE1;END=188000444;SVLEN=1033;AN=20618;AC=3;AF=0.00014550392860607236;N_HET=3;N_HOMALT=0;N_HOMREF=10306;FREQ_HET=0.0002910078572121447;FREQ_HOMALT=0.0;FREQ_HOMREF=0.9997089921427879;N_BI_GENOS=10309;East_Han_AN=6554;East_Han_AC=3;East_Han_A
chr2	188525079	.	C	<INS:ME:LINE1>	.	PASS	SVTYPE=INS:ME:LINE1;END=188525095;SVLEN=494;AN=20618;AC=3;AF=0.00014550392860607236;N_HET=3;N_HOMALT=0;N_HOMREF=10306;FREQ_HET=0.0002910078572121447;FREQ_HOMALT=0.0;FREQ_HOMREF=0.9997089921427879;N_BI_GENOS=10309;South_Han_AN=1496;South_Han_AC=1;South_Han
chr2	188629859	.	A	<INS:ME:LINE1>	.	PASS	SVTYPE=INS:ME:LINE1;END=188629866;SVLEN=214;AN=20618;AC=1;AF=4.850130953535746e-05;N_HET=1;N_HOMALT=0;N_HOMREF=10308;FREQ_HET=9.700261907071492e-05;FREQ_HOMALT=0.0;FREQ_HOMREF=0.9999029973809292;N_BI_GENOS=10309;North_Han_AN=2272;North_Han_AC=1;North_Han_
chr2	188774175	.	A	<INS:ME:LINE1>	.	PASS	SVTYPE=INS:ME:LINE1;END=188774175;SVLEN=471;AN=20618;AC=1;AF=4.850130953535746e-05;N_HET=1;N_HOMALT=0;N_HOMREF=10308;FREQ_HET=9.700261907071492e-05;FREQ_HOMALT=0.0;FREQ_HOMREF=0.9999029973809292;N_BI_GENOS=10309;Tibetan_AN=414;Tibetan_AC=1;Tibetan_AF=0.00
chr2	189071106	.	C	<INS:ME:LINE1>	.	PASS	SVTYPE=INS:ME:LINE1;END=189071107;SVLEN=1627;AN=20618;AC=5;AF=0.00024250654767678728;N_HET=3;N_HOMALT=1;N_HOMREF=10305;FREQ_HET=0.0002910078572121447;FREQ_HOMALT=9.700261907071492e-05;FREQ_HOMREF=0.9996119895237171;N_BI_GENOS=10309;South_Han_AN=1496;South
chr2	189375838	.	A	<INS:ME:LINE1>	.	PASS	SVTYPE=INS:ME:LINE1;END=189375849;SVLEN=5999;AN=20618;AC=5;AF=0.00024250654767678728;N_HET=5;N_HOMALT=0;N_HOMREF=10304;FREQ_HET=0.00048501309535357455;FREQ_HOMALT=0.0;FREQ_HOMREF=0.9995149869046465;N_BI_GENOS=10309;Lingnan_Han_AN=1888;Lingnan_Han_AC=1;Lin
chr2	189769002	.	A	<INS:ME:LINE1>	.	PASS	SVTYPE=INS:ME:LINE1;END=189769017;SVLEN=5979;AN=20618;AC=1;AF=4.850130953535746e-05;N_HET=1;N_HOMALT=0;N_HOMREF=10308;FREQ_HET=9.700261907071492e-05;FREQ_HOMALT=0.0;FREQ_HOMREF=0.9999029973809292;N_BI_GENOS=10309;South_Han_AN=1496;South_Han_AC=1;South_Han
chr2	189896183	.	T	<INS:ME:LINE1>	.	PASS	SVTYPE=INS:ME:LINE1;END=189896193;SVLEN=92;AN=20618;AC=86;AF=0.0041711112620040741;N_HET=82;N_HOMALT=2;N_HOMREF=10225;FREQ_HET=0.007954214763798623;FREQ_HOMALT=0.00019400523814142983;FREQ_HOMREF=0.99185177999806;N_BI_GENOS=10309;South_Han_AN=1496;South_Han
chr2	189928619	.	T	<INS:ME:LINE1>	.	PASS	SVTYPE=INS:ME:LINE1;END=189928633;SVLEN=921;AN=20618;AC=1;AF=4.850130953535746e-05;N_HET=1;N_HOMALT=0;N_HOMREF=10308;FREQ_HET=9.700261907071492e-05;FREQ_HOMALT=0.0;FREQ_HOMREF=0.9999029973809292;N_BI_GENOS=10309;Northwest_Han_AN=1296;Northwest_Han_AC=1;No
chr2	190516333	.	A	<INS:ME:LINE1>	.	PASS	SVTYPE=INS:ME:LINE1;END=190516346;SVLEN=1369;AN=20618;AC=1;AF=4.850130953535746e-05;N_HET=1;N_HOMALT=0;N_HOMREF=10308;FREQ_HET=9.700261907071492e-05;FREQ_HOMALT=0.0;FREQ_HOMREF=0.9999029973809292;N_BI_GENOS=10309;East_Han_AN=6554;East_Han_AC=1;East_Han_AF
chr2	190613974	.	T	<INS:ME:LINE1>	.	PASS	SVTYPE=INS:ME:LINE1;END=190613988;SVLEN=5997;AN=20618;AC=3757;AF=0.18221941992433796;N_HET=2969;N_HOMALT=394;N_HOMREF=6946;FREQ_HET=0.2880007760209526;FREQ_HOMALT=0.038219031913861674;FREQ_HOMREF=0.6737801920651858;N_BI_GENOS=10309;South_Han_AN=1496;South
chr2	191144629	.	A	<INS:ME:LINE1>	.	PASS	SVTYPE=INS:ME:LINE1;END=191144634;SVLEN=828;AN=20618;AC=503;AF=0.0243961586962848;N_HET=461;N_HOMALT=21;N_HOMREF=9827;FREQ_HET=0.044718207391599575;FREQ_HOMALT=0.002037055000485013;FREQ_HOMREF=0.9532447376079154;N_BI_GENOS=10309;South_Han_AN=1496;South_Ha
chr2	191628773	.	A	<INS:ME:LINE1>	.	PASS	SVTYPE=INS:ME:LINE1;END=191628787;SVLEN=671;AN=20618;AC=79;AF=0.0038316034532932387;N_HET=79;N_HOMALT=0;N_HOMREF=10230;FREQ_HET=0.007663206906586477;FREQ_HOMALT=0.0;FREQ_HOMREF=0.9923367930934135;N_BI_GENOS=10309;South_Han_AN=1496;South_Han_AC=9;South_Han
chr2	191801794	.	A	<INS:ME:LINE1>	.	PASS	SVTYPE=INS:ME:LINE1;END=191801810;SVLEN=2519;AN=20618;AC=1;AF=4.850130953535746e-05;N_HET=1;N_HOMALT=0;N_HOMREF=10308;FREQ_HET=9.700261907071492e-05;FREQ_HOMALT=0.0;FREQ_HOMREF=0.9999029973809292;N_BI_GENOS=10309;East_Han_AN=6554;East_Han_AC=1;East_Han_AF
chr2	192087541	.	T	<INS:ME:LINE1>	.	PASS	SVTYPE=INS:ME:LINE1;END=192087556;SVLEN=1194;AN=20618;AC=3;AF=0.00014550392860607236;N_HET=3;N_HOMALT=0;N_HOMREF=10306;FREQ_HET=0.0002910078572121447;FREQ_HOMALT=0.0;FREQ_HOMREF=0.9997089921427879;N_BI_GENOS=10309;East_Han_AN=6554;East_Han_AC=1;East_Han_A
chr2	192267972	.	T	<INS:ME:LINE1>	.	PASS	SVTYPE=INS:ME:LINE1;END=192267990;SVLEN=6016;AN=20618;AC=1;AF=4.850130953535746e-05;N_HET=1;N_HOMALT=0;N_HOMREF=10308;FREQ_HET=9.700261907071492e-05;FREQ_HOMALT=0.0;FREQ_HOMREF=0.9999029973809292;N_BI_GENOS=10309;Central_Han_AN=2128;Central_Han_AC=1;Centr
chr2	192801580	.	A	<INS:ME:LINE1>	.	PASS	SVTYPE=INS:ME:LINE1;END=192801599;SVLEN=5999;AN=20618;AC=11;AF=0.000533514404888932;N_HET=11;N_HOMALT=0;N_HOMREF=10298;FREQ_HET=0.001067028809777864;FREQ_HOMALT=0.0;FREQ_HOMREF=0.9989329711902222;N_BI_GENOS=10309;South_Han_AN=1496;South_Han_AC=2;South_Han
chr2	192942761	.	G	<INS:ME:LINE1>	.	PASS	SVTYPE=INS:ME:LINE1;END=192942775;SVLEN=762;AN=20618;AC=7;AF=0.0003395091667475022;N_HET=7;N_HOMALT=0;N_HOMREF=10302;FREQ_HET=0.0006790183334950044;FREQ_HOMALT=0.0;FREQ_HOMREF=0.999320981666505;N_BI_GENOS=10309;South_Han_AN=1496;South_Han_AC=1;South_Han_A
chr2	193208677	.	A	<INS:ME:LINE1>	.	PASS	SVTYPE=INS:ME:LINE1;END=193208695;SVLEN=914;AN=20618;AC=5;AF=0.00024250654767678728;N_HET=5;N_HOMALT=0;N_HOMREF=10304;FREQ_HET=0.00048501309535357455;FREQ_HOMALT=0.0;FREQ_HOMREF=0.9995149869046465;N_BI_GENOS=10309;South_Han_AN=1496;South_Han_AC=1;South_Ha
chr2	193566799	.	A	<INS:ME:LINE1>	.	PASS	SVTYPE=INS:ME:LINE1;END=193566814;SVLEN=797;AN=20618;AC=1;AF=4.850130953535746e-05;N_HET=1;N_HOMALT=0;N_HOMREF=10308;FREQ_HET=9.700261907071492e-05;FREQ_HOMALT=0.0;FREQ_HOMREF=0.9999029973809292;N_BI_GENOS=10309;East_Han_AN=6554;East_Han_AC=1;East_Han_AF=
chr2	193704391	.	A	<INS:ME:LINE1>	.	PASS	SVTYPE=INS:ME:LINE1;END=193704406;SVLEN=1434;AN=20618;AC=1;AF=4.850130953535746e-05;N_HET=1;N_HOMALT=0;N_HOMREF=10308;FREQ_HET=9.700261907071492e-05;FREQ_HOMALT=0.0;FREQ_HOMREF=0.9999029973809292;N_BI_GENOS=10309;North_Han_AN=2272;North_Han_AC=1;North_Han
chr2	194245509	.	A	<INS:ME:LINE1>	.	PASS	SVTYPE=INS:ME:LINE1;END=194245526;SVLEN=1214;AN=20618;AC=1;AF=4.850130953535746e-05;N_HET=1;N_HOMALT=0;N_HOMREF=10308;FREQ_HET=9.700261907071492e-05;FREQ_HOMALT=0.0;FREQ_HOMREF=0.9999029973809292;N_BI_GENOS=10309;Southeast_Han_AN=1908;Southeast_Han_AC=1;S

chr2	194401780	.	A	<INS:ME:LINE1>	.	PASS	SVTYPE=INS:ME:LINE1;END=194401797;SVLEN=3251;AN=20618;AC=1;AF=4.850130953535746e-05;N_HET=1;N_HOMALT=0;N_HOMREF=10308;FREQ_HET=9.700261907071492e-05;FREQ_HOMALT=0.0;FREQ_HOMREF=0.9999029973809292;N_BI_GENOS=10309;Southeast_Han_AN=1908;Southeast_Han_AC=1;S
chr2	194411892	.	A	<INS:ME:LINE1>	.	PASS	SVTYPE=INS:ME:LINE1;END=194411901;SVLEN=1671;AN=20618;AC=1;AF=4.850130953535746e-05;N_HET=1;N_HOMALT=0;N_HOMREF=10308;FREQ_HET=9.700261907071492e-05;FREQ_HOMALT=0.0;FREQ_HOMREF=0.9999029973809292;N_BI_GENOS=10309;Lingnan_Han_AN=1888;Lingnan_Han_AC=1;Lingn
chr2	194437920	.	G	<INS:ME:LINE1>	.	PASS	SVTYPE=INS:ME:LINE1;END=194437920;SVLEN=183;AN=20618;AC=1;AF=4.850130953535746e-05;N_HET=1;N_HOMALT=0;N_HOMREF=10308;FREQ_HET=9.700261907071492e-05;FREQ_HOMALT=0.0;FREQ_HOMREF=0.9999029973809292;N_BI_GENOS=10309;East_Han_AN=6554;East_Han_AC=1;East_Han_AF=
chr2	194777488	.	T	<INS:ME:LINE1>	.	PASS	SVTYPE=INS:ME:LINE1;END=194777504;SVLEN=463;AN=20618;AC=1;AF=4.850130953535746e-05;N_HET=1;N_HOMALT=0;N_HOMREF=10308;FREQ_HET=9.700261907071492e-05;FREQ_HOMALT=0.0;FREQ_HOMREF=0.9999029973809292;N_BI_GENOS=10309;East_Han_AN=6554;East_Han_AC=1;East_Han_AF=
chr2	194794793	.	A	<INS:ME:LINE1>	.	PASS	SVTYPE=INS:ME:LINE1;END=194794811;SVLEN=1493;AN=20618;AC=2;AF=9.700261907071492e-05;N_HET=2;N_HOMALT=0;N_HOMREF=10307;FREQ_HET=0.00019400523814142983;FREQ_HOMALT=0.0;FREQ_HOMREF=0.9998059947618586;N_BI_GENOS=10309;Northwest_Han_AN=1296;Northwest_Han_AC=1;
chr2	194923195	.	C	<INS:ME:LINE1>	.	PASS	SVTYPE=INS:ME:LINE1;END=194923203;SVLEN=545;AN=20618;AC=3;AF=0.00014550392860607236;N_HET=3;N_HOMALT=0;N_HOMREF=10306;FREQ_HET=0.00029100785572121447;FREQ_HOMALT=0.0;FREQ_HOMREF=0.9997089921427879;N_BI_GENOS=10309;Lingnan_Han_AN=1888;Lingnan_Han_AC=1;Lingn
chr2	194934660	.	C	<INS:ME:LINE1>	.	PASS	SVTYPE=INS:ME:LINE1;END=194934676;SVLEN=806;AN=20618;AC=1;AF=4.850130953535746e-05;N_HET=1;N_HOMALT=0;N_HOMREF=10308;FREQ_HET=9.700261907071492e-05;FREQ_HOMALT=0.0;FREQ_HOMREF=0.9999029973809292;N_BI_GENOS=10309;Southeast_Han_AN=1908;Southeast_Han_AC=1;So
chr2	195008070	.	A	<INS:ME:LINE1>	.	PASS	SVTYPE=INS:ME:LINE1;END=195008080;SVLEN=425;AN=20618;AC=1;AF=4.850130953535746e-05;N_HET=1;N_HOMALT=0;N_HOMREF=10308;FREQ_HET=9.700261907071492e-05;FREQ_HOMALT=0.0;FREQ_HOMREF=0.9999029973809292;N_BI_GENOS=10309;East_Han_AN=6554;East_Han_AC=1;East_Han_AF=
chr2	195046356	.	A	<INS:ME:LINE1>	.	PASS	SVTYPE=INS:ME:LINE1;END=195046369;SVLEN=5998;AN=20618;AC=2;AF=9.700261907071492e-05;N_HET=2;N_HOMALT=0;N_HOMREF=10307;FREQ_HET=0.00019400523814142983;FREQ_HOMALT=0.0;FREQ_HOMREF=0.9998059947618586;N_BI_GENOS=10309;Hui_AN=444;Hui_AC=2;Hui_AF=0.0045045045045045045045045045045045
chr2	195315669	.	A	<INS:ME:LINE1>	.	PASS	SVTYPE=INS:ME:LINE1;END=195315685;SVLEN=6022;AN=20618;AC=2;AF=9.700261907071492e-05;N_HET=2;N_HOMALT=0;N_HOMREF=10307;FREQ_HET=0.00019400523814142983;FREQ_HOMALT=0.0;FREQ_HOMREF=0.9998059947618586;N_BI_GENOS=10309;Central_Han_AN=2128;Central_Han_AC=1;Cent
chr2	195537101	.	C	<INS:ME:LINE1>	.	PASS	SVTYPE=INS:ME:LINE1;END=195537101;SVLEN=13;AN=20618;AC=5;AF=0.00024250654767678728;N_HET=3;N_HOMALT=1;N_HOMREF=10305;FREQ_HET=0.0002910078572121447;FREQ_HOMALT=9.700261907071492e-05;FREQ_HOMREF=0.9996119895237171;N_BI_GENOS=10309;East_Han_AN=6554;East_Han
chr2	195896921	.	A	<INS:ME:LINE1>	.	PASS	SVTYPE=INS:ME:LINE1;END=195896926;SVLEN=1387;AN=20618;AC=8;AF=0.00038801047628285966;N_HET=8;N_HOMALT=0;N_HOMREF=10301;FREQ_HET=0.0007760209525657193;FREQ_HOMALT=0.0;FREQ_HOMREF=0.9992239790474343;N_BI_GENOS=10309;Lingnan_Han_AN=1888;Lingnan_Han_AC=1;Ling
chr2	196677088	.	A	<INS:ME:LINE1>	.	PASS	SVTYPE=INS:ME:LINE1;END=196677106;SVLEN=819;AN=20618;AC=1;AF=4.850130953535746e-05;N_HET=1;N_HOMALT=0;N_HOMREF=10308;FREQ_HET=9.700261907071492e-05;FREQ_HOMALT=0.0;FREQ_HOMREF=0.9999029973809292;N_BI_GENOS=10309;Southeast_Han_AN=1908;Southeast_Han_AC=1;So
chr2	197204637	.	G	<INS:ME:LINE1>	.	PASS	SVTYPE=INS:ME:LINE1;END=197204654;SVLEN=927;AN=20618;AC=1;AF=4.850130953535746e-05;N_HET=1;N_HOMALT=0;N_HOMREF=10308;FREQ_HET=9.700261907071492e-05;FREQ_HOMALT=0.0;FREQ_HOMREF=0.9999029973809292;N_BI_GENOS=10309;Northwest_Han_AN=1296;Northwest_Han_AC=1;No
chr2	197282969	.	G	<INS:ME:LINE1>	.	PASS	SVTYPE=INS:ME:LINE1;END=197282969;SVLEN=1181;AN=20618;AC=1;AF=4.850130953535746e-05;N_HET=1;N_HOMALT=0;N_HOMREF=10308;FREQ_HET=9.700261907071492e-05;FREQ_HOMALT=0.0;FREQ_HOMREF=0.9999029973809292;N_BI_GENOS=10309;East_Han_AN=6554;East_Han_AC=1;East_Han_AF
chr2	197774436	.	T	<INS:ME:LINE1>	.	PASS	SVTYPE=INS:ME:LINE1;END=197774439;SVLEN=43;AN=20618;AC=1;AF=4.850130953535746e-05;N_HET=1;N_HOMALT=0;N_HOMREF=10308;FREQ_HET=9.700261907071492e-05;FREQ_HOMALT=0.0;FREQ_HOMREF=0.9999029973809292;N_BI_GENOS=10309;Southeast_Han_AN=1908;Southeast_Han_AC=1;Sou
chr2	197873019	.	C	<INS:ME:LINE1>	.	PASS	SVTYPE=INS:ME:LINE1;END=197873036;SVLEN=2592;AN=20618;AC=1;AF=4.850130953535746e-05;N_HET=1;N_HOMALT=0;N_HOMREF=10308;FREQ_HET=9.700261907071492e-05;FREQ_HOMALT=0.0;FREQ_HOMREF=0.9999029973809292;N_BI_GENOS=10309;North_Han_AN=2272;North_Han_AC=1;North_Han
chr2	197874328	.	A	<INS:ME:LINE1>	.	PASS	SVTYPE=INS:ME:LINE1;END=197874346;SVLEN=1230;AN=20618;AC=1;AF=4.850130953535746e-05;N_HET=1;N_HOMALT=0;N_HOMREF=10308;FREQ_HET=9.700261907071492e-05;FREQ_HOMALT=0.0;FREQ_HOMREF=0.9999029973809292;N_BI_GENOS=10309;Central_Han_AN=2128;Central_Han_AC=1;Centr
chr2	197889892	.	G	<INS:ME:LINE1>	.	PASS	SVTYPE=INS:ME:LINE1;END=197889900;SVLEN=5988;AN=20618;AC=1;AF=4.850130953535746e-05;N_HET=1;N_HOMALT=0;N_HOMREF=10308;FREQ_HET=9.700261907071492e-05;FREQ_HOMALT=0.0;FREQ_HOMREF=0.9999029973809292;N_BI_GENOS=10309;East_Han_AN=6554;East_Han_AC=1;East_Han_AF
chr2	198241507	.	A	<INS:ME:LINE1>	.	PASS	SVTYPE=INS:ME:LINE1;END=198241509;SVLEN=690;AN=20618;AC=1;AF=4.850130953535746e-05;N_HET=1;N_HOMALT=0;N_HOMREF=10308;FREQ_HET=9.700261907071492e-05;FREQ_HOMALT=0.0;FREQ_HOMREF=0.9999029973809292;N_BI_GENOS=10309;South_Han_AN=1496;South_Han_AC=1;South_Han_
chr2	198364866	.	A	<INS:ME:LINE1>	.	PASS	SVTYPE=INS:ME:LINE1;END=198364870;SVLEN=828;AN=20618;AC=1;AF=4.850130953535746e-05;N_HET=1;N_HOMALT=0;N_HOMREF=10308;FREQ_HET=9.700261907071492e-05;FREQ_HOMALT=0.0;FREQ_HOMREF=0.9999029973809292;N_BI_GENOS=10309;Northwest_Han_AN=1296;Northwest_Han_AC=1;No
chr2	198410378	.	G	<INS:ME:LINE1>	.	PASS	SVTYPE=INS:ME:LINE1;END=198410393;SVLEN=6083;AN=20618;AC=1;AF=4.850130953535746e-05;N_HET=1;N_HOMALT=0;N_HOMREF=10308;FREQ_HET=9.700261907071492e-05;FREQ_HOMALT=0.0;FREQ_HOMREF=0.9999029973809292;N_BI_GENOS=10309;Southeast_Han_AN=1908;Southeast_Han_AC=1;S
chr2	198473371	.	A	<INS:ME:LINE1>	.	PASS	SVTYPE=INS:ME:LINE1;END=198473371;SVLEN=1430;AN=20618;AC=1;AF=4.850130953535746e-05;N_HET=1;N_HOMALT=0;N_HOMREF=10308;FREQ_HET=9.700261907071492e-05;FREQ_HOMALT=0.0;FREQ_HOMREF=0.9999029973809292;N_BI_GENOS=10309;South_Han_AN=1496;South_Han_AC=1;South_Han
chr2	198482346	.	T	<INS:ME:LINE1>	.	PASS	SVTYPE=INS:ME:LINE1;END=198482363;SVLEN=906;AN=20618;AC=1;AF=4.850130953535746e-05;N_HET=1;N_HOMALT=0;N_HOMREF=10308;FREQ_HET=9.700261907071492e-05;FREQ_HOMALT=0.0;FREQ_HOMREF=0.9999029973809292;N_BI_GENOS=10309;East_Han_AN=6554;East_Han_AC=1;East_Han_AF=
chr2	198826901	.	A	<INS:ME:LINE1>	.	PASS	SVTYPE=INS:ME:LINE1;END=198826915;SVLEN=5998;AN=20618;AC=1;AF=4.850130953535746e-05;N_HET=1;N_HOMALT=0;N_HOMREF=10308;FREQ_HET=9.700261907071492e-05;FREQ_HOMALT=0.0;FREQ_HOMREF=0.9999029973809292;N_BI_GENOS=10309;Mongolian_AN=454;Mongolian_AC=1;Mongolian_
chr2	198877869	.	A	<INS:ME:LINE1>	.	PASS	SVTYPE=INS:ME:LINE1;END=198877885;SVLEN=2600;AN=20618;AC=2;AF=9.700261907071492e-05;N_HET=2;N_HOMALT=0;N_HOMREF=10307;FREQ_HET=0.00019400523814142983;FREQ_HOMALT=0.0;FREQ_HOMREF=0.9998059947618586;N_BI_GENOS=10309;East_Han_AN=6554;East_Han_AC=2;East_Han_A
chr2	198895611	.	T	<INS:ME:LINE1>	.	PASS	SVTYPE=INS:ME:LINE1;END=198895618;SVLEN=2621;AN=20618;AC=1;AF=4.850130953535746e-05;N_HET=1;N_HOMALT=0;N_HOMREF=10308;FREQ_HET=9.700261907071492e-05;FREQ_HOMALT=0.0;FREQ_HOMREF=0.9999029973809292;N_BI_GENOS=10309;East_Han_AN=6554;East_Han_AC=1;East_Han_AF
chr2	198896725	.	A	<INS:ME:LINE1>	.	PASS	SVTYPE=INS:ME:LINE1;END=198896746;SVLEN=1363;AN=20618;AC=1;AF=4.850130953535746e-05;N_HET=1;N_HOMALT=0;N_HOMREF=10308;FREQ_HET=9.700261907071492e-05;FREQ_HOMALT=0.0;FREQ_HOMREF=0.9999029973809292;N_BI_GENOS=10309;East_Han_AN=6554;East_Han_AC=1;East_Han_AF
chr2	198915174	.	A	<INS:ME:LINE1>	.	PASS	SVTYPE=INS:ME:LINE1;END=198915190;SVLEN=6006;AN=20618;AC=11;AF=0.000533514404888932;N_HET=11;N_HOMALT=0;N_HOMREF=10298;FREQ_HET=0.001067028809777864;FREQ_HOMALT=0.0;FREQ_HOMREF=0.9989329711902222;N_BI_GENOS=10309;Northwest_Han_AN=1296;Northwest_Han_AC=4;N
chr2	198936324	.	A	<INS:ME:LINE1>	.	PASS	SVTYPE=INS:ME:LINE1;END=198936334;SVLEN=1185;AN=20618;AC=1;AF=4.850130953535746e-05;N_HET=1;N_HOMALT=0;N_HOMREF=10308;FREQ_HET=9.700261907071492e-05;FREQ_HOMALT=0.0;FREQ_HOMREF=0.9999029973809292;N_BI_GENOS=10309;East_Han_AN=6554;East_Han_AC=1;East_Han_AF
chr2	199332633	.	T	<INS:ME:LINE1>	.	PASS	SVTYPE=INS:ME:LINE1;END=199332633;SVLEN=2859;AN=20618;AC=1;AF=4.850130953535746e-05;N_HET=1;N_HOMALT=0;N_HOMREF=10308;FREQ_HET=9.700261907071492e-05;FREQ_HOMALT=0.0;FREQ_HOMREF=0.9999029973809292;N_BI_GENOS=10309;South_Han_AN=1496;South_Han_AC=1;South_Han
chr2	199625667	.	A	<INS:ME:LINE1>	.	PASS	SVTYPE=INS:ME:LINE1;END=199625683;SVLEN=1230;AN=20618;AC=2;AF=9.700261907071492e-05;N_HET=2;N_HOMALT=0;N_HOMREF=10307;FREQ_HET=0.00019400523814142983;FREQ_HOMALT=0.0;FREQ_HOMREF=0.9998059947618586;N_BI_GENOS=10309;North_Han_AN=2272;North_Han_AC=1;North_Ha
chr2	199797746	.	A	<INS:ME:LINE1>	.	PASS	SVTYPE=INS:ME:LINE1;END=199797762;SVLEN=1032;AN=20618;AC=7;AF=0.0003395091667475022;N_HET=7;N_HOMALT=0;N_HOMREF=10302;FREQ_HET=0.0006790183334950044;FREQ_HOMALT=0.0;FREQ_HOMREF=0.999320981666505;N_BI_GENOS=10309;South_Han_AN=1496;South_Han_AC=1;South_Han_
chr2	199830314	.	A	<INS:ME:LINE1>	.	PASS	SVTYPE=INS:ME:LINE1;END=199830331;SVLEN=5998;AN=20618;AC=6;AF=0.0002910078572121447;N_HET=6;N_HOMALT=0;N_HOMREF=10303;FREQ_HET=0.0005820157144242894;FREQ_HOMALT=0.0;FREQ_HOMREF=0.9994179842855757;N_BI_GENOS=10309;South_Han_AN=1496;South_Han_AC=1;South_Han
chr2	200059514	.	T	<INS:ME:LINE1>	.	PASS	SVTYPE=INS:ME:LINE1;END=200059514;SVLEN=685;AN=20618;AC=1;AF=4.850130953535746e-05;N_HET=1;N_HOMALT=0;N_HOMREF=10308;FREQ_HET=9.700261907071492e-05;FREQ_HOMALT=0.0;FREQ_HOMREF=0.9999029973809292;N_BI_GENOS=10309;East_Han_AN=6554;East_Han_AC=1;East_Han_AF=
chr2	200059625	.	A	<INS:ME:LINE1>	.	PASS	SVTYPE=INS:ME:LINE1;END=200059625;SVLEN=925;AN=20618;AC=2;AF=9.700261907071492e-05;N_HET=2;N_HOMALT=0;N_HOMREF=10307;FREQ_HET=0.00019400523814142983;FREQ_HOMALT=0.0;FREQ_HOMREF=0.9998059947618586;N_BI_GENOS=10309;East_Han_AN=6554;East_Han_AC=2;East_Han_AF
chr2	200320832	.	A	<INS:ME:LINE1>	.	PASS	SVTYPE=INS:ME:LINE1;END=200320837;SVLEN=547;AN=20618;AC=1;AF=4.850130953535746e-05;N_HET=1;N_HOMALT=0;N_HOMREF=10308;FREQ_HET=9.700261907071492e-05;FREQ_HOMALT=0.0;FREQ_HOMREF=0.9999029973809292;N_BI_GENOS=10309;South_Han_AN=1496;South_Han_AC=1;South_Han_
chr2	200638624	.	A	<INS:ME:LINE1>	.	PASS	SVTYPE=INS:ME:LINE1;END=200638635;SVLEN=5998;AN=20618;AC=1;AF=4.850130953535746e-05;N_HET=1;N_HOMALT=0;N_HOMREF=10308;FREQ_HET=9.700261907071492e-05;FREQ_HOMALT=0.0;FREQ_HOMREF=0.9999029973809292;N_BI_GENOS=10309;South_Han_AN=1496;South_Han_AC=1;South_Han
chr2	200773344	.	A	<INS:ME:LINE1>	.	PASS	SVTYPE=INS:ME:LINE1;END=200773362;SVLEN=916;AN=20618;AC=1260;AF=0.06111165001455039;N_HET=1064;N_HOMALT=98;N_HOMREF=9147;FREQ_HET=0.10321078669124066;FREQ_HOMALT=0.009506256668930062;FREQ_HOMREF=0.8872829566398293;N_BI_GENOS=10309;South_Han_AN=1496;South_
chr2	201588679	.	A	<INS:ME:LINE1>	.	PASS	SVTYPE=INS:ME:LINE1;END=201588679;SVLEN=746;AN=20618;AC=4;AF=0.00019400523814142983;N_HET=4;N_HOMALT=0;N_HOMREF=10305;FREQ_HET=0.00038801047628285966;FREQ_HOMALT=0.0;FREQ_HOMREF=0.9996119895237171;N_BI_GENOS=10309;East_Han_AN=6554;East_Han_AC=4;East_Han_A
chr2	201589490	.	A	<INS:ME:LINE1>	.	PASS	SVTYPE=INS:ME:LINE1;END=201589490;SVLEN=1430;AN=20618;AC=3;AF=0.00014550392860607236;N_HET=3;N_HOMALT=0;N_HOMREF=10306;FREQ_HET=0.0002910078572121447;FREQ_HOMALT=0.0;FREQ_HOMREF=0.9997089921427879;N_BI_GENOS=10309;East_Han_AN=6554;East_Han_AC=2;East_Han_A
chr2	202099623	.	A	<INS:ME:LINE1>	.	PASS	SVTYPE=INS:ME:LINE1;END=202099641;SVLEN=704;AN=20618;AC=4;AF=0.00019400523814142983;N_HET=4;N_HOMALT=0;N_HOMREF=10305;FREQ_HET=0.00038801047628285966;FREQ_HOMALT=0.0;FREQ_HOMREF=0.9996119895237171;N_BI_GENOS=10309;North_Han_AN=2272;North_Han_AC=1;North_Ha
chr2	202142150	.	A	<INS:ME:LINE1>	.	PASS	SVTYPE=INS:ME:LINE1;END=202142161;SVLEN=5999;AN=20618;AC=1;AF=4.850130953535746e-05;N_HET=1;N_HOMALT=0;N_HOMREF=10308;FREQ_HET=9.700261907071492e-05;FREQ_HOMALT=0.0;FREQ_HOMREF=0.9999029973809292;N_BI_GENOS=10309;South_Han_AN=1496;South_Han_AC=1;South_Han
chr2	202577448	.	T	<INS:ME:LINE1>	.	PASS	SVTYPE=INS:ME:LINE1;END=202577466;SVLEN=1351;AN=20618;AC=1;AF=4.850130953535746e-05;N_HET=1;N_HOMALT=0;N_HOMREF=10308;FREQ_HET=9.700261907071492e-05;FREQ_HOMALT=0.0;FREQ_HOMREF=0.9999029973809292;N_BI_GENOS=10309;North_Han_AN=2272;North_Han_AC=1;North_Han
chr2	202588773	.	A	<INS:ME:LINE1>	.	PASS	SVTYPE=INS:ME:LINE1;END=202588784;SVLEN=744;AN=20618;AC=1;AF=4.850130953535746e-05;N_HET=1;N_HOMALT=0;N_HOMREF=10308;FREQ_HET=9.700261907071492e-05;FREQ_HOMALT=0.0;FREQ_HOMREF=0.9999029973809292;N_BI_GENOS=10309;North_Han_AN=2272;North_Han_AC=1;North_Han_
chr2	203197454	.	A	<INS:ME:LINE1>	.	PASS	SVTYPE=INS:ME:LINE1;END=203197472;SVLEN=1120;AN=20618;AC=1;AF=4.850130953535746e-05;N_HET=1;N_HOMALT=0;N_HOMREF=10308;FREQ_HET=9.700261907071492e-05;FREQ_HOMALT=0.0;FREQ_HOMREF=0.9999029973809292;N_BI_GENOS=10309;East_Han_AN=6554;East_Han_AC=1;East_Han_AF
chr2	203323854	.	A	<INS:ME:LINE1>	.	PASS	SVTYPE=INS:ME:LINE1;END=203323868;SVLEN=301;AN=20618;AC=1;AF=4.850130953535746e-05;N_HET=1;N_HOMALT=0;N_HOMREF=10308;FREQ_HET=9.700261907071492e-05;FREQ_HOMALT=0.0;FREQ_HOMREF=0.9999029973809292;N_BI_GENOS=10309;South_Han_AN=1496;South_Han_AC=1;South_Han_
chr2	203675351	.	A	<INS:ME:LINE1>	.	PASS	SVTYPE=INS:ME:LINE1;END=203675367;SVLEN=6019;AN=20618;AC=1;AF=4.850130953535746e-05;N_HET=1;N_HOMALT=0;N_HOMREF=10308;FREQ_HET=9.700261907071492e-05;FREQ_HOMALT=0.0;FREQ_HOMREF=0.9999029973809292;N_BI_GENOS=10309;Southeast_Han_AN=1908;Southeast_Han_AC=1;S
chr2	203855178	.	T	<INS:ME:LINE1>	.	PASS	SVTYPE=INS:ME:LINE1;END=203855191;SVLEN=2955;AN=20618;AC=1;AF=4.850130953535746e-05;N_HET=1;N_HOMALT=0;N_HOMREF=10308;FREQ_HET=9.700261907071492e-05;FREQ_HOMALT=0.0;FREQ_HOMREF=0.9999029973809292;N_BI_GENOS=10309;Southeast_Han_AN=1908;Southeast_Han_AC=1;S
chr2	204786220	.	A	<INS:ME:LINE1>	.	PASS	SVTYPE=INS:ME:LINE1;END=204786236;SVLEN=2134;AN=20618;AC=1;AF=4.850130953535746e-05;N_HET=1;N_HOMALT=0;N_HOMREF=10308;FREQ_HET=9.700261907071492e-05;FREQ_HOMALT=0.0;FREQ_HOMREF=0.9999029973809292;N_BI_GENOS=10309;Lingnan_Han_AN=1888;Lingnan_Han_AC=1;Lingn
chr2	205009194	.	A	<INS:ME:LINE1>	.	PASS	SVTYPE=INS:ME:LINE1;END=205009211;SVLEN=1660;AN=20618;AC=1;AF=4.850130953535746e-05;N_HET=1;N_HOMALT=0;N_HOMREF=10308;FREQ_HET=9.700261907071492e-05;FREQ_HOMALT=0.0;FREQ_HOMREF=0.9999029973809292;N_BI_GENOS=10309;East_Han_AN=6554;East_Han_AC=1;East_Han_AF
chr2	205027242	.	T	<INS:ME:LINE1>	.	PASS	SVTYPE=INS:ME:LINE1;END=205027242;SVLEN=397;AN=20618;AC=1;AF=4.850130953535746e-05;N_HET=1;N_HOMALT=0;N_HOMREF=10308;FREQ_HET=9.700261907071492e-05;FREQ_HOMALT=0.0;FREQ_HOMREF=0.9999029973809292;N_BI_GENOS=10309;East_Han_AN=6554;East_Han_AC=1;East_Han_AF=
chr2	205066194	.	T	<INS:ME:LINE1>	.	PASS	SVTYPE=INS:ME:LINE1;END=205066209;SVLEN=2625;AN=20618;AC=1;AF=4.850130953535746e-05;N_HET=1;N_HOMALT=0;N_HOMREF=10308;FREQ_HET=9.700261907071492e-05;FREQ_HOMALT=0.0;FREQ_HOMREF=0.9999029973809292;N_BI_GENOS=10309;North_Han_AN=2272;North_Han_AC=1;North_Han
chr2	205076053	.	G	<INS:ME:LINE1>	.	PASS	SVTYPE=INS:ME:LINE1;END=205076071;SVLEN=5999;AN=20618;AC=2;AF=9.700261907071492e-05;N_HET=2;N_HOMALT=0;N_HOMREF=10307;FREQ_HET=0.00019400523814142983;FREQ_HOMALT=0.0;FREQ_HOMREF=0.9998059947618586;N_BI_GENOS=10309;South_Han_AN=1496;South_Han_AC=1;South_Ha
chr2	205156820	.	A	<INS:ME:LINE1>	.	PASS	SVTYPE=INS:ME:LINE1;END=205156834;SVLEN=6012;AN=20618;AC=1;AF=4.850130953535746e-05;N_HET=1;N_HOMALT=0;N_HOMREF=10308;FREQ_HET=9.700261907071492e-05;FREQ_HOMALT=0.0;FREQ_HOMREF=0.9999029973809292;N_BI_GENOS=10309;Mongolian_AN=454;Mongolian_AC=1;Mongolian_
chr2	205193381	.	A	<INS:ME:LINE1>	.	PASS	SVTYPE=INS:ME:LINE1;END=205193381;SVLEN=143;AN=20618;AC=1;AF=4.850130953535746e-05;N_HET=1;N_HOMALT=0;N_HOMREF=10308;FREQ_HET=9.700261907071492e-05;FREQ_HOMALT=0.0;FREQ_HOMREF=0.9999029973809292;N_BI_GENOS=10309;Central_Han_AN=2128;Central_Han_AC=1;Centra
chr2	205282542	.	A	<INS:ME:LINE1>	.	PASS	SVTYPE=INS:ME:LINE1;END=205282558;SVLEN=232;AN=20618;AC=433;AF=0.02100106702880978;N_HET=383;N_HOMALT=25;N_HOMREF=9901;FREQ_HET=0.03715200310408381;FREQ_HOMALT=0.002425065476767727;FREQ_HOMREF=0.9604229314191484;N_BI_GENOS=10309;South_Han_AN=1496;South_H
chr2	205356890	.	A	<INS:ME:LINE1>	.	PASS	SVTYPE=INS:ME:LINE1;END=205356906;SVLEN=6000;AN=20618;AC=1;AF=4.850130953535746e-05;N_HET=1;N_HOMALT=0;N_HOMREF=10308;FREQ_HET=9.700261907071492e-05;FREQ_HOMALT=0.0;FREQ_HOMREF=0.9999029973809292;N_BI_GENOS=10309;Central_Han_AN=2128;Central_Han_AC=1;Centr
chr2	205702149	.	T	<INS:ME:LINE1>	.	PASS	SVTYPE=INS:ME:LINE1;END=205702165;SVLEN=248;AN=20618;AC=162;AF=0.007857212144727907;N_HET=160;N_HOMALT=1;N_HOMREF=10148;FREQ_HET=0.015520419051314386;FREQ_HOMALT=9.700261907071492e-05;FREQ_HOMREF=0.984382578329615;N_BI_GENOS=10309;South_Han_AN=1496;South_
chr2	206549489	.	A	<INS:ME:LINE1>	.	PASS	SVTYPE=INS:ME:LINE1;END=206549502;SVLEN=393;AN=20618;AC=1;AF=4.850130953535746e-05;N_HET=1;N_HOMALT=0;N_HOMREF=10308;FREQ_HET=9.700261907071492e-05;FREQ_HOMALT=0.0;FREQ_HOMREF=0.9999029973809292;N_BI_GENOS=10309;North_Han_AN=2272;North_Han_AC=1;North_Han_
chr2	207337694	.	A	<INS:ME:LINE1>	.	PASS	SVTYPE=INS:ME:LINE1;END=207337697;SVLEN=3161;AN=20618;AC=1;AF=4.850130953535746e-05;N_HET=1;N_HOMALT=0;N_HOMREF=10308;FREQ_HET=9.700261907071492e-05;FREQ_HOMALT=0.0;FREQ_HOMREF=0.9999029973809292;N_BI_GENOS=10309;Manchu_AN=416;Manchu_AC=1;Manchu_AF=0.0024
chr2	207354195	.	A	<INS:ME:LINE1>	.	PASS	SVTYPE=INS:ME:LINE1;END=207354211;SVLEN=893;AN=20618;AC=2;AF=9.700261907071492e-05;N_HET=2;N_HOMALT=0;N_HOMREF=10307;FREQ_HET=0.00019400523814142983;FREQ_HOMALT=0.0;FREQ_HOMREF=0.9998059947618586;N_BI_GENOS=10309;South_Han_AN=1496;South_Han_AC=1;South_Han
chr2	208012837	.	A	<INS:ME:LINE1>	.	PASS	SVTYPE=INS:ME:LINE1;END=208012854;SVLEN=2046;AN=20618;AC=2;AF=9.700261907071492e-05;N_HET=2;N_HOMALT=0;N_HOMREF=10307;FREQ_HET=0.00019400523814142983;FREQ_HOMALT=0.0;FREQ_HOMREF=0.9998059947618586;N_BI_GENOS=10309;North_Han_AN=2272;North_Han_AC=1;North_Ha
chr2	208416436	.	A	<INS:ME:LINE1>	.	PASS	SVTYPE=INS:ME:LINE1;END=208416453;SVLEN=591;AN=20618;AC=1;AF=4.850130953535746e-05;N_HET=1;N_HOMALT=0;N_HOMREF=10308;FREQ_HET=9.700261907071492e-05;FREQ_HOMALT=0.0;FREQ_HOMREF=0.9999029973809292;N_BI_GENOS=10309;Northwest_Han_AN=1296;Northwest_Han_AC=1;No
chr2	208797961	.	A	<INS:ME:LINE1>	.	PASS	SVTYPE=INS:ME:LINE1;END=208797976;SVLEN=6009;AN=20618;AC=2;AF=9.700261907071492e-05;N_HET=2;N_HOMALT=0;N_HOMREF=10307;FREQ_HET=0.00019400523814142983;FREQ_HOMALT=0.0;FREQ_HOMREF=0.9998059947618586;N_BI_GENOS=10309;Southeast_Han_AN=1908;Southeast_Han_AC=1;
chr2	209055261	.	A	<INS:ME:LINE1>	.	PASS	SVTYPE=INS:ME:LINE1;END=209055261;SVLEN=446;AN=20618;AC=1;AF=4.850130953535746e-05;N_HET=1;N_HOMALT=0;N_HOMREF=10308;FREQ_HET=9.700261907071492e-05;FREQ_HOMALT=0.0;FREQ_HOMREF=0.9999029973809292;N_BI_GENOS=10309;East_Han_AN=6554;East_Han_AC=1;East_Han_AF=
chr2	209156937	.	A	<INS:ME:LINE1>	.	PASS	SVTYPE=INS:ME:LINE1;END=209156951;SVLEN=1106;AN=20618;AC=3;AF=0.00014550392860607236;N_HET=3;N_HOMALT=0;N_HOMREF=10306;FREQ_HET=0.0002910078572121447;FREQ_HOMALT=0.0;FREQ_HOMREF=0.9997089921427879;N_BI_GENOS=10309;Tibetan_AN=414;Tibetan_AC=3;Tibetan_AF=0.
chr2	209182533	.	T	<INS:ME:LINE1>	.	PASS	SVTYPE=INS:ME:LINE1;END=209182551;SVLEN=3424;AN=20618;AC=1;AF=4.850130953535746e-05;N_HET=1;N_HOMALT=0;N_HOMREF=10308;FREQ_HET=9.700261907071492e-05;FREQ_HOMALT=0.0;FREQ_HOMREF=0.9999029973809292;N_BI_GENOS=10309;East_Han_AN=6554;East_Han_AC=1;East_Han_AF
chr2	209463357	.	G	<INS:ME:LINE1>	.	PASS	SVTYPE=INS:ME:LINE1;END=209463365;SVLEN=591;AN=20618;AC=1;AF=4.850130953535746e-05;N_HET=1;N_HOMALT=0;N_HOMREF=10308;FREQ_HET=9.700261907071492e-05;FREQ_HOMALT=0.0;FREQ_HOMREF=0.9999029973809292;N_BI_GENOS=10309;Lingnan_Han_AN=1888;Lingnan_Han_AC=1;Lingna
chr2	209677626	.	A	<INS:ME:LINE1>	.	PASS	SVTYPE=INS:ME:LINE1;END=209677636;SVLEN=5998;AN=20618;AC=1;AF=4.850130953535746e-05;N_HET=1;N_HOMALT=0;N_HOMREF=10308;FREQ_HET=9.700261907071492e-05;FREQ_HOMALT=0.0;FREQ_HOMREF=0.9999029973809292;N_BI_GENOS=10309;East_Han_AN=6554;East_Han_AC=1;East_Han_AF
chr2	209759644	.	T	<INS:ME:LINE1>	.	PASS	SVTYPE=INS:ME:LINE1;END=209759644;SVLEN=1664;AN=20618;AC=1;AF=4.850130953535746e-05;N_HET=1;N_HOMALT=0;N_HOMREF=10308;FREQ_HET=9.700261907071492e-05;FREQ_HOMALT=0.0;FREQ_HOMREF=0.9999029973809292;N_BI_GENOS=10309;East_Han_AN=6554;East_Han_AC=1;East_Han_AF
chr2	209760089	.	T	<INS:ME:LINE1>	.	PASS	SVTYPE=INS:ME:LINE1;END=209760136;SVLEN=1071;AN=20618;AC=1;AF=4.850130953535746e-05;N_HET=1;N_HOMALT=0;N_HOMREF=10308;FREQ_HET=9.700261907071492e-05;FREQ_HOMALT=0.0;FREQ_HOMREF=0.9999029973809292;N_BI_GENOS=10309;East_Han_AN=6554;East_Han_AC=1;East_Han_AF
chr2	210577681	.	A	<INS:ME:LINE1>	.	PASS	SVTYPE=INS:ME:LINE1;END=210577695;SVLEN=6019;AN=20618;AC=3;AF=0.00014550392860607236;N_HET=3;N_HOMALT=0;N_HOMREF=10306;FREQ_HET=0.0002910078572121447;FREQ_HOMALT=0.0;FREQ_HOMREF=0.9997089921427879;N_BI_GENOS=10309;South_Han_AN=1496;South_Han_AC=2;South_Ha
chr2	210619098	.	G	<INS:ME:LINE1>	.	PASS	SVTYPE=INS:ME:LINE1;END=210619107;SVLEN=507;AN=20618;AC=3;AF=0.00014550392860607236;N_HET=3;N_HOMALT=0;N_HOMREF=10306;FREQ_HET=0.0002910078572121447;FREQ_HOMALT=0.0;FREQ_HOMREF=0.9997089921427879;N_BI_GENOS=10309;Lingnan_Han_AN=1888;Lingnan_Han_AC=3;Lingn
chr2	210717695	.	G	<INS:ME:LINE1>	.	PASS	SVTYPE=INS:ME:LINE1;END=210717704;SVLEN=169;AN=20618;AC=2;AF=9.700261907071492e-05;N_HET=2;N_HOMALT=0;N_HOMREF=10307;FREQ_HET=0.00019400523814142983;FREQ_HOMALT=0.0;FREQ_HOMREF=0.9998059947618586;N_BI_GENOS=10309;East_Han_AN=6554;East_Han_AC=2;East_Han_AF
chr2	210752053	.	G	<INS:ME:LINE1>	.	PASS	SVTYPE=INS:ME:LINE1;END=210752069;SVLEN=2417;AN=20618;AC=3;AF=0.00014550392860607236;N_HET=3;N_HOMALT=0;N_HOMREF=10306;FREQ_HET=0.0002910078572121447;FREQ_HOMALT=0.0;FREQ_HOMREF=0.9997089921427879;N_BI_GENOS=10309;Northwest_Han_AN=1296;Northwest_Han_AC=1;
chr2	210941875	.	A	<INS:ME:LINE1>	.	PASS	SVTYPE=INS:ME:LINE1;END=210941890;SVLEN=1551;AN=20618;AC=1;AF=4.850130953535746e-05;N_HET=1;N_HOMALT=0;N_HOMREF=10308;FREQ_HET=9.700261907071492e-05;FREQ_HOMALT=0.0;FREQ_HOMREF=0.9999029973809292;N_BI_GENOS=10309;East_Han_AN=6554;East_Han_AC=1;East_Han_AF
chr2	211232798	.	C	<INS:ME:LINE1>	.	PASS	SVTYPE=INS:ME:LINE1;END=211232798;SVLEN=2537;AN=20618;AC=1;AF=4.850130953535746e-05;N_HET=1;N_HOMALT=0;N_HOMREF=10308;FREQ_HET=9.700261907071492e-05;FREQ_HOMALT=0.0;FREQ_HOMREF=0.9999029973809292;N_BI_GENOS=10309;East_Han_AN=6554;East_Han_AC=1;East_Han_AF
chr2	211554958	.	A	<INS:ME:LINE1>	.	PASS	SVTYPE=INS:ME:LINE1;END=211554974;SVLEN=5999;AN=20618;AC=1;AF=4.850130953535746e-05;N_HET=1;N_HOMALT=0;N_HOMREF=10308;FREQ_HET=9.700261907071492e-05;FREQ_HOMALT=0.0;FREQ_HOMREF=0.9999029973809292;N_BI_GENOS=10309;Zhuang_AN=444;Zhuang_AC=1;Zhuang_AF=0.0022
chr2	211825952	.	A	<INS:ME:LINE1>	.	PASS	SVTYPE=INS:ME:LINE1;END=211825957;SVLEN=991;AN=20618;AC=1;AF=4.850130953535746e-05;N_HET=1;N_HOMALT=0;N_HOMREF=10308;FREQ_HET=9.700261907071492e-05;FREQ_HOMALT=0.0;FREQ_HOMREF=0.9999029973809292;N_BI_GENOS=10309;Southeast_Han_AN=1908;Southeast_Han_AC=1;So
chr2	211842455	.	A	<INS:ME:LINE1>	.	PASS	SVTYPE=INS:ME:LINE1;END=211842472;SVLEN=1008;AN=20618;AC=35;AF=0.0016975458337375108;N_HET=31;N_HOMALT=2;N_HOMREF=10276;FREQ_HET=0.0030070811911921624;FREQ_HOMALT=0.00019400523814142983;FREQ_HOMREF=0.9967989135706664;N_BI_GENOS=10309;Northwest_Han_AN=1296
chr2	212249437	.	A	<INS:ME:LINE1>	.	PASS	SVTYPE=INS:ME:LINE1;END=212249449;SVLEN=10;AN=20618;AC=2;AF=9.700261907071492e-05;N_HET=2;N_HOMALT=0;N_HOMREF=10307;FREQ_HET=0.00019400523814142983;FREQ_HOMALT=0.0;FREQ_HOMREF=0.9998059947618586;N_BI_GENOS=10309;South_Han_AN=1496;South_Han_AC=1;South_Han_
chr2	212315481	.	A	<INS:ME:LINE1>	.	PASS	SVTYPE=INS:ME:LINE1;END=212315492;SVLEN=2159;AN=20618;AC=3;AF=0.00014550392860607236;N_HET=3;N_HOMALT=0;N_HOMREF=10306;FREQ_HET=0.0002910078572121447;FREQ_HOMALT=0.0;FREQ_HOMREF=0.9997089921427879;N_BI_GENOS=10309;North_Han_AN=2272;North_Han_AC=1;North_Ha
chr2	212379548	.	A	<INS:ME:LINE1>	.	PASS	SVTYPE=INS:ME:LINE1;END=212379548;SVLEN=131;AN=20618;AC=1;AF=4.850130953535746e-05;N_HET=1;N_HOMALT=0;N_HOMREF=10308;FREQ_HET=9.700261907071492e-05;FREQ_HOMALT=0.0;FREQ_HOMREF=0.9999029973809292;N_BI_GENOS=10309;Mongolian_AN=454;Mongolian_AC=1;Mongolian_A
chr2	212415744	.	A	<INS:ME:LINE1>	.	PASS	SVTYPE=INS:ME:LINE1;END=212415744;SVLEN=1175;AN=20618;AC=1;AF=4.850130953535746e-05;N_HET=1;N_HOMALT=0;N_HOMREF=10308;FREQ_HET=9.700261907071492e-05;FREQ_HOMALT=0.0;FREQ_HOMREF=0.9999029973809292;N_BI_GENOS=10309;Southeast_Han_AN=1908;Southeast_Han_AC=1;S
chr2	212553194	.	C	<INS:ME:LINE1>	.	PASS	SVTYPE=INS:ME:LINE1;END=212553199;SVLEN=1093;AN=20618;AC=1;AF=4.850130953535746e-05;N_HET=1;N_HOMALT=0;N_HOMREF=10308;FREQ_HET=9.700261907071492e-05;FREQ_HOMALT=0.0;FREQ_HOMREF=0.9999029973809292;N_BI_GENOS=10309;East_Han_AN=6554;East_Han_AC=1;East_Han_AF
chr2	212613481	.	T	<INS:ME:LINE1>	.	PASS	SVTYPE=INS:ME:LINE1;END=212613481;SVLEN=681;AN=20618;AC=1;AF=4.850130953535746e-05;N_HET=1;N_HOMALT=0;N_HOMREF=10308;FREQ_HET=9.700261907071492e-05;FREQ_HOMALT=0.0;FREQ_HOMREF=0.9999029973809292;N_BI_GENOS=10309;East_Han_AN=6554;East_Han_AC=1;East_Han_AF=
chr2	212693348	.	C	<INS:ME:LINE1>	.	PASS	SVTYPE=INS:ME:LINE1;END=212693362;SVLEN=774;AN=20618;AC=1092;AF=0.05296343001261034;N_HET=980;N_HOMALT=56;N_HOMREF=9273;FREQ_HET=0.09506256668930062;FREQ_HOMALT=0.005432146667960035;FREQ_HOMREF=0.8995052866427393;N_BI_GENOS=10309;South_Han_AN=1496;South_H
chr2	212803870	.	A	<INS:ME:LINE1>	.	PASS	SVTYPE=INS:ME:LINE1;END=212803886;SVLEN=1186;AN=20618;AC=3;AF=0.00014550392860607236;N_HET=3;N_HOMALT=0;N_HOMREF=10306;FREQ_HET=0.0002910078572121447;FREQ_HOMALT=0.0;FREQ_HOMREF=0.9997089921427879;N_BI_GENOS=10309;South_Han_AN=1496;South_Han_AC=2;South_Ha
chr2	212839028	.	C	<INS:ME:LINE1>	.	PASS	SVTYPE=INS:ME:LINE1;END=212839045;SVLEN=5999;AN=20618;AC=4;AF=0.00019400523814142983;N_HET=4;N_HOMALT=0;N_HOMREF=10305;FREQ_HET=0.00038801047628285966;FREQ_HOMALT=0.0;FREQ_HOMREF=0.9996119895237171;N_BI_GENOS=10309;South_Han_AN=1496;South_Han_AC=1;South_H
chr2	213058649	.	A	<INS:ME:LINE1>	.	PASS	SVTYPE=INS:ME:LINE1;END=213058650;SVLEN=309;AN=20618;AC=2;AF=9.700261907071492e-05;N_HET=2;N_HOMALT=0;N_HOMREF=10307;FREQ_HET=0.00019400523814142983;FREQ_HOMALT=0.0;FREQ_HOMREF=0.9998059947618586;N_BI_GENOS=10309;South_Han_AN=1496;South_Han_AC=1;South_Han
chr2	213099387	.	T	<INS:ME:LINE1>	.	PASS	SVTYPE=INS:ME:LINE1;END=213099395;SVLEN=407;AN=20618;AC=1;AF=4.850130953535746e-05;N_HET=1;N_HOMALT=0;N_HOMREF=10308;FREQ_HET=9.700261907071492e-05;FREQ_HOMALT=0.0;FREQ_HOMREF=0.9999029973809292;N_BI_GENOS=10309;Central_Han_AN=2128;Central_Han_AC=1;Centra
chr2	213173118	.	A	<INS:ME:LINE1>	.	PASS	SVTYPE=INS:ME:LINE1;END=213173132;SVLEN=1366;AN=20618;AC=1;AF=4.850130953535746e-05;N_HET=1;N_HOMALT=0;N_HOMREF=10308;FREQ_HET=9.700261907071492e-05;FREQ_HOMALT=0.0;FREQ_HOMREF=0.9999029973809292;N_BI_GENOS=10309;Central_Han_AN=2128;Central_Han_AC=1;Centr
chr2	213288827	.	C	<INS:ME:LINE1>	.	PASS	SVTYPE=INS:ME:LINE1;END=213288845;SVLEN=5989;AN=20618;AC=2;AF=9.700261907071492e-05;N_HET=2;N_HOMALT=0;N_HOMREF=10307;FREQ_HET=0.00019400523814142983;FREQ_HOMALT=0.0;FREQ_HOMREF=0.9998059947618586;N_BI_GENOS=10309;East_Han_AN=6554;East_Han_AC=1;East_Han_A
chr2	213327251	.	T	<INS:ME:LINE1>	.	PASS	SVTYPE=INS:ME:LINE1;END=213327266;SVLEN=6000;AN=20618;AC=3;AF=0.00014550392860607236;N_HET=3;N_HOMALT=0;N_HOMREF=10306;FREQ_HET=0.0002910078572121447;FREQ_HOMALT=0.0;FREQ_HOMREF=0.9997089921427879;N_BI_GENOS=10309;Central_Han_AN=2128;Central_Han_AC=1;Cent
chr2	213412499	.	A	<INS:ME:LINE1>	.	PASS	SVTYPE=INS:ME:LINE1;END=213412506;SVLEN=5998;AN=20618;AC=25;AF=0.0012125327383839363;N_HET=25;N_HOMALT=0;N_HOMREF=10284;FREQ_HET=0.0024250654767678727;FREQ_HOMALT=0.0;FREQ_HOMREF=0.9975749345232321;N_BI_GENOS=10309;South_Han_AN=1496;South_Han_AC=2;South_H

chr2	213791810	.	G	<INS:ME:LINE1>	.	PASS	SVTYPE=INS:ME:LINE1;END=213791811;SVLEN=306;AN=20618;AC=1;AF=4.850130953535746e-05;N_HET=1;N_HOMALT=0;N_HOMREF=10308;FREQ_HET=9.700261907071492e-05;FREQ_HOMALT=0.0;FREQ_HOMREF=0.9999029973809292;N_BI_GENOS=10309;Lingnan_Han_AN=1888;Lingnan_Han_AC=1;Lingna
chr2	213852331	.	G	<INS:ME:LINE1>	.	PASS	SVTYPE=INS:ME:LINE1;END=213852342;SVLEN=751;AN=20618;AC=26;AF=0.0012610340479192938;N_HET=26;N_HOMALT=0;N_HOMREF=10283;FREQ_HET=0.0025220680958385876;FREQ_HOMALT=0.0;FREQ_HOMREF=0.9974779319041615;N_BI_GENOS=10309;South_Han_AN=1496;South_Han_AC=1;South_Ha
chr2	214089483	.	G	<INS:ME:LINE1>	.	PASS	SVTYPE=INS:ME:LINE1;END=214089496;SVLEN=5996;AN=20618;AC=1;AF=4.850130953535746e-05;N_HET=1;N_HOMALT=0;N_HOMREF=10308;FREQ_HET=9.700261907071492e-05;FREQ_HOMALT=0.0;FREQ_HOMREF=0.9999029973809292;N_BI_GENOS=10309;Central_Han_AN=2128;Central_Han_AC=1;Centr
chr2	214104681	.	A	<INS:ME:LINE1>	.	PASS	SVTYPE=INS:ME:LINE1;END=214104696;SVLEN=1573;AN=20618;AC=9;AF=0.0004365117858182171;N_HET=9;N_HOMALT=0;N_HOMREF=10300;FREQ_HET=0.0008730235716364342;FREQ_HOMALT=0.0;FREQ_HOMREF=0.9991269764283636;N_BI_GENOS=10309;South_Han_AN=1496;South_Han_AC=1;South_Han
chr2	214280337	.	T	<INS:ME:LINE1>	.	PASS	SVTYPE=INS:ME:LINE1;END=214280337;SVLEN=3313;AN=20618;AC=1;AF=4.850130953535746e-05;N_HET=1;N_HOMALT=0;N_HOMREF=10308;FREQ_HET=9.700261907071492e-05;FREQ_HOMALT=0.0;FREQ_HOMREF=0.9999029973809292;N_BI_GENOS=10309;Southeast_Han_AN=1908;Southeast_Han_AC=1;S
chr2	214319018	.	A	<INS:ME:LINE1>	.	PASS	SVTYPE=INS:ME:LINE1;END=214319032;SVLEN=1957;AN=20618;AC=1;AF=4.850130953535746e-05;N_HET=1;N_HOMALT=0;N_HOMREF=10308;FREQ_HET=9.700261907071492e-05;FREQ_HOMALT=0.0;FREQ_HOMREF=0.9999029973809292;N_BI_GENOS=10309;North_Han_AN=2272;North_Han_AC=1;North_Han
chr2	214339521	.	T	<INS:ME:LINE1>	.	PASS	SVTYPE=INS:ME:LINE1;END=214339526;SVLEN=1544;AN=20618;AC=1;AF=4.850130953535746e-05;N_HET=1;N_HOMALT=0;N_HOMREF=10308;FREQ_HET=9.700261907071492e-05;FREQ_HOMALT=0.0;FREQ_HOMREF=0.9999029973809292;N_BI_GENOS=10309;Miao_AN=482;Miao_AC=1;Miao_AF=0.0020746887
chr2	214487618	.	A	<INS:ME:LINE1>	.	PASS	SVTYPE=INS:ME:LINE1;END=214487621;SVLEN=353;AN=20618;AC=42;AF=0.002037055000485013;N_HET=38;N_HOMALT=2;N_HOMREF=10269;FREQ_HET=0.0036860995246871665;FREQ_HOMALT=0.00019400523814142983;FREQ_HOMREF=0.9961198952371714;N_BI_GENOS=10309;South_Han_AN=1496;South
chr2	214575493	.	A	<INS:ME:LINE1>	.	PASS	SVTYPE=INS:ME:LINE1;END=214575510;SVLEN=725;AN=20618;AC=1;AF=4.850130953535746e-05;N_HET=1;N_HOMALT=0;N_HOMREF=10308;FREQ_HET=9.700261907071492e-05;FREQ_HOMALT=0.0;FREQ_HOMREF=0.9999029973809292;N_BI_GENOS=10309;Central_Han_AN=2128;Central_Han_AC=1;Centra
chr2	214579487	.	A	<INS:ME:LINE1>	.	PASS	SVTYPE=INS:ME:LINE1;END=214579507;SVLEN=1411;AN=20618;AC=1;AF=4.850130953535746e-05;N_HET=1;N_HOMALT=0;N_HOMREF=10308;FREQ_HET=9.700261907071492e-05;FREQ_HOMALT=0.0;FREQ_HOMREF=0.9999029973809292;N_BI_GENOS=10309;Central_Han_AN=2128;Central_Han_AC=1;Centr
chr2	214611860	.	A	<INS:ME:LINE1>	.	PASS	SVTYPE=INS:ME:LINE1;END=214611872;SVLEN=754;AN=20618;AC=1;AF=4.850130953535746e-05;N_HET=1;N_HOMALT=0;N_HOMREF=10308;FREQ_HET=9.700261907071492e-05;FREQ_HOMALT=0.0;FREQ_HOMREF=0.9999029973809292;N_BI_GENOS=10309;North_Han_AN=2272;North_Han_AC=1;North_Han_
chr2	214661286	.	G	<INS:ME:LINE1>	.	PASS	SVTYPE=INS:ME:LINE1;END=214661286;SVLEN=1;AN=20618;AC=1;AF=4.850130953535746e-05;N_HET=1;N_HOMALT=0;N_HOMREF=10308;FREQ_HET=9.700261907071492e-05;FREQ_HOMALT=0.0;FREQ_HOMREF=0.9999029973809292;N_BI_GENOS=10309;East_Han_AN=6554;East_Han_AC=1;East_Han_AF=0.
chr2	214662843	.	A	<INS:ME:LINE1>	.	PASS	SVTYPE=INS:ME:LINE1;END=214662860;SVLEN=184;AN=20618;AC=1;AF=4.850130953535746e-05;N_HET=1;N_HOMALT=0;N_HOMREF=10308;FREQ_HET=9.700261907071492e-05;FREQ_HOMALT=0.0;FREQ_HOMREF=0.9999029973809292;N_BI_GENOS=10309;North_Han_AN=2272;North_Han_AC=1;North_Han_
chr2	214704452	.	T	<INS:ME:LINE1>	.	PASS	SVTYPE=INS:ME:LINE1;END=214704452;SVLEN=1868;AN=20618;AC=1;AF=4.850130953535746e-05;N_HET=1;N_HOMALT=0;N_HOMREF=10308;FREQ_HET=9.700261907071492e-05;FREQ_HOMALT=0.0;FREQ_HOMREF=0.9999029973809292;N_BI_GENOS=10309;East_Han_AN=6554;East_Han_AC=1;East_Han_AF
chr2	214916824	.	T	<INS:ME:LINE1>	.	PASS	SVTYPE=INS:ME:LINE1;END=214916825;SVLEN=1212;AN=20618;AC=1;AF=4.850130953535746e-05;N_HET=1;N_HOMALT=0;N_HOMREF=10308;FREQ_HET=9.700261907071492e-05;FREQ_HOMALT=0.0;FREQ_HOMREF=0.9999029973809292;N_BI_GENOS=10309;South_Han_AN=1496;South_Han_AC=1;South_Han
chr2	215052202	.	T	<INS:ME:LINE1>	.	PASS	SVTYPE=INS:ME:LINE1;END=215052213;SVLEN=6151;AN=20618;AC=1;AF=4.850130953535746e-05;N_HET=1;N_HOMALT=0;N_HOMREF=10308;FREQ_HET=9.700261907071492e-05;FREQ_HOMALT=0.0;FREQ_HOMREF=0.9999029973809292;N_BI_GENOS=10309;East_Han_AN=6554;East_Han_AC=1;East_Han_AF
chr2	215102813	.	A	<INS:ME:LINE1>	.	PASS	SVTYPE=INS:ME:LINE1;END=215102827;SVLEN=3311;AN=20618;AC=2;AF=9.700261907071492e-05;N_HET=2;N_HOMALT=0;N_HOMREF=10307;FREQ_HET=0.00019400523814142983;FREQ_HOMALT=0.0;FREQ_HOMREF=0.9998059947618586;N_BI_GENOS=10309;North_Han_AN=2272;North_Han_AC=1;North_Ha
chr2	215136397	.	A	<INS:ME:LINE1>	.	PASS	SVTYPE=INS:ME:LINE1;END=215136408;SVLEN=381;AN=20618;AC=5;AF=0.00024250654767678728;N_HET=5;N_HOMALT=0;N_HOMREF=10304;FREQ_HET=0.0004850130953535745;FREQ_HOMALT=0.0;FREQ_HOMREF=0.9995149869046465;N_BI_GENOS=10309;Lingnan_Han_AN=1888;Lingnan_Han_AC=1;Ling
chr2	215236886	.	A	<INS:ME:LINE1>	.	PASS	SVTYPE=INS:ME:LINE1;END=215236899;SVLEN=6000;AN=20618;AC=58;AF=0.0028130759530507325;N_HET=56;N_HOMALT=1;N_HOMREF=10252;FREQ_HET=0.005432146667960035;FREQ_HOMALT=9.700261907071492e-05;FREQ_HOMREF=0.9944708507129693;N_BI_GENOS=10309;South_Han_AN=1496;South
chr2	215586522	.	C	<INS:ME:LINE1>	.	PASS	SVTYPE=INS:ME:LINE1;END=215586522;SVLEN=412;AN=20618;AC=1;AF=4.850130953535746e-05;N_HET=1;N_HOMALT=0;N_HOMREF=10308;FREQ_HET=9.700261907071492e-05;FREQ_HOMALT=0.0;FREQ_HOMREF=0.9999029973809292;N_BI_GENOS=10309;Central_Han_AN=2128;Central_Han_AC=1;Centra
chr2	216846592	.	A	<INS:ME:LINE1>	.	PASS	SVTYPE=INS:ME:LINE1;END=216846609;SVLEN=1079;AN=20618;AC=1;AF=4.850130953535746e-05;N_HET=1;N_HOMALT=0;N_HOMREF=10308;FREQ_HET=9.700261907071492e-05;FREQ_HOMALT=0.0;FREQ_HOMREF=0.9999029973809292;N_BI_GENOS=10309;Central_Han_AN=2128;Central_Han_AC=1;Centr
chr2	216994063	.	C	<INS:ME:LINE1>	.	PASS	SVTYPE=INS:ME:LINE1;END=216994074;SVLEN=6010;AN=20618;AC=1;AF=4.850130953535746e-05;N_HET=1;N_HOMALT=0;N_HOMREF=10308;FREQ_HET=9.700261907071492e-05;FREQ_HOMALT=0.0;FREQ_HOMREF=0.9999029973809292;N_BI_GENOS=10309;Southeast_Han_AN=1908;Southeast_Han_AC=1;S
chr2	217020848	.	A	<INS:ME:LINE1>	.	PASS	SVTYPE=INS:ME:LINE1;END=217020863;SVLEN=4179;AN=20618;AC=19;AF=0.0009215248811717916;N_HET=19;N_HOMALT=0;N_HOMREF=10290;FREQ_HET=0.0018430497623435833;FREQ_HOMALT=0.0;FREQ_HOMREF=0.9981569502376564;N_BI_GENOS=10309;South_Han_AN=1496;South_Han_AC=3;South_H
chr2	217312605	.	T	<INS:ME:LINE1>	.	PASS	SVTYPE=INS:ME:LINE1;END=217312605;SVLEN=324;AN=20618;AC=1;AF=4.850130953535746e-05;N_HET=1;N_HOMALT=0;N_HOMREF=10308;FREQ_HET=9.700261907071492e-05;FREQ_HOMALT=0.0;FREQ_HOMREF=0.9999029973809292;N_BI_GENOS=10309;South_Han_AN=1496;South_Han_AC=1;South_Han_
chr2	217318157	.	A	<INS:ME:LINE1>	.	PASS	SVTYPE=INS:ME:LINE1;END=217318157;SVLEN=265;AN=20618;AC=1;AF=4.850130953535746e-05;N_HET=1;N_HOMALT=0;N_HOMREF=10308;FREQ_HET=9.700261907071492e-05;FREQ_HOMALT=0.0;FREQ_HOMREF=0.9999029973809292;N_BI_GENOS=10309;Southeast_Han_AN=1908;Southeast_Han_AC=1;So
chr2	217644904	.	A	<INS:ME:LINE1>	.	PASS	SVTYPE=INS:ME:LINE1;END=217644913;SVLEN=502;AN=20618;AC=2;AF=9.700261907071492e-05;N_HET=0;N_HOMALT=1;N_HOMREF=10308;FREQ_HET=0.0;FREQ_HOMALT=9.700261907071492e-05;FREQ_HOMREF=0.9999029973809292;N_BI_GENOS=10309;East_Han_AN=6554;East_Han_AC=2;East_Han_AF=
chr2	218845895	.	C	<INS:ME:LINE1>	.	PASS	SVTYPE=INS:ME:LINE1;END=218845895;SVLEN=4049;AN=20618;AC=1;AF=4.850130953535746e-05;N_HET=1;N_HOMALT=0;N_HOMREF=10308;FREQ_HET=9.700261907071492e-05;FREQ_HOMALT=0.0;FREQ_HOMREF=0.9999029973809292;N_BI_GENOS=10309;North_Han_AN=2272;North_Han_AC=1;North_Han
chr2	219744736	.	G	<INS:ME:LINE1>	.	PASS	SVTYPE=INS:ME:LINE1;END=219744754;SVLEN=3147;AN=20618;AC=1;AF=4.850130953535746e-05;N_HET=1;N_HOMALT=0;N_HOMREF=10308;FREQ_HET=9.700261907071492e-05;FREQ_HOMALT=0.0;FREQ_HOMREF=0.9999029973809292;N_BI_GENOS=10309;Central_Han_AN=2128;Central_Han_AC=1;Centr
chr2	219792357	.	G	<INS:ME:LINE1>	.	PASS	SVTYPE=INS:ME:LINE1;END=219792369;SVLEN=6005;AN=20618;AC=1;AF=4.850130953535746e-05;N_HET=1;N_HOMALT=0;N_HOMREF=10308;FREQ_HET=9.700261907071492e-05;FREQ_HOMALT=0.0;FREQ_HOMREF=0.9999029973809292;N_BI_GENOS=10309;Southeast_Han_AN=1908;Southeast_Han_AC=1;S
chr2	219958414	.	C	<INS:ME:LINE1>	.	PASS	SVTYPE=INS:ME:LINE1;END=219958432;SVLEN=6005;AN=20618;AC=1;AF=4.850130953535746e-05;N_HET=1;N_HOMALT=0;N_HOMREF=10308;FREQ_HET=9.700261907071492e-05;FREQ_HOMALT=0.0;FREQ_HOMREF=0.9999029973809292;N_BI_GENOS=10309;Central_Han_AN=2128;Central_Han_AC=1;Centr
chr2	220202791	.	A	<INS:ME:LINE1>	.	PASS	SVTYPE=INS:ME:LINE1;END=220202807;SVLEN=1617;AN=20618;AC=1;AF=4.850130953535746e-05;N_HET=1;N_HOMALT=0;N_HOMREF=10308;FREQ_HET=9.700261907071492e-05;FREQ_HOMALT=0.0;FREQ_HOMREF=0.9999029973809292;N_BI_GENOS=10309;North_Han_AN=2272;North_Han_AC=1;North_Han
chr2	220358842	.	T	<INS:ME:LINE1>	.	PASS	SVTYPE=INS:ME:LINE1;END=220358854;SVLEN=932;AN=20618;AC=1;AF=4.850130953535746e-05;N_HET=1;N_HOMALT=0;N_HOMREF=10308;FREQ_HET=9.700261907071492e-05;FREQ_HOMALT=0.0;FREQ_HOMREF=0.9999029973809292;N_BI_GENOS=10309;East_Han_AN=6554;East_Han_AC=1;East_Han_AF=
chr2	220413501	.	A	<INS:ME:LINE1>	.	PASS	SVTYPE=INS:ME:LINE1;END=220413511;SVLEN=5997;AN=20618;AC=3481;AF=0.1688330584925793;N_HET=2221;N_HOMALT=630;N_HOMREF=7458;FREQ_HET=0.21544281695605783;FREQ_HOMALT=0.06111165001455039;FREQ_HOMREF=0.7234455330293917;N_BI_GENOS=10309;South_Han_AN=1496;South_
chr2	220533183	.	A	<INS:ME:LINE1>	.	PASS	SVTYPE=INS:ME:LINE1;END=220533196;SVLEN=1141;AN=20618;AC=10;AF=0.0004850130953535455;N_HET=10;N_HOMALT=0;N_HOMREF=10299;FREQ_HET=0.0009700261907071491;FREQ_HOMALT=0.0;FREQ_HOMREF=0.9990299738092928;N_BI_GENOS=10309;North_Han_AN=2272;North_Han_AC=3;North_
chr2	220639029	.	A	<INS:ME:LINE1>	.	PASS	SVTYPE=INS:ME:LINE1;END=220639048;SVLEN=759;AN=20618;AC=3956;AF=0.1918711805218741;N_HET=2982;N_HOMALT=487;N_HOMREF=6840;FREQ_HET=0.28926181006887186;FREQ_HOMALT=0.04724027548743816;FREQ_HOMREF=0.66349791444369;N_BI_GENOS=10309;South_Han_AN=1496;South_Han
chr2	221016362	.	A	<INS:ME:LINE1>	.	PASS	SVTYPE=INS:ME:LINE1;END=221016372;SVLEN=725;AN=20618;AC=2;AF=9.700261907071492e-05;N_HET=2;N_HOMALT=0;N_HOMREF=10307;FREQ_HET=0.00019400523814142983;FREQ_HOMALT=0.0;FREQ_HOMREF=0.9998059947618586;N_BI_GENOS=10309;East_Han_AN=6554;East_Han_AC=1;East_Han_AF
chr2	221198489	.	A	<INS:ME:LINE1>	.	PASS	SVTYPE=INS:ME:LINE1;END=221198503;SVLEN=3591;AN=20618;AC=1;AF=4.850130953535746e-05;N_HET=1;N_HOMALT=0;N_HOMREF=10308;FREQ_HET=9.700261907071492e-05;FREQ_HOMALT=0.0;FREQ_HOMREF=0.9999029973809292;N_BI_GENOS=10309;Central_Han_AN=2128;Central_Han_AC=1;Centr
chr2	221555704	.	T	<INS:ME:LINE1>	.	PASS	SVTYPE=INS:ME:LINE1;END=221555704;SVLEN=138;AN=20618;AC=1;AF=4.850130953535746e-05;N_HET=1;N_HOMALT=0;N_HOMREF=10308;FREQ_HET=9.700261907071492e-05;FREQ_HOMALT=0.0;FREQ_HOMREF=0.9999029973809292;N_BI_GENOS=10309;Southeast_Han_AN=1908;Southeast_Han_AC=1;So
chr2	221555761	.	A	<INS:ME:LINE1>	.	PASS	SVTYPE=INS:ME:LINE1;END=221555773;SVLEN=136;AN=20618;AC=12;AF=0.0005820157144242894;N_HET=12;N_HOMALT=0;N_HOMREF=10297;FREQ_HET=0.0011640314288485789;FREQ_HOMALT=0.0;FREQ_HOMREF=0.9988359685711514;N_BI_GENOS=10309;South_Han_AN=1496;South_Han_AC=1;South_Ha
chr2	222518218	.	C	<INS:ME:LINE1>	.	PASS	SVTYPE=INS:ME:LINE1;END=222518234;SVLEN=5988;AN=20618;AC=6;AF=0.0002910078572121447;N_HET=6;N_HOMALT=0;N_HOMREF=10303;FREQ_HET=0.0005820157144242894;FREQ_HOMALT=0.0;FREQ_HOMREF=0.9994179842855757;N_BI_GENOS=10309;South_Han_AN=1496;South_Han_AC=2;South_Han
chr2	223102598	.	G	<INS:ME:LINE1>	.	PASS	SVTYPE=INS:ME:LINE1;END=223102610;SVLEN=5997;AN=20618;AC=3;AF=0.0001455039286060 7236;N_HET=3;N_HOMALT=0;N_HOMREF=10306;FREQ_HET=0.0002910078572121447;FREQ_HOMALT=0.0;FREQ_HOMREF=0.9997089921427879;N_BI_GENOS=10309;Lingnan_Han_AN=1888;Lingnan_Han_AC=1;Ling
chr2	223139137	.	A	<INS:ME:LINE1>	.	PASS	SVTYPE=INS:ME:LINE1;END=223139147;SVLEN=5921;AN=20618;AC=17;AF=0.0008245222621010767;N_HET=15;N_HOMALT=1;N_HOMREF=10293;FREQ_HET=0.0014550392860607237;FREQ_HOMALT=9.700261907071492e-05;FREQ_HOMREF=0.9984479580948685;N_BI_GENOS=10309;South_Han_AN=1496;Sout
chr2	223141843	.	A	<INS:ME:LINE1>	.	PASS	SVTYPE=INS:ME:LINE1;END=223141853;SVLEN=5998;AN=20618;AC=1;AF=4.850130953535746e-05;N_HET=1;N_HOMALT=0;N_HOMREF=10308;FREQ_HET=9.700261907071492e-05;FREQ_HOMALT=0.0;FREQ_HOMREF=0.9999029973809292;N_BI_GENOS=10309;South_Han_AN=1496;South_Han_AC=1;South_Han
chr2	223202976	.	A	<INS:ME:LINE1>	.	PASS	SVTYPE=INS:ME:LINE1;END=223202991;SVLEN=227;AN=20618;AC=3;AF=0.00014550392860607236;N_HET=3;N_HOMALT=0;N_HOMREF=10306;FREQ_HET=0.0002910078572121447;FREQ_HOMALT=0.0;FREQ_HOMREF=0.9997089921427879;N_BI_GENOS=10309;South_Han_AN=1496;South_Han_AC=1;South_Han
chr2	223453704	.	A	<INS:ME:LINE1>	.	PASS	SVTYPE=INS:ME:LINE1;END=223453720;SVLEN=3064;AN=20618;AC=3;AF=0.00014550392860607236;N_HET=3;N_HOMALT=0;N_HOMREF=10306;FREQ_HET=0.0002910078572121447;FREQ_HOMALT=0.0;FREQ_HOMREF=0.9997089921427879;N_BI_GENOS=10309;East_Han_AN=6554;East_Han_AC=2;East_Han_A
chr2	223520906	.	A	<INS:ME:LINE1>	.	PASS	SVTYPE=INS:ME:LINE1;END=223520915;SVLEN=899;AN=20618;AC=9;AF=0.0004365117858182171;N_HET=9;N_HOMALT=0;N_HOMREF=10300;FREQ_HET=0.0008730235716364342;FREQ_HOMALT=0.0;FREQ_HOMREF=0.9991269764283636;N_BI_GENOS=10309;Northwest_Han_AN=1296;Northwest_Han_AC=2;No
chr2	223718829	.	G	<INS:ME:LINE1>	.	PASS	SVTYPE=INS:ME:LINE1;END=223718838;SVLEN=4356;AN=20618;AC=1;AF=4.850130953535746e-05;N_HET=1;N_HOMALT=0;N_HOMREF=10308;FREQ_HET=9.700261907071492e-05;FREQ_HOMALT=0.0;FREQ_HOMREF=0.9999029973809292;N_BI_GENOS=10309;Lingnan_Han_AN=1888;Lingnan_Han_AC=1;Lingn
chr2	224296939	.	C	<INS:ME:LINE1>	.	PASS	SVTYPE=INS:ME:LINE1;END=224296939;SVLEN=0;AN=20618;AC=2;AF=9.700261907071492e-05;N_HET=2;N_HOMALT=0;N_HOMREF=10307;FREQ_HET=0.00019400523814142983;FREQ_HOMALT=0.0;FREQ_HOMREF=0.9998059947618586;N_BI_GENOS=10309;Lingnan_Han_AN=1888;Lingnan_Han_AC=1;Lingnan
chr2	224917084	.	T	<INS:ME:LINE1>	.	PASS	SVTYPE=INS:ME:LINE1;END=224917123;SVLEN=6052;AN=20618;AC=2;AF=9.700261907071492e-05;N_HET=0;N_HOMALT=1;N_HOMREF=10308;FREQ_HET=0.0;FREQ_HOMALT=9.700261907071492e-05;FREQ_HOMREF=0.9999029973809292;N_BI_GENOS=10309;Southeast_Han_AN=1908;Southeast_Han_AC=2;S
chr2	224994709	.	T	<INS:ME:LINE1>	.	PASS	SVTYPE=INS:ME:LINE1;END=224994726;SVLEN=5998;AN=20618;AC=1;AF=4.850130953535746e-05;N_HET=1;N_HOMALT=0;N_HOMREF=10308;FREQ_HET=9.700261907071492e-05;FREQ_HOMALT=0.0;FREQ_HOMREF=0.9999029973809292;N_BI_GENOS=10309;Lingnan_Han_AN=1888;Lingnan_Han_AC=1;Lingn
chr2	225016260	.	A	<INS:ME:LINE1>	.	PASS	SVTYPE=INS:ME:LINE1;END=225016274;SVLEN=693;AN=20618;AC=1;AF=4.850130953535746e-05;N_HET=1;N_HOMALT=0;N_HOMREF=10308;FREQ_HET=9.700261907071492e-05;FREQ_HOMALT=0.0;FREQ_HOMREF=0.9999029973809292;N_BI_GENOS=10309;East_Han_AN=6554;East_Han_AC=1;East_Han_AF=
chr2	225870592	.	A	<INS:ME:LINE1>	.	PASS	SVTYPE=INS:ME:LINE1;END=225870599;SVLEN=668;AN=20618;AC=3;AF=0.00014550392860607236;N_HET=3;N_HOMALT=0;N_HOMREF=10306;FREQ_HET=0.0002910078572121447;FREQ_HOMALT=0.0;FREQ_HOMREF=0.9997089921427879;N_BI_GENOS=10309;South_Han_AN=1496;South_Han_AC=1;South_Han
chr2	226113517	.	A	<INS:ME:LINE1>	.	PASS	SVTYPE=INS:ME:LINE1;END=226113533;SVLEN=535;AN=20618;AC=1;AF=4.850130953535746e-05;N_HET=1;N_HOMALT=0;N_HOMREF=10308;FREQ_HET=9.700261907071492e-05;FREQ_HOMALT=0.0;FREQ_HOMREF=0.9999029973809292;N_BI_GENOS=10309;Southeast_Han_AN=1908;Southeast_Han_AC=1;So
chr2	226288512	.	A	<INS:ME:LINE1>	.	PASS	SVTYPE=INS:ME:LINE1;END=226288527;SVLEN=5997;AN=20618;AC=2;AF=9.700261907071492e-05;N_HET=2;N_HOMALT=0;N_HOMREF=10307;FREQ_HET=0.00019400523814142983;FREQ_HOMALT=0.0;FREQ_HOMREF=0.9998059947618586;N_BI_GENOS=10309;North_Han_AN=2272;North_Han_AC=1;North_Ha
chr2	226485969	.	T	<INS:ME:LINE1>	.	PASS	SVTYPE=INS:ME:LINE1;END=226485969;SVLEN=0;AN=20618;AC=2;AF=9.700261907071492e-05;N_HET=0;N_HOMALT=1;N_HOMREF=10308;FREQ_HET=0.0;FREQ_HOMALT=9.700261907071492e-05;FREQ_HOMREF=0.9999029973809292;N_BI_GENOS=10309;Northwest_Han_AN=1296;Northwest_Han_AC=2;Nort
chr2	226490188	.	A	<INS:ME:LINE1>	.	PASS	SVTYPE=INS:ME:LINE1;END=226490194;SVLEN=1388;AN=20618;AC=9;AF=0.0004365117858182171;N_HET=9;N_HOMALT=0;N_HOMREF=10300;FREQ_HET=0.0008730235716364342;FREQ_HOMALT=0.0;FREQ_HOMREF=0.9991269764283636;N_BI_GENOS=10309;South_Han_AN=1496;South_Han_AC=4;South_Han
chr2	226712611	.	A	<INS:ME:LINE1>	.	PASS	SVTYPE=INS:ME:LINE1;END=226712627;SVLEN=5998;AN=20618;AC=1;AF=4.850130953535746e-05;N_HET=1;N_HOMALT=0;N_HOMREF=10308;FREQ_HET=9.700261907071492e-05;FREQ_HOMALT=0.0;FREQ_HOMREF=0.9999029973809292;N_BI_GENOS=10309;Lingnan_Han_AN=1888;Lingnan_Han_AC=1;Lingn
chr2	226868596	.	T	<INS:ME:LINE1>	.	PASS	SVTYPE=INS:ME:LINE1;END=226868614;SVLEN=515;AN=20618;AC=1;AF=4.850130953535746e-05;N_HET=1;N_HOMALT=0;N_HOMREF=10308;FREQ_HET=9.700261907071492e-05;FREQ_HOMALT=0.0;FREQ_HOMREF=0.9999029973809292;N_BI_GENOS=10309;Lingnan_Han_AN=1888;Lingnan_Han_AC=1;Lingna
chr2	228048037	.	A	<INS:ME:LINE1>	.	PASS	SVTYPE=INS:ME:LINE1;END=228048054;SVLEN=2220;AN=20618;AC=2;AF=9.700261907071492e-05;N_HET=2;N_HOMALT=0;N_HOMREF=10307;FREQ_HET=0.00019400523814142983;FREQ_HOMALT=0.0;FREQ_HOMREF=0.9998059947618586;N_BI_GENOS=10309;Northwest_Han_AN=1296;Northwest_Han_AC=1;
chr2	228365745	.	G	<INS:ME:LINE1>	.	PASS	SVTYPE=INS:ME:LINE1;END=228365745;SVLEN=496;AN=20618;AC=1;AF=4.850130953535746e-05;N_HET=1;N_HOMALT=0;N_HOMREF=10308;FREQ_HET=9.700261907071492e-05;FREQ_HOMALT=0.0;FREQ_HOMREF=0.9999029973809292;N_BI_GENOS=10309;Lingnan_Han_AN=1888;Lingnan_Han_AC=1;Lingna
chr2	228708776	.	A	<INS:ME:LINE1>	.	PASS	SVTYPE=INS:ME:LINE1;END=228708790;SVLEN=6007;AN=20618;AC=14;AF=0.0006790183334950044;N_HET=14;N_HOMALT=0;N_HOMREF=10295;FREQ_HET=0.0013580366669900088;FREQ_HOMALT=0.0;FREQ_HOMREF=0.99864196333301;N_BI_GENOS=10309;Northwest_Han_AN=1296;Northwest_Han_AC=1;N
chr2	228739554	.	C	<INS:ME:LINE1>	.	PASS	SVTYPE=INS:ME:LINE1;END=228739554;SVLEN=152;AN=20618;AC=1;AF=4.850130953535746e-05;N_HET=1;N_HOMALT=0;N_HOMREF=10308;FREQ_HET=9.700261907071492e-05;FREQ_HOMALT=0.0;FREQ_HOMREF=0.9999029973809292;N_BI_GENOS=10309;East_Han_AN=6554;East_Han_AC=1;East_Han_AF=
chr2	229019287	.	T	<INS:ME:LINE1>	.	PASS	SVTYPE=INS:ME:LINE1;END=229019293;SVLEN=548;AN=20618;AC=1;AF=4.850130953535746e-05;N_HET=1;N_HOMALT=0;N_HOMREF=10308;FREQ_HET=9.700261907071492e-05;FREQ_HOMALT=0.0;FREQ_HOMREF=0.9999029973809292;N_BI_GENOS=10309;Southeast_Han_AN=1908;Southeast_Han_AC=1;So
chr2	229074483	.	A	<INS:ME:LINE1>	.	PASS	SVTYPE=INS:ME:LINE1;END=229074498;SVLEN=1315;AN=20618;AC=13;AF=0.0006305170239596469;N_HET=13;N_HOMALT=0;N_HOMREF=10296;FREQ_HET=0.001261034047919293 8;FREQ_HOMALT=0.0;FREQ_HOMREF=0.9987389659520807;N_BI_GENOS=10309;East_Han_AN=6554;East_Han_AC=7;East_Han_
chr2	229476839	.	A	<INS:ME:LINE1>	.	PASS	SVTYPE=INS:ME:LINE1;END=229476843;SVLEN=6000;AN=20618;AC=1657;AF=0.0803666699000873;N_HET=1385;N_HOMALT=136;N_HOMREF=8788;FREQ_HET=0.13434862741294015;FREQ_HOMALT=0.013192356193617227;FREQ_HOMREF=0.8524590163934426;N_BI_GENOS=10309;South_Han_AN=1496;South
chr2	229619656	.	C	<INS:ME:LINE1>	.	PASS	SVTYPE=INS:ME:LINE1;END=229619673;SVLEN=6001;AN=20618;AC=1;AF=4.850130953535746e-05;N_HET=1;N_HOMALT=0;N_HOMREF=10308;FREQ_HET=9.700261907071492e-05;FREQ_HOMALT=0.0;FREQ_HOMREF=0.9999029973809292;N_BI_GENOS=10309;Zhuang_AN=444;Zhuang_AC=1;Zhuang_AF=0.0022
chr2	229624089	.	A	<INS:ME:LINE1>	.	PASS	SVTYPE=INS:ME:LINE1;END=229624100;SVLEN=333;AN=20618;AC=1;AF=4.850130953535746e-05;N_HET=1;N_HOMALT=0;N_HOMREF=10308;FREQ_HET=9.700261907071492e-05;FREQ_HOMALT=0.0;FREQ_HOMREF=0.9999029973809292;N_BI_GENOS=10309;North_Han_AN=2272;North_Han_AC=1;North_Han_
chr2	229703323	.	A	<INS:ME:LINE1>	.	PASS	SVTYPE=INS:ME:LINE1;END=229703339;SVLEN=1263;AN=20618;AC=1;AF=4.850130953535746e-05;N_HET=1;N_HOMALT=0;N_HOMREF=10308;FREQ_HET=9.700261907071492e-05;FREQ_HOMALT=0.0;FREQ_HOMREF=0.9999029973809292;N_BI_GENOS=10309;Southeast_Han_AN=1908;Southeast_Han_AC=1;S
chr2	230278036	.	A	<INS:ME:LINE1>	.	PASS	SVTYPE=INS:ME:LINE1;END=230278044;SVLEN=1061;AN=20618;AC=1;AF=4.850130953535746e-05;N_HET=1;N_HOMALT=0;N_HOMREF=10308;FREQ_HET=9.700261907071492e-05;FREQ_HOMALT=0.0;FREQ_HOMREF=0.9999029973809292;N_BI_GENOS=10309;Miao_AN=482;Miao_AC=1;Miao_AF=0.0020746887
chr2	231389085	.	A	<INS:ME:LINE1>	.	PASS	SVTYPE=INS:ME:LINE1;END=231389099;SVLEN=116;AN=20618;AC=1;AF=4.850130953535746e-05;N_HET=1;N_HOMALT=0;N_HOMREF=10308;FREQ_HET=9.700261907071492e-05;FREQ_HOMALT=0.0;FREQ_HOMREF=0.9999029973809292;N_BI_GENOS=10309;North_Han_AN=2272;North_Han_AC=1;North_Han_
chr2	231618013	.	A	<INS:ME:LINE1>	.	PASS	SVTYPE=INS:ME:LINE1;END=231618029;SVLEN=178;AN=20618;AC=2154;AF=0.10447182073915996;N_HET=1626;N_HOMALT=264;N_HOMREF=8419;FREQ_HET=0.15772625860898246;FREQ_HOMALT=0.025608691434668734;FREQ_HOMREF=0.8166650499563488;N_BI_GENOS=10309;South_Han_AN=1496;South
chr2	231917693	.	A	<INS:ME:LINE1>	.	PASS	SVTYPE=INS:ME:LINE1;END=231917709;SVLEN=705;AN=20618;AC=11;AF=0.000533514404888932;N_HET=11;N_HOMALT=0;N_HOMREF=10298;FREQ_HET=0.001067028809777864;FREQ_HOMALT=0.0;FREQ_HOMREF=0.9989329711902222;N_BI_GENOS=10309;South_Han_AN=1496;South_Han_AC=1;South_Han_
chr2	232285515	.	A	<INS:ME:LINE1>	.	PASS	SVTYPE=INS:ME:LINE1;END=232285525;SVLEN=1054;AN=20618;AC=1;AF=4.850130953535746e-05;N_HET=1;N_HOMALT=0;N_HOMREF=10308;FREQ_HET=9.700261907071492e-05;FREQ_HOMALT=0.0;FREQ_HOMREF=0.9999029973809292;N_BI_GENOS=10309;Mongolian_AN=454;Mongolian_AC=1;Mongolian_
chr2	232349470	.	G	<INS:ME:LINE1>	.	PASS	SVTYPE=INS:ME:LINE1;END=232349470;SVLEN=1553;AN=20618;AC=2;AF=9.700261907071492e-05;N_HET=2;N_HOMALT=0;N_HOMREF=10307;FREQ_HET=0.00019400523814142983;FREQ_HOMALT=0.0;FREQ_HOMREF=0.9998059947618586;N_BI_GENOS=10309;East_Han_AN=6554;East_Han_AC=2;East_Han_A
chr2	232726813	.	A	<INS:ME:LINE1>	.	PASS	SVTYPE=INS:ME:LINE1;END=232726828;SVLEN=821;AN=20618;AC=1;AF=4.850130953535746e-05;N_HET=1;N_HOMALT=0;N_HOMREF=10308;FREQ_HET=9.700261907071492e-05;FREQ_HOMALT=0.0;FREQ_HOMREF=0.9999029973809292;N_BI_GENOS=10309;East_Han_AN=6554;East_Han_AC=1;East_Han_AF=
chr2	233504035	.	A	<INS:ME:LINE1>	.	PASS	SVTYPE=INS:ME:LINE1;END=233504052;SVLEN=5992;AN=20618;AC=1;AF=4.850130953535746e-05;N_HET=1;N_HOMALT=0;N_HOMREF=10308;FREQ_HET=9.700261907071492e-05;FREQ_HOMALT=0.0;FREQ_HOMREF=0.9999029973809292;N_BI_GENOS=10309;South_Han_AN=1496;South_Han_AC=1;South_Han
chr2	233599444	.	A	<INS:ME:LINE1>	.	PASS	SVTYPE=INS:ME:LINE1;END=233599461;SVLEN=967;AN=20618;AC=2;AF=9.700261907071492e-05;N_HET=2;N_HOMALT=0;N_HOMREF=10307;FREQ_HET=0.00019400523814142983;FREQ_HOMALT=0.0;FREQ_HOMREF=0.9998059947618586;N_BI_GENOS=10309;East_Han_AN=6554;East_Han_AC=2;East_Han_AF
chr2	233749336	.	T	<INS:ME:LINE1>	.	PASS	SVTYPE=INS:ME:LINE1;END=233749337;SVLEN=900;AN=20618;AC=1;AF=4.850130953535746e-05;N_HET=1;N_HOMALT=0;N_HOMREF=10308;FREQ_HET=9.700261907071492e-05;FREQ_HOMALT=0.0;FREQ_HOMREF=0.9999029973809292;N_BI_GENOS=10309;Southeast_Han_AN=1908;Southeast_Han_AC=1;So
chr2	233904123	.	T	<INS:ME:LINE1>	.	PASS	SVTYPE=INS:ME:LINE1;END=233904130;SVLEN=6263;AN=20618;AC=1;AF=4.850130953535746e-05;N_HET=1;N_HOMALT=0;N_HOMREF=10308;FREQ_HET=9.700261907071492e-05;FREQ_HOMALT=0.0;FREQ_HOMREF=0.9999029973809292;N_BI_GENOS=10309;North_Han_AN=2272;North_Han_AC=1;North_Han
chr2	234611149	.	A	<INS:ME:LINE1>	.	PASS	SVTYPE=INS:ME:LINE1;END=234611166;SVLEN=1039;AN=20618;AC=992;AF=0.0481132990590746;N_HET=830;N_HOMALT=81;N_HOMREF=9398;FREQ_HET=0.08051217382869337;FREQ_HOMALT=0.007857212144727907;FREQ_HOMREF=0.9116306140265787;N_BI_GENOS=10309;South_Han_AN=1496;South_Ha
chr2	234638091	.	T	<INS:ME:LINE1>	.	PASS	SVTYPE=INS:ME:LINE1;END=234638091;SVLEN=0;AN=20618;AC=2;AF=9.700261907071492e-05;N_HET=0;N_HOMALT=1;N_HOMREF=10308;FREQ_HET=0.0;FREQ_HOMALT=9.700261907071492e-05;FREQ_HOMREF=0.9999029973809292;N_BI_GENOS=10309;Southeast_Han_AN=1908;Southeast_Han_AC=2;Sout
chr2	235188250	.	G	<INS:ME:LINE1>	.	PASS	SVTYPE=INS:ME:LINE1;END=235188267;SVLEN=5996;AN=20618;AC=137;AF=0.006644679406343971;N_HET=129;N_HOMALT=4;N_HOMREF=10176;FREQ_HET=0.012513337860122223;FREQ_HOMALT=0.0003880104762828596 6;FREQ_HOMREF=0.9870986516635949;N_BI_GENOS=10309;South_Han_AN=1496;Sou
chr2	235274991	.	A	<INS:ME:LINE1>	.	PASS	SVTYPE=INS:ME:LINE1;END=235275006;SVLEN=2063;AN=20618;AC=3;AF=0.00014550392860607236;N_HET=3;N_HOMALT=0;N_HOMREF=10306;FREQ_HET=0.0002910078572121447;FREQ_HOMALT=0.0;FREQ_HOMREF=0.9997089921427879;N_BI_GENOS=10309;East_Han_AN=6554;East_Han_AC=1;East_Han_A
chr2	235461058	.	C	<INS:ME:LINE1>	.	PASS	SVTYPE=INS:ME:LINE1;END=235461074;SVLEN=1537;AN=20618;AC=10;AF=0.0004850130953535455;N_HET=10;N_HOMALT=0;N_HOMREF=10299;FREQ_HET=0.0009700261907071491;FREQ_HOMALT=0.0;FREQ_HOMREF=0.9990299738092928;N_BI_GENOS=10309;Northwest_Han_AN=1296;Northwest_Han_AC=
chr2	235679037	.	A	<INS:ME:LINE1>	.	PASS	SVTYPE=INS:ME:LINE1;END=235679051;SVLEN=1749;AN=20618;AC=5;AF=0.00024250654767678728;N_HET=5;N_HOMALT=0;N_HOMREF=10304;FREQ_HET=0.0004850130953535745 5;FREQ_HOMALT=0.0;FREQ_HOMREF=0.9995149869046465;N_BI_GENOS=10309;South_Han_AN=1496;South_Han_AC=1;South_H
chr2	236264584	.	A	<INS:ME:LINE1>	.	PASS	SVTYPE=INS:ME:LINE1;END=236264603;SVLEN=622;AN=20618;AC=1;AF=4.850130953535746e-05;N_HET=1;N_HOMALT=0;N_HOMREF=10308;FREQ_HET=9.700261907071492e-05;FREQ_HOMALT=0.0;FREQ_HOMREF=0.9999029973809292;N_BI_GENOS=10309;East_Han_AN=6554;East_Han_AC=1;East_Han_AF=
chr2	236659815	.	C	<INS:ME:LINE1>	.	PASS	SVTYPE=INS:ME:LINE1;END=236659825;SVLEN=206;AN=20618;AC=1;AF=4.850130953535746e-05;N_HET=1;N_HOMALT=0;N_HOMREF=10308;FREQ_HET=9.700261907071492e-05;FREQ_HOMALT=0.0;FREQ_HOMREF=0.9999029973809292;N_BI_GENOS=10309;Northwest_Han_AN=1296;Northwest_Han_AC=1;No
chr2	237608353	.	C	<INS:ME:LINE1>	.	PASS	SVTYPE=INS:ME:LINE1;END=237608353;SVLEN=0;AN=20618;AC=3;AF=0.00014550392860607236;N_HET=1;N_HOMALT=1;N_HOMREF=10307;FREQ_HET=9.700261907071492e-05;FREQ_HOMALT=9.700261907071492e-05;FREQ_HOMREF=0.9998059947618586;N_BI_GENOS=10309;East_Han_AN=6554;East_Han_
chr2	237723449	.	A	<INS:ME:LINE1>	.	PASS	SVTYPE=INS:ME:LINE1;END=237723461;SVLEN=6017;AN=20618;AC=1;AF=4.850130953535746e-05;N_HET=1;N_HOMALT=0;N_HOMREF=10308;FREQ_HET=9.700261907071492e-05;FREQ_HOMALT=0.0;FREQ_HOMREF=0.9999029973809292;N_BI_GENOS=10309;Tibetan_AN=414;Tibetan_AC=1;Tibetan_AF=0.0
chr2	238805487	.	G	<INS:ME:LINE1>	.	PASS	SVTYPE=INS:ME:LINE1;END=238805498;SVLEN=1598;AN=20618;AC=1;AF=4.850130953535746e-05;N_HET=1;N_HOMALT=0;N_HOMREF=10308;FREQ_HET=9.700261907071492e-05;FREQ_HOMALT=0.0;FREQ_HOMREF=0.9999029973809292;N_BI_GENOS=10309;Miao_AN=482;Miao_AC=1;Miao_AF=0.0020746887
chr2	238820797	.	A	<INS:ME:LINE1>	.	PASS	SVTYPE=INS:ME:LINE1;END=238820810;SVLEN=5992;AN=20618;AC=2;AF=9.700261907071492e-05;N_HET=2;N_HOMALT=0;N_HOMREF=10307;FREQ_HET=0.00019400523814142983;FREQ_HOMALT=0.0;FREQ_HOMREF=0.9998059947618586;N_BI_GENOS=10309;East_Han_AN=6554;East_Han_AC=2;East_Han_A
chr2	238974999	.	G	<INS:ME:LINE1>	.	PASS	SVTYPE=INS:ME:LINE1;END=238975013;SVLEN=6000;AN=20618;AC=1;AF=4.850130953535746e-05;N_HET=1;N_HOMALT=0;N_HOMREF=10308;FREQ_HET=9.700261907071492e-05;FREQ_HOMALT=0.0;FREQ_HOMREF=0.9999029973809292;N_BI_GENOS=10309;South_Han_AN=1496;South_Han_AC=1;South_Han
chr2	240202474	.	A	<INS:ME:LINE1>	.	PASS	SVTYPE=INS:ME:LINE1;END=240202487;SVLEN=1004;AN=20618;AC=1;AF=4.850130953535746e-05;N_HET=1;N_HOMALT=0;N_HOMREF=10308;FREQ_HET=9.700261907071492e-05;FREQ_HOMALT=0.0;FREQ_HOMREF=0.9999029973809292;N_BI_GENOS=10309;East_Han_AN=6554;East_Han_AC=1;East_Han_AF
chr3	112058	.	A	<INS:ME:LINE1>	.	PASS	SVTYPE=INS:ME:LINE1;END=112074;SVLEN=1730;AN=20618;AC=3;AF=0.00014550392860607236;N_HET=3;N_HOMALT=0;N_HOMREF=10306;FREQ_HET=0.0002910078572121447;FREQ_HOMALT=0.0;FREQ_HOMREF=0.9997089921427879;N_BI_GENOS=10309;North_Han_AN=2272;North_Han_AC=2;North_Han_A
chr3	545473	.	T	<INS:ME:LINE1>	.	PASS	SVTYPE=INS:ME:LINE1;END=545483;SVLEN=2240;AN=20618;AC=1;AF=4.850130953535746e-05;N_HET=1;N_HOMALT=0;N_HOMREF=10308;FREQ_HET=9.700261907071492e-05;FREQ_HOMALT=0.0;FREQ_HOMREF=0.9999029973809292;N_BI_GENOS=10309;Central_Han_AN=2128;Central_Han_AC=1;Central_
chr3	722998	.	A	<INS:ME:LINE1>	.	PASS	SVTYPE=INS:ME:LINE1;END=723014;SVLEN=3570;AN=20618;AC=1;AF=4.850130953535746e-05;N_HET=1;N_HOMALT=0;N_HOMREF=10308;FREQ_HET=9.700261907071492e-05;FREQ_HOMALT=0.0;FREQ_HOMREF=0.9999029973809292;N_BI_GENOS=10309;Tibetan_AN=414;Tibetan_AC=1;Tibetan_AF=0.0024

chr3	933378	.	C	<INS:ME:LINE1>	.	PASS	SVTYPE=INS:ME:LINE1;END=933392;SVLEN=6005;AN=20618;AC=3;AF=0.00014550392860607236;N_HET=1;N_HOMALT=1;N_HOMREF=10307;FREQ_HET=9.700261907071492e-05;FREQ_HOMALT=9.700261907071492e-05;FREQ_HOMREF=0.9998059947618586;N_BI_GENOS=10309;Southeast_Han_AN=1908;Sout
chr3	1040025	.	G	<INS:ME:LINE1>	.	PASS	SVTYPE=INS:ME:LINE1;END=1040025;SVLEN=106;AN=20618;AC=2;AF=9.700261907071492e-05;N_HET=2;N_HOMALT=0;N_HOMREF=10307;FREQ_HET=0.00019400523814142983;FREQ_HOMALT=0.0;FREQ_HOMREF=0.9998059947618586;N_BI_GENOS=10309;Central_Han_AN=2128;Central_Han_AC=2;Central
chr3	1669207	.	A	<INS:ME:LINE1>	.	PASS	SVTYPE=INS:ME:LINE1;END=1669223;SVLEN=91;AN=20618;AC=1;AF=4.850130953535746e-05;N_HET=1;N_HOMALT=0;N_HOMREF=10308;FREQ_HET=9.700261907071492e-05;FREQ_HOMALT=0.0;FREQ_HOMREF=0.9999029973809292;N_BI_GENOS=10309;East_Han_AN=6554;East_Han_AC=1;East_Han_AF=0.0
chr3	1816545	.	T	<INS:ME:LINE1>	.	PASS	SVTYPE=INS:ME:LINE1;END=1816550;SVLEN=568;AN=20618;AC=1;AF=4.850130953535746e-05;N_HET=1;N_HOMALT=0;N_HOMREF=10308;FREQ_HET=9.700261907071492e-05;FREQ_HOMALT=0.0;FREQ_HOMREF=0.9999029973809292;N_BI_GENOS=10309;Lingnan_Han_AN=1888;Lingnan_Han_AC=1;Lingnan_
chr3	2284670	.	C	<INS:ME:LINE1>	.	PASS	SVTYPE=INS:ME:LINE1;END=2284705;SVLEN=6000;AN=20618;AC=1;AF=4.850130953535746e-05;N_HET=1;N_HOMALT=0;N_HOMREF=10308;FREQ_HET=9.700261907071492e-05;FREQ_HOMALT=0.0;FREQ_HOMREF=0.9999029973809292;N_BI_GENOS=10309;Central_Han_AN=2128;Central_Han_AC=1;Central
chr3	2523428	.	A	<INS:ME:LINE1>	.	PASS	SVTYPE=INS:ME:LINE1;END=2523443;SVLEN=6020;AN=20618;AC=1;AF=4.850130953535746e-05;N_HET=1;N_HOMALT=0;N_HOMREF=10308;FREQ_HET=9.700261907071492e-05;FREQ_HOMALT=0.0;FREQ_HOMREF=0.9999029973809292;N_BI_GENOS=10309;Lingnan_Han_AN=1888;Lingnan_Han_AC=1;Lingnan
chr3	2818661	.	G	<INS:ME:LINE1>	.	PASS	SVTYPE=INS:ME:LINE1;END=2818673;SVLEN=1126;AN=20618;AC=8;AF=0.00038801047628285966;N_HET=8;N_HOMALT=0;N_HOMREF=10301;FREQ_HET=0.0007760209525657193;FREQ_HOMALT=0.0;FREQ_HOMREF=0.9992239790474343;N_BI_GENOS=10309;Lingnan_Han_AN=1888;Lingnan_Han_AC=1;Lingna
chr3	3115930	.	A	<INS:ME:LINE1>	.	PASS	SVTYPE=INS:ME:LINE1;END=3115945;SVLEN=5998;AN=20618;AC=2;AF=9.700261907071492e-05;N_HET=2;N_HOMALT=0;N_HOMREF=10307;FREQ_HET=0.00019400523814142983;FREQ_HOMALT=0.0;FREQ_HOMREF=0.9998059947618586;N_BI_GENOS=10309;South_Han_AN=1496;South_Han_AC=1;South_Han_
chr3	3239699	.	G	<INS:ME:LINE1>	.	PASS	SVTYPE=INS:ME:LINE1;END=3239712;SVLEN=2800;AN=20618;AC=1;AF=4.850130953535746e-05;N_HET=1;N_HOMALT=0;N_HOMREF=10308;FREQ_HET=9.700261907071492e-05;FREQ_HOMALT=0.0;FREQ_HOMREF=0.9999029973809292;N_BI_GENOS=10309;Lingnan_Han_AN=1888;Lingnan_Han_AC=1;Lingnan
chr3	3305946	.	A	<INS:ME:LINE1>	.	PASS	SVTYPE=INS:ME:LINE1;END=3305964;SVLEN=5786;AN=20618;AC=1;AF=4.850130953535746e-05;N_HET=1;N_HOMALT=0;N_HOMREF=10308;FREQ_HET=9.700261907071492e-05;FREQ_HOMALT=0.0;FREQ_HOMREF=0.9999029973809292;N_BI_GENOS=10309;East_Han_AN=6554;East_Han_AC=1;East_Han_AF=0
chr3	3382711	.	A	<INS:ME:LINE1>	.	PASS	SVTYPE=INS:ME:LINE1;END=3382726;SVLEN=49;AN=20618;AC=3;AF=0.00014550392860607236;N_HET=3;N_HOMALT=0;N_HOMREF=10306;FREQ_HET=0.0002910078572121447;FREQ_HOMALT=0.0;FREQ_HOMREF=0.9997089921427879;N_BI_GENOS=10309;Central_Han_AN=2128;Central_Han_AC=2;Central_
chr3	3453118	.	G	<INS:ME:LINE1>	.	PASS	SVTYPE=INS:ME:LINE1;END=3453135;SVLEN=2680;AN=20618;AC=3;AF=0.00014550392860607236;N_HET=3;N_HOMALT=0;N_HOMREF=10306;FREQ_HET=0.0002910078572121447;FREQ_HOMALT=0.0;FREQ_HOMREF=0.9997089921427879;N_BI_GENOS=10309;Lingnan_Han_AN=1888;Lingnan_Han_AC=2;Lingna
chr3	3498546	.	T	<INS:ME:LINE1>	.	PASS	SVTYPE=INS:ME:LINE1;END=3498561;SVLEN=5993;AN=20618;AC=1;AF=4.850130953535746e-05;N_HET=1;N_HOMALT=0;N_HOMREF=10308;FREQ_HET=9.700261907071492e-05;FREQ_HOMALT=0.0;FREQ_HOMREF=0.9999029973809292;N_BI_GENOS=10309;Central_Han_AN=2128;Central_Han_AC=1;Central
chr3	3785743	.	C	<INS:ME:LINE1>	.	PASS	SVTYPE=INS:ME:LINE1;END=3785761;SVLEN=2049;AN=20618;AC=1;AF=4.850130953535746e-05;N_HET=1;N_HOMALT=0;N_HOMREF=10308;FREQ_HET=9.700261907071492e-05;FREQ_HOMALT=0.0;FREQ_HOMREF=0.9999029973809292;N_BI_GENOS=10309;Southeast_Han_AN=1908;Southeast_Han_AC=1;Sou
chr3	3974744	.	C	<INS:ME:LINE1>	.	PASS	SVTYPE=INS:ME:LINE1;END=3974744;SVLEN=657;AN=20618;AC=1;AF=4.850130953535746e-05;N_HET=1;N_HOMALT=0;N_HOMREF=10308;FREQ_HET=9.700261907071492e-05;FREQ_HOMALT=0.0;FREQ_HOMREF=0.9999029973809292;N_BI_GENOS=10309;Lingnan_Han_AN=1888;Lingnan_Han_AC=1;Lingnan_
chr3	3975105	.	A	<INS:ME:LINE1>	.	PASS	SVTYPE=INS:ME:LINE1;END=3975105;SVLEN=924;AN=20618;AC=1;AF=4.850130953535746e-05;N_HET=1;N_HOMALT=0;N_HOMREF=10308;FREQ_HET=9.700261907071492e-05;FREQ_HOMALT=0.0;FREQ_HOMREF=0.9999029973809292;N_BI_GENOS=10309;East_Han_AN=6554;East_Han_AC=1;East_Han_AF=0.
chr3	4063190	.	T	<INS:ME:LINE1>	.	PASS	SVTYPE=INS:ME:LINE1;END=4063207;SVLEN=1355;AN=20618;AC=1;AF=4.850130953535746e-05;N_HET=1;N_HOMALT=0;N_HOMREF=10308;FREQ_HET=9.700261907071492e-05;FREQ_HOMALT=0.0;FREQ_HOMREF=0.9999029973809292;N_BI_GENOS=10309;East_Han_AN=6554;East_Han_AC=1;East_Han_AF=0
chr3	4093579	.	A	<INS:ME:LINE1>	.	PASS	SVTYPE=INS:ME:LINE1;END=4093595;SVLEN=5998;AN=20618;AC=1;AF=4.850130953535746e-05;N_HET=1;N_HOMALT=0;N_HOMREF=10308;FREQ_HET=9.700261907071492e-05;FREQ_HOMALT=0.0;FREQ_HOMREF=0.9999029973809292;N_BI_GENOS=10309;South_Han_AN=1496;South_Han_AC=1;South_Han_A
chr3	4160294	.	A	<INS:ME:LINE1>	.	PASS	SVTYPE=INS:ME:LINE1;END=4160308;SVLEN=366;AN=20618;AC=2;AF=9.700261907071492e-05;N_HET=2;N_HOMALT=0;N_HOMREF=10307;FREQ_HET=0.00019400523814142983;FREQ_HOMALT=0.0;FREQ_HOMREF=0.9998059947618586;N_BI_GENOS=10309;South_Han_AN=1496;South_Han_AC=1;South_Han_A
chr3	4225009	.	A	<INS:ME:LINE1>	.	PASS	SVTYPE=INS:ME:LINE1;END=4225025;SVLEN=3834;AN=20618;AC=1;AF=4.850130953535746e-05;N_HET=1;N_HOMALT=0;N_HOMREF=10308;FREQ_HET=9.700261907071492e-05;FREQ_HOMALT=0.0;FREQ_HOMREF=0.9999029973809292;N_BI_GENOS=10309;South_Han_AN=1496;South_Han_AC=1;South_Han_A
chr3	4388992	.	A	<INS:ME:LINE1>	.	PASS	SVTYPE=INS:ME:LINE1;END=4389008;SVLEN=1368;AN=20618;AC=4;AF=0.00019400523814142983;N_HET=4;N_HOMALT=0;N_HOMREF=10305;FREQ_HET=0.00038801047628285966;FREQ_HOMALT=0.0;FREQ_HOMREF=0.9996119895237171;N_BI_GENOS=10309;South_Han_AN=1496;South_Han_AC=2;South_Han
chr3	4960041	.	A	<INS:ME:LINE1>	.	PASS	SVTYPE=INS:ME:LINE1;END=4960044;SVLEN=469;AN=20618;AC=15;AF=0.0007275196430303619;N_HET=15;N_HOMALT=0;N_HOMREF=10294;FREQ_HET=0.0014550392860607237;FREQ_HOMALT=0.0;FREQ_HOMREF=0.9985449607139393;N_BI_GENOS=10309;South_Han_AN=1496;South_Han_AC=1;South_Han_
chr3	5456970	.	C	<INS:ME:LINE1>	.	PASS	SVTYPE=INS:ME:LINE1;END=5456983;SVLEN=6000;AN=20618;AC=2;AF=9.700261907071492e-05;N_HET=2;N_HOMALT=0;N_HOMREF=10307;FREQ_HET=0.00019400523814142983;FREQ_HOMALT=0.0;FREQ_HOMREF=0.9998059947618586;N_BI_GENOS=10309;East_Han_AN=6554;East_Han_AC=1;East_Han_AF=
chr3	5575311	.	A	<INS:ME:LINE1>	.	PASS	SVTYPE=INS:ME:LINE1;END=5575324;SVLEN=5998;AN=20618;AC=4;AF=0.00019400523814142983;N_HET=4;N_HOMALT=0;N_HOMREF=10305;FREQ_HET=0.00038801047628285966;FREQ_HOMALT=0.0;FREQ_HOMREF=0.9996119895237171;N_BI_GENOS=10309;South_Han_AN=1496;South_Han_AC=2;South_Han
chr3	5604518	.	G	<INS:ME:LINE1>	.	PASS	SVTYPE=INS:ME:LINE1;END=5604529;SVLEN=1563;AN=20618;AC=1;AF=4.850130953535746e-05;N_HET=1;N_HOMALT=0;N_HOMREF=10308;FREQ_HET=9.700261907071492e-05;FREQ_HOMALT=0.0;FREQ_HOMREF=0.9999029973809292;N_BI_GENOS=10309;East_Han_AN=6554;East_Han_AC=1;East_Han_AF=0
chr3	5638044	.	A	<INS:ME:LINE1>	.	PASS	SVTYPE=INS:ME:LINE1;END=5638060;SVLEN=3632;AN=20618;AC=1;AF=4.850130953535746e-05;N_HET=1;N_HOMALT=0;N_HOMREF=10308;FREQ_HET=9.700261907071492e-05;FREQ_HOMALT=0.0;FREQ_HOMREF=0.9999029973809292;N_BI_GENOS=10309;Lingnan_Han_AN=1888;Lingnan_Han_AC=1;Lingnan
chr3	5809779	.	A	<INS:ME:LINE1>	.	PASS	SVTYPE=INS:ME:LINE1;END=5809792;SVLEN=2035;AN=20618;AC=1;AF=4.850130953535746e-05;N_HET=1;N_HOMALT=0;N_HOMREF=10308;FREQ_HET=9.700261907071492e-05;FREQ_HOMALT=0.0;FREQ_HOMREF=0.9999029973809292;N_BI_GENOS=10309;East_Han_AN=6554;East_Han_AC=1;East_Han_AF=0
chr3	6635729	.	A	<INS:ME:LINE1>	.	PASS	SVTYPE=INS:ME:LINE1;END=6635731;SVLEN=1800;AN=20618;AC=10;AF=0.00048501309535357455;N_HET=8;N_HOMALT=1;N_HOMREF=10300;FREQ_HET=0.0007760209525657193;FREQ_HOMALT=9.700261907071492e-05;FREQ_HOMREF=0.9991269764283636;N_BI_GENOS=10309;North_Han_AN=2272;North_
chr3	6702913	.	G	<INS:ME:LINE1>	.	PASS	SVTYPE=INS:ME:LINE1;END=6702954;SVLEN=2682;AN=20618;AC=1;AF=4.850130953535746e-05;N_HET=1;N_HOMALT=0;N_HOMREF=10308;FREQ_HET=9.700261907071492e-05;FREQ_HOMALT=0.0;FREQ_HOMREF=0.9999029973809292;N_BI_GENOS=10309;East_Han_AN=6554;East_Han_AC=1;East_Han_AF=0
chr3	6806643	.	T	<INS:ME:LINE1>	.	PASS	SVTYPE=INS:ME:LINE1;END=6806656;SVLEN=397;AN=20618;AC=64;AF=0.0031040838102628773;N_HET=62;N_HOMALT=1;N_HOMREF=10246;FREQ_HET=0.006014162382384325;FREQ_HOMALT=9.700261907071492e-05;FREQ_HOMREF=0.993888834998545;N_BI_GENOS=10309;South_Han_AN=1496;South_Han
chr3	6809774	.	T	<INS:ME:LINE1>	.	PASS	SVTYPE=INS:ME:LINE1;END=6809791;SVLEN=235;AN=20618;AC=1;AF=4.850130953535746e-05;N_HET=1;N_HOMALT=0;N_HOMREF=10308;FREQ_HET=9.700261907071492e-05;FREQ_HOMALT=0.0;FREQ_HOMREF=0.9999029973809292;N_BI_GENOS=10309;Lingnan_Han_AN=1888;Lingnan_Han_AC=1;Lingnan_
chr3	6940516	.	G	<INS:ME:LINE1>	.	PASS	SVTYPE=INS:ME:LINE1;END=6940516;SVLEN=2714;AN=20618;AC=1;AF=4.850130953535746e-05;N_HET=1;N_HOMALT=0;N_HOMREF=10308;FREQ_HET=9.700261907071492e-05;FREQ_HOMALT=0.0;FREQ_HOMREF=0.9999029973809292;N_BI_GENOS=10309;East_Han_AN=6554;East_Han_AC=1;East_Han_AF=0
chr3	7005699	.	A	<INS:ME:LINE1>	.	PASS	SVTYPE=INS:ME:LINE1;END=7005703;SVLEN=3546;AN=20618;AC=1;AF=4.850130953535746e-05;N_HET=1;N_HOMALT=0;N_HOMREF=10308;FREQ_HET=9.700261907071492e-05;FREQ_HOMALT=0.0;FREQ_HOMREF=0.9999029973809292;N_BI_GENOS=10309;Southeast_Han_AN=1908;Southeast_Han_AC=1;Sou
chr3	7080566	.	C	<INS:ME:LINE1>	.	PASS	SVTYPE=INS:ME:LINE1;END=7080584;SVLEN=6022;AN=20618;AC=1;AF=4.850130953535746e-05;N_HET=1;N_HOMALT=0;N_HOMREF=10308;FREQ_HET=9.700261907071492e-05;FREQ_HOMALT=0.0;FREQ_HOMREF=0.9999029973809292;N_BI_GENOS=10309;East_Han_AN=6554;East_Han_AC=1;East_Han_AF=0
chr3	7091714	.	A	<INS:ME:LINE1>	.	PASS	SVTYPE=INS:ME:LINE1;END=7091714;SVLEN=15;AN=20618;AC=1;AF=4.850130953535746e-05;N_HET=1;N_HOMALT=0;N_HOMREF=10308;FREQ_HET=9.700261907071492e-05;FREQ_HOMALT=0.0;FREQ_HOMREF=0.9999029973809292;N_BI_GENOS=10309;Southeast_Han_AN=1908;Southeast_Han_AC=1;South
chr3	7106177	.	T	<INS:ME:LINE1>	.	PASS	SVTYPE=INS:ME:LINE1;END=7106191;SVLEN=1361;AN=20618;AC=1;AF=4.850130953535746e-05;N_HET=1;N_HOMALT=0;N_HOMREF=10308;FREQ_HET=9.700261907071492e-05;FREQ_HOMALT=0.0;FREQ_HOMREF=0.9999029973809292;N_BI_GENOS=10309;North_Han_AN=2272;North_Han_AC=1;North_Han_A
chr3	7106267	.	A	<INS:ME:LINE1>	.	PASS	SVTYPE=INS:ME:LINE1;END=7106317;SVLEN=69;AN=20618;AC=1;AF=4.850130953535746e-05;N_HET=1;N_HOMALT=0;N_HOMREF=10308;FREQ_HET=9.700261907071492e-05;FREQ_HOMALT=0.0;FREQ_HOMREF=0.9999029973809292;N_BI_GENOS=10309;East_Han_AN=6554;East_Han_AC=1;East_Han_AF=0.0
chr3	7318584	.	A	<INS:ME:LINE1>	.	PASS	SVTYPE=INS:ME:LINE1;END=7318597;SVLEN=6059;AN=20618;AC=1;AF=4.850130953535746e-05;N_HET=1;N_HOMALT=0;N_HOMREF=10308;FREQ_HET=9.700261907071492e-05;FREQ_HOMALT=0.0;FREQ_HOMREF=0.9999029973809292;N_BI_GENOS=10309;South_Han_AN=1496;South_Han_AC=1;South_Han_A
chr3	7511697	.	A	<INS:ME:LINE1>	.	PASS	SVTYPE=INS:ME:LINE1;END=7511702;SVLEN=133;AN=20618;AC=1;AF=4.850130953535746e-05;N_HET=1;N_HOMALT=0;N_HOMREF=10308;FREQ_HET=9.700261907071492e-05;FREQ_HOMALT=0.0;FREQ_HOMREF=0.9999029973809292;N_BI_GENOS=10309;Yi_AN=422;Yi_AC=1;Yi_AF=0.002369668246445498;
chr3	7526382	.	A	<INS:ME:LINE1>	.	PASS	SVTYPE=INS:ME:LINE1;END=7526382;SVLEN=535;AN=20618;AC=1;AF=4.850130953535746e-05;N_HET=1;N_HOMALT=0;N_HOMREF=10308;FREQ_HET=9.700261907071492e-05;FREQ_HOMALT=0.0;FREQ_HOMREF=0.9999029973809292;N_BI_GENOS=10309;East_Han_AN=6554;East_Han_AC=1;East_Han_AF=0.
chr3	7548259	.	A	<INS:ME:LINE1>	.	PASS	SVTYPE=INS:ME:LINE1;END=7548262;SVLEN=270;AN=20618;AC=1;AF=4.850130953535746e-05;N_HET=1;N_HOMALT=0;N_HOMREF=10308;FREQ_HET=9.700261907071492e-05;FREQ_HOMALT=0.0;FREQ_HOMREF=0.9999029973809292;N_BI_GENOS=10309;Northwest_Han_AN=1296;Northwest_Han_AC=1;Nort
chr3	7561594	.	G	<INS:ME:LINE1>	.	PASS	SVTYPE=INS:ME:LINE1;END=7561610;SVLEN=854;AN=20618;AC=29;AF=0.0014065379765253662;N_HET=29;N_HOMALT=0;N_HOMREF=10280;FREQ_HET=0.0028130759530507325;FREQ_HOMALT=0.0;FREQ_HOMREF=0.9971869240469493;N_BI_GENOS=10309;South_Han_AN=1496;South_Han_AC=6;South_Han_
chr3	7641413	.	A	<INS:ME:LINE1>	.	PASS	SVTYPE=INS:ME:LINE1;END=7641417;SVLEN=1043;AN=20618;AC=2;AF=9.700261907071492e-05;N_HET=2;N_HOMALT=0;N_HOMREF=10307;FREQ_HET=0.00019400523814142983;FREQ_HOMALT=0.0;FREQ_HOMREF=0.9998059947618586;N_BI_GENOS=10309;North_Han_AN=2272;North_Han_AC=1;North_Han_
chr3	7707878	.	A	<INS:ME:LINE1>	.	PASS	SVTYPE=INS:ME:LINE1;END=7707895;SVLEN=6008;AN=20618;AC=1;AF=4.850130953535746e-05;N_HET=1;N_HOMALT=0;N_HOMREF=10308;FREQ_HET=9.700261907071492e-05;FREQ_HOMALT=0.0;FREQ_HOMREF=0.9999029973809292;N_BI_GENOS=10309;Northwest_Han_AN=1296;Northwest_Han_AC=1;Nor
chr3	7882249	.	T	<INS:ME:LINE1>	.	PASS	SVTYPE=INS:ME:LINE1;END=7882266;SVLEN=2875;AN=20618;AC=1;AF=4.850130953535746e-05;N_HET=1;N_HOMALT=0;N_HOMREF=10308;FREQ_HET=9.700261907071492e-05;FREQ_HOMALT=0.0;FREQ_HOMREF=0.9999029973809292;N_BI_GENOS=10309;East_Han_AN=6554;East_Han_AC=1;East_Han_AF=0
chr3	7954592	.	A	<INS:ME:LINE1>	.	PASS	SVTYPE=INS:ME:LINE1;END=7954609;SVLEN=622;AN=20618;AC=11;AF=0.000533514404888932;N_HET=11;N_HOMALT=0;N_HOMREF=10298;FREQ_HET=0.001067028809777864;FREQ_HOMALT=0.0;FREQ_HOMREF=0.9989329711902222;N_BI_GENOS=10309;Northwest_Han_AN=1296;Northwest_Han_AC=2;Nort
chr3	7964720	.	A	<INS:ME:LINE1>	.	PASS	SVTYPE=INS:ME:LINE1;END=7964722;SVLEN=2192;AN=20618;AC=1;AF=4.850130953535746e-05;N_HET=1;N_HOMALT=0;N_HOMREF=10308;FREQ_HET=9.700261907071492e-05;FREQ_HOMALT=0.0;FREQ_HOMREF=0.9999029973809292;N_BI_GENOS=10309;East_Han_AN=6554;East_Han_AC=1;East_Han_AF=0
chr3	7971345	.	A	<INS:ME:LINE1>	.	PASS	SVTYPE=INS:ME:LINE1;END=7971361;SVLEN=6011;AN=20618;AC=1;AF=4.850130953535746e-05;N_HET=1;N_HOMALT=0;N_HOMREF=10308;FREQ_HET=9.700261907071492e-05;FREQ_HOMALT=0.0;FREQ_HOMREF=0.9999029973809292;N_BI_GENOS=10309;East_Han_AN=6554;East_Han_AC=1;East_Han_AF=0
chr3	8327916	.	T	<INS:ME:LINE1>	.	PASS	SVTYPE=INS:ME:LINE1;END=8327924;SVLEN=1301;AN=20618;AC=61;AF=0.0029585798816568047;N_HET=59;N_HOMALT=1;N_HOMREF=10249;FREQ_HET=0.0057231545251721794;FREQ_HOMALT=9.700261907071492e-05;FREQ_HOMREF=0.9941798428557571;N_BI_GENOS=10309;South_Han_AN=1496;South_
chr3	8540805	.	G	<INS:ME:LINE1>	.	PASS	SVTYPE=INS:ME:LINE1;END=8540821;SVLEN=1570;AN=20618;AC=3;AF=0.00014550392860607236;N_HET=3;N_HOMALT=0;N_HOMREF=10306;FREQ_HET=0.0002910078572121447;FREQ_HOMALT=0.0;FREQ_HOMREF=0.9997089921427879;N_BI_GENOS=10309;East_Han_AN=6554;East_Han_AC=3;East_Han_AF=
chr3	8704579	.	A	<INS:ME:LINE1>	.	PASS	SVTYPE=INS:ME:LINE1;END=8704595;SVLEN=710;AN=20618;AC=5;AF=0.00024250654767678728;N_HET=5;N_HOMALT=0;N_HOMREF=10304;FREQ_HET=0.00048501309535357455;FREQ_HOMALT=0.0;FREQ_HOMREF=0.9995149869046465;N_BI_GENOS=10309;South_Han_AN=1496;South_Han_AC=1;South_Han_
chr3	8791164	.	A	<INS:ME:LINE1>	.	PASS	SVTYPE=INS:ME:LINE1;END=8791181;SVLEN=1970;AN=20618;AC=1;AF=4.850130953535746e-05;N_HET=1;N_HOMALT=0;N_HOMREF=10308;FREQ_HET=9.700261907071492e-05;FREQ_HOMALT=0.0;FREQ_HOMREF=0.9999029973809292;N_BI_GENOS=10309;Central_Han_AN=2128;Central_Han_AC=1;Central
chr3	9031874	.	A	<INS:ME:LINE1>	.	PASS	SVTYPE=INS:ME:LINE1;END=9031880;SVLEN=520;AN=20618;AC=30;AF=0.0014550392860607237;N_HET=30;N_HOMALT=0;N_HOMREF=10279;FREQ_HET=0.0029100785721214474;FREQ_HOMALT=0.0;FREQ_HOMREF=0.9970899214278786;N_BI_GENOS=10309;South_Han_AN=1496;South_Han_AC=1;South_Han_
chr3	9070258	.	A	<INS:ME:LINE1>	.	PASS	SVTYPE=INS:ME:LINE1;END=9070273;SVLEN=247;AN=20618;AC=2;AF=9.700261907071492e-05;N_HET=2;N_HOMALT=0;N_HOMREF=10307;FREQ_HET=0.00019400523814142983;FREQ_HOMALT=0.0;FREQ_HOMREF=0.9998059947618586;N_BI_GENOS=10309;Lingnan_Han_AN=1888;Lingnan_Han_AC=1;Lingnan
chr3	9246014	.	A	<INS:ME:LINE1>	.	PASS	SVTYPE=INS:ME:LINE1;END=9246024;SVLEN=5997;AN=20618;AC=1;AF=4.850130953535746e-05;N_HET=1;N_HOMALT=0;N_HOMREF=10308;FREQ_HET=9.700261907071492e-05;FREQ_HOMALT=0.0;FREQ_HOMREF=0.9999029973809292;N_BI_GENOS=10309;East_Han_AN=6554;East_Han_AC=1;East_Han_AF=0
chr3	10639199	.	A	<INS:ME:LINE1>	.	PASS	SVTYPE=INS:ME:LINE1;END=10639210;SVLEN=1647;AN=20618;AC=1;AF=4.850130953535746e-05;N_HET=1;N_HOMALT=0;N_HOMREF=10308;FREQ_HET=9.700261907071492e-05;FREQ_HOMALT=0.0;FREQ_HOMREF=0.9999029973809292;N_BI_GENOS=10309;East_Han_AN=6554;East_Han_AC=1;East_Han_AF=
chr3	11029991	.	A	<INS:ME:LINE1>	.	PASS	SVTYPE=INS:ME:LINE1;END=11029991;SVLEN=0;AN=20618;AC=1;AF=4.850130953535746e-05;N_HET=1;N_HOMALT=0;N_HOMREF=10308;FREQ_HET=9.700261907071492e-05;FREQ_HOMALT=0.0;FREQ_HOMREF=0.9999029973809292;N_BI_GENOS=10309;South_Han_AN=1496;South_Han_AC=1;South_Han_AF=
chr3	11100605	.	T	<INS:ME:LINE1>	.	PASS	SVTYPE=INS:ME:LINE1;END=11100635;SVLEN=578;AN=20618;AC=1;AF=4.850130953535746e-05;N_HET=1;N_HOMALT=0;N_HOMREF=10308;FREQ_HET=9.700261907071492e-05;FREQ_HOMALT=0.0;FREQ_HOMREF=0.9999029973809292;N_BI_GENOS=10309;East_Han_AN=6554;East_Han_AC=1;East_Han_AF=0
chr3	11122891	.	T	<INS:ME:LINE1>	.	PASS	SVTYPE=INS:ME:LINE1;END=11122892;SVLEN=74;AN=20618;AC=1;AF=4.850130953535746e-05;N_HET=1;N_HOMALT=0;N_HOMREF=10308;FREQ_HET=9.700261907071492e-05;FREQ_HOMALT=0.0;FREQ_HOMREF=0.9999029973809292;N_BI_GENOS=10309;East_Han_AN=6554;East_Han_AC=1;East_Han_AF=0.
chr3	12052713	.	A	<INS:ME:LINE1>	.	PASS	SVTYPE=INS:ME:LINE1;END=12052725;SVLEN=2287;AN=20618;AC=1;AF=4.850130953535746e-05;N_HET=1;N_HOMALT=0;N_HOMREF=10308;FREQ_HET=9.700261907071492e-05;FREQ_HOMALT=0.0;FREQ_HOMREF=0.9999029973809292;N_BI_GENOS=10309;Northwest_Han_AN=1296;Northwest_Han_AC=1;No
chr3	12325882	.	A	<INS:ME:LINE1>	.	PASS	SVTYPE=INS:ME:LINE1;END=12325899;SVLEN=6021;AN=20618;AC=1;AF=4.850130953535746e-05;N_HET=1;N_HOMALT=0;N_HOMREF=10308;FREQ_HET=9.700261907071492e-05;FREQ_HOMALT=0.0;FREQ_HOMREF=0.9999029973809292;N_BI_GENOS=10309;East_Han_AN=6554;East_Han_AC=1;East_Han_AF=
chr3	14174320	.	A	<INS:ME:LINE1>	.	PASS	SVTYPE=INS:ME:LINE1;END=14174328;SVLEN=2105;AN=20618;AC=3;AF=0.00014550392860607236;N_HET=3;N_HOMALT=0;N_HOMREF=10306;FREQ_HET=0.0002910078572121447;FREQ_HOMALT=0.0;FREQ_HOMREF=0.9997089921427879;N_BI_GENOS=10309;Northwest_Han_AN=1296;Northwest_Han_AC=1;N
chr3	15145743	.	G	<INS:ME:LINE1>	.	PASS	SVTYPE=INS:ME:LINE1;END=15145743;SVLEN=0;AN=20618;AC=5;AF=0.00024250654767678728;N_HET=5;N_HOMALT=0;N_HOMREF=10304;FREQ_HET=0.00048501309535357455;FREQ_HOMALT=0.0;FREQ_HOMREF=0.9995149869046465;N_BI_GENOS=10309;North_Han_AN=2272;North_Han_AC=3;North_Han_A
chr3	15914195	.	C	<INS:ME:LINE1>	.	PASS	SVTYPE=INS:ME:LINE1;END=15914211;SVLEN=1868;AN=20618;AC=145;AF=0.007032689882626831;N_HET=141;N_HOMALT=2;N_HOMREF=10166;FREQ_HET=0.013677369288970802;FREQ_HOMALT=0.00019400523814142983;FREQ_HOMREF=0.9861286254728878;N_BI_GENOS=10309;South_Han_AN=1496;Sout
chr3	16568203	.	G	<INS:ME:LINE1>	.	PASS	SVTYPE=INS:ME:LINE1;END=16568219;SVLEN=6017;AN=20618;AC=2;AF=9.700261907071492e-05;N_HET=2;N_HOMALT=0;N_HOMREF=10307;FREQ_HET=0.00019400523814142983;FREQ_HOMALT=0.0;FREQ_HOMREF=0.9998059947618586;N_BI_GENOS=10309;East_Han_AN=6554;East_Han_AC=1;East_Han_AF
chr3	16711782	.	A	<INS:ME:LINE1>	.	PASS	SVTYPE=INS:ME:LINE1;END=16711782;SVLEN=400;AN=20618;AC=1;AF=4.850130953535746e-05;N_HET=1;N_HOMALT=0;N_HOMREF=10308;FREQ_HET=9.700261907071492e-05;FREQ_HOMALT=0.0;FREQ_HOMREF=0.9999029973809292;N_BI_GENOS=10309;East_Han_AN=6554;East_Han_AC=1;East_Han_AF=0
chr3	16712553	.	C	<INS:ME:LINE1>	.	PASS	SVTYPE=INS:ME:LINE1;END=16712553;SVLEN=1283;AN=20618;AC=1;AF=4.850130953535746e-05;N_HET=1;N_HOMALT=0;N_HOMREF=10308;FREQ_HET=9.700261907071492e-05;FREQ_HOMALT=0.0;FREQ_HOMREF=0.9999029973809292;N_BI_GENOS=10309;East_Han_AN=6554;East_Han_AC=1;East_Han_AF=
chr3	17300778	.	A	<INS:ME:LINE1>	.	PASS	SVTYPE=INS:ME:LINE1;END=17300780;SVLEN=946;AN=20618;AC=1;AF=4.850130953535746e-05;N_HET=1;N_HOMALT=0;N_HOMREF=10308;FREQ_HET=9.700261907071492e-05;FREQ_HOMALT=0.0;FREQ_HOMREF=0.9999029973809292;N_BI_GENOS=10309;Tibetan_AN=414;Tibetan_AC=1;Tibetan_AF=0.002
chr3	17395879	.	G	<INS:ME:LINE1>	.	PASS	SVTYPE=INS:ME:LINE1;END=17395895;SVLEN=6002;AN=20618;AC=1;AF=4.850130953535746e-05;N_HET=1;N_HOMALT=0;N_HOMREF=10308;FREQ_HET=9.700261907071492e-05;FREQ_HOMALT=0.0;FREQ_HOMREF=0.9999029973809292;N_BI_GENOS=10309;East_Han_AN=6554;East_Han_AC=1;East_Han_AF=
chr3	17626230	.	A	<INS:ME:LINE1>	.	PASS	SVTYPE=INS:ME:LINE1;END=17626230;SVLEN=5978;AN=20618;AC=1;AF=4.850130953535746e-05;N_HET=1;N_HOMALT=0;N_HOMREF=10308;FREQ_HET=9.700261907071492e-05;FREQ_HOMALT=0.0;FREQ_HOMREF=0.9999029973809292;N_BI_GENOS=10309;North_Han_AN=2272;North_Han_AC=1;North_Han_
chr3	17842908	.	T	<INS:ME:LINE1>	.	PASS	SVTYPE=INS:ME:LINE1;END=17842924;SVLEN=6001;AN=20618;AC=1;AF=4.850130953535746e-05;N_HET=1;N_HOMALT=0;N_HOMREF=10308;FREQ_HET=9.700261907071492e-05;FREQ_HOMALT=0.0;FREQ_HOMREF=0.9999029973809292;N_BI_GENOS=10309;Southeast_Han_AN=1908;Southeast_Han_AC=1;So
chr3	18092716	.	G	<INS:ME:LINE1>	.	PASS	SVTYPE=INS:ME:LINE1;END=18092732;SVLEN=5979;AN=20618;AC=44;AF=0.002134057619555728;N_HET=38;N_HOMALT=3;N_HOMREF=10268;FREQ_HET=0.0036860995246871665;FREQ_HOMALT=0.0002910078572121447;FREQ_HOMREF=0.9960228926181007;N_BI_GENOS=10309;South_Han_AN=1496;South_
chr3	18305645	.	G	<INS:ME:LINE1>	.	PASS	SVTYPE=INS:ME:LINE1;END=18305660;SVLEN=556;AN=20618;AC=1;AF=4.850130953535746e-05;N_HET=1;N_HOMALT=0;N_HOMREF=10308;FREQ_HET=9.700261907071492e-05;FREQ_HOMALT=0.0;FREQ_HOMREF=0.9999029973809292;N_BI_GENOS=10309;East_Han_AN=6554;East_Han_AC=1;East_Han_AF=0
chr3	18588946	.	T	<INS:ME:LINE1>	.	PASS	SVTYPE=INS:ME:LINE1;END=18588960;SVLEN=6009;AN=20618;AC=12;AF=0.0005820157144242894;N_HET=12;N_HOMALT=0;N_HOMREF=10297;FREQ_HET=0.0011640314288485789;FREQ_HOMALT=0.0;FREQ_HOMREF=0.9988359685711514;N_BI_GENOS=10309;South_Han_AN=1496;South_Han_AC=2;South_Ha
chr3	19340482	.	T	<INS:ME:LINE1>	.	PASS	SVTYPE=INS:ME:LINE1;END=19340482;SVLEN=264;AN=20618;AC=1;AF=4.850130953535746e-05;N_HET=1;N_HOMALT=0;N_HOMREF=10308;FREQ_HET=9.700261907071492e-05;FREQ_HOMALT=0.0;FREQ_HOMREF=0.9999029973809292;N_BI_GENOS=10309;East_Han_AN=6554;East_Han_AC=1;East_Han_AF=0
chr3	19481455	.	A	<INS:ME:LINE1>	.	PASS	SVTYPE=INS:ME:LINE1;END=19481472;SVLEN=5997;AN=20618;AC=2;AF=9.700261907071492e-05;N_HET=2;N_HOMALT=0;N_HOMREF=10307;FREQ_HET=0.00019400523814142983;FREQ_HOMALT=0.0;FREQ_HOMREF=0.9998059947618586;N_BI_GENOS=10309;South_Han_AN=1496;South_Han_AC=1;South_Han
chr3	19484161	.	A	<INS:ME:LINE1>	.	PASS	SVTYPE=INS:ME:LINE1;END=19484168;SVLEN=1899;AN=20618;AC=1;AF=4.850130953535746e-05;N_HET=1;N_HOMALT=0;N_HOMREF=10308;FREQ_HET=9.700261907071492e-05;FREQ_HOMALT=0.0;FREQ_HOMREF=0.9999029973809292;N_BI_GENOS=10309;South_Han_AN=1496;South_Han_AC=1;South_Han_
chr3	20049024	.	C	<INS:ME:LINE1>	.	PASS	SVTYPE=INS:ME:LINE1;END=20049041;SVLEN=5997;AN=20618;AC=12;AF=0.0005820157144242894;N_HET=12;N_HOMALT=0;N_HOMREF=10297;FREQ_HET=0.0011640314288485789;FREQ_HOMALT=0.0;FREQ_HOMREF=0.9988359685711514;N_BI_GENOS=10309;South_Han_AN=1496;South_Han_AC=2;South_Ha
chr3	20222907	.	T	<INS:ME:LINE1>	.	PASS	SVTYPE=INS:ME:LINE1;END=20222921;SVLEN=5993;AN=20618;AC=58;AF=0.0028130759530507325;N_HET=58;N_HOMALT=0;N_HOMREF=10251;FREQ_HET=0.005626151906101465;FREQ_HOMALT=0.0;FREQ_HOMREF=0.9943738480938985;N_BI_GENOS=10309;South_Han_AN=1496;South_Han_AC=6;South_Han
chr3	20407824	.	T	<INS:ME:LINE1>	.	PASS	SVTYPE=INS:ME:LINE1;END=20407840;SVLEN=407;AN=20618;AC=1;AF=4.850130953535746e-05;N_HET=1;N_HOMALT=0;N_HOMREF=10308;FREQ_HET=9.700261907071492e-05;FREQ_HOMALT=0.0;FREQ_HOMREF=0.9999029973809292;N_BI_GENOS=10309;Central_Han_AN=2128;Central_Han_AC=1;Central
chr3	20505385	.	G	<INS:ME:LINE1>	.	PASS	SVTYPE=INS:ME:LINE1;END=20505385;SVLEN=278;AN=20618;AC=1;AF=4.850130953535746e-05;N_HET=1;N_HOMALT=0;N_HOMREF=10308;FREQ_HET=9.700261907071492e-05;FREQ_HOMALT=0.0;FREQ_HOMREF=0.9999029973809292;N_BI_GENOS=10309;East_Han_AN=6554;East_Han_AC=1;East_Han_AF=0
chr3	20707403	.	G	<INS:ME:LINE1>	.	PASS	SVTYPE=INS:ME:LINE1;END=20707412;SVLEN=6000;AN=20618;AC=616;AF=0.029876806673780194;N_HET=592;N_HOMALT=12;N_HOMREF=9705;FREQ_HET=0.057425550489863225;FREQ_HOMALT=0.0011640314288485789;FREQ_HOMREF=0.9414104180812882;N_BI_GENOS=10309;South_Han_AN=1496;South
chr3	21101158	.	A	<INS:ME:LINE1>	.	PASS	SVTYPE=INS:ME:LINE1;END=21101179;SVLEN=922;AN=20618;AC=1;AF=4.850130953535746e-05;N_HET=1;N_HOMALT=0;N_HOMREF=10308;FREQ_HET=9.700261907071492e-05;FREQ_HOMALT=0.0;FREQ_HOMREF=0.9999029973809292;N_BI_GENOS=10309;East_Han_AN=6554;East_Han_AC=1;East_Han_AF=0
chr3	21193422	.	A	<INS:ME:LINE1>	.	PASS	SVTYPE=INS:ME:LINE1;END=21193435;SVLEN=683;AN=20618;AC=80;AF=0.0038801047628285964;N_HET=74;N_HOMALT=3;N_HOMREF=10232;FREQ_HET=0.007178193811232903;FREQ_HOMALT=0.0002910078572121447;FREQ_HOMREF=0.992530798331555;N_BI_GENOS=10309;South_Han_AN=1496;South_Ha
chr3	21227964	.	T	<INS:ME:LINE1>	.	PASS	SVTYPE=INS:ME:LINE1;END=21227985;SVLEN=6002;AN=20618;AC=8;AF=0.00038801047628285966;N_HET=8;N_HOMALT=0;N_HOMREF=10301;FREQ_HET=0.0007760209525657193;FREQ_HOMALT=0.0;FREQ_HOMREF=0.9992239790474343;N_BI_GENOS=10309;South_Han_AN=1496;South_Han_AC=1;South_Han
chr3	21624942	.	A	<INS:ME:LINE1>	.	PASS	SVTYPE=INS:ME:LINE1;END=21624955;SVLEN=5977;AN=20618;AC=1;AF=4.850130953535746e-05;N_HET=1;N_HOMALT=0;N_HOMREF=10308;FREQ_HET=9.700261907071492e-05;FREQ_HOMALT=0.0;FREQ_HOMREF=0.9999029973809292;N_BI_GENOS=10309;Southeast_Han_AN=1908;Southeast_Han_AC=1;So
chr3	21678752	.	T	<INS:ME:LINE1>	.	PASS	SVTYPE=INS:ME:LINE1;END=21678767;SVLEN=395;AN=20618;AC=1;AF=4.850130953535746e-05;N_HET=1;N_HOMALT=0;N_HOMREF=10308;FREQ_HET=9.700261907071492e-05;FREQ_HOMALT=0.0;FREQ_HOMREF=0.9999029973809292;N_BI_GENOS=10309;Manchu_AN=416;Manchu_AC=1;Manchu_AF=0.002403
chr3	21945707	.	A	<INS:ME:LINE1>	.	PASS	SVTYPE=INS:ME:LINE1;END=21945708;SVLEN=1257;AN=20618;AC=1;AF=4.850130953535746e-05;N_HET=1;N_HOMALT=0;N_HOMREF=10308;FREQ_HET=9.700261907071492e-05;FREQ_HOMALT=0.0;FREQ_HOMREF=0.9999029973809292;N_BI_GENOS=10309;Central_Han_AN=2128;Central_Han_AC=1;Centra
chr3	22144136	.	C	<INS:ME:LINE1>	.	PASS	SVTYPE=INS:ME:LINE1;END=22144154;SVLEN=3077;AN=20618;AC=1;AF=4.850130953535746e-05;N_HET=1;N_HOMALT=0;N_HOMREF=10308;FREQ_HET=9.700261907071492e-05;FREQ_HOMALT=0.0;FREQ_HOMREF=0.9999029973809292;N_BI_GENOS=10309;North_Han_AN=2272;North_Han_AC=1;North_Han_
chr3	22363672	.	A	<INS:ME:LINE1>	.	PASS	SVTYPE=INS:ME:LINE1;END=22363688;SVLEN=6005;AN=20618;AC=1;AF=4.850130953535746e-05;N_HET=1;N_HOMALT=0;N_HOMREF=10308;FREQ_HET=9.700261907071492e-05;FREQ_HOMALT=0.0;FREQ_HOMREF=0.9999029973809292;N_BI_GENOS=10309;North_Han_AN=2272;North_Han_AC=1;North_Han_
chr3	22786287	.	A	<INS:ME:LINE1>	.	PASS	SVTYPE=INS:ME:LINE1;END=22786287;SVLEN=3796;AN=20618;AC=1;AF=4.850130953535746e-05;N_HET=1;N_HOMALT=0;N_HOMREF=10308;FREQ_HET=9.700261907071492e-05;FREQ_HOMALT=0.0;FREQ_HOMREF=0.9999029973809292;N_BI_GENOS=10309;Central_Han_AN=2128;Central_Han_AC=1;Centra
chr3	22884576	.	A	<INS:ME:LINE1>	.	PASS	SVTYPE=INS:ME:LINE1;END=22884593;SVLEN=6000;AN=20618;AC=1;AF=4.850130953535746e-05;N_HET=1;N_HOMALT=0;N_HOMREF=10308;FREQ_HET=9.700261907071492e-05;FREQ_HOMALT=0.0;FREQ_HOMREF=0.9999029973809292;N_BI_GENOS=10309;Zhuang_AN=444;Zhuang_AC=1;Zhuang_AF=0.00225
chr3	23214632	.	G	<INS:ME:LINE1>	.	PASS	SVTYPE=INS:ME:LINE1;END=23214648;SVLEN=5998;AN=20618;AC=1;AF=4.850130953535746e-05;N_HET=1;N_HOMALT=0;N_HOMREF=10308;FREQ_HET=9.700261907071492e-05;FREQ_HOMALT=0.0;FREQ_HOMREF=0.9999029973809292;N_BI_GENOS=10309;Lingnan_Han_AN=1888;Lingnan_Han_AC=1;Lingna
chr3	23569507	.	A	<INS:ME:LINE1>	.	PASS	SVTYPE=INS:ME:LINE1;END=23569519;SVLEN=5138;AN=20618;AC=1;AF=4.850130953535746e-05;N_HET=1;N_HOMALT=0;N_HOMREF=10308;FREQ_HET=9.700261907071492e-05;FREQ_HOMALT=0.0;FREQ_HOMREF=0.9999029973809292;N_BI_GENOS=10309;East_Han_AN=6554;East_Han_AC=1;East_Han_AF=
chr3	23804904	.	A	<INS:ME:LINE1>	.	PASS	SVTYPE=INS:ME:LINE1;END=23804917;SVLEN=6000;AN=20618;AC=1;AF=4.850130953535746e-05;N_HET=1;N_HOMALT=0;N_HOMREF=10308;FREQ_HET=9.700261907071492e-05;FREQ_HOMALT=0.0;FREQ_HOMREF=0.9999029973809292;N_BI_GENOS=10309;North_Han_AN=2272;North_Han_AC=1;North_Han_
chr3	24184113	.	T	<INS:ME:LINE1>	.	PASS	SVTYPE=INS:ME:LINE1;END=24184131;SVLEN=216;AN=20618;AC=1;AF=4.850130953535746e-05;N_HET=1;N_HOMALT=0;N_HOMREF=10308;FREQ_HET=9.700261907071492e-05;FREQ_HOMALT=0.0;FREQ_HOMREF=0.9999029973809292;N_BI_GENOS=10309;Lingnan_Han_AN=1888;Lingnan_Han_AC=1;Lingnan

chr3	24349817	.	A	<INS:ME:LINE1>	.	PASS	SVTYPE=INS:ME:LINE1;END=24349832;SVLEN=6000;AN=20618;AC=2;AF=9.700261907071492e-05;N_HET=2;N_HOMALT=0;N_HOMREF=10307;FREQ_HET=0.0001940052381414298 3;FREQ_HOMALT=0.0;FREQ_HOMREF=0.9998059947618586;N_BI_GENOS=10309;East_Han_AN=6554;East_Han_AC=2;East_Han_AF
chr3	24350109	.	A	<INS:ME:LINE1>	.	PASS	SVTYPE=INS:ME:LINE1;END=24350127;SVLEN=1146;AN=20618;AC=3;AF=0.00014550392860607236;N_HET=3;N_HOMALT=0;N_HOMREF=10306;FREQ_HET=0.0002910078572121447;FREQ_HOMALT=0.0;FREQ_HOMREF=0.9997089921427879;N_BI_GENOS=10309;South_Han_AN=1496;South_Han_AC=2;South_Han
chr3	24414929	.	A	<INS:ME:LINE1>	.	PASS	SVTYPE=INS:ME:LINE1;END=24414929;SVLEN=113;AN=20618;AC=1;AF=4.850130953535746e-05;N_HET=1;N_HOMALT=0;N_HOMREF=10308;FREQ_HET=9.700261907071492e-05;FREQ_HOMALT=0.0;FREQ_HOMREF=0.9999029973809292;N_BI_GENOS=10309;East_Han_AN=6554;East_Han_AC=1;East_Han_AF=0
chr3	24509663	.	A	<INS:ME:LINE1>	.	PASS	SVTYPE=INS:ME:LINE1;END=24509671;SVLEN=41;AN=20618;AC=2;AF=9.700261907071492e-05;N_HET=2;N_HOMALT=0;N_HOMREF=10307;FREQ_HET=0.0001940052381414298 3;FREQ_HOMALT=0.0;FREQ_HOMREF=0.9998059947618586;N_BI_GENOS=10309;East_Han_AN=6554;East_Han_AC=2;East_Han_AF=0
chr3	24946366	.	C	<INS:ME:LINE1>	.	PASS	SVTYPE=INS:ME:LINE1;END=24946383;SVLEN=2901;AN=20618;AC=6989;AF=0.33897565234261323;N_HET=4331;N_HOMALT=1329;N_HOMREF=4649;FREQ_HET=0.42011834319526625;FREQ_HOMALT=0.1289164807449801;FREQ_HOMREF=0.4509651760597536;N_BI_GENOS=10309;South_Han_AN=1496;South_
chr3	26123507	.	C	<INS:ME:LINE1>	.	PASS	SVTYPE=INS:ME:LINE1;END=26123520;SVLEN=970;AN=20618;AC=1;AF=4.850130953535746e-05;N_HET=1;N_HOMALT=0;N_HOMREF=10308;FREQ_HET=9.700261907071492e-05;FREQ_HOMALT=0.0;FREQ_HOMREF=0.9999029973809292;N_BI_GENOS=10309;East_Han_AN=6554;East_Han_AC=1;East_Han_AF=0
chr3	26153570	.	T	<INS:ME:LINE1>	.	PASS	SVTYPE=INS:ME:LINE1;END=26153588;SVLEN=957;AN=20618;AC=1;AF=4.850130953535746e-05;N_HET=1;N_HOMALT=0;N_HOMREF=10308;FREQ_HET=9.700261907071492e-05;FREQ_HOMALT=0.0;FREQ_HOMREF=0.9999029973809292;N_BI_GENOS=10309;East_Han_AN=6554;East_Han_AC=1;East_Han_AF=0
chr3	26482955	.	A	<INS:ME:LINE1>	.	PASS	SVTYPE=INS:ME:LINE1;END=26482973;SVLEN=1305;AN=20618;AC=4806;AF=0.23309729362692794;N_HET=3270;N_HOMALT=768;N_HOMREF=6271;FREQ_HET=0.3171985643612378;FREQ_HOMALT=0.07449801144630905;FREQ_HOMREF=0.6083034241924532;N_BI_GENOS=10309;South_Han_AN=1496;South_H
chr3	26486112	.	C	<INS:ME:LINE1>	.	PASS	SVTYPE=INS:ME:LINE1;END=26486112;SVLEN=45;AN=20618;AC=3;AF=0.00014550392860607236;N_HET=3;N_HOMALT=0;N_HOMREF=10306;FREQ_HET=0.0002910078572121447;FREQ_HOMALT=0.0;FREQ_HOMREF=0.9997089921427879;N_BI_GENOS=10309;Central_Han_AN=2128;Central_Han_AC=3;Central
chr3	26486367	.	T	<INS:ME:LINE1>	.	PASS	SVTYPE=INS:ME:LINE1;END=26486367;SVLEN=45;AN=20618;AC=3;AF=0.00014550392860607236;N_HET=3;N_HOMALT=0;N_HOMREF=10306;FREQ_HET=0.0002910078572121447;FREQ_HOMALT=0.0;FREQ_HOMREF=0.9997089921427879;N_BI_GENOS=10309;Central_Han_AN=2128;Central_Han_AC=3;Central
chr3	26700343	.	A	<INS:ME:LINE1>	.	PASS	SVTYPE=INS:ME:LINE1;END=26700358;SVLEN=712;AN=20618;AC=1;AF=4.850130953535746e-05;N_HET=1;N_HOMALT=0;N_HOMREF=10308;FREQ_HET=9.700261907071492e-05;FREQ_HOMALT=0.0;FREQ_HOMREF=0.9999029973809292;N_BI_GENOS=10309;East_Han_AN=6554;East_Han_AC=1;East_Han_AF=0
chr3	26804547	.	C	<INS:ME:LINE1>	.	PASS	SVTYPE=INS:ME:LINE1;END=26804561;SVLEN=13;AN=20618;AC=1;AF=4.850130953535746e-05;N_HET=1;N_HOMALT=0;N_HOMREF=10308;FREQ_HET=9.700261907071492e-05;FREQ_HOMALT=0.0;FREQ_HOMREF=0.9999029973809292;N_BI_GENOS=10309;East_Han_AN=6554;East_Han_AC=1;East_Han_AF=0.
chr3	26864583	.	A	<INS:ME:LINE1>	.	PASS	SVTYPE=INS:ME:LINE1;END=26864596;SVLEN=2137;AN=20618;AC=8;AF=0.0003880104762828 5966;N_HET=8;N_HOMALT=0;N_HOMREF=10301;FREQ_HET=0.0007760209525657193;FREQ_HOMALT=0.0;FREQ_HOMREF=0.9992239790474343;N_BI_GENOS=10309;Lingnan_Han_AN=1888;Lingnan_Han_AC=1;Lingn
chr3	27199868	.	A	<INS:ME:LINE1>	.	PASS	SVTYPE=INS:ME:LINE1;END=27199884;SVLEN=3304;AN=20618;AC=36;AF=0.0017460471432728683;N_HET=34;N_HOMALT=1;N_HOMREF=10274;FREQ_HET=0.0032980890484043067;FREQ_HOMALT=9.700261907071492e-05;FREQ_HOMREF=0.9966049083325 25;N_BI_GENOS=10309;South_Han_AN=1496;South_
chr3	28220479	.	C	<INS:ME:LINE1>	.	PASS	SVTYPE=INS:ME:LINE1;END=28220493;SVLEN=1146;AN=20618;AC=751;AF=0.03642448346105 3446;N_HET=695;N_HOMALT=28;N_HOMREF=9586;FREQ_HET=0.06741682025414686;FREQ_HOMALT=0.0027160733339800175;FREQ_HOMREF=0.9298671064118731;N_BI_GENOS=10309;South_Han_AN=1496;South_
chr3	28237045	.	T	<INS:ME:LINE1>	.	PASS	SVTYPE=INS:ME:LINE1;END=28237062;SVLEN=726;AN=20618;AC=1;AF=4.850130953535746e-05;N_HET=1;N_HOMALT=0;N_HOMREF=10308;FREQ_HET=9.700261907071492e-05;FREQ_HOMALT=0.0;FREQ_HOMREF=0.9999029973809292;N_BI_GENOS=10309;East_Han_AN=6554;East_Han_AC=1;East_Han_AF=0
chr3	28615215	.	A	<INS:ME:LINE1>	.	PASS	SVTYPE=INS:ME:LINE1;END=28615215;SVLEN=3334;AN=20618;AC=1;AF=4.850130953535746e-05;N_HET=1;N_HOMALT=0;N_HOMREF=10308;FREQ_HET=9.700261907071492e-05;FREQ_HOMALT=0.0;FREQ_HOMREF=0.9999029973809292;N_BI_GENOS=10309;Central_Han_AN=2128;Central_Han_AC=1;Centra
chr3	28631670	.	C	<INS:ME:LINE1>	.	PASS	SVTYPE=INS:ME:LINE1;END=28631683;SVLEN=6002;AN=20618;AC=1;AF=4.850130953535746e-05;N_HET=1;N_HOMALT=0;N_HOMREF=10308;FREQ_HET=9.700261907071492e-05;FREQ_HOMALT=0.0;FREQ_HOMREF=0.9999029973809292;N_BI_GENOS=10309;Tibetan_AN=414;Tibetan_AC=1;Tibetan_AF=0.00
chr3	28731613	.	A	<INS:ME:LINE1>	.	PASS	SVTYPE=INS:ME:LINE1;END=28731616;SVLEN=4097;AN=20618;AC=1;AF=4.850130953535746e-05;N_HET=1;N_HOMALT=0;N_HOMREF=10308;FREQ_HET=9.700261907071492e-05;FREQ_HOMALT=0.0;FREQ_HOMREF=0.9999029973809292;N_BI_GENOS=10309;Zhuang_AN=444;Zhuang_AC=1;Zhuang_AF=0.00225
chr3	28944959	.	T	<INS:ME:LINE1>	.	PASS	SVTYPE=INS:ME:LINE1;END=28944975;SVLEN=421;AN=20618;AC=4;AF=0.00019400523814142983;N_HET=4;N_HOMALT=0;N_HOMREF=10305;FREQ_HET=0.00038801047628285966;FREQ_HOMALT=0.0;FREQ_HOMREF=0.9996119895237171;N_BI_GENOS=10309;Northwest_Han_AN=1296;Northwest_Han_AC=1;N
chr3	29095797	.	G	<INS:ME:LINE1>	.	PASS	SVTYPE=INS:ME:LINE1;END=29095797;SVLEN=2450;AN=20618;AC=1;AF=4.850130953535746e-05;N_HET=1;N_HOMALT=0;N_HOMREF=10308;FREQ_HET=9.700261907071492e-05;FREQ_HOMALT=0.0;FREQ_HOMREF=0.9999029973809292;N_BI_GENOS=10309;East_Han_AN=6554;East_Han_AC=1;East_Han_AF=
chr3	29271839	.	C	<INS:ME:LINE1>	.	PASS	SVTYPE=INS:ME:LINE1;END=29271839;SVLEN=533;AN=20618;AC=2;AF=9.700261907071492e-05;N_HET=2;N_HOMALT=0;N_HOMREF=10307;FREQ_HET=0.00019400523814142983;FREQ_HOMALT=0.0;FREQ_HOMREF=0.9998059947618586;N_BI_GENOS=10309;East_Han_AN=6554;East_Han_AC=2;East_Han_AF=
chr3	29631093	.	A	<INS:ME:LINE1>	.	PASS	SVTYPE=INS:ME:LINE1;END=29631103;SVLEN=6017;AN=20618;AC=2;AF=9.700261907071492e-05;N_HET=2;N_HOMALT=0;N_HOMREF=10307;FREQ_HET=0.00019400523814142983;FREQ_HOMALT=0.0;FREQ_HOMREF=0.9998059947618586;N_BI_GENOS=10309;Central_Han_AN=2128;Central_Han_AC=2;Centr
chr3	30016239	.	G	<INS:ME:LINE1>	.	PASS	SVTYPE=INS:ME:LINE1;END=30016254;SVLEN=1048;AN=20618;AC=5;AF=0.0002425065476767 8728;N_HET=5;N_HOMALT=0;N_HOMREF=10304;FREQ_HET=0.00048501309535357455;FREQ_HOMALT=0.0;FREQ_HOMREF=0.9995149869046465;N_BI_GENOS=10309;North_Han_AN=2272;North_Han_AC=1;North_Ha
chr3	30124853	.	T	<INS:ME:LINE1>	.	PASS	SVTYPE=INS:ME:LINE1;END=30124867;SVLEN=5993;AN=20618;AC=1;AF=4.850130953535746e-05;N_HET=1;N_HOMALT=0;N_HOMREF=10308;FREQ_HET=9.700261907071492e-05;FREQ_HOMALT=0.0;FREQ_HOMREF=0.9999029973809292;N_BI_GENOS=10309;Mongolian_AN=454;Mongolian_AC=1;Mongolian_A
chr3	30199239	.	A	<INS:ME:LINE1>	.	PASS	SVTYPE=INS:ME:LINE1;END=30199252;SVLEN=5949;AN=20618;AC=1;AF=4.850130953535746e-05;N_HET=1;N_HOMALT=0;N_HOMREF=10308;FREQ_HET=9.700261907071492e-05;FREQ_HOMALT=0.0;FREQ_HOMREF=0.9999029973809292;N_BI_GENOS=10309;Northwest_Han_AN=1296;Northwest_Han_AC=1;No
chr3	30250861	.	G	<INS:ME:LINE1>	.	PASS	SVTYPE=INS:ME:LINE1;END=30250861;SVLEN=435;AN=20618;AC=1;AF=4.850130953535746e-05;N_HET=1;N_HOMALT=0;N_HOMREF=10308;FREQ_HET=9.700261907071492e-05;FREQ_HOMALT=0.0;FREQ_HOMREF=0.9999029973809292;N_BI_GENOS=10309;Central_Han_AN=2128;Central_Han_AC=1;Central
chr3	30377890	.	G	<INS:ME:LINE1>	.	PASS	SVTYPE=INS:ME:LINE1;END=30377903;SVLEN=5998;AN=20618;AC=3;AF=0.00014550392860607236;N_HET=3;N_HOMALT=0;N_HOMREF=10306;FREQ_HET=0.0002910078572121447;FREQ_HOMALT=0.0;FREQ_HOMREF=0.9997089921427879;N_BI_GENOS=10309;Northwest_Han_AN=1296;Northwest_Han_AC=1;N
chr3	30408244	.	A	<INS:ME:LINE1>	.	PASS	SVTYPE=INS:ME:LINE1;END=30408261;SVLEN=261;AN=20618;AC=1;AF=4.850130953535746e-05;N_HET=1;N_HOMALT=0;N_HOMREF=10308;FREQ_HET=9.700261907071492e-05;FREQ_HOMALT=0.0;FREQ_HOMREF=0.9999029973809292;N_BI_GENOS=10309;North_Han_AN=2272;North_Han_AC=1;North_Han_A
chr3	30427334	.	C	<INS:ME:LINE1>	.	PASS	SVTYPE=INS:ME:LINE1;END=30427349;SVLEN=5986;AN=20618;AC=2;AF=9.700261907071492e-05;N_HET=2;N_HOMALT=0;N_HOMREF=10307;FREQ_HET=0.00019400523814142983;FREQ_HOMALT=0.0;FREQ_HOMREF=0.9998059947618586;N_BI_GENOS=10309;South_Han_AN=1496;South_Han_AC=1;South_Han
chr3	30587620	.	G	<INS:ME:LINE1>	.	PASS	SVTYPE=INS:ME:LINE1;END=30587636;SVLEN=4452;AN=20618;AC=2;AF=9.700261907071492e-05;N_HET=2;N_HOMALT=0;N_HOMREF=10307;FREQ_HET=0.00019400523814142983;FREQ_HOMALT=0.0;FREQ_HOMREF=0.9998059947618586;N_BI_GENOS=10309;Lingnan_Han_AN=1888;Lingnan_Han_AC=1;Lingn
chr3	30771656	.	A	<INS:ME:LINE1>	.	PASS	SVTYPE=INS:ME:LINE1;END=30771679;SVLEN=3670;AN=20618;AC=1;AF=4.850130953535746e-05;N_HET=1;N_HOMALT=0;N_HOMREF=10308;FREQ_HET=9.700261907071492e-05;FREQ_HOMALT=0.0;FREQ_HOMREF=0.9999029973809292;N_BI_GENOS=10309;Zhuang_AN=444;Zhuang_AC=1;Zhuang_AF=0.00225
chr3	31065252	.	T	<INS:ME:LINE1>	.	PASS	SVTYPE=INS:ME:LINE1;END=31065271;SVLEN=376;AN=20618;AC=1;AF=4.850130953535746e-05;N_HET=1;N_HOMALT=0;N_HOMREF=10308;FREQ_HET=9.700261907071492e-05;FREQ_HOMALT=0.0;FREQ_HOMREF=0.9999029973809292;N_BI_GENOS=10309;North_Han_AN=2272;North_Han_AC=1;North_Han_A
chr3	31170526	.	T	<INS:ME:LINE1>	.	PASS	SVTYPE=INS:ME:LINE1;END=31170542;SVLEN=5996;AN=20618;AC=1;AF=4.850130953535746e-05;N_HET=1;N_HOMALT=0;N_HOMREF=10308;FREQ_HET=9.700261907071492e-05;FREQ_HOMALT=0.0;FREQ_HOMREF=0.9999029973809292;N_BI_GENOS=10309;Manchu_AN=416;Manchu_AC=1;Manchu_AF=0.00240
chr3	31275963	.	T	<INS:ME:LINE1>	.	PASS	SVTYPE=INS:ME:LINE1;END=31275975;SVLEN=1208;AN=20618;AC=1;AF=4.850130953535746e-05;N_HET=1;N_HOMALT=0;N_HOMREF=10308;FREQ_HET=9.700261907071492e-05;FREQ_HOMALT=0.0;FREQ_HOMREF=0.9999029973809292;N_BI_GENOS=10309;Lingnan_Han_AN=1888;Lingnan_Han_AC=1;Lingna
chr3	31359164	.	T	<INS:ME:LINE1>	.	PASS	SVTYPE=INS:ME:LINE1;END=31359164;SVLEN=11;AN=20618;AC=1;AF=4.850130953535746e-05;N_HET=1;N_HOMALT=0;N_HOMREF=10308;FREQ_HET=9.700261907071492e-05;FREQ_HOMALT=0.0;FREQ_HOMREF=0.9999029973809292;N_BI_GENOS=10309;Northwest_Han_AN=1296;Northwest_Han_AC=1;Nort
chr3	31423514	.	A	<INS:ME:LINE1>	.	PASS	SVTYPE=INS:ME:LINE1;END=31423529;SVLEN=1132;AN=20618;AC=1;AF=4.850130953535746e-05;N_HET=1;N_HOMALT=0;N_HOMREF=10308;FREQ_HET=9.700261907071492e-05;FREQ_HOMALT=0.0;FREQ_HOMREF=0.9999029973809292;N_BI_GENOS=10309;Central_Han_AN=2128;Central_Han_AC=1;Centra
chr3	31498269	.	G	<INS:ME:LINE1>	.	PASS	SVTYPE=INS:ME:LINE1;END=31498281;SVLEN=354;AN=20618;AC=1;AF=4.850130953535746e-05;N_HET=1;N_HOMALT=0;N_HOMREF=10308;FREQ_HET=9.700261907071492e-05;FREQ_HOMALT=0.0;FREQ_HOMREF=0.9999029973809292;N_BI_GENOS=10309;Southeast_Han_AN=1908;Southeast_Han_AC=1;Sou
chr3	31584365	.	T	<INS:ME:LINE1>	.	PASS	SVTYPE=INS:ME:LINE1;END=31584386;SVLEN=6008;AN=20618;AC=2;AF=9.700261907071492e-05;N_HET=2;N_HOMALT=0;N_HOMREF=10307;FREQ_HET=0.00019400523814142983;FREQ_HOMALT=0.0;FREQ_HOMREF=0.9998059947618586;N_BI_GENOS=10309;East_Han_AN=6554;East_Han_AC=1;East_Han_AF
chr3	31628642	.	C	<INS:ME:LINE1>	.	PASS	SVTYPE=INS:ME:LINE1;END=31628657;SVLEN=1871;AN=20618;AC=1;AF=4.850130953535746e-05;N_HET=1;N_HOMALT=0;N_HOMREF=10308;FREQ_HET=9.700261907071492e-05;FREQ_HOMALT=0.0;FREQ_HOMREF=0.9999029973809292;N_BI_GENOS=10309;Mongolian_AN=454;Mongolian_AC=1;Mongolian_A
chr3	31649200	.	G	<INS:ME:LINE1>	.	PASS	SVTYPE=INS:ME:LINE1;END=31649208;SVLEN=229;AN=20618;AC=1;AF=4.850130953535746e-05;N_HET=1;N_HOMALT=0;N_HOMREF=10308;FREQ_HET=9.700261907071492e-05;FREQ_HOMALT=0.0;FREQ_HOMREF=0.9999029973809292;N_BI_GENOS=10309;Lingnan_Han_AN=1888;Lingnan_Han_AC=1;Lingnan
chr3	32193180	.	C	<INS:ME:LINE1>	.	PASS	SVTYPE=INS:ME:LINE1;END=32193188;SVLEN=3652;AN=20618;AC=1;AF=4.850130953535746e-05;N_HET=1;N_HOMALT=0;N_HOMREF=10308;FREQ_HET=9.700261907071492e-05;FREQ_HOMALT=0.0;FREQ_HOMREF=0.9999029973809292;N_BI_GENOS=10309;Tibetan_AN=414;Tibetan_AC=1;Tibetan_AF=0.00
chr3	32215798	.	A	<INS:ME:LINE1>	.	PASS	SVTYPE=INS:ME:LINE1;END=32215813;SVLEN=1062;AN=20618;AC=1;AF=4.850130953535746e-05;N_HET=1;N_HOMALT=0;N_HOMREF=10308;FREQ_HET=9.700261907071492e-05;FREQ_HOMALT=0.0;FREQ_HOMREF=0.9999029973809292;N_BI_GENOS=10309;Southeast_Han_AN=1908;Southeast_Han_AC=1;So
chr3	33185950	.	A	<INS:ME:LINE1>	.	PASS	SVTYPE=INS:ME:LINE1;END=33185967;SVLEN=5993;AN=20618;AC=1;AF=4.850130953535746e-05;N_HET=1;N_HOMALT=0;N_HOMREF=10308;FREQ_HET=9.700261907071492e-05;FREQ_HOMALT=0.0;FREQ_HOMREF=0.9999029973809292;N_BI_GENOS=10309;Hui_AN=444;Hui_AC=1;Hui_AF=0.00225225225225
chr3	33856690	.	A	<INS:ME:LINE1>	.	PASS	SVTYPE=INS:ME:LINE1;END=33856706;SVLEN=6004;AN=20618;AC=1;AF=4.850130953535746e-05;N_HET=1;N_HOMALT=0;N_HOMREF=10308;FREQ_HET=9.700261907071492e-05;FREQ_HOMALT=0.0;FREQ_HOMREF=0.9999029973809292;N_BI_GENOS=10309;Lingnan_Han_AN=1888;Lingnan_Han_AC=1;Lingna
chr3	34053165	.	T	<INS:ME:LINE1>	.	PASS	SVTYPE=INS:ME:LINE1;END=34053177;SVLEN=6009;AN=20618;AC=2;AF=9.700261907071492e-05;N_HET=2;N_HOMALT=0;N_HOMREF=10307;FREQ_HET=0.00019400523814142983;FREQ_HOMALT=0.0;FREQ_HOMREF=0.9998059947618586;N_BI_GENOS=10309;East_Han_AN=6554;East_Han_AC=2;East_Han_AF
chr3	34135335	.	T	<INS:ME:LINE1>	.	PASS	SVTYPE=INS:ME:LINE1;END=34135335;SVLEN=162;AN=20618;AC=1;AF=4.850130953535746e-05;N_HET=1;N_HOMALT=0;N_HOMREF=10308;FREQ_HET=9.700261907071492e-05;FREQ_HOMALT=0.0;FREQ_HOMREF=0.9999029973809292;N_BI_GENOS=10309;East_Han_AN=6554;East_Han_AC=1;East_Han_AF=0
chr3	34318920	.	A	<INS:ME:LINE1>	.	PASS	SVTYPE=INS:ME:LINE1;END=34318932;SVLEN=6030;AN=20618;AC=1;AF=4.850130953535746e-05;N_HET=1;N_HOMALT=0;N_HOMREF=10308;FREQ_HET=9.700261907071492e-05;FREQ_HOMALT=0.0;FREQ_HOMREF=0.9999029973809292;N_BI_GENOS=10309;East_Han_AN=6554;East_Han_AC=1;East_Han_AF=
chr3	34393279	.	A	<INS:ME:LINE1>	.	PASS	SVTYPE=INS:ME:LINE1;END=34393284;SVLEN=1715;AN=20618;AC=3;AF=0.00014550392860607236;N_HET=3;N_HOMALT=0;N_HOMREF=10306;FREQ_HET=0.0002910078572121447;FREQ_HOMALT=0.0;FREQ_HOMREF=0.9997089921427879;N_BI_GENOS=10309;North_Han_AN=2272;North_Han_AC=1;North_Han
chr3	34510867	.	A	<INS:ME:LINE1>	.	PASS	SVTYPE=INS:ME:LINE1;END=34510877;SVLEN=501;AN=20618;AC=7;AF=0.0003395091667475022;N_HET=7;N_HOMALT=0;N_HOMREF=10302;FREQ_HET=0.0006790183334950044;FREQ_HOMALT=0.0;FREQ_HOMREF=0.999320981666505;N_BI_GENOS=10309;South_Han_AN=1496;South_Han_AC=1;South_Han_AF
chr3	34589063	.	T	<INS:ME:LINE1>	.	PASS	SVTYPE=INS:ME:LINE1;END=34589097;SVLEN=2205;AN=20618;AC=1;AF=4.850130953535746e-05;N_HET=1;N_HOMALT=0;N_HOMREF=10308;FREQ_HET=9.700261907071492e-05;FREQ_HOMALT=0.0;FREQ_HOMREF=0.9999029973809292;N_BI_GENOS=10309;East_Han_AN=6554;East_Han_AC=1;East_Han_AF=
chr3	34844989	.	A	<INS:ME:LINE1>	.	PASS	SVTYPE=INS:ME:LINE1;END=34845000;SVLEN=1004;AN=20618;AC=1;AF=4.850130953535746e-05;N_HET=1;N_HOMALT=0;N_HOMREF=10308;FREQ_HET=9.700261907071492e-05;FREQ_HOMALT=0.0;FREQ_HOMREF=0.9999029973809292;N_BI_GENOS=10309;North_Han_AN=2272;North_Han_AC=1;North_Han_
chr3	34975816	.	T	<INS:ME:LINE1>	.	PASS	SVTYPE=INS:ME:LINE1;END=34975816;SVLEN=26;AN=20618;AC=1;AF=4.850130953535746e-05;N_HET=1;N_HOMALT=0;N_HOMREF=10308;FREQ_HET=9.700261907071492e-05;FREQ_HOMALT=0.0;FREQ_HOMREF=0.9999029973809292;N_BI_GENOS=10309;East_Han_AN=6554;East_Han_AC=1;East_Han_AF=0.
chr3	35186643	.	A	<INS:ME:LINE1>	.	PASS	SVTYPE=INS:ME:LINE1;END=35186658;SVLEN=1890;AN=20618;AC=2;AF=9.700261907071492e-05;N_HET=2;N_HOMALT=0;N_HOMREF=10307;FREQ_HET=0.00019400523814142983;FREQ_HOMALT=0.0;FREQ_HOMREF=0.9998059947618586;N_BI_GENOS=10309;East_Han_AN=6554;East_Han_AC=1;East_Han_AF
chr3	35245423	.	T	<INS:ME:LINE1>	.	PASS	SVTYPE=INS:ME:LINE1;END=35245423;SVLEN=799;AN=20618;AC=4;AF=0.00019400523814142983;N_HET=4;N_HOMALT=0;N_HOMREF=10305;FREQ_HET=0.00038801047628285966;FREQ_HOMALT=0.0;FREQ_HOMREF=0.9996119895237171;N_BI_GENOS=10309;South_Han_AN=1496;South_Han_AC=1;South_Han
chr3	35398956	.	A	<INS:ME:LINE1>	.	PASS	SVTYPE=INS:ME:LINE1;END=35398984;SVLEN=493;AN=20618;AC=1;AF=4.850130953535746e-05;N_HET=1;N_HOMALT=0;N_HOMREF=10308;FREQ_HET=9.700261907071492e-05;FREQ_HOMALT=0.0;FREQ_HOMREF=0.9999029973809292;N_BI_GENOS=10309;Central_Han_AN=2128;Central_Han_AC=1;Central
chr3	35423466	.	T	<INS:ME:LINE1>	.	PASS	SVTYPE=INS:ME:LINE1;END=35423480;SVLEN=716;AN=20618;AC=1;AF=4.850130953535746e-05;N_HET=1;N_HOMALT=0;N_HOMREF=10308;FREQ_HET=9.700261907071492e-05;FREQ_HOMALT=0.0;FREQ_HOMREF=0.9999029973809292;N_BI_GENOS=10309;East_Han_AN=6554;East_Han_AC=1;East_Han_AF=0
chr3	35469109	.	G	<INS:ME:LINE1>	.	PASS	SVTYPE=INS:ME:LINE1;END=35469109;SVLEN=0;AN=20618;AC=2;AF=9.700261907071492e-05;N_HET=0;N_HOMALT=1;N_HOMREF=10308;FREQ_HET=0.0;FREQ_HOMALT=9.700261907071492e-05;FREQ_HOMREF=0.9999029973809292;N_BI_GENOS=10309;Northwest_Han_AN=1296;Northwest_Han_AC=2;North
chr3	35586652	.	T	<INS:ME:LINE1>	.	PASS	SVTYPE=INS:ME:LINE1;END=35586655;SVLEN=294;AN=20618;AC=2;AF=9.700261907071492e-05;N_HET=2;N_HOMALT=0;N_HOMREF=10307;FREQ_HET=0.00019400523814142983;FREQ_HOMALT=0.0;FREQ_HOMREF=0.9998059947618586;N_BI_GENOS=10309;North_Han_AN=2272;North_Han_AC=1;North_Han_
chr3	35657317	.	G	<INS:ME:LINE1>	.	PASS	SVTYPE=INS:ME:LINE1;END=35657336;SVLEN=213;AN=20618;AC=1;AF=4.850130953535746e-05;N_HET=1;N_HOMALT=0;N_HOMREF=10308;FREQ_HET=9.700261907071492e-05;FREQ_HOMALT=0.0;FREQ_HOMREF=0.9999029973809292;N_BI_GENOS=10309;Hui_AN=444;Hui_AC=1;Hui_AF=0.002252252252252
chr3	35954772	.	T	<INS:ME:LINE1>	.	PASS	SVTYPE=INS:ME:LINE1;END=35954781;SVLEN=5998;AN=20618;AC=14;AF=0.0006790183334950044;N_HET=14;N_HOMALT=0;N_HOMREF=10295;FREQ_HET=0.0013580366669900088;FREQ_HOMALT=0.0;FREQ_HOMREF=0.99864196333301;N_BI_GENOS=10309;South_Han_AN=1496;South_Han_AC=3;South_Han_
chr3	36057412	.	C	<INS:ME:LINE1>	.	PASS	SVTYPE=INS:ME:LINE1;END=36057431;SVLEN=1751;AN=20618;AC=1;AF=4.850130953535746e-05;N_HET=1;N_HOMALT=0;N_HOMREF=10308;FREQ_HET=9.700261907071492e-05;FREQ_HOMALT=0.0;FREQ_HOMREF=0.9999029973809292;N_BI_GENOS=10309;Central_Han_AN=2128;Central_Han_AC=1;Centra
chr3	36081561	.	T	<INS:ME:LINE1>	.	PASS	SVTYPE=INS:ME:LINE1;END=36081562;SVLEN=491;AN=20618;AC=1;AF=4.850130953535746e-05;N_HET=1;N_HOMALT=0;N_HOMREF=10308;FREQ_HET=9.700261907071492e-05;FREQ_HOMALT=0.0;FREQ_HOMREF=0.9999029973809292;N_BI_GENOS=10309;North_Han_AN=2272;North_Han_AC=1;North_Han_A
chr3	36091972	.	T	<INS:ME:LINE1>	.	PASS	SVTYPE=INS:ME:LINE1;END=36091987;SVLEN=1500;AN=20618;AC=1;AF=4.850130953535746e-05;N_HET=1;N_HOMALT=0;N_HOMREF=10308;FREQ_HET=9.700261907071492e-05;FREQ_HOMALT=0.0;FREQ_HOMREF=0.9999029973809292;N_BI_GENOS=10309;North_Han_AN=2272;North_Han_AC=1;North_Han_
chr3	36112150	.	A	<INS:ME:LINE1>	.	PASS	SVTYPE=INS:ME:LINE1;END=36112164;SVLEN=1631;AN=20618;AC=1;AF=4.850130953535746e-05;N_HET=1;N_HOMALT=0;N_HOMREF=10308;FREQ_HET=9.700261907071492e-05;FREQ_HOMALT=0.0;FREQ_HOMREF=0.9999029973809292;N_BI_GENOS=10309;Hui_AN=444;Hui_AC=1;Hui_AF=0.00225225225225
chr3	36273749	.	A	<INS:ME:LINE1>	.	PASS	SVTYPE=INS:ME:LINE1;END=36273750;SVLEN=695;AN=20618;AC=1;AF=4.850130953535746e-05;N_HET=1;N_HOMALT=0;N_HOMREF=10308;FREQ_HET=9.700261907071492e-05;FREQ_HOMALT=0.0;FREQ_HOMREF=0.9999029973809292;N_BI_GENOS=10309;North_Han_AN=2272;North_Han_AC=1;North_Han_A
chr3	36530922	.	C	<INS:ME:LINE1>	.	PASS	SVTYPE=INS:ME:LINE1;END=36530935;SVLEN=5997;AN=20618;AC=2;AF=9.700261907071492e-05;N_HET=2;N_HOMALT=0;N_HOMREF=10307;FREQ_HET=0.00019400523814142983;FREQ_HOMALT=0.0;FREQ_HOMREF=0.9998059947618586;N_BI_GENOS=10309;Southeast_Han_AN=1908;Southeast_Han_AC=1;S
chr3	36558378	.	T	<INS:ME:LINE1>	.	PASS	SVTYPE=INS:ME:LINE1;END=36558393;SVLEN=643;AN=20618;AC=11;AF=0.0005335144404888932;N_HET=11;N_HOMALT=0;N_HOMREF=10298;FREQ_HET=0.0010670288809777864;FREQ_HOMALT=0.0;FREQ_HOMREF=0.9989329711902222;N_BI_GENOS=10309;South_Han_AN=1496;South_Han_AC=3;South_Han_A
chr3	36712023	.	A	<INS:ME:LINE1>	.	PASS	SVTYPE=INS:ME:LINE1;END=36712042;SVLEN=6010;AN=20618;AC=1;AF=4.850130953535746e-05;N_HET=1;N_HOMALT=0;N_HOMREF=10308;FREQ_HET=9.700261907071492e-05;FREQ_HOMALT=0.0;FREQ_HOMREF=0.9999029973809292;N_BI_GENOS=10309;Southeast_Han_AN=1908;Southeast_Han_AC=1;So
chr3	36727465	.	A	<INS:ME:LINE1>	.	PASS	SVTYPE=INS:ME:LINE1;END=36727480;SVLEN=348;AN=20618;AC=1;AF=4.850130953535746e-05;N_HET=1;N_HOMALT=0;N_HOMREF=10308;FREQ_HET=9.700261907071492e-05;FREQ_HOMALT=0.0;FREQ_HOMREF=0.9999029973809292;N_BI_GENOS=10309;Southeast_Han_AN=1908;Southeast_Han_AC=1;Sou
chr3	37120916	.	A	<INS:ME:LINE1>	.	PASS	SVTYPE=INS:ME:LINE1;END=37120931;SVLEN=559;AN=20618;AC=1;AF=4.850130953535746e-05;N_HET=1;N_HOMALT=0;N_HOMREF=10308;FREQ_HET=9.700261907071492e-05;FREQ_HOMALT=0.0;FREQ_HOMREF=0.9999029973809292;N_BI_GENOS=10309;Lingnan_Han_AN=1888;Lingnan_Han_AC=1;Lingnan
chr3	37268499	.	A	<INS:ME:LINE1>	.	PASS	SVTYPE=INS:ME:LINE1;END=37268514;SVLEN=283;AN=20618;AC=1;AF=4.850130953535746e-05;N_HET=1;N_HOMALT=0;N_HOMREF=10308;FREQ_HET=9.700261907071492e-05;FREQ_HOMALT=0.0;FREQ_HOMREF=0.9999029973809292;N_BI_GENOS=10309;Lingnan_Han_AN=1888;Lingnan_Han_AC=1;Lingnan
chr3	37467272	.	A	<INS:ME:LINE1>	.	PASS	SVTYPE=INS:ME:LINE1;END=37467272;SVLEN=5991;AN=20618;AC=1;AF=4.850130953535746e-05;N_HET=1;N_HOMALT=0;N_HOMREF=10308;FREQ_HET=9.700261907071492e-05;FREQ_HOMALT=0.0;FREQ_HOMREF=0.9999029973809292;N_BI_GENOS=10309;Central_Han_AN=2128;Central_Han_AC=1;Centra
chr3	37723221	.	A	<INS:ME:LINE1>	.	PASS	SVTYPE=INS:ME:LINE1;END=37723235;SVLEN=6069;AN=20618;AC=2;AF=9.700261907071492e-05;N_HET=2;N_HOMALT=0;N_HOMREF=10307;FREQ_HET=0.00019400523814142983;FREQ_HOMALT=0.0;FREQ_HOMREF=0.9998059947618586;N_BI_GENOS=10309;North_Han_AN=2272;North_Han_AC=1;North_Han
chr3	37758732	.	A	<INS:ME:LINE1>	.	PASS	SVTYPE=INS:ME:LINE1;END=37758751;SVLEN=355;AN=20618;AC=2;AF=9.700261907071492e-05;N_HET=2;N_HOMALT=0;N_HOMREF=10307;FREQ_HET=0.00019400523814142983;FREQ_HOMALT=0.0;FREQ_HOMREF=0.9998059947618586;N_BI_GENOS=10309;East_Han_AN=6554;East_Han_AC=1;East_Han_AF=
chr3	38083397	.	G	<INS:ME:LINE1>	.	PASS	SVTYPE=INS:ME:LINE1;END=38083397;SVLEN=0;AN=20618;AC=1;AF=4.850130953535746e-05;N_HET=1;N_HOMALT=0;N_HOMREF=10308;FREQ_HET=9.700261907071492e-05;FREQ_HOMALT=0.0;FREQ_HOMREF=0.9999029973809292;N_BI_GENOS=10309;North_Han_AN=2272;North_Han_AC=1;North_Han_AF=
chr3	38212101	.	G	<INS:ME:LINE1>	.	PASS	SVTYPE=INS:ME:LINE1;END=38212116;SVLEN=3801;AN=20618;AC=1032;AF=0.05005335144048889;N_HET=930;N_HOMALT=51;N_HOMREF=9328;FREQ_HET=0.09021243573576486;FREQ_HOMALT=0.004947133572606461;FREQ_HOMREF=0.9048404306916287;N_BI_GENOS=10309;South_Han_AN=1496;South_H
chr3	38584574	.	A	<INS:ME:LINE1>	.	PASS	SVTYPE=INS:ME:LINE1;END=38584591;SVLEN=5999;AN=20618;AC=7098;AF=0.3442622950819672;N_HET=2954;N_HOMALT=2072;N_HOMREF=5283;FREQ_HET=0.28654573673489186;FREQ_HOMALT=0.2009894267145213;FREQ_HOMREF=0.5124648365505868;N_BI_GENOS=10309;South_Han_AN=1496;South_H
chr3	38996357	.	G	<INS:ME:LINE1>	.	PASS	SVTYPE=INS:ME:LINE1;END=38996375;SVLEN=4396;AN=20618;AC=12;AF=0.0005820157144242894;N_HET=12;N_HOMALT=0;N_HOMREF=10297;FREQ_HET=0.0011640314288485789;FREQ_HOMALT=0.0;FREQ_HOMREF=0.9988359685711514;N_BI_GENOS=10309;Lingnan_Han_AN=1888;Lingnan_Han_AC=1;Ling
chr3	39852461	.	T	<INS:ME:LINE1>	.	PASS	SVTYPE=INS:ME:LINE1;END=39852465;SVLEN=4193;AN=20618;AC=1;AF=4.850130953535746e-05;N_HET=1;N_HOMALT=0;N_HOMREF=10308;FREQ_HET=9.700261907071492e-05;FREQ_HOMALT=0.0;FREQ_HOMREF=0.9999029973809292;N_BI_GENOS=10309;Lingnan_Han_AN=1888;Lingnan_Han_AC=1;Lingna
chr3	39923129	.	A	<INS:ME:LINE1>	.	PASS	SVTYPE=INS:ME:LINE1;END=39923146;SVLEN=665;AN=20618;AC=1;AF=4.850130953535746e-05;N_HET=1;N_HOMALT=0;N_HOMREF=10308;FREQ_HET=9.700261907071492e-05;FREQ_HOMALT=0.0;FREQ_HOMREF=0.9999029973809292;N_BI_GENOS=10309;East_Han_AN=6554;East_Han_AC=1;East_Han_AF=0
chr3	40419981	.	A	<INS:ME:LINE1>	.	PASS	SVTYPE=INS:ME:LINE1;END=40419981;SVLEN=668;AN=20618;AC=1;AF=4.850130953535746e-05;N_HET=1;N_HOMALT=0;N_HOMREF=10308;FREQ_HET=9.700261907071492e-05;FREQ_HOMALT=0.0;FREQ_HOMREF=0.9999029973809292;N_BI_GENOS=10309;Lingnan_Han_AN=1888;Lingnan_Han_AC=1;Lingnan
chr3	41239350	.	T	<INS:ME:LINE1>	.	PASS	SVTYPE=INS:ME:LINE1;END=41239366;SVLEN=702;AN=20618;AC=1;AF=4.850130953535746e-05;N_HET=1;N_HOMALT=0;N_HOMREF=10308;FREQ_HET=9.700261907071492e-05;FREQ_HOMALT=0.0;FREQ_HOMREF=0.9999029973809292;N_BI_GENOS=10309;Lingnan_Han_AN=1888;Lingnan_Han_AC=1;Lingnan
chr3	41412859	.	C	<INS:ME:LINE1>	.	PASS	SVTYPE=INS:ME:LINE1;END=41412876;SVLEN=826;AN=20618;AC=1;AF=4.850130953535746e-05;N_HET=1;N_HOMALT=0;N_HOMREF=10308;FREQ_HET=9.700261907071492e-05;FREQ_HOMALT=0.0;FREQ_HOMREF=0.9999029973809292;N_BI_GENOS=10309;Southeast_Han_AN=1908;Southeast_Han_AC=1;Sou
chr3	41535062	.	T	<INS:ME:LINE1>	.	PASS	SVTYPE=INS:ME:LINE1;END=41535077;SVLEN=640;AN=20618;AC=1;AF=4.850130953535746e-05;N_HET=1;N_HOMALT=0;N_HOMREF=10308;FREQ_HET=9.700261907071492e-05;FREQ_HOMALT=0.0;FREQ_HOMREF=0.9999029973809292;N_BI_GENOS=10309;South_Han_AN=1496;South_Han_AC=1;South_Han_A
chr3	41549411	.	A	<INS:ME:LINE1>	.	PASS	SVTYPE=INS:ME:LINE1;END=41549425;SVLEN=2440;AN=20618;AC=1;AF=4.850130953535746e-05;N_HET=1;N_HOMALT=0;N_HOMREF=10308;FREQ_HET=9.700261907071492e-05;FREQ_HOMALT=0.0;FREQ_HOMREF=0.9999029973809292;N_BI_GENOS=10309;East_Han_AN=6554;East_Han_AC=1;East_Han_AF=
chr3	41922518	.	T	<INS:ME:LINE1>	.	PASS	SVTYPE=INS:ME:LINE1;END=41922518;SVLEN=5983;AN=20618;AC=1;AF=4.850130953535746e-05;N_HET=1;N_HOMALT=0;N_HOMREF=10308;FREQ_HET=9.700261907071492e-05;FREQ_HOMALT=0.0;FREQ_HOMREF=0.9999029973809292;N_BI_GENOS=10309;East_Han_AN=6554;East_Han_AC=1;East_Han_AF=
chr3	42061166	.	C	<INS:ME:LINE1>	.	PASS	SVTYPE=INS:ME:LINE1;END=42061166;SVLEN=2943;AN=20618;AC=1;AF=4.850130953535746e-05;N_HET=1;N_HOMALT=0;N_HOMREF=10308;FREQ_HET=9.700261907071492e-05;FREQ_HOMALT=0.0;FREQ_HOMREF=0.9999029973809292;N_BI_GENOS=10309;Lingnan_Han_AN=1888;Lingnan_Han_AC=1;Lingna
chr3	42736225	.	A	<INS:ME:LINE1>	.	PASS	SVTYPE=INS:ME:LINE1;END=42736239;SVLEN=410;AN=20618;AC=1;AF=4.850130953535746e-05;N_HET=1;N_HOMALT=0;N_HOMREF=10308;FREQ_HET=9.700261907071492e-05;FREQ_HOMALT=0.0;FREQ_HOMREF=0.9999029973809292;N_BI_GENOS=10309;East_Han_AN=6554;East_Han_AC=1;East_Han_AF=0
chr3	42964217	.	G	<INS:ME:LINE1>	.	PASS	SVTYPE=INS:ME:LINE1;END=42964230;SVLEN=6003;AN=20618;AC=1;AF=4.850130953535746e-05;N_HET=1;N_HOMALT=0;N_HOMREF=10308;FREQ_HET=9.700261907071492e-05;FREQ_HOMALT=0.0;FREQ_HOMREF=0.9999029973809292;N_BI_GENOS=10309;South_Han_AN=1496;South_Han_AC=1;South_Han_
chr3	43038703	.	G	<INS:ME:LINE1>	.	PASS	SVTYPE=INS:ME:LINE1;END=43038715;SVLEN=6000;AN=20618;AC=3;AF=0.00014550392860607236;N_HET=3;N_HOMALT=0;N_HOMREF=10306;FREQ_HET=0.0002910078572121447;FREQ_HOMALT=0.0;FREQ_HOMREF=0.9997089921427879;N_BI_GENOS=10309;East_Han_AN=6554;East_Han_AC=2;East_Han_AF
chr3	43221187	.	A	<INS:ME:LINE1>	.	PASS	SVTYPE=INS:ME:LINE1;END=43221188;SVLEN=2033;AN=20618;AC=1;AF=4.850130953535746e-05;N_HET=1;N_HOMALT=0;N_HOMREF=10308;FREQ_HET=9.700261907071492e-05;FREQ_HOMALT=0.0;FREQ_HOMREF=0.9999029973809292;N_BI_GENOS=10309;North_Han_AN=2272;North_Han_AC=1;North_Han_
chr3	43589823	.	A	<INS:ME:LINE1>	.	PASS	SVTYPE=INS:ME:LINE1;END=43589837;SVLEN=6000;AN=20618;AC=1;AF=4.850130953535746e-05;N_HET=1;N_HOMALT=0;N_HOMREF=10308;FREQ_HET=9.700261907071492e-05;FREQ_HOMALT=0.0;FREQ_HOMREF=0.9999029973809292;N_BI_GENOS=10309;Mongolian_AN=454;Mongolian_AC=1;Mongolian_A
chr3	43602735	.	G	<INS:ME:LINE1>	.	PASS	SVTYPE=INS:ME:LINE1;END=43602747;SVLEN=525;AN=20618;AC=1;AF=4.850130953535746e-05;N_HET=1;N_HOMALT=0;N_HOMREF=10308;FREQ_HET=9.700261907071492e-05;FREQ_HOMALT=0.0;FREQ_HOMREF=0.9999029973809292;N_BI_GENOS=10309;Northwest_Han_AN=1296;Northwest_Han_AC=1;Nor
chr3	43874433	.	A	<INS:ME:LINE1>	.	PASS	SVTYPE=INS:ME:LINE1;END=43874448;SVLEN=5997;AN=20618;AC=1;AF=4.850130953535746e-05;N_HET=1;N_HOMALT=0;N_HOMREF=10308;FREQ_HET=9.700261907071492e-05;FREQ_HOMALT=0.0;FREQ_HOMREF=0.9999029973809292;N_BI_GENOS=10309;East_Han_AN=6554;East_Han_AC=1;East_Han_AF=
chr3	44193011	.	A	<INS:ME:LINE1>	.	PASS	SVTYPE=INS:ME:LINE1;END=44193011;SVLEN=5970;AN=20618;AC=2;AF=9.700261907071492e-05;N_HET=2;N_HOMALT=0;N_HOMREF=10307;FREQ_HET=0.00019400523814142983;FREQ_HOMALT=0.0;FREQ_HOMREF=0.9998059947618586;N_BI_GENOS=10309;South_Han_AN=1496;South_Han_AC=1;South_Han

chr3	44259250	.	T	<INS:ME:LINE1>	.	PASS	SVTYPE=INS:ME:LINE1;END=44259265;SVLEN=1018;AN=20618;AC=2;AF=9.700261907071492e-05;N_HET=2;N_HOMALT=0;N_HOMREF=10307;FREQ_HET=0.00019400523814142983;FREQ_HOMALT=0.0;FREQ_HOMREF=0.9998059947618586;N_BI_GENOS=10309;Mongolian_AN=454;Mongolian_AC=2;Mongolian_
chr3	45271511	.	G	<INS:ME:LINE1>	.	PASS	SVTYPE=INS:ME:LINE1;END=45271526;SVLEN=6011;AN=20618;AC=1;AF=4.850130953535746e-05;N_HET=1;N_HOMALT=0;N_HOMREF=10308;FREQ_HET=9.700261907071492e-05;FREQ_HOMALT=0.0;FREQ_HOMREF=0.9999029973809292;N_BI_GENOS=10309;East_Han_AN=6554;East_Han_AC=1;East_Han_AF=
chr3	45744435	.	A	<INS:ME:LINE1>	.	PASS	SVTYPE=INS:ME:LINE1;END=45744435;SVLEN=6000;AN=20618;AC=6;AF=0.0002910078572121447;N_HET=6;N_HOMALT=0;N_HOMREF=10303;FREQ_HET=0.0005820157144242894;FREQ_HOMALT=0.0;FREQ_HOMREF=0.9994179842855757;N_BI_GENOS=10309;East_Han_AN=6554;East_Han_AC=6;East_Han_AF=
chr3	46180513	.	A	<INS:ME:LINE1>	.	PASS	SVTYPE=INS:ME:LINE1;END=46180525;SVLEN=509;AN=20618;AC=22;AF=0.001067028809777864;N_HET=20;N_HOMALT=1;N_HOMREF=10288;FREQ_HET=0.0019400523814142982;FREQ_HOMALT=9.700261907071492e-05;FREQ_HOMREF=0.997962944999515;N_BI_GENOS=10309;South_Han_AN=1496;South_Ha
chr3	46239269	.	T	<INS:ME:LINE1>	.	PASS	SVTYPE=INS:ME:LINE1;END=46239285;SVLEN=6002;AN=20618;AC=1;AF=4.850130953535746e-05;N_HET=1;N_HOMALT=0;N_HOMREF=10308;FREQ_HET=9.700261907071492e-05;FREQ_HOMALT=0.0;FREQ_HOMREF=0.9999029973809292;N_BI_GENOS=10309;East_Han_AN=6554;East_Han_AC=1;East_Han_AF=
chr3	46631771	.	A	<INS:ME:LINE1>	.	PASS	SVTYPE=INS:ME:LINE1;END=46631790;SVLEN=414;AN=20618;AC=1;AF=4.850130953535746e-05;N_HET=1;N_HOMALT=0;N_HOMREF=10308;FREQ_HET=9.700261907071492e-05;FREQ_HOMALT=0.0;FREQ_HOMREF=0.9999029973809292;N_BI_GENOS=10309;South_Han_AN=1496;South_Han_AC=1;South_Han_A
chr3	49200475	.	A	<INS:ME:LINE1>	.	PASS	SVTYPE=INS:ME:LINE1;END=49200475;SVLEN=0;AN=20618;AC=5;AF=0.00024250654767678728;N_HET=3;N_HOMALT=1;N_HOMREF=10305;FREQ_HET=0.0002910078572121447;FREQ_HOMALT=9.700261907071492e-05;FREQ_HOMREF=0.9996119895237171;N_BI_GENOS=10309;North_Han_AN=2272;North_Han
chr3	49404094	.	T	<INS:ME:LINE1>	.	PASS	SVTYPE=INS:ME:LINE1;END=49404095;SVLEN=882;AN=20618;AC=63;AF=0.0030555825007275196;N_HET=59;N_HOMALT=2;N_HOMREF=10248;FREQ_HET=0.0057231545251721794;FREQ_HOMALT=0.00019400523814142983;FREQ_HOMREF=0.9940828402366864;N_BI_GENOS=10309;South_Han_AN=1496;South
chr3	50019378	.	T	<INS:ME:LINE1>	.	PASS	SVTYPE=INS:ME:LINE1;END=50019393;SVLEN=1374;AN=20618;AC=2;AF=9.700261907071492e-05;N_HET=2;N_HOMALT=0;N_HOMREF=10307;FREQ_HET=0.00019400523814142983;FREQ_HOMALT=0.0;FREQ_HOMREF=0.9998059947618586;N_BI_GENOS=10309;Lingnan_Han_AN=1888;Lingnan_Han_AC=1;Lingn
chr3	50981808	.	T	<INS:ME:LINE1>	.	PASS	SVTYPE=INS:ME:LINE1;END=50981808;SVLEN=39;AN=20618;AC=1;AF=4.850130953535746e-05;N_HET=1;N_HOMALT=0;N_HOMREF=10308;FREQ_HET=9.700261907071492e-05;FREQ_HOMALT=0.0;FREQ_HOMREF=0.9999029973809292;N_BI_GENOS=10309;Central_Han_AN=2128;Central_Han_AC=1;Central_
chr3	51184768	.	T	<INS:ME:LINE1>	.	PASS	SVTYPE=INS:ME:LINE1;END=51184786;SVLEN=1830;AN=20618;AC=2;AF=9.700261907071492e-05;N_HET=2;N_HOMALT=0;N_HOMREF=10307;FREQ_HET=0.00019400523814142983;FREQ_HOMALT=0.0;FREQ_HOMREF=0.9998059947618586;N_BI_GENOS=10309;Northwest_Han_AN=1296;Northwest_Han_AC=1;N
chr3	51207661	.	C	<INS:ME:LINE1>	.	PASS	SVTYPE=INS:ME:LINE1;END=51207667;SVLEN=1424;AN=20618;AC=1;AF=4.850130953535746e-05;N_HET=1;N_HOMALT=0;N_HOMREF=10308;FREQ_HET=9.700261907071492e-05;FREQ_HOMALT=0.0;FREQ_HOMREF=0.9999029973809292;N_BI_GENOS=10309;North_Han_AN=2272;North_Han_AC=1;North_Han_
chr3	51328625	.	G	<INS:ME:LINE1>	.	PASS	SVTYPE=INS:ME:LINE1;END=51328641;SVLEN=6310;AN=20618;AC=1;AF=4.850130953535746e-05;N_HET=1;N_HOMALT=0;N_HOMREF=10308;FREQ_HET=9.700261907071492e-05;FREQ_HOMALT=0.0;FREQ_HOMREF=0.9999029973809292;N_BI_GENOS=10309;North_Han_AN=2272;North_Han_AC=1;North_Han_
chr3	51447412	.	A	<INS:ME:LINE1>	.	PASS	SVTYPE=INS:ME:LINE1;END=51447426;SVLEN=5998;AN=20618;AC=1;AF=4.850130953535746e-05;N_HET=1;N_HOMALT=0;N_HOMREF=10308;FREQ_HET=9.700261907071492e-05;FREQ_HOMALT=0.0;FREQ_HOMREF=0.9999029973809292;N_BI_GENOS=10309;Lingnan_Han_AN=1888;Lingnan_Han_AC=1;Lingna
chr3	52799315	.	G	<INS:ME:LINE1>	.	PASS	SVTYPE=INS:ME:LINE1;END=52799324;SVLEN=890;AN=20618;AC=1;AF=4.850130953535746e-05;N_HET=1;N_HOMALT=0;N_HOMREF=10308;FREQ_HET=9.700261907071492e-05;FREQ_HOMALT=0.0;FREQ_HOMREF=0.9999029973809292;N_BI_GENOS=10309;Northwest_Han_AN=1296;Northwest_Han_AC=1;Nor
chr3	53387827	.	T	<INS:ME:LINE1>	.	PASS	SVTYPE=INS:ME:LINE1;END=53387843;SVLEN=373;AN=20618;AC=1;AF=4.850130953535746e-05;N_HET=1;N_HOMALT=0;N_HOMREF=10308;FREQ_HET=9.700261907071492e-05;FREQ_HOMALT=0.0;FREQ_HOMREF=0.9999029973809292;N_BI_GENOS=10309;North_Han_AN=2272;North_Han_AC=1;North_Han_A
chr3	53706090	.	C	<INS:ME:LINE1>	.	PASS	SVTYPE=INS:ME:LINE1;END=53706105;SVLEN=6000;AN=20618;AC=7;AF=0.0003395091667475022;N_HET=7;N_HOMALT=0;N_HOMREF=10302;FREQ_HET=0.0006790183334950044;FREQ_HOMALT=0.0;FREQ_HOMREF=0.999320981666505;N_BI_GENOS=10309;South_Han_AN=1496;South_Han_AC=2;South_Han_A
chr3	54145418	.	G	<INS:ME:LINE1>	.	PASS	SVTYPE=INS:ME:LINE1;END=54145438;SVLEN=310;AN=20618;AC=1;AF=4.850130953535746e-05;N_HET=1;N_HOMALT=0;N_HOMREF=10308;FREQ_HET=9.700261907071492e-05;FREQ_HOMALT=0.0;FREQ_HOMREF=0.9999029973809292;N_BI_GENOS=10309;Northwest_Han_AN=1296;Northwest_Han_AC=1;Nor
chr3	54269760	.	T	<INS:ME:LINE1>	.	PASS	SVTYPE=INS:ME:LINE1;END=54269773;SVLEN=287;AN=20618;AC=1;AF=4.850130953535746e-05;N_HET=1;N_HOMALT=0;N_HOMREF=10308;FREQ_HET=9.700261907071492e-05;FREQ_HOMALT=0.0;FREQ_HOMREF=0.9999029973809292;N_BI_GENOS=10309;South_Han_AN=1496;South_Han_AC=1;South_Han_A
chr3	55419464	.	C	<INS:ME:LINE1>	.	PASS	SVTYPE=INS:ME:LINE1;END=55419478;SVLEN=6006;AN=20618;AC=13;AF=0.0006305170239596469;N_HET=13;N_HOMALT=0;N_HOMREF=10296;FREQ_HET=0.0012610340479192938;FREQ_HOMALT=0.0;FREQ_HOMREF=0.9987389659520807;N_BI_GENOS=10309;North_Han_AN=2272;North_Han_AC=3;North_Ha
chr3	55742523	.	A	<INS:ME:LINE1>	.	PASS	SVTYPE=INS:ME:LINE1;END=55742538;SVLEN=5998;AN=20618;AC=1;AF=4.850130953535746e-05;N_HET=1;N_HOMALT=0;N_HOMREF=10308;FREQ_HET=9.700261907071492e-05;FREQ_HOMALT=0.0;FREQ_HOMREF=0.9999029973809292;N_BI_GENOS=10309;East_Han_AN=6554;East_Han_AC=1;East_Han_AF=
chr3	55754539	.	A	<INS:ME:LINE1>	.	PASS	SVTYPE=INS:ME:LINE1;END=55754552;SVLEN=5998;AN=20618;AC=9711;AF=0.47099621689785626;N_HET=3963;N_HOMALT=2874;N_HOMREF=3472;FREQ_HET=0.3844213793772432;FREQ_HOMALT=0.27878552720923466;FREQ_HOMREF=0.33679309341352215;N_BI_GENOS=10309;South_Han_AN=1496;South
chr3	55754683	.	T	<INS:ME:LINE1>	.	PASS	SVTYPE=INS:ME:LINE1;END=55754683;SVLEN=419;AN=20618;AC=1;AF=4.850130953535746e-05;N_HET=1;N_HOMALT=0;N_HOMREF=10308;FREQ_HET=9.700261907071492e-05;FREQ_HOMALT=0.0;FREQ_HOMREF=0.9999029973809292;N_BI_GENOS=10309;Tibetan_AN=414;Tibetan_AC=1;Tibetan_AF=0.002
chr3	55754771	.	T	<INS:ME:LINE1>	.	PASS	SVTYPE=INS:ME:LINE1;END=55754771;SVLEN=364;AN=20618;AC=29;AF=0.0014065379765253662;N_HET=29;N_HOMALT=0;N_HOMREF=10280;FREQ_HET=0.0028130759530507325;FREQ_HOMALT=0.0;FREQ_HOMREF=0.9971869240469493;N_BI_GENOS=10309;South_Han_AN=1496;South_Han_AC=1;South_Han
chr3	55754916	.	T	<INS:ME:LINE1>	.	PASS	SVTYPE=INS:ME:LINE1;END=55754933;SVLEN=5994;AN=20618;AC=1;AF=4.850130953535746e-05;N_HET=1;N_HOMALT=0;N_HOMREF=10308;FREQ_HET=9.700261907071492e-05;FREQ_HOMALT=0.0;FREQ_HOMREF=0.9999029973809292;N_BI_GENOS=10309;North_Han_AN=2272;North_Han_AC=1;North_Han_
chr3	55915075	.	G	<INS:ME:LINE1>	.	PASS	SVTYPE=INS:ME:LINE1;END=55915092;SVLEN=1732;AN=20618;AC=2;AF=9.700261907071492e-05;N_HET=2;N_HOMALT=0;N_HOMREF=10307;FREQ_HET=0.00019400523814142983;FREQ_HOMALT=0.0;FREQ_HOMREF=0.9998059947618586;N_BI_GENOS=10309;East_Han_AN=6554;East_Han_AC=2;East_Han_AF
chr3	55939494	.	A	<INS:ME:LINE1>	.	PASS	SVTYPE=INS:ME:LINE1;END=55939512;SVLEN=428;AN=20618;AC=4;AF=0.00019400523814142983;N_HET=4;N_HOMALT=0;N_HOMREF=10305;FREQ_HET=0.00038801047628285966;FREQ_HOMALT=0.0;FREQ_HOMREF=0.9996119895237171;N_BI_GENOS=10309;Lingnan_Han_AN=1888;Lingnan_Han_AC=1;Lingn
chr3	56731610	.	A	<INS:ME:LINE1>	.	PASS	SVTYPE=INS:ME:LINE1;END=56731610;SVLEN=0;AN=20618;AC=18;AF=0.0008730235716364342;N_HET=14;N_HOMALT=2;N_HOMREF=10293;FREQ_HET=0.0013580366669900088;FREQ_HOMALT=0.00019400523814142983;FREQ_HOMREF=0.9984479580948685;N_BI_GENOS=10309;South_Han_AN=1496;South_H
chr3	56911683	.	T	<INS:ME:LINE1>	.	PASS	SVTYPE=INS:ME:LINE1;END=56911698;SVLEN=688;AN=20618;AC=1;AF=4.850130953535746e-05;N_HET=1;N_HOMALT=0;N_HOMREF=10308;FREQ_HET=9.700261907071492e-05;FREQ_HOMALT=0.0;FREQ_HOMREF=0.9999029973809292;N_BI_GENOS=10309;Central_Han_AN=2128;Central_Han_AC=1;Central
chr3	57459502	.	T	<INS:ME:LINE1>	.	PASS	SVTYPE=INS:ME:LINE1;END=57459513;SVLEN=1118;AN=20618;AC=5;AF=0.00024250654767678728;N_HET=5;N_HOMALT=0;N_HOMREF=10304;FREQ_HET=0.00048501309535357455;FREQ_HOMALT=0.0;FREQ_HOMREF=0.9995149869046465;N_BI_GENOS=10309;Lingnan_Han_AN=1888;Lingnan_Han_AC=2;Ling
chr3	57474015	.	A	<INS:ME:LINE1>	.	PASS	SVTYPE=INS:ME:LINE1;END=57474015;SVLEN=356;AN=20618;AC=1;AF=4.850130953535746e-05;N_HET=1;N_HOMALT=0;N_HOMREF=10308;FREQ_HET=9.700261907071492e-05;FREQ_HOMALT=0.0;FREQ_HOMREF=0.9999029973809292;N_BI_GENOS=10309;East_Han_AN=6554;East_Han_AC=1;East_Han_AF=0
chr3	57801900	.	T	<INS:ME:LINE1>	.	PASS	SVTYPE=INS:ME:LINE1;END=57801915;SVLEN=4724;AN=20618;AC=1;AF=4.850130953535746e-05;N_HET=1;N_HOMALT=0;N_HOMREF=10308;FREQ_HET=9.700261907071492e-05;FREQ_HOMALT=0.0;FREQ_HOMREF=0.9999029973809292;N_BI_GENOS=10309;Central_Han_AN=2128;Central_Han_AC=1;Centra
chr3	58744776	.	T	<INS:ME:LINE1>	.	PASS	SVTYPE=INS:ME:LINE1;END=58744797;SVLEN=2038;AN=20618;AC=3;AF=0.00014550392860607236;N_HET=3;N_HOMALT=0;N_HOMREF=10306;FREQ_HET=0.0002910078572121447;FREQ_HOMALT=0.0;FREQ_HOMREF=0.9997089921427879;N_BI_GENOS=10309;Northwest_Han_AN=1296;Northwest_Han_AC=2;N
chr3	58782989	.	A	<INS:ME:LINE1>	.	PASS	SVTYPE=INS:ME:LINE1;END=58782989;SVLEN=832;AN=20618;AC=1;AF=4.850130953535746e-05;N_HET=1;N_HOMALT=0;N_HOMREF=10308;FREQ_HET=9.700261907071492e-05;FREQ_HOMALT=0.0;FREQ_HOMREF=0.9999029973809292;N_BI_GENOS=10309;East_Han_AN=6554;East_Han_AC=1;East_Han_AF=0
chr3	58869502	.	T	<INS:ME:LINE1>	.	PASS	SVTYPE=INS:ME:LINE1;END=58869502;SVLEN=6000;AN=20618;AC=3;AF=0.00014550392860607236;N_HET=3;N_HOMALT=0;N_HOMREF=10306;FREQ_HET=0.0002910078572121447;FREQ_HOMALT=0.0;FREQ_HOMREF=0.9997089921427879;N_BI_GENOS=10309;East_Han_AN=6554;East_Han_AC=3;East_Han_AF
chr3	58876389	.	C	<INS:ME:LINE1>	.	PASS	SVTYPE=INS:ME:LINE1;END=58876389;SVLEN=63;AN=20618;AC=1;AF=4.850130953535746e-05;N_HET=1;N_HOMALT=0;N_HOMREF=10308;FREQ_HET=9.700261907071492e-05;FREQ_HOMALT=0.0;FREQ_HOMREF=0.9999029973809292;N_BI_GENOS=10309;Southeast_Han_AN=1908;Southeast_Han_AC=1;Sout
chr3	59004316	.	A	<INS:ME:LINE1>	.	PASS	SVTYPE=INS:ME:LINE1;END=59004332;SVLEN=2848;AN=20618;AC=1;AF=4.850130953535746e-05;N_HET=1;N_HOMALT=0;N_HOMREF=10308;FREQ_HET=9.700261907071492e-05;FREQ_HOMALT=0.0;FREQ_HOMREF=0.9999029973809292;N_BI_GENOS=10309;Northwest_Han_AN=1296;Northwest_Han_AC=1;No
chr3	59024741	.	A	<INS:ME:LINE1>	.	PASS	SVTYPE=INS:ME:LINE1;END=59024754;SVLEN=646;AN=20618;AC=1;AF=4.850130953535746e-05;N_HET=1;N_HOMALT=0;N_HOMREF=10308;FREQ_HET=9.700261907071492e-05;FREQ_HOMALT=0.0;FREQ_HOMREF=0.9999029973809292;N_BI_GENOS=10309;North_Han_AN=2272;North_Han_AC=1;North_Han_A
chr3	59116849	.	A	<INS:ME:LINE1>	.	PASS	SVTYPE=INS:ME:LINE1;END=59116864;SVLEN=1529;AN=20618;AC=2;AF=9.700261907071492e-05;N_HET=2;N_HOMALT=0;N_HOMREF=10307;FREQ_HET=0.00019400523814142983;FREQ_HOMALT=0.0;FREQ_HOMREF=0.9998059947618586;N_BI_GENOS=10309;East_Han_AN=6554;East_Han_AC=2;East_Han_AF
chr3	59128844	.	A	<INS:ME:LINE1>	.	PASS	SVTYPE=INS:ME:LINE1;END=59128859;SVLEN=809;AN=20618;AC=1;AF=4.850130953535746e-05;N_HET=1;N_HOMALT=0;N_HOMREF=10308;FREQ_HET=9.700261907071492e-05;FREQ_HOMALT=0.0;FREQ_HOMREF=0.9999029973809292;N_BI_GENOS=10309;Mongolian_AN=454;Mongolian_AC=1;Mongolian_AF
chr3	59212178	.	A	<INS:ME:LINE1>	.	PASS	SVTYPE=INS:ME:LINE1;END=59212193;SVLEN=98;AN=20618;AC=1;AF=4.850130953535746e-05;N_HET=1;N_HOMALT=0;N_HOMREF=10308;FREQ_HET=9.700261907071492e-05;FREQ_HOMALT=0.0;FREQ_HOMREF=0.9999029973809292;N_BI_GENOS=10309;East_Han_AN=6554;East_Han_AC=1;East_Han_AF=0.
chr3	59413929	.	T	<INS:ME:LINE1>	.	PASS	SVTYPE=INS:ME:LINE1;END=59413946;SVLEN=211;AN=20618;AC=1;AF=4.850130953535746e-05;N_HET=1;N_HOMALT=0;N_HOMREF=10308;FREQ_HET=9.700261907071492e-05;FREQ_HOMALT=0.0;FREQ_HOMREF=0.9999029973809292;N_BI_GENOS=10309;North_Han_AN=2272;North_Han_AC=1;North_Han_A
chr3	59828780	.	A	<INS:ME:LINE1>	.	PASS	SVTYPE=INS:ME:LINE1;END=59828797;SVLEN=5998;AN=20618;AC=1;AF=4.850130953535746e-05;N_HET=1;N_HOMALT=0;N_HOMREF=10308;FREQ_HET=9.700261907071492e-05;FREQ_HOMALT=0.0;FREQ_HOMREF=0.9999029973809292;N_BI_GENOS=10309;East_Han_AN=6554;East_Han_AC=1;East_Han_AF=
chr3	59867163	.	A	<INS:ME:LINE1>	.	PASS	SVTYPE=INS:ME:LINE1;END=59867177;SVLEN=902;AN=20618;AC=12651;AF=0.6135900669318072;N_HET=4843;N_HOMALT=3904;N_HOMREF=1562;FREQ_HET=0.4697836841594723;FREQ_HOMALT=0.378698224852071;FREQ_HOMREF=0.1515180909884567;N_BI_GENOS=10309;South_Han_AN=1496;South_Han
chr3	60069274	.	G	<INS:ME:LINE1>	.	PASS	SVTYPE=INS:ME:LINE1;END=60069284;SVLEN=5998;AN=20618;AC=1;AF=4.850130953535746e-05;N_HET=1;N_HOMALT=0;N_HOMREF=10308;FREQ_HET=9.700261907071492e-05;FREQ_HOMALT=0.0;FREQ_HOMREF=0.9999029973809292;N_BI_GENOS=10309;Mongolian_AN=454;Mongolian_AC=1;Mongolian_A
chr3	60172911	.	A	<INS:ME:LINE1>	.	PASS	SVTYPE=INS:ME:LINE1;END=60172920;SVLEN=6010;AN=20618;AC=2;AF=9.700261907071492e-05;N_HET=2;N_HOMALT=0;N_HOMREF=10307;FREQ_HET=0.00019400523814142983;FREQ_HOMALT=0.0;FREQ_HOMREF=0.9998059947618586;N_BI_GENOS=10309;East_Han_AN=6554;East_Han_AC=2;East_Han_AF
chr3	60188818	.	A	<INS:ME:LINE1>	.	PASS	SVTYPE=INS:ME:LINE1;END=60188834;SVLEN=5997;AN=20618;AC=1;AF=4.850130953535746e-05;N_HET=1;N_HOMALT=0;N_HOMREF=10308;FREQ_HET=9.700261907071492e-05;FREQ_HOMALT=0.0;FREQ_HOMREF=0.9999029973809292;N_BI_GENOS=10309;Southeast_Han_AN=1908;Southeast_Han_AC=1;So
chr3	60897079	.	T	<INS:ME:LINE1>	.	PASS	SVTYPE=INS:ME:LINE1;END=60897096;SVLEN=1343;AN=20618;AC=4;AF=0.00019400523814142983;N_HET=4;N_HOMALT=0;N_HOMREF=10305;FREQ_HET=0.00038801047628285966;FREQ_HOMALT=0.0;FREQ_HOMREF=0.9996119895237171;N_BI_GENOS=10309;Lingnan_Han_AN=1888;Lingnan_Han_AC=1;Ling
chr3	61010975	.	T	<INS:ME:LINE1>	.	PASS	SVTYPE=INS:ME:LINE1;END=61010989;SVLEN=2446;AN=20618;AC=7;AF=0.0003395091667475022;N_HET=7;N_HOMALT=0;N_HOMREF=10302;FREQ_HET=0.0006790183334950044;FREQ_HOMALT=0.0;FREQ_HOMREF=0.999320981666505;N_BI_GENOS=10309;Northwest_Han_AN=1296;Northwest_Han_AC=1;Nor
chr3	61116694	.	G	<INS:ME:LINE1>	.	PASS	SVTYPE=INS:ME:LINE1;END=61116703;SVLEN=477;AN=20618;AC=1;AF=4.850130953535746e-05;N_HET=1;N_HOMALT=0;N_HOMREF=10308;FREQ_HET=9.700261907071492e-05;FREQ_HOMALT=0.0;FREQ_HOMREF=0.9999029973809292;N_BI_GENOS=10309;North_Han_AN=2272;North_Han_AC=1;North_Han_A
chr3	61199872	.	C	<INS:ME:LINE1>	.	PASS	SVTYPE=INS:ME:LINE1;END=61199887;SVLEN=1452;AN=20618;AC=15;AF=0.0007275196430303619;N_HET=15;N_HOMALT=0;N_HOMREF=10294;FREQ_HET=0.0014550392860607237;FREQ_HOMALT=0.0;FREQ_HOMREF=0.9985449607139393;N_BI_GENOS=10309;South_Han_AN=1496;South_Han_AC=2;South_Ha
chr3	61233921	.	T	<INS:ME:LINE1>	.	PASS	SVTYPE=INS:ME:LINE1;END=61233937;SVLEN=103;AN=20618;AC=1;AF=4.850130953535746e-05;N_HET=1;N_HOMALT=0;N_HOMREF=10308;FREQ_HET=9.700261907071492e-05;FREQ_HOMALT=0.0;FREQ_HOMREF=0.9999029973809292;N_BI_GENOS=10309;South_Han_AN=1496;South_Han_AC=1;South_Han_A
chr3	61957486	.	G	<INS:ME:LINE1>	.	PASS	SVTYPE=INS:ME:LINE1;END=61957501;SVLEN=6003;AN=20618;AC=1;AF=4.850130953535746e-05;N_HET=1;N_HOMALT=0;N_HOMREF=10308;FREQ_HET=9.700261907071492e-05;FREQ_HOMALT=0.0;FREQ_HOMREF=0.9999029973809292;N_BI_GENOS=10309;East_Han_AN=6554;East_Han_AC=1;East_Han_AF=
chr3	62607954	.	A	<INS:ME:LINE1>	.	PASS	SVTYPE=INS:ME:LINE1;END=62607971;SVLEN=5683;AN=20618;AC=1;AF=4.850130953535746e-05;N_HET=1;N_HOMALT=0;N_HOMREF=10308;FREQ_HET=9.700261907071492e-05;FREQ_HOMALT=0.0;FREQ_HOMREF=0.9999029973809292;N_BI_GENOS=10309;East_Han_AN=6554;East_Han_AC=1;East_Han_AF=
chr3	62768544	.	A	<INS:ME:LINE1>	.	PASS	SVTYPE=INS:ME:LINE1;END=62768560;SVLEN=5999;AN=20618;AC=1;AF=4.850130953535746e-05;N_HET=1;N_HOMALT=0;N_HOMREF=10308;FREQ_HET=9.700261907071492e-05;FREQ_HOMALT=0.0;FREQ_HOMREF=0.9999029973809292;N_BI_GENOS=10309;South_Han_AN=1496;South_Han_AC=1;South_Han_
chr3	63167742	.	T	<INS:ME:LINE1>	.	PASS	SVTYPE=INS:ME:LINE1;END=63167758;SVLEN=5998;AN=20618;AC=1;AF=4.850130953535746e-05;N_HET=1;N_HOMALT=0;N_HOMREF=10308;FREQ_HET=9.700261907071492e-05;FREQ_HOMALT=0.0;FREQ_HOMREF=0.9999029973809292;N_BI_GENOS=10309;Lingnan_Han_AN=1888;Lingnan_Han_AC=1;Lingna
chr3	63369655	.	T	<INS:ME:LINE1>	.	PASS	SVTYPE=INS:ME:LINE1;END=63369669;SVLEN=6023;AN=20618;AC=1;AF=4.850130953535746e-05;N_HET=1;N_HOMALT=0;N_HOMREF=10308;FREQ_HET=9.700261907071492e-05;FREQ_HOMALT=0.0;FREQ_HOMREF=0.9999029973809292;N_BI_GENOS=10309;Southeast_Han_AN=1908;Southeast_Han_AC=1;So
chr3	63423018	.	A	<INS:ME:LINE1>	.	PASS	SVTYPE=INS:ME:LINE1;END=63423030;SVLEN=4601;AN=20618;AC=2;AF=9.700261907071492e-05;N_HET=2;N_HOMALT=0;N_HOMREF=10307;FREQ_HET=0.00019400523814142983;FREQ_HOMALT=0.0;FREQ_HOMREF=0.9998059947618586;N_BI_GENOS=10309;Central_Han_AN=2128;Central_Han_AC=1;Centr
chr3	63483739	.	A	<INS:ME:LINE1>	.	PASS	SVTYPE=INS:ME:LINE1;END=63483755;SVLEN=6000;AN=20618;AC=1;AF=4.850130953535746e-05;N_HET=1;N_HOMALT=0;N_HOMREF=10308;FREQ_HET=9.700261907071492e-05;FREQ_HOMALT=0.0;FREQ_HOMREF=0.9999029973809292;N_BI_GENOS=10309;South_Han_AN=1496;South_Han_AC=1;South_Han_
chr3	64155148	.	A	<INS:ME:LINE1>	.	PASS	SVTYPE=INS:ME:LINE1;END=64155148;SVLEN=167;AN=20618;AC=1;AF=4.850130953535746e-05;N_HET=1;N_HOMALT=0;N_HOMREF=10308;FREQ_HET=9.700261907071492e-05;FREQ_HOMALT=0.0;FREQ_HOMREF=0.9999029973809292;N_BI_GENOS=10309;Southeast_Han_AN=1908;Southeast_Han_AC=1;Sou
chr3	64258845	.	A	<INS:ME:LINE1>	.	PASS	SVTYPE=INS:ME:LINE1;END=64258845;SVLEN=0;AN=20618;AC=2;AF=9.700261907071492e-05;N_HET=0;N_HOMALT=1;N_HOMREF=10308;FREQ_HET=0.0;FREQ_HOMALT=9.700261907071492e-05;FREQ_HOMREF=0.9999029973809292;N_BI_GENOS=10309;East_Han_AN=6554;East_Han_AC=2;East_Han_AF=0.0
chr3	65061445	.	A	<INS:ME:LINE1>	.	PASS	SVTYPE=INS:ME:LINE1;END=65061459;SVLEN=733;AN=20618;AC=1;AF=4.850130953535746e-05;N_HET=1;N_HOMALT=0;N_HOMREF=10308;FREQ_HET=9.700261907071492e-05;FREQ_HOMALT=0.0;FREQ_HOMREF=0.9999029973809292;N_BI_GENOS=10309;South_Han_AN=1496;South_Han_AC=1;South_Han_A
chr3	65161552	.	A	<INS:ME:LINE1>	.	PASS	SVTYPE=INS:ME:LINE1;END=65161565;SVLEN=6038;AN=20618;AC=1;AF=4.850130953535746e-05;N_HET=1;N_HOMALT=0;N_HOMREF=10308;FREQ_HET=9.700261907071492e-05;FREQ_HOMALT=0.0;FREQ_HOMREF=0.9999029973809292;N_BI_GENOS=10309;Manchu_AN=416;Manchu_AC=1;Manchu_AF=0.00240
chr3	65228006	.	T	<INS:ME:LINE1>	.	PASS	SVTYPE=INS:ME:LINE1;END=65228009;SVLEN=3170;AN=20618;AC=1;AF=4.850130953535746e-05;N_HET=1;N_HOMALT=0;N_HOMREF=10308;FREQ_HET=9.700261907071492e-05;FREQ_HOMALT=0.0;FREQ_HOMREF=0.9999029973809292;N_BI_GENOS=10309;Northwest_Han_AN=1296;Northwest_Han_AC=1;No
chr3	66262688	.	G	<INS:ME:LINE1>	.	PASS	SVTYPE=INS:ME:LINE1;END=66262698;SVLEN=6000;AN=20618;AC=1;AF=4.850130953535746e-05;N_HET=1;N_HOMALT=0;N_HOMREF=10308;FREQ_HET=9.700261907071492e-05;FREQ_HOMALT=0.0;FREQ_HOMREF=0.9999029973809292;N_BI_GENOS=10309;East_Han_AN=6554;East_Han_AC=1;East_Han_AF=
chr3	66360505	.	A	<INS:ME:LINE1>	.	PASS	SVTYPE=INS:ME:LINE1;END=66360519;SVLEN=536;AN=20618;AC=1;AF=4.850130953535746e-05;N_HET=1;N_HOMALT=0;N_HOMREF=10308;FREQ_HET=9.700261907071492e-05;FREQ_HOMALT=0.0;FREQ_HOMREF=0.9999029973809292;N_BI_GENOS=10309;East_Han_AN=6554;East_Han_AC=1;East_Han_AF=0
chr3	66516735	.	C	<INS:ME:LINE1>	.	PASS	SVTYPE=INS:ME:LINE1;END=66516749;SVLEN=6093;AN=20618;AC=1;AF=4.850130953535746e-05;N_HET=1;N_HOMALT=0;N_HOMREF=10308;FREQ_HET=9.700261907071492e-05;FREQ_HOMALT=0.0;FREQ_HOMREF=0.9999029973809292;N_BI_GENOS=10309;East_Han_AN=6554;East_Han_AC=1;East_Han_AF=
chr3	67026650	.	A	<INS:ME:LINE1>	.	PASS	SVTYPE=INS:ME:LINE1;END=67026666;SVLEN=762;AN=20618;AC=1;AF=4.850130953535746e-05;N_HET=1;N_HOMALT=0;N_HOMREF=10308;FREQ_HET=9.700261907071492e-05;FREQ_HOMALT=0.0;FREQ_HOMREF=0.9999029973809292;N_BI_GENOS=10309;Southeast_Han_AN=1908;Southeast_Han_AC=1;Sou
chr3	67354355	.	A	<INS:ME:LINE1>	.	PASS	SVTYPE=INS:ME:LINE1;END=67354370;SVLEN=1210;AN=20618;AC=1;AF=4.850130953535746e-05;N_HET=1;N_HOMALT=0;N_HOMREF=10308;FREQ_HET=9.700261907071492e-05;FREQ_HOMALT=0.0;FREQ_HOMREF=0.9999029973809292;N_BI_GENOS=10309;North_Han_AN=2272;North_Han_AC=1;North_Han_
chr3	67404792	.	C	<INS:ME:LINE1>	.	PASS	SVTYPE=INS:ME:LINE1;END=67404792;SVLEN=1081;AN=20618;AC=2;AF=9.700261907071492e-05;N_HET=2;N_HOMALT=0;N_HOMREF=10307;FREQ_HET=0.00019400523814142983;FREQ_HOMALT=0.0;FREQ_HOMREF=0.9998059947618586;N_BI_GENOS=10309;Southeast_Han_AN=1908;Southeast_Han_AC=2;S
chr3	67533938	.	C	<INS:ME:LINE1>	.	PASS	SVTYPE=INS:ME:LINE1;END=67533939;SVLEN=1062;AN=20618;AC=1088;AF=0.05276942477446891;N_HET=1000;N_HOMALT=44;N_HOMREF=9265;FREQ_HET=0.0970026190707149;FREQ_HOMALT=0.004268115239111456;FREQ_HOMREF=0.8987292656901736;N_BI_GENOS=10309;South_Han_AN=1496;South_H
chr3	67743277	.	A	<INS:ME:LINE1>	.	PASS	SVTYPE=INS:ME:LINE1;END=67743277;SVLEN=219;AN=20618;AC=1;AF=4.850130953535746e-05;N_HET=1;N_HOMALT=0;N_HOMREF=10308;FREQ_HET=9.700261907071492e-05;FREQ_HOMALT=0.0;FREQ_HOMREF=0.9999029973809292;N_BI_GENOS=10309;East_Han_AN=6554;East_Han_AC=1;East_Han_AF=0
chr3	67800468	.	C	<INS:ME:LINE1>	.	PASS	SVTYPE=INS:ME:LINE1;END=67800468;SVLEN=496;AN=20618;AC=1;AF=4.850130953535746e-05;N_HET=1;N_HOMALT=0;N_HOMREF=10308;FREQ_HET=9.700261907071492e-05;FREQ_HOMALT=0.0;FREQ_HOMREF=0.9999029973809292;N_BI_GENOS=10309;East_Han_AN=6554;East_Han_AC=1;East_Han_AF=0
chr3	68499706	.	A	<INS:ME:LINE1>	.	PASS	SVTYPE=INS:ME:LINE1;END=68499722;SVLEN=1006;AN=20618;AC=2;AF=9.700261907071492e-05;N_HET=0;N_HOMALT=1;N_HOMREF=10308;FREQ_HET=0.0;FREQ_HOMALT=9.700261907071492e-05;FREQ_HOMREF=0.9999029973809292;N_BI_GENOS=10309;North_Han_AN=2272;North_Han_AC=2;North_Han_
chr3	68605478	.	C	<INS:ME:LINE1>	.	PASS	SVTYPE=INS:ME:LINE1;END=68605478;SVLEN=748;AN=20618;AC=1;AF=4.850130953535746e-05;N_HET=1;N_HOMALT=0;N_HOMREF=10308;FREQ_HET=9.700261907071492e-05;FREQ_HOMALT=0.0;FREQ_HOMREF=0.9999029973809292;N_BI_GENOS=10309;East_Han_AN=6554;East_Han_AC=1;East_Han_AF=0
chr3	68818062	.	G	<INS:ME:LINE1>	.	PASS	SVTYPE=INS:ME:LINE1;END=68818077;SVLEN=391;AN=20618;AC=2;AF=9.700261907071492e-05;N_HET=2;N_HOMALT=0;N_HOMREF=10307;FREQ_HET=0.00019400523814142983;FREQ_HOMALT=0.0;FREQ_HOMREF=0.9998059947618586;N_BI_GENOS=10309;North_Han_AN=2272;North_Han_AC=1;North_Han_
chr3	68849228	.	A	<INS:ME:LINE1>	.	PASS	SVTYPE=INS:ME:LINE1;END=68849241;SVLEN=6057;AN=20618;AC=1;AF=4.850130953535746e-05;N_HET=1;N_HOMALT=0;N_HOMREF=10308;FREQ_HET=9.700261907071492e-05;FREQ_HOMALT=0.0;FREQ_HOMREF=0.9999029973809292;N_BI_GENOS=10309;East_Han_AN=6554;East_Han_AC=1;East_Han_AF=
chr3	69026185	.	A	<INS:ME:LINE1>	.	PASS	SVTYPE=INS:ME:LINE1;END=69026200;SVLEN=6007;AN=20618;AC=1;AF=4.850130953535746e-05;N_HET=1;N_HOMALT=0;N_HOMREF=10308;FREQ_HET=9.700261907071492e-05;FREQ_HOMALT=0.0;FREQ_HOMREF=0.9999029973809292;N_BI_GENOS=10309;Tibetan_AN=414;Tibetan_AC=1;Tibetan_AF=0.00
chr3	69132390	.	T	<INS:ME:LINE1>	.	PASS	SVTYPE=INS:ME:LINE1;END=69132390;SVLEN=426;AN=20618;AC=537;AF=0.026045203220486954;N_HET=363;N_HOMALT=87;N_HOMREF=9859;FREQ_HET=0.03521195072266951;FREQ_HOMALT=0.008439227859152198;FREQ_HOMREF=0.9563488214181783;N_BI_GENOS=10309;South_Han_AN=1496;South_Ha
chr3	69325128	.	A	<INS:ME:LINE1>	.	PASS	SVTYPE=INS:ME:LINE1;END=69325128;SVLEN=0;AN=20618;AC=4;AF=0.00019400523814142983;N_HET=0;N_HOMALT=2;N_HOMREF=10307;FREQ_HET=0.0;FREQ_HOMALT=0.00019400523814142983;FREQ_HOMREF=0.9998059947618586;N_BI_GENOS=10309;East_Han_AN=6554;East_Han_AC=4;East_Han_AF=0
chr3	69510261	.	G	<INS:ME:LINE1>	.	PASS	SVTYPE=INS:ME:LINE1;END=69510272;SVLEN=479;AN=20618;AC=1;AF=4.850130953535746e-05;N_HET=1;N_HOMALT=0;N_HOMREF=10308;FREQ_HET=9.700261907071492e-05;FREQ_HOMALT=0.0;FREQ_HOMREF=0.9999029973809292;N_BI_GENOS=10309;Zhuang_AN=444;Zhuang_AC=1;Zhuang_AF=0.002252
chr3	69528027	.	C	<INS:ME:LINE1>	.	PASS	SVTYPE=INS:ME:LINE1;END=69528027;SVLEN=5952;AN=20618;AC=1;AF=4.850130953535746e-05;N_HET=1;N_HOMALT=0;N_HOMREF=10308;FREQ_HET=9.700261907071492e-05;FREQ_HOMALT=0.0;FREQ_HOMREF=0.9999029973809292;N_BI_GENOS=10309;Southeast_Han_AN=1908;Southeast_Han_AC=1;So
chr3	69824134	.	A	<INS:ME:LINE1>	.	PASS	SVTYPE=INS:ME:LINE1;END=69824134;SVLEN=564;AN=20618;AC=3;AF=0.00014550392860607236;N_HET=3;N_HOMALT=0;N_HOMREF=10306;FREQ_HET=0.0002910078572121447;FREQ_HOMALT=0.0;FREQ_HOMREF=0.9997089921427879;N_BI_GENOS=10309;Lingnan_Han_AN=1888;Lingnan_Han_AC=2;Lingna
chr3	69839402	.	C	<INS:ME:LINE1>	.	PASS	SVTYPE=INS:ME:LINE1;END=69839402;SVLEN=6015;AN=20618;AC=1;AF=4.850130953535746e-05;N_HET=1;N_HOMALT=0;N_HOMREF=10308;FREQ_HET=9.700261907071492e-05;FREQ_HOMALT=0.0;FREQ_HOMREF=0.9999029973809292;N_BI_GENOS=10309;East_Han_AN=6554;East_Han_AC=1;East_Han_AF=
chr3	70300436	.	A	<INS:ME:LINE1>	.	PASS	SVTYPE=INS:ME:LINE1;END=70300450;SVLEN=6000;AN=20618;AC=1;AF=4.850130953535746e-05;N_HET=1;N_HOMALT=0;N_HOMREF=10308;FREQ_HET=9.700261907071492e-05;FREQ_HOMALT=0.0;FREQ_HOMREF=0.9999029973809292;N_BI_GENOS=10309;South_Han_AN=1496;South_Han_AC=1;South_Han_
chr3	70350833	.	G	<INS:ME:LINE1>	.	PASS	SVTYPE=INS:ME:LINE1;END=70350847;SVLEN=3813;AN=20618;AC=1;AF=4.850130953535746e-05;N_HET=1;N_HOMALT=0;N_HOMREF=10308;FREQ_HET=9.700261907071492e-05;FREQ_HOMALT=0.0;FREQ_HOMREF=0.9999029973809292;N_BI_GENOS=10309;South_Han_AN=1496;South_Han_AC=1;South_Han_
chr3	70439182	.	T	<INS:ME:LINE1>	.	PASS	SVTYPE=INS:ME:LINE1;END=70439182;SVLEN=0;AN=20618;AC=1;AF=4.850130953535746e-05;N_HET=1;N_HOMALT=0;N_HOMREF=10308;FREQ_HET=9.700261907071492e-05;FREQ_HOMALT=0.0;FREQ_HOMREF=0.9999029973809292;N_BI_GENOS=10309;Northwest_Han_AN=1296;Northwest_Han_AC=1;North
chr3	70728322	.	A	<INS:ME:LINE1>	.	PASS	SVTYPE=INS:ME:LINE1;END=70728339;SVLEN=5997;AN=20618;AC=10;AF=0.00048501309535357455;N_HET=10;N_HOMALT=0;N_HOMREF=10299;FREQ_HET=0.0009700261907071491;FREQ_HOMALT=0.0;FREQ_HOMREF=0.9990299738092928;N_BI_GENOS=10309;South_Han_AN=1496;South_Han_AC=1;South_H
chr3	70864213	.	G	<INS:ME:LINE1>	.	PASS	SVTYPE=INS:ME:LINE1;END=70864229;SVLEN=5998;AN=20618;AC=4;AF=0.00019400523814142983;N_HET=4;N_HOMALT=0;N_HOMREF=10305;FREQ_HET=0.00038801047628285966;FREQ_HOMALT=0.0;FREQ_HOMREF=0.9996119895237171;N_BI_GENOS=10309;Miao_AN=482;Miao_AC=4;Miao_AF=0.008298755
chr3	70937021	.	C	<INS:ME:LINE1>	.	PASS	SVTYPE=INS:ME:LINE1;END=70937038;SVLEN=1673;AN=20618;AC=6;AF=0.0002910078572121447;N_HET=4;N_HOMALT=1;N_HOMREF=10304;FREQ_HET=0.00038801047628285966;FREQ_HOMALT=9.700261907071492e-05;FREQ_HOMREF=0.9995149869046465;N_BI_GENOS=10309;South_Han_AN=1496;South_
chr3	71217028	.	A	<INS:ME:LINE1>	.	PASS	SVTYPE=INS:ME:LINE1;END=71217048;SVLEN=1471;AN=20618;AC=3;AF=0.00014550392860607236;N_HET=3;N_HOMALT=0;N_HOMREF=10306;FREQ_HET=0.0002910078572121447;FREQ_HOMALT=0.0;FREQ_HOMREF=0.9997089921427879;N_BI_GENOS=10309;Lingnan_Han_AN=1888;Lingnan_Han_AC=1;Lingn
chr3	71658126	.	G	<INS:ME:LINE1>	.	PASS	SVTYPE=INS:ME:LINE1;END=71658136;SVLEN=6002;AN=20618;AC=1;AF=4.850130953535746e-05;N_HET=1;N_HOMALT=0;N_HOMREF=10308;FREQ_HET=9.700261907071492e-05;FREQ_HOMALT=0.0;FREQ_HOMREF=0.9999029973809292;N_BI_GENOS=10309;South_Han_AN=1496;South_Han_AC=1;South_Han_
chr3	73056087	.	A	<INS:ME:LINE1>	.	PASS	SVTYPE=INS:ME:LINE1;END=73056104;SVLEN=434;AN=20618;AC=1;AF=4.850130953535746e-05;N_HET=1;N_HOMALT=0;N_HOMREF=10308;FREQ_HET=9.700261907071492e-05;FREQ_HOMALT=0.0;FREQ_HOMREF=0.9999029973809292;N_BI_GENOS=10309;Northwest_Han_AN=1296;Northwest_Han_AC=1;Nor
chr3	73115957	.	A	<INS:ME:LINE1>	.	PASS	SVTYPE=INS:ME:LINE1;END=73115972;SVLEN=6006;AN=20618;AC=1;AF=4.850130953535746e-05;N_HET=1;N_HOMALT=0;N_HOMREF=10308;FREQ_HET=9.700261907071492e-05;FREQ_HOMALT=0.0;FREQ_HOMREF=0.9999029973809292;N_BI_GENOS=10309;Central_Han_AN=2128;Central_Han_AC=1;Centra
chr3	73234352	.	A	<INS:ME:LINE1>	.	PASS	SVTYPE=INS:ME:LINE1;END=73234365;SVLEN=6008;AN=20618;AC=1;AF=4.850130953535746e-05;N_HET=1;N_HOMALT=0;N_HOMREF=10308;FREQ_HET=9.700261907071492e-05;FREQ_HOMALT=0.0;FREQ_HOMREF=0.9999029973809292;N_BI_GENOS=10309;North_Han_AN=2272;North_Han_AC=1;North_Han_
chr3	73394756	.	G	<INS:ME:LINE1>	.	PASS	SVTYPE=INS:ME:LINE1;END=73394756;SVLEN=333;AN=20618;AC=1;AF=4.850130953535746e-05;N_HET=1;N_HOMALT=0;N_HOMREF=10308;FREQ_HET=9.700261907071492e-05;FREQ_HOMALT=0.0;FREQ_HOMREF=0.9999029973809292;N_BI_GENOS=10309;North_Han_AN=2272;North_Han_AC=1;North_Han_A

chr3	73779889	.	A	<INS:ME:LINE1>	.	PASS	SVTYPE=INS:ME:LINE1;END=73779906;SVLEN=1358;AN=20618;AC=1;AF=4.850130953535746e-05;N_HET=1;N_HOMALT=0;N_HOMREF=10308;FREQ_HET=9.700261907071492e-05;FREQ_HOMALT=0.0;FREQ_HOMREF=0.9999029973809292;N_BI_GENOS=10309;Central_Han_AN=2128;Central_Han_AC=1;Centra
chr3	73805931	.	A	<INS:ME:LINE1>	.	PASS	SVTYPE=INS:ME:LINE1;END=73805931;SVLEN=5978;AN=20618;AC=1;AF=4.850130953535746e-05;N_HET=1;N_HOMALT=0;N_HOMREF=10308;FREQ_HET=9.700261907071492e-05;FREQ_HOMALT=0.0;FREQ_HOMREF=0.9999029973809292;N_BI_GENOS=10309;South_Han_AN=1496;South_Han_AC=1;South_Han_
chr3	73914274	.	A	<INS:ME:LINE1>	.	PASS	SVTYPE=INS:ME:LINE1;END=73914289;SVLEN=865;AN=20618;AC=3;AF=0.0001455039286060 7236;N_HET=3;N_HOMALT=0;N_HOMREF=10306;FREQ_HET=0.0002910078572121447;FREQ_HOMALT=0.0;FREQ_HOMREF=0.9997089921427879;N_BI_GENOS=10309;Northwest_Han_AN=1296;Northwest_Han_AC=1;No
chr3	74041861	.	A	<INS:ME:LINE1>	.	PASS	SVTYPE=INS:ME:LINE1;END=74041861;SVLEN=1308;AN=20618;AC=1;AF=4.850130953535746e-05;N_HET=1;N_HOMALT=0;N_HOMREF=10308;FREQ_HET=9.700261907071492e-05;FREQ_HOMALT=0.0;FREQ_HOMREF=0.9999029973809292;N_BI_GENOS=10309;Northwest_Han_AN=1296;Northwest_Han_AC=1;No
chr3	74155068	.	G	<INS:ME:LINE1>	.	PASS	SVTYPE=INS:ME:LINE1;END=74155079;SVLEN=528;AN=20618;AC=1;AF=4.850130953535746e-05;N_HET=1;N_HOMALT=0;N_HOMREF=10308;FREQ_HET=9.700261907071492e-05;FREQ_HOMALT=0.0;FREQ_HOMREF=0.9999029973809292;N_BI_GENOS=10309;North_Han_AN=2272;North_Han_AC=1;North_Han_A
chr3	74250458	.	A	<INS:ME:LINE1>	.	PASS	SVTYPE=INS:ME:LINE1;END=74250476;SVLEN=875;AN=20618;AC=6;AF=0.0002910078572121447;N_HET=6;N_HOMALT=0;N_HOMREF=10303;FREQ_HET=0.0005820157144242894;FREQ_HOMALT=0.0;FREQ_HOMREF=0.9994179842855757;N_BI_GENOS=10309;Northwest_Han_AN=1296;Northwest_Han_AC=2;Nor
chr3	74629711	.	A	<INS:ME:LINE1>	.	PASS	SVTYPE=INS:ME:LINE1;END=74629722;SVLEN=6000;AN=20618;AC=17;AF=0.0008245222621010767;N_HET=17;N_HOMALT=0;N_HOMREF=10292;FREQ_HET=0.001649044524202153 4;FREQ_HOMALT=0.0;FREQ_HOMREF=0.9983509554757979;N_BI_GENOS=10309;South_Han_AN=1496;South_Han_AC=1;South_Ha
chr3	74631910	.	A	<INS:ME:LINE1>	.	PASS	SVTYPE=INS:ME:LINE1;END=74631910;SVLEN=1542;AN=20618;AC=5;AF=0.0002425065476767872 8;N_HET=5;N_HOMALT=0;N_HOMREF=10304;FREQ_HET=0.0004850130953535745 5;FREQ_HOMALT=0.0;FREQ_HOMREF=0.9995149869046465;N_BI_GENOS=10309;South_Han_AN=1496;South_Han_AC=2;South_Ha
chr3	74831207	.	G	<INS:ME:LINE1>	.	PASS	SVTYPE=INS:ME:LINE1;END=74831222;SVLEN=2145;AN=20618;AC=1;AF=4.850130953535746e-05;N_HET=1;N_HOMALT=0;N_HOMREF=10308;FREQ_HET=9.700261907071492e-05;FREQ_HOMALT=0.0;FREQ_HOMREF=0.9999029973809292;N_BI_GENOS=10309;Central_Han_AN=2128;Central_Han_AC=1;Centra
chr3	74981695	.	T	<INS:ME:LINE1>	.	PASS	SVTYPE=INS:ME:LINE1;END=74981712;SVLEN=5987;AN=20618;AC=1;AF=4.850130953535746e-05;N_HET=1;N_HOMALT=0;N_HOMREF=10308;FREQ_HET=9.700261907071492e-05;FREQ_HOMALT=0.0;FREQ_HOMREF=0.9999029973809292;N_BI_GENOS=10309;East_Han_AN=6554;East_Han_AC=1;East_Han_AF=
chr3	74982317	.	A	<INS:ME:LINE1>	.	PASS	SVTYPE=INS:ME:LINE1;END=74982317;SVLEN=97;AN=20618;AC=1;AF=4.850130953535746e-05;N_HET=1;N_HOMALT=0;N_HOMREF=10308;FREQ_HET=9.700261907071492e-05;FREQ_HOMALT=0.0;FREQ_HOMREF=0.9999029973809292;N_BI_GENOS=10309;East_Han_AN=6554;East_Han_AC=1;East_Han_AF=0.
chr3	74997383	.	A	<INS:ME:LINE1>	.	PASS	SVTYPE=INS:ME:LINE1;END=74997399;SVLEN=74;AN=20618;AC=1;AF=4.850130953535746e-05;N_HET=1;N_HOMALT=0;N_HOMREF=10308;FREQ_HET=9.700261907071492e-05;FREQ_HOMALT=0.0;FREQ_HOMREF=0.9999029973809292;N_BI_GENOS=10309;East_Han_AN=6554;East_Han_AC=1;East_Han_AF=0.
chr3	75052065	.	T	<INS:ME:LINE1>	.	PASS	SVTYPE=INS:ME:LINE1;END=75052066;SVLEN=190;AN=20618;AC=12498;AF=0.6061693665728974;N_HET=4496;N_HOMALT=4001;N_HOMREF=1812;FREQ_HET=0.43612377534193425;FREQ_HOMALT=0.38810747890193037;FREQ_HOMREF=0.1757687457561354;N_BI_GENOS=10309;South_Han_AN=1496;South_
chr3	75143966	.	A	<INS:ME:LINE1>	.	PASS	SVTYPE=INS:ME:LINE1;END=75143978;SVLEN=6021;AN=20618;AC=1;AF=4.850130953535746e-05;N_HET=1;N_HOMALT=0;N_HOMREF=10308;FREQ_HET=9.700261907071492e-05;FREQ_HOMALT=0.0;FREQ_HOMREF=0.9999029973809292;N_BI_GENOS=10309;Central_Han_AN=2128;Central_Han_AC=1;Centra
chr3	75538328	.	A	<INS:ME:LINE1>	.	PASS	SVTYPE=INS:ME:LINE1;END=75538328;SVLEN=0;AN=20618;AC=58;AF=0.0028130759530507325;N_HET=56;N_HOMALT=1;N_HOMREF=10252;FREQ_HET=0.005432146667960035;FREQ_HOMALT=9.700261907071492e-05;FREQ_HOMREF=0.9944708507129693;N_BI_GENOS=10309;South_Han_AN=1496;South_Han
chr3	75540674	.	C	<INS:ME:LINE1>	.	PASS	SVTYPE=INS:ME:LINE1;END=75540674;SVLEN=0;AN=20618;AC=12;AF=0.0005820157144242894;N_HET=4;N_HOMALT=4;N_HOMREF=10301;FREQ_HET=0.00038801047628285966;FREQ_HOMALT=0.00038801047628285966;FREQ_HOMREF=0.9992239790474343;N_BI_GENOS=10309;South_Han_AN=1496;South_H
chr3	75689121	.	C	<INS:ME:LINE1>	.	PASS	SVTYPE=INS:ME:LINE1;END=75689121;SVLEN=0;AN=20618;AC=1;AF=4.850130953535746e-05;N_HET=1;N_HOMALT=0;N_HOMREF=10308;FREQ_HET=9.700261907071492e-05;FREQ_HOMALT=0.0;FREQ_HOMREF=0.9999029973809292;N_BI_GENOS=10309;Lingnan_Han_AN=1888;Lingnan_Han_AC=1;Lingnan_H
chr3	75693544	.	A	<INS:ME:LINE1>	.	PASS	SVTYPE=INS:ME:LINE1;END=75693544;SVLEN=0;AN=20618;AC=2;AF=9.700261907071492e-05;N_HET=2;N_HOMALT=0;N_HOMREF=10307;FREQ_HET=0.00019400523814142983;FREQ_HOMALT=0.0;FREQ_HOMREF=0.9998059947618586;N_BI_GENOS=10309;North_Han_AN=2272;North_Han_AC=1;North_Han_AF
chr3	75694083	.	A	<INS:ME:LINE1>	.	PASS	SVTYPE=INS:ME:LINE1;END=75694083;SVLEN=0;AN=20618;AC=2;AF=9.700261907071492e-05;N_HET=2;N_HOMALT=0;N_HOMREF=10307;FREQ_HET=0.00019400523814142983;FREQ_HOMALT=0.0;FREQ_HOMREF=0.9998059947618586;N_BI_GENOS=10309;Northwest_Han_AN=1296;Northwest_Han_AC=1;Nort
chr3	75695142	.	G	<INS:ME:LINE1>	.	PASS	SVTYPE=INS:ME:LINE1;END=75695142;SVLEN=0;AN=20618;AC=1;AF=4.850130953535746e-05;N_HET=1;N_HOMALT=0;N_HOMREF=10308;FREQ_HET=9.700261907071492e-05;FREQ_HOMALT=0.0;FREQ_HOMREF=0.9999029973809292;N_BI_GENOS=10309;East_Han_AN=6554;East_Han_AC=1;East_Han_AF=0.0
chr3	75696022	.	C	<INS:ME:LINE1>	.	PASS	SVTYPE=INS:ME:LINE1;END=75696022;SVLEN=2402;AN=20618;AC=1;AF=4.850130953535746e-05;N_HET=1;N_HOMALT=0;N_HOMREF=10308;FREQ_HET=9.700261907071492e-05;FREQ_HOMALT=0.0;FREQ_HOMREF=0.9999029973809292;N_BI_GENOS=10309;East_Han_AN=6554;East_Han_AC=1;East_Han_AF=
chr3	75706830	.	T	<INS:ME:LINE1>	.	PASS	SVTYPE=INS:ME:LINE1;END=75706830;SVLEN=0;AN=20618;AC=1;AF=4.850130953535746e-05;N_HET=1;N_HOMALT=0;N_HOMREF=10308;FREQ_HET=9.700261907071492e-05;FREQ_HOMALT=0.0;FREQ_HOMREF=0.9999029973809292;N_BI_GENOS=10309;East_Han_AN=6554;East_Han_AC=1;East_Han_AF=0.0
chr3	75951637	.	G	<INS:ME:LINE1>	.	PASS	SVTYPE=INS:ME:LINE1;END=75951654;SVLEN=929;AN=20618;AC=2;AF=9.700261907071492e-05;N_HET=2;N_HOMALT=0;N_HOMREF=10307;FREQ_HET=0.00019400523814142983;FREQ_HOMALT=0.0;FREQ_HOMREF=0.9998059947618586;N_BI_GENOS=10309;East_Han_AN=6554;East_Han_AC=1;East_Han_AF=
chr3	76268550	.	A	<INS:ME:LINE1>	.	PASS	SVTYPE=INS:ME:LINE1;END=76268557;SVLEN=469;AN=20618;AC=2;AF=9.700261907071492e-05;N_HET=0;N_HOMALT=1;N_HOMREF=10308;FREQ_HET=0.0;FREQ_HOMALT=9.700261907071492e-05;FREQ_HOMREF=0.9999029973809292;N_BI_GENOS=10309;South_Han_AN=1496;South_Han_AC=2;South_Han_A
chr3	76289217	.	G	<INS:ME:LINE1>	.	PASS	SVTYPE=INS:ME:LINE1;END=76289239;SVLEN=732;AN=20618;AC=1;AF=4.850130953535746e-05;N_HET=1;N_HOMALT=0;N_HOMREF=10308;FREQ_HET=9.700261907071492e-05;FREQ_HOMALT=0.0;FREQ_HOMREF=0.9999029973809292;N_BI_GENOS=10309;East_Han_AN=6554;East_Han_AC=1;East_Han_AF=0
chr3	76886974	.	A	<INS:ME:LINE1>	.	PASS	SVTYPE=INS:ME:LINE1;END=76886990;SVLEN=638;AN=20618;AC=1;AF=4.850130953535746e-05;N_HET=1;N_HOMALT=0;N_HOMREF=10308;FREQ_HET=9.700261907071492e-05;FREQ_HOMALT=0.0;FREQ_HOMREF=0.9999029973809292;N_BI_GENOS=10309;Tibetan_AN=414;Tibetan_AC=1;Tibetan_AF=0.002
chr3	77130474	.	A	<INS:ME:LINE1>	.	PASS	SVTYPE=INS:ME:LINE1;END=77130483;SVLEN=4515;AN=20618;AC=1;AF=4.850130953535746e-05;N_HET=1;N_HOMALT=0;N_HOMREF=10308;FREQ_HET=9.700261907071492e-05;FREQ_HOMALT=0.0;FREQ_HOMREF=0.9999029973809292;N_BI_GENOS=10309;North_Han_AN=2272;North_Han_AC=1;North_Han_
chr3	77440716	.	T	<INS:ME:LINE1>	.	PASS	SVTYPE=INS:ME:LINE1;END=77440729;SVLEN=691;AN=20618;AC=1;AF=4.850130953535746e-05;N_HET=1;N_HOMALT=0;N_HOMREF=10308;FREQ_HET=9.700261907071492e-05;FREQ_HOMALT=0.0;FREQ_HOMREF=0.9999029973809292;N_BI_GENOS=10309;North_Han_AN=2272;North_Han_AC=1;North_Han_A
chr3	77501901	.	A	<INS:ME:LINE1>	.	PASS	SVTYPE=INS:ME:LINE1;END=77501906;SVLEN=257;AN=20618;AC=8;AF=0.00038801047628285966;N_HET=8;N_HOMALT=0;N_HOMREF=10301;FREQ_HET=0.0007760209525657193;FREQ_HOMALT=0.0;FREQ_HOMREF=0.9992239790474343;N_BI_GENOS=10309;North_Han_AN=2272;North_Han_AC=3;North_Han_
chr3	77684998	.	G	<INS:ME:LINE1>	.	PASS	SVTYPE=INS:ME:LINE1;END=77685012;SVLEN=604;AN=20618;AC=1;AF=4.850130953535746e-05;N_HET=1;N_HOMALT=0;N_HOMREF=10308;FREQ_HET=9.700261907071492e-05;FREQ_HOMALT=0.0;FREQ_HOMREF=0.9999029973809292;N_BI_GENOS=10309;Miao_AN=482;Miao_AC=1;Miao_AF=0.002074688796
chr3	77875058	.	A	<INS:ME:LINE1>	.	PASS	SVTYPE=INS:ME:LINE1;END=77875074;SVLEN=932;AN=20618;AC=303;AF=0.01469589678921331;N_HET=279;N_HOMALT=12;N_HOMREF=10018;FREQ_HET=0.02706373072072946;FREQ_HOMALT=0.0011640314288485789;FREQ_HOMREF=0.971772237850422;N_BI_GENOS=10309;South_Han_AN=1496;South_Ha
chr3	78044514	.	T	<INS:ME:LINE1>	.	PASS	SVTYPE=INS:ME:LINE1;END=78044529;SVLEN=484;AN=20618;AC=1;AF=4.850130953535746e-05;N_HET=1;N_HOMALT=0;N_HOMREF=10308;FREQ_HET=9.700261907071492e-05;FREQ_HOMALT=0.0;FREQ_HOMREF=0.9999029973809292;N_BI_GENOS=10309;Southeast_Han_AN=1908;Southeast_Han_AC=1;Sou
chr3	78316826	.	A	<INS:ME:LINE1>	.	PASS	SVTYPE=INS:ME:LINE1;END=78316842;SVLEN=1345;AN=20618;AC=2;AF=9.700261907071492e-05;N_HET=2;N_HOMALT=0;N_HOMREF=10307;FREQ_HET=0.00019400523814142983;FREQ_HOMALT=0.0;FREQ_HOMREF=0.9998059947618586;N_BI_GENOS=10309;Hui_AN=444;Hui_AC=2;Hui_AF=0.0045045045045
chr3	78475711	.	A	<INS:ME:LINE1>	.	PASS	SVTYPE=INS:ME:LINE1;END=78475711;SVLEN=718;AN=20618;AC=1;AF=4.850130953535746e-05;N_HET=1;N_HOMALT=0;N_HOMREF=10308;FREQ_HET=9.700261907071492e-05;FREQ_HOMALT=0.0;FREQ_HOMREF=0.9999029973809292;N_BI_GENOS=10309;East_Han_AN=6554;East_Han_AC=1;East_Han_AF=0
chr3	78587779	.	C	<INS:ME:LINE1>	.	PASS	SVTYPE=INS:ME:LINE1;END=78587779;SVLEN=0;AN=20618;AC=27;AF=0.0013095353574546513;N_HET=7;N_HOMALT=10;N_HOMREF=10292;FREQ_HET=0.0006790183334950044;FREQ_HOMALT=0.0009700261907071491;FREQ_HOMREF=0.9983509554757979;N_BI_GENOS=10309;South_Han_AN=1496;South_Ha
chr3	78787302	.	A	<INS:ME:LINE1>	.	PASS	SVTYPE=INS:ME:LINE1;END=78787315;SVLEN=858;AN=20618;AC=1;AF=4.850130953535746e-05;N_HET=1;N_HOMALT=0;N_HOMREF=10308;FREQ_HET=9.700261907071492e-05;FREQ_HOMALT=0.0;FREQ_HOMREF=0.9999029973809292;N_BI_GENOS=10309;Central_Han_AN=2128;Central_Han_AC=1;Central
chr3	79087354	.	C	<INS:ME:LINE1>	.	PASS	SVTYPE=INS:ME:LINE1;END=79087354;SVLEN=742;AN=20618;AC=1;AF=4.850130953535746e-05;N_HET=1;N_HOMALT=0;N_HOMREF=10308;FREQ_HET=9.700261907071492e-05;FREQ_HOMALT=0.0;FREQ_HOMREF=0.9999029973809292;N_BI_GENOS=10309;East_Han_AN=6554;East_Han_AC=1;East_Han_AF=0
chr3	79104776	.	C	<INS:ME:LINE1>	.	PASS	SVTYPE=INS:ME:LINE1;END=79104791;SVLEN=3151;AN=20618;AC=2;AF=9.700261907071492e-05;N_HET=2;N_HOMALT=0;N_HOMREF=10307;FREQ_HET=0.00019400523814142983;FREQ_HOMALT=0.0;FREQ_HOMREF=0.9998059947618586;N_BI_GENOS=10309;South_Han_AN=1496;South_Han_AC=1;South_Han
chr3	79167148	.	A	<INS:ME:LINE1>	.	PASS	SVTYPE=INS:ME:LINE1;END=79167163;SVLEN=6000;AN=20618;AC=33;AF=0.0016005432146667 96;N_HET=31;N_HOMALT=1;N_HOMREF=10277;FREQ_HET=0.0030070811911921624;FREQ_HOMALT=9.700261907071492e-05;FREQ_HOMREF=0.9968959161897372;N_BI_GENOS=10309;South_Han_AN=1496;South_
chr3	79200662	.	G	<INS:ME:LINE1>	.	PASS	SVTYPE=INS:ME:LINE1;END=79200677;SVLEN=1980;AN=20618;AC=1;AF=4.850130953535746e-05;N_HET=1;N_HOMALT=0;N_HOMREF=10308;FREQ_HET=9.700261907071492e-05;FREQ_HOMALT=0.0;FREQ_HOMREF=0.9999029973809292;N_BI_GENOS=10309;East_Han_AN=6554;East_Han_AC=1;East_Han_AF=
chr3	79290695	.	A	<INS:ME:LINE1>	.	PASS	SVTYPE=INS:ME:LINE1;END=79290713;SVLEN=5238;AN=20618;AC=32;AF=0.0015520419051314386;N_HET=30;N_HOMALT=1;N_HOMREF=10278;FREQ_HET=0.0029100785721214474;FREQ_HOMALT=9.700261907071492e-05;FREQ_HOMREF=0.9969929188088078;N_BI_GENOS=10309;South_Han_AN=1496;South
chr3	79496387	.	T	<INS:ME:LINE1>	.	PASS	SVTYPE=INS:ME:LINE1;END=79496387;SVLEN=0;AN=20618;AC=2;AF=9.700261907071492e-05;N_HET=0;N_HOMALT=1;N_HOMREF=10308;FREQ_HET=0.0;FREQ_HOMALT=9.700261907071492e-05;FREQ_HOMREF=0.9999029973809292;N_BI_GENOS=10309;North_Han_AN=2272;North_Han_AC=2;North_Han_AF=
chr3	80172454	.	T	<INS:ME:LINE1>	.	PASS	SVTYPE=INS:ME:LINE1;END=80172457;SVLEN=1398;AN=20618;AC=2;AF=9.700261907071492e-05;N_HET=2;N_HOMALT=0;N_HOMREF=10307;FREQ_HET=0.00019400523814142983;FREQ_HOMALT=0.0;FREQ_HOMREF=0.9998059947618586;N_BI_GENOS=10309;Northwest_Han_AN=1296;Northwest_Han_AC=1;N
chr3	80199334	.	A	<INS:ME:LINE1>	.	PASS	SVTYPE=INS:ME:LINE1;END=80199349;SVLEN=2662;AN=20618;AC=1;AF=4.850130953535746e-05;N_HET=1;N_HOMALT=0;N_HOMREF=10308;FREQ_HET=9.700261907071492e-05;FREQ_HOMALT=0.0;FREQ_HOMREF=0.9999029973809292;N_BI_GENOS=10309;North_Han_AN=2272;North_Han_AC=1;North_Han_
chr3	80236902	.	T	<INS:ME:LINE1>	.	PASS	SVTYPE=INS:ME:LINE1;END=80236909;SVLEN=995;AN=20618;AC=1;AF=4.850130953535746e-05;N_HET=1;N_HOMALT=0;N_HOMREF=10308;FREQ_HET=9.700261907071492e-05;FREQ_HOMALT=0.0;FREQ_HOMREF=0.9999029973809292;N_BI_GENOS=10309;South_Han_AN=1496;South_Han_AC=1;South_Han_A
chr3	80241669	.	A	<INS:ME:LINE1>	.	PASS	SVTYPE=INS:ME:LINE1;END=80241681;SVLEN=1275;AN=20618;AC=1;AF=4.850130953535746e-05;N_HET=1;N_HOMALT=0;N_HOMREF=10308;FREQ_HET=9.700261907071492e-05;FREQ_HOMALT=0.0;FREQ_HOMREF=0.9999029973809292;N_BI_GENOS=10309;North_Han_AN=2272;North_Han_AC=1;North_Han_
chr3	80276039	.	A	<INS:ME:LINE1>	.	PASS	SVTYPE=INS:ME:LINE1;END=80276039;SVLEN=0;AN=20618;AC=1;AF=4.850130953535746e-05;N_HET=1;N_HOMALT=0;N_HOMREF=10308;FREQ_HET=9.700261907071492e-05;FREQ_HOMALT=0.0;FREQ_HOMREF=0.9999029973809292;N_BI_GENOS=10309;North_Han_AN=2272;North_Han_AC=1;North_Han_AF=
chr3	80328564	.	A	<INS:ME:LINE1>	.	PASS	SVTYPE=INS:ME:LINE1;END=80328575;SVLEN=732;AN=20618;AC=1;AF=4.850130953535746e-05;N_HET=1;N_HOMALT=0;N_HOMREF=10308;FREQ_HET=9.700261907071492e-05;FREQ_HOMALT=0.0;FREQ_HOMREF=0.9999029973809292;N_BI_GENOS=10309;Central_Han_AN=2128;Central_Han_AC=1;Central
chr3	80509089	.	A	<INS:ME:LINE1>	.	PASS	SVTYPE=INS:ME:LINE1;END=80509090;SVLEN=590;AN=20618;AC=2;AF=9.700261907071492e-05;N_HET=2;N_HOMALT=0;N_HOMREF=10307;FREQ_HET=0.00019400523814142983;FREQ_HOMALT=0.0;FREQ_HOMREF=0.9998059947618586;N_BI_GENOS=10309;South_Han_AN=1496;South_Han_AC=1;South_Han_
chr3	80541012	.	C	<INS:ME:LINE1>	.	PASS	SVTYPE=INS:ME:LINE1;END=80541015;SVLEN=6000;AN=20618;AC=4941;AF=0.2396449704142011 9;N_HET=2925;N_HOMALT=1008;N_HOMREF=6376;FREQ_HET=0.2837326607818411;FREQ_HOMALT=0.09777864002328063;FREQ_HOMREF=0.6184886991948783;N_BI_GENOS=10309;South_Han_AN=1496;South_
chr3	80831066	.	A	<INS:ME:LINE1>	.	PASS	SVTYPE=INS:ME:LINE1;END=80831081;SVLEN=252;AN=20618;AC=27;AF=0.0013095353574546513;N_HET=27;N_HOMALT=0;N_HOMREF=10282;FREQ_HET=0.0026190707149093026;FREQ_HOMALT=0.0;FREQ_HOMREF=0.9973809292850907;N_BI_GENOS=10309;South_Han_AN=1496;South_Han_AC=1;South_Han
chr3	80850009	.	G	<INS:ME:LINE1>	.	PASS	SVTYPE=INS:ME:LINE1;END=80850024;SVLEN=2095;AN=20618;AC=1;AF=4.850130953535746e-05;N_HET=1;N_HOMALT=0;N_HOMREF=10308;FREQ_HET=9.700261907071492e-05;FREQ_HOMALT=0.0;FREQ_HOMREF=0.9999029973809292;N_BI_GENOS=10309;Miao_AN=482;Miao_AC=1;Miao_AF=0.00207468879
chr3	80978839	.	A	<INS:ME:LINE1>	.	PASS	SVTYPE=INS:ME:LINE1;END=80978854;SVLEN=166;AN=20618;AC=11;AF=0.000533514404888932;N_HET=11;N_HOMALT=0;N_HOMREF=10298;FREQ_HET=0.001067028809777864;FREQ_HOMALT=0.0;FREQ_HOMREF=0.9989329711902222;N_BI_GENOS=10309;Northwest_Han_AN=1296;Northwest_Han_AC=1;Nor
chr3	81626380	.	A	<INS:ME:LINE1>	.	PASS	SVTYPE=INS:ME:LINE1;END=81626395;SVLEN=314;AN=20618;AC=3;AF=0.00014550392860607236;N_HET=3;N_HOMALT=0;N_HOMREF=10306;FREQ_HET=0.0002910078572121447;FREQ_HOMALT=0.0;FREQ_HOMREF=0.9997089921427879;N_BI_GENOS=10309;East_Han_AN=6554;East_Han_AC=2;East_Han_AF=
chr3	81708541	.	G	<INS:ME:LINE1>	.	PASS	SVTYPE=INS:ME:LINE1;END=81708557;SVLEN=6018;AN=20618;AC=2;AF=9.700261907071492e-05;N_HET=2;N_HOMALT=0;N_HOMREF=10307;FREQ_HET=0.00019400523814142983;FREQ_HOMALT=0.0;FREQ_HOMREF=0.9998059947618586;N_BI_GENOS=10309;East_Han_AN=6554;East_Han_AC=2;East_Han_AF
chr3	81845423	.	A	<INS:ME:LINE1>	.	PASS	SVTYPE=INS:ME:LINE1;END=81845437;SVLEN=997;AN=20618;AC=1;AF=4.850130953535746e-05;N_HET=1;N_HOMALT=0;N_HOMREF=10308;FREQ_HET=9.700261907071492e-05;FREQ_HOMALT=0.0;FREQ_HOMREF=0.9999029973809292;N_BI_GENOS=10309;East_Han_AN=6554;East_Han_AC=1;East_Han_AF=0
chr3	81941841	.	T	<INS:ME:LINE1>	.	PASS	SVTYPE=INS:ME:LINE1;END=81941855;SVLEN=5999;AN=20618;AC=5938;AF=0.2880007760209526;N_HET=3686;N_HOMALT=1126;N_HOMREF=5497;FREQ_HET=0.35755165389465515;FREQ_HOMALT=0.10922494907362498;FREQ_HOMREF=0.5332233970317198;N_BI_GENOS=10309;South_Han_AN=1496;South_
chr3	82029905	.	A	<INS:ME:LINE1>	.	PASS	SVTYPE=INS:ME:LINE1;END=82029921;SVLEN=1084;AN=20618;AC=1;AF=4.850130953535746e-05;N_HET=1;N_HOMALT=0;N_HOMREF=10308;FREQ_HET=9.700261907071492e-05;FREQ_HOMALT=0.0;FREQ_HOMREF=0.9999029973809292;N_BI_GENOS=10309;East_Han_AN=6554;East_Han_AC=1;East_Han_AF=
chr3	82042878	.	G	<INS:ME:LINE1>	.	PASS	SVTYPE=INS:ME:LINE1;END=82042890;SVLEN=6022;AN=20618;AC=2;AF=9.700261907071492e-05;N_HET=2;N_HOMALT=0;N_HOMREF=10307;FREQ_HET=0.00019400523814142983;FREQ_HOMALT=0.0;FREQ_HOMREF=0.9998059947618586;N_BI_GENOS=10309;Central_Han_AN=2128;Central_Han_AC=2;Centr
chr3	82095702	.	G	<INS:ME:LINE1>	.	PASS	SVTYPE=INS:ME:LINE1;END=82095718;SVLEN=5999;AN=20618;AC=1354;AF=0.06567077311087399;N_HET=1158;N_HOMALT=98;N_HOMREF=9053;FREQ_HET=0.11232903288388786;FREQ_HOMALT=0.009506256668930062;FREQ_HOMREF=0.878164710447182;N_BI_GENOS=10309;South_Han_AN=1496;South_H
chr3	82315860	.	A	<INS:ME:LINE1>	.	PASS	SVTYPE=INS:ME:LINE1;END=82315877;SVLEN=6004;AN=20618;AC=1;AF=4.850130953535746e-05;N_HET=1;N_HOMALT=0;N_HOMREF=10308;FREQ_HET=9.700261907071492e-05;FREQ_HOMALT=0.0;FREQ_HOMREF=0.9999029973809292;N_BI_GENOS=10309;North_Han_AN=2272;North_Han_AC=1;North_Han_
chr3	82537672	.	G	<INS:ME:LINE1>	.	PASS	SVTYPE=INS:ME:LINE1;END=82537685;SVLEN=754;AN=20618;AC=1;AF=4.850130953535746e-05;N_HET=1;N_HOMALT=0;N_HOMREF=10308;FREQ_HET=9.700261907071492e-05;FREQ_HOMALT=0.0;FREQ_HOMREF=0.9999029973809292;N_BI_GENOS=10309;South_Han_AN=1496;South_Han_AC=1;South_Han_A
chr3	82660969	.	A	<INS:ME:LINE1>	.	PASS	SVTYPE=INS:ME:LINE1;END=82660986;SVLEN=1470;AN=20618;AC=865;AF=0.041953632748084196;N_HET=621;N_HOMALT=122;N_HOMREF=9566;FREQ_HET=0.06023862644291396;FREQ_HOMALT=0.011834319526627219;FREQ_HOMREF=0.9279270540304588;N_BI_GENOS=10309;South_Han_AN=1496;South_
chr3	82672793	.	T	<INS:ME:LINE1>	.	PASS	SVTYPE=INS:ME:LINE1;END=82672808;SVLEN=482;AN=20618;AC=1;AF=4.850130953535746e-05;N_HET=1;N_HOMALT=0;N_HOMREF=10308;FREQ_HET=9.700261907071492e-05;FREQ_HOMALT=0.0;FREQ_HOMREF=0.9999029973809292;N_BI_GENOS=10309;Central_Han_AN=2128;Central_Han_AC=1;Central
chr3	82734267	.	A	<INS:ME:LINE1>	.	PASS	SVTYPE=INS:ME:LINE1;END=82734283;SVLEN=6003;AN=20618;AC=2;AF=9.700261907071492e-05;N_HET=2;N_HOMALT=0;N_HOMREF=10307;FREQ_HET=0.00019400523814142983;FREQ_HOMALT=0.0;FREQ_HOMREF=0.9998059947618586;N_BI_GENOS=10309;East_Han_AN=6554;East_Han_AC=1;East_Han_AF
chr3	82998994	.	A	<INS:ME:LINE1>	.	PASS	SVTYPE=INS:ME:LINE1;END=82999011;SVLEN=1674;AN=20618;AC=1;AF=4.850130953535746e-05;N_HET=1;N_HOMALT=0;N_HOMREF=10308;FREQ_HET=9.700261907071492e-05;FREQ_HOMALT=0.0;FREQ_HOMREF=0.9999029973809292;N_BI_GENOS=10309;North_Han_AN=2272;North_Han_AC=1;North_Han_
chr3	83099677	.	A	<INS:ME:LINE1>	.	PASS	SVTYPE=INS:ME:LINE1;END=83099678;SVLEN=76;AN=20618;AC=2;AF=9.700261907071492e-05;N_HET=0;N_HOMALT=1;N_HOMREF=10308;FREQ_HET=0.0;FREQ_HOMALT=9.700261907071492e-05;FREQ_HOMREF=0.9999029973809292;N_BI_GENOS=10309;Lingnan_Han_AN=1888;Lingnan_Han_AC=2;Lingnan_
chr3	83388332	.	G	<INS:ME:LINE1>	.	PASS	SVTYPE=INS:ME:LINE1;END=83388338;SVLEN=670;AN=20618;AC=1;AF=4.850130953535746e-05;N_HET=1;N_HOMALT=0;N_HOMREF=10308;FREQ_HET=9.700261907071492e-05;FREQ_HOMALT=0.0;FREQ_HOMREF=0.9999029973809292;N_BI_GENOS=10309;Southeast_Han_AN=1908;Southeast_Han_AC=1;Sou
chr3	83398712	.	C	<INS:ME:LINE1>	.	PASS	SVTYPE=INS:ME:LINE1;END=83398761;SVLEN=1092;AN=20618;AC=1;AF=4.850130953535746e-05;N_HET=1;N_HOMALT=0;N_HOMREF=10308;FREQ_HET=9.700261907071492e-05;FREQ_HOMALT=0.0;FREQ_HOMREF=0.9999029973809292;N_BI_GENOS=10309;East_Han_AN=6554;East_Han_AC=1;East_Han_AF=
chr3	83634388	.	T	<INS:ME:LINE1>	.	PASS	SVTYPE=INS:ME:LINE1;END=83634391;SVLEN=953;AN=20618;AC=1;AF=4.850130953535746e-05;N_HET=1;N_HOMALT=0;N_HOMREF=10308;FREQ_HET=9.700261907071492e-05;FREQ_HOMALT=0.0;FREQ_HOMREF=0.9999029973809292;N_BI_GENOS=10309;North_Han_AN=2272;North_Han_AC=1;North_Han_A
chr3	83710418	.	A	<INS:ME:LINE1>	.	PASS	SVTYPE=INS:ME:LINE1;END=83710436;SVLEN=1004;AN=20618;AC=1;AF=4.850130953535746e-05;N_HET=1;N_HOMALT=0;N_HOMREF=10308;FREQ_HET=9.700261907071492e-05;FREQ_HOMALT=0.0;FREQ_HOMREF=0.9999029973809292;N_BI_GENOS=10309;North_Han_AN=2272;North_Han_AC=1;North_Han_
chr3	83734015	.	A	<INS:ME:LINE1>	.	PASS	SVTYPE=INS:ME:LINE1;END=83734027;SVLEN=1695;AN=20618;AC=1;AF=4.850130953535746e-05;N_HET=1;N_HOMALT=0;N_HOMREF=10308;FREQ_HET=9.700261907071492e-05;FREQ_HOMALT=0.0;FREQ_HOMREF=0.9999029973809292;N_BI_GENOS=10309;East_Han_AN=6554;East_Han_AC=1;East_Han_AF=
chr3	83990281	.	C	<INS:ME:LINE1>	.	PASS	SVTYPE=INS:ME:LINE1;END=83990296;SVLEN=1327;AN=20618;AC=150;AF=0.007275196430303618 5;N_HET=140;N_HOMALT=5;N_HOMREF=10164;FREQ_HET=0.013580366669900087;FREQ_HOMALT=0.0004850130953535745 5;FREQ_HOMREF=0.9859346202347463;N_BI_GENOS=10309;South_Han_AN=1496;Sou
chr3	84035380	.	C	<INS:ME:LINE1>	.	PASS	SVTYPE=INS:ME:LINE1;END=84035380;SVLEN=504;AN=20618;AC=1;AF=4.850130953535746e-05;N_HET=1;N_HOMALT=0;N_HOMREF=10308;FREQ_HET=9.700261907071492e-05;FREQ_HOMALT=0.0;FREQ_HOMREF=0.9999029973809292;N_BI_GENOS=10309;East_Han_AN=6554;East_Han_AC=1;East_Han_AF=0
chr3	84062444	.	A	<INS:ME:LINE1>	.	PASS	SVTYPE=INS:ME:LINE1;END=84062459;SVLEN=1643;AN=20618;AC=3;AF=0.00014550392860607236;N_HET=3;N_HOMALT=0;N_HOMREF=10306;FREQ_HET=0.0002910078572121447;FREQ_HOMALT=0.0;FREQ_HOMREF=0.9997089921427879;N_BI_GENOS=10309;East_Han_AN=6554;East_Han_AC=3;East_Han_AF
chr3	84176284	.	G	<INS:ME:LINE1>	.	PASS	SVTYPE=INS:ME:LINE1;END=84176284;SVLEN=1459;AN=20618;AC=1;AF=4.850130953535746e-05;N_HET=1;N_HOMALT=0;N_HOMREF=10308;FREQ_HET=9.700261907071492e-05;FREQ_HOMALT=0.0;FREQ_HOMREF=0.9999029973809292;N_BI_GENOS=10309;East_Han_AN=6554;East_Han_AC=1;East_Han_AF=
chr3	84184015	.	T	<INS:ME:LINE1>	.	PASS	SVTYPE=INS:ME:LINE1;END=84184034;SVLEN=360;AN=20618;AC=1;AF=4.850130953535746e-05;N_HET=1;N_HOMALT=0;N_HOMREF=10308;FREQ_HET=9.700261907071492e-05;FREQ_HOMALT=0.0;FREQ_HOMREF=0.9999029973809292;N_BI_GENOS=10309;Hui_AN=444;Hui_AC=1;Hui_AF=0.002252252252252
chr3	84381184	.	A	<INS:ME:LINE1>	.	PASS	SVTYPE=INS:ME:LINE1;END=84381184;SVLEN=2616;AN=20618;AC=1;AF=4.850130953535746e-05;N_HET=1;N_HOMALT=0;N_HOMREF=10308;FREQ_HET=9.700261907071492e-05;FREQ_HOMALT=0.0;FREQ_HOMREF=0.9999029973809292;N_BI_GENOS=10309;Tibetan_AN=414;Tibetan_AC=1;Tibetan_AF=0.00
chr3	84384233	.	T	<INS:ME:LINE1>	.	PASS	SVTYPE=INS:ME:LINE1;END=84384244;SVLEN=5998;AN=20618;AC=2506;AF=0.12154428169560579;N_HET=1820;N_HOMALT=343;N_HOMREF=8146;FREQ_HET=0.17654476670870115;FREQ_HOMALT=0.03327189834125521;FREQ_HOMREF=0.7901833349500437;N_BI_GENOS=10309;South_Han_AN=1496;South_
chr3	84423453	.	G	<INS:ME:LINE1>	.	PASS	SVTYPE=INS:ME:LINE1;END=84423453;SVLEN=761;AN=20618;AC=1;AF=4.850130953535746e-05;N_HET=1;N_HOMALT=0;N_HOMREF=10308;FREQ_HET=9.700261907071492e-05;FREQ_HOMALT=0.0;FREQ_HOMREF=0.9999029973809292;N_BI_GENOS=10309;North_Han_AN=2272;North_Han_AC=1;North_Han_A
chr3	84544122	.	A	<INS:ME:LINE1>	.	PASS	SVTYPE=INS:ME:LINE1;END=84544137;SVLEN=865;AN=20618;AC=6816;AF=0.3305849257929964;N_HET=3974;N_HOMALT=1421;N_HOMREF=4914;FREQ_HET=0.38548840818702107;FREQ_HOMALT=0.13784072169948589;FREQ_HOMREF=0.4766708701134931;N_BI_GENOS=10309;South_Han_AN=1496;South_H
chr3	84566172	.	C	<INS:ME:LINE1>	.	PASS	SVTYPE=INS:ME:LINE1;END=84566172;SVLEN=10;AN=20618;AC=1;AF=4.850130953535746e-05;N_HET=1;N_HOMALT=0;N_HOMREF=10308;FREQ_HET=9.700261907071492e-05;FREQ_HOMALT=0.0;FREQ_HOMREF=0.9999029973809292;N_BI_GENOS=10309;Southeast_Han_AN=1908;Southeast_Han_AC=1;Sout
chr3	84743425	.	A	<INS:ME:LINE1>	.	PASS	SVTYPE=INS:ME:LINE1;END=84743441;SVLEN=975;AN=20618;AC=1;AF=4.850130953535746e-05;N_HET=1;N_HOMALT=0;N_HOMREF=10308;FREQ_HET=9.700261907071492e-05;FREQ_HOMALT=0.0;FREQ_HOMREF=0.9999029973809292;N_BI_GENOS=10309;Northwest_Han_AN=1296;Northwest_Han_AC=1;Nor
chr3	84747904	.	A	<INS:ME:LINE1>	.	PASS	SVTYPE=INS:ME:LINE1;END=84747905;SVLEN=763;AN=20618;AC=1;AF=4.850130953535746e-05;N_HET=1;N_HOMALT=0;N_HOMREF=10308;FREQ_HET=9.700261907071492e-05;FREQ_HOMALT=0.0;FREQ_HOMREF=0.9999029973809292;N_BI_GENOS=10309;East_Han_AN=6554;East_Han_AC=1;East_Han_AF=0
chr3	84876333	.	T	<INS:ME:LINE1>	.	PASS	SVTYPE=INS:ME:LINE1;END=84876349;SVLEN=623;AN=20618;AC=1;AF=4.850130953535746e-05;N_HET=1;N_HOMALT=0;N_HOMREF=10308;FREQ_HET=9.700261907071492e-05;FREQ_HOMALT=0.0;FREQ_HOMREF=0.9999029973809292;N_BI_GENOS=10309;East_Han_AN=6554;East_Han_AC=1;East_Han_AF=0
chr3	85237035	.	A	<INS:ME:LINE1>	.	PASS	SVTYPE=INS:ME:LINE1;END=85237049;SVLEN=2822;AN=20618;AC=1;AF=4.850130953535746e-05;N_HET=1;N_HOMALT=0;N_HOMREF=10308;FREQ_HET=9.700261907071492e-05;FREQ_HOMALT=0.0;FREQ_HOMREF=0.9999029973809292;N_BI_GENOS=10309;Southeast_Han_AN=1908;Southeast_Han_AC=1;So
chr3	85299159	.	T	<INS:ME:LINE1>	.	PASS	SVTYPE=INS:ME:LINE1;END=85299174;SVLEN=1708;AN=20618;AC=1;AF=4.850130953535746e-05;N_HET=1;N_HOMALT=0;N_HOMREF=10308;FREQ_HET=9.700261907071492e-05;FREQ_HOMALT=0.0;FREQ_HOMREF=0.9999029973809292;N_BI_GENOS=10309;Lingnan_Han_AN=1888;Lingnan_Han_AC=1;Lingna
chr3	85403303	.	A	<INS:ME:LINE1>	.	PASS	SVTYPE=INS:ME:LINE1;END=85403317;SVLEN=127;AN=20618;AC=2;AF=9.700261907071492e-05;N_HET=2;N_HOMALT=0;N_HOMREF=10307;FREQ_HET=0.00019400523814142983;FREQ_HOMALT=0.0;FREQ_HOMREF=0.9998059947618586;N_BI_GENOS=10309;South_Han_AN=1496;South_Han_AC=1;South_Han_
chr3	85415270	.	G	<INS:ME:LINE1>	.	PASS	SVTYPE=INS:ME:LINE1;END=85415274;SVLEN=3816;AN=20618;AC=35;AF=0.0016975458337375108;N_HET=35;N_HOMALT=0;N_HOMREF=10274;FREQ_HET=0.0033950916674750217;FREQ_HOMALT=0.0;FREQ_HOMREF=0.996604908332525;N_BI_GENOS=10309;South_Han_AN=1496;South_Han_AC=9;South_Han
chr3	85418439	.	A	<INS:ME:LINE1>	.	PASS	SVTYPE=INS:ME:LINE1;END=85418456;SVLEN=3111;AN=20618;AC=1;AF=4.850130953535746e-05;N_HET=1;N_HOMALT=0;N_HOMREF=10308;FREQ_HET=9.700261907071492e-05;FREQ_HOMALT=0.0;FREQ_HOMREF=0.9999029973809292;N_BI_GENOS=10309;Northwest_Han_AN=1296;Northwest_Han_AC=1;No
chr3	85502377	.	T	<INS:ME:LINE1>	.	PASS	SVTYPE=INS:ME:LINE1;END=85502388;SVLEN=914;AN=20618;AC=2;AF=9.700261907071492e-05;N_HET=2;N_HOMALT=0;N_HOMREF=10307;FREQ_HET=0.00019400523814142983;FREQ_HOMALT=0.0;FREQ_HOMREF=0.9998059947618586;N_BI_GENOS=10309;Central_Han_AN=2128;Central_Han_AC=1;Centra
chr3	85505111	.	G	<INS:ME:LINE1>	.	PASS	SVTYPE=INS:ME:LINE1;END=85505111;SVLEN=0;AN=20618;AC=1;AF=4.850130953535746e-05;N_HET=1;N_HOMALT=0;N_HOMREF=10308;FREQ_HET=9.700261907071492e-05;FREQ_HOMALT=0.0;FREQ_HOMREF=0.9999029973809292;N_BI_GENOS=10309;East_Han_AN=6554;East_Han_AC=1;East_Han_AF=0.0
chr3	85527399	.	A	<INS:ME:LINE1>	.	PASS	SVTYPE=INS:ME:LINE1;END=85527418;SVLEN=5998;AN=20618;AC=8367;AF=0.4058104568823358;N_HET=3041;N_HOMALT=2663;N_HOMREF=4605;FREQ_HET=0.29498496459404405;FREQ_HOMALT=0.2583179745853138;FREQ_HOMREF=0.44669706082064214;N_BI_GENOS=10309;South_Han_AN=1496;South_
chr3	85639738	.	A	<INS:ME:LINE1>	.	PASS	SVTYPE=INS:ME:LINE1;END=85639754;SVLEN=771;AN=20618;AC=1;AF=4.850130953535746e-05;N_HET=1;N_HOMALT=0;N_HOMREF=10308;FREQ_HET=9.700261907071492e-05;FREQ_HOMALT=0.0;FREQ_HOMREF=0.9999029973809292;N_BI_GENOS=10309;Lingnan_Han_AN=1888;Lingnan_Han_AC=1;Lingnan
chr3	85657376	.	C	<INS:ME:LINE1>	.	PASS	SVTYPE=INS:ME:LINE1;END=85657379;SVLEN=867;AN=20618;AC=2;AF=9.700261907071492e-05;N_HET=2;N_HOMALT=0;N_HOMREF=10307;FREQ_HET=0.00019400523814142983;FREQ_HOMALT=0.0;FREQ_HOMREF=0.9998059947618586;N_BI_GENOS=10309;Central_Han_AN=2128;Central_Han_AC=1;Centra
chr3	85964963	.	G	<INS:ME:LINE1>	.	PASS	SVTYPE=INS:ME:LINE1;END=85964980;SVLEN=895;AN=20618;AC=16;AF=0.0007760209525657193;N_HET=16;N_HOMALT=0;N_HOMREF=10293;FREQ_HET=0.0015520419051314386;FREQ_HOMALT=0.0;FREQ_HOMREF=0.9984479580948685;N_BI_GENOS=10309;South_Han_AN=1496;South_Han_AC=6;South_Han

chr3	85977976	.	T	<INS:ME:LINE1>	.	PASS	SVTYPE=INS:ME:LINE1;END=85977991;SVLEN=5997;AN=20618;AC=1;AF=4.850130953535746e-05;N_HET=1;N_HOMALT=0;N_HOMREF=10308;FREQ_HET=9.700261907071492e-05;FREQ_HOMALT=0.0;FREQ_HOMREF=0.9999029973809292;N_BI_GENOS=10309;Northwest_Han_AN=1296;Northwest_Han_AC=1;No
chr3	86071788	.	A	<INS:ME:LINE1>	.	PASS	SVTYPE=INS:ME:LINE1;END=86071804;SVLEN=6003;AN=20618;AC=1;AF=4.850130953535746e-05;N_HET=1;N_HOMALT=0;N_HOMREF=10308;FREQ_HET=9.700261907071492e-05;FREQ_HOMALT=0.0;FREQ_HOMREF=0.9999029973809292;N_BI_GENOS=10309;North_Han_AN=2272;North_Han_AC=1;North_Han_
chr3	86152060	.	A	<INS:ME:LINE1>	.	PASS	SVTYPE=INS:ME:LINE1;END=86152070;SVLEN=495;AN=20618;AC=1;AF=4.850130953535746e-05;N_HET=1;N_HOMALT=0;N_HOMREF=10308;FREQ_HET=9.700261907071492e-05;FREQ_HOMALT=0.0;FREQ_HOMREF=0.9999029973809292;N_BI_GENOS=10309;Central_Han_AN=2128;Central_Han_AC=1;Central
chr3	86215788	.	T	<INS:ME:LINE1>	.	PASS	SVTYPE=INS:ME:LINE1;END=86215796;SVLEN=760;AN=20618;AC=1011;AF=0.049034823940246386;N_HET=965;N_HOMALT=23;N_HOMREF=9321;FREQ_HET=0.09360752740323988;FREQ_HOMALT=0.002231060238626443;FREQ_HOMREF=0.9041614123581336;N_BI_GENOS=10309;South_Han_AN=1496;South_H
chr3	86926749	.	A	<INS:ME:LINE1>	.	PASS	SVTYPE=INS:ME:LINE1;END=86926751;SVLEN=832;AN=20618;AC=9;AF=0.0004365117858182171;N_HET=9;N_HOMALT=0;N_HOMREF=10300;FREQ_HET=0.0008730235716364342;FREQ_HOMALT=0.0;FREQ_HOMREF=0.9991269764283636;N_BI_GENOS=10309;Northwest_Han_AN=1296;Northwest_Han_AC=2;Nor
chr3	87164290	.	A	<INS:ME:LINE1>	.	PASS	SVTYPE=INS:ME:LINE1;END=87164305;SVLEN=2157;AN=20618;AC=1;AF=4.850130953535746e-05;N_HET=1;N_HOMALT=0;N_HOMREF=10308;FREQ_HET=9.700261907071492e-05;FREQ_HOMALT=0.0;FREQ_HOMREF=0.9999029973809292;N_BI_GENOS=10309;Northwest_Han_AN=1296;Northwest_Han_AC=1;No
chr3	87190131	.	G	<INS:ME:LINE1>	.	PASS	SVTYPE=INS:ME:LINE1;END=87190146;SVLEN=1318;AN=20618;AC=3;AF=0.00014550392860607236;N_HET=1;N_HOMALT=1;N_HOMREF=10307;FREQ_HET=9.700261907071492e-05;FREQ_HOMALT=9.700261907071492e-05;FREQ_HOMREF=0.9998059947618586;N_BI_GENOS=10309;Lingnan_Han_AN=1888;Ling
chr3	87440910	.	A	<INS:ME:LINE1>	.	PASS	SVTYPE=INS:ME:LINE1;END=87440918;SVLEN=450;AN=20618;AC=1;AF=4.850130953535746e-05;N_HET=1;N_HOMALT=0;N_HOMREF=10308;FREQ_HET=9.700261907071492e-05;FREQ_HOMALT=0.0;FREQ_HOMREF=0.9999029973809292;N_BI_GENOS=10309;Central_Han_AN=2128;Central_Han_AC=1;Central
chr3	87543889	.	T	<INS:ME:LINE1>	.	PASS	SVTYPE=INS:ME:LINE1;END=87543889;SVLEN=942;AN=20618;AC=2;AF=9.700261907071492e-05;N_HET=2;N_HOMALT=0;N_HOMREF=10307;FREQ_HET=0.00019400523814142983;FREQ_HOMALT=0.0;FREQ_HOMREF=0.9998059947618586;N_BI_GENOS=10309;Southeast_Han_AN=1908;Southeast_Han_AC=1;So
chr3	87730613	.	G	<INS:ME:LINE1>	.	PASS	SVTYPE=INS:ME:LINE1;END=87730630;SVLEN=109;AN=20618;AC=2;AF=9.700261907071492e-05;N_HET=2;N_HOMALT=0;N_HOMREF=10307;FREQ_HET=0.00019400523814142983;FREQ_HOMALT=0.0;FREQ_HOMREF=0.9998059947618586;N_BI_GENOS=10309;East_Han_AN=6554;East_Han_AC=2;East_Han_AF=
chr3	87768907	.	C	<INS:ME:LINE1>	.	PASS	SVTYPE=INS:ME:LINE1;END=87768907;SVLEN=839;AN=20618;AC=1;AF=4.850130953535746e-05;N_HET=1;N_HOMALT=0;N_HOMREF=10308;FREQ_HET=9.700261907071492e-05;FREQ_HOMALT=0.0;FREQ_HOMREF=0.9999029973809292;N_BI_GENOS=10309;East_Han_AN=6554;East_Han_AC=1;East_Han_AF=0
chr3	87947356	.	G	<INS:ME:LINE1>	.	PASS	SVTYPE=INS:ME:LINE1;END=87947371;SVLEN=6016;AN=20618;AC=1;AF=4.850130953535746e-05;N_HET=1;N_HOMALT=0;N_HOMREF=10308;FREQ_HET=9.700261907071492e-05;FREQ_HOMALT=0.0;FREQ_HOMREF=0.9999029973809292;N_BI_GENOS=10309;Lingnan_Han_AN=1888;Lingnan_Han_AC=1;Lingna
chr3	88309184	.	G	<INS:ME:LINE1>	.	PASS	SVTYPE=INS:ME:LINE1;END=88309192;SVLEN=802;AN=20618;AC=1;AF=4.850130953535746e-05;N_HET=1;N_HOMALT=0;N_HOMREF=10308;FREQ_HET=9.700261907071492e-05;FREQ_HOMALT=0.0;FREQ_HOMREF=0.9999029973809292;N_BI_GENOS=10309;Lingnan_Han_AN=1888;Lingnan_Han_AC=1;Lingnan
chr3	88418283	.	A	<INS:ME:LINE1>	.	PASS	SVTYPE=INS:ME:LINE1;END=88418300;SVLEN=1452;AN=20618;AC=100;AF=0.004850130953535745;N_HET=100;N_HOMALT=0;N_HOMREF=10209;FREQ_HET=0.00970026190707149;FREQ_HOMALT=0.0;FREQ_HOMREF=0.9902997380929285;N_BI_GENOS=10309;South_Han_AN=1496;South_Han_AC=14;South_Ha
chr3	88467490	.	G	<INS:ME:LINE1>	.	PASS	SVTYPE=INS:ME:LINE1;END=88467490;SVLEN=1345;AN=20618;AC=5;AF=0.00024250654767678728;N_HET=5;N_HOMALT=0;N_HOMREF=10304;FREQ_HET=0.0004850130953535455;FREQ_HOMALT=0.0;FREQ_HOMREF=0.9995149869046465;N_BI_GENOS=10309;South_Han_AN=1496;South_Han_AC=3;South_Ha
chr3	88481745	.	T	<INS:ME:LINE1>	.	PASS	SVTYPE=INS:ME:LINE1;END=88481760;SVLEN=4404;AN=20618;AC=1;AF=4.850130953535746e-05;N_HET=1;N_HOMALT=0;N_HOMREF=10308;FREQ_HET=9.700261907071492e-05;FREQ_HOMALT=0.0;FREQ_HOMREF=0.9999029973809292;N_BI_GENOS=10309;Central_Han_AN=2128;Central_Han_AC=1;Centra
chr3	88517708	.	T	<INS:ME:LINE1>	.	PASS	SVTYPE=INS:ME:LINE1;END=88517708;SVLEN=95;AN=20618;AC=1;AF=4.850130953535746e-05;N_HET=1;N_HOMALT=0;N_HOMREF=10308;FREQ_HET=9.700261907071492e-05;FREQ_HOMALT=0.0;FREQ_HOMREF=0.9999029973809292;N_BI_GENOS=10309;East_Han_AN=6554;East_Han_AC=1;East_Han_AF=0.
chr3	88631105	.	T	<INS:ME:LINE1>	.	PASS	SVTYPE=INS:ME:LINE1;END=88631105;SVLEN=677;AN=20618;AC=1;AF=4.850130953535746e-05;N_HET=1;N_HOMALT=0;N_HOMREF=10308;FREQ_HET=9.700261907071492e-05;FREQ_HOMALT=0.0;FREQ_HOMREF=0.9999029973809292;N_BI_GENOS=10309;Central_Han_AN=2128;Central_Han_AC=1;Central
chr3	88651654	.	G	<INS:ME:LINE1>	.	PASS	SVTYPE=INS:ME:LINE1;END=88651668;SVLEN=912;AN=20618;AC=1;AF=4.850130953535746e-05;N_HET=1;N_HOMALT=0;N_HOMREF=10308;FREQ_HET=9.700261907071492e-05;FREQ_HOMALT=0.0;FREQ_HOMREF=0.9999029973809292;N_BI_GENOS=10309;North_Han_AN=2272;North_Han_AC=1;North_Han_A
chr3	88652382	.	A	<INS:ME:LINE1>	.	PASS	SVTYPE=INS:ME:LINE1;END=88652395;SVLEN=1420;AN=20618;AC=3;AF=0.00014550392860607236;N_HET=3;N_HOMALT=0;N_HOMREF=10306;FREQ_HET=0.0002910078572121447;FREQ_HOMALT=0.0;FREQ_HOMREF=0.9997089921427879;N_BI_GENOS=10309;South_Han_AN=1496;South_Han_AC=1;South_Han
chr3	88666224	.	A	<INS:ME:LINE1>	.	PASS	SVTYPE=INS:ME:LINE1;END=88666224;SVLEN=2862;AN=20618;AC=1;AF=4.850130953535746e-05;N_HET=1;N_HOMALT=0;N_HOMREF=10308;FREQ_HET=9.700261907071492e-05;FREQ_HOMALT=0.0;FREQ_HOMREF=0.9999029973809292;N_BI_GENOS=10309;North_Han_AN=2272;North_Han_AC=1;North_Han_
chr3	88735308	.	A	<INS:ME:LINE1>	.	PASS	SVTYPE=INS:ME:LINE1;END=88735317;SVLEN=645;AN=20618;AC=1;AF=4.850130953535746e-05;N_HET=1;N_HOMALT=0;N_HOMREF=10308;FREQ_HET=9.700261907071492e-05;FREQ_HOMALT=0.0;FREQ_HOMREF=0.9999029973809292;N_BI_GENOS=10309;South_Han_AN=1496;South_Han_AC=1;South_Han_A
chr3	88809621	.	A	<INS:ME:LINE1>	.	PASS	SVTYPE=INS:ME:LINE1;END=88809637;SVLEN=597;AN=20618;AC=6;AF=0.0002910078572121447;N_HET=6;N_HOMALT=0;N_HOMREF=10303;FREQ_HET=0.0005820157144242894;FREQ_HOMALT=0.0;FREQ_HOMREF=0.9994179842855757;N_BI_GENOS=10309;South_Han_AN=1496;South_Han_AC=1;South_Han_A
chr3	88884289	.	T	<INS:ME:LINE1>	.	PASS	SVTYPE=INS:ME:LINE1;END=88884305;SVLEN=414;AN=20618;AC=1;AF=4.850130953535746e-05;N_HET=1;N_HOMALT=0;N_HOMREF=10308;FREQ_HET=9.700261907071492e-05;FREQ_HOMALT=0.0;FREQ_HOMREF=0.9999029973809292;N_BI_GENOS=10309;Central_Han_AN=2128;Central_Han_AC=1;Central
chr3	88973180	.	A	<INS:ME:LINE1>	.	PASS	SVTYPE=INS:ME:LINE1;END=88973196;SVLEN=1761;AN=20618;AC=8508;AF=0.41264914152682125;N_HET=3530;N_HOMALT=2489;N_HOMREF=4290;FREQ_HET=0.34241924531962364;FREQ_HOMALT=0.2414395188670094;FREQ_HOMREF=0.416141235813367;N_BI_GENOS=10309;South_Han_AN=1496;South_H
chr3	89032428	.	T	<INS:ME:LINE1>	.	PASS	SVTYPE=INS:ME:LINE1;END=89032435;SVLEN=3075;AN=20618;AC=2;AF=9.700261907071492e-05;N_HET=0;N_HOMALT=1;N_HOMREF=10308;FREQ_HET=0.0;FREQ_HOMALT=9.700261907071492e-05;FREQ_HOMREF=0.9999029973809292;N_BI_GENOS=10309;Lingnan_Han_AN=1888;Lingnan_Han_AC=2;Lingna
chr3	89344873	.	T	<INS:ME:LINE1>	.	PASS	SVTYPE=INS:ME:LINE1;END=89344873;SVLEN=379;AN=20618;AC=1;AF=4.850130953535746e-05;N_HET=1;N_HOMALT=0;N_HOMREF=10308;FREQ_HET=9.700261907071492e-05;FREQ_HOMALT=0.0;FREQ_HOMREF=0.9999029973809292;N_BI_GENOS=10309;North_Han_AN=2272;North_Han_AC=1;North_Han_A
chr3	89594300	.	C	<INS:ME:LINE1>	.	PASS	SVTYPE=INS:ME:LINE1;END=89594312;SVLEN=1853;AN=20618;AC=3;AF=0.00014550392860607236;N_HET=3;N_HOMALT=0;N_HOMREF=10306;FREQ_HET=0.0002910078572121447;FREQ_HOMALT=0.0;FREQ_HOMREF=0.9997089921427879;N_BI_GENOS=10309;North_Han_AN=2272;North_Han_AC=1;North_Han
chr3	89867626	.	A	<INS:ME:LINE1>	.	PASS	SVTYPE=INS:ME:LINE1;END=89867626;SVLEN=1085;AN=20618;AC=1;AF=4.850130953535746e-05;N_HET=1;N_HOMALT=0;N_HOMREF=10308;FREQ_HET=9.700261907071492e-05;FREQ_HOMALT=0.0;FREQ_HOMREF=0.9999029973809292;N_BI_GENOS=10309;East_Han_AN=6554;East_Han_AC=1;East_Han_AF=
chr3	89868216	.	A	<INS:ME:LINE1>	.	PASS	SVTYPE=INS:ME:LINE1;END=89868232;SVLEN=1220;AN=20618;AC=2;AF=9.700261907071492e-05;N_HET=0;N_HOMALT=1;N_HOMREF=10308;FREQ_HET=0.0;FREQ_HOMALT=9.700261907071492e-05;FREQ_HOMREF=0.9999029973809292;N_BI_GENOS=10309;East_Han_AN=6554;East_Han_AC=2;East_Han_AF=
chr3	89976372	.	T	<INS:ME:LINE1>	.	PASS	SVTYPE=INS:ME:LINE1;END=89976388;SVLEN=5999;AN=20618;AC=1;AF=4.850130953535746e-05;N_HET=1;N_HOMALT=0;N_HOMREF=10308;FREQ_HET=9.700261907071492e-05;FREQ_HOMALT=0.0;FREQ_HOMREF=0.9999029973809292;N_BI_GENOS=10309;Southeast_Han_AN=1908;Southeast_Han_AC=1;So
chr3	90217055	.	T	<INS:ME:LINE1>	.	PASS	SVTYPE=INS:ME:LINE1;END=90217071;SVLEN=1847;AN=20618;AC=1;AF=4.850130953535746e-05;N_HET=1;N_HOMALT=0;N_HOMREF=10308;FREQ_HET=9.700261907071492e-05;FREQ_HOMALT=0.0;FREQ_HOMREF=0.9999029973809292;N_BI_GENOS=10309;South_Han_AN=1496;South_Han_AC=1;South_Han_
chr3	90449914	.	C	<INS:ME:LINE1>	.	PASS	SVTYPE=INS:ME:LINE1;END=90449926;SVLEN=383;AN=20618;AC=5;AF=0.00024250654767678728;N_HET=5;N_HOMALT=0;N_HOMREF=10304;FREQ_HET=0.0004850130953535455;FREQ_HOMALT=0.0;FREQ_HOMREF=0.9995149869046465;N_BI_GENOS=10309;South_Han_AN=1496;South_Han_AC=3;South_Han
chr3	90479383	.	A	<INS:ME:LINE1>	.	PASS	SVTYPE=INS:ME:LINE1;END=90479391;SVLEN=1278;AN=20618;AC=8737;AF=0.42375594141041806;N_HET=1405;N_HOMALT=3666;N_HOMREF=5238;FREQ_HET=0.13628867979435444;FREQ_HOMALT=0.35561160151324084;FREQ_HOMREF=0.5080997186924047;N_BI_GENOS=10309;South_Han_AN=1496;South
chr3	90480013	.	C	<INS:ME:LINE1>	.	PASS	SVTYPE=INS:ME:LINE1;END=90480027;SVLEN=2271;AN=20618;AC=1;AF=4.850130953535746e-05;N_HET=1;N_HOMALT=0;N_HOMREF=10308;FREQ_HET=9.700261907071492e-05;FREQ_HOMALT=0.0;FREQ_HOMREF=0.9999029973809292;N_BI_GENOS=10309;South_Han_AN=1496;South_Han_AC=1;South_Han_
chr3	90495519	.	T	<INS:ME:LINE1>	.	PASS	SVTYPE=INS:ME:LINE1;END=90495535;SVLEN=1099;AN=20618;AC=1;AF=4.850130953535746e-05;N_HET=1;N_HOMALT=0;N_HOMREF=10308;FREQ_HET=9.700261907071492e-05;FREQ_HOMALT=0.0;FREQ_HOMREF=0.9999029973809292;N_BI_GENOS=10309;South_Han_AN=1496;South_Han_AC=1;South_Han_
chr3	90705638	.	A	<INS:ME:LINE1>	.	PASS	SVTYPE=INS:ME:LINE1;END=90705642;SVLEN=3099;AN=20618;AC=12;AF=0.0005820157144242894;N_HET=8;N_HOMALT=2;N_HOMREF=10299;FREQ_HET=0.0007760209525657193;FREQ_HOMALT=0.00019400523814142983;FREQ_HOMREF=0.9990299738092928;N_BI_GENOS=10309;East_Han_AN=6554;East_H
chr3	91525337	.	C	<INS:ME:LINE1>	.	PASS	SVTYPE=INS:ME:LINE1;END=91525352;SVLEN=1041;AN=20618;AC=1;AF=4.850130953535746e-05;N_HET=1;N_HOMALT=0;N_HOMREF=10308;FREQ_HET=9.700261907071492e-05;FREQ_HOMALT=0.0;FREQ_HOMREF=0.9999029973809292;N_BI_GENOS=10309;Zhuang_AN=444;Zhuang_AC=1;Zhuang_AF=0.00225
chr3	91532022	.	A	<INS:ME:LINE1>	.	PASS	SVTYPE=INS:ME:LINE1;END=91532033;SVLEN=5948;AN=20618;AC=1;AF=4.850130953535746e-05;N_HET=1;N_HOMALT=0;N_HOMREF=10308;FREQ_HET=9.700261907071492e-05;FREQ_HOMALT=0.0;FREQ_HOMREF=0.9999029973809292;N_BI_GENOS=10309;East_Han_AN=6554;East_Han_AC=1;East_Han_AF=
chr3	91532537	.	A	<INS:ME:LINE1>	.	PASS	SVTYPE=INS:ME:LINE1;END=91532555;SVLEN=6000;AN=20618;AC=1753;AF=0.08502279561548162;N_HET=1227;N_HOMALT=263;N_HOMREF=8819;FREQ_HET=0.1190222135997672;FREQ_HOMALT=0.02551168881559802;FREQ_HOMREF=0.8554660975846348;N_BI_GENOS=10309;South_Han_AN=1496;South_H
chr3	93719397	.	T	<INS:ME:LINE1>	.	PASS	SVTYPE=INS:ME:LINE1;END=93719411;SVLEN=569;AN=20618;AC=1;AF=4.850130953535746e-05;N_HET=1;N_HOMALT=0;N_HOMREF=10308;FREQ_HET=9.700261907071492e-05;FREQ_HOMALT=0.0;FREQ_HOMREF=0.9999029973809292;N_BI_GENOS=10309;South_Han_AN=1496;South_Han_AC=1;South_Han_A
chr3	93818009	.	G	<INS:ME:LINE1>	.	PASS	SVTYPE=INS:ME:LINE1;END=93818026;SVLEN=5998;AN=20618;AC=1;AF=4.850130953535746e-05;N_HET=1;N_HOMALT=0;N_HOMREF=10308;FREQ_HET=9.700261907071492e-05;FREQ_HOMALT=0.0;FREQ_HOMREF=0.9999029973809292;N_BI_GENOS=10309;South_Han_AN=1496;South_Han_AC=1;South_Han_
chr3	93964932	.	A	<INS:ME:LINE1>	.	PASS	SVTYPE=INS:ME:LINE1;END=93964941;SVLEN=2305;AN=20618;AC=1;AF=4.850130953535746e-05;N_HET=1;N_HOMALT=0;N_HOMREF=10308;FREQ_HET=9.700261907071492e-05;FREQ_HOMALT=0.0;FREQ_HOMREF=0.9999029973809292;N_BI_GENOS=10309;East_Han_AN=6554;East_Han_AC=1;East_Han_AF=
chr3	94212714	.	A	<INS:ME:LINE1>	.	PASS	SVTYPE=INS:ME:LINE1;END=94212714;SVLEN=991;AN=20618;AC=2;AF=9.700261907071492e-05;N_HET=0;N_HOMALT=1;N_HOMREF=10308;FREQ_HET=0.0;FREQ_HOMALT=9.700261907071492e-05;FREQ_HOMREF=0.9999029973809292;N_BI_GENOS=10309;East_Han_AN=6554;East_Han_AC=2;East_Han_AF=0
chr3	94360578	.	G	<INS:ME:LINE1>	.	PASS	SVTYPE=INS:ME:LINE1;END=94360578;SVLEN=0;AN=20618;AC=1;AF=4.850130953535746e-05;N_HET=1;N_HOMALT=0;N_HOMREF=10308;FREQ_HET=9.700261907071492e-05;FREQ_HOMALT=0.0;FREQ_HOMREF=0.9999029973809292;N_BI_GENOS=10309;Manchu_AN=416;Manchu_AC=1;Manchu_AF=0.00240384
chr3	94681188	.	A	<INS:ME:LINE1>	.	PASS	SVTYPE=INS:ME:LINE1;END=94681215;SVLEN=1540;AN=20618;AC=1;AF=4.850130953535746e-05;N_HET=1;N_HOMALT=0;N_HOMREF=10308;FREQ_HET=9.700261907071492e-05;FREQ_HOMALT=0.0;FREQ_HOMREF=0.9999029973809292;N_BI_GENOS=10309;East_Han_AN=6554;East_Han_AC=1;East_Han_AF=
chr3	94810978	.	A	<INS:ME:LINE1>	.	PASS	SVTYPE=INS:ME:LINE1;END=94810994;SVLEN=1218;AN=20618;AC=1;AF=4.850130953535746e-05;N_HET=1;N_HOMALT=0;N_HOMREF=10308;FREQ_HET=9.700261907071492e-05;FREQ_HOMALT=0.0;FREQ_HOMREF=0.9999029973809292;N_BI_GENOS=10309;Southeast_Han_AN=1908;Southeast_Han_AC=1;So
chr3	94899485	.	C	<INS:ME:LINE1>	.	PASS	SVTYPE=INS:ME:LINE1;END=94899504;SVLEN=5994;AN=20618;AC=1;AF=4.850130953535746e-05;N_HET=1;N_HOMALT=0;N_HOMREF=10308;FREQ_HET=9.700261907071492e-05;FREQ_HOMALT=0.0;FREQ_HOMREF=0.9999029973809292;N_BI_GENOS=10309;Central_Han_AN=2128;Central_Han_AC=1;Centra
chr3	94903738	.	G	<INS:ME:LINE1>	.	PASS	SVTYPE=INS:ME:LINE1;END=94903751;SVLEN=5998;AN=20618;AC=6;AF=0.0002910078572121447;N_HET=4;N_HOMALT=1;N_HOMREF=10304;FREQ_HET=0.00038801047628285966;FREQ_HOMALT=9.700261907071492e-05;FREQ_HOMREF=0.9995149869046465;N_BI_GENOS=10309;South_Han_AN=1496;South_
chr3	95020717	.	A	<INS:ME:LINE1>	.	PASS	SVTYPE=INS:ME:LINE1;END=95020717;SVLEN=512;AN=20618;AC=1;AF=4.850130953535746e-05;N_HET=1;N_HOMALT=0;N_HOMREF=10308;FREQ_HET=9.700261907071492e-05;FREQ_HOMALT=0.0;FREQ_HOMREF=0.9999029973809292;N_BI_GENOS=10309;East_Han_AN=6554;East_Han_AC=1;East_Han_AF=0
chr3	95993702	.	T	<INS:ME:LINE1>	.	PASS	SVTYPE=INS:ME:LINE1;END=95993717;SVLEN=2248;AN=20618;AC=1;AF=4.850130953535746e-05;N_HET=1;N_HOMALT=0;N_HOMREF=10308;FREQ_HET=9.700261907071492e-05;FREQ_HOMALT=0.0;FREQ_HOMREF=0.9999029973809292;N_BI_GENOS=10309;East_Han_AN=6554;East_Han_AC=1;East_Han_AF=
chr3	96100088	.	T	<INS:ME:LINE1>	.	PASS	SVTYPE=INS:ME:LINE1;END=96100098;SVLEN=1073;AN=20618;AC=1;AF=4.850130953535746e-05;N_HET=1;N_HOMALT=0;N_HOMREF=10308;FREQ_HET=9.700261907071492e-05;FREQ_HOMALT=0.0;FREQ_HOMREF=0.9999029973809292;N_BI_GENOS=10309;South_Han_AN=1496;South_Han_AC=1;South_Han_
chr3	96223995	.	A	<INS:ME:LINE1>	.	PASS	SVTYPE=INS:ME:LINE1;END=96224014;SVLEN=2200;AN=20618;AC=1;AF=4.850130953535746e-05;N_HET=1;N_HOMALT=0;N_HOMREF=10308;FREQ_HET=9.700261907071492e-05;FREQ_HOMALT=0.0;FREQ_HOMREF=0.9999029973809292;N_BI_GENOS=10309;Hui_AN=444;Hui_AC=1;Hui_AF=0.00225225225225
chr3	96266852	.	A	<INS:ME:LINE1>	.	PASS	SVTYPE=INS:ME:LINE1;END=96266868;SVLEN=921;AN=20618;AC=1;AF=4.850130953535746e-05;N_HET=1;N_HOMALT=0;N_HOMREF=10308;FREQ_HET=9.700261907071492e-05;FREQ_HOMALT=0.0;FREQ_HOMREF=0.9999029973809292;N_BI_GENOS=10309;East_Han_AN=6554;East_Han_AC=1;East_Han_AF=0
chr3	96309660	.	T	<INS:ME:LINE1>	.	PASS	SVTYPE=INS:ME:LINE1;END=96309679;SVLEN=671;AN=20618;AC=2;AF=9.700261907071492e-05;N_HET=2;N_HOMALT=0;N_HOMREF=10307;FREQ_HET=0.00019400523814142983;FREQ_HOMALT=0.0;FREQ_HOMREF=0.9998059947618586;N_BI_GENOS=10309;East_Han_AN=6554;East_Han_AC=2;East_Han_AF=
chr3	96383259	.	A	<INS:ME:LINE1>	.	PASS	SVTYPE=INS:ME:LINE1;END=96383259;SVLEN=267;AN=20618;AC=1;AF=4.850130953535746e-05;N_HET=1;N_HOMALT=0;N_HOMREF=10308;FREQ_HET=9.700261907071492e-05;FREQ_HOMALT=0.0;FREQ_HOMREF=0.9999029973809292;N_BI_GENOS=10309;Zhuang_AN=444;Zhuang_AC=1;Zhuang_AF=0.002252
chr3	96409938	.	A	<INS:ME:LINE1>	.	PASS	SVTYPE=INS:ME:LINE1;END=96409952;SVLEN=284;AN=20618;AC=2;AF=9.700261907071492e-05;N_HET=2;N_HOMALT=0;N_HOMREF=10307;FREQ_HET=0.00019400523814142983;FREQ_HOMALT=0.0;FREQ_HOMREF=0.9998059947618586;N_BI_GENOS=10309;East_Han_AN=6554;East_Han_AC=2;East_Han_AF=
chr3	96474518	.	A	<INS:ME:LINE1>	.	PASS	SVTYPE=INS:ME:LINE1;END=96474518;SVLEN=1578;AN=20618;AC=1;AF=4.850130953535746e-05;N_HET=1;N_HOMALT=0;N_HOMREF=10308;FREQ_HET=9.700261907071492e-05;FREQ_HOMALT=0.0;FREQ_HOMREF=0.9999029973809292;N_BI_GENOS=10309;North_Han_AN=2272;North_Han_AC=1;North_Han_
chr3	96756524	.	A	<INS:ME:LINE1>	.	PASS	SVTYPE=INS:ME:LINE1;END=96756540;SVLEN=5997;AN=20618;AC=1;AF=4.850130953535746e-05;N_HET=1;N_HOMALT=0;N_HOMREF=10308;FREQ_HET=9.700261907071492e-05;FREQ_HOMALT=0.0;FREQ_HOMREF=0.9999029973809292;N_BI_GENOS=10309;South_Han_AN=1496;South_Han_AC=1;South_Han_
chr3	96976590	.	A	<INS:ME:LINE1>	.	PASS	SVTYPE=INS:ME:LINE1;END=96976599;SVLEN=215;AN=20618;AC=2;AF=9.700261907071492e-05;N_HET=2;N_HOMALT=0;N_HOMREF=10307;FREQ_HET=0.00019400523814142983;FREQ_HOMALT=0.0;FREQ_HOMREF=0.9998059947618586;N_BI_GENOS=10309;North_Han_AN=2272;North_Han_AC=1;North_Han_
chr3	97050487	.	A	<INS:ME:LINE1>	.	PASS	SVTYPE=INS:ME:LINE1;END=97050487;SVLEN=0;AN=20618;AC=1;AF=4.850130953535746e-05;N_HET=1;N_HOMALT=0;N_HOMREF=10308;FREQ_HET=9.700261907071492e-05;FREQ_HOMALT=0.0;FREQ_HOMREF=0.9999029973809292;N_BI_GENOS=10309;Southeast_Han_AN=1908;Southeast_Han_AC=1;South
chr3	97189603	.	A	<INS:ME:LINE1>	.	PASS	SVTYPE=INS:ME:LINE1;END=97189621;SVLEN=373;AN=20618;AC=1;AF=4.850130953535746e-05;N_HET=1;N_HOMALT=0;N_HOMREF=10308;FREQ_HET=9.700261907071492e-05;FREQ_HOMALT=0.0;FREQ_HOMREF=0.9999029973809292;N_BI_GENOS=10309;Northwest_Han_AN=1296;Northwest_Han_AC=1;Nor
chr3	97202196	.	G	<INS:ME:LINE1>	.	PASS	SVTYPE=INS:ME:LINE1;END=97202212;SVLEN=6008;AN=20618;AC=1;AF=4.850130953535746e-05;N_HET=1;N_HOMALT=0;N_HOMREF=10308;FREQ_HET=9.700261907071492e-05;FREQ_HOMALT=0.0;FREQ_HOMREF=0.9999029973809292;N_BI_GENOS=10309;Central_Han_AN=2128;Central_Han_AC=1;Centra
chr3	97373678	.	G	<INS:ME:LINE1>	.	PASS	SVTYPE=INS:ME:LINE1;END=97373692;SVLEN=1767;AN=20618;AC=1;AF=4.850130953535746e-05;N_HET=1;N_HOMALT=0;N_HOMREF=10308;FREQ_HET=9.700261907071492e-05;FREQ_HOMALT=0.0;FREQ_HOMREF=0.9999029973809292;N_BI_GENOS=10309;East_Han_AN=6554;East_Han_AC=1;East_Han_AF=
chr3	97482755	.	A	<INS:ME:LINE1>	.	PASS	SVTYPE=INS:ME:LINE1;END=97482765;SVLEN=747;AN=20618;AC=1;AF=4.850130953535746e-05;N_HET=1;N_HOMALT=0;N_HOMREF=10308;FREQ_HET=9.700261907071492e-05;FREQ_HOMALT=0.0;FREQ_HOMREF=0.9999029973809292;N_BI_GENOS=10309;Southeast_Han_AN=1908;Southeast_Han_AC=1;Sou
chr3	97782247	.	T	<INS:ME:LINE1>	.	PASS	SVTYPE=INS:ME:LINE1;END=97782263;SVLEN=2008;AN=20618;AC=1;AF=4.850130953535746e-05;N_HET=1;N_HOMALT=0;N_HOMREF=10308;FREQ_HET=9.700261907071492e-05;FREQ_HOMALT=0.0;FREQ_HOMREF=0.9999029973809292;N_BI_GENOS=10309;Southeast_Han_AN=1908;Southeast_Han_AC=1;So
chr3	98079739	.	T	<INS:ME:LINE1>	.	PASS	SVTYPE=INS:ME:LINE1;END=98079754;SVLEN=604;AN=20618;AC=1;AF=4.850130953535746e-05;N_HET=1;N_HOMALT=0;N_HOMREF=10308;FREQ_HET=9.700261907071492e-05;FREQ_HOMALT=0.0;FREQ_HOMREF=0.9999029973809292;N_BI_GENOS=10309;East_Han_AN=6554;East_Han_AC=1;East_Han_AF=0
chr3	98098130	.	C	<INS:ME:LINE1>	.	PASS	SVTYPE=INS:ME:LINE1;END=98098147;SVLEN=340;AN=20618;AC=3;AF=0.00014550392860607236;N_HET=3;N_HOMALT=0;N_HOMREF=10306;FREQ_HET=0.0002910078572121447;FREQ_HOMALT=0.0;FREQ_HOMREF=0.9997089921427879;N_BI_GENOS=10309;South_Han_AN=1496;South_Han_AC=2;South_Han_
chr3	98119661	.	T	<INS:ME:LINE1>	.	PASS	SVTYPE=INS:ME:LINE1;END=98119672;SVLEN=1078;AN=20618;AC=1;AF=4.850130953535746e-05;N_HET=1;N_HOMALT=0;N_HOMREF=10308;FREQ_HET=9.700261907071492e-05;FREQ_HOMALT=0.0;FREQ_HOMREF=0.9999029973809292;N_BI_GENOS=10309;East_Han_AN=6554;East_Han_AC=1;East_Han_AF=
chr3	98191447	.	C	<INS:ME:LINE1>	.	PASS	SVTYPE=INS:ME:LINE1;END=98191447;SVLEN=0;AN=20618;AC=1;AF=4.850130953535746e-05;N_HET=1;N_HOMALT=0;N_HOMREF=10308;FREQ_HET=9.700261907071492e-05;FREQ_HOMALT=0.0;FREQ_HOMREF=0.9999029973809292;N_BI_GENOS=10309;East_Han_AN=6554;East_Han_AC=1;East_Han_AF=0.0
chr3	98291706	.	A	<INS:ME:LINE1>	.	PASS	SVTYPE=INS:ME:LINE1;END=98291718;SVLEN=2062;AN=20618;AC=1;AF=4.850130953535746e-05;N_HET=1;N_HOMALT=0;N_HOMREF=10308;FREQ_HET=9.700261907071492e-05;FREQ_HOMALT=0.0;FREQ_HOMREF=0.9999029973809292;N_BI_GENOS=10309;East_Han_AN=6554;East_Han_AC=1;East_Han_AF=
chr3	98392689	.	T	<INS:ME:LINE1>	.	PASS	SVTYPE=INS:ME:LINE1;END=98392690;SVLEN=5998;AN=20618;AC=1;AF=4.850130953535746e-05;N_HET=1;N_HOMALT=0;N_HOMREF=10308;FREQ_HET=9.700261907071492e-05;FREQ_HOMALT=0.0;FREQ_HOMREF=0.9999029973809292;N_BI_GENOS=10309;Hui_AN=444;Hui_AC=1;Hui_AF=0.00225225225225
chr3	98636154	.	A	<INS:ME:LINE1>	.	PASS	SVTYPE=INS:ME:LINE1;END=98636164;SVLEN=2541;AN=20618;AC=1;AF=4.850130953535746e-05;N_HET=1;N_HOMALT=0;N_HOMREF=10308;FREQ_HET=9.700261907071492e-05;FREQ_HOMALT=0.0;FREQ_HOMREF=0.9999029973809292;N_BI_GENOS=10309;Tibetan_AN=414;Tibetan_AC=1;Tibetan_AF=0.00
chr3	98999801	.	T	<INS:ME:LINE1>	.	PASS	SVTYPE=INS:ME:LINE1;END=98999801;SVLEN=94;AN=20618;AC=1;AF=4.850130953535746e-05;N_HET=1;N_HOMALT=0;N_HOMREF=10308;FREQ_HET=9.700261907071492e-05;FREQ_HOMALT=0.0;FREQ_HOMREF=0.9999029973809292;N_BI_GENOS=10309;East_Han_AN=6554;East_Han_AC=1;East_Han_AF=0.
chr3	99029974	.	A	<INS:ME:LINE1>	.	PASS	SVTYPE=INS:ME:LINE1;END=99029991;SVLEN=876;AN=20618;AC=1;AF=4.850130953535746e-05;N_HET=1;N_HOMALT=0;N_HOMREF=10308;FREQ_HET=9.700261907071492e-05;FREQ_HOMALT=0.0;FREQ_HOMREF=0.9999029973809292;N_BI_GENOS=10309;Tibetan_AN=414;Tibetan_AC=1;Tibetan_AF=0.002
chr3	99030774	.	A	<INS:ME:LINE1>	.	PASS	SVTYPE=INS:ME:LINE1;END=99030784;SVLEN=6000;AN=20618;AC=1;AF=4.850130953535746e-05;N_HET=1;N_HOMALT=0;N_HOMREF=10308;FREQ_HET=9.700261907071492e-05;FREQ_HOMALT=0.0;FREQ_HOMREF=0.9999029973809292;N_BI_GENOS=10309;East_Han_AN=6554;East_Han_AC=1;East_Han_AF=
chr3	99056640	.	C	<INS:ME:LINE1>	.	PASS	SVTYPE=INS:ME:LINE1;END=99056656;SVLEN=6005;AN=20618;AC=7;AF=0.0003395091667475022;N_HET=7;N_HOMALT=0;N_HOMREF=10302;FREQ_HET=0.0006790183334950044;FREQ_HOMALT=0.0;FREQ_HOMREF=0.999320981666505;N_BI_GENOS=10309;Northwest_Han_AN=1296;Northwest_Han_AC=1;Nor
chr3	99119754	.	T	<INS:ME:LINE1>	.	PASS	SVTYPE=INS:ME:LINE1;END=99119771;SVLEN=434;AN=20618;AC=2;AF=9.700261907071492e-05;N_HET=2;N_HOMALT=0;N_HOMREF=10307;FREQ_HET=0.00019400523814142983;FREQ_HOMALT=0.0;FREQ_HOMREF=0.9998059947618586;N_BI_GENOS=10309;Northwest_Han_AN=1296;Northwest_Han_AC=1;No
chr3	99331271	.	A	<INS:ME:LINE1>	.	PASS	SVTYPE=INS:ME:LINE1;END=99331283;SVLEN=5988;AN=20618;AC=1;AF=4.850130953535746e-05;N_HET=1;N_HOMALT=0;N_HOMREF=10308;FREQ_HET=9.700261907071492e-05;FREQ_HOMALT=0.0;FREQ_HOMREF=0.9999029973809292;N_BI_GENOS=10309;North_Han_AN=2272;North_Han_AC=1;North_Han_
chr3	99381524	.	A	<INS:ME:LINE1>	.	PASS	SVTYPE=INS:ME:LINE1;END=99381524;SVLEN=1444;AN=20618;AC=1;AF=4.850130953535746e-05;N_HET=1;N_HOMALT=0;N_HOMREF=10308;FREQ_HET=9.700261907071492e-05;FREQ_HOMALT=0.0;FREQ_HOMREF=0.9999029973809292;N_BI_GENOS=10309;East_Han_AN=6554;East_Han_AC=1;East_Han_AF=
chr3	99539279	.	A	<INS:ME:LINE1>	.	PASS	SVTYPE=INS:ME:LINE1;END=99539294;SVLEN=6064;AN=20618;AC=1;AF=4.850130953535746e-05;N_HET=1;N_HOMALT=0;N_HOMREF=10308;FREQ_HET=9.700261907071492e-05;FREQ_HOMALT=0.0;FREQ_HOMREF=0.9999029973809292;N_BI_GENOS=10309;East_Han_AN=6554;East_Han_AC=1;East_Han_AF=
chr3	99616983	.	A	<INS:ME:LINE1>	.	PASS	SVTYPE=INS:ME:LINE1;END=99616999;SVLEN=3404;AN=20618;AC=1;AF=4.850130953535746e-05;N_HET=1;N_HOMALT=0;N_HOMREF=10308;FREQ_HET=9.700261907071492e-05;FREQ_HOMALT=0.0;FREQ_HOMREF=0.9999029973809292;N_BI_GENOS=10309;Lingnan_Han_AN=1888;Lingnan_Han_AC=1;Lingna
chr3	99637085	.	A	<INS:ME:LINE1>	.	PASS	SVTYPE=INS:ME:LINE1;END=99637103;SVLEN=879;AN=20618;AC=2;AF=9.700261907071492e-05;N_HET=2;N_HOMALT=0;N_HOMREF=10307;FREQ_HET=0.00019400523814142983;FREQ_HOMALT=0.0;FREQ_HOMREF=0.9998059947618586;N_BI_GENOS=10309;North_Han_AN=2272;North_Han_AC=1;North_Han_
chr3	99669953	.	T	<INS:ME:LINE1>	.	PASS	SVTYPE=INS:ME:LINE1;END=99669970;SVLEN=1459;AN=20618;AC=1;AF=4.850130953535746e-05;N_HET=1;N_HOMALT=0;N_HOMREF=10308;FREQ_HET=9.700261907071492e-05;FREQ_HOMALT=0.0;FREQ_HOMREF=0.9999029973809292;N_BI_GENOS=10309;Hui_AN=444;Hui_AC=1;Hui_AF=0.00225225225225
chr3	99682793	.	A	<INS:ME:LINE1>	.	PASS	SVTYPE=INS:ME:LINE1;END=99682794;SVLEN=1298;AN=20618;AC=6;AF=0.0002910078572121447;N_HET=6;N_HOMALT=0;N_HOMREF=10303;FREQ_HET=0.0005820157144242894;FREQ_HOMALT=0.0;FREQ_HOMREF=0.9994179842855757;N_BI_GENOS=10309;East_Han_AN=6554;East_Han_AC=1;East_Han_AF=
chr3	99763236	.	A	<INS:ME:LINE1>	.	PASS	SVTYPE=INS:ME:LINE1;END=99763245;SVLEN=384;AN=20618;AC=1;AF=4.850130953535746e-05;N_HET=1;N_HOMALT=0;N_HOMREF=10308;FREQ_HET=9.700261907071492e-05;FREQ_HOMALT=0.0;FREQ_HOMREF=0.9999029973809292;N_BI_GENOS=10309;East_Han_AN=6554;East_Han_AC=1;East_Han_AF=0
chr3	99783791	.	A	<INS:ME:LINE1>	.	PASS	SVTYPE=INS:ME:LINE1;END=99783791;SVLEN=1561;AN=20618;AC=1;AF=4.850130953535746e-05;N_HET=1;N_HOMALT=0;N_HOMREF=10308;FREQ_HET=9.700261907071492e-05;FREQ_HOMALT=0.0;FREQ_HOMREF=0.9999029973809292;N_BI_GENOS=10309;Southeast_Han_AN=1908;Southeast_Han_AC=1;So
chr3	99972730	.	A	<INS:ME:LINE1>	.	PASS	SVTYPE=INS:ME:LINE1;END=99972744;SVLEN=910;AN=20618;AC=1;AF=4.850130953535746e-05;N_HET=1;N_HOMALT=0;N_HOMREF=10308;FREQ_HET=9.700261907071492e-05;FREQ_HOMALT=0.0;FREQ_HOMREF=0.9999029973809292;N_BI_GENOS=10309;South_Han_AN=1496;South_Han_AC=1;South_Han_A
chr3	99976646	.	T	<INS:ME:LINE1>	.	PASS	SVTYPE=INS:ME:LINE1;END=99976662;SVLEN=699;AN=20618;AC=2;AF=9.700261907071492e-05;N_HET=2;N_HOMALT=0;N_HOMREF=10307;FREQ_HET=0.00019400523814142983;FREQ_HOMALT=0.0;FREQ_HOMREF=0.9998059947618586;N_BI_GENOS=10309;North_Han_AN=2272;North_Han_AC=1;North_Han_
chr3	100860433	.	A	<INS:ME:LINE1>	.	PASS	SVTYPE=INS:ME:LINE1;END=100860448;SVLEN=1637;AN=20618;AC=1;AF=4.850130953535746e-05;N_HET=1;N_HOMALT=0;N_HOMREF=10308;FREQ_HET=9.700261907071492e-05;FREQ_HOMALT=0.0;FREQ_HOMREF=0.9999029973809292;N_BI_GENOS=10309;East_Han_AN=6554;East_Han_AC=1;East_Han_AF
chr3	100925932	.	G	<INS:ME:LINE1>	.	PASS	SVTYPE=INS:ME:LINE1;END=100925946;SVLEN=5993;AN=20618;AC=14;AF=0.0006790183334950044;N_HET=14;N_HOMALT=0;N_HOMREF=10295;FREQ_HET=0.001358036666900088;FREQ_HOMALT=0.0;FREQ_HOMREF=0.99864196333301;N_BI_GENOS=10309;South_Han_AN=1496;South_Han_AC=1;South_Han
chr3	101032037	.	C	<INS:ME:LINE1>	.	PASS	SVTYPE=INS:ME:LINE1;END=101032048;SVLEN=744;AN=20618;AC=2;AF=9.700261907071492e-05;N_HET=2;N_HOMALT=0;N_HOMREF=10307;FREQ_HET=0.00019400523814142983;FREQ_HOMALT=0.0;FREQ_HOMREF=0.9998059947618586;N_BI_GENOS=10309;North_Han_AN=2272;North_Han_AC=1;North_Han
chr3	101067017	.	G	<INS:ME:LINE1>	.	PASS	SVTYPE=INS:ME:LINE1;END=101067033;SVLEN=667;AN=20618;AC=3;AF=0.00014550392860607236;N_HET=3;N_HOMALT=0;N_HOMREF=10306;FREQ_HET=0.0002910078572121447;FREQ_HOMALT=0.0;FREQ_HOMREF=0.9997089921427879;N_BI_GENOS=10309;Lingnan_Han_AN=1888;Lingnan_Han_AC=2;Lingn
chr3	101082847	.	A	<INS:ME:LINE1>	.	PASS	SVTYPE=INS:ME:LINE1;END=101082863;SVLEN=751;AN=20618;AC=1;AF=4.850130953535746e-05;N_HET=1;N_HOMALT=0;N_HOMREF=10308;FREQ_HET=9.700261907071492e-05;FREQ_HOMALT=0.0;FREQ_HOMREF=0.9999029973809292;N_BI_GENOS=10309;Central_Han_AN=2128;Central_Han_AC=1;Centra
chr3	101262022	.	T	<INS:ME:LINE1>	.	PASS	SVTYPE=INS:ME:LINE1;END=101262040;SVLEN=6000;AN=20618;AC=1;AF=4.850130953535746e-05;N_HET=1;N_HOMALT=0;N_HOMREF=10308;FREQ_HET=9.700261907071492e-05;FREQ_HOMALT=0.0;FREQ_HOMREF=0.9999029973809292;N_BI_GENOS=10309;Southeast_Han_AN=1908;Southeast_Han_AC=1;S
chr3	101326151	.	T	<INS:ME:LINE1>	.	PASS	SVTYPE=INS:ME:LINE1;END=101326151;SVLEN=1682;AN=20618;AC=1;AF=4.850130953535746e-05;N_HET=1;N_HOMALT=0;N_HOMREF=10308;FREQ_HET=9.700261907071492e-05;FREQ_HOMALT=0.0;FREQ_HOMREF=0.9999029973809292;N_BI_GENOS=10309;Yi_AN=422;Yi_AC=1;Yi_AF=0.0023696682464454

chr3	101326359	.	A	<INS:ME:LINE1>	.	PASS	SVTYPE=INS:ME:LINE1;END=101326371;SVLEN=565;AN=20618;AC=1;AF=4.850130953535746e-05;N_HET=1;N_HOMALT=0;N_HOMREF=10308;FREQ_HET=9.700261907071492e-05;FREQ_HOMALT=0.0;FREQ_HOMREF=0.9999029973809292;N_BI_GENOS=10309;Hui_AN=444;Hui_AC=1;Hui_AF=0.0022522522525225
chr3	101399628	.	T	<INS:ME:LINE1>	.	PASS	SVTYPE=INS:ME:LINE1;END=101399629;SVLEN=309;AN=20618;AC=1;AF=4.850130953535746e-05;N_HET=1;N_HOMALT=0;N_HOMREF=10308;FREQ_HET=9.700261907071492e-05;FREQ_HOMALT=0.0;FREQ_HOMREF=0.9999029973809292;N_BI_GENOS=10309;East_Han_AN=6554;East_Han_AC=1;East_Han_AF=
chr3	101417915	.	A	<INS:ME:LINE1>	.	PASS	SVTYPE=INS:ME:LINE1;END=101417932;SVLEN=1103;AN=20618;AC=10;AF=0.00048501309535357455;N_HET=10;N_HOMALT=0;N_HOMREF=10299;FREQ_HET=0.0009700261907071491;FREQ_HOMALT=0.0;FREQ_HOMREF=0.9990299738092928;N_BI_GENOS=10309;South_Han_AN=1496;South_Han_AC=1;South_
chr3	101480371	.	T	<INS:ME:LINE1>	.	PASS	SVTYPE=INS:ME:LINE1;END=101480374;SVLEN=1097;AN=20618;AC=1;AF=4.850130953535746e-05;N_HET=1;N_HOMALT=0;N_HOMREF=10308;FREQ_HET=9.700261907071492e-05;FREQ_HOMALT=0.0;FREQ_HOMREF=0.9999029973809292;N_BI_GENOS=10309;Lingnan_Han_AN=1888;Lingnan_Han_AC=1;Lingn
chr3	101480829	.	T	<INS:ME:LINE1>	.	PASS	SVTYPE=INS:ME:LINE1;END=101480835;SVLEN=692;AN=20618;AC=1;AF=4.850130953535746e-05;N_HET=1;N_HOMALT=0;N_HOMREF=10308;FREQ_HET=9.700261907071492e-05;FREQ_HOMALT=0.0;FREQ_HOMREF=0.9999029973809292;N_BI_GENOS=10309;Southeast_Han_AN=1908;Southeast_Han_AC=1;So
chr3	101560799	.	A	<INS:ME:LINE1>	.	PASS	SVTYPE=INS:ME:LINE1;END=101560813;SVLEN=5998;AN=20618;AC=4536;AF=0.2200019400523814;N_HET=3204;N_HOMALT=666;N_HOMREF=6439;FREQ_HET=0.31079639150257055;FREQ_HOMALT=0.06460374430109612;FREQ_HOMREF=0.6245998641963333;N_BI_GENOS=10309;South_Han_AN=1496;South_
chr3	101918377	.	A	<INS:ME:LINE1>	.	PASS	SVTYPE=INS:ME:LINE1;END=101918391;SVLEN=478;AN=20618;AC=1;AF=4.850130953535746e-05;N_HET=1;N_HOMALT=0;N_HOMREF=10308;FREQ_HET=9.700261907071492e-05;FREQ_HOMALT=0.0;FREQ_HOMREF=0.9999029973809292;N_BI_GENOS=10309;East_Han_AN=6554;East_Han_AC=1;East_Han_AF=
chr3	102044514	.	A	<INS:ME:LINE1>	.	PASS	SVTYPE=INS:ME:LINE1;END=102044529;SVLEN=1703;AN=20618;AC=86;AF=0.004171112620040741;N_HET=0;N_HOMALT=43;N_HOMREF=10266;FREQ_HET=0.0;FREQ_HOMALT=0.004171112620040741;FREQ_HOMREF=0.9958288873799592;N_BI_GENOS=10309;South_Han_AN=1496;South_Han_AC=10;South_Ha
chr3	102170449	.	A	<INS:ME:LINE1>	.	PASS	SVTYPE=INS:ME:LINE1;END=102170465;SVLEN=5987;AN=20618;AC=1;AF=4.850130953535746e-05;N_HET=1;N_HOMALT=0;N_HOMREF=10308;FREQ_HET=9.700261907071492e-05;FREQ_HOMALT=0.0;FREQ_HOMREF=0.9999029973809292;N_BI_GENOS=10309;Southeast_Han_AN=1908;Southeast_Han_AC=1;S
chr3	102172422	.	A	<INS:ME:LINE1>	.	PASS	SVTYPE=INS:ME:LINE1;END=102172439;SVLEN=6016;AN=20618;AC=1;AF=4.850130953535746e-05;N_HET=1;N_HOMALT=0;N_HOMREF=10308;FREQ_HET=9.700261907071492e-05;FREQ_HOMALT=0.0;FREQ_HOMREF=0.9999029973809292;N_BI_GENOS=10309;Northwest_Han_AN=1296;Northwest_Han_AC=1;N
chr3	102433527	.	T	<INS:ME:LINE1>	.	PASS	SVTYPE=INS:ME:LINE1;END=102433540;SVLEN=44;AN=20618;AC=1;AF=4.850130953535746e-05;N_HET=1;N_HOMALT=0;N_HOMREF=10308;FREQ_HET=9.700261907071492e-05;FREQ_HOMALT=0.0;FREQ_HOMREF=0.9999029973809292;N_BI_GENOS=10309;Lingnan_Han_AN=1888;Lingnan_Han_AC=1;Lingnan
chr3	102484874	.	A	<INS:ME:LINE1>	.	PASS	SVTYPE=INS:ME:LINE1;END=102484888;SVLEN=277;AN=20618;AC=1;AF=4.850130953535746e-05;N_HET=1;N_HOMALT=0;N_HOMREF=10308;FREQ_HET=9.700261907071492e-05;FREQ_HOMALT=0.0;FREQ_HOMREF=0.9999029973809292;N_BI_GENOS=10309;Southeast_Han_AN=1908;Southeast_Han_AC=1;So
chr3	102490018	.	A	<INS:ME:LINE1>	.	PASS	SVTYPE=INS:ME:LINE1;END=102490027;SVLEN=5998;AN=20618;AC=1;AF=4.850130953535746e-05;N_HET=1;N_HOMALT=0;N_HOMREF=10308;FREQ_HET=9.700261907071492e-05;FREQ_HOMALT=0.0;FREQ_HOMREF=0.9999029973809292;N_BI_GENOS=10309;Central_Han_AN=2128;Central_Han_AC=1;Centr
chr3	102729561	.	A	<INS:ME:LINE1>	.	PASS	SVTYPE=INS:ME:LINE1;END=102729609;SVLEN=1981;AN=20618;AC=2;AF=9.700261907071492e-05;N_HET=2;N_HOMALT=0;N_HOMREF=10307;FREQ_HET=0.00019400523814142983;FREQ_HOMALT=0.0;FREQ_HOMREF=0.9998059947618586;N_BI_GENOS=10309;East_Han_AN=6554;East_Han_AC=2;East_Han_A
chr3	102790172	.	G	<INS:ME:LINE1>	.	PASS	SVTYPE=INS:ME:LINE1;END=102790187;SVLEN=6006;AN=20618;AC=1;AF=4.850130953535746e-05;N_HET=1;N_HOMALT=0;N_HOMREF=10308;FREQ_HET=9.700261907071492e-05;FREQ_HOMALT=0.0;FREQ_HOMREF=0.9999029973809292;N_BI_GENOS=10309;Southeast_Han_AN=1908;Southeast_Han_AC=1;S
chr3	102824528	.	A	<INS:ME:LINE1>	.	PASS	SVTYPE=INS:ME:LINE1;END=102824540;SVLEN=538;AN=20618;AC=1;AF=4.850130953535746e-05;N_HET=1;N_HOMALT=0;N_HOMREF=10308;FREQ_HET=9.700261907071492e-05;FREQ_HOMALT=0.0;FREQ_HOMREF=0.9999029973809292;N_BI_GENOS=10309;Manchu_AN=416;Manchu_AC=1;Manchu_AF=0.00240
chr3	102874095	.	A	<INS:ME:LINE1>	.	PASS	SVTYPE=INS:ME:LINE1;END=102874111;SVLEN=2160;AN=20618;AC=1;AF=4.850130953535746e-05;N_HET=1;N_HOMALT=0;N_HOMREF=10308;FREQ_HET=9.700261907071492e-05;FREQ_HOMALT=0.0;FREQ_HOMREF=0.9999029973809292;N_BI_GENOS=10309;South_Han_AN=1496;South_Han_AC=1;South_Han
chr3	103627787	.	A	<INS:ME:LINE1>	.	PASS	SVTYPE=INS:ME:LINE1;END=103627798;SVLEN=100;AN=20618;AC=1;AF=4.850130953535746e-05;N_HET=1;N_HOMALT=0;N_HOMREF=10308;FREQ_HET=9.700261907071492e-05;FREQ_HOMALT=0.0;FREQ_HOMREF=0.9999029973809292;N_BI_GENOS=10309;Lingnan_Han_AN=1888;Lingnan_Han_AC=1;Lingna
chr3	103666745	.	C	<INS:ME:LINE1>	.	PASS	SVTYPE=INS:ME:LINE1;END=103666762;SVLEN=1124;AN=20618;AC=3;AF=0.00014550392860607236;N_HET=3;N_HOMALT=0;N_HOMREF=10306;FREQ_HET=0.0002910078572121447;FREQ_HOMALT=0.0;FREQ_HOMREF=0.9997089921427879;N_BI_GENOS=10309;Lingnan_Han_AN=1888;Lingnan_Han_AC=1;Ling
chr3	104106367	.	A	<INS:ME:LINE1>	.	PASS	SVTYPE=INS:ME:LINE1;END=104106367;SVLEN=220;AN=20618;AC=1;AF=4.850130953535746e-05;N_HET=1;N_HOMALT=0;N_HOMREF=10308;FREQ_HET=9.700261907071492e-05;FREQ_HOMALT=0.0;FREQ_HOMREF=0.9999029973809292;N_BI_GENOS=10309;East_Han_AN=6554;East_Han_AC=1;East_Han_AF=
chr3	104291130	.	A	<INS:ME:LINE1>	.	PASS	SVTYPE=INS:ME:LINE1;END=104291145;SVLEN=1329;AN=20618;AC=7;AF=0.0003395091667475022;N_HET=7;N_HOMALT=0;N_HOMREF=10302;FREQ_HET=0.0006790183334950044;FREQ_HOMALT=0.0;FREQ_HOMREF=0.999320981666505;N_BI_GENOS=10309;North_Han_AN=2272;North_Han_AC=4;North_Han_
chr3	104588500	.	A	<INS:ME:LINE1>	.	PASS	SVTYPE=INS:ME:LINE1;END=104588500;SVLEN=1029;AN=20618;AC=1;AF=4.850130953535746e-05;N_HET=1;N_HOMALT=0;N_HOMREF=10308;FREQ_HET=9.700261907071492e-05;FREQ_HOMALT=0.0;FREQ_HOMREF=0.9999029973809292;N_BI_GENOS=10309;South_Han_AN=1496;South_Han_AC=1;South_Han
chr3	104889161	.	T	<INS:ME:LINE1>	.	PASS	SVTYPE=INS:ME:LINE1;END=104889173;SVLEN=1463;AN=20618;AC=82;AF=0.003977107381899311;N_HET=82;N_HOMALT=0;N_HOMREF=10227;FREQ_HET=0.007954214763798623;FREQ_HOMALT=0.0;FREQ_HOMREF=0.9920457852362013;N_BI_GENOS=10309;South_Han_AN=1496;South_Han_AC=4;South_Han
chr3	104966252	.	C	<INS:ME:LINE1>	.	PASS	SVTYPE=INS:ME:LINE1;END=104966264;SVLEN=168;AN=20618;AC=2;AF=9.700261907071492e-05;N_HET=2;N_HOMALT=0;N_HOMREF=10307;FREQ_HET=0.00019400523814142983;FREQ_HOMALT=0.0;FREQ_HOMREF=0.9998059947618586;N_BI_GENOS=10309;Lingnan_Han_AN=1888;Lingnan_Han_AC=1;Lingn
chr3	105128855	.	G	<INS:ME:LINE1>	.	PASS	SVTYPE=INS:ME:LINE1;END=105128855;SVLEN=59;AN=20618;AC=1;AF=4.850130953535746e-05;N_HET=1;N_HOMALT=0;N_HOMREF=10308;FREQ_HET=9.700261907071492e-05;FREQ_HOMALT=0.0;FREQ_HOMREF=0.9999029973809292;N_BI_GENOS=10309;East_Han_AN=6554;East_Han_AC=1;East_Han_AF=0
chr3	105140129	.	A	<INS:ME:LINE1>	.	PASS	SVTYPE=INS:ME:LINE1;END=105140141;SVLEN=2808;AN=20618;AC=2;AF=9.700261907071492e-05;N_HET=2;N_HOMALT=0;N_HOMREF=10307;FREQ_HET=0.00019400523814142983;FREQ_HOMALT=0.0;FREQ_HOMREF=0.9998059947618586;N_BI_GENOS=10309;Hui_AN=444;Hui_AC=1;Hui_AF=0.002252252252
chr3	105686573	.	A	<INS:ME:LINE1>	.	PASS	SVTYPE=INS:ME:LINE1;END=105686587;SVLEN=5526;AN=20618;AC=7;AF=0.0003395091667475022;N_HET=7;N_HOMALT=0;N_HOMREF=10302;FREQ_HET=0.0006790183334950044;FREQ_HOMALT=0.0;FREQ_HOMREF=0.999320981666505;N_BI_GENOS=10309;Northwest_Han_AN=1296;Northwest_Han_AC=1;No
chr3	105927666	.	A	<INS:ME:LINE1>	.	PASS	SVTYPE=INS:ME:LINE1;END=105927682;SVLEN=595;AN=20618;AC=1;AF=4.850130953535746e-05;N_HET=1;N_HOMALT=0;N_HOMREF=10308;FREQ_HET=9.700261907071492e-05;FREQ_HOMALT=0.0;FREQ_HOMREF=0.9999029973809292;N_BI_GENOS=10309;North_Han_AN=2272;North_Han_AC=1;North_Han_
chr3	106311308	.	T	<INS:ME:LINE1>	.	PASS	SVTYPE=INS:ME:LINE1;END=106311323;SVLEN=6001;AN=20618;AC=1;AF=4.850130953535746e-05;N_HET=1;N_HOMALT=0;N_HOMREF=10308;FREQ_HET=9.700261907071492e-05;FREQ_HOMALT=0.0;FREQ_HOMREF=0.9999029973809292;N_BI_GENOS=10309;East_Han_AN=6554;East_Han_AC=1;East_Han_AF
chr3	106560308	.	T	<INS:ME:LINE1>	.	PASS	SVTYPE=INS:ME:LINE1;END=106560323;SVLEN=1285;AN=20618;AC=1;AF=4.850130953535746e-05;N_HET=1;N_HOMALT=0;N_HOMREF=10308;FREQ_HET=9.700261907071492e-05;FREQ_HOMALT=0.0;FREQ_HOMREF=0.9999029973809292;N_BI_GENOS=10309;South_Han_AN=1496;South_Han_AC=1;South_Han
chr3	106616462	.	A	<INS:ME:LINE1>	.	PASS	SVTYPE=INS:ME:LINE1;END=106616468;SVLEN=1274;AN=20618;AC=1;AF=4.850130953535746e-05;N_HET=1;N_HOMALT=0;N_HOMREF=10308;FREQ_HET=9.700261907071492e-05;FREQ_HOMALT=0.0;FREQ_HOMREF=0.9999029973809292;N_BI_GENOS=10309;North_Han_AN=2272;North_Han_AC=1;North_Han
chr3	107169122	.	G	<INS:ME:LINE1>	.	PASS	SVTYPE=INS:ME:LINE1;END=107169168;SVLEN=1897;AN=20618;AC=1;AF=4.850130953535746e-05;N_HET=1;N_HOMALT=0;N_HOMREF=10308;FREQ_HET=9.700261907071492e-05;FREQ_HOMALT=0.0;FREQ_HOMREF=0.9999029973809292;N_BI_GENOS=10309;East_Han_AN=6554;East_Han_AC=1;East_Han_AF
chr3	107169926	.	C	<INS:ME:LINE1>	.	PASS	SVTYPE=INS:ME:LINE1;END=107169926;SVLEN=404;AN=20618;AC=1;AF=4.850130953535746e-05;N_HET=1;N_HOMALT=0;N_HOMREF=10308;FREQ_HET=9.700261907071492e-05;FREQ_HOMALT=0.0;FREQ_HOMREF=0.9999029973809292;N_BI_GENOS=10309;East_Han_AN=6554;East_Han_AC=1;East_Han_AF=
chr3	107358735	.	A	<INS:ME:LINE1>	.	PASS	SVTYPE=INS:ME:LINE1;END=107358750;SVLEN=6030;AN=20618;AC=1;AF=4.850130953535746e-05;N_HET=1;N_HOMALT=0;N_HOMREF=10308;FREQ_HET=9.700261907071492e-05;FREQ_HOMALT=0.0;FREQ_HOMREF=0.9999029973809292;N_BI_GENOS=10309;Tibetan_AN=414;Tibetan_AC=1;Tibetan_AF=0.0
chr3	107591286	.	A	<INS:ME:LINE1>	.	PASS	SVTYPE=INS:ME:LINE1;END=107591303;SVLEN=5994;AN=20618;AC=1;AF=4.850130953535746e-05;N_HET=1;N_HOMALT=0;N_HOMREF=10308;FREQ_HET=9.700261907071492e-05;FREQ_HOMALT=0.0;FREQ_HOMREF=0.9999029973809292;N_BI_GENOS=10309;Southeast_Han_AN=1908;Southeast_Han_AC=1;S
chr3	107638758	.	A	<INS:ME:LINE1>	.	PASS	SVTYPE=INS:ME:LINE1;END=107638818;SVLEN=1992;AN=20618;AC=1;AF=4.850130953535746e-05;N_HET=1;N_HOMALT=0;N_HOMREF=10308;FREQ_HET=9.700261907071492e-05;FREQ_HOMALT=0.0;FREQ_HOMREF=0.9999029973809292;N_BI_GENOS=10309;East_Han_AN=6554;East_Han_AC=1;East_Han_AF
chr3	107761461	.	A	<INS:ME:LINE1>	.	PASS	SVTYPE=INS:ME:LINE1;END=107761476;SVLEN=1218;AN=20618;AC=1;AF=4.850130953535746e-05;N_HET=1;N_HOMALT=0;N_HOMREF=10308;FREQ_HET=9.700261907071492e-05;FREQ_HOMALT=0.0;FREQ_HOMREF=0.9999029973809292;N_BI_GENOS=10309;Lingnan_Han_AN=1888;Lingnan_Han_AC=1;Lingn
chr3	108282987	.	G	<INS:ME:LINE1>	.	PASS	SVTYPE=INS:ME:LINE1;END=108283001;SVLEN=704;AN=20618;AC=1;AF=4.850130953535746e-05;N_HET=1;N_HOMALT=0;N_HOMREF=10308;FREQ_HET=9.700261907071492e-05;FREQ_HOMALT=0.0;FREQ_HOMREF=0.9999029973809292;N_BI_GENOS=10309;North_Han_AN=2272;North_Han_AC=1;North_Han_
chr3	108419835	.	A	<INS:ME:LINE1>	.	PASS	SVTYPE=INS:ME:LINE1;END=108419850;SVLEN=429;AN=20618;AC=601;AF=0.02914928703074983;N_HET=557;N_HOMALT=22;N_HOMREF=9730;FREQ_HET=0.0540304588223882;FREQ_HOMALT=0.002134057619555728;FREQ_HOMREF=0.943835483558056;N_BI_GENOS=10309;South_Han_AN=1496;South_Han_
chr3	108727888	.	A	<INS:ME:LINE1>	.	PASS	SVTYPE=INS:ME:LINE1;END=108727895;SVLEN=489;AN=20618;AC=1;AF=4.850130953535746e-05;N_HET=1;N_HOMALT=0;N_HOMREF=10308;FREQ_HET=9.700261907071492e-05;FREQ_HOMALT=0.0;FREQ_HOMREF=0.9999029973809292;N_BI_GENOS=10309;Lingnan_Han_AN=1888;Lingnan_Han_AC=1;Lingna
chr3	108999198	.	T	<INS:ME:LINE1>	.	PASS	SVTYPE=INS:ME:LINE1;END=108999216;SVLEN=448;AN=20618;AC=1;AF=4.850130953535746e-05;N_HET=1;N_HOMALT=0;N_HOMREF=10308;FREQ_HET=9.700261907071492e-05;FREQ_HOMALT=0.0;FREQ_HOMREF=0.9999029973809292;N_BI_GENOS=10309;Central_Han_AN=2128;Central_Han_AC=1;Centra
chr3	109037161	.	C	<INS:ME:LINE1>	.	PASS	SVTYPE=INS:ME:LINE1;END=109037179;SVLEN=392;AN=20618;AC=2;AF=9.700261907071492e-05;N_HET=2;N_HOMALT=0;N_HOMREF=10307;FREQ_HET=0.00019400523814142983;FREQ_HOMALT=0.0;FREQ_HOMREF=0.9998059947618586;N_BI_GENOS=10309;South_Han_AN=1496;South_Han_AC=1;South_Han
chr3	109484143	.	G	<INS:ME:LINE1>	.	PASS	SVTYPE=INS:ME:LINE1;END=109484143;SVLEN=231;AN=20618;AC=3;AF=0.00014550392860607236;N_HET=3;N_HOMALT=0;N_HOMREF=10306;FREQ_HET=0.0002910078572121447;FREQ_HOMALT=0.0;FREQ_HOMREF=0.9997089921427879;N_BI_GENOS=10309;South_Han_AN=1496;South_Han_AC=1;South_Han
chr3	109568343	.	T	<INS:ME:LINE1>	.	PASS	SVTYPE=INS:ME:LINE1;END=109568344;SVLEN=981;AN=20618;AC=1;AF=4.850130953535746e-05;N_HET=1;N_HOMALT=0;N_HOMREF=10308;FREQ_HET=9.700261907071492e-05;FREQ_HOMALT=0.0;FREQ_HOMREF=0.9999029973809292;N_BI_GENOS=10309;East_Han_AN=6554;East_Han_AC=1;East_Han_AF=
chr3	109744078	.	G	<INS:ME:LINE1>	.	PASS	SVTYPE=INS:ME:LINE1;END=109744095;SVLEN=1987;AN=20618;AC=48;AF=0.0023280628576971578;N_HET=48;N_HOMALT=0;N_HOMREF=10261;FREQ_HET=0.004656125715394315;FREQ_HOMALT=0.0;FREQ_HOMREF=0.9953438742846057;N_BI_GENOS=10309;South_Han_AN=1496;South_Han_AC=8;South_H
chr3	109786885	.	G	<INS:ME:LINE1>	.	PASS	SVTYPE=INS:ME:LINE1;END=109786885;SVLEN=5997;AN=20618;AC=1;AF=4.850130953535746e-05;N_HET=1;N_HOMALT=0;N_HOMREF=10308;FREQ_HET=9.700261907071492e-05;FREQ_HOMALT=0.0;FREQ_HOMREF=0.9999029973809292;N_BI_GENOS=10309;Manchu_AN=416;Manchu_AC=1;Manchu_AF=0.0024
chr3	110032909	.	A	<INS:ME:LINE1>	.	PASS	SVTYPE=INS:ME:LINE1;END=110032923;SVLEN=253;AN=20618;AC=1109;AF=0.05378795227471142;N_HET=853;N_HOMALT=128;N_HOMREF=9328;FREQ_HET=0.08274323406731982;FREQ_HOMALT=0.01241633524105151;FREQ_HOMREF=0.9048404306916287;N_BI_GENOS=10309;South_Han_AN=1496;South_H
chr3	110492918	.	A	<INS:ME:LINE1>	.	PASS	SVTYPE=INS:ME:LINE1;END=110492934;SVLEN=5998;AN=20618;AC=1;AF=4.850130953535746e-05;N_HET=1;N_HOMALT=0;N_HOMREF=10308;FREQ_HET=9.700261907071492e-05;FREQ_HOMALT=0.0;FREQ_HOMREF=0.9999029973809292;N_BI_GENOS=10309;East_Han_AN=6554;East_Han_AC=1;East_Han_AF
chr3	110552177	.	C	<INS:ME:LINE1>	.	PASS	SVTYPE=INS:ME:LINE1;END=110552177;SVLEN=1;AN=20618;AC=3;AF=0.00014550392860607236;N_HET=1;N_HOMALT=1;N_HOMREF=10307;FREQ_HET=9.700261907071492e-05;FREQ_HOMALT=9.700261907071492e-05;FREQ_HOMREF=0.9998059947618586;N_BI_GENOS=10309;South_Han_AN=1496;South_Ha
chr3	110743252	.	T	<INS:ME:LINE1>	.	PASS	SVTYPE=INS:ME:LINE1;END=110743268;SVLEN=375;AN=20618;AC=2;AF=9.700261907071492e-05;N_HET=2;N_HOMALT=0;N_HOMREF=10307;FREQ_HET=0.00019400523814142983;FREQ_HOMALT=0.0;FREQ_HOMREF=0.9998059947618586;N_BI_GENOS=10309;East_Han_AN=6554;East_Han_AC=2;East_Han_AF
chr3	110872124	.	G	<INS:ME:LINE1>	.	PASS	SVTYPE=INS:ME:LINE1;END=110872134;SVLEN=5998;AN=20618;AC=7;AF=0.0003395091667475022;N_HET=5;N_HOMALT=1;N_HOMREF=10303;FREQ_HET=0.00048501309535357455;FREQ_HOMALT=9.700261907071492e-05;FREQ_HOMREF=0.9994179842855757;N_BI_GENOS=10309;North_Han_AN=2272;North
chr3	110909199	.	T	<INS:ME:LINE1>	.	PASS	SVTYPE=INS:ME:LINE1;END=110909201;SVLEN=1524;AN=20618;AC=2;AF=9.700261907071492e-05;N_HET=2;N_HOMALT=0;N_HOMREF=10307;FREQ_HET=0.00019400523814142983;FREQ_HOMALT=0.0;FREQ_HOMREF=0.9998059947618586;N_BI_GENOS=10309;East_Han_AN=6554;East_Han_AC=1;East_Han_A
chr3	111043023	.	T	<INS:ME:LINE1>	.	PASS	SVTYPE=INS:ME:LINE1;END=111043023;SVLEN=149;AN=20618;AC=1;AF=4.850130953535746e-05;N_HET=1;N_HOMALT=0;N_HOMREF=10308;FREQ_HET=9.700261907071492e-05;FREQ_HOMALT=0.0;FREQ_HOMREF=0.9999029973809292;N_BI_GENOS=10309;East_Han_AN=6554;East_Han_AC=1;East_Han_AF=
chr3	111320735	.	T	<INS:ME:LINE1>	.	PASS	SVTYPE=INS:ME:LINE1;END=111320736;SVLEN=758;AN=20618;AC=1;AF=4.850130953535746e-05;N_HET=1;N_HOMALT=0;N_HOMREF=10308;FREQ_HET=9.700261907071492e-05;FREQ_HOMALT=0.0;FREQ_HOMREF=0.9999029973809292;N_BI_GENOS=10309;Tibetan_AN=414;Tibetan_AC=1;Tibetan_AF=0.00
chr3	111426515	.	T	<INS:ME:LINE1>	.	PASS	SVTYPE=INS:ME:LINE1;END=111426515;SVLEN=826;AN=20618;AC=1;AF=4.850130953535746e-05;N_HET=1;N_HOMALT=0;N_HOMREF=10308;FREQ_HET=9.700261907071492e-05;FREQ_HOMALT=0.0;FREQ_HOMREF=0.9999029973809292;N_BI_GENOS=10309;East_Han_AN=6554;East_Han_AC=1;East_Han_AF=
chr3	111484512	.	T	<INS:ME:LINE1>	.	PASS	SVTYPE=INS:ME:LINE1;END=111484512;SVLEN=0;AN=20618;AC=31;AF=0.0015035405955960812;N_HET=1;N_HOMALT=15;N_HOMREF=10293;FREQ_HET=9.700261907071492e-05;FREQ_HOMALT=0.0014550392860607237;FREQ_HOMREF=0.9984479580948685;N_BI_GENOS=10309;South_Han_AN=1496;South_H
chr3	111493207	.	A	<INS:ME:LINE1>	.	PASS	SVTYPE=INS:ME:LINE1;END=111493211;SVLEN=4151;AN=20618;AC=6;AF=0.0002910078572121447;N_HET=4;N_HOMALT=1;N_HOMREF=10304;FREQ_HET=0.0003880104762828596;FREQ_HOMALT=9.700261907071492e-05;FREQ_HOMREF=0.9995149869046465;N_BI_GENOS=10309;North_Han_AN=2272;North
chr3	111586078	.	A	<INS:ME:LINE1>	.	PASS	SVTYPE=INS:ME:LINE1;END=111586093;SVLEN=6000;AN=20618;AC=3;AF=0.00014550392860607236;N_HET=3;N_HOMALT=0;N_HOMREF=10306;FREQ_HET=0.0002910078572121447;FREQ_HOMALT=0.0;FREQ_HOMREF=0.9997089921427879;N_BI_GENOS=10309;Lingnan_Han_AN=1888;Lingnan_Han_AC=3;Ling
chr3	111871984	.	C	<INS:ME:LINE1>	.	PASS	SVTYPE=INS:ME:LINE1;END=111871984;SVLEN=1168;AN=20618;AC=1;AF=4.850130953535746e-05;N_HET=1;N_HOMALT=0;N_HOMREF=10308;FREQ_HET=9.700261907071492e-05;FREQ_HOMALT=0.0;FREQ_HOMREF=0.9999029973809292;N_BI_GENOS=10309;East_Han_AN=6554;East_Han_AC=1;East_Han_AF
chr3	112186367	.	T	<INS:ME:LINE1>	.	PASS	SVTYPE=INS:ME:LINE1;END=112186367;SVLEN=1902;AN=20618;AC=1;AF=4.850130953535746e-05;N_HET=1;N_HOMALT=0;N_HOMREF=10308;FREQ_HET=9.700261907071492e-05;FREQ_HOMALT=0.0;FREQ_HOMREF=0.9999029973809292;N_BI_GENOS=10309;Central_Han_AN=2128;Central_Han_AC=1;Centr
chr3	112187070	.	T	<INS:ME:LINE1>	.	PASS	SVTYPE=INS:ME:LINE1;END=112187074;SVLEN=475;AN=20618;AC=2;AF=9.700261907071492e-05;N_HET=2;N_HOMALT=0;N_HOMREF=10307;FREQ_HET=0.00019400523814142983;FREQ_HOMALT=0.0;FREQ_HOMREF=0.9998059947618586;N_BI_GENOS=10309;Lingnan_Han_AN=1888;Lingnan_Han_AC=2;Lingn
chr3	112208784	.	A	<INS:ME:LINE1>	.	PASS	SVTYPE=INS:ME:LINE1;END=112208798;SVLEN=5998;AN=20618;AC=91;AF=0.004413619167717529;N_HET=81;N_HOMALT=5;N_HOMREF=10223;FREQ_HET=0.007857212144727907;FREQ_HOMALT=0.00048501309535357455;FREQ_HOMREF=0.9916577747599186;N_BI_GENOS=10309;South_Han_AN=1496;South
chr3	112390538	.	A	<INS:ME:LINE1>	.	PASS	SVTYPE=INS:ME:LINE1;END=112390555;SVLEN=6006;AN=20618;AC=7;AF=0.0003395091667475022;N_HET=5;N_HOMALT=1;N_HOMREF=10303;FREQ_HET=0.00048501309535357455;FREQ_HOMALT=9.700261907071492e-05;FREQ_HOMREF=0.9994179842855757;N_BI_GENOS=10309;South_Han_AN=1496;South
chr3	112747857	.	T	<INS:ME:LINE1>	.	PASS	SVTYPE=INS:ME:LINE1;END=112747857;SVLEN=5;AN=20618;AC=6;AF=0.0002910078572121447;N_HET=4;N_HOMALT=1;N_HOMREF=10304;FREQ_HET=0.0003880104762828596;FREQ_HOMALT=9.700261907071492e-05;FREQ_HOMREF=0.9995149869046465;N_BI_GENOS=10309;East_Han_AN=6554;East_Han_
chr3	112843002	.	A	<INS:ME:LINE1>	.	PASS	SVTYPE=INS:ME:LINE1;END=112843019;SVLEN=139;AN=20618;AC=1;AF=4.850130953535746e-05;N_HET=1;N_HOMALT=0;N_HOMREF=10308;FREQ_HET=9.700261907071492e-05;FREQ_HOMALT=0.0;FREQ_HOMREF=0.9999029973809292;N_BI_GENOS=10309;Zhuang_AN=444;Zhuang_AC=1;Zhuang_AF=0.00225
chr3	112854810	.	A	<INS:ME:LINE1>	.	PASS	SVTYPE=INS:ME:LINE1;END=112854810;SVLEN=3328;AN=20618;AC=28;AF=0.0013580366669900088;N_HET=20;N_HOMALT=4;N_HOMREF=10285;FREQ_HET=0.0019400523814142982;FREQ_HOMALT=0.0003880104762828596;FREQ_HOMREF=0.9976719371423028;N_BI_GENOS=10309;South_Han_AN=1496;Sou
chr3	113079582	.	A	<INS:ME:LINE1>	.	PASS	SVTYPE=INS:ME:LINE1;END=113079597;SVLEN=1030;AN=20618;AC=1;AF=4.850130953535746e-05;N_HET=1;N_HOMALT=0;N_HOMREF=10308;FREQ_HET=9.700261907071492e-05;FREQ_HOMALT=0.0;FREQ_HOMREF=0.9999029973809292;N_BI_GENOS=10309;Southeast_Han_AN=1908;Southeast_Han_AC=1;S
chr3	113169113	.	C	<INS:ME:LINE1>	.	PASS	SVTYPE=INS:ME:LINE1;END=113169131;SVLEN=5998;AN=20618;AC=1;AF=4.850130953535746e-05;N_HET=1;N_HOMALT=0;N_HOMREF=10308;FREQ_HET=9.700261907071492e-05;FREQ_HOMALT=0.0;FREQ_HOMREF=0.9999029973809292;N_BI_GENOS=10309;North_Han_AN=2272;North_Han_AC=1;North_Han
chr3	113210610	.	C	<INS:ME:LINE1>	.	PASS	SVTYPE=INS:ME:LINE1;END=113210628;SVLEN=247;AN=20618;AC=1;AF=4.850130953535746e-05;N_HET=1;N_HOMALT=0;N_HOMREF=10308;FREQ_HET=9.700261907071492e-05;FREQ_HOMALT=0.0;FREQ_HOMREF=0.9999029973809292;N_BI_GENOS=10309;East_Han_AN=6554;East_Han_AC=1;East_Han_AF=
chr3	113307597	.	T	<INS:ME:LINE1>	.	PASS	SVTYPE=INS:ME:LINE1;END=113307610;SVLEN=498;AN=20618;AC=1;AF=4.850130953535746e-05;N_HET=1;N_HOMALT=0;N_HOMREF=10308;FREQ_HET=9.700261907071492e-05;FREQ_HOMALT=0.0;FREQ_HOMREF=0.9999029973809292;N_BI_GENOS=10309;Central_Han_AN=2128;Central_Han_AC=1;Centra
chr3	113480912	.	A	<INS:ME:LINE1>	.	PASS	SVTYPE=INS:ME:LINE1;END=113480912;SVLEN=0;AN=20618;AC=5;AF=0.00024250654767678728;N_HET=1;N_HOMALT=2;N_HOMREF=10306;FREQ_HET=9.700261907071492e-05;FREQ_HOMALT=0.00019400523814142983;FREQ_HOMREF=0.9997089921427879;N_BI_GENOS=10309;Lingnan_Han_AN=1888;Lingn
chr3	113662617	.	A	<INS:ME:LINE1>	.	PASS	SVTYPE=INS:ME:LINE1;END=113662635;SVLEN=56;AN=20618;AC=1;AF=4.850130953535746e-05;N_HET=1;N_HOMALT=0;N_HOMREF=10308;FREQ_HET=9.700261907071492e-05;FREQ_HOMALT=0.0;FREQ_HOMREF=0.9999029973809292;N_BI_GENOS=10309;Central_Han_AN=2128;Central_Han_AC=1;Central
chr3	113886226	.	G	<INS:ME:LINE1>	.	PASS	SVTYPE=INS:ME:LINE1;END=113886240;SVLEN=6000;AN=20618;AC=1;AF=4.850130953535746e-05;N_HET=1;N_HOMALT=0;N_HOMREF=10308;FREQ_HET=9.700261907071492e-05;FREQ_HOMALT=0.0;FREQ_HOMREF=0.9999029973809292;N_BI_GENOS=10309;Lingnan_Han_AN=1888;Lingnan_Han_AC=1;Lingn
chr3	114220267	.	A	<INS:ME:LINE1>	.	PASS	SVTYPE=INS:ME:LINE1;END=114220284;SVLEN=5999;AN=20618;AC=1;AF=4.850130953535746e-05;N_HET=1;N_HOMALT=0;N_HOMREF=10308;FREQ_HET=9.700261907071492e-05;FREQ_HOMALT=0.0;FREQ_HOMREF=0.9999029973809292;N_BI_GENOS=10309;North_Han_AN=2272;North_Han_AC=1;North_Han
chr3	114446740	.	T	<INS:ME:LINE1>	.	PASS	SVTYPE=INS:ME:LINE1;END=114446757;SVLEN=1588;AN=20618;AC=1;AF=4.850130953535746e-05;N_HET=1;N_HOMALT=0;N_HOMREF=10308;FREQ_HET=9.700261907071492e-05;FREQ_HOMALT=0.0;FREQ_HOMREF=0.9999029973809292;N_BI_GENOS=10309;North_Han_AN=2272;North_Han_AC=1;North_Han
chr3	114575584	.	A	<INS:ME:LINE1>	.	PASS	SVTYPE=INS:ME:LINE1;END=114575584;SVLEN=4194;AN=20618;AC=3;AF=0.00014550392860607236;N_HET=3;N_HOMALT=0;N_HOMREF=10306;FREQ_HET=0.0002910078572121447;FREQ_HOMALT=0.0;FREQ_HOMREF=0.9997089921427879;N_BI_GENOS=10309;Northwest_Han_AN=1296;Northwest_Han_AC=1;
chr3	114665502	.	A	<INS:ME:LINE1>	.	PASS	SVTYPE=INS:ME:LINE1;END=114665502;SVLEN=3661;AN=20618;AC=8;AF=0.0003880104762828596;N_HET=8;N_HOMALT=0;N_HOMREF=10301;FREQ_HET=0.0007760209525657193;FREQ_HOMALT=0.0;FREQ_HOMREF=0.9992239790474343;N_BI_GENOS=10309;South_Han_AN=1496;South_Han_AC=1;South_Ha
chr3	114889070	.	A	<INS:ME:LINE1>	.	PASS	SVTYPE=INS:ME:LINE1;END=114889085;SVLEN=1308;AN=20618;AC=4;AF=0.00019400523814142983;N_HET=0;N_HOMALT=2;N_HOMREF=10307;FREQ_HET=0.0;FREQ_HOMALT=0.00019400523814142983;FREQ_HOMREF=0.9998059947618586;N_BI_GENOS=10309;Lingnan_Han_AN=1888;Lingnan_Han_AC=2;Lin
chr3	114969486	.	C	<INS:ME:LINE1>	.	PASS	SVTYPE=INS:ME:LINE1;END=114969498;SVLEN=2315;AN=20618;AC=6;AF=0.0002910078572121447;N_HET=6;N_HOMALT=0;N_HOMREF=10303;FREQ_HET=0.0005820157144242894;FREQ_HOMALT=0.0;FREQ_HOMREF=0.9994179842855757;N_BI_GENOS=10309;Northwest_Han_AN=1296;Northwest_Han_AC=1;N
chr3	114969746	.	A	<INS:ME:LINE1>	.	PASS	SVTYPE=INS:ME:LINE1;END=114969759;SVLEN=5998;AN=20618;AC=1;AF=4.850130953535746e-05;N_HET=1;N_HOMALT=0;N_HOMREF=10308;FREQ_HET=9.700261907071492e-05;FREQ_HOMALT=0.0;FREQ_HOMREF=0.9999029973809292;N_BI_GENOS=10309;Central_Han_AN=2128;Central_Han_AC=1;Centr
chr3	114983154	.	A	<INS:ME:LINE1>	.	PASS	SVTYPE=INS:ME:LINE1;END=114983154;SVLEN=862;AN=20618;AC=1;AF=4.850130953535746e-05;N_HET=1;N_HOMALT=0;N_HOMREF=10308;FREQ_HET=9.700261907071492e-05;FREQ_HOMALT=0.0;FREQ_HOMREF=0.9999029973809292;N_BI_GENOS=10309;East_Han_AN=6554;East_Han_AC=1;East_Han_AF=
chr3	115124528	.	A	<INS:ME:LINE1>	.	PASS	SVTYPE=INS:ME:LINE1;END=115124546;SVLEN=5997;AN=20618;AC=1;AF=4.850130953535746e-05;N_HET=1;N_HOMALT=0;N_HOMREF=10308;FREQ_HET=9.700261907071492e-05;FREQ_HOMALT=0.0;FREQ_HOMREF=0.9999029973809292;N_BI_GENOS=10309;East_Han_AN=6554;East_Han_AC=1;East_Han_AF
chr3	115262785	.	C	<INS:ME:LINE1>	.	PASS	SVTYPE=INS:ME:LINE1;END=115262798;SVLEN=5993;AN=20618;AC=2;AF=9.700261907071492e-05;N_HET=2;N_HOMALT=0;N_HOMREF=10307;FREQ_HET=0.00019400523814142983;FREQ_HOMALT=0.0;FREQ_HOMREF=0.9998059947618586;N_BI_GENOS=10309;East_Han_AN=6554;East_Han_AC=2;East_Han_A
chr3	115408427	.	A	<INS:ME:LINE1>	.	PASS	SVTYPE=INS:ME:LINE1;END=115408441;SVLEN=5999;AN=20618;AC=11;AF=0.000533514404888932;N_HET=11;N_HOMALT=0;N_HOMREF=10298;FREQ_HET=0.001067028809777864;FREQ_HOMALT=0.0;FREQ_HOMREF=0.9989329711902222;N_BI_GENOS=10309;South_Han_AN=1496;South_Han_AC=1;South_Han
chr3	115766377	.	A	<INS:ME:LINE1>	.	PASS	SVTYPE=INS:ME:LINE1;END=115766391;SVLEN=674;AN=20618;AC=1;AF=4.850130953535746e-05;N_HET=1;N_HOMALT=0;N_HOMREF=10308;FREQ_HET=9.700261907071492e-05;FREQ_HOMALT=0.0;FREQ_HOMREF=0.9999029973809292;N_BI_GENOS=10309;East_Han_AN=6554;East_Han_AC=1;East_Han_AF=
chr3	116115715	.	T	<INS:ME:LINE1>	.	PASS	SVTYPE=INS:ME:LINE1;END=116115731;SVLEN=6000;AN=20618;AC=12;AF=0.0005820157144242894;N_HET=12;N_HOMALT=0;N_HOMREF=10297;FREQ_HET=0.0011640314288485789;FREQ_HOMALT=0.0;FREQ_HOMREF=0.9988359685711514;N_BI_GENOS=10309;Northwest_Han_AN=1296;Northwest_Han_AC=2
chr3	116543262	.	T	<INS:ME:LINE1>	.	PASS	SVTYPE=INS:ME:LINE1;END=116543262;SVLEN=566;AN=20618;AC=1;AF=4.850130953535746e-05;N_HET=1;N_HOMALT=0;N_HOMREF=10308;FREQ_HET=9.700261907071492e-05;FREQ_HOMALT=0.0;FREQ_HOMREF=0.9999029973809292;N_BI_GENOS=10309;East_Han_AN=6554;East_Han_AC=1;East_Han_AF=
chr3	116649369	.	A	<INS:ME:LINE1>	.	PASS	SVTYPE=INS:ME:LINE1;END=116649384;SVLEN=610;AN=20618;AC=1;AF=4.850130953535746e-05;N_HET=1;N_HOMALT=0;N_HOMREF=10308;FREQ_HET=9.700261907071492e-05;FREQ_HOMALT=0.0;FREQ_HOMREF=0.9999029973809292;N_BI_GENOS=10309;North_Han_AN=2272;North_Han_AC=1;North_Han_
chr3	116687060	.	G	<INS:ME:LINE1>	.	PASS	SVTYPE=INS:ME:LINE1;END=116687072;SVLEN=47;AN=20618;AC=3;AF=0.00014550392860607236;N_HET=3;N_HOMALT=0;N_HOMREF=10306;FREQ_HET=0.0002910078572121447;FREQ_HOMALT=0.0;FREQ_HOMREF=0.9997089921427879;N_BI_GENOS=10309;South_Han_AN=1496;South_Han_AC=1;South_Han_
chr3	116717406	.	T	<INS:ME:LINE1>	.	PASS	SVTYPE=INS:ME:LINE1;END=116717425;SVLEN=73;AN=20618;AC=2;AF=9.700261907071492e-05;N_HET=2;N_HOMALT=0;N_HOMREF=10307;FREQ_HET=0.00019400523814142983;FREQ_HOMALT=0.0;FREQ_HOMREF=0.9998059947618586;N_BI_GENOS=10309;South_Han_AN=1496;South_Han_AC=1;South_Han_
chr3	116726583	.	G	<INS:ME:LINE1>	.	PASS	SVTYPE=INS:ME:LINE1;END=116726583;SVLEN=1034;AN=20618;AC=1;AF=4.850130953535746e-05;N_HET=1;N_HOMALT=0;N_HOMREF=10308;FREQ_HET=9.700261907071492e-05;FREQ_HOMALT=0.0;FREQ_HOMREF=0.9999029973809292;N_BI_GENOS=10309;Southeast_Han_AN=1908;Southeast_Han_AC=1;S
chr3	117050499	.	G	<INS:ME:LINE1>	.	PASS	SVTYPE=INS:ME:LINE1;END=117050499;SVLEN=0;AN=20618;AC=2;AF=9.700261907071492e-05;N_HET=0;N_HOMALT=1;N_HOMREF=10308;FREQ_HET=0.0;FREQ_HOMALT=9.700261907071492e-05;FREQ_HOMREF=0.9999029973809292;N_BI_GENOS=10309;South_Han_AN=1496;South_Han_AC=2;South_Han_AF
chr3	117224570	.	A	<INS:ME:LINE1>	.	PASS	SVTYPE=INS:ME:LINE1;END=117224580;SVLEN=5985;AN=20618;AC=1;AF=4.850130953535746e-05;N_HET=1;N_HOMALT=0;N_HOMREF=10308;FREQ_HET=9.700261907071492e-05;FREQ_HOMALT=0.0;FREQ_HOMREF=0.9999029973809292;N_BI_GENOS=10309;East_Han_AN=6554;East_Han_AC=1;East_Han_AF
chr3	117261110	.	A	<INS:ME:LINE1>	.	PASS	SVTYPE=INS:ME:LINE1;END=117261124;SVLEN=651;AN=20618;AC=5;AF=0.00024250654767678728;N_HET=5;N_HOMALT=0;N_HOMREF=10304;FREQ_HET=0.00048501309535357455;FREQ_HOMALT=0.0;FREQ_HOMREF=0.9995149869046465;N_BI_GENOS=10309;North_Han_AN=2272;North_Han_AC=2;North_Ha
chr3	117436967	.	A	<INS:ME:LINE1>	.	PASS	SVTYPE=INS:ME:LINE1;END=117436982;SVLEN=431;AN=20618;AC=3;AF=0.00014550392860607236;N_HET=3;N_HOMALT=0;N_HOMREF=10306;FREQ_HET=0.0002910078572121447;FREQ_HOMALT=0.0;FREQ_HOMREF=0.9997089921427879;N_BI_GENOS=10309;North_Han_AN=2272;North_Han_AC=1;North_Han
chr3	117719080	.	A	<INS:ME:LINE1>	.	PASS	SVTYPE=INS:ME:LINE1;END=117719097;SVLEN=1358;AN=20618;AC=1;AF=4.850130953535746e-05;N_HET=1;N_HOMALT=0;N_HOMREF=10308;FREQ_HET=9.700261907071492e-05;FREQ_HOMALT=0.0;FREQ_HOMREF=0.9999029973809292;N_BI_GENOS=10309;Lingnan_Han_AN=1888;Lingnan_Han_AC=1;Lingn

chr3	117858375	.	T	<INS:ME:LINE1>	.	PASS	SVTYPE=INS:ME:LINE1;END=117858381;SVLEN=1693;AN=20618;AC=1;AF=4.850130953535746e-05;N_HET=1;N_HOMALT=0;N_HOMREF=10308;FREQ_HET=9.700261907071492e-05;FREQ_HOMALT=0.0;FREQ_HOMREF=0.9999029973809292;N_BI_GENOS=10309;Northwest_Han_AN=1296;Northwest_Han_AC=1;N
chr3	117879772	.	T	<INS:ME:LINE1>	.	PASS	SVTYPE=INS:ME:LINE1;END=117879787;SVLEN=370;AN=20618;AC=1;AF=4.850130953535746e-05;N_HET=1;N_HOMALT=0;N_HOMREF=10308;FREQ_HET=9.700261907071492e-05;FREQ_HOMALT=0.0;FREQ_HOMREF=0.9999029973809292;N_BI_GENOS=10309;South_Han_AN=1496;South_Han_AC=1;South_Han_
chr3	117949671	.	A	<INS:ME:LINE1>	.	PASS	SVTYPE=INS:ME:LINE1;END=117949708;SVLEN=1596;AN=20618;AC=1;AF=4.850130953535746e-05;N_HET=1;N_HOMALT=0;N_HOMREF=10308;FREQ_HET=9.700261907071492e-05;FREQ_HOMALT=0.0;FREQ_HOMREF=0.9999029973809292;N_BI_GENOS=10309;East_Han_AN=6554;East_Han_AC=1;East_Han_AF
chr3	118022511	.	T	<INS:ME:LINE1>	.	PASS	SVTYPE=INS:ME:LINE1;END=118022513;SVLEN=4943;AN=20618;AC=1;AF=4.850130953535746e-05;N_HET=1;N_HOMALT=0;N_HOMREF=10308;FREQ_HET=9.700261907071492e-05;FREQ_HOMALT=0.0;FREQ_HOMREF=0.9999029973809292;N_BI_GENOS=10309;Northwest_Han_AN=1296;Northwest_Han_AC=1;N
chr3	118290650	.	T	<INS:ME:LINE1>	.	PASS	SVTYPE=INS:ME:LINE1;END=118290650;SVLEN=314;AN=20618;AC=1;AF=4.850130953535746e-05;N_HET=1;N_HOMALT=0;N_HOMREF=10308;FREQ_HET=9.700261907071492e-05;FREQ_HOMALT=0.0;FREQ_HOMREF=0.9999029973809292;N_BI_GENOS=10309;East_Han_AN=6554;East_Han_AC=1;East_Han_AF=
chr3	118404252	.	A	<INS:ME:LINE1>	.	PASS	SVTYPE=INS:ME:LINE1;END=118404265;SVLEN=858;AN=20618;AC=1;AF=4.850130953535746e-05;N_HET=1;N_HOMALT=0;N_HOMREF=10308;FREQ_HET=9.700261907071492e-05;FREQ_HOMALT=0.0;FREQ_HOMREF=0.9999029973809292;N_BI_GENOS=10309;Southeast_Han_AN=1908;Southeast_Han_AC=1;So
chr3	118834963	.	A	<INS:ME:LINE1>	.	PASS	SVTYPE=INS:ME:LINE1;END=118834977;SVLEN=6004;AN=20618;AC=6;AF=0.0002910078572121447;N_HET=6;N_HOMALT=0;N_HOMREF=10303;FREQ_HET=0.0005820157144242894;FREQ_HOMALT=0.0;FREQ_HOMREF=0.9994179842855757;N_BI_GENOS=10309;Northwest_Han_AN=1296;Northwest_Han_AC=1;N
chr3	118884934	.	A	<INS:ME:LINE1>	.	PASS	SVTYPE=INS:ME:LINE1;END=118884948;SVLEN=993;AN=20618;AC=1;AF=4.850130953535746e-05;N_HET=1;N_HOMALT=0;N_HOMREF=10308;FREQ_HET=9.700261907071492e-05;FREQ_HOMALT=0.0;FREQ_HOMREF=0.9999029973809292;N_BI_GENOS=10309;East_Han_AN=6554;East_Han_AC=1;East_Han_AF=
chr3	118890449	.	C	<INS:ME:LINE1>	.	PASS	SVTYPE=INS:ME:LINE1;END=118890465;SVLEN=6055;AN=20618;AC=1;AF=4.850130953535746e-05;N_HET=1;N_HOMALT=0;N_HOMREF=10308;FREQ_HET=9.700261907071492e-05;FREQ_HOMALT=0.0;FREQ_HOMREF=0.9999029973809292;N_BI_GENOS=10309;Northwest_Han_AN=1296;Northwest_Han_AC=1;N
chr3	119359860	.	A	<INS:ME:LINE1>	.	PASS	SVTYPE=INS:ME:LINE1;END=119359876;SVLEN=5446;AN=20618;AC=3;AF=0.00014550392860607236;N_HET=3;N_HOMALT=0;N_HOMREF=10306;FREQ_HET=0.0002910078572121447;FREQ_HOMALT=0.0;FREQ_HOMREF=0.9997089921427879;N_BI_GENOS=10309;North_Han_AN=2272;North_Han_AC=1;North_Ha
chr3	119514499	.	A	<INS:ME:LINE1>	.	PASS	SVTYPE=INS:ME:LINE1;END=119514514;SVLEN=5996;AN=20618;AC=1;AF=4.850130953535746e-05;N_HET=1;N_HOMALT=0;N_HOMREF=10308;FREQ_HET=9.700261907071492e-05;FREQ_HOMALT=0.0;FREQ_HOMREF=0.9999029973809292;N_BI_GENOS=10309;Southeast_Han_AN=1908;Southeast_Han_AC=1;S
chr3	119842326	.	G	<INS:ME:LINE1>	.	PASS	SVTYPE=INS:ME:LINE1;END=119842326;SVLEN=2110;AN=20618;AC=1;AF=4.850130953535746e-05;N_HET=1;N_HOMALT=0;N_HOMREF=10308;FREQ_HET=9.700261907071492e-05;FREQ_HOMALT=0.0;FREQ_HOMREF=0.9999029973809292;N_BI_GENOS=10309;East_Han_AN=6554;East_Han_AC=1;East_Han_AF
chr3	120285804	.	A	<INS:ME:LINE1>	.	PASS	SVTYPE=INS:ME:LINE1;END=120285820;SVLEN=652;AN=20618;AC=7;AF=0.0003395091667475022;N_HET=7;N_HOMALT=0;N_HOMREF=10302;FREQ_HET=0.0006790183334950044;FREQ_HOMALT=0.0;FREQ_HOMREF=0.999320981666505;N_BI_GENOS=10309;North_Han_AN=2272;North_Han_AC=1;North_Han_A
chr3	120587130	.	G	<INS:ME:LINE1>	.	PASS	SVTYPE=INS:ME:LINE1;END=120587145;SVLEN=816;AN=20618;AC=5;AF=0.00024250654767678728;N_HET=3;N_HOMALT=1;N_HOMREF=10305;FREQ_HET=0.0002910078572121447;FREQ_HOMALT=9.700261907071492e-05;FREQ_HOMREF=0.9996119895237171;N_BI_GENOS=10309;Hui_AN=444;Hui_AC=1;Hui_
chr3	120875537	.	A	<INS:ME:LINE1>	.	PASS	SVTYPE=INS:ME:LINE1;END=120875552;SVLEN=5998;AN=20618;AC=3;AF=0.00014550392860607236;N_HET=3;N_HOMALT=0;N_HOMREF=10306;FREQ_HET=0.0002910078572121447;FREQ_HOMALT=0.0;FREQ_HOMREF=0.9997089921427879;N_BI_GENOS=10309;Lingnan_Han_AN=1888;Lingnan_Han_AC=1;Ling
chr3	120921247	.	A	<INS:ME:LINE1>	.	PASS	SVTYPE=INS:ME:LINE1;END=120921247;SVLEN=194;AN=20618;AC=1;AF=4.850130953535746e-05;N_HET=1;N_HOMALT=0;N_HOMREF=10308;FREQ_HET=9.700261907071492e-05;FREQ_HOMALT=0.0;FREQ_HOMREF=0.9999029973809292;N_BI_GENOS=10309;East_Han_AN=6554;East_Han_AC=1;East_Han_AF=
chr3	121024825	.	A	<INS:ME:LINE1>	.	PASS	SVTYPE=INS:ME:LINE1;END=121024838;SVLEN=103;AN=20618;AC=2;AF=9.700261907071492e-05;N_HET=2;N_HOMALT=0;N_HOMREF=10307;FREQ_HET=0.00019400523814142983;FREQ_HOMALT=0.0;FREQ_HOMREF=0.9998059947618586;N_BI_GENOS=10309;East_Han_AN=6554;East_Han_AC=1;East_Han_AF
chr3	121068816	.	G	<INS:ME:LINE1>	.	PASS	SVTYPE=INS:ME:LINE1;END=121068830;SVLEN=6002;AN=20618;AC=1;AF=4.850130953535746e-05;N_HET=1;N_HOMALT=0;N_HOMREF=10308;FREQ_HET=9.700261907071492e-05;FREQ_HOMALT=0.0;FREQ_HOMREF=0.9999029973809292;N_BI_GENOS=10309;South_Han_AN=1496;South_Han_AC=1;South_Han
chr3	121086742	.	A	<INS:ME:LINE1>	.	PASS	SVTYPE=INS:ME:LINE1;END=121086759;SVLEN=605;AN=20618;AC=1;AF=4.850130953535746e-05;N_HET=1;N_HOMALT=0;N_HOMREF=10308;FREQ_HET=9.700261907071492e-05;FREQ_HOMALT=0.0;FREQ_HOMREF=0.9999029973809292;N_BI_GENOS=10309;East_Han_AN=6554;East_Han_AC=1;East_Han_AF=
chr3	121090589	.	T	<INS:ME:LINE1>	.	PASS	SVTYPE=INS:ME:LINE1;END=121090604;SVLEN=5997;AN=20618;AC=1;AF=4.850130953535746e-05;N_HET=1;N_HOMALT=0;N_HOMREF=10308;FREQ_HET=9.700261907071492e-05;FREQ_HOMALT=0.0;FREQ_HOMREF=0.9999029973809292;N_BI_GENOS=10309;Southeast_Han_AN=1908;Southeast_Han_AC=1;S
chr3	121220220	.	A	<INS:ME:LINE1>	.	PASS	SVTYPE=INS:ME:LINE1;END=121220237;SVLEN=1377;AN=20618;AC=1;AF=4.850130953535746e-05;N_HET=1;N_HOMALT=0;N_HOMREF=10308;FREQ_HET=9.700261907071492e-05;FREQ_HOMALT=0.0;FREQ_HOMREF=0.9999029973809292;N_BI_GENOS=10309;South_Han_AN=1496;South_Han_AC=1;South_Han
chr3	121894873	.	T	<INS:ME:LINE1>	.	PASS	SVTYPE=INS:ME:LINE1;END=121894887;SVLEN=1257;AN=20618;AC=2;AF=9.700261907071492e-05;N_HET=2;N_HOMALT=0;N_HOMREF=10307;FREQ_HET=0.00019400523814142983;FREQ_HOMALT=0.0;FREQ_HOMREF=0.9998059947618586;N_BI_GENOS=10309;Lingnan_Han_AN=1888;Lingnan_Han_AC=1;Ling
chr3	122207815	.	A	<INS:ME:LINE1>	.	PASS	SVTYPE=INS:ME:LINE1;END=122207827;SVLEN=1866;AN=20618;AC=2;AF=9.700261907071492e-05;N_HET=2;N_HOMALT=0;N_HOMREF=10307;FREQ_HET=0.00019400523814142983;FREQ_HOMALT=0.0;FREQ_HOMREF=0.9998059947618586;N_BI_GENOS=10309;East_Han_AN=6554;East_Han_AC=2;East_Han_A
chr3	122726900	.	A	<INS:ME:LINE1>	.	PASS	SVTYPE=INS:ME:LINE1;END=122726900;SVLEN=5;AN=20618;AC=1;AF=4.850130953535746e-05;N_HET=1;N_HOMALT=0;N_HOMREF=10308;FREQ_HET=9.700261907071492e-05;FREQ_HOMALT=0.0;FREQ_HOMREF=0.9999029973809292;N_BI_GENOS=10309;Hui_AN=444;Hui_AC=1;Hui_AF=0.0022522522522522
chr3	122832929	.	A	<INS:ME:LINE1>	.	PASS	SVTYPE=INS:ME:LINE1;END=122832929;SVLEN=277;AN=20618;AC=1;AF=4.850130953535746e-05;N_HET=1;N_HOMALT=0;N_HOMREF=10308;FREQ_HET=9.700261907071492e-05;FREQ_HOMALT=0.0;FREQ_HOMREF=0.9999029973809292;N_BI_GENOS=10309;North_Han_AN=2272;North_Han_AC=1;North_Han_
chr3	122844458	.	C	<INS:ME:LINE1>	.	PASS	SVTYPE=INS:ME:LINE1;END=122844473;SVLEN=1540;AN=20618;AC=1;AF=4.850130953535746e-05;N_HET=1;N_HOMALT=0;N_HOMREF=10308;FREQ_HET=9.700261907071492e-05;FREQ_HOMALT=0.0;FREQ_HOMREF=0.9999029973809292;N_BI_GENOS=10309;North_Han_AN=2272;North_Han_AC=1;North_Han
chr3	123299235	.	T	<INS:ME:LINE1>	.	PASS	SVTYPE=INS:ME:LINE1;END=123299244;SVLEN=847;AN=20618;AC=1;AF=4.850130953535746e-05;N_HET=1;N_HOMALT=0;N_HOMREF=10308;FREQ_HET=9.700261907071492e-05;FREQ_HOMALT=0.0;FREQ_HOMREF=0.9999029973809292;N_BI_GENOS=10309;Southeast_Han_AN=1908;Southeast_Han_AC=1;So
chr3	123400650	.	A	<INS:ME:LINE1>	.	PASS	SVTYPE=INS:ME:LINE1;END=123400664;SVLEN=5994;AN=20618;AC=1;AF=4.850130953535746e-05;N_HET=1;N_HOMALT=0;N_HOMREF=10308;FREQ_HET=9.700261907071492e-05;FREQ_HOMALT=0.0;FREQ_HOMREF=0.9999029973809292;N_BI_GENOS=10309;Central_Han_AN=2128;Central_Han_AC=1;Centr
chr3	123547973	.	A	<INS:ME:LINE1>	.	PASS	SVTYPE=INS:ME:LINE1;END=123547988;SVLEN=3807;AN=20618;AC=1;AF=4.850130953535746e-05;N_HET=1;N_HOMALT=0;N_HOMREF=10308;FREQ_HET=9.700261907071492e-05;FREQ_HOMALT=0.0;FREQ_HOMREF=0.9999029973809292;N_BI_GENOS=10309;Southeast_Han_AN=1908;Southeast_Han_AC=1;S
chr3	123871865	.	G	<INS:ME:LINE1>	.	PASS	SVTYPE=INS:ME:LINE1;END=123871879;SVLEN=5999;AN=20618;AC=845;AF=0.040983606557377046;N_HET=737;N_HOMALT=54;N_HOMREF=9518;FREQ_HET=0.0714909302551169;FREQ_HOMALT=0.005238141429818605;FREQ_HOMREF=0.9232709283150645;N_BI_GENOS=10309;South_Han_AN=1496;South_H
chr3	123970713	.	T	<INS:ME:LINE1>	.	PASS	SVTYPE=INS:ME:LINE1;END=123970728;SVLEN=512;AN=20618;AC=14;AF=0.0006790183334950044;N_HET=14;N_HOMALT=0;N_HOMREF=10295;FREQ_HET=0.0013580366669900088;FREQ_HOMALT=0.0;FREQ_HOMREF=0.99864196333301;N_BI_GENOS=10309;South_Han_AN=1496;South_Han_AC=1;South_Han_
chr3	124657262	.	A	<INS:ME:LINE1>	.	PASS	SVTYPE=INS:ME:LINE1;END=124657273;SVLEN=6006;AN=20618;AC=1;AF=4.850130953535746e-05;N_HET=1;N_HOMALT=0;N_HOMREF=10308;FREQ_HET=9.700261907071492e-05;FREQ_HOMALT=0.0;FREQ_HOMREF=0.9999029973809292;N_BI_GENOS=10309;East_Han_AN=6554;East_Han_AC=1;East_Han_AF
chr3	124756899	.	C	<INS:ME:LINE1>	.	PASS	SVTYPE=INS:ME:LINE1;END=124756899;SVLEN=0;AN=20618;AC=1;AF=4.850130953535746e-05;N_HET=1;N_HOMALT=0;N_HOMREF=10308;FREQ_HET=9.700261907071492e-05;FREQ_HOMALT=0.0;FREQ_HOMREF=0.9999029973809292;N_BI_GENOS=10309;East_Han_AN=6554;East_Han_AC=1;East_Han_AF=0.
chr3	125024148	.	A	<INS:ME:LINE1>	.	PASS	SVTYPE=INS:ME:LINE1;END=125024167;SVLEN=398;AN=20618;AC=1;AF=4.850130953535746e-05;N_HET=1;N_HOMALT=0;N_HOMREF=10308;FREQ_HET=9.700261907071492e-05;FREQ_HOMALT=0.0;FREQ_HOMREF=0.9999029973809292;N_BI_GENOS=10309;East_Han_AN=6554;East_Han_AC=1;East_Han_AF=
chr3	126230493	.	C	<INS:ME:LINE1>	.	PASS	SVTYPE=INS:ME:LINE1;END=126230493;SVLEN=118;AN=20618;AC=1;AF=4.850130953535746e-05;N_HET=1;N_HOMALT=0;N_HOMREF=10308;FREQ_HET=9.700261907071492e-05;FREQ_HOMALT=0.0;FREQ_HOMREF=0.9999029973809292;N_BI_GENOS=10309;East_Han_AN=6554;East_Han_AC=1;East_Han_AF=
chr3	126606912	.	A	<INS:ME:LINE1>	.	PASS	SVTYPE=INS:ME:LINE1;END=126606928;SVLEN=4047;AN=20618;AC=1;AF=4.850130953535746e-05;N_HET=1;N_HOMALT=0;N_HOMREF=10308;FREQ_HET=9.700261907071492e-05;FREQ_HOMALT=0.0;FREQ_HOMREF=0.9999029973809292;N_BI_GENOS=10309;North_Han_AN=2272;North_Han_AC=1;North_Han
chr3	126903509	.	A	<INS:ME:LINE1>	.	PASS	SVTYPE=INS:ME:LINE1;END=126903524;SVLEN=5955;AN=20618;AC=2;AF=9.700261907071492e-05;N_HET=2;N_HOMALT=0;N_HOMREF=10307;FREQ_HET=0.00019400523814142983;FREQ_HOMALT=0.0;FREQ_HOMREF=0.9998059947618586;N_BI_GENOS=10309;Central_Han_AN=2128;Central_Han_AC=1;Cent
chr3	127126547	.	A	<INS:ME:LINE1>	.	PASS	SVTYPE=INS:ME:LINE1;END=127126557;SVLEN=6008;AN=20618;AC=1;AF=4.850130953535746e-05;N_HET=1;N_HOMALT=0;N_HOMREF=10308;FREQ_HET=9.700261907071492e-05;FREQ_HOMALT=0.0;FREQ_HOMREF=0.9999029973809292;N_BI_GENOS=10309;East_Han_AN=6554;East_Han_AC=1;East_Han_AF
chr3	127826629	.	G	<INS:ME:LINE1>	.	PASS	SVTYPE=INS:ME:LINE1;END=127826641;SVLEN=5997;AN=20618;AC=6;AF=0.0002910078572121447;N_HET=6;N_HOMALT=0;N_HOMREF=10303;FREQ_HET=0.0005820157144242894;FREQ_HOMALT=0.0;FREQ_HOMREF=0.9994179842855757;N_BI_GENOS=10309;North_Han_AN=2272;North_Han_AC=1;North_Han
chr3	127952450	.	A	<INS:ME:LINE1>	.	PASS	SVTYPE=INS:ME:LINE1;END=127952465;SVLEN=5999;AN=20618;AC=22;AF=0.001067028809777864;N_HET=22;N_HOMALT=0;N_HOMREF=10287;FREQ_HET=0.002134057619555728;FREQ_HOMALT=0.0;FREQ_HOMREF=0.9978659423804442;N_BI_GENOS=10309;Lingnan_Han_AN=1888;Lingnan_Han_AC=4;Lingn
chr3	128513652	.	A	<INS:ME:LINE1>	.	PASS	SVTYPE=INS:ME:LINE1;END=128513652;SVLEN=15;AN=20618;AC=1;AF=4.850130953535746e-05;N_HET=1;N_HOMALT=0;N_HOMREF=10308;FREQ_HET=9.700261907071492e-05;FREQ_HOMALT=0.0;FREQ_HOMREF=0.9999029973809292;N_BI_GENOS=10309;South_Han_AN=1496;South_Han_AC=1;South_Han_A
chr3	129110674	.	A	<INS:ME:LINE1>	.	PASS	SVTYPE=INS:ME:LINE1;END=129110688;SVLEN=535;AN=20618;AC=1;AF=4.850130953535746e-05;N_HET=1;N_HOMALT=0;N_HOMREF=10308;FREQ_HET=9.700261907071492e-05;FREQ_HOMALT=0.0;FREQ_HOMREF=0.9999029973809292;N_BI_GENOS=10309;Northwest_Han_AN=1296;Northwest_Han_AC=1;No
chr3	129775735	.	T	<INS:ME:LINE1>	.	PASS	SVTYPE=INS:ME:LINE1;END=129775736;SVLEN=806;AN=20618;AC=1;AF=4.850130953535746e-05;N_HET=1;N_HOMALT=0;N_HOMREF=10308;FREQ_HET=9.700261907071492e-05;FREQ_HOMALT=0.0;FREQ_HOMREF=0.9999029973809292;N_BI_GENOS=10309;South_Han_AN=1496;South_Han_AC=1;South_Han_
chr3	130015939	.	C	<INS:ME:LINE1>	.	PASS	SVTYPE=INS:ME:LINE1;END=130015939;SVLEN=0;AN=20618;AC=20;AF=0.0009700261907071491;N_HET=18;N_HOMALT=1;N_HOMREF=10290;FREQ_HET=0.0017460471432728683;FREQ_HOMALT=9.700261907071492e-05;FREQ_HOMREF=0.9981569502376564;N_BI_GENOS=10309;Northwest_Han_AN=1296;Nor
chr3	130038611	.	C	<INS:ME:LINE1>	.	PASS	SVTYPE=INS:ME:LINE1;END=130038611;SVLEN=0;AN=20618;AC=1;AF=4.850130953535746e-05;N_HET=1;N_HOMALT=0;N_HOMREF=10308;FREQ_HET=9.700261907071492e-05;FREQ_HOMALT=0.0;FREQ_HOMREF=0.9999029973809292;N_BI_GENOS=10309;Lingnan_Han_AN=1888;Lingnan_Han_AC=1;Lingnan_
chr3	130124204	.	C	<INS:ME:LINE1>	.	PASS	SVTYPE=INS:ME:LINE1;END=130124204;SVLEN=0;AN=20618;AC=1;AF=4.850130953535746e-05;N_HET=1;N_HOMALT=0;N_HOMREF=10308;FREQ_HET=9.700261907071492e-05;FREQ_HOMALT=0.0;FREQ_HOMREF=0.9999029973809292;N_BI_GENOS=10309;East_Han_AN=6554;East_Han_AC=1;East_Han_AF=0.
chr3	130124318	.	G	<INS:ME:LINE1>	.	PASS	SVTYPE=INS:ME:LINE1;END=130124318;SVLEN=0;AN=20618;AC=2;AF=9.700261907071492e-05;N_HET=2;N_HOMALT=0;N_HOMREF=10307;FREQ_HET=0.00019400523814142983;FREQ_HOMALT=0.0;FREQ_HOMREF=0.9998059947618586;N_BI_GENOS=10309;Northwest_Han_AN=1296;Northwest_Han_AC=1;Nor
chr3	130125080	.	T	<INS:ME:LINE1>	.	PASS	SVTYPE=INS:ME:LINE1;END=130125080;SVLEN=0;AN=20618;AC=1;AF=4.850130953535746e-05;N_HET=1;N_HOMALT=0;N_HOMREF=10308;FREQ_HET=9.700261907071492e-05;FREQ_HOMALT=0.0;FREQ_HOMREF=0.9999029973809292;N_BI_GENOS=10309;Northwest_Han_AN=1296;Northwest_Han_AC=1;Nort
chr3	130271765	.	C	<INS:ME:LINE1>	.	PASS	SVTYPE=INS:ME:LINE1;END=130271775;SVLEN=526;AN=20618;AC=2;AF=9.700261907071492e-05;N_HET=2;N_HOMALT=0;N_HOMREF=10307;FREQ_HET=0.00019400523814142983;FREQ_HOMALT=0.0;FREQ_HOMREF=0.9998059947618586;N_BI_GENOS=10309;East_Han_AN=6554;East_Han_AC=1;East_Han_AF
chr3	130431000	.	C	<INS:ME:LINE1>	.	PASS	SVTYPE=INS:ME:LINE1;END=130431001;SVLEN=364;AN=20618;AC=1;AF=4.850130953535746e-05;N_HET=1;N_HOMALT=0;N_HOMREF=10308;FREQ_HET=9.700261907071492e-05;FREQ_HOMALT=0.0;FREQ_HOMREF=0.9999029973809292;N_BI_GENOS=10309;East_Han_AN=6554;East_Han_AC=1;East_Han_AF=
chr3	130484675	.	T	<INS:ME:LINE1>	.	PASS	SVTYPE=INS:ME:LINE1;END=130484692;SVLEN=153;AN=20618;AC=15;AF=0.0007275196430303619;N_HET=15;N_HOMALT=0;N_HOMREF=10294;FREQ_HET=0.0014550392860607237;FREQ_HOMALT=0.0;FREQ_HOMREF=0.9985449607139393;N_BI_GENOS=10309;Northwest_Han_AN=1296;Northwest_Han_AC=1;
chr3	130507506	.	A	<INS:ME:LINE1>	.	PASS	SVTYPE=INS:ME:LINE1;END=130507517;SVLEN=1363;AN=20618;AC=1;AF=4.850130953535746e-05;N_HET=1;N_HOMALT=0;N_HOMREF=10308;FREQ_HET=9.700261907071492e-05;FREQ_HOMALT=0.0;FREQ_HOMREF=0.9999029973809292;N_BI_GENOS=10309;Central_Han_AN=2128;Central_Han_AC=1;Centr
chr3	130551118	.	C	<INS:ME:LINE1>	.	PASS	SVTYPE=INS:ME:LINE1;END=130551150;SVLEN=3118;AN=20618;AC=1;AF=4.850130953535746e-05;N_HET=1;N_HOMALT=0;N_HOMREF=10308;FREQ_HET=9.700261907071492e-05;FREQ_HOMALT=0.0;FREQ_HOMREF=0.9999029973809292;N_BI_GENOS=10309;East_Han_AN=6554;East_Han_AC=1;East_Han_AF
chr3	130570128	.	A	<INS:ME:LINE1>	.	PASS	SVTYPE=INS:ME:LINE1;END=130570142;SVLEN=6000;AN=20618;AC=1;AF=4.850130953535746e-05;N_HET=1;N_HOMALT=0;N_HOMREF=10308;FREQ_HET=9.700261907071492e-05;FREQ_HOMALT=0.0;FREQ_HOMREF=0.9999029973809292;N_BI_GENOS=10309;East_Han_AN=6554;East_Han_AC=1;East_Han_AF
chr3	130586182	.	T	<INS:ME:LINE1>	.	PASS	SVTYPE=INS:ME:LINE1;END=130586189;SVLEN=817;AN=20618;AC=1;AF=4.850130953535746e-05;N_HET=1;N_HOMALT=0;N_HOMREF=10308;FREQ_HET=9.700261907071492e-05;FREQ_HOMALT=0.0;FREQ_HOMREF=0.9999029973809292;N_BI_GENOS=10309;East_Han_AN=6554;East_Han_AC=1;East_Han_AF=
chr3	130669034	.	A	<INS:ME:LINE1>	.	PASS	SVTYPE=INS:ME:LINE1;END=130669051;SVLEN=6082;AN=20618;AC=1;AF=4.850130953535746e-05;N_HET=1;N_HOMALT=0;N_HOMREF=10308;FREQ_HET=9.700261907071492e-05;FREQ_HOMALT=0.0;FREQ_HOMREF=0.9999029973809292;N_BI_GENOS=10309;East_Han_AN=6554;East_Han_AC=1;East_Han_AF
chr3	130759983	.	A	<INS:ME:LINE1>	.	PASS	SVTYPE=INS:ME:LINE1;END=130759983;SVLEN=448;AN=20618;AC=1;AF=4.850130953535746e-05;N_HET=1;N_HOMALT=0;N_HOMREF=10308;FREQ_HET=9.700261907071492e-05;FREQ_HOMALT=0.0;FREQ_HOMREF=0.9999029973809292;N_BI_GENOS=10309;North_Han_AN=2272;North_Han_AC=1;North_Han_
chr3	131110534	.	T	<INS:ME:LINE1>	.	PASS	SVTYPE=INS:ME:LINE1;END=131110537;SVLEN=433;AN=20618;AC=1;AF=4.850130953535746e-05;N_HET=1;N_HOMALT=0;N_HOMREF=10308;FREQ_HET=9.700261907071492e-05;FREQ_HOMALT=0.0;FREQ_HOMREF=0.9999029973809292;N_BI_GENOS=10309;North_Han_AN=2272;North_Han_AC=1;North_Han_
chr3	131193827	.	G	<INS:ME:LINE1>	.	PASS	SVTYPE=INS:ME:LINE1;END=131193837;SVLEN=6001;AN=20618;AC=1;AF=4.850130953535746e-05;N_HET=1;N_HOMALT=0;N_HOMREF=10308;FREQ_HET=9.700261907071492e-05;FREQ_HOMALT=0.0;FREQ_HOMREF=0.9999029973809292;N_BI_GENOS=10309;Central_Han_AN=2128;Central_Han_AC=1;Centr
chr3	131254521	.	G	<INS:ME:LINE1>	.	PASS	SVTYPE=INS:ME:LINE1;END=131254538;SVLEN=196;AN=20618;AC=1;AF=4.850130953535746e-05;N_HET=1;N_HOMALT=0;N_HOMREF=10308;FREQ_HET=9.700261907071492e-05;FREQ_HOMALT=0.0;FREQ_HOMREF=0.9999029973809292;N_BI_GENOS=10309;Southeast_Han_AN=1908;Southeast_Han_AC=1;So
chr3	131300488	.	C	<INS:ME:LINE1>	.	PASS	SVTYPE=INS:ME:LINE1;END=131300499;SVLEN=1137;AN=20618;AC=1;AF=4.850130953535746e-05;N_HET=1;N_HOMALT=0;N_HOMREF=10308;FREQ_HET=9.700261907071492e-05;FREQ_HOMALT=0.0;FREQ_HOMREF=0.9999029973809292;N_BI_GENOS=10309;East_Han_AN=6554;East_Han_AC=1;East_Han_AF
chr3	131300713	.	C	<INS:ME:LINE1>	.	PASS	SVTYPE=INS:ME:LINE1;END=131300713;SVLEN=121;AN=20618;AC=1;AF=4.850130953535746e-05;N_HET=1;N_HOMALT=0;N_HOMREF=10308;FREQ_HET=9.700261907071492e-05;FREQ_HOMALT=0.0;FREQ_HOMREF=0.9999029973809292;N_BI_GENOS=10309;East_Han_AN=6554;East_Han_AC=1;East_Han_AF=
chr3	131609588	.	A	<INS:ME:LINE1>	.	PASS	SVTYPE=INS:ME:LINE1;END=131609603;SVLEN=5998;AN=20618;AC=1;AF=4.850130953535746e-05;N_HET=1;N_HOMALT=0;N_HOMREF=10308;FREQ_HET=9.700261907071492e-05;FREQ_HOMALT=0.0;FREQ_HOMREF=0.9999029973809292;N_BI_GENOS=10309;East_Han_AN=6554;East_Han_AC=1;East_Han_AF
chr3	131613938	.	A	<INS:ME:LINE1>	.	PASS	SVTYPE=INS:ME:LINE1;END=131613952;SVLEN=660;AN=20618;AC=3;AF=0.00014550392860607236;N_HET=3;N_HOMALT=0;N_HOMREF=10306;FREQ_HET=0.0002910078572121447;FREQ_HOMALT=0.0;FREQ_HOMREF=0.9997089921427879;N_BI_GENOS=10309;North_Han_AN=2272;North_Han_AC=1;North_Han
chr3	131810472	.	A	<INS:ME:LINE1>	.	PASS	SVTYPE=INS:ME:LINE1;END=131810472;SVLEN=355;AN=20618;AC=1;AF=4.850130953535746e-05;N_HET=1;N_HOMALT=0;N_HOMREF=10308;FREQ_HET=9.700261907071492e-05;FREQ_HOMALT=0.0;FREQ_HOMREF=0.9999029973809292;N_BI_GENOS=10309;East_Han_AN=6554;East_Han_AC=1;East_Han_AF=
chr3	131817992	.	T	<INS:ME:LINE1>	.	PASS	SVTYPE=INS:ME:LINE1;END=131817992;SVLEN=2159;AN=20618;AC=1;AF=4.850130953535746e-05;N_HET=1;N_HOMALT=0;N_HOMREF=10308;FREQ_HET=9.700261907071492e-05;FREQ_HOMALT=0.0;FREQ_HOMREF=0.9999029973809292;N_BI_GENOS=10309;East_Han_AN=6554;East_Han_AC=1;East_Han_AF
chr3	132109074	.	A	<INS:ME:LINE1>	.	PASS	SVTYPE=INS:ME:LINE1;END=132109074;SVLEN=728;AN=20618;AC=1;AF=4.850130953535746e-05;N_HET=1;N_HOMALT=0;N_HOMREF=10308;FREQ_HET=9.700261907071492e-05;FREQ_HOMALT=0.0;FREQ_HOMREF=0.9999029973809292;N_BI_GENOS=10309;East_Han_AN=6554;East_Han_AC=1;East_Han_AF=
chr3	132135723	.	A	<INS:ME:LINE1>	.	PASS	SVTYPE=INS:ME:LINE1;END=132135728;SVLEN=353;AN=20618;AC=10;AF=0.00048501309535357455;N_HET=10;N_HOMALT=0;N_HOMREF=10299;FREQ_HET=0.0009700261907071491;FREQ_HOMALT=0.0;FREQ_HOMREF=0.9990299738092928;N_BI_GENOS=10309;Northwest_Han_AN=1296;Northwest_Han_AC=1
chr3	132928631	.	A	<INS:ME:LINE1>	.	PASS	SVTYPE=INS:ME:LINE1;END=132928641;SVLEN=977;AN=20618;AC=2;AF=9.700261907071492e-05;N_HET=2;N_HOMALT=0;N_HOMREF=10307;FREQ_HET=0.00019400523814142983;FREQ_HOMALT=0.0;FREQ_HOMREF=0.9998059947618586;N_BI_GENOS=10309;Northwest_Han_AN=1296;Northwest_Han_AC=1;N
chr3	133069141	.	A	<INS:ME:LINE1>	.	PASS	SVTYPE=INS:ME:LINE1;END=133069156;SVLEN=456;AN=20618;AC=5;AF=0.00024250654767678728;N_HET=5;N_HOMALT=0;N_HOMREF=10304;FREQ_HET=0.00048501309535357455;FREQ_HOMALT=0.0;FREQ_HOMREF=0.9995149869046465;N_BI_GENOS=10309;Lingnan_Han_AN=1888;Lingnan_Han_AC=1;Ling
chr3	133217206	.	A	<INS:ME:LINE1>	.	PASS	SVTYPE=INS:ME:LINE1;END=133217206;SVLEN=881;AN=20618;AC=1;AF=4.850130953535746e-05;N_HET=1;N_HOMALT=0;N_HOMREF=10308;FREQ_HET=9.700261907071492e-05;FREQ_HOMALT=0.0;FREQ_HOMREF=0.9999029973809292;N_BI_GENOS=10309;East_Han_AN=6554;East_Han_AC=1;East_Han_AF=
chr3	133521596	.	A	<INS:ME:LINE1>	.	PASS	SVTYPE=INS:ME:LINE1;END=133521612;SVLEN=5997;AN=20618;AC=1;AF=4.850130953535746e-05;N_HET=1;N_HOMALT=0;N_HOMREF=10308;FREQ_HET=9.700261907071492e-05;FREQ_HOMALT=0.0;FREQ_HOMREF=0.9999029973809292;N_BI_GENOS=10309;East_Han_AN=6554;East_Han_AC=1;East_Han_AF
chr3	134119019	.	G	<INS:ME:LINE1>	.	PASS	SVTYPE=INS:ME:LINE1;END=134119019;SVLEN=350;AN=20618;AC=1;AF=4.850130953535746e-05;N_HET=1;N_HOMALT=0;N_HOMREF=10308;FREQ_HET=9.700261907071492e-05;FREQ_HOMALT=0.0;FREQ_HOMREF=0.9999029973809292;N_BI_GENOS=10309;East_Han_AN=6554;East_Han_AC=1;East_Han_AF=
chr3	134192441	.	A	<INS:ME:LINE1>	.	PASS	SVTYPE=INS:ME:LINE1;END=134192456;SVLEN=413;AN=20618;AC=862;AF=0.041808128819478124;N_HET=728;N_HOMALT=67;N_HOMREF=9514;FREQ_HET=0.07061790668348046;FREQ_HOMALT=0.006499175477737899;FREQ_HOMREF=0.9228829178387816;N_BI_GENOS=10309;South_Han_AN=1496;South_H
chr3	134502596	.	C	<INS:ME:LINE1>	.	PASS	SVTYPE=INS:ME:LINE1;END=134502596;SVLEN=1252;AN=20618;AC=1;AF=4.850130953535746e-05;N_HET=1;N_HOMALT=0;N_HOMREF=10308;FREQ_HET=9.700261907071492e-05;FREQ_HOMALT=0.0;FREQ_HOMREF=0.9999029973809292;N_BI_GENOS=10309;East_Han_AN=6554;East_Han_AC=1;East_Han_AF
chr3	135088155	.	G	<INS:ME:LINE1>	.	PASS	SVTYPE=INS:ME:LINE1;END=135088171;SVLEN=748;AN=20618;AC=1;AF=4.850130953535746e-05;N_HET=1;N_HOMALT=0;N_HOMREF=10308;FREQ_HET=9.700261907071492e-05;FREQ_HOMALT=0.0;FREQ_HOMREF=0.9999029973809292;N_BI_GENOS=10309;Lingnan_Han_AN=1888;Lingnan_Han_AC=1;Lingna
chr3	135180942	.	A	<INS:ME:LINE1>	.	PASS	SVTYPE=INS:ME:LINE1;END=135180957;SVLEN=3759;AN=20618;AC=1;AF=4.850130953535746e-05;N_HET=1;N_HOMALT=0;N_HOMREF=10308;FREQ_HET=9.700261907071492e-05;FREQ_HOMALT=0.0;FREQ_HOMREF=0.9999029973809292;N_BI_GENOS=10309;Hui_AN=444;Hui_AC=1;Hui_AF=0.0022522522522
chr3	135199508	.	A	<INS:ME:LINE1>	.	PASS	SVTYPE=INS:ME:LINE1;END=135199512;SVLEN=2863;AN=20618;AC=7;AF=0.0003395091667475022;N_HET=7;N_HOMALT=0;N_HOMREF=10302;FREQ_HET=0.0006790183334950044;FREQ_HOMALT=0.0;FREQ_HOMREF=0.999320981666505;N_BI_GENOS=10309;South_Han_AN=1496;South_Han_AC=2;South_Han_
chr3	135273844	.	T	<INS:ME:LINE1>	.	PASS	SVTYPE=INS:ME:LINE1;END=135273864;SVLEN=1978;AN=20618;AC=1;AF=4.850130953535746e-05;N_HET=1;N_HOMALT=0;N_HOMREF=10308;FREQ_HET=9.700261907071492e-05;FREQ_HOMALT=0.0;FREQ_HOMREF=0.9999029973809292;N_BI_GENOS=10309;Central_Han_AN=2128;Central_Han_AC=1;Centr
chr3	135337578	.	A	<INS:ME:LINE1>	.	PASS	SVTYPE=INS:ME:LINE1;END=135337588;SVLEN=795;AN=20618;AC=1;AF=4.850130953535746e-05;N_HET=1;N_HOMALT=0;N_HOMREF=10308;FREQ_HET=9.700261907071492e-05;FREQ_HOMALT=0.0;FREQ_HOMREF=0.9999029973809292;N_BI_GENOS=10309;North_Han_AN=2272;North_Han_AC=1;North_Han_
chr3	135371144	.	G	<INS:ME:LINE1>	.	PASS	SVTYPE=INS:ME:LINE1;END=135371159;SVLEN=341;AN=20618;AC=9;AF=0.0004365117858182171;N_HET=9;N_HOMALT=0;N_HOMREF=10300;FREQ_HET=0.0008730235716364342;FREQ_HOMALT=0.0;FREQ_HOMREF=0.9991269764283636;N_BI_GENOS=10309;Northwest_Han_AN=1296;Northwest_Han_AC=1;No
chr3	135406693	.	G	<INS:ME:LINE1>	.	PASS	SVTYPE=INS:ME:LINE1;END=135406693;SVLEN=5999;AN=20618;AC=1;AF=4.850130953535746e-05;N_HET=1;N_HOMALT=0;N_HOMREF=10308;FREQ_HET=9.700261907071492e-05;FREQ_HOMALT=0.0;FREQ_HOMREF=0.9999029973809292;N_BI_GENOS=10309;East_Han_AN=6554;East_Han_AC=1;East_Han_AF
chr3	136497371	.	A	<INS:ME:LINE1>	.	PASS	SVTYPE=INS:ME:LINE1;END=136497382;SVLEN=5999;AN=20618;AC=1;AF=4.850130953535746e-05;N_HET=1;N_HOMALT=0;N_HOMREF=10308;FREQ_HET=9.700261907071492e-05;FREQ_HOMALT=0.0;FREQ_HOMREF=0.9999029973809292;N_BI_GENOS=10309;South_Han_AN=1496;South_Han_AC=1;South_Han
chr3	137105515	.	A	<INS:ME:LINE1>	.	PASS	SVTYPE=INS:ME:LINE1;END=137105532;SVLEN=6031;AN=20618;AC=1;AF=4.850130953535746e-05;N_HET=1;N_HOMALT=0;N_HOMREF=10308;FREQ_HET=9.700261907071492e-05;FREQ_HOMALT=0.0;FREQ_HOMREF=0.9999029973809292;N_BI_GENOS=10309;North_Han_AN=2272;North_Han_AC=1;North_Han
chr3	137139751	.	G	<INS:ME:LINE1>	.	PASS	SVTYPE=INS:ME:LINE1;END=137139795;SVLEN=3871;AN=20618;AC=2;AF=9.700261907071492e-05;N_HET=2;N_HOMALT=0;N_HOMREF=10307;FREQ_HET=0.00019400523814142983;FREQ_HOMALT=0.0;FREQ_HOMREF=0.9998059947618586;N_BI_GENOS=10309;Lingnan_Han_AN=1888;Lingnan_Han_AC=1;Ling
chr3	137356444	.	G	<INS:ME:LINE1>	.	PASS	SVTYPE=INS:ME:LINE1;END=137356459;SVLEN=619;AN=20618;AC=1;AF=4.850130953535746e-05;N_HET=1;N_HOMALT=0;N_HOMREF=10308;FREQ_HET=9.700261907071492e-05;FREQ_HOMALT=0.0;FREQ_HOMREF=0.9999029973809292;N_BI_GENOS=10309;Lingnan_Han_AN=1888;Lingnan_Han_AC=1;Lingna
chr3	137388681	.	A	<INS:ME:LINE1>	.	PASS	SVTYPE=INS:ME:LINE1;END=137388699;SVLEN=2046;AN=20618;AC=19;AF=0.0009215248811717916;N_HET=19;N_HOMALT=0;N_HOMREF=10290;FREQ_HET=0.0018430497623435833;FREQ_HOMALT=0.0;FREQ_HOMREF=0.9981569502376564;N_BI_GENOS=10309;South_Han_AN=1496;South_Han_AC=1;South_H
chr3	137485879	.	C	<INS:ME:LINE1>	.	PASS	SVTYPE=INS:ME:LINE1;END=137485879;SVLEN=1401;AN=20618;AC=1;AF=4.850130953535746e-05;N_HET=1;N_HOMALT=0;N_HOMREF=10308;FREQ_HET=9.700261907071492e-05;FREQ_HOMALT=0.0;FREQ_HOMREF=0.9999029973809292;N_BI_GENOS=10309;East_Han_AN=6554;East_Han_AC=1;East_Han_AF
chr3	137720508	.	T	<INS:ME:LINE1>	.	PASS	SVTYPE=INS:ME:LINE1;END=137720521;SVLEN=684;AN=20618;AC=4;AF=0.00019400523814142983;N_HET=4;N_HOMALT=0;N_HOMREF=10305;FREQ_HET=0.0003880104762828596 6;FREQ_HOMALT=0.0;FREQ_HOMREF=0.9996119895237171;N_BI_GENOS=10309;Lingnan_Han_AN=1888;Lingnan_Han_AC=1;Ling
chr3	137778678	.	G	<INS:ME:LINE1>	.	PASS	SVTYPE=INS:ME:LINE1;END=137778693;SVLEN=1187;AN=20618;AC=4;AF=0.00019400523814142983;N_HET=4;N_HOMALT=0;N_HOMREF=10305;FREQ_HET=0.00038801047628285966;FREQ_HOMALT=0.0;FREQ_HOMREF=0.9996119895237171;N_BI_GENOS=10309;East_Han_AN=6554;East_Han_AC=4;East_Han_
chr3	138957113	.	A	<INS:ME:LINE1>	.	PASS	SVTYPE=INS:ME:LINE1;END=138957130;SVLEN=227;AN=20618;AC=1;AF=4.850130953535746e-05;N_HET=1;N_HOMALT=0;N_HOMREF=10308;FREQ_HET=9.700261907071492e-05;FREQ_HOMALT=0.0;FREQ_HOMREF=0.9999029973809292;N_BI_GENOS=10309;Southeast_Han_AN=1908;Southeast_Han_AC=1;So
chr3	139139188	.	T	<INS:ME:LINE1>	.	PASS	SVTYPE=INS:ME:LINE1;END=139139199;SVLEN=5998;AN=20618;AC=1;AF=4.850130953535746e-05;N_HET=1;N_HOMALT=0;N_HOMREF=10308;FREQ_HET=9.700261907071492e-05;FREQ_HOMALT=0.0;FREQ_HOMREF=0.9999029973809292;N_BI_GENOS=10309;Zhuang_AN=444;Zhuang_AC=1;Zhuang_AF=0.0022
chr3	139149398	.	A	<INS:ME:LINE1>	.	PASS	SVTYPE=INS:ME:LINE1;END=139149420;SVLEN=1616;AN=20618;AC=1;AF=4.850130953535746e-05;N_HET=1;N_HOMALT=0;N_HOMREF=10308;FREQ_HET=9.700261907071492e-05;FREQ_HOMALT=0.0;FREQ_HOMREF=0.9999029973809292;N_BI_GENOS=10309;East_Han_AN=6554;East_Han_AC=1;East_Han_AF
chr3	139325584	.	A	<INS:ME:LINE1>	.	PASS	SVTYPE=INS:ME:LINE1;END=139325599;SVLEN=5993;AN=20618;AC=665;AF=0.03225337084101271;N_HET=625;N_HOMALT=20;N_HOMREF=9664;FREQ_HET=0.06062663691919682;FREQ_HOMALT=0.0019400523814142982;FREQ_HOMREF=0.9374333106993888;N_BI_GENOS=10309;South_Han_AN=1496;South_
chr3	139416423	.	G	<INS:ME:LINE1>	.	PASS	SVTYPE=INS:ME:LINE1;END=139416438;SVLEN=6003;AN=20618;AC=2;AF=9.700261907071492e-05;N_HET=2;N_HOMALT=0;N_HOMREF=10307;FREQ_HET=0.00019400523814142983;FREQ_HOMALT=0.0;FREQ_HOMREF=0.9998059947618586;N_BI_GENOS=10309;North_Han_AN=2272;North_Han_AC=1;North_Ha
chr3	139746609	.	A	<INS:ME:LINE1>	.	PASS	SVTYPE=INS:ME:LINE1;END=139746625;SVLEN=878;AN=20618;AC=1;AF=4.850130953535746e-05;N_HET=1;N_HOMALT=0;N_HOMREF=10308;FREQ_HET=9.700261907071492e-05;FREQ_HOMALT=0.0;FREQ_HOMREF=0.9999029973809292;N_BI_GENOS=10309;North_Han_AN=2272;North_Han_AC=1;North_Han_

chr3	139908094	.	A	<INS:ME:LINE1>	.	PASS	SVTYPE=INS:ME:LINE1;END=139908108;SVLEN=1538;AN=20618;AC=13;AF=0.0006305170239596469;N_HET=11;N_HOMALT=1;N_HOMREF=10297;FREQ_HET=0.001067028809777864;FREQ_HOMALT=9.700261907071492e-05;FREQ_HOMREF=0.9988359685711514;N_BI_GENOS=10309;Lingnan_Han_AN=1888;Lin
chr3	139918775	.	A	<INS:ME:LINE1>	.	PASS	SVTYPE=INS:ME:LINE1;END=139918789;SVLEN=1347;AN=20618;AC=1;AF=4.850130953535746e-05;N_HET=1;N_HOMALT=0;N_HOMREF=10308;FREQ_HET=9.700261907071492e-05;FREQ_HOMALT=0.0;FREQ_HOMREF=0.9999029973809292;N_BI_GENOS=10309;Central_Han_AN=2128;Central_Han_AC=1;Centr
chr3	139971966	.	A	<INS:ME:LINE1>	.	PASS	SVTYPE=INS:ME:LINE1;END=139971981;SVLEN=6000;AN=20618;AC=1;AF=4.850130953535746e-05;N_HET=1;N_HOMALT=0;N_HOMREF=10308;FREQ_HET=9.700261907071492e-05;FREQ_HOMALT=0.0;FREQ_HOMREF=0.9999029973809292;N_BI_GENOS=10309;East_Han_AN=6554;East_Han_AC=1;East_Han_AF
chr3	140257069	.	A	<INS:ME:LINE1>	.	PASS	SVTYPE=INS:ME:LINE1;END=140257071;SVLEN=1095;AN=20618;AC=7;AF=0.0003395091667475022;N_HET=7;N_HOMALT=0;N_HOMREF=10302;FREQ_HET=0.0006790183334950044;FREQ_HOMALT=0.0;FREQ_HOMREF=0.999320981666505;N_BI_GENOS=10309;South_Han_AN=1496;South_Han_AC=1;South_Han_
chr3	140598991	.	A	<INS:ME:LINE1>	.	PASS	SVTYPE=INS:ME:LINE1;END=140599008;SVLEN=5997;AN=20618;AC=2;AF=9.700261907071492e-05;N_HET=0;N_HOMALT=1;N_HOMREF=10308;FREQ_HET=0.0;FREQ_HOMALT=9.700261907071492e-05;FREQ_HOMREF=0.9999029973809292;N_BI_GENOS=10309;Southeast_Han_AN=1908;Southeast_Han_AC=2;S
chr3	140643555	.	A	<INS:ME:LINE1>	.	PASS	SVTYPE=INS:ME:LINE1;END=140643571;SVLEN=5996;AN=20618;AC=3;AF=0.00014550392860607236;N_HET=3;N_HOMALT=0;N_HOMREF=10306;FREQ_HET=0.0002910078572121447;FREQ_HOMALT=0.0;FREQ_HOMREF=0.9997089921427879;N_BI_GENOS=10309;East_Han_AN=6554;East_Han_AC=1;East_Han_A
chr3	140873227	.	C	<INS:ME:LINE1>	.	PASS	SVTYPE=INS:ME:LINE1;END=140873243;SVLEN=894;AN=20618;AC=1;AF=4.850130953535746e-05;N_HET=1;N_HOMALT=0;N_HOMREF=10308;FREQ_HET=9.700261907071492e-05;FREQ_HOMALT=0.0;FREQ_HOMREF=0.9999029973809292;N_BI_GENOS=10309;East_Han_AN=6554;East_Han_AC=1;East_Han_AF=
chr3	140884189	.	A	<INS:ME:LINE1>	.	PASS	SVTYPE=INS:ME:LINE1;END=140884199;SVLEN=100;AN=20618;AC=1;AF=4.850130953535746e-05;N_HET=1;N_HOMALT=0;N_HOMREF=10308;FREQ_HET=9.700261907071492e-05;FREQ_HOMALT=0.0;FREQ_HOMREF=0.9999029973809292;N_BI_GENOS=10309;Northwest_Han_AN=1296;Northwest_Han_AC=1;No
chr3	141090794	.	G	<INS:ME:LINE1>	.	PASS	SVTYPE=INS:ME:LINE1;END=141090813;SVLEN=5998;AN=20618;AC=1;AF=4.850130953535746e-05;N_HET=1;N_HOMALT=0;N_HOMREF=10308;FREQ_HET=9.700261907071492e-05;FREQ_HOMALT=0.0;FREQ_HOMREF=0.9999029973809292;N_BI_GENOS=10309;East_Han_AN=6554;East_Han_AC=1;East_Han_AF
chr3	141767083	.	A	<INS:ME:LINE1>	.	PASS	SVTYPE=INS:ME:LINE1;END=141767096;SVLEN=6000;AN=20618;AC=1;AF=4.850130953535746e-05;N_HET=1;N_HOMALT=0;N_HOMREF=10308;FREQ_HET=9.700261907071492e-05;FREQ_HOMALT=0.0;FREQ_HOMREF=0.9999029973809292;N_BI_GENOS=10309;South_Han_AN=1496;South_Han_AC=1;South_Han
chr3	142071139	.	A	<INS:ME:LINE1>	.	PASS	SVTYPE=INS:ME:LINE1;END=142071154;SVLEN=3118;AN=20618;AC=14;AF=0.0006790183334950044;N_HET=14;N_HOMALT=0;N_HOMREF=10295;FREQ_HET=0.0013580366669900088;FREQ_HOMALT=0.0;FREQ_HOMREF=0.99864196333301;N_BI_GENOS=10309;South_Han_AN=1496;South_Han_AC=2;South_Han
chr3	142175439	.	T	<INS:ME:LINE1>	.	PASS	SVTYPE=INS:ME:LINE1;END=142175440;SVLEN=1460;AN=20618;AC=6;AF=0.0002910078572121447;N_HET=6;N_HOMALT=0;N_HOMREF=10303;FREQ_HET=0.0005820157144242894;FREQ_HOMALT=0.0;FREQ_HOMREF=0.9994179842855757;N_BI_GENOS=10309;South_Han_AN=1496;South_Han_AC=1;South_Han
chr3	142613556	.	G	<INS:ME:LINE1>	.	PASS	SVTYPE=INS:ME:LINE1;END=142613573;SVLEN=142;AN=20618;AC=1;AF=4.850130953535746e-05;N_HET=1;N_HOMALT=0;N_HOMREF=10308;FREQ_HET=9.700261907071492e-05;FREQ_HOMALT=0.0;FREQ_HOMREF=0.9999029973809292;N_BI_GENOS=10309;Zhuang_AN=444;Zhuang_AC=1;Zhuang_AF=0.00225
chr3	142645498	.	A	<INS:ME:LINE1>	.	PASS	SVTYPE=INS:ME:LINE1;END=142645512;SVLEN=600;AN=20618;AC=1;AF=4.850130953535746e-05;N_HET=1;N_HOMALT=0;N_HOMREF=10308;FREQ_HET=9.700261907071492e-05;FREQ_HOMALT=0.0;FREQ_HOMREF=0.9999029973809292;N_BI_GENOS=10309;Hui_AN=444;Hui_AC=1;Hui_AF=0.0022522522522 5
chr3	142735713	.	A	<INS:ME:LINE1>	.	PASS	SVTYPE=INS:ME:LINE1;END=142735714;SVLEN=401;AN=20618;AC=1;AF=4.850130953535746e-05;N_HET=1;N_HOMALT=0;N_HOMREF=10308;FREQ_HET=9.700261907071492e-05;FREQ_HOMALT=0.0;FREQ_HOMREF=0.9999029973809292;N_BI_GENOS=10309;Northwest_Han_AN=1296;Northwest_Han_AC=1;No
chr3	143402916	.	T	<INS:ME:LINE1>	.	PASS	SVTYPE=INS:ME:LINE1;END=143402931;SVLEN=5994;AN=20618;AC=2434;AF=0.11805218740906004;N_HET=1848;N_HOMALT=293;N_HOMREF=8168;FREQ_HET=0.17926084004268114;FREQ_HOMALT=0.028421767387719468;FREQ_HOMREF=0.7923173925695994;N_BI_GENOS=10309;South_Han_AN=1496;Sout
chr3	143754377	.	G	<INS:ME:LINE1>	.	PASS	SVTYPE=INS:ME:LINE1;END=143754382;SVLEN=2523;AN=20618;AC=1;AF=4.850130953535746e-05;N_HET=1;N_HOMALT=0;N_HOMREF=10308;FREQ_HET=9.700261907071492e-05;FREQ_HOMALT=0.0;FREQ_HOMREF=0.9999029973809292;N_BI_GENOS=10309;South_Han_AN=1496;South_Han_AC=1;South_Han
chr3	143773302	.	C	<INS:ME:LINE1>	.	PASS	SVTYPE=INS:ME:LINE1;END=143773317;SVLEN=1193;AN=20618;AC=2;AF=9.700261907071492e-05;N_HET=2;N_HOMALT=0;N_HOMREF=10307;FREQ_HET=0.00019400523814142983;FREQ_HOMALT=0.0;FREQ_HOMREF=0.9998059947618586;N_BI_GENOS=10309;East_Han_AN=6554;East_Han_AC=1;East_Han_A
chr3	143896013	.	C	<INS:ME:LINE1>	.	PASS	SVTYPE=INS:ME:LINE1;END=143896025;SVLEN=707;AN=20618;AC=1;AF=4.850130953535746e-05;N_HET=1;N_HOMALT=0;N_HOMREF=10308;FREQ_HET=9.700261907071492e-05;FREQ_HOMALT=0.0;FREQ_HOMREF=0.9999029973809292;N_BI_GENOS=10309;East_Han_AN=6554;East_Han_AC=1;East_Han_AF=
chr3	143943561	.	A	<INS:ME:LINE1>	.	PASS	SVTYPE=INS:ME:LINE1;END=143943578;SVLEN=5996;AN=20618;AC=1;AF=4.850130953535746e-05;N_HET=1;N_HOMALT=0;N_HOMREF=10308;FREQ_HET=9.700261907071492e-05;FREQ_HOMALT=0.0;FREQ_HOMREF=0.9999029973809292;N_BI_GENOS=10309;North_Han_AN=2272;North_Han_AC=1;North_Han
chr3	143951489	.	T	<INS:ME:LINE1>	.	PASS	SVTYPE=INS:ME:LINE1;END=143951492;SVLEN=805;AN=20618;AC=1;AF=4.850130953535746e-05;N_HET=1;N_HOMALT=0;N_HOMREF=10308;FREQ_HET=9.700261907071492e-05;FREQ_HOMALT=0.0;FREQ_HOMREF=0.9999029973809292;N_BI_GENOS=10309;North_Han_AN=2272;North_Han_AC=1;North_Han_
chr3	144066772	.	A	<INS:ME:LINE1>	.	PASS	SVTYPE=INS:ME:LINE1;END=144066786;SVLEN=2860;AN=20618;AC=1;AF=4.850130953535746e-05;N_HET=1;N_HOMALT=0;N_HOMREF=10308;FREQ_HET=9.700261907071492e-05;FREQ_HOMALT=0.0;FREQ_HOMREF=0.9999029973809292;N_BI_GENOS=10309;Southeast_Han_AN=1908;Southeast_Han_AC=1;S
chr3	144481968	.	G	<INS:ME:LINE1>	.	PASS	SVTYPE=INS:ME:LINE1;END=144481981;SVLEN=6007;AN=20618;AC=1;AF=4.850130953535746e-05;N_HET=1;N_HOMALT=0;N_HOMREF=10308;FREQ_HET=9.700261907071492e-05;FREQ_HOMALT=0.0;FREQ_HOMREF=0.9999029973809292;N_BI_GENOS=10309;East_Han_AN=6554;East_Han_AC=1;East_Han_AF
chr3	144610260	.	A	<INS:ME:LINE1>	.	PASS	SVTYPE=INS:ME:LINE1;END=144610260;SVLEN=1119;AN=20618;AC=2;AF=9.700261907071492e-05;N_HET=2;N_HOMALT=0;N_HOMREF=10307;FREQ_HET=0.00019400523814142983;FREQ_HOMALT=0.0;FREQ_HOMREF=0.9998059947618586;N_BI_GENOS=10309;Northwest_Han_AN=1296;Northwest_Han_AC=1;
chr3	144614621	.	T	<INS:ME:LINE1>	.	PASS	SVTYPE=INS:ME:LINE1;END=144614627;SVLEN=1254;AN=20618;AC=1;AF=4.850130953535746e-05;N_HET=1;N_HOMALT=0;N_HOMREF=10308;FREQ_HET=9.700261907071492e-05;FREQ_HOMALT=0.0;FREQ_HOMREF=0.9999029973809292;N_BI_GENOS=10309;East_Han_AN=6554;East_Han_AC=1;East_Han_AF
chr3	144697691	.	T	<INS:ME:LINE1>	.	PASS	SVTYPE=INS:ME:LINE1;END=144697691;SVLEN=383;AN=20618;AC=2;AF=9.700261907071492e-05;N_HET=0;N_HOMALT=1;N_HOMREF=10308;FREQ_HET=0.0;FREQ_HOMALT=9.700261907071492e-05;FREQ_HOMREF=0.9999029973809292;N_BI_GENOS=10309;East_Han_AN=6554;East_Han_AC=2;East_Han_AF=
chr3	144710806	.	T	<INS:ME:LINE1>	.	PASS	SVTYPE=INS:ME:LINE1;END=144710822;SVLEN=397;AN=20618;AC=5;AF=0.00024250654767678728;N_HET=5;N_HOMALT=0;N_HOMREF=10304;FREQ_HET=0.00048501309535357455;FREQ_HOMALT=0.0;FREQ_HOMREF=0.9995149869046465;N_BI_GENOS=10309;East_Han_AN=6554;East_Han_AC=3;East_Han_A
chr3	144847226	.	A	<INS:ME:LINE1>	.	PASS	SVTYPE=INS:ME:LINE1;END=144847236;SVLEN=1826;AN=20618;AC=1;AF=4.850130953535746e-05;N_HET=1;N_HOMALT=0;N_HOMREF=10308;FREQ_HET=9.700261907071492e-05;FREQ_HOMALT=0.0;FREQ_HOMREF=0.9999029973809292;N_BI_GENOS=10309;East_Han_AN=6554;East_Han_AC=1;East_Han_AF
chr3	144891229	.	T	<INS:ME:LINE1>	.	PASS	SVTYPE=INS:ME:LINE1;END=144891241;SVLEN=5230;AN=20618;AC=9;AF=0.0004365117858182171;N_HET=9;N_HOMALT=0;N_HOMREF=10300;FREQ_HET=0.0008730235716364342;FREQ_HOMALT=0.0;FREQ_HOMREF=0.9991269764283636;N_BI_GENOS=10309;Northwest_Han_AN=1296;Northwest_Han_AC=4;N
chr3	144964158	.	A	<INS:ME:LINE1>	.	PASS	SVTYPE=INS:ME:LINE1;END=144964158;SVLEN=18;AN=20618;AC=8;AF=0.0003880104762828596 6;N_HET=8;N_HOMALT=0;N_HOMREF=10301;FREQ_HET=0.0007760209525657193;FREQ_HOMALT=0.0;FREQ_HOMREF=0.9992239790474343;N_BI_GENOS=10309;South_Han_AN=1496;South_Han_AC=2;South_Han_
chr3	145416393	.	A	<INS:ME:LINE1>	.	PASS	SVTYPE=INS:ME:LINE1;END=145416408;SVLEN=5997;AN=20618;AC=1;AF=4.850130953535746e-05;N_HET=1;N_HOMALT=0;N_HOMREF=10308;FREQ_HET=9.700261907071492e-05;FREQ_HOMALT=0.0;FREQ_HOMREF=0.9999029973809292;N_BI_GENOS=10309;Central_Han_AN=2128;Central_Han_AC=1;Centr
chr3	145472048	.	A	<INS:ME:LINE1>	.	PASS	SVTYPE=INS:ME:LINE1;END=145472063;SVLEN=123;AN=20618;AC=1;AF=4.850130953535746e-05;N_HET=1;N_HOMALT=0;N_HOMREF=10308;FREQ_HET=9.700261907071492e-05;FREQ_HOMALT=0.0;FREQ_HOMREF=0.9999029973809292;N_BI_GENOS=10309;Southeast_Han_AN=1908;Southeast_Han_AC=1;So
chr3	145745619	.	C	<INS:ME:LINE1>	.	PASS	SVTYPE=INS:ME:LINE1;END=145745636;SVLEN=5975;AN=20618;AC=1;AF=4.850130953535746e-05;N_HET=1;N_HOMALT=0;N_HOMREF=10308;FREQ_HET=9.700261907071492e-05;FREQ_HOMALT=0.0;FREQ_HOMREF=0.9999029973809292;N_BI_GENOS=10309;Tibetan_AN=414;Tibetan_AC=1;Tibetan_AF=0.0
chr3	146053189	.	A	<INS:ME:LINE1>	.	PASS	SVTYPE=INS:ME:LINE1;END=146053204;SVLEN=6000;AN=20618;AC=8;AF=0.00038801047628285966;N_HET=8;N_HOMALT=0;N_HOMREF=10301;FREQ_HET=0.0007760209525657193;FREQ_HOMALT=0.0;FREQ_HOMREF=0.9992239790474343;N_BI_GENOS=10309;Northwest_Han_AN=1296;Northwest_Han_AC=1;
chr3	146114246	.	C	<INS:ME:LINE1>	.	PASS	SVTYPE=INS:ME:LINE1;END=146114246;SVLEN=0;AN=20618;AC=2;AF=9.700261907071492e-05;N_HET=0;N_HOMALT=1;N_HOMREF=10308;FREQ_HET=0.0;FREQ_HOMALT=9.700261907071492e-05;FREQ_HOMREF=0.9999029973809292;N_BI_GENOS=10309;East_Han_AN=6554;East_Han_AC=2;East_Han_AF=0.
chr3	146223105	.	A	<INS:ME:LINE1>	.	PASS	SVTYPE=INS:ME:LINE1;END=146223116;SVLEN=1226;AN=20618;AC=2;AF=9.700261907071492e-05;N_HET=0;N_HOMALT=1;N_HOMREF=10308;FREQ_HET=0.0;FREQ_HOMALT=9.700261907071492e-05;FREQ_HOMREF=0.9999029973809292;N_BI_GENOS=10309;East_Han_AN=6554;East_Han_AC=2;East_Han_AF
chr3	146377768	.	T	<INS:ME:LINE1>	.	PASS	SVTYPE=INS:ME:LINE1;END=146377785;SVLEN=6201;AN=20618;AC=1;AF=4.850130953535746e-05;N_HET=1;N_HOMALT=0;N_HOMREF=10308;FREQ_HET=9.700261907071492e-05;FREQ_HOMALT=0.0;FREQ_HOMREF=0.9999029973809292;N_BI_GENOS=10309;South_Han_AN=1496;South_Han_AC=1;South_Han
chr3	146392658	.	T	<INS:ME:LINE1>	.	PASS	SVTYPE=INS:ME:LINE1;END=146392675;SVLEN=1463;AN=20618;AC=2;AF=9.700261907071492e-05;N_HET=0;N_HOMALT=1;N_HOMREF=10308;FREQ_HET=0.0;FREQ_HOMALT=9.700261907071492e-05;FREQ_HOMREF=0.9999029973809292;N_BI_GENOS=10309;South_Han_AN=1496;South_Han_AC=2;South_Han
chr3	146535130	.	C	<INS:ME:LINE1>	.	PASS	SVTYPE=INS:ME:LINE1;END=146535145;SVLEN=6027;AN=20618;AC=1;AF=4.850130953535746e-05;N_HET=1;N_HOMALT=0;N_HOMREF=10308;FREQ_HET=9.700261907071492e-05;FREQ_HOMALT=0.0;FREQ_HOMREF=0.9999029973809292;N_BI_GENOS=10309;Central_Han_AN=2128;Central_Han_AC=1;Centr
chr3	146653651	.	A	<INS:ME:LINE1>	.	PASS	SVTYPE=INS:ME:LINE1;END=146653670;SVLEN=1589;AN=20618;AC=1;AF=4.850130953535746e-05;N_HET=1;N_HOMALT=0;N_HOMREF=10308;FREQ_HET=9.700261907071492e-05;FREQ_HOMALT=0.0;FREQ_HOMREF=0.9999029973809292;N_BI_GENOS=10309;Manchu_AN=416;Manchu_AC=1;Manchu_AF=0.0024
chr3	146827887	.	T	<INS:ME:LINE1>	.	PASS	SVTYPE=INS:ME:LINE1;END=146827899;SVLEN=6003;AN=20618;AC=1;AF=4.850130953535746e-05;N_HET=1;N_HOMALT=0;N_HOMREF=10308;FREQ_HET=9.700261907071492e-05;FREQ_HOMALT=0.0;FREQ_HOMREF=0.9999029973809292;N_BI_GENOS=10309;Central_Han_AN=2128;Central_Han_AC=1;Centr
chr3	146834425	.	T	<INS:ME:LINE1>	.	PASS	SVTYPE=INS:ME:LINE1;END=146834442;SVLEN=485;AN=20618;AC=1;AF=4.850130953535746e-05;N_HET=1;N_HOMALT=0;N_HOMREF=10308;FREQ_HET=9.700261907071492e-05;FREQ_HOMALT=0.0;FREQ_HOMREF=0.9999029973809292;N_BI_GENOS=10309;Southeast_Han_AN=1908;Southeast_Han_AC=1;So
chr3	146835042	.	C	<INS:ME:LINE1>	.	PASS	SVTYPE=INS:ME:LINE1;END=146835042;SVLEN=5955;AN=20618;AC=1;AF=4.850130953535746e-05;N_HET=1;N_HOMALT=0;N_HOMREF=10308;FREQ_HET=9.700261907071492e-05;FREQ_HOMALT=0.0;FREQ_HOMREF=0.9999029973809292;N_BI_GENOS=10309;Mongolian_AN=454;Mongolian_AC=1;Mongolian_
chr3	147088767	.	T	<INS:ME:LINE1>	.	PASS	SVTYPE=INS:ME:LINE1;END=147088767;SVLEN=2764;AN=20618;AC=1;AF=4.850130953535746e-05;N_HET=1;N_HOMALT=0;N_HOMREF=10308;FREQ_HET=9.700261907071492e-05;FREQ_HOMALT=0.0;FREQ_HOMREF=0.9999029973809292;N_BI_GENOS=10309;Manchu_AN=416;Manchu_AC=1;Manchu_AF=0.0024
chr3	147253448	.	A	<INS:ME:LINE1>	.	PASS	SVTYPE=INS:ME:LINE1;END=147253464;SVLEN=329;AN=20618;AC=1;AF=4.850130953535746e-05;N_HET=1;N_HOMALT=0;N_HOMREF=10308;FREQ_HET=9.700261907071492e-05;FREQ_HOMALT=0.0;FREQ_HOMREF=0.9999029973809292;N_BI_GENOS=10309;East_Han_AN=6554;East_Han_AC=1;East_Han_AF=
chr3	147295725	.	A	<INS:ME:LINE1>	.	PASS	SVTYPE=INS:ME:LINE1;END=147295725;SVLEN=450;AN=20618;AC=1;AF=4.850130953535746e-05;N_HET=1;N_HOMALT=0;N_HOMREF=10308;FREQ_HET=9.700261907071492e-05;FREQ_HOMALT=0.0;FREQ_HOMREF=0.9999029973809292;N_BI_GENOS=10309;East_Han_AN=6554;East_Han_AC=1;East_Han_AF=
chr3	147323350	.	A	<INS:ME:LINE1>	.	PASS	SVTYPE=INS:ME:LINE1;END=147323350;SVLEN=639;AN=20618;AC=1;AF=4.850130953535746e-05;N_HET=1;N_HOMALT=0;N_HOMREF=10308;FREQ_HET=9.700261907071492e-05;FREQ_HOMALT=0.0;FREQ_HOMREF=0.9999029973809292;N_BI_GENOS=10309;East_Han_AN=6554;East_Han_AC=1;East_Han_AF=
chr3	147499982	.	C	<INS:ME:LINE1>	.	PASS	SVTYPE=INS:ME:LINE1;END=147499996;SVLEN=357;AN=20618;AC=1;AF=4.850130953535746e-05;N_HET=1;N_HOMALT=0;N_HOMREF=10308;FREQ_HET=9.700261907071492e-05;FREQ_HOMALT=0.0;FREQ_HOMREF=0.9999029973809292;N_BI_GENOS=10309;South_Han_AN=1496;South_Han_AC=1;South_Han_
chr3	147603593	.	A	<INS:ME:LINE1>	.	PASS	SVTYPE=INS:ME:LINE1;END=147603608;SVLEN=1995;AN=20618;AC=1;AF=4.850130953535746e-05;N_HET=1;N_HOMALT=0;N_HOMREF=10308;FREQ_HET=9.700261907071492e-05;FREQ_HOMALT=0.0;FREQ_HOMREF=0.9999029973809292;N_BI_GENOS=10309;East_Han_AN=6554;East_Han_AC=1;East_Han_AF
chr3	147826949	.	A	<INS:ME:LINE1>	.	PASS	SVTYPE=INS:ME:LINE1;END=147826949;SVLEN=1217;AN=20618;AC=1;AF=4.850130953535746e-05;N_HET=1;N_HOMALT=0;N_HOMREF=10308;FREQ_HET=9.700261907071492e-05;FREQ_HOMALT=0.0;FREQ_HOMREF=0.9999029973809292;N_BI_GENOS=10309;Northwest_Han_AN=1296;Northwest_Han_AC=1;N
chr3	148246007	.	A	<INS:ME:LINE1>	.	PASS	SVTYPE=INS:ME:LINE1;END=148246015;SVLEN=387;AN=20618;AC=2;AF=9.700261907071492e-05;N_HET=2;N_HOMALT=0;N_HOMREF=10307;FREQ_HET=0.00019400523814142983;FREQ_HOMALT=0.0;FREQ_HOMREF=0.9998059947618586;N_BI_GENOS=10309;East_Han_AN=6554;East_Han_AC=1;East_Han_AF
chr3	148363407	.	A	<INS:ME:LINE1>	.	PASS	SVTYPE=INS:ME:LINE1;END=148363421;SVLEN=1804;AN=20618;AC=1;AF=4.850130953535746e-05;N_HET=1;N_HOMALT=0;N_HOMREF=10308;FREQ_HET=9.700261907071492e-05;FREQ_HOMALT=0.0;FREQ_HOMREF=0.9999029973809292;N_BI_GENOS=10309;Yi_AN=422;Yi_AC=1;Yi_AF=0.0023696682464454
chr3	148647576	.	A	<INS:ME:LINE1>	.	PASS	SVTYPE=INS:ME:LINE1;END=148647587;SVLEN=586;AN=20618;AC=4;AF=0.00019400523814142983;N_HET=0;N_HOMALT=2;N_HOMREF=10307;FREQ_HET=0.0;FREQ_HOMALT=0.00019400523814142983;FREQ_HOMREF=0.9998059947618586;N_BI_GENOS=10309;North_Han_AN=2272;North_Han_AC=4;North_Ha
chr3	148897171	.	A	<INS:ME:LINE1>	.	PASS	SVTYPE=INS:ME:LINE1;END=148897196;SVLEN=5351;AN=20618;AC=2;AF=9.700261907071492e-05;N_HET=0;N_HOMALT=1;N_HOMREF=10308;FREQ_HET=0.0;FREQ_HOMALT=9.700261907071492e-05;FREQ_HOMREF=0.9999029973809292;N_BI_GENOS=10309;Yi_AN=422;Yi_AC=2;Yi_AF=0.0047393364928909
chr3	148936666	.	A	<INS:ME:LINE1>	.	PASS	SVTYPE=INS:ME:LINE1;END=148936684;SVLEN=6036;AN=20618;AC=6;AF=0.0002910078572121447;N_HET=6;N_HOMALT=0;N_HOMREF=10303;FREQ_HET=0.0005820157144242894;FREQ_HOMALT=0.0;FREQ_HOMREF=0.9994179842855757;N_BI_GENOS=10309;South_Han_AN=1496;South_Han_AC=1;South_Han
chr3	150270727	.	G	<INS:ME:LINE1>	.	PASS	SVTYPE=INS:ME:LINE1;END=150270727;SVLEN=5047;AN=20618;AC=2;AF=9.700261907071492e-05;N_HET=2;N_HOMALT=0;N_HOMREF=10307;FREQ_HET=0.00019400523814142983;FREQ_HOMALT=0.0;FREQ_HOMREF=0.9998059947618586;N_BI_GENOS=10309;East_Han_AN=6554;East_Han_AC=2;East_Han_A
chr3	150614020	.	A	<INS:ME:LINE1>	.	PASS	SVTYPE=INS:ME:LINE1;END=150614023;SVLEN=4520;AN=20618;AC=1;AF=4.850130953535746e-05;N_HET=1;N_HOMALT=0;N_HOMREF=10308;FREQ_HET=9.700261907071492e-05;FREQ_HOMALT=0.0;FREQ_HOMREF=0.9999029973809292;N_BI_GENOS=10309;Southeast_Han_AN=1908;Southeast_Han_AC=1;S
chr3	151050726	.	T	<INS:ME:LINE1>	.	PASS	SVTYPE=INS:ME:LINE1;END=151050743;SVLEN=1132;AN=20618;AC=4;AF=0.00019400523814142983;N_HET=4;N_HOMALT=0;N_HOMREF=10305;FREQ_HET=0.00038801047628285966;FREQ_HOMALT=0.0;FREQ_HOMREF=0.9996119895237171;N_BI_GENOS=10309;South_Han_AN=1496;South_Han_AC=2;South_H
chr3	151103234	.	C	<INS:ME:LINE1>	.	PASS	SVTYPE=INS:ME:LINE1;END=151103248;SVLEN=6000;AN=20618;AC=1;AF=4.850130953535746e-05;N_HET=1;N_HOMALT=0;N_HOMREF=10308;FREQ_HET=9.700261907071492e-05;FREQ_HOMALT=0.0;FREQ_HOMREF=0.9999029973809292;N_BI_GENOS=10309;East_Han_AN=6554;East_Han_AC=1;East_Han_AF
chr3	151387178	.	A	<INS:ME:LINE1>	.	PASS	SVTYPE=INS:ME:LINE1;END=151387193;SVLEN=5994;AN=20618;AC=1;AF=4.850130953535746e-05;N_HET=1;N_HOMALT=0;N_HOMREF=10308;FREQ_HET=9.700261907071492e-05;FREQ_HOMALT=0.0;FREQ_HOMREF=0.9999029973809292;N_BI_GENOS=10309;East_Han_AN=6554;East_Han_AC=1;East_Han_AF
chr3	151396993	.	C	<INS:ME:LINE1>	.	PASS	SVTYPE=INS:ME:LINE1;END=151397010;SVLEN=5519;AN=20618;AC=6;AF=0.0002910078572121447;N_HET=6;N_HOMALT=0;N_HOMREF=10303;FREQ_HET=0.0005820157144242894;FREQ_HOMALT=0.0;FREQ_HOMREF=0.9994179842855757;N_BI_GENOS=10309;South_Han_AN=1496;South_Han_AC=1;South_Han
chr3	151430755	.	C	<INS:ME:LINE1>	.	PASS	SVTYPE=INS:ME:LINE1;END=151430767;SVLEN=5997;AN=20618;AC=8862;AF=0.42981860510233777;N_HET=4402;N_HOMALT=2230;N_HOMREF=3677;FREQ_HET=0.427005529149287;FREQ_HOMALT=0.21631584052769426;FREQ_HOMREF=0.3566786303230187;N_BI_GENOS=10309;South_Han_AN=1496;South_
chr3	151549431	.	C	<INS:ME:LINE1>	.	PASS	SVTYPE=INS:ME:LINE1;END=151549448;SVLEN=5979;AN=20618;AC=1;AF=4.850130953535746e-05;N_HET=1;N_HOMALT=0;N_HOMREF=10308;FREQ_HET=9.700261907071492e-05;FREQ_HOMALT=0.0;FREQ_HOMREF=0.9999029973809292;N_BI_GENOS=10309;East_Han_AN=6554;East_Han_AC=1;East_Han_AF
chr3	151953393	.	A	<INS:ME:LINE1>	.	PASS	SVTYPE=INS:ME:LINE1;END=151953394;SVLEN=712;AN=20618;AC=1;AF=4.850130953535746e-05;N_HET=1;N_HOMALT=0;N_HOMREF=10308;FREQ_HET=9.700261907071492e-05;FREQ_HOMALT=0.0;FREQ_HOMREF=0.9999029973809292;N_BI_GENOS=10309;Southeast_Han_AN=1908;Southeast_Han_AC=1;So
chr3	151970298	.	T	<INS:ME:LINE1>	.	PASS	SVTYPE=INS:ME:LINE1;END=151970313;SVLEN=5997;AN=20618;AC=339;AF=0.016441943932486176;N_HET=331;N_HOMALT=4;N_HOMREF=9974;FREQ_HET=0.032107866912406635;FREQ_HOMALT=0.00038801047628285966;FREQ_HOMREF=0.9675041226113105;N_BI_GENOS=10309;South_Han_AN=1496;Sout
chr3	151974839	.	A	<INS:ME:LINE1>	.	PASS	SVTYPE=INS:ME:LINE1;END=151974839;SVLEN=505;AN=20618;AC=3;AF=0.00014550392860607236;N_HET=3;N_HOMALT=0;N_HOMREF=10306;FREQ_HET=0.0002910078572121447;FREQ_HOMALT=0.0;FREQ_HOMREF=0.9997089921427879;N_BI_GENOS=10309;East_Han_AN=6554;East_Han_AC=1;East_Han_AF
chr3	152309955	.	C	<INS:ME:LINE1>	.	PASS	SVTYPE=INS:ME:LINE1;END=152309971;SVLEN=1003;AN=20618;AC=1;AF=4.850130953535746e-05;N_HET=1;N_HOMALT=0;N_HOMREF=10308;FREQ_HET=9.700261907071492e-05;FREQ_HOMALT=0.0;FREQ_HOMREF=0.9999029973809292;N_BI_GENOS=10309;East_Han_AN=6554;East_Han_AC=1;East_Han_AF
chr3	152449505	.	T	<INS:ME:LINE1>	.	PASS	SVTYPE=INS:ME:LINE1;END=152449520;SVLEN=169;AN=20618;AC=2;AF=9.700261907071492e-05;N_HET=2;N_HOMALT=0;N_HOMREF=10307;FREQ_HET=0.00019400523814142983;FREQ_HOMALT=0.0;FREQ_HOMREF=0.9998059947618586;N_BI_GENOS=10309;Southeast_Han_AN=1908;Southeast_Han_AC=1;S
chr3	152462662	.	A	<INS:ME:LINE1>	.	PASS	SVTYPE=INS:ME:LINE1;END=152462676;SVLEN=988;AN=20618;AC=9;AF=0.0004365117858182171;N_HET=9;N_HOMALT=0;N_HOMREF=10300;FREQ_HET=0.0008730235716364342;FREQ_HOMALT=0.0;FREQ_HOMREF=0.9991269764283636;N_BI_GENOS=10309;North_Han_AN=2272;North_Han_AC=1;North_Han_
chr3	152707400	.	G	<INS:ME:LINE1>	.	PASS	SVTYPE=INS:ME:LINE1;END=152707415;SVLEN=1288;AN=20618;AC=1;AF=4.850130953535746e-05;N_HET=1;N_HOMALT=0;N_HOMREF=10308;FREQ_HET=9.700261907071492e-05;FREQ_HOMALT=0.0;FREQ_HOMREF=0.9999029973809292;N_BI_GENOS=10309;Northwest_Han_AN=1296;Northwest_Han_AC=1;N
chr3	152831196	.	T	<INS:ME:LINE1>	.	PASS	SVTYPE=INS:ME:LINE1;END=152831196;SVLEN=3972;AN=20618;AC=1;AF=4.850130953535746e-05;N_HET=1;N_HOMALT=0;N_HOMREF=10308;FREQ_HET=9.700261907071492e-05;FREQ_HOMALT=0.0;FREQ_HOMREF=0.9999029973809292;N_BI_GENOS=10309;Lingnan_Han_AN=1888;Lingnan_Han_AC=1;Lingn
chr3	152961194	.	A	<INS:ME:LINE1>	.	PASS	SVTYPE=INS:ME:LINE1;END=152961210;SVLEN=6034;AN=20618;AC=1;AF=4.850130953535746e-05;N_HET=1;N_HOMALT=0;N_HOMREF=10308;FREQ_HET=9.700261907071492e-05;FREQ_HOMALT=0.0;FREQ_HOMREF=0.9999029973809292;N_BI_GENOS=10309;South_Han_AN=1496;South_Han_AC=1;South_Han
chr3	152983528	.	A	<INS:ME:LINE1>	.	PASS	SVTYPE=INS:ME:LINE1;END=152983540;SVLEN=5971;AN=20618;AC=4;AF=0.00019400523814142983;N_HET=0;N_HOMALT=2;N_HOMREF=10307;FREQ_HET=0.0;FREQ_HOMALT=0.00019400523814142983;FREQ_HOMREF=0.9998059947618586;N_BI_GENOS=10309;North_Han_AN=2272;North_Han_AC=2;North_H
chr3	153350985	.	A	<INS:ME:LINE1>	.	PASS	SVTYPE=INS:ME:LINE1;END=153351000;SVLEN=6015;AN=20618;AC=2;AF=9.700261907071492e-05;N_HET=2;N_HOMALT=0;N_HOMREF=10307;FREQ_HET=0.00019400523814142983;FREQ_HOMALT=0.0;FREQ_HOMREF=0.9998059947618586;N_BI_GENOS=10309;Southeast_Han_AN=1908;Southeast_Han_AC=1;
chr3	153429940	.	A	<INS:ME:LINE1>	.	PASS	SVTYPE=INS:ME:LINE1;END=153429946;SVLEN=732;AN=20618;AC=3;AF=0.00014550392860607236;N_HET=3;N_HOMALT=0;N_HOMREF=10306;FREQ_HET=0.0002910078572121447;FREQ_HOMALT=0.0;FREQ_HOMREF=0.9997089921427879;N_BI_GENOS=10309;North_Han_AN=2272;North_Han_AC=1;North_Han
chr3	153578240	.	A	<INS:ME:LINE1>	.	PASS	SVTYPE=INS:ME:LINE1;END=153578255;SVLEN=5991;AN=20618;AC=4;AF=0.00019400523814142983;N_HET=4;N_HOMALT=0;N_HOMREF=10305;FREQ_HET=0.00038801047628285966;FREQ_HOMALT=0.0;FREQ_HOMREF=0.9996119895237171;N_BI_GENOS=10309;Lingnan_Han_AN=1888;Lingnan_Han_AC=1;Lin
chr3	153951897	.	A	<INS:ME:LINE1>	.	PASS	SVTYPE=INS:ME:LINE1;END=153951914;SVLEN=1945;AN=20618;AC=1;AF=4.850130953535746e-05;N_HET=1;N_HOMALT=0;N_HOMREF=10308;FREQ_HET=9.700261907071492e-05;FREQ_HOMALT=0.0;FREQ_HOMREF=0.9999029973809292;N_BI_GENOS=10309;East_Han_AN=6554;East_Han_AC=1;East_Han_AF
chr3	153955136	.	A	<INS:ME:LINE1>	.	PASS	SVTYPE=INS:ME:LINE1;END=153955136;SVLEN=240;AN=20618;AC=1;AF=4.850130953535746e-05;N_HET=1;N_HOMALT=0;N_HOMREF=10308;FREQ_HET=9.700261907071492e-05;FREQ_HOMALT=0.0;FREQ_HOMREF=0.9999029973809292;N_BI_GENOS=10309;Manchu_AN=416;Manchu_AC=1;Manchu_AF=0.00240
chr3	153961159	.	A	<INS:ME:LINE1>	.	PASS	SVTYPE=INS:ME:LINE1;END=153961160;SVLEN=1993;AN=20618;AC=1;AF=4.850130953535746e-05;N_HET=1;N_HOMALT=0;N_HOMREF=10308;FREQ_HET=9.700261907071492e-05;FREQ_HOMALT=0.0;FREQ_HOMREF=0.9999029973809292;N_BI_GENOS=10309;East_Han_AN=6554;East_Han_AC=1;East_Han_AF
chr3	154572191	.	A	<INS:ME:LINE1>	.	PASS	SVTYPE=INS:ME:LINE1;END=154572213;SVLEN=313;AN=20618;AC=1;AF=4.850130953535746e-05;N_HET=1;N_HOMALT=0;N_HOMREF=10308;FREQ_HET=9.700261907071492e-05;FREQ_HOMALT=0.0;FREQ_HOMREF=0.9999029973809292;N_BI_GENOS=10309;Southeast_Han_AN=1908;Southeast_Han_AC=1;So
chr3	154715909	.	A	<INS:ME:LINE1>	.	PASS	SVTYPE=INS:ME:LINE1;END=154715923;SVLEN=6000;AN=20618;AC=4;AF=0.00019400523814142983;N_HET=4;N_HOMALT=0;N_HOMREF=10305;FREQ_HET=0.00038801047628285966;FREQ_HOMALT=0.0;FREQ_HOMREF=0.9996119895237171;N_BI_GENOS=10309;Northwest_Han_AN=1296;Northwest_Han_AC=1
chr3	154796961	.	A	<INS:ME:LINE1>	.	PASS	SVTYPE=INS:ME:LINE1;END=154796975;SVLEN=2058;AN=20618;AC=2;AF=9.700261907071492e-05;N_HET=2;N_HOMALT=0;N_HOMREF=10307;FREQ_HET=0.00019400523814142983;FREQ_HOMALT=0.0;FREQ_HOMREF=0.9998059947618586;N_BI_GENOS=10309;North_Han_AN=2272;North_Han_AC=1;North_Ha
chr3	155148777	.	G	<INS:ME:LINE1>	.	PASS	SVTYPE=INS:ME:LINE1;END=155148788;SVLEN=573;AN=20618;AC=3;AF=0.00014550392860607236;N_HET=3;N_HOMALT=0;N_HOMREF=10306;FREQ_HET=0.0002910078572121447;FREQ_HOMALT=0.0;FREQ_HOMREF=0.9997089921427879;N_BI_GENOS=10309;East_Han_AN=6554;East_Han_AC=1;East_Han_AF
chr3	155182955	.	G	<INS:ME:LINE1>	.	PASS	SVTYPE=INS:ME:LINE1;END=155182958;SVLEN=337;AN=20618;AC=5;AF=0.00024250654767678728;N_HET=5;N_HOMALT=0;N_HOMREF=10304;FREQ_HET=0.00048501309535357455;FREQ_HOMALT=0.0;FREQ_HOMREF=0.9995149869046465;N_BI_GENOS=10309;South_Han_AN=1496;South_Han_AC=1;South_Ha
chr3	155674786	.	A	<INS:ME:LINE1>	.	PASS	SVTYPE=INS:ME:LINE1;END=155674802;SVLEN=5998;AN=20618;AC=1;AF=4.850130953535746e-05;N_HET=1;N_HOMALT=0;N_HOMREF=10308;FREQ_HET=9.700261907071492e-05;FREQ_HOMALT=0.0;FREQ_HOMREF=0.9999029973809292;N_BI_GENOS=10309;Tibetan_AN=414;Tibetan_AC=1;Tibetan_AF=0.0
chr3	155682689	.	A	<INS:ME:LINE1>	.	PASS	SVTYPE=INS:ME:LINE1;END=155682704;SVLEN=2461;AN=20618;AC=1;AF=4.850130953535746e-05;N_HET=1;N_HOMALT=0;N_HOMREF=10308;FREQ_HET=9.700261907071492e-05;FREQ_HOMALT=0.0;FREQ_HOMREF=0.9999029973809292;N_BI_GENOS=10309;Southeast_Han_AN=1908;Southeast_Han_AC=1;S
chr3	155693954	.	A	<INS:ME:LINE1>	.	PASS	SVTYPE=INS:ME:LINE1;END=155693968;SVLEN=6309;AN=20618;AC=1;AF=4.850130953535746e-05;N_HET=1;N_HOMALT=0;N_HOMREF=10308;FREQ_HET=9.700261907071492e-05;FREQ_HOMALT=0.0;FREQ_HOMREF=0.9999029973809292;N_BI_GENOS=10309;Lingnan_Han_AN=1888;Lingnan_Han_AC=1;Lingn
chr3	155762323	.	T	<INS:ME:LINE1>	.	PASS	SVTYPE=INS:ME:LINE1;END=155762338;SVLEN=5997;AN=20618;AC=1;AF=4.850130953535746e-05;N_HET=1;N_HOMALT=0;N_HOMREF=10308;FREQ_HET=9.700261907071492e-05;FREQ_HOMALT=0.0;FREQ_HOMREF=0.9999029973809292;N_BI_GENOS=10309;Central_Han_AN=2128;Central_Han_AC=1;Centr
chr3	155938935	.	A	<INS:ME:LINE1>	.	PASS	SVTYPE=INS:ME:LINE1;END=155938948;SVLEN=839;AN=20618;AC=44;AF=0.002134057619555728;N_HET=44;N_HOMALT=0;N_HOMREF=10265;FREQ_HET=0.004268115239111456;FREQ_HOMALT=0.0;FREQ_HOMREF=0.9957318847608886;N_BI_GENOS=10309;South_Han_AN=1496;South_Han_AC=3;South_Han_
chr3	156295368	.	A	<INS:ME:LINE1>	.	PASS	SVTYPE=INS:ME:LINE1;END=156295384;SVLEN=5997;AN=20618;AC=1;AF=4.850130953535746e-05;N_HET=1;N_HOMALT=0;N_HOMREF=10308;FREQ_HET=9.700261907071492e-05;FREQ_HOMALT=0.0;FREQ_HOMREF=0.9999029973809292;N_BI_GENOS=10309;North_Han_AN=2272;North_Han_AC=1;North_Han
chr3	156471630	.	T	<INS:ME:LINE1>	.	PASS	SVTYPE=INS:ME:LINE1;END=156471642;SVLEN=65;AN=20618;AC=1;AF=4.850130953535746e-05;N_HET=1;N_HOMALT=0;N_HOMREF=10308;FREQ_HET=9.700261907071492e-05;FREQ_HOMALT=0.0;FREQ_HOMREF=0.9999029973809292;N_BI_GENOS=10309;East_Han_AN=6554;East_Han_AC=1;East_Han_AF=0
chr3	156573309	.	A	<INS:ME:LINE1>	.	PASS	SVTYPE=INS:ME:LINE1;END=156573309;SVLEN=5999;AN=20618;AC=2;AF=9.700261907071492e-05;N_HET=2;N_HOMALT=0;N_HOMREF=10307;FREQ_HET=0.00019400523814142983;FREQ_HOMALT=0.0;FREQ_HOMREF=0.9998059947618586;N_BI_GENOS=10309;Northwest_Han_AN=1296;Northwest_Han_AC=1;
chr3	156857053	.	A	<INS:ME:LINE1>	.	PASS	SVTYPE=INS:ME:LINE1;END=156857069;SVLEN=676;AN=20618;AC=1;AF=4.850130953535746e-05;N_HET=1;N_HOMALT=0;N_HOMREF=10308;FREQ_HET=9.700261907071492e-05;FREQ_HOMALT=0.0;FREQ_HOMREF=0.9999029973809292;N_BI_GENOS=10309;Central_Han_AN=2128;Central_Han_AC=1;Centra
chr3	156858766	.	T	<INS:ME:LINE1>	.	PASS	SVTYPE=INS:ME:LINE1;END=156858782;SVLEN=1290;AN=20618;AC=29;AF=0.0014065379765253662;N_HET=29;N_HOMALT=0;N_HOMREF=10280;FREQ_HET=0.0028130759530507325;FREQ_HOMALT=0.0;FREQ_HOMREF=0.9971869240469493;N_BI_GENOS=10309;Northwest_Han_AN=1296;Northwest_Han_AC=3
chr3	156985671	.	A	<INS:ME:LINE1>	.	PASS	SVTYPE=INS:ME:LINE1;END=156985687;SVLEN=710;AN=20618;AC=1;AF=4.850130953535746e-05;N_HET=1;N_HOMALT=0;N_HOMREF=10308;FREQ_HET=9.700261907071492e-05;FREQ_HOMALT=0.0;FREQ_HOMREF=0.9999029973809292;N_BI_GENOS=10309;East_Han_AN=6554;East_Han_AC=1;East_Han_AF=
chr3	157274446	.	A	<INS:ME:LINE1>	.	PASS	SVTYPE=INS:ME:LINE1;END=157274446;SVLEN=549;AN=20618;AC=1;AF=4.850130953535746e-05;N_HET=1;N_HOMALT=0;N_HOMREF=10308;FREQ_HET=9.700261907071492e-05;FREQ_HOMALT=0.0;FREQ_HOMREF=0.9999029973809292;N_BI_GENOS=10309;Central_Han_AN=2128;Central_Han_AC=1;Centra

chr3	157460964	.	A	<INS:ME:LINE1>	.	PASS	SVTYPE=INS:ME:LINE1;END=157460976;SVLEN=6000;AN=20618;AC=1;AF=4.850130953535746e-05;N_HET=1;N_HOMALT=0;N_HOMREF=10308;FREQ_HET=9.700261907071492e-05;FREQ_HOMALT=0.0;FREQ_HOMREF=0.9999029973809292;N_BI_GENOS=10309;East_Han_AN=6554;East_Han_AC=1;East_Han_AF
chr3	157817838	.	A	<INS:ME:LINE1>	.	PASS	SVTYPE=INS:ME:LINE1;END=157817852;SVLEN=539;AN=20618;AC=1;AF=4.850130953535746e-05;N_HET=1;N_HOMALT=0;N_HOMREF=10308;FREQ_HET=9.700261907071492e-05;FREQ_HOMALT=0.0;FREQ_HOMREF=0.9999029973809292;N_BI_GENOS=10309;Tibetan_AN=414;Tibetan_AC=1;Tibetan_AF=0.00
chr3	157863272	.	A	<INS:ME:LINE1>	.	PASS	SVTYPE=INS:ME:LINE1;END=157863292;SVLEN=6231;AN=20618;AC=5;AF=0.00024250654767678728;N_HET=5;N_HOMALT=0;N_HOMREF=10304;FREQ_HET=0.00048501309535357455;FREQ_HOMALT=0.0;FREQ_HOMREF=0.9995149869046465;N_BI_GENOS=10309;South_Han_AN=1496;South_Han_AC=1;South_H
chr3	158064364	.	A	<INS:ME:LINE1>	.	PASS	SVTYPE=INS:ME:LINE1;END=158064376;SVLEN=1102;AN=20618;AC=31;AF=0.0015035405955960812;N_HET=31;N_HOMALT=0;N_HOMREF=10278;FREQ_HET=0.0030070811911921624;FREQ_HOMALT=0.0;FREQ_HOMREF=0.9969929188088078;N_BI_GENOS=10309;South_Han_AN=1496;South_Han_AC=2;South_H
chr3	158179387	.	T	<INS:ME:LINE1>	.	PASS	SVTYPE=INS:ME:LINE1;END=158179402;SVLEN=1254;AN=20618;AC=4;AF=0.00019400523814142983;N_HET=4;N_HOMALT=0;N_HOMREF=10305;FREQ_HET=0.00038801047628285966;FREQ_HOMALT=0.0;FREQ_HOMREF=0.9996119895237171;N_BI_GENOS=10309;South_Han_AN=1496;South_Han_AC=1;South_H
chr3	158405552	.	T	<INS:ME:LINE1>	.	PASS	SVTYPE=INS:ME:LINE1;END=158405555;SVLEN=105;AN=20618;AC=1;AF=4.850130953535746e-05;N_HET=1;N_HOMALT=0;N_HOMREF=10308;FREQ_HET=9.700261907071492e-05;FREQ_HOMALT=0.0;FREQ_HOMREF=0.9999029973809292;N_BI_GENOS=10309;Central_Han_AN=2128;Central_Han_AC=1;Centra
chr3	158530903	.	A	<INS:ME:LINE1>	.	PASS	SVTYPE=INS:ME:LINE1;END=158530903;SVLEN=17;AN=20618;AC=2;AF=9.700261907071492e-05;N_HET=2;N_HOMALT=0;N_HOMREF=10307;FREQ_HET=0.00019400523814142983;FREQ_HOMALT=0.0;FREQ_HOMREF=0.9998059947618586;N_BI_GENOS=10309;Northwest_Han_AN=1296;Northwest_Han_AC=1;No
chr3	158818292	.	A	<INS:ME:LINE1>	.	PASS	SVTYPE=INS:ME:LINE1;END=158818301;SVLEN=443;AN=20618;AC=3;AF=0.00014550392860607236;N_HET=3;N_HOMALT=0;N_HOMREF=10306;FREQ_HET=0.0002910078572121447;FREQ_HOMALT=0.0;FREQ_HOMREF=0.9997089921427879;N_BI_GENOS=10309;Miao_AN=482;Miao_AC=3;Miao_AF=0.0062240663
chr3	158829106	.	C	<INS:ME:LINE1>	.	PASS	SVTYPE=INS:ME:LINE1;END=158829122;SVLEN=328;AN=20618;AC=69;AF=0.0033465903579396644;N_HET=63;N_HOMALT=3;N_HOMREF=10243;FREQ_HET=0.006111165001455039;FREQ_HOMALT=0.0002910078572121447;FREQ_HOMREF=0.9935978271413328;N_BI_GENOS=10309;South_Han_AN=1496;South_
chr3	158849405	.	A	<INS:ME:LINE1>	.	PASS	SVTYPE=INS:ME:LINE1;END=158849422;SVLEN=612;AN=20618;AC=1;AF=4.850130953535746e-05;N_HET=1;N_HOMALT=0;N_HOMREF=10308;FREQ_HET=9.700261907071492e-05;FREQ_HOMALT=0.0;FREQ_HOMREF=0.9999029973809292;N_BI_GENOS=10309;Central_Han_AN=2128;Central_Han_AC=1;Centra
chr3	158864142	.	A	<INS:ME:LINE1>	.	PASS	SVTYPE=INS:ME:LINE1;END=158864142;SVLEN=5988;AN=20618;AC=1;AF=4.850130953535746e-05;N_HET=1;N_HOMALT=0;N_HOMREF=10308;FREQ_HET=9.700261907071492e-05;FREQ_HOMALT=0.0;FREQ_HOMREF=0.9999029973809292;N_BI_GENOS=10309;Mongolian_AN=454;Mongolian_AC=1;Mongolian_
chr3	159327150	.	T	<INS:ME:LINE1>	.	PASS	SVTYPE=INS:ME:LINE1;END=159327164;SVLEN=2513;AN=20618;AC=2;AF=9.700261907071492e-05;N_HET=2;N_HOMALT=0;N_HOMREF=10307;FREQ_HET=0.00019400523814142983;FREQ_HOMALT=0.0;FREQ_HOMREF=0.9998059947618586;N_BI_GENOS=10309;South_Han_AN=1496;South_Han_AC=1;South_Ha
chr3	159372990	.	A	<INS:ME:LINE1>	.	PASS	SVTYPE=INS:ME:LINE1;END=159373008;SVLEN=1543;AN=20618;AC=1;AF=4.850130953535746e-05;N_HET=1;N_HOMALT=0;N_HOMREF=10308;FREQ_HET=9.700261907071492e-05;FREQ_HOMALT=0.0;FREQ_HOMREF=0.9999029973809292;N_BI_GENOS=10309;East_Han_AN=6554;East_Han_AC=1;East_Han_AF
chr3	159473161	.	T	<INS:ME:LINE1>	.	PASS	SVTYPE=INS:ME:LINE1;END=159473175;SVLEN=5999;AN=20618;AC=3;AF=0.00014550392860607236;N_HET=3;N_HOMALT=0;N_HOMREF=10306;FREQ_HET=0.0002910078572121447;FREQ_HOMALT=0.0;FREQ_HOMREF=0.9997089921427879;N_BI_GENOS=10309;South_Han_AN=1496;South_Han_AC=2;South_Ha
chr3	159473896	.	G	<INS:ME:LINE1>	.	PASS	SVTYPE=INS:ME:LINE1;END=159473914;SVLEN=5960;AN=20618;AC=17;AF=0.0008245222621010767;N_HET=15;N_HOMALT=1;N_HOMREF=10293;FREQ_HET=0.0014550392860607237;FREQ_HOMALT=9.700261907071492e-05;FREQ_HOMREF=0.9984479580948685;N_BI_GENOS=10309;Lingnan_Han_AN=1888;Li
chr3	159552753	.	G	<INS:ME:LINE1>	.	PASS	SVTYPE=INS:ME:LINE1;END=159552762;SVLEN=1102;AN=20618;AC=13;AF=0.0006305170239596469;N_HET=13;N_HOMALT=0;N_HOMREF=10296;FREQ_HET=0.0012610340479192938;FREQ_HOMALT=0.0;FREQ_HOMREF=0.9987389659520807;N_BI_GENOS=10309;South_Han_AN=1496;South_Han_AC=1;South_H
chr3	159602408	.	T	<INS:ME:LINE1>	.	PASS	SVTYPE=INS:ME:LINE1;END=159602425;SVLEN=6000;AN=20618;AC=5;AF=0.00024250654767678728;N_HET=5;N_HOMALT=0;N_HOMREF=10304;FREQ_HET=0.00048501309535357455;FREQ_HOMALT=0.0;FREQ_HOMREF=0.9995149869046465;N_BI_GENOS=10309;Lingnan_Han_AN=1888;Lingnan_Han_AC=1;Lin
chr3	160032311	.	G	<INS:ME:LINE1>	.	PASS	SVTYPE=INS:ME:LINE1;END=160032319;SVLEN=690;AN=20618;AC=5;AF=0.00024250654767678728;N_HET=5;N_HOMALT=0;N_HOMREF=10304;FREQ_HET=0.00048501309535357455;FREQ_HOMALT=0.0;FREQ_HOMREF=0.9995149869046465;N_BI_GENOS=10309;South_Han_AN=1496;South_Han_AC=2;South_Ha
chr3	160251320	.	A	<INS:ME:LINE1>	.	PASS	SVTYPE=INS:ME:LINE1;END=160251334;SVLEN=381;AN=20618;AC=6;AF=0.0002910078572121447;N_HET=6;N_HOMALT=0;N_HOMREF=10303;FREQ_HET=0.0005820157144242894;FREQ_HOMALT=0.0;FREQ_HOMREF=0.9994179842855757;N_BI_GENOS=10309;North_Han_AN=2272;North_Han_AC=1;North_Han_
chr3	160271288	.	A	<INS:ME:LINE1>	.	PASS	SVTYPE=INS:ME:LINE1;END=160271288;SVLEN=769;AN=20618;AC=1;AF=4.850130953535746e-05;N_HET=1;N_HOMALT=0;N_HOMREF=10308;FREQ_HET=9.700261907071492e-05;FREQ_HOMALT=0.0;FREQ_HOMREF=0.9999029973809292;N_BI_GENOS=10309;East_Han_AN=6554;East_Han_AC=1;East_Han_AF=
chr3	160280287	.	T	<INS:ME:LINE1>	.	PASS	SVTYPE=INS:ME:LINE1;END=160280289;SVLEN=1121;AN=20618;AC=2;AF=9.700261907071492e-05;N_HET=2;N_HOMALT=0;N_HOMREF=10307;FREQ_HET=0.00019400523814142983;FREQ_HOMALT=0.0;FREQ_HOMREF=0.9998059947618586;N_BI_GENOS=10309;South_Han_AN=1496;South_Han_AC=1;South_Ha
chr3	160602344	.	T	<INS:ME:LINE1>	.	PASS	SVTYPE=INS:ME:LINE1;END=160602360;SVLEN=3996;AN=20618;AC=2;AF=9.700261907071492e-05;N_HET=2;N_HOMALT=0;N_HOMREF=10307;FREQ_HET=0.00019400523814142983;FREQ_HOMALT=0.0;FREQ_HOMREF=0.9998059947618586;N_BI_GENOS=10309;East_Han_AN=6554;East_Han_AC=1;East_Han_A
chr3	160708546	.	C	<INS:ME:LINE1>	.	PASS	SVTYPE=INS:ME:LINE1;END=160708563;SVLEN=628;AN=20618;AC=1;AF=4.850130953535746e-05;N_HET=1;N_HOMALT=0;N_HOMREF=10308;FREQ_HET=9.700261907071492e-05;FREQ_HOMALT=0.0;FREQ_HOMREF=0.9999029973809292;N_BI_GENOS=10309;Southeast_Han_AN=1908;Southeast_Han_AC=1;So
chr3	160943713	.	A	<INS:ME:LINE1>	.	PASS	SVTYPE=INS:ME:LINE1;END=160943729;SVLEN=734;AN=20618;AC=1;AF=4.850130953535746e-05;N_HET=1;N_HOMALT=0;N_HOMREF=10308;FREQ_HET=9.700261907071492e-05;FREQ_HOMALT=0.0;FREQ_HOMREF=0.9999029973809292;N_BI_GENOS=10309;East_Han_AN=6554;East_Han_AC=1;East_Han_AF=
chr3	161009235	.	C	<INS:ME:LINE1>	.	PASS	SVTYPE=INS:ME:LINE1;END=161009248;SVLEN=1137;AN=20618;AC=6;AF=0.0002910078572121447;N_HET=6;N_HOMALT=0;N_HOMREF=10303;FREQ_HET=0.0005820157144242894;FREQ_HOMALT=0.0;FREQ_HOMREF=0.9994179842855757;N_BI_GENOS=10309;East_Han_AN=6554;East_Han_AC=5;East_Han_AF
chr3	161332754	.	A	<INS:ME:LINE1>	.	PASS	SVTYPE=INS:ME:LINE1;END=161332772;SVLEN=2989;AN=20618;AC=124;AF=0.006014162382384325;N_HET=120;N_HOMALT=2;N_HOMREF=10187;FREQ_HET=0.01164031428848579;FREQ_HOMALT=0.00019400523814142983;FREQ_HOMREF=0.9881656804733728;N_BI_GENOS=10309;South_Han_AN=1496;Sout
chr3	161396175	.	C	<INS:ME:LINE1>	.	PASS	SVTYPE=INS:ME:LINE1;END=161396190;SVLEN=5998;AN=20618;AC=1;AF=4.850130953535746e-05;N_HET=1;N_HOMALT=0;N_HOMREF=10308;FREQ_HET=9.700261907071492e-05;FREQ_HOMALT=0.0;FREQ_HOMREF=0.9999029973809292;N_BI_GENOS=10309;East_Han_AN=6554;East_Han_AC=1;East_Han_AF
chr3	162202209	.	A	<INS:ME:LINE1>	.	PASS	SVTYPE=INS:ME:LINE1;END=162202224;SVLEN=2544;AN=20618;AC=10;AF=0.00048501309535357455;N_HET=10;N_HOMALT=0;N_HOMREF=10299;FREQ_HET=0.0009700261907071491;FREQ_HOMALT=0.0;FREQ_HOMREF=0.9990299738092928;N_BI_GENOS=10309;South_Han_AN=1496;South_Han_AC=1;South_
chr3	162323018	.	A	<INS:ME:LINE1>	.	PASS	SVTYPE=INS:ME:LINE1;END=162323033;SVLEN=2761;AN=20618;AC=2;AF=9.700261907071492e-05;N_HET=2;N_HOMALT=0;N_HOMREF=10307;FREQ_HET=0.00019400523814142983;FREQ_HOMALT=0.0;FREQ_HOMREF=0.9998059947618586;N_BI_GENOS=10309;Northwest_Han_AN=1296;Northwest_Han_AC=2;
chr3	162426811	.	T	<INS:ME:LINE1>	.	PASS	SVTYPE=INS:ME:LINE1;END=162426817;SVLEN=1402;AN=20618;AC=1;AF=4.850130953535746e-05;N_HET=1;N_HOMALT=0;N_HOMREF=10308;FREQ_HET=9.700261907071492e-05;FREQ_HOMALT=0.0;FREQ_HOMREF=0.9999029973809292;N_BI_GENOS=10309;Southeast_Han_AN=1908;Southeast_Han_AC=1;S
chr3	162726442	.	C	<INS:ME:LINE1>	.	PASS	SVTYPE=INS:ME:LINE1;END=162726442;SVLEN=0;AN=20618;AC=5;AF=0.00024250654767678728;N_HET=5;N_HOMALT=0;N_HOMREF=10304;FREQ_HET=0.00048501309535357455;FREQ_HOMALT=0.0;FREQ_HOMREF=0.9995149869046465;N_BI_GENOS=10309;South_Han_AN=1496;South_Han_AC=1;South_Han_
chr3	162788690	.	A	<INS:ME:LINE1>	.	PASS	SVTYPE=INS:ME:LINE1;END=162788702;SVLEN=431;AN=20618;AC=1;AF=4.850130953535746e-05;N_HET=1;N_HOMALT=0;N_HOMREF=10308;FREQ_HET=9.700261907071492e-05;FREQ_HOMALT=0.0;FREQ_HOMREF=0.9999029973809292;N_BI_GENOS=10309;Northwest_Han_AN=1296;Northwest_Han_AC=1;No
chr3	162873951	.	T	<INS:ME:LINE1>	.	PASS	SVTYPE=INS:ME:LINE1;END=162873956;SVLEN=387;AN=20618;AC=2;AF=9.700261907071492e-05;N_HET=0;N_HOMALT=1;N_HOMREF=10308;FREQ_HET=0.0;FREQ_HOMALT=9.700261907071492e-05;FREQ_HOMREF=0.9999029973809292;N_BI_GENOS=10309;Tibetan_AN=414;Tibetan_AC=2;Tibetan_AF=0.00
chr3	162956396	.	A	<INS:ME:LINE1>	.	PASS	SVTYPE=INS:ME:LINE1;END=162956413;SVLEN=4125;AN=20618;AC=1;AF=4.850130953535746e-05;N_HET=1;N_HOMALT=0;N_HOMREF=10308;FREQ_HET=9.700261907071492e-05;FREQ_HOMALT=0.0;FREQ_HOMREF=0.9999029973809292;N_BI_GENOS=10309;North_Han_AN=2272;North_Han_AC=1;North_Han
chr3	162999518	.	A	<INS:ME:LINE1>	.	PASS	SVTYPE=INS:ME:LINE1;END=162999518;SVLEN=305;AN=20618;AC=43;AF=0.0020855563100203706;N_HET=13;N_HOMALT=15;N_HOMREF=10281;FREQ_HET=0.0012610340479192938;FREQ_HOMALT=0.0014550392860607237;FREQ_HOMREF=0.99728392666602;N_BI_GENOS=10309;South_Han_AN=1496;South_
chr3	163086420	.	T	<INS:ME:LINE1>	.	PASS	SVTYPE=INS:ME:LINE1;END=163086426;SVLEN=1140;AN=20618;AC=53;AF=0.002570569405373945;N_HET=43;N_HOMALT=5;N_HOMREF=10261;FREQ_HET=0.004171112620040741;FREQ_HOMALT=0.00048501309535357455;FREQ_HOMREF=0.9953438742846057;N_BI_GENOS=10309;South_Han_AN=1496;South
chr3	163159627	.	A	<INS:ME:LINE1>	.	PASS	SVTYPE=INS:ME:LINE1;END=163159641;SVLEN=1094;AN=20618;AC=2;AF=9.700261907071492e-05;N_HET=2;N_HOMALT=0;N_HOMREF=10307;FREQ_HET=0.00019400523814142983;FREQ_HOMALT=0.0;FREQ_HOMREF=0.9998059947618586;N_BI_GENOS=10309;Southeast_Han_AN=1908;Southeast_Han_AC=2;
chr3	163320951	.	A	<INS:ME:LINE1>	.	PASS	SVTYPE=INS:ME:LINE1;END=163320956;SVLEN=263;AN=20618;AC=1;AF=4.850130953535746e-05;N_HET=1;N_HOMALT=0;N_HOMREF=10308;FREQ_HET=9.700261907071492e-05;FREQ_HOMALT=0.0;FREQ_HOMREF=0.9999029973809292;N_BI_GENOS=10309;South_Han_AN=1496;South_Han_AC=1;South_Han_
chr3	163389532	.	T	<INS:ME:LINE1>	.	PASS	SVTYPE=INS:ME:LINE1;END=163389547;SVLEN=6024;AN=20618;AC=1;AF=4.850130953535746e-05;N_HET=1;N_HOMALT=0;N_HOMREF=10308;FREQ_HET=9.700261907071492e-05;FREQ_HOMALT=0.0;FREQ_HOMREF=0.9999029973809292;N_BI_GENOS=10309;Manchu_AN=416;Manchu_AC=1;Manchu_AF=0.0024
chr3	163623691	.	G	<INS:ME:LINE1>	.	PASS	SVTYPE=INS:ME:LINE1;END=163623700;SVLEN=184;AN=20618;AC=1;AF=4.850130953535746e-05;N_HET=1;N_HOMALT=0;N_HOMREF=10308;FREQ_HET=9.700261907071492e-05;FREQ_HOMALT=0.0;FREQ_HOMREF=0.9999029973809292;N_BI_GENOS=10309;East_Han_AN=6554;East_Han_AC=1;East_Han_AF=
chr3	163755375	.	T	<INS:ME:LINE1>	.	PASS	SVTYPE=INS:ME:LINE1;END=163755375;SVLEN=939;AN=20618;AC=1;AF=4.850130953535746e-05;N_HET=1;N_HOMALT=0;N_HOMREF=10308;FREQ_HET=9.700261907071492e-05;FREQ_HOMALT=0.0;FREQ_HOMREF=0.9999029973809292;N_BI_GENOS=10309;East_Han_AN=6554;East_Han_AC=1;East_Han_AF=
chr3	163868539	.	T	<INS:ME:LINE1>	.	PASS	SVTYPE=INS:ME:LINE1;END=163868555;SVLEN=1032;AN=20618;AC=1;AF=4.850130953535746e-05;N_HET=1;N_HOMALT=0;N_HOMREF=10308;FREQ_HET=9.700261907071492e-05;FREQ_HOMALT=0.0;FREQ_HOMREF=0.9999029973809292;N_BI_GENOS=10309;North_Han_AN=2272;North_Han_AC=1;North_Han
chr3	163929256	.	A	<INS:ME:LINE1>	.	PASS	SVTYPE=INS:ME:LINE1;END=163929270;SVLEN=857;AN=20618;AC=1;AF=4.850130953535746e-05;N_HET=1;N_HOMALT=0;N_HOMREF=10308;FREQ_HET=9.700261907071492e-05;FREQ_HOMALT=0.0;FREQ_HOMREF=0.9999029973809292;N_BI_GENOS=10309;North_Han_AN=2272;North_Han_AC=1;North_Han_
chr3	163986211	.	T	<INS:ME:LINE1>	.	PASS	SVTYPE=INS:ME:LINE1;END=163986237;SVLEN=2934;AN=20618;AC=1;AF=4.850130953535746e-05;N_HET=1;N_HOMALT=0;N_HOMREF=10308;FREQ_HET=9.700261907071492e-05;FREQ_HOMALT=0.0;FREQ_HOMREF=0.9999029973809292;N_BI_GENOS=10309;South_Han_AN=1496;South_Han_AC=1;South_Han
chr3	163996561	.	G	<INS:ME:LINE1>	.	PASS	SVTYPE=INS:ME:LINE1;END=163996561;SVLEN=1356;AN=20618;AC=7;AF=0.0003395091667475022;N_HET=7;N_HOMALT=0;N_HOMREF=10302;FREQ_HET=0.0006790183334950044;FREQ_HOMALT=0.0;FREQ_HOMREF=0.999320981666505;N_BI_GENOS=10309;Lingnan_Han_AN=1888;Lingnan_Han_AC=1;Lingna
chr3	164070514	.	A	<INS:ME:LINE1>	.	PASS	SVTYPE=INS:ME:LINE1;END=164070514;SVLEN=76;AN=20618;AC=2;AF=9.700261907071492e-05;N_HET=2;N_HOMALT=0;N_HOMREF=10307;FREQ_HET=0.00019400523814142983;FREQ_HOMALT=0.0;FREQ_HOMREF=0.9998059947618586;N_BI_GENOS=10309;East_Han_AN=6554;East_Han_AC=1;East_Han_AF=
chr3	164245031	.	T	<INS:ME:LINE1>	.	PASS	SVTYPE=INS:ME:LINE1;END=164245047;SVLEN=385;AN=20618;AC=1;AF=4.850130953535746e-05;N_HET=1;N_HOMALT=0;N_HOMREF=10308;FREQ_HET=9.700261907071492e-05;FREQ_HOMALT=0.0;FREQ_HOMREF=0.9999029973809292;N_BI_GENOS=10309;Southeast_Han_AN=1908;Southeast_Han_AC=1;So
chr3	165020741	.	T	<INS:ME:LINE1>	.	PASS	SVTYPE=INS:ME:LINE1;END=165020745;SVLEN=334;AN=20618;AC=6;AF=0.0002910078572121447;N_HET=6;N_HOMALT=0;N_HOMREF=10303;FREQ_HET=0.0005820157144242894;FREQ_HOMALT=0.0;FREQ_HOMREF=0.9994179842855757;N_BI_GENOS=10309;South_Han_AN=1496;South_Han_AC=2;South_Han_
chr3	165149777	.	A	<INS:ME:LINE1>	.	PASS	SVTYPE=INS:ME:LINE1;END=165149793;SVLEN=6000;AN=20618;AC=1;AF=4.850130953535746e-05;N_HET=1;N_HOMALT=0;N_HOMREF=10308;FREQ_HET=9.700261907071492e-05;FREQ_HOMALT=0.0;FREQ_HOMREF=0.9999029973809292;N_BI_GENOS=10309;North_Han_AN=2272;North_Han_AC=1;North_Han
chr3	165215424	.	G	<INS:ME:LINE1>	.	PASS	SVTYPE=INS:ME:LINE1;END=165215424;SVLEN=320;AN=20618;AC=2;AF=9.700261907071492e-05;N_HET=0;N_HOMALT=1;N_HOMREF=10308;FREQ_HET=0.0;FREQ_HOMALT=9.700261907071492e-05;FREQ_HOMREF=0.9999029973809292;N_BI_GENOS=10309;Lingnan_Han_AN=1888;Lingnan_Han_AC=2;Lingna
chr3	165234168	.	G	<INS:ME:LINE1>	.	PASS	SVTYPE=INS:ME:LINE1;END=165234182;SVLEN=617;AN=20618;AC=2;AF=9.700261907071492e-05;N_HET=2;N_HOMALT=0;N_HOMREF=10307;FREQ_HET=0.00019400523814142983;FREQ_HOMALT=0.0;FREQ_HOMREF=0.9998059947618586;N_BI_GENOS=10309;East_Han_AN=6554;East_Han_AC=2;East_Han_AF
chr3	165290893	.	T	<INS:ME:LINE1>	.	PASS	SVTYPE=INS:ME:LINE1;END=165290893;SVLEN=91;AN=20618;AC=1;AF=4.850130953535746e-05;N_HET=1;N_HOMALT=0;N_HOMREF=10308;FREQ_HET=9.700261907071492e-05;FREQ_HOMALT=0.0;FREQ_HOMREF=0.9999029973809292;N_BI_GENOS=10309;East_Han_AN=6554;East_Han_AC=1;East_Han_AF=0
chr3	165463439	.	A	<INS:ME:LINE1>	.	PASS	SVTYPE=INS:ME:LINE1;END=165463452;SVLEN=600;AN=20618;AC=1;AF=4.850130953535746e-05;N_HET=1;N_HOMALT=0;N_HOMREF=10308;FREQ_HET=9.700261907071492e-05;FREQ_HOMALT=0.0;FREQ_HOMREF=0.9999029973809292;N_BI_GENOS=10309;Southeast_Han_AN=1908;Southeast_Han_AC=1;So
chr3	165596421	.	A	<INS:ME:LINE1>	.	PASS	SVTYPE=INS:ME:LINE1;END=165596438;SVLEN=326;AN=20618;AC=31;AF=0.0015035405955960812;N_HET=29;N_HOMALT=1;N_HOMREF=10279;FREQ_HET=0.0028130759530507325;FREQ_HOMALT=9.700261907071492e-05;FREQ_HOMREF=0.9970899214278786;N_BI_GENOS=10309;South_Han_AN=1496;South
chr3	165834846	.	A	<INS:ME:LINE1>	.	PASS	SVTYPE=INS:ME:LINE1;END=165834846;SVLEN=65;AN=20618;AC=1;AF=4.850130953535746e-05;N_HET=1;N_HOMALT=0;N_HOMREF=10308;FREQ_HET=9.700261907071492e-05;FREQ_HOMALT=0.0;FREQ_HOMREF=0.9999029973809292;N_BI_GENOS=10309;East_Han_AN=6554;East_Han_AC=1;East_Han_AF=0
chr3	166010841	.	A	<INS:ME:LINE1>	.	PASS	SVTYPE=INS:ME:LINE1;END=166010841;SVLEN=210;AN=20618;AC=1;AF=4.850130953535746e-05;N_HET=1;N_HOMALT=0;N_HOMREF=10308;FREQ_HET=9.700261907071492e-05;FREQ_HOMALT=0.0;FREQ_HOMREF=0.9999029973809292;N_BI_GENOS=10309;East_Han_AN=6554;East_Han_AC=1;East_Han_AF=
chr3	166043933	.	T	<INS:ME:LINE1>	.	PASS	SVTYPE=INS:ME:LINE1;END=166043949;SVLEN=479;AN=20618;AC=1;AF=4.850130953535746e-05;N_HET=1;N_HOMALT=0;N_HOMREF=10308;FREQ_HET=9.700261907071492e-05;FREQ_HOMALT=0.0;FREQ_HOMREF=0.9999029973809292;N_BI_GENOS=10309;East_Han_AN=6554;East_Han_AC=1;East_Han_AF=
chr3	166178463	.	G	<INS:ME:LINE1>	.	PASS	SVTYPE=INS:ME:LINE1;END=166178464;SVLEN=5991;AN=20618;AC=4;AF=0.00019400523814142983;N_HET=4;N_HOMALT=0;N_HOMREF=10305;FREQ_HET=0.00038801047628285966;FREQ_HOMALT=0.0;FREQ_HOMREF=0.9996119895237171;N_BI_GENOS=10309;North_Han_AN=2272;North_Han_AC=2;North_H
chr3	166196239	.	C	<INS:ME:LINE1>	.	PASS	SVTYPE=INS:ME:LINE1;END=166196256;SVLEN=1425;AN=20618;AC=26;AF=0.0012610340479192938;N_HET=26;N_HOMALT=0;N_HOMREF=10283;FREQ_HET=0.0025220680958385876;FREQ_HOMALT=0.0;FREQ_HOMREF=0.9974779319041615;N_BI_GENOS=10309;South_Han_AN=1496;South_Han_AC=1;South_H
chr3	166548383	.	C	<INS:ME:LINE1>	.	PASS	SVTYPE=INS:ME:LINE1;END=166548460;SVLEN=1234;AN=20618;AC=1;AF=4.850130953535746e-05;N_HET=1;N_HOMALT=0;N_HOMREF=10308;FREQ_HET=9.700261907071492e-05;FREQ_HOMALT=0.0;FREQ_HOMREF=0.9999029973809292;N_BI_GENOS=10309;East_Han_AN=6554;East_Han_AC=1;East_Han_AF
chr3	166580619	.	T	<INS:ME:LINE1>	.	PASS	SVTYPE=INS:ME:LINE1;END=166580634;SVLEN=325;AN=20618;AC=1;AF=4.850130953535746e-05;N_HET=1;N_HOMALT=0;N_HOMREF=10308;FREQ_HET=9.700261907071492e-05;FREQ_HOMALT=0.0;FREQ_HOMREF=0.9999029973809292;N_BI_GENOS=10309;East_Han_AN=6554;East_Han_AC=1;East_Han_AF=
chr3	166582446	.	C	<INS:ME:LINE1>	.	PASS	SVTYPE=INS:ME:LINE1;END=166582446;SVLEN=102;AN=20618;AC=1;AF=4.850130953535746e-05;N_HET=1;N_HOMALT=0;N_HOMREF=10308;FREQ_HET=9.700261907071492e-05;FREQ_HOMALT=0.0;FREQ_HOMREF=0.9999029973809292;N_BI_GENOS=10309;East_Han_AN=6554;East_Han_AC=1;East_Han_AF=
chr3	166583177	.	G	<INS:ME:LINE1>	.	PASS	SVTYPE=INS:ME:LINE1;END=166583177;SVLEN=1003;AN=20618;AC=1;AF=4.850130953535746e-05;N_HET=1;N_HOMALT=0;N_HOMREF=10308;FREQ_HET=9.700261907071492e-05;FREQ_HOMALT=0.0;FREQ_HOMREF=0.9999029973809292;N_BI_GENOS=10309;East_Han_AN=6554;East_Han_AC=1;East_Han_AF
chr3	166640380	.	T	<INS:ME:LINE1>	.	PASS	SVTYPE=INS:ME:LINE1;END=166640390;SVLEN=1083;AN=20618;AC=1;AF=4.850130953535746e-05;N_HET=1;N_HOMALT=0;N_HOMREF=10308;FREQ_HET=9.700261907071492e-05;FREQ_HOMALT=0.0;FREQ_HOMREF=0.9999029973809292;N_BI_GENOS=10309;Central_Han_AN=2128;Central_Han_AC=1;Centr
chr3	166648734	.	A	<INS:ME:LINE1>	.	PASS	SVTYPE=INS:ME:LINE1;END=166648739;SVLEN=698;AN=20618;AC=1;AF=4.850130953535746e-05;N_HET=1;N_HOMALT=0;N_HOMREF=10308;FREQ_HET=9.700261907071492e-05;FREQ_HOMALT=0.0;FREQ_HOMREF=0.9999029973809292;N_BI_GENOS=10309;East_Han_AN=6554;East_Han_AC=1;East_Han_AF=
chr3	166778884	.	T	<INS:ME:LINE1>	.	PASS	SVTYPE=INS:ME:LINE1;END=166778884;SVLEN=196;AN=20618;AC=1;AF=4.850130953535746e-05;N_HET=1;N_HOMALT=0;N_HOMREF=10308;FREQ_HET=9.700261907071492e-05;FREQ_HOMALT=0.0;FREQ_HOMREF=0.9999029973809292;N_BI_GENOS=10309;East_Han_AN=6554;East_Han_AC=1;East_Han_AF=
chr3	166827862	.	C	<INS:ME:LINE1>	.	PASS	SVTYPE=INS:ME:LINE1;END=166827862;SVLEN=0;AN=20618;AC=1;AF=4.850130953535746e-05;N_HET=1;N_HOMALT=0;N_HOMREF=10308;FREQ_HET=9.700261907071492e-05;FREQ_HOMALT=0.0;FREQ_HOMREF=0.9999029973809292;N_BI_GENOS=10309;Central_Han_AN=2128;Central_Han_AC=1;Central_
chr3	166867976	.	A	<INS:ME:LINE1>	.	PASS	SVTYPE=INS:ME:LINE1;END=166867976;SVLEN=68;AN=20618;AC=1;AF=4.850130953535746e-05;N_HET=1;N_HOMALT=0;N_HOMREF=10308;FREQ_HET=9.700261907071492e-05;FREQ_HOMALT=0.0;FREQ_HOMREF=0.9999029973809292;N_BI_GENOS=10309;North_Han_AN=2272;North_Han_AC=1;North_Han_A
chr3	166868086	.	A	<INS:ME:LINE1>	.	PASS	SVTYPE=INS:ME:LINE1;END=166868102;SVLEN=343;AN=20618;AC=1;AF=4.850130953535746e-05;N_HET=1;N_HOMALT=0;N_HOMREF=10308;FREQ_HET=9.700261907071492e-05;FREQ_HOMALT=0.0;FREQ_HOMREF=0.9999029973809292;N_BI_GENOS=10309;East_Han_AN=6554;East_Han_AC=1;East_Han_AF=
chr3	166939633	.	T	<INS:ME:LINE1>	.	PASS	SVTYPE=INS:ME:LINE1;END=166939633;SVLEN=238;AN=20618;AC=1;AF=4.850130953535746e-05;N_HET=1;N_HOMALT=0;N_HOMREF=10308;FREQ_HET=9.700261907071492e-05;FREQ_HOMALT=0.0;FREQ_HOMREF=0.9999029973809292;N_BI_GENOS=10309;East_Han_AN=6554;East_Han_AC=1;East_Han_AF=
chr3	167023208	.	A	<INS:ME:LINE1>	.	PASS	SVTYPE=INS:ME:LINE1;END=167023219;SVLEN=6000;AN=20618;AC=1;AF=4.850130953535746e-05;N_HET=1;N_HOMALT=0;N_HOMREF=10308;FREQ_HET=9.700261907071492e-05;FREQ_HOMALT=0.0;FREQ_HOMREF=0.9999029973809292;N_BI_GENOS=10309;Central_Han_AN=2128;Central_Han_AC=1;Centr
chr3	167053676	.	A	<INS:ME:LINE1>	.	PASS	SVTYPE=INS:ME:LINE1;END=167053692;SVLEN=2497;AN=20618;AC=2;AF=9.700261907071492e-05;N_HET=2;N_HOMALT=0;N_HOMREF=10307;FREQ_HET=0.00019400523814142983;FREQ_HOMALT=0.0;FREQ_HOMREF=0.9998059947618586;N_BI_GENOS=10309;Southeast_Han_AN=1908;Southeast_Han_AC=2;
chr3	167245354	.	T	<INS:ME:LINE1>	.	PASS	SVTYPE=INS:ME:LINE1;END=167245369;SVLEN=6049;AN=20618;AC=1;AF=4.850130953535746e-05;N_HET=1;N_HOMALT=0;N_HOMREF=10308;FREQ_HET=9.700261907071492e-05;FREQ_HOMALT=0.0;FREQ_HOMREF=0.9999029973809292;N_BI_GENOS=10309;Lingnan_Han_AN=1888;Lingnan_Han_AC=1;Lingn
chr3	167547408	.	A	<INS:ME:LINE1>	.	PASS	SVTYPE=INS:ME:LINE1;END=167547421;SVLEN=3476;AN=20618;AC=92;AF=0.004462120477252886;N_HET=90;N_HOMALT=1;N_HOMREF=10218;FREQ_HET=0.008730235716364342;FREQ_HOMALT=9.700261907071492e-05;FREQ_HOMREF=0.9911727616645649;N_BI_GENOS=10309;South_Han_AN=1496;South_
chr3	167592058	.	T	<INS:ME:LINE1>	.	PASS	SVTYPE=INS:ME:LINE1;END=167592074;SVLEN=5993;AN=20618;AC=1;AF=4.850130953535746e-05;N_HET=1;N_HOMALT=0;N_HOMREF=10308;FREQ_HET=9.700261907071492e-05;FREQ_HOMALT=0.0;FREQ_HOMREF=0.9999029973809292;N_BI_GENOS=10309;Hui_AN=444;Hui_AC=1;Hui_AF=0.0022522522522
chr3	167603685	.	T	<INS:ME:LINE1>	.	PASS	SVTYPE=INS:ME:LINE1;END=167603704;SVLEN=6005;AN=20618;AC=1;AF=4.850130953535746e-05;N_HET=1;N_HOMALT=0;N_HOMREF=10308;FREQ_HET=9.700261907071492e-05;FREQ_HOMALT=0.0;FREQ_HOMREF=0.9999029973809292;N_BI_GENOS=10309;Central_Han_AN=2128;Central_Han_AC=1;Centr
chr3	167770190	.	G	<INS:ME:LINE1>	.	PASS	SVTYPE=INS:ME:LINE1;END=167770204;SVLEN=5994;AN=20618;AC=1;AF=4.850130953535746e-05;N_HET=1;N_HOMALT=0;N_HOMREF=10308;FREQ_HET=9.700261907071492e-05;FREQ_HOMALT=0.0;FREQ_HOMREF=0.9999029973809292;N_BI_GENOS=10309;Northwest_Han_AN=1296;Northwest_Han_AC=1;N
chr3	167846373	.	T	<INS:ME:LINE1>	.	PASS	SVTYPE=INS:ME:LINE1;END=167846388;SVLEN=970;AN=20618;AC=3;AF=0.00014550392860607236;N_HET=3;N_HOMALT=0;N_HOMREF=10306;FREQ_HET=0.0002910078572121447;FREQ_HOMALT=0.0;FREQ_HOMREF=0.9997089921427879;N_BI_GENOS=10309;Lingnan_Han_AN=1888;Lingnan_Han_AC=1;Lingn
chr3	167908640	.	A	<INS:ME:LINE1>	.	PASS	SVTYPE=INS:ME:LINE1;END=167908653;SVLEN=6014;AN=20618;AC=1;AF=4.850130953535746e-05;N_HET=1;N_HOMALT=0;N_HOMREF=10308;FREQ_HET=9.700261907071492e-05;FREQ_HOMALT=0.0;FREQ_HOMREF=0.9999029973809292;N_BI_GENOS=10309;Southeast_Han_AN=1908;Southeast_Han_AC=1;S
chr3	167969965	.	T	<INS:ME:LINE1>	.	PASS	SVTYPE=INS:ME:LINE1;END=167969965;SVLEN=656;AN=20618;AC=1;AF=4.850130953535746e-05;N_HET=1;N_HOMALT=0;N_HOMREF=10308;FREQ_HET=9.700261907071492e-05;FREQ_HOMALT=0.0;FREQ_HOMREF=0.9999029973809292;N_BI_GENOS=10309;East_Han_AN=6554;East_Han_AC=1;East_Han_AF=
chr3	168008971	.	A	<INS:ME:LINE1>	.	PASS	SVTYPE=INS:ME:LINE1;END=168008988;SVLEN=6001;AN=20618;AC=1;AF=4.850130953535746e-05;N_HET=1;N_HOMALT=0;N_HOMREF=10308;FREQ_HET=9.700261907071492e-05;FREQ_HOMALT=0.0;FREQ_HOMREF=0.9999029973809292;N_BI_GENOS=10309;East_Han_AN=6554;East_Han_AC=1;East_Han_AF
chr3	168607778	.	A	<INS:ME:LINE1>	.	PASS	SVTYPE=INS:ME:LINE1;END=168607791;SVLEN=1549;AN=20618;AC=4;AF=0.00019400523814142983;N_HET=4;N_HOMALT=0;N_HOMREF=10305;FREQ_HET=0.00038801047628285966;FREQ_HOMALT=0.0;FREQ_HOMREF=0.9996119895237171;N_BI_GENOS=10309;South_Han_AN=1496;South_Han_AC=1;South_H
chr3	169232314	.	A	<INS:ME:LINE1>	.	PASS	SVTYPE=INS:ME:LINE1;END=169232319;SVLEN=227;AN=20618;AC=2;AF=9.700261907071492e-05;N_HET=2;N_HOMALT=0;N_HOMREF=10307;FREQ_HET=0.00019400523814142983;FREQ_HOMALT=0.0;FREQ_HOMREF=0.9998059947618586;N_BI_GENOS=10309;South_Han_AN=1496;South_Han_AC=1;South_Han
chr3	169379441	.	A	<INS:ME:LINE1>	.	PASS	SVTYPE=INS:ME:LINE1;END=169379458;SVLEN=759;AN=20618;AC=1;AF=4.850130953535746e-05;N_HET=1;N_HOMALT=0;N_HOMREF=10308;FREQ_HET=9.700261907071492e-05;FREQ_HOMALT=0.0;FREQ_HOMREF=0.9999029973809292;N_BI_GENOS=10309;South_Han_AN=1496;South_Han_AC=1;South_Han_
chr3	169386442	.	A	<INS:ME:LINE1>	.	PASS	SVTYPE=INS:ME:LINE1;END=169386456;SVLEN=1741;AN=20618;AC=1;AF=4.850130953535746e-05;N_HET=1;N_HOMALT=0;N_HOMREF=10308;FREQ_HET=9.700261907071492e-05;FREQ_HOMALT=0.0;FREQ_HOMREF=0.9999029973809292;N_BI_GENOS=10309;North_Han_AN=2272;North_Han_AC=1;North_Han
chr3	169408963	.	A	<INS:ME:LINE1>	.	PASS	SVTYPE=INS:ME:LINE1;END=169408982;SVLEN=3352;AN=20618;AC=2;AF=9.700261907071492e-05;N_HET=2;N_HOMALT=0;N_HOMREF=10307;FREQ_HET=0.00019400523814142983;FREQ_HOMALT=0.0;FREQ_HOMREF=0.9998059947618586;N_BI_GENOS=10309;Mongolian_AN=454;Mongolian_AC=2;Mongolian
chr3	169456985	.	A	<INS:ME:LINE1>	.	PASS	SVTYPE=INS:ME:LINE1;END=169457000;SVLEN=834;AN=20618;AC=1;AF=4.850130953535746e-05;N_HET=1;N_HOMALT=0;N_HOMREF=10308;FREQ_HET=9.700261907071492e-05;FREQ_HOMALT=0.0;FREQ_HOMREF=0.9999029973809292;N_BI_GENOS=10309;North_Han_AN=2272;North_Han_AC=1;North_Han_
chr3	169552167	.	A	<INS:ME:LINE1>	.	PASS	SVTYPE=INS:ME:LINE1;END=169552168;SVLEN=5997;AN=20618;AC=14880;AF=0.7216994858861189;N_HET=3902;N_HOMALT=5489;N_HOMREF=918;FREQ_HET=0.37850421961392955;FREQ_HOMALT=0.5324473760791542;FREQ_HOMREF=0.08904840430691628;N_BI_GENOS=10309;South_Han_AN=1496;South
chr3	170525609	.	T	<INS:ME:LINE1>	.	PASS	SVTYPE=INS:ME:LINE1;END=170525621;SVLEN=6055;AN=20618;AC=2;AF=9.700261907071492e-05;N_HET=2;N_HOMALT=0;N_HOMREF=10307;FREQ_HET=0.00019400523814142983;FREQ_HOMALT=0.0;FREQ_HOMREF=0.9998059947618586;N_BI_GENOS=10309;Lingnan_Han_AN=1888;Lingnan_Han_AC=1;Ling
chr3	170674970	.	G	<INS:ME:LINE1>	.	PASS	SVTYPE=INS:ME:LINE1;END=170674970;SVLEN=0;AN=20618;AC=2;AF=9.700261907071492e-05;N_HET=2;N_HOMALT=0;N_HOMREF=10307;FREQ_HET=0.00019400523814142983;FREQ_HOMALT=0.0;FREQ_HOMREF=0.9998059947618586;N_BI_GENOS=10309;East_Han_AN=6554;East_Han_AC=2;East_Han_AF=0
chr3	170825869	.	T	<INS:ME:LINE1>	.	PASS	SVTYPE=INS:ME:LINE1;END=170825885;SVLEN=6005;AN=20618;AC=2;AF=9.700261907071492e-05;N_HET=2;N_HOMALT=0;N_HOMREF=10307;FREQ_HET=0.00019400523814142983;FREQ_HOMALT=0.0;FREQ_HOMREF=0.9998059947618586;N_BI_GENOS=10309;South_Han_AN=1496;South_Han_AC=1;South_Ha
chr3	170993157	.	A	<INS:ME:LINE1>	.	PASS	SVTYPE=INS:ME:LINE1;END=170993157;SVLEN=928;AN=20618;AC=1;AF=4.850130953535746e-05;N_HET=1;N_HOMALT=0;N_HOMREF=10308;FREQ_HET=9.700261907071492e-05;FREQ_HOMALT=0.0;FREQ_HOMREF=0.9999029973809292;N_BI_GENOS=10309;East_Han_AN=6554;East_Han_AC=1;East_Han_AF=
chr3	171238673	.	T	<INS:ME:LINE1>	.	PASS	SVTYPE=INS:ME:LINE1;END=171238693;SVLEN=6019;AN=20618;AC=2;AF=9.700261907071492e-05;N_HET=2;N_HOMALT=0;N_HOMREF=10307;FREQ_HET=0.00019400523814142983;FREQ_HOMALT=0.0;FREQ_HOMREF=0.9998059947618586;N_BI_GENOS=10309;Northwest_Han_AN=1296;Northwest_Han_AC=1;
chr3	171319306	.	A	<INS:ME:LINE1>	.	PASS	SVTYPE=INS:ME:LINE1;END=171319316;SVLEN=826;AN=20618;AC=1;AF=4.850130953535746e-05;N_HET=1;N_HOMALT=0;N_HOMREF=10308;FREQ_HET=9.700261907071492e-05;FREQ_HOMALT=0.0;FREQ_HOMREF=0.9999029973809292;N_BI_GENOS=10309;Tibetan_AN=414;Tibetan_AC=1;Tibetan_AF=0.00
chr3	172291732	.	A	<INS:ME:LINE1>	.	PASS	SVTYPE=INS:ME:LINE1;END=172291747;SVLEN=1152;AN=20618;AC=1;AF=4.850130953535746e-05;N_HET=1;N_HOMALT=0;N_HOMREF=10308;FREQ_HET=9.700261907071492e-05;FREQ_HOMALT=0.0;FREQ_HOMREF=0.9999029973809292;N_BI_GENOS=10309;Central_Han_AN=2128;Central_Han_AC=1;Centr
chr3	172611719	.	A	<INS:ME:LINE1>	.	PASS	SVTYPE=INS:ME:LINE1;END=172611736;SVLEN=6025;AN=20618;AC=2;AF=9.700261907071492e-05;N_HET=2;N_HOMALT=0;N_HOMREF=10307;FREQ_HET=0.00019400523814142983;FREQ_HOMALT=0.0;FREQ_HOMREF=0.9998059947618586;N_BI_GENOS=10309;North_Han_AN=2272;North_Han_AC=1;North_Ha

chr3	173181357	.	T	<INS:ME:LINE1>	.	PASS	SVTYPE=INS:ME:LINE1;END=173181373;SVLEN=737;AN=20618;AC=1;AF=4.850130953535746e-05;N_HET=1;N_HOMALT=0;N_HOMREF=10308;FREQ_HET=9.700261907071492e-05;FREQ_HOMALT=0.0;FREQ_HOMREF=0.9999029973809292;N_BI_GENOS=10309;North_Han_AN=2272;North_Han_AC=1;North_Han_
chr3	173545069	.	T	<INS:ME:LINE1>	.	PASS	SVTYPE=INS:ME:LINE1;END=173545112;SVLEN=5754;AN=20618;AC=3;AF=0.00014550392860607236;N_HET=1;N_HOMALT=1;N_HOMREF=10307;FREQ_HET=9.700261907071492e-05;FREQ_HOMALT=9.700261907071492e-05;FREQ_HOMREF=0.9998059947618586;N_BI_GENOS=10309;East_Han_AN=6554;East_H
chr3	173872275	.	C	<INS:ME:LINE1>	.	PASS	SVTYPE=INS:ME:LINE1;END=173872291;SVLEN=1590;AN=20618;AC=3;AF=0.00014550392860607236;N_HET=3;N_HOMALT=0;N_HOMREF=10306;FREQ_HET=0.0002910078572121447;FREQ_HOMALT=0.0;FREQ_HOMREF=0.9997089921427879;N_BI_GENOS=10309;Northwest_Han_AN=1296;Northwest_Han_AC=1;
chr3	174037906	.	G	<INS:ME:LINE1>	.	PASS	SVTYPE=INS:ME:LINE1;END=174037918;SVLEN=643;AN=20618;AC=1;AF=4.850130953535746e-05;N_HET=1;N_HOMALT=0;N_HOMREF=10308;FREQ_HET=9.700261907071492e-05;FREQ_HOMALT=0.0;FREQ_HOMREF=0.9999029973809292;N_BI_GENOS=10309;Manchu_AN=416;Manchu_AC=1;Manchu_AF=0.00240
chr3	174199422	.	C	<INS:ME:LINE1>	.	PASS	SVTYPE=INS:ME:LINE1;END=174199438;SVLEN=123;AN=20618;AC=62;AF=0.0030070811911921624;N_HET=62;N_HOMALT=0;N_HOMREF=10247;FREQ_HET=0.006014162382384325;FREQ_HOMALT=0.0;FREQ_HOMREF=0.9939858376176157;N_BI_GENOS=10309;South_Han_AN=1496;South_Han_AC=4;South_Han
chr3	174730596	.	A	<INS:ME:LINE1>	.	PASS	SVTYPE=INS:ME:LINE1;END=174730612;SVLEN=3851;AN=20618;AC=1;AF=4.850130953535746e-05;N_HET=1;N_HOMALT=0;N_HOMREF=10308;FREQ_HET=9.700261907071492e-05;FREQ_HOMALT=0.0;FREQ_HOMREF=0.9999029973809292;N_BI_GENOS=10309;East_Han_AN=6554;East_Han_AC=1;East_Han_AF
chr3	175209039	.	A	<INS:ME:LINE1>	.	PASS	SVTYPE=INS:ME:LINE1;END=175209039;SVLEN=91;AN=20618;AC=2;AF=9.700261907071492e-05;N_HET=2;N_HOMALT=0;N_HOMREF=10307;FREQ_HET=0.00019400523814142983;FREQ_HOMALT=0.0;FREQ_HOMREF=0.9998059947618586;N_BI_GENOS=10309;Lingnan_Han_AN=1888;Lingnan_Han_AC=1;Lingna
chr3	175222858	.	C	<INS:ME:LINE1>	.	PASS	SVTYPE=INS:ME:LINE1;END=175222874;SVLEN=1845;AN=20618;AC=1;AF=4.850130953535746e-05;N_HET=1;N_HOMALT=0;N_HOMREF=10308;FREQ_HET=9.700261907071492e-05;FREQ_HOMALT=0.0;FREQ_HOMREF=0.9999029973809292;N_BI_GENOS=10309;Lingnan_Han_AN=1888;Lingnan_Han_AC=1;Lingn
chr3	175279947	.	A	<INS:ME:LINE1>	.	PASS	SVTYPE=INS:ME:LINE1;END=175279961;SVLEN=935;AN=20618;AC=3;AF=0.00014550392860607236;N_HET=1;N_HOMALT=1;N_HOMREF=10307;FREQ_HET=9.700261907071492e-05;FREQ_HOMALT=9.700261907071492e-05;FREQ_HOMREF=0.9998059947618586;N_BI_GENOS=10309;East_Han_AN=6554;East_Ha
chr3	175960181	.	C	<INS:ME:LINE1>	.	PASS	SVTYPE=INS:ME:LINE1;END=175960196;SVLEN=904;AN=20618;AC=6321;AF=0.30657677757299445;N_HET=4033;N_HOMALT=1144;N_HOMREF=5132;FREQ_HET=0.3912115627121932;FREQ_HOMALT=0.11097099621689786;FREQ_HOMREF=0.4978174410709089;N_BI_GENOS=10309;South_Han_AN=1496;South_
chr3	176036535	.	A	<INS:ME:LINE1>	.	PASS	SVTYPE=INS:ME:LINE1;END=176036539;SVLEN=2395;AN=20618;AC=1;AF=4.850130953535746e-05;N_HET=1;N_HOMALT=0;N_HOMREF=10308;FREQ_HET=9.700261907071492e-05;FREQ_HOMALT=0.0;FREQ_HOMREF=0.9999029973809292;N_BI_GENOS=10309;East_Han_AN=6554;East_Han_AC=1;East_Han_AF
chr3	176321844	.	T	<INS:ME:LINE1>	.	PASS	SVTYPE=INS:ME:LINE1;END=176321844;SVLEN=0;AN=20618;AC=6;AF=0.0002910078572121447;N_HET=4;N_HOMALT=1;N_HOMREF=10304;FREQ_HET=0.00038801047628285966;FREQ_HOMALT=9.700261907071492e-05;FREQ_HOMREF=0.9995149869046465;N_BI_GENOS=10309;South_Han_AN=1496;South_Ha
chr3	176373529	.	A	<INS:ME:LINE1>	.	PASS	SVTYPE=INS:ME:LINE1;END=176373545;SVLEN=223;AN=20618;AC=12;AF=0.0005820157144242894;N_HET=0;N_HOMALT=6;N_HOMREF=10303;FREQ_HET=0.0;FREQ_HOMALT=0.0005820157144242894;FREQ_HOMREF=0.9994179842855757;N_BI_GENOS=10309;East_Han_AN=6554;East_Han_AC=6;East_Han_AF
chr3	176467389	.	T	<INS:ME:LINE1>	.	PASS	SVTYPE=INS:ME:LINE1;END=176467402;SVLEN=1237;AN=20618;AC=1;AF=4.850130953535746e-05;N_HET=1;N_HOMALT=0;N_HOMREF=10308;FREQ_HET=9.700261907071492e-05;FREQ_HOMALT=0.0;FREQ_HOMREF=0.9999029973809292;N_BI_GENOS=10309;Northwest_Han_AN=1296;Northwest_Han_AC=1;N
chr3	176697211	.	G	<INS:ME:LINE1>	.	PASS	SVTYPE=INS:ME:LINE1;END=176697225;SVLEN=451;AN=20618;AC=1;AF=4.850130953535746e-05;N_HET=1;N_HOMALT=0;N_HOMREF=10308;FREQ_HET=9.700261907071492e-05;FREQ_HOMALT=0.0;FREQ_HOMREF=0.9999029973809292;N_BI_GENOS=10309;Central_Han_AN=2128;Central_Han_AC=1;Centra
chr3	177599700	.	A	<INS:ME:LINE1>	.	PASS	SVTYPE=INS:ME:LINE1;END=177599718;SVLEN=5998;AN=20618;AC=2;AF=9.700261907071492e-05;N_HET=2;N_HOMALT=0;N_HOMREF=10307;FREQ_HET=0.00019400523814142983;FREQ_HOMALT=0.0;FREQ_HOMREF=0.9998059947618586;N_BI_GENOS=10309;East_Han_AN=6554;East_Han_AC=1;East_Han_A
chr3	177669024	.	G	<INS:ME:LINE1>	.	PASS	SVTYPE=INS:ME:LINE1;END=177669024;SVLEN=0;AN=20618;AC=1;AF=4.850130953535746e-05;N_HET=1;N_HOMALT=0;N_HOMREF=10308;FREQ_HET=9.700261907071492e-05;FREQ_HOMALT=0.0;FREQ_HOMREF=0.9999029973809292;N_BI_GENOS=10309;Northwest_Han_AN=1296;Northwest_Han_AC=1;Nort
chr3	177694171	.	C	<INS:ME:LINE1>	.	PASS	SVTYPE=INS:ME:LINE1;END=177694189;SVLEN=2306;AN=20618;AC=1;AF=4.850130953535746e-05;N_HET=1;N_HOMALT=0;N_HOMREF=10308;FREQ_HET=9.700261907071492e-05;FREQ_HOMALT=0.0;FREQ_HOMREF=0.9999029973809292;N_BI_GENOS=10309;Southeast_Han_AN=1908;Southeast_Han_AC=1;S
chr3	177725501	.	G	<INS:ME:LINE1>	.	PASS	SVTYPE=INS:ME:LINE1;END=177725516;SVLEN=6000;AN=20618;AC=1;AF=4.850130953535746e-05;N_HET=1;N_HOMALT=0;N_HOMREF=10308;FREQ_HET=9.700261907071492e-05;FREQ_HOMALT=0.0;FREQ_HOMREF=0.9999029973809292;N_BI_GENOS=10309;North_Han_AN=2272;North_Han_AC=1;North_Han
chr3	177933539	.	C	<INS:ME:LINE1>	.	PASS	SVTYPE=INS:ME:LINE1;END=177933555;SVLEN=929;AN=20618;AC=1;AF=4.850130953535746e-05;N_HET=1;N_HOMALT=0;N_HOMREF=10308;FREQ_HET=9.700261907071492e-05;FREQ_HOMALT=0.0;FREQ_HOMREF=0.9999029973809292;N_BI_GENOS=10309;North_Han_AN=2272;North_Han_AC=1;North_Han_
chr3	178093926	.	T	<INS:ME:LINE1>	.	PASS	SVTYPE=INS:ME:LINE1;END=178093969;SVLEN=2684;AN=20618;AC=1;AF=4.850130953535746e-05;N_HET=1;N_HOMALT=0;N_HOMREF=10308;FREQ_HET=9.700261907071492e-05;FREQ_HOMALT=0.0;FREQ_HOMREF=0.9999029973809292;N_BI_GENOS=10309;East_Han_AN=6554;East_Han_AC=1;East_Han_AF
chr3	178093992	.	C	<INS:ME:LINE1>	.	PASS	SVTYPE=INS:ME:LINE1;END=178093992;SVLEN=876;AN=20618;AC=1;AF=4.850130953535746e-05;N_HET=1;N_HOMALT=0;N_HOMREF=10308;FREQ_HET=9.700261907071492e-05;FREQ_HOMALT=0.0;FREQ_HOMREF=0.9999029973809292;N_BI_GENOS=10309;East_Han_AN=6554;East_Han_AC=1;East_Han_AF=
chr3	178404341	.	T	<INS:ME:LINE1>	.	PASS	SVTYPE=INS:ME:LINE1;END=178404359;SVLEN=6005;AN=20618;AC=1;AF=4.850130953535746e-05;N_HET=1;N_HOMALT=0;N_HOMREF=10308;FREQ_HET=9.700261907071492e-05;FREQ_HOMALT=0.0;FREQ_HOMREF=0.9999029973809292;N_BI_GENOS=10309;North_Han_AN=2272;North_Han_AC=1;North_Han
chr3	179182105	.	C	<INS:ME:LINE1>	.	PASS	SVTYPE=INS:ME:LINE1;END=179182124;SVLEN=6000;AN=20618;AC=1;AF=4.850130953535746e-05;N_HET=1;N_HOMALT=0;N_HOMREF=10308;FREQ_HET=9.700261907071492e-05;FREQ_HOMALT=0.0;FREQ_HOMREF=0.9999029973809292;N_BI_GENOS=10309;Manchu_AN=416;Manchu_AC=1;Manchu_AF=0.0024
chr3	179288127	.	A	<INS:ME:LINE1>	.	PASS	SVTYPE=INS:ME:LINE1;END=179288140;SVLEN=848;AN=20618;AC=1;AF=4.850130953535746e-05;N_HET=1;N_HOMALT=0;N_HOMREF=10308;FREQ_HET=9.700261907071492e-05;FREQ_HOMALT=0.0;FREQ_HOMREF=0.9999029973809292;N_BI_GENOS=10309;East_Han_AN=6554;East_Han_AC=1;East_Han_AF=
chr3	180293166	.	A	<INS:ME:LINE1>	.	PASS	SVTYPE=INS:ME:LINE1;END=180293180;SVLEN=518;AN=20618;AC=1;AF=4.850130953535746e-05;N_HET=1;N_HOMALT=0;N_HOMREF=10308;FREQ_HET=9.700261907071492e-05;FREQ_HOMALT=0.0;FREQ_HOMREF=0.9999029973809292;N_BI_GENOS=10309;East_Han_AN=6554;East_Han_AC=1;East_Han_AF=
chr3	180336652	.	G	<INS:ME:LINE1>	.	PASS	SVTYPE=INS:ME:LINE1;END=180336667;SVLEN=5988;AN=20618;AC=3;AF=0.00014550392860607236;N_HET=3;N_HOMALT=0;N_HOMREF=10306;FREQ_HET=0.0002910078572121447;FREQ_HOMALT=0.0;FREQ_HOMREF=0.9997089921427879;N_BI_GENOS=10309;East_Han_AN=6554;East_Han_AC=1;East_Han_A
chr3	180412826	.	A	<INS:ME:LINE1>	.	PASS	SVTYPE=INS:ME:LINE1;END=180412840;SVLEN=1319;AN=20618;AC=3;AF=0.00014550392860607236;N_HET=3;N_HOMALT=0;N_HOMREF=10306;FREQ_HET=0.0002910078572121447;FREQ_HOMALT=0.0;FREQ_HOMREF=0.9997089921427879;N_BI_GENOS=10309;East_Han_AN=6554;East_Han_AC=1;East_Han_A
chr3	180441471	.	A	<INS:ME:LINE1>	.	PASS	SVTYPE=INS:ME:LINE1;END=180441478;SVLEN=840;AN=20618;AC=1;AF=4.850130953535746e-05;N_HET=1;N_HOMALT=0;N_HOMREF=10308;FREQ_HET=9.700261907071492e-05;FREQ_HOMALT=0.0;FREQ_HOMREF=0.9999029973809292;N_BI_GENOS=10309;Zhuang_AN=444;Zhuang_AC=1;Zhuang_AF=0.00225
chr3	180447867	.	A	<INS:ME:LINE1>	.	PASS	SVTYPE=INS:ME:LINE1;END=180447881;SVLEN=6004;AN=20618;AC=1;AF=4.850130953535746e-05;N_HET=1;N_HOMALT=0;N_HOMREF=10308;FREQ_HET=9.700261907071492e-05;FREQ_HOMALT=0.0;FREQ_HOMREF=0.9999029973809292;N_BI_GENOS=10309;East_Han_AN=6554;East_Han_AC=1;East_Han_AF
chr3	180514729	.	A	<INS:ME:LINE1>	.	PASS	SVTYPE=INS:ME:LINE1;END=180514740;SVLEN=4071;AN=20618;AC=2;AF=9.700261907071492e-05;N_HET=2;N_HOMALT=0;N_HOMREF=10307;FREQ_HET=0.00019400523814142983;FREQ_HOMALT=0.0;FREQ_HOMREF=0.9998059947618586;N_BI_GENOS=10309;South_Han_AN=1496;South_Han_AC=1;South_Ha
chr3	181019308	.	T	<INS:ME:LINE1>	.	PASS	SVTYPE=INS:ME:LINE1;END=181019323;SVLEN=983;AN=20618;AC=1;AF=4.850130953535746e-05;N_HET=1;N_HOMALT=0;N_HOMREF=10308;FREQ_HET=9.700261907071492e-05;FREQ_HOMALT=0.0;FREQ_HOMREF=0.9999029973809292;N_BI_GENOS=10309;North_Han_AN=2272;North_Han_AC=1;North_Han_
chr3	181122475	.	T	<INS:ME:LINE1>	.	PASS	SVTYPE=INS:ME:LINE1;END=181122475;SVLEN=1774;AN=20618;AC=1;AF=4.850130953535746e-05;N_HET=1;N_HOMALT=0;N_HOMREF=10308;FREQ_HET=9.700261907071492e-05;FREQ_HOMALT=0.0;FREQ_HOMREF=0.9999029973809292;N_BI_GENOS=10309;Zhuang_AN=444;Zhuang_AC=1;Zhuang_AF=0.0022
chr3	181299519	.	G	<INS:ME:LINE1>	.	PASS	SVTYPE=INS:ME:LINE1;END=181299527;SVLEN=6008;AN=20618;AC=1;AF=4.850130953535746e-05;N_HET=1;N_HOMALT=0;N_HOMREF=10308;FREQ_HET=9.700261907071492e-05;FREQ_HOMALT=0.0;FREQ_HOMREF=0.9999029973809292;N_BI_GENOS=10309;Southeast_Han_AN=1908;Southeast_Han_AC=1;S
chr3	181474229	.	T	<INS:ME:LINE1>	.	PASS	SVTYPE=INS:ME:LINE1;END=181474229;SVLEN=1120;AN=20618;AC=2;AF=9.700261907071492e-05;N_HET=2;N_HOMALT=0;N_HOMREF=10307;FREQ_HET=0.00019400523814142983;FREQ_HOMALT=0.0;FREQ_HOMREF=0.9998059947618586;N_BI_GENOS=10309;East_Han_AN=6554;East_Han_AC=2;East_Han_A
chr3	181475038	.	A	<INS:ME:LINE1>	.	PASS	SVTYPE=INS:ME:LINE1;END=181475057;SVLEN=3108;AN=20618;AC=5810;AF=0.2817926084004268;N_HET=3724;N_HOMALT=1043;N_HOMREF=5542;FREQ_HET=0.36123775341934233;FREQ_HOMALT=0.10117373169075565;FREQ_HOMREF=0.537588514889902;N_BI_GENOS=10309;South_Han_AN=1496;South_
chr3	181781832	.	T	<INS:ME:LINE1>	.	PASS	SVTYPE=INS:ME:LINE1;END=181781848;SVLEN=708;AN=20618;AC=1;AF=4.850130953535746e-05;N_HET=1;N_HOMALT=0;N_HOMREF=10308;FREQ_HET=9.700261907071492e-05;FREQ_HOMALT=0.0;FREQ_HOMREF=0.9999029973809292;N_BI_GENOS=10309;South_Han_AN=1496;South_Han_AC=1;South_Han_
chr3	182204645	.	C	<INS:ME:LINE1>	.	PASS	SVTYPE=INS:ME:LINE1;END=182204657;SVLEN=6000;AN=20618;AC=3;AF=0.00014550392860607236;N_HET=3;N_HOMALT=0;N_HOMREF=10306;FREQ_HET=0.0002910078572121447;FREQ_HOMALT=0.0;FREQ_HOMREF=0.9997089921427879;N_BI_GENOS=10309;East_Han_AN=6554;East_Han_AC=1;East_Han_A
chr3	182330271	.	A	<INS:ME:LINE1>	.	PASS	SVTYPE=INS:ME:LINE1;END=182330283;SVLEN=6001;AN=20618;AC=1;AF=4.850130953535746e-05;N_HET=1;N_HOMALT=0;N_HOMREF=10308;FREQ_HET=9.700261907071492e-05;FREQ_HOMALT=0.0;FREQ_HOMREF=0.9999029973809292;N_BI_GENOS=10309;North_Han_AN=2272;North_Han_AC=1;North_Han
chr3	182460644	.	A	<INS:ME:LINE1>	.	PASS	SVTYPE=INS:ME:LINE1;END=182460659;SVLEN=1649;AN=20618;AC=3;AF=0.00014550392860607236;N_HET=3;N_HOMALT=0;N_HOMREF=10306;FREQ_HET=0.0002910078572121447;FREQ_HOMALT=0.0;FREQ_HOMREF=0.9997089921427879;N_BI_GENOS=10309;Central_Han_AN=2128;Central_Han_AC=2;Cent
chr3	182571672	.	T	<INS:ME:LINE1>	.	PASS	SVTYPE=INS:ME:LINE1;END=182571687;SVLEN=6020;AN=20618;AC=1;AF=4.850130953535746e-05;N_HET=1;N_HOMALT=0;N_HOMREF=10308;FREQ_HET=9.700261907071492e-05;FREQ_HOMALT=0.0;FREQ_HOMREF=0.9999029973809292;N_BI_GENOS=10309;Lingnan_Han_AN=1888;Lingnan_Han_AC=1;Lingn
chr3	182669682	.	A	<INS:ME:LINE1>	.	PASS	SVTYPE=INS:ME:LINE1;END=182669699;SVLEN=314;AN=20618;AC=1;AF=4.850130953535746e-05;N_HET=1;N_HOMALT=0;N_HOMREF=10308;FREQ_HET=9.700261907071492e-05;FREQ_HOMALT=0.0;FREQ_HOMREF=0.9999029973809292;N_BI_GENOS=10309;Hui_AN=444;Hui_AC=1;Hui_AF=0.00225225225225
chr3	182735293	.	A	<INS:ME:LINE1>	.	PASS	SVTYPE=INS:ME:LINE1;END=182735296;SVLEN=503;AN=20618;AC=2;AF=9.700261907071492e-05;N_HET=2;N_HOMALT=0;N_HOMREF=10307;FREQ_HET=0.00019400523814142983;FREQ_HOMALT=0.0;FREQ_HOMREF=0.9998059947618586;N_BI_GENOS=10309;South_Han_AN=1496;South_Han_AC=1;South_Han
chr3	182835597	.	C	<INS:ME:LINE1>	.	PASS	SVTYPE=INS:ME:LINE1;END=182835612;SVLEN=35;AN=20618;AC=5;AF=0.00024250654767678728;N_HET=5;N_HOMALT=0;N_HOMREF=10304;FREQ_HET=0.00048501309535357455;FREQ_HOMALT=0.0;FREQ_HOMREF=0.9995149869046465;N_BI_GENOS=10309;South_Han_AN=1496;South_Han_AC=3;South_Han
chr3	182899816	.	T	<INS:ME:LINE1>	.	PASS	SVTYPE=INS:ME:LINE1;END=182899828;SVLEN=6009;AN=20618;AC=1;AF=4.850130953535746e-05;N_HET=1;N_HOMALT=0;N_HOMREF=10308;FREQ_HET=9.700261907071492e-05;FREQ_HOMALT=0.0;FREQ_HOMREF=0.9999029973809292;N_BI_GENOS=10309;East_Han_AN=6554;East_Han_AC=1;East_Han_AF
chr3	183198205	.	A	<INS:ME:LINE1>	.	PASS	SVTYPE=INS:ME:LINE1;END=183198213;SVLEN=357;AN=20618;AC=1;AF=4.850130953535746e-05;N_HET=1;N_HOMALT=0;N_HOMREF=10308;FREQ_HET=9.700261907071492e-05;FREQ_HOMALT=0.0;FREQ_HOMREF=0.9999029973809292;N_BI_GENOS=10309;Central_Han_AN=2128;Central_Han_AC=1;Centra
chr3	183982018	.	A	<INS:ME:LINE1>	.	PASS	SVTYPE=INS:ME:LINE1;END=183982020;SVLEN=305;AN=20618;AC=1;AF=4.850130953535746e-05;N_HET=1;N_HOMALT=0;N_HOMREF=10308;FREQ_HET=9.700261907071492e-05;FREQ_HOMALT=0.0;FREQ_HOMREF=0.9999029973809292;N_BI_GENOS=10309;North_Han_AN=2272;North_Han_AC=1;North_Han_
chr3	183985370	.	T	<INS:ME:LINE1>	.	PASS	SVTYPE=INS:ME:LINE1;END=183985380;SVLEN=766;AN=20618;AC=1;AF=4.850130953535746e-05;N_HET=1;N_HOMALT=0;N_HOMREF=10308;FREQ_HET=9.700261907071492e-05;FREQ_HOMALT=0.0;FREQ_HOMREF=0.9999029973809292;N_BI_GENOS=10309;East_Han_AN=6554;East_Han_AC=1;East_Han_AF=
chr3	184648454	.	T	<INS:ME:LINE1>	.	PASS	SVTYPE=INS:ME:LINE1;END=184648470;SVLEN=748;AN=20618;AC=1;AF=4.850130953535746e-05;N_HET=1;N_HOMALT=0;N_HOMREF=10308;FREQ_HET=9.700261907071492e-05;FREQ_HOMALT=0.0;FREQ_HOMREF=0.9999029973809292;N_BI_GENOS=10309;Northwest_Han_AN=1296;Northwest_Han_AC=1;No
chr3	184898343	.	G	<INS:ME:LINE1>	.	PASS	SVTYPE=INS:ME:LINE1;END=184898354;SVLEN=760;AN=20618;AC=1;AF=4.850130953535746e-05;N_HET=1;N_HOMALT=0;N_HOMREF=10308;FREQ_HET=9.700261907071492e-05;FREQ_HOMALT=0.0;FREQ_HOMREF=0.9999029973809292;N_BI_GENOS=10309;Lingnan_Han_AN=1888;Lingnan_Han_AC=1;Lingna
chr3	184985122	.	G	<INS:ME:LINE1>	.	PASS	SVTYPE=INS:ME:LINE1;END=184985138;SVLEN=269;AN=20618;AC=4;AF=0.00019400523814142983;N_HET=4;N_HOMALT=0;N_HOMREF=10305;FREQ_HET=0.00038801047628285966;FREQ_HOMALT=0.0;FREQ_HOMREF=0.9996119895237171;N_BI_GENOS=10309;North_Han_AN=2272;North_Han_AC=1;North_Ha
chr3	185381638	.	A	<INS:ME:LINE1>	.	PASS	SVTYPE=INS:ME:LINE1;END=185381651;SVLEN=2155;AN=20618;AC=2;AF=9.700261907071492e-05;N_HET=2;N_HOMALT=0;N_HOMREF=10307;FREQ_HET=0.00019400523814142983;FREQ_HOMALT=0.0;FREQ_HOMREF=0.9998059947618586;N_BI_GENOS=10309;East_Han_AN=6554;East_Han_AC=2;East_Han_A
chr3	186231611	.	T	<INS:ME:LINE1>	.	PASS	SVTYPE=INS:ME:LINE1;END=186231611;SVLEN=5952;AN=20618;AC=1;AF=4.850130953535746e-05;N_HET=1;N_HOMALT=0;N_HOMREF=10308;FREQ_HET=9.700261907071492e-05;FREQ_HOMALT=0.0;FREQ_HOMREF=0.9999029973809292;N_BI_GENOS=10309;North_Han_AN=2272;North_Han_AC=1;North_Han
chr3	186654334	.	A	<INS:ME:LINE1>	.	PASS	SVTYPE=INS:ME:LINE1;END=186654352;SVLEN=6000;AN=20618;AC=9232;AF=0.44776408963042;N_HET=4724;N_HOMALT=2254;N_HOMREF=3331;FREQ_HET=0.4582403724900572;FREQ_HOMALT=0.2186439033853914;FREQ_HOMREF=0.32311572412455136;N_BI_GENOS=10309;South_Han_AN=1496;South_Ha
chr3	186664935	.	C	<INS:ME:LINE1>	.	PASS	SVTYPE=INS:ME:LINE1;END=186664935;SVLEN=5926;AN=20618;AC=1;AF=4.850130953535746e-05;N_HET=1;N_HOMALT=0;N_HOMREF=10308;FREQ_HET=9.700261907071492e-05;FREQ_HOMALT=0.0;FREQ_HOMREF=0.9999029973809292;N_BI_GENOS=10309;Zhuang_AN=444;Zhuang_AC=1;Zhuang_AF=0.0022
chr3	187175728	.	G	<INS:ME:LINE1>	.	PASS	SVTYPE=INS:ME:LINE1;END=187175728;SVLEN=0;AN=20618;AC=1;AF=4.850130953535746e-05;N_HET=1;N_HOMALT=0;N_HOMREF=10308;FREQ_HET=9.700261907071492e-05;FREQ_HOMALT=0.0;FREQ_HOMREF=0.9999029973809292;N_BI_GENOS=10309;Southeast_Han_AN=1908;Southeast_Han_AC=1;Sout
chr3	187523549	.	C	<INS:ME:LINE1>	.	PASS	SVTYPE=INS:ME:LINE1;END=187523567;SVLEN=6002;AN=20618;AC=2;AF=9.700261907071492e-05;N_HET=2;N_HOMALT=0;N_HOMREF=10307;FREQ_HET=0.00019400523814142983;FREQ_HOMALT=0.0;FREQ_HOMREF=0.9998059947618586;N_BI_GENOS=10309;Lingnan_Han_AN=1888;Lingnan_Han_AC=1;Ling
chr3	187776835	.	A	<INS:ME:LINE1>	.	PASS	SVTYPE=INS:ME:LINE1;END=187776851;SVLEN=1980;AN=20618;AC=1;AF=4.850130953535746e-05;N_HET=1;N_HOMALT=0;N_HOMREF=10308;FREQ_HET=9.700261907071492e-05;FREQ_HOMALT=0.0;FREQ_HOMREF=0.9999029973809292;N_BI_GENOS=10309;East_Han_AN=6554;East_Han_AC=1;East_Han_AF
chr3	187786361	.	C	<INS:ME:LINE1>	.	PASS	SVTYPE=INS:ME:LINE1;END=187786361;SVLEN=6010;AN=20618;AC=4;AF=0.00019400523814142983;N_HET=4;N_HOMALT=0;N_HOMREF=10305;FREQ_HET=0.00038801047628285966;FREQ_HOMALT=0.0;FREQ_HOMREF=0.9996119895237171;N_BI_GENOS=10309;South_Han_AN=1496;South_Han_AC=1;South_H
chr3	188033939	.	A	<INS:ME:LINE1>	.	PASS	SVTYPE=INS:ME:LINE1;END=188033950;SVLEN=1917;AN=20618;AC=2;AF=9.700261907071492e-05;N_HET=2;N_HOMALT=0;N_HOMREF=10307;FREQ_HET=0.00019400523814142983;FREQ_HOMALT=0.0;FREQ_HOMREF=0.9998059947618586;N_BI_GENOS=10309;East_Han_AN=6554;East_Han_AC=1;East_Han_A
chr3	188117521	.	A	<INS:ME:LINE1>	.	PASS	SVTYPE=INS:ME:LINE1;END=188117521;SVLEN=5531;AN=20618;AC=63;AF=0.0030555825007275196;N_HET=63;N_HOMALT=0;N_HOMREF=10246;FREQ_HET=0.006111165001455039;FREQ_HOMALT=0.0;FREQ_HOMREF=0.993888834998545;N_BI_GENOS=10309;South_Han_AN=1496;South_Han_AC=5;South_Han
chr3	188516629	.	A	<INS:ME:LINE1>	.	PASS	SVTYPE=INS:ME:LINE1;END=188516644;SVLEN=526;AN=20618;AC=3467;AF=0.1681540401590843;N_HET=2477;N_HOMALT=495;N_HOMREF=7337;FREQ_HET=0.24027548743816082;FREQ_HOMALT=0.04801629644000388;FREQ_HOMREF=0.7117082161218353;N_BI_GENOS=10309;South_Han_AN=1496;South_H
chr3	188629964	.	T	<INS:ME:LINE1>	.	PASS	SVTYPE=INS:ME:LINE1;END=188629964;SVLEN=478;AN=20618;AC=1;AF=4.850130953535746e-05;N_HET=1;N_HOMALT=0;N_HOMREF=10308;FREQ_HET=9.700261907071492e-05;FREQ_HOMALT=0.0;FREQ_HOMREF=0.9999029973809292;N_BI_GENOS=10309;East_Han_AN=6554;East_Han_AC=1;East_Han_AF=
chr3	189001587	.	A	<INS:ME:LINE1>	.	PASS	SVTYPE=INS:ME:LINE1;END=189001600;SVLEN=2011;AN=20618;AC=1;AF=4.850130953535746e-05;N_HET=1;N_HOMALT=0;N_HOMREF=10308;FREQ_HET=9.700261907071492e-05;FREQ_HOMALT=0.0;FREQ_HOMREF=0.9999029973809292;N_BI_GENOS=10309;East_Han_AN=6554;East_Han_AC=1;East_Han_AF
chr3	189327649	.	A	<INS:ME:LINE1>	.	PASS	SVTYPE=INS:ME:LINE1;END=189327664;SVLEN=439;AN=20618;AC=1;AF=4.850130953535746e-05;N_HET=1;N_HOMALT=0;N_HOMREF=10308;FREQ_HET=9.700261907071492e-05;FREQ_HOMALT=0.0;FREQ_HOMREF=0.9999029973809292;N_BI_GENOS=10309;Southeast_Han_AN=1908;Southeast_Han_AC=1;So
chr3	189961609	.	C	<INS:ME:LINE1>	.	PASS	SVTYPE=INS:ME:LINE1;END=189961609;SVLEN=6;AN=20618;AC=1;AF=4.850130953535746e-05;N_HET=1;N_HOMALT=0;N_HOMREF=10308;FREQ_HET=9.700261907071492e-05;FREQ_HOMALT=0.0;FREQ_HOMREF=0.9999029973809292;N_BI_GENOS=10309;Central_Han_AN=2128;Central_Han_AC=1;Central_
chr3	190188475	.	T	<INS:ME:LINE1>	.	PASS	SVTYPE=INS:ME:LINE1;END=190188475;SVLEN=5977;AN=20618;AC=1;AF=4.850130953535746e-05;N_HET=1;N_HOMALT=0;N_HOMREF=10308;FREQ_HET=9.700261907071492e-05;FREQ_HOMALT=0.0;FREQ_HOMREF=0.9999029973809292;N_BI_GENOS=10309;North_Han_AN=2272;North_Han_AC=1;North_Han
chr3	190254651	.	T	<INS:ME:LINE1>	.	PASS	SVTYPE=INS:ME:LINE1;END=190254664;SVLEN=1495;AN=20618;AC=1;AF=4.850130953535746e-05;N_HET=1;N_HOMALT=0;N_HOMREF=10308;FREQ_HET=9.700261907071492e-05;FREQ_HOMALT=0.0;FREQ_HOMREF=0.9999029973809292;N_BI_GENOS=10309;Southeast_Han_AN=1908;Southeast_Han_AC=1;S
chr3	190418647	.	C	<INS:ME:LINE1>	.	PASS	SVTYPE=INS:ME:LINE1;END=190418647;SVLEN=67;AN=20618;AC=2;AF=9.700261907071492e-05;N_HET=2;N_HOMALT=0;N_HOMREF=10307;FREQ_HET=0.00019400523814142983;FREQ_HOMALT=0.0;FREQ_HOMREF=0.9998059947618586;N_BI_GENOS=10309;Tibetan_AN=414;Tibetan_AC=2;Tibetan_AF=0.00
chr3	190522956	.	G	<INS:ME:LINE1>	.	PASS	SVTYPE=INS:ME:LINE1;END=190522976;SVLEN=1145;AN=20618;AC=5;AF=0.00024250654767678728;N_HET=5;N_HOMALT=0;N_HOMREF=10304;FREQ_HET=0.00048501309535357455;FREQ_HOMALT=0.0;FREQ_HOMREF=0.9995149869046465;N_BI_GENOS=10309;Miao_AN=482;Miao_AC=5;Miao_AF=0.01037344
chr3	190689470	.	A	<INS:ME:LINE1>	.	PASS	SVTYPE=INS:ME:LINE1;END=190689488;SVLEN=2299;AN=20618;AC=62;AF=0.0030070811911921624;N_HET=62;N_HOMALT=0;N_HOMREF=10247;FREQ_HET=0.006014162382384325;FREQ_HOMALT=0.0;FREQ_HOMREF=0.9939858376176157;N_BI_GENOS=10309;South_Han_AN=1496;South_Han_AC=3;South_Ha
chr3	190706350	.	A	<INS:ME:LINE1>	.	PASS	SVTYPE=INS:ME:LINE1;END=190706363;SVLEN=1620;AN=20618;AC=3;AF=0.00014550392860607236;N_HET=3;N_HOMALT=0;N_HOMREF=10306;FREQ_HET=0.0002910078572121447;FREQ_HOMALT=0.0;FREQ_HOMREF=0.9997089921427879;N_BI_GENOS=10309;North_Han_AN=2272;North_Han_AC=1;North_Ha
chr3	191085104	.	A	<INS:ME:LINE1>	.	PASS	SVTYPE=INS:ME:LINE1;END=191085119;SVLEN=1520;AN=20618;AC=1;AF=4.850130953535746e-05;N_HET=1;N_HOMALT=0;N_HOMREF=10308;FREQ_HET=9.700261907071492e-05;FREQ_HOMALT=0.0;FREQ_HOMREF=0.9999029973809292;N_BI_GENOS=10309;East_Han_AN=6554;East_Han_AC=1;East_Han_AF
chr3	191645556	.	A	<INS:ME:LINE1>	.	PASS	SVTYPE=INS:ME:LINE1;END=191645556;SVLEN=581;AN=20618;AC=1;AF=4.850130953535746e-05;N_HET=1;N_HOMALT=0;N_HOMREF=10308;FREQ_HET=9.700261907071492e-05;FREQ_HOMALT=0.0;FREQ_HOMREF=0.9999029973809292;N_BI_GENOS=10309;East_Han_AN=6554;East_Han_AC=1;East_Han_AF=
chr3	191672130	.	C	<INS:ME:LINE1>	.	PASS	SVTYPE=INS:ME:LINE1;END=191672130;SVLEN=0;AN=20618;AC=2;AF=9.700261907071492e-05;N_HET=0;N_HOMALT=1;N_HOMREF=10308;FREQ_HET=0.0;FREQ_HOMALT=9.700261907071492e-05;FREQ_HOMREF=0.9999029973809292;N_BI_GENOS=10309;South_Han_AN=1496;South_Han_AC=2;South_Han_AF
chr3	192176369	.	G	<INS:ME:LINE1>	.	PASS	SVTYPE=INS:ME:LINE1;END=192176379;SVLEN=59;AN=20618;AC=1;AF=4.850130953535746e-05;N_HET=1;N_HOMALT=0;N_HOMREF=10308;FREQ_HET=9.700261907071492e-05;FREQ_HOMALT=0.0;FREQ_HOMREF=0.9999029973809292;N_BI_GENOS=10309;East_Han_AN=6554;East_Han_AC=1;East_Han_AF=0
chr3	192233311	.	A	<INS:ME:LINE1>	.	PASS	SVTYPE=INS:ME:LINE1;END=192233311;SVLEN=575;AN=20618;AC=1;AF=4.850130953535746e-05;N_HET=1;N_HOMALT=0;N_HOMREF=10308;FREQ_HET=9.700261907071492e-05;FREQ_HOMALT=0.0;FREQ_HOMREF=0.9999029973809292;N_BI_GENOS=10309;East_Han_AN=6554;East_Han_AC=1;East_Han_AF=
chr3	192428470	.	G	<INS:ME:LINE1>	.	PASS	SVTYPE=INS:ME:LINE1;END=192428475;SVLEN=725;AN=20618;AC=47;AF=0.0022795615481618005;N_HET=47;N_HOMALT=0;N_HOMREF=10262;FREQ_HET=0.004559123096323601;FREQ_HOMALT=0.0;FREQ_HOMREF=0.9954408769036764;N_BI_GENOS=10309;South_Han_AN=1496;South_Han_AC=6;South_Han
chr3	192493524	.	A	<INS:ME:LINE1>	.	PASS	SVTYPE=INS:ME:LINE1;END=192493537;SVLEN=670;AN=20618;AC=10;AF=0.00048501309535357455;N_HET=10;N_HOMALT=0;N_HOMREF=10299;FREQ_HET=0.0009700261907071491;FREQ_HOMALT=0.0;FREQ_HOMREF=0.9990299738092928;N_BI_GENOS=10309;South_Han_AN=1496;South_Han_AC=1;South_H
chr3	193209885	.	A	<INS:ME:LINE1>	.	PASS	SVTYPE=INS:ME:LINE1;END=193209899;SVLEN=100;AN=20618;AC=1;AF=4.850130953535746e-05;N_HET=1;N_HOMALT=0;N_HOMREF=10308;FREQ_HET=9.700261907071492e-05;FREQ_HOMALT=0.0;FREQ_HOMREF=0.9999029973809292;N_BI_GENOS=10309;Yi_AN=422;Yi_AC=1;Yi_AF=0.0023696682464549
chr3	193260685	.	A	<INS:ME:LINE1>	.	PASS	SVTYPE=INS:ME:LINE1;END=193260699;SVLEN=754;AN=20618;AC=1;AF=4.850130953535746e-05;N_HET=1;N_HOMALT=0;N_HOMREF=10308;FREQ_HET=9.700261907071492e-05;FREQ_HOMALT=0.0;FREQ_HOMREF=0.9999029973809292;N_BI_GENOS=10309;North_Han_AN=2272;North_Han_AC=1;North_Han_
chr3	193437736	.	C	<INS:ME:LINE1>	.	PASS	SVTYPE=INS:ME:LINE1;END=193437753;SVLEN=6070;AN=20618;AC=1;AF=4.850130953535746e-05;N_HET=1;N_HOMALT=0;N_HOMREF=10308;FREQ_HET=9.700261907071492e-05;FREQ_HOMALT=0.0;FREQ_HOMREF=0.9999029973809292;N_BI_GENOS=10309;North_Han_AN=2272;North_Han_AC=1;North_Han
chr3	193521092	.	T	<INS:ME:LINE1>	.	PASS	SVTYPE=INS:ME:LINE1;END=193521107;SVLEN=482;AN=20618;AC=1;AF=4.850130953535746e-05;N_HET=1;N_HOMALT=0;N_HOMREF=10308;FREQ_HET=9.700261907071492e-05;FREQ_HOMALT=0.0;FREQ_HOMREF=0.9999029973809292;N_BI_GENOS=10309;Northwest_Han_AN=1296;Northwest_Han_AC=1;No
chr3	193540965	.	A	<INS:ME:LINE1>	.	PASS	SVTYPE=INS:ME:LINE1;END=193540967;SVLEN=3494;AN=20618;AC=1;AF=4.850130953535746e-05;N_HET=1;N_HOMALT=0;N_HOMREF=10308;FREQ_HET=9.700261907071492e-05;FREQ_HOMALT=0.0;FREQ_HOMREF=0.9999029973809292;N_BI_GENOS=10309;East_Han_AN=6554;East_Han_AC=1;East_Han_AF
chr3	193541248	.	A	<INS:ME:LINE1>	.	PASS	SVTYPE=INS:ME:LINE1;END=193541259;SVLEN=3998;AN=20618;AC=50;AF=0.0024250654767678727;N_HET=30;N_HOMALT=10;N_HOMREF=10269;FREQ_HET=0.0029100785721214474;FREQ_HOMALT=0.0009700261907071491;FREQ_HOMREF=0.9961198952371714;N_BI_GENOS=10309;South_Han_AN=1496;Sou
chr3	193636369	.	G	<INS:ME:LINE1>	.	PASS	SVTYPE=INS:ME:LINE1;END=193636386;SVLEN=1007;AN=20618;AC=6364;AF=0.30866233388301484;N_HET=4342;N_HOMALT=1011;N_HOMREF=4956;FREQ_HET=0.42118537200504413;FREQ_HOMALT=0.09806964788049277;FREQ_HOMREF=0.4807449801144631;N_BI_GENOS=10309;South_Han_AN=1496;Sout
chr3	193712822	.	G	<INS:ME:LINE1>	.	PASS	SVTYPE=INS:ME:LINE1;END=193712822;SVLEN=0;AN=20618;AC=6;AF=0.0002910078572121447;N_HET=0;N_HOMALT=3;N_HOMREF=10306;FREQ_HET=0.0;FREQ_HOMALT=0.0002910078572121447;FREQ_HOMREF=0.9997089921427879;N_BI_GENOS=10309;South_Han_AN=1496;South_Han_AC=2;South_Han_AF
chr3	194430335	.	A	<INS:ME:LINE1>	.	PASS	SVTYPE=INS:ME:LINE1;END=194430344;SVLEN=2689;AN=20618;AC=1;AF=4.850130953535746e-05;N_HET=1;N_HOMALT=0;N_HOMREF=10308;FREQ_HET=9.700261907071492e-05;FREQ_HOMALT=0.0;FREQ_HOMREF=0.9999029973809292;N_BI_GENOS=10309;East_Han_AN=6554;East_Han_AC=1;East_Han_AF
chr3	194783874	.	G	<INS:ME:LINE1>	.	PASS	SVTYPE=INS:ME:LINE1;END=194783889;SVLEN=6014;AN=20618;AC=1;AF=4.850130953535746e-05;N_HET=1;N_HOMALT=0;N_HOMREF=10308;FREQ_HET=9.700261907071492e-05;FREQ_HOMALT=0.0;FREQ_HOMREF=0.9999029973809292;N_BI_GENOS=10309;East_Han_AN=6554;East_Han_AC=1;East_Han_AF
chr3	195018885	.	A	<INS:ME:LINE1>	.	PASS	SVTYPE=INS:ME:LINE1;END=195018902;SVLEN=5268;AN=20618;AC=1;AF=4.850130953535746e-05;N_HET=1;N_HOMALT=0;N_HOMREF=10308;FREQ_HET=9.700261907071492e-05;FREQ_HOMALT=0.0;FREQ_HOMREF=0.9999029973809292;N_BI_GENOS=10309;East_Han_AN=6554;East_Han_AC=1;East_Han_AF
chr3	195193457	.	T	<INS:ME:LINE1>	.	PASS	SVTYPE=INS:ME:LINE1;END=195193457;SVLEN=1323;AN=20618;AC=1;AF=4.850130953535746e-05;N_HET=1;N_HOMALT=0;N_HOMREF=10308;FREQ_HET=9.700261907071492e-05;FREQ_HOMALT=0.0;FREQ_HOMREF=0.9999029973809292;N_BI_GENOS=10309;Tibetan_AN=414;Tibetan_AC=1;Tibetan_AF=0.0
chr3	195194071	.	A	<INS:ME:LINE1>	.	PASS	SVTYPE=INS:ME:LINE1;END=195194084;SVLEN=951;AN=20618;AC=1;AF=4.850130953535746e-05;N_HET=1;N_HOMALT=0;N_HOMREF=10308;FREQ_HET=9.700261907071492e-05;FREQ_HOMALT=0.0;FREQ_HOMREF=0.9999029973809292;N_BI_GENOS=10309;East_Han_AN=6554;East_Han_AC=1;East_Han_AF=
chr3	195201184	.	C	<INS:ME:LINE1>	.	PASS	SVTYPE=INS:ME:LINE1;END=195201194;SVLEN=5960;AN=20618;AC=21;AF=0.0010185275002425065;N_HET=21;N_HOMALT=0;N_HOMREF=10288;FREQ_HET=0.002037055000485013;FREQ_HOMALT=0.0;FREQ_HOMREF=0.997962944999515;N_BI_GENOS=10309;South_Han_AN=1496;South_Han_AC=5;South_Han
chr3	195252692	.	C	<INS:ME:LINE1>	.	PASS	SVTYPE=INS:ME:LINE1;END=195252692;SVLEN=0;AN=20618;AC=1;AF=4.850130953535746e-05;N_HET=1;N_HOMALT=0;N_HOMREF=10308;FREQ_HET=9.700261907071492e-05;FREQ_HOMALT=0.0;FREQ_HOMREF=0.9999029973809292;N_BI_GENOS=10309;Zhuang_AN=444;Zhuang_AC=1;Zhuang_AF=0.0022522
chr3	195322517	.	A	<INS:ME:LINE1>	.	PASS	SVTYPE=INS:ME:LINE1;END=195322534;SVLEN=690;AN=20618;AC=2;AF=9.700261907071492e-05;N_HET=2;N_HOMALT=0;N_HOMREF=10307;FREQ_HET=0.00019400523814142983;FREQ_HOMALT=0.0;FREQ_HOMREF=0.9998059947618586;N_BI_GENOS=10309;East_Han_AN=6554;East_Han_AC=1;East_Han_AF
chr3	197109538	.	A	<INS:ME:LINE1>	.	PASS	SVTYPE=INS:ME:LINE1;END=197109549;SVLEN=86;AN=20618;AC=1;AF=4.850130953535746e-05;N_HET=1;N_HOMALT=0;N_HOMREF=10308;FREQ_HET=9.700261907071492e-05;FREQ_HOMALT=0.0;FREQ_HOMREF=0.9999029973809292;N_BI_GENOS=10309;Lingnan_Han_AN=1888;Lingnan_Han_AC=1;Lingnan

chr3	197118730	.	A	<INS:ME:LINE1>	.	PASS	SVTYPE=INS:ME:LINE1;END=197118746;SVLEN=6003;AN=20618;AC=1;AF=4.850130953535746e-05;N_HET=1;N_HOMALT=0;N_HOMREF=10308;FREQ_HET=9.700261907071492e-05;FREQ_HOMALT=0.0;FREQ_HOMREF=0.9999029973809292;N_BI_GENOS=10309;Northwest_Han_AN=1296;Northwest_Han_AC=1;N
chr3	197233118	.	A	<INS:ME:LINE1>	.	PASS	SVTYPE=INS:ME:LINE1;END=197233130;SVLEN=5998;AN=20618;AC=1;AF=4.850130953535746e-05;N_HET=1;N_HOMALT=0;N_HOMREF=10308;FREQ_HET=9.700261907071492e-05;FREQ_HOMALT=0.0;FREQ_HOMREF=0.9999029973809292;N_BI_GENOS=10309;East_Han_AN=6554;East_Han_AC=1;East_Han_AF
chr3	197568273	.	T	<INS:ME:LINE1>	.	PASS	SVTYPE=INS:ME:LINE1;END=197568273;SVLEN=5998;AN=20618;AC=5;AF=0.00024250654767678728;N_HET=5;N_HOMALT=0;N_HOMREF=10304;FREQ_HET=0.00048501309535357455;FREQ_HOMALT=0.0;FREQ_HOMREF=0.9995149869046465;N_BI_GENOS=10309;East_Han_AN=6554;East_Han_AC=3;East_Han_
chr4	466407	.	T	<INS:ME:LINE1>	.	PASS	SVTYPE=INS:ME:LINE1;END=466420;SVLEN=220;AN=20618;AC=2;AF=9.700261907071492e-05;N_HET=2;N_HOMALT=0;N_HOMREF=10307;FREQ_HET=0.00019400523814142983;FREQ_HOMALT=0.0;FREQ_HOMREF=0.9998059947618586;N_BI_GENOS=10309;Southeast_Han_AN=1908;Southeast_Han_AC=2;Sout
chr4	2333822	.	A	<INS:ME:LINE1>	.	PASS	SVTYPE=INS:ME:LINE1;END=2333834;SVLEN=3227;AN=20618;AC=1;AF=4.850130953535746e-05;N_HET=1;N_HOMALT=0;N_HOMREF=10308;FREQ_HET=9.700261907071492e-05;FREQ_HOMALT=0.0;FREQ_HOMREF=0.9999029973809292;N_BI_GENOS=10309;East_Han_AN=6554;East_Han_AC=1;East_Han_AF=0
chr4	3114704	.	T	<INS:ME:LINE1>	.	PASS	SVTYPE=INS:ME:LINE1;END=3114716;SVLEN=5979;AN=20618;AC=1;AF=4.850130953535746e-05;N_HET=1;N_HOMALT=0;N_HOMREF=10308;FREQ_HET=9.700261907071492e-05;FREQ_HOMALT=0.0;FREQ_HOMREF=0.9999029973809292;N_BI_GENOS=10309;Yi_AN=422;Yi_AC=1;Yi_AF=0.002369668246445498
chr4	4074596	.	A	<INS:ME:LINE1>	.	PASS	SVTYPE=INS:ME:LINE1;END=4074596;SVLEN=0;AN=20618;AC=2;AF=9.700261907071492e-05;N_HET=2;N_HOMALT=0;N_HOMREF=10307;FREQ_HET=0.00019400523814142983;FREQ_HOMALT=0.0;FREQ_HOMREF=0.9998059947618586;N_BI_GENOS=10309;East_Han_AN=6554;East_Han_AC=2;East_Han_AF=0.0
chr4	4074779	.	C	<INS:ME:LINE1>	.	PASS	SVTYPE=INS:ME:LINE1;END=4074779;SVLEN=0;AN=20618;AC=5;AF=0.00024250654767678728;N_HET=5;N_HOMALT=0;N_HOMREF=10304;FREQ_HET=0.00048501309535357455;FREQ_HOMALT=0.0;FREQ_HOMREF=0.9995149869046465;N_BI_GENOS=10309;South_Han_AN=1496;South_Han_AC=1;South_Han_AF
chr4	4120520	.	G	<INS:ME:LINE1>	.	PASS	SVTYPE=INS:ME:LINE1;END=4120520;SVLEN=0;AN=20618;AC=1;AF=4.850130953535746e-05;N_HET=1;N_HOMALT=0;N_HOMREF=10308;FREQ_HET=9.700261907071492e-05;FREQ_HOMALT=0.0;FREQ_HOMREF=0.9999029973809292;N_BI_GENOS=10309;Lingnan_Han_AN=1888;Lingnan_Han_AC=1;Lingnan_Ha
chr4	4493700	.	A	<INS:ME:LINE1>	.	PASS	SVTYPE=INS:ME:LINE1;END=4493711;SVLEN=5688;AN=20618;AC=1;AF=4.850130953535746e-05;N_HET=1;N_HOMALT=0;N_HOMREF=10308;FREQ_HET=9.700261907071492e-05;FREQ_HOMALT=0.0;FREQ_HOMREF=0.9999029973809292;N_BI_GENOS=10309;Manchu_AN=416;Manchu_AC=1;Manchu_AF=0.002403
chr4	4880613	.	A	<INS:ME:LINE1>	.	PASS	SVTYPE=INS:ME:LINE1;END=4880628;SVLEN=5999;AN=20618;AC=1;AF=4.850130953535746e-05;N_HET=1;N_HOMALT=0;N_HOMREF=10308;FREQ_HET=9.700261907071492e-05;FREQ_HOMALT=0.0;FREQ_HOMREF=0.9999029973809292;N_BI_GENOS=10309;Lingnan_Han_AN=1888;Lingnan_Han_AC=1;Lingnan
chr4	5666267	.	G	<INS:ME:LINE1>	.	PASS	SVTYPE=INS:ME:LINE1;END=5666267;SVLEN=766;AN=20618;AC=1551;AF=0.07522553108933941;N_HET=1401;N_HOMALT=75;N_HOMREF=8833;FREQ_HET=0.1359006693180716;FREQ_HOMALT=0.007275196430303618 5;FREQ_HOMREF=0.8568241342516248;N_BI_GENOS=10309;South_Han_AN=1496;South_Ha
chr4	5720569	.	C	<INS:ME:LINE1>	.	PASS	SVTYPE=INS:ME:LINE1;END=5720569;SVLEN=6000;AN=20618;AC=1;AF=4.850130953535746e-05;N_HET=1;N_HOMALT=0;N_HOMREF=10308;FREQ_HET=9.700261907071492e-05;FREQ_HOMALT=0.0;FREQ_HOMREF=0.9999029973809292;N_BI_GENOS=10309;Lingnan_Han_AN=1888;Lingnan_Han_AC=1;Lingnan
chr4	5776156	.	C	<INS:ME:LINE1>	.	PASS	SVTYPE=INS:ME:LINE1;END=5776166;SVLEN=2073;AN=20618;AC=3;AF=0.00014550392860607236;N_HET=3;N_HOMALT=0;N_HOMREF=10306;FREQ_HET=0.0002910078572121447;FREQ_HOMALT=0.0;FREQ_HOMREF=0.9997089921427879;N_BI_GENOS=10309;South_Han_AN=1496;South_Han_AC=1;South_Han_
chr4	5824893	.	A	<INS:ME:LINE1>	.	PASS	SVTYPE=INS:ME:LINE1;END=5824908;SVLEN=1362;AN=20618;AC=1;AF=4.850130953535746e-05;N_HET=1;N_HOMALT=0;N_HOMREF=10308;FREQ_HET=9.700261907071492e-05;FREQ_HOMALT=0.0;FREQ_HOMREF=0.9999029973809292;N_BI_GENOS=10309;Northwest_Han_AN=1296;Northwest_Han_AC=1;Nor
chr4	6173536	.	A	<INS:ME:LINE1>	.	PASS	SVTYPE=INS:ME:LINE1;END=6173552;SVLEN=6071;AN=20618;AC=2;AF=9.700261907071492e-05;N_HET=2;N_HOMALT=0;N_HOMREF=10307;FREQ_HET=0.00019400523814142983;FREQ_HOMALT=0.0;FREQ_HOMREF=0.9998059947618586;N_BI_GENOS=10309;Northwest_Han_AN=1296;Northwest_Han_AC=1;No
chr4	6422171	.	G	<INS:ME:LINE1>	.	PASS	SVTYPE=INS:ME:LINE1;END=6422171;SVLEN=433;AN=20618;AC=2;AF=9.700261907071492e-05;N_HET=2;N_HOMALT=0;N_HOMREF=10307;FREQ_HET=0.00019400523814142983;FREQ_HOMALT=0.0;FREQ_HOMREF=0.9998059947618586;N_BI_GENOS=10309;East_Han_AN=6554;East_Han_AC=2;East_Han_AF=0
chr4	6449079	.	A	<INS:ME:LINE1>	.	PASS	SVTYPE=INS:ME:LINE1;END=6449081;SVLEN=894;AN=20618;AC=2;AF=9.700261907071492e-05;N_HET=2;N_HOMALT=0;N_HOMREF=10307;FREQ_HET=0.00019400523814142983;FREQ_HOMALT=0.0;FREQ_HOMREF=0.9998059947618586;N_BI_GENOS=10309;Lingnan_Han_AN=1888;Lingnan_Han_AC=1;Lingnan
chr4	7093093	.	T	<INS:ME:LINE1>	.	PASS	SVTYPE=INS:ME:LINE1;END=7093103;SVLEN=131;AN=20618;AC=7;AF=0.0003395091667475022;N_HET=7;N_HOMALT=0;N_HOMREF=10302;FREQ_HET=0.0006790183334950044;FREQ_HOMALT=0.0;FREQ_HOMREF=0.999320981666505;N_BI_GENOS=10309;South_Han_AN=1496;South_Han_AC=1;South_Han_AF=
chr4	9177030	.	T	<INS:ME:LINE1>	.	PASS	SVTYPE=INS:ME:LINE1;END=9177030;SVLEN=0;AN=20618;AC=2;AF=9.700261907071492e-05;N_HET=0;N_HOMALT=1;N_HOMREF=10308;FREQ_HET=0.0;FREQ_HOMALT=9.700261907071492e-05;FREQ_HOMREF=0.9999029973809292;N_BI_GENOS=10309;East_Han_AN=6554;East_Han_AC=2;East_Han_AF=0.00
chr4	9421412	.	C	<INS:ME:LINE1>	.	PASS	SVTYPE=INS:ME:LINE1;END=9421430;SVLEN=6055;AN=20618;AC=1;AF=4.850130953535746e-05;N_HET=1;N_HOMALT=0;N_HOMREF=10308;FREQ_HET=9.700261907071492e-05;FREQ_HOMALT=0.0;FREQ_HOMREF=0.9999029973809292;N_BI_GENOS=10309;East_Han_AN=6554;East_Han_AC=1;East_Han_AF=0
chr4	9455323	.	G	<INS:ME:LINE1>	.	PASS	SVTYPE=INS:ME:LINE1;END=9455338;SVLEN=1095;AN=20618;AC=4;AF=0.00019400523814142983;N_HET=4;N_HOMALT=0;N_HOMREF=10305;FREQ_HET=0.00038801047628285966;FREQ_HOMALT=0.0;FREQ_HOMREF=0.9996119895237171;N_BI_GENOS=10309;South_Han_AN=1496;South_Han_AC=1;South_Han
chr4	9519681	.	C	<INS:ME:LINE1>	.	PASS	SVTYPE=INS:ME:LINE1;END=9519681;SVLEN=0;AN=20618;AC=16;AF=0.0007760209525657193;N_HET=16;N_HOMALT=0;N_HOMREF=10293;FREQ_HET=0.001552041905131438 6;FREQ_HOMALT=0.0;FREQ_HOMREF=0.9984479580948685;N_BI_GENOS=10309;South_Han_AN=1496;South_Han_AC=2;South_Han_AF
chr4	9810615	.	A	<INS:ME:LINE1>	.	PASS	SVTYPE=INS:ME:LINE1;END=9810618;SVLEN=1598;AN=20618;AC=11;AF=0.000533514404888932;N_HET=11;N_HOMALT=0;N_HOMREF=10298;FREQ_HET=0.001067028809777864;FREQ_HOMALT=0.0;FREQ_HOMREF=0.9989329711902222;N_BI_GENOS=10309;Northwest_Han_AN=1296;Northwest_Han_AC=2;Nor
chr4	9871014	.	C	<INS:ME:LINE1>	.	PASS	SVTYPE=INS:ME:LINE1;END=9871032;SVLEN=1997;AN=20618;AC=2;AF=9.700261907071492e-05;N_HET=2;N_HOMALT=0;N_HOMREF=10307;FREQ_HET=0.00019400523814142983;FREQ_HOMALT=0.0;FREQ_HOMREF=0.9998059947618586;N_BI_GENOS=10309;Northwest_Han_AN=1296;Northwest_Han_AC=1;No
chr4	10263815	.	T	<INS:ME:LINE1>	.	PASS	SVTYPE=INS:ME:LINE1;END=10263830;SVLEN=5993;AN=20618;AC=1;AF=4.850130953535746e-05;N_HET=1;N_HOMALT=0;N_HOMREF=10308;FREQ_HET=9.700261907071492e-05;FREQ_HOMALT=0.0;FREQ_HOMREF=0.9999029973809292;N_BI_GENOS=10309;Central_Han_AN=2128;Central_Han_AC=1;Centra
chr4	10631054	.	C	<INS:ME:LINE1>	.	PASS	SVTYPE=INS:ME:LINE1;END=10631070;SVLEN=5996;AN=20618;AC=6;AF=0.0002910078572121447;N_HET=6;N_HOMALT=0;N_HOMREF=10303;FREQ_HET=0.0005820157144242894;FREQ_HOMALT=0.0;FREQ_HOMREF=0.9994179842855757;N_BI_GENOS=10309;Northwest_Han_AN=1296;Northwest_Han_AC=1;No
chr4	10647340	.	T	<INS:ME:LINE1>	.	PASS	SVTYPE=INS:ME:LINE1;END=10647356;SVLEN=479;AN=20618;AC=1;AF=4.850130953535746e-05;N_HET=1;N_HOMALT=0;N_HOMREF=10308;FREQ_HET=9.700261907071492e-05;FREQ_HOMALT=0.0;FREQ_HOMREF=0.9999029973809292;N_BI_GENOS=10309;Central_Han_AN=2128;Central_Han_AC=1;Central
chr4	10714335	.	G	<INS:ME:LINE1>	.	PASS	SVTYPE=INS:ME:LINE1;END=10714355;SVLEN=2098;AN=20618;AC=1;AF=4.850130953535746e-05;N_HET=1;N_HOMALT=0;N_HOMREF=10308;FREQ_HET=9.700261907071492e-05;FREQ_HOMALT=0.0;FREQ_HOMREF=0.9999029973809292;N_BI_GENOS=10309;South_Han_AN=1496;South_Han_AC=1;South_Han_
chr4	11070485	.	A	<INS:ME:LINE1>	.	PASS	SVTYPE=INS:ME:LINE1;END=11070501;SVLEN=6003;AN=20618;AC=1;AF=4.850130953535746e-05;N_HET=1;N_HOMALT=0;N_HOMREF=10308;FREQ_HET=9.700261907071492e-05;FREQ_HOMALT=0.0;FREQ_HOMREF=0.9999029973809292;N_BI_GENOS=10309;Tibetan_AN=414;Tibetan_AC=1;Tibetan_AF=0.00
chr4	11687621	.	A	<INS:ME:LINE1>	.	PASS	SVTYPE=INS:ME:LINE1;END=11687636;SVLEN=878;AN=20618;AC=1;AF=4.850130953535746e-05;N_HET=1;N_HOMALT=0;N_HOMREF=10308;FREQ_HET=9.700261907071492e-05;FREQ_HOMALT=0.0;FREQ_HOMREF=0.9999029973809292;N_BI_GENOS=10309;East_Han_AN=6554;East_Han_AC=1;East_Han_AF=0
chr4	12528472	.	A	<INS:ME:LINE1>	.	PASS	SVTYPE=INS:ME:LINE1;END=12528472;SVLEN=923;AN=20618;AC=1;AF=4.850130953535746e-05;N_HET=1;N_HOMALT=0;N_HOMREF=10308;FREQ_HET=9.700261907071492e-05;FREQ_HOMALT=0.0;FREQ_HOMREF=0.9999029973809292;N_BI_GENOS=10309;East_Han_AN=6554;East_Han_AC=1;East_Han_AF=0
chr4	12587851	.	A	<INS:ME:LINE1>	.	PASS	SVTYPE=INS:ME:LINE1;END=12587856;SVLEN=559;AN=20618;AC=1;AF=4.850130953535746e-05;N_HET=1;N_HOMALT=0;N_HOMREF=10308;FREQ_HET=9.700261907071492e-05;FREQ_HOMALT=0.0;FREQ_HOMREF=0.9999029973809292;N_BI_GENOS=10309;East_Han_AN=6554;East_Han_AC=1;East_Han_AF=0
chr4	12645756	.	A	<INS:ME:LINE1>	.	PASS	SVTYPE=INS:ME:LINE1;END=12645756;SVLEN=6000;AN=20618;AC=2;AF=9.700261907071492e-05;N_HET=2;N_HOMALT=0;N_HOMREF=10307;FREQ_HET=0.00019400523814142983;FREQ_HOMALT=0.0;FREQ_HOMREF=0.9998059947618586;N_BI_GENOS=10309;South_Han_AN=1496;South_Han_AC=1;South_Han
chr4	12674257	.	A	<INS:ME:LINE1>	.	PASS	SVTYPE=INS:ME:LINE1;END=12674268;SVLEN=468;AN=20618;AC=5;AF=0.00024250654767678728;N_HET=5;N_HOMALT=0;N_HOMREF=10304;FREQ_HET=0.00048501309535357455;FREQ_HOMALT=0.0;FREQ_HOMREF=0.9995149869046465;N_BI_GENOS=10309;North_Han_AN=2272;North_Han_AC=3;North_Han
chr4	12729984	.	A	<INS:ME:LINE1>	.	PASS	SVTYPE=INS:ME:LINE1;END=12729999;SVLEN=697;AN=20618;AC=2;AF=9.700261907071492e-05;N_HET=2;N_HOMALT=0;N_HOMREF=10307;FREQ_HET=0.00019400523814142983;FREQ_HOMALT=0.0;FREQ_HOMREF=0.9998059947618586;N_BI_GENOS=10309;Lingnan_Han_AN=1888;Lingnan_Han_AC=1;Lingna
chr4	12926792	.	C	<INS:ME:LINE1>	.	PASS	SVTYPE=INS:ME:LINE1;END=12926806;SVLEN=368;AN=20618;AC=1;AF=4.850130953535746e-05;N_HET=1;N_HOMALT=0;N_HOMREF=10308;FREQ_HET=9.700261907071492e-05;FREQ_HOMALT=0.0;FREQ_HOMREF=0.9999029973809292;N_BI_GENOS=10309;Northwest_Han_AN=1296;Northwest_Han_AC=1;Nor
chr4	13325799	.	A	<INS:ME:LINE1>	.	PASS	SVTYPE=INS:ME:LINE1;END=13325807;SVLEN=2175;AN=20618;AC=2;AF=9.700261907071492e-05;N_HET=2;N_HOMALT=0;N_HOMREF=10307;FREQ_HET=0.00019400523814142983;FREQ_HOMALT=0.0;FREQ_HOMREF=0.9998059947618586;N_BI_GENOS=10309;Northwest_Han_AN=1296;Northwest_Han_AC=1;N
chr4	13403902	.	A	<INS:ME:LINE1>	.	PASS	SVTYPE=INS:ME:LINE1;END=13403920;SVLEN=848;AN=20618;AC=1;AF=4.850130953535746e-05;N_HET=1;N_HOMALT=0;N_HOMREF=10308;FREQ_HET=9.700261907071492e-05;FREQ_HOMALT=0.0;FREQ_HOMREF=0.9999029973809292;N_BI_GENOS=10309;Southeast_Han_AN=1908;Southeast_Han_AC=1;Sou
chr4	13439923	.	A	<INS:ME:LINE1>	.	PASS	SVTYPE=INS:ME:LINE1;END=13439939;SVLEN=5997;AN=20618;AC=6;AF=0.0002910078572121447;N_HET=6;N_HOMALT=0;N_HOMREF=10303;FREQ_HET=0.0005820157144242894;FREQ_HOMALT=0.0;FREQ_HOMREF=0.9994179842855757;N_BI_GENOS=10309;Lingnan_Han_AN=1888;Lingnan_Han_AC=1;Lingna
chr4	13504481	.	A	<INS:ME:LINE1>	.	PASS	SVTYPE=INS:ME:LINE1;END=13504490;SVLEN=3031;AN=20618;AC=7;AF=0.0003395091667475022;N_HET=7;N_HOMALT=0;N_HOMREF=10302;FREQ_HET=0.0006790183334950044;FREQ_HOMALT=0.0;FREQ_HOMREF=0.999320981666505;N_BI_GENOS=10309;South_Han_AN=1496;South_Han_AC=1;South_Han_A
chr4	13609170	.	A	<INS:ME:LINE1>	.	PASS	SVTYPE=INS:ME:LINE1;END=13609184;SVLEN=6005;AN=20618;AC=1;AF=4.850130953535746e-05;N_HET=1;N_HOMALT=0;N_HOMREF=10308;FREQ_HET=9.700261907071492e-05;FREQ_HOMALT=0.0;FREQ_HOMREF=0.9999029973809292;N_BI_GENOS=10309;Zhuang_AN=444;Zhuang_AC=1;Zhuang_AF=0.00225
chr4	13754047	.	A	<INS:ME:LINE1>	.	PASS	SVTYPE=INS:ME:LINE1;END=13754059;SVLEN=376;AN=20618;AC=1;AF=4.850130953535746e-05;N_HET=1;N_HOMALT=0;N_HOMREF=10308;FREQ_HET=9.700261907071492e-05;FREQ_HOMALT=0.0;FREQ_HOMREF=0.9999029973809292;N_BI_GENOS=10309;North_Han_AN=2272;North_Han_AC=1;North_Han_A
chr4	13841854	.	T	<INS:ME:LINE1>	.	PASS	SVTYPE=INS:ME:LINE1;END=13841862;SVLEN=3740;AN=20618;AC=1;AF=4.850130953535746e-05;N_HET=1;N_HOMALT=0;N_HOMREF=10308;FREQ_HET=9.700261907071492e-05;FREQ_HOMALT=0.0;FREQ_HOMREF=0.9999029973809292;N_BI_GENOS=10309;East_Han_AN=6554;East_Han_AC=1;East_Han_AF=
chr4	13889892	.	C	<INS:ME:LINE1>	.	PASS	SVTYPE=INS:ME:LINE1;END=13889906;SVLEN=6012;AN=20618;AC=1;AF=4.850130953535746e-05;N_HET=1;N_HOMALT=0;N_HOMREF=10308;FREQ_HET=9.700261907071492e-05;FREQ_HOMALT=0.0;FREQ_HOMREF=0.9999029973809292;N_BI_GENOS=10309;Tibetan_AN=414;Tibetan_AC=1;Tibetan_AF=0.00
chr4	13905621	.	A	<INS:ME:LINE1>	.	PASS	SVTYPE=INS:ME:LINE1;END=13905635;SVLEN=734;AN=20618;AC=1;AF=4.850130953535746e-05;N_HET=1;N_HOMALT=0;N_HOMREF=10308;FREQ_HET=9.700261907071492e-05;FREQ_HOMALT=0.0;FREQ_HOMREF=0.9999029973809292;N_BI_GENOS=10309;North_Han_AN=2272;North_Han_AC=1;North_Han_A
chr4	13965303	.	A	<INS:ME:LINE1>	.	PASS	SVTYPE=INS:ME:LINE1;END=13965320;SVLEN=1140;AN=20618;AC=1;AF=4.850130953535746e-05;N_HET=1;N_HOMALT=0;N_HOMREF=10308;FREQ_HET=9.700261907071492e-05;FREQ_HOMALT=0.0;FREQ_HOMREF=0.9999029973809292;N_BI_GENOS=10309;South_Han_AN=1496;South_Han_AC=1;South_Han_
chr4	14257413	.	G	<INS:ME:LINE1>	.	PASS	SVTYPE=INS:ME:LINE1;END=14257428;SVLEN=928;AN=20618;AC=1;AF=4.850130953535746e-05;N_HET=1;N_HOMALT=0;N_HOMREF=10308;FREQ_HET=9.700261907071492e-05;FREQ_HOMALT=0.0;FREQ_HOMREF=0.9999029973809292;N_BI_GENOS=10309;Lingnan_Han_AN=1888;Lingnan_Han_AC=1;Lingnan
chr4	14262865	.	G	<INS:ME:LINE1>	.	PASS	SVTYPE=INS:ME:LINE1;END=14262883;SVLEN=2055;AN=20618;AC=1;AF=4.850130953535746e-05;N_HET=1;N_HOMALT=0;N_HOMREF=10308;FREQ_HET=9.700261907071492e-05;FREQ_HOMALT=0.0;FREQ_HOMREF=0.9999029973809292;N_BI_GENOS=10309;North_Han_AN=2272;North_Han_AC=1;North_Han_
chr4	14502798	.	A	<INS:ME:LINE1>	.	PASS	SVTYPE=INS:ME:LINE1;END=14502798;SVLEN=1070;AN=20618;AC=1;AF=4.850130953535746e-05;N_HET=1;N_HOMALT=0;N_HOMREF=10308;FREQ_HET=9.700261907071492e-05;FREQ_HOMALT=0.0;FREQ_HOMREF=0.9999029973809292;N_BI_GENOS=10309;East_Han_AN=6554;East_Han_AC=1;East_Han_AF=
chr4	14528613	.	G	<INS:ME:LINE1>	.	PASS	SVTYPE=INS:ME:LINE1;END=14528613;SVLEN=741;AN=20618;AC=1;AF=4.850130953535746e-05;N_HET=1;N_HOMALT=0;N_HOMREF=10308;FREQ_HET=9.700261907071492e-05;FREQ_HOMALT=0.0;FREQ_HOMREF=0.9999029973809292;N_BI_GENOS=10309;East_Han_AN=6554;East_Han_AC=1;East_Han_AF=0
chr4	14587236	.	C	<INS:ME:LINE1>	.	PASS	SVTYPE=INS:ME:LINE1;END=14587248;SVLEN=6000;AN=20618;AC=2;AF=9.700261907071492e-05;N_HET=2;N_HOMALT=0;N_HOMREF=10307;FREQ_HET=0.00019400523814142983;FREQ_HOMALT=0.0;FREQ_HOMREF=0.9998059947618586;N_BI_GENOS=10309;East_Han_AN=6554;East_Han_AC=2;East_Han_AF
chr4	14634181	.	A	<INS:ME:LINE1>	.	PASS	SVTYPE=INS:ME:LINE1;END=14634196;SVLEN=350;AN=20618;AC=3;AF=0.00014550392860607236;N_HET=3;N_HOMALT=0;N_HOMREF=10306;FREQ_HET=0.0002910078572121447;FREQ_HOMALT=0.0;FREQ_HOMREF=0.9997089921427879;N_BI_GENOS=10309;Northwest_Han_AN=1296;Northwest_Han_AC=1;No
chr4	14737846	.	A	<INS:ME:LINE1>	.	PASS	SVTYPE=INS:ME:LINE1;END=14737864;SVLEN=1296;AN=20618;AC=1;AF=4.850130953535746e-05;N_HET=1;N_HOMALT=0;N_HOMREF=10308;FREQ_HET=9.700261907071492e-05;FREQ_HOMALT=0.0;FREQ_HOMREF=0.9999029973809292;N_BI_GENOS=10309;East_Han_AN=6554;East_Han_AC=1;East_Han_AF=
chr4	14773615	.	G	<INS:ME:LINE1>	.	PASS	SVTYPE=INS:ME:LINE1;END=14773615;SVLEN=293;AN=20618;AC=1;AF=4.850130953535746e-05;N_HET=1;N_HOMALT=0;N_HOMREF=10308;FREQ_HET=9.700261907071492e-05;FREQ_HOMALT=0.0;FREQ_HOMREF=0.9999029973809292;N_BI_GENOS=10309;East_Han_AN=6554;East_Han_AC=1;East_Han_AF=0
chr4	14847400	.	A	<INS:ME:LINE1>	.	PASS	SVTYPE=INS:ME:LINE1;END=14847414;SVLEN=5410;AN=20618;AC=31;AF=0.0015035405955960812;N_HET=31;N_HOMALT=0;N_HOMREF=10278;FREQ_HET=0.0030070811911921624;FREQ_HOMALT=0.0;FREQ_HOMREF=0.9969929188088078;N_BI_GENOS=10309;South_Han_AN=1496;South_Han_AC=1;South_Ha
chr4	14896392	.	A	<INS:ME:LINE1>	.	PASS	SVTYPE=INS:ME:LINE1;END=14896404;SVLEN=215;AN=20618;AC=1;AF=4.850130953535746e-05;N_HET=1;N_HOMALT=0;N_HOMREF=10308;FREQ_HET=9.700261907071492e-05;FREQ_HOMALT=0.0;FREQ_HOMREF=0.9999029973809292;N_BI_GENOS=10309;East_Han_AN=6554;East_Han_AC=1;East_Han_AF=0
chr4	15172053	.	T	<INS:ME:LINE1>	.	PASS	SVTYPE=INS:ME:LINE1;END=15172064;SVLEN=535;AN=20618;AC=1;AF=4.850130953535746e-05;N_HET=1;N_HOMALT=0;N_HOMREF=10308;FREQ_HET=9.700261907071492e-05;FREQ_HOMALT=0.0;FREQ_HOMREF=0.9999029973809292;N_BI_GENOS=10309;Central_Han_AN=2128;Central_Han_AC=1;Central
chr4	15502890	.	A	<INS:ME:LINE1>	.	PASS	SVTYPE=INS:ME:LINE1;END=15502903;SVLEN=6027;AN=20618;AC=3;AF=0.00014550392860607236;N_HET=3;N_HOMALT=0;N_HOMREF=10306;FREQ_HET=0.0002910078572121447;FREQ_HOMALT=0.0;FREQ_HOMREF=0.9997089921427879;N_BI_GENOS=10309;South_Han_AN=1496;South_Han_AC=1;South_Han
chr4	15870545	.	T	<INS:ME:LINE1>	.	PASS	SVTYPE=INS:ME:LINE1;END=15870558;SVLEN=5990;AN=20618;AC=2;AF=9.700261907071492e-05;N_HET=2;N_HOMALT=0;N_HOMREF=10307;FREQ_HET=0.00019400523814142983;FREQ_HOMALT=0.0;FREQ_HOMREF=0.9998059947618586;N_BI_GENOS=10309;North_Han_AN=2272;North_Han_AC=1;North_Han
chr4	16470699	.	G	<INS:ME:LINE1>	.	PASS	SVTYPE=INS:ME:LINE1;END=16470715;SVLEN=6000;AN=20618;AC=1;AF=4.850130953535746e-05;N_HET=1;N_HOMALT=0;N_HOMREF=10308;FREQ_HET=9.700261907071492e-05;FREQ_HOMALT=0.0;FREQ_HOMREF=0.9999029973809292;N_BI_GENOS=10309;East_Han_AN=6554;East_Han_AC=1;East_Han_AF=
chr4	16590042	.	A	<INS:ME:LINE1>	.	PASS	SVTYPE=INS:ME:LINE1;END=16590058;SVLEN=1084;AN=20618;AC=1;AF=4.850130953535746e-05;N_HET=1;N_HOMALT=0;N_HOMREF=10308;FREQ_HET=9.700261907071492e-05;FREQ_HOMALT=0.0;FREQ_HOMREF=0.9999029973809292;N_BI_GENOS=10309;Zhuang_AN=444;Zhuang_AC=1;Zhuang_AF=0.00225
chr4	17135645	.	A	<INS:ME:LINE1>	.	PASS	SVTYPE=INS:ME:LINE1;END=17135659;SVLEN=5998;AN=20618;AC=1;AF=4.850130953535746e-05;N_HET=1;N_HOMALT=0;N_HOMREF=10308;FREQ_HET=9.700261907071492e-05;FREQ_HOMALT=0.0;FREQ_HOMREF=0.9999029973809292;N_BI_GENOS=10309;Manchu_AN=416;Manchu_AC=1;Manchu_AF=0.00240
chr4	17181683	.	A	<INS:ME:LINE1>	.	PASS	SVTYPE=INS:ME:LINE1;END=17181699;SVLEN=2177;AN=20618;AC=1;AF=4.850130953535746e-05;N_HET=1;N_HOMALT=0;N_HOMREF=10308;FREQ_HET=9.700261907071492e-05;FREQ_HOMALT=0.0;FREQ_HOMREF=0.9999029973809292;N_BI_GENOS=10309;East_Han_AN=6554;East_Han_AC=1;East_Han_AF=
chr4	17381440	.	G	<INS:ME:LINE1>	.	PASS	SVTYPE=INS:ME:LINE1;END=17381454;SVLEN=6002;AN=20618;AC=1;AF=4.850130953535746e-05;N_HET=1;N_HOMALT=0;N_HOMREF=10308;FREQ_HET=9.700261907071492e-05;FREQ_HOMALT=0.0;FREQ_HOMREF=0.9999029973809292;N_BI_GENOS=10309;North_Han_AN=2272;North_Han_AC=1;North_Han_
chr4	17791257	.	T	<INS:ME:LINE1>	.	PASS	SVTYPE=INS:ME:LINE1;END=17791257;SVLEN=6000;AN=20618;AC=1;AF=4.850130953535746e-05;N_HET=1;N_HOMALT=0;N_HOMREF=10308;FREQ_HET=9.700261907071492e-05;FREQ_HOMALT=0.0;FREQ_HOMREF=0.9999029973809292;N_BI_GENOS=10309;East_Han_AN=6554;East_Han_AC=1;East_Han_AF=
chr4	18083277	.	A	<INS:ME:LINE1>	.	PASS	SVTYPE=INS:ME:LINE1;END=18083277;SVLEN=809;AN=20618;AC=1;AF=4.850130953535746e-05;N_HET=1;N_HOMALT=0;N_HOMREF=10308;FREQ_HET=9.700261907071492e-05;FREQ_HOMALT=0.0;FREQ_HOMREF=0.9999029973809292;N_BI_GENOS=10309;Lingnan_Han_AN=1888;Lingnan_Han_AC=1;Lingnan
chr4	18128790	.	T	<INS:ME:LINE1>	.	PASS	SVTYPE=INS:ME:LINE1;END=18128805;SVLEN=6009;AN=20618;AC=1;AF=4.850130953535746e-05;N_HET=1;N_HOMALT=0;N_HOMREF=10308;FREQ_HET=9.700261907071492e-05;FREQ_HOMALT=0.0;FREQ_HOMREF=0.9999029973809292;N_BI_GENOS=10309;East_Han_AN=6554;East_Han_AC=1;East_Han_AF=
chr4	18161056	.	A	<INS:ME:LINE1>	.	PASS	SVTYPE=INS:ME:LINE1;END=18161066;SVLEN=1554;AN=20618;AC=14057;AF=0.6817829081385197;N_HET=4267;N_HOMALT=4895;N_HOMREF=1147;FREQ_HET=0.4139101755747405;FREQ_HOMALT=0.47482782035114945;FREQ_HOMREF=0.11126200407411001;N_BI_GENOS=10309;South_Han_AN=1496;South
chr4	18192723	.	A	<INS:ME:LINE1>	.	PASS	SVTYPE=INS:ME:LINE1;END=18192734;SVLEN=2542;AN=20618;AC=1863;AF=0.09035793966437093;N_HET=1679;N_HOMALT=92;N_HOMREF=8538;FREQ_HET=0.16286739741973033;FREQ_HOMALT=0.008924240954505771;FREQ_HOMREF=0.828208361625764;N_BI_GENOS=10309;South_Han_AN=1496;South_H
chr4	18280203	.	G	<INS:ME:LINE1>	.	PASS	SVTYPE=INS:ME:LINE1;END=18280217;SVLEN=981;AN=20618;AC=1;AF=4.850130953535746e-05;N_HET=1;N_HOMALT=0;N_HOMREF=10308;FREQ_HET=9.700261907071492e-05;FREQ_HOMALT=0.0;FREQ_HOMREF=0.9999029973809292;N_BI_GENOS=10309;Southeast_Han_AN=1908;Southeast_Han_AC=1;Sou
chr4	18361121	.	T	<INS:ME:LINE1>	.	PASS	SVTYPE=INS:ME:LINE1;END=18361136;SVLEN=771;AN=20618;AC=2;AF=9.700261907071492e-05;N_HET=2;N_HOMALT=0;N_HOMREF=10307;FREQ_HET=0.00019400523814142983;FREQ_HOMALT=0.0;FREQ_HOMREF=0.9998059947618586;N_BI_GENOS=10309;North_Han_AN=2272;North_Han_AC=1;North_Han_
chr4	18427367	.	T	<INS:ME:LINE1>	.	PASS	SVTYPE=INS:ME:LINE1;END=18427384;SVLEN=2873;AN=20618;AC=1;AF=4.850130953535746e-05;N_HET=1;N_HOMALT=0;N_HOMREF=10308;FREQ_HET=9.700261907071492e-05;FREQ_HOMALT=0.0;FREQ_HOMREF=0.9999029973809292;N_BI_GENOS=10309;East_Han_AN=6554;East_Han_AC=1;East_Han_AF=
chr4	18458019	.	A	<INS:ME:LINE1>	.	PASS	SVTYPE=INS:ME:LINE1;END=18458035;SVLEN=517;AN=20618;AC=2;AF=9.700261907071492e-05;N_HET=2;N_HOMALT=0;N_HOMREF=10307;FREQ_HET=0.00019400523814142983;FREQ_HOMALT=0.0;FREQ_HOMREF=0.9998059947618586;N_BI_GENOS=10309;Lingnan_Han_AN=1888;Lingnan_Han_AC=1;Lingna
chr4	18975140	.	A	<INS:ME:LINE1>	.	PASS	SVTYPE=INS:ME:LINE1;END=18975152;SVLEN=2859;AN=20618;AC=1;AF=4.850130953535746e-05;N_HET=1;N_HOMALT=0;N_HOMREF=10308;FREQ_HET=9.700261907071492e-05;FREQ_HOMALT=0.0;FREQ_HOMREF=0.9999029973809292;N_BI_GENOS=10309;East_Han_AN=6554;East_Han_AC=1;East_Han_AF=
chr4	19228297	.	A	<INS:ME:LINE1>	.	PASS	SVTYPE=INS:ME:LINE1;END=19228310;SVLEN=1873;AN=20618;AC=1;AF=4.850130953535746e-05;N_HET=1;N_HOMALT=0;N_HOMREF=10308;FREQ_HET=9.700261907071492e-05;FREQ_HOMALT=0.0;FREQ_HOMREF=0.9999029973809292;N_BI_GENOS=10309;East_Han_AN=6554;East_Han_AC=1;East_Han_AF=
chr4	19390186	.	C	<INS:ME:LINE1>	.	PASS	SVTYPE=INS:ME:LINE1;END=19390186;SVLEN=6000;AN=20618;AC=1;AF=4.850130953535746e-05;N_HET=1;N_HOMALT=0;N_HOMREF=10308;FREQ_HET=9.700261907071492e-05;FREQ_HOMALT=0.0;FREQ_HOMREF=0.9999029973809292;N_BI_GENOS=10309;Central_Han_AN=2128;Central_Han_AC=1;Centra
chr4	19557114	.	A	<INS:ME:LINE1>	.	PASS	SVTYPE=INS:ME:LINE1;END=19557132;SVLEN=1904;AN=20618;AC=6;AF=0.0002910078572121447;N_HET=6;N_HOMALT=0;N_HOMREF=10303;FREQ_HET=0.0005820157144242894;FREQ_HOMALT=0.0;FREQ_HOMREF=0.9994179842855757;N_BI_GENOS=10309;Lingnan_Han_AN=1888;Lingnan_Han_AC=2;Lingna
chr4	20115552	.	A	<INS:ME:LINE1>	.	PASS	SVTYPE=INS:ME:LINE1;END=20115568;SVLEN=322;AN=20618;AC=3;AF=0.00014550392860607236;N_HET=3;N_HOMALT=0;N_HOMREF=10306;FREQ_HET=0.0002910078572121447;FREQ_HOMALT=0.0;FREQ_HOMREF=0.9997089921427879;N_BI_GENOS=10309;North_Han_AN=2272;North_Han_AC=1;North_Han_
chr4	20181952	.	C	<INS:ME:LINE1>	.	PASS	SVTYPE=INS:ME:LINE1;END=20181970;SVLEN=671;AN=20618;AC=1;AF=4.850130953535746e-05;N_HET=1;N_HOMALT=0;N_HOMREF=10308;FREQ_HET=9.700261907071492e-05;FREQ_HOMALT=0.0;FREQ_HOMREF=0.9999029973809292;N_BI_GENOS=10309;Central_Han_AN=2128;Central_Han_AC=1;Central
chr4	20189267	.	T	<INS:ME:LINE1>	.	PASS	SVTYPE=INS:ME:LINE1;END=20189272;SVLEN=767;AN=20618;AC=1;AF=4.850130953535746e-05;N_HET=1;N_HOMALT=0;N_HOMREF=10308;FREQ_HET=9.700261907071492e-05;FREQ_HOMALT=0.0;FREQ_HOMREF=0.9999029973809292;N_BI_GENOS=10309;Hui_AN=444;Hui_AC=1;Hui_AF=0.002252252252252
chr4	20193946	.	A	<INS:ME:LINE1>	.	PASS	SVTYPE=INS:ME:LINE1;END=20193963;SVLEN=504;AN=20618;AC=1;AF=4.850130953535746e-05;N_HET=1;N_HOMALT=0;N_HOMREF=10308;FREQ_HET=9.700261907071492e-05;FREQ_HOMALT=0.0;FREQ_HOMREF=0.9999029973809292;N_BI_GENOS=10309;Zhuang_AN=444;Zhuang_AC=1;Zhuang_AF=0.002252
chr4	20427501	.	A	<INS:ME:LINE1>	.	PASS	SVTYPE=INS:ME:LINE1;END=20427514;SVLEN=860;AN=20618;AC=1;AF=4.850130953535746e-05;N_HET=1;N_HOMALT=0;N_HOMREF=10308;FREQ_HET=9.700261907071492e-05;FREQ_HOMALT=0.0;FREQ_HOMREF=0.9999029973809292;N_BI_GENOS=10309;Northwest_Han_AN=1296;Northwest_Han_AC=1;Nor
chr4	20645171	.	A	<INS:ME:LINE1>	.	PASS	SVTYPE=INS:ME:LINE1;END=20645182;SVLEN=6086;AN=20618;AC=3;AF=0.00014550392860607236;N_HET=3;N_HOMALT=0;N_HOMREF=10306;FREQ_HET=0.0002910078572121447;FREQ_HOMALT=0.0;FREQ_HOMREF=0.9997089921427879;N_BI_GENOS=10309;North_Han_AN=2272;North_Han_AC=1;North_Han
chr4	20798078	.	C	<INS:ME:LINE1>	.	PASS	SVTYPE=INS:ME:LINE1;END=20798097;SVLEN=342;AN=20618;AC=2;AF=9.700261907071492e-05;N_HET=2;N_HOMALT=0;N_HOMREF=10307;FREQ_HET=0.00019400523814142983;FREQ_HOMALT=0.0;FREQ_HOMREF=0.9998059947618586;N_BI_GENOS=10309;East_Han_AN=6554;East_Han_AC=2;East_Han_AF=
chr4	20875742	.	T	<INS:ME:LINE1>	.	PASS	SVTYPE=INS:ME:LINE1;END=20875742;SVLEN=194;AN=20618;AC=1;AF=4.850130953535746e-05;N_HET=1;N_HOMALT=0;N_HOMREF=10308;FREQ_HET=9.700261907071492e-05;FREQ_HOMALT=0.0;FREQ_HOMREF=0.9999029973809292;N_BI_GENOS=10309;Lingnan_Han_AN=1888;Lingnan_Han_AC=1;Lingnan
chr4	21382296	.	T	<INS:ME:LINE1>	.	PASS	SVTYPE=INS:ME:LINE1;END=21382296;SVLEN=376;AN=20618;AC=3;AF=0.00014550392860607236;N_HET=1;N_HOMALT=1;N_HOMREF=10307;FREQ_HET=9.700261907071492e-05;FREQ_HOMALT=9.700261907071492e-05;FREQ_HOMREF=0.9998059947618586;N_BI_GENOS=10309;East_Han_AN=6554;East_Han
chr4	21468220	.	A	<INS:ME:LINE1>	.	PASS	SVTYPE=INS:ME:LINE1;END=21468236;SVLEN=738;AN=20618;AC=1;AF=4.850130953535746e-05;N_HET=1;N_HOMALT=0;N_HOMREF=10308;FREQ_HET=9.700261907071492e-05;FREQ_HOMALT=0.0;FREQ_HOMREF=0.9999029973809292;N_BI_GENOS=10309;Northwest_Han_AN=1296;Northwest_Han_AC=1;Nor
chr4	21922548	.	A	<INS:ME:LINE1>	.	PASS	SVTYPE=INS:ME:LINE1;END=21922556;SVLEN=58;AN=20618;AC=5;AF=0.00024250654767678728;N_HET=5;N_HOMALT=0;N_HOMREF=10304;FREQ_HET=0.00048501309535357455;FREQ_HOMALT=0.0;FREQ_HOMREF=0.9995149869046465;N_BI_GENOS=10309;Northwest_Han_AN=1296;Northwest_Han_AC=2;No
chr4	21947476	.	G	<INS:ME:LINE1>	.	PASS	SVTYPE=INS:ME:LINE1;END=21947492;SVLEN=1803;AN=20618;AC=1;AF=4.850130953535746e-05;N_HET=1;N_HOMALT=0;N_HOMREF=10308;FREQ_HET=9.700261907071492e-05;FREQ_HOMALT=0.0;FREQ_HOMREF=0.9999029973809292;N_BI_GENOS=10309;Southeast_Han_AN=1908;Southeast_Han_AC=1;So
chr4	22059622	.	T	<INS:ME:LINE1>	.	PASS	SVTYPE=INS:ME:LINE1;END=22059637;SVLEN=1916;AN=20618;AC=4;AF=0.00019400523814142983;N_HET=4;N_HOMALT=0;N_HOMREF=10305;FREQ_HET=0.00038801047628285966;FREQ_HOMALT=0.0;FREQ_HOMREF=0.9996119895237171;N_BI_GENOS=10309;Lingnan_Han_AN=1888;Lingnan_Han_AC=2;Ling
chr4	22155090	.	T	<INS:ME:LINE1>	.	PASS	SVTYPE=INS:ME:LINE1;END=22155091;SVLEN=5289;AN=20618;AC=1;AF=4.850130953535746e-05;N_HET=1;N_HOMALT=0;N_HOMREF=10308;FREQ_HET=9.700261907071492e-05;FREQ_HOMALT=0.0;FREQ_HOMREF=0.9999029973809292;N_BI_GENOS=10309;East_Han_AN=6554;East_Han_AC=1;East_Han_AF=
chr4	22205385	.	A	<INS:ME:LINE1>	.	PASS	SVTYPE=INS:ME:LINE1;END=22205400;SVLEN=5999;AN=20618;AC=2;AF=9.700261907071492e-05;N_HET=2;N_HOMALT=0;N_HOMREF=10307;FREQ_HET=0.00019400523814142983;FREQ_HOMALT=0.0;FREQ_HOMREF=0.9998059947618586;N_BI_GENOS=10309;Miao_AN=482;Miao_AC=2;Miao_AF=0.0041493775
chr4	22368385	.	A	<INS:ME:LINE1>	.	PASS	SVTYPE=INS:ME:LINE1;END=22368395;SVLEN=5988;AN=20618;AC=37;AF=0.0017945484528082258;N_HET=37;N_HOMALT=0;N_HOMREF=10272;FREQ_HET=0.0035890969056164516;FREQ_HOMALT=0.0;FREQ_HOMREF=0.9964109030943835;N_BI_GENOS=10309;South_Han_AN=1496;South_Han_AC=5;South_Ha
chr4	22712153	.	A	<INS:ME:LINE1>	.	PASS	SVTYPE=INS:ME:LINE1;END=22712153;SVLEN=102;AN=20618;AC=1;AF=4.850130953535746e-05;N_HET=1;N_HOMALT=0;N_HOMREF=10308;FREQ_HET=9.700261907071492e-05;FREQ_HOMALT=0.0;FREQ_HOMREF=0.9999029973809292;N_BI_GENOS=10309;North_Han_AN=2272;North_Han_AC=1;North_Han_A
chr4	22821180	.	G	<INS:ME:LINE1>	.	PASS	SVTYPE=INS:ME:LINE1;END=22821191;SVLEN=5999;AN=20618;AC=1;AF=4.850130953535746e-05;N_HET=1;N_HOMALT=0;N_HOMREF=10308;FREQ_HET=9.700261907071492e-05;FREQ_HOMALT=0.0;FREQ_HOMREF=0.9999029973809292;N_BI_GENOS=10309;East_Han_AN=6554;East_Han_AC=1;East_Han_AF=

chr4	22882880	.	A	<INS:ME:LINE1>	.	PASS	SVTYPE=INS:ME:LINE1;END=22882890;SVLEN=6065;AN=20618;AC=1;AF=4.850130953535746e-05;N_HET=1;N_HOMALT=0;N_HOMREF=10308;FREQ_HET=9.700261907071492e-05;FREQ_HOMALT=0.0;FREQ_HOMREF=0.9999029973809292;N_BI_GENOS=10309;Tibetan_AN=414;Tibetan_AC=1;Tibetan_AF=0.00
chr4	22974954	.	A	<INS:ME:LINE1>	.	PASS	SVTYPE=INS:ME:LINE1;END=22974955;SVLEN=462;AN=20618;AC=2;AF=9.700261907071492e-05;N_HET=2;N_HOMALT=0;N_HOMREF=10307;FREQ_HET=0.0001940052381442983;FREQ_HOMALT=0.0;FREQ_HOMREF=0.9998059947618586;N_BI_GENOS=10309;Mongolian_AN=454;Mongolian_AC=2;Mongolian_A
chr4	23017275	.	T	<INS:ME:LINE1>	.	PASS	SVTYPE=INS:ME:LINE1;END=23017276;SVLEN=78;AN=20618;AC=2;AF=9.700261907071492e-05;N_HET=2;N_HOMALT=0;N_HOMREF=10307;FREQ_HET=0.0001940052381442983;FREQ_HOMALT=0.0;FREQ_HOMREF=0.9998059947618586;N_BI_GENOS=10309;East_Han_AN=6554;East_Han_AC=1;East_Han_AF=0
chr4	23175424	.	T	<INS:ME:LINE1>	.	PASS	SVTYPE=INS:ME:LINE1;END=23175433;SVLEN=557;AN=20618;AC=1;AF=4.850130953535746e-05;N_HET=1;N_HOMALT=0;N_HOMREF=10308;FREQ_HET=9.700261907071492e-05;FREQ_HOMALT=0.0;FREQ_HOMREF=0.9999029973809292;N_BI_GENOS=10309;Manchu_AN=416;Manchu_AC=1;Manchu_AF=0.002403
chr4	23300811	.	A	<INS:ME:LINE1>	.	PASS	SVTYPE=INS:ME:LINE1;END=23300826;SVLEN=468;AN=20618;AC=3;AF=0.00014550392860607236;N_HET=3;N_HOMALT=0;N_HOMREF=10306;FREQ_HET=0.0002910078572121447;FREQ_HOMALT=0.0;FREQ_HOMREF=0.9997089921427879;N_BI_GENOS=10309;South_Han_AN=1496;South_Han_AC=1;South_Han_
chr4	23404230	.	G	<INS:ME:LINE1>	.	PASS	SVTYPE=INS:ME:LINE1;END=23404230;SVLEN=269;AN=20618;AC=1;AF=4.850130953535746e-05;N_HET=1;N_HOMALT=0;N_HOMREF=10308;FREQ_HET=9.700261907071492e-05;FREQ_HOMALT=0.0;FREQ_HOMREF=0.9999029973809292;N_BI_GENOS=10309;East_Han_AN=6554;East_Han_AC=1;East_Han_AF=0
chr4	23525712	.	A	<INS:ME:LINE1>	.	PASS	SVTYPE=INS:ME:LINE1;END=23525714;SVLEN=4096;AN=20618;AC=1;AF=4.850130953535746e-05;N_HET=1;N_HOMALT=0;N_HOMREF=10308;FREQ_HET=9.700261907071492e-05;FREQ_HOMALT=0.0;FREQ_HOMREF=0.9999029973809292;N_BI_GENOS=10309;East_Han_AN=6554;East_Han_AC=1;East_Han_AF=
chr4	23671438	.	C	<INS:ME:LINE1>	.	PASS	SVTYPE=INS:ME:LINE1;END=23671453;SVLEN=5790;AN=20618;AC=1;AF=4.850130953535746e-05;N_HET=1;N_HOMALT=0;N_HOMREF=10308;FREQ_HET=9.700261907071492e-05;FREQ_HOMALT=0.0;FREQ_HOMREF=0.9999029973809292;N_BI_GENOS=10309;South_Han_AN=1496;South_Han_AC=1;South_Han_
chr4	24040773	.	T	<INS:ME:LINE1>	.	PASS	SVTYPE=INS:ME:LINE1;END=24040782;SVLEN=1839;AN=20618;AC=1;AF=4.850130953535746e-05;N_HET=1;N_HOMALT=0;N_HOMREF=10308;FREQ_HET=9.700261907071492e-05;FREQ_HOMALT=0.0;FREQ_HOMREF=0.9999029973809292;N_BI_GENOS=10309;East_Han_AN=6554;East_Han_AC=1;East_Han_AF=
chr4	24057312	.	G	<INS:ME:LINE1>	.	PASS	SVTYPE=INS:ME:LINE1;END=24057327;SVLEN=6006;AN=20618;AC=3;AF=0.00014550392860607236;N_HET=3;N_HOMALT=0;N_HOMREF=10306;FREQ_HET=0.0002910078572121447;FREQ_HOMALT=0.0;FREQ_HOMREF=0.9997089921427879;N_BI_GENOS=10309;East_Han_AN=6554;East_Han_AC=3;East_Han_AF
chr4	24207838	.	A	<INS:ME:LINE1>	.	PASS	SVTYPE=INS:ME:LINE1;END=24207853;SVLEN=1822;AN=20618;AC=1;AF=4.850130953535746e-05;N_HET=1;N_HOMALT=0;N_HOMREF=10308;FREQ_HET=9.700261907071492e-05;FREQ_HOMALT=0.0;FREQ_HOMREF=0.9999029973809292;N_BI_GENOS=10309;East_Han_AN=6554;East_Han_AC=1;East_Han_AF=
chr4	24270472	.	A	<INS:ME:LINE1>	.	PASS	SVTYPE=INS:ME:LINE1;END=24270488;SVLEN=6004;AN=20618;AC=1;AF=4.850130953535746e-05;N_HET=1;N_HOMALT=0;N_HOMREF=10308;FREQ_HET=9.700261907071492e-05;FREQ_HOMALT=0.0;FREQ_HOMREF=0.9999029973809292;N_BI_GENOS=10309;Northwest_Han_AN=1296;Northwest_Han_AC=1;No
chr4	24614864	.	T	<INS:ME:LINE1>	.	PASS	SVTYPE=INS:ME:LINE1;END=24614873;SVLEN=6010;AN=20618;AC=3;AF=0.00014550392860607236;N_HET=3;N_HOMALT=0;N_HOMREF=10306;FREQ_HET=0.0002910078572121447;FREQ_HOMALT=0.0;FREQ_HOMREF=0.9997089921427879;N_BI_GENOS=10309;North_Han_AN=2272;North_Han_AC=1;North_Han
chr4	24724407	.	T	<INS:ME:LINE1>	.	PASS	SVTYPE=INS:ME:LINE1;END=24724423;SVLEN=6000;AN=20618;AC=1;AF=4.850130953535746e-05;N_HET=1;N_HOMALT=0;N_HOMREF=10308;FREQ_HET=9.700261907071492e-05;FREQ_HOMALT=0.0;FREQ_HOMREF=0.9999029973809292;N_BI_GENOS=10309;North_Han_AN=2272;North_Han_AC=1;North_Han_
chr4	24823298	.	A	<INS:ME:LINE1>	.	PASS	SVTYPE=INS:ME:LINE1;END=24823315;SVLEN=330;AN=20618;AC=1;AF=4.850130953535746e-05;N_HET=1;N_HOMALT=0;N_HOMREF=10308;FREQ_HET=9.700261907071492e-05;FREQ_HOMALT=0.0;FREQ_HOMREF=0.9999029973809292;N_BI_GENOS=10309;Central_Han_AN=2128;Central_Han_AC=1;Central
chr4	25491953	.	G	<INS:ME:LINE1>	.	PASS	SVTYPE=INS:ME:LINE1;END=25491970;SVLEN=546;AN=20618;AC=125;AF=0.006062566369191968;N_HET=125;N_HOMALT=0;N_HOMREF=10184;FREQ_HET=0.012125327383839363;FREQ_HOMALT=0.0;FREQ_HOMREF=0.9878746726161607;N_BI_GENOS=10309;South_Han_AN=1496;South_Han_AC=9;South_Ha
chr4	25644471	.	T	<INS:ME:LINE1>	.	PASS	SVTYPE=INS:ME:LINE1;END=25644487;SVLEN=5996;AN=20618;AC=1;AF=4.850130953535746e-05;N_HET=1;N_HOMALT=0;N_HOMREF=10308;FREQ_HET=9.700261907071492e-05;FREQ_HOMALT=0.0;FREQ_HOMREF=0.9999029973809292;N_BI_GENOS=10309;South_Han_AN=1496;South_Han_AC=1;South_Han_
chr4	26154640	.	A	<INS:ME:LINE1>	.	PASS	SVTYPE=INS:ME:LINE1;END=26154645;SVLEN=718;AN=20618;AC=2;AF=9.700261907071492e-05;N_HET=2;N_HOMALT=0;N_HOMREF=10307;FREQ_HET=0.0001940052381442983;FREQ_HOMALT=0.0;FREQ_HOMREF=0.9998059947618586;N_BI_GENOS=10309;East_Han_AN=6554;East_Han_AC=2;East_Han_AF=
chr4	26770136	.	A	<INS:ME:LINE1>	.	PASS	SVTYPE=INS:ME:LINE1;END=26770147;SVLEN=1010;AN=20618;AC=1;AF=4.850130953535746e-05;N_HET=1;N_HOMALT=0;N_HOMREF=10308;FREQ_HET=9.700261907071492e-05;FREQ_HOMALT=0.0;FREQ_HOMREF=0.9999029973809292;N_BI_GENOS=10309;Central_Han_AN=2128;Central_Han_AC=1;Centra
chr4	26775496	.	G	<INS:ME:LINE1>	.	PASS	SVTYPE=INS:ME:LINE1;END=26775512;SVLEN=1253;AN=20618;AC=1;AF=4.850130953535746e-05;N_HET=1;N_HOMALT=0;N_HOMREF=10308;FREQ_HET=9.700261907071492e-05;FREQ_HOMALT=0.0;FREQ_HOMREF=0.9999029973809292;N_BI_GENOS=10309;Tibetan_AN=414;Tibetan_AC=1;Tibetan_AF=0.00
chr4	27354483	.	G	<INS:ME:LINE1>	.	PASS	SVTYPE=INS:ME:LINE1;END=27354498;SVLEN=6003;AN=20618;AC=1;AF=4.850130953535746e-05;N_HET=1;N_HOMALT=0;N_HOMREF=10308;FREQ_HET=9.700261907071492e-05;FREQ_HOMALT=0.0;FREQ_HOMREF=0.9999029973809292;N_BI_GENOS=10309;East_Han_AN=6554;East_Han_AC=1;East_Han_AF=
chr4	27598929	.	T	<INS:ME:LINE1>	.	PASS	SVTYPE=INS:ME:LINE1;END=27598945;SVLEN=1947;AN=20618;AC=1;AF=4.850130953535746e-05;N_HET=1;N_HOMALT=0;N_HOMREF=10308;FREQ_HET=9.700261907071492e-05;FREQ_HOMALT=0.0;FREQ_HOMREF=0.9999029973809292;N_BI_GENOS=10309;North_Han_AN=2272;North_Han_AC=1;North_Han_
chr4	27672988	.	A	<INS:ME:LINE1>	.	PASS	SVTYPE=INS:ME:LINE1;END=27673005;SVLEN=1445;AN=20618;AC=1;AF=4.850130953535746e-05;N_HET=1;N_HOMALT=0;N_HOMREF=10308;FREQ_HET=9.700261907071492e-05;FREQ_HOMALT=0.0;FREQ_HOMREF=0.9999029973809292;N_BI_GENOS=10309;Miao_AN=482;Miao_AC=1;Miao_AF=0.00207468879
chr4	27739365	.	C	<INS:ME:LINE1>	.	PASS	SVTYPE=INS:ME:LINE1;END=27739380;SVLEN=803;AN=20618;AC=5;AF=0.00024250654767678728;N_HET=5;N_HOMALT=0;N_HOMREF=10304;FREQ_HET=0.0004850130953535455;FREQ_HOMALT=0.0;FREQ_HOMREF=0.9995149869046465;N_BI_GENOS=10309;South_Han_AN=1496;South_Han_AC=1;South_Han
chr4	27773152	.	C	<INS:ME:LINE1>	.	PASS	SVTYPE=INS:ME:LINE1;END=27773164;SVLEN=91;AN=20618;AC=2;AF=9.700261907071492e-05;N_HET=2;N_HOMALT=0;N_HOMREF=10307;FREQ_HET=0.0001940052381442983;FREQ_HOMALT=0.0;FREQ_HOMREF=0.9998059947618586;N_BI_GENOS=10309;East_Han_AN=6554;East_Han_AC=2;East_Han_AF=0
chr4	27849390	.	A	<INS:ME:LINE1>	.	PASS	SVTYPE=INS:ME:LINE1;END=27849391;SVLEN=5249;AN=20618;AC=1;AF=4.850130953535746e-05;N_HET=1;N_HOMALT=0;N_HOMREF=10308;FREQ_HET=9.700261907071492e-05;FREQ_HOMALT=0.0;FREQ_HOMREF=0.9999029973809292;N_BI_GENOS=10309;North_Han_AN=2272;North_Han_AC=1;North_Han_
chr4	27882679	.	A	<INS:ME:LINE1>	.	PASS	SVTYPE=INS:ME:LINE1;END=27882703;SVLEN=745;AN=20618;AC=1;AF=4.850130953535746e-05;N_HET=1;N_HOMALT=0;N_HOMREF=10308;FREQ_HET=9.700261907071492e-05;FREQ_HOMALT=0.0;FREQ_HOMREF=0.9999029973809292;N_BI_GENOS=10309;Central_Han_AN=2128;Central_Han_AC=1;Central
chr4	28151689	.	G	<INS:ME:LINE1>	.	PASS	SVTYPE=INS:ME:LINE1;END=28151704;SVLEN=784;AN=20618;AC=2;AF=9.700261907071492e-05;N_HET=2;N_HOMALT=0;N_HOMREF=10307;FREQ_HET=0.0001940052381442983;FREQ_HOMALT=0.0;FREQ_HOMREF=0.9998059947618586;N_BI_GENOS=10309;Lingnan_Han_AN=1888;Lingnan_Han_AC=1;Lingna
chr4	28164676	.	A	<INS:ME:LINE1>	.	PASS	SVTYPE=INS:ME:LINE1;END=28164692;SVLEN=1659;AN=20618;AC=1;AF=4.850130953535746e-05;N_HET=1;N_HOMALT=0;N_HOMREF=10308;FREQ_HET=9.700261907071492e-05;FREQ_HOMALT=0.0;FREQ_HOMREF=0.9999029973809292;N_BI_GENOS=10309;Central_Han_AN=2128;Central_Han_AC=1;Centra
chr4	28238160	.	G	<INS:ME:LINE1>	.	PASS	SVTYPE=INS:ME:LINE1;END=28238193;SVLEN=1983;AN=20618;AC=21;AF=0.0010185275002425065;N_HET=19;N_HOMALT=1;N_HOMREF=10289;FREQ_HET=0.0018430497623435833;FREQ_HOMALT=9.700261907071492e-05;FREQ_HOMREF=0.9980599476185857;N_BI_GENOS=10309;South_Han_AN=1496;South
chr4	28249225	.	A	<INS:ME:LINE1>	.	PASS	SVTYPE=INS:ME:LINE1;END=28249241;SVLEN=32;AN=20618;AC=1;AF=4.850130953535746e-05;N_HET=1;N_HOMALT=0;N_HOMREF=10308;FREQ_HET=9.700261907071492e-05;FREQ_HOMALT=0.0;FREQ_HOMREF=0.9999029973809292;N_BI_GENOS=10309;South_Han_AN=1496;South_Han_AC=1;South_Han_AF
chr4	28435257	.	G	<INS:ME:LINE1>	.	PASS	SVTYPE=INS:ME:LINE1;END=28435284;SVLEN=641;AN=20618;AC=1;AF=4.850130953535746e-05;N_HET=1;N_HOMALT=0;N_HOMREF=10308;FREQ_HET=9.700261907071492e-05;FREQ_HOMALT=0.0;FREQ_HOMREF=0.9999029973809292;N_BI_GENOS=10309;East_Han_AN=6554;East_Han_AC=1;East_Han_AF=0
chr4	28590637	.	A	<INS:ME:LINE1>	.	PASS	SVTYPE=INS:ME:LINE1;END=28590649;SVLEN=5994;AN=20618;AC=1;AF=4.850130953535746e-05;N_HET=1;N_HOMALT=0;N_HOMREF=10308;FREQ_HET=9.700261907071492e-05;FREQ_HOMALT=0.0;FREQ_HOMREF=0.9999029973809292;N_BI_GENOS=10309;East_Han_AN=6554;East_Han_AC=1;East_Han_AF=
chr4	28768656	.	A	<INS:ME:LINE1>	.	PASS	SVTYPE=INS:ME:LINE1;END=28768656;SVLEN=1004;AN=20618;AC=1;AF=4.850130953535746e-05;N_HET=1;N_HOMALT=0;N_HOMREF=10308;FREQ_HET=9.700261907071492e-05;FREQ_HOMALT=0.0;FREQ_HOMREF=0.9999029973809292;N_BI_GENOS=10309;East_Han_AN=6554;East_Han_AC=1;East_Han_AF=
chr4	28769433	.	T	<INS:ME:LINE1>	.	PASS	SVTYPE=INS:ME:LINE1;END=28769433;SVLEN=558;AN=20618;AC=1;AF=4.850130953535746e-05;N_HET=1;N_HOMALT=0;N_HOMREF=10308;FREQ_HET=9.700261907071492e-05;FREQ_HOMALT=0.0;FREQ_HOMREF=0.9999029973809292;N_BI_GENOS=10309;East_Han_AN=6554;East_Han_AC=1;East_Han_AF=0
chr4	28776079	.	C	<INS:ME:LINE1>	.	PASS	SVTYPE=INS:ME:LINE1;END=28776095;SVLEN=6000;AN=20618;AC=1;AF=4.850130953535746e-05;N_HET=1;N_HOMALT=0;N_HOMREF=10308;FREQ_HET=9.700261907071492e-05;FREQ_HOMALT=0.0;FREQ_HOMREF=0.9999029973809292;N_BI_GENOS=10309;Central_Han_AN=2128;Central_Han_AC=1;Centra
chr4	29152188	.	T	<INS:ME:LINE1>	.	PASS	SVTYPE=INS:ME:LINE1;END=29152192;SVLEN=762;AN=20618;AC=1;AF=4.850130953535746e-05;N_HET=1;N_HOMALT=0;N_HOMREF=10308;FREQ_HET=9.700261907071492e-05;FREQ_HOMALT=0.0;FREQ_HOMREF=0.9999029973809292;N_BI_GENOS=10309;South_Han_AN=1496;South_Han_AC=1;South_Han_A
chr4	29320228	.	C	<INS:ME:LINE1>	.	PASS	SVTYPE=INS:ME:LINE1;END=29320239;SVLEN=1135;AN=20618;AC=146;AF=0.007081191192162189;N_HET=130;N_HOMALT=8;N_HOMREF=10171;FREQ_HET=0.012610340479192938;FREQ_HOMALT=0.0007760209525657193;FREQ_HOMREF=0.9866136385682414;N_BI_GENOS=10309;South_Han_AN=1496;South
chr4	29327749	.	A	<INS:ME:LINE1>	.	PASS	SVTYPE=INS:ME:LINE1;END=29327750;SVLEN=5999;AN=20618;AC=1;AF=4.850130953535746e-05;N_HET=1;N_HOMALT=0;N_HOMREF=10308;FREQ_HET=9.700261907071492e-05;FREQ_HOMALT=0.0;FREQ_HOMREF=0.9999029973809292;N_BI_GENOS=10309;East_Han_AN=6554;East_Han_AC=1;East_Han_AF=
chr4	29479085	.	T	<INS:ME:LINE1>	.	PASS	SVTYPE=INS:ME:LINE1;END=29479096;SVLEN=272;AN=20618;AC=5;AF=0.00024250654767678728;N_HET=5;N_HOMALT=0;N_HOMREF=10304;FREQ_HET=0.0004850130953535455;FREQ_HOMALT=0.0;FREQ_HOMREF=0.9995149869046465;N_BI_GENOS=10309;East_Han_AN=6554;East_Han_AC=2;East_Han_AF
chr4	29569818	.	A	<INS:ME:LINE1>	.	PASS	SVTYPE=INS:ME:LINE1;END=29569834;SVLEN=1567;AN=20618;AC=2;AF=9.700261907071492e-05;N_HET=2;N_HOMALT=0;N_HOMREF=10307;FREQ_HET=0.0001940052381442983;FREQ_HOMALT=0.0;FREQ_HOMREF=0.9998059947618586;N_BI_GENOS=10309;North_Han_AN=2272;North_Han_AC=1;North_Han
chr4	29952079	.	A	<INS:ME:LINE1>	.	PASS	SVTYPE=INS:ME:LINE1;END=29952097;SVLEN=1666;AN=20618;AC=4;AF=0.0001940052381442983;N_HET=4;N_HOMALT=0;N_HOMREF=10305;FREQ_HET=0.0003880104762885966;FREQ_HOMALT=0.0;FREQ_HOMREF=0.9996119895237171;N_BI_GENOS=10309;East_Han_AN=6554;East_Han_AC=2;East_Han_A
chr4	30281782	.	A	<INS:ME:LINE1>	.	PASS	SVTYPE=INS:ME:LINE1;END=30281788;SVLEN=450;AN=20618;AC=1;AF=4.850130953535746e-05;N_HET=1;N_HOMALT=0;N_HOMREF=10308;FREQ_HET=9.700261907071492e-05;FREQ_HOMALT=0.0;FREQ_HOMREF=0.9999029973809292;N_BI_GENOS=10309;Northwest_Han_AN=1296;Northwest_Han_AC=1;Nor
chr4	30282114	.	A	<INS:ME:LINE1>	.	PASS	SVTYPE=INS:ME:LINE1;END=30282130;SVLEN=2656;AN=20618;AC=1;AF=4.850130953535746e-05;N_HET=1;N_HOMALT=0;N_HOMREF=10308;FREQ_HET=9.700261907071492e-05;FREQ_HOMALT=0.0;FREQ_HOMREF=0.9999029973809292;N_BI_GENOS=10309;Miao_AN=482;Miao_AC=1;Miao_AF=0.00207468879
chr4	30626319	.	A	<INS:ME:LINE1>	.	PASS	SVTYPE=INS:ME:LINE1;END=30626331;SVLEN=784;AN=20618;AC=1;AF=4.850130953535746e-05;N_HET=1;N_HOMALT=0;N_HOMREF=10308;FREQ_HET=9.700261907071492e-05;FREQ_HOMALT=0.0;FREQ_HOMREF=0.9999029973809292;N_BI_GENOS=10309;East_Han_AN=6554;East_Han_AC=1;East_Han_AF=0
chr4	30655871	.	A	<INS:ME:LINE1>	.	PASS	SVTYPE=INS:ME:LINE1;END=30655888;SVLEN=2160;AN=20618;AC=1;AF=4.850130953535746e-05;N_HET=1;N_HOMALT=0;N_HOMREF=10308;FREQ_HET=9.700261907071492e-05;FREQ_HOMALT=0.0;FREQ_HOMREF=0.9999029973809292;N_BI_GENOS=10309;Lingnan_Han_AN=1888;Lingnan_Han_AC=1;Lingna
chr4	30805081	.	T	<INS:ME:LINE1>	.	PASS	SVTYPE=INS:ME:LINE1;END=30805086;SVLEN=3165;AN=20618;AC=1;AF=4.850130953535746e-05;N_HET=1;N_HOMALT=0;N_HOMREF=10308;FREQ_HET=9.700261907071492e-05;FREQ_HOMALT=0.0;FREQ_HOMREF=0.9999029973809292;N_BI_GENOS=10309;East_Han_AN=6554;East_Han_AC=1;East_Han_AF=
chr4	30959243	.	G	<INS:ME:LINE1>	.	PASS	SVTYPE=INS:ME:LINE1;END=30959259;SVLEN=1139;AN=20618;AC=1;AF=4.850130953535746e-05;N_HET=1;N_HOMALT=0;N_HOMREF=10308;FREQ_HET=9.700261907071492e-05;FREQ_HOMALT=0.0;FREQ_HOMREF=0.9999029973809292;N_BI_GENOS=10309;Lingnan_Han_AN=1888;Lingnan_Han_AC=1;Lingna
chr4	30986389	.	C	<INS:ME:LINE1>	.	PASS	SVTYPE=INS:ME:LINE1;END=30986396;SVLEN=1403;AN=20618;AC=4;AF=0.0001940052381442983;N_HET=4;N_HOMALT=0;N_HOMREF=10305;FREQ_HET=0.0003880104762885966;FREQ_HOMALT=0.0;FREQ_HOMREF=0.9996119895237171;N_BI_GENOS=10309;Lingnan_Han_AN=1888;Lingnan_Han_AC=2;Ling
chr4	31282091	.	A	<INS:ME:LINE1>	.	PASS	SVTYPE=INS:ME:LINE1;END=31282091;SVLEN=822;AN=20618;AC=2;AF=9.700261907071492e-05;N_HET=0;N_HOMALT=1;N_HOMREF=10308;FREQ_HET=0.0;FREQ_HOMALT=9.700261907071492e-05;FREQ_HOMREF=0.9999029973809292;N_BI_GENOS=10309;East_Han_AN=6554;East_Han_AC=2;East_Han_AF=0
chr4	31411041	.	G	<INS:ME:LINE1>	.	PASS	SVTYPE=INS:ME:LINE1;END=31411051;SVLEN=1245;AN=20618;AC=1;AF=4.850130953535746e-05;N_HET=1;N_HOMALT=0;N_HOMREF=10308;FREQ_HET=9.700261907071492e-05;FREQ_HOMALT=0.0;FREQ_HOMREF=0.9999029973809292;N_BI_GENOS=10309;North_Han_AN=2272;North_Han_AC=1;North_Han_
chr4	31536055	.	G	<INS:ME:LINE1>	.	PASS	SVTYPE=INS:ME:LINE1;END=31536071;SVLEN=2576;AN=20618;AC=2;AF=9.700261907071492e-05;N_HET=2;N_HOMALT=0;N_HOMREF=10307;FREQ_HET=0.0001940052381442983;FREQ_HOMALT=0.0;FREQ_HOMREF=0.9998059947618586;N_BI_GENOS=10309;South_Han_AN=1496;South_Han_AC=1;South_Han
chr4	31661085	.	A	<INS:ME:LINE1>	.	PASS	SVTYPE=INS:ME:LINE1;END=31661089;SVLEN=304;AN=20618;AC=1;AF=4.850130953535746e-05;N_HET=1;N_HOMALT=0;N_HOMREF=10308;FREQ_HET=9.700261907071492e-05;FREQ_HOMALT=0.0;FREQ_HOMREF=0.9999029973809292;N_BI_GENOS=10309;Northwest_Han_AN=1296;Northwest_Han_AC=1;Nor
chr4	31734737	.	A	<INS:ME:LINE1>	.	PASS	SVTYPE=INS:ME:LINE1;END=31734753;SVLEN=1140;AN=20618;AC=1;AF=4.850130953535746e-05;N_HET=1;N_HOMALT=0;N_HOMREF=10308;FREQ_HET=9.700261907071492e-05;FREQ_HOMALT=0.0;FREQ_HOMREF=0.9999029973809292;N_BI_GENOS=10309;East_Han_AN=6554;East_Han_AC=1;East_Han_AF=
chr4	31919462	.	G	<INS:ME:LINE1>	.	PASS	SVTYPE=INS:ME:LINE1;END=31919479;SVLEN=929;AN=20618;AC=1;AF=4.850130953535746e-05;N_HET=1;N_HOMALT=0;N_HOMREF=10308;FREQ_HET=9.700261907071492e-05;FREQ_HOMALT=0.0;FREQ_HOMREF=0.9999029973809292;N_BI_GENOS=10309;North_Han_AN=2272;North_Han_AC=1;North_Han_A
chr4	32180008	.	G	<INS:ME:LINE1>	.	PASS	SVTYPE=INS:ME:LINE1;END=32180024;SVLEN=1963;AN=20618;AC=2;AF=9.700261907071492e-05;N_HET=2;N_HOMALT=0;N_HOMREF=10307;FREQ_HET=0.0001940052381442983;FREQ_HOMALT=0.0;FREQ_HOMREF=0.9998059947618586;N_BI_GENOS=10309;Lingnan_Han_AN=1888;Lingnan_Han_AC=1;Lingn
chr4	32758228	.	A	<INS:ME:LINE1>	.	PASS	SVTYPE=INS:ME:LINE1;END=32758228;SVLEN=690;AN=20618;AC=1;AF=4.850130953535746e-05;N_HET=1;N_HOMALT=0;N_HOMREF=10308;FREQ_HET=9.700261907071492e-05;FREQ_HOMALT=0.0;FREQ_HOMREF=0.9999029973809292;N_BI_GENOS=10309;East_Han_AN=6554;East_Han_AC=1;East_Han_AF=0
chr4	32889766	.	A	<INS:ME:LINE1>	.	PASS	SVTYPE=INS:ME:LINE1;END=32889766;SVLEN=92;AN=20618;AC=14;AF=0.0006790183334950044;N_HET=14;N_HOMALT=0;N_HOMREF=10295;FREQ_HET=0.0013580366669900088;FREQ_HOMALT=0.0;FREQ_HOMREF=0.99864196333301;N_BI_GENOS=10309;North_Han_AN=2272;North_Han_AC=3;North_Han_AF
chr4	33112795	.	A	<INS:ME:LINE1>	.	PASS	SVTYPE=INS:ME:LINE1;END=33112824;SVLEN=1337;AN=20618;AC=1;AF=4.850130953535746e-05;N_HET=1;N_HOMALT=0;N_HOMREF=10308;FREQ_HET=9.700261907071492e-05;FREQ_HOMALT=0.0;FREQ_HOMREF=0.9999029973809292;N_BI_GENOS=10309;East_Han_AN=6554;East_Han_AC=1;East_Han_AF=
chr4	33172957	.	A	<INS:ME:LINE1>	.	PASS	SVTYPE=INS:ME:LINE1;END=33172973;SVLEN=505;AN=20618;AC=2;AF=9.700261907071492e-05;N_HET=2;N_HOMALT=0;N_HOMREF=10307;FREQ_HET=0.0001940052381442983;FREQ_HOMALT=0.0;FREQ_HOMREF=0.9998059947618586;N_BI_GENOS=10309;East_Han_AN=6554;East_Han_AC=2;East_Han_AF=
chr4	33180225	.	A	<INS:ME:LINE1>	.	PASS	SVTYPE=INS:ME:LINE1;END=33180265;SVLEN=786;AN=20618;AC=1;AF=4.850130953535746e-05;N_HET=1;N_HOMALT=0;N_HOMREF=10308;FREQ_HET=9.700261907071492e-05;FREQ_HOMALT=0.0;FREQ_HOMREF=0.9999029973809292;N_BI_GENOS=10309;East_Han_AN=6554;East_Han_AC=1;East_Han_AF=0
chr4	33224431	.	A	<INS:ME:LINE1>	.	PASS	SVTYPE=INS:ME:LINE1;END=33224446;SVLEN=749;AN=20618;AC=3;AF=0.00014550392860607236;N_HET=3;N_HOMALT=0;N_HOMREF=10306;FREQ_HET=0.0002910078572121447;FREQ_HOMALT=0.0;FREQ_HOMREF=0.9997089921427879;N_BI_GENOS=10309;Lingnan_Han_AN=1888;Lingnan_Han_AC=1;Lingna
chr4	33254640	.	T	<INS:ME:LINE1>	.	PASS	SVTYPE=INS:ME:LINE1;END=33254654;SVLEN=950;AN=20618;AC=9;AF=0.0004365117858182171;N_HET=9;N_HOMALT=0;N_HOMREF=10300;FREQ_HET=0.0008730235716364342;FREQ_HOMALT=0.0;FREQ_HOMREF=0.9991269764283636;N_BI_GENOS=10309;South_Han_AN=1496;South_Han_AC=1;South_Han_A
chr4	33300866	.	T	<INS:ME:LINE1>	.	PASS	SVTYPE=INS:ME:LINE1;END=33300870;SVLEN=1342;AN=20618;AC=5;AF=0.00024250654767678728;N_HET=1;N_HOMALT=2;N_HOMREF=10306;FREQ_HET=9.700261907071492e-05;FREQ_HOMALT=0.0001940052381442983;FREQ_HOMREF=0.9997089921427879;N_BI_GENOS=10309;North_Han_AN=2272;North
chr4	33469668	.	T	<INS:ME:LINE1>	.	PASS	SVTYPE=INS:ME:LINE1;END=33469684;SVLEN=6001;AN=20618;AC=2;AF=9.700261907071492e-05;N_HET=2;N_HOMALT=0;N_HOMREF=10307;FREQ_HET=0.0001940052381442983;FREQ_HOMALT=0.0;FREQ_HOMREF=0.9998059947618586;N_BI_GENOS=10309;South_Han_AN=1496;South_Han_AC=1;South_Han
chr4	33622420	.	A	<INS:ME:LINE1>	.	PASS	SVTYPE=INS:ME:LINE1;END=33622436;SVLEN=2972;AN=20618;AC=2;AF=9.700261907071492e-05;N_HET=2;N_HOMALT=0;N_HOMREF=10307;FREQ_HET=0.0001940052381442983;FREQ_HOMALT=0.0;FREQ_HOMREF=0.9998059947618586;N_BI_GENOS=10309;Central_Han_AN=2128;Central_Han_AC=2;Centr
chr4	34036370	.	T	<INS:ME:LINE1>	.	PASS	SVTYPE=INS:ME:LINE1;END=34036384;SVLEN=6042;AN=20618;AC=1;AF=4.850130953535746e-05;N_HET=1;N_HOMALT=0;N_HOMREF=10308;FREQ_HET=9.700261907071492e-05;FREQ_HOMALT=0.0;FREQ_HOMREF=0.9999029973809292;N_BI_GENOS=10309;North_Han_AN=2272;North_Han_AC=1;North_Han_
chr4	34121428	.	A	<INS:ME:LINE1>	.	PASS	SVTYPE=INS:ME:LINE1;END=34121444;SVLEN=882;AN=20618;AC=1;AF=4.850130953535746e-05;N_HET=1;N_HOMALT=0;N_HOMREF=10308;FREQ_HET=9.700261907071492e-05;FREQ_HOMALT=0.0;FREQ_HOMREF=0.9999029973809292;N_BI_GENOS=10309;Hui_AN=444;Hui_AC=1;Hui_AF=0.002252252252252
chr4	34175649	.	C	<INS:ME:LINE1>	.	PASS	SVTYPE=INS:ME:LINE1;END=34175672;SVLEN=1564;AN=20618;AC=1;AF=4.850130953535746e-05;N_HET=1;N_HOMALT=0;N_HOMREF=10308;FREQ_HET=9.700261907071492e-05;FREQ_HOMALT=0.0;FREQ_HOMREF=0.9999029973809292;N_BI_GENOS=10309;East_Han_AN=6554;East_Han_AC=1;East_Han_AF=
chr4	34176725	.	A	<INS:ME:LINE1>	.	PASS	SVTYPE=INS:ME:LINE1;END=34176725;SVLEN=221;AN=20618;AC=1;AF=4.850130953535746e-05;N_HET=1;N_HOMALT=0;N_HOMREF=10308;FREQ_HET=9.700261907071492e-05;FREQ_HOMALT=0.0;FREQ_HOMREF=0.9999029973809292;N_BI_GENOS=10309;East_Han_AN=6554;East_Han_AC=1;East_Han_AF=0
chr4	34241712	.	A	<INS:ME:LINE1>	.	PASS	SVTYPE=INS:ME:LINE1;END=34241727;SVLEN=2046;AN=20618;AC=1;AF=4.850130953535746e-05;N_HET=1;N_HOMALT=0;N_HOMREF=10308;FREQ_HET=9.700261907071492e-05;FREQ_HOMALT=0.0;FREQ_HOMREF=0.9999029973809292;N_BI_GENOS=10309;Zhuang_AN=444;Zhuang_AC=1;Zhuang_AF=0.00225
chr4	34380909	.	G	<INS:ME:LINE1>	.	PASS	SVTYPE=INS:ME:LINE1;END=34380909;SVLEN=1044;AN=20618;AC=1;AF=4.850130953535746e-05;N_HET=1;N_HOMALT=0;N_HOMREF=10308;FREQ_HET=9.700261907071492e-05;FREQ_HOMALT=0.0;FREQ_HOMREF=0.9999029973809292;N_BI_GENOS=10309;Southeast_Han_AN=1908;Southeast_Han_AC=1;So
chr4	35156916	.	A	<INS:ME:LINE1>	.	PASS	SVTYPE=INS:ME:LINE1;END=35156934;SVLEN=2677;AN=20618;AC=1;AF=4.850130953535746e-05;N_HET=1;N_HOMALT=0;N_HOMREF=10308;FREQ_HET=9.700261907071492e-05;FREQ_HOMALT=0.0;FREQ_HOMREF=0.9999029973809292;N_BI_GENOS=10309;North_Han_AN=2272;North_Han_AC=1;North_Han_
chr4	35272224	.	T	<INS:ME:LINE1>	.	PASS	SVTYPE=INS:ME:LINE1;END=35272241;SVLEN=1193;AN=20618;AC=1;AF=4.850130953535746e-05;N_HET=1;N_HOMALT=0;N_HOMREF=10308;FREQ_HET=9.700261907071492e-05;FREQ_HOMALT=0.0;FREQ_HOMREF=0.9999029973809292;N_BI_GENOS=10309;North_Han_AN=2272;North_Han_AC=1;North_Han_
chr4	35331105	.	A	<INS:ME:LINE1>	.	PASS	SVTYPE=INS:ME:LINE1;END=35331119;SVLEN=600;AN=20618;AC=1;AF=4.850130953535746e-05;N_HET=1;N_HOMALT=0;N_HOMREF=10308;FREQ_HET=9.700261907071492e-05;FREQ_HOMALT=0.0;FREQ_HOMREF=0.9999029973809292;N_BI_GENOS=10309;Mongolian_AN=454;Mongolian_AC=1;Mongolian_AF
chr4	35459137	.	T	<INS:ME:LINE1>	.	PASS	SVTYPE=INS:ME:LINE1;END=35459151;SVLEN=557;AN=20618;AC=2;AF=9.700261907071492e-05;N_HET=2;N_HOMALT=0;N_HOMREF=10307;FREQ_HET=0.0001940052381442983;FREQ_HOMALT=0.0;FREQ_HOMREF=0.9998059947618586;N_BI_GENOS=10309;South_Han_AN=1496;South_Han_AC=1;South_Han_
chr4	35654660	.	T	<INS:ME:LINE1>	.	PASS	SVTYPE=INS:ME:LINE1;END=35654673;SVLEN=6062;AN=20618;AC=1;AF=4.850130953535746e-05;N_HET=1;N_HOMALT=0;N_HOMREF=10308;FREQ_HET=9.700261907071492e-05;FREQ_HOMALT=0.0;FREQ_HOMREF=0.9999029973809292;N_BI_GENOS=10309;East_Han_AN=6554;East_Han_AC=1;East_Han_AF=
chr4	35669772	.	T	<INS:ME:LINE1>	.	PASS	SVTYPE=INS:ME:LINE1;END=35669772;SVLEN=1701;AN=20618;AC=1;AF=4.850130953535746e-05;N_HET=1;N_HOMALT=0;N_HOMREF=10308;FREQ_HET=9.700261907071492e-05;FREQ_HOMALT=0.0;FREQ_HOMREF=0.9999029973809292;N_BI_GENOS=10309;Lingnan_Han_AN=1888;Lingnan_Han_AC=1;Lingna
chr4	35706262	.	G	<INS:ME:LINE1>	.	PASS	SVTYPE=INS:ME:LINE1;END=35706262;SVLEN=115;AN=20618;AC=1;AF=4.850130953535746e-05;N_HET=1;N_HOMALT=0;N_HOMREF=10308;FREQ_HET=9.700261907071492e-05;FREQ_HOMALT=0.0;FREQ_HOMREF=0.9999029973809292;N_BI_GENOS=10309;East_Han_AN=6554;East_Han_AC=1;East_Han_AF=0
chr4	35775682	.	A	<INS:ME:LINE1>	.	PASS	SVTYPE=INS:ME:LINE1;END=35775699;SVLEN=3135;AN=20618;AC=11;AF=0.0005335144048888932;N_HET=11;N_HOMALT=0;N_HOMREF=10298;FREQ_HET=0.0010670288097777864;FREQ_HOMALT=0.0;FREQ_HOMREF=0.9989329711902222;N_BI_GENOS=10309;South_Han_AN=1496;South_Han_AC=1;South_Han_
chr4	35846031	.	T	<INS:ME:LINE1>	.	PASS	SVTYPE=INS:ME:LINE1;END=35846031;SVLEN=680;AN=20618;AC=1;AF=4.850130953535746e-05;N_HET=1;N_HOMALT=0;N_HOMREF=10308;FREQ_HET=9.700261907071492e-05;FREQ_HOMALT=0.0;FREQ_HOMREF=0.9999029973809292;N_BI_GENOS=10309;East_Han_AN=6554;East_Han_AC=1;East_Han_AF=0
chr4	35847612	.	T	<INS:ME:LINE1>	.	PASS	SVTYPE=INS:ME:LINE1;END=35847614;SVLEN=460;AN=20618;AC=1;AF=4.850130953535746e-05;N_HET=1;N_HOMALT=0;N_HOMREF=10308;FREQ_HET=9.700261907071492e-05;FREQ_HOMALT=0.0;FREQ_HOMREF=0.9999029973809292;N_BI_GENOS=10309;Southeast_Han_AN=1908;Southeast_Han_AC=1;Sou
chr4	35935608	.	A	<INS:ME:LINE1>	.	PASS	SVTYPE=INS:ME:LINE1;END=35935625;SVLEN=2764;AN=20618;AC=3;AF=0.00014550392860607236;N_HET=3;N_HOMALT=0;N_HOMREF=10306;FREQ_HET=0.0002910078572121447;FREQ_HOMALT=0.0;FREQ_HOMREF=0.9997089921427879;N_BI_GENOS=10309;Northwest_Han_AN=1296;Northwest_Han_AC=1;N
chr4	36067264	.	T	<INS:ME:LINE1>	.	PASS	SVTYPE=INS:ME:LINE1;END=36067277;SVLEN=612;AN=20618;AC=1;AF=4.850130953535746e-05;N_HET=1;N_HOMALT=0;N_HOMREF=10308;FREQ_HET=9.700261907071492e-05;FREQ_HOMALT=0.0;FREQ_HOMREF=0.9999029973809292;N_BI_GENOS=10309;Zhuang_AN=444;Zhuang_AC=1;Zhuang_AF=0.002252
chr4	36280144	.	G	<INS:ME:LINE1>	.	PASS	SVTYPE=INS:ME:LINE1;END=36280154;SVLEN=1258;AN=20618;AC=1;AF=4.850130953535746e-05;N_HET=1;N_HOMALT=0;N_HOMREF=10308;FREQ_HET=9.700261907071492e-05;FREQ_HOMALT=0.0;FREQ_HOMREF=0.9999029973809292;N_BI_GENOS=10309;East_Han_AN=6554;East_Han_AC=1;East_Han_AF=
chr4	36424280	.	A	<INS:ME:LINE1>	.	PASS	SVTYPE=INS:ME:LINE1;END=36424290;SVLEN=1926;AN=20618;AC=3;AF=0.00014550392860607236;N_HET=3;N_HOMALT=0;N_HOMREF=10306;FREQ_HET=0.0002910078572121447;FREQ_HOMALT=0.0;FREQ_HOMREF=0.9997089921427879;N_BI_GENOS=10309;Lingnan_Han_AN=1888;Lingnan_Han_AC=2;Lingn
chr4	36501634	.	T	<INS:ME:LINE1>	.	PASS	SVTYPE=INS:ME:LINE1;END=36501650;SVLEN=5999;AN=20618;AC=1;AF=4.850130953535746e-05;N_HET=1;N_HOMALT=0;N_HOMREF=10308;FREQ_HET=9.700261907071492e-05;FREQ_HOMALT=0.0;FREQ_HOMREF=0.9999029973809292;N_BI_GENOS=10309;Southeast_Han_AN=1908;Southeast_Han_AC=1;So
chr4	36701054	.	T	<INS:ME:LINE1>	.	PASS	SVTYPE=INS:ME:LINE1;END=36701063;SVLEN=3574;AN=20618;AC=1;AF=4.850130953535746e-05;N_HET=1;N_HOMALT=0;N_HOMREF=10308;FREQ_HET=9.700261907071492e-05;FREQ_HOMALT=0.0;FREQ_HOMREF=0.9999029973809292;N_BI_GENOS=10309;South_Han_AN=1496;South_Han_AC=1;South_Han_
chr4	36706379	.	A	<INS:ME:LINE1>	.	PASS	SVTYPE=INS:ME:LINE1;END=36706394;SVLEN=1067;AN=20618;AC=1;AF=4.850130953535746e-05;N_HET=1;N_HOMALT=0;N_HOMREF=10308;FREQ_HET=9.700261907071492e-05;FREQ_HOMALT=0.0;FREQ_HOMREF=0.9999029973809292;N_BI_GENOS=10309;East_Han_AN=6554;East_Han_AC=1;East_Han_AF=
chr4	36828191	.	A	<INS:ME:LINE1>	.	PASS	SVTYPE=INS:ME:LINE1;END=36828209;SVLEN=1015;AN=20618;AC=1;AF=4.850130953535746e-05;N_HET=1;N_HOMALT=0;N_HOMREF=10308;FREQ_HET=9.700261907071492e-05;FREQ_HOMALT=0.0;FREQ_HOMREF=0.9999029973809292;N_BI_GENOS=10309;Northwest_Han_AN=1296;Northwest_Han_AC=1;No
chr4	36854227	.	A	<INS:ME:LINE1>	.	PASS	SVTYPE=INS:ME:LINE1;END=36854234;SVLEN=1361;AN=20618;AC=1;AF=4.850130953535746e-05;N_HET=1;N_HOMALT=0;N_HOMREF=10308;FREQ_HET=9.700261907071492e-05;FREQ_HOMALT=0.0;FREQ_HOMREF=0.9999029973809292;N_BI_GENOS=10309;East_Han_AN=6554;East_Han_AC=1;East_Han_AF=
chr4	37215598	.	A	<INS:ME:LINE1>	.	PASS	SVTYPE=INS:ME:LINE1;END=37215611;SVLEN=5998;AN=20618;AC=1;AF=4.850130953535746e-05;N_HET=1;N_HOMALT=0;N_HOMREF=10308;FREQ_HET=9.700261907071492e-05;FREQ_HOMALT=0.0;FREQ_HOMREF=0.9999029973809292;N_BI_GENOS=10309;East_Han_AN=6554;East_Han_AC=1;East_Han_AF=
chr4	37335350	.	T	<INS:ME:LINE1>	.	PASS	SVTYPE=INS:ME:LINE1;END=37335355;SVLEN=6154;AN=20618;AC=1;AF=4.850130953535746e-05;N_HET=1;N_HOMALT=0;N_HOMREF=10308;FREQ_HET=9.700261907071492e-05;FREQ_HOMALT=0.0;FREQ_HOMREF=0.9999029973809292;N_BI_GENOS=10309;East_Han_AN=6554;East_Han_AC=1;East_Han_AF=
chr4	37434693	.	A	<INS:ME:LINE1>	.	PASS	SVTYPE=INS:ME:LINE1;END=37434709;SVLEN=1902;AN=20618;AC=1;AF=4.850130953535746e-05;N_HET=1;N_HOMALT=0;N_HOMREF=10308;FREQ_HET=9.700261907071492e-05;FREQ_HOMALT=0.0;FREQ_HOMREF=0.9999029973809292;N_BI_GENOS=10309;Southeast_Han_AN=1908;Southeast_Han_AC=1;So
chr4	37574985	.	T	<INS:ME:LINE1>	.	PASS	SVTYPE=INS:ME:LINE1;END=37574998;SVLEN=2636;AN=20618;AC=1;AF=4.850130953535746e-05;N_HET=1;N_HOMALT=0;N_HOMREF=10308;FREQ_HET=9.700261907071492e-05;FREQ_HOMALT=0.0;FREQ_HOMREF=0.9999029973809292;N_BI_GENOS=10309;North_Han_AN=2272;North_Han_AC=1;North_Han_
chr4	37763212	.	A	<INS:ME:LINE1>	.	PASS	SVTYPE=INS:ME:LINE1;END=37763229;SVLEN=5999;AN=20618;AC=3;AF=0.00014550392860607236;N_HET=3;N_HOMALT=0;N_HOMREF=10306;FREQ_HET=0.0002910078572121447;FREQ_HOMALT=0.0;FREQ_HOMREF=0.9997089921427879;N_BI_GENOS=10309;Northwest_Han_AN=1296;Northwest_Han_AC=1;N

chr4	37950491 .	T	<INS:ME:LINE1>	.	PASS	SVTYPE=INS:ME:LINE1;END=37950492;SVLEN=4116;AN=20618;AC=2796;AF=0.13560966146085945;N_HET=2044;N_HOMALT=376;N_HOMREF=7889;FREQ_HET=0.19827335338054128;FREQ_HOMALT=0.03647298477058881;FREQ_HOMREF=0.7652536618488699;N_BI_GENOS=10309;South_Han_AN=1496;South_
chr4	38412684 .	G	<INS:ME:LINE1>	.	PASS	SVTYPE=INS:ME:LINE1;END=38412700;SVLEN=5998;AN=20618;AC=5;AF=0.00024250654767678728;N_HET=5;N_HOMALT=0;N_HOMREF=10304;FREQ_HET=0.0004850130953535457455;FREQ_HOMALT=0.0;FREQ_HOMREF=0.9995149869046465;N_BI_GENOS=10309;North_Han_AN=2272;North_Han_AC=1;North_Ha
chr4	38505712 .	A	<INS:ME:LINE1>	.	PASS	SVTYPE=INS:ME:LINE1;END=38505728;SVLEN=5998;AN=20618;AC=12;AF=0.0005820157144242894;N_HET=10;N_HOMALT=1;N_HOMREF=10298;FREQ_HET=0.0009700261907071491;FREQ_HOMALT=9.700261907071492e-05;FREQ_HOMREF=0.9989329711902222;N_BI_GENOS=10309;South_Han_AN=1496;South
chr4	38521315 .	T	<INS:ME:LINE1>	.	PASS	SVTYPE=INS:ME:LINE1;END=38521315;SVLEN=2878;AN=20618;AC=2;AF=9.700261907071492e-05;N_HET=2;N_HOMALT=0;N_HOMREF=10307;FREQ_HET=0.00019400523814142983;FREQ_HOMALT=0.0;FREQ_HOMREF=0.9998059947618586;N_BI_GENOS=10309;Lingnan_Han_AN=1888;Lingnan_Han_AC=1;Lingn
chr4	39211589 .	A	<INS:ME:LINE1>	.	PASS	SVTYPE=INS:ME:LINE1;END=39211591;SVLEN=3562;AN=20618;AC=1;AF=4.850130953535746e-05;N_HET=1;N_HOMALT=0;N_HOMREF=10308;FREQ_HET=9.700261907071492e-05;FREQ_HOMALT=0.0;FREQ_HOMREF=0.9999029973809292;N_BI_GENOS=10309;Central_Han_AN=2128;Central_Han_AC=1;Centra
chr4	40978730 .	G	<INS:ME:LINE1>	.	PASS	SVTYPE=INS:ME:LINE1;END=40978748;SVLEN=5994;AN=20618;AC=3;AF=0.00014550392860607236;N_HET=3;N_HOMALT=0;N_HOMREF=10306;FREQ_HET=0.0002910078572121447;FREQ_HOMALT=0.0;FREQ_HOMREF=0.9997089921427879;N_BI_GENOS=10309;South_Han_AN=1496;South_Han_AC=1;South_Han
chr4	41156728 .	A	<INS:ME:LINE1>	.	PASS	SVTYPE=INS:ME:LINE1;END=41156743;SVLEN=5997;AN=20618;AC=27;AF=0.0013095353574546513;N_HET=27;N_HOMALT=0;N_HOMREF=10282;FREQ_HET=0.0026190707149093026;FREQ_HOMALT=0.0;FREQ_HOMREF=0.9973809292850907;N_BI_GENOS=10309;South_Han_AN=1496;South_Han_AC=2;South_Ha
chr4	41317136 .	T	<INS:ME:LINE1>	.	PASS	SVTYPE=INS:ME:LINE1;END=41317151;SVLEN=5998;AN=20618;AC=27;AF=0.0013095353574546513;N_HET=27;N_HOMALT=0;N_HOMREF=10282;FREQ_HET=0.0026190707149093026;FREQ_HOMALT=0.0;FREQ_HOMREF=0.9973809292850907;N_BI_GENOS=10309;South_Han_AN=1496;South_Han_AC=2;South_Ha
chr4	41393713 .	A	<INS:ME:LINE1>	.	PASS	SVTYPE=INS:ME:LINE1;END=41393728;SVLEN=6000;AN=20618;AC=1;AF=4.850130953535746e-05;N_HET=1;N_HOMALT=0;N_HOMREF=10308;FREQ_HET=9.700261907071492e-05;FREQ_HOMALT=0.0;FREQ_HOMREF=0.9999029973809292;N_BI_GENOS=10309;Central_Han_AN=2128;Central_Han_AC=1;Centra
chr4	41407918 .	G	<INS:ME:LINE1>	.	PASS	SVTYPE=INS:ME:LINE1;END=41407933;SVLEN=778;AN=20618;AC=1;AF=4.850130953535746e-05;N_HET=1;N_HOMALT=0;N_HOMREF=10308;FREQ_HET=9.700261907071492e-05;FREQ_HOMALT=0.0;FREQ_HOMREF=0.9999029973809292;N_BI_GENOS=10309;Central_Han_AN=2128;Central_Han_AC=1;Central
chr4	41467327 .	A	<INS:ME:LINE1>	.	PASS	SVTYPE=INS:ME:LINE1;END=41467327;SVLEN=2248;AN=20618;AC=1;AF=4.850130953535746e-05;N_HET=1;N_HOMALT=0;N_HOMREF=10308;FREQ_HET=9.700261907071492e-05;FREQ_HOMALT=0.0;FREQ_HOMREF=0.9999029973809292;N_BI_GENOS=10309;Lingnan_Han_AN=1888;Lingnan_Han_AC=1;Lingna
chr4	41523651 .	C	<INS:ME:LINE1>	.	PASS	SVTYPE=INS:ME:LINE1;END=41523668;SVLEN=5998;AN=20618;AC=2;AF=9.700261907071492e-05;N_HET=2;N_HOMALT=0;N_HOMREF=10307;FREQ_HET=0.00019400523814142983;FREQ_HOMALT=0.0;FREQ_HOMREF=0.9998059947618586;N_BI_GENOS=10309;Southeast_Han_AN=1908;Southeast_Han_AC=1;S
chr4	41652946 .	T	<INS:ME:LINE1>	.	PASS	SVTYPE=INS:ME:LINE1;END=41652964;SVLEN=602;AN=20618;AC=3;AF=0.00014550392860607236;N_HET=3;N_HOMALT=0;N_HOMREF=10306;FREQ_HET=0.0002910078572121447;FREQ_HOMALT=0.0;FREQ_HOMREF=0.9997089921427879;N_BI_GENOS=10309;North_Han_AN=2272;North_Han_AC=1;North_Han_
chr4	42095567 .	T	<INS:ME:LINE1>	.	PASS	SVTYPE=INS:ME:LINE1;END=42095585;SVLEN=4683;AN=20618;AC=1;AF=4.850130953535746e-05;N_HET=1;N_HOMALT=0;N_HOMREF=10308;FREQ_HET=9.700261907071492e-05;FREQ_HOMALT=0.0;FREQ_HOMREF=0.9999029973809292;N_BI_GENOS=10309;East_Han_AN=6554;East_Han_AC=1;East_Han_AF=
chr4	42730073 .	A	<INS:ME:LINE1>	.	PASS	SVTYPE=INS:ME:LINE1;END=42730089;SVLEN=5998;AN=20618;AC=43;AF=0.0020855563100203706;N_HET=43;N_HOMALT=0;N_HOMREF=10266;FREQ_HET=0.004171112620040741;FREQ_HOMALT=0.0;FREQ_HOMREF=0.9958288873799592;N_BI_GENOS=10309;South_Han_AN=1496;South_Han_AC=6;South_Han
chr4	42796452 .	T	<INS:ME:LINE1>	.	PASS	SVTYPE=INS:ME:LINE1;END=42796461;SVLEN=6007;AN=20618;AC=3;AF=0.00014550392860607236;N_HET=3;N_HOMALT=0;N_HOMREF=10306;FREQ_HET=0.0002910078572121447;FREQ_HOMALT=0.0;FREQ_HOMREF=0.9997089921427879;N_BI_GENOS=10309;Northwest_Han_AN=1296;Northwest_Han_AC=1;N
chr4	42978139 .	T	<INS:ME:LINE1>	.	PASS	SVTYPE=INS:ME:LINE1;END=42978139;SVLEN=547;AN=20618;AC=1;AF=4.850130953535746e-05;N_HET=1;N_HOMALT=0;N_HOMREF=10308;FREQ_HET=9.700261907071492e-05;FREQ_HOMALT=0.0;FREQ_HOMREF=0.9999029973809292;N_BI_GENOS=10309;East_Han_AN=6554;East_Han_AC=1;East_Han_AF=0
chr4	43028607 .	A	<INS:ME:LINE1>	.	PASS	SVTYPE=INS:ME:LINE1;END=43028624;SVLEN=211;AN=20618;AC=2;AF=9.700261907071492e-05;N_HET=2;N_HOMALT=0;N_HOMREF=10307;FREQ_HET=0.00019400523814142983;FREQ_HOMALT=0.0;FREQ_HOMREF=0.9998059947618586;N_BI_GENOS=10309;East_Han_AN=6554;East_Han_AC=1;East_Han_AF=
chr4	43139514 .	T	<INS:ME:LINE1>	.	PASS	SVTYPE=INS:ME:LINE1;END=43139528;SVLEN=1048;AN=20618;AC=2;AF=9.700261907071492e-05;N_HET=2;N_HOMALT=0;N_HOMREF=10307;FREQ_HET=0.00019400523814142983;FREQ_HOMALT=0.0;FREQ_HOMREF=0.9998059947618586;N_BI_GENOS=10309;Lingnan_Han_AN=1888;Lingnan_Han_AC=2;Lingn
chr4	43155357 .	G	<INS:ME:LINE1>	.	PASS	SVTYPE=INS:ME:LINE1;END=43155357;SVLEN=657;AN=20618;AC=1;AF=4.850130953535746e-05;N_HET=1;N_HOMALT=0;N_HOMREF=10308;FREQ_HET=9.700261907071492e-05;FREQ_HOMALT=0.0;FREQ_HOMREF=0.9999029973809292;N_BI_GENOS=10309;East_Han_AN=6554;East_Han_AC=1;East_Han_AF=0
chr4	43156029 .	G	<INS:ME:LINE1>	.	PASS	SVTYPE=INS:ME:LINE1;END=43156029;SVLEN=744;AN=20618;AC=1;AF=4.850130953535746e-05;N_HET=1;N_HOMALT=0;N_HOMREF=10308;FREQ_HET=9.700261907071492e-05;FREQ_HOMALT=0.0;FREQ_HOMREF=0.9999029973809292;N_BI_GENOS=10309;East_Han_AN=6554;East_Han_AC=1;East_Han_AF=0
chr4	43414829 .	A	<INS:ME:LINE1>	.	PASS	SVTYPE=INS:ME:LINE1;END=43414842;SVLEN=1122;AN=20618;AC=1;AF=4.850130953535746e-05;N_HET=1;N_HOMALT=0;N_HOMREF=10308;FREQ_HET=9.700261907071492e-05;FREQ_HOMALT=0.0;FREQ_HOMREF=0.9999029973809292;N_BI_GENOS=10309;Central_Han_AN=2128;Central_Han_AC=1;Centra
chr4	43500977 .	C	<INS:ME:LINE1>	.	PASS	SVTYPE=INS:ME:LINE1;END=43500988;SVLEN=6000;AN=20618;AC=1;AF=4.850130953535746e-05;N_HET=1;N_HOMALT=0;N_HOMREF=10308;FREQ_HET=9.700261907071492e-05;FREQ_HOMALT=0.0;FREQ_HOMREF=0.9999029973809292;N_BI_GENOS=10309;Central_Han_AN=2128;Central_Han_AC=1;Centra
chr4	43521329 .	G	<INS:ME:LINE1>	.	PASS	SVTYPE=INS:ME:LINE1;END=43521338;SVLEN=1874;AN=20618;AC=1;AF=4.850130953535746e-05;N_HET=1;N_HOMALT=0;N_HOMREF=10308;FREQ_HET=9.700261907071492e-05;FREQ_HOMALT=0.0;FREQ_HOMREF=0.9999029973809292;N_BI_GENOS=10309;East_Han_AN=6554;East_Han_AC=1;East_Han_AF=
chr4	43889916 .	T	<INS:ME:LINE1>	.	PASS	SVTYPE=INS:ME:LINE1;END=43889916;SVLEN=575;AN=20618;AC=3;AF=0.00014550392860607236;N_HET=3;N_HOMALT=0;N_HOMREF=10306;FREQ_HET=0.0002910078572121447;FREQ_HOMALT=0.0;FREQ_HOMREF=0.9997089921427879;N_BI_GENOS=10309;South_Han_AN=1496;South_Han_AC=1;South_Han
chr4	44045722 .	A	<INS:ME:LINE1>	.	PASS	SVTYPE=INS:ME:LINE1;END=44045737;SVLEN=274;AN=20618;AC=1;AF=4.850130953535746e-05;N_HET=1;N_HOMALT=0;N_HOMREF=10308;FREQ_HET=9.700261907071492e-05;FREQ_HOMALT=0.0;FREQ_HOMREF=0.9999029973809292;N_BI_GENOS=10309;Yi_AN=422;Yi_AC=1;Yi_AF=0.002369668246445498
chr4	44179714 .	A	<INS:ME:LINE1>	.	PASS	SVTYPE=INS:ME:LINE1;END=44179737;SVLEN=701;AN=20618;AC=705;AF=0.03419342322242701;N_HET=665;N_HOMALT=20;N_HOMREF=9624;FREQ_HET=0.06450674168202541;FREQ_HOMALT=0.0019400523814142982;FREQ_HOMREF=0.9335532059365603;N_BI_GENOS=10309;South_Han_AN=1496;South_Ha
chr4	44286812 .	A	<INS:ME:LINE1>	.	PASS	SVTYPE=INS:ME:LINE1;END=44286812;SVLEN=6014;AN=20618;AC=1;AF=4.850130953535746e-05;N_HET=1;N_HOMALT=0;N_HOMREF=10308;FREQ_HET=9.700261907071492e-05;FREQ_HOMALT=0.0;FREQ_HOMREF=0.9999029973809292;N_BI_GENOS=10309;East_Han_AN=6554;East_Han_AC=1;East_Han_AF=
chr4	44480940 .	A	<INS:ME:LINE1>	.	PASS	SVTYPE=INS:ME:LINE1;END=44480960;SVLEN=509;AN=20618;AC=3;AF=0.00014550392860607236;N_HET=3;N_HOMALT=0;N_HOMREF=10306;FREQ_HET=0.0002910078572121447;FREQ_HOMALT=0.0;FREQ_HOMREF=0.9997089921427879;N_BI_GENOS=10309;South_Han_AN=1496;South_Han_AC=1;South_
chr4	44588576 .	G	<INS:ME:LINE1>	.	PASS	SVTYPE=INS:ME:LINE1;END=44588577;SVLEN=373;AN=20618;AC=1;AF=4.850130953535746e-05;N_HET=1;N_HOMALT=0;N_HOMREF=10308;FREQ_HET=9.700261907071492e-05;FREQ_HOMALT=0.0;FREQ_HOMREF=0.9999029973809292;N_BI_GENOS=10309;East_Han_AN=6554;East_Han_AC=1;East_Han_AF=0
chr4	44599779 .	A	<INS:ME:LINE1>	.	PASS	SVTYPE=INS:ME:LINE1;END=44599792;SVLEN=1154;AN=20618;AC=1;AF=4.850130953535746e-05;N_HET=1;N_HOMALT=0;N_HOMREF=10308;FREQ_HET=9.700261907071492e-05;FREQ_HOMALT=0.0;FREQ_HOMREF=0.9999029973809292;N_BI_GENOS=10309;Miao_AN=482;Miao_AC=1;Miao_AF=0.00207468879
chr4	44726788 .	G	<INS:ME:LINE1>	.	PASS	SVTYPE=INS:ME:LINE1;END=44726788;SVLEN=695;AN=20618;AC=2;AF=9.700261907071492e-05;N_HET=2;N_HOMALT=0;N_HOMREF=10307;FREQ_HET=0.00019400523814142983;FREQ_HOMALT=0.0;FREQ_HOMREF=0.9998059947618586;N_BI_GENOS=10309;Lingnan_Han_AN=1888;Lingnan_Han_AC=2;Lingna
chr4	44782093 .	G	<INS:ME:LINE1>	.	PASS	SVTYPE=INS:ME:LINE1;END=44782109;SVLEN=183;AN=20618;AC=1;AF=4.850130953535746e-05;N_HET=1;N_HOMALT=0;N_HOMREF=10308;FREQ_HET=9.700261907071492e-05;FREQ_HOMALT=0.0;FREQ_HOMREF=0.9999029973809292;N_BI_GENOS=10309;Northwest_Han_AN=1296;Northwest_Han_AC=1;Nor
chr4	44788192 .	A	<INS:ME:LINE1>	.	PASS	SVTYPE=INS:ME:LINE1;END=44788207;SVLEN=483;AN=20618;AC=2;AF=9.700261907071492e-05;N_HET=2;N_HOMALT=0;N_HOMREF=10307;FREQ_HET=0.00019400523814142983;FREQ_HOMALT=0.0;FREQ_HOMREF=0.9998059947618586;N_BI_GENOS=10309;Lingnan_Han_AN=1888;Lingnan_Han_AC=1;Lingna
chr4	45122766 .	A	<INS:ME:LINE1>	.	PASS	SVTYPE=INS:ME:LINE1;END=45122766;SVLEN=12;AN=20618;AC=1;AF=4.850130953535746e-05;N_HET=1;N_HOMALT=0;N_HOMREF=10308;FREQ_HET=9.700261907071492e-05;FREQ_HOMALT=0.0;FREQ_HOMREF=0.9999029973809292;N_BI_GENOS=10309;Tibetan_AN=414;Tibetan_AC=1;Tibetan_AF=0.0024
chr4	45155377 .	G	<INS:ME:LINE1>	.	PASS	SVTYPE=INS:ME:LINE1;END=45155390;SVLEN=465;AN=20618;AC=1;AF=4.850130953535746e-05;N_HET=1;N_HOMALT=0;N_HOMREF=10308;FREQ_HET=9.700261907071492e-05;FREQ_HOMALT=0.0;FREQ_HOMREF=0.9999029973809292;N_BI_GENOS=10309;East_Han_AN=6554;East_Han_AC=1;East_Han_AF=0
chr4	45230043 .	A	<INS:ME:LINE1>	.	PASS	SVTYPE=INS:ME:LINE1;END=45230059;SVLEN=357;AN=20618;AC=1;AF=4.850130953535746e-05;N_HET=1;N_HOMALT=0;N_HOMREF=10308;FREQ_HET=9.700261907071492e-05;FREQ_HOMALT=0.0;FREQ_HOMREF=0.9999029973809292;N_BI_GENOS=10309;Lingnan_Han_AN=1888;Lingnan_Han_AC=1;Lingnan
chr4	45322789 .	T	<INS:ME:LINE1>	.	PASS	SVTYPE=INS:ME:LINE1;END=45322804;SVLEN=911;AN=20618;AC=1;AF=4.850130953535746e-05;N_HET=1;N_HOMALT=0;N_HOMREF=10308;FREQ_HET=9.700261907071492e-05;FREQ_HOMALT=0.0;FREQ_HOMREF=0.9999029973809292;N_BI_GENOS=10309;East_Han_AN=6554;East_Han_AC=1;East_Han_AF=0
chr4	45707353 .	T	<INS:ME:LINE1>	.	PASS	SVTYPE=INS:ME:LINE1;END=45707353;SVLEN=894;AN=20618;AC=1;AF=4.850130953535746e-05;N_HET=1;N_HOMALT=0;N_HOMREF=10308;FREQ_HET=9.700261907071492e-05;FREQ_HOMALT=0.0;FREQ_HOMREF=0.9999029973809292;N_BI_GENOS=10309;East_Han_AN=6554;East_Han_AC=1;East_Han_AF=0
chr4	45740252 .	T	<INS:ME:LINE1>	.	PASS	SVTYPE=INS:ME:LINE1;END=45740252;SVLEN=901;AN=20618;AC=1;AF=4.850130953535746e-05;N_HET=1;N_HOMALT=0;N_HOMREF=10308;FREQ_HET=9.700261907071492e-05;FREQ_HOMALT=0.0;FREQ_HOMREF=0.9999029973809292;N_BI_GENOS=10309;East_Han_AN=6554;East_Han_AC=1;East_Han_AF=0
chr4	45740945 .	T	<INS:ME:LINE1>	.	PASS	SVTYPE=INS:ME:LINE1;END=45740945;SVLEN=917;AN=20618;AC=1;AF=4.850130953535746e-05;N_HET=1;N_HOMALT=0;N_HOMREF=10308;FREQ_HET=9.700261907071492e-05;FREQ_HOMALT=0.0;FREQ_HOMREF=0.9999029973809292;N_BI_GENOS=10309;East_Han_AN=6554;East_Han_AC=1;East_Han_AF=0
chr4	45741071 .	A	<INS:ME:LINE1>	.	PASS	SVTYPE=INS:ME:LINE1;END=45741071;SVLEN=302;AN=20618;AC=1;AF=4.850130953535746e-05;N_HET=1;N_HOMALT=0;N_HOMREF=10308;FREQ_HET=9.700261907071492e-05;FREQ_HOMALT=0.0;FREQ_HOMREF=0.9999029973809292;N_BI_GENOS=10309;East_Han_AN=6554;East_Han_AC=1;East_Han_AF=0
chr4	45795608 .	C	<INS:ME:LINE1>	.	PASS	SVTYPE=INS:ME:LINE1;END=45795623;SVLEN=2107;AN=20618;AC=1;AF=4.850130953535746e-05;N_HET=1;N_HOMALT=0;N_HOMREF=10308;FREQ_HET=9.700261907071492e-05;FREQ_HOMALT=0.0;FREQ_HOMREF=0.9999029973809292;N_BI_GENOS=10309;East_Han_AN=6554;East_Han_AC=1;East_Han_AF=
chr4	45865600 .	T	<INS:ME:LINE1>	.	PASS	SVTYPE=INS:ME:LINE1;END=45865600;SVLEN=3859;AN=20618;AC=1;AF=4.850130953535746e-05;N_HET=1;N_HOMALT=0;N_HOMREF=10308;FREQ_HET=9.700261907071492e-05;FREQ_HOMALT=0.0;FREQ_HOMREF=0.9999029973809292;N_BI_GENOS=10309;Lingnan_Han_AN=1888;Lingnan_Han_AC=1;Lingna
chr4	45885774 .	A	<INS:ME:LINE1>	.	PASS	SVTYPE=INS:ME:LINE1;END=45885778;SVLEN=204;AN=20618;AC=1;AF=4.850130953535746e-05;N_HET=1;N_HOMALT=0;N_HOMREF=10308;FREQ_HET=9.700261907071492e-05;FREQ_HOMALT=0.0;FREQ_HOMREF=0.9999029973809292;N_BI_GENOS=10309;Lingnan_Han_AN=1888;Lingnan_Han_AC=1;Lingnan
chr4	46028505 .	T	<INS:ME:LINE1>	.	PASS	SVTYPE=INS:ME:LINE1;END=46028505;SVLEN=507;AN=20618;AC=1;AF=4.850130953535746e-05;N_HET=1;N_HOMALT=0;N_HOMREF=10308;FREQ_HET=9.700261907071492e-05;FREQ_HOMALT=0.0;FREQ_HOMREF=0.9999029973809292;N_BI_GENOS=10309;East_Han_AN=6554;East_Han_AC=1;East_Han_AF=0
chr4	46038630 .	G	<INS:ME:LINE1>	.	PASS	SVTYPE=INS:ME:LINE1;END=46038647;SVLEN=422;AN=20618;AC=2;AF=9.700261907071492e-05;N_HET=2;N_HOMALT=0;N_HOMREF=10307;FREQ_HET=0.00019400523814142983;FREQ_HOMALT=0.0;FREQ_HOMREF=0.9998059947618586;N_BI_GENOS=10309;East_Han_AN=6554;East_Han_AC=2;East_Han_AF=
chr4	46191655 .	A	<INS:ME:LINE1>	.	PASS	SVTYPE=INS:ME:LINE1;END=46191655;SVLEN=92;AN=20618;AC=1;AF=4.850130953535746e-05;N_HET=1;N_HOMALT=0;N_HOMREF=10308;FREQ_HET=9.700261907071492e-05;FREQ_HOMALT=0.0;FREQ_HOMREF=0.9999029973809292;N_BI_GENOS=10309;Mongolian_AN=454;Mongolian_AC=1;Mongolian_AF=
chr4	46274943 .	A	<INS:ME:LINE1>	.	PASS	SVTYPE=INS:ME:LINE1;END=46274960;SVLEN=5884;AN=20618;AC=1;AF=4.850130953535746e-05;N_HET=1;N_HOMALT=0;N_HOMREF=10308;FREQ_HET=9.700261907071492e-05;FREQ_HOMALT=0.0;FREQ_HOMREF=0.9999029973809292;N_BI_GENOS=10309;Southeast_Han_AN=1908;Southeast_Han_AC=1;So
chr4	46279427 .	T	<INS:ME:LINE1>	.	PASS	SVTYPE=INS:ME:LINE1;END=46279439;SVLEN=6000;AN=20618;AC=1;AF=4.850130953535746e-05;N_HET=1;N_HOMALT=0;N_HOMREF=10308;FREQ_HET=9.700261907071492e-05;FREQ_HOMALT=0.0;FREQ_HOMREF=0.9999029973809292;N_BI_GENOS=10309;East_Han_AN=6554;East_Han_AC=1;East_Han_AF=
chr4	46325837 .	A	<INS:ME:LINE1>	.	PASS	SVTYPE=INS:ME:LINE1;END=46325860;SVLEN=1360;AN=20618;AC=1;AF=4.850130953535746e-05;N_HET=1;N_HOMALT=0;N_HOMREF=10308;FREQ_HET=9.700261907071492e-05;FREQ_HOMALT=0.0;FREQ_HOMREF=0.9999029973809292;N_BI_GENOS=10309;East_Han_AN=6554;East_Han_AC=1;East_Han_AF=
chr4	46354759 .	A	<INS:ME:LINE1>	.	PASS	SVTYPE=INS:ME:LINE1;END=46354767;SVLEN=295;AN=20618;AC=3;AF=0.00014550392860607236;N_HET=3;N_HOMALT=0;N_HOMREF=10306;FREQ_HET=0.0002910078572121447;FREQ_HOMALT=0.0;FREQ_HOMREF=0.9997089921427879;N_BI_GENOS=10309;East_Han_AN=6554;East_Han_AC=3;East_Han_AF=
chr4	46584662 .	T	<INS:ME:LINE1>	.	PASS	SVTYPE=INS:ME:LINE1;END=46584662;SVLEN=29;AN=20618;AC=10;AF=0.0004850130953557455;N_HET=8;N_HOMALT=1;N_HOMREF=10300;FREQ_HET=0.0007760209525657455;FREQ_HOMALT=9.700261907071492e-05;FREQ_HOMREF=0.9991269764283636;N_BI_GENOS=10309;North_Han_AN=2272;North_H
chr4	46654464 .	A	<INS:ME:LINE1>	.	PASS	SVTYPE=INS:ME:LINE1;END=46654477;SVLEN=316;AN=20618;AC=3;AF=0.00014550392860607236;N_HET=3;N_HOMALT=0;N_HOMREF=10306;FREQ_HET=0.0002910078572121447;FREQ_HOMALT=0.0;FREQ_HOMREF=0.9997089921427879;N_BI_GENOS=10309;Northwest_Han_AN=1296;Northwest_Han_AC=1;No
chr4	46756769 .	G	<INS:ME:LINE1>	.	PASS	SVTYPE=INS:ME:LINE1;END=46756786;SVLEN=788;AN=20618;AC=2;AF=9.700261907071492e-05;N_HET=0;N_HOMALT=1;N_HOMREF=10308;FREQ_HET=0.0;FREQ_HOMALT=9.700261907071492e-05;FREQ_HOMREF=0.9999029973809292;N_BI_GENOS=10309;East_Han_AN=6554;East_Han_AC=2;East_Han_AF=0
chr4	46756904 .	T	<INS:ME:LINE1>	.	PASS	SVTYPE=INS:ME:LINE1;END=46756941;SVLEN=730;AN=20618;AC=1;AF=4.850130953535746e-05;N_HET=1;N_HOMALT=0;N_HOMREF=10308;FREQ_HET=9.700261907071492e-05;FREQ_HOMALT=0.0;FREQ_HOMREF=0.9999029973809292;N_BI_GENOS=10309;East_Han_AN=6554;East_Han_AC=1;East_Han_AF=0
chr4	46921177 .	A	<INS:ME:LINE1>	.	PASS	SVTYPE=INS:ME:LINE1;END=46921194;SVLEN=1501;AN=20618;AC=1;AF=4.850130953535746e-05;N_HET=1;N_HOMALT=0;N_HOMREF=10308;FREQ_HET=9.700261907071492e-05;FREQ_HOMALT=0.0;FREQ_HOMREF=0.9999029973809292;N_BI_GENOS=10309;Hui_AN=444;Hui_AC=1;Hui_AF=0.002252252252225
chr4	46954253 .	T	<INS:ME:LINE1>	.	PASS	SVTYPE=INS:ME:LINE1;END=46954270;SVLEN=171;AN=20618;AC=1;AF=4.850130953535746e-05;N_HET=1;N_HOMALT=0;N_HOMREF=10308;FREQ_HET=9.700261907071492e-05;FREQ_HOMALT=0.0;FREQ_HOMREF=0.9999029973809292;N_BI_GENOS=10309;Northwest_Han_AN=1296;Northwest_Han_AC=1;Nor
chr4	46992703 .	A	<INS:ME:LINE1>	.	PASS	SVTYPE=INS:ME:LINE1;END=46992713;SVLEN=168;AN=20618;AC=1;AF=4.850130953535746e-05;N_HET=1;N_HOMALT=0;N_HOMREF=10308;FREQ_HET=9.700261907071492e-05;FREQ_HOMALT=0.0;FREQ_HOMREF=0.9999029973809292;N_BI_GENOS=10309;Southeast_Han_AN=1908;Southeast_Han_AC=1;Sou
chr4	47005768 .	A	<INS:ME:LINE1>	.	PASS	SVTYPE=INS:ME:LINE1;END=47005772;SVLEN=391;AN=20618;AC=11039;AF=0.535405955960811;N_HET=3659;N_HOMALT=3690;N_HOMREF=2960;FREQ_HET=0.35493258317974585;FREQ_HOMALT=0.357939664370938;FREQ_HOMREF=0.28712775244931615;N_BI_GENOS=10309;South_Han_AN=1496;South_Ha
chr4	47005865 .	T	<INS:ME:LINE1>	.	PASS	SVTYPE=INS:ME:LINE1;END=47005877;SVLEN=2054;AN=20618;AC=1558;AF=0.07556504025608692;N_HET=1230;N_HOMALT=164;N_HOMREF=8915;FREQ_HET=0.11931322145697934;FREQ_HOMALT=0.015908429527597245;FREQ_HOMREF=0.8647783490154234;N_BI_GENOS=10309;South_Han_AN=1496;South
chr4	47093282 .	T	<INS:ME:LINE1>	.	PASS	SVTYPE=INS:ME:LINE1;END=47093298;SVLEN=6000;AN=20618;AC=6;AF=0.0002910078572121447;N_HET=6;N_HOMALT=0;N_HOMREF=10303;FREQ_HET=0.0005820157144242894;FREQ_HOMALT=0.0;FREQ_HOMREF=0.9994179842855757;N_BI_GENOS=10309;South_Han_AN=1496;South_Han_AC=1;South_Han_
chr4	47187129 .	C	<INS:ME:LINE1>	.	PASS	SVTYPE=INS:ME:LINE1;END=47187129;SVLEN=1149;AN=20618;AC=1;AF=4.850130953535746e-05;N_HET=1;N_HOMALT=0;N_HOMREF=10308;FREQ_HET=9.700261907071492e-05;FREQ_HOMALT=0.0;FREQ_HOMREF=0.9999029973809292;N_BI_GENOS=10309;North_Han_AN=2272;North_Han_AC=1;North_Han_
chr4	47429576 .	A	<INS:ME:LINE1>	.	PASS	SVTYPE=INS:ME:LINE1;END=47429588;SVLEN=1431;AN=20618;AC=1;AF=4.850130953535746e-05;N_HET=1;N_HOMALT=0;N_HOMREF=10308;FREQ_HET=9.700261907071492e-05;FREQ_HOMALT=0.0;FREQ_HOMREF=0.9999029973809292;N_BI_GENOS=10309;South_Han_AN=1496;South_Han_AC=1;South_Han_
chr4	47529239 .	C	<INS:ME:LINE1>	.	PASS	SVTYPE=INS:ME:LINE1;END=47529239;SVLEN=237;AN=20618;AC=1;AF=4.850130953535746e-05;N_HET=1;N_HOMALT=0;N_HOMREF=10308;FREQ_HET=9.700261907071492e-05;FREQ_HOMALT=0.0;FREQ_HOMREF=0.9999029973809292;N_BI_GENOS=10309;East_Han_AN=6554;East_Han_AC=1;East_Han_AF=0
chr4	47531682 .	T	<INS:ME:LINE1>	.	PASS	SVTYPE=INS:ME:LINE1;END=47531690;SVLEN=94;AN=20618;AC=4;AF=0.00019400523814142983;N_HET=4;N_HOMALT=0;N_HOMREF=10305;FREQ_HET=0.00038801047628285966;FREQ_HOMALT=0.0;FREQ_HOMREF=0.9996119895237171;N_BI_GENOS=10309;North_Han_AN=2272;North_Han_AC=1;North_Han
chr4	47537805 .	A	<INS:ME:LINE1>	.	PASS	SVTYPE=INS:ME:LINE1;END=47537821;SVLEN=5998;AN=20618;AC=2;AF=9.700261907071492e-05;N_HET=2;N_HOMALT=0;N_HOMREF=10307;FREQ_HET=0.00019400523814142983;FREQ_HOMALT=0.0;FREQ_HOMREF=0.9998059947618586;N_BI_GENOS=10309;North_Han_AN=2272;North_Han_AC=1;North_Han
chr4	47935856 .	T	<INS:ME:LINE1>	.	PASS	SVTYPE=INS:ME:LINE1;END=47935871;SVLEN=1660;AN=20618;AC=1;AF=4.850130953535746e-05;N_HET=1;N_HOMALT=0;N_HOMREF=10308;FREQ_HET=9.700261907071492e-05;FREQ_HOMALT=0.0;FREQ_HOMREF=0.9999029973809292;N_BI_GENOS=10309;North_Han_AN=2272;North_Han_AC=1;North_Han_
chr4	48454949 .	A	<INS:ME:LINE1>	.	PASS	SVTYPE=INS:ME:LINE1;END=48454963;SVLEN=6014;AN=20618;AC=1;AF=4.850130953535746e-05;N_HET=1;N_HOMALT=0;N_HOMREF=10308;FREQ_HET=9.700261907071492e-05;FREQ_HOMALT=0.0;FREQ_HOMREF=0.9999029973809292;N_BI_GENOS=10309;North_Han_AN=2272;North_Han_AC=1;North_Han_
chr4	48574813 .	A	<INS:ME:LINE1>	.	PASS	SVTYPE=INS:ME:LINE1;END=48574813;SVLEN=108;AN=20618;AC=1;AF=4.850130953535746e-05;N_HET=1;N_HOMALT=0;N_HOMREF=10308;FREQ_HET=9.700261907071492e-05;FREQ_HOMALT=0.0;FREQ_HOMREF=0.9999029973809292;N_BI_GENOS=10309;Zhuang_AN=444;Zhuang_AC=1;Zhuang_AF=0.002252
chr4	48840259 .	G	<INS:ME:LINE1>	.	PASS	SVTYPE=INS:ME:LINE1;END=48840272;SVLEN=5998;AN=20618;AC=1;AF=4.850130953535746e-05;N_HET=1;N_HOMALT=0;N_HOMREF=10308;FREQ_HET=9.700261907071492e-05;FREQ_HOMALT=0.0;FREQ_HOMREF=0.9999029973809292;N_BI_GENOS=10309;Southeast_Han_AN=1908;Southeast_Han_AC=1;So
chr4	49044000 .	C	<INS:ME:LINE1>	.	PASS	SVTYPE=INS:ME:LINE1;END=49044016;SVLEN=6001;AN=20618;AC=1;AF=4.850130953535746e-05;N_HET=1;N_HOMALT=0;N_HOMREF=10308;FREQ_HET=9.700261907071492e-05;FREQ_HOMALT=0.0;FREQ_HOMREF=0.9999029973809292;N_BI_GENOS=10309;Northwest_Han_AN=1296;Northwest_Han_AC=1;No
chr4	49080645 .	A	<INS:ME:LINE1>	.	PASS	SVTYPE=INS:ME:LINE1;END=49080651;SVLEN=681;AN=20618;AC=2;AF=9.700261907071492e-05;N_HET=2;N_HOMALT=0;N_HOMREF=10307;FREQ_HET=0.00019400523814142983;FREQ_HOMALT=0.0;FREQ_HOMREF=0.9998059947618586;N_BI_GENOS=10309;North_Han_AN=2272;North_Han_AC=1;North_Han_
chr4	51954807 .	C	<INS:ME:LINE1>	.	PASS	SVTYPE=INS:ME:LINE1;END=51954822;SVLEN=5997;AN=20618;AC=1;AF=4.850130953535746e-05;N_HET=1;N_HOMALT=0;N_HOMREF=10308;FREQ_HET=9.700261907071492e-05;FREQ_HOMALT=0.0;FREQ_HOMREF=0.9999029973809292;N_BI_GENOS=10309;Central_Han_AN=2128;Central_Han_AC=1;Centra
chr4	52002301 .	T	<INS:ME:LINE1>	.	PASS	SVTYPE=INS:ME:LINE1;END=52002301;SVLEN=73;AN=20618;AC=1;AF=4.850130953535746e-05;N_HET=1;N_HOMALT=0;N_HOMREF=10308;FREQ_HET=9.700261907071492e-05;FREQ_HOMALT=0.0;FREQ_HOMREF=0.9999029973809292;N_BI_GENOS=10309;Central_Han_AN=2128;Central_Han_AC=1;Central_
chr4	52148020 .	A	<INS:ME:LINE1>	.	PASS	SVTYPE=INS:ME:LINE1;END=52148037;SVLEN=5980;AN=20618;AC=2;AF=9.700261907071492e-05;N_HET=2;N_HOMALT=0;N_HOMREF=10307;FREQ_HET=0.00019400523814142983;FREQ_HOMALT=0.0;FREQ_HOMREF=0.9998059947618586;N_BI_GENOS=10309;Southeast_Han_AN=1908;Southeast_Han_AC=2;S
chr4	52232759 .	A	<INS:ME:LINE1>	.	PASS	SVTYPE=INS:ME:LINE1;END=52232778;SVLEN=6060;AN=20618;AC=2;AF=9.700261907071492e-05;N_HET=2;N_HOMALT=0;N_HOMREF=10307;FREQ_HET=0.00019400523814142983;FREQ_HOMALT=0.0;FREQ_HOMREF=0.9998059947618586;N_BI_GENOS=10309;North_Han_AN=2272;North_Han_AC=2;North_Han
chr4	52307086 .	T	<INS:ME:LINE1>	.	PASS	SVTYPE=INS:ME:LINE1;END=52307099;SVLEN=5997;AN=20618;AC=1;AF=4.850130953535746e-05;N_HET=1;N_HOMALT=0;N_HOMREF=10308;FREQ_HET=9.700261907071492e-05;FREQ_HOMALT=0.0;FREQ_HOMREF=0.9999029973809292;N_BI_GENOS=10309;Central_Han_AN=2128;Central_Han_AC=1;Centra
chr4	52356257 .	T	<INS:ME:LINE1>	.	PASS	SVTYPE=INS:ME:LINE1;END=52356268;SVLEN=431;AN=20618;AC=4;AF=0.00019400523814142983;N_HET=2;N_HOMALT=1;N_HOMREF=10306;FREQ_HET=0.00019400523814142983;FREQ_HOMALT=9.700261907071492e-05;FREQ_HOMREF=0.9997089921427879;N_BI_GENOS=10309;Northwest_Han_AN=1296;No
chr4	52762309 .	T	<INS:ME:LINE1>	.	PASS	SVTYPE=INS:ME:LINE1;END=52762323;SVLEN=151;AN=20618;AC=2596;AF=0.12590939955378797;N_HET=1788;N_HOMALT=404;N_HOMREF=8117;FREQ_HET=0.17344068289843825;FREQ_HOMALT=0.03918905818104568825;FREQ_HOMREF=0.7873702589969929;N_BI_GENOS=10309;South_Han_AN=1496;South_
chr4	52762564 .	G	<INS:ME:LINE1>	.	PASS	SVTYPE=INS:ME:LINE1;END=52762576;SVLEN=137;AN=20618;AC=2;AF=9.700261907071492e-05;N_HET=2;N_HOMALT=0;N_HOMREF=10307;FREQ_HET=0.00019400523814142983;FREQ_HOMALT=0.0;FREQ_HOMREF=0.9998059947618586;N_BI_GENOS=10309;Central_Han_AN=2128;Central_Han_AC=1;Centra
chr4	52835092 .	T	<INS:ME:LINE1>	.	PASS	SVTYPE=INS:ME:LINE1;END=52835092;SVLEN=0;AN=20618;AC=72;AF=0.0034920942865457366;N_HET=70;N_HOMALT=1;N_HOMREF=10238;FREQ_HET=0.006790183334950043;FREQ_HOMALT=9.700261907071492e-05;FREQ_HOMREF=0.9931128140459793;N_BI_GENOS=10309;South_Han_AN=1496;South_Han
chr4	52889557 .	T	<INS:ME:LINE1>	.	PASS	SVTYPE=INS:ME:LINE1;END=52889573;SVLEN=3600;AN=20618;AC=1;AF=4.850130953535746e-05;N_HET=1;N_HOMALT=0;N_HOMREF=10308;FREQ_HET=9.700261907071492e-05;FREQ_HOMALT=0.0;FREQ_HOMREF=0.9999029973809292;N_BI_GENOS=10309;Miao_AN=482;Miao_AC=1;Miao_AF=0.00207468879
chr4	53125425 .	C	<INS:ME:LINE1>	.	PASS	SVTYPE=INS:ME:LINE1;END=53125442;SVLEN=6018;AN=20618;AC=4;AF=0.00019400523814142983;N_HET=4;N_HOMALT=0;N_HOMREF=10305;FREQ_HET=0.00038801047628285966;FREQ_HOMALT=0.0;FREQ_HOMREF=0.9996119895237171;N_BI_GENOS=10309;South_Han_AN=1496;South_Han_AC=1;South_Ha
chr4	53188316 .	C	<INS:ME:LINE1>	.	PASS	SVTYPE=INS:ME:LINE1;END=53188316;SVLEN=1182;AN=20618;AC=1;AF=4.850130953535746e-05;N_HET=1;N_HOMALT=0;N_HOMREF=10308;FREQ_HET=9.700261907071492e-05;FREQ_HOMALT=0.0;FREQ_HOMREF=0.9999029973809292;N_BI_GENOS=10309;South_Han_AN=1496;South_Han_AC=1;South_Han_
chr4	53190908 .	A	<INS:ME:LINE1>	.	PASS	SVTYPE=INS:ME:LINE1;END=53190958;SVLEN=500;AN=20618;AC=1;AF=4.850130953535746e-05;N_HET=1;N_HOMALT=0;N_HOMREF=10308;FREQ_HET=9.700261907071492e-05;FREQ_HOMALT=0.0;FREQ_HOMREF=0.9999029973809292;N_BI_GENOS=10309;North_Han_AN=2272;North_Han_AC=1;North_Han_A
chr4	53311509 .	A	<INS:ME:LINE1>	.	PASS	SVTYPE=INS:ME:LINE1;END=53311523;SVLEN=434;AN=20618;AC=1;AF=4.850130953535746e-05;N_HET=1;N_HOMALT=0;N_HOMREF=10308;FREQ_HET=9.700261907071492e-05;FREQ_HOMALT=0.0;FREQ_HOMREF=0.9999029973809292;N_BI_GENOS=10309;North_Han_AN=2272;North_Han_AC=1;North_Han_A
chr4	53463517 .	C	<INS:ME:LINE1>	.	PASS	SVTYPE=INS:ME:LINE1;END=53463517;SVLEN=1468;AN=20618;AC=1;AF=4.850130953535746e-05;N_HET=1;N_HOMALT=0;N_HOMREF=10308;FREQ_HET=9.700261907071492e-05;FREQ_HOMALT=0.0;FREQ_HOMREF=0.9999029973809292;N_BI_GENOS=10309;Mongolian_AN=454;Mongolian_AC=1;Mongolian_A
chr4	53502122 .	T	<INS:ME:LINE1>	.	PASS	SVTYPE=INS:ME:LINE1;END=53502136;SVLEN=6000;AN=20618;AC=342;AF=0.016587447861092248;N_HET=332;N_HOMALT=5;N_HOMREF=9972;FREQ_HET=0.03220486953147735;FREQ_HOMALT=0.0004850130953357455;FREQ_HOMREF=0.9673101173731691;N_BI_GENOS=10309;South_Han_AN=1496;South_
chr4	53960116 .	T	<INS:ME:LINE1>	.	PASS	SVTYPE=INS:ME:LINE1;END=53960116;SVLEN=392;AN=20618;AC=1;AF=4.850130953535746e-05;N_HET=1;N_HOMALT=0;N_HOMREF=10308;FREQ_HET=9.700261907071492e-05;FREQ_HOMALT=0.0;FREQ_HOMREF=0.9999029973809292;N_BI_GENOS=10309;East_Han_AN=6554;East_Han_AC=1;East_Han_AF=0
chr4	54858211 .	G	<INS:ME:LINE1>	.	PASS	SVTYPE=INS:ME:LINE1;END=54858211;SVLEN=0;AN=20618;AC=3;AF=0.00014550392860607236;N_HET=1;N_HOMALT=1;N_HOMREF=10307;FREQ_HET=9.700261907071492e-05;FREQ_HOMALT=9.700261907071492e-05;FREQ_HOMREF=0.9998059947618586;N_BI_GENOS=10309;South_Han_AN=1496;South_Han
chr4	55863782 .	A	<INS:ME:LINE1>	.	PASS	SVTYPE=INS:ME:LINE1;END=55863797;SVLEN=5975;AN=20618;AC=4;AF=0.00019400523814142983;N_HET=4;N_HOMALT=0;N_HOMREF=10305;FREQ_HET=0.00038801047628285966;FREQ_HOMALT=0.0;FREQ_HOMREF=0.9996119895237171;N_BI_GENOS=10309;North_Han_AN=2272;North_Han_AC=1;North_Ha
chr4	56187670 .	A	<INS:ME:LINE1>	.	PASS	SVTYPE=INS:ME:LINE1;END=56187684;SVLEN=5999;AN=20618;AC=3;AF=0.00014550392860607236;N_HET=3;N_HOMALT=0;N_HOMREF=10306;FREQ_HET=0.0002910078572121447;FREQ_HOMALT=0.0;FREQ_HOMREF=0.9997089921427879;N_BI_GENOS=10309;Northwest_Han_AN=1296;Northwest_Han_AC=1;N
chr4	56320387 .	C	<INS:ME:LINE1>	.	PASS	SVTYPE=INS:ME:LINE1;END=56320402;SVLEN=6000;AN=20618;AC=63;AF=0.0030555825007275196;N_HET=61;N_HOMALT=1;N_HOMREF=10247;FREQ_HET=0.005917159763313609;FREQ_HOMALT=9.700261907071492e-05;FREQ_HOMREF=0.9939858376176157;N_BI_GENOS=10309;Northwest_Han_AN=1296;No
chr4	56347855 .	A	<INS:ME:LINE1>	.	PASS	SVTYPE=INS:ME:LINE1;END=56347855;SVLEN=32;AN=20618;AC=1;AF=4.850130953535746e-05;N_HET=1;N_HOMALT=0;N_HOMREF=10308;FREQ_HET=9.700261907071492e-05;FREQ_HOMALT=0.0;FREQ_HOMREF=0.9999029973809292;N_BI_GENOS=10309;North_Han_AN=2272;North_Han_AC=1;North_Han_AF
chr4	56630886 .	G	<INS:ME:LINE1>	.	PASS	SVTYPE=INS:ME:LINE1;END=56630901;SVLEN=503;AN=20618;AC=1560;AF=0.07566204287515763;N_HET=1444;N_HOMALT=58;N_HOMREF=8807;FREQ_HET=0.14007178193811232;FREQ_HOMALT=0.005626151906101465;FREQ_HOMREF=0.8543020661557862;N_BI_GENOS=10309;South_Han_AN=1496;South_H

chr4	57260496	.	T	<INS:ME:LINE1>	.	PASS	SVTYPE=INS:ME:LINE1;END=57260511;SVLEN=1137;AN=20618;AC=3047;AF=0.14778349015423417;N_HET=1289;N_HOMALT=879;N_HOMREF=8141;FREQ_HET=0.12503637598215153;FREQ_HOMALT=0.0852653021631584;FREQ_HOMREF=0.78969832185469;N_BI_GENOS=10309;South_Han_AN=1496;South_Han
chr4	57334031	.	A	<INS:ME:LINE1>	.	PASS	SVTYPE=INS:ME:LINE1;END=57334046;SVLEN=6000;AN=20618;AC=1;AF=4.850130953535746e-05;N_HET=1;N_HOMALT=0;N_HOMREF=10308;FREQ_HET=9.700261907071492e-05;FREQ_HOMALT=0.0;FREQ_HOMREF=0.9999029973809292;N_BI_GENOS=10309;North_Han_AN=2272;North_Han_AC=1;North_Han_
chr4	57342638	.	A	<INS:ME:LINE1>	.	PASS	SVTYPE=INS:ME:LINE1;END=57342655;SVLEN=5998;AN=20618;AC=1;AF=4.850130953535746e-05;N_HET=1;N_HOMALT=0;N_HOMREF=10308;FREQ_HET=9.700261907071492e-05;FREQ_HOMALT=0.0;FREQ_HOMREF=0.9999029973809292;N_BI_GENOS=10309;Zhuang_AN=444;Zhuang_AC=1;Zhuang_AF=0.00225
chr4	57360766	.	C	<INS:ME:LINE1>	.	PASS	SVTYPE=INS:ME:LINE1;END=57360782;SVLEN=153;AN=20618;AC=1;AF=4.850130953535746e-05;N_HET=1;N_HOMALT=0;N_HOMREF=10308;FREQ_HET=9.700261907071492e-05;FREQ_HOMALT=0.0;FREQ_HOMREF=0.9999029973809292;N_BI_GENOS=10309;Central_Han_AN=2128;Central_Han_AC=1;Central
chr4	57396160	.	A	<INS:ME:LINE1>	.	PASS	SVTYPE=INS:ME:LINE1;END=57396194;SVLEN=2861;AN=20618;AC=1;AF=4.850130953535746e-05;N_HET=1;N_HOMALT=0;N_HOMREF=10308;FREQ_HET=9.700261907071492e-05;FREQ_HOMALT=0.0;FREQ_HOMREF=0.9999029973809292;N_BI_GENOS=10309;Central_Han_AN=2128;Central_Han_AC=1;Centra
chr4	57415778	.	C	<INS:ME:LINE1>	.	PASS	SVTYPE=INS:ME:LINE1;END=57415790;SVLEN=6000;AN=20618;AC=1;AF=4.850130953535746e-05;N_HET=1;N_HOMALT=0;N_HOMREF=10308;FREQ_HET=9.700261907071492e-05;FREQ_HOMALT=0.0;FREQ_HOMREF=0.9999029973809292;N_BI_GENOS=10309;Central_Han_AN=2128;Central_Han_AC=1;Centra
chr4	57462448	.	A	<INS:ME:LINE1>	.	PASS	SVTYPE=INS:ME:LINE1;END=57462462;SVLEN=876;AN=20618;AC=4;AF=0.00019400523814142983;N_HET=2;N_HOMALT=1;N_HOMREF=10306;FREQ_HET=0.00019400523814142983;FREQ_HOMALT=9.700261907071492e-05;FREQ_HOMREF=0.9997089921427879;N_BI_GENOS=10309;East_Han_AN=6554;East_Ha
chr4	57493534	.	G	<INS:ME:LINE1>	.	PASS	SVTYPE=INS:ME:LINE1;END=57493544;SVLEN=5982;AN=20618;AC=1;AF=4.850130953535746e-05;N_HET=1;N_HOMALT=0;N_HOMREF=10308;FREQ_HET=9.700261907071492e-05;FREQ_HOMALT=0.0;FREQ_HOMREF=0.9999029973809292;N_BI_GENOS=10309;South_Han_AN=1496;South_Han_AC=1;South_Han_
chr4	57520590	.	A	<INS:ME:LINE1>	.	PASS	SVTYPE=INS:ME:LINE1;END=57520604;SVLEN=5997;AN=20618;AC=39;AF=0.0018915510718789407;N_HET=35;N_HOMALT=2;N_HOMREF=10272;FREQ_HET=0.0033950916674750217;FREQ_HOMALT=0.00019400523814142983;FREQ_HOMREF=0.9964109030943835;N_BI_GENOS=10309;South_Han_AN=1496;Sout
chr4	57594293	.	T	<INS:ME:LINE1>	.	PASS	SVTYPE=INS:ME:LINE1;END=57594305;SVLEN=5421;AN=20618;AC=1;AF=4.850130953535746e-05;N_HET=1;N_HOMALT=0;N_HOMREF=10308;FREQ_HET=9.700261907071492e-05;FREQ_HOMALT=0.0;FREQ_HOMREF=0.9999029973809292;N_BI_GENOS=10309;Central_Han_AN=2128;Central_Han_AC=1;Centra
chr4	57617767	.	A	<INS:ME:LINE1>	.	PASS	SVTYPE=INS:ME:LINE1;END=57617780;SVLEN=666;AN=20618;AC=1;AF=4.850130953535746e-05;N_HET=1;N_HOMALT=0;N_HOMREF=10308;FREQ_HET=9.700261907071492e-05;FREQ_HOMALT=0.0;FREQ_HOMREF=0.9999029973809292;N_BI_GENOS=10309;Southeast_Han_AN=1908;Southeast_Han_AC=1;Sou
chr4	57735556	.	G	<INS:ME:LINE1>	.	PASS	SVTYPE=INS:ME:LINE1;END=57735567;SVLEN=568;AN=20618;AC=1;AF=4.850130953535746e-05;N_HET=1;N_HOMALT=0;N_HOMREF=10308;FREQ_HET=9.700261907071492e-05;FREQ_HOMALT=0.0;FREQ_HOMREF=0.9999029973809292;N_BI_GENOS=10309;North_Han_AN=2272;North_Han_AC=1;North_Han_A
chr4	58018480	.	C	<INS:ME:LINE1>	.	PASS	SVTYPE=INS:ME:LINE1;END=58018493;SVLEN=699;AN=20618;AC=2;AF=9.700261907071492e-05;N_HET=2;N_HOMALT=0;N_HOMREF=10307;FREQ_HET=0.00019400523814142983;FREQ_HOMALT=0.0;FREQ_HOMREF=0.9998059947618586;N_BI_GENOS=10309;East_Han_AN=6554;East_Han_AC=2;East_Han_AF=
chr4	58051348	.	T	<INS:ME:LINE1>	.	PASS	SVTYPE=INS:ME:LINE1;END=58051363;SVLEN=1030;AN=20618;AC=8883;AF=0.4308371326025803;N_HET=4537;N_HOMALT=2173;N_HOMREF=3599;FREQ_HET=0.44010088272383335;FREQ_HOMALT=0.2107866912406635;FREQ_HOMREF=0.34911242603550297;N_BI_GENOS=10309;South_Han_AN=1496;South_H
chr4	58184509	.	A	<INS:ME:LINE1>	.	PASS	SVTYPE=INS:ME:LINE1;END=58184524;SVLEN=416;AN=20618;AC=2;AF=9.700261907071492e-05;N_HET=2;N_HOMALT=0;N_HOMREF=10307;FREQ_HET=0.00019400523814142983;FREQ_HOMALT=0.0;FREQ_HOMREF=0.9998059947618586;N_BI_GENOS=10309;East_Han_AN=6554;East_Han_AC=2;East_Han_AF=
chr4	58281208	.	A	<INS:ME:LINE1>	.	PASS	SVTYPE=INS:ME:LINE1;END=58281222;SVLEN=6000;AN=20618;AC=1;AF=4.850130953535746e-05;N_HET=1;N_HOMALT=0;N_HOMREF=10308;FREQ_HET=9.700261907071492e-05;FREQ_HOMALT=0.0;FREQ_HOMREF=0.9999029973809292;N_BI_GENOS=10309;Southeast_Han_AN=1908;Southeast_Han_AC=1;So
chr4	58298940	.	A	<INS:ME:LINE1>	.	PASS	SVTYPE=INS:ME:LINE1;END=58298956;SVLEN=161;AN=20618;AC=1;AF=4.850130953535746e-05;N_HET=1;N_HOMALT=0;N_HOMREF=10308;FREQ_HET=9.700261907071492e-05;FREQ_HOMALT=0.0;FREQ_HOMREF=0.9999029973809292;N_BI_GENOS=10309;Central_Han_AN=2128;Central_Han_AC=1;Central
chr4	58546124	.	T	<INS:ME:LINE1>	.	PASS	SVTYPE=INS:ME:LINE1;END=58546124;SVLEN=0;AN=20618;AC=1;AF=4.850130953535746e-05;N_HET=1;N_HOMALT=0;N_HOMREF=10308;FREQ_HET=9.700261907071492e-05;FREQ_HOMALT=0.0;FREQ_HOMREF=0.9999029973809292;N_BI_GENOS=10309;Zhuang_AN=444;Zhuang_AC=1;Zhuang_AF=0.00225225
chr4	58756275	.	A	<INS:ME:LINE1>	.	PASS	SVTYPE=INS:ME:LINE1;END=58756285;SVLEN=745;AN=20618;AC=10;AF=0.00048501309535357455;N_HET=10;N_HOMALT=0;N_HOMREF=10299;FREQ_HET=0.0009700261907071491;FREQ_HOMALT=0.0;FREQ_HOMREF=0.9990299738092928;N_BI_GENOS=10309;Northwest_Han_AN=1296;Northwest_Han_AC=3;
chr4	58779586	.	A	<INS:ME:LINE1>	.	PASS	SVTYPE=INS:ME:LINE1;END=58779599;SVLEN=1467;AN=20618;AC=1;AF=4.850130953535746e-05;N_HET=1;N_HOMALT=0;N_HOMREF=10308;FREQ_HET=9.700261907071492e-05;FREQ_HOMALT=0.0;FREQ_HOMREF=0.9999029973809292;N_BI_GENOS=10309;Southeast_Han_AN=1908;Southeast_Han_AC=1;So
chr4	58782413	.	G	<INS:ME:LINE1>	.	PASS	SVTYPE=INS:ME:LINE1;END=58782429;SVLEN=2053;AN=20618;AC=3;AF=0.00014550392860607236;N_HET=3;N_HOMALT=0;N_HOMREF=10306;FREQ_HET=0.0002910078572121447;FREQ_HOMALT=0.0;FREQ_HOMREF=0.9997089921427879;N_BI_GENOS=10309;Northwest_Han_AN=1296;Northwest_Han_AC=1;N
chr4	58825753	.	A	<INS:ME:LINE1>	.	PASS	SVTYPE=INS:ME:LINE1;END=58825763;SVLEN=882;AN=20618;AC=5;AF=0.00024250654767678728;N_HET=5;N_HOMALT=0;N_HOMREF=10304;FREQ_HET=0.00048501309535357455;FREQ_HOMALT=0.0;FREQ_HOMREF=0.9995149869046465;N_BI_GENOS=10309;Lingnan_Han_AN=1888;Lingnan_Han_AC=1;Lingn
chr4	58940606	.	A	<INS:ME:LINE1>	.	PASS	SVTYPE=INS:ME:LINE1;END=58940623;SVLEN=1818;AN=20618;AC=2;AF=9.700261907071492e-05;N_HET=2;N_HOMALT=0;N_HOMREF=10307;FREQ_HET=0.00019400523814142983;FREQ_HOMALT=0.0;FREQ_HOMREF=0.9998059947618586;N_BI_GENOS=10309;South_Han_AN=1496;South_Han_AC=1;South_Han
chr4	59060826	.	G	<INS:ME:LINE1>	.	PASS	SVTYPE=INS:ME:LINE1;END=59060839;SVLEN=5141;AN=20618;AC=1;AF=4.850130953535746e-05;N_HET=1;N_HOMALT=0;N_HOMREF=10308;FREQ_HET=9.700261907071492e-05;FREQ_HOMALT=0.0;FREQ_HOMREF=0.9999029973809292;N_BI_GENOS=10309;North_Han_AN=2272;North_Han_AC=1;North_Han_
chr4	59481174	.	A	<INS:ME:LINE1>	.	PASS	SVTYPE=INS:ME:LINE1;END=59481189;SVLEN=3373;AN=20618;AC=1;AF=4.850130953535746e-05;N_HET=1;N_HOMALT=0;N_HOMREF=10308;FREQ_HET=9.700261907071492e-05;FREQ_HOMALT=0.0;FREQ_HOMREF=0.9999029973809292;N_BI_GENOS=10309;Lingnan_Han_AN=1888;Lingnan_Han_AC=1;Lingna
chr4	59539253	.	T	<INS:ME:LINE1>	.	PASS	SVTYPE=INS:ME:LINE1;END=59539267;SVLEN=1173;AN=20618;AC=1;AF=4.850130953535746e-05;N_HET=1;N_HOMALT=0;N_HOMREF=10308;FREQ_HET=9.700261907071492e-05;FREQ_HOMALT=0.0;FREQ_HOMREF=0.9999029973809292;N_BI_GENOS=10309;East_Han_AN=6554;East_Han_AC=1;East_Han_AF=
chr4	59668675	.	T	<INS:ME:LINE1>	.	PASS	SVTYPE=INS:ME:LINE1;END=59668691;SVLEN=2001;AN=20618;AC=1;AF=4.850130953535746e-05;N_HET=1;N_HOMALT=0;N_HOMREF=10308;FREQ_HET=9.700261907071492e-05;FREQ_HOMALT=0.0;FREQ_HOMREF=0.9999029973809292;N_BI_GENOS=10309;Central_Han_AN=2128;Central_Han_AC=1;Centra
chr4	59726185	.	A	<INS:ME:LINE1>	.	PASS	SVTYPE=INS:ME:LINE1;END=59726201;SVLEN=724;AN=20618;AC=2;AF=9.700261907071492e-05;N_HET=2;N_HOMALT=0;N_HOMREF=10307;FREQ_HET=0.00019400523814142983;FREQ_HOMALT=0.0;FREQ_HOMREF=0.9998059947618586;N_BI_GENOS=10309;Central_Han_AN=2128;Central_Han_AC=2;Centra
chr4	59867160	.	A	<INS:ME:LINE1>	.	PASS	SVTYPE=INS:ME:LINE1;END=59867179;SVLEN=1698;AN=20618;AC=4;AF=0.00019400523814142983;N_HET=4;N_HOMALT=0;N_HOMREF=10305;FREQ_HET=0.00038801047628285966;FREQ_HOMALT=0.0;FREQ_HOMREF=0.9996119895237171;N_BI_GENOS=10309;Lingnan_Han_AN=1888;Lingnan_Han_AC=1;Ling
chr4	60014700	.	A	<INS:ME:LINE1>	.	PASS	SVTYPE=INS:ME:LINE1;END=60014700;SVLEN=617;AN=20618;AC=1;AF=4.850130953535746e-05;N_HET=1;N_HOMALT=0;N_HOMREF=10308;FREQ_HET=9.700261907071492e-05;FREQ_HOMALT=0.0;FREQ_HOMREF=0.9999029973809292;N_BI_GENOS=10309;East_Han_AN=6554;East_Han_AC=1;East_Han_AF=0
chr4	60018284	.	A	<INS:ME:LINE1>	.	PASS	SVTYPE=INS:ME:LINE1;END=60018291;SVLEN=460;AN=20618;AC=1;AF=4.850130953535746e-05;N_HET=1;N_HOMALT=0;N_HOMREF=10308;FREQ_HET=9.700261907071492e-05;FREQ_HOMALT=0.0;FREQ_HOMREF=0.9999029973809292;N_BI_GENOS=10309;East_Han_AN=6554;East_Han_AC=1;East_Han_AF=0
chr4	60055765	.	A	<INS:ME:LINE1>	.	PASS	SVTYPE=INS:ME:LINE1;END=60055776;SVLEN=3678;AN=20618;AC=1;AF=4.850130953535746e-05;N_HET=1;N_HOMALT=0;N_HOMREF=10308;FREQ_HET=9.700261907071492e-05;FREQ_HOMALT=0.0;FREQ_HOMREF=0.9999029973809292;N_BI_GENOS=10309;Tibetan_AN=414;Tibetan_AC=1;Tibetan_AF=0.00
chr4	60076365	.	A	<INS:ME:LINE1>	.	PASS	SVTYPE=INS:ME:LINE1;END=60076382;SVLEN=219;AN=20618;AC=6;AF=0.0002910078572121447;N_HET=6;N_HOMALT=0;N_HOMREF=10303;FREQ_HET=0.0005820157144242894;FREQ_HOMALT=0.0;FREQ_HOMREF=0.9994179842855757;N_BI_GENOS=10309;Northwest_Han_AN=1296;Northwest_Han_AC=1;Nor
chr4	60113839	.	G	<INS:ME:LINE1>	.	PASS	SVTYPE=INS:ME:LINE1;END=60113855;SVLEN=3398;AN=20618;AC=26;AF=0.001261034047919 2938;N_HET=24;N_HOMALT=1;N_HOMREF=10284;FREQ_HET=0.0023280628576971578;FREQ_HOMALT=9.700261907071492e-05;FREQ_HOMREF=0.9975749345232321;N_BI_GENOS=10309;Northwest_Han_AN=1296;N
chr4	60262536	.	G	<INS:ME:LINE1>	.	PASS	SVTYPE=INS:ME:LINE1;END=60262550;SVLEN=864;AN=20618;AC=1;AF=4.850130953535746e-05;N_HET=1;N_HOMALT=0;N_HOMREF=10308;FREQ_HET=9.700261907071492e-05;FREQ_HOMALT=0.0;FREQ_HOMREF=0.9999029973809292;N_BI_GENOS=10309;East_Han_AN=6554;East_Han_AC=1;East_Han_AF=0
chr4	60322748	.	A	<INS:ME:LINE1>	.	PASS	SVTYPE=INS:ME:LINE1;END=60322762;SVLEN=840;AN=20618;AC=1;AF=4.850130953535746e-05;N_HET=1;N_HOMALT=0;N_HOMREF=10308;FREQ_HET=9.700261907071492e-05;FREQ_HOMALT=0.0;FREQ_HOMREF=0.9999029973809292;N_BI_GENOS=10309;Hui_AN=444;Hui_AC=1;Hui_AF=0.002252252252252
chr4	60330369	.	T	<INS:ME:LINE1>	.	PASS	SVTYPE=INS:ME:LINE1;END=60330370;SVLEN=787;AN=20618;AC=5;AF=0.00024250654767678728;N_HET=5;N_HOMALT=0;N_HOMREF=10304;FREQ_HET=0.00048501309535357455;FREQ_HOMALT=0.0;FREQ_HOMREF=0.9995149869046465;N_BI_GENOS=10309;East_Han_AN=6554;East_Han_AC=4;East_Han_AF
chr4	60358148	.	T	<INS:ME:LINE1>	.	PASS	SVTYPE=INS:ME:LINE1;END=60358173;SVLEN=19;AN=20618;AC=2;AF=9.700261907071492e-05;N_HET=0;N_HOMALT=1;N_HOMREF=10308;FREQ_HET=0.0;FREQ_HOMALT=9.700261907071492e-05;FREQ_HOMREF=0.9999029973809292;N_BI_GENOS=10309;East_Han_AN=6554;East_Han_AC=2;East_Han_AF=0.
chr4	60433619	.	C	<INS:ME:LINE1>	.	PASS	SVTYPE=INS:ME:LINE1;END=60433619;SVLEN=389;AN=20618;AC=1;AF=4.850130953535746e-05;N_HET=1;N_HOMALT=0;N_HOMREF=10308;FREQ_HET=9.700261907071492e-05;FREQ_HOMALT=0.0;FREQ_HOMREF=0.9999029973809292;N_BI_GENOS=10309;East_Han_AN=6554;East_Han_AC=1;East_Han_AF=0
chr4	61006200	.	A	<INS:ME:LINE1>	.	PASS	SVTYPE=INS:ME:LINE1;END=61006214;SVLEN=1126;AN=20618;AC=5;AF=0.00024250654767678728;N_HET=5;N_HOMALT=0;N_HOMREF=10304;FREQ_HET=0.00048501309535357455;FREQ_HOMALT=0.0;FREQ_HOMREF=0.9995149869046465;N_BI_GENOS=10309;South_Han_AN=1496;South_Han_AC=3;South_Ha
chr4	61148119	.	G	<INS:ME:LINE1>	.	PASS	SVTYPE=INS:ME:LINE1;END=61148131;SVLEN=6000;AN=20618;AC=10;AF=0.00048501309535357455;N_HET=8;N_HOMALT=1;N_HOMREF=10300;FREQ_HET=0.0007760209525657193;FREQ_HOMALT=9.700261907071492e-05;FREQ_HOMREF=0.9991269764283636;N_BI_GENOS=10309;East_Han_AN=6554;East_H
chr4	61189671	.	A	<INS:ME:LINE1>	.	PASS	SVTYPE=INS:ME:LINE1;END=61189671;SVLEN=0;AN=20618;AC=2;AF=9.700261907071492e-05;N_HET=0;N_HOMALT=1;N_HOMREF=10308;FREQ_HET=0.0;FREQ_HOMALT=9.700261907071492e-05;FREQ_HOMREF=0.9999029973809292;N_BI_GENOS=10309;East_Han_AN=6554;East_Han_AC=2;East_Han_AF=0.0
chr4	61357089	.	A	<INS:ME:LINE1>	.	PASS	SVTYPE=INS:ME:LINE1;END=61357104;SVLEN=6035;AN=20618;AC=1;AF=4.850130953535746e-05;N_HET=1;N_HOMALT=0;N_HOMREF=10308;FREQ_HET=9.700261907071492e-05;FREQ_HOMALT=0.0;FREQ_HOMREF=0.9999029973809292;N_BI_GENOS=10309;North_Han_AN=2272;North_Han_AC=1;North_Han_
chr4	61448300	.	A	<INS:ME:LINE1>	.	PASS	SVTYPE=INS:ME:LINE1;END=61448316;SVLEN=509;AN=20618;AC=1;AF=4.850130953535746e-05;N_HET=1;N_HOMALT=0;N_HOMREF=10308;FREQ_HET=9.700261907071492e-05;FREQ_HOMALT=0.0;FREQ_HOMREF=0.9999029973809292;N_BI_GENOS=10309;Hui_AN=444;Hui_AC=1;Hui_AF=0.002252252252252
chr4	61562326	.	G	<INS:ME:LINE1>	.	PASS	SVTYPE=INS:ME:LINE1;END=61562337;SVLEN=6000;AN=20618;AC=23;AF=0.0011155301193132214;N_HET=23;N_HOMALT=0;N_HOMREF=10286;FREQ_HET=0.002231060238626443;FREQ_HOMALT=0.0;FREQ_HOMREF=0.9977689397613736;N_BI_GENOS=10309;South_Han_AN=1496;South_Han_AC=1;South_Han
chr4	61831231	.	G	<INS:ME:LINE1>	.	PASS	SVTYPE=INS:ME:LINE1;END=61831247;SVLEN=2066;AN=20618;AC=3;AF=0.00014550392860607236;N_HET=3;N_HOMALT=0;N_HOMREF=10306;FREQ_HET=0.0002910078572121447;FREQ_HOMALT=0.0;FREQ_HOMREF=0.9997089921427879;N_BI_GENOS=10309;Lingnan_Han_AN=1888;Lingnan_Han_AC=2;Lingn
chr4	62479707	.	A	<INS:ME:LINE1>	.	PASS	SVTYPE=INS:ME:LINE1;END=62479721;SVLEN=923;AN=20618;AC=1;AF=4.850130953535746e-05;N_HET=1;N_HOMALT=0;N_HOMREF=10308;FREQ_HET=9.700261907071492e-05;FREQ_HOMALT=0.0;FREQ_HOMREF=0.9999029973809292;N_BI_GENOS=10309;Central_Han_AN=2128;Central_Han_AC=1;Central
chr4	62554019	.	T	<INS:ME:LINE1>	.	PASS	SVTYPE=INS:ME:LINE1;END=62554019;SVLEN=1121;AN=20618;AC=1;AF=4.850130953535746e-05;N_HET=1;N_HOMALT=0;N_HOMREF=10308;FREQ_HET=9.700261907071492e-05;FREQ_HOMALT=0.0;FREQ_HOMREF=0.9999029973809292;N_BI_GENOS=10309;East_Han_AN=6554;East_Han_AC=1;East_Han_AF=
chr4	62663490	.	T	<INS:ME:LINE1>	.	PASS	SVTYPE=INS:ME:LINE1;END=62663503;SVLEN=5998;AN=20618;AC=2;AF=9.700261907071492e-05;N_HET=2;N_HOMALT=0;N_HOMREF=10307;FREQ_HET=0.00019400523814142983;FREQ_HOMALT=0.0;FREQ_HOMREF=0.9998059947618586;N_BI_GENOS=10309;Lingnan_Han_AN=1888;Lingnan_Han_AC=1;Lingn
chr4	62675505	.	A	<INS:ME:LINE1>	.	PASS	SVTYPE=INS:ME:LINE1;END=62675517;SVLEN=2000;AN=20618;AC=6;AF=0.0002910078572121447;N_HET=6;N_HOMALT=0;N_HOMREF=10303;FREQ_HET=0.0005820157144242894;FREQ_HOMALT=0.0;FREQ_HOMREF=0.9994179842855757;N_BI_GENOS=10309;South_Han_AN=1496;South_Han_AC=1;South_Han_
chr4	63062068	.	C	<INS:ME:LINE1>	.	PASS	SVTYPE=INS:ME:LINE1;END=63062068;SVLEN=0;AN=20618;AC=2;AF=9.700261907071492e-05;N_HET=2;N_HOMALT=0;N_HOMREF=10307;FREQ_HET=0.00019400523814142983;FREQ_HOMALT=0.0;FREQ_HOMREF=0.9998059947618586;N_BI_GENOS=10309;Northwest_Han_AN=1296;Northwest_Han_AC=1;Nort
chr4	63123236	.	T	<INS:ME:LINE1>	.	PASS	SVTYPE=INS:ME:LINE1;END=63123252;SVLEN=1248;AN=20618;AC=1;AF=4.850130953535746e-05;N_HET=1;N_HOMALT=0;N_HOMREF=10308;FREQ_HET=9.700261907071492e-05;FREQ_HOMALT=0.0;FREQ_HOMREF=0.9999029973809292;N_BI_GENOS=10309;East_Han_AN=6554;East_Han_AC=1;East_Han_AF=
chr4	63278182	.	A	<INS:ME:LINE1>	.	PASS	SVTYPE=INS:ME:LINE1;END=63278196;SVLEN=5960;AN=20618;AC=131;AF=0.006353671549131827;N_HET=111;N_HOMALT=10;N_HOMREF=10188;FREQ_HET=0.010767290716849355;FREQ_HOMALT=0.0009700261907071491;FREQ_HOMREF=0.9882626830924435;N_BI_GENOS=10309;South_Han_AN=1496;Sout
chr4	63279189	.	C	<INS:ME:LINE1>	.	PASS	SVTYPE=INS:ME:LINE1;END=63279189;SVLEN=2421;AN=20618;AC=1;AF=4.850130953535746e-05;N_HET=1;N_HOMALT=0;N_HOMREF=10308;FREQ_HET=9.700261907071492e-05;FREQ_HOMALT=0.0;FREQ_HOMREF=0.9999029973809292;N_BI_GENOS=10309;East_Han_AN=6554;East_Han_AC=1;East_Han_AF=
chr4	63302658	.	T	<INS:ME:LINE1>	.	PASS	SVTYPE=INS:ME:LINE1;END=63302659;SVLEN=467;AN=20618;AC=13;AF=0.0006305170239596469;N_HET=11;N_HOMALT=1;N_HOMREF=10297;FREQ_HET=0.001067028809777864;FREQ_HOMALT=9.700261907071492e-05;FREQ_HOMREF=0.9988359685711514;N_BI_GENOS=10309;South_Han_AN=1496;South_H
chr4	63773361	.	A	<INS:ME:LINE1>	.	PASS	SVTYPE=INS:ME:LINE1;END=63773376;SVLEN=518;AN=20618;AC=1;AF=4.850130953535746e-05;N_HET=1;N_HOMALT=0;N_HOMREF=10308;FREQ_HET=9.700261907071492e-05;FREQ_HOMALT=0.0;FREQ_HOMREF=0.9999029973809292;N_BI_GENOS=10309;North_Han_AN=2272;North_Han_AC=1;North_Han_A
chr4	63810190	.	G	<INS:ME:LINE1>	.	PASS	SVTYPE=INS:ME:LINE1;END=63810234;SVLEN=1459;AN=20618;AC=1;AF=4.850130953535746e-05;N_HET=1;N_HOMALT=0;N_HOMREF=10308;FREQ_HET=9.700261907071492e-05;FREQ_HOMALT=0.0;FREQ_HOMREF=0.9999029973809292;N_BI_GENOS=10309;East_Han_AN=6554;East_Han_AC=1;East_Han_AF=
chr4	63810364	.	G	<INS:ME:LINE1>	.	PASS	SVTYPE=INS:ME:LINE1;END=63810376;SVLEN=1666;AN=20618;AC=1;AF=4.850130953535746e-05;N_HET=1;N_HOMALT=0;N_HOMREF=10308;FREQ_HET=9.700261907071492e-05;FREQ_HOMALT=0.0;FREQ_HOMREF=0.9999029973809292;N_BI_GENOS=10309;East_Han_AN=6554;East_Han_AC=1;East_Han_AF=
chr4	63886966	.	A	<INS:ME:LINE1>	.	PASS	SVTYPE=INS:ME:LINE1;END=63886966;SVLEN=865;AN=20618;AC=1;AF=4.850130953535746e-05;N_HET=1;N_HOMALT=0;N_HOMREF=10308;FREQ_HET=9.700261907071492e-05;FREQ_HOMALT=0.0;FREQ_HOMREF=0.9999029973809292;N_BI_GENOS=10309;East_Han_AN=6554;East_Han_AC=1;East_Han_AF=0
chr4	64242108	.	G	<INS:ME:LINE1>	.	PASS	SVTYPE=INS:ME:LINE1;END=64242118;SVLEN=5997;AN=20618;AC=1;AF=4.850130953535746e-05;N_HET=1;N_HOMALT=0;N_HOMREF=10308;FREQ_HET=9.700261907071492e-05;FREQ_HOMALT=0.0;FREQ_HOMREF=0.9999029973809292;N_BI_GENOS=10309;Lingnan_Han_AN=1888;Lingnan_Han_AC=1;Lingna
chr4	64819107	.	G	<INS:ME:LINE1>	.	PASS	SVTYPE=INS:ME:LINE1;END=64819123;SVLEN=1803;AN=20618;AC=1;AF=4.850130953535746e-05;N_HET=1;N_HOMALT=0;N_HOMREF=10308;FREQ_HET=9.700261907071492e-05;FREQ_HOMALT=0.0;FREQ_HOMREF=0.9999029973809292;N_BI_GENOS=10309;North_Han_AN=2272;North_Han_AC=1;North_Han_
chr4	64975342	.	A	<INS:ME:LINE1>	.	PASS	SVTYPE=INS:ME:LINE1;END=64975358;SVLEN=456;AN=20618;AC=1;AF=4.850130953535746e-05;N_HET=1;N_HOMALT=0;N_HOMREF=10308;FREQ_HET=9.700261907071492e-05;FREQ_HOMALT=0.0;FREQ_HOMREF=0.9999029973809292;N_BI_GENOS=10309;East_Han_AN=6554;East_Han_AC=1;East_Han_AF=0
chr4	65007521	.	T	<INS:ME:LINE1>	.	PASS	SVTYPE=INS:ME:LINE1;END=65007535;SVLEN=5997;AN=20618;AC=3;AF=0.00014550392860607236;N_HET=3;N_HOMALT=0;N_HOMREF=10306;FREQ_HET=0.0002910078572121447;FREQ_HOMALT=0.0;FREQ_HOMREF=0.9997089921427879;N_BI_GENOS=10309;Southeast_Han_AN=1908;Southeast_Han_AC=3;S
chr4	65105581	.	T	<INS:ME:LINE1>	.	PASS	SVTYPE=INS:ME:LINE1;END=65105581;SVLEN=2408;AN=20618;AC=1;AF=4.850130953535746e-05;N_HET=1;N_HOMALT=0;N_HOMREF=10308;FREQ_HET=9.700261907071492e-05;FREQ_HOMALT=0.0;FREQ_HOMREF=0.9999029973809292;N_BI_GENOS=10309;East_Han_AN=6554;East_Han_AC=1;East_Han_AF=
chr4	65120676	.	T	<INS:ME:LINE1>	.	PASS	SVTYPE=INS:ME:LINE1;END=65120684;SVLEN=1147;AN=20618;AC=37;AF=0.0017945484528082258;N_HET=37;N_HOMALT=0;N_HOMREF=10272;FREQ_HET=0.0035890969056164516;FREQ_HOMALT=0.0;FREQ_HOMREF=0.9964109030943835;N_BI_GENOS=10309;South_Han_AN=1496;South_Han_AC=5;South_Ha
chr4	65218333	.	G	<INS:ME:LINE1>	.	PASS	SVTYPE=INS:ME:LINE1;END=65218333;SVLEN=122;AN=20618;AC=1;AF=4.850130953535746e-05;N_HET=1;N_HOMALT=0;N_HOMREF=10308;FREQ_HET=9.700261907071492e-05;FREQ_HOMALT=0.0;FREQ_HOMREF=0.9999029973809292;N_BI_GENOS=10309;East_Han_AN=6554;East_Han_AC=1;East_Han_AF=0
chr4	65280438	.	A	<INS:ME:LINE1>	.	PASS	SVTYPE=INS:ME:LINE1;END=65280440;SVLEN=1862;AN=20618;AC=1;AF=4.850130953535746e-05;N_HET=1;N_HOMALT=0;N_HOMREF=10308;FREQ_HET=9.700261907071492e-05;FREQ_HOMALT=0.0;FREQ_HOMREF=0.9999029973809292;N_BI_GENOS=10309;Tibetan_AN=414;Tibetan_AC=1;Tibetan_AF=0.00
chr4	65548212	.	A	<INS:ME:LINE1>	.	PASS	SVTYPE=INS:ME:LINE1;END=65548214;SVLEN=227;AN=20618;AC=6046;AF=0.2932389174507712;N_HET=3380;N_HOMALT=1333;N_HOMREF=5596;FREQ_HET=0.32786885245901637;FREQ_HOMALT=0.12930449122126297;FREQ_HOMREF=0.5428266563197206;N_BI_GENOS=10309;South_Han_AN=1496;South_H
chr4	65565680	.	A	<INS:ME:LINE1>	.	PASS	SVTYPE=INS:ME:LINE1;END=65565691;SVLEN=1626;AN=20618;AC=1;AF=4.850130953535746e-05;N_HET=1;N_HOMALT=0;N_HOMREF=10308;FREQ_HET=9.700261907071492e-05;FREQ_HOMALT=0.0;FREQ_HOMREF=0.9999029973809292;N_BI_GENOS=10309;South_Han_AN=1496;South_Han_AC=1;South_Han_
chr4	65618725	.	A	<INS:ME:LINE1>	.	PASS	SVTYPE=INS:ME:LINE1;END=65618742;SVLEN=319;AN=20618;AC=1;AF=4.850130953535746e-05;N_HET=1;N_HOMALT=0;N_HOMREF=10308;FREQ_HET=9.700261907071492e-05;FREQ_HOMALT=0.0;FREQ_HOMREF=0.9999029973809292;N_BI_GENOS=10309;East_Han_AN=6554;East_Han_AC=1;East_Han_AF=0
chr4	65754500	.	A	<INS:ME:LINE1>	.	PASS	SVTYPE=INS:ME:LINE1;END=65754516;SVLEN=5997;AN=20618;AC=1;AF=4.850130953535746e-05;N_HET=1;N_HOMALT=0;N_HOMREF=10308;FREQ_HET=9.700261907071492e-05;FREQ_HOMALT=0.0;FREQ_HOMREF=0.9999029973809292;N_BI_GENOS=10309;Central_Han_AN=2128;Central_Han_AC=1;Centra
chr4	65788090	.	T	<INS:ME:LINE1>	.	PASS	SVTYPE=INS:ME:LINE1;END=65788103;SVLEN=2115;AN=20618;AC=16;AF=0.0007760209525657193;N_HET=16;N_HOMALT=0;N_HOMREF=10293;FREQ_HET=0.0015520419051314386;FREQ_HOMALT=0.0;FREQ_HOMREF=0.9984479580948685;N_BI_GENOS=10309;South_Han_AN=1496;South_Han_AC=4;South_Ha
chr4	65855594	.	C	<INS:ME:LINE1>	.	PASS	SVTYPE=INS:ME:LINE1;END=65855607;SVLEN=2238;AN=20618;AC=1;AF=4.850130953535746e-05;N_HET=1;N_HOMALT=0;N_HOMREF=10308;FREQ_HET=9.700261907071492e-05;FREQ_HOMALT=0.0;FREQ_HOMREF=0.9999029973809292;N_BI_GENOS=10309;Central_Han_AN=2128;Central_Han_AC=1;Centra
chr4	65943352	.	C	<INS:ME:LINE1>	.	PASS	SVTYPE=INS:ME:LINE1;END=65943360;SVLEN=1974;AN=20618;AC=1597;AF=0.07745659132796585;N_HET=1425;N_HOMALT=86;N_HOMREF=8798;FREQ_HET=0.13822873217576875;FREQ_HOMALT=0.008342225240081482;FREQ_HOMREF=0.8534290425841498;N_BI_GENOS=10309;South_Han_AN=1496;South_
chr4	65958784	.	C	<INS:ME:LINE1>	.	PASS	SVTYPE=INS:ME:LINE1;END=65958784;SVLEN=1397;AN=20618;AC=1;AF=4.850130953535746e-05;N_HET=1;N_HOMALT=0;N_HOMREF=10308;FREQ_HET=9.700261907071492e-05;FREQ_HOMALT=0.0;FREQ_HOMREF=0.9999029973809292;N_BI_GENOS=10309;East_Han_AN=6554;East_Han_AC=1;East_Han_AF=
chr4	65962405	.	A	<INS:ME:LINE1>	.	PASS	SVTYPE=INS:ME:LINE1;END=65962405;SVLEN=207;AN=20618;AC=1;AF=4.850130953535746e-05;N_HET=1;N_HOMALT=0;N_HOMREF=10308;FREQ_HET=9.700261907071492e-05;FREQ_HOMALT=0.0;FREQ_HOMREF=0.9999029973809292;N_BI_GENOS=10309;East_Han_AN=6554;East_Han_AC=1;East_Han_AF=0
chr4	66033699	.	T	<INS:ME:LINE1>	.	PASS	SVTYPE=INS:ME:LINE1;END=66033715;SVLEN=1685;AN=20618;AC=1;AF=4.850130953535746e-05;N_HET=1;N_HOMALT=0;N_HOMREF=10308;FREQ_HET=9.700261907071492e-05;FREQ_HOMALT=0.0;FREQ_HOMREF=0.9999029973809292;N_BI_GENOS=10309;Northwest_Han_AN=1296;Northwest_Han_AC=1;No
chr4	66392678	.	A	<INS:ME:LINE1>	.	PASS	SVTYPE=INS:ME:LINE1;END=66392692;SVLEN=6028;AN=20618;AC=2;AF=9.700261907071492e-05;N_HET=2;N_HOMALT=0;N_HOMREF=10307;FREQ_HET=0.00019400523814142983;FREQ_HOMALT=0.0;FREQ_HOMREF=0.9998059947618586;N_BI_GENOS=10309;East_Han_AN=6554;East_Han_AC=1;East_Han_AF
chr4	66585553	.	G	<INS:ME:LINE1>	.	PASS	SVTYPE=INS:ME:LINE1;END=66585559;SVLEN=3174;AN=20618;AC=1;AF=4.850130953535746e-05;N_HET=1;N_HOMALT=0;N_HOMREF=10308;FREQ_HET=9.700261907071492e-05;FREQ_HOMALT=0.0;FREQ_HOMREF=0.9999029973809292;N_BI_GENOS=10309;Southeast_Han_AN=1908;Southeast_Han_AC=1;So
chr4	66604183	.	A	<INS:ME:LINE1>	.	PASS	SVTYPE=INS:ME:LINE1;END=66604197;SVLEN=381;AN=20618;AC=1;AF=4.850130953535746e-05;N_HET=1;N_HOMALT=0;N_HOMREF=10308;FREQ_HET=9.700261907071492e-05;FREQ_HOMALT=0.0;FREQ_HOMREF=0.9999029973809292;N_BI_GENOS=10309;Southeast_Han_AN=1908;Southeast_Han_AC=1;Sou
chr4	66613899	.	G	<INS:ME:LINE1>	.	PASS	SVTYPE=INS:ME:LINE1;END=66613899;SVLEN=1504;AN=20618;AC=1;AF=4.850130953535746e-05;N_HET=1;N_HOMALT=0;N_HOMREF=10308;FREQ_HET=9.700261907071492e-05;FREQ_HOMALT=0.0;FREQ_HOMREF=0.9999029973809292;N_BI_GENOS=10309;East_Han_AN=6554;East_Han_AC=1;East_Han_AF=
chr4	66762591	.	T	<INS:ME:LINE1>	.	PASS	SVTYPE=INS:ME:LINE1;END=66762600;SVLEN=711;AN=20618;AC=2;AF=9.700261907071492e-05;N_HET=2;N_HOMALT=0;N_HOMREF=10307;FREQ_HET=0.00019400523814142983;FREQ_HOMALT=0.0;FREQ_HOMREF=0.9998059947618586;N_BI_GENOS=10309;South_Han_AN=1496;South_Han_AC=1;South_Han_
chr4	66957316	.	A	<INS:ME:LINE1>	.	PASS	SVTYPE=INS:ME:LINE1;END=66957335;SVLEN=2545;AN=20618;AC=6;AF=0.0002910078572121447;N_HET=6;N_HOMALT=0;N_HOMREF=10303;FREQ_HET=0.0005820157144242894;FREQ_HOMALT=0.0;FREQ_HOMREF=0.9994179842855757;N_BI_GENOS=10309;South_Han_AN=1496;South_Han_AC=1;South_Han_
chr4	67078532	.	A	<INS:ME:LINE1>	.	PASS	SVTYPE=INS:ME:LINE1;END=67078549;SVLEN=1820;AN=20618;AC=1;AF=4.850130953535746e-05;N_HET=1;N_HOMALT=0;N_HOMREF=10308;FREQ_HET=9.700261907071492e-05;FREQ_HOMALT=0.0;FREQ_HOMREF=0.9999029973809292;N_BI_GENOS=10309;North_Han_AN=2272;North_Han_AC=1;North_Han_
chr4	67223967	.	T	<INS:ME:LINE1>	.	PASS	SVTYPE=INS:ME:LINE1;END=67223980;SVLEN=6003;AN=20618;AC=1;AF=4.850130953535746e-05;N_HET=1;N_HOMALT=0;N_HOMREF=10308;FREQ_HET=9.700261907071492e-05;FREQ_HOMALT=0.0;FREQ_HOMREF=0.9999029973809292;N_BI_GENOS=10309;Central_Han_AN=2128;Central_Han_AC=1;Centra
chr4	67923508	.	T	<INS:ME:LINE1>	.	PASS	SVTYPE=INS:ME:LINE1;END=67923524;SVLEN=800;AN=20618;AC=6;AF=0.0002910078572121447;N_HET=4;N_HOMALT=1;N_HOMREF=10304;FREQ_HET=0.00038801047628285966;FREQ_HOMALT=9.700261907071492e-05;FREQ_HOMREF=0.9995149869046465;N_BI_GENOS=10309;East_Han_AN=6554;East_Han
chr4	67989973	.	T	<INS:ME:LINE1>	.	PASS	SVTYPE=INS:ME:LINE1;END=67989992;SVLEN=5372;AN=20618;AC=1;AF=4.850130953535746e-05;N_HET=1;N_HOMALT=0;N_HOMREF=10308;FREQ_HET=9.700261907071492e-05;FREQ_HOMALT=0.0;FREQ_HOMREF=0.9999029973809292;N_BI_GENOS=10309;East_Han_AN=6554;East_Han_AC=1;East_Han_AF=
chr4	68028936	.	A	<INS:ME:LINE1>	.	PASS	SVTYPE=INS:ME:LINE1;END=68028946;SVLEN=6002;AN=20618;AC=1;AF=4.850130953535746e-05;N_HET=1;N_HOMALT=0;N_HOMREF=10308;FREQ_HET=9.700261907071492e-05;FREQ_HOMALT=0.0;FREQ_HOMREF=0.9999029973809292;N_BI_GENOS=10309;Lingnan_Han_AN=1888;Lingnan_Han_AC=1;Lingna
chr4	68217638	.	C	<INS:ME:LINE1>	.	PASS	SVTYPE=INS:ME:LINE1;END=68217651;SVLEN=314;AN=20618;AC=1;AF=4.850130953535746e-05;N_HET=1;N_HOMALT=0;N_HOMREF=10308;FREQ_HET=9.700261907071492e-05;FREQ_HOMALT=0.0;FREQ_HOMREF=0.9999029973809292;N_BI_GENOS=10309;North_Han_AN=2272;North_Han_AC=1;North_Han_A
chr4	69191234	.	G	<INS:ME:LINE1>	.	PASS	SVTYPE=INS:ME:LINE1;END=69191248;SVLEN=1086;AN=20618;AC=1;AF=4.850130953535746e-05;N_HET=1;N_HOMALT=0;N_HOMREF=10308;FREQ_HET=9.700261907071492e-05;FREQ_HOMALT=0.0;FREQ_HOMREF=0.9999029973809292;N_BI_GENOS=10309;Central_Han_AN=2128;Central_Han_AC=1;Centra
chr4	69240305	.	G	<INS:ME:LINE1>	.	PASS	SVTYPE=INS:ME:LINE1;END=69240305;SVLEN=2525;AN=20618;AC=1;AF=4.850130953535746e-05;N_HET=1;N_HOMALT=0;N_HOMREF=10308;FREQ_HET=9.700261907071492e-05;FREQ_HOMALT=0.0;FREQ_HOMREF=0.9999029973809292;N_BI_GENOS=10309;Central_Han_AN=2128;Central_Han_AC=1;Centra
chr4	69444133	.	G	<INS:ME:LINE1>	.	PASS	SVTYPE=INS:ME:LINE1;END=69444148;SVLEN=715;AN=20618;AC=1;AF=4.850130953535746e-05;N_HET=1;N_HOMALT=0;N_HOMREF=10308;FREQ_HET=9.700261907071492e-05;FREQ_HOMALT=0.0;FREQ_HOMREF=0.9999029973809292;N_BI_GENOS=10309;Tibetan_AN=414;Tibetan_AC=1;Tibetan_AF=0.002
chr4	69459258	.	A	<INS:ME:LINE1>	.	PASS	SVTYPE=INS:ME:LINE1;END=69459275;SVLEN=621;AN=20618;AC=6;AF=0.0002910078572121447;N_HET=6;N_HOMALT=0;N_HOMREF=10303;FREQ_HET=0.0005820157144242894;FREQ_HOMALT=0.0;FREQ_HOMREF=0.9994179842855757;N_BI_GENOS=10309;North_Han_AN=2272;North_Han_AC=2;North_Han_A
chr4	69541468	.	A	<INS:ME:LINE1>	.	PASS	SVTYPE=INS:ME:LINE1;END=69541478;SVLEN=11;AN=20618;AC=3;AF=0.00014550392860607236;N_HET=1;N_HOMALT=1;N_HOMREF=10307;FREQ_HET=9.700261907071492e-05;FREQ_HOMALT=9.700261907071492e-05;FREQ_HOMREF=0.9998059947618586;N_BI_GENOS=10309;Central_Han_AN=2128;Centra
chr4	69614608	.	T	<INS:ME:LINE1>	.	PASS	SVTYPE=INS:ME:LINE1;END=69614617;SVLEN=1099;AN=20618;AC=1;AF=4.850130953535746e-05;N_HET=1;N_HOMALT=0;N_HOMREF=10308;FREQ_HET=9.700261907071492e-05;FREQ_HOMALT=0.0;FREQ_HOMREF=0.9999029973809292;N_BI_GENOS=10309;East_Han_AN=6554;East_Han_AC=1;East_Han_AF=
chr4	69706255	.	A	<INS:ME:LINE1>	.	PASS	SVTYPE=INS:ME:LINE1;END=69706271;SVLEN=2533;AN=20618;AC=1;AF=4.850130953535746e-05;N_HET=1;N_HOMALT=0;N_HOMREF=10308;FREQ_HET=9.700261907071492e-05;FREQ_HOMALT=0.0;FREQ_HOMREF=0.9999029973809292;N_BI_GENOS=10309;East_Han_AN=6554;East_Han_AC=1;East_Han_AF=

chr4	69884157	.	A	<INS:ME:LINE1>	.	PASS	SVTYPE=INS:ME:LINE1;END=69884175;SVLEN=3515;AN=20618;AC=1;AF=4.850130953535746e-05;N_HET=1;N_HOMALT=0;N_HOMREF=10308;FREQ_HET=9.700261907071492e-05;FREQ_HOMALT=0.0;FREQ_HOMREF=0.9999029973809292;N_BI_GENOS=10309;Central_Han_AN=2128;Central_Han_AC=1;Centra
chr4	69953200	.	T	<INS:ME:LINE1>	.	PASS	SVTYPE=INS:ME:LINE1;END=69953201;SVLEN=72;AN=20618;AC=1;AF=4.850130953535746e-05;N_HET=1;N_HOMALT=0;N_HOMREF=10308;FREQ_HET=9.700261907071492e-05;FREQ_HOMALT=0.0;FREQ_HOMREF=0.9999029973809292;N_BI_GENOS=10309;Central_Han_AN=2128;Central_Han_AC=1;Central_
chr4	70029284	.	A	<INS:ME:LINE1>	.	PASS	SVTYPE=INS:ME:LINE1;END=70029296;SVLEN=1514;AN=20618;AC=121;AF=0.005868658453778252;N_HET=109;N_HOMALT=6;N_HOMREF=10194;FREQ_HET=0.010573285478707926;FREQ_HOMALT=0.0005820157144242894;FREQ_HOMREF=0.9888446988068678;N_BI_GENOS=10309;South_Han_AN=1496;South
chr4	70160474	.	T	<INS:ME:LINE1>	.	PASS	SVTYPE=INS:ME:LINE1;END=70160474;SVLEN=0;AN=20618;AC=7;AF=0.0003395091667475022;N_HET=1;N_HOMALT=3;N_HOMREF=10305;FREQ_HET=9.700261907071492e-05;FREQ_HOMALT=0.0002910078572121447;FREQ_HOMREF=0.9996119895237171;N_BI_GENOS=10309;Northwest_Han_AN=1296;Northw
chr4	70227583	.	A	<INS:ME:LINE1>	.	PASS	SVTYPE=INS:ME:LINE1;END=70227595;SVLEN=794;AN=20618;AC=4965;AF=0.24080900184304976;N_HET=3457;N_HOMALT=754;N_HOMREF=6098;FREQ_HET=0.3353380541274614;FREQ_HOMALT=0.07313997477931904;FREQ_HOMREF=0.5915219710932195;N_BI_GENOS=10309;South_Han_AN=1496;South_Ha
chr4	70359744	.	T	<INS:ME:LINE1>	.	PASS	SVTYPE=INS:ME:LINE1;END=70359747;SVLEN=834;AN=20618;AC=2;AF=9.700261907071492e-05;N_HET=2;N_HOMALT=0;N_HOMREF=10307;FREQ_HET=0.0001940052381414 2983;FREQ_HOMALT=0.0;FREQ_HOMREF=0.9998059947618586;N_BI_GENOS=10309;Zhuang_AN=444;Zhuang_AC=1;Zhuang_AF=0.00225
chr4	70585334	.	A	<INS:ME:LINE1>	.	PASS	SVTYPE=INS:ME:LINE1;END=70585350;SVLEN=6006;AN=20618;AC=1;AF=4.850130953535746e-05;N_HET=1;N_HOMALT=0;N_HOMREF=10308;FREQ_HET=9.700261907071492e-05;FREQ_HOMALT=0.0;FREQ_HOMREF=0.9999029973809292;N_BI_GENOS=10309;Central_Han_AN=2128;Central_Han_AC=1;Centra
chr4	71229839	.	C	<INS:ME:LINE1>	.	PASS	SVTYPE=INS:ME:LINE1;END=71229853;SVLEN=761;AN=20618;AC=2;AF=9.700261907071492e-05;N_HET=2;N_HOMALT=0;N_HOMREF=10307;FREQ_HET=0.00019400523814142983;FREQ_HOMALT=0.0;FREQ_HOMREF=0.9998059947618586;N_BI_GENOS=10309;Lingnan_Han_AN=1888;Lingnan_Han_AC=1;Lingna
chr4	71246734	.	T	<INS:ME:LINE1>	.	PASS	SVTYPE=INS:ME:LINE1;END=71246741;SVLEN=246;AN=20618;AC=2;AF=9.700261907071492e-05;N_HET=2;N_HOMALT=0;N_HOMREF=10307;FREQ_HET=0.00019400523814142983;FREQ_HOMALT=0.0;FREQ_HOMREF=0.9998059947618586;N_BI_GENOS=10309;South_Han_AN=1496;South_Han_AC=1;South_Han_
chr4	71322155	.	A	<INS:ME:LINE1>	.	PASS	SVTYPE=INS:ME:LINE1;END=71322169;SVLEN=300;AN=20618;AC=1;AF=4.850130953535746e-05;N_HET=1;N_HOMALT=0;N_HOMREF=10308;FREQ_HET=9.700261907071492e-05;FREQ_HOMALT=0.0;FREQ_HOMREF=0.9999029973809292;N_BI_GENOS=10309;East_Han_AN=6554;East_Han_AC=1;East_Han_AF=0
chr4	71400283	.	A	<INS:ME:LINE1>	.	PASS	SVTYPE=INS:ME:LINE1;END=71400290;SVLEN=924;AN=20618;AC=1;AF=4.850130953535746e-05;N_HET=1;N_HOMALT=0;N_HOMREF=10308;FREQ_HET=9.700261907071492e-05;FREQ_HOMALT=0.0;FREQ_HOMREF=0.9999029973809292;N_BI_GENOS=10309;Yi_AN=422;Yi_AC=1;Yi_AF=0.002369668246445498
chr4	71473714	.	A	<INS:ME:LINE1>	.	PASS	SVTYPE=INS:ME:LINE1;END=71473714;SVLEN=365;AN=20618;AC=1;AF=4.850130953535746e-05;N_HET=1;N_HOMALT=0;N_HOMREF=10308;FREQ_HET=9.700261907071492e-05;FREQ_HOMALT=0.0;FREQ_HOMREF=0.9999029973809292;N_BI_GENOS=10309;North_Han_AN=2272;North_Han_AC=1;North_Han_A
chr4	71512436	.	T	<INS:ME:LINE1>	.	PASS	SVTYPE=INS:ME:LINE1;END=71512479;SVLEN=949;AN=20618;AC=1;AF=4.850130953535746e-05;N_HET=1;N_HOMALT=0;N_HOMREF=10308;FREQ_HET=9.700261907071492e-05;FREQ_HOMALT=0.0;FREQ_HOMREF=0.9999029973809292;N_BI_GENOS=10309;East_Han_AN=6554;East_Han_AC=1;East_Han_AF=0
chr4	71713730	.	G	<INS:ME:LINE1>	.	PASS	SVTYPE=INS:ME:LINE1;END=71713734;SVLEN=562;AN=20618;AC=1;AF=4.850130953535746e-05;N_HET=1;N_HOMALT=0;N_HOMREF=10308;FREQ_HET=9.700261907071492e-05;FREQ_HOMALT=0.0;FREQ_HOMREF=0.9999029973809292;N_BI_GENOS=10309;Southeast_Han_AN=1908;Southeast_Han_AC=1;Sou
chr4	72161537	.	T	<INS:ME:LINE1>	.	PASS	SVTYPE=INS:ME:LINE1;END=72161537;SVLEN=180;AN=20618;AC=1;AF=4.850130953535746e-05;N_HET=1;N_HOMALT=0;N_HOMREF=10308;FREQ_HET=9.700261907071492e-05;FREQ_HOMALT=0.0;FREQ_HOMREF=0.9999029973809292;N_BI_GENOS=10309;East_Han_AN=6554;East_Han_AC=1;East_Han_AF=0
chr4	72629209	.	A	<INS:ME:LINE1>	.	PASS	SVTYPE=INS:ME:LINE1;END=72629222;SVLEN=2323;AN=20618;AC=1;AF=4.850130953535746e-05;N_HET=1;N_HOMALT=0;N_HOMREF=10308;FREQ_HET=9.700261907071492e-05;FREQ_HOMALT=0.0;FREQ_HOMREF=0.9999029973809292;N_BI_GENOS=10309;Southeast_Han_AN=1908;Southeast_Han_AC=1;So
chr4	73158209	.	A	<INS:ME:LINE1>	.	PASS	SVTYPE=INS:ME:LINE1;END=73158209;SVLEN=20;AN=20618;AC=1;AF=4.850130953535746e-05;N_HET=1;N_HOMALT=0;N_HOMREF=10308;FREQ_HET=9.700261907071492e-05;FREQ_HOMALT=0.0;FREQ_HOMREF=0.9999029973809292;N_BI_GENOS=10309;East_Han_AN=6554;East_Han_AC=1;East_Han_AF=0.
chr4	73377970	.	A	<INS:ME:LINE1>	.	PASS	SVTYPE=INS:ME:LINE1;END=73377988;SVLEN=2169;AN=20618;AC=6;AF=0.0002910078572121447;N_HET=6;N_HOMALT=0;N_HOMREF=10303;FREQ_HET=0.0005820157144242894;FREQ_HOMALT=0.0;FREQ_HOMREF=0.9994179842855757;N_BI_GENOS=10309;South_Han_AN=1496;South_Han_AC=1;South_Han_
chr4	74185151	.	A	<INS:ME:LINE1>	.	PASS	SVTYPE=INS:ME:LINE1;END=74185170;SVLEN=921;AN=20618;AC=10;AF=0.0004850130953535 7455;N_HET=2;N_HOMALT=4;N_HOMREF=10303;FREQ_HET=0.00019400523814142983;FREQ_HOMALT=0.00038801047628285966;FREQ_HOMREF=0.9994179842855757;N_BI_GENOS=10309;East_Han_AN=6554;East_
chr4	74198605	.	T	<INS:ME:LINE1>	.	PASS	SVTYPE=INS:ME:LINE1;END=74198606;SVLEN=1199;AN=20618;AC=1;AF=4.850130953535746e-05;N_HET=1;N_HOMALT=0;N_HOMREF=10308;FREQ_HET=9.700261907071492e-05;FREQ_HOMALT=0.0;FREQ_HOMREF=0.9999029973809292;N_BI_GENOS=10309;South_Han_AN=1496;South_Han_AC=1;South_Han_
chr4	74294084	.	A	<INS:ME:LINE1>	.	PASS	SVTYPE=INS:ME:LINE1;END=74294097;SVLEN=95;AN=20618;AC=1;AF=4.850130953535746e-05;N_HET=1;N_HOMALT=0;N_HOMREF=10308;FREQ_HET=9.700261907071492e-05;FREQ_HOMALT=0.0;FREQ_HOMREF=0.9999029973809292;N_BI_GENOS=10309;North_Han_AN=2272;North_Han_AC=1;North_Han_AF
chr4	74329673	.	A	<INS:ME:LINE1>	.	PASS	SVTYPE=INS:ME:LINE1;END=74329690;SVLEN=1125;AN=20618;AC=1;AF=4.850130953535746e-05;N_HET=1;N_HOMALT=0;N_HOMREF=10308;FREQ_HET=9.700261907071492e-05;FREQ_HOMALT=0.0;FREQ_HOMREF=0.9999029973809292;N_BI_GENOS=10309;North_Han_AN=2272;North_Han_AC=1;North_Han_
chr4	74407346	.	G	<INS:ME:LINE1>	.	PASS	SVTYPE=INS:ME:LINE1;END=74407362;SVLEN=204;AN=20618;AC=1;AF=4.850130953535746e-05;N_HET=1;N_HOMALT=0;N_HOMREF=10308;FREQ_HET=9.700261907071492e-05;FREQ_HOMALT=0.0;FREQ_HOMREF=0.9999029973809292;N_BI_GENOS=10309;Lingnan_Han_AN=1888;Lingnan_Han_AC=1;Lingnan
chr4	74627866	.	A	<INS:ME:LINE1>	.	PASS	SVTYPE=INS:ME:LINE1;END=74627880;SVLEN=5998;AN=20618;AC=3;AF=0.00014550392860607236;N_HET=1;N_HOMALT=1;N_HOMREF=10307;FREQ_HET=9.700261907071492e-05;FREQ_HOMALT=9.700261907071492e-05;FREQ_HOMREF=0.9998059947618586;N_BI_GENOS=10309;South_Han_AN=1496;South_
chr4	74716282	.	T	<INS:ME:LINE1>	.	PASS	SVTYPE=INS:ME:LINE1;END=74716284;SVLEN=0;AN=20618;AC=4;AF=0.00019400523814142983;N_HET=4;N_HOMALT=0;N_HOMREF=10305;FREQ_HET=0.00038801047628285966;FREQ_HOMALT=0.0;FREQ_HOMREF=0.9996119895237171;N_BI_GENOS=10309;Lingnan_Han_AN=1888;Lingnan_Han_AC=1;Lingnan
chr4	74992485	.	A	<INS:ME:LINE1>	.	PASS	SVTYPE=INS:ME:LINE1;END=74992485;SVLEN=0;AN=20618;AC=33;AF=0.0016005432146667 96;N_HET=31;N_HOMALT=1;N_HOMREF=10277;FREQ_HET=0.0030070811911921624;FREQ_HOMALT=9.700261907071492e-05;FREQ_HOMREF=0.9968959161897372;N_BI_GENOS=10309;South_Han_AN=1496;South_Han
chr4	75159569	.	A	<INS:ME:LINE1>	.	PASS	SVTYPE=INS:ME:LINE1;END=75159583;SVLEN=878;AN=20618;AC=1;AF=4.850130953535746e-05;N_HET=1;N_HOMALT=0;N_HOMREF=10308;FREQ_HET=9.700261907071492e-05;FREQ_HOMALT=0.0;FREQ_HOMREF=0.9999029973809292;N_BI_GENOS=10309;South_Han_AN=1496;South_Han_AC=1;South_Han_A
chr4	75199352	.	C	<INS:ME:LINE1>	.	PASS	SVTYPE=INS:ME:LINE1;END=75199365;SVLEN=6002;AN=20618;AC=1;AF=4.850130953535746e-05;N_HET=1;N_HOMALT=0;N_HOMREF=10308;FREQ_HET=9.700261907071492e-05;FREQ_HOMALT=0.0;FREQ_HOMREF=0.9999029973809292;N_BI_GENOS=10309;East_Han_AN=6554;East_Han_AC=1;East_Han_AF=
chr4	75483217	.	A	<INS:ME:LINE1>	.	PASS	SVTYPE=INS:ME:LINE1;END=75483231;SVLEN=5983;AN=20618;AC=2;AF=9.700261907071492e-05;N_HET=2;N_HOMALT=0;N_HOMREF=10307;FREQ_HET=0.00019400523814142983;FREQ_HOMALT=0.0;FREQ_HOMREF=0.9998059947618586;N_BI_GENOS=10309;Northwest_Han_AN=1296;Northwest_Han_AC=1;N
chr4	75877041	.	A	<INS:ME:LINE1>	.	PASS	SVTYPE=INS:ME:LINE1;END=75877041;SVLEN=0;AN=20618;AC=3;AF=0.00014550392860607236;N_HET=1;N_HOMALT=1;N_HOMREF=10307;FREQ_HET=9.700261907071492e-05;FREQ_HOMALT=9.700261907071492e-05;FREQ_HOMREF=0.9998059947618586;N_BI_GENOS=10309;Northwest_Han_AN=1296;North
chr4	76014519	.	A	<INS:ME:LINE1>	.	PASS	SVTYPE=INS:ME:LINE1;END=76014519;SVLEN=5997;AN=20618;AC=1;AF=4.850130953535746e-05;N_HET=1;N_HOMALT=0;N_HOMREF=10308;FREQ_HET=9.700261907071492e-05;FREQ_HOMALT=0.0;FREQ_HOMREF=0.9999029973809292;N_BI_GENOS=10309;Southeast_Han_AN=1908;Southeast_Han_AC=1;So
chr4	76260535	.	C	<INS:ME:LINE1>	.	PASS	SVTYPE=INS:ME:LINE1;END=76260535;SVLEN=388;AN=20618;AC=1;AF=4.850130953535746e-05;N_HET=1;N_HOMALT=0;N_HOMREF=10308;FREQ_HET=9.700261907071492e-05;FREQ_HOMALT=0.0;FREQ_HOMREF=0.9999029973809292;N_BI_GENOS=10309;East_Han_AN=6554;East_Han_AC=1;East_Han_AF=0
chr4	76788511	.	T	<INS:ME:LINE1>	.	PASS	SVTYPE=INS:ME:LINE1;END=76788512;SVLEN=907;AN=20618;AC=24;AF=0.0011640314288485789;N_HET=20;N_HOMALT=2;N_HOMREF=10287;FREQ_HET=0.0019400523814142982;FREQ_HOMALT=0.00019400523814142983;FREQ_HOMREF=0.9978659423804442;N_BI_GENOS=10309;South_Han_AN=1496;South
chr4	77069063	.	A	<INS:ME:LINE1>	.	PASS	SVTYPE=INS:ME:LINE1;END=77069080;SVLEN=6005;AN=20618;AC=1;AF=4.850130953535746e-05;N_HET=1;N_HOMALT=0;N_HOMREF=10308;FREQ_HET=9.700261907071492e-05;FREQ_HOMALT=0.0;FREQ_HOMREF=0.9999029973809292;N_BI_GENOS=10309;Lingnan_Han_AN=1888;Lingnan_Han_AC=1;Lingna
chr4	77477433	.	T	<INS:ME:LINE1>	.	PASS	SVTYPE=INS:ME:LINE1;END=77477433;SVLEN=525;AN=20618;AC=1;AF=4.850130953535746e-05;N_HET=1;N_HOMALT=0;N_HOMREF=10308;FREQ_HET=9.700261907071492e-05;FREQ_HOMALT=0.0;FREQ_HOMREF=0.9999029973809292;N_BI_GENOS=10309;East_Han_AN=6554;East_Han_AC=1;East_Han_AF=0
chr4	77576719	.	G	<INS:ME:LINE1>	.	PASS	SVTYPE=INS:ME:LINE1;END=77576733;SVLEN=5993;AN=20618;AC=35;AF=0.0016975458337375108;N_HET=35;N_HOMALT=0;N_HOMREF=10274;FREQ_HET=0.003395091667475 0217;FREQ_HOMALT=0.0;FREQ_HOMREF=0.996604908332525;N_BI_GENOS=10309;South_Han_AN=1496;South_Han_AC=6;South_Han
chr4	77615673	.	A	<INS:ME:LINE1>	.	PASS	SVTYPE=INS:ME:LINE1;END=77615684;SVLEN=6014;AN=20618;AC=1;AF=4.850130953535746e-05;N_HET=1;N_HOMALT=0;N_HOMREF=10308;FREQ_HET=9.700261907071492e-05;FREQ_HOMALT=0.0;FREQ_HOMREF=0.9999029973809292;N_BI_GENOS=10309;Tibetan_AN=414;Tibetan_AC=1;Tibetan_AF=0.00
chr4	78439803	.	T	<INS:ME:LINE1>	.	PASS	SVTYPE=INS:ME:LINE1;END=78439818;SVLEN=255;AN=20618;AC=1;AF=4.850130953535746e-05;N_HET=1;N_HOMALT=0;N_HOMREF=10308;FREQ_HET=9.700261907071492e-05;FREQ_HOMALT=0.0;FREQ_HOMREF=0.9999029973809292;N_BI_GENOS=10309;Northwest_Han_AN=1296;Northwest_Han_AC=1;Nor
chr4	78600927	.	A	<INS:ME:LINE1>	.	PASS	SVTYPE=INS:ME:LINE1;END=78600946;SVLEN=5966;AN=20618;AC=1;AF=4.850130953535746e-05;N_HET=1;N_HOMALT=0;N_HOMREF=10308;FREQ_HET=9.700261907071492e-05;FREQ_HOMALT=0.0;FREQ_HOMREF=0.9999029973809292;N_BI_GENOS=10309;South_Han_AN=1496;South_Han_AC=1;South_Han_
chr4	79021713	.	T	<INS:ME:LINE1>	.	PASS	SVTYPE=INS:ME:LINE1;END=79021728;SVLEN=5997;AN=20618;AC=1;AF=4.850130953535746e-05;N_HET=1;N_HOMALT=0;N_HOMREF=10308;FREQ_HET=9.700261907071492e-05;FREQ_HOMALT=0.0;FREQ_HOMREF=0.9999029973809292;N_BI_GENOS=10309;East_Han_AN=6554;East_Han_AC=1;East_Han_AF=
chr4	79185607	.	A	<INS:ME:LINE1>	.	PASS	SVTYPE=INS:ME:LINE1;END=79185620;SVLEN=617;AN=20618;AC=1;AF=4.850130953535746e-05;N_HET=1;N_HOMALT=0;N_HOMREF=10308;FREQ_HET=9.700261907071492e-05;FREQ_HOMALT=0.0;FREQ_HOMREF=0.9999029973809292;N_BI_GENOS=10309;North_Han_AN=2272;North_Han_AC=1;North_Han_A
chr4	79378041	.	C	<INS:ME:LINE1>	.	PASS	SVTYPE=INS:ME:LINE1;END=79378041;SVLEN=5996;AN=20618;AC=1;AF=4.850130953535746e-05;N_HET=1;N_HOMALT=0;N_HOMREF=10308;FREQ_HET=9.700261907071492e-05;FREQ_HOMALT=0.0;FREQ_HOMREF=0.9999029973809292;N_BI_GENOS=10309;Mongolian_AN=454;Mongolian_AC=1;Mongolian_A
chr4	79419523	.	T	<INS:ME:LINE1>	.	PASS	SVTYPE=INS:ME:LINE1;END=79419549;SVLEN=1621;AN=20618;AC=1;AF=4.850130953535746e-05;N_HET=1;N_HOMALT=0;N_HOMREF=10308;FREQ_HET=9.700261907071492e-05;FREQ_HOMALT=0.0;FREQ_HOMREF=0.9999029973809292;N_BI_GENOS=10309;East_Han_AN=6554;East_Han_AC=1;East_Han_AF=
chr4	79422490	.	A	<INS:ME:LINE1>	.	PASS	SVTYPE=INS:ME:LINE1;END=79422503;SVLEN=5999;AN=20618;AC=5;AF=0.00024250654767678728;N_HET=5;N_HOMALT=0;N_HOMREF=10304;FREQ_HET=0.0004850130953535 7455;FREQ_HOMALT=0.0;FREQ_HOMREF=0.9995149869046465;N_BI_GENOS=10309;South_Han_AN=1496;South_Han_AC=1;South_Ha
chr4	79426649	.	C	<INS:ME:LINE1>	.	PASS	SVTYPE=INS:ME:LINE1;END=79426665;SVLEN=2663;AN=20618;AC=1;AF=4.850130953535746e-05;N_HET=1;N_HOMALT=0;N_HOMREF=10308;FREQ_HET=9.700261907071492e-05;FREQ_HOMALT=0.0;FREQ_HOMREF=0.9999029973809292;N_BI_GENOS=10309;Northwest_Han_AN=1296;Northwest_Han_AC=1;No
chr4	79433570	.	A	<INS:ME:LINE1>	.	PASS	SVTYPE=INS:ME:LINE1;END=79433587;SVLEN=5997;AN=20618;AC=1;AF=4.850130953535746e-05;N_HET=1;N_HOMALT=0;N_HOMREF=10308;FREQ_HET=9.700261907071492e-05;FREQ_HOMALT=0.0;FREQ_HOMREF=0.9999029973809292;N_BI_GENOS=10309;East_Han_AN=6554;East_Han_AC=1;East_Han_AF=
chr4	79847385	.	A	<INS:ME:LINE1>	.	PASS	SVTYPE=INS:ME:LINE1;END=79847387;SVLEN=601;AN=20618;AC=4798;AF=0.23270928315064507;N_HET=2486;N_HOMALT=1156;N_HOMREF=6667;FREQ_HET=0.24114851100979726;FREQ_HOMALT=0.11213502764574644;FREQ_HOMREF=0.6467164613444563;N_BI_GENOS=10309;South_Han_AN=1496;South_
chr4	79884209	.	A	<INS:ME:LINE1>	.	PASS	SVTYPE=INS:ME:LINE1;END=79884225;SVLEN=956;AN=20618;AC=1;AF=4.850130953535746e-05;N_HET=1;N_HOMALT=0;N_HOMREF=10308;FREQ_HET=9.700261907071492e-05;FREQ_HOMALT=0.0;FREQ_HOMREF=0.9999029973809292;N_BI_GENOS=10309;East_Han_AN=6554;East_Han_AC=1;East_Han_AF=0
chr4	80027241	.	A	<INS:ME:LINE1>	.	PASS	SVTYPE=INS:ME:LINE1;END=80027257;SVLEN=6003;AN=20618;AC=1;AF=4.850130953535746e-05;N_HET=1;N_HOMALT=0;N_HOMREF=10308;FREQ_HET=9.700261907071492e-05;FREQ_HOMALT=0.0;FREQ_HOMREF=0.9999029973809292;N_BI_GENOS=10309;East_Han_AN=6554;East_Han_AC=1;East_Han_AF=
chr4	80286364	.	A	<INS:ME:LINE1>	.	PASS	SVTYPE=INS:ME:LINE1;END=80286376;SVLEN=6002;AN=20618;AC=2;AF=9.700261907071492e-05;N_HET=2;N_HOMALT=0;N_HOMREF=10307;FREQ_HET=0.00019400523814142983;FREQ_HOMALT=0.0;FREQ_HOMREF=0.9998059947618586;N_BI_GENOS=10309;East_Han_AN=6554;East_Han_AC=1;East_Han_AF
chr4	80622563	.	T	<INS:ME:LINE1>	.	PASS	SVTYPE=INS:ME:LINE1;END=80622564;SVLEN=240;AN=20618;AC=1;AF=4.850130953535746e-05;N_HET=1;N_HOMALT=0;N_HOMREF=10308;FREQ_HET=9.700261907071492e-05;FREQ_HOMALT=0.0;FREQ_HOMREF=0.9999029973809292;N_BI_GENOS=10309;North_Han_AN=2272;North_Han_AC=1;North_Han_A
chr4	80745635	.	A	<INS:ME:LINE1>	.	PASS	SVTYPE=INS:ME:LINE1;END=80745643;SVLEN=484;AN=20618;AC=2;AF=9.700261907071492e-05;N_HET=2;N_HOMALT=0;N_HOMREF=10307;FREQ_HET=0.00019400523814142983;FREQ_HOMALT=0.0;FREQ_HOMREF=0.9998059947618586;N_BI_GENOS=10309;Southeast_Han_AN=1908;Southeast_Han_AC=1;So
chr4	80782276	.	A	<INS:ME:LINE1>	.	PASS	SVTYPE=INS:ME:LINE1;END=80782285;SVLEN=626;AN=20618;AC=34;AF=0.0016490445242021534;N_HET=32;N_HOMALT=1;N_HOMREF=10276;FREQ_HET=0.0031040838102628773;FREQ_HOMALT=9.700261907071492e-05;FREQ_HOMREF=0.9967989135706664;N_BI_GENOS=10309;South_Han_AN=1496;South_
chr4	81262847	.	G	<INS:ME:LINE1>	.	PASS	SVTYPE=INS:ME:LINE1;END=81262852;SVLEN=6066;AN=20618;AC=1;AF=4.850130953535746e-05;N_HET=1;N_HOMALT=0;N_HOMREF=10308;FREQ_HET=9.700261907071492e-05;FREQ_HOMALT=0.0;FREQ_HOMREF=0.9999029973809292;N_BI_GENOS=10309;North_Han_AN=2272;North_Han_AC=1;North_Han_
chr4	81276386	.	A	<INS:ME:LINE1>	.	PASS	SVTYPE=INS:ME:LINE1;END=81276402;SVLEN=1195;AN=20618;AC=1;AF=4.850130953535746e-05;N_HET=1;N_HOMALT=0;N_HOMREF=10308;FREQ_HET=9.700261907071492e-05;FREQ_HOMALT=0.0;FREQ_HOMREF=0.9999029973809292;N_BI_GENOS=10309;Southeast_Han_AN=1908;Southeast_Han_AC=1;So
chr4	81485279	.	T	<INS:ME:LINE1>	.	PASS	SVTYPE=INS:ME:LINE1;END=81485292;SVLEN=6007;AN=20618;AC=1;AF=4.850130953535746e-05;N_HET=1;N_HOMALT=0;N_HOMREF=10308;FREQ_HET=9.700261907071492e-05;FREQ_HOMALT=0.0;FREQ_HOMREF=0.9999029973809292;N_BI_GENOS=10309;South_Han_AN=1496;South_Han_AC=1;South_Han_
chr4	81615622	.	T	<INS:ME:LINE1>	.	PASS	SVTYPE=INS:ME:LINE1;END=81615633;SVLEN=5997;AN=20618;AC=3;AF=0.00014550392860607236;N_HET=3;N_HOMALT=0;N_HOMREF=10306;FREQ_HET=0.0002910078572121447;FREQ_HOMALT=0.0;FREQ_HOMREF=0.9997089921427879;N_BI_GENOS=10309;East_Han_AN=6554;East_Han_AC=3;East_Han_AF
chr4	81616037	.	A	<INS:ME:LINE1>	.	PASS	SVTYPE=INS:ME:LINE1;END=81616053;SVLEN=5998;AN=20618;AC=1;AF=4.850130953535746e-05;N_HET=1;N_HOMALT=0;N_HOMREF=10308;FREQ_HET=9.700261907071492e-05;FREQ_HOMALT=0.0;FREQ_HOMREF=0.9999029973809292;N_BI_GENOS=10309;East_Han_AN=6554;East_Han_AC=1;East_Han_AF=
chr4	81688238	.	A	<INS:ME:LINE1>	.	PASS	SVTYPE=INS:ME:LINE1;END=81688259;SVLEN=1713;AN=20618;AC=1;AF=4.850130953535746e-05;N_HET=1;N_HOMALT=0;N_HOMREF=10308;FREQ_HET=9.700261907071492e-05;FREQ_HOMALT=0.0;FREQ_HOMREF=0.9999029973809292;N_BI_GENOS=10309;Lingnan_Han_AN=1888;Lingnan_Han_AC=1;Lingna
chr4	81767844	.	T	<INS:ME:LINE1>	.	PASS	SVTYPE=INS:ME:LINE1;END=81767858;SVLEN=6005;AN=20618;AC=1;AF=4.850130953535746e-05;N_HET=1;N_HOMALT=0;N_HOMREF=10308;FREQ_HET=9.700261907071492e-05;FREQ_HOMALT=0.0;FREQ_HOMREF=0.9999029973809292;N_BI_GENOS=10309;Southeast_Han_AN=1908;Southeast_Han_AC=1;So
chr4	81799230	.	A	<INS:ME:LINE1>	.	PASS	SVTYPE=INS:ME:LINE1;END=81799244;SVLEN=477;AN=20618;AC=1;AF=4.850130953535746e-05;N_HET=1;N_HOMALT=0;N_HOMREF=10308;FREQ_HET=9.700261907071492e-05;FREQ_HOMALT=0.0;FREQ_HOMREF=0.9999029973809292;N_BI_GENOS=10309;Central_Han_AN=2128;Central_Han_AC=1;Central
chr4	81977499	.	A	<INS:ME:LINE1>	.	PASS	SVTYPE=INS:ME:LINE1;END=81977516;SVLEN=727;AN=20618;AC=1;AF=4.850130953535746e-05;N_HET=1;N_HOMALT=0;N_HOMREF=10308;FREQ_HET=9.700261907071492e-05;FREQ_HOMALT=0.0;FREQ_HOMREF=0.9999029973809292;N_BI_GENOS=10309;Mongolian_AN=454;Mongolian_AC=1;Mongolian_AF
chr4	82263572	.	A	<INS:ME:LINE1>	.	PASS	SVTYPE=INS:ME:LINE1;END=82263586;SVLEN=5999;AN=20618;AC=1;AF=4.850130953535746e-05;N_HET=1;N_HOMALT=0;N_HOMREF=10308;FREQ_HET=9.700261907071492e-05;FREQ_HOMALT=0.0;FREQ_HOMREF=0.9999029973809292;N_BI_GENOS=10309;Hui_AN=444;Hui_AC=1;Hui_AF=0.00225225225225
chr4	82860836	.	C	<INS:ME:LINE1>	.	PASS	SVTYPE=INS:ME:LINE1;END=82860853;SVLEN=5998;AN=20618;AC=1;AF=4.850130953535746e-05;N_HET=1;N_HOMALT=0;N_HOMREF=10308;FREQ_HET=9.700261907071492e-05;FREQ_HOMALT=0.0;FREQ_HOMREF=0.9999029973809292;N_BI_GENOS=10309;South_Han_AN=1496;South_Han_AC=1;South_Han_
chr4	83148072	.	A	<INS:ME:LINE1>	.	PASS	SVTYPE=INS:ME:LINE1;END=83148086;SVLEN=449;AN=20618;AC=1;AF=4.850130953535746e-05;N_HET=1;N_HOMALT=0;N_HOMREF=10308;FREQ_HET=9.700261907071492e-05;FREQ_HOMALT=0.0;FREQ_HOMREF=0.9999029973809292;N_BI_GENOS=10309;Manchu_AN=416;Manchu_AC=1;Manchu_AF=0.002403
chr4	83445999	.	G	<INS:ME:LINE1>	.	PASS	SVTYPE=INS:ME:LINE1;END=83446010;SVLEN=6048;AN=20618;AC=1;AF=4.850130953535746e-05;N_HET=1;N_HOMALT=0;N_HOMREF=10308;FREQ_HET=9.700261907071492e-05;FREQ_HOMALT=0.0;FREQ_HOMREF=0.9999029973809292;N_BI_GENOS=10309;Central_Han_AN=2128;Central_Han_AC=1;Centra
chr4	83913828	.	T	<INS:ME:LINE1>	.	PASS	SVTYPE=INS:ME:LINE1;END=83913844;SVLEN=245;AN=20618;AC=14;AF=0.0006790183334950044;N_HET=14;N_HOMALT=0;N_HOMREF=10295;FREQ_HET=0.0013580366669900088;FREQ_HOMALT=0.0;FREQ_HOMREF=0.99864196333301;N_BI_GENOS=10309;North_Han_AN=2272;North_Han_AC=2;North_Han_A
chr4	83976945	.	A	<INS:ME:LINE1>	.	PASS	SVTYPE=INS:ME:LINE1;END=83976962;SVLEN=4026;AN=20618;AC=1;AF=4.850130953535746e-05;N_HET=1;N_HOMALT=0;N_HOMREF=10308;FREQ_HET=9.700261907071492e-05;FREQ_HOMALT=0.0;FREQ_HOMREF=0.9999029973809292;N_BI_GENOS=10309;South_Han_AN=1496;South_Han_AC=1;South_Han_
chr4	84187055	.	T	<INS:ME:LINE1>	.	PASS	SVTYPE=INS:ME:LINE1;END=84187067;SVLEN=706;AN=20618;AC=26;AF=0.0012610340479192938;N_HET=26;N_HOMALT=0;N_HOMREF=10283;FREQ_HET=0.0025220680958385876;FREQ_HOMALT=0.0;FREQ_HOMREF=0.9974779319041615;N_BI_GENOS=10309;South_Han_AN=1496;South_Han_AC=2;South_Han
chr4	84256998	.	C	<INS:ME:LINE1>	.	PASS	SVTYPE=INS:ME:LINE1;END=84257013;SVLEN=6043;AN=20618;AC=1;AF=4.850130953535746e-05;N_HET=1;N_HOMALT=0;N_HOMREF=10308;FREQ_HET=9.700261907071492e-05;FREQ_HOMALT=0.0;FREQ_HOMREF=0.9999029973809292;N_BI_GENOS=10309;Central_Han_AN=2128;Central_Han_AC=1;Centra
chr4	84336682	.	A	<INS:ME:LINE1>	.	PASS	SVTYPE=INS:ME:LINE1;END=84336682;SVLEN=3993;AN=20618;AC=1;AF=4.850130953535746e-05;N_HET=1;N_HOMALT=0;N_HOMREF=10308;FREQ_HET=9.700261907071492e-05;FREQ_HOMALT=0.0;FREQ_HOMREF=0.9999029973809292;N_BI_GENOS=10309;East_Han_AN=6554;East_Han_AC=1;East_Han_AF=
chr4	84396305	.	T	<INS:ME:LINE1>	.	PASS	SVTYPE=INS:ME:LINE1;END=84396322;SVLEN=219;AN=20618;AC=734;AF=0.035599961198952375;N_HET=514;N_HOMALT=110;N_HOMREF=9685;FREQ_HET=0.049859346202347464;FREQ_HOMALT=0.01067028809777864;FREQ_HOMREF=0.9394703656998739;N_BI_GENOS=10309;South_Han_AN=1496;South_H
chr4	84634910	.	A	<INS:ME:LINE1>	.	PASS	SVTYPE=INS:ME:LINE1;END=84634927;SVLEN=339;AN=20618;AC=1;AF=4.850130953535746e-05;N_HET=1;N_HOMALT=0;N_HOMREF=10308;FREQ_HET=9.700261907071492e-05;FREQ_HOMALT=0.0;FREQ_HOMREF=0.9999029973809292;N_BI_GENOS=10309;Central_Han_AN=2128;Central_Han_AC=1;Central
chr4	85040905	.	G	<INS:ME:LINE1>	.	PASS	SVTYPE=INS:ME:LINE1;END=85040917;SVLEN=1652;AN=20618;AC=1;AF=4.850130953535746e-05;N_HET=1;N_HOMALT=0;N_HOMREF=10308;FREQ_HET=9.700261907071492e-05;FREQ_HOMALT=0.0;FREQ_HOMREF=0.9999029973809292;N_BI_GENOS=10309;Mongolian_AN=454;Mongolian_AC=1;Mongolian_A
chr4	85275433	.	A	<INS:ME:LINE1>	.	PASS	SVTYPE=INS:ME:LINE1;END=85275441;SVLEN=514;AN=20618;AC=1;AF=4.850130953535746e-05;N_HET=1;N_HOMALT=0;N_HOMREF=10308;FREQ_HET=9.700261907071492e-05;FREQ_HOMALT=0.0;FREQ_HOMREF=0.9999029973809292;N_BI_GENOS=10309;Tibetan_AN=414;Tibetan_AC=1;Tibetan_AF=0.002
chr4	85301427	.	T	<INS:ME:LINE1>	.	PASS	SVTYPE=INS:ME:LINE1;END=85301443;SVLEN=6004;AN=20618;AC=1;AF=4.850130953535746e-05;N_HET=1;N_HOMALT=0;N_HOMREF=10308;FREQ_HET=9.700261907071492e-05;FREQ_HOMALT=0.0;FREQ_HOMREF=0.9999029973809292;N_BI_GENOS=10309;Central_Han_AN=2128;Central_Han_AC=1;Centra
chr4	85424360	.	A	<INS:ME:LINE1>	.	PASS	SVTYPE=INS:ME:LINE1;END=85424360;SVLEN=6018;AN=20618;AC=1;AF=4.850130953535746e-05;N_HET=1;N_HOMALT=0;N_HOMREF=10308;FREQ_HET=9.700261907071492e-05;FREQ_HOMALT=0.0;FREQ_HOMREF=0.9999029973809292;N_BI_GENOS=10309;Lingnan_Han_AN=1888;Lingnan_Han_AC=1;Lingna
chr4	85439523	.	A	<INS:ME:LINE1>	.	PASS	SVTYPE=INS:ME:LINE1;END=85439523;SVLEN=955;AN=20618;AC=1;AF=4.850130953535746e-05;N_HET=1;N_HOMALT=0;N_HOMREF=10308;FREQ_HET=9.700261907071492e-05;FREQ_HOMALT=0.0;FREQ_HOMREF=0.9999029973809292;N_BI_GENOS=10309;East_Han_AN=6554;East_Han_AC=1;East_Han_AF=0
chr4	85558292	.	C	<INS:ME:LINE1>	.	PASS	SVTYPE=INS:ME:LINE1;END=85558301;SVLEN=871;AN=20618;AC=2;AF=9.700261907071492e-05;N_HET=2;N_HOMALT=0;N_HOMREF=10307;FREQ_HET=0.00019400523814142983;FREQ_HOMALT=0.0;FREQ_HOMREF=0.9998059947618586;N_BI_GENOS=10309;East_Han_AN=6554;East_Han_AC=2;East_Han_AF=
chr4	85583925	.	A	<INS:ME:LINE1>	.	PASS	SVTYPE=INS:ME:LINE1;END=85583926;SVLEN=243;AN=20618;AC=6;AF=0.0002910078572121447;N_HET=0;N_HOMALT=3;N_HOMREF=10306;FREQ_HET=0.0;FREQ_HOMALT=0.0002910078572121447;FREQ_HOMREF=0.9997089921427879;N_BI_GENOS=10309;Lingnan_Han_AN=1888;Lingnan_Han_AC=2;Lingnan
chr4	85686796	.	A	<INS:ME:LINE1>	.	PASS	SVTYPE=INS:ME:LINE1;END=85686803;SVLEN=410;AN=20618;AC=19;AF=0.0009215248811717916;N_HET=17;N_HOMALT=1;N_HOMREF=10291;FREQ_HET=0.0016490445242021534;FREQ_HOMALT=9.700261907071492e-05;FREQ_HOMREF=0.9982539528567271;N_BI_GENOS=10309;Northwest_Han_AN=1296;No
chr4	86156525	.	A	<INS:ME:LINE1>	.	PASS	SVTYPE=INS:ME:LINE1;END=86156525;SVLEN=987;AN=20618;AC=1;AF=4.850130953535746e-05;N_HET=1;N_HOMALT=0;N_HOMREF=10308;FREQ_HET=9.700261907071492e-05;FREQ_HOMALT=0.0;FREQ_HOMREF=0.9999029973809292;N_BI_GENOS=10309;East_Han_AN=6554;East_Han_AC=1;East_Han_AF=0
chr4	86359289	.	G	<INS:ME:LINE1>	.	PASS	SVTYPE=INS:ME:LINE1;END=86359289;SVLEN=0;AN=20618;AC=6;AF=0.0002910078572121447;N_HET=0;N_HOMALT=3;N_HOMREF=10306;FREQ_HET=0.0;FREQ_HOMALT=0.0002910078572121447;FREQ_HOMREF=0.9997089921427879;N_BI_GENOS=10309;East_Han_AN=6554;East_Han_AC=4;East_Han_AF=0.0
chr4	86369787	.	A	<INS:ME:LINE1>	.	PASS	SVTYPE=INS:ME:LINE1;END=86369802;SVLEN=5988;AN=20618;AC=1;AF=4.850130953535746e-05;N_HET=1;N_HOMALT=0;N_HOMREF=10308;FREQ_HET=9.700261907071492e-05;FREQ_HOMALT=0.0;FREQ_HOMREF=0.9999029973809292;N_BI_GENOS=10309;Lingnan_Han_AN=1888;Lingnan_Han_AC=1;Lingna
chr4	86401452	.	A	<INS:ME:LINE1>	.	PASS	SVTYPE=INS:ME:LINE1;END=86401466;SVLEN=6009;AN=20618;AC=1;AF=4.850130953535746e-05;N_HET=1;N_HOMALT=0;N_HOMREF=10308;FREQ_HET=9.700261907071492e-05;FREQ_HOMALT=0.0;FREQ_HOMREF=0.9999029973809292;N_BI_GENOS=10309;Southeast_Han_AN=1908;Southeast_Han_AC=1;So
chr4	86554391	.	T	<INS:ME:LINE1>	.	PASS	SVTYPE=INS:ME:LINE1;END=86554410;SVLEN=3300;AN=20618;AC=15;AF=0.0007275196430303619;N_HET=15;N_HOMALT=0;N_HOMREF=10294;FREQ_HET=0.0014550392860607237;FREQ_HOMALT=0.0;FREQ_HOMREF=0.9985449607139393;N_BI_GENOS=10309;North_Han_AN=2272;North_Han_AC=2;North_Ha
chr4	86609245	.	A	<INS:ME:LINE1>	.	PASS	SVTYPE=INS:ME:LINE1;END=86609246;SVLEN=1410;AN=20618;AC=4;AF=0.00019400523814142983;N_HET=4;N_HOMALT=0;N_HOMREF=10305;FREQ_HET=0.00038801047628285966;FREQ_HOMALT=0.0;FREQ_HOMREF=0.9996119895237171;N_BI_GENOS=10309;East_Han_AN=6554;East_Han_AC=2;East_Han_A
chr4	86612860	.	A	<INS:ME:LINE1>	.	PASS	SVTYPE=INS:ME:LINE1;END=86612878;SVLEN=729;AN=20618;AC=1;AF=4.850130953535746e-05;N_HET=1;N_HOMALT=0;N_HOMREF=10308;FREQ_HET=9.700261907071492e-05;FREQ_HOMALT=0.0;FREQ_HOMREF=0.9999029973809292;N_BI_GENOS=10309;Northwest_Han_AN=1296;Northwest_Han_AC=1;Nor
chr4	86706395	.	A	<INS:ME:LINE1>	.	PASS	SVTYPE=INS:ME:LINE1;END=86706408;SVLEN=1272;AN=20618;AC=1;AF=4.850130953535746e-05;N_HET=1;N_HOMALT=0;N_HOMREF=10308;FREQ_HET=9.700261907071492e-05;FREQ_HOMALT=0.0;FREQ_HOMREF=0.9999029973809292;N_BI_GENOS=10309;East_Han_AN=6554;East_Han_AC=1;East_Han_AF=
chr4	86758082	.	A	<INS:ME:LINE1>	.	PASS	SVTYPE=INS:ME:LINE1;END=86758096;SVLEN=2337;AN=20618;AC=1;AF=4.850130953535746e-05;N_HET=1;N_HOMALT=0;N_HOMREF=10308;FREQ_HET=9.700261907071492e-05;FREQ_HOMALT=0.0;FREQ_HOMREF=0.9999029973809292;N_BI_GENOS=10309;East_Han_AN=6554;East_Han_AC=1;East_Han_AF=
chr4	87431857	.	G	<INS:ME:LINE1>	.	PASS	SVTYPE=INS:ME:LINE1;END=87431865;SVLEN=579;AN=20618;AC=66;AF=0.003201086429333592;N_HET=66;N_HOMALT=0;N_HOMREF=10243;FREQ_HET=0.006402172858667184;FREQ_HOMALT=0.0;FREQ_HOMREF=0.9935978271413328;N_BI_GENOS=10309;South_Han_AN=1496;South_Han_AC=6;South_Han_A
chr4	87432776	.	T	<INS:ME:LINE1>	.	PASS	SVTYPE=INS:ME:LINE1;END=87432788;SVLEN=5999;AN=20618;AC=5;AF=0.00024250654767678728;N_HET=5;N_HOMALT=0;N_HOMREF=10304;FREQ_HET=0.0004850130953535 7455;FREQ_HOMALT=0.0;FREQ_HOMREF=0.9995149869046465;N_BI_GENOS=10309;North_Han_AN=2272;North_Han_AC=1;North_Ha
chr4	87729162	.	T	<INS:ME:LINE1>	.	PASS	SVTYPE=INS:ME:LINE1;END=87729175;SVLEN=4209;AN=20618;AC=1;AF=4.850130953535746e-05;N_HET=1;N_HOMALT=0;N_HOMREF=10308;FREQ_HET=9.700261907071492e-05;FREQ_HOMALT=0.0;FREQ_HOMREF=0.9999029973809292;N_BI_GENOS=10309;Lingnan_Han_AN=1888;Lingnan_Han_AC=1;Lingna
chr4	87783045	.	A	<INS:ME:LINE1>	.	PASS	SVTYPE=INS:ME:LINE1;END=87783057;SVLEN=2265;AN=20618;AC=1;AF=4.850130953535746e-05;N_HET=1;N_HOMALT=0;N_HOMREF=10308;FREQ_HET=9.700261907071492e-05;FREQ_HOMALT=0.0;FREQ_HOMREF=0.9999029973809292;N_BI_GENOS=10309;Yi_AN=422;Yi_AC=1;Yi_AF=0.002369668246 44549
chr4	87936046	.	G	<INS:ME:LINE1>	.	PASS	SVTYPE=INS:ME:LINE1;END=87936046;SVLEN=0;AN=20618;AC=3;AF=0.00014550392860607236;N_HET=1;N_HOMALT=1;N_HOMREF=10307;FREQ_HET=9.700261907071492e-05;FREQ_HOMALT=9.700261907071492e-05;FREQ_HOMREF=0.9998059947618586;N_BI_GENOS=10309;Northwest_Han_AN=1296;North
chr4	88153542	.	G	<INS:ME:LINE1>	.	PASS	SVTYPE=INS:ME:LINE1;END=88153542;SVLEN=0;AN=20618;AC=1;AF=4.850130953535746e-05;N_HET=1;N_HOMALT=0;N_HOMREF=10308;FREQ_HET=9.700261907071492e-05;FREQ_HOMALT=0.0;FREQ_HOMREF=0.9999029973809292;N_BI_GENOS=10309;Central_Han_AN=2128;Central_Han_AC=1;Central_H

chr4	88358785	.	C	<INS:ME:LINE1>	.	PASS	SVTYPE=INS:ME:LINE1;END=88358805;SVLEN=5993;AN=20618;AC=3;AF=0.00014550392860607236;N_HET=3;N_HOMALT=0;N_HOMREF=10306;FREQ_HET=0.0002910078572121447;FREQ_HOMALT=0.0;FREQ_HOMREF=0.9997089921427879;N_BI_GENOS=10309;North_Han_AN=2272;North_Han_AC=2;North_Han
chr4	88710622	.	A	<INS:ME:LINE1>	.	PASS	SVTYPE=INS:ME:LINE1;END=88710638;SVLEN=5999;AN=20618;AC=3;AF=0.00014550392860607236;N_HET=3;N_HOMALT=0;N_HOMREF=10306;FREQ_HET=0.0002910078572121447;FREQ_HOMALT=0.0;FREQ_HOMREF=0.9997089921427879;N_BI_GENOS=10309;South_Han_AN=1496;South_Han_AC=2;South_Han
chr4	88806385	.	T	<INS:ME:LINE1>	.	PASS	SVTYPE=INS:ME:LINE1;END=88806391;SVLEN=1513;AN=20618;AC=165;AF=0.00800271607333398;N_HET=165;N_HOMALT=0;N_HOMREF=10144;FREQ_HET=0.01600543214666796;FREQ_HOMALT=0.0;FREQ_HOMREF=0.9839945678533321;N_BI_GENOS=10309;South_Han_AN=1496;South_Han_AC=8;South_Han_
chr4	88938151	.	T	<INS:ME:LINE1>	.	PASS	SVTYPE=INS:ME:LINE1;END=88938167;SVLEN=5995;AN=20618;AC=1;AF=4.850130953535746e-05;N_HET=1;N_HOMALT=0;N_HOMREF=10308;FREQ_HET=9.700261907071492e-05;FREQ_HOMALT=0.0;FREQ_HOMREF=0.9999029973809292;N_BI_GENOS=10309;Southeast_Han_AN=1908;Southeast_Han_AC=1;So
chr4	89137125	.	A	<INS:ME:LINE1>	.	PASS	SVTYPE=INS:ME:LINE1;END=89137139;SVLEN=450;AN=20618;AC=1;AF=4.850130953535746e-05;N_HET=1;N_HOMALT=0;N_HOMREF=10308;FREQ_HET=9.700261907071492e-05;FREQ_HOMALT=0.0;FREQ_HOMREF=0.9999029973809292;N_BI_GENOS=10309;East_Han_AN=6554;East_Han_AC=1;East_Han_AF=0
chr4	89339073	.	T	<INS:ME:LINE1>	.	PASS	SVTYPE=INS:ME:LINE1;END=89339088;SVLEN=2284;AN=20618;AC=1;AF=4.850130953535746e-05;N_HET=1;N_HOMALT=0;N_HOMREF=10308;FREQ_HET=9.700261907071492e-05;FREQ_HOMALT=0.0;FREQ_HOMREF=0.9999029973809292;N_BI_GENOS=10309;Central_Han_AN=2128;Central_Han_AC=1;Centra
chr4	89968802	.	G	<INS:ME:LINE1>	.	PASS	SVTYPE=INS:ME:LINE1;END=89968802;SVLEN=0;AN=20618;AC=6;AF=0.0002910078572121447;N_HET=4;N_HOMALT=1;N_HOMREF=10304;FREQ_HET=0.0003880104762828966;FREQ_HOMALT=9.700261907071492e-05;FREQ_HOMREF=0.9995149869046465;N_BI_GENOS=10309;South_Han_AN=1496;South_Han
chr4	90426193	.	A	<INS:ME:LINE1>	.	PASS	SVTYPE=INS:ME:LINE1;END=90426212;SVLEN=430;AN=20618;AC=4;AF=0.00019400523814142983;N_HET=4;N_HOMALT=0;N_HOMREF=10305;FREQ_HET=0.0003880104762828966;FREQ_HOMALT=0.0;FREQ_HOMREF=0.9996119895237171;N_BI_GENOS=10309;North_Han_AN=2272;North_Han_AC=1;North_Han
chr4	90606069	.	A	<INS:ME:LINE1>	.	PASS	SVTYPE=INS:ME:LINE1;END=90606084;SVLEN=126;AN=20618;AC=1;AF=4.850130953535746e-05;N_HET=1;N_HOMALT=0;N_HOMREF=10308;FREQ_HET=9.700261907071492e-05;FREQ_HOMALT=0.0;FREQ_HOMREF=0.9999029973809292;N_BI_GENOS=10309;South_Han_AN=1496;South_Han_AC=1;South_Han_A
chr4	90709983	.	A	<INS:ME:LINE1>	.	PASS	SVTYPE=INS:ME:LINE1;END=90709983;SVLEN=210;AN=20618;AC=1;AF=4.850130953535746e-05;N_HET=1;N_HOMALT=0;N_HOMREF=10308;FREQ_HET=9.700261907071492e-05;FREQ_HOMALT=0.0;FREQ_HOMREF=0.9999029973809292;N_BI_GENOS=10309;East_Han_AN=6554;East_Han_AC=1;East_Han_AF=0
chr4	90711474	.	T	<INS:ME:LINE1>	.	PASS	SVTYPE=INS:ME:LINE1;END=90711474;SVLEN=180;AN=20618;AC=1;AF=4.850130953535746e-05;N_HET=1;N_HOMALT=0;N_HOMREF=10308;FREQ_HET=9.700261907071492e-05;FREQ_HOMALT=0.0;FREQ_HOMREF=0.9999029973809292;N_BI_GENOS=10309;East_Han_AN=6554;East_Han_AC=1;East_Han_AF=0
chr4	90712929	.	T	<INS:ME:LINE1>	.	PASS	SVTYPE=INS:ME:LINE1;END=90712981;SVLEN=3929;AN=20618;AC=2;AF=9.700261907071492e-05;N_HET=2;N_HOMALT=0;N_HOMREF=10307;FREQ_HET=0.00019400523814142983;FREQ_HOMALT=0.0;FREQ_HOMREF=0.9998059947618586;N_BI_GENOS=10309;East_Han_AN=6554;East_Han_AC=2;East_Han_AF
chr4	90943227	.	A	<INS:ME:LINE1>	.	PASS	SVTYPE=INS:ME:LINE1;END=90943242;SVLEN=363;AN=20618;AC=105;AF=0.005092637501212533;N_HET=99;N_HOMALT=3;N_HOMREF=10207;FREQ_HET=0.009603259288000775;FREQ_HOMALT=0.0002910078572121447;FREQ_HOMREF=0.9901057328547871;N_BI_GENOS=10309;South_Han_AN=1496;South_H
chr4	91581459	.	A	<INS:ME:LINE1>	.	PASS	SVTYPE=INS:ME:LINE1;END=91581471;SVLEN=3092;AN=20618;AC=13;AF=0.0006305170239596469;N_HET=11;N_HOMALT=1;N_HOMREF=10297;FREQ_HET=0.001067028809777864;FREQ_HOMALT=9.700261907071492e-05;FREQ_HOMREF=0.9988359685711514;N_BI_GENOS=10309;South_Han_AN=1496;South_
chr4	91618019	.	A	<INS:ME:LINE1>	.	PASS	SVTYPE=INS:ME:LINE1;END=91618019;SVLEN=706;AN=20618;AC=1;AF=4.850130953535746e-05;N_HET=1;N_HOMALT=0;N_HOMREF=10308;FREQ_HET=9.700261907071492e-05;FREQ_HOMALT=0.0;FREQ_HOMREF=0.9999029973809292;N_BI_GENOS=10309;East_Han_AN=6554;East_Han_AC=1;East_Han_AF=0
chr4	91728677	.	A	<INS:ME:LINE1>	.	PASS	SVTYPE=INS:ME:LINE1;END=91728686;SVLEN=5838;AN=20618;AC=1;AF=4.850130953535746e-05;N_HET=1;N_HOMALT=0;N_HOMREF=10308;FREQ_HET=9.700261907071492e-05;FREQ_HOMALT=0.0;FREQ_HOMREF=0.9999029973809292;N_BI_GENOS=10309;North_Han_AN=2272;North_Han_AC=1;North_Han_
chr4	91953899	.	A	<INS:ME:LINE1>	.	PASS	SVTYPE=INS:ME:LINE1;END=91953916;SVLEN=525;AN=20618;AC=1;AF=4.850130953535746e-05;N_HET=1;N_HOMALT=0;N_HOMREF=10308;FREQ_HET=9.700261907071492e-05;FREQ_HOMALT=0.0;FREQ_HOMREF=0.9999029973809292;N_BI_GENOS=10309;Northwest_Han_AN=1296;Northwest_Han_AC=1;Nor
chr4	92147776	.	G	<INS:ME:LINE1>	.	PASS	SVTYPE=INS:ME:LINE1;END=92147776;SVLEN=715;AN=20618;AC=3;AF=0.00014550392860607236;N_HET=3;N_HOMALT=0;N_HOMREF=10306;FREQ_HET=0.0002910078572121447;FREQ_HOMALT=0.0;FREQ_HOMREF=0.9997089921427879;N_BI_GENOS=10309;East_Han_AN=6554;East_Han_AC=3;East_Han_AF=
chr4	92148226	.	A	<INS:ME:LINE1>	.	PASS	SVTYPE=INS:ME:LINE1;END=92148260;SVLEN=2151;AN=20618;AC=1;AF=4.850130953535746e-05;N_HET=1;N_HOMALT=0;N_HOMREF=10308;FREQ_HET=9.700261907071492e-05;FREQ_HOMALT=0.0;FREQ_HOMREF=0.9999029973809292;N_BI_GENOS=10309;East_Han_AN=6554;East_Han_AC=1;East_Han_AF=
chr4	92149908	.	A	<INS:ME:LINE1>	.	PASS	SVTYPE=INS:ME:LINE1;END=92149908;SVLEN=825;AN=20618;AC=1;AF=4.850130953535746e-05;N_HET=1;N_HOMALT=0;N_HOMREF=10308;FREQ_HET=9.700261907071492e-05;FREQ_HOMALT=0.0;FREQ_HOMREF=0.9999029973809292;N_BI_GENOS=10309;East_Han_AN=6554;East_Han_AC=1;East_Han_AF=0
chr4	92150132	.	A	<INS:ME:LINE1>	.	PASS	SVTYPE=INS:ME:LINE1;END=92150156;SVLEN=11;AN=20618;AC=1;AF=4.850130953535746e-05;N_HET=1;N_HOMALT=0;N_HOMREF=10308;FREQ_HET=9.700261907071492e-05;FREQ_HOMALT=0.0;FREQ_HOMREF=0.9999029973809292;N_BI_GENOS=10309;East_Han_AN=6554;East_Han_AC=1;East_Han_AF=0.
chr4	92573280	.	T	<INS:ME:LINE1>	.	PASS	SVTYPE=INS:ME:LINE1;END=92573280;SVLEN=1161;AN=20618;AC=6;AF=0.0002910078572121447;N_HET=6;N_HOMALT=0;N_HOMREF=10303;FREQ_HET=0.0005820157144242894;FREQ_HOMALT=0.0;FREQ_HOMREF=0.9994179842855757;N_BI_GENOS=10309;East_Han_AN=6554;East_Han_AC=6;East_Han_AF=
chr4	92573917	.	A	<INS:ME:LINE1>	.	PASS	SVTYPE=INS:ME:LINE1;END=92573917;SVLEN=149;AN=20618;AC=1;AF=4.850130953535746e-05;N_HET=1;N_HOMALT=0;N_HOMREF=10308;FREQ_HET=9.700261907071492e-05;FREQ_HOMALT=0.0;FREQ_HOMREF=0.9999029973809292;N_BI_GENOS=10309;East_Han_AN=6554;East_Han_AC=1;East_Han_AF=0
chr4	92588159	.	A	<INS:ME:LINE1>	.	PASS	SVTYPE=INS:ME:LINE1;END=92588171;SVLEN=6000;AN=20618;AC=1;AF=4.850130953535746e-05;N_HET=1;N_HOMALT=0;N_HOMREF=10308;FREQ_HET=9.700261907071492e-05;FREQ_HOMALT=0.0;FREQ_HOMREF=0.9999029973809292;N_BI_GENOS=10309;East_Han_AN=6554;East_Han_AC=1;East_Han_AF=
chr4	92762914	.	T	<INS:ME:LINE1>	.	PASS	SVTYPE=INS:ME:LINE1;END=92762927;SVLEN=2700;AN=20618;AC=1;AF=4.850130953535746e-05;N_HET=1;N_HOMALT=0;N_HOMREF=10308;FREQ_HET=9.700261907071492e-05;FREQ_HOMALT=0.0;FREQ_HOMREF=0.9999029973809292;N_BI_GENOS=10309;Northwest_Han_AN=1296;Northwest_Han_AC=1;No
chr4	92870929	.	G	<INS:ME:LINE1>	.	PASS	SVTYPE=INS:ME:LINE1;END=92870941;SVLEN=1119;AN=20618;AC=25;AF=0.0012125327383839363;N_HET=21;N_HOMALT=2;N_HOMREF=10286;FREQ_HET=0.002037055000485013;FREQ_HOMALT=0.00019400523814142983;FREQ_HOMREF=0.9977689397613736;N_BI_GENOS=10309;South_Han_AN=1496;South
chr4	92985211	.	C	<INS:ME:LINE1>	.	PASS	SVTYPE=INS:ME:LINE1;END=92985216;SVLEN=85;AN=20618;AC=1;AF=4.850130953535746e-05;N_HET=1;N_HOMALT=0;N_HOMREF=10308;FREQ_HET=9.700261907071492e-05;FREQ_HOMALT=0.0;FREQ_HOMREF=0.9999029973809292;N_BI_GENOS=10309;Southeast_Han_AN=1908;Southeast_Han_AC=1;Sout
chr4	93093801	.	A	<INS:ME:LINE1>	.	PASS	SVTYPE=INS:ME:LINE1;END=93093816;SVLEN=949;AN=20618;AC=4;AF=0.00019400523814142983;N_HET=4;N_HOMALT=0;N_HOMREF=10305;FREQ_HET=0.0003880104762828966;FREQ_HOMALT=0.0;FREQ_HOMREF=0.9996119895237171;N_BI_GENOS=10309;East_Han_AN=6554;East_Han_AC=3;East_Han_AF
chr4	93709686	.	G	<INS:ME:LINE1>	.	PASS	SVTYPE=INS:ME:LINE1;END=93709699;SVLEN=673;AN=20618;AC=1;AF=4.850130953535746e-05;N_HET=1;N_HOMALT=0;N_HOMREF=10308;FREQ_HET=9.700261907071492e-05;FREQ_HOMALT=0.0;FREQ_HOMREF=0.9999029973809292;N_BI_GENOS=10309;South_Han_AN=1496;South_Han_AC=1;South_Han_A
chr4	93819121	.	G	<INS:ME:LINE1>	.	PASS	SVTYPE=INS:ME:LINE1;END=93819134;SVLEN=2054;AN=20618;AC=1;AF=4.850130953535746e-05;N_HET=1;N_HOMALT=0;N_HOMREF=10308;FREQ_HET=9.700261907071492e-05;FREQ_HOMALT=0.0;FREQ_HOMREF=0.9999029973809292;N_BI_GENOS=10309;East_Han_AN=6554;East_Han_AC=1;East_Han_AF=
chr4	94064208	.	T	<INS:ME:LINE1>	.	PASS	SVTYPE=INS:ME:LINE1;END=94064226;SVLEN=217;AN=20618;AC=1;AF=4.850130953535746e-05;N_HET=1;N_HOMALT=0;N_HOMREF=10308;FREQ_HET=9.700261907071492e-05;FREQ_HOMALT=0.0;FREQ_HOMREF=0.9999029973809292;N_BI_GENOS=10309;Lingnan_Han_AN=1888;Lingnan_Han_AC=1;Lingnan
chr4	94088514	.	A	<INS:ME:LINE1>	.	PASS	SVTYPE=INS:ME:LINE1;END=94088514;SVLEN=0;AN=20618;AC=16;AF=0.0007760209525657193;N_HET=6;N_HOMALT=5;N_HOMREF=10298;FREQ_HET=0.0005820157144242894;FREQ_HOMALT=0.0004850130953535457455;FREQ_HOMREF=0.9989329711902222;N_BI_GENOS=10309;East_Han_AN=6554;East_Han_
chr4	94317043	.	A	<INS:ME:LINE1>	.	PASS	SVTYPE=INS:ME:LINE1;END=94317059;SVLEN=1523;AN=20618;AC=1;AF=4.850130953535746e-05;N_HET=1;N_HOMALT=0;N_HOMREF=10308;FREQ_HET=9.700261907071492e-05;FREQ_HOMALT=0.0;FREQ_HOMREF=0.9999029973809292;N_BI_GENOS=10309;North_Han_AN=2272;North_Han_AC=1;North_Han_
chr4	94717484	.	T	<INS:ME:LINE1>	.	PASS	SVTYPE=INS:ME:LINE1;END=94717484;SVLEN=6034;AN=20618;AC=1;AF=4.850130953535746e-05;N_HET=1;N_HOMALT=0;N_HOMREF=10308;FREQ_HET=9.700261907071492e-05;FREQ_HOMALT=0.0;FREQ_HOMREF=0.9999029973809292;N_BI_GENOS=10309;Southeast_Han_AN=1908;Southeast_Han_AC=1;So
chr4	95002354	.	C	<INS:ME:LINE1>	.	PASS	SVTYPE=INS:ME:LINE1;END=95002367;SVLEN=5954;AN=20618;AC=1;AF=4.850130953535746e-05;N_HET=1;N_HOMALT=0;N_HOMREF=10308;FREQ_HET=9.700261907071492e-05;FREQ_HOMALT=0.0;FREQ_HOMREF=0.9999029973809292;N_BI_GENOS=10309;Tibetan_AN=414;Tibetan_AC=1;Tibetan_AF=0.00
chr4	95125489	.	A	<INS:ME:LINE1>	.	PASS	SVTYPE=INS:ME:LINE1;END=95125506;SVLEN=963;AN=20618;AC=11;AF=0.000533514404888932;N_HET=11;N_HOMALT=0;N_HOMREF=10298;FREQ_HET=0.001067028809777864;FREQ_HOMALT=0.0;FREQ_HOMREF=0.9989329711902222;N_BI_GENOS=10309;Northwest_Han_AN=1296;Northwest_Han_AC=2;Nor
chr4	95756438	.	C	<INS:ME:LINE1>	.	PASS	SVTYPE=INS:ME:LINE1;END=95756438;SVLEN=2645;AN=20618;AC=1;AF=4.850130953535746e-05;N_HET=1;N_HOMALT=0;N_HOMREF=10308;FREQ_HET=9.700261907071492e-05;FREQ_HOMALT=0.0;FREQ_HOMREF=0.9999029973809292;N_BI_GENOS=10309;Central_Han_AN=2128;Central_Han_AC=1;Centra
chr4	96090379	.	A	<INS:ME:LINE1>	.	PASS	SVTYPE=INS:ME:LINE1;END=96090379;SVLEN=665;AN=20618;AC=1;AF=4.850130953535746e-05;N_HET=1;N_HOMALT=0;N_HOMREF=10308;FREQ_HET=9.700261907071492e-05;FREQ_HOMALT=0.0;FREQ_HOMREF=0.9999029973809292;N_BI_GENOS=10309;East_Han_AN=6554;East_Han_AC=1;East_Han_AF=0
chr4	96200913	.	A	<INS:ME:LINE1>	.	PASS	SVTYPE=INS:ME:LINE1;END=96200930;SVLEN=651;AN=20618;AC=1;AF=4.850130953535746e-05;N_HET=1;N_HOMALT=0;N_HOMREF=10308;FREQ_HET=9.700261907071492e-05;FREQ_HOMALT=0.0;FREQ_HOMREF=0.9999029973809292;N_BI_GENOS=10309;Northwest_Han_AN=1296;Northwest_Han_AC=1;Nor
chr4	96360645	.	C	<INS:ME:LINE1>	.	PASS	SVTYPE=INS:ME:LINE1;END=96360645;SVLEN=4929;AN=20618;AC=1;AF=4.850130953535746e-05;N_HET=1;N_HOMALT=0;N_HOMREF=10308;FREQ_HET=9.700261907071492e-05;FREQ_HOMALT=0.0;FREQ_HOMREF=0.9999029973809292;N_BI_GENOS=10309;Southeast_Han_AN=1908;Southeast_Han_AC=1;So
chr4	96490387	.	G	<INS:ME:LINE1>	.	PASS	SVTYPE=INS:ME:LINE1;END=96490403;SVLEN=6000;AN=20618;AC=1;AF=4.850130953535746e-05;N_HET=1;N_HOMALT=0;N_HOMREF=10308;FREQ_HET=9.700261907071492e-05;FREQ_HOMALT=0.0;FREQ_HOMREF=0.9999029973809292;N_BI_GENOS=10309;East_Han_AN=6554;East_Han_AC=1;East_Han_AF=
chr4	96505147	.	A	<INS:ME:LINE1>	.	PASS	SVTYPE=INS:ME:LINE1;END=96505162;SVLEN=6065;AN=20618;AC=1;AF=4.850130953535746e-05;N_HET=1;N_HOMALT=0;N_HOMREF=10308;FREQ_HET=9.700261907071492e-05;FREQ_HOMALT=0.0;FREQ_HOMREF=0.9999029973809292;N_BI_GENOS=10309;Southeast_Han_AN=1908;Southeast_Han_AC=1;So
chr4	96556594	.	T	<INS:ME:LINE1>	.	PASS	SVTYPE=INS:ME:LINE1;END=96556601;SVLEN=613;AN=20618;AC=1;AF=4.850130953535746e-05;N_HET=1;N_HOMALT=0;N_HOMREF=10308;FREQ_HET=9.700261907071492e-05;FREQ_HOMALT=0.0;FREQ_HOMREF=0.9999029973809292;N_BI_GENOS=10309;South_Han_AN=1496;South_Han_AC=1;South_Han_A
chr4	96843309	.	A	<INS:ME:LINE1>	.	PASS	SVTYPE=INS:ME:LINE1;END=96843326;SVLEN=1678;AN=20618;AC=1;AF=4.850130953535746e-05;N_HET=1;N_HOMALT=0;N_HOMREF=10308;FREQ_HET=9.700261907071492e-05;FREQ_HOMALT=0.0;FREQ_HOMREF=0.9999029973809292;N_BI_GENOS=10309;South_Han_AN=1496;South_Han_AC=1;South_Han_
chr4	96850112	.	A	<INS:ME:LINE1>	.	PASS	SVTYPE=INS:ME:LINE1;END=96850123;SVLEN=6014;AN=20618;AC=1;AF=4.850130953535746e-05;N_HET=1;N_HOMALT=0;N_HOMREF=10308;FREQ_HET=9.700261907071492e-05;FREQ_HOMALT=0.0;FREQ_HOMREF=0.9999029973809292;N_BI_GENOS=10309;North_Han_AN=2272;North_Han_AC=1;North_Han_
chr4	97052173	.	C	<INS:ME:LINE1>	.	PASS	SVTYPE=INS:ME:LINE1;END=97052190;SVLEN=6000;AN=20618;AC=1;AF=4.850130953535746e-05;N_HET=1;N_HOMALT=0;N_HOMREF=10308;FREQ_HET=9.700261907071492e-05;FREQ_HOMALT=0.0;FREQ_HOMREF=0.9999029973809292;N_BI_GENOS=10309;South_Han_AN=1496;South_Han_AC=1;South_Han_
chr4	97130306	.	G	<INS:ME:LINE1>	.	PASS	SVTYPE=INS:ME:LINE1;END=97130306;SVLEN=778;AN=20618;AC=1;AF=4.850130953535746e-05;N_HET=1;N_HOMALT=0;N_HOMREF=10308;FREQ_HET=9.700261907071492e-05;FREQ_HOMALT=0.0;FREQ_HOMREF=0.9999029973809292;N_BI_GENOS=10309;East_Han_AN=6554;East_Han_AC=1;East_Han_AF=0
chr4	97391485	.	T	<INS:ME:LINE1>	.	PASS	SVTYPE=INS:ME:LINE1;END=97391487;SVLEN=1804;AN=20618;AC=65;AF=0.0031525851197982345;N_HET=65;N_HOMALT=0;N_HOMREF=10244;FREQ_HET=0.006305170239596469;FREQ_HOMALT=0.0;FREQ_HOMREF=0.9936948297604036;N_BI_GENOS=10309;South_Han_AN=1496;South_Han_AC=6;South_Han
chr4	97605227	.	T	<INS:ME:LINE1>	.	PASS	SVTYPE=INS:ME:LINE1;END=97605243;SVLEN=553;AN=20618;AC=3;AF=0.00014550392860607236;N_HET=3;N_HOMALT=0;N_HOMREF=10306;FREQ_HET=0.0002910078572121447;FREQ_HOMALT=0.0;FREQ_HOMREF=0.9997089921427879;N_BI_GENOS=10309;East_Han_AN=6554;East_Han_AC=3;East_Han_AF=
chr4	97725511	.	A	<INS:ME:LINE1>	.	PASS	SVTYPE=INS:ME:LINE1;END=97725528;SVLEN=865;AN=20618;AC=1;AF=4.850130953535746e-05;N_HET=1;N_HOMALT=0;N_HOMREF=10308;FREQ_HET=9.700261907071492e-05;FREQ_HOMALT=0.0;FREQ_HOMREF=0.9999029973809292;N_BI_GENOS=10309;East_Han_AN=6554;East_Han_AC=1;East_Han_AF=0
chr4	97811979	.	C	<INS:ME:LINE1>	.	PASS	SVTYPE=INS:ME:LINE1;END=97811979;SVLEN=1806;AN=20618;AC=1;AF=4.850130953535746e-05;N_HET=1;N_HOMALT=0;N_HOMREF=10308;FREQ_HET=9.700261907071492e-05;FREQ_HOMALT=0.0;FREQ_HOMREF=0.9999029973809292;N_BI_GENOS=10309;Mongolian_AN=454;Mongolian_AC=1;Mongolian_A
chr4	98189692	.	G	<INS:ME:LINE1>	.	PASS	SVTYPE=INS:ME:LINE1;END=98189705;SVLEN=5999;AN=20618;AC=1;AF=4.850130953535746e-05;N_HET=1;N_HOMALT=0;N_HOMREF=10308;FREQ_HET=9.700261907071492e-05;FREQ_HOMALT=0.0;FREQ_HOMREF=0.9999029973809292;N_BI_GENOS=10309;Northwest_Han_AN=1296;Northwest_Han_AC=1;No
chr4	98347112	.	T	<INS:ME:LINE1>	.	PASS	SVTYPE=INS:ME:LINE1;END=98347126;SVLEN=862;AN=20618;AC=5;AF=0.00024250654767678728;N_HET=5;N_HOMALT=0;N_HOMREF=10304;FREQ_HET=0.0004850130953535457455;FREQ_HOMALT=0.0;FREQ_HOMREF=0.9995149869046465;N_BI_GENOS=10309;South_Han_AN=1496;South_Han_AC=1;South_Han
chr4	98410365	.	A	<INS:ME:LINE1>	.	PASS	SVTYPE=INS:ME:LINE1;END=98410379;SVLEN=75;AN=20618;AC=1;AF=4.850130953535746e-05;N_HET=1;N_HOMALT=0;N_HOMREF=10308;FREQ_HET=9.700261907071492e-05;FREQ_HOMALT=0.0;FREQ_HOMREF=0.9999029973809292;N_BI_GENOS=10309;East_Han_AN=6554;East_Han_AC=1;East_Han_AF=0.
chr4	98560276	.	G	<INS:ME:LINE1>	.	PASS	SVTYPE=INS:ME:LINE1;END=98560293;SVLEN=127;AN=20618;AC=1;AF=4.850130953535746e-05;N_HET=1;N_HOMALT=0;N_HOMREF=10308;FREQ_HET=9.700261907071492e-05;FREQ_HOMALT=0.0;FREQ_HOMREF=0.9999029973809292;N_BI_GENOS=10309;Lingnan_Han_AN=1888;Lingnan_Han_AC=1;Lingnan
chr4	98715096	.	A	<INS:ME:LINE1>	.	PASS	SVTYPE=INS:ME:LINE1;END=98715112;SVLEN=6000;AN=20618;AC=1;AF=4.850130953535746e-05;N_HET=1;N_HOMALT=0;N_HOMREF=10308;FREQ_HET=9.700261907071492e-05;FREQ_HOMALT=0.0;FREQ_HOMREF=0.9999029973809292;N_BI_GENOS=10309;Mongolian_AN=454;Mongolian_AC=1;Mongolian_A
chr4	99105032	.	T	<INS:ME:LINE1>	.	PASS	SVTYPE=INS:ME:LINE1;END=99105049;SVLEN=537;AN=20618;AC=1;AF=4.850130953535746e-05;N_HET=1;N_HOMALT=0;N_HOMREF=10308;FREQ_HET=9.700261907071492e-05;FREQ_HOMALT=0.0;FREQ_HOMREF=0.9999029973809292;N_BI_GENOS=10309;Yi_AN=422;Yi_AC=1;Yi_AF=0.002369668246445498
chr4	99133062	.	A	<INS:ME:LINE1>	.	PASS	SVTYPE=INS:ME:LINE1;END=99133076;SVLEN=801;AN=20618;AC=1;AF=4.850130953535746e-05;N_HET=1;N_HOMALT=0;N_HOMREF=10308;FREQ_HET=9.700261907071492e-05;FREQ_HOMALT=0.0;FREQ_HOMREF=0.9999029973809292;N_BI_GENOS=10309;Southeast_Han_AN=1908;Southeast_Han_AC=1;Sou
chr4	99264731	.	A	<INS:ME:LINE1>	.	PASS	SVTYPE=INS:ME:LINE1;END=99264748;SVLEN=5993;AN=20618;AC=22;AF=0.001067028809777864;N_HET=22;N_HOMALT=0;N_HOMREF=10287;FREQ_HET=0.002134057619555728;FREQ_HOMALT=0.0;FREQ_HOMREF=0.9978659423804442;N_BI_GENOS=10309;South_Han_AN=1496;South_Han_AC=3;South_Han_
chr4	99318687	.	A	<INS:ME:LINE1>	.	PASS	SVTYPE=INS:ME:LINE1;END=99318694;SVLEN=1052;AN=20618;AC=1;AF=4.850130953535746e-05;N_HET=1;N_HOMALT=0;N_HOMREF=10308;FREQ_HET=9.700261907071492e-05;FREQ_HOMALT=0.0;FREQ_HOMREF=0.9999029973809292;N_BI_GENOS=10309;East_Han_AN=6554;East_Han_AC=1;East_Han_AF=
chr4	99428210	.	T	<INS:ME:LINE1>	.	PASS	SVTYPE=INS:ME:LINE1;END=99428210;SVLEN=2503;AN=20618;AC=1;AF=4.850130953535746e-05;N_HET=1;N_HOMALT=0;N_HOMREF=10308;FREQ_HET=9.700261907071492e-05;FREQ_HOMALT=0.0;FREQ_HOMREF=0.9999029973809292;N_BI_GENOS=10309;East_Han_AN=6554;East_Han_AC=1;East_Han_AF=
chr4	99472673	.	T	<INS:ME:LINE1>	.	PASS	SVTYPE=INS:ME:LINE1;END=99472687;SVLEN=6005;AN=20618;AC=1;AF=4.850130953535746e-05;N_HET=1;N_HOMALT=0;N_HOMREF=10308;FREQ_HET=9.700261907071492e-05;FREQ_HOMALT=0.0;FREQ_HOMREF=0.9999029973809292;N_BI_GENOS=10309;Lingnan_Han_AN=1888;Lingnan_Han_AC=1;Lingna
chr4	99548721	.	A	<INS:ME:LINE1>	.	PASS	SVTYPE=INS:ME:LINE1;END=99548738;SVLEN=6071;AN=20618;AC=1;AF=4.850130953535746e-05;N_HET=1;N_HOMALT=0;N_HOMREF=10308;FREQ_HET=9.700261907071492e-05;FREQ_HOMALT=0.0;FREQ_HOMREF=0.9999029973809292;N_BI_GENOS=10309;Southeast_Han_AN=1908;Southeast_Han_AC=1;So
chr4	99593268	.	C	<INS:ME:LINE1>	.	PASS	SVTYPE=INS:ME:LINE1;END=99593284;SVLEN=679;AN=20618;AC=1;AF=4.850130953535746e-05;N_HET=1;N_HOMALT=0;N_HOMREF=10308;FREQ_HET=9.700261907071492e-05;FREQ_HOMALT=0.0;FREQ_HOMREF=0.9999029973809292;N_BI_GENOS=10309;East_Han_AN=6554;East_Han_AC=1;East_Han_AF=0
chr4	100217782	.	T	<INS:ME:LINE1>	.	PASS	SVTYPE=INS:ME:LINE1;END=100217782;SVLEN=249;AN=20618;AC=1;AF=4.850130953535746e-05;N_HET=1;N_HOMALT=0;N_HOMREF=10308;FREQ_HET=9.700261907071492e-05;FREQ_HOMALT=0.0;FREQ_HOMREF=0.9999029973809292;N_BI_GENOS=10309;East_Han_AN=6554;East_Han_AC=1;East_Han_AF=
chr4	100342964	.	T	<INS:ME:LINE1>	.	PASS	SVTYPE=INS:ME:LINE1;END=100342965;SVLEN=700;AN=20618;AC=1;AF=4.850130953535746e-05;N_HET=1;N_HOMALT=0;N_HOMREF=10308;FREQ_HET=9.700261907071492e-05;FREQ_HOMALT=0.0;FREQ_HOMREF=0.9999029973809292;N_BI_GENOS=10309;Northwest_Han_AN=1296;Northwest_Han_AC=1;No
chr4	100462389	.	A	<INS:ME:LINE1>	.	PASS	SVTYPE=INS:ME:LINE1;END=100462407;SVLEN=4033;AN=20618;AC=1;AF=4.850130953535746e-05;N_HET=1;N_HOMALT=0;N_HOMREF=10308;FREQ_HET=9.700261907071492e-05;FREQ_HOMALT=0.0;FREQ_HOMREF=0.9999029973809292;N_BI_GENOS=10309;North_Han_AN=2272;North_Han_AC=1;North_Han
chr4	100871815	.	A	<INS:ME:LINE1>	.	PASS	SVTYPE=INS:ME:LINE1;END=100871831;SVLEN=1089;AN=20618;AC=1;AF=4.850130953535746e-05;N_HET=1;N_HOMALT=0;N_HOMREF=10308;FREQ_HET=9.700261907071492e-05;FREQ_HOMALT=0.0;FREQ_HOMREF=0.9999029973809292;N_BI_GENOS=10309;Central_Han_AN=2128;Central_Han_AC=1;Centr
chr4	101321980	.	A	<INS:ME:LINE1>	.	PASS	SVTYPE=INS:ME:LINE1;END=101321998;SVLEN=314;AN=20618;AC=15;AF=0.0007275196430303619;N_HET=15;N_HOMALT=0;N_HOMREF=10294;FREQ_HET=0.0014550392860607237;FREQ_HOMALT=0.0;FREQ_HOMREF=0.9985449607139393;N_BI_GENOS=10309;South_Han_AN=1496;South_Han_AC=2;South_Ha
chr4	101436467	.	A	<INS:ME:LINE1>	.	PASS	SVTYPE=INS:ME:LINE1;END=101436483;SVLEN=1084;AN=20618;AC=1;AF=4.850130953535746e-05;N_HET=1;N_HOMALT=0;N_HOMREF=10308;FREQ_HET=9.700261907071492e-05;FREQ_HOMALT=0.0;FREQ_HOMREF=0.9999029973809292;N_BI_GENOS=10309;East_Han_AN=6554;East_Han_AC=1;East_Han_AF
chr4	101686702	.	T	<INS:ME:LINE1>	.	PASS	SVTYPE=INS:ME:LINE1;END=101686712;SVLEN=3893;AN=20618;AC=1;AF=4.850130953535746e-05;N_HET=1;N_HOMALT=0;N_HOMREF=10308;FREQ_HET=9.700261907071492e-05;FREQ_HOMALT=0.0;FREQ_HOMREF=0.9999029973809292;N_BI_GENOS=10309;Hui_AN=444;Hui_AC=1;Hui_AF=0.0022522522522
chr4	101730821	.	A	<INS:ME:LINE1>	.	PASS	SVTYPE=INS:ME:LINE1;END=101730821;SVLEN=98;AN=20618;AC=1;AF=4.850130953535746e-05;N_HET=1;N_HOMALT=0;N_HOMREF=10308;FREQ_HET=9.700261907071492e-05;FREQ_HOMALT=0.0;FREQ_HOMREF=0.9999029973809292;N_BI_GENOS=10309;Tibetan_AN=414;Tibetan_AC=1;Tibetan_AF=0.002
chr4	101743809	.	G	<INS:ME:LINE1>	.	PASS	SVTYPE=INS:ME:LINE1;END=101743825;SVLEN=5999;AN=20618;AC=1;AF=4.850130953535746e-05;N_HET=1;N_HOMALT=0;N_HOMREF=10308;FREQ_HET=9.700261907071492e-05;FREQ_HOMALT=0.0;FREQ_HOMREF=0.9999029973809292;N_BI_GENOS=10309;Lingnan_Han_AN=1888;Lingnan_Han_AC=1;Lingn
chr4	101844983	.	C	<INS:ME:LINE1>	.	PASS	SVTYPE=INS:ME:LINE1;END=101845001;SVLEN=2583;AN=20618;AC=1;AF=4.850130953535746e-05;N_HET=1;N_HOMALT=0;N_HOMREF=10308;FREQ_HET=9.700261907071492e-05;FREQ_HOMALT=0.0;FREQ_HOMREF=0.9999029973809292;N_BI_GENOS=10309;East_Han_AN=6554;East_Han_AC=1;East_Han_AF
chr4	101863994	.	A	<INS:ME:LINE1>	.	PASS	SVTYPE=INS:ME:LINE1;END=101864002;SVLEN=1528;AN=20618;AC=1;AF=4.850130953535746e-05;N_HET=1;N_HOMALT=0;N_HOMREF=10308;FREQ_HET=9.700261907071492e-05;FREQ_HOMALT=0.0;FREQ_HOMREF=0.9999029973809292;N_BI_GENOS=10309;East_Han_AN=6554;East_Han_AC=1;East_Han_AF
chr4	102422588	.	A	<INS:ME:LINE1>	.	PASS	SVTYPE=INS:ME:LINE1;END=102422595;SVLEN=1797;AN=20618;AC=4215;AF=0.20443301969153166;N_HET=2401;N_HOMALT=907;N_HOMREF=7001;FREQ_HET=0.2329032883887865;FREQ_HOMALT=0.08798137549713843;FREQ_HOMREF=0.679115336114075;N_BI_GENOS=10309;South_Han_AN=1496;South_H
chr4	102452282	.	A	<INS:ME:LINE1>	.	PASS	SVTYPE=INS:ME:LINE1;END=102452297;SVLEN=118;AN=20618;AC=1;AF=4.850130953535746e-05;N_HET=1;N_HOMALT=0;N_HOMREF=10308;FREQ_HET=9.700261907071492e-05;FREQ_HOMALT=0.0;FREQ_HOMREF=0.9999029973809292;N_BI_GENOS=10309;Central_Han_AN=2128;Central_Han_AC=1;Centra
chr4	102661985	.	A	<INS:ME:LINE1>	.	PASS	SVTYPE=INS:ME:LINE1;END=102662002;SVLEN=716;AN=20618;AC=7;AF=0.0003395091667475022;N_HET=7;N_HOMALT=0;N_HOMREF=10302;FREQ_HET=0.0006790183334950044;FREQ_HOMALT=0.0;FREQ_HOMREF=0.999320981666505;N_BI_GENOS=10309;Lingnan_Han_AN=1888;Lingnan_Han_AC=1;Lingnan
chr4	102914224	.	C	<INS:ME:LINE1>	.	PASS	SVTYPE=INS:ME:LINE1;END=102914224;SVLEN=0;AN=20618;AC=339;AF=0.016441943932486176;N_HET=83;N_HOMALT=128;N_HOMREF=10098;FREQ_HET=0.008051217382869338;FREQ_HOMALT=0.01241633524105151;FREQ_HOMREF=0.9795324473760791;N_BI_GENOS=10309;South_Han_AN=1496;South_Ha
chr4	102940545	.	A	<INS:ME:LINE1>	.	PASS	SVTYPE=INS:ME:LINE1;END=102940545;SVLEN=583;AN=20618;AC=1;AF=4.850130953535746e-05;N_HET=1;N_HOMALT=0;N_HOMREF=10308;FREQ_HET=9.700261907071492e-05;FREQ_HOMALT=0.0;FREQ_HOMREF=0.9999029973809292;N_BI_GENOS=10309;East_Han_AN=6554;East_Han_AC=1;East_Han_AF=
chr4	103011238	.	A	<INS:ME:LINE1>	.	PASS	SVTYPE=INS:ME:LINE1;END=103011254;SVLEN=3357;AN=20618;AC=1;AF=4.850130953535746e-05;N_HET=1;N_HOMALT=0;N_HOMREF=10308;FREQ_HET=9.700261907071492e-05;FREQ_HOMALT=0.0;FREQ_HOMREF=0.9999029973809292;N_BI_GENOS=10309;East_Han_AN=6554;East_Han_AC=1;East_Han_AF
chr4	103293513	.	C	<INS:ME:LINE1>	.	PASS	SVTYPE=INS:ME:LINE1;END=103293529;SVLEN=793;AN=20618;AC=1192;AF=0.05781356096614609;N_HET=972;N_HOMALT=110;N_HOMREF=9227;FREQ_HET=0.0942865457367349;FREQ_HOMALT=0.01067028809777864;FREQ_HOMREF=0.8950431661654865;N_BI_GENOS=10309;South_Han_AN=1496;South_Ha
chr4	103773361	.	T	<INS:ME:LINE1>	.	PASS	SVTYPE=INS:ME:LINE1;END=103773379;SVLEN=892;AN=20618;AC=1;AF=4.850130953535746e-05;N_HET=1;N_HOMALT=0;N_HOMREF=10308;FREQ_HET=9.700261907071492e-05;FREQ_HOMALT=0.0;FREQ_HOMREF=0.9999029973809292;N_BI_GENOS=10309;East_Han_AN=6554;East_Han_AC=1;East_Han_AF=
chr4	103808082	.	T	<INS:ME:LINE1>	.	PASS	SVTYPE=INS:ME:LINE1;END=103808082;SVLEN=219;AN=20618;AC=2;AF=9.700261907071492e-05;N_HET=2;N_HOMALT=0;N_HOMREF=10307;FREQ_HET=0.00019400523814142983;FREQ_HOMALT=0.0;FREQ_HOMREF=0.9998059947618586;N_BI_GENOS=10309;East_Han_AN=6554;East_Han_AC=2;East_Han_AF
chr4	104355530	.	A	<INS:ME:LINE1>	.	PASS	SVTYPE=INS:ME:LINE1;END=104355545;SVLEN=5985;AN=20618;AC=1;AF=4.850130953535746e-05;N_HET=1;N_HOMALT=0;N_HOMREF=10308;FREQ_HET=9.700261907071492e-05;FREQ_HOMALT=0.0;FREQ_HOMREF=0.9999029973809292;N_BI_GENOS=10309;Southeast_Han_AN=1908;Southeast_Han_AC=1;S
chr4	104573052	.	T	<INS:ME:LINE1>	.	PASS	SVTYPE=INS:ME:LINE1;END=104573067;SVLEN=558;AN=20618;AC=1;AF=4.850130953535746e-05;N_HET=1;N_HOMALT=0;N_HOMREF=10308;FREQ_HET=9.700261907071492e-05;FREQ_HOMALT=0.0;FREQ_HOMREF=0.9999029973809292;N_BI_GENOS=10309;Lingnan_Han_AN=1888;Lingnan_Han_AC=1;Lingna
chr4	104725397	.	A	<INS:ME:LINE1>	.	PASS	SVTYPE=INS:ME:LINE1;END=104725414;SVLEN=2440;AN=20618;AC=1;AF=4.850130953535746e-05;N_HET=1;N_HOMALT=0;N_HOMREF=10308;FREQ_HET=9.700261907071492e-05;FREQ_HOMALT=0.0;FREQ_HOMREF=0.9999029973809292;N_BI_GENOS=10309;Northwest_Han_AN=1296;Northwest_Han_AC=1;N
chr4	105015528	.	A	<INS:ME:LINE1>	.	PASS	SVTYPE=INS:ME:LINE1;END=105015543;SVLEN=215;AN=20618;AC=1;AF=4.850130953535746e-05;N_HET=1;N_HOMALT=0;N_HOMREF=10308;FREQ_HET=9.700261907071492e-05;FREQ_HOMALT=0.0;FREQ_HOMREF=0.9999029973809292;N_BI_GENOS=10309;Southeast_Han_AN=1908;Southeast_Han_AC=1;So
chr4	105053475	.	T	<INS:ME:LINE1>	.	PASS	SVTYPE=INS:ME:LINE1;END=105053500;SVLEN=2786;AN=20618;AC=2;AF=9.700261907071492e-05;N_HET=2;N_HOMALT=0;N_HOMREF=10307;FREQ_HET=0.00019400523814142983;FREQ_HOMALT=0.0;FREQ_HOMREF=0.9998059947618586;N_BI_GENOS=10309;East_Han_AN=6554;East_Han_AC=2;East_Han_A
chr4	105450150	.	A	<INS:ME:LINE1>	.	PASS	SVTYPE=INS:ME:LINE1;END=105450150;SVLEN=5978;AN=20618;AC=2;AF=9.700261907071492e-05;N_HET=2;N_HOMALT=0;N_HOMREF=10307;FREQ_HET=0.00019400523814142983;FREQ_HOMALT=0.0;FREQ_HOMREF=0.9998059947618586;N_BI_GENOS=10309;North_Han_AN=2272;North_Han_AC=1;North_Ha
chr4	105810712	.	A	<INS:ME:LINE1>	.	PASS	SVTYPE=INS:ME:LINE1;END=105810726;SVLEN=5990;AN=20618;AC=2;AF=9.700261907071492e-05;N_HET=2;N_HOMALT=0;N_HOMREF=10307;FREQ_HET=0.00019400523814142983;FREQ_HOMALT=0.0;FREQ_HOMREF=0.9998059947618586;N_BI_GENOS=10309;Central_Han_AN=2128;Central_Han_AC=1;Cent
chr4	105975858	.	C	<INS:ME:LINE1>	.	PASS	SVTYPE=INS:ME:LINE1;END=105975874;SVLEN=6039;AN=20618;AC=1;AF=4.850130953535746e-05;N_HET=1;N_HOMALT=0;N_HOMREF=10308;FREQ_HET=9.700261907071492e-05;FREQ_HOMALT=0.0;FREQ_HOMREF=0.9999029973809292;N_BI_GENOS=10309;East_Han_AN=6554;East_Han_AC=1;East_Han_AF
chr4	106439208	.	A	<INS:ME:LINE1>	.	PASS	SVTYPE=INS:ME:LINE1;END=106439222;SVLEN=6010;AN=20618;AC=2;AF=9.700261907071492e-05;N_HET=2;N_HOMALT=0;N_HOMREF=10307;FREQ_HET=0.00019400523814142983;FREQ_HOMALT=0.0;FREQ_HOMREF=0.9998059947618586;N_BI_GENOS=10309;Central_Han_AN=2128;Central_Han_AC=2;Cent
chr4	106455071	.	G	<INS:ME:LINE1>	.	PASS	SVTYPE=INS:ME:LINE1;END=106455087;SVLEN=6000;AN=20618;AC=1;AF=4.850130953535746e-05;N_HET=1;N_HOMALT=0;N_HOMREF=10308;FREQ_HET=9.700261907071492e-05;FREQ_HOMALT=0.0;FREQ_HOMREF=0.9999029973809292;N_BI_GENOS=10309;Lingnan_Han_AN=1888;Lingnan_Han_AC=1;Lingn
chr4	106515846	.	A	<INS:ME:LINE1>	.	PASS	SVTYPE=INS:ME:LINE1;END=106515846;SVLEN=168;AN=20618;AC=2;AF=9.700261907071492e-05;N_HET=2;N_HOMALT=0;N_HOMREF=10307;FREQ_HET=0.00019400523814142983;FREQ_HOMALT=0.0;FREQ_HOMREF=0.9998059947618586;N_BI_GENOS=10309;East_Han_AN=6554;East_Han_AC=2;East_Han_AF
chr4	106516021	.	C	<INS:ME:LINE1>	.	PASS	SVTYPE=INS:ME:LINE1;END=106516021;SVLEN=229;AN=20618;AC=1;AF=4.850130953535746e-05;N_HET=1;N_HOMALT=0;N_HOMREF=10308;FREQ_HET=9.700261907071492e-05;FREQ_HOMALT=0.0;FREQ_HOMREF=0.9999029973809292;N_BI_GENOS=10309;East_Han_AN=6554;East_Han_AC=1;East_Han_AF=

chr4	106516782	.	C	<INS:ME:LINE1>	.	PASS	SVTYPE=INS:ME:LINE1;END=106516796;SVLEN=812;AN=20618;AC=2;AF=9.700261907071492e-05;N_HET=0;N_HOMALT=1;N_HOMREF=10308;FREQ_HET=0.0;FREQ_HOMALT=9.700261907071492e-05;FREQ_HOMREF=0.9999029973809292;N_BI_GENOS=10309;North_Han_AN=2272;North_Han_AC=2;North_Han_
chr4	107637291	.	G	<INS:ME:LINE1>	.	PASS	SVTYPE=INS:ME:LINE1;END=107637307;SVLEN=1201;AN=20618;AC=1;AF=4.850130953535746e-05;N_HET=1;N_HOMALT=0;N_HOMREF=10308;FREQ_HET=9.700261907071492e-05;FREQ_HOMALT=0.0;FREQ_HOMREF=0.9999029973809292;N_BI_GENOS=10309;East_Han_AN=6554;East_Han_AC=1;East_Han_AF
chr4	108131696	.	G	<INS:ME:LINE1>	.	PASS	SVTYPE=INS:ME:LINE1;END=108131715;SVLEN=868;AN=20618;AC=1;AF=4.850130953535746e-05;N_HET=1;N_HOMALT=0;N_HOMREF=10308;FREQ_HET=9.700261907071492e-05;FREQ_HOMALT=0.0;FREQ_HOMREF=0.9999029973809292;N_BI_GENOS=10309;East_Han_AN=6554;East_Han_AC=1;East_Han_AF=
chr4	108228501	.	C	<INS:ME:LINE1>	.	PASS	SVTYPE=INS:ME:LINE1;END=108228517;SVLEN=5998;AN=20618;AC=4;AF=0.0001940052381414298 3;N_HET=4;N_HOMALT=0;N_HOMREF=10305;FREQ_HET=0.0003880104762828596 6;FREQ_HOMALT=0.0;FREQ_HOMREF=0.9996119895237171;N_BI_GENOS=10309;South_Han_AN=1496;South_Han_AC=1;South_H
chr4	109104012	.	T	<INS:ME:LINE1>	.	PASS	SVTYPE=INS:ME:LINE1;END=109104030;SVLEN=564;AN=20618;AC=1;AF=4.850130953535746e-05;N_HET=1;N_HOMALT=0;N_HOMREF=10308;FREQ_HET=9.700261907071492e-05;FREQ_HOMALT=0.0;FREQ_HOMREF=0.9999029973809292;N_BI_GENOS=10309;Central_Han_AN=2128;Central_Han_AC=1;Centra
chr4	109327159	.	A	<INS:ME:LINE1>	.	PASS	SVTYPE=INS:ME:LINE1;END=109327176;SVLEN=5982;AN=20618;AC=10;AF=0.00048501309535357455;N_HET=8;N_HOMALT=1;N_HOMREF=10300;FREQ_HET=0.0007760209525657193;FREQ_HOMALT=9.700261907071492e-05;FREQ_HOMREF=0.9991269764283636;N_BI_GENOS=10309;South_Han_AN=1496;Sout
chr4	109972656	.	T	<INS:ME:LINE1>	.	PASS	SVTYPE=INS:ME:LINE1;END=109972670;SVLEN=6075;AN=20618;AC=1;AF=4.850130953535746e-05;N_HET=1;N_HOMALT=0;N_HOMREF=10308;FREQ_HET=9.700261907071492e-05;FREQ_HOMALT=0.0;FREQ_HOMREF=0.9999029973809292;N_BI_GENOS=10309;Miao_AN=482;Miao_AC=1;Miao_AF=0.0020746887
chr4	109991648	.	A	<INS:ME:LINE1>	.	PASS	SVTYPE=INS:ME:LINE1;END=109991659;SVLEN=6008;AN=20618;AC=1;AF=4.850130953535746e-05;N_HET=1;N_HOMALT=0;N_HOMREF=10308;FREQ_HET=9.700261907071492e-05;FREQ_HOMALT=0.0;FREQ_HOMREF=0.9999029973809292;N_BI_GENOS=10309;East_Han_AN=6554;East_Han_AC=1;East_Han_AF
chr4	110293919	.	G	<INS:ME:LINE1>	.	PASS	SVTYPE=INS:ME:LINE1;END=110293923;SVLEN=91;AN=20618;AC=2;AF=9.700261907071492e-05;N_HET=2;N_HOMALT=0;N_HOMREF=10307;FREQ_HET=0.00019400523814142983;FREQ_HOMALT=0.0;FREQ_HOMREF=0.9998059947618586;N_BI_GENOS=10309;Lingnan_Han_AN=1888;Lingnan_Han_AC=1;Lingna
chr4	110495036	.	T	<INS:ME:LINE1>	.	PASS	SVTYPE=INS:ME:LINE1;END=110495036;SVLEN=5975;AN=20618;AC=1;AF=4.850130953535746e-05;N_HET=1;N_HOMALT=0;N_HOMREF=10308;FREQ_HET=9.700261907071492e-05;FREQ_HOMALT=0.0;FREQ_HOMREF=0.9999029973809292;N_BI_GENOS=10309;Central_Han_AN=2128;Central_Han_AC=1;Centr
chr4	110499662	.	C	<INS:ME:LINE1>	.	PASS	SVTYPE=INS:ME:LINE1;END=110499674;SVLEN=5998;AN=20618;AC=2;AF=9.700261907071492e-05;N_HET=2;N_HOMALT=0;N_HOMREF=10307;FREQ_HET=0.00019400523814142983;FREQ_HOMALT=0.0;FREQ_HOMREF=0.9998059947618586;N_BI_GENOS=10309;East_Han_AN=6554;East_Han_AC=1;East_Han_A
chr4	110850429	.	A	<INS:ME:LINE1>	.	PASS	SVTYPE=INS:ME:LINE1;END=110850432;SVLEN=560;AN=20618;AC=20;AF=0.0009700261907071491;N_HET=20;N_HOMALT=0;N_HOMREF=10289;FREQ_HET=0.0019400523814142982;FREQ_HOMALT=0.0;FREQ_HOMREF=0.9980599476185857;N_BI_GENOS=10309;South_Han_AN=1496;South_Han_AC=1;South_Ha
chr4	111158939	.	A	<INS:ME:LINE1>	.	PASS	SVTYPE=INS:ME:LINE1;END=111158953;SVLEN=504;AN=20618;AC=6;AF=0.0002910078572121447;N_HET=4;N_HOMALT=1;N_HOMREF=10304;FREQ_HET=0.0003880104762828596 6;FREQ_HOMALT=9.700261907071492e-05;FREQ_HOMREF=0.9995149869046465;N_BI_GENOS=10309;East_Han_AN=6554;East_Ha
chr4	111407506	.	A	<INS:ME:LINE1>	.	PASS	SVTYPE=INS:ME:LINE1;END=111407516;SVLEN=387;AN=20618;AC=1;AF=4.850130953535746e-05;N_HET=1;N_HOMALT=0;N_HOMREF=10308;FREQ_HET=9.700261907071492e-05;FREQ_HOMALT=0.0;FREQ_HOMREF=0.9999029973809292;N_BI_GENOS=10309;Central_Han_AN=2128;Central_Han_AC=1;Centra
chr4	111706105	.	G	<INS:ME:LINE1>	.	PASS	SVTYPE=INS:ME:LINE1;END=111706110;SVLEN=97;AN=20618;AC=2;AF=9.700261907071492e-05;N_HET=2;N_HOMALT=0;N_HOMREF=10307;FREQ_HET=0.00019400523814142983;FREQ_HOMALT=0.0;FREQ_HOMREF=0.9998059947618586;N_BI_GENOS=10309;North_Han_AN=2272;North_Han_AC=2;North_Han_
chr4	111707803	.	T	<INS:ME:LINE1>	.	PASS	SVTYPE=INS:ME:LINE1;END=111707817;SVLEN=5993;AN=20618;AC=8850;AF=0.4292365893879135;N_HET=3890;N_HOMALT=2480;N_HOMREF=3939;FREQ_HET=0.37734018818508097;FREQ_HOMALT=0.24056649529537297;FREQ_HOMREF=0.38209331651954603;N_BI_GENOS=10309;South_Han_AN=1496;Sout
chr4	111795818	.	T	<INS:ME:LINE1>	.	PASS	SVTYPE=INS:ME:LINE1;END=111795832;SVLEN=5928;AN=20618;AC=9;AF=0.0004365117858182171;N_HET=9;N_HOMALT=0;N_HOMREF=10300;FREQ_HET=0.0008730235716364342;FREQ_HOMALT=0.0;FREQ_HOMREF=0.9991269764283636;N_BI_GENOS=10309;Lingnan_Han_AN=1888;Lingnan_Han_AC=1;Lingn
chr4	111850578	.	A	<INS:ME:LINE1>	.	PASS	SVTYPE=INS:ME:LINE1;END=111850589;SVLEN=1653;AN=20618;AC=1;AF=4.850130953535746e-05;N_HET=1;N_HOMALT=0;N_HOMREF=10308;FREQ_HET=9.700261907071492e-05;FREQ_HOMALT=0.0;FREQ_HOMREF=0.9999029973809292;N_BI_GENOS=10309;East_Han_AN=6554;East_Han_AC=1;East_Han_AF
chr4	111852248	.	T	<INS:ME:LINE1>	.	PASS	SVTYPE=INS:ME:LINE1;END=111852248;SVLEN=359;AN=20618;AC=1;AF=4.850130953535746e-05;N_HET=1;N_HOMALT=0;N_HOMREF=10308;FREQ_HET=9.700261907071492e-05;FREQ_HOMALT=0.0;FREQ_HOMREF=0.9999029973809292;N_BI_GENOS=10309;Southeast_Han_AN=1908;Southeast_Han_AC=1;So
chr4	111857064	.	G	<INS:ME:LINE1>	.	PASS	SVTYPE=INS:ME:LINE1;END=111857082;SVLEN=5994;AN=20618;AC=1;AF=4.850130953535746e-05;N_HET=1;N_HOMALT=0;N_HOMREF=10308;FREQ_HET=9.700261907071492e-05;FREQ_HOMALT=0.0;FREQ_HOMREF=0.9999029973809292;N_BI_GENOS=10309;East_Han_AN=6554;East_Han_AC=1;East_Han_AF
chr4	111945244	.	A	<INS:ME:LINE1>	.	PASS	SVTYPE=INS:ME:LINE1;END=111945260;SVLEN=811;AN=20618;AC=15;AF=0.0007275196430303619;N_HET=15;N_HOMALT=0;N_HOMREF=10294;FREQ_HET=0.0014550392860607237;FREQ_HOMALT=0.0;FREQ_HOMREF=0.9985449607139393;N_BI_GENOS=10309;South_Han_AN=1496;South_Han_AC=2;South_Ha
chr4	112226010	.	C	<INS:ME:LINE1>	.	PASS	SVTYPE=INS:ME:LINE1;END=112226023;SVLEN=5997;AN=20618;AC=1;AF=4.850130953535746e-05;N_HET=1;N_HOMALT=0;N_HOMREF=10308;FREQ_HET=9.700261907071492e-05;FREQ_HOMALT=0.0;FREQ_HOMREF=0.9999029973809292;N_BI_GENOS=10309;East_Han_AN=6554;East_Han_AC=1;East_Han_AF
chr4	112920715	.	T	<INS:ME:LINE1>	.	PASS	SVTYPE=INS:ME:LINE1;END=112920731;SVLEN=5999;AN=20618;AC=1;AF=4.850130953535746e-05;N_HET=1;N_HOMALT=0;N_HOMREF=10308;FREQ_HET=9.700261907071492e-05;FREQ_HOMALT=0.0;FREQ_HOMREF=0.9999029973809292;N_BI_GENOS=10309;Central_Han_AN=2128;Central_Han_AC=1;Centr
chr4	113258692	.	A	<INS:ME:LINE1>	.	PASS	SVTYPE=INS:ME:LINE1;END=113258709;SVLEN=1013;AN=20618;AC=1;AF=4.850130953535746e-05;N_HET=1;N_HOMALT=0;N_HOMREF=10308;FREQ_HET=9.700261907071492e-05;FREQ_HOMALT=0.0;FREQ_HOMREF=0.9999029973809292;N_BI_GENOS=10309;Central_Han_AN=2128;Central_Han_AC=1;Centr
chr4	113705480	.	T	<INS:ME:LINE1>	.	PASS	SVTYPE=INS:ME:LINE1;END=113705494;SVLEN=773;AN=20618;AC=1;AF=4.850130953535746e-05;N_HET=1;N_HOMALT=0;N_HOMREF=10308;FREQ_HET=9.700261907071492e-05;FREQ_HOMALT=0.0;FREQ_HOMREF=0.9999029973809292;N_BI_GENOS=10309;Northwest_Han_AN=1296;Northwest_Han_AC=1;No
chr4	113858787	.	G	<INS:ME:LINE1>	.	PASS	SVTYPE=INS:ME:LINE1;END=113858787;SVLEN=983;AN=20618;AC=2;AF=9.700261907071492e-05;N_HET=2;N_HOMALT=0;N_HOMREF=10307;FREQ_HET=0.00019400523814142983;FREQ_HOMALT=0.0;FREQ_HOMREF=0.9998059947618586;N_BI_GENOS=10309;East_Han_AN=6554;East_Han_AC=2;East_Han_AF
chr4	113876931	.	A	<INS:ME:LINE1>	.	PASS	SVTYPE=INS:ME:LINE1;END=113876931;SVLEN=0;AN=20618;AC=1;AF=4.850130953535746e-05;N_HET=1;N_HOMALT=0;N_HOMREF=10308;FREQ_HET=9.700261907071492e-05;FREQ_HOMALT=0.0;FREQ_HOMREF=0.9999029973809292;N_BI_GENOS=10309;Northwest_Han_AN=1296;Northwest_Han_AC=1;Nort
chr4	113926211	.	T	<INS:ME:LINE1>	.	PASS	SVTYPE=INS:ME:LINE1;END=113926227;SVLEN=3600;AN=20618;AC=2;AF=9.700261907071492e-05;N_HET=2;N_HOMALT=0;N_HOMREF=10307;FREQ_HET=0.00019400523814142983;FREQ_HOMALT=0.0;FREQ_HOMREF=0.9998059947618586;N_BI_GENOS=10309;Lingnan_Han_AN=1888;Lingnan_Han_AC=1;Ling
chr4	114005757	.	A	<INS:ME:LINE1>	.	PASS	SVTYPE=INS:ME:LINE1;END=114005758;SVLEN=1798;AN=20618;AC=3;AF=0.00014550392860607236;N_HET=3;N_HOMALT=0;N_HOMREF=10306;FREQ_HET=0.0002910078572121447;FREQ_HOMALT=0.0;FREQ_HOMREF=0.9997089921427879;N_BI_GENOS=10309;Central_Han_AN=2128;Central_Han_AC=2;Cent
chr4	114075902	.	A	<INS:ME:LINE1>	.	PASS	SVTYPE=INS:ME:LINE1;END=114075902;SVLEN=633;AN=20618;AC=1;AF=4.850130953535746e-05;N_HET=1;N_HOMALT=0;N_HOMREF=10308;FREQ_HET=9.700261907071492e-05;FREQ_HOMALT=0.0;FREQ_HOMREF=0.9999029973809292;N_BI_GENOS=10309;East_Han_AN=6554;East_Han_AC=1;East_Han_AF=
chr4	114085300	.	A	<INS:ME:LINE1>	.	PASS	SVTYPE=INS:ME:LINE1;END=114085300;SVLEN=38;AN=20618;AC=1;AF=4.850130953535746e-05;N_HET=1;N_HOMALT=0;N_HOMREF=10308;FREQ_HET=9.700261907071492e-05;FREQ_HOMALT=0.0;FREQ_HOMREF=0.9999029973809292;N_BI_GENOS=10309;Northwest_Han_AN=1296;Northwest_Han_AC=1;Nor
chr4	114100688	.	A	<INS:ME:LINE1>	.	PASS	SVTYPE=INS:ME:LINE1;END=114100699;SVLEN=1776;AN=20618;AC=1;AF=4.850130953535746e-05;N_HET=1;N_HOMALT=0;N_HOMREF=10308;FREQ_HET=9.700261907071492e-05;FREQ_HOMALT=0.0;FREQ_HOMREF=0.9999029973809292;N_BI_GENOS=10309;East_Han_AN=6554;East_Han_AC=1;East_Han_AF
chr4	114211538	.	A	<INS:ME:LINE1>	.	PASS	SVTYPE=INS:ME:LINE1;END=114211547;SVLEN=2367;AN=20618;AC=6924;AF=0.335823067222815;N_HET=4136;N_HOMALT=1394;N_HOMREF=4779;FREQ_HET=0.40120283247647687;FREQ_HOMALT=0.13522165098457659;FREQ_HOMREF=0.4635755165389466;N_BI_GENOS=10309;South_Han_AN=1496;South_
chr4	114409676	.	A	<INS:ME:LINE1>	.	PASS	SVTYPE=INS:ME:LINE1;END=114409691;SVLEN=6007;AN=20618;AC=2;AF=9.700261907071492e-05;N_HET=2;N_HOMALT=0;N_HOMREF=10307;FREQ_HET=0.00019400523814142983;FREQ_HOMALT=0.0;FREQ_HOMREF=0.9998059947618586;N_BI_GENOS=10309;Tibetan_AN=414;Tibetan_AC=2;Tibetan_AF=0.
chr4	114450296	.	C	<INS:ME:LINE1>	.	PASS	SVTYPE=INS:ME:LINE1;END=114450325;SVLEN=3907;AN=20618;AC=3;AF=0.00014550392860607236;N_HET=1;N_HOMALT=1;N_HOMREF=10307;FREQ_HET=9.700261907071492e-05;FREQ_HOMALT=9.700261907071492e-05;FREQ_HOMREF=0.9998059947618586;N_BI_GENOS=10309;East_Han_AN=6554;East_H
chr4	114451731	.	A	<INS:ME:LINE1>	.	PASS	SVTYPE=INS:ME:LINE1;END=114451731;SVLEN=511;AN=20618;AC=1;AF=4.850130953535746e-05;N_HET=1;N_HOMALT=0;N_HOMREF=10308;FREQ_HET=9.700261907071492e-05;FREQ_HOMALT=0.0;FREQ_HOMREF=0.9999029973809292;N_BI_GENOS=10309;East_Han_AN=6554;East_Han_AC=1;East_Han_AF=
chr4	114605580	.	C	<INS:ME:LINE1>	.	PASS	SVTYPE=INS:ME:LINE1;END=114605597;SVLEN=6000;AN=20618;AC=14;AF=0.0006790183334950044;N_HET=14;N_HOMALT=0;N_HOMREF=10295;FREQ_HET=0.0013580366669900088;FREQ_HOMALT=0.0;FREQ_HOMREF=0.99864196333301;N_BI_GENOS=10309;South_Han_AN=1496;South_Han_AC=1;South_Han
chr4	114689313	.	A	<INS:ME:LINE1>	.	PASS	SVTYPE=INS:ME:LINE1;END=114689328;SVLEN=408;AN=20618;AC=1;AF=4.850130953535746e-05;N_HET=1;N_HOMALT=0;N_HOMREF=10308;FREQ_HET=9.700261907071492e-05;FREQ_HOMALT=0.0;FREQ_HOMREF=0.9999029973809292;N_BI_GENOS=10309;Mongolian_AN=454;Mongolian_AC=1;Mongolian_A
chr4	114811191	.	A	<INS:ME:LINE1>	.	PASS	SVTYPE=INS:ME:LINE1;END=114811206;SVLEN=6012;AN=20618;AC=3;AF=0.00014550392860607236;N_HET=3;N_HOMALT=0;N_HOMREF=10306;FREQ_HET=0.0002910078572121447;FREQ_HOMALT=0.0;FREQ_HOMREF=0.9997089921427879;N_BI_GENOS=10309;North_Han_AN=2272;North_Han_AC=2;North_Ha
chr4	114836255	.	T	<INS:ME:LINE1>	.	PASS	SVTYPE=INS:ME:LINE1;END=114836270;SVLEN=653;AN=20618;AC=1;AF=4.850130953535746e-05;N_HET=1;N_HOMALT=0;N_HOMREF=10308;FREQ_HET=9.700261907071492e-05;FREQ_HOMALT=0.0;FREQ_HOMREF=0.9999029973809292;N_BI_GENOS=10309;East_Han_AN=6554;East_Han_AC=1;East_Han_AF=
chr4	114972559	.	C	<INS:ME:LINE1>	.	PASS	SVTYPE=INS:ME:LINE1;END=114972561;SVLEN=1616;AN=20618;AC=3;AF=0.00014550392860607236;N_HET=3;N_HOMALT=0;N_HOMREF=10306;FREQ_HET=0.0002910078572121447;FREQ_HOMALT=0.0;FREQ_HOMREF=0.9997089921427879;N_BI_GENOS=10309;East_Han_AN=6554;East_Han_AC=1;East_Han_A
chr4	115018368	.	T	<INS:ME:LINE1>	.	PASS	SVTYPE=INS:ME:LINE1;END=115018384;SVLEN=879;AN=20618;AC=32;AF=0.0015520419051314386;N_HET=32;N_HOMALT=0;N_HOMREF=10277;FREQ_HET=0.0031040838102628773;FREQ_HOMALT=0.0;FREQ_HOMREF=0.9968959161897372;N_BI_GENOS=10309;South_Han_AN=1496;South_Han_AC=3;South_Ha
chr4	115287248	.	A	<INS:ME:LINE1>	.	PASS	SVTYPE=INS:ME:LINE1;END=115287248;SVLEN=29;AN=20618;AC=1;AF=4.850130953535746e-05;N_HET=1;N_HOMALT=0;N_HOMREF=10308;FREQ_HET=9.700261907071492e-05;FREQ_HOMALT=0.0;FREQ_HOMREF=0.9999029973809292;N_BI_GENOS=10309;East_Han_AN=6554;East_Han_AC=1;East_Han_AF=0
chr4	115342019	.	A	<INS:ME:LINE1>	.	PASS	SVTYPE=INS:ME:LINE1;END=115342030;SVLEN=2171;AN=20618;AC=2;AF=9.700261907071492e-05;N_HET=2;N_HOMALT=0;N_HOMREF=10307;FREQ_HET=0.00019400523814142983;FREQ_HOMALT=0.0;FREQ_HOMREF=0.9998059947618586;N_BI_GENOS=10309;Lingnan_Han_AN=1888;Lingnan_Han_AC=1;Ling
chr4	115475335	.	A	<INS:ME:LINE1>	.	PASS	SVTYPE=INS:ME:LINE1;END=115475347;SVLEN=6000;AN=20618;AC=1;AF=4.850130953535746e-05;N_HET=1;N_HOMALT=0;N_HOMREF=10308;FREQ_HET=9.700261907071492e-05;FREQ_HOMALT=0.0;FREQ_HOMREF=0.9999029973809292;N_BI_GENOS=10309;Southeast_Han_AN=1908;Southeast_Han_AC=1;S
chr4	115579610	.	A	<INS:ME:LINE1>	.	PASS	SVTYPE=INS:ME:LINE1;END=115579624;SVLEN=514;AN=20618;AC=1;AF=4.850130953535746e-05;N_HET=1;N_HOMALT=0;N_HOMREF=10308;FREQ_HET=9.700261907071492e-05;FREQ_HOMALT=0.0;FREQ_HOMREF=0.9999029973809292;N_BI_GENOS=10309;South_Han_AN=1496;South_Han_AC=1;South_Han_
chr4	115738995	.	A	<INS:ME:LINE1>	.	PASS	SVTYPE=INS:ME:LINE1;END=115739006;SVLEN=6002;AN=20618;AC=1;AF=4.850130953535746e-05;N_HET=1;N_HOMALT=0;N_HOMREF=10308;FREQ_HET=9.700261907071492e-05;FREQ_HOMALT=0.0;FREQ_HOMREF=0.9999029973809292;N_BI_GENOS=10309;Mongolian_AN=454;Mongolian_AC=1;Mongolian_
chr4	115757155	.	A	<INS:ME:LINE1>	.	PASS	SVTYPE=INS:ME:LINE1;END=115757159;SVLEN=1215;AN=20618;AC=1;AF=4.850130953535746e-05;N_HET=1;N_HOMALT=0;N_HOMREF=10308;FREQ_HET=9.700261907071492e-05;FREQ_HOMALT=0.0;FREQ_HOMREF=0.9999029973809292;N_BI_GENOS=10309;North_Han_AN=2272;North_Han_AC=1;North_Han
chr4	116374225	.	C	<INS:ME:LINE1>	.	PASS	SVTYPE=INS:ME:LINE1;END=116374244;SVLEN=928;AN=20618;AC=2;AF=9.700261907071492e-05;N_HET=2;N_HOMALT=0;N_HOMREF=10307;FREQ_HET=0.00019400523814142983;FREQ_HOMALT=0.0;FREQ_HOMREF=0.9998059947618586;N_BI_GENOS=10309;East_Han_AN=6554;East_Han_AC=1;East_Han_AF
chr4	116638256	.	C	<INS:ME:LINE1>	.	PASS	SVTYPE=INS:ME:LINE1;END=116638256;SVLEN=832;AN=20618;AC=1;AF=4.850130953535746e-05;N_HET=1;N_HOMALT=0;N_HOMREF=10308;FREQ_HET=9.700261907071492e-05;FREQ_HOMALT=0.0;FREQ_HOMREF=0.9999029973809292;N_BI_GENOS=10309;East_Han_AN=6554;East_Han_AC=1;East_Han_AF=
chr4	116981170	.	T	<INS:ME:LINE1>	.	PASS	SVTYPE=INS:ME:LINE1;END=116981170;SVLEN=103;AN=20618;AC=2;AF=9.700261907071492e-05;N_HET=2;N_HOMALT=0;N_HOMREF=10307;FREQ_HET=0.00019400523814142983;FREQ_HOMALT=0.0;FREQ_HOMREF=0.9998059947618586;N_BI_GENOS=10309;East_Han_AN=6554;East_Han_AC=2;East_Han_AF
chr4	117074882	.	A	<INS:ME:LINE1>	.	PASS	SVTYPE=INS:ME:LINE1;END=117074895;SVLEN=357;AN=20618;AC=1;AF=4.850130953535746e-05;N_HET=1;N_HOMALT=0;N_HOMREF=10308;FREQ_HET=9.700261907071492e-05;FREQ_HOMALT=0.0;FREQ_HOMREF=0.9999029973809292;N_BI_GENOS=10309;Lingnan_Han_AN=1888;Lingnan_Han_AC=1;Lingna
chr4	117211338	.	A	<INS:ME:LINE1>	.	PASS	SVTYPE=INS:ME:LINE1;END=117211355;SVLEN=295;AN=20618;AC=1;AF=4.850130953535746e-05;N_HET=1;N_HOMALT=0;N_HOMREF=10308;FREQ_HET=9.700261907071492e-05;FREQ_HOMALT=0.0;FREQ_HOMREF=0.9999029973809292;N_BI_GENOS=10309;Northwest_Han_AN=1296;Northwest_Han_AC=1;No
chr4	117330519	.	G	<INS:ME:LINE1>	.	PASS	SVTYPE=INS:ME:LINE1;END=117330534;SVLEN=856;AN=20618;AC=1;AF=4.850130953535746e-05;N_HET=1;N_HOMALT=0;N_HOMREF=10308;FREQ_HET=9.700261907071492e-05;FREQ_HOMALT=0.0;FREQ_HOMREF=0.9999029973809292;N_BI_GENOS=10309;Northwest_Han_AN=1296;Northwest_Han_AC=1;No
chr4	117411240	.	T	<INS:ME:LINE1>	.	PASS	SVTYPE=INS:ME:LINE1;END=117411255;SVLEN=911;AN=20618;AC=12;AF=0.0005820157144242894;N_HET=12;N_HOMALT=0;N_HOMREF=10297;FREQ_HET=0.0011640314288485789;FREQ_HOMALT=0.0;FREQ_HOMREF=0.9988359685711514;N_BI_GENOS=10309;South_Han_AN=1496;South_Han_AC=2;South_Ha
chr4	117430392	.	G	<INS:ME:LINE1>	.	PASS	SVTYPE=INS:ME:LINE1;END=117430407;SVLEN=1298;AN=20618;AC=2;AF=9.700261907071492e-05;N_HET=2;N_HOMALT=0;N_HOMREF=10307;FREQ_HET=0.00019400523814142983;FREQ_HOMALT=0.0;FREQ_HOMREF=0.9998059947618586;N_BI_GENOS=10309;Northwest_Han_AN=1296;Northwest_Han_AC=1;
chr4	117641563	.	A	<INS:ME:LINE1>	.	PASS	SVTYPE=INS:ME:LINE1;END=117641583;SVLEN=964;AN=20618;AC=1;AF=4.850130953535746e-05;N_HET=1;N_HOMALT=0;N_HOMREF=10308;FREQ_HET=9.700261907071492e-05;FREQ_HOMALT=0.0;FREQ_HOMREF=0.9999029973809292;N_BI_GENOS=10309;East_Han_AN=6554;East_Han_AC=1;East_Han_AF=
chr4	117647523	.	C	<INS:ME:LINE1>	.	PASS	SVTYPE=INS:ME:LINE1;END=117647523;SVLEN=25;AN=20618;AC=1;AF=4.850130953535746e-05;N_HET=1;N_HOMALT=0;N_HOMREF=10308;FREQ_HET=9.700261907071492e-05;FREQ_HOMALT=0.0;FREQ_HOMREF=0.9999029973809292;N_BI_GENOS=10309;East_Han_AN=6554;East_Han_AC=1;East_Han_AF=0
chr4	117844099	.	A	<INS:ME:LINE1>	.	PASS	SVTYPE=INS:ME:LINE1;END=117844104;SVLEN=1697;AN=20618;AC=2759;AF=0.1338151130080512;N_HET=2183;N_HOMALT=288;N_HOMREF=7838;FREQ_HET=0.21175671743137064;FREQ_HOMALT=0.027936754292365893;FREQ_HOMREF=0.7603065282762634;N_BI_GENOS=10309;South_Han_AN=1496;South
chr4	117847472	.	C	<INS:ME:LINE1>	.	PASS	SVTYPE=INS:ME:LINE1;END=117847489;SVLEN=6000;AN=20618;AC=9;AF=0.0004365117858182171;N_HET=9;N_HOMALT=0;N_HOMREF=10300;FREQ_HET=0.0008730235716364342;FREQ_HOMALT=0.0;FREQ_HOMREF=0.9991269764283636;N_BI_GENOS=10309;South_Han_AN=1496;South_Han_AC=1;South_Han
chr4	117973354	.	G	<INS:ME:LINE1>	.	PASS	SVTYPE=INS:ME:LINE1;END=117973373;SVLEN=304;AN=20618;AC=1;AF=4.850130953535746e-05;N_HET=1;N_HOMALT=0;N_HOMREF=10308;FREQ_HET=9.700261907071492e-05;FREQ_HOMALT=0.0;FREQ_HOMREF=0.9999029973809292;N_BI_GENOS=10309;Tibetan_AN=414;Tibetan_AC=1;Tibetan_AF=0.00
chr4	117981808	.	T	<INS:ME:LINE1>	.	PASS	SVTYPE=INS:ME:LINE1;END=117981836;SVLEN=1043;AN=20618;AC=1;AF=4.850130953535746e-05;N_HET=1;N_HOMALT=0;N_HOMREF=10308;FREQ_HET=9.700261907071492e-05;FREQ_HOMALT=0.0;FREQ_HOMREF=0.9999029973809292;N_BI_GENOS=10309;East_Han_AN=6554;East_Han_AC=1;East_Han_AF
chr4	118023219	.	A	<INS:ME:LINE1>	.	PASS	SVTYPE=INS:ME:LINE1;END=118023234;SVLEN=6072;AN=20618;AC=1;AF=4.850130953535746e-05;N_HET=1;N_HOMALT=0;N_HOMREF=10308;FREQ_HET=9.700261907071492e-05;FREQ_HOMALT=0.0;FREQ_HOMREF=0.9999029973809292;N_BI_GENOS=10309;Southeast_Han_AN=1908;Southeast_Han_AC=1;S
chr4	118262532	.	G	<INS:ME:LINE1>	.	PASS	SVTYPE=INS:ME:LINE1;END=118262550;SVLEN=5994;AN=20618;AC=1;AF=4.850130953535746e-05;N_HET=1;N_HOMALT=0;N_HOMREF=10308;FREQ_HET=9.700261907071492e-05;FREQ_HOMALT=0.0;FREQ_HOMREF=0.9999029973809292;N_BI_GENOS=10309;East_Han_AN=6554;East_Han_AC=1;East_Han_AF
chr4	118270823	.	T	<INS:ME:LINE1>	.	PASS	SVTYPE=INS:ME:LINE1;END=118270842;SVLEN=2428;AN=20618;AC=1;AF=4.850130953535746e-05;N_HET=1;N_HOMALT=0;N_HOMREF=10308;FREQ_HET=9.700261907071492e-05;FREQ_HOMALT=0.0;FREQ_HOMREF=0.9999029973809292;N_BI_GENOS=10309;South_Han_AN=1496;South_Han_AC=1;South_Han
chr4	118648113	.	G	<INS:ME:LINE1>	.	PASS	SVTYPE=INS:ME:LINE1;END=118648132;SVLEN=749;AN=20618;AC=1;AF=4.850130953535746e-05;N_HET=1;N_HOMALT=0;N_HOMREF=10308;FREQ_HET=9.700261907071492e-05;FREQ_HOMALT=0.0;FREQ_HOMREF=0.9999029973809292;N_BI_GENOS=10309;Central_Han_AN=2128;Central_Han_AC=1;Centra
chr4	118896466	.	A	<INS:ME:LINE1>	.	PASS	SVTYPE=INS:ME:LINE1;END=118896473;SVLEN=105;AN=20618;AC=1;AF=4.850130953535746e-05;N_HET=1;N_HOMALT=0;N_HOMREF=10308;FREQ_HET=9.700261907071492e-05;FREQ_HOMALT=0.0;FREQ_HOMREF=0.9999029973809292;N_BI_GENOS=10309;Lingnan_Han_AN=1888;Lingnan_Han_AC=1;Lingna
chr4	119306064	.	A	<INS:ME:LINE1>	.	PASS	SVTYPE=INS:ME:LINE1;END=119306078;SVLEN=6000;AN=20618;AC=1;AF=4.850130953535746e-05;N_HET=1;N_HOMALT=0;N_HOMREF=10308;FREQ_HET=9.700261907071492e-05;FREQ_HOMALT=0.0;FREQ_HOMREF=0.9999029973809292;N_BI_GENOS=10309;North_Han_AN=2272;North_Han_AC=1;North_Han
chr4	119518983	.	A	<INS:ME:LINE1>	.	PASS	SVTYPE=INS:ME:LINE1;END=119519000;SVLEN=763;AN=20618;AC=1;AF=4.850130953535746e-05;N_HET=1;N_HOMALT=0;N_HOMREF=10308;FREQ_HET=9.700261907071492e-05;FREQ_HOMALT=0.0;FREQ_HOMREF=0.9999029973809292;N_BI_GENOS=10309;North_Han_AN=2272;North_Han_AC=1;North_Han_
chr4	119554201	.	T	<INS:ME:LINE1>	.	PASS	SVTYPE=INS:ME:LINE1;END=119554213;SVLEN=416;AN=20618;AC=4;AF=0.00019400523814142983;N_HET=4;N_HOMALT=0;N_HOMREF=10305;FREQ_HET=0.00038801047628285966;FREQ_HOMALT=0.0;FREQ_HOMREF=0.9996119895237171;N_BI_GENOS=10309;Lingnan_Han_AN=1888;Lingnan_Han_AC=1;Ling
chr4	119554329	.	G	<INS:ME:LINE1>	.	PASS	SVTYPE=INS:ME:LINE1;END=119554339;SVLEN=835;AN=20618;AC=1;AF=4.850130953535746e-05;N_HET=1;N_HOMALT=0;N_HOMREF=10308;FREQ_HET=9.700261907071492e-05;FREQ_HOMALT=0.0;FREQ_HOMREF=0.9999029973809292;N_BI_GENOS=10309;Central_Han_AN=2128;Central_Han_AC=1;Centra
chr4	119796374	.	T	<INS:ME:LINE1>	.	PASS	SVTYPE=INS:ME:LINE1;END=119796389;SVLEN=527;AN=20618;AC=1020;AF=0.0494713357260646;N_HET=870;N_HOMALT=75;N_HOMREF=9364;FREQ_HET=0.08439227859152197;FREQ_HOMALT=0.007275196430303618;FREQ_HOMREF=0.9083325249781744;N_BI_GENOS=10309;South_Han_AN=1496;South_H
chr4	119941181	.	G	<INS:ME:LINE1>	.	PASS	SVTYPE=INS:ME:LINE1;END=119941181;SVLEN=1797;AN=20618;AC=1;AF=4.850130953535746e-05;N_HET=1;N_HOMALT=0;N_HOMREF=10308;FREQ_HET=9.700261907071492e-05;FREQ_HOMALT=0.0;FREQ_HOMREF=0.9999029973809292;N_BI_GENOS=10309;Southeast_Han_AN=1908;Southeast_Han_AC=1;S
chr4	120379202	.	A	<INS:ME:LINE1>	.	PASS	SVTYPE=INS:ME:LINE1;END=120379212;SVLEN=884;AN=20618;AC=2;AF=9.700261907071492e-05;N_HET=2;N_HOMALT=0;N_HOMREF=10307;FREQ_HET=0.00019400523814142983;FREQ_HOMALT=0.0;FREQ_HOMREF=0.9998059947618586;N_BI_GENOS=10309;South_Han_AN=1496;South_Han_AC=1;South_Han
chr4	120384279	.	A	<INS:ME:LINE1>	.	PASS	SVTYPE=INS:ME:LINE1;END=120384293;SVLEN=844;AN=20618;AC=9;AF=0.0004365117858182171;N_HET=7;N_HOMALT=1;N_HOMREF=10301;FREQ_HET=0.0006790183334950044;FREQ_HOMALT=9.700261907071492e-05;FREQ_HOMREF=0.9992239790474343;N_BI_GENOS=10309;Lingnan_Han_AN=1888;Lingn
chr4	120415568	.	T	<INS:ME:LINE1>	.	PASS	SVTYPE=INS:ME:LINE1;END=120415569;SVLEN=1554;AN=20618;AC=1;AF=4.850130953535746e-05;N_HET=1;N_HOMALT=0;N_HOMREF=10308;FREQ_HET=9.700261907071492e-05;FREQ_HOMALT=0.0;FREQ_HOMREF=0.9999029973809292;N_BI_GENOS=10309;East_Han_AN=6554;East_Han_AC=1;East_Han_AF
chr4	120471730	.	T	<INS:ME:LINE1>	.	PASS	SVTYPE=INS:ME:LINE1;END=120471732;SVLEN=2324;AN=20618;AC=41;AF=0.001988553690949 6557;N_HET=37;N_HOMALT=2;N_HOMREF=10270;FREQ_HET=0.0035890969056164516;FREQ_HOMALT=0.00019400523814142983;FREQ_HOMREF=0.9962168978562421;N_BI_GENOS=10309;South_Han_AN=1496;Sou
chr4	120473173	.	T	<INS:ME:LINE1>	.	PASS	SVTYPE=INS:ME:LINE1;END=120473173;SVLEN=107;AN=20618;AC=3;AF=0.00014550392860607236;N_HET=3;N_HOMALT=0;N_HOMREF=10306;FREQ_HET=0.0002910078572121447;FREQ_HOMALT=0.0;FREQ_HOMREF=0.9997089921427879;N_BI_GENOS=10309;East_Han_AN=6554;East_Han_AC=2;East_Han_AF
chr4	120576175	.	C	<INS:ME:LINE1>	.	PASS	SVTYPE=INS:ME:LINE1;END=120576214;SVLEN=1736;AN=20618;AC=1;AF=4.850130953535746e-05;N_HET=1;N_HOMALT=0;N_HOMREF=10308;FREQ_HET=9.700261907071492e-05;FREQ_HOMALT=0.0;FREQ_HOMREF=0.9999029973809292;N_BI_GENOS=10309;East_Han_AN=6554;East_Han_AC=1;East_Han_AF
chr4	120576868	.	G	<INS:ME:LINE1>	.	PASS	SVTYPE=INS:ME:LINE1;END=120576904;SVLEN=704;AN=20618;AC=1;AF=4.850130953535746e-05;N_HET=1;N_HOMALT=0;N_HOMREF=10308;FREQ_HET=9.700261907071492e-05;FREQ_HOMALT=0.0;FREQ_HOMREF=0.9999029973809292;N_BI_GENOS=10309;East_Han_AN=6554;East_Han_AC=1;East_Han_AF=
chr4	120596913	.	A	<INS:ME:LINE1>	.	PASS	SVTYPE=INS:ME:LINE1;END=120596938;SVLEN=2661;AN=20618;AC=11;AF=0.000533514404888932;N_HET=9;N_HOMALT=1;N_HOMREF=10299;FREQ_HET=0.0008730235716364342;FREQ_HOMALT=9.700261907071492e-05;FREQ_HOMREF=0.9990299738092928;N_BI_GENOS=10309;South_Han_AN=1496;South_
chr4	120762113	.	A	<INS:ME:LINE1>	.	PASS	SVTYPE=INS:ME:LINE1;END=120762130;SVLEN=5998;AN=20618;AC=1;AF=4.850130953535746e-05;N_HET=1;N_HOMALT=0;N_HOMREF=10308;FREQ_HET=9.700261907071492e-05;FREQ_HOMALT=0.0;FREQ_HOMREF=0.9999029973809292;N_BI_GENOS=10309;North_Han_AN=2272;North_Han_AC=1;North_Han
chr4	120801054	.	G	<INS:ME:LINE1>	.	PASS	SVTYPE=INS:ME:LINE1;END=120801054;SVLEN=985;AN=20618;AC=1;AF=4.850130953535746e-05;N_HET=1;N_HOMALT=0;N_HOMREF=10308;FREQ_HET=9.700261907071492e-05;FREQ_HOMALT=0.0;FREQ_HOMREF=0.9999029973809292;N_BI_GENOS=10309;Southeast_Han_AN=1908;Southeast_Han_AC=1;So
chr4	121102353	.	A	<INS:ME:LINE1>	.	PASS	SVTYPE=INS:ME:LINE1;END=121102354;SVLEN=646;AN=20618;AC=39;AF=0.0018915510718789407;N_HET=35;N_HOMALT=2;N_HOMREF=10272;FREQ_HET=0.0033950916674750217;FREQ_HOMALT=0.00019400523814142983;FREQ_HOMREF=0.9964109030943835;N_BI_GENOS=10309;South_Han_AN=1496;Sout
chr4	121682449	.	C	<INS:ME:LINE1>	.	PASS	SVTYPE=INS:ME:LINE1;END=121682462;SVLEN=5984;AN=20618;AC=1;AF=4.850130953535746e-05;N_HET=1;N_HOMALT=0;N_HOMREF=10308;FREQ_HET=9.700261907071492e-05;FREQ_HOMALT=0.0;FREQ_HOMREF=0.9999029973809292;N_BI_GENOS=10309;East_Han_AN=6554;East_Han_AC=1;East_Han_AF
chr4	122009760	.	A	<INS:ME:LINE1>	.	PASS	SVTYPE=INS:ME:LINE1;END=122009777;SVLEN=641;AN=20618;AC=1;AF=4.850130953535746e-05;N_HET=1;N_HOMALT=0;N_HOMREF=10308;FREQ_HET=9.700261907071492e-05;FREQ_HOMALT=0.0;FREQ_HOMREF=0.9999029973809292;N_BI_GENOS=10309;Mongolian_AN=454;Mongolian_AC=1;Mongolian_A
chr4	122044333	.	C	<INS:ME:LINE1>	.	PASS	SVTYPE=INS:ME:LINE1;END=122044345;SVLEN=6016;AN=20618;AC=1;AF=4.850130953535746e-05;N_HET=1;N_HOMALT=0;N_HOMREF=10308;FREQ_HET=9.700261907071492e-05;FREQ_HOMALT=0.0;FREQ_HOMREF=0.9999029973809292;N_BI_GENOS=10309;Lingnan_Han_AN=1888;Lingnan_Han_AC=1;Lingn
chr4	122261664	.	G	<INS:ME:LINE1>	.	PASS	SVTYPE=INS:ME:LINE1;END=122261664;SVLEN=0;AN=20618;AC=2;AF=9.700261907071492e-05;N_HET=2;N_HOMALT=0;N_HOMREF=10307;FREQ_HET=0.00019400523814142983;FREQ_HOMALT=0.0;FREQ_HOMREF=0.9998059947618586;N_BI_GENOS=10309;Southeast_Han_AN=1908;Southeast_Han_AC=2;Sou
chr4	122324290	.	G	<INS:ME:LINE1>	.	PASS	SVTYPE=INS:ME:LINE1;END=122324303;SVLEN=1794;AN=20618;AC=8;AF=0.00038801047628285966;N_HET=8;N_HOMALT=0;N_HOMREF=10301;FREQ_HET=0.0007760209525657193;FREQ_HOMALT=0.0;FREQ_HOMREF=0.9992239790474343;N_BI_GENOS=10309;North_Han_AN=2272;North_Han_AC=1;North_Ha
chr4	122431644	.	G	<INS:ME:LINE1>	.	PASS	SVTYPE=INS:ME:LINE1;END=122431648;SVLEN=897;AN=20618;AC=1;AF=4.850130953535746e-05;N_HET=1;N_HOMALT=0;N_HOMREF=10308;FREQ_HET=9.700261907071492e-05;FREQ_HOMALT=0.0;FREQ_HOMREF=0.9999029973809292;N_BI_GENOS=10309;Mongolian_AN=454;Mongolian_AC=1;Mongolian_A
chr4	122511677	.	A	<INS:ME:LINE1>	.	PASS	SVTYPE=INS:ME:LINE1;END=122511695;SVLEN=863;AN=20618;AC=1;AF=4.850130953535746e-05;N_HET=1;N_HOMALT=0;N_HOMREF=10308;FREQ_HET=9.700261907071492e-05;FREQ_HOMALT=0.0;FREQ_HOMREF=0.9999029973809292;N_BI_GENOS=10309;South_Han_AN=1496;South_Han_AC=1;South_Han_
chr4	122814920	.	A	<INS:ME:LINE1>	.	PASS	SVTYPE=INS:ME:LINE1;END=122814935;SVLEN=5998;AN=20618;AC=1;AF=4.850130953535746e-05;N_HET=1;N_HOMALT=0;N_HOMREF=10308;FREQ_HET=9.700261907071492e-05;FREQ_HOMALT=0.0;FREQ_HOMREF=0.9999029973809292;N_BI_GENOS=10309;Lingnan_Han_AN=1888;Lingnan_Han_AC=1;Lingn
chr4	123412707	.	G	<INS:ME:LINE1>	.	PASS	SVTYPE=INS:ME:LINE1;END=123412720;SVLEN=6009;AN=20618;AC=2;AF=9.700261907071492e-05;N_HET=2;N_HOMALT=0;N_HOMREF=10307;FREQ_HET=0.00019400523814142983;FREQ_HOMALT=0.0;FREQ_HOMREF=0.9998059947618586;N_BI_GENOS=10309;East_Han_AN=6554;East_Han_AC=1;East_Han_A
chr4	123704452	.	A	<INS:ME:LINE1>	.	PASS	SVTYPE=INS:ME:LINE1;END=123704456;SVLEN=687;AN=20618;AC=2;AF=9.700261907071492e-05;N_HET=2;N_HOMALT=0;N_HOMREF=10307;FREQ_HET=0.00019400523814142983;FREQ_HOMALT=0.0;FREQ_HOMREF=0.9998059947618586;N_BI_GENOS=10309;Lingnan_Han_AN=1888;Lingnan_Han_AC=1;Lingn
chr4	123798774	.	T	<INS:ME:LINE1>	.	PASS	SVTYPE=INS:ME:LINE1;END=123798775;SVLEN=1508;AN=20618;AC=1;AF=4.850130953535746e-05;N_HET=1;N_HOMALT=0;N_HOMREF=10308;FREQ_HET=9.700261907071492e-05;FREQ_HOMALT=0.0;FREQ_HOMREF=0.9999029973809292;N_BI_GENOS=10309;East_Han_AN=6554;East_Han_AC=1;East_Han_AF
chr4	123898040	.	A	<INS:ME:LINE1>	.	PASS	SVTYPE=INS:ME:LINE1;END=123898054;SVLEN=1726;AN=20618;AC=27;AF=0.0013095353574546513;N_HET=27;N_HOMALT=0;N_HOMREF=10282;FREQ_HET=0.0026190707149093026;FREQ_HOMALT=0.0;FREQ_HOMREF=0.997380929285 0907;N_BI_GENOS=10309;South_Han_AN=1496;South_Han_AC=5;South_H

chr4	123936217	.	G	<INS:ME:LINE1>	.	PASS	SVTYPE=INS:ME:LINE1;END=123936224;SVLEN=760;AN=20618;AC=11;AF=0.000533514404888932;N_HET=11;N_HOMALT=0;N_HOMREF=10298;FREQ_HET=0.00106702880977864;FREQ_HOMALT=0.0;FREQ_HOMREF=0.9989329711902222;N_BI_GENOS=10309;South_Han_AN=1496;South_Han_AC=1;South_Han_
chr4	124002987	.	A	<INS:ME:LINE1>	.	PASS	SVTYPE=INS:ME:LINE1;END=124003003;SVLEN=970;AN=20618;AC=1;AF=4.850130953535746e-05;N_HET=1;N_HOMALT=0;N_HOMREF=10308;FREQ_HET=9.700261907071492e-05;FREQ_HOMALT=0.0;FREQ_HOMREF=0.9999029973809292;N_BI_GENOS=10309;Manchu_AN=416;Manchu_AC=1;Manchu_AF=0.00240
chr4	124103077	.	G	<INS:ME:LINE1>	.	PASS	SVTYPE=INS:ME:LINE1;END=124103077;SVLEN=72;AN=20618;AC=1;AF=4.850130953535746e-05;N_HET=1;N_HOMALT=0;N_HOMREF=10308;FREQ_HET=9.700261907071492e-05;FREQ_HOMALT=0.0;FREQ_HOMREF=0.9999029973809292;N_BI_GENOS=10309;East_Han_AN=6554;East_Han_AC=1;East_Han_AF=0
chr4	124183686	.	C	<INS:ME:LINE1>	.	PASS	SVTYPE=INS:ME:LINE1;END=124183702;SVLEN=1832;AN=20618;AC=1;AF=4.850130953535746e-05;N_HET=1;N_HOMALT=0;N_HOMREF=10308;FREQ_HET=9.700261907071492e-05;FREQ_HOMALT=0.0;FREQ_HOMREF=0.9999029973809292;N_BI_GENOS=10309;East_Han_AN=6554;East_Han_AC=1;East_Han_AF
chr4	124262370	.	A	<INS:ME:LINE1>	.	PASS	SVTYPE=INS:ME:LINE1;END=124262370;SVLEN=1492;AN=20618;AC=1;AF=4.850130953535746e-05;N_HET=1;N_HOMALT=0;N_HOMREF=10308;FREQ_HET=9.700261907071492e-05;FREQ_HOMALT=0.0;FREQ_HOMREF=0.9999029973809292;N_BI_GENOS=10309;Central_Han_AN=2128;Central_Han_AC=1;Centr
chr4	124283426	.	G	<INS:ME:LINE1>	.	PASS	SVTYPE=INS:ME:LINE1;END=124283441;SVLEN=5983;AN=20618;AC=1;AF=4.850130953535746e-05;N_HET=1;N_HOMALT=0;N_HOMREF=10308;FREQ_HET=9.700261907071492e-05;FREQ_HOMALT=0.0;FREQ_HOMREF=0.9999029973809292;N_BI_GENOS=10309;Southeast_Han_AN=1908;Southeast_Han_AC=1;S
chr4	124309116	.	A	<INS:ME:LINE1>	.	PASS	SVTYPE=INS:ME:LINE1;END=124309118;SVLEN=65;AN=20618;AC=1447;AF=0.07018139489766223;N_HET=1025;N_HOMALT=211;N_HOMREF=9073;FREQ_HET=0.09942768454748278;FREQ_HOMALT=0.020467552623920846;FREQ_HOMREF=0.880104762885964;N_BI_GENOS=10309;South_Han_AN=1496;South_
chr4	124313613	.	A	<INS:ME:LINE1>	.	PASS	SVTYPE=INS:ME:LINE1;END=124313629;SVLEN=182;AN=20618;AC=10;AF=0.00048501309535357455;N_HET=10;N_HOMALT=0;N_HOMREF=10299;FREQ_HET=0.0009700261907071491;FREQ_HOMALT=0.0;FREQ_HOMREF=0.9990299738092928;N_BI_GENOS=10309;North_Han_AN=2272;North_Han_AC=2;North_H
chr4	124485498	.	A	<INS:ME:LINE1>	.	PASS	SVTYPE=INS:ME:LINE1;END=124485513;SVLEN=322;AN=20618;AC=1;AF=4.850130953535746e-05;N_HET=1;N_HOMALT=0;N_HOMREF=10308;FREQ_HET=9.700261907071492e-05;FREQ_HOMALT=0.0;FREQ_HOMREF=0.9999029973809292;N_BI_GENOS=10309;Yi_AN=422;Yi_AC=1;Yi_AF=0.0023696682464549
chr4	124595766	.	G	<INS:ME:LINE1>	.	PASS	SVTYPE=INS:ME:LINE1;END=124595781;SVLEN=5998;AN=20618;AC=1;AF=4.850130953535746e-05;N_HET=1;N_HOMALT=0;N_HOMREF=10308;FREQ_HET=9.700261907071492e-05;FREQ_HOMALT=0.0;FREQ_HOMREF=0.9999029973809292;N_BI_GENOS=10309;North_Han_AN=2272;North_Han_AC=1;North_Han
chr4	124629142	.	T	<INS:ME:LINE1>	.	PASS	SVTYPE=INS:ME:LINE1;END=124629148;SVLEN=3207;AN=20618;AC=1;AF=4.850130953535746e-05;N_HET=1;N_HOMALT=0;N_HOMREF=10308;FREQ_HET=9.700261907071492e-05;FREQ_HOMALT=0.0;FREQ_HOMREF=0.9999029973809292;N_BI_GENOS=10309;Lingnan_Han_AN=1888;Lingnan_Han_AC=1;Lingn
chr4	124743050	.	A	<INS:ME:LINE1>	.	PASS	SVTYPE=INS:ME:LINE1;END=124743068;SVLEN=6000;AN=20618;AC=1;AF=4.850130953535746e-05;N_HET=1;N_HOMALT=0;N_HOMREF=10308;FREQ_HET=9.700261907071492e-05;FREQ_HOMALT=0.0;FREQ_HOMREF=0.9999029973809292;N_BI_GENOS=10309;Tibetan_AN=414;Tibetan_AC=1;Tibetan_AF=0.0
chr4	124783223	.	T	<INS:ME:LINE1>	.	PASS	SVTYPE=INS:ME:LINE1;END=124783224;SVLEN=645;AN=20618;AC=1;AF=4.850130953535746e-05;N_HET=1;N_HOMALT=0;N_HOMREF=10308;FREQ_HET=9.700261907071492e-05;FREQ_HOMALT=0.0;FREQ_HOMREF=0.9999029973809292;N_BI_GENOS=10309;East_Han_AN=6554;East_Han_AC=1;East_Han_AF=
chr4	125034409	.	A	<INS:ME:LINE1>	.	PASS	SVTYPE=INS:ME:LINE1;END=125034425;SVLEN=6006;AN=20618;AC=2;AF=9.700261907071492e-05;N_HET=0;N_HOMALT=1;N_HOMREF=10308;FREQ_HET=0.0;FREQ_HOMALT=9.700261907071492e-05;FREQ_HOMREF=0.9999029973809292;N_BI_GENOS=10309;East_Han_AN=6554;East_Han_AC=2;East_Han_AF
chr4	125131152	.	T	<INS:ME:LINE1>	.	PASS	SVTYPE=INS:ME:LINE1;END=125131156;SVLEN=1185;AN=20618;AC=339;AF=0.016441943932486176;N_HET=325;N_HOMALT=7;N_HOMREF=9977;FREQ_HET=0.03152585119798234 6;FREQ_HOMALT=0.0006790183334950044;FREQ_HOMREF=0.9677951304685226;N_BI_GENOS=10309;South_Han_AN=1496;South
chr4	125268637	.	C	<INS:ME:LINE1>	.	PASS	SVTYPE=INS:ME:LINE1;END=125268637;SVLEN=419;AN=20618;AC=1;AF=4.850130953535746e-05;N_HET=1;N_HOMALT=0;N_HOMREF=10308;FREQ_HET=9.700261907071492e-05;FREQ_HOMALT=0.0;FREQ_HOMREF=0.9999029973809292;N_BI_GENOS=10309;East_Han_AN=6554;East_Han_AC=1;East_Han_AF=
chr4	125566365	.	G	<INS:ME:LINE1>	.	PASS	SVTYPE=INS:ME:LINE1;END=125566365;SVLEN=0;AN=20618;AC=12;AF=0.0005820157144242894;N_HET=12;N_HOMALT=0;N_HOMREF=10297;FREQ_HET=0.001164031428848578 9;FREQ_HOMALT=0.0;FREQ_HOMREF=0.9988359685711514;N_BI_GENOS=10309;South_Han_AN=1496;South_Han_AC=1;South_Han_
chr4	125566493	.	C	<INS:ME:LINE1>	.	PASS	SVTYPE=INS:ME:LINE1;END=125566493;SVLEN=0;AN=20618;AC=5;AF=0.00024250654767678728;N_HET=5;N_HOMALT=0;N_HOMREF=10304;FREQ_HET=0.00048501309535357455;FREQ_HOMALT=0.0;FREQ_HOMREF=0.9995149869046465;N_BI_GENOS=10309;East_Han_AN=6554;East_Han_AC=3;East_Han_AF=
chr4	125746494	.	A	<INS:ME:LINE1>	.	PASS	SVTYPE=INS:ME:LINE1;END=125746509;SVLEN=5997;AN=20618;AC=17;AF=0.0008245222621010767;N_HET=17;N_HOMALT=0;N_HOMREF=10292;FREQ_HET=0.0016490445242021534;FREQ_HOMALT=0.0;FREQ_HOMREF=0.9983509554757979;N_BI_GENOS=10309;South_Han_AN=1496;South_Han_AC=1;South_H
chr4	125869190	.	G	<INS:ME:LINE1>	.	PASS	SVTYPE=INS:ME:LINE1;END=125869256;SVLEN=1581;AN=20618;AC=1;AF=4.850130953535746e-05;N_HET=1;N_HOMALT=0;N_HOMREF=10308;FREQ_HET=9.700261907071492e-05;FREQ_HOMALT=0.0;FREQ_HOMREF=0.9999029973809292;N_BI_GENOS=10309;Southeast_Han_AN=1908;Southeast_Han_AC=1;S
chr4	125919014	.	A	<INS:ME:LINE1>	.	PASS	SVTYPE=INS:ME:LINE1;END=125919031;SVLEN=6000;AN=20618;AC=3;AF=0.0001455039286060 7236;N_HET=3;N_HOMALT=0;N_HOMREF=10306;FREQ_HET=0.00029100785721214 47;FREQ_HOMALT=0.0;FREQ_HOMREF=0.9997089921427879;N_BI_GENOS=10309;South_Han_AN=1496;South_Han_AC=1;South_Ha
chr4	126122795	.	G	<INS:ME:LINE1>	.	PASS	SVTYPE=INS:ME:LINE1;END=126122800;SVLEN=697;AN=20618;AC=1;AF=4.850130953535746e-05;N_HET=1;N_HOMALT=0;N_HOMREF=10308;FREQ_HET=9.700261907071492e-05;FREQ_HOMALT=0.0;FREQ_HOMREF=0.9999029973809292;N_BI_GENOS=10309;South_Han_AN=1496;South_Han_AC=1;South_Han_
chr4	126155343	.	T	<INS:ME:LINE1>	.	PASS	SVTYPE=INS:ME:LINE1;END=126155343;SVLEN=4971;AN=20618;AC=1;AF=4.850130953535746e-05;N_HET=1;N_HOMALT=0;N_HOMREF=10308;FREQ_HET=9.700261907071492e-05;FREQ_HOMALT=0.0;FREQ_HOMREF=0.9999029973809292;N_BI_GENOS=10309;Central_Han_AN=2128;Central_Han_AC=1;Centr
chr4	126221436	.	G	<INS:ME:LINE1>	.	PASS	SVTYPE=INS:ME:LINE1;END=126221451;SVLEN=5997;AN=20618;AC=21;AF=0.0010185275002425065;N_HET=21;N_HOMALT=0;N_HOMREF=10288;FREQ_HET=0.002037055000485013;FREQ_HOMALT=0.0;FREQ_HOMREF=0.997962944999515;N_BI_GENOS=10309;South_Han_AN=1496;South_Han_AC=2;South_Han
chr4	126253212	.	A	<INS:ME:LINE1>	.	PASS	SVTYPE=INS:ME:LINE1;END=126253225;SVLEN=1227;AN=20618;AC=1;AF=4.850130953535746e-05;N_HET=1;N_HOMALT=0;N_HOMREF=10308;FREQ_HET=9.700261907071492e-05;FREQ_HOMALT=0.0;FREQ_HOMREF=0.9999029973809292;N_BI_GENOS=10309;North_Han_AN=2272;North_Han_AC=1;North_Han
chr4	126541238	.	A	<INS:ME:LINE1>	.	PASS	SVTYPE=INS:ME:LINE1;END=126541253;SVLEN=5999;AN=20618;AC=48;AF=0.0023280628576971578;N_HET=44;N_HOMALT=2;N_HOMREF=10263;FREQ_HET=0.004268115239111456;FREQ_HOMALT=0.00019400523814142983;FREQ_HOMREF=0.9955378795227471;N_BI_GENOS=10309;South_Han_AN=1496;Sout
chr4	126553085	.	A	<INS:ME:LINE1>	.	PASS	SVTYPE=INS:ME:LINE1;END=126553095;SVLEN=1427;AN=20618;AC=1;AF=4.850130953535746e-05;N_HET=1;N_HOMALT=0;N_HOMREF=10308;FREQ_HET=9.700261907071492e-05;FREQ_HOMALT=0.0;FREQ_HOMREF=0.9999029973809292;N_BI_GENOS=10309;Southeast_Han_AN=1908;Southeast_Han_AC=1;S
chr4	126620159	.	T	<INS:ME:LINE1>	.	PASS	SVTYPE=INS:ME:LINE1;END=126620171;SVLEN=6000;AN=20618;AC=4;AF=0.00019400523814142983;N_HET=4;N_HOMALT=0;N_HOMREF=10305;FREQ_HET=0.0003880104762828 5966;FREQ_HOMALT=0.0;FREQ_HOMREF=0.9996119895237171;N_BI_GENOS=10309;South_Han_AN=1496;South_Han_AC=1;South_H
chr4	126623288	.	A	<INS:ME:LINE1>	.	PASS	SVTYPE=INS:ME:LINE1;END=126623288;SVLEN=55;AN=20618;AC=1;AF=4.850130953535746e-05;N_HET=1;N_HOMALT=0;N_HOMREF=10308;FREQ_HET=9.700261907071492e-05;FREQ_HOMALT=0.0;FREQ_HOMREF=0.9999029973809292;N_BI_GENOS=10309;East_Han_AN=6554;East_Han_AC=1;East_Han_AF=0
chr4	126675037	.	T	<INS:ME:LINE1>	.	PASS	SVTYPE=INS:ME:LINE1;END=126675054;SVLEN=452;AN=20618;AC=5519;AF=0.2676787273256378;N_HET=861;N_HOMALT=2329;N_HOMREF=7119;FREQ_HET=0.08351925501988554;FREQ_HOMALT=0.22591909981569502;FREQ_HOMREF=0.6905616451644194;N_BI_GENOS=10309;South_Han_AN=1496;South_H
chr4	127049755	.	C	<INS:ME:LINE1>	.	PASS	SVTYPE=INS:ME:LINE1;END=127049771;SVLEN=775;AN=20618;AC=1;AF=4.850130953535746e-05;N_HET=1;N_HOMALT=0;N_HOMREF=10308;FREQ_HET=9.700261907071492e-05;FREQ_HOMALT=0.0;FREQ_HOMREF=0.9999029973809292;N_BI_GENOS=10309;Southeast_Han_AN=1908;Southeast_Han_AC=1;So
chr4	127109237	.	C	<INS:ME:LINE1>	.	PASS	SVTYPE=INS:ME:LINE1;END=127109237;SVLEN=870;AN=20618;AC=1;AF=4.850130953535746e-05;N_HET=1;N_HOMALT=0;N_HOMREF=10308;FREQ_HET=9.700261907071492e-05;FREQ_HOMALT=0.0;FREQ_HOMREF=0.9999029973809292;N_BI_GENOS=10309;East_Han_AN=6554;East_Han_AC=1;East_Han_AF=
chr4	127109887	.	G	<INS:ME:LINE1>	.	PASS	SVTYPE=INS:ME:LINE1;END=127109929;SVLEN=2048;AN=20618;AC=1;AF=4.850130953535746e-05;N_HET=1;N_HOMALT=0;N_HOMREF=10308;FREQ_HET=9.700261907071492e-05;FREQ_HOMALT=0.0;FREQ_HOMREF=0.9999029973809292;N_BI_GENOS=10309;East_Han_AN=6554;East_Han_AC=1;East_Han_AF
chr4	127110746	.	G	<INS:ME:LINE1>	.	PASS	SVTYPE=INS:ME:LINE1;END=127110746;SVLEN=461;AN=20618;AC=1;AF=4.850130953535746e-05;N_HET=1;N_HOMALT=0;N_HOMREF=10308;FREQ_HET=9.700261907071492e-05;FREQ_HOMALT=0.0;FREQ_HOMREF=0.9999029973809292;N_BI_GENOS=10309;East_Han_AN=6554;East_Han_AC=1;East_Han_AF=
chr4	127159338	.	A	<INS:ME:LINE1>	.	PASS	SVTYPE=INS:ME:LINE1;END=127159353;SVLEN=578;AN=20618;AC=1;AF=4.850130953535746e-05;N_HET=1;N_HOMALT=0;N_HOMREF=10308;FREQ_HET=9.700261907071492e-05;FREQ_HOMALT=0.0;FREQ_HOMREF=0.9999029973809292;N_BI_GENOS=10309;Central_Han_AN=2128;Central_Han_AC=1;Centra
chr4	127220541	.	T	<INS:ME:LINE1>	.	PASS	SVTYPE=INS:ME:LINE1;END=127220541;SVLEN=0;AN=20618;AC=2;AF=9.700261907071492e-05;N_HET=0;N_HOMALT=1;N_HOMREF=10308;FREQ_HET=0.0;FREQ_HOMALT=9.700261907071492e-05;FREQ_HOMREF=0.9999029973809292;N_BI_GENOS=10309;East_Han_AN=6554;East_Han_AC=2;East_Han_AF=0.
chr4	127244346	.	G	<INS:ME:LINE1>	.	PASS	SVTYPE=INS:ME:LINE1;END=127244366;SVLEN=966;AN=20618;AC=4;AF=0.00019400523814142983;N_HET=4;N_HOMALT=0;N_HOMREF=10305;FREQ_HET=0.00038801047628285966;FREQ_HOMALT=0.0;FREQ_HOMREF=0.9996119895237171;N_BI_GENOS=10309;Northwest_Han_AN=1296;Northwest_Han_AC=1;
chr4	127282720	.	G	<INS:ME:LINE1>	.	PASS	SVTYPE=INS:ME:LINE1;END=127282725;SVLEN=1855;AN=20618;AC=1;AF=4.850130953535746e-05;N_HET=1;N_HOMALT=0;N_HOMREF=10308;FREQ_HET=9.700261907071492e-05;FREQ_HOMALT=0.0;FREQ_HOMREF=0.9999029973809292;N_BI_GENOS=10309;Tibetan_AN=414;Tibetan_AC=1;Tibetan_AF=0.0
chr4	127338029	.	A	<INS:ME:LINE1>	.	PASS	SVTYPE=INS:ME:LINE1;END=127338041;SVLEN=490;AN=20618;AC=10;AF=0.00048501309535357455;N_HET=10;N_HOMALT=0;N_HOMREF=10299;FREQ_HET=0.0009700261907071491;FREQ_HOMALT=0.0;FREQ_HOMREF=0.9990299738092928;N_BI_GENOS=10309;Lingnan_Han_AN=1888;Lingnan_Han_AC=1;Lin
chr4	127353329	.	T	<INS:ME:LINE1>	.	PASS	SVTYPE=INS:ME:LINE1;END=127353343;SVLEN=1131;AN=20618;AC=4;AF=0.00019400523814142983;N_HET=4;N_HOMALT=0;N_HOMREF=10305;FREQ_HET=0.00038801047628285966;FREQ_HOMALT=0.0;FREQ_HOMREF=0.9996119895237171;N_BI_GENOS=10309;South_Han_AN=1496;South_Han_AC=1;South_H
chr4	127398400	.	A	<INS:ME:LINE1>	.	PASS	SVTYPE=INS:ME:LINE1;END=127398414;SVLEN=6022;AN=20618;AC=1;AF=4.850130953535746e-05;N_HET=1;N_HOMALT=0;N_HOMREF=10308;FREQ_HET=9.700261907071492e-05;FREQ_HOMALT=0.0;FREQ_HOMREF=0.9999029973809292;N_BI_GENOS=10309;Lingnan_Han_AN=1888;Lingnan_Han_AC=1;Lingn
chr4	127918206	.	T	<INS:ME:LINE1>	.	PASS	SVTYPE=INS:ME:LINE1;END=127918217;SVLEN=369;AN=20618;AC=2;AF=9.700261907071492e-05;N_HET=2;N_HOMALT=0;N_HOMREF=10307;FREQ_HET=0.00019400523814142983;FREQ_HOMALT=0.0;FREQ_HOMREF=0.9998059947618586;N_BI_GENOS=10309;East_Han_AN=6554;East_Han_AC=1;East_Han_AF
chr4	128256453	.	T	<INS:ME:LINE1>	.	PASS	SVTYPE=INS:ME:LINE1;END=128256453;SVLEN=5998;AN=20618;AC=1;AF=4.850130953535746e-05;N_HET=1;N_HOMALT=0;N_HOMREF=10308;FREQ_HET=9.700261907071492e-05;FREQ_HOMALT=0.0;FREQ_HOMREF=0.9999029973809292;N_BI_GENOS=10309;Central_Han_AN=2128;Central_Han_AC=1;Centr
chr4	128913895	.	C	<INS:ME:LINE1>	.	PASS	SVTYPE=INS:ME:LINE1;END=128913910;SVLEN=516;AN=20618;AC=4;AF=0.00019400523814142983;N_HET=4;N_HOMALT=0;N_HOMREF=10305;FREQ_HET=0.00038801047628285966;FREQ_HOMALT=0.0;FREQ_HOMREF=0.9996119895237171;N_BI_GENOS=10309;East_Han_AN=6554;East_Han_AC=3;East_Han_A
chr4	129041228	.	A	<INS:ME:LINE1>	.	PASS	SVTYPE=INS:ME:LINE1;END=129041243;SVLEN=5963;AN=20618;AC=1;AF=4.850130953535746e-05;N_HET=1;N_HOMALT=0;N_HOMREF=10308;FREQ_HET=9.700261907071492e-05;FREQ_HOMALT=0.0;FREQ_HOMREF=0.9999029973809292;N_BI_GENOS=10309;North_Han_AN=2272;North_Han_AC=1;North_Han
chr4	129427787	.	T	<INS:ME:LINE1>	.	PASS	SVTYPE=INS:ME:LINE1;END=129427802;SVLEN=531;AN=20618;AC=3;AF=0.00014550392860607236;N_HET=3;N_HOMALT=0;N_HOMREF=10306;FREQ_HET=0.00029100785721214 47;FREQ_HOMALT=0.0;FREQ_HOMREF=0.9997089921427879;N_BI_GENOS=10309;East_Han_AN=6554;East_Han_AC=2;East_Han_AF
chr4	129440499	.	G	<INS:ME:LINE1>	.	PASS	SVTYPE=INS:ME:LINE1;END=129440515;SVLEN=701;AN=20618;AC=865;AF=0.04195363274808419 6;N_HET=735;N_HOMALT=65;N_HOMREF=9509;FREQ_HET=0.07129692501697546;FREQ_HOMALT=0.006305170239596469;FREQ_HOMREF=0.9223979047434281;N_BI_GENOS=10309;South_Han_AN=1496;South_H
chr4	129605922	.	A	<INS:ME:LINE1>	.	PASS	SVTYPE=INS:ME:LINE1;END=129605936;SVLEN=6003;AN=20618;AC=1;AF=4.850130953535746e-05;N_HET=1;N_HOMALT=0;N_HOMREF=10308;FREQ_HET=9.700261907071492e-05;FREQ_HOMALT=0.0;FREQ_HOMREF=0.9999029973809292;N_BI_GENOS=10309;Central_Han_AN=2128;Central_Han_AC=1;Centr
chr4	129680270	.	A	<INS:ME:LINE1>	.	PASS	SVTYPE=INS:ME:LINE1;END=129680280;SVLEN=765;AN=20618;AC=1445;AF=0.07008439227859152;N_HET=1321;N_HOMALT=62;N_HOMREF=8926;FREQ_HET=0.1281404597924144;FREQ_HOMALT=0.006014162382384325;FREQ_HOMREF=0.8658453778252013;N_BI_GENOS=10309;South_Han_AN=1496;South_H
chr4	129908957	.	A	<INS:ME:LINE1>	.	PASS	SVTYPE=INS:ME:LINE1;END=129908965;SVLEN=812;AN=20618;AC=1;AF=4.850130953535746e-05;N_HET=1;N_HOMALT=0;N_HOMREF=10308;FREQ_HET=9.700261907071492e-05;FREQ_HOMALT=0.0;FREQ_HOMREF=0.9999029973809292;N_BI_GENOS=10309;Central_Han_AN=2128;Central_Han_AC=1;Centra
chr4	130026615	.	G	<INS:ME:LINE1>	.	PASS	SVTYPE=INS:ME:LINE1;END=130026615;SVLEN=95;AN=20618;AC=1;AF=4.850130953535746e-05;N_HET=1;N_HOMALT=0;N_HOMREF=10308;FREQ_HET=9.700261907071492e-05;FREQ_HOMALT=0.0;FREQ_HOMREF=0.9999029973809292;N_BI_GENOS=10309;Tibetan_AN=414;Tibetan_AC=1;Tibetan_AF=0.002
chr4	130092723	.	A	<INS:ME:LINE1>	.	PASS	SVTYPE=INS:ME:LINE1;END=130092723;SVLEN=5965;AN=20618;AC=3;AF=0.00014550392860607236;N_HET=3;N_HOMALT=0;N_HOMREF=10306;FREQ_HET=0.00029100785721214 47;FREQ_HOMALT=0.0;FREQ_HOMREF=0.9997089921427879;N_BI_GENOS=10309;South_Han_AN=1496;South_Han_AC=1;South_Ha
chr4	130227158	.	T	<INS:ME:LINE1>	.	PASS	SVTYPE=INS:ME:LINE1;END=130227159;SVLEN=433;AN=20618;AC=1;AF=4.850130953535746e-05;N_HET=1;N_HOMALT=0;N_HOMREF=10308;FREQ_HET=9.700261907071492e-05;FREQ_HOMALT=0.0;FREQ_HOMREF=0.9999029973809292;N_BI_GENOS=10309;Central_Han_AN=2128;Central_Han_AC=1;Centra
chr4	130326351	.	A	<INS:ME:LINE1>	.	PASS	SVTYPE=INS:ME:LINE1;END=130326355;SVLEN=917;AN=20618;AC=1;AF=4.850130953535746e-05;N_HET=1;N_HOMALT=0;N_HOMREF=10308;FREQ_HET=9.700261907071492e-05;FREQ_HOMALT=0.0;FREQ_HOMREF=0.9999029973809292;N_BI_GENOS=10309;South_Han_AN=1496;South_Han_AC=1;South_Han_
chr4	130428503	.	G	<INS:ME:LINE1>	.	PASS	SVTYPE=INS:ME:LINE1;END=130428517;SVLEN=5997;AN=20618;AC=8;AF=0.00038801047628285966;N_HET=8;N_HOMALT=0;N_HOMREF=10301;FREQ_HET=0.0007760209525657193;FREQ_HOMALT=0.0;FREQ_HOMREF=0.9992239790474343;N_BI_GENOS=10309;Northwest_Han_AN=1296;Northwest_Han_AC=1;
chr4	130453965	.	A	<INS:ME:LINE1>	.	PASS	SVTYPE=INS:ME:LINE1;END=130453967;SVLEN=69;AN=20618;AC=1;AF=4.850130953535746e-05;N_HET=1;N_HOMALT=0;N_HOMREF=10308;FREQ_HET=9.700261907071492e-05;FREQ_HOMALT=0.0;FREQ_HOMREF=0.9999029973809292;N_BI_GENOS=10309;Central_Han_AN=2128;Central_Han_AC=1;Central
chr4	130758249	.	A	<INS:ME:LINE1>	.	PASS	SVTYPE=INS:ME:LINE1;END=130758258;SVLEN=1290;AN=20618;AC=1;AF=4.850130953535746e-05;N_HET=1;N_HOMALT=0;N_HOMREF=10308;FREQ_HET=9.700261907071492e-05;FREQ_HOMALT=0.0;FREQ_HOMREF=0.9999029973809292;N_BI_GENOS=10309;East_Han_AN=6554;East_Han_AC=1;East_Han_AF
chr4	130824727	.	T	<INS:ME:LINE1>	.	PASS	SVTYPE=INS:ME:LINE1;END=130824750;SVLEN=287;AN=20618;AC=1;AF=4.850130953535746e-05;N_HET=1;N_HOMALT=0;N_HOMREF=10308;FREQ_HET=9.700261907071492e-05;FREQ_HOMALT=0.0;FREQ_HOMREF=0.9999029973809292;N_BI_GENOS=10309;South_Han_AN=1496;South_Han_AC=1;South_Han_
chr4	131197578	.	T	<INS:ME:LINE1>	.	PASS	SVTYPE=INS:ME:LINE1;END=131197591;SVLEN=135;AN=20618;AC=2;AF=9.700261907071492e-05;N_HET=0;N_HOMALT=1;N_HOMREF=10308;FREQ_HET=0.0;FREQ_HOMALT=9.700261907071492e-05;FREQ_HOMREF=0.9999029973809292;N_BI_GENOS=10309;South_Han_AN=1496;South_Han_AC=2;South_Han_
chr4	131211608	.	T	<INS:ME:LINE1>	.	PASS	SVTYPE=INS:ME:LINE1;END=131211615;SVLEN=1407;AN=20618;AC=1;AF=4.850130953535746e-05;N_HET=1;N_HOMALT=0;N_HOMREF=10308;FREQ_HET=9.700261907071492e-05;FREQ_HOMALT=0.0;FREQ_HOMREF=0.9999029973809292;N_BI_GENOS=10309;Central_Han_AN=2128;Central_Han_AC=1;Centr
chr4	131260492	.	G	<INS:ME:LINE1>	.	PASS	SVTYPE=INS:ME:LINE1;END=131260507;SVLEN=6000;AN=20618;AC=1833;AF=0.08890290037831021;N_HET=1611;N_HOMALT=111;N_HOMREF=8587;FREQ_HET=0.1562712193229217;FREQ_HOMALT=0.010767290716849355;FREQ_HOMREF=0.8329614899602289;N_BI_GENOS=10309;South_Han_AN=1496;South
chr4	131316960	.	G	<INS:ME:LINE1>	.	PASS	SVTYPE=INS:ME:LINE1;END=131316960;SVLEN=181;AN=20618;AC=1;AF=4.850130953535746e-05;N_HET=1;N_HOMALT=0;N_HOMREF=10308;FREQ_HET=9.700261907071492e-05;FREQ_HOMALT=0.0;FREQ_HOMREF=0.9999029973809292;N_BI_GENOS=10309;East_Han_AN=6554;East_Han_AC=1;East_Han_AF=
chr4	131331738	.	G	<INS:ME:LINE1>	.	PASS	SVTYPE=INS:ME:LINE1;END=131331746;SVLEN=208;AN=20618;AC=201;AF=0.009748763216606848;N_HET=201;N_HOMALT=0;N_HOMREF=10108;FREQ_HET=0.019497526433213695;FREQ_HOMALT=0.0;FREQ_HOMREF=0.9805024735667863;N_BI_GENOS=10309;South_Han_AN=1496;South_Han_AC=10;South_H
chr4	131344732	.	T	<INS:ME:LINE1>	.	PASS	SVTYPE=INS:ME:LINE1;END=131344748;SVLEN=519;AN=20618;AC=5;AF=0.00024250654767678728;N_HET=5;N_HOMALT=0;N_HOMREF=10304;FREQ_HET=0.00048501309535357455;FREQ_HOMALT=0.0;FREQ_HOMREF=0.9995149869046465;N_BI_GENOS=10309;Mongolian_AN=454;Mongolian_AC=1;Mongolian
chr4	131437504	.	C	<INS:ME:LINE1>	.	PASS	SVTYPE=INS:ME:LINE1;END=131437532;SVLEN=2625;AN=20618;AC=2;AF=9.700261907071492e-05;N_HET=2;N_HOMALT=0;N_HOMREF=10307;FREQ_HET=0.00019400523814142983;FREQ_HOMALT=0.0;FREQ_HOMREF=0.9998059947618586;N_BI_GENOS=10309;East_Han_AN=6554;East_Han_AC=2;East_Han_A
chr4	131516954	.	A	<INS:ME:LINE1>	.	PASS	SVTYPE=INS:ME:LINE1;END=131516966;SVLEN=2447;AN=20618;AC=2;AF=9.700261907071492e-05;N_HET=2;N_HOMALT=0;N_HOMREF=10307;FREQ_HET=0.00019400523814142983;FREQ_HOMALT=0.0;FREQ_HOMREF=0.9998059947618586;N_BI_GENOS=10309;South_Han_AN=1496;South_Han_AC=1;South_Ha
chr4	131537619	.	A	<INS:ME:LINE1>	.	PASS	SVTYPE=INS:ME:LINE1;END=131537633;SVLEN=817;AN=20618;AC=2;AF=9.700261907071492e-05;N_HET=2;N_HOMALT=0;N_HOMREF=10307;FREQ_HET=0.00019400523814142983;FREQ_HOMALT=0.0;FREQ_HOMREF=0.9998059947618586;N_BI_GENOS=10309;East_Han_AN=6554;East_Han_AC=2;East_Han_AF
chr4	131616612	.	C	<INS:ME:LINE1>	.	PASS	SVTYPE=INS:ME:LINE1;END=131616612;SVLEN=2576;AN=20618;AC=1;AF=4.850130953535746e-05;N_HET=1;N_HOMALT=0;N_HOMREF=10308;FREQ_HET=9.700261907071492e-05;FREQ_HOMALT=0.0;FREQ_HOMREF=0.9999029973809292;N_BI_GENOS=10309;East_Han_AN=6554;East_Han_AC=1;East_Han_AF
chr4	131623517	.	C	<INS:ME:LINE1>	.	PASS	SVTYPE=INS:ME:LINE1;END=131623533;SVLEN=998;AN=20618;AC=1;AF=4.850130953535746e-05;N_HET=1;N_HOMALT=0;N_HOMREF=10308;FREQ_HET=9.700261907071492e-05;FREQ_HOMALT=0.0;FREQ_HOMREF=0.9999029973809292;N_BI_GENOS=10309;East_Han_AN=6554;East_Han_AC=1;East_Han_AF=
chr4	131667938	.	G	<INS:ME:LINE1>	.	PASS	SVTYPE=INS:ME:LINE1;END=131667938;SVLEN=0;AN=20618;AC=1;AF=4.850130953535746e-05;N_HET=1;N_HOMALT=0;N_HOMREF=10308;FREQ_HET=9.700261907071492e-05;FREQ_HOMALT=0.0;FREQ_HOMREF=0.9999029973809292;N_BI_GENOS=10309;South_Han_AN=1496;South_Han_AC=1;South_Han_AF
chr4	131814536	.	G	<INS:ME:LINE1>	.	PASS	SVTYPE=INS:ME:LINE1;END=131814536;SVLEN=2055;AN=20618;AC=1;AF=4.850130953535746e-05;N_HET=1;N_HOMALT=0;N_HOMREF=10308;FREQ_HET=9.700261907071492e-05;FREQ_HOMALT=0.0;FREQ_HOMREF=0.9999029973809292;N_BI_GENOS=10309;East_Han_AN=6554;East_Han_AC=1;East_Han_AF
chr4	131986843	.	T	<INS:ME:LINE1>	.	PASS	SVTYPE=INS:ME:LINE1;END=131986862;SVLEN=736;AN=20618;AC=2;AF=9.700261907071492e-05;N_HET=2;N_HOMALT=0;N_HOMREF=10307;FREQ_HET=0.00019400523814142983;FREQ_HOMALT=0.0;FREQ_HOMREF=0.9998059947618586;N_BI_GENOS=10309;Lingnan_Han_AN=1888;Lingnan_Han_AC=1;Lingn
chr4	132120511	.	C	<INS:ME:LINE1>	.	PASS	SVTYPE=INS:ME:LINE1;END=132120511;SVLEN=0;AN=20618;AC=2;AF=9.700261907071492e-05;N_HET=0;N_HOMALT=1;N_HOMREF=10308;FREQ_HET=0.0;FREQ_HOMALT=9.700261907071492e-05;FREQ_HOMREF=0.9999029973809292;N_BI_GENOS=10309;East_Han_AN=6554;East_Han_AC=2;East_Han_AF=0.
chr4	132203216	.	A	<INS:ME:LINE1>	.	PASS	SVTYPE=INS:ME:LINE1;END=132203235;SVLEN=1632;AN=20618;AC=2;AF=9.700261907071492e-05;N_HET=2;N_HOMALT=0;N_HOMREF=10307;FREQ_HET=0.00019400523814142983;FREQ_HOMALT=0.0;FREQ_HOMREF=0.9998059947618586;N_BI_GENOS=10309;Northwest_Han_AN=1296;Northwest_Han_AC=1;
chr4	132910196	.	T	<INS:ME:LINE1>	.	PASS	SVTYPE=INS:ME:LINE1;END=132910196;SVLEN=1655;AN=20618;AC=1;AF=4.850130953535746e-05;N_HET=1;N_HOMALT=0;N_HOMREF=10308;FREQ_HET=9.700261907071492e-05;FREQ_HOMALT=0.0;FREQ_HOMREF=0.9999029973809292;N_BI_GENOS=10309;North_Han_AN=2272;North_Han_AC=1;North_Han
chr4	133000000	.	T	<INS:ME:LINE1>	.	PASS	SVTYPE=INS:ME:LINE1;END=133000005;SVLEN=943;AN=20618;AC=4;AF=0.00019400523814142983;N_HET=4;N_HOMALT=0;N_HOMREF=10305;FREQ_HET=0.00038801047628285966;FREQ_HOMALT=0.0;FREQ_HOMREF=0.9996119895237171;N_BI_GENOS=10309;South_Han_AN=1496;South_Han_AC=2;South_Ha
chr4	133036065	.	A	<INS:ME:LINE1>	.	PASS	SVTYPE=INS:ME:LINE1;END=133036082;SVLEN=2938;AN=20618;AC=1;AF=4.850130953535746e-05;N_HET=1;N_HOMALT=0;N_HOMREF=10308;FREQ_HET=9.700261907071492e-05;FREQ_HOMALT=0.0;FREQ_HOMREF=0.9999029973809292;N_BI_GENOS=10309;Miao_AN=482;Miao_AC=1;Miao_AF=0.0020746887
chr4	133049967	.	C	<INS:ME:LINE1>	.	PASS	SVTYPE=INS:ME:LINE1;END=133049974;SVLEN=2741;AN=20618;AC=1;AF=4.850130953535746e-05;N_HET=1;N_HOMALT=0;N_HOMREF=10308;FREQ_HET=9.700261907071492e-05;FREQ_HOMALT=0.0;FREQ_HOMREF=0.9999029973809292;N_BI_GENOS=10309;East_Han_AN=6554;East_Han_AC=1;East_Han_AF
chr4	133050326	.	A	<INS:ME:LINE1>	.	PASS	SVTYPE=INS:ME:LINE1;END=133050326;SVLEN=2349;AN=20618;AC=1;AF=4.850130953535746e-05;N_HET=1;N_HOMALT=0;N_HOMREF=10308;FREQ_HET=9.700261907071492e-05;FREQ_HOMALT=0.0;FREQ_HOMREF=0.9999029973809292;N_BI_GENOS=10309;East_Han_AN=6554;East_Han_AC=1;East_Han_AF
chr4	133598577	.	T	<INS:ME:LINE1>	.	PASS	SVTYPE=INS:ME:LINE1;END=133598577;SVLEN=1305;AN=20618;AC=4;AF=0.00019400523814142983;N_HET=2;N_HOMALT=1;N_HOMREF=10306;FREQ_HET=0.00019400523814142983;FREQ_HOMALT=9.700261907071492e-05;FREQ_HOMREF=0.9997089921427879;N_BI_GENOS=10309;Lingnan_Han_AN=1888;Li
chr4	133675267	.	A	<INS:ME:LINE1>	.	PASS	SVTYPE=INS:ME:LINE1;END=133675268;SVLEN=382;AN=20618;AC=10222;AF=0.4957803860704239;N_HET=4828;N_HOMALT=2697;N_HOMREF=2784;FREQ_HET=0.4683286448734116;FREQ_HOMALT=0.2616160636337181;FREQ_HOMREF=0.27005529149287033;N_BI_GENOS=10309;South_Han_AN=1496;South_
chr4	133972904	.	T	<INS:ME:LINE1>	.	PASS	SVTYPE=INS:ME:LINE1;END=133972917;SVLEN=1514;AN=20618;AC=1;AF=4.850130953535746e-05;N_HET=1;N_HOMALT=0;N_HOMREF=10308;FREQ_HET=9.700261907071492e-05;FREQ_HOMALT=0.0;FREQ_HOMREF=0.9999029973809292;N_BI_GENOS=10309;Lingnan_Han_AN=1888;Lingnan_Han_AC=1;Lingn
chr4	134065859	.	A	<INS:ME:LINE1>	.	PASS	SVTYPE=INS:ME:LINE1;END=134065859;SVLEN=729;AN=20618;AC=1;AF=4.850130953535746e-05;N_HET=1;N_HOMALT=0;N_HOMREF=10308;FREQ_HET=9.700261907071492e-05;FREQ_HOMALT=0.0;FREQ_HOMREF=0.9999029973809292;N_BI_GENOS=10309;East_Han_AN=6554;East_Han_AC=1;East_Han_AF=
chr4	134182200	.	A	<INS:ME:LINE1>	.	PASS	SVTYPE=INS:ME:LINE1;END=134182200;SVLEN=823;AN=20618;AC=1;AF=4.850130953535746e-05;N_HET=1;N_HOMALT=0;N_HOMREF=10308;FREQ_HET=9.700261907071492e-05;FREQ_HOMALT=0.0;FREQ_HOMREF=0.9999029973809292;N_BI_GENOS=10309;East_Han_AN=6554;East_Han_AC=1;East_Han_AF=
chr4	134182606	.	T	<INS:ME:LINE1>	.	PASS	SVTYPE=INS:ME:LINE1;END=134182643;SVLEN=971;AN=20618;AC=1;AF=4.850130953535746e-05;N_HET=1;N_HOMALT=0;N_HOMREF=10308;FREQ_HET=9.700261907071492e-05;FREQ_HOMALT=0.0;FREQ_HOMREF=0.9999029973809292;N_BI_GENOS=10309;East_Han_AN=6554;East_Han_AC=1;East_Han_AF=
chr4	134284643	.	T	<INS:ME:LINE1>	.	PASS	SVTYPE=INS:ME:LINE1;END=134284643;SVLEN=5981;AN=20618;AC=1;AF=4.850130953535746e-05;N_HET=1;N_HOMALT=0;N_HOMREF=10308;FREQ_HET=9.700261907071492e-05;FREQ_HOMALT=0.0;FREQ_HOMREF=0.9999029973809292;N_BI_GENOS=10309;Tibetan_AN=414;Tibetan_AC=1;Tibetan_AF=0.0
chr4	134341738	.	T	<INS:ME:LINE1>	.	PASS	SVTYPE=INS:ME:LINE1;END=134341758;SVLEN=927;AN=20618;AC=1;AF=4.850130953535746e-05;N_HET=1;N_HOMALT=0;N_HOMREF=10308;FREQ_HET=9.700261907071492e-05;FREQ_HOMALT=0.0;FREQ_HOMREF=0.9999029973809292;N_BI_GENOS=10309;Northwest_Han_AN=1296;Northwest_Han_AC=1;No
chr4	134499544	.	A	<INS:ME:LINE1>	.	PASS	SVTYPE=INS:ME:LINE1;END=134499558;SVLEN=4551;AN=20618;AC=20;AF=0.0009700261907071491;N_HET=18;N_HOMALT=1;N_HOMREF=10290;FREQ_HET=0.0017460471432728683;FREQ_HOMALT=9.700261907071492e-05;FREQ_HOMREF=0.9981569502376564;N_BI_GENOS=10309;South_Han_AN=1496;Sout
chr4	135121936	.	T	<INS:ME:LINE1>	.	PASS	SVTYPE=INS:ME:LINE1;END=135121946;SVLEN=1240;AN=20618;AC=3;AF=0.00014550392860607236;N_HET=3;N_HOMALT=0;N_HOMREF=10306;FREQ_HET=0.00029100785721214 47;FREQ_HOMALT=0.0;FREQ_HOMREF=0.9997089921427879;N_BI_GENOS=10309;North_Han_AN=2272;North_Han_AC=1;North_Ha
chr4	135498480	.	T	<INS:ME:LINE1>	.	PASS	SVTYPE=INS:ME:LINE1;END=135498480;SVLEN=141;AN=20618;AC=1;AF=4.850130953535746e-05;N_HET=1;N_HOMALT=0;N_HOMREF=10308;FREQ_HET=9.700261907071492e-05;FREQ_HOMALT=0.0;FREQ_HOMREF=0.9999029973809292;N_BI_GENOS=10309;East_Han_AN=6554;East_Han_AC=1;East_Han_AF=
chr4	135684717	.	G	<INS:ME:LINE1>	.	PASS	SVTYPE=INS:ME:LINE1;END=135684717;SVLEN=50;AN=20618;AC=1;AF=4.850130953535746e-05;N_HET=1;N_HOMALT=0;N_HOMREF=10308;FREQ_HET=9.700261907071492e-05;FREQ_HOMALT=0.0;FREQ_HOMREF=0.9999029973809292;N_BI_GENOS=10309;Central_Han_AN=2128;Central_Han_AC=1;Central
chr4	135735476	.	A	<INS:ME:LINE1>	.	PASS	SVTYPE=INS:ME:LINE1;END=135735485;SVLEN=824;AN=20618;AC=2;AF=9.700261907071492e-05;N_HET=2;N_HOMALT=0;N_HOMREF=10307;FREQ_HET=0.00019400523814142983;FREQ_HOMALT=0.0;FREQ_HOMREF=0.9998059947618586;N_BI_GENOS=10309;East_Han_AN=6554;East_Han_AC=1;East_Han_AF
chr4	136017445	.	A	<INS:ME:LINE1>	.	PASS	SVTYPE=INS:ME:LINE1;END=136017445;SVLEN=5998;AN=20618;AC=1;AF=4.850130953535746e-05;N_HET=1;N_HOMALT=0;N_HOMREF=10308;FREQ_HET=9.700261907071492e-05;FREQ_HOMALT=0.0;FREQ_HOMREF=0.9999029973809292;N_BI_GENOS=10309;Lingnan_Han_AN=1888;Lingnan_Han_AC=1;Lingn
chr4	136066304	.	T	<INS:ME:LINE1>	.	PASS	SVTYPE=INS:ME:LINE1;END=136066304;SVLEN=139;AN=20618;AC=1;AF=4.850130953535746e-05;N_HET=1;N_HOMALT=0;N_HOMREF=10308;FREQ_HET=9.700261907071492e-05;FREQ_HOMALT=0.0;FREQ_HOMREF=0.9999029973809292;N_BI_GENOS=10309;East_Han_AN=6554;East_Han_AC=1;East_Han_AF=
chr4	136854893	.	A	<INS:ME:LINE1>	.	PASS	SVTYPE=INS:ME:LINE1;END=136854906;SVLEN=486;AN=20618;AC=8;AF=0.00038801047628285966;N_HET=6;N_HOMALT=1;N_HOMREF=10302;FREQ_HET=0.0005820157144242894;FREQ_HOMALT=9.700261907071492e-05;FREQ_HOMREF=0.999320981666505;N_BI_GENOS=10309;Northwest_Han_AN=1296;Nor
chr4	137051854	.	G	<INS:ME:LINE1>	.	PASS	SVTYPE=INS:ME:LINE1;END=137051869;SVLEN=1029;AN=20618;AC=1;AF=4.850130953535746e-05;N_HET=1;N_HOMALT=0;N_HOMREF=10308;FREQ_HET=9.700261907071492e-05;FREQ_HOMALT=0.0;FREQ_HOMREF=0.9999029973809292;N_BI_GENOS=10309;Yi_AN=422;Yi_AC=1;Yi_AF=0.0023696682464454

chr4	137256170	.	A	<INS:ME:LINE1>	.	PASS	SVTYPE=INS:ME:LINE1;END=137256180;SVLEN=1484;AN=20618;AC=8133;AF=0.39446115045106217;N_HET=4619;N_HOMALT=1757;N_HOMREF=3933;FREQ_HET=0.44805509748763217;FREQ_HOMALT=0.17043360170724609;FREQ_HOMREF=0.38151130080512174;N_BI_GENOS=10309;South_Han_AN=1496;Sou
chr4	137326771	.	G	<INS:ME:LINE1>	.	PASS	SVTYPE=INS:ME:LINE1;END=137326771;SVLEN=190;AN=20618;AC=1;AF=4.850130953535746e-05;N_HET=1;N_HOMALT=0;N_HOMREF=10308;FREQ_HET=9.700261907071492e-05;FREQ_HOMALT=0.0;FREQ_HOMREF=0.9999029973809292;N_BI_GENOS=10309;East_Han_AN=6554;East_Han_AC=1;East_Han_AF=
chr4	137492406	.	T	<INS:ME:LINE1>	.	PASS	SVTYPE=INS:ME:LINE1;END=137492407;SVLEN=633;AN=20618;AC=19;AF=0.0009215248811717916;N_HET=19;N_HOMALT=0;N_HOMREF=10290;FREQ_HET=0.0018430497623435833;FREQ_HOMALT=0.0;FREQ_HOMREF=0.9981569502376564;N_BI_GENOS=10309;South_Han_AN=1496;South_Han_AC=2;South_Ha
chr4	137748954	.	A	<INS:ME:LINE1>	.	PASS	SVTYPE=INS:ME:LINE1;END=137748968;SVLEN=1049;AN=20618;AC=3;AF=0.00014550392860607236;N_HET=3;N_HOMALT=0;N_HOMREF=10306;FREQ_HET=0.00029100785721214447;FREQ_HOMALT=0.0;FREQ_HOMREF=0.9997089921427879;N_BI_GENOS=10309;North_Han_AN=2272;North_Han_AC=1;North_Ha
chr4	137811548	.	A	<INS:ME:LINE1>	.	PASS	SVTYPE=INS:ME:LINE1;END=137811561;SVLEN=642;AN=20618;AC=1;AF=4.850130953535746e-05;N_HET=1;N_HOMALT=0;N_HOMREF=10308;FREQ_HET=9.700261907071492e-05;FREQ_HOMALT=0.0;FREQ_HOMREF=0.9999029973809292;N_BI_GENOS=10309;East_Han_AN=6554;East_Han_AC=1;East_Han_AF=
chr4	138014520	.	A	<INS:ME:LINE1>	.	PASS	SVTYPE=INS:ME:LINE1;END=138014535;SVLEN=1323;AN=20618;AC=3;AF=0.00014550392860607236;N_HET=3;N_HOMALT=0;N_HOMREF=10306;FREQ_HET=0.00029100785721214447;FREQ_HOMALT=0.0;FREQ_HOMREF=0.9997089921427879;N_BI_GENOS=10309;East_Han_AN=6554;East_Han_AC=3;East_Han_A
chr4	138458064	.	T	<INS:ME:LINE1>	.	PASS	SVTYPE=INS:ME:LINE1;END=138458084;SVLEN=5981;AN=20618;AC=1;AF=4.850130953535746e-05;N_HET=1;N_HOMALT=0;N_HOMREF=10308;FREQ_HET=9.700261907071492e-05;FREQ_HOMALT=0.0;FREQ_HOMREF=0.9999029973809292;N_BI_GENOS=10309;Northwest_Han_AN=1296;Northwest_Han_AC=1;N
chr4	138518377	.	G	<INS:ME:LINE1>	.	PASS	SVTYPE=INS:ME:LINE1;END=138518391;SVLEN=5998;AN=20618;AC=1;AF=4.850130953535746e-05;N_HET=1;N_HOMALT=0;N_HOMREF=10308;FREQ_HET=9.700261907071492e-05;FREQ_HOMALT=0.0;FREQ_HOMREF=0.9999029973809292;N_BI_GENOS=10309;North_Han_AN=2272;North_Han_AC=1;North_Han
chr4	138810949	.	G	<INS:ME:LINE1>	.	PASS	SVTYPE=INS:ME:LINE1;END=138810965;SVLEN=5999;AN=20618;AC=1;AF=4.850130953535746e-05;N_HET=1;N_HOMALT=0;N_HOMREF=10308;FREQ_HET=9.700261907071492e-05;FREQ_HOMALT=0.0;FREQ_HOMREF=0.9999029973809292;N_BI_GENOS=10309;Northwest_Han_AN=1296;Northwest_Han_AC=1;N
chr4	138811550	.	G	<INS:ME:LINE1>	.	PASS	SVTYPE=INS:ME:LINE1;END=138811564;SVLEN=145;AN=20618;AC=1;AF=4.850130953535746e-05;N_HET=1;N_HOMALT=0;N_HOMREF=10308;FREQ_HET=9.700261907071492e-05;FREQ_HOMALT=0.0;FREQ_HOMREF=0.9999029973809292;N_BI_GENOS=10309;Northwest_Han_AN=1296;Northwest_Han_AC=1;No
chr4	138839235	.	A	<INS:ME:LINE1>	.	PASS	SVTYPE=INS:ME:LINE1;END=138839250;SVLEN=89;AN=20618;AC=1;AF=4.850130953535746e-05;N_HET=1;N_HOMALT=0;N_HOMREF=10308;FREQ_HET=9.700261907071492e-05;FREQ_HOMALT=0.0;FREQ_HOMREF=0.9999029973809292;N_BI_GENOS=10309;South_Han_AN=1496;South_Han_AC=1;South_Han_A
chr4	138928346	.	A	<INS:ME:LINE1>	.	PASS	SVTYPE=INS:ME:LINE1;END=138928346;SVLEN=0;AN=20618;AC=1;AF=4.850130953535746e-05;N_HET=1;N_HOMALT=0;N_HOMREF=10308;FREQ_HET=9.700261907071492e-05;FREQ_HOMALT=0.0;FREQ_HOMREF=0.9999029973809292;N_BI_GENOS=10309;South_Han_AN=1496;South_Han_AC=1;South_Han_AF
chr4	138941363	.	A	<INS:ME:LINE1>	.	PASS	SVTYPE=INS:ME:LINE1;END=138941381;SVLEN=879;AN=20618;AC=1;AF=4.850130953535746e-05;N_HET=1;N_HOMALT=0;N_HOMREF=10308;FREQ_HET=9.700261907071492e-05;FREQ_HOMALT=0.0;FREQ_HOMREF=0.9999029973809292;N_BI_GENOS=10309;East_Han_AN=6554;East_Han_AC=1;East_Han_AF=
chr4	139726624	.	A	<INS:ME:LINE1>	.	PASS	SVTYPE=INS:ME:LINE1;END=139726640;SVLEN=800;AN=20618;AC=2717;AF=0.1317780580075662;N_HET=2175;N_HOMALT=271;N_HOMREF=7863;FREQ_HET=0.21098069647880494;FREQ_HOMALT=0.026287770976816374;FREQ_HOMREF=0.7627315937530313;N_BI_GENOS=10309;South_Han_AN=1496;South_H
chr4	140612319	.	A	<INS:ME:LINE1>	.	PASS	SVTYPE=INS:ME:LINE1;END=140612335;SVLEN=6011;AN=20618;AC=1;AF=4.850130953535746e-05;N_HET=1;N_HOMALT=0;N_HOMREF=10308;FREQ_HET=9.700261907071492e-05;FREQ_HOMALT=0.0;FREQ_HOMREF=0.9999029973809292;N_BI_GENOS=10309;South_Han_AN=1496;South_Han_AC=1;South_Han
chr4	140682075	.	T	<INS:ME:LINE1>	.	PASS	SVTYPE=INS:ME:LINE1;END=140682079;SVLEN=456;AN=20618;AC=2;AF=9.700261907071492e-05;N_HET=2;N_HOMALT=0;N_HOMREF=10307;FREQ_HET=0.00019400523814142983;FREQ_HOMALT=0.0;FREQ_HOMREF=0.9998059947618586;N_BI_GENOS=10309;Northwest_Han_AN=1296;Northwest_Han_AC=1;N
chr4	140877383	.	G	<INS:ME:LINE1>	.	PASS	SVTYPE=INS:ME:LINE1;END=140877397;SVLEN=5997;AN=20618;AC=1;AF=4.850130953535746e-05;N_HET=1;N_HOMALT=0;N_HOMREF=10308;FREQ_HET=9.700261907071492e-05;FREQ_HOMALT=0.0;FREQ_HOMREF=0.9999029973809292;N_BI_GENOS=10309;Manchu_AN=416;Manchu_AC=1;Manchu_AF=0.0024
chr4	140950509	.	G	<INS:ME:LINE1>	.	PASS	SVTYPE=INS:ME:LINE1;END=140950524;SVLEN=4291;AN=20618;AC=1;AF=4.850130953535746e-05;N_HET=1;N_HOMALT=0;N_HOMREF=10308;FREQ_HET=9.700261907071492e-05;FREQ_HOMALT=0.0;FREQ_HOMREF=0.9999029973809292;N_BI_GENOS=10309;Central_Han_AN=2128;Central_Han_AC=1;Centr
chr4	141020348	.	G	<INS:ME:LINE1>	.	PASS	SVTYPE=INS:ME:LINE1;END=141020364;SVLEN=6004;AN=20618;AC=1;AF=4.850130953535746e-05;N_HET=1;N_HOMALT=0;N_HOMREF=10308;FREQ_HET=9.700261907071492e-05;FREQ_HOMALT=0.0;FREQ_HOMREF=0.9999029973809292;N_BI_GENOS=10309;East_Han_AN=6554;East_Han_AC=1;East_Han_AF
chr4	141024321	.	T	<INS:ME:LINE1>	.	PASS	SVTYPE=INS:ME:LINE1;END=141024336;SVLEN=776;AN=20618;AC=1;AF=4.850130953535746e-05;N_HET=1;N_HOMALT=0;N_HOMREF=10308;FREQ_HET=9.700261907071492e-05;FREQ_HOMALT=0.0;FREQ_HOMREF=0.9999029973809292;N_BI_GENOS=10309;Northwest_Han_AN=1296;Northwest_Han_AC=1;No
chr4	141210130	.	T	<INS:ME:LINE1>	.	PASS	SVTYPE=INS:ME:LINE1;END=141210145;SVLEN=6012;AN=20618;AC=1;AF=4.850130953535746e-05;N_HET=1;N_HOMALT=0;N_HOMREF=10308;FREQ_HET=9.700261907071492e-05;FREQ_HOMALT=0.0;FREQ_HOMREF=0.9999029973809292;N_BI_GENOS=10309;South_Han_AN=1496;South_Han_AC=1;South_Han
chr4	141210601	.	G	<INS:ME:LINE1>	.	PASS	SVTYPE=INS:ME:LINE1;END=141210615;SVLEN=6013;AN=20618;AC=1;AF=4.850130953535746e-05;N_HET=1;N_HOMALT=0;N_HOMREF=10308;FREQ_HET=9.700261907071492e-05;FREQ_HOMALT=0.0;FREQ_HOMREF=0.9999029973809292;N_BI_GENOS=10309;Southeast_Han_AN=1908;Southeast_Han_AC=1;S
chr4	141301307	.	A	<INS:ME:LINE1>	.	PASS	SVTYPE=INS:ME:LINE1;END=141301324;SVLEN=2405;AN=20618;AC=2;AF=9.700261907071492e-05;N_HET=2;N_HOMALT=0;N_HOMREF=10307;FREQ_HET=0.00019400523814142983;FREQ_HOMALT=0.0;FREQ_HOMREF=0.9998059947618586;N_BI_GENOS=10309;East_Han_AN=6554;East_Han_AC=1;East_Han_A
chr4	141558147	.	C	<INS:ME:LINE1>	.	PASS	SVTYPE=INS:ME:LINE1;END=141558188;SVLEN=1733;AN=20618;AC=1;AF=4.850130953535746e-05;N_HET=1;N_HOMALT=0;N_HOMREF=10308;FREQ_HET=9.700261907071492e-05;FREQ_HOMALT=0.0;FREQ_HOMREF=0.9999029973809292;N_BI_GENOS=10309;South_Han_AN=1496;South_Han_AC=1;South_Han
chr4	141825299	.	A	<INS:ME:LINE1>	.	PASS	SVTYPE=INS:ME:LINE1;END=141825316;SVLEN=1658;AN=20618;AC=2;AF=9.700261907071492e-05;N_HET=2;N_HOMALT=0;N_HOMREF=10307;FREQ_HET=0.00019400523814142983;FREQ_HOMALT=0.0;FREQ_HOMREF=0.9998059947618586;N_BI_GENOS=10309;Lingnan_Han_AN=1888;Lingnan_Han_AC=1;Ling
chr4	142142726	.	T	<INS:ME:LINE1>	.	PASS	SVTYPE=INS:ME:LINE1;END=142142728;SVLEN=1100;AN=20618;AC=18;AF=0.0008730235716364342;N_HET=18;N_HOMALT=0;N_HOMREF=10291;FREQ_HET=0.0017460471432728683;FREQ_HOMALT=0.0;FREQ_HOMREF=0.9982539528567271;N_BI_GENOS=10309;South_Han_AN=1496;South_Han_AC=3;South_H
chr4	142156020	.	A	<INS:ME:LINE1>	.	PASS	SVTYPE=INS:ME:LINE1;END=142156036;SVLEN=1097;AN=20618;AC=1;AF=4.850130953535746e-05;N_HET=1;N_HOMALT=0;N_HOMREF=10308;FREQ_HET=9.700261907071492e-05;FREQ_HOMALT=0.0;FREQ_HOMREF=0.9999029973809292;N_BI_GENOS=10309;East_Han_AN=6554;East_Han_AC=1;East_Han_AF
chr4	142236956	.	A	<INS:ME:LINE1>	.	PASS	SVTYPE=INS:ME:LINE1;END=142236967;SVLEN=581;AN=20618;AC=1;AF=4.850130953535746e-05;N_HET=1;N_HOMALT=0;N_HOMREF=10308;FREQ_HET=9.700261907071492e-05;FREQ_HOMALT=0.0;FREQ_HOMREF=0.9999029973809292;N_BI_GENOS=10309;Lingnan_Han_AN=1888;Lingnan_Han_AC=1;Lingna
chr4	142403565	.	G	<INS:ME:LINE1>	.	PASS	SVTYPE=INS:ME:LINE1;END=142403580;SVLEN=6011;AN=20618;AC=2;AF=9.700261907071492e-05;N_HET=2;N_HOMALT=0;N_HOMREF=10307;FREQ_HET=0.00019400523814142983;FREQ_HOMALT=0.0;FREQ_HOMREF=0.9998059947618586;N_BI_GENOS=10309;East_Han_AN=6554;East_Han_AC=1;East_Han_A
chr4	142577075	.	A	<INS:ME:LINE1>	.	PASS	SVTYPE=INS:ME:LINE1;END=142577087;SVLEN=891;AN=20618;AC=1;AF=4.850130953535746e-05;N_HET=1;N_HOMALT=0;N_HOMREF=10308;FREQ_HET=9.700261907071492e-05;FREQ_HOMALT=0.0;FREQ_HOMREF=0.9999029973809292;N_BI_GENOS=10309;East_Han_AN=6554;East_Han_AC=1;East_Han_AF=
chr4	142581811	.	A	<INS:ME:LINE1>	.	PASS	SVTYPE=INS:ME:LINE1;END=142581820;SVLEN=5999;AN=20618;AC=7;AF=0.0003395091667475022;N_HET=7;N_HOMALT=0;N_HOMREF=10302;FREQ_HET=0.0006790183334950044;FREQ_HOMALT=0.0;FREQ_HOMREF=0.999320981666505;N_BI_GENOS=10309;Lingnan_Han_AN=1888;Lingnan_Han_AC=2;Lingna
chr4	142585944	.	G	<INS:ME:LINE1>	.	PASS	SVTYPE=INS:ME:LINE1;END=142585958;SVLEN=4139;AN=20618;AC=1;AF=4.850130953535746e-05;N_HET=1;N_HOMALT=0;N_HOMREF=10308;FREQ_HET=9.700261907071492e-05;FREQ_HOMALT=0.0;FREQ_HOMREF=0.9999029973809292;N_BI_GENOS=10309;East_Han_AN=6554;East_Han_AC=1;East_Han_AF
chr4	142604870	.	T	<INS:ME:LINE1>	.	PASS	SVTYPE=INS:ME:LINE1;END=142604870;SVLEN=195;AN=20618;AC=1;AF=4.850130953535746e-05;N_HET=1;N_HOMALT=0;N_HOMREF=10308;FREQ_HET=9.700261907071492e-05;FREQ_HOMALT=0.0;FREQ_HOMREF=0.9999029973809292;N_BI_GENOS=10309;East_Han_AN=6554;East_Han_AC=1;East_Han_AF=
chr4	142716254	.	A	<INS:ME:LINE1>	.	PASS	SVTYPE=INS:ME:LINE1;END=142716263;SVLEN=471;AN=20618;AC=1;AF=4.850130953535746e-05;N_HET=1;N_HOMALT=0;N_HOMREF=10308;FREQ_HET=9.700261907071492e-05;FREQ_HOMALT=0.0;FREQ_HOMREF=0.9999029973809292;N_BI_GENOS=10309;Central_Han_AN=2128;Central_Han_AC=1;Centra
chr4	142720171	.	A	<INS:ME:LINE1>	.	PASS	SVTYPE=INS:ME:LINE1;END=142720190;SVLEN=93;AN=20618;AC=1;AF=4.850130953535746e-05;N_HET=1;N_HOMALT=0;N_HOMREF=10308;FREQ_HET=9.700261907071492e-05;FREQ_HOMALT=0.0;FREQ_HOMREF=0.9999029973809292;N_BI_GENOS=10309;Manchu_AN=416;Manchu_AC=1;Manchu_AF=0.002403
chr4	142744614	.	A	<INS:ME:LINE1>	.	PASS	SVTYPE=INS:ME:LINE1;END=142744631;SVLEN=849;AN=20618;AC=1;AF=4.850130953535746e-05;N_HET=1;N_HOMALT=0;N_HOMREF=10308;FREQ_HET=9.700261907071492e-05;FREQ_HOMALT=0.0;FREQ_HOMREF=0.9999029973809292;N_BI_GENOS=10309;East_Han_AN=6554;East_Han_AC=1;East_Han_AF=
chr4	142755354	.	T	<INS:ME:LINE1>	.	PASS	SVTYPE=INS:ME:LINE1;END=142755368;SVLEN=618;AN=20618;AC=1;AF=4.850130953535746e-05;N_HET=1;N_HOMALT=0;N_HOMREF=10308;FREQ_HET=9.700261907071492e-05;FREQ_HOMALT=0.0;FREQ_HOMREF=0.9999029973809292;N_BI_GENOS=10309;North_Han_AN=2272;North_Han_AC=1;North_Han_
chr4	143041820	.	T	<INS:ME:LINE1>	.	PASS	SVTYPE=INS:ME:LINE1;END=143041820;SVLEN=566;AN=20618;AC=1;AF=4.850130953535746e-05;N_HET=1;N_HOMALT=0;N_HOMREF=10308;FREQ_HET=9.700261907071492e-05;FREQ_HOMALT=0.0;FREQ_HOMREF=0.9999029973809292;N_BI_GENOS=10309;East_Han_AN=6554;East_Han_AC=1;East_Han_AF=
chr4	143086809	.	T	<INS:ME:LINE1>	.	PASS	SVTYPE=INS:ME:LINE1;END=143086822;SVLEN=400;AN=20618;AC=1;AF=4.850130953535746e-05;N_HET=1;N_HOMALT=0;N_HOMREF=10308;FREQ_HET=9.700261907071492e-05;FREQ_HOMALT=0.0;FREQ_HOMREF=0.9999029973809292;N_BI_GENOS=10309;South_Han_AN=1496;South_Han_AC=1;South_Han_
chr4	143099486	.	C	<INS:ME:LINE1>	.	PASS	SVTYPE=INS:ME:LINE1;END=143099503;SVLEN=5998;AN=20618;AC=385;AF=0.01867300417111262;N_HET=367;N_HOMALT=9;N_HOMREF=9933;FREQ_HET=0.035599961198952375;FREQ_HOMALT=0.0008730235716364342;FREQ_HOMREF=0.9635270152294112;N_BI_GENOS=10309;South_Han_AN=1496;South_
chr4	143236613	.	A	<INS:ME:LINE1>	.	PASS	SVTYPE=INS:ME:LINE1;END=143236625;SVLEN=5960;AN=20618;AC=8;AF=0.0003880104762885966;N_HET=8;N_HOMALT=0;N_HOMREF=10301;FREQ_HET=0.0007760209525657193;FREQ_HOMALT=0.0;FREQ_HOMREF=0.9992239790474343;N_BI_GENOS=10309;South_Han_AN=1496;South_Han_AC=1;South_Ha
chr4	143255627	.	A	<INS:ME:LINE1>	.	PASS	SVTYPE=INS:ME:LINE1;END=143255627;SVLEN=0;AN=20618;AC=1;AF=4.850130953535746e-05;N_HET=1;N_HOMALT=0;N_HOMREF=10308;FREQ_HET=9.700261907071492e-05;FREQ_HOMALT=0.0;FREQ_HOMREF=0.9999029973809292;N_BI_GENOS=10309;Southeast_Han_AN=1908;Southeast_Han_AC=1;Sout
chr4	143642541	.	A	<INS:ME:LINE1>	.	PASS	SVTYPE=INS:ME:LINE1;END=143642541;SVLEN=1463;AN=20618;AC=1;AF=4.850130953535746e-05;N_HET=1;N_HOMALT=0;N_HOMREF=10308;FREQ_HET=9.700261907071492e-05;FREQ_HOMALT=0.0;FREQ_HOMREF=0.9999029973809292;N_BI_GENOS=10309;East_Han_AN=6554;East_Han_AC=1;East_Han_AF
chr4	143769541	.	G	<INS:ME:LINE1>	.	PASS	SVTYPE=INS:ME:LINE1;END=143769567;SVLEN=1067;AN=20618;AC=1;AF=4.850130953535746e-05;N_HET=1;N_HOMALT=0;N_HOMREF=10308;FREQ_HET=9.700261907071492e-05;FREQ_HOMALT=0.0;FREQ_HOMREF=0.9999029973809292;N_BI_GENOS=10309;East_Han_AN=6554;East_Han_AC=1;East_Han_AF
chr4	143769936	.	T	<INS:ME:LINE1>	.	PASS	SVTYPE=INS:ME:LINE1;END=143769936;SVLEN=1111;AN=20618;AC=1;AF=4.850130953535746e-05;N_HET=1;N_HOMALT=0;N_HOMREF=10308;FREQ_HET=9.700261907071492e-05;FREQ_HOMALT=0.0;FREQ_HOMREF=0.9999029973809292;N_BI_GENOS=10309;East_Han_AN=6554;East_Han_AC=1;East_Han_AF
chr4	143770213	.	A	<INS:ME:LINE1>	.	PASS	SVTYPE=INS:ME:LINE1;END=143770213;SVLEN=343;AN=20618;AC=2;AF=9.700261907071492e-05;N_HET=0;N_HOMALT=1;N_HOMREF=10308;FREQ_HET=0.0;FREQ_HOMALT=9.700261907071492e-05;FREQ_HOMREF=0.9999029973809292;N_BI_GENOS=10309;East_Han_AN=6554;East_Han_AC=2;East_Han_AF=
chr4	143770312	.	T	<INS:ME:LINE1>	.	PASS	SVTYPE=INS:ME:LINE1;END=143770312;SVLEN=154;AN=20618;AC=1;AF=4.850130953535746e-05;N_HET=1;N_HOMALT=0;N_HOMREF=10308;FREQ_HET=9.700261907071492e-05;FREQ_HOMALT=0.0;FREQ_HOMREF=0.9999029973809292;N_BI_GENOS=10309;East_Han_AN=6554;East_Han_AC=1;East_Han_AF=
chr4	143901116	.	A	<INS:ME:LINE1>	.	PASS	SVTYPE=INS:ME:LINE1;END=143901131;SVLEN=5997;AN=20618;AC=1;AF=4.850130953535746e-05;N_HET=1;N_HOMALT=0;N_HOMREF=10308;FREQ_HET=9.700261907071492e-05;FREQ_HOMALT=0.0;FREQ_HOMREF=0.9999029973809292;N_BI_GENOS=10309;Southeast_Han_AN=1908;Southeast_Han_AC=1;S
chr4	144250980	.	A	<INS:ME:LINE1>	.	PASS	SVTYPE=INS:ME:LINE1;END=144250995;SVLEN=1077;AN=20618;AC=1;AF=4.850130953535746e-05;N_HET=1;N_HOMALT=0;N_HOMREF=10308;FREQ_HET=9.700261907071492e-05;FREQ_HOMALT=0.0;FREQ_HOMREF=0.9999029973809292;N_BI_GENOS=10309;East_Han_AN=6554;East_Han_AC=1;East_Han_AF
chr4	144398753	.	A	<INS:ME:LINE1>	.	PASS	SVTYPE=INS:ME:LINE1;END=144398753;SVLEN=2555;AN=20618;AC=1652;AF=0.08012416335241052;N_HET=674;N_HOMALT=489;N_HOMREF=9146;FREQ_HET=0.06537976525366185;FREQ_HOMALT=0.04743428072557959;FREQ_HOMREF=0.8871859540207586;N_BI_GENOS=10309;South_Han_AN=1496;South_
chr4	144474156	.	A	<INS:ME:LINE1>	.	PASS	SVTYPE=INS:ME:LINE1;END=144474156;SVLEN=13;AN=20618;AC=1;AF=4.850130953535746e-05;N_HET=1;N_HOMALT=0;N_HOMREF=10308;FREQ_HET=9.700261907071492e-05;FREQ_HOMALT=0.0;FREQ_HOMREF=0.9999029973809292;N_BI_GENOS=10309;North_Han_AN=2272;North_Han_AC=1;North_Han_A
chr4	144478533	.	A	<INS:ME:LINE1>	.	PASS	SVTYPE=INS:ME:LINE1;END=144478533;SVLEN=39;AN=20618;AC=1;AF=4.850130953535746e-05;N_HET=1;N_HOMALT=0;N_HOMREF=10308;FREQ_HET=9.700261907071492e-05;FREQ_HOMALT=0.0;FREQ_HOMREF=0.9999029973809292;N_BI_GENOS=10309;East_Han_AN=6554;East_Han_AC=1;East_Han_AF=0
chr4	144561091	.	T	<INS:ME:LINE1>	.	PASS	SVTYPE=INS:ME:LINE1;END=144561113;SVLEN=3847;AN=20618;AC=1;AF=4.850130953535746e-05;N_HET=1;N_HOMALT=0;N_HOMREF=10308;FREQ_HET=9.700261907071492e-05;FREQ_HOMALT=0.0;FREQ_HOMREF=0.9999029973809292;N_BI_GENOS=10309;Miao_AN=482;Miao_AC=1;Miao_AF=0.0020746887
chr4	144629468	.	T	<INS:ME:LINE1>	.	PASS	SVTYPE=INS:ME:LINE1;END=144629482;SVLEN=567;AN=20618;AC=1;AF=4.850130953535746e-05;N_HET=1;N_HOMALT=0;N_HOMREF=10308;FREQ_HET=9.700261907071492e-05;FREQ_HOMALT=0.0;FREQ_HOMREF=0.9999029973809292;N_BI_GENOS=10309;Mongolian_AN=454;Mongolian_AC=1;Mongolian_A
chr4	144712518	.	T	<INS:ME:LINE1>	.	PASS	SVTYPE=INS:ME:LINE1;END=144712522;SVLEN=260;AN=20618;AC=1;AF=4.850130953535746e-05;N_HET=1;N_HOMALT=0;N_HOMREF=10308;FREQ_HET=9.700261907071492e-05;FREQ_HOMALT=0.0;FREQ_HOMREF=0.9999029973809292;N_BI_GENOS=10309;Central_Han_AN=2128;Central_Han_AC=1;Centra
chr4	144755534	.	C	<INS:ME:LINE1>	.	PASS	SVTYPE=INS:ME:LINE1;END=144755534;SVLEN=5997;AN=20618;AC=1;AF=4.850130953535746e-05;N_HET=1;N_HOMALT=0;N_HOMREF=10308;FREQ_HET=9.700261907071492e-05;FREQ_HOMALT=0.0;FREQ_HOMREF=0.9999029973809292;N_BI_GENOS=10309;Zhuang_AN=444;Zhuang_AC=1;Zhuang_AF=0.0022
chr4	145182888	.	A	<INS:ME:LINE1>	.	PASS	SVTYPE=INS:ME:LINE1;END=145182904;SVLEN=5977;AN=20618;AC=1;AF=4.850130953535746e-05;N_HET=1;N_HOMALT=0;N_HOMREF=10308;FREQ_HET=9.700261907071492e-05;FREQ_HOMALT=0.0;FREQ_HOMREF=0.9999029973809292;N_BI_GENOS=10309;North_Han_AN=2272;North_Han_AC=1;North_Han
chr4	145448979	.	A	<INS:ME:LINE1>	.	PASS	SVTYPE=INS:ME:LINE1;END=145448994;SVLEN=5997;AN=20618;AC=1;AF=4.850130953535746e-05;N_HET=1;N_HOMALT=0;N_HOMREF=10308;FREQ_HET=9.700261907071492e-05;FREQ_HOMALT=0.0;FREQ_HOMREF=0.9999029973809292;N_BI_GENOS=10309;Central_Han_AN=2128;Central_Han_AC=1;Centr
chr4	145700881	.	A	<INS:ME:LINE1>	.	PASS	SVTYPE=INS:ME:LINE1;END=145700881;SVLEN=0;AN=20618;AC=1;AF=4.850130953535746e-05;N_HET=1;N_HOMALT=0;N_HOMREF=10308;FREQ_HET=9.700261907071492e-05;FREQ_HOMALT=0.0;FREQ_HOMREF=0.9999029973809292;N_BI_GENOS=10309;Southeast_Han_AN=1908;Southeast_Han_AC=1;Sout
chr4	146006588	.	C	<INS:ME:LINE1>	.	PASS	SVTYPE=INS:ME:LINE1;END=146006588;SVLEN=642;AN=20618;AC=1;AF=4.850130953535746e-05;N_HET=1;N_HOMALT=0;N_HOMREF=10308;FREQ_HET=9.700261907071492e-05;FREQ_HOMALT=0.0;FREQ_HOMREF=0.9999029973809292;N_BI_GENOS=10309;Central_Han_AN=2128;Central_Han_AC=1;Centra
chr4	146220471	.	T	<INS:ME:LINE1>	.	PASS	SVTYPE=INS:ME:LINE1;END=146220485;SVLEN=6005;AN=20618;AC=22;AF=0.001067028809777864;N_HET=22;N_HOMALT=0;N_HOMREF=10287;FREQ_HET=0.002134057619555728;FREQ_HOMALT=0.0;FREQ_HOMREF=0.9978659423804442;N_BI_GENOS=10309;South_Han_AN=1496;South_Han_AC=1;South_Han
chr4	146304129	.	A	<INS:ME:LINE1>	.	PASS	SVTYPE=INS:ME:LINE1;END=146304144;SVLEN=6006;AN=20618;AC=9797;AF=0.475167329517897;N_HET=2435;N_HOMALT=3681;N_HOMREF=4193;FREQ_HET=0.2362013774371908;FREQ_HOMALT=0.35706664079930156;FREQ_HOMREF=0.4067319817635076;N_BI_GENOS=10309;South_Han_AN=1496;South_H
chr4	146334319	.	A	<INS:ME:LINE1>	.	PASS	SVTYPE=INS:ME:LINE1;END=146334333;SVLEN=363;AN=20618;AC=1;AF=4.850130953535746e-05;N_HET=1;N_HOMALT=0;N_HOMREF=10308;FREQ_HET=9.700261907071492e-05;FREQ_HOMALT=0.0;FREQ_HOMREF=0.9999029973809292;N_BI_GENOS=10309;East_Han_AN=6554;East_Han_AC=1;East_Han_AF=
chr4	146573298	.	A	<INS:ME:LINE1>	.	PASS	SVTYPE=INS:ME:LINE1;END=146573312;SVLEN=866;AN=20618;AC=1;AF=4.850130953535746e-05;N_HET=1;N_HOMALT=0;N_HOMREF=10308;FREQ_HET=9.700261907071492e-05;FREQ_HOMALT=0.0;FREQ_HOMREF=0.9999029973809292;N_BI_GENOS=10309;Central_Han_AN=2128;Central_Han_AC=1;Centra
chr4	146771632	.	A	<INS:ME:LINE1>	.	PASS	SVTYPE=INS:ME:LINE1;END=146771646;SVLEN=976;AN=20618;AC=2;AF=9.700261907071492e-05;N_HET=2;N_HOMALT=0;N_HOMREF=10307;FREQ_HET=0.00019400523814142983;FREQ_HOMALT=0.0;FREQ_HOMREF=0.9998059947618586;N_BI_GENOS=10309;North_Han_AN=2272;North_Han_AC=1;North_Han
chr4	146772652	.	A	<INS:ME:LINE1>	.	PASS	SVTYPE=INS:ME:LINE1;END=146772652;SVLEN=1090;AN=20618;AC=1;AF=4.850130953535746e-05;N_HET=1;N_HOMALT=0;N_HOMREF=10308;FREQ_HET=9.700261907071492e-05;FREQ_HOMALT=0.0;FREQ_HOMREF=0.9999029973809292;N_BI_GENOS=10309;East_Han_AN=6554;East_Han_AC=1;East_Han_AF
chr4	146773066	.	T	<INS:ME:LINE1>	.	PASS	SVTYPE=INS:ME:LINE1;END=146773102;SVLEN=1875;AN=20618;AC=2;AF=9.700261907071492e-05;N_HET=0;N_HOMALT=1;N_HOMREF=10308;FREQ_HET=0.0;FREQ_HOMALT=9.700261907071492e-05;FREQ_HOMREF=0.9999029973809292;N_BI_GENOS=10309;East_Han_AN=6554;East_Han_AC=2;East_Han_AF
chr4	146773838	.	T	<INS:ME:LINE1>	.	PASS	SVTYPE=INS:ME:LINE1;END=146773876;SVLEN=2862;AN=20618;AC=1;AF=4.850130953535746e-05;N_HET=1;N_HOMALT=0;N_HOMREF=10308;FREQ_HET=9.700261907071492e-05;FREQ_HOMALT=0.0;FREQ_HOMREF=0.9999029973809292;N_BI_GENOS=10309;East_Han_AN=6554;East_Han_AC=1;East_Han_AF
chr4	146875624	.	A	<INS:ME:LINE1>	.	PASS	SVTYPE=INS:ME:LINE1;END=146875641;SVLEN=579;AN=20618;AC=1;AF=4.850130953535746e-05;N_HET=1;N_HOMALT=0;N_HOMREF=10308;FREQ_HET=9.700261907071492e-05;FREQ_HOMALT=0.0;FREQ_HOMREF=0.9999029973809292;N_BI_GENOS=10309;East_Han_AN=6554;East_Han_AC=1;East_Han_AF=
chr4	147032790	.	C	<INS:ME:LINE1>	.	PASS	SVTYPE=INS:ME:LINE1;END=147032791;SVLEN=153;AN=20618;AC=1;AF=4.850130953535746e-05;N_HET=1;N_HOMALT=0;N_HOMREF=10308;FREQ_HET=9.700261907071492e-05;FREQ_HOMALT=0.0;FREQ_HOMREF=0.9999029973809292;N_BI_GENOS=10309;North_Han_AN=2272;North_Han_AC=1;North_Han_
chr4	147038036	.	A	<INS:ME:LINE1>	.	PASS	SVTYPE=INS:ME:LINE1;END=147038051;SVLEN=282;AN=20618;AC=1;AF=4.850130953535746e-05;N_HET=1;N_HOMALT=0;N_HOMREF=10308;FREQ_HET=9.700261907071492e-05;FREQ_HOMALT=0.0;FREQ_HOMREF=0.9999029973809292;N_BI_GENOS=10309;East_Han_AN=6554;East_Han_AC=1;East_Han_AF=
chr4	148043095	.	A	<INS:ME:LINE1>	.	PASS	SVTYPE=INS:ME:LINE1;END=148043109;SVLEN=6001;AN=20618;AC=3;AF=0.00014550392860607236;N_HET=3;N_HOMALT=0;N_HOMREF=10306;FREQ_HET=0.00029100785721214447;FREQ_HOMALT=0.0;FREQ_HOMREF=0.9997089921427879;N_BI_GENOS=10309;South_Han_AN=1496;South_Han_AC=1;South_Ha
chr4	148121417	.	A	<INS:ME:LINE1>	.	PASS	SVTYPE=INS:ME:LINE1;END=148121433;SVLEN=1091;AN=20618;AC=9;AF=0.0004365117858182171;N_HET=9;N_HOMALT=0;N_HOMREF=10300;FREQ_HET=0.0008730235716364342;FREQ_HOMALT=0.0;FREQ_HOMREF=0.9991269764283636;N_BI_GENOS=10309;South_Han_AN=1496;South_Han_AC=2;South_Han
chr4	148307865	.	A	<INS:ME:LINE1>	.	PASS	SVTYPE=INS:ME:LINE1;END=148307882;SVLEN=5998;AN=20618;AC=9;AF=0.0004365117858182171;N_HET=9;N_HOMALT=0;N_HOMREF=10300;FREQ_HET=0.0008730235716364342;FREQ_HOMALT=0.0;FREQ_HOMREF=0.9991269764283636;N_BI_GENOS=10309;South_Han_AN=1496;South_Han_AC=1;South_Han
chr4	148524207	.	C	<INS:ME:LINE1>	.	PASS	SVTYPE=INS:ME:LINE1;END=148524222;SVLEN=6008;AN=20618;AC=1;AF=4.850130953535746e-05;N_HET=1;N_HOMALT=0;N_HOMREF=10308;FREQ_HET=9.700261907071492e-05;FREQ_HOMALT=0.0;FREQ_HOMREF=0.9999029973809292;N_BI_GENOS=10309;South_Han_AN=1496;South_Han_AC=1;South_Han
chr4	148614678	.	A	<INS:ME:LINE1>	.	PASS	SVTYPE=INS:ME:LINE1;END=148614693;SVLEN=6010;AN=20618;AC=1;AF=4.850130953535746e-05;N_HET=1;N_HOMALT=0;N_HOMREF=10308;FREQ_HET=9.700261907071492e-05;FREQ_HOMALT=0.0;FREQ_HOMREF=0.9999029973809292;N_BI_GENOS=10309;South_Han_AN=1496;South_Han_AC=1;South_Han
chr4	148651484	.	T	<INS:ME:LINE1>	.	PASS	SVTYPE=INS:ME:LINE1;END=148651484;SVLEN=144;AN=20618;AC=1;AF=4.850130953535746e-05;N_HET=1;N_HOMALT=0;N_HOMREF=10308;FREQ_HET=9.700261907071492e-05;FREQ_HOMALT=0.0;FREQ_HOMREF=0.9999029973809292;N_BI_GENOS=10309;Tibetan_AN=414;Tibetan_AC=1;Tibetan_AF=0.00
chr4	149445821	.	G	<INS:ME:LINE1>	.	PASS	SVTYPE=INS:ME:LINE1;END=149445836;SVLEN=470;AN=20618;AC=1;AF=4.850130953535746e-05;N_HET=1;N_HOMALT=0;N_HOMREF=10308;FREQ_HET=9.700261907071492e-05;FREQ_HOMALT=0.0;FREQ_HOMREF=0.9999029973809292;N_BI_GENOS=10309;North_Han_AN=2272;North_Han_AC=1;North_Han_
chr4	149877675	.	G	<INS:ME:LINE1>	.	PASS	SVTYPE=INS:ME:LINE1;END=149877690;SVLEN=5998;AN=20618;AC=2818;AF=0.13667669027063733;N_HET=2232;N_HOMALT=293;N_HOMREF=7784;FREQ_HET=0.21650984576583568;FREQ_HOMALT=0.028421767387719468;FREQ_HOMREF=0.7550683868464448;N_BI_GENOS=10309;South_Han_AN=1496;South
chr4	149886387	.	A	<INS:ME:LINE1>	.	PASS	SVTYPE=INS:ME:LINE1;END=149886394;SVLEN=6008;AN=20618;AC=2;AF=9.700261907071492e-05;N_HET=2;N_HOMALT=0;N_HOMREF=10307;FREQ_HET=0.00019400523814142983;FREQ_HOMALT=0.0;FREQ_HOMREF=0.9998059947618586;N_BI_GENOS=10309;Southeast_Han_AN=1908;Southeast_Han_AC=2;
chr4	150128993	.	A	<INS:ME:LINE1>	.	PASS	SVTYPE=INS:ME:LINE1;END=150129007;SVLEN=1201;AN=20618;AC=1;AF=4.850130953535746e-05;N_HET=1;N_HOMALT=0;N_HOMREF=10308;FREQ_HET=9.700261907071492e-05;FREQ_HOMALT=0.0;FREQ_HOMREF=0.9999029973809292;N_BI_GENOS=10309;Yi_AN=422;Yi_AC=1;Yi_AF=0.0023696682464454
chr4	150156243	.	A	<INS:ME:LINE1>	.	PASS	SVTYPE=INS:ME:LINE1;END=150156258;SVLEN=6005;AN=20618;AC=1;AF=4.850130953535746e-05;N_HET=1;N_HOMALT=0;N_HOMREF=10308;FREQ_HET=9.700261907071492e-05;FREQ_HOMALT=0.0;FREQ_HOMREF=0.9999029973809292;N_BI_GENOS=10309;South_Han_AN=1496;South_Han_AC=1;South_Han
chr4	151409259	.	C	<INS:ME:LINE1>	.	PASS	SVTYPE=INS:ME:LINE1;END=151409259;SVLEN=0;AN=20618;AC=19;AF=0.0009215248811717916;N_HET=19;N_HOMALT=0;N_HOMREF=10290;FREQ_HET=0.0018430497623435833;FREQ_HOMALT=0.0;FREQ_HOMREF=0.9981569502376564;N_BI_GENOS=10309;South_Han_AN=1496;South_Han_AC=1;South_Han_
chr4	151427683	.	T	<INS:ME:LINE1>	.	PASS	SVTYPE=INS:ME:LINE1;END=151427693;SVLEN=5999;AN=20618;AC=1;AF=4.850130953535746e-05;N_HET=1;N_HOMALT=0;N_HOMREF=10308;FREQ_HET=9.700261907071492e-05;FREQ_HOMALT=0.0;FREQ_HOMREF=0.9999029973809292;N_BI_GENOS=10309;Southeast_Han_AN=1908;Southeast_Han_AC=1;S
chr4	151507628	.	A	<INS:ME:LINE1>	.	PASS	SVTYPE=INS:ME:LINE1;END=151507641;SVLEN=5989;AN=20618;AC=1;AF=4.850130953535746e-05;N_HET=1;N_HOMALT=0;N_HOMREF=10308;FREQ_HET=9.700261907071492e-05;FREQ_HOMALT=0.0;FREQ_HOMREF=0.9999029973809292;N_BI_GENOS=10309;Mongolian_AN=454;Mongolian_AC=1;Mongolian_
chr4	151585975	.	A	<INS:ME:LINE1>	.	PASS	SVTYPE=INS:ME:LINE1;END=151585980;SVLEN=107;AN=20618;AC=1;AF=4.850130953535746e-05;N_HET=1;N_HOMALT=0;N_HOMREF=10308;FREQ_HET=9.700261907071492e-05;FREQ_HOMALT=0.0;FREQ_HOMREF=0.9999029973809292;N_BI_GENOS=10309;Hui_AN=444;Hui_AC=1;Hui_AF=0.00225225225225
chr4	151637640	.	T	<INS:ME:LINE1>	.	PASS	SVTYPE=INS:ME:LINE1;END=151637645;SVLEN=211;AN=20618;AC=2;AF=9.700261907071492e-05;N_HET=2;N_HOMALT=0;N_HOMREF=10307;FREQ_HET=0.00019400523814142983;FREQ_HOMALT=0.0;FREQ_HOMREF=0.9998059947618586;N_BI_GENOS=10309;East_Han_AN=6554;East_Han_AC=2;East_Han_AF
chr4	151811657	.	T	<INS:ME:LINE1>	.	PASS	SVTYPE=INS:ME:LINE1;END=151811671;SVLEN=5998;AN=20618;AC=14;AF=0.0006790183334950044;N_HET=14;N_HOMALT=0;N_HOMREF=10295;FREQ_HET=0.0013580366669900088;FREQ_HOMALT=0.0;FREQ_HOMREF=0.99864196333301;N_BI_GENOS=10309;Northwest_Han_AN=1296;Northwest_Han_AC=1;N
chr4	152155887	.	A	<INS:ME:LINE1>	.	PASS	SVTYPE=INS:ME:LINE1;END=152155887;SVLEN=6040;AN=20618;AC=1;AF=4.850130953535746e-05;N_HET=1;N_HOMALT=0;N_HOMREF=10308;FREQ_HET=9.700261907071492e-05;FREQ_HOMALT=0.0;FREQ_HOMREF=0.9999029973809292;N_BI_GENOS=10309;East_Han_AN=6554;East_Han_AC=1;East_Han_AF
chr4	152424617	.	C	<INS:ME:LINE1>	.	PASS	SVTYPE=INS:ME:LINE1;END=152424637;SVLEN=1296;AN=20618;AC=2;AF=9.700261907071492e-05;N_HET=2;N_HOMALT=0;N_HOMREF=10307;FREQ_HET=0.00019400523814142983;FREQ_HOMALT=0.0;FREQ_HOMREF=0.9998059947618586;N_BI_GENOS=10309;South_Han_AN=1496;South_Han_AC=2;South_Ha
chr4	152701104	.	A	<INS:ME:LINE1>	.	PASS	SVTYPE=INS:ME:LINE1;END=152701104;SVLEN=0;AN=20618;AC=4;AF=0.00019400523814142983;N_HET=0;N_HOMALT=2;N_HOMREF=10307;FREQ_HET=0.0;FREQ_HOMALT=0.00019400523814142983;FREQ_HOMREF=0.9998059947618586;N_BI_GENOS=10309;North_Han_AN=2272;North_Han_AC=2;North_Han_
chr4	153097554	.	A	<INS:ME:LINE1>	.	PASS	SVTYPE=INS:ME:LINE1;END=153097554;SVLEN=6045;AN=20618;AC=1;AF=4.850130953535746e-05;N_HET=1;N_HOMALT=0;N_HOMREF=10308;FREQ_HET=9.700261907071492e-05;FREQ_HOMALT=0.0;FREQ_HOMREF=0.9999029973809292;N_BI_GENOS=10309;North_Han_AN=2272;North_Han_AC=1;North_Han
chr4	153356775	.	G	<INS:ME:LINE1>	.	PASS	SVTYPE=INS:ME:LINE1;END=153356789;SVLEN=6008;AN=20618;AC=2;AF=9.700261907071492e-05;N_HET=2;N_HOMALT=0;N_HOMREF=10307;FREQ_HET=0.00019400523814142983;FREQ_HOMALT=0.0;FREQ_HOMREF=0.9998059947618586;N_BI_GENOS=10309;North_Han_AN=2272;North_Han_AC=1;North_Ha
chr4	153543231	.	T	<INS:ME:LINE1>	.	PASS	SVTYPE=INS:ME:LINE1;END=153543246;SVLEN=6066;AN=20618;AC=1;AF=4.850130953535746e-05;N_HET=1;N_HOMALT=0;N_HOMREF=10308;FREQ_HET=9.700261907071492e-05;FREQ_HOMALT=0.0;FREQ_HOMREF=0.9999029973809292;N_BI_GENOS=10309;Yi_AN=422;Yi_AC=1;Yi_AF=0.0023696682464454
chr4	153674461	.	A	<INS:ME:LINE1>	.	PASS	SVTYPE=INS:ME:LINE1;END=153674477;SVLEN=1255;AN=20618;AC=1;AF=4.850130953535746e-05;N_HET=1;N_HOMALT=0;N_HOMREF=10308;FREQ_HET=9.700261907071492e-05;FREQ_HOMALT=0.0;FREQ_HOMREF=0.9999029973809292;N_BI_GENOS=10309;Mongolian_AN=454;Mongolian_AC=1;Mongolian_
chr4	153835649	.	A	<INS:ME:LINE1>	.	PASS	SVTYPE=INS:ME:LINE1;END=153835655;SVLEN=268;AN=20618;AC=1;AF=4.850130953535746e-05;N_HET=1;N_HOMALT=0;N_HOMREF=10308;FREQ_HET=9.700261907071492e-05;FREQ_HOMALT=0.0;FREQ_HOMREF=0.9999029973809292;N_BI_GENOS=10309;East_Han_AN=6554;East_Han_AC=1;East_Han_AF=

chr4	153913412	.	T	<INS:ME:LINE1>	.	PASS	SVTYPE=INS:ME:LINE1;END=153913416;SVLEN=1389;AN=20618;AC=3;AF=0.00014550392860607236;N_HET=3;N_HOMALT=0;N_HOMREF=10306;FREQ_HET=0.0002910078572121447;FREQ_HOMALT=0.0;FREQ_HOMREF=0.9997089921427879;N_BI_GENOS=10309;North_Han_AN=2272;North_Han_AC=1;North_Ha
chr4	154085965	.	C	<INS:ME:LINE1>	.	PASS	SVTYPE=INS:ME:LINE1;END=154085980;SVLEN=6000;AN=20618;AC=5;AF=0.00024250654767678728;N_HET=5;N_HOMALT=0;N_HOMREF=10304;FREQ_HET=0.00048501309535357455;FREQ_HOMALT=0.0;FREQ_HOMREF=0.9995149869046465;N_BI_GENOS=10309;Lingnan_Han_AN=1888;Lingnan_Han_AC=2;Lin
chr4	154169613	.	T	<INS:ME:LINE1>	.	PASS	SVTYPE=INS:ME:LINE1;END=154169663;SVLEN=2234;AN=20618;AC=2;AF=9.700261907071492e-05;N_HET=0;N_HOMALT=1;N_HOMREF=10308;FREQ_HET=0.0;FREQ_HOMALT=9.700261907071492e-05;FREQ_HOMREF=0.9999029973809292;N_BI_GENOS=10309;Central_Han_AN=2128;Central_Han_AC=2;Centr
chr4	154201529	.	T	<INS:ME:LINE1>	.	PASS	SVTYPE=INS:ME:LINE1;END=154201529;SVLEN=4;AN=20618;AC=2;AF=9.700261907071492e-05;N_HET=2;N_HOMALT=0;N_HOMREF=10307;FREQ_HET=0.00019400523814142983;FREQ_HOMALT=0.0;FREQ_HOMREF=0.9998059947618586;N_BI_GENOS=10309;Central_Han_AN=2128;Central_Han_AC=1;Central
chr4	154594227	.	A	<INS:ME:LINE1>	.	PASS	SVTYPE=INS:ME:LINE1;END=154594243;SVLEN=6004;AN=20618;AC=2;AF=9.700261907071492e-05;N_HET=2;N_HOMALT=0;N_HOMREF=10307;FREQ_HET=0.00019400523814142983;FREQ_HOMALT=0.0;FREQ_HOMREF=0.9998059947618586;N_BI_GENOS=10309;South_Han_AN=1496;South_Han_AC=1;South_Ha
chr4	154901880	.	C	<INS:ME:LINE1>	.	PASS	SVTYPE=INS:ME:LINE1;END=154901894;SVLEN=1035;AN=20618;AC=1;AF=4.850130953535746e-05;N_HET=1;N_HOMALT=0;N_HOMREF=10308;FREQ_HET=9.700261907071492e-05;FREQ_HOMALT=0.0;FREQ_HOMREF=0.9999029973809292;N_BI_GENOS=10309;North_Han_AN=2272;North_Han_AC=1;North_Han
chr4	155143395	.	C	<INS:ME:LINE1>	.	PASS	SVTYPE=INS:ME:LINE1;END=155143405;SVLEN=690;AN=20618;AC=1;AF=4.850130953535746e-05;N_HET=1;N_HOMALT=0;N_HOMREF=10308;FREQ_HET=9.700261907071492e-05;FREQ_HOMALT=0.0;FREQ_HOMREF=0.9999029973809292;N_BI_GENOS=10309;Central_Han_AN=2128;Central_Han_AC=1;Centra
chr4	155189509	.	T	<INS:ME:LINE1>	.	PASS	SVTYPE=INS:ME:LINE1;END=155189524;SVLEN=698;AN=20618;AC=2;AF=9.700261907071492e-05;N_HET=2;N_HOMALT=0;N_HOMREF=10307;FREQ_HET=0.00019400523814142983;FREQ_HOMALT=0.0;FREQ_HOMREF=0.9998059947618586;N_BI_GENOS=10309;East_Han_AN=6554;East_Han_AC=1;East_Han_AF
chr4	155235068	.	C	<INS:ME:LINE1>	.	PASS	SVTYPE=INS:ME:LINE1;END=155235077;SVLEN=6197;AN=20618;AC=1;AF=4.850130953535746e-05;N_HET=1;N_HOMALT=0;N_HOMREF=10308;FREQ_HET=9.700261907071492e-05;FREQ_HOMALT=0.0;FREQ_HOMREF=0.9999029973809292;N_BI_GENOS=10309;East_Han_AN=6554;East_Han_AC=1;East_Han_AF
chr4	156195195	.	A	<INS:ME:LINE1>	.	PASS	SVTYPE=INS:ME:LINE1;END=156195213;SVLEN=637;AN=20618;AC=1;AF=4.850130953535746e-05;N_HET=1;N_HOMALT=0;N_HOMREF=10308;FREQ_HET=9.700261907071492e-05;FREQ_HOMALT=0.0;FREQ_HOMREF=0.9999029973809292;N_BI_GENOS=10309;Lingnan_Han_AN=1888;Lingnan_Han_AC=1;Lingna
chr4	156236532	.	T	<INS:ME:LINE1>	.	PASS	SVTYPE=INS:ME:LINE1;END=156236533;SVLEN=1711;AN=20618;AC=25;AF=0.0012125327383839363;N_HET=25;N_HOMALT=0;N_HOMREF=10284;FREQ_HET=0.0024250654767678727;FREQ_HOMALT=0.0;FREQ_HOMREF=0.9975749345232321;N_BI_GENOS=10309;South_Han_AN=1496;South_Han_AC=2;South_H
chr4	156272387	.	C	<INS:ME:LINE1>	.	PASS	SVTYPE=INS:ME:LINE1;END=156272409;SVLEN=845;AN=20618;AC=1;AF=4.850130953535746e-05;N_HET=1;N_HOMALT=0;N_HOMREF=10308;FREQ_HET=9.700261907071492e-05;FREQ_HOMALT=0.0;FREQ_HOMREF=0.9999029973809292;N_BI_GENOS=10309;East_Han_AN=6554;East_Han_AC=1;East_Han_AF=
chr4	156605929	.	G	<INS:ME:LINE1>	.	PASS	SVTYPE=INS:ME:LINE1;END=156605929;SVLEN=0;AN=20618;AC=2;AF=9.700261907071492e-05;N_HET=2;N_HOMALT=0;N_HOMREF=10307;FREQ_HET=0.00019400523814142983;FREQ_HOMALT=0.0;FREQ_HOMREF=0.9998059947618586;N_BI_GENOS=10309;North_Han_AN=2272;North_Han_AC=1;North_Han_A
chr4	156606021	.	G	<INS:ME:LINE1>	.	PASS	SVTYPE=INS:ME:LINE1;END=156606021;SVLEN=0;AN=20618;AC=1;AF=4.850130953535746e-05;N_HET=1;N_HOMALT=0;N_HOMREF=10308;FREQ_HET=9.700261907071492e-05;FREQ_HOMALT=0.0;FREQ_HOMREF=0.9999029973809292;N_BI_GENOS=10309;Southeast_Han_AN=1908;Southeast_Han_AC=1;Sout
chr4	156634105	.	A	<INS:ME:LINE1>	.	PASS	SVTYPE=INS:ME:LINE1;END=156634105;SVLEN=1942;AN=20618;AC=2;AF=9.700261907071492e-05;N_HET=2;N_HOMALT=0;N_HOMREF=10307;FREQ_HET=0.00019400523814142983;FREQ_HOMALT=0.0;FREQ_HOMREF=0.9998059947618586;N_BI_GENOS=10309;East_Han_AN=6554;East_Han_AC=2;East_Han_A
chr4	156673278	.	C	<INS:ME:LINE1>	.	PASS	SVTYPE=INS:ME:LINE1;END=156673294;SVLEN=2109;AN=20618;AC=3;AF=0.00014550392860607236;N_HET=3;N_HOMALT=0;N_HOMREF=10306;FREQ_HET=0.0002910078572121447;FREQ_HOMALT=0.0;FREQ_HOMREF=0.9997089921427879;N_BI_GENOS=10309;Lingnan_Han_AN=1888;Lingnan_Han_AC=1;Ling
chr4	156777135	.	T	<INS:ME:LINE1>	.	PASS	SVTYPE=INS:ME:LINE1;END=156777148;SVLEN=114;AN=20618;AC=6;AF=0.0002910078572121447;N_HET=4;N_HOMALT=1;N_HOMREF=10304;FREQ_HET=0.00038801047628285966;FREQ_HOMALT=9.700261907071492e-05;FREQ_HOMREF=0.9995149869046465;N_BI_GENOS=10309;South_Han_AN=1496;South_
chr4	157319285	.	A	<INS:ME:LINE1>	.	PASS	SVTYPE=INS:ME:LINE1;END=157319300;SVLEN=270;AN=20618;AC=1;AF=4.850130953535746e-05;N_HET=1;N_HOMALT=0;N_HOMREF=10308;FREQ_HET=9.700261907071492e-05;FREQ_HOMALT=0.0;FREQ_HOMREF=0.9999029973809292;N_BI_GENOS=10309;Tibetan_AN=414;Tibetan_AC=1;Tibetan_AF=0.00
chr4	157509201	.	A	<INS:ME:LINE1>	.	PASS	SVTYPE=INS:ME:LINE1;END=157509214;SVLEN=6081;AN=20618;AC=3;AF=0.00014550392860607236;N_HET=3;N_HOMALT=0;N_HOMREF=10306;FREQ_HET=0.0002910078572121447;FREQ_HOMALT=0.0;FREQ_HOMREF=0.9997089921427879;N_BI_GENOS=10309;Northwest_Han_AN=1296;Northwest_Han_AC=1;
chr4	157685860	.	A	<INS:ME:LINE1>	.	PASS	SVTYPE=INS:ME:LINE1;END=157685872;SVLEN=2066;AN=20618;AC=1;AF=4.850130953535746e-05;N_HET=1;N_HOMALT=0;N_HOMREF=10308;FREQ_HET=9.700261907071492e-05;FREQ_HOMALT=0.0;FREQ_HOMREF=0.9999029973809292;N_BI_GENOS=10309;Southeast_Han_AN=1908;Southeast_Han_AC=1;S
chr4	157744295	.	T	<INS:ME:LINE1>	.	PASS	SVTYPE=INS:ME:LINE1;END=157744296;SVLEN=381;AN=20618;AC=1;AF=4.850130953535746e-05;N_HET=1;N_HOMALT=0;N_HOMREF=10308;FREQ_HET=9.700261907071492e-05;FREQ_HOMALT=0.0;FREQ_HOMREF=0.9999029973809292;N_BI_GENOS=10309;Central_Han_AN=2128;Central_Han_AC=1;Centra
chr4	157986119	.	G	<INS:ME:LINE1>	.	PASS	SVTYPE=INS:ME:LINE1;END=157986137;SVLEN=5997;AN=20618;AC=3399;AF=0.16485595111068;N_HET=2695;N_HOMALT=352;N_HOMREF=7262;FREQ_HET=0.2614220583955767;FREQ_HOMALT=0.034144921912891646;FREQ_HOMREF=0.7044330196915317;N_BI_GENOS=10309;South_Han_AN=1496;South_Ha
chr4	158309553	.	A	<INS:ME:LINE1>	.	PASS	SVTYPE=INS:ME:LINE1;END=158309553;SVLEN=184;AN=20618;AC=1;AF=4.850130953535746e-05;N_HET=1;N_HOMALT=0;N_HOMREF=10308;FREQ_HET=9.700261907071492e-05;FREQ_HOMALT=0.0;FREQ_HOMREF=0.9999029973809292;N_BI_GENOS=10309;North_Han_AN=2272;North_Han_AC=1;North_Han_
chr4	158391839	.	A	<INS:ME:LINE1>	.	PASS	SVTYPE=INS:ME:LINE1;END=158391844;SVLEN=1805;AN=20618;AC=1;AF=4.850130953535746e-05;N_HET=1;N_HOMALT=0;N_HOMREF=10308;FREQ_HET=9.700261907071492e-05;FREQ_HOMALT=0.0;FREQ_HOMREF=0.9999029973809292;N_BI_GENOS=10309;South_Han_AN=1496;South_Han_AC=1;South_Han
chr4	158551988	.	A	<INS:ME:LINE1>	.	PASS	SVTYPE=INS:ME:LINE1;END=158552003;SVLEN=5999;AN=20618;AC=1;AF=4.850130953535746e-05;N_HET=1;N_HOMALT=0;N_HOMREF=10308;FREQ_HET=9.700261907071492e-05;FREQ_HOMALT=0.0;FREQ_HOMREF=0.9999029973809292;N_BI_GENOS=10309;Northwest_Han_AN=1296;Northwest_Han_AC=1;N
chr4	158878862	.	G	<INS:ME:LINE1>	.	PASS	SVTYPE=INS:ME:LINE1;END=158878862;SVLEN=411;AN=20618;AC=3;AF=0.00014550392860607236;N_HET=3;N_HOMALT=0;N_HOMREF=10306;FREQ_HET=0.0002910078572121447;FREQ_HOMALT=0.0;FREQ_HOMREF=0.9997089921427879;N_BI_GENOS=10309;Lingnan_Han_AN=1888;Lingnan_Han_AC=1;Lingn
chr4	158994197	.	C	<INS:ME:LINE1>	.	PASS	SVTYPE=INS:ME:LINE1;END=158994211;SVLEN=5986;AN=20618;AC=2;AF=9.700261907071492e-05;N_HET=2;N_HOMALT=0;N_HOMREF=10307;FREQ_HET=0.00019400523814142983;FREQ_HOMALT=0.0;FREQ_HOMREF=0.9998059947618586;N_BI_GENOS=10309;South_Han_AN=1496;South_Han_AC=1;South_Ha
chr4	159299173	.	T	<INS:ME:LINE1>	.	PASS	SVTYPE=INS:ME:LINE1;END=159299186;SVLEN=977;AN=20618;AC=1;AF=4.850130953535746e-05;N_HET=1;N_HOMALT=0;N_HOMREF=10308;FREQ_HET=9.700261907071492e-05;FREQ_HOMALT=0.0;FREQ_HOMREF=0.9999029973809292;N_BI_GENOS=10309;Southeast_Han_AN=1908;Southeast_Han_AC=1;So
chr4	159339430	.	T	<INS:ME:LINE1>	.	PASS	SVTYPE=INS:ME:LINE1;END=159339444;SVLEN=6047;AN=20618;AC=1;AF=4.850130953535746e-05;N_HET=1;N_HOMALT=0;N_HOMREF=10308;FREQ_HET=9.700261907071492e-05;FREQ_HOMALT=0.0;FREQ_HOMREF=0.9999029973809292;N_BI_GENOS=10309;Central_Han_AN=2128;Central_Han_AC=1;Centr
chr4	159745996	.	A	<INS:ME:LINE1>	.	PASS	SVTYPE=INS:ME:LINE1;END=159746012;SVLEN=1174;AN=20618;AC=1;AF=4.850130953535746e-05;N_HET=1;N_HOMALT=0;N_HOMREF=10308;FREQ_HET=9.700261907071492e-05;FREQ_HOMALT=0.0;FREQ_HOMREF=0.9999029973809292;N_BI_GENOS=10309;East_Han_AN=6554;East_Han_AC=1;East_Han_AF
chr4	159985847	.	G	<INS:ME:LINE1>	.	PASS	SVTYPE=INS:ME:LINE1;END=159985847;SVLEN=142;AN=20618;AC=1;AF=4.850130953535746e-05;N_HET=1;N_HOMALT=0;N_HOMREF=10308;FREQ_HET=9.700261907071492e-05;FREQ_HOMALT=0.0;FREQ_HOMREF=0.9999029973809292;N_BI_GENOS=10309;Manchu_AN=416;Manchu_AC=1;Manchu_AF=0.00240
chr4	160033445	.	A	<INS:ME:LINE1>	.	PASS	SVTYPE=INS:ME:LINE1;END=160033461;SVLEN=757;AN=20618;AC=22;AF=0.001067028809777864;N_HET=22;N_HOMALT=0;N_HOMREF=10287;FREQ_HET=0.002134057619555728;FREQ_HOMALT=0.0;FREQ_HOMREF=0.9978659423804442;N_BI_GENOS=10309;South_Han_AN=1496;South_Han_AC=1;South_Han_
chr4	160103250	.	T	<INS:ME:LINE1>	.	PASS	SVTYPE=INS:ME:LINE1;END=160103250;SVLEN=171;AN=20618;AC=1;AF=4.850130953535746e-05;N_HET=1;N_HOMALT=0;N_HOMREF=10308;FREQ_HET=9.700261907071492e-05;FREQ_HOMALT=0.0;FREQ_HOMREF=0.9999029973809292;N_BI_GENOS=10309;East_Han_AN=6554;East_Han_AC=1;East_Han_AF=
chr4	160345503	.	T	<INS:ME:LINE1>	.	PASS	SVTYPE=INS:ME:LINE1;END=160345513;SVLEN=1105;AN=20618;AC=1;AF=4.850130953535746e-05;N_HET=1;N_HOMALT=0;N_HOMREF=10308;FREQ_HET=9.700261907071492e-05;FREQ_HOMALT=0.0;FREQ_HOMREF=0.9999029973809292;N_BI_GENOS=10309;Lingnan_Han_AN=1888;Lingnan_Han_AC=1;Lingn
chr4	160519465	.	T	<INS:ME:LINE1>	.	PASS	SVTYPE=INS:ME:LINE1;END=160519481;SVLEN=527;AN=20618;AC=5;AF=0.00024250654767678728;N_HET=5;N_HOMALT=0;N_HOMREF=10304;FREQ_HET=0.00048501309535357455;FREQ_HOMALT=0.0;FREQ_HOMREF=0.9995149869046465;N_BI_GENOS=10309;North_Han_AN=2272;North_Han_AC=1;North_Ha
chr4	160519725	.	T	<INS:ME:LINE1>	.	PASS	SVTYPE=INS:ME:LINE1;END=160519741;SVLEN=992;AN=20618;AC=24;AF=0.0011640314288485789;N_HET=24;N_HOMALT=0;N_HOMREF=10285;FREQ_HET=0.0023280628576971578;FREQ_HOMALT=0.0;FREQ_HOMREF=0.9976719371423028;N_BI_GENOS=10309;South_Han_AN=1496;South_Han_AC=3;South_Ha
chr4	160613386	.	A	<INS:ME:LINE1>	.	PASS	SVTYPE=INS:ME:LINE1;END=160613403;SVLEN=811;AN=20618;AC=1;AF=4.850130953535746e-05;N_HET=1;N_HOMALT=0;N_HOMREF=10308;FREQ_HET=9.700261907071492e-05;FREQ_HOMALT=0.0;FREQ_HOMREF=0.9999029973809292;N_BI_GENOS=10309;Tibetan_AN=414;Tibetan_AC=1;Tibetan_AF=0.00
chr4	160895021	.	T	<INS:ME:LINE1>	.	PASS	SVTYPE=INS:ME:LINE1;END=160895032;SVLEN=2199;AN=20618;AC=2;AF=9.700261907071492e-05;N_HET=2;N_HOMALT=0;N_HOMREF=10307;FREQ_HET=0.00019400523814142983;FREQ_HOMALT=0.0;FREQ_HOMREF=0.9998059947618586;N_BI_GENOS=10309;South_Han_AN=1496;South_Han_AC=2;South_Ha
chr4	160908268	.	A	<INS:ME:LINE1>	.	PASS	SVTYPE=INS:ME:LINE1;END=160908268;SVLEN=801;AN=20618;AC=1;AF=4.850130953535746e-05;N_HET=1;N_HOMALT=0;N_HOMREF=10308;FREQ_HET=9.700261907071492e-05;FREQ_HOMALT=0.0;FREQ_HOMREF=0.9999029973809292;N_BI_GENOS=10309;Lingnan_Han_AN=1888;Lingnan_Han_AC=1;Lingna
chr4	160950885	.	T	<INS:ME:LINE1>	.	PASS	SVTYPE=INS:ME:LINE1;END=160950885;SVLEN=197;AN=20618;AC=1;AF=4.850130953535746e-05;N_HET=1;N_HOMALT=0;N_HOMREF=10308;FREQ_HET=9.700261907071492e-05;FREQ_HOMALT=0.0;FREQ_HOMREF=0.9999029973809292;N_BI_GENOS=10309;East_Han_AN=6554;East_Han_AC=1;East_Han_AF=
chr4	161165142	.	T	<INS:ME:LINE1>	.	PASS	SVTYPE=INS:ME:LINE1;END=161165156;SVLEN=1589;AN=20618;AC=8;AF=0.00038801047628285966;N_HET=8;N_HOMALT=0;N_HOMREF=10301;FREQ_HET=0.0007760209525657193;FREQ_HOMALT=0.0;FREQ_HOMREF=0.9992239790474343;N_BI_GENOS=10309;South_Han_AN=1496;South_Han_AC=3;South_Ha
chr4	161530514	.	A	<INS:ME:LINE1>	.	PASS	SVTYPE=INS:ME:LINE1;END=161530514;SVLEN=111;AN=20618;AC=8;AF=0.00038801047628285966;N_HET=4;N_HOMALT=2;N_HOMREF=10303;FREQ_HET=0.00038801047628285966;FREQ_HOMALT=0.00019400523814142983;FREQ_HOMREF=0.9994179842855757;N_BI_GENOS=10309;Lingnan_Han_AN=1888;Li
chr4	162018653	.	A	<INS:ME:LINE1>	.	PASS	SVTYPE=INS:ME:LINE1;END=162018663;SVLEN=1333;AN=20618;AC=1;AF=4.850130953535746e-05;N_HET=1;N_HOMALT=0;N_HOMREF=10308;FREQ_HET=9.700261907071492e-05;FREQ_HOMALT=0.0;FREQ_HOMREF=0.9999029973809292;N_BI_GENOS=10309;Northwest_Han_AN=1296;Northwest_Han_AC=1;N
chr4	162173047	.	T	<INS:ME:LINE1>	.	PASS	SVTYPE=INS:ME:LINE1;END=162173057;SVLEN=40;AN=20618;AC=4;AF=0.00019400523814142983;N_HET=4;N_HOMALT=0;N_HOMREF=10305;FREQ_HET=0.00038801047628285966;FREQ_HOMALT=0.0;FREQ_HOMREF=0.9996119895237171;N_BI_GENOS=10309;Lingnan_Han_AN=1888;Lingnan_Han_AC=1;Lingn
chr4	162242917	.	A	<INS:ME:LINE1>	.	PASS	SVTYPE=INS:ME:LINE1;END=162242934;SVLEN=384;AN=20618;AC=8;AF=0.00038801047628285966;N_HET=8;N_HOMALT=0;N_HOMREF=10301;FREQ_HET=0.0007760209525657193;FREQ_HOMALT=0.0;FREQ_HOMREF=0.9992239790474343;N_BI_GENOS=10309;Northwest_Han_AN=1296;Northwest_Han_AC=1;N
chr4	162434086	.	T	<INS:ME:LINE1>	.	PASS	SVTYPE=INS:ME:LINE1;END=162434086;SVLEN=84;AN=20618;AC=1;AF=4.850130953535746e-05;N_HET=1;N_HOMALT=0;N_HOMREF=10308;FREQ_HET=9.700261907071492e-05;FREQ_HOMALT=0.0;FREQ_HOMREF=0.9999029973809292;N_BI_GENOS=10309;East_Han_AN=6554;East_Han_AC=1;East_Han_AF=0
chr4	162434432	.	G	<INS:ME:LINE1>	.	PASS	SVTYPE=INS:ME:LINE1;END=162434432;SVLEN=210;AN=20618;AC=1;AF=4.850130953535746e-05;N_HET=1;N_HOMALT=0;N_HOMREF=10308;FREQ_HET=9.700261907071492e-05;FREQ_HOMALT=0.0;FREQ_HOMREF=0.9999029973809292;N_BI_GENOS=10309;East_Han_AN=6554;East_Han_AC=1;East_Han_AF=
chr4	162436403	.	A	<INS:ME:LINE1>	.	PASS	SVTYPE=INS:ME:LINE1;END=162436434;SVLEN=2560;AN=20618;AC=1;AF=4.850130953535746e-05;N_HET=1;N_HOMALT=0;N_HOMREF=10308;FREQ_HET=9.700261907071492e-05;FREQ_HOMALT=0.0;FREQ_HOMREF=0.9999029973809292;N_BI_GENOS=10309;East_Han_AN=6554;East_Han_AC=1;East_Han_AF
chr4	162688009	.	A	<INS:ME:LINE1>	.	PASS	SVTYPE=INS:ME:LINE1;END=162688024;SVLEN=5998;AN=20618;AC=1;AF=4.850130953535746e-05;N_HET=1;N_HOMALT=0;N_HOMREF=10308;FREQ_HET=9.700261907071492e-05;FREQ_HOMALT=0.0;FREQ_HOMREF=0.9999029973809292;N_BI_GENOS=10309;Hui_AN=444;Hui_AC=1;Hui_AF=0.0022522522522
chr4	162715167	.	C	<INS:ME:LINE1>	.	PASS	SVTYPE=INS:ME:LINE1;END=162715167;SVLEN=825;AN=20618;AC=1;AF=4.850130953535746e-05;N_HET=1;N_HOMALT=0;N_HOMREF=10308;FREQ_HET=9.700261907071492e-05;FREQ_HOMALT=0.0;FREQ_HOMREF=0.9999029973809292;N_BI_GENOS=10309;East_Han_AN=6554;East_Han_AC=1;East_Han_AF=
chr4	162771250	.	G	<INS:ME:LINE1>	.	PASS	SVTYPE=INS:ME:LINE1;END=162771262;SVLEN=1313;AN=20618;AC=42;AF=0.002037055000485013;N_HET=42;N_HOMALT=0;N_HOMREF=10267;FREQ_HET=0.004074110000970026;FREQ_HOMALT=0.0;FREQ_HOMREF=0.995925889999903;N_BI_GENOS=10309;South_Han_AN=1496;South_Han_AC=5;South_Han_A
chr4	162779059	.	T	<INS:ME:LINE1>	.	PASS	SVTYPE=INS:ME:LINE1;END=162779070;SVLEN=814;AN=20618;AC=1;AF=4.850130953535746e-05;N_HET=1;N_HOMALT=0;N_HOMREF=10308;FREQ_HET=9.700261907071492e-05;FREQ_HOMALT=0.0;FREQ_HOMREF=0.9999029973809292;N_BI_GENOS=10309;North_Han_AN=2272;North_Han_AC=1;North_Han_
chr4	162791033	.	A	<INS:ME:LINE1>	.	PASS	SVTYPE=INS:ME:LINE1;END=162791047;SVLEN=589;AN=20618;AC=1;AF=4.850130953535746e-05;N_HET=1;N_HOMALT=0;N_HOMREF=10308;FREQ_HET=9.700261907071492e-05;FREQ_HOMALT=0.0;FREQ_HOMREF=0.9999029973809292;N_BI_GENOS=10309;North_Han_AN=2272;North_Han_AC=1;North_Han_
chr4	163751901	.	A	<INS:ME:LINE1>	.	PASS	SVTYPE=INS:ME:LINE1;END=163751917;SVLEN=1722;AN=20618;AC=65;AF=0.003152585119798 2345;N_HET=53;N_HOMALT=6;N_HOMREF=10250;FREQ_HET=0.00514113881074789;FREQ_HOMALT=0.0005820157144242894;FREQ_HOMREF=0.9942768454748279;N_BI_GENOS=10309;South_Han_AN=1496;South_
chr4	163774416	.	C	<INS:ME:LINE1>	.	PASS	SVTYPE=INS:ME:LINE1;END=163774431;SVLEN=1022;AN=20618;AC=1;AF=4.850130953535746e-05;N_HET=1;N_HOMALT=0;N_HOMREF=10308;FREQ_HET=9.700261907071492e-05;FREQ_HOMALT=0.0;FREQ_HOMREF=0.9999029973809292;N_BI_GENOS=10309;East_Han_AN=6554;East_Han_AC=1;East_Han_AF
chr4	163794369	.	C	<INS:ME:LINE1>	.	PASS	SVTYPE=INS:ME:LINE1;END=163794370;SVLEN=482;AN=20618;AC=1;AF=4.850130953535746e-05;N_HET=1;N_HOMALT=0;N_HOMREF=10308;FREQ_HET=9.700261907071492e-05;FREQ_HOMALT=0.0;FREQ_HOMREF=0.9999029973809292;N_BI_GENOS=10309;Lingnan_Han_AN=1888;Lingnan_Han_AC=1;Lingna
chr4	163869007	.	T	<INS:ME:LINE1>	.	PASS	SVTYPE=INS:ME:LINE1;END=163869016;SVLEN=751;AN=20618;AC=433;AF=0.0210010670288 0978;N_HET=373;N_HOMALT=30;N_HOMREF=9906;FREQ_HET=0.036181976913376664;FREQ_HOMALT=0.0029100785721214474;FREQ_HOMREF=0.9609079445145019;N_BI_GENOS=10309;South_Han_AN=1496;South_
chr4	164027473	.	C	<INS:ME:LINE1>	.	PASS	SVTYPE=INS:ME:LINE1;END=164027473;SVLEN=691;AN=20618;AC=1;AF=4.850130953535746e-05;N_HET=1;N_HOMALT=0;N_HOMREF=10308;FREQ_HET=9.700261907071492e-05;FREQ_HOMALT=0.0;FREQ_HOMREF=0.9999029973809292;N_BI_GENOS=10309;East_Han_AN=6554;East_Han_AC=1;East_Han_AF=
chr4	164095757	.	A	<INS:ME:LINE1>	.	PASS	SVTYPE=INS:ME:LINE1;END=164095757;SVLEN=613;AN=20618;AC=1;AF=4.850130953535746e-05;N_HET=1;N_HOMALT=0;N_HOMREF=10308;FREQ_HET=9.700261907071492e-05;FREQ_HOMALT=0.0;FREQ_HOMREF=0.9999029973809292;N_BI_GENOS=10309;East_Han_AN=6554;East_Han_AC=1;East_Han_AF=
chr4	164180067	.	G	<INS:ME:LINE1>	.	PASS	SVTYPE=INS:ME:LINE1;END=164180078;SVLEN=552;AN=20618;AC=1;AF=4.850130953535746e-05;N_HET=1;N_HOMALT=0;N_HOMREF=10308;FREQ_HET=9.700261907071492e-05;FREQ_HOMALT=0.0;FREQ_HOMREF=0.9999029973809292;N_BI_GENOS=10309;Central_Han_AN=2128;Central_Han_AC=1;Centra
chr4	164310577	.	G	<INS:ME:LINE1>	.	PASS	SVTYPE=INS:ME:LINE1;END=164310577;SVLEN=300;AN=20618;AC=6255;AF=0.30337569114366086;N_HET=4047;N_HOMALT=1104;N_HOMREF=5158;FREQ_HET=0.39256959937918323;FREQ_HOMALT=0.10709089145406926;FREQ_HOMREF=0.5003395091667475;N_BI_GENOS=10309;South_Han_AN=1496;South
chr4	164404669	.	A	<INS:ME:LINE1>	.	PASS	SVTYPE=INS:ME:LINE1;END=164404677;SVLEN=1157;AN=20618;AC=1;AF=4.850130953535746e-05;N_HET=1;N_HOMALT=0;N_HOMREF=10308;FREQ_HET=9.700261907071492e-05;FREQ_HOMALT=0.0;FREQ_HOMREF=0.9999029973809292;N_BI_GENOS=10309;East_Han_AN=6554;East_Han_AC=1;East_Han_AF
chr4	164537882	.	A	<INS:ME:LINE1>	.	PASS	SVTYPE=INS:ME:LINE1;END=164537896;SVLEN=6000;AN=20618;AC=1;AF=4.850130953535746e-05;N_HET=1;N_HOMALT=0;N_HOMREF=10308;FREQ_HET=9.700261907071492e-05;FREQ_HOMALT=0.0;FREQ_HOMREF=0.9999029973809292;N_BI_GENOS=10309;Lingnan_Han_AN=1888;Lingnan_Han_AC=1;Lingn
chr4	164610876	.	A	<INS:ME:LINE1>	.	PASS	SVTYPE=INS:ME:LINE1;END=164610876;SVLEN=0;AN=20618;AC=90;AF=0.004365117858182171;N_HET=20;N_HOMALT=35;N_HOMREF=10254;FREQ_HET=0.0019400523814142982;FREQ_HOMALT=0.0033950916674750217;FREQ_HOMREF=0.9946648559511106;N_BI_GENOS=10309;South_Han_AN=1496;South_H
chr4	164620106	.	A	<INS:ME:LINE1>	.	PASS	SVTYPE=INS:ME:LINE1;END=164620118;SVLEN=6031;AN=20618;AC=2;AF=9.700261907071492e-05;N_HET=2;N_HOMALT=0;N_HOMREF=10307;FREQ_HET=0.00019400523814142983;FREQ_HOMALT=0.0;FREQ_HOMREF=0.9998059947618586;N_BI_GENOS=10309;East_Han_AN=6554;East_Han_AC=1;East_Han_A
chr4	165161235	.	A	<INS:ME:LINE1>	.	PASS	SVTYPE=INS:ME:LINE1;END=165161250;SVLEN=831;AN=20618;AC=9;AF=0.0004365117858182171;N_HET=9;N_HOMALT=0;N_HOMREF=10300;FREQ_HET=0.0008730235716364342;FREQ_HOMALT=0.0;FREQ_HOMREF=0.9991269764283636;N_BI_GENOS=10309;Lingnan_Han_AN=1888;Lingnan_Han_AC=4;Lingna
chr4	165701966	.	A	<INS:ME:LINE1>	.	PASS	SVTYPE=INS:ME:LINE1;END=165701981;SVLEN=2028;AN=20618;AC=1;AF=4.850130953535746e-05;N_HET=1;N_HOMALT=0;N_HOMREF=10308;FREQ_HET=9.700261907071492e-05;FREQ_HOMALT=0.0;FREQ_HOMREF=0.9999029973809292;N_BI_GENOS=10309;Southeast_Han_AN=1908;Southeast_Han_AC=1;S
chr4	165765402	.	T	<INS:ME:LINE1>	.	PASS	SVTYPE=INS:ME:LINE1;END=165765419;SVLEN=509;AN=20618;AC=1;AF=4.850130953535746e-05;N_HET=1;N_HOMALT=0;N_HOMREF=10308;FREQ_HET=9.700261907071492e-05;FREQ_HOMALT=0.0;FREQ_HOMREF=0.9999029973809292;N_BI_GENOS=10309;North_Han_AN=2272;North_Han_AC=1;North_Han_
chr4	166439754	.	A	<INS:ME:LINE1>	.	PASS	SVTYPE=INS:ME:LINE1;END=166439754;SVLEN=239;AN=20618;AC=1;AF=4.850130953535746e-05;N_HET=1;N_HOMALT=0;N_HOMREF=10308;FREQ_HET=9.700261907071492e-05;FREQ_HOMALT=0.0;FREQ_HOMREF=0.9999029973809292;N_BI_GENOS=10309;East_Han_AN=6554;East_Han_AC=1;East_Han_AF=
chr4	166525395	.	G	<INS:ME:LINE1>	.	PASS	SVTYPE=INS:ME:LINE1;END=166525395;SVLEN=0;AN=20618;AC=9;AF=0.0004365117858182171;N_HET=3;N_HOMALT=3;N_HOMREF=10303;FREQ_HET=0.0002910078572121447;FREQ_HOMALT=0.0002910078572121447;FREQ_HOMREF=0.9994179842855757;N_BI_GENOS=10309;Northwest_Han_AN=1296;North
chr4	166553788	.	A	<INS:ME:LINE1>	.	PASS	SVTYPE=INS:ME:LINE1;END=166553798;SVLEN=5999;AN=20618;AC=1;AF=4.850130953535746e-05;N_HET=1;N_HOMALT=0;N_HOMREF=10308;FREQ_HET=9.700261907071492e-05;FREQ_HOMALT=0.0;FREQ_HOMREF=0.9999029973809292;N_BI_GENOS=10309;Northwest_Han_AN=1296;Northwest_Han_AC=1;N
chr4	166717044	.	T	<INS:ME:LINE1>	.	PASS	SVTYPE=INS:ME:LINE1;END=166717044;SVLEN=0;AN=20618;AC=1;AF=4.850130953535746e-05;N_HET=1;N_HOMALT=0;N_HOMREF=10308;FREQ_HET=9.700261907071492e-05;FREQ_HOMALT=0.0;FREQ_HOMREF=0.9999029973809292;N_BI_GENOS=10309;East_Han_AN=6554;East_Han_AC=1;East_Han_AF=0.
chr4	166870496	.	T	<INS:ME:LINE1>	.	PASS	SVTYPE=INS:ME:LINE1;END=166870512;SVLEN=5201;AN=20618;AC=1;AF=4.850130953535746e-05;N_HET=1;N_HOMALT=0;N_HOMREF=10308;FREQ_HET=9.700261907071492e-05;FREQ_HOMALT=0.0;FREQ_HOMREF=0.9999029973809292;N_BI_GENOS=10309;East_Han_AN=6554;East_Han_AC=1;East_Han_AF
chr4	166926473	.	G	<INS:ME:LINE1>	.	PASS	SVTYPE=INS:ME:LINE1;END=166926491;SVLEN=623;AN=20618;AC=1;AF=4.850130953535746e-05;N_HET=1;N_HOMALT=0;N_HOMREF=10308;FREQ_HET=9.700261907071492e-05;FREQ_HOMALT=0.0;FREQ_HOMREF=0.9999029973809292;N_BI_GENOS=10309;East_Han_AN=6554;East_Han_AC=1;East_Han_AF=
chr4	167029920	.	T	<INS:ME:LINE1>	.	PASS	SVTYPE=INS:ME:LINE1;END=167029920;SVLEN=2105;AN=20618;AC=2;AF=9.700261907071492e-05;N_HET=2;N_HOMALT=0;N_HOMREF=10307;FREQ_HET=0.00019400523814142983;FREQ_HOMALT=0.0;FREQ_HOMREF=0.9998059947618586;N_BI_GENOS=10309;Lingnan_Han_AN=1888;Lingnan_Han_AC=1;Ling
chr4	167474718	.	T	<INS:ME:LINE1>	.	PASS	SVTYPE=INS:ME:LINE1;END=167474734;SVLEN=725;AN=20618;AC=4;AF=0.00019400523814142983;N_HET=4;N_HOMALT=0;N_HOMREF=10305;FREQ_HET=0.00038801047628285966;FREQ_HOMALT=0.0;FREQ_HOMREF=0.9996119895237171;N_BI_GENOS=10309;Lingnan_Han_AN=1888;Lingnan_Han_AC=1;Ling
chr4	167617914	.	T	<INS:ME:LINE1>	.	PASS	SVTYPE=INS:ME:LINE1;END=167617914;SVLEN=0;AN=20618;AC=1;AF=4.850130953535746e-05;N_HET=1;N_HOMALT=0;N_HOMREF=10308;FREQ_HET=9.700261907071492e-05;FREQ_HOMALT=0.0;FREQ_HOMREF=0.9999029973809292;N_BI_GENOS=10309;North_Han_AN=2272;North_Han_AC=1;North_Han_AF
chr4	167767896	.	T	<INS:ME:LINE1>	.	PASS	SVTYPE=INS:ME:LINE1;END=167767910;SVLEN=5398;AN=20618;AC=4;AF=0.00019400523814142983;N_HET=4;N_HOMALT=0;N_HOMREF=10305;FREQ_HET=0.00038801047628285966;FREQ_HOMALT=0.0;FREQ_HOMREF=0.9996119895237171;N_BI_GENOS=10309;South_Han_AN=1496;South_Han_AC=1;South_H
chr4	167775314	.	G	<INS:ME:LINE1>	.	PASS	SVTYPE=INS:ME:LINE1;END=167775327;SVLEN=2836;AN=20618;AC=1;AF=4.850130953535746e-05;N_HET=1;N_HOMALT=0;N_HOMREF=10308;FREQ_HET=9.700261907071492e-05;FREQ_HOMALT=0.0;FREQ_HOMREF=0.9999029973809292;N_BI_GENOS=10309;South_Han_AN=1496;South_Han_AC=1;South_Han
chr4	168028760	.	A	<INS:ME:LINE1>	.	PASS	SVTYPE=INS:ME:LINE1;END=168028775;SVLEN=2718;AN=20618;AC=2;AF=9.700261907071492e-05;N_HET=2;N_HOMALT=0;N_HOMREF=10307;FREQ_HET=0.00019400523814142983;FREQ_HOMALT=0.0;FREQ_HOMREF=0.9998059947618586;N_BI_GENOS=10309;East_Han_AN=6554;East_Han_AC=2;East_Han_A
chr4	168070406	.	T	<INS:ME:LINE1>	.	PASS	SVTYPE=INS:ME:LINE1;END=168070420;SVLEN=366;AN=20618;AC=1;AF=4.850130953535746e-05;N_HET=1;N_HOMALT=0;N_HOMREF=10308;FREQ_HET=9.700261907071492e-05;FREQ_HOMALT=0.0;FREQ_HOMREF=0.9999029973809292;N_BI_GENOS=10309;East_Han_AN=6554;East_Han_AC=1;East_Han_AF=
chr4	168080930	.	T	<INS:ME:LINE1>	.	PASS	SVTYPE=INS:ME:LINE1;END=168080938;SVLEN=1625;AN=20618;AC=6;AF=0.0002910078572121447;N_HET=6;N_HOMALT=0;N_HOMREF=10303;FREQ_HET=0.0005820157144242894;FREQ_HOMALT=0.0;FREQ_HOMREF=0.9994179842855757;N_BI_GENOS=10309;South_Han_AN=1496;South_Han_AC=1;South_Han
chr4	168491947	.	T	<INS:ME:LINE1>	.	PASS	SVTYPE=INS:ME:LINE1;END=168491960;SVLEN=5997;AN=20618;AC=3;AF=0.00014550392860607236;N_HET=3;N_HOMALT=0;N_HOMREF=10306;FREQ_HET=0.0002910078572121447;FREQ_HOMALT=0.0;FREQ_HOMREF=0.9997089921427879;N_BI_GENOS=10309;North_Han_AN=2272;North_Han_AC=1;North_Ha
chr4	168586024	.	C	<INS:ME:LINE1>	.	PASS	SVTYPE=INS:ME:LINE1;END=168586065;SVLEN=5999;AN=20618;AC=1;AF=4.850130953535746e-05;N_HET=1;N_HOMALT=0;N_HOMREF=10308;FREQ_HET=9.700261907071492e-05;FREQ_HOMALT=0.0;FREQ_HOMREF=0.9999029973809292;N_BI_GENOS=10309;Northwest_Han_AN=1296;Northwest_Han_AC=1;N
chr4	168647299	.	A	<INS:ME:LINE1>	.	PASS	SVTYPE=INS:ME:LINE1;END=168647312;SVLEN=6033;AN=20618;AC=1;AF=4.850130953535746e-05;N_HET=1;N_HOMALT=0;N_HOMREF=10308;FREQ_HET=9.700261907071492e-05;FREQ_HOMALT=0.0;FREQ_HOMREF=0.9999029973809292;N_BI_GENOS=10309;North_Han_AN=2272;North_Han_AC=1;North_Han
chr4	168705093	.	C	<INS:ME:LINE1>	.	PASS	SVTYPE=INS:ME:LINE1;END=168705108;SVLEN=376;AN=20618;AC=5;AF=0.00024250654767678728;N_HET=5;N_HOMALT=0;N_HOMREF=10304;FREQ_HET=0.00048501309535357455;FREQ_HOMALT=0.0;FREQ_HOMREF=0.9995149869046465;N_BI_GENOS=10309;South_Han_AN=1496;South_Han_AC=1;South_Ha
chr4	168800401	.	A	<INS:ME:LINE1>	.	PASS	SVTYPE=INS:ME:LINE1;END=168800415;SVLEN=290;AN=20618;AC=2;AF=9.700261907071492e-05;N_HET=2;N_HOMALT=0;N_HOMREF=10307;FREQ_HET=0.00019400523814142983;FREQ_HOMALT=0.0;FREQ_HOMREF=0.9998059947618586;N_BI_GENOS=10309;North_Han_AN=2272;North_Han_AC=2;North_Han
chr4	169186119	.	A	<INS:ME:LINE1>	.	PASS	SVTYPE=INS:ME:LINE1;END=169186138;SVLEN=951;AN=20618;AC=6;AF=0.0002910078572121447;N_HET=6;N_HOMALT=0;N_HOMREF=10303;FREQ_HET=0.0005820157144242894;FREQ_HOMALT=0.0;FREQ_HOMREF=0.9994179842855757;N_BI_GENOS=10309;North_Han_AN=2272;North_Han_AC=1;North_Han_
chr4	169364638	.	A	<INS:ME:LINE1>	.	PASS	SVTYPE=INS:ME:LINE1;END=169364653;SVLEN=6000;AN=20618;AC=2;AF=9.700261907071492e-05;N_HET=0;N_HOMALT=1;N_HOMREF=10308;FREQ_HET=0.0;FREQ_HOMALT=9.700261907071492e-05;FREQ_HOMREF=0.9999029973809292;N_BI_GENOS=10309;Yi_AN=422;Yi_AC=2;Yi_AF=0.0047393364928909
chr4	169387173	.	A	<INS:ME:LINE1>	.	PASS	SVTYPE=INS:ME:LINE1;END=169387173;SVLEN=14;AN=20618;AC=1;AF=4.850130953535746e-05;N_HET=1;N_HOMALT=0;N_HOMREF=10308;FREQ_HET=9.700261907071492e-05;FREQ_HOMALT=0.0;FREQ_HOMREF=0.9999029973809292;N_BI_GENOS=10309;Southeast_Han_AN=1908;Southeast_Han_AC=1;Sou
chr4	169466664	.	A	<INS:ME:LINE1>	.	PASS	SVTYPE=INS:ME:LINE1;END=169466678;SVLEN=721;AN=20618;AC=1;AF=4.850130953535746e-05;N_HET=1;N_HOMALT=0;N_HOMREF=10308;FREQ_HET=9.700261907071492e-05;FREQ_HOMALT=0.0;FREQ_HOMREF=0.9999029973809292;N_BI_GENOS=10309;East_Han_AN=6554;East_Han_AC=1;East_Han_AF=
chr4	169801913	.	A	<INS:ME:LINE1>	.	PASS	SVTYPE=INS:ME:LINE1;END=169801920;SVLEN=931;AN=20618;AC=1;AF=4.850130953535746e-05;N_HET=1;N_HOMALT=0;N_HOMREF=10308;FREQ_HET=9.700261907071492e-05;FREQ_HOMALT=0.0;FREQ_HOMREF=0.9999029973809292;N_BI_GENOS=10309;East_Han_AN=6554;East_Han_AC=1;East_Han_AF=
chr4	169840342	.	A	<INS:ME:LINE1>	.	PASS	SVTYPE=INS:ME:LINE1;END=169840350;SVLEN=2360;AN=20618;AC=27;AF=0.0013095353574546513;N_HET=27;N_HOMALT=0;N_HOMREF=10282;FREQ_HET=0.0026190707149093026;FREQ_HOMALT=0.0;FREQ_HOMREF=0.9973809292850907;N_BI_GENOS=10309;South_Han_AN=1496;South_Han_AC=1;South_H
chr4	170149903	.	A	<INS:ME:LINE1>	.	PASS	SVTYPE=INS:ME:LINE1;END=170149903;SVLEN=2238;AN=20618;AC=1;AF=4.850130953535746e-05;N_HET=1;N_HOMALT=0;N_HOMREF=10308;FREQ_HET=9.700261907071492e-05;FREQ_HOMALT=0.0;FREQ_HOMREF=0.9999029973809292;N_BI_GENOS=10309;East_Han_AN=6554;East_Han_AC=1;East_Han_AF
chr4	170149980	.	G	<INS:ME:LINE1>	.	PASS	SVTYPE=INS:ME:LINE1;END=170150000;SVLEN=2254;AN=20618;AC=1;AF=4.850130953535746e-05;N_HET=1;N_HOMALT=0;N_HOMREF=10308;FREQ_HET=9.700261907071492e-05;FREQ_HOMALT=0.0;FREQ_HOMREF=0.9999029973809292;N_BI_GENOS=10309;East_Han_AN=6554;East_Han_AC=1;East_Han_AF
chr4	170204938	.	C	<INS:ME:LINE1>	.	PASS	SVTYPE=INS:ME:LINE1;END=170204952;SVLEN=1585;AN=20618;AC=1;AF=4.850130953535746e-05;N_HET=1;N_HOMALT=0;N_HOMREF=10308;FREQ_HET=9.700261907071492e-05;FREQ_HOMALT=0.0;FREQ_HOMREF=0.9999029973809292;N_BI_GENOS=10309;Tibetan_AN=414;Tibetan_AC=1;Tibetan_AF=0.0

chr4	170336208 .	A	<INS:ME:LINE1>	.	PASS	SVTYPE=INS:ME:LINE1;END=170336210;SVLEN=822;AN=20618;AC=1;AF=4.850130953535746e-05;N_HET=1;N_HOMALT=0;N_HOMREF=10308;FREQ_HET=9.700261907071492e-05;FREQ_HOMALT=0.0;FREQ_HOMREF=0.9999029973809292;N_BI_GENOS=10309;East_Han_AN=6554;East_Han_AC=1;East_Han_AF=
chr4	170344820 .	G	<INS:ME:LINE1>	.	PASS	SVTYPE=INS:ME:LINE1;END=170344830;SVLEN=176;AN=20618;AC=1;AF=4.850130953535746e-05;N_HET=1;N_HOMALT=0;N_HOMREF=10308;FREQ_HET=9.700261907071492e-05;FREQ_HOMALT=0.0;FREQ_HOMREF=0.9999029973809292;N_BI_GENOS=10309;Central_Han_AN=2128;Central_Han_AC=1;Centra
chr4	170448005 .	G	<INS:ME:LINE1>	.	PASS	SVTYPE=INS:ME:LINE1;END=170448019;SVLEN=5883;AN=20618;AC=2;AF=9.700261907071492e-05;N_HET=2;N_HOMALT=0;N_HOMREF=10307;FREQ_HET=0.00019400523814142983;FREQ_HOMALT=0.0;FREQ_HOMREF=0.9998059947618586;N_BI_GENOS=10309;North_Han_AN=2272;North_Han_AC=1;North_Ha
chr4	170682664 .	A	<INS:ME:LINE1>	.	PASS	SVTYPE=INS:ME:LINE1;END=170682680;SVLEN=690;AN=20618;AC=1;AF=4.850130953535746e-05;N_HET=1;N_HOMALT=0;N_HOMREF=10308;FREQ_HET=9.700261907071492e-05;FREQ_HOMALT=0.0;FREQ_HOMREF=0.9999029973809292;N_BI_GENOS=10309;Southeast_Han_AN=1908;Southeast_Han_AC=1;So
chr4	170698184 .	A	<INS:ME:LINE1>	.	PASS	SVTYPE=INS:ME:LINE1;END=170698201;SVLEN=3537;AN=20618;AC=1;AF=4.850130953535746e-05;N_HET=1;N_HOMALT=0;N_HOMREF=10308;FREQ_HET=9.700261907071492e-05;FREQ_HOMALT=0.0;FREQ_HOMREF=0.9999029973809292;N_BI_GENOS=10309;East_Han_AN=6554;East_Han_AC=1;East_Han_AF
chr4	170769001 .	A	<INS:ME:LINE1>	.	PASS	SVTYPE=INS:ME:LINE1;END=170769011;SVLEN=697;AN=20618;AC=1;AF=4.850130953535746e-05;N_HET=1;N_HOMALT=0;N_HOMREF=10308;FREQ_HET=9.700261907071492e-05;FREQ_HOMALT=0.0;FREQ_HOMREF=0.9999029973809292;N_BI_GENOS=10309;East_Han_AN=6554;East_Han_AC=1;East_Han_AF=
chr4	171178261 .	A	<INS:ME:LINE1>	.	PASS	SVTYPE=INS:ME:LINE1;END=171178280;SVLEN=1171;AN=20618;AC=1;AF=4.850130953535746e-05;N_HET=1;N_HOMALT=0;N_HOMREF=10308;FREQ_HET=9.700261907071492e-05;FREQ_HOMALT=0.0;FREQ_HOMREF=0.9999029973809292;N_BI_GENOS=10309;Zhuang_AN=444;Zhuang_AC=1;Zhuang_AF=0.0022
chr4	171213320 .	A	<INS:ME:LINE1>	.	PASS	SVTYPE=INS:ME:LINE1;END=171213337;SVLEN=1087;AN=20618;AC=1;AF=4.850130953535746e-05;N_HET=1;N_HOMALT=0;N_HOMREF=10308;FREQ_HET=9.700261907071492e-05;FREQ_HOMALT=0.0;FREQ_HOMREF=0.9999029973809292;N_BI_GENOS=10309;Zhuang_AN=444;Zhuang_AC=1;Zhuang_AF=0.0022
chr4	171355080 .	G	<INS:ME:LINE1>	.	PASS	SVTYPE=INS:ME:LINE1;END=171355099;SVLEN=1235;AN=20618;AC=1;AF=4.850130953535746e-05;N_HET=1;N_HOMALT=0;N_HOMREF=10308;FREQ_HET=9.700261907071492e-05;FREQ_HOMALT=0.0;FREQ_HOMREF=0.9999029973809292;N_BI_GENOS=10309;East_Han_AN=6554;East_Han_AC=1;East_Han_AF
chr4	171355779 .	T	<INS:ME:LINE1>	.	PASS	SVTYPE=INS:ME:LINE1;END=171355797;SVLEN=2530;AN=20618;AC=2;AF=9.700261907071492e-05;N_HET=2;N_HOMALT=0;N_HOMREF=10307;FREQ_HET=0.00019400523814142983;FREQ_HOMALT=0.0;FREQ_HOMREF=0.9998059947618586;N_BI_GENOS=10309;East_Han_AN=6554;East_Han_AC=2;East_Han_A
chr4	171840147 .	A	<INS:ME:LINE1>	.	PASS	SVTYPE=INS:ME:LINE1;END=171840151;SVLEN=397;AN=20618;AC=4;AF=0.00019400523814142983;N_HET=4;N_HOMALT=0;N_HOMREF=10305;FREQ_HET=0.00038801047628285966;FREQ_HOMALT=0.0;FREQ_HOMREF=0.9996119895237171;N_BI_GENOS=10309;South_Han_AN=1496;South_Han_AC=4;South_Ha
chr4	171864641 .	T	<INS:ME:LINE1>	.	PASS	SVTYPE=INS:ME:LINE1;END=171864657;SVLEN=1121;AN=20618;AC=7;AF=0.0003395091667475022;N_HET=7;N_HOMALT=0;N_HOMREF=10302;FREQ_HET=0.0006790183334950044;FREQ_HOMALT=0.0;FREQ_HOMREF=0.999320981666505;N_BI_GENOS=10309;Northwest_Han_AN=1296;Northwest_Han_AC=2;No
chr4	171937527 .	T	<INS:ME:LINE1>	.	PASS	SVTYPE=INS:ME:LINE1;END=171937530;SVLEN=1358;AN=20618;AC=2;AF=9.700261907071492e-05;N_HET=2;N_HOMALT=0;N_HOMREF=10307;FREQ_HET=0.00019400523814142983;FREQ_HOMALT=0.0;FREQ_HOMREF=0.9998059947618586;N_BI_GENOS=10309;Central_Han_AN=2128;Central_Han_AC=1;Cent
chr4	172615935 .	T	<INS:ME:LINE1>	.	PASS	SVTYPE=INS:ME:LINE1;END=172615946;SVLEN=6000;AN=20618;AC=86;AF=0.004171111262004741;N_HET=86;N_HOMALT=0;N_HOMREF=10223;FREQ_HET=0.008342225240081482;FREQ_HOMALT=0.0;FREQ_HOMREF=0.9916577747599186;N_BI_GENOS=10309;South_Han_AN=1496;South_Han_AC=6;South_Han
chr4	173114443 .	A	<INS:ME:LINE1>	.	PASS	SVTYPE=INS:ME:LINE1;END=173114460;SVLEN=3149;AN=20618;AC=18;AF=0.0008730235716364342;N_HET=18;N_HOMALT=0;N_HOMREF=10291;FREQ_HET=0.0017460471432728683;FREQ_HOMALT=0.0;FREQ_HOMREF=0.9982539528567271;N_BI_GENOS=10309;South_Han_AN=1496;South_Han_AC=1;South_H
chr4	173758422 .	A	<INS:ME:LINE1>	.	PASS	SVTYPE=INS:ME:LINE1;END=173758440;SVLEN=415;AN=20618;AC=3;AF=0.00014550392860607236;N_HET=3;N_HOMALT=0;N_HOMREF=10306;FREQ_HET=0.0002910078572121447;FREQ_HOMALT=0.0;FREQ_HOMREF=0.9997089921427879;N_BI_GENOS=10309;Miao_AN=482;Miao_AC=3;Miao_AF=0.0062240663
chr4	173770518 .	C	<INS:ME:LINE1>	.	PASS	SVTYPE=INS:ME:LINE1;END=173770535;SVLEN=565;AN=20618;AC=9;AF=0.0004365117858182171;N_HET=9;N_HOMALT=0;N_HOMREF=10300;FREQ_HET=0.0008730235716364342;FREQ_HOMALT=0.0;FREQ_HOMREF=0.9991269764283636;N_BI_GENOS=10309;South_Han_AN=1496;South_Han_AC=1;South_Han_
chr4	174069579 .	A	<INS:ME:LINE1>	.	PASS	SVTYPE=INS:ME:LINE1;END=174069579;SVLEN=0;AN=20618;AC=1;AF=4.850130953535746e-05;N_HET=1;N_HOMALT=0;N_HOMREF=10308;FREQ_HET=9.700261907071492e-05;FREQ_HOMALT=0.0;FREQ_HOMREF=0.9999029973809292;N_BI_GENOS=10309;Lingnan_Han_AN=1888;Lingnan_Han_AC=1;Lingnan_
chr4	174199648 .	C	<INS:ME:LINE1>	.	PASS	SVTYPE=INS:ME:LINE1;END=174199660;SVLEN=6000;AN=20618;AC=3;AF=0.00014550392860607236;N_HET=3;N_HOMALT=0;N_HOMREF=10306;FREQ_HET=0.0002910078572121447;FREQ_HOMALT=0.0;FREQ_HOMREF=0.9997089921427879;N_BI_GENOS=10309;Central_Han_AN=2128;Central_Han_AC=1;Cent
chr4	174382771 .	G	<INS:ME:LINE1>	.	PASS	SVTYPE=INS:ME:LINE1;END=174382773;SVLEN=565;AN=20618;AC=3;AF=0.00014550392860607236;N_HET=3;N_HOMALT=0;N_HOMREF=10306;FREQ_HET=0.0002910078572121447;FREQ_HOMALT=0.0;FREQ_HOMREF=0.9997089921427879;N_BI_GENOS=10309;Northwest_Han_AN=1296;Northwest_Han_AC=1;N
chr4	174737232 .	T	<INS:ME:LINE1>	.	PASS	SVTYPE=INS:ME:LINE1;END=174737244;SVLEN=1445;AN=20618;AC=1;AF=4.850130953535746e-05;N_HET=1;N_HOMALT=0;N_HOMREF=10308;FREQ_HET=9.700261907071492e-05;FREQ_HOMALT=0.0;FREQ_HOMREF=0.9999029973809292;N_BI_GENOS=10309;Southeast_Han_AN=1908;Southeast_Han_AC=1;S
chr4	174810370 .	C	<INS:ME:LINE1>	.	PASS	SVTYPE=INS:ME:LINE1;END=174810387;SVLEN=6004;AN=20618;AC=1;AF=4.850130953535746e-05;N_HET=1;N_HOMALT=0;N_HOMREF=10308;FREQ_HET=9.700261907071492e-05;FREQ_HOMALT=0.0;FREQ_HOMREF=0.9999029973809292;N_BI_GENOS=10309;East_Han_AN=6554;East_Han_AC=1;East_Han_AF
chr4	175354932 .	A	<INS:ME:LINE1>	.	PASS	SVTYPE=INS:ME:LINE1;END=175354932;SVLEN=1295;AN=20618;AC=1;AF=4.850130953535746e-05;N_HET=1;N_HOMALT=0;N_HOMREF=10308;FREQ_HET=9.700261907071492e-05;FREQ_HOMALT=0.0;FREQ_HOMREF=0.9999029973809292;N_BI_GENOS=10309;East_Han_AN=6554;East_Han_AC=1;East_Han_AF
chr4	175406490 .	G	<INS:ME:LINE1>	.	PASS	SVTYPE=INS:ME:LINE1;END=175406490;SVLEN=3431;AN=20618;AC=1;AF=4.850130953535746e-05;N_HET=1;N_HOMALT=0;N_HOMREF=10308;FREQ_HET=9.700261907071492e-05;FREQ_HOMALT=0.0;FREQ_HOMREF=0.9999029973809292;N_BI_GENOS=10309;North_Han_AN=2272;North_Han_AC=1;North_Han
chr4	175523331 .	A	<INS:ME:LINE1>	.	PASS	SVTYPE=INS:ME:LINE1;END=175523347;SVLEN=5998;AN=20618;AC=9;AF=0.0004365117858182171;N_HET=9;N_HOMALT=0;N_HOMREF=10300;FREQ_HET=0.0008730235716364342;FREQ_HOMALT=0.0;FREQ_HOMREF=0.9991269764283636;N_BI_GENOS=10309;South_Han_AN=1496;South_Han_AC=1;South_Han
chr4	175591260 .	G	<INS:ME:LINE1>	.	PASS	SVTYPE=INS:ME:LINE1;END=175591275;SVLEN=1974;AN=20618;AC=1;AF=4.850130953535746e-05;N_HET=1;N_HOMALT=0;N_HOMREF=10308;FREQ_HET=9.700261907071492e-05;FREQ_HOMALT=0.0;FREQ_HOMREF=0.9999029973809292;N_BI_GENOS=10309;Southeast_Han_AN=1908;Southeast_Han_AC=1;S
chr4	176046805 .	A	<INS:ME:LINE1>	.	PASS	SVTYPE=INS:ME:LINE1;END=176046818;SVLEN=6000;AN=20618;AC=1;AF=4.850130953535746e-05;N_HET=1;N_HOMALT=0;N_HOMREF=10308;FREQ_HET=9.700261907071492e-05;FREQ_HOMALT=0.0;FREQ_HOMREF=0.9999029973809292;N_BI_GENOS=10309;East_Han_AN=6554;East_Han_AC=1;East_Han_AF
chr4	176100924 .	T	<INS:ME:LINE1>	.	PASS	SVTYPE=INS:ME:LINE1;END=176100924;SVLEN=85;AN=20618;AC=1;AF=4.850130953535746e-05;N_HET=1;N_HOMALT=0;N_HOMREF=10308;FREQ_HET=9.700261907071492e-05;FREQ_HOMALT=0.0;FREQ_HOMREF=0.9999029973809292;N_BI_GENOS=10309;East_Han_AN=6554;East_Han_AC=1;East_Han_AF=0
chr4	176434056 .	A	<INS:ME:LINE1>	.	PASS	SVTYPE=INS:ME:LINE1;END=176434070;SVLEN=6010;AN=20618;AC=1;AF=4.850130953535746e-05;N_HET=1;N_HOMALT=0;N_HOMREF=10308;FREQ_HET=9.700261907071492e-05;FREQ_HOMALT=0.0;FREQ_HOMREF=0.9999029973809292;N_BI_GENOS=10309;Mongolian_AN=454;Mongolian_AC=1;Mongolian_
chr4	176456133 .	A	<INS:ME:LINE1>	.	PASS	SVTYPE=INS:ME:LINE1;END=176456133;SVLEN=271;AN=20618;AC=1;AF=4.850130953535746e-05;N_HET=1;N_HOMALT=0;N_HOMREF=10308;FREQ_HET=9.700261907071492e-05;FREQ_HOMALT=0.0;FREQ_HOMREF=0.9999029973809292;N_BI_GENOS=10309;East_Han_AN=6554;East_Han_AC=1;East_Han_AF=
chr4	176457193 .	A	<INS:ME:LINE1>	.	PASS	SVTYPE=INS:ME:LINE1;END=176457193;SVLEN=321;AN=20618;AC=2;AF=9.700261907071492e-05;N_HET=2;N_HOMALT=0;N_HOMREF=10307;FREQ_HET=0.00019400523814142983;FREQ_HOMALT=0.0;FREQ_HOMREF=0.9998059947618586;N_BI_GENOS=10309;East_Han_AN=6554;East_Han_AC=2;East_Han_AF
chr4	176775332 .	T	<INS:ME:LINE1>	.	PASS	SVTYPE=INS:ME:LINE1;END=176775337;SVLEN=336;AN=20618;AC=3;AF=0.00014550392860607236;N_HET=3;N_HOMALT=0;N_HOMREF=10306;FREQ_HET=0.0002910078572121447;FREQ_HOMALT=0.0;FREQ_HOMREF=0.9997089921427879;N_BI_GENOS=10309;East_Han_AN=6554;East_Han_AC=2;East_Han_AF
chr4	177229231 .	A	<INS:ME:LINE1>	.	PASS	SVTYPE=INS:ME:LINE1;END=177229247;SVLEN=3749;AN=20618;AC=1;AF=4.850130953535746e-05;N_HET=1;N_HOMALT=0;N_HOMREF=10308;FREQ_HET=9.700261907071492e-05;FREQ_HOMALT=0.0;FREQ_HOMREF=0.9999029973809292;N_BI_GENOS=10309;Northwest_Han_AN=1296;Northwest_Han_AC=1;N
chr4	177517187 .	A	<INS:ME:LINE1>	.	PASS	SVTYPE=INS:ME:LINE1;END=177517204;SVLEN=6006;AN=20618;AC=2;AF=9.700261907071492e-05;N_HET=2;N_HOMALT=0;N_HOMREF=10307;FREQ_HET=0.00019400523814142983;FREQ_HOMALT=0.0;FREQ_HOMREF=0.9998059947618586;N_BI_GENOS=10309;East_Han_AN=6554;East_Han_AC=2;East_Han_A
chr4	177596962 .	T	<INS:ME:LINE1>	.	PASS	SVTYPE=INS:ME:LINE1;END=177596962;SVLEN=0;AN=20618;AC=4;AF=0.00019400523814142983;N_HET=4;N_HOMALT=0;N_HOMREF=10305;FREQ_HET=0.00038801047628285966;FREQ_HOMALT=0.0;FREQ_HOMREF=0.9996119895237171;N_BI_GENOS=10309;East_Han_AN=6554;East_Han_AC=1;East_Han_AF=
chr4	177635015 .	A	<INS:ME:LINE1>	.	PASS	SVTYPE=INS:ME:LINE1;END=177635028;SVLEN=6155;AN=20618;AC=3;AF=0.00014550392860607236;N_HET=3;N_HOMALT=0;N_HOMREF=10306;FREQ_HET=0.0002910078572121447;FREQ_HOMALT=0.0;FREQ_HOMREF=0.9997089921427879;N_BI_GENOS=10309;South_Han_AN=1496;South_Han_AC=1;South_Ha
chr4	177768617 .	A	<INS:ME:LINE1>	.	PASS	SVTYPE=INS:ME:LINE1;END=177768635;SVLEN=5998;AN=20618;AC=2;AF=9.700261907071492e-05;N_HET=2;N_HOMALT=0;N_HOMREF=10307;FREQ_HET=0.00019400523814142983;FREQ_HOMALT=0.0;FREQ_HOMREF=0.9998059947618586;N_BI_GENOS=10309;Lingnan_Han_AN=1888;Lingnan_Han_AC=1;Ling
chr4	177787085 .	A	<INS:ME:LINE1>	.	PASS	SVTYPE=INS:ME:LINE1;END=177787086;SVLEN=1929;AN=20618;AC=4;AF=0.00019400523814142983;N_HET=4;N_HOMALT=0;N_HOMREF=10305;FREQ_HET=0.00038801047628285966;FREQ_HOMALT=0.0;FREQ_HOMREF=0.9996119895237171;N_BI_GENOS=10309;Northwest_Han_AN=1296;Northwest_Han_AC=1
chr4	177957782 .	A	<INS:ME:LINE1>	.	PASS	SVTYPE=INS:ME:LINE1;END=177957786;SVLEN=4310;AN=20618;AC=1;AF=4.850130953535746e-05;N_HET=1;N_HOMALT=0;N_HOMREF=10308;FREQ_HET=9.700261907071492e-05;FREQ_HOMALT=0.0;FREQ_HOMREF=0.9999029973809292;N_BI_GENOS=10309;East_Han_AN=6554;East_Han_AC=1;East_Han_AF
chr4	178294566 .	T	<INS:ME:LINE1>	.	PASS	SVTYPE=INS:ME:LINE1;END=178294566;SVLEN=348;AN=20618;AC=1;AF=4.850130953535746e-05;N_HET=1;N_HOMALT=0;N_HOMREF=10308;FREQ_HET=9.700261907071492e-05;FREQ_HOMALT=0.0;FREQ_HOMREF=0.9999029973809292;N_BI_GENOS=10309;East_Han_AN=6554;East_Han_AC=1;East_Han_AF=
chr4	178325557 .	A	<INS:ME:LINE1>	.	PASS	SVTYPE=INS:ME:LINE1;END=178325557;SVLEN=625;AN=20618;AC=1;AF=4.850130953535746e-05;N_HET=1;N_HOMALT=0;N_HOMREF=10308;FREQ_HET=9.700261907071492e-05;FREQ_HOMALT=0.0;FREQ_HOMREF=0.9999029973809292;N_BI_GENOS=10309;East_Han_AN=6554;East_Han_AC=1;East_Han_AF=
chr4	178338629 .	T	<INS:ME:LINE1>	.	PASS	SVTYPE=INS:ME:LINE1;END=178338644;SVLEN=673;AN=20618;AC=1;AF=4.850130953535746e-05;N_HET=1;N_HOMALT=0;N_HOMREF=10308;FREQ_HET=9.700261907071492e-05;FREQ_HOMALT=0.0;FREQ_HOMREF=0.9999029973809292;N_BI_GENOS=10309;North_Han_AN=2272;North_Han_AC=1;North_Han_
chr4	178728857 .	C	<INS:ME:LINE1>	.	PASS	SVTYPE=INS:ME:LINE1;END=178728857;SVLEN=391;AN=20618;AC=1;AF=4.850130953535746e-05;N_HET=1;N_HOMALT=0;N_HOMREF=10308;FREQ_HET=9.700261907071492e-05;FREQ_HOMALT=0.0;FREQ_HOMREF=0.9999029973809292;N_BI_GENOS=10309;East_Han_AN=6554;East_Han_AC=1;East_Han_AF=
chr4	179169701 .	T	<INS:ME:LINE1>	.	PASS	SVTYPE=INS:ME:LINE1;END=179169701;SVLEN=0;AN=20618;AC=1;AF=4.850130953535746e-05;N_HET=1;N_HOMALT=0;N_HOMREF=10308;FREQ_HET=9.700261907071492e-05;FREQ_HOMALT=0.0;FREQ_HOMREF=0.9999029973809292;N_BI_GENOS=10309;Lingnan_Han_AN=1888;Lingnan_Han_AC=1;Lingnan_
chr4	179291943 .	A	<INS:ME:LINE1>	.	PASS	SVTYPE=INS:ME:LINE1;END=179291955;SVLEN=1162;AN=20618;AC=3;AF=0.00014550392860607236;N_HET=3;N_HOMALT=0;N_HOMREF=10306;FREQ_HET=0.0002910078572121447;FREQ_HOMALT=0.0;FREQ_HOMREF=0.9997089921427879;N_BI_GENOS=10309;South_Han_AN=1496;South_Han_AC=1;South_Ha
chr4	179360139 .	A	<INS:ME:LINE1>	.	PASS	SVTYPE=INS:ME:LINE1;END=179360139;SVLEN=2011;AN=20618;AC=1;AF=4.850130953535746e-05;N_HET=1;N_HOMALT=0;N_HOMREF=10308;FREQ_HET=9.700261907071492e-05;FREQ_HOMALT=0.0;FREQ_HOMREF=0.9999029973809292;N_BI_GENOS=10309;Central_Han_AN=2128;Central_Han_AC=1;Centr
chr4	179572841 .	T	<INS:ME:LINE1>	.	PASS	SVTYPE=INS:ME:LINE1;END=179572841;SVLEN=706;AN=20618;AC=1;AF=4.850130953535746e-05;N_HET=1;N_HOMALT=0;N_HOMREF=10308;FREQ_HET=9.700261907071492e-05;FREQ_HOMALT=0.0;FREQ_HOMREF=0.9999029973809292;N_BI_GENOS=10309;Southeast_Han_AN=1908;Southeast_Han_AC=1;So
chr4	179857398 .	A	<INS:ME:LINE1>	.	PASS	SVTYPE=INS:ME:LINE1;END=179857398;SVLEN=0;AN=20618;AC=2;AF=9.700261907071492e-05;N_HET=0;N_HOMALT=1;N_HOMREF=10308;FREQ_HET=0.0;FREQ_HOMALT=9.700261907071492e-05;FREQ_HOMREF=0.9999029973809292;N_BI_GENOS=10309;East_Han_AN=6554;East_Han_AC=2;East_Han_AF=0.
chr4	179908261 .	A	<INS:ME:LINE1>	.	PASS	SVTYPE=INS:ME:LINE1;END=179908274;SVLEN=206;AN=20618;AC=2;AF=9.700261907071492e-05;N_HET=0;N_HOMALT=1;N_HOMREF=10308;FREQ_HET=0.0;FREQ_HOMALT=9.700261907071492e-05;FREQ_HOMREF=0.9999029973809292;N_BI_GENOS=10309;Central_Han_AN=2128;Central_Han_AC=2;Centra
chr4	180204265 .	A	<INS:ME:LINE1>	.	PASS	SVTYPE=INS:ME:LINE1;END=180204283;SVLEN=497;AN=20618;AC=1;AF=4.850130953535746e-05;N_HET=1;N_HOMALT=0;N_HOMREF=10308;FREQ_HET=9.700261907071492e-05;FREQ_HOMALT=0.0;FREQ_HOMREF=0.9999029973809292;N_BI_GENOS=10309;South_Han_AN=1496;South_Han_AC=1;South_Han_
chr4	180774332 .	A	<INS:ME:LINE1>	.	PASS	SVTYPE=INS:ME:LINE1;END=180774349;SVLEN=629;AN=20618;AC=1;AF=4.850130953535746e-05;N_HET=1;N_HOMALT=0;N_HOMREF=10308;FREQ_HET=9.700261907071492e-05;FREQ_HOMALT=0.0;FREQ_HOMREF=0.9999029973809292;N_BI_GENOS=10309;Zhuang_AN=444;Zhuang_AC=1;Zhuang_AF=0.00225
chr4	180798203 .	T	<INS:ME:LINE1>	.	PASS	SVTYPE=INS:ME:LINE1;END=180798217;SVLEN=540;AN=20618;AC=1;AF=4.850130953535746e-05;N_HET=1;N_HOMALT=0;N_HOMREF=10308;FREQ_HET=9.700261907071492e-05;FREQ_HOMALT=0.0;FREQ_HOMREF=0.9999029973809292;N_BI_GENOS=10309;North_Han_AN=2272;North_Han_AC=1;North_Han_
chr4	181083996 .	T	<INS:ME:LINE1>	.	PASS	SVTYPE=INS:ME:LINE1;END=181084009;SVLEN=171;AN=20618;AC=17;AF=0.0008245222621010767;N_HET=17;N_HOMALT=0;N_HOMREF=10292;FREQ_HET=0.0016490445242021534;FREQ_HOMALT=0.0;FREQ_HOMREF=0.9983509554757979;N_BI_GENOS=10309;Northwest_Han_AN=1296;Northwest_Han_AC=2;
chr4	181117582 .	C	<INS:ME:LINE1>	.	PASS	SVTYPE=INS:ME:LINE1;END=181117593;SVLEN=52;AN=20618;AC=3;AF=0.00014550392860607236;N_HET=3;N_HOMALT=0;N_HOMREF=10306;FREQ_HET=0.0002910078572121447;FREQ_HOMALT=0.0;FREQ_HOMREF=0.9997089921427879;N_BI_GENOS=10309;Northwest_Han_AN=1296;Northwest_Han_AC=2;No
chr4	181496905 .	C	<INS:ME:LINE1>	.	PASS	SVTYPE=INS:ME:LINE1;END=181496954;SVLEN=112;AN=20618;AC=2;AF=9.700261907071492e-05;N_HET=2;N_HOMALT=0;N_HOMREF=10307;FREQ_HET=0.00019400523814142983;FREQ_HOMALT=0.0;FREQ_HOMREF=0.9998059947618586;N_BI_GENOS=10309;Lingnan_Han_AN=1888;Lingnan_Han_AC=1;Lingn
chr4	181856326 .	A	<INS:ME:LINE1>	.	PASS	SVTYPE=INS:ME:LINE1;END=181856341;SVLEN=2613;AN=20618;AC=1;AF=4.850130953535746e-05;N_HET=1;N_HOMALT=0;N_HOMREF=10308;FREQ_HET=9.700261907071492e-05;FREQ_HOMALT=0.0;FREQ_HOMREF=0.9999029973809292;N_BI_GENOS=10309;Southeast_Han_AN=1908;Southeast_Han_AC=1;S
chr4	182738336 .	A	<INS:ME:LINE1>	.	PASS	SVTYPE=INS:ME:LINE1;END=182738351;SVLEN=1596;AN=20618;AC=3;AF=0.00014550392860607236;N_HET=3;N_HOMALT=0;N_HOMREF=10306;FREQ_HET=0.0002910078572121447;FREQ_HOMALT=0.0;FREQ_HOMREF=0.9997089921427879;N_BI_GENOS=10309;Miao_AN=482;Miao_AC=3;Miao_AF=0.006224066
chr4	183201929 .	G	<INS:ME:LINE1>	.	PASS	SVTYPE=INS:ME:LINE1;END=183201945;SVLEN=742;AN=20618;AC=4;AF=0.00019400523814142983;N_HET=4;N_HOMALT=0;N_HOMREF=10305;FREQ_HET=0.00038801047628285966;FREQ_HOMALT=0.0;FREQ_HOMREF=0.9996119895237171;N_BI_GENOS=10309;Northwest_Han_AN=1296;Northwest_Han_AC=1;
chr4	183980628 .	C	<INS:ME:LINE1>	.	PASS	SVTYPE=INS:ME:LINE1;END=183980646;SVLEN=5997;AN=20618;AC=1;AF=4.850130953535746e-05;N_HET=1;N_HOMALT=0;N_HOMREF=10308;FREQ_HET=9.700261907071492e-05;FREQ_HOMALT=0.0;FREQ_HOMREF=0.9999029973809292;N_BI_GENOS=10309;East_Han_AN=6554;East_Han_AC=1;East_Han_AF
chr4	184163908 .	G	<INS:ME:LINE1>	.	PASS	SVTYPE=INS:ME:LINE1;END=184163925;SVLEN=860;AN=20618;AC=2;AF=9.700261907071492e-05;N_HET=2;N_HOMALT=0;N_HOMREF=10307;FREQ_HET=0.00019400523814142983;FREQ_HOMALT=0.0;FREQ_HOMREF=0.9998059947618586;N_BI_GENOS=10309;Lingnan_Han_AN=1888;Lingnan_Han_AC=1;Lingn
chr4	184360538 .	G	<INS:ME:LINE1>	.	PASS	SVTYPE=INS:ME:LINE1;END=184360551;SVLEN=443;AN=20618;AC=1;AF=4.850130953535746e-05;N_HET=1;N_HOMALT=0;N_HOMREF=10308;FREQ_HET=9.700261907071492e-05;FREQ_HOMALT=0.0;FREQ_HOMREF=0.9999029973809292;N_BI_GENOS=10309;Central_Han_AN=2128;Central_Han_AC=1;Centra
chr4	184421762 .	A	<INS:ME:LINE1>	.	PASS	SVTYPE=INS:ME:LINE1;END=184421778;SVLEN=5997;AN=20618;AC=4;AF=0.00019400523814142983;N_HET=4;N_HOMALT=0;N_HOMREF=10305;FREQ_HET=0.00038801047628285966;FREQ_HOMALT=0.0;FREQ_HOMREF=0.9996119895237171;N_BI_GENOS=10309;South_Han_AN=1496;South_Han_AC=1;South_H
chr4	184567572 .	T	<INS:ME:LINE1>	.	PASS	SVTYPE=INS:ME:LINE1;END=184567572;SVLEN=996;AN=20618;AC=1;AF=4.850130953535746e-05;N_HET=1;N_HOMALT=0;N_HOMREF=10308;FREQ_HET=9.700261907071492e-05;FREQ_HOMALT=0.0;FREQ_HOMREF=0.9999029973809292;N_BI_GENOS=10309;East_Han_AN=6554;East_Han_AC=1;East_Han_AF=
chr4	185274754 .	A	<INS:ME:LINE1>	.	PASS	SVTYPE=INS:ME:LINE1;END=185274770;SVLEN=882;AN=20618;AC=1;AF=4.850130953535746e-05;N_HET=1;N_HOMALT=0;N_HOMREF=10308;FREQ_HET=9.700261907071492e-05;FREQ_HOMALT=0.0;FREQ_HOMREF=0.9999029973809292;N_BI_GENOS=10309;East_Han_AN=6554;East_Han_AC=1;East_Han_AF=
chr4	185549292 .	T	<INS:ME:LINE1>	.	PASS	SVTYPE=INS:ME:LINE1;END=185549310;SVLEN=5075;AN=20618;AC=1;AF=4.850130953535746e-05;N_HET=1;N_HOMALT=0;N_HOMREF=10308;FREQ_HET=9.700261907071492e-05;FREQ_HOMALT=0.0;FREQ_HOMREF=0.9999029973809292;N_BI_GENOS=10309;Northwest_Han_AN=1296;Northwest_Han_AC=1;N
chr4	185986072 .	A	<INS:ME:LINE1>	.	PASS	SVTYPE=INS:ME:LINE1;END=185986090;SVLEN=6038;AN=20618;AC=1;AF=4.850130953535746e-05;N_HET=1;N_HOMALT=0;N_HOMREF=10308;FREQ_HET=9.700261907071492e-05;FREQ_HOMALT=0.0;FREQ_HOMREF=0.9999029973809292;N_BI_GENOS=10309;East_Han_AN=6554;East_Han_AC=1;East_Han_AF
chr4	186278358 .	G	<INS:ME:LINE1>	.	PASS	SVTYPE=INS:ME:LINE1;END=186278376;SVLEN=718;AN=20618;AC=1;AF=4.850130953535746e-05;N_HET=1;N_HOMALT=0;N_HOMREF=10308;FREQ_HET=9.700261907071492e-05;FREQ_HOMALT=0.0;FREQ_HOMREF=0.9999029973809292;N_BI_GENOS=10309;East_Han_AN=6554;East_Han_AC=1;East_Han_AF=
chr4	187321701 .	T	<INS:ME:LINE1>	.	PASS	SVTYPE=INS:ME:LINE1;END=187321701;SVLEN=510;AN=20618;AC=1;AF=4.850130953535746e-05;N_HET=1;N_HOMALT=0;N_HOMREF=10308;FREQ_HET=9.700261907071492e-05;FREQ_HOMALT=0.0;FREQ_HOMREF=0.9999029973809292;N_BI_GENOS=10309;East_Han_AN=6554;East_Han_AC=1;East_Han_AF=
chr4	187338138 .	A	<INS:ME:LINE1>	.	PASS	SVTYPE=INS:ME:LINE1;END=187338149;SVLEN=1616;AN=20618;AC=1;AF=4.850130953535746e-05;N_HET=1;N_HOMALT=0;N_HOMREF=10308;FREQ_HET=9.700261907071492e-05;FREQ_HOMALT=0.0;FREQ_HOMREF=0.9999029973809292;N_BI_GENOS=10309;Yi_AN=422;Yi_AC=1;Yi_AF=0.0023696682464454
chr4	187341228 .	A	<INS:ME:LINE1>	.	PASS	SVTYPE=INS:ME:LINE1;END=187341228;SVLEN=506;AN=20618;AC=1;AF=4.850130953535746e-05;N_HET=1;N_HOMALT=0;N_HOMREF=10308;FREQ_HET=9.700261907071492e-05;FREQ_HOMALT=0.0;FREQ_HOMREF=0.9999029973809292;N_BI_GENOS=10309;East_Han_AN=6554;East_Han_AC=1;East_Han_AF=
chr4	187426246 .	A	<INS:ME:LINE1>	.	PASS	SVTYPE=INS:ME:LINE1;END=187426257;SVLEN=97;AN=20618;AC=1;AF=4.850130953535746e-05;N_HET=1;N_HOMALT=0;N_HOMREF=10308;FREQ_HET=9.700261907071492e-05;FREQ_HOMALT=0.0;FREQ_HOMREF=0.9999029973809292;N_BI_GENOS=10309;East_Han_AN=6554;East_Han_AC=1;East_Han_AF=0
chr4	187547428 .	T	<INS:ME:LINE1>	.	PASS	SVTYPE=INS:ME:LINE1;END=187547444;SVLEN=3544;AN=20618;AC=4;AF=0.00019400523814142983;N_HET=4;N_HOMALT=0;N_HOMREF=10305;FREQ_HET=0.00038801047628285966;FREQ_HOMALT=0.0;FREQ_HOMREF=0.9996119895237171;N_BI_GENOS=10309;South_Han_AN=1496;South_Han_AC=1;South_H
chr4	187570311 .	A	<INS:ME:LINE1>	.	PASS	SVTYPE=INS:ME:LINE1;END=187570326;SVLEN=6075;AN=20618;AC=1;AF=4.850130953535746e-05;N_HET=1;N_HOMALT=0;N_HOMREF=10308;FREQ_HET=9.700261907071492e-05;FREQ_HOMALT=0.0;FREQ_HOMREF=0.9999029973809292;N_BI_GENOS=10309;Northwest_Han_AN=1296;Northwest_Han_AC=1;N
chr4	187826491 .	A	<INS:ME:LINE1>	.	PASS	SVTYPE=INS:ME:LINE1;END=187826508;SVLEN=5997;AN=20618;AC=1;AF=4.850130953535746e-05;N_HET=1;N_HOMALT=0;N_HOMREF=10308;FREQ_HET=9.700261907071492e-05;FREQ_HOMALT=0.0;FREQ_HOMREF=0.9999029973809292;N_BI_GENOS=10309;North_Han_AN=2272;North_Han_AC=1;North_Han
chr4	187958823 .	G	<INS:ME:LINE1>	.	PASS	SVTYPE=INS:ME:LINE1;END=187958838;SVLEN=6036;AN=20618;AC=1;AF=4.850130953535746e-05;N_HET=1;N_HOMALT=0;N_HOMREF=10308;FREQ_HET=9.700261907071492e-05;FREQ_HOMALT=0.0;FREQ_HOMREF=0.9999029973809292;N_BI_GENOS=10309;Northwest_Han_AN=1296;Northwest_Han_AC=1;N
chr4	188736737 .	A	<INS:ME:LINE1>	.	PASS	SVTYPE=INS:ME:LINE1;END=188736754;SVLEN=5999;AN=20618;AC=2;AF=9.700261907071492e-05;N_HET=2;N_HOMALT=0;N_HOMREF=10307;FREQ_HET=0.00019400523814142983;FREQ_HOMALT=0.0;FREQ_HOMREF=0.9998059947618586;N_BI_GENOS=10309;East_Han_AN=6554;East_Han_AC=1;East_Han_A
chr5	197057 .	G	<INS:ME:LINE1>	.	PASS	SVTYPE=INS:ME:LINE1;END=197057;SVLEN=0;AN=20618;AC=30;AF=0.0014550392860607237;N_HET=30;N_HOMALT=0;N_HOMREF=10279;FREQ_HET=0.0029100785721214474;FREQ_HOMALT=0.0;FREQ_HOMREF=0.9970899214278786;N_BI_GENOS=10309;South_Han_AN=1496;South_Han_AC=5;South_Han_AF=
chr5	197168 .	G	<INS:ME:LINE1>	.	PASS	SVTYPE=INS:ME:LINE1;END=197168;SVLEN=0;AN=20618;AC=11;AF=0.0005335144404888932;N_HET=11;N_HOMALT=0;N_HOMREF=10298;FREQ_HET=0.0010670288809777864;FREQ_HOMALT=0.0;FREQ_HOMREF=0.9989329711902222;N_BI_GENOS=10309;South_Han_AN=1496;South_Han_AC=3;South_Han_AF=0.
chr5	1585387 .	A	<INS:ME:LINE1>	.	PASS	SVTYPE=INS:ME:LINE1;END=1585387;SVLEN=1392;AN=20618;AC=1;AF=4.850130953535746e-05;N_HET=1;N_HOMALT=0;N_HOMREF=10308;FREQ_HET=9.700261907071492e-05;FREQ_HOMALT=0.0;FREQ_HOMREF=0.9999029973809292;N_BI_GENOS=10309;South_Han_AN=1496;South_Han_AC=1;South_Han_A
chr5	1645658 .	T	<INS:ME:LINE1>	.	PASS	SVTYPE=INS:ME:LINE1;END=1645659;SVLEN=794;AN=20618;AC=564;AF=0.027354738577941604;N_HET=198;N_HOMALT=183;N_HOMREF=9928;FREQ_HET=0.019206518557600155;FREQ_HOMALT=0.01775147928994083;FREQ_HOMREF=0.9630420021340577;N_BI_GENOS=10309;South_Han_AN=1496;South_Han
chr5	2150655 .	G	<INS:ME:LINE1>	.	PASS	SVTYPE=INS:ME:LINE1;END=2150669;SVLEN=6007;AN=20618;AC=1;AF=4.850130953535746e-05;N_HET=1;N_HOMALT=0;N_HOMREF=10308;FREQ_HET=9.700261907071492e-05;FREQ_HOMALT=0.0;FREQ_HOMREF=0.9999029973809292;N_BI_GENOS=10309;North_Han_AN=2272;North_Han_AC=1;North_Han_A
chr5	2250302 .	A	<INS:ME:LINE1>	.	PASS	SVTYPE=INS:ME:LINE1;END=2250316;SVLEN=6000;AN=20618;AC=3;AF=0.00014550392860607236;N_HET=3;N_HOMALT=0;N_HOMREF=10306;FREQ_HET=0.0002910078572121447;FREQ_HOMALT=0.0;FREQ_HOMREF=0.9997089921427879;N_BI_GENOS=10309;North_Han_AN=2272;North_Han_AC=1;North_Han_
chr5	2383696 .	C	<INS:ME:LINE1>	.	PASS	SVTYPE=INS:ME:LINE1;END=2383704;SVLEN=1285;AN=20618;AC=1;AF=4.850130953535746e-05;N_HET=1;N_HOMALT=0;N_HOMREF=10308;FREQ_HET=9.700261907071492e-05;FREQ_HOMALT=0.0;FREQ_HOMREF=0.9999029973809292;N_BI_GENOS=10309;North_Han_AN=2272;North_Han_AC=1;North_Han_A
chr5	2768914 .	T	<INS:ME:LINE1>	.	PASS	SVTYPE=INS:ME:LINE1;END=2768931;SVLEN=1549;AN=20618;AC=3;AF=0.00014550392860607236;N_HET=1;N_HOMALT=1;N_HOMREF=10307;FREQ_HET=9.700261907071492e-05;FREQ_HOMALT=9.700261907071492e-05;FREQ_HOMREF=0.9998059947618586;N_BI_GENOS=10309;South_Han_AN=1496;South_H
chr5	2816764 .	G	<INS:ME:LINE1>	.	PASS	SVTYPE=INS:ME:LINE1;END=2816774;SVLEN=693;AN=20618;AC=1;AF=4.850130953535746e-05;N_HET=1;N_HOMALT=0;N_HOMREF=10308;FREQ_HET=9.700261907071492e-05;FREQ_HOMALT=0.0;FREQ_HOMREF=0.9999029973809292;N_BI_GENOS=10309;East_Han_AN=6554;East_Han_AC=1;East_Han_AF=0.
chr5	3428480 .	C	<INS:ME:LINE1>	.	PASS	SVTYPE=INS:ME:LINE1;END=3428480;SVLEN=2445;AN=20618;AC=1;AF=4.850130953535746e-05;N_HET=1;N_HOMALT=0;N_HOMREF=10308;FREQ_HET=9.700261907071492e-05;FREQ_HOMALT=0.0;FREQ_HOMREF=0.9999029973809292;N_BI_GENOS=10309;Lingnan_Han_AN=1888;Lingnan_Han_AC=1;Lingnan
chr5	4243264 .	G	<INS:ME:LINE1>	.	PASS	SVTYPE=INS:ME:LINE1;END=4243283;SVLEN=5996;AN=20618;AC=1;AF=4.850130953535746e-05;N_HET=1;N_HOMALT=0;N_HOMREF=10308;FREQ_HET=9.700261907071492e-05;FREQ_HOMALT=0.0;FREQ_HOMREF=0.9999029973809292;N_BI_GENOS=10309;East_Han_AN=6554;East_Han_AC=1;East_Han_AF=0
chr5	4295015 .	C	<INS:ME:LINE1>	.	PASS	SVTYPE=INS:ME:LINE1;END=4295031;SVLEN=1693;AN=20618;AC=2;AF=9.700261907071492e-05;N_HET=2;N_HOMALT=0;N_HOMREF=10307;FREQ_HET=0.00019400523814142983;FREQ_HOMALT=0.0;FREQ_HOMREF=0.9998059947618586;N_BI_GENOS=10309;North_Han_AN=2272;North_Han_AC=1;North_Han_
chr5	4520723 .	A	<INS:ME:LINE1>	.	PASS	SVTYPE=INS:ME:LINE1;END=4520741;SVLEN=326;AN=20618;AC=1;AF=4.850130953535746e-05;N_HET=1;N_HOMALT=0;N_HOMREF=10308;FREQ_HET=9.700261907071492e-05;FREQ_HOMALT=0.0;FREQ_HOMREF=0.9999029973809292;N_BI_GENOS=10309;East_Han_AN=6554;East_Han_AC=1;East_Han_AF=0.
chr5	4679669 .	A	<INS:ME:LINE1>	.	PASS	SVTYPE=INS:ME:LINE1;END=4679685;SVLEN=290;AN=20618;AC=3;AF=0.00014550392860607236;N_HET=3;N_HOMALT=0;N_HOMREF=10306;FREQ_HET=0.0002910078572121447;FREQ_HOMALT=0.0;FREQ_HOMREF=0.9997089921427879;N_BI_GENOS=10309;South_Han_AN=1496;South_Han_AC=1;South_Han_A
chr5	4793717 .	A	<INS:ME:LINE1>	.	PASS	SVTYPE=INS:ME:LINE1;END=4793730;SVLEN=1072;AN=20618;AC=1;AF=4.850130953535746e-05;N_HET=1;N_HOMALT=0;N_HOMREF=10308;FREQ_HET=9.700261907071492e-05;FREQ_HOMALT=0.0;FREQ_HOMREF=0.9999029973809292;N_BI_GENOS=10309;Lingnan_Han_AN=1888;Lingnan_Han_AC=1;Lingnan
chr5	4920025 .	A	<INS:ME:LINE1>	.	PASS	SVTYPE=INS:ME:LINE1;END=4920039;SVLEN=1039;AN=20618;AC=1;AF=4.850130953535746e-05;N_HET=1;N_HOMALT=0;N_HOMREF=10308;FREQ_HET=9.700261907071492e-05;FREQ_HOMALT=0.0;FREQ_HOMREF=0.9999029973809292;N_BI_GENOS=10309;Central_Han_AN=2128;Central_Han_AC=1;Central
chr5	5141376 .	T	<INS:ME:LINE1>	.	PASS	SVTYPE=INS:ME:LINE1;END=5141390;SVLEN=1848;AN=20618;AC=4;AF=0.00019400523814142983;N_HET=4;N_HOMALT=0;N_HOMREF=10305;FREQ_HET=0.00038801047628285966;FREQ_HOMALT=0.0;FREQ_HOMREF=0.9996119895237171;N_BI_GENOS=10309;South_Han_AN=1496;South_Han_AC=1;South_Han
chr5	5603892 .	T	<INS:ME:LINE1>	.	PASS	SVTYPE=INS:ME:LINE1;END=5603894;SVLEN=467;AN=20618;AC=1;AF=4.850130953535746e-05;N_HET=1;N_HOMALT=0;N_HOMREF=10308;FREQ_HET=9.700261907071492e-05;FREQ_HOMALT=0.0;FREQ_HOMREF=0.9999029973809292;N_BI_GENOS=10309;Miao_AN=482;Miao_AC=1;Miao_AF=0.0020746887966
chr5	5950186 .	T	<INS:ME:LINE1>	.	PASS	SVTYPE=INS:ME:LINE1;END=5950190;SVLEN=1352;AN=20618;AC=3;AF=0.00014550392860607236;N_HET=3;N_HOMALT=0;N_HOMREF=10306;FREQ_HET=0.0002910078572121447;FREQ_HOMALT=0.0;FREQ_HOMREF=0.9997089921427879;N_BI_GENOS=10309;Northwest_Han_AN=1296;Northwest_Han_AC=1;No
chr5	6475617 .	G	<INS:ME:LINE1>	.	PASS	SVTYPE=INS:ME:LINE1;END=6475632;SVLEN=853;AN=20618;AC=9;AF=0.0004365117858182171;N_HET=7;N_HOMALT=1;N_HOMREF=10301;FREQ_HET=0.0006790183334950044;FREQ_HOMALT=9.700261907071492e-05;FREQ_HOMREF=0.9992239790474343;N_BI_GENOS=10309;Lingnan_Han_AN=1888;Lingnan

chr5	6548838	.	G	<INS:ME:LINE1>	.	PASS	SVTYPE=INS:ME:LINE1;END=6548852;SVLEN=6064;AN=20618;AC=1;AF=4.850130953535746e-05;N_HET=1;N_HOMALT=0;N_HOMREF=10308;FREQ_HET=9.700261907071492e-05;FREQ_HOMALT=0.0;FREQ_HOMREF=0.9999029973809292;N_BI_GENOS=10309;Southeast_Han_AN=1908;Southeast_Han_AC=1;Sou
chr5	6696869	.	T	<INS:ME:LINE1>	.	PASS	SVTYPE=INS:ME:LINE1;END=6696869;SVLEN=206;AN=20618;AC=1;AF=4.850130953535746e-05;N_HET=1;N_HOMALT=0;N_HOMREF=10308;FREQ_HET=9.700261907071492e-05;FREQ_HOMALT=0.0;FREQ_HOMREF=0.9999029973809292;N_BI_GENOS=10309;East_Han_AN=6554;East_Han_AC=1;East_Han_AF=0.
chr5	6744442	.	T	<INS:ME:LINE1>	.	PASS	SVTYPE=INS:ME:LINE1;END=6744454;SVLEN=5976;AN=20618;AC=4;AF=0.00019400523814142983;N_HET=4;N_HOMALT=0;N_HOMREF=10305;FREQ_HET=0.00038801047628285966;FREQ_HOMALT=0.0;FREQ_HOMREF=0.9996119895237171;N_BI_GENOS=10309;Northwest_Han_AN=1296;Northwest_Han_AC=1;N
chr5	6804604	.	G	<INS:ME:LINE1>	.	PASS	SVTYPE=INS:ME:LINE1;END=6804621;SVLEN=5971;AN=20618;AC=1;AF=4.850130953535746e-05;N_HET=1;N_HOMALT=0;N_HOMREF=10308;FREQ_HET=9.700261907071492e-05;FREQ_HOMALT=0.0;FREQ_HOMREF=0.9999029973809292;N_BI_GENOS=10309;Lingnan_Han_AN=1888;Lingnan_Han_AC=1;Lingnan
chr5	7008801	.	A	<INS:ME:LINE1>	.	PASS	SVTYPE=INS:ME:LINE1;END=7008801;SVLEN=508;AN=20618;AC=1;AF=4.850130953535746e-05;N_HET=1;N_HOMALT=0;N_HOMREF=10308;FREQ_HET=9.700261907071492e-05;FREQ_HOMALT=0.0;FREQ_HOMREF=0.9999029973809292;N_BI_GENOS=10309;East_Han_AN=6554;East_Han_AC=1;East_Han_AF=0.
chr5	7035847	.	T	<INS:ME:LINE1>	.	PASS	SVTYPE=INS:ME:LINE1;END=7035861;SVLEN=6000;AN=20618;AC=1;AF=4.850130953535746e-05;N_HET=1;N_HOMALT=0;N_HOMREF=10308;FREQ_HET=9.700261907071492e-05;FREQ_HOMALT=0.0;FREQ_HOMREF=0.9999029973809292;N_BI_GENOS=10309;Lingnan_Han_AN=1888;Lingnan_Han_AC=1;Lingnan
chr5	7153787	.	A	<INS:ME:LINE1>	.	PASS	SVTYPE=INS:ME:LINE1;END=7153787;SVLEN=10;AN=20618;AC=1;AF=4.850130953535746e-05;N_HET=1;N_HOMALT=0;N_HOMREF=10308;FREQ_HET=9.700261907071492e-05;FREQ_HOMALT=0.0;FREQ_HOMREF=0.9999029973809292;N_BI_GENOS=10309;East_Han_AN=6554;East_Han_AC=1;East_Han_AF=0.0
chr5	8467537	.	T	<INS:ME:LINE1>	.	PASS	SVTYPE=INS:ME:LINE1;END=8467537;SVLEN=89;AN=20618;AC=1;AF=4.850130953535746e-05;N_HET=1;N_HOMALT=0;N_HOMREF=10308;FREQ_HET=9.700261907071492e-05;FREQ_HOMALT=0.0;FREQ_HOMREF=0.9999029973809292;N_BI_GENOS=10309;Tibetan_AN=414;Tibetan_AC=1;Tibetan_AF=0.00241
chr5	8515781	.	A	<INS:ME:LINE1>	.	PASS	SVTYPE=INS:ME:LINE1;END=8515792;SVLEN=5995;AN=20618;AC=2;AF=9.700261907071492e-05;N_HET=2;N_HOMALT=0;N_HOMREF=10307;FREQ_HET=0.00019400523814142983;FREQ_HOMALT=0.0;FREQ_HOMREF=0.9998059947618586;N_BI_GENOS=10309;South_Han_AN=1496;South_Han_AC=1;South_Han_
chr5	8622965	.	A	<INS:ME:LINE1>	.	PASS	SVTYPE=INS:ME:LINE1;END=8622970;SVLEN=2055;AN=20618;AC=1;AF=4.850130953535746e-05;N_HET=1;N_HOMALT=0;N_HOMREF=10308;FREQ_HET=9.700261907071492e-05;FREQ_HOMALT=0.0;FREQ_HOMREF=0.9999029973809292;N_BI_GENOS=10309;East_Han_AN=6554;East_Han_AC=1;East_Han_AF=0
chr5	8644699	.	A	<INS:ME:LINE1>	.	PASS	SVTYPE=INS:ME:LINE1;END=8644712;SVLEN=698;AN=20618;AC=1;AF=4.850130953535746e-05;N_HET=1;N_HOMALT=0;N_HOMREF=10308;FREQ_HET=9.700261907071492e-05;FREQ_HOMALT=0.0;FREQ_HOMREF=0.9999029973809292;N_BI_GENOS=10309;South_Han_AN=1496;South_Han_AC=1;South_Han_AF
chr5	8666411	.	G	<INS:ME:LINE1>	.	PASS	SVTYPE=INS:ME:LINE1;END=8666422;SVLEN=677;AN=20618;AC=1;AF=4.850130953535746e-05;N_HET=1;N_HOMALT=0;N_HOMREF=10308;FREQ_HET=9.700261907071492e-05;FREQ_HOMALT=0.0;FREQ_HOMREF=0.9999029973809292;N_BI_GENOS=10309;East_Han_AN=6554;East_Han_AC=1;East_Han_AF=0.
chr5	8749402	.	A	<INS:ME:LINE1>	.	PASS	SVTYPE=INS:ME:LINE1;END=8749416;SVLEN=1465;AN=20618;AC=10946;AF=0.5308953341740227;N_HET=4176;N_HOMALT=3385;N_HOMREF=2748;FREQ_HET=0.40508293723930544;FREQ_HOMALT=0.32835386555436996;FREQ_HOMREF=0.26656319720632466;N_BI_GENOS=10309;South_Han_AN=1496;South_
chr5	8749893	.	A	<INS:ME:LINE1>	.	PASS	SVTYPE=INS:ME:LINE1;END=8749910;SVLEN=1346;AN=20618;AC=16;AF=0.0007760209525657193;N_HET=16;N_HOMALT=0;N_HOMREF=10293;FREQ_HET=0.0015520419051314386;FREQ_HOMALT=0.0;FREQ_HOMREF=0.9984479580948685;N_BI_GENOS=10309;South_Han_AN=1496;South_Han_AC=1;South_Han
chr5	9099614	.	T	<INS:ME:LINE1>	.	PASS	SVTYPE=INS:ME:LINE1;END=9099619;SVLEN=633;AN=20618;AC=2;AF=9.700261907071492e-05;N_HET=2;N_HOMALT=0;N_HOMREF=10307;FREQ_HET=0.00019400523814142983;FREQ_HOMALT=0.0;FREQ_HOMREF=0.9998059947618586;N_BI_GENOS=10309;East_Han_AN=6554;East_Han_AC=2;East_Han_AF=0
chr5	9288560	.	A	<INS:ME:LINE1>	.	PASS	SVTYPE=INS:ME:LINE1;END=9288577;SVLEN=1741;AN=20618;AC=1;AF=4.850130953535746e-05;N_HET=1;N_HOMALT=0;N_HOMREF=10308;FREQ_HET=9.700261907071492e-05;FREQ_HOMALT=0.0;FREQ_HOMREF=0.9999029973809292;N_BI_GENOS=10309;East_Han_AN=6554;East_Han_AC=1;East_Han_AF=0
chr5	9749265	.	A	<INS:ME:LINE1>	.	PASS	SVTYPE=INS:ME:LINE1;END=9749275;SVLEN=3579;AN=20618;AC=2;AF=9.700261907071492e-05;N_HET=2;N_HOMALT=0;N_HOMREF=10307;FREQ_HET=0.00019400523814142983;FREQ_HOMALT=0.0;FREQ_HOMREF=0.9998059947618586;N_BI_GENOS=10309;East_Han_AN=6554;East_Han_AC=2;East_Han_AF=
chr5	9978143	.	G	<INS:ME:LINE1>	.	PASS	SVTYPE=INS:ME:LINE1;END=9978148;SVLEN=1050;AN=20618;AC=1;AF=4.850130953535746e-05;N_HET=1;N_HOMALT=0;N_HOMREF=10308;FREQ_HET=9.700261907071492e-05;FREQ_HOMALT=0.0;FREQ_HOMREF=0.9999029973809292;N_BI_GENOS=10309;Central_Han_AN=2128;Central_Han_AC=1;Central
chr5	10887129	.	T	<INS:ME:LINE1>	.	PASS	SVTYPE=INS:ME:LINE1;END=10887146;SVLEN=6000;AN=20618;AC=3;AF=0.00014550392860607236;N_HET=3;N_HOMALT=0;N_HOMREF=10306;FREQ_HET=0.0002910078572121447;FREQ_HOMALT=0.0;FREQ_HOMREF=0.9997089921427879;N_BI_GENOS=10309;Northwest_Han_AN=1296;Northwest_Han_AC=1;N
chr5	11007962	.	A	<INS:ME:LINE1>	.	PASS	SVTYPE=INS:ME:LINE1;END=11007975;SVLEN=5999;AN=20618;AC=1;AF=4.850130953535746e-05;N_HET=1;N_HOMALT=0;N_HOMREF=10308;FREQ_HET=9.700261907071492e-05;FREQ_HOMALT=0.0;FREQ_HOMREF=0.9999029973809292;N_BI_GENOS=10309;Yi_AN=422;Yi_AC=1;Yi_AF=0.0023696682464549
chr5	11391150	.	A	<INS:ME:LINE1>	.	PASS	SVTYPE=INS:ME:LINE1;END=11391164;SVLEN=210;AN=20618;AC=702;AF=0.034047919293820936;N_HET=538;N_HOMALT=82;N_HOMREF=9689;FREQ_HET=0.05218740906004462;FREQ_HOMALT=0.007954214763798623;FREQ_HOMREF=0.9398583761761568;N_BI_GENOS=10309;South_Han_AN=1496;South_Ha
chr5	11875672	.	A	<INS:ME:LINE1>	.	PASS	SVTYPE=INS:ME:LINE1;END=11875686;SVLEN=1080;AN=20618;AC=3;AF=0.00014550392860607236;N_HET=3;N_HOMALT=0;N_HOMREF=10306;FREQ_HET=0.0002910078572121447;FREQ_HOMALT=0.0;FREQ_HOMREF=0.9997089921427879;N_BI_GENOS=10309;East_Han_AN=6554;East_Han_AC=3;East_Han_AF
chr5	11894869	.	C	<INS:ME:LINE1>	.	PASS	SVTYPE=INS:ME:LINE1;END=11894883;SVLEN=586;AN=20618;AC=1;AF=4.850130953535746e-05;N_HET=1;N_HOMALT=0;N_HOMREF=10308;FREQ_HET=9.700261907071492e-05;FREQ_HOMALT=0.0;FREQ_HOMREF=0.9999029973809292;N_BI_GENOS=10309;East_Han_AN=6554;East_Han_AC=1;East_Han_AF=0
chr5	11933519	.	G	<INS:ME:LINE1>	.	PASS	SVTYPE=INS:ME:LINE1;END=11933519;SVLEN=2358;AN=20618;AC=1;AF=4.850130953535746e-05;N_HET=1;N_HOMALT=0;N_HOMREF=10308;FREQ_HET=9.700261907071492e-05;FREQ_HOMALT=0.0;FREQ_HOMREF=0.9999029973809292;N_BI_GENOS=10309;Tibetan_AN=414;Tibetan_AC=1;Tibetan_AF=0.00
chr5	11999459	.	C	<INS:ME:LINE1>	.	PASS	SVTYPE=INS:ME:LINE1;END=11999472;SVLEN=1432;AN=20618;AC=1;AF=4.850130953535746e-05;N_HET=1;N_HOMALT=0;N_HOMREF=10308;FREQ_HET=9.700261907071492e-05;FREQ_HOMALT=0.0;FREQ_HOMREF=0.9999029973809292;N_BI_GENOS=10309;East_Han_AN=6554;East_Han_AC=1;East_Han_AF=
chr5	12380072	.	G	<INS:ME:LINE1>	.	PASS	SVTYPE=INS:ME:LINE1;END=12380072;SVLEN=713;AN=20618;AC=1;AF=4.850130953535746e-05;N_HET=1;N_HOMALT=0;N_HOMREF=10308;FREQ_HET=9.700261907071492e-05;FREQ_HOMALT=0.0;FREQ_HOMREF=0.9999029973809292;N_BI_GENOS=10309;East_Han_AN=6554;East_Han_AC=1;East_Han_AF=0
chr5	12955506	.	A	<INS:ME:LINE1>	.	PASS	SVTYPE=INS:ME:LINE1;END=12955523;SVLEN=1525;AN=20618;AC=1;AF=4.850130953535746e-05;N_HET=1;N_HOMALT=0;N_HOMREF=10308;FREQ_HET=9.700261907071492e-05;FREQ_HOMALT=0.0;FREQ_HOMREF=0.9999029973809292;N_BI_GENOS=10309;South_Han_AN=1496;South_Han_AC=1;South_Han_
chr5	13033891	.	C	<INS:ME:LINE1>	.	PASS	SVTYPE=INS:ME:LINE1;END=13033905;SVLEN=6007;AN=20618;AC=3;AF=0.00014550392860607236;N_HET=3;N_HOMALT=0;N_HOMREF=10306;FREQ_HET=0.0002910078572121447;FREQ_HOMALT=0.0;FREQ_HOMREF=0.9997089921427879;N_BI_GENOS=10309;Lingnan_Han_AN=1888;Lingnan_Han_AC=1;Lingn
chr5	13179144	.	A	<INS:ME:LINE1>	.	PASS	SVTYPE=INS:ME:LINE1;END=13179159;SVLEN=743;AN=20618;AC=1;AF=4.850130953535746e-05;N_HET=1;N_HOMALT=0;N_HOMREF=10308;FREQ_HET=9.700261907071492e-05;FREQ_HOMALT=0.0;FREQ_HOMREF=0.9999029973809292;N_BI_GENOS=10309;East_Han_AN=6554;East_Han_AC=1;East_Han_AF=0
chr5	13231778	.	T	<INS:ME:LINE1>	.	PASS	SVTYPE=INS:ME:LINE1;END=13231794;SVLEN=6000;AN=20618;AC=245;AF=0.011882820836162577;N_HET=243;N_HOMALT=1;N_HOMREF=10065;FREQ_HET=0.023571636434183724;FREQ_HOMALT=9.700261907071492e-05;FREQ_HOMREF=0.9763313609467456;N_BI_GENOS=10309;South_Han_AN=1496;South
chr5	13561016	.	A	<INS:ME:LINE1>	.	PASS	SVTYPE=INS:ME:LINE1;END=13561028;SVLEN=5992;AN=20618;AC=1;AF=4.850130953535746e-05;N_HET=1;N_HOMALT=0;N_HOMREF=10308;FREQ_HET=9.700261907071492e-05;FREQ_HOMALT=0.0;FREQ_HOMREF=0.9999029973809292;N_BI_GENOS=10309;Southeast_Han_AN=1908;Southeast_Han_AC=1;So
chr5	13658626	.	G	<INS:ME:LINE1>	.	PASS	SVTYPE=INS:ME:LINE1;END=13658636;SVLEN=3505;AN=20618;AC=1;AF=4.850130953535746e-05;N_HET=1;N_HOMALT=0;N_HOMREF=10308;FREQ_HET=9.700261907071492e-05;FREQ_HOMALT=0.0;FREQ_HOMREF=0.9999029973809292;N_BI_GENOS=10309;Lingnan_Han_AN=1888;Lingnan_Han_AC=1;Lingna
chr5	13908241	.	G	<INS:ME:LINE1>	.	PASS	SVTYPE=INS:ME:LINE1;END=13908261;SVLEN=2604;AN=20618;AC=3;AF=0.00014550392860607236;N_HET=3;N_HOMALT=0;N_HOMREF=10306;FREQ_HET=0.0002910078572121447;FREQ_HOMALT=0.0;FREQ_HOMREF=0.9997089921427879;N_BI_GENOS=10309;Southeast_Han_AN=1908;Southeast_Han_AC=1;S
chr5	13956282	.	T	<INS:ME:LINE1>	.	PASS	SVTYPE=INS:ME:LINE1;END=13956299;SVLEN=1528;AN=20618;AC=2;AF=9.700261907071492e-05;N_HET=2;N_HOMALT=0;N_HOMREF=10307;FREQ_HET=0.00019400523814142983;FREQ_HOMALT=0.0;FREQ_HOMREF=0.9998059947618586;N_BI_GENOS=10309;South_Han_AN=1496;South_Han_AC=1;South_Han
chr5	14237963	.	A	<INS:ME:LINE1>	.	PASS	SVTYPE=INS:ME:LINE1;END=14237978;SVLEN=103;AN=20618;AC=5;AF=0.0002425065476767878728;N_HET=5;N_HOMALT=0;N_HOMREF=10304;FREQ_HET=0.0004850130953535357455;FREQ_HOMALT=0.0;FREQ_HOMREF=0.9995149869046465;N_BI_GENOS=10309;South_Han_AN=1496;South_Han_AC=2;South_Han
chr5	14585384	.	C	<INS:ME:LINE1>	.	PASS	SVTYPE=INS:ME:LINE1;END=14585400;SVLEN=5998;AN=20618;AC=12;AF=0.0005820157144242894;N_HET=12;N_HOMALT=0;N_HOMREF=10297;FREQ_HET=0.0011640314288485789;FREQ_HOMALT=0.0;FREQ_HOMREF=0.9988359685711514;N_BI_GENOS=10309;South_Han_AN=1496;South_Han_AC=1;South_Ha
chr5	15020795	.	T	<INS:ME:LINE1>	.	PASS	SVTYPE=INS:ME:LINE1;END=15020805;SVLEN=2237;AN=20618;AC=3;AF=0.00014550392860607236;N_HET=3;N_HOMALT=0;N_HOMREF=10306;FREQ_HET=0.0002910078572121447;FREQ_HOMALT=0.0;FREQ_HOMREF=0.9997089921427879;N_BI_GENOS=10309;North_Han_AN=2272;North_Han_AC=2;North_Han
chr5	15060631	.	A	<INS:ME:LINE1>	.	PASS	SVTYPE=INS:ME:LINE1;END=15060648;SVLEN=6021;AN=20618;AC=1;AF=4.850130953535746e-05;N_HET=1;N_HOMALT=0;N_HOMREF=10308;FREQ_HET=9.700261907071492e-05;FREQ_HOMALT=0.0;FREQ_HOMREF=0.9999029973809292;N_BI_GENOS=10309;Lingnan_Han_AN=1888;Lingnan_Han_AC=1;Lingna
chr5	15127318	.	A	<INS:ME:LINE1>	.	PASS	SVTYPE=INS:ME:LINE1;END=15127344;SVLEN=840;AN=20618;AC=1;AF=4.850130953535746e-05;N_HET=1;N_HOMALT=0;N_HOMREF=10308;FREQ_HET=9.700261907071492e-05;FREQ_HOMALT=0.0;FREQ_HOMREF=0.9999029973809292;N_BI_GENOS=10309;East_Han_AN=6554;East_Han_AC=1;East_Han_AF=0
chr5	15155772	.	G	<INS:ME:LINE1>	.	PASS	SVTYPE=INS:ME:LINE1;END=15155780;SVLEN=1991;AN=20618;AC=1;AF=4.850130953535746e-05;N_HET=1;N_HOMALT=0;N_HOMREF=10308;FREQ_HET=9.700261907071492e-05;FREQ_HOMALT=0.0;FREQ_HOMREF=0.9999029973809292;N_BI_GENOS=10309;Central_Han_AN=2128;Central_Han_AC=1;Centra
chr5	15186097	.	A	<INS:ME:LINE1>	.	PASS	SVTYPE=INS:ME:LINE1;END=15186112;SVLEN=5999;AN=20618;AC=1;AF=4.850130953535746e-05;N_HET=1;N_HOMALT=0;N_HOMREF=10308;FREQ_HET=9.700261907071492e-05;FREQ_HOMALT=0.0;FREQ_HOMREF=0.9999029973809292;N_BI_GENOS=10309;Lingnan_Han_AN=1888;Lingnan_Han_AC=1;Lingna
chr5	15571442	.	A	<INS:ME:LINE1>	.	PASS	SVTYPE=INS:ME:LINE1;END=15571454;SVLEN=6002;AN=20618;AC=2;AF=9.700261907071492e-05;N_HET=2;N_HOMALT=0;N_HOMREF=10307;FREQ_HET=0.00019400523814142983;FREQ_HOMALT=0.0;FREQ_HOMREF=0.9998059947618586;N_BI_GENOS=10309;Southeast_Han_AN=1908;Southeast_Han_AC=2;S
chr5	16008482	.	T	<INS:ME:LINE1>	.	PASS	SVTYPE=INS:ME:LINE1;END=16008499;SVLEN=329;AN=20618;AC=75;AF=0.0036375982151518093;N_HET=75;N_HOMALT=0;N_HOMREF=10234;FREQ_HET=0.007275196430303618;FREQ_HOMALT=0.0;FREQ_HOMREF=0.9927248035696964;N_BI_GENOS=10309;South_Han_AN=1496;South_Han_AC=6;South_Han
chr5	16035568	.	A	<INS:ME:LINE1>	.	PASS	SVTYPE=INS:ME:LINE1;END=16035585;SVLEN=404;AN=20618;AC=1;AF=4.850130953535746e-05;N_HET=1;N_HOMALT=0;N_HOMREF=10308;FREQ_HET=9.700261907071492e-05;FREQ_HOMALT=0.0;FREQ_HOMREF=0.9999029973809292;N_BI_GENOS=10309;North_Han_AN=2272;North_Han_AC=1;North_Han_A
chr5	16081285	.	C	<INS:ME:LINE1>	.	PASS	SVTYPE=INS:ME:LINE1;END=16081303;SVLEN=1345;AN=20618;AC=5;AF=0.0002425065476767878728;N_HET=5;N_HOMALT=0;N_HOMREF=10304;FREQ_HET=0.0004850130953535357455;FREQ_HOMALT=0.0;FREQ_HOMREF=0.9995149869046465;N_BI_GENOS=10309;Lingnan_Han_AN=1888;Lingnan_Han_AC=1;Ling
chr5	16091558	.	T	<INS:ME:LINE1>	.	PASS	SVTYPE=INS:ME:LINE1;END=16091572;SVLEN=382;AN=20618;AC=1;AF=4.850130953535746e-05;N_HET=1;N_HOMALT=0;N_HOMREF=10308;FREQ_HET=9.700261907071492e-05;FREQ_HOMALT=0.0;FREQ_HOMREF=0.9999029973809292;N_BI_GENOS=10309;Lingnan_Han_AN=1888;Lingnan_Han_AC=1;Lingnan
chr5	16139855	.	T	<INS:ME:LINE1>	.	PASS	SVTYPE=INS:ME:LINE1;END=16139865;SVLEN=1034;AN=20618;AC=10;AF=0.00048501309535357455;N_HET=10;N_HOMALT=0;N_HOMREF=10299;FREQ_HET=0.0009700261907071491;FREQ_HOMALT=0.0;FREQ_HOMREF=0.9990299738092928;N_BI_GENOS=10309;North_Han_AN=2272;North_Han_AC=1;North_H
chr5	16464186	.	G	<INS:ME:LINE1>	.	PASS	SVTYPE=INS:ME:LINE1;END=16464200;SVLEN=5997;AN=20618;AC=106;AF=0.00514113881074789;N_HET=106;N_HOMALT=0;N_HOMREF=10203;FREQ_HET=0.01028227762149578;FREQ_HOMALT=0.0;FREQ_HOMREF=0.9897177223785042;N_BI_GENOS=10309;South_Han_AN=1496;South_Han_AC=8;South_Han_
chr5	16656973	.	A	<INS:ME:LINE1>	.	PASS	SVTYPE=INS:ME:LINE1;END=16656978;SVLEN=1641;AN=20618;AC=1;AF=4.850130953535746e-05;N_HET=1;N_HOMALT=0;N_HOMREF=10308;FREQ_HET=9.700261907071492e-05;FREQ_HOMALT=0.0;FREQ_HOMREF=0.9999029973809292;N_BI_GENOS=10309;Southeast_Han_AN=1908;Southeast_Han_AC=1;So
chr5	16882901	.	T	<INS:ME:LINE1>	.	PASS	SVTYPE=INS:ME:LINE1;END=16882908;SVLEN=5999;AN=20618;AC=1394;AF=0.06761082549228829;N_HET=1068;N_HOMALT=163;N_HOMREF=9078;FREQ_HET=0.10359879716752353;FREQ_HOMALT=0.015811426908526532;FREQ_HOMREF=0.8805897759239499;N_BI_GENOS=10309;South_Han_AN=1496;South
chr5	17712496	.	A	<INS:ME:LINE1>	.	PASS	SVTYPE=INS:ME:LINE1;END=17712496;SVLEN=478;AN=20618;AC=384;AF=0.018624502861577262;N_HET=356;N_HOMALT=14;N_HOMREF=9939;FREQ_HET=0.03453293238917451;FREQ_HOMALT=0.0013580366669900088;FREQ_HOMREF=0.9641090309438355;N_BI_GENOS=10309;South_Han_AN=1496;South_H
chr5	17712708	.	A	<INS:ME:LINE1>	.	PASS	SVTYPE=INS:ME:LINE1;END=17712708;SVLEN=478;AN=20618;AC=1;AF=4.850130953535746e-05;N_HET=1;N_HOMALT=0;N_HOMREF=10308;FREQ_HET=9.700261907071492e-05;FREQ_HOMALT=0.0;FREQ_HOMREF=0.9999029973809292;N_BI_GENOS=10309;East_Han_AN=6554;East_Han_AC=1;East_Han_AF=0
chr5	17713953	.	G	<INS:ME:LINE1>	.	PASS	SVTYPE=INS:ME:LINE1;END=17713968;SVLEN=396;AN=20618;AC=1;AF=4.850130953535746e-05;N_HET=1;N_HOMALT=0;N_HOMREF=10308;FREQ_HET=9.700261907071492e-05;FREQ_HOMALT=0.0;FREQ_HOMREF=0.9999029973809292;N_BI_GENOS=10309;North_Han_AN=2272;North_Han_AC=1;North_Han_A
chr5	17911420	.	A	<INS:ME:LINE1>	.	PASS	SVTYPE=INS:ME:LINE1;END=17911438;SVLEN=5997;AN=20618;AC=2;AF=9.700261907071492e-05;N_HET=2;N_HOMALT=0;N_HOMREF=10307;FREQ_HET=0.00019400523814142983;FREQ_HOMALT=0.0;FREQ_HOMREF=0.9998059947618586;N_BI_GENOS=10309;East_Han_AN=6554;East_Han_AC=2;East_Han_AF
chr5	17935215	.	T	<INS:ME:LINE1>	.	PASS	SVTYPE=INS:ME:LINE1;END=17935231;SVLEN=3417;AN=20618;AC=1;AF=4.850130953535746e-05;N_HET=1;N_HOMALT=0;N_HOMREF=10308;FREQ_HET=9.700261907071492e-05;FREQ_HOMALT=0.0;FREQ_HOMREF=0.9999029973809292;N_BI_GENOS=10309;East_Han_AN=6554;East_Han_AC=1;East_Han_AF=
chr5	17998916	.	T	<INS:ME:LINE1>	.	PASS	SVTYPE=INS:ME:LINE1;END=17998932;SVLEN=811;AN=20618;AC=1;AF=4.850130953535746e-05;N_HET=1;N_HOMALT=0;N_HOMREF=10308;FREQ_HET=9.700261907071492e-05;FREQ_HOMALT=0.0;FREQ_HOMREF=0.9999029973809292;N_BI_GENOS=10309;Central_Han_AN=2128;Central_Han_AC=1;Central
chr5	18051614	.	C	<INS:ME:LINE1>	.	PASS	SVTYPE=INS:ME:LINE1;END=18051629;SVLEN=937;AN=20618;AC=1;AF=4.850130953535746e-05;N_HET=1;N_HOMALT=0;N_HOMREF=10308;FREQ_HET=9.700261907071492e-05;FREQ_HOMALT=0.0;FREQ_HOMREF=0.9999029973809292;N_BI_GENOS=10309;East_Han_AN=6554;East_Han_AC=1;East_Han_AF=0
chr5	18187490	.	T	<INS:ME:LINE1>	.	PASS	SVTYPE=INS:ME:LINE1;END=18187490;SVLEN=927;AN=20618;AC=2;AF=9.700261907071492e-05;N_HET=2;N_HOMALT=0;N_HOMREF=10307;FREQ_HET=0.00019400523814142983;FREQ_HOMALT=0.0;FREQ_HOMREF=0.9998059947618586;N_BI_GENOS=10309;East_Han_AN=6554;East_Han_AC=2;East_Han_AF=
chr5	18188543	.	T	<INS:ME:LINE1>	.	PASS	SVTYPE=INS:ME:LINE1;END=18188557;SVLEN=2533;AN=20618;AC=1;AF=4.850130953535746e-05;N_HET=1;N_HOMALT=0;N_HOMREF=10308;FREQ_HET=9.700261907071492e-05;FREQ_HOMALT=0.0;FREQ_HOMREF=0.9999029973809292;N_BI_GENOS=10309;Tibetan_AN=414;Tibetan_AC=1;Tibetan_AF=0.00
chr5	18650008	.	A	<INS:ME:LINE1>	.	PASS	SVTYPE=INS:ME:LINE1;END=18650027;SVLEN=1128;AN=20618;AC=11;AF=0.000533514404888932;N_HET=9;N_HOMALT=1;N_HOMREF=10299;FREQ_HET=0.0008730235716364342;FREQ_HOMALT=9.700261907071492e-05;FREQ_HOMREF=0.9990299738092928;N_BI_GENOS=10309;South_Han_AN=1496;South_H
chr5	18673995	.	T	<INS:ME:LINE1>	.	PASS	SVTYPE=INS:ME:LINE1;END=18673995;SVLEN=0;AN=20618;AC=13;AF=0.0006305170239596469;N_HET=1;N_HOMALT=6;N_HOMREF=10302;FREQ_HET=9.700261907071492e-05;FREQ_HOMALT=0.0005820157144242894;FREQ_HOMREF=0.999320981666505;N_BI_GENOS=10309;South_Han_AN=1496;South_Han_
chr5	19118457	.	A	<INS:ME:LINE1>	.	PASS	SVTYPE=INS:ME:LINE1;END=19118457;SVLEN=750;AN=20618;AC=1;AF=4.850130953535746e-05;N_HET=1;N_HOMALT=0;N_HOMREF=10308;FREQ_HET=9.700261907071492e-05;FREQ_HOMALT=0.0;FREQ_HOMREF=0.9999029973809292;N_BI_GENOS=10309;East_Han_AN=6554;East_Han_AC=1;East_Han_AF=0
chr5	19179891	.	A	<INS:ME:LINE1>	.	PASS	SVTYPE=INS:ME:LINE1;END=19179907;SVLEN=1058;AN=20618;AC=1;AF=4.850130953535746e-05;N_HET=1;N_HOMALT=0;N_HOMREF=10308;FREQ_HET=9.700261907071492e-05;FREQ_HOMALT=0.0;FREQ_HOMREF=0.9999029973809292;N_BI_GENOS=10309;Southeast_Han_AN=1908;Southeast_Han_AC=1;So
chr5	19356923	.	A	<INS:ME:LINE1>	.	PASS	SVTYPE=INS:ME:LINE1;END=19356923;SVLEN=105;AN=20618;AC=15;AF=0.0007275196430303619;N_HET=15;N_HOMALT=0;N_HOMREF=10294;FREQ_HET=0.0014550392860607237;FREQ_HOMALT=0.0;FREQ_HOMREF=0.9985449607139393;N_BI_GENOS=10309;South_Han_AN=1496;South_Han_AC=1;South_Han
chr5	19429777	.	T	<INS:ME:LINE1>	.	PASS	SVTYPE=INS:ME:LINE1;END=19429787;SVLEN=871;AN=20618;AC=2;AF=9.700261907071492e-05;N_HET=2;N_HOMALT=0;N_HOMREF=10307;FREQ_HET=0.00019400523814142983;FREQ_HOMALT=0.0;FREQ_HOMREF=0.9998059947618586;N_BI_GENOS=10309;North_Han_AN=2272;North_Han_AC=2;North_Han_
chr5	19438218	.	C	<INS:ME:LINE1>	.	PASS	SVTYPE=INS:ME:LINE1;END=19438233;SVLEN=251;AN=20618;AC=1;AF=4.850130953535746e-05;N_HET=1;N_HOMALT=0;N_HOMREF=10308;FREQ_HET=9.700261907071492e-05;FREQ_HOMALT=0.0;FREQ_HOMREF=0.9999029973809292;N_BI_GENOS=10309;Manchu_AN=416;Manchu_AC=1;Manchu_AF=0.002403
chr5	19574628	.	T	<INS:ME:LINE1>	.	PASS	SVTYPE=INS:ME:LINE1;END=19574646;SVLEN=1124;AN=20618;AC=22;AF=0.0010670288097777864;N_HET=22;N_HOMALT=0;N_HOMREF=10287;FREQ_HET=0.002134057619555728;FREQ_HOMALT=0.0;FREQ_HOMREF=0.9978659423804442;N_BI_GENOS=10309;South_Han_AN=1496;South_Han_AC=3;South_Han_
chr5	19678829	.	G	<INS:ME:LINE1>	.	PASS	SVTYPE=INS:ME:LINE1;END=19678829;SVLEN=851;AN=20618;AC=1;AF=4.850130953535746e-05;N_HET=1;N_HOMALT=0;N_HOMREF=10308;FREQ_HET=9.700261907071492e-05;FREQ_HOMALT=0.0;FREQ_HOMREF=0.9999029973809292;N_BI_GENOS=10309;East_Han_AN=6554;East_Han_AC=1;East_Han_AF=0
chr5	19680739	.	A	<INS:ME:LINE1>	.	PASS	SVTYPE=INS:ME:LINE1;END=19680739;SVLEN=908;AN=20618;AC=2;AF=9.700261907071492e-05;N_HET=2;N_HOMALT=0;N_HOMREF=10307;FREQ_HET=0.00019400523814142983;FREQ_HOMALT=0.0;FREQ_HOMREF=0.9998059947618586;N_BI_GENOS=10309;East_Han_AN=6554;East_Han_AC=2;East_Han_AF=
chr5	19966121	.	A	<INS:ME:LINE1>	.	PASS	SVTYPE=INS:ME:LINE1;END=19966135;SVLEN=414;AN=20618;AC=1;AF=4.850130953535746e-05;N_HET=1;N_HOMALT=0;N_HOMREF=10308;FREQ_HET=9.700261907071492e-05;FREQ_HOMALT=0.0;FREQ_HOMREF=0.9999029973809292;N_BI_GENOS=10309;Lingnan_Han_AN=1888;Lingnan_Han_AC=1;Lingnan
chr5	20090534	.	A	<INS:ME:LINE1>	.	PASS	SVTYPE=INS:ME:LINE1;END=20090548;SVLEN=418;AN=20618;AC=51;AF=0.0024735667863032304;N_HET=43;N_HOMALT=4;N_HOMREF=10262;FREQ_HET=0.004171126200407141;FREQ_HOMALT=0.00038801047628285966;FREQ_HOMREF=0.9954408769036764;N_BI_GENOS=10309;South_Han_AN=1496;South_
chr5	20174697	.	G	<INS:ME:LINE1>	.	PASS	SVTYPE=INS:ME:LINE1;END=20174714;SVLEN=2358;AN=20618;AC=1;AF=4.850130953535746e-05;N_HET=1;N_HOMALT=0;N_HOMREF=10308;FREQ_HET=9.700261907071492e-05;FREQ_HOMALT=0.0;FREQ_HOMREF=0.9999029973809292;N_BI_GENOS=10309;Lingnan_Han_AN=1888;Lingnan_Han_AC=1;Lingna
chr5	20358719	.	T	<INS:ME:LINE1>	.	PASS	SVTYPE=INS:ME:LINE1;END=20358736;SVLEN=953;AN=20618;AC=2;AF=9.700261907071492e-05;N_HET=2;N_HOMALT=0;N_HOMREF=10307;FREQ_HET=0.00019400523814142983;FREQ_HOMALT=0.0;FREQ_HOMREF=0.9998059947618586;N_BI_GENOS=10309;Northwest_Han_AN=1296;Northwest_Han_AC=1;No
chr5	20486506	.	T	<INS:ME:LINE1>	.	PASS	SVTYPE=INS:ME:LINE1;END=20486520;SVLEN=188;AN=20618;AC=11;AF=0.000533514404888932;N_HET=9;N_HOMALT=1;N_HOMREF=10299;FREQ_HET=0.0008730235716364342;FREQ_HOMALT=9.700261907071492e-05;FREQ_HOMREF=0.9990299738092928;N_BI_GENOS=10309;Lingnan_Han_AN=1888;Lingna
chr5	20511206	.	A	<INS:ME:LINE1>	.	PASS	SVTYPE=INS:ME:LINE1;END=20511221;SVLEN=537;AN=20618;AC=1;AF=4.850130953535746e-05;N_HET=1;N_HOMALT=0;N_HOMREF=10308;FREQ_HET=9.700261907071492e-05;FREQ_HOMALT=0.0;FREQ_HOMREF=0.9999029973809292;N_BI_GENOS=10309;Southeast_Han_AN=1908;Southeast_Han_AC=1;Sou
chr5	20534238	.	A	<INS:ME:LINE1>	.	PASS	SVTYPE=INS:ME:LINE1;END=20534238;SVLEN=970;AN=20618;AC=2;AF=9.700261907071492e-05;N_HET=2;N_HOMALT=0;N_HOMREF=10307;FREQ_HET=0.00019400523814142983;FREQ_HOMALT=0.0;FREQ_HOMREF=0.9998059947618586;N_BI_GENOS=10309;East_Han_AN=6554;East_Han_AC=1;East_Han_AF=
chr5	20705395	.	G	<INS:ME:LINE1>	.	PASS	SVTYPE=INS:ME:LINE1;END=20705408;SVLEN=5995;AN=20618;AC=1;AF=4.850130953535746e-05;N_HET=1;N_HOMALT=0;N_HOMREF=10308;FREQ_HET=9.700261907071492e-05;FREQ_HOMALT=0.0;FREQ_HOMREF=0.9999029973809292;N_BI_GENOS=10309;East_Han_AN=6554;East_Han_AC=1;East_Han_AF=
chr5	20739162	.	T	<INS:ME:LINE1>	.	PASS	SVTYPE=INS:ME:LINE1;END=20739171;SVLEN=512;AN=20618;AC=1;AF=4.850130953535746e-05;N_HET=1;N_HOMALT=0;N_HOMREF=10308;FREQ_HET=9.700261907071492e-05;FREQ_HOMALT=0.0;FREQ_HOMREF=0.9999029973809292;N_BI_GENOS=10309;Lingnan_Han_AN=1888;Lingnan_Han_AC=1;Lingnan
chr5	21207604	.	A	<INS:ME:LINE1>	.	PASS	SVTYPE=INS:ME:LINE1;END=21207623;SVLEN=5999;AN=20618;AC=4304;AF=0.20874963624017848;N_HET=2512;N_HOMALT=896;N_HOMREF=6901;FREQ_HET=0.24367057910563586;FREQ_HOMALT=0.08691434668736056;FREQ_HOMREF=0.6694150742070036;N_BI_GENOS=10309;South_Han_AN=1496;South_
chr5	21207834	.	G	<INS:ME:LINE1>	.	PASS	SVTYPE=INS:ME:LINE1;END=21207834;SVLEN=0;AN=20618;AC=1;AF=4.850130953535746e-05;N_HET=1;N_HOMALT=0;N_HOMREF=10308;FREQ_HET=9.700261907071492e-05;FREQ_HOMALT=0.0;FREQ_HOMREF=0.9999029973809292;N_BI_GENOS=10309;East_Han_AN=6554;East_Han_AC=1;East_Han_AF=0.0
chr5	21526219	.	A	<INS:ME:LINE1>	.	PASS	SVTYPE=INS:ME:LINE1;END=21526232;SVLEN=630;AN=20618;AC=1;AF=4.850130953535746e-05;N_HET=1;N_HOMALT=0;N_HOMREF=10308;FREQ_HET=9.700261907071492e-05;FREQ_HOMALT=0.0;FREQ_HOMREF=0.9999029973809292;N_BI_GENOS=10309;Tibetan_AN=414;Tibetan_AC=1;Tibetan_AF=0.002
chr5	21689170	.	A	<INS:ME:LINE1>	.	PASS	SVTYPE=INS:ME:LINE1;END=21689186;SVLEN=984;AN=20618;AC=1;AF=4.850130953535746e-05;N_HET=1;N_HOMALT=0;N_HOMREF=10308;FREQ_HET=9.700261907071492e-05;FREQ_HOMALT=0.0;FREQ_HOMREF=0.9999029973809292;N_BI_GENOS=10309;Northwest_Han_AN=1296;Northwest_Han_AC=1;Nor
chr5	21715019	.	T	<INS:ME:LINE1>	.	PASS	SVTYPE=INS:ME:LINE1;END=21715032;SVLEN=62;AN=20618;AC=57;AF=0.0027645746435153748;N_HET=57;N_HOMALT=0;N_HOMREF=10252;FREQ_HET=0.005529149287030749 6;FREQ_HOMALT=0.0;FREQ_HOMREF=0.9944708507129693;N_BI_GENOS=10309;South_Han_AN=1496;South_Han_AC=9;South_Han_
chr5	21735377	.	A	<INS:ME:LINE1>	.	PASS	SVTYPE=INS:ME:LINE1;END=21735390;SVLEN=1503;AN=20618;AC=1;AF=4.850130953535746e-05;N_HET=1;N_HOMALT=0;N_HOMREF=10308;FREQ_HET=9.700261907071492e-05;FREQ_HOMALT=0.0;FREQ_HOMREF=0.9999029973809292;N_BI_GENOS=10309;North_Han_AN=2272;North_Han_AC=1;North_Han_
chr5	21899726	.	A	<INS:ME:LINE1>	.	PASS	SVTYPE=INS:ME:LINE1;END=21899736;SVLEN=5999;AN=20618;AC=123;AF=0.005965661072848967;N_HET=107;N_HOMALT=8;N_HOMREF=10194;FREQ_HET=0.010379280240566495;FREQ_HOMALT=0.0007760209525657193;FREQ_HOMREF=0.9888446988068678;N_BI_GENOS=10309;South_Han_AN=1496;South
chr5	22284834	.	A	<INS:ME:LINE1>	.	PASS	SVTYPE=INS:ME:LINE1;END=22284848;SVLEN=623;AN=20618;AC=7;AF=0.0003395091667475022;N_HET=7;N_HOMALT=0;N_HOMREF=10302;FREQ_HET=0.0006790183334950044;FREQ_HOMALT=0.0;FREQ_HOMREF=0.999320981666505;N_BI_GENOS=10309;South_Han_AN=1496;South_Han_AC=1;South_Han_AF
chr5	22313658	.	A	<INS:ME:LINE1>	.	PASS	SVTYPE=INS:ME:LINE1;END=22313673;SVLEN=1751;AN=20618;AC=1;AF=4.850130953535746e-05;N_HET=1;N_HOMALT=0;N_HOMREF=10308;FREQ_HET=9.700261907071492e-05;FREQ_HOMALT=0.0;FREQ_HOMREF=0.9999029973809292;N_BI_GENOS=10309;North_Han_AN=2272;North_Han_AC=1;North_Han_
chr5	22389565	.	A	<INS:ME:LINE1>	.	PASS	SVTYPE=INS:ME:LINE1;END=22389579;SVLEN=1158;AN=20618;AC=2;AF=9.700261907071492e-05;N_HET=2;N_HOMALT=0;N_HOMREF=10307;FREQ_HET=0.00019400523814142983;FREQ_HOMALT=0.0;FREQ_HOMREF=0.9998059947618586;N_BI_GENOS=10309;Yi_AN=422;Yi_AC=2;Yi_AF=0.0047393364928909
chr5	22391843	.	A	<INS:ME:LINE1>	.	PASS	SVTYPE=INS:ME:LINE1;END=22391856;SVLEN=589;AN=20618;AC=1;AF=4.850130953535746e-05;N_HET=1;N_HOMALT=0;N_HOMREF=10308;FREQ_HET=9.700261907071492e-05;FREQ_HOMALT=0.0;FREQ_HOMREF=0.9999029973809292;N_BI_GENOS=10309;Central_Han_AN=2128;Central_Han_AC=1;Central
chr5	22687310	.	A	<INS:ME:LINE1>	.	PASS	SVTYPE=INS:ME:LINE1;END=22687320;SVLEN=441;AN=20618;AC=3;AF=0.00014550392860607236;N_HET=3;N_HOMALT=0;N_HOMREF=10306;FREQ_HET=0.0002910078572121447;FREQ_HOMALT=0.0;FREQ_HOMREF=0.9997089921427879;N_BI_GENOS=10309;North_Han_AN=2272;North_Han_AC=2;North_Han_
chr5	22860973	.	A	<INS:ME:LINE1>	.	PASS	SVTYPE=INS:ME:LINE1;END=22860977;SVLEN=63;AN=20618;AC=1;AF=4.850130953535746e-05;N_HET=1;N_HOMALT=0;N_HOMREF=10308;FREQ_HET=9.700261907071492e-05;FREQ_HOMALT=0.0;FREQ_HOMREF=0.9999029973809292;N_BI_GENOS=10309;East_Han_AN=6554;East_Han_AC=1;East_Han_AF=0.
chr5	22863853	.	G	<INS:ME:LINE1>	.	PASS	SVTYPE=INS:ME:LINE1;END=22863869;SVLEN=634;AN=20618;AC=1;AF=4.850130953535746e-05;N_HET=1;N_HOMALT=0;N_HOMREF=10308;FREQ_HET=9.700261907071492e-05;FREQ_HOMALT=0.0;FREQ_HOMREF=0.9999029973809292;N_BI_GENOS=10309;Hui_AN=444;Hui_AC=1;Hui_AF=0.002252252252252
chr5	22889016	.	T	<INS:ME:LINE1>	.	PASS	SVTYPE=INS:ME:LINE1;END=22889029;SVLEN=511;AN=20618;AC=50;AF=0.0024250654767678727;N_HET=50;N_HOMALT=0;N_HOMREF=10259;FREQ_HET=0.004850130953535745;FREQ_HOMALT=0.0;FREQ_HOMREF=0.9951498690464643;N_BI_GENOS=10309;South_Han_AN=1496;South_Han_AC=2;South_Han_
chr5	22892897	.	T	<INS:ME:LINE1>	.	PASS	SVTYPE=INS:ME:LINE1;END=22892918;SVLEN=2955;AN=20618;AC=2;AF=9.700261907071492e-05;N_HET=2;N_HOMALT=0;N_HOMREF=10307;FREQ_HET=0.00019400523814142983;FREQ_HOMALT=0.0;FREQ_HOMREF=0.9998059947618586;N_BI_GENOS=10309;Lingnan_Han_AN=1888;Lingnan_Han_AC=1;Lingn
chr5	23022150	.	T	<INS:ME:LINE1>	.	PASS	SVTYPE=INS:ME:LINE1;END=23022151;SVLEN=1764;AN=20618;AC=1;AF=4.850130953535746e-05;N_HET=1;N_HOMALT=0;N_HOMREF=10308;FREQ_HET=9.700261907071492e-05;FREQ_HOMALT=0.0;FREQ_HOMREF=0.9999029973809292;N_BI_GENOS=10309;Tibetan_AN=414;Tibetan_AC=1;Tibetan_AF=0.00
chr5	23057581	.	T	<INS:ME:LINE1>	.	PASS	SVTYPE=INS:ME:LINE1;END=23057594;SVLEN=5999;AN=20618;AC=1;AF=4.850130953535746e-05;N_HET=1;N_HOMALT=0;N_HOMREF=10308;FREQ_HET=9.700261907071492e-05;FREQ_HOMALT=0.0;FREQ_HOMREF=0.9999029973809292;N_BI_GENOS=10309;Lingnan_Han_AN=1888;Lingnan_Han_AC=1;Lingna

chr5	23070163	.	G	<INS:ME:LINE1>	.	PASS	SVTYPE=INS:ME:LINE1;END=23070169;SVLEN=953;AN=20618;AC=4;AF=0.00019400523814142983;N_HET=4;N_HOMALT=0;N_HOMREF=10305;FREQ_HET=0.00038801047628285966;FREQ_HOMALT=0.0;FREQ_HOMREF=0.9996119895237171;N_BI_GENOS=10309;Northwest_Han_AN=1296;Northwest_Han_AC=2;N
chr5	23328858	.	A	<INS:ME:LINE1>	.	PASS	SVTYPE=INS:ME:LINE1;END=23328872;SVLEN=6003;AN=20618;AC=1;AF=4.850130953535746e-05;N_HET=1;N_HOMALT=0;N_HOMREF=10308;FREQ_HET=9.700261907071492e-05;FREQ_HOMALT=0.0;FREQ_HOMREF=0.9999029973809292;N_BI_GENOS=10309;Lingnan_Han_AN=1888;Lingnan_Han_AC=1;Lingna
chr5	23753793	.	T	<INS:ME:LINE1>	.	PASS	SVTYPE=INS:ME:LINE1;END=23753830;SVLEN=125;AN=20618;AC=3;AF=0.00014550392860607236;N_HET=3;N_HOMALT=0;N_HOMREF=10306;FREQ_HET=0.0002910078572121447;FREQ_HOMALT=0.0;FREQ_HOMREF=0.9997089921427879;N_BI_GENOS=10309;East_Han_AN=6554;East_Han_AC=3;East_Han_AF=
chr5	23774507	.	A	<INS:ME:LINE1>	.	PASS	SVTYPE=INS:ME:LINE1;END=23774517;SVLEN=80;AN=20618;AC=1;AF=4.850130953535746e-05;N_HET=1;N_HOMALT=0;N_HOMREF=10308;FREQ_HET=9.700261907071492e-05;FREQ_HOMALT=0.0;FREQ_HOMREF=0.9999029973809292;N_BI_GENOS=10309;East_Han_AN=6554;East_Han_AC=1;East_Han_AF=0.
chr5	23786366	.	G	<INS:ME:LINE1>	.	PASS	SVTYPE=INS:ME:LINE1;END=23786383;SVLEN=6019;AN=20618;AC=1;AF=4.850130953535746e-05;N_HET=1;N_HOMALT=0;N_HOMREF=10308;FREQ_HET=9.700261907071492e-05;FREQ_HOMALT=0.0;FREQ_HOMREF=0.9999029973809292;N_BI_GENOS=10309;North_Han_AN=2272;North_Han_AC=1;North_Han_
chr5	23837099	.	A	<INS:ME:LINE1>	.	PASS	SVTYPE=INS:ME:LINE1;END=23837099;SVLEN=569;AN=20618;AC=1;AF=4.850130953535746e-05;N_HET=1;N_HOMALT=0;N_HOMREF=10308;FREQ_HET=9.700261907071492e-05;FREQ_HOMALT=0.0;FREQ_HOMREF=0.9999029973809292;N_BI_GENOS=10309;East_Han_AN=6554;East_Han_AC=1;East_Han_AF=0
chr5	23872600	.	A	<INS:ME:LINE1>	.	PASS	SVTYPE=INS:ME:LINE1;END=23872600;SVLEN=310;AN=20618;AC=2;AF=9.700261907071492e-05;N_HET=2;N_HOMALT=0;N_HOMREF=10307;FREQ_HET=0.00019400523814142983;FREQ_HOMALT=0.0;FREQ_HOMREF=0.9998059947618586;N_BI_GENOS=10309;East_Han_AN=6554;East_Han_AC=2;East_Han_AF=
chr5	23891425	.	T	<INS:ME:LINE1>	.	PASS	SVTYPE=INS:ME:LINE1;END=23891425;SVLEN=1280;AN=20618;AC=1;AF=4.850130953535746e-05;N_HET=1;N_HOMALT=0;N_HOMREF=10308;FREQ_HET=9.700261907071492e-05;FREQ_HOMALT=0.0;FREQ_HOMREF=0.9999029973809292;N_BI_GENOS=10309;East_Han_AN=6554;East_Han_AC=1;East_Han_AF=
chr5	24008453	.	T	<INS:ME:LINE1>	.	PASS	SVTYPE=INS:ME:LINE1;END=24008453;SVLEN=12;AN=20618;AC=1;AF=4.850130953535746e-05;N_HET=1;N_HOMALT=0;N_HOMREF=10308;FREQ_HET=9.700261907071492e-05;FREQ_HOMALT=0.0;FREQ_HOMREF=0.9999029973809292;N_BI_GENOS=10309;East_Han_AN=6554;East_Han_AC=1;East_Han_AF=0.
chr5	24008902	.	A	<INS:ME:LINE1>	.	PASS	SVTYPE=INS:ME:LINE1;END=24008943;SVLEN=1475;AN=20618;AC=1;AF=4.850130953535746e-05;N_HET=1;N_HOMALT=0;N_HOMREF=10308;FREQ_HET=9.700261907071492e-05;FREQ_HOMALT=0.0;FREQ_HOMREF=0.9999029973809292;N_BI_GENOS=10309;East_Han_AN=6554;East_Han_AC=1;East_Han_AF=
chr5	24102069	.	T	<INS:ME:LINE1>	.	PASS	SVTYPE=INS:ME:LINE1;END=24102085;SVLEN=3170;AN=20618;AC=1;AF=4.850130953535746e-05;N_HET=1;N_HOMALT=0;N_HOMREF=10308;FREQ_HET=9.700261907071492e-05;FREQ_HOMALT=0.0;FREQ_HOMREF=0.9999029973809292;N_BI_GENOS=10309;Central_Han_AN=2128;Central_Han_AC=1;Centra
chr5	24175003	.	G	<INS:ME:LINE1>	.	PASS	SVTYPE=INS:ME:LINE1;END=24175017;SVLEN=6020;AN=20618;AC=1;AF=4.850130953535746e-05;N_HET=1;N_HOMALT=0;N_HOMREF=10308;FREQ_HET=9.700261907071492e-05;FREQ_HOMALT=0.0;FREQ_HOMREF=0.9999029973809292;N_BI_GENOS=10309;South_Han_AN=1496;South_Han_AC=1;South_Han_
chr5	24207710	.	A	<INS:ME:LINE1>	.	PASS	SVTYPE=INS:ME:LINE1;END=24207721;SVLEN=1176;AN=20618;AC=6;AF=0.0002910078572121447;N_HET=6;N_HOMALT=0;N_HOMREF=10303;FREQ_HET=0.0005820157144242894;FREQ_HOMALT=0.0;FREQ_HOMREF=0.9994179842855757;N_BI_GENOS=10309;Northwest_Han_AN=1296;Northwest_Han_AC=1;No
chr5	24287076	.	A	<INS:ME:LINE1>	.	PASS	SVTYPE=INS:ME:LINE1;END=24287091;SVLEN=1284;AN=20618;AC=3;AF=0.00014550392860607236;N_HET=3;N_HOMALT=0;N_HOMREF=10306;FREQ_HET=0.0002910078572121447;FREQ_HOMALT=0.0;FREQ_HOMREF=0.9997089921427879;N_BI_GENOS=10309;South_Han_AN=1496;South_Han_AC=1;South_Han
chr5	24427939	.	T	<INS:ME:LINE1>	.	PASS	SVTYPE=INS:ME:LINE1;END=24427955;SVLEN=707;AN=20618;AC=5;AF=0.00024250654767678728;N_HET=5;N_HOMALT=0;N_HOMREF=10304;FREQ_HET=0.00048501309535357455;FREQ_HOMALT=0.0;FREQ_HOMREF=0.9995149869046465;N_BI_GENOS=10309;Miao_AN=482;Miao_AC=5;Miao_AF=0.0103734439
chr5	24458029	.	C	<INS:ME:LINE1>	.	PASS	SVTYPE=INS:ME:LINE1;END=24458029;SVLEN=0;AN=20618;AC=1;AF=4.850130953535746e-05;N_HET=1;N_HOMALT=0;N_HOMREF=10308;FREQ_HET=9.700261907071492e-05;FREQ_HOMALT=0.0;FREQ_HOMREF=0.9999029973809292;N_BI_GENOS=10309;Central_Han_AN=2128;Central_Han_AC=1;Central_H
chr5	24522373	.	C	<INS:ME:LINE1>	.	PASS	SVTYPE=INS:ME:LINE1;END=24522389;SVLEN=791;AN=20618;AC=24;AF=0.0011640314288485789;N_HET=24;N_HOMALT=0;N_HOMREF=10285;FREQ_HET=0.0023280628576971578;FREQ_HOMALT=0.0;FREQ_HOMREF=0.9976719371423028;N_BI_GENOS=10309;South_Han_AN=1496;South_Han_AC=1;South_Han
chr5	24742965	.	T	<INS:ME:LINE1>	.	PASS	SVTYPE=INS:ME:LINE1;END=24742965;SVLEN=0;AN=20618;AC=1;AF=4.850130953535746e-05;N_HET=1;N_HOMALT=0;N_HOMREF=10308;FREQ_HET=9.700261907071492e-05;FREQ_HOMALT=0.0;FREQ_HOMREF=0.9999029973809292;N_BI_GENOS=10309;East_Han_AN=6554;East_Han_AC=1;East_Han_AF=0.0
chr5	24767708	.	C	<INS:ME:LINE1>	.	PASS	SVTYPE=INS:ME:LINE1;END=24767722;SVLEN=609;AN=20618;AC=161;AF=0.0078087108351925505;N_HET=155;N_HOMALT=3;N_HOMREF=10151;FREQ_HET=0.01503540595596081;FREQ_HOMALT=0.0002910078572121447;FREQ_HOMREF=0.9846735861868271;N_BI_GENOS=10309;South_Han_AN=1496;South_
chr5	24777200	.	A	<INS:ME:LINE1>	.	PASS	SVTYPE=INS:ME:LINE1;END=24777214;SVLEN=925;AN=20618;AC=4;AF=0.00019400523814142983;N_HET=4;N_HOMALT=0;N_HOMREF=10305;FREQ_HET=0.00038801047628285966;FREQ_HOMALT=0.0;FREQ_HOMREF=0.9996119895237171;N_BI_GENOS=10309;Central_Han_AN=2128;Central_Han_AC=2;Centr
chr5	24778643	.	T	<INS:ME:LINE1>	.	PASS	SVTYPE=INS:ME:LINE1;END=24778652;SVLEN=2275;AN=20618;AC=1;AF=4.850130953535746e-05;N_HET=1;N_HOMALT=0;N_HOMREF=10308;FREQ_HET=9.700261907071492e-05;FREQ_HOMALT=0.0;FREQ_HOMREF=0.9999029973809292;N_BI_GENOS=10309;East_Han_AN=6554;East_Han_AC=1;East_Han_AF=
chr5	24814905	.	A	<INS:ME:LINE1>	.	PASS	SVTYPE=INS:ME:LINE1;END=24814922;SVLEN=5999;AN=20618;AC=24;AF=0.0011640314288485789;N_HET=24;N_HOMALT=0;N_HOMREF=10285;FREQ_HET=0.0023280628576971578;FREQ_HOMALT=0.0;FREQ_HOMREF=0.9976719371423028;N_BI_GENOS=10309;South_Han_AN=1496;South_Han_AC=1;South_Ha
chr5	24994618	.	C	<INS:ME:LINE1>	.	PASS	SVTYPE=INS:ME:LINE1;END=24994618;SVLEN=380;AN=20618;AC=1;AF=4.850130953535746e-05;N_HET=1;N_HOMALT=0;N_HOMREF=10308;FREQ_HET=9.700261907071492e-05;FREQ_HOMALT=0.0;FREQ_HOMREF=0.9999029973809292;N_BI_GENOS=10309;East_Han_AN=6554;East_Han_AC=1;East_Han_AF=0
chr5	24996346	.	T	<INS:ME:LINE1>	.	PASS	SVTYPE=INS:ME:LINE1;END=24996346;SVLEN=453;AN=20618;AC=2;AF=9.700261907071492e-05;N_HET=0;N_HOMALT=1;N_HOMREF=10308;FREQ_HET=0.0;FREQ_HOMALT=9.700261907071492e-05;FREQ_HOMREF=0.9999029973809292;N_BI_GENOS=10309;East_Han_AN=6554;East_Han_AC=2;East_Han_AF=0
chr5	25172770	.	G	<INS:ME:LINE1>	.	PASS	SVTYPE=INS:ME:LINE1;END=25172778;SVLEN=809;AN=20618;AC=14;AF=0.0006790183334950044;N_HET=14;N_HOMALT=0;N_HOMREF=10295;FREQ_HET=0.0013580366669900088;FREQ_HOMALT=0.0;FREQ_HOMREF=0.99864196333301;N_BI_GENOS=10309;Northwest_Han_AN=1296;Northwest_Han_AC=1;Nor
chr5	25325190	.	T	<INS:ME:LINE1>	.	PASS	SVTYPE=INS:ME:LINE1;END=25325190;SVLEN=452;AN=20618;AC=1;AF=4.850130953535746e-05;N_HET=1;N_HOMALT=0;N_HOMREF=10308;FREQ_HET=9.700261907071492e-05;FREQ_HOMALT=0.0;FREQ_HOMREF=0.9999029973809292;N_BI_GENOS=10309;Southeast_Han_AN=1908;Southeast_Han_AC=1;Sou
chr5	25624396	.	A	<INS:ME:LINE1>	.	PASS	SVTYPE=INS:ME:LINE1;END=25624397;SVLEN=2545;AN=20618;AC=6;AF=0.0002910078572121447;N_HET=2;N_HOMALT=2;N_HOMREF=10305;FREQ_HET=0.00019400523814142983;FREQ_HOMALT=0.00019400523814142983;FREQ_HOMREF=0.9996119895237171;N_BI_GENOS=10309;East_Han_AN=6554;East_H
chr5	25681441	.	A	<INS:ME:LINE1>	.	PASS	SVTYPE=INS:ME:LINE1;END=25681456;SVLEN=1105;AN=20618;AC=4;AF=0.00019400523814142983;N_HET=2;N_HOMALT=1;N_HOMREF=10306;FREQ_HET=0.00019400523814142983;FREQ_HOMALT=9.700261907071492e-05;FREQ_HOMREF=0.9997089921427879;N_BI_GENOS=10309;Lingnan_Han_AN=1888;Lin
chr5	25708541	.	A	<INS:ME:LINE1>	.	PASS	SVTYPE=INS:ME:LINE1;END=25708556;SVLEN=5998;AN=20618;AC=11;AF=0.000533514404888932;N_HET=11;N_HOMALT=0;N_HOMREF=10298;FREQ_HET=0.001067028809777864;FREQ_HOMALT=0.0;FREQ_HOMREF=0.9989329711902222;N_BI_GENOS=10309;Lingnan_Han_AN=1888;Lingnan_Han_AC=3;Lingna
chr5	25990834	.	T	<INS:ME:LINE1>	.	PASS	SVTYPE=INS:ME:LINE1;END=25990847;SVLEN=1252;AN=20618;AC=2;AF=9.700261907071492e-05;N_HET=2;N_HOMALT=0;N_HOMREF=10307;FREQ_HET=0.00019400523814142983;FREQ_HOMALT=0.0;FREQ_HOMREF=0.9998059947618586;N_BI_GENOS=10309;East_Han_AN=6554;East_Han_AC=2;East_Han_AF
chr5	26093030	.	T	<INS:ME:LINE1>	.	PASS	SVTYPE=INS:ME:LINE1;END=26093046;SVLEN=833;AN=20618;AC=11142;AF=0.5404015908429528;N_HET=4618;N_HOMALT=3262;N_HOMREF=2429;FREQ_HET=0.4479580948685615;FREQ_HOMALT=0.31642254340867204;FREQ_HOMREF=0.2356193617227665;N_BI_GENOS=10309;South_Han_AN=1496;South_H
chr5	26139088	.	A	<INS:ME:LINE1>	.	PASS	SVTYPE=INS:ME:LINE1;END=26139100;SVLEN=158;AN=20618;AC=1;AF=4.850130953535746e-05;N_HET=1;N_HOMALT=0;N_HOMREF=10308;FREQ_HET=9.700261907071492e-05;FREQ_HOMALT=0.0;FREQ_HOMREF=0.9999029973809292;N_BI_GENOS=10309;Central_Han_AN=2128;Central_Han_AC=1;Central
chr5	26346910	.	A	<INS:ME:LINE1>	.	PASS	SVTYPE=INS:ME:LINE1;END=26346924;SVLEN=936;AN=20618;AC=26;AF=0.0012610340479192938;N_HET=22;N_HOMALT=2;N_HOMREF=10285;FREQ_HET=0.002134057619555728;FREQ_HOMALT=0.00019400523814142983;FREQ_HOMREF=0.9976719371423028;N_BI_GENOS=10309;South_Han_AN=1496;South_
chr5	27075895	.	A	<INS:ME:LINE1>	.	PASS	SVTYPE=INS:ME:LINE1;END=27075895;SVLEN=276;AN=20618;AC=1;AF=4.850130953535746e-05;N_HET=1;N_HOMALT=0;N_HOMREF=10308;FREQ_HET=9.700261907071492e-05;FREQ_HOMALT=0.0;FREQ_HOMREF=0.9999029973809292;N_BI_GENOS=10309;East_Han_AN=6554;East_Han_AC=1;East_Han_AF=0
chr5	27290387	.	A	<INS:ME:LINE1>	.	PASS	SVTYPE=INS:ME:LINE1;END=27290398;SVLEN=524;AN=20618;AC=1;AF=4.850130953535746e-05;N_HET=1;N_HOMALT=0;N_HOMREF=10308;FREQ_HET=9.700261907071492e-05;FREQ_HOMALT=0.0;FREQ_HOMREF=0.9999029973809292;N_BI_GENOS=10309;East_Han_AN=6554;East_Han_AC=1;East_Han_AF=0
chr5	27334257	.	A	<INS:ME:LINE1>	.	PASS	SVTYPE=INS:ME:LINE1;END=27334273;SVLEN=1114;AN=20618;AC=2;AF=9.700261907071492e-05;N_HET=2;N_HOMALT=0;N_HOMREF=10307;FREQ_HET=0.00019400523814142983;FREQ_HOMALT=0.0;FREQ_HOMREF=0.9998059947618586;N_BI_GENOS=10309;East_Han_AN=6554;East_Han_AC=1;East_Han_AF
chr5	27628192	.	G	<INS:ME:LINE1>	.	PASS	SVTYPE=INS:ME:LINE1;END=27628207;SVLEN=398;AN=20618;AC=1;AF=4.850130953535746e-05;N_HET=1;N_HOMALT=0;N_HOMREF=10308;FREQ_HET=9.700261907071492e-05;FREQ_HOMALT=0.0;FREQ_HOMREF=0.9999029973809292;N_BI_GENOS=10309;North_Han_AN=2272;North_Han_AC=1;North_Han_A
chr5	27752193	.	A	<INS:ME:LINE1>	.	PASS	SVTYPE=INS:ME:LINE1;END=27752193;SVLEN=3896;AN=20618;AC=2;AF=9.700261907071492e-05;N_HET=0;N_HOMALT=1;N_HOMREF=10308;FREQ_HET=0.0;FREQ_HOMALT=9.700261907071492e-05;FREQ_HOMREF=0.9999029973809292;N_BI_GENOS=10309;East_Han_AN=6554;East_Han_AC=2;East_Han_AF=
chr5	27770672	.	T	<INS:ME:LINE1>	.	PASS	SVTYPE=INS:ME:LINE1;END=27770673;SVLEN=417;AN=20618;AC=1;AF=4.850130953535746e-05;N_HET=1;N_HOMALT=0;N_HOMREF=10308;FREQ_HET=9.700261907071492e-05;FREQ_HOMALT=0.0;FREQ_HOMREF=0.9999029973809292;N_BI_GENOS=10309;East_Han_AN=6554;East_Han_AC=1;East_Han_AF=0
chr5	27787805	.	T	<INS:ME:LINE1>	.	PASS	SVTYPE=INS:ME:LINE1;END=27787805;SVLEN=755;AN=20618;AC=1;AF=4.850130953535746e-05;N_HET=1;N_HOMALT=0;N_HOMREF=10308;FREQ_HET=9.700261907071492e-05;FREQ_HOMALT=0.0;FREQ_HOMREF=0.9999029973809292;N_BI_GENOS=10309;Northwest_Han_AN=1296;Northwest_Han_AC=1;Nor
chr5	27798902	.	A	<INS:ME:LINE1>	.	PASS	SVTYPE=INS:ME:LINE1;END=27798917;SVLEN=3096;AN=20618;AC=1;AF=4.850130953535746e-05;N_HET=1;N_HOMALT=0;N_HOMREF=10308;FREQ_HET=9.700261907071492e-05;FREQ_HOMALT=0.0;FREQ_HOMREF=0.9999029973809292;N_BI_GENOS=10309;Tibetan_AN=414;Tibetan_AC=1;Tibetan_AF=0.00
chr5	27834285	.	T	<INS:ME:LINE1>	.	PASS	SVTYPE=INS:ME:LINE1;END=27834299;SVLEN=383;AN=20618;AC=1;AF=4.850130953535746e-05;N_HET=1;N_HOMALT=0;N_HOMREF=10308;FREQ_HET=9.700261907071492e-05;FREQ_HOMALT=0.0;FREQ_HOMREF=0.9999029973809292;N_BI_GENOS=10309;North_Han_AN=2272;North_Han_AC=1;North_Han_A
chr5	27848404	.	C	<INS:ME:LINE1>	.	PASS	SVTYPE=INS:ME:LINE1;END=27848404;SVLEN=146;AN=20618;AC=1;AF=4.850130953535746e-05;N_HET=1;N_HOMALT=0;N_HOMREF=10308;FREQ_HET=9.700261907071492e-05;FREQ_HOMALT=0.0;FREQ_HOMREF=0.9999029973809292;N_BI_GENOS=10309;East_Han_AN=6554;East_Han_AC=1;East_Han_AF=0
chr5	27893802	.	A	<INS:ME:LINE1>	.	PASS	SVTYPE=INS:ME:LINE1;END=27893812;SVLEN=676;AN=20618;AC=5;AF=0.00024250654767678728;N_HET=5;N_HOMALT=0;N_HOMREF=10304;FREQ_HET=0.00048501309535357455;FREQ_HOMALT=0.0;FREQ_HOMREF=0.9995149869046465;N_BI_GENOS=10309;South_Han_AN=1496;South_Han_AC=2;South_Han
chr5	28001482	.	T	<INS:ME:LINE1>	.	PASS	SVTYPE=INS:ME:LINE1;END=28001482;SVLEN=220;AN=20618;AC=1;AF=4.850130953535746e-05;N_HET=1;N_HOMALT=0;N_HOMREF=10308;FREQ_HET=9.700261907071492e-05;FREQ_HOMALT=0.0;FREQ_HOMREF=0.9999029973809292;N_BI_GENOS=10309;East_Han_AN=6554;East_Han_AC=1;East_Han_AF=0
chr5	28260775	.	A	<INS:ME:LINE1>	.	PASS	SVTYPE=INS:ME:LINE1;END=28260789;SVLEN=1270;AN=20618;AC=1;AF=4.850130953535746e-05;N_HET=1;N_HOMALT=0;N_HOMREF=10308;FREQ_HET=9.700261907071492e-05;FREQ_HOMALT=0.0;FREQ_HOMREF=0.9999029973809292;N_BI_GENOS=10309;East_Han_AN=6554;East_Han_AC=1;East_Han_AF=
chr5	28334240	.	G	<INS:ME:LINE1>	.	PASS	SVTYPE=INS:ME:LINE1;END=28334252;SVLEN=1550;AN=20618;AC=4;AF=0.00019400523814142983;N_HET=4;N_HOMALT=0;N_HOMREF=10305;FREQ_HET=0.00038801047628285966;FREQ_HOMALT=0.0;FREQ_HOMREF=0.9996119895237171;N_BI_GENOS=10309;East_Han_AN=6554;East_Han_AC=4;East_Han_A
chr5	28417636	.	A	<INS:ME:LINE1>	.	PASS	SVTYPE=INS:ME:LINE1;END=28417652;SVLEN=501;AN=20618;AC=1;AF=4.850130953535746e-05;N_HET=1;N_HOMALT=0;N_HOMREF=10308;FREQ_HET=9.700261907071492e-05;FREQ_HOMALT=0.0;FREQ_HOMREF=0.9999029973809292;N_BI_GENOS=10309;East_Han_AN=6554;East_Han_AC=1;East_Han_AF=0
chr5	28488221	.	T	<INS:ME:LINE1>	.	PASS	SVTYPE=INS:ME:LINE1;END=28488237;SVLEN=805;AN=20618;AC=1;AF=4.850130953535746e-05;N_HET=1;N_HOMALT=0;N_HOMREF=10308;FREQ_HET=9.700261907071492e-05;FREQ_HOMALT=0.0;FREQ_HOMREF=0.9999029973809292;N_BI_GENOS=10309;North_Han_AN=2272;North_Han_AC=1;North_Han_A
chr5	28562197	.	A	<INS:ME:LINE1>	.	PASS	SVTYPE=INS:ME:LINE1;END=28562208;SVLEN=522;AN=20618;AC=5;AF=0.00024250654767678728;N_HET=5;N_HOMALT=0;N_HOMREF=10304;FREQ_HET=0.00048501309535357455;FREQ_HOMALT=0.0;FREQ_HOMREF=0.9995149869046465;N_BI_GENOS=10309;South_Han_AN=1496;South_Han_AC=1;South_Han
chr5	28814106	.	T	<INS:ME:LINE1>	.	PASS	SVTYPE=INS:ME:LINE1;END=28814121;SVLEN=1741;AN=20618;AC=3;AF=0.00014550392860607236;N_HET=3;N_HOMALT=0;N_HOMREF=10306;FREQ_HET=0.0002910078572121447;FREQ_HOMALT=0.0;FREQ_HOMREF=0.9997089921427879;N_BI_GENOS=10309;Lingnan_Han_AN=1888;Lingnan_Han_AC=3;Lingn
chr5	29006374	.	A	<INS:ME:LINE1>	.	PASS	SVTYPE=INS:ME:LINE1;END=29006390;SVLEN=4535;AN=20618;AC=7;AF=0.0003395091667475022;N_HET=7;N_HOMALT=0;N_HOMREF=10302;FREQ_HET=0.0006790183334950044;FREQ_HOMALT=0.0;FREQ_HOMREF=0.999320981666505;N_BI_GENOS=10309;Lingnan_Han_AN=1888;Lingnan_Han_AC=1;Lingnan
chr5	29289979	.	A	<INS:ME:LINE1>	.	PASS	SVTYPE=INS:ME:LINE1;END=29289992;SVLEN=2261;AN=20618;AC=1;AF=4.850130953535746e-05;N_HET=1;N_HOMALT=0;N_HOMREF=10308;FREQ_HET=9.700261907071492e-05;FREQ_HOMALT=0.0;FREQ_HOMREF=0.9999029973809292;N_BI_GENOS=10309;Southeast_Han_AN=1908;Southeast_Han_AC=1;So
chr5	29343459	.	A	<INS:ME:LINE1>	.	PASS	SVTYPE=INS:ME:LINE1;END=29343475;SVLEN=1364;AN=20618;AC=1;AF=4.850130953535746e-05;N_HET=1;N_HOMALT=0;N_HOMREF=10308;FREQ_HET=9.700261907071492e-05;FREQ_HOMALT=0.0;FREQ_HOMREF=0.9999029973809292;N_BI_GENOS=10309;Yi_AN=422;Yi_AC=1;Yi_AF=0.002369668246444549
chr5	29514450	.	G	<INS:ME:LINE1>	.	PASS	SVTYPE=INS:ME:LINE1;END=29514464;SVLEN=5997;AN=20618;AC=1;AF=4.850130953535746e-05;N_HET=1;N_HOMALT=0;N_HOMREF=10308;FREQ_HET=9.700261907071492e-05;FREQ_HOMALT=0.0;FREQ_HOMREF=0.9999029973809292;N_BI_GENOS=10309;Northwest_Han_AN=1296;Northwest_Han_AC=1;No
chr5	29742876	.	A	<INS:ME:LINE1>	.	PASS	SVTYPE=INS:ME:LINE1;END=29742926;SVLEN=1350;AN=20618;AC=1;AF=4.850130953535746e-05;N_HET=1;N_HOMALT=0;N_HOMREF=10308;FREQ_HET=9.700261907071492e-05;FREQ_HOMALT=0.0;FREQ_HOMREF=0.9999029973809292;N_BI_GENOS=10309;East_Han_AN=6554;East_Han_AC=1;East_Han_AF=
chr5	29938066	.	A	<INS:ME:LINE1>	.	PASS	SVTYPE=INS:ME:LINE1;END=29938085;SVLEN=762;AN=20618;AC=1;AF=4.850130953535746e-05;N_HET=1;N_HOMALT=0;N_HOMREF=10308;FREQ_HET=9.700261907071492e-05;FREQ_HOMALT=0.0;FREQ_HOMREF=0.9999029973809292;N_BI_GENOS=10309;Central_Han_AN=2128;Central_Han_AC=1;Central
chr5	29971468	.	A	<INS:ME:LINE1>	.	PASS	SVTYPE=INS:ME:LINE1;END=29971486;SVLEN=3427;AN=20618;AC=1;AF=4.850130953535746e-05;N_HET=1;N_HOMALT=0;N_HOMREF=10308;FREQ_HET=9.700261907071492e-05;FREQ_HOMALT=0.0;FREQ_HOMREF=0.9999029973809292;N_BI_GENOS=10309;Northwest_Han_AN=1296;Northwest_Han_AC=1;No
chr5	29985283	.	T	<INS:ME:LINE1>	.	PASS	SVTYPE=INS:ME:LINE1;END=29985283;SVLEN=857;AN=20618;AC=1;AF=4.850130953535746e-05;N_HET=1;N_HOMALT=0;N_HOMREF=10308;FREQ_HET=9.700261907071492e-05;FREQ_HOMALT=0.0;FREQ_HOMREF=0.9999029973809292;N_BI_GENOS=10309;East_Han_AN=6554;East_Han_AC=1;East_Han_AF=0
chr5	30102700	.	T	<INS:ME:LINE1>	.	PASS	SVTYPE=INS:ME:LINE1;END=30102700;SVLEN=2655;AN=20618;AC=1;AF=4.850130953535746e-05;N_HET=1;N_HOMALT=0;N_HOMREF=10308;FREQ_HET=9.700261907071492e-05;FREQ_HOMALT=0.0;FREQ_HOMREF=0.9999029973809292;N_BI_GENOS=10309;Central_Han_AN=2128;Central_Han_AC=1;Centra
chr5	30197430	.	T	<INS:ME:LINE1>	.	PASS	SVTYPE=INS:ME:LINE1;END=30197444;SVLEN=1497;AN=20618;AC=1;AF=4.850130953535746e-05;N_HET=1;N_HOMALT=0;N_HOMREF=10308;FREQ_HET=9.700261907071492e-05;FREQ_HOMALT=0.0;FREQ_HOMREF=0.9999029973809292;N_BI_GENOS=10309;Hui_AN=444;Hui_AC=1;Hui_AF=0.00225225225225
chr5	30309750	.	A	<INS:ME:LINE1>	.	PASS	SVTYPE=INS:ME:LINE1;END=30309762;SVLEN=1393;AN=20618;AC=1;AF=4.850130953535746e-05;N_HET=1;N_HOMALT=0;N_HOMREF=10308;FREQ_HET=9.700261907071492e-05;FREQ_HOMALT=0.0;FREQ_HOMREF=0.9999029973809292;N_BI_GENOS=10309;Manchu_AN=416;Manchu_AC=1;Manchu_AF=0.00240
chr5	30563193	.	A	<INS:ME:LINE1>	.	PASS	SVTYPE=INS:ME:LINE1;END=30563215;SVLEN=599;AN=20618;AC=1;AF=4.850130953535746e-05;N_HET=1;N_HOMALT=0;N_HOMREF=10308;FREQ_HET=9.700261907071492e-05;FREQ_HOMALT=0.0;FREQ_HOMREF=0.9999029973809292;N_BI_GENOS=10309;South_Han_AN=1496;South_Han_AC=1;South_Han_A
chr5	30583029	.	A	<INS:ME:LINE1>	.	PASS	SVTYPE=INS:ME:LINE1;END=30583046;SVLEN=120;AN=20618;AC=19;AF=0.0009215248811717916;N_HET=19;N_HOMALT=0;N_HOMREF=10290;FREQ_HET=0.0018430497623435833;FREQ_HOMALT=0.0;FREQ_HOMREF=0.9981569502376564;N_BI_GENOS=10309;Lingnan_Han_AN=1888;Lingnan_Han_AC=3;Lingn
chr5	30663730	.	C	<INS:ME:LINE1>	.	PASS	SVTYPE=INS:ME:LINE1;END=30663746;SVLEN=1249;AN=20618;AC=1;AF=4.850130953535746e-05;N_HET=1;N_HOMALT=0;N_HOMREF=10308;FREQ_HET=9.700261907071492e-05;FREQ_HOMALT=0.0;FREQ_HOMREF=0.9999029973809292;N_BI_GENOS=10309;Manchu_AN=416;Manchu_AC=1;Manchu_AF=0.00240
chr5	30902686	.	A	<INS:ME:LINE1>	.	PASS	SVTYPE=INS:ME:LINE1;END=30902700;SVLEN=1434;AN=20618;AC=1;AF=4.850130953535746e-05;N_HET=1;N_HOMALT=0;N_HOMREF=10308;FREQ_HET=9.700261907071492e-05;FREQ_HOMALT=0.0;FREQ_HOMREF=0.9999029973809292;N_BI_GENOS=10309;East_Han_AN=6554;East_Han_AC=1;East_Han_AF=
chr5	31048648	.	G	<INS:ME:LINE1>	.	PASS	SVTYPE=INS:ME:LINE1;END=31048663;SVLEN=6000;AN=20618;AC=1;AF=4.850130953535746e-05;N_HET=1;N_HOMALT=0;N_HOMREF=10308;FREQ_HET=9.700261907071492e-05;FREQ_HOMALT=0.0;FREQ_HOMREF=0.9999029973809292;N_BI_GENOS=10309;Southeast_Han_AN=1908;Southeast_Han_AC=1;So
chr5	31105854	.	A	<INS:ME:LINE1>	.	PASS	SVTYPE=INS:ME:LINE1;END=31105868;SVLEN=626;AN=20618;AC=1;AF=4.850130953535746e-05;N_HET=1;N_HOMALT=0;N_HOMREF=10308;FREQ_HET=9.700261907071492e-05;FREQ_HOMALT=0.0;FREQ_HOMREF=0.9999029973809292;N_BI_GENOS=10309;South_Han_AN=1496;South_Han_AC=1;South_Han_A
chr5	31213584	.	T	<INS:ME:LINE1>	.	PASS	SVTYPE=INS:ME:LINE1;END=31213588;SVLEN=65;AN=20618;AC=1;AF=4.850130953535746e-05;N_HET=1;N_HOMALT=0;N_HOMREF=10308;FREQ_HET=9.700261907071492e-05;FREQ_HOMALT=0.0;FREQ_HOMREF=0.9999029973809292;N_BI_GENOS=10309;East_Han_AN=6554;East_Han_AC=1;East_Han_AF=0.
chr5	31709999	.	G	<INS:ME:LINE1>	.	PASS	SVTYPE=INS:ME:LINE1;END=31710014;SVLEN=6018;AN=20618;AC=1;AF=4.850130953535746e-05;N_HET=1;N_HOMALT=0;N_HOMREF=10308;FREQ_HET=9.700261907071492e-05;FREQ_HOMALT=0.0;FREQ_HOMREF=0.9999029973809292;N_BI_GENOS=10309;Central_Han_AN=2128;Central_Han_AC=1;Centra
chr5	32463728	.	A	<INS:ME:LINE1>	.	PASS	SVTYPE=INS:ME:LINE1;END=32463741;SVLEN=532;AN=20618;AC=7;AF=0.0003395091667475022;N_HET=3;N_HOMALT=2;N_HOMREF=10304;FREQ_HET=0.0002910078572121447;FREQ_HOMALT=0.00019400523814142983;FREQ_HOMREF=0.9995149869046465;N_BI_GENOS=10309;North_Han_AN=2272;North_H
chr5	32633222	.	A	<INS:ME:LINE1>	.	PASS	SVTYPE=INS:ME:LINE1;END=32633222;SVLEN=6047;AN=20618;AC=2;AF=9.700261907071492e-05;N_HET=2;N_HOMALT=0;N_HOMREF=10307;FREQ_HET=0.00019400523814142983;FREQ_HOMALT=0.0;FREQ_HOMREF=0.9998059947618586;N_BI_GENOS=10309;South_Han_AN=1496;South_Han_AC=1;South_Han
chr5	32643851	.	G	<INS:ME:LINE1>	.	PASS	SVTYPE=INS:ME:LINE1;END=32643866;SVLEN=4515;AN=20618;AC=4;AF=0.00019400523814142983;N_HET=4;N_HOMALT=0;N_HOMREF=10305;FREQ_HET=0.00038801047628285966;FREQ_HOMALT=0.0;FREQ_HOMREF=0.9996119895237171;N_BI_GENOS=10309;Southeast_Han_AN=1908;Southeast_Han_AC=3;
chr5	32735823	.	A	<INS:ME:LINE1>	.	PASS	SVTYPE=INS:ME:LINE1;END=32735834;SVLEN=1380;AN=20618;AC=2;AF=9.700261907071492e-05;N_HET=2;N_HOMALT=0;N_HOMREF=10307;FREQ_HET=0.00019400523814142983;FREQ_HOMALT=0.0;FREQ_HOMREF=0.9998059947618586;N_BI_GENOS=10309;East_Han_AN=6554;East_Han_AC=2;East_Han_AF
chr5	32838802	.	A	<INS:ME:LINE1>	.	PASS	SVTYPE=INS:ME:LINE1;END=32838818;SVLEN=1717;AN=20618;AC=1;AF=4.850130953535746e-05;N_HET=1;N_HOMALT=0;N_HOMREF=10308;FREQ_HET=9.700261907071492e-05;FREQ_HOMALT=0.0;FREQ_HOMREF=0.9999029973809292;N_BI_GENOS=10309;Northwest_Han_AN=1296;Northwest_Han_AC=1;No
chr5	32967388	.	A	<INS:ME:LINE1>	.	PASS	SVTYPE=INS:ME:LINE1;END=32967395;SVLEN=1071;AN=20618;AC=6;AF=0.0002910078572121447;N_HET=4;N_HOMALT=1;N_HOMREF=10304;FREQ_HET=0.00038801047628285966;FREQ_HOMALT=9.700261907071492e-05;FREQ_HOMREF=0.9995149869046465;N_BI_GENOS=10309;Northwest_Han_AN=1296;No
chr5	33086681	.	G	<INS:ME:LINE1>	.	PASS	SVTYPE=INS:ME:LINE1;END=33086692;SVLEN=780;AN=20618;AC=2;AF=9.700261907071492e-05;N_HET=2;N_HOMALT=0;N_HOMREF=10307;FREQ_HET=0.00019400523814142983;FREQ_HOMALT=0.0;FREQ_HOMREF=0.9998059947618586;N_BI_GENOS=10309;East_Han_AN=6554;East_Han_AC=1;East_Han_AF=
chr5	33206375	.	A	<INS:ME:LINE1>	.	PASS	SVTYPE=INS:ME:LINE1;END=33206395;SVLEN=759;AN=20618;AC=1;AF=4.850130953535746e-05;N_HET=1;N_HOMALT=0;N_HOMREF=10308;FREQ_HET=9.700261907071492e-05;FREQ_HOMALT=0.0;FREQ_HOMREF=0.9999029973809292;N_BI_GENOS=10309;Hui_AN=444;Hui_AC=1;Hui_AF=0.002252252252252
chr5	33265398	.	A	<INS:ME:LINE1>	.	PASS	SVTYPE=INS:ME:LINE1;END=33265412;SVLEN=5999;AN=20618;AC=3;AF=0.00014550392860607236;N_HET=3;N_HOMALT=0;N_HOMREF=10306;FREQ_HET=0.0002910078572121447;FREQ_HOMALT=0.0;FREQ_HOMREF=0.9997089921427879;N_BI_GENOS=10309;South_Han_AN=1496;South_Han_AC=1;South_Han
chr5	33306026	.	T	<INS:ME:LINE1>	.	PASS	SVTYPE=INS:ME:LINE1;END=33306045;SVLEN=5999;AN=20618;AC=1;AF=4.850130953535746e-05;N_HET=1;N_HOMALT=0;N_HOMREF=10308;FREQ_HET=9.700261907071492e-05;FREQ_HOMALT=0.0;FREQ_HOMREF=0.9999029973809292;N_BI_GENOS=10309;Southeast_Han_AN=1908;Southeast_Han_AC=1;So
chr5	33565540	.	G	<INS:ME:LINE1>	.	PASS	SVTYPE=INS:ME:LINE1;END=33565550;SVLEN=2954;AN=20618;AC=2;AF=9.700261907071492e-05;N_HET=2;N_HOMALT=0;N_HOMREF=10307;FREQ_HET=0.00019400523814142983;FREQ_HOMALT=0.0;FREQ_HOMREF=0.9998059947618586;N_BI_GENOS=10309;Tibetan_AN=414;Tibetan_AC=2;Tibetan_AF=0.0
chr5	33632396	.	A	<INS:ME:LINE1>	.	PASS	SVTYPE=INS:ME:LINE1;END=33632415;SVLEN=24;AN=20618;AC=1;AF=4.850130953535746e-05;N_HET=1;N_HOMALT=0;N_HOMREF=10308;FREQ_HET=9.700261907071492e-05;FREQ_HOMALT=0.0;FREQ_HOMREF=0.9999029973809292;N_BI_GENOS=10309;Central_Han_AN=2128;Central_Han_AC=1;Central_
chr5	33633229	.	G	<INS:ME:LINE1>	.	PASS	SVTYPE=INS:ME:LINE1;END=33633243;SVLEN=2495;AN=20618;AC=13468;AF=0.6532156368221942;N_HET=3574;N_HOMALT=4947;N_HOMREF=1788;FREQ_HET=0.34668736055873506;FREQ_HOMALT=0.47987195654282666;FREQ_HOMREF=0.17344068289843825;N_BI_GENOS=10309;South_Han_AN=1496;Sout
chr5	33642233	.	C	<INS:ME:LINE1>	.	PASS	SVTYPE=INS:ME:LINE1;END=33642248;SVLEN=1862;AN=20618;AC=1;AF=4.850130953535746e-05;N_HET=1;N_HOMALT=0;N_HOMREF=10308;FREQ_HET=9.700261907071492e-05;FREQ_HOMALT=0.0;FREQ_HOMREF=0.9999029973809292;N_BI_GENOS=10309;Central_Han_AN=2128;Central_Han_AC=1;Centra
chr5	33737040	.	A	<INS:ME:LINE1>	.	PASS	SVTYPE=INS:ME:LINE1;END=33737056;SVLEN=1447;AN=20618;AC=1;AF=4.850130953535746e-05;N_HET=1;N_HOMALT=0;N_HOMREF=10308;FREQ_HET=9.700261907071492e-05;FREQ_HOMALT=0.0;FREQ_HOMREF=0.9999029973809292;N_BI_GENOS=10309;Central_Han_AN=2128;Central_Han_AC=1;Centra
chr5	33797438	.	A	<INS:ME:LINE1>	.	PASS	SVTYPE=INS:ME:LINE1;END=33797452;SVLEN=6000;AN=20618;AC=1299;AF=0.06300320108642933;N_HET=1217;N_HOMALT=41;N_HOMREF=9051;FREQ_HET=0.11805218740906004;FREQ_HOMALT=0.003977107381899311;FREQ_HOMREF=0.8779707052090406;N_BI_GENOS=10309;South_Han_AN=1496;South_
chr5	34003288	.	C	<INS:ME:LINE1>	.	PASS	SVTYPE=INS:ME:LINE1;END=34003288;SVLEN=5820;AN=20618;AC=2;AF=9.700261907071492e-05;N_HET=2;N_HOMALT=0;N_HOMREF=10307;FREQ_HET=0.00019400523814142983;FREQ_HOMALT=0.0;FREQ_HOMREF=0.9998059947618586;N_BI_GENOS=10309;Southeast_Han_AN=1908;Southeast_Han_AC=2;S
chr5	34459883	.	A	<INS:ME:LINE1>	.	PASS	SVTYPE=INS:ME:LINE1;END=34459899;SVLEN=1893;AN=20618;AC=11;AF=0.000533514404888932;N_HET=11;N_HOMALT=0;N_HOMREF=10298;FREQ_HET=0.001067028809777864;FREQ_HOMALT=0.0;FREQ_HOMREF=0.9989329711902222;N_BI_GENOS=10309;Northwest_Han_AN=1296;Northwest_Han_AC=1;No
chr5	35010192	.	T	<INS:ME:LINE1>	.	PASS	SVTYPE=INS:ME:LINE1;END=35010193;SVLEN=1759;AN=20618;AC=10;AF=0.00048501309535357455;N_HET=10;N_HOMALT=0;N_HOMREF=10299;FREQ_HET=0.0009700261907071491;FREQ_HOMALT=0.0;FREQ_HOMREF=0.9990299738092928;N_BI_GENOS=10309;North_Han_AN=2272;North_Han_AC=1;North_H
chr5	35059777	.	T	<INS:ME:LINE1>	.	PASS	SVTYPE=INS:ME:LINE1;END=35059792;SVLEN=5992;AN=20618;AC=1;AF=4.850130953535746e-05;N_HET=1;N_HOMALT=0;N_HOMREF=10308;FREQ_HET=9.700261907071492e-05;FREQ_HOMALT=0.0;FREQ_HOMREF=0.9999029973809292;N_BI_GENOS=10309;Central_Han_AN=2128;Central_Han_AC=1;Centra
chr5	35519544	.	A	<INS:ME:LINE1>	.	PASS	SVTYPE=INS:ME:LINE1;END=35519557;SVLEN=5958;AN=20618;AC=1;AF=4.850130953535746e-05;N_HET=1;N_HOMALT=0;N_HOMREF=10308;FREQ_HET=9.700261907071492e-05;FREQ_HOMALT=0.0;FREQ_HOMREF=0.9999029973809292;N_BI_GENOS=10309;East_Han_AN=6554;East_Han_AC=1;East_Han_AF=
chr5	35566678	.	G	<INS:ME:LINE1>	.	PASS	SVTYPE=INS:ME:LINE1;END=35566694;SVLEN=1109;AN=20618;AC=1;AF=4.850130953535746e-05;N_HET=1;N_HOMALT=0;N_HOMREF=10308;FREQ_HET=9.700261907071492e-05;FREQ_HOMALT=0.0;FREQ_HOMREF=0.9999029973809292;N_BI_GENOS=10309;Northwest_Han_AN=1296;Northwest_Han_AC=1;No
chr5	35815024	.	T	<INS:ME:LINE1>	.	PASS	SVTYPE=INS:ME:LINE1;END=35815039;SVLEN=6002;AN=20618;AC=6;AF=0.0002910078572121447;N_HET=6;N_HOMALT=0;N_HOMREF=10303;FREQ_HET=0.0005820157144242894;FREQ_HOMALT=0.0;FREQ_HOMREF=0.9994179842855757;N_BI_GENOS=10309;South_Han_AN=1496;South_Han_AC=2;South_Han_
chr5	35856594	.	A	<INS:ME:LINE1>	.	PASS	SVTYPE=INS:ME:LINE1;END=35856611;SVLEN=983;AN=20618;AC=3;AF=0.00014550392860607236;N_HET=3;N_HOMALT=0;N_HOMREF=10306;FREQ_HET=0.0002910078572121447;FREQ_HOMALT=0.0;FREQ_HOMREF=0.9997089921427879;N_BI_GENOS=10309;East_Han_AN=6554;East_Han_AC=2;East_Han_AF=
chr5	35857819	.	A	<INS:ME:LINE1>	.	PASS	SVTYPE=INS:ME:LINE1;END=35857836;SVLEN=3690;AN=20618;AC=1;AF=4.850130953535746e-05;N_HET=1;N_HOMALT=0;N_HOMREF=10308;FREQ_HET=9.700261907071492e-05;FREQ_HOMALT=0.0;FREQ_HOMREF=0.9999029973809292;N_BI_GENOS=10309;South_Han_AN=1496;South_Han_AC=1;South_Han_
chr5	36003358	.	A	<INS:ME:LINE1>	.	PASS	SVTYPE=INS:ME:LINE1;END=36003374;SVLEN=605;AN=20618;AC=1;AF=4.850130953535746e-05;N_HET=1;N_HOMALT=0;N_HOMREF=10308;FREQ_HET=9.700261907071492e-05;FREQ_HOMALT=0.0;FREQ_HOMREF=0.9999029973809292;N_BI_GENOS=10309;Hui_AN=444;Hui_AC=1;Hui_AF=0.002252252252252

chr5	36124182	.	T	<INS:ME:LINE1>	.	PASS	SVTYPE=INS:ME:LINE1;END=36124196;SVLEN=2275;AN=20618;AC=2;AF=9.700261907071492e-05;N_HET=2;N_HOMALT=0;N_HOMREF=10307;FREQ_HET=0.00019400523814142983;FREQ_HOMALT=0.0;FREQ_HOMREF=0.9998059947618586;N_BI_GENOS=10309;East_Han_AN=6554;East_Han_AC=1;East_Han_AF
chr5	36204031	.	G	<INS:ME:LINE1>	.	PASS	SVTYPE=INS:ME:LINE1;END=36204031;SVLEN=1986;AN=20618;AC=3;AF=0.00014550392860607236;N_HET=3;N_HOMALT=0;N_HOMREF=10306;FREQ_HET=0.0002910078572121447;FREQ_HOMALT=0.0;FREQ_HOMREF=0.9997089921427879;N_BI_GENOS=10309;Lingnan_Han_AN=1888;Lingnan_Han_AC=1;Lingn
chr5	36257904	.	A	<INS:ME:LINE1>	.	PASS	SVTYPE=INS:ME:LINE1;END=36257918;SVLEN=4945;AN=20618;AC=1;AF=4.850130953535746e-05;N_HET=1;N_HOMALT=0;N_HOMREF=10308;FREQ_HET=9.700261907071492e-05;FREQ_HOMALT=0.0;FREQ_HOMREF=0.9999029973809292;N_BI_GENOS=10309;East_Han_AN=6554;East_Han_AC=1;East_Han_AF=
chr5	36976936	.	T	<INS:ME:LINE1>	.	PASS	SVTYPE=INS:ME:LINE1;END=36976937;SVLEN=136;AN=20618;AC=1;AF=4.850130953535746e-05;N_HET=1;N_HOMALT=0;N_HOMREF=10308;FREQ_HET=9.700261907071492e-05;FREQ_HOMALT=0.0;FREQ_HOMREF=0.9999029973809292;N_BI_GENOS=10309;South_Han_AN=1496;South_Han_AC=1;South_Han_A
chr5	37002585	.	A	<INS:ME:LINE1>	.	PASS	SVTYPE=INS:ME:LINE1;END=37002601;SVLEN=2231;AN=20618;AC=2;AF=9.700261907071492e-05;N_HET=2;N_HOMALT=0;N_HOMREF=10307;FREQ_HET=0.00019400523814142983;FREQ_HOMALT=0.0;FREQ_HOMREF=0.9998059947618586;N_BI_GENOS=10309;East_Han_AN=6554;East_Han_AC=2;East_Han_AF
chr5	37202081	.	G	<INS:ME:LINE1>	.	PASS	SVTYPE=INS:ME:LINE1;END=37202099;SVLEN=2991;AN=20618;AC=1;AF=4.850130953535746e-05;N_HET=1;N_HOMALT=0;N_HOMREF=10308;FREQ_HET=9.700261907071492e-05;FREQ_HOMALT=0.0;FREQ_HOMREF=0.9999029973809292;N_BI_GENOS=10309;Northwest_Han_AN=1296;Northwest_Han_AC=1;No
chr5	37604101	.	G	<INS:ME:LINE1>	.	PASS	SVTYPE=INS:ME:LINE1;END=37604118;SVLEN=5998;AN=20618;AC=2;AF=9.700261907071492e-05;N_HET=2;N_HOMALT=0;N_HOMREF=10307;FREQ_HET=0.00019400523814142983;FREQ_HOMALT=0.0;FREQ_HOMREF=0.9998059947618586;N_BI_GENOS=10309;Southeast_Han_AN=1908;Southeast_Han_AC=2;S
chr5	37922667	.	T	<INS:ME:LINE1>	.	PASS	SVTYPE=INS:ME:LINE1;END=37922686;SVLEN=2768;AN=20618;AC=1;AF=4.850130953535746e-05;N_HET=1;N_HOMALT=0;N_HOMREF=10308;FREQ_HET=9.700261907071492e-05;FREQ_HOMALT=0.0;FREQ_HOMREF=0.9999029973809292;N_BI_GENOS=10309;East_Han_AN=6554;East_Han_AC=1;East_Han_AF=
chr5	37975784	.	G	<INS:ME:LINE1>	.	PASS	SVTYPE=INS:ME:LINE1;END=37975799;SVLEN=1925;AN=20618;AC=1;AF=4.850130953535746e-05;N_HET=1;N_HOMALT=0;N_HOMREF=10308;FREQ_HET=9.700261907071492e-05;FREQ_HOMALT=0.0;FREQ_HOMREF=0.9999029973809292;N_BI_GENOS=10309;South_Han_AN=1496;South_Han_AC=1;South_Han_
chr5	37978870	.	C	<INS:ME:LINE1>	.	PASS	SVTYPE=INS:ME:LINE1;END=37978885;SVLEN=6003;AN=20618;AC=2;AF=9.700261907071492e-05;N_HET=2;N_HOMALT=0;N_HOMREF=10307;FREQ_HET=0.00019400523814142983;FREQ_HOMALT=0.0;FREQ_HOMREF=0.9998059947618586;N_BI_GENOS=10309;South_Han_AN=1496;South_Han_AC=1;South_Han
chr5	38042326	.	A	<INS:ME:LINE1>	.	PASS	SVTYPE=INS:ME:LINE1;END=38042326;SVLEN=0;AN=20618;AC=13;AF=0.0006305170239596469;N_HET=13;N_HOMALT=0;N_HOMREF=10296;FREQ_HET=0.0012610340479192938;FREQ_HOMALT=0.0;FREQ_HOMREF=0.9987389659520807;N_BI_GENOS=10309;East_Han_AN=6554;East_Han_AC=8;East_Han_AF=0
chr5	38053891	.	C	<INS:ME:LINE1>	.	PASS	SVTYPE=INS:ME:LINE1;END=38053891;SVLEN=4819;AN=20618;AC=1;AF=4.850130953535746e-05;N_HET=1;N_HOMALT=0;N_HOMREF=10308;FREQ_HET=9.700261907071492e-05;FREQ_HOMALT=0.0;FREQ_HOMREF=0.9999029973809292;N_BI_GENOS=10309;Central_Han_AN=2128;Central_Han_AC=1;Centra
chr5	38090569	.	T	<INS:ME:LINE1>	.	PASS	SVTYPE=INS:ME:LINE1;END=38090575;SVLEN=871;AN=20618;AC=1;AF=4.850130953535746e-05;N_HET=1;N_HOMALT=0;N_HOMREF=10308;FREQ_HET=9.700261907071492e-05;FREQ_HOMALT=0.0;FREQ_HOMREF=0.9999029973809292;N_BI_GENOS=10309;Yi_AN=422;Yi_AC=1;Yi_AF=0.002369668246445498
chr5	38599734	.	A	<INS:ME:LINE1>	.	PASS	SVTYPE=INS:ME:LINE1;END=38599750;SVLEN=6000;AN=20618;AC=4;AF=0.00019400523814142983;N_HET=4;N_HOMALT=0;N_HOMREF=10305;FREQ_HET=0.0003880104762828966;FREQ_HOMALT=0.0;FREQ_HOMREF=0.9996119895237171;N_BI_GENOS=10309;South_Han_AN=1496;South_Han_AC=1;South_Ha
chr5	38774269	.	G	<INS:ME:LINE1>	.	PASS	SVTYPE=INS:ME:LINE1;END=38774287;SVLEN=6039;AN=20618;AC=2;AF=9.700261907071492e-05;N_HET=2;N_HOMALT=0;N_HOMREF=10307;FREQ_HET=0.00019400523814142983;FREQ_HOMALT=0.0;FREQ_HOMREF=0.9998059947618586;N_BI_GENOS=10309;Lingnan_Han_AN=1888;Lingnan_Han_AC=1;Lingn
chr5	38946411	.	G	<INS:ME:LINE1>	.	PASS	SVTYPE=INS:ME:LINE1;END=38946424;SVLEN=360;AN=20618;AC=1;AF=4.850130953535746e-05;N_HET=1;N_HOMALT=0;N_HOMREF=10308;FREQ_HET=9.700261907071492e-05;FREQ_HOMALT=0.0;FREQ_HOMREF=0.9999029973809292;N_BI_GENOS=10309;Central_Han_AN=2128;Central_Han_AC=1;Central
chr5	39129406	.	T	<INS:ME:LINE1>	.	PASS	SVTYPE=INS:ME:LINE1;END=39129406;SVLEN=341;AN=20618;AC=1;AF=4.850130953535746e-05;N_HET=1;N_HOMALT=0;N_HOMREF=10308;FREQ_HET=9.700261907071492e-05;FREQ_HOMALT=0.0;FREQ_HOMREF=0.9999029973809292;N_BI_GENOS=10309;East_Han_AN=6554;East_Han_AC=1;East_Han_AF=0
chr5	39183165	.	A	<INS:ME:LINE1>	.	PASS	SVTYPE=INS:ME:LINE1;END=39183180;SVLEN=6000;AN=20618;AC=1;AF=4.850130953535746e-05;N_HET=1;N_HOMALT=0;N_HOMREF=10308;FREQ_HET=9.700261907071492e-05;FREQ_HOMALT=0.0;FREQ_HOMREF=0.9999029973809292;N_BI_GENOS=10309;South_Han_AN=1496;South_Han_AC=1;South_Han_
chr5	39217089	.	C	<INS:ME:LINE1>	.	PASS	SVTYPE=INS:ME:LINE1;END=39217092;SVLEN=5312;AN=20618;AC=1;AF=4.850130953535746e-05;N_HET=1;N_HOMALT=0;N_HOMREF=10308;FREQ_HET=9.700261907071492e-05;FREQ_HOMALT=0.0;FREQ_HOMREF=0.9999029973809292;N_BI_GENOS=10309;Mongolian_AN=454;Mongolian_AC=1;Mongolian_A
chr5	39310795	.	A	<INS:ME:LINE1>	.	PASS	SVTYPE=INS:ME:LINE1;END=39310800;SVLEN=1246;AN=20618;AC=1;AF=4.850130953535746e-05;N_HET=1;N_HOMALT=0;N_HOMREF=10308;FREQ_HET=9.700261907071492e-05;FREQ_HOMALT=0.0;FREQ_HOMREF=0.9999029973809292;N_BI_GENOS=10309;East_Han_AN=6554;East_Han_AC=1;East_Han_AF=
chr5	39399460	.	A	<INS:ME:LINE1>	.	PASS	SVTYPE=INS:ME:LINE1;END=39399479;SVLEN=4537;AN=20618;AC=1;AF=4.850130953535746e-05;N_HET=1;N_HOMALT=0;N_HOMREF=10308;FREQ_HET=9.700261907071492e-05;FREQ_HOMALT=0.0;FREQ_HOMREF=0.9999029973809292;N_BI_GENOS=10309;Central_Han_AN=2128;Central_Han_AC=1;Centra
chr5	39462727	.	T	<INS:ME:LINE1>	.	PASS	SVTYPE=INS:ME:LINE1;END=39462740;SVLEN=910;AN=20618;AC=7;AF=0.0003395091667475022;N_HET=7;N_HOMALT=0;N_HOMREF=10302;FREQ_HET=0.0006790183334950044;FREQ_HOMALT=0.0;FREQ_HOMREF=0.999320981666505;N_BI_GENOS=10309;Northwest_Han_AN=1296;Northwest_Han_AC=1;Nort
chr5	39744926	.	T	<INS:ME:LINE1>	.	PASS	SVTYPE=INS:ME:LINE1;END=39744926;SVLEN=5998;AN=20618;AC=1;AF=4.850130953535746e-05;N_HET=1;N_HOMALT=0;N_HOMREF=10308;FREQ_HET=9.700261907071492e-05;FREQ_HOMALT=0.0;FREQ_HOMREF=0.9999029973809292;N_BI_GENOS=10309;Northwest_Han_AN=1296;Northwest_Han_AC=1;No
chr5	39926001	.	A	<INS:ME:LINE1>	.	PASS	SVTYPE=INS:ME:LINE1;END=39926016;SVLEN=1892;AN=20618;AC=10;AF=0.0004850130953535746;N_HET=10;N_HOMALT=0;N_HOMREF=10299;FREQ_HET=0.0009700261907071491;FREQ_HOMALT=0.0;FREQ_HOMREF=0.9990299738092928;N_BI_GENOS=10309;Northwest_Han_AN=1296;Northwest_Han_AC=1
chr5	39940779	.	T	<INS:ME:LINE1>	.	PASS	SVTYPE=INS:ME:LINE1;END=39940795;SVLEN=6003;AN=20618;AC=1;AF=4.850130953535746e-05;N_HET=1;N_HOMALT=0;N_HOMREF=10308;FREQ_HET=9.700261907071492e-05;FREQ_HOMALT=0.0;FREQ_HOMREF=0.9999029973809292;N_BI_GENOS=10309;South_Han_AN=1496;South_Han_AC=1;South_Han_
chr5	39958711	.	C	<INS:ME:LINE1>	.	PASS	SVTYPE=INS:ME:LINE1;END=39958720;SVLEN=1356;AN=20618;AC=17;AF=0.0008245222621010767;N_HET=17;N_HOMALT=0;N_HOMREF=10292;FREQ_HET=0.0016490445242021534;FREQ_HOMALT=0.0;FREQ_HOMREF=0.9983509554757979;N_BI_GENOS=10309;South_Han_AN=1496;South_Han_AC=2;South_Ha
chr5	39958998	.	A	<INS:ME:LINE1>	.	PASS	SVTYPE=INS:ME:LINE1;END=39959009;SVLEN=1498;AN=20618;AC=1;AF=4.850130953535746e-05;N_HET=1;N_HOMALT=0;N_HOMREF=10308;FREQ_HET=9.700261907071492e-05;FREQ_HOMALT=0.0;FREQ_HOMREF=0.9999029973809292;N_BI_GENOS=10309;Central_Han_AN=2128;Central_Han_AC=1;Centra
chr5	40041233	.	C	<INS:ME:LINE1>	.	PASS	SVTYPE=INS:ME:LINE1;END=40041243;SVLEN=5996;AN=20618;AC=853;AF=0.04137161703365991;N_HET=785;N_HOMALT=34;N_HOMREF=9490;FREQ_HET=0.0761470559705112;FREQ_HOMALT=0.0032980890484043067;FREQ_HOMREF=0.9205548549810845;N_BI_GENOS=10309;South_Han_AN=1496;South_Ha
chr5	40158510	.	T	<INS:ME:LINE1>	.	PASS	SVTYPE=INS:ME:LINE1;END=40158523;SVLEN=6002;AN=20618;AC=1;AF=4.850130953535746e-05;N_HET=1;N_HOMALT=0;N_HOMREF=10308;FREQ_HET=9.700261907071492e-05;FREQ_HOMALT=0.0;FREQ_HOMREF=0.9999029973809292;N_BI_GENOS=10309;Northwest_Han_AN=1296;Northwest_Han_AC=1;No
chr5	40191587	.	C	<INS:ME:LINE1>	.	PASS	SVTYPE=INS:ME:LINE1;END=40191587;SVLEN=342;AN=20618;AC=1;AF=4.850130953535746e-05;N_HET=1;N_HOMALT=0;N_HOMREF=10308;FREQ_HET=9.700261907071492e-05;FREQ_HOMALT=0.0;FREQ_HOMREF=0.9999029973809292;N_BI_GENOS=10309;North_Han_AN=2272;North_Han_AC=1;North_Han_A
chr5	40196719	.	A	<INS:ME:LINE1>	.	PASS	SVTYPE=INS:ME:LINE1;END=40196736;SVLEN=832;AN=20618;AC=1;AF=4.850130953535746e-05;N_HET=1;N_HOMALT=0;N_HOMREF=10308;FREQ_HET=9.700261907071492e-05;FREQ_HOMALT=0.0;FREQ_HOMREF=0.9999029973809292;N_BI_GENOS=10309;Zhuang_AN=444;Zhuang_AC=1;Zhuang_AF=0.002252
chr5	40490274	.	A	<INS:ME:LINE1>	.	PASS	SVTYPE=INS:ME:LINE1;END=40490285;SVLEN=601;AN=20618;AC=5;AF=0.00024250654767678728;N_HET=5;N_HOMALT=0;N_HOMREF=10304;FREQ_HET=0.0004850130953535746;FREQ_HOMALT=0.0;FREQ_HOMREF=0.9995149869046465;N_BI_GENOS=10309;Lingnan_Han_AN=1888;Lingnan_Han_AC=2;Lingn
chr5	40521776	.	A	<INS:ME:LINE1>	.	PASS	SVTYPE=INS:ME:LINE1;END=40521790;SVLEN=6019;AN=20618;AC=1;AF=4.850130953535746e-05;N_HET=1;N_HOMALT=0;N_HOMREF=10308;FREQ_HET=9.700261907071492e-05;FREQ_HOMALT=0.0;FREQ_HOMREF=0.9999029973809292;N_BI_GENOS=10309;Northwest_Han_AN=1296;Northwest_Han_AC=1;No
chr5	40660527	.	C	<INS:ME:LINE1>	.	PASS	SVTYPE=INS:ME:LINE1;END=40660542;SVLEN=6006;AN=20618;AC=7;AF=0.0003395091667475022;N_HET=7;N_HOMALT=0;N_HOMREF=10302;FREQ_HET=0.0006790183334950044;FREQ_HOMALT=0.0;FREQ_HOMREF=0.999320981666505;N_BI_GENOS=10309;East_Han_AN=6554;East_Han_AC=5;East_Han_AF=0
chr5	40724743	.	T	<INS:ME:LINE1>	.	PASS	SVTYPE=INS:ME:LINE1;END=40724754;SVLEN=97;AN=20618;AC=1;AF=4.850130953535746e-05;N_HET=1;N_HOMALT=0;N_HOMREF=10308;FREQ_HET=9.700261907071492e-05;FREQ_HOMALT=0.0;FREQ_HOMREF=0.9999029973809292;N_BI_GENOS=10309;East_Han_AN=6554;East_Han_AC=1;East_Han_AF=0.
chr5	41357009	.	C	<INS:ME:LINE1>	.	PASS	SVTYPE=INS:ME:LINE1;END=41357023;SVLEN=6000;AN=20618;AC=1;AF=4.850130953535746e-05;N_HET=1;N_HOMALT=0;N_HOMREF=10308;FREQ_HET=9.700261907071492e-05;FREQ_HOMALT=0.0;FREQ_HOMREF=0.9999029973809292;N_BI_GENOS=10309;South_Han_AN=1496;South_Han_AC=1;South_Han_
chr5	41373033	.	T	<INS:ME:LINE1>	.	PASS	SVTYPE=INS:ME:LINE1;END=41373050;SVLEN=114;AN=20618;AC=2;AF=9.700261907071492e-05;N_HET=2;N_HOMALT=0;N_HOMREF=10307;FREQ_HET=0.00019400523814142983;FREQ_HOMALT=0.0;FREQ_HOMREF=0.9998059947618586;N_BI_GENOS=10309;Lingnan_Han_AN=1888;Lingnan_Han_AC=2;Lingna
chr5	41448454	.	A	<INS:ME:LINE1>	.	PASS	SVTYPE=INS:ME:LINE1;END=41448473;SVLEN=1763;AN=20618;AC=1;AF=4.850130953535746e-05;N_HET=1;N_HOMALT=0;N_HOMREF=10308;FREQ_HET=9.700261907071492e-05;FREQ_HOMALT=0.0;FREQ_HOMREF=0.9999029973809292;N_BI_GENOS=10309;Central_Han_AN=2128;Central_Han_AC=1;Centra
chr5	41449873	.	A	<INS:ME:LINE1>	.	PASS	SVTYPE=INS:ME:LINE1;END=41449873;SVLEN=2079;AN=20618;AC=1;AF=4.850130953535746e-05;N_HET=1;N_HOMALT=0;N_HOMREF=10308;FREQ_HET=9.700261907071492e-05;FREQ_HOMALT=0.0;FREQ_HOMREF=0.9999029973809292;N_BI_GENOS=10309;Southeast_Han_AN=1908;Southeast_Han_AC=1;So
chr5	41514798	.	A	<INS:ME:LINE1>	.	PASS	SVTYPE=INS:ME:LINE1;END=41514815;SVLEN=3600;AN=20618;AC=1;AF=4.850130953535746e-05;N_HET=1;N_HOMALT=0;N_HOMREF=10308;FREQ_HET=9.700261907071492e-05;FREQ_HOMALT=0.0;FREQ_HOMREF=0.9999029973809292;N_BI_GENOS=10309;Yi_AN=422;Yi_AC=1;Yi_AF=0.00236966824644549
chr5	41601925	.	A	<INS:ME:LINE1>	.	PASS	SVTYPE=INS:ME:LINE1;END=41601941;SVLEN=1086;AN=20618;AC=20;AF=0.0009700261907071491;N_HET=20;N_HOMALT=0;N_HOMREF=10289;FREQ_HET=0.0019400523814142982;FREQ_HOMALT=0.0;FREQ_HOMREF=0.9980599476185857;N_BI_GENOS=10309;Northwest_Han_AN=1296;Northwest_Han_AC=1;
chr5	41695579	.	C	<INS:ME:LINE1>	.	PASS	SVTYPE=INS:ME:LINE1;END=41695579;SVLEN=2828;AN=20618;AC=3;AF=0.00014550392860607236;N_HET=1;N_HOMALT=1;N_HOMREF=10307;FREQ_HET=9.700261907071492e-05;FREQ_HOMALT=9.700261907071492e-05;FREQ_HOMREF=0.9998059947618586;N_BI_GENOS=10309;North_Han_AN=2272;North_
chr5	41821032	.	A	<INS:ME:LINE1>	.	PASS	SVTYPE=INS:ME:LINE1;END=41821046;SVLEN=1885;AN=20618;AC=1;AF=4.850130953535746e-05;N_HET=1;N_HOMALT=0;N_HOMREF=10308;FREQ_HET=9.700261907071492e-05;FREQ_HOMALT=0.0;FREQ_HOMREF=0.9999029973809292;N_BI_GENOS=10309;Lingnan_Han_AN=1888;Lingnan_Han_AC=1;Lingna
chr5	42057941	.	C	<INS:ME:LINE1>	.	PASS	SVTYPE=INS:ME:LINE1;END=42057955;SVLEN=6000;AN=20618;AC=11;AF=0.0005335144404888932;N_HET=11;N_HOMALT=0;N_HOMREF=10298;FREQ_HET=0.0010670288809777864;FREQ_HOMALT=0.0;FREQ_HOMREF=0.9989329711902222;N_BI_GENOS=10309;North_Han_AN=2272;North_Han_AC=1;North_Han_
chr5	42118396	.	A	<INS:ME:LINE1>	.	PASS	SVTYPE=INS:ME:LINE1;END=42118410;SVLEN=1415;AN=20618;AC=1;AF=4.850130953535746e-05;N_HET=1;N_HOMALT=0;N_HOMREF=10308;FREQ_HET=9.700261907071492e-05;FREQ_HOMALT=0.0;FREQ_HOMREF=0.9999029973809292;N_BI_GENOS=10309;North_Han_AN=2272;North_Han_AC=1;North_Han_
chr5	42409601	.	G	<INS:ME:LINE1>	.	PASS	SVTYPE=INS:ME:LINE1;END=42409618;SVLEN=6002;AN=20618;AC=1;AF=4.850130953535746e-05;N_HET=1;N_HOMALT=0;N_HOMREF=10308;FREQ_HET=9.700261907071492e-05;FREQ_HOMALT=0.0;FREQ_HOMREF=0.9999029973809292;N_BI_GENOS=10309;East_Han_AN=6554;East_Han_AC=1;East_Han_AF=
chr5	42667944	.	C	<INS:ME:LINE1>	.	PASS	SVTYPE=INS:ME:LINE1;END=42667958;SVLEN=295;AN=20618;AC=1;AF=4.850130953535746e-05;N_HET=1;N_HOMALT=0;N_HOMREF=10308;FREQ_HET=9.700261907071492e-05;FREQ_HOMALT=0.0;FREQ_HOMREF=0.9999029973809292;N_BI_GENOS=10309;Southeast_Han_AN=1908;Southeast_Han_AC=1;Sou
chr5	42783432	.	A	<INS:ME:LINE1>	.	PASS	SVTYPE=INS:ME:LINE1;END=42783432;SVLEN=0;AN=20618;AC=1;AF=4.850130953535746e-05;N_HET=1;N_HOMALT=0;N_HOMREF=10308;FREQ_HET=9.700261907071492e-05;FREQ_HOMALT=0.0;FREQ_HOMREF=0.9999029973809292;N_BI_GENOS=10309;Southeast_Han_AN=1908;Southeast_Han_AC=1;South
chr5	43800759	.	T	<INS:ME:LINE1>	.	PASS	SVTYPE=INS:ME:LINE1;END=43800770;SVLEN=1520;AN=20618;AC=944;AF=0.04578536201377436;N_HET=830;N_HOMALT=57;N_HOMREF=9422;FREQ_HET=0.08051217382869337;FREQ_HOMALT=0.0055291492870307496;FREQ_HOMREF=0.9139586768842759;N_BI_GENOS=10309;South_Han_AN=1496;South_
chr5	44167470	.	T	<INS:ME:LINE1>	.	PASS	SVTYPE=INS:ME:LINE1;END=44167489;SVLEN=2619;AN=20618;AC=3;AF=0.00014550392860607236;N_HET=1;N_HOMALT=1;N_HOMREF=10307;FREQ_HET=9.700261907071492e-05;FREQ_HOMALT=9.700261907071492e-05;FREQ_HOMREF=0.9998059947618586;N_BI_GENOS=10309;East_Han_AN=6554;East_Ha
chr5	44639258	.	A	<INS:ME:LINE1>	.	PASS	SVTYPE=INS:ME:LINE1;END=44639276;SVLEN=5997;AN=20618;AC=3;AF=0.00014550392860607236;N_HET=3;N_HOMALT=0;N_HOMREF=10306;FREQ_HET=0.0002910078572121447;FREQ_HOMALT=0.0;FREQ_HOMREF=0.9997089921427879;N_BI_GENOS=10309;Lingnan_Han_AN=1888;Lingnan_Han_AC=1;Lingn
chr5	45132898	.	C	<INS:ME:LINE1>	.	PASS	SVTYPE=INS:ME:LINE1;END=45132917;SVLEN=770;AN=20618;AC=1;AF=4.850130953535746e-05;N_HET=1;N_HOMALT=0;N_HOMREF=10308;FREQ_HET=9.700261907071492e-05;FREQ_HOMALT=0.0;FREQ_HOMREF=0.9999029973809292;N_BI_GENOS=10309;Lingnan_Han_AN=1888;Lingnan_Han_AC=1;Lingnan
chr5	45195747	.	T	<INS:ME:LINE1>	.	PASS	SVTYPE=INS:ME:LINE1;END=45195752;SVLEN=1489;AN=20618;AC=11;AF=0.0005335144404888932;N_HET=11;N_HOMALT=0;N_HOMREF=10298;FREQ_HET=0.0010670288809777864;FREQ_HOMALT=0.0;FREQ_HOMREF=0.9989329711902222;N_BI_GENOS=10309;Northwest_Han_AN=1296;Northwest_Han_AC=1;No
chr5	46090232	.	G	<INS:ME:LINE1>	.	PASS	SVTYPE=INS:ME:LINE1;END=46090246;SVLEN=1871;AN=20618;AC=2;AF=9.700261907071492e-05;N_HET=2;N_HOMALT=0;N_HOMREF=10307;FREQ_HET=0.00019400523814142983;FREQ_HOMALT=0.0;FREQ_HOMREF=0.9998059947618586;N_BI_GENOS=10309;Miao_AN=482;Miao_AC=2;Miao_AF=0.0041493775
chr5	46118678	.	T	<INS:ME:LINE1>	.	PASS	SVTYPE=INS:ME:LINE1;END=46118689;SVLEN=1406;AN=20618;AC=1;AF=4.850130953535746e-05;N_HET=1;N_HOMALT=0;N_HOMREF=10308;FREQ_HET=9.700261907071492e-05;FREQ_HOMALT=0.0;FREQ_HOMREF=0.9999029973809292;N_BI_GENOS=10309;Lingnan_Han_AN=1888;Lingnan_Han_AC=1;Lingna
chr5	46241154	.	G	<INS:ME:LINE1>	.	PASS	SVTYPE=INS:ME:LINE1;END=46241160;SVLEN=109;AN=20618;AC=2;AF=9.700261907071492e-05;N_HET=2;N_HOMALT=0;N_HOMREF=10307;FREQ_HET=0.00019400523814142983;FREQ_HOMALT=0.0;FREQ_HOMREF=0.9998059947618586;N_BI_GENOS=10309;North_Han_AN=2272;North_Han_AC=1;North_Han_
chr5	46256584	.	T	<INS:ME:LINE1>	.	PASS	SVTYPE=INS:ME:LINE1;END=46256594;SVLEN=327;AN=20618;AC=1;AF=4.850130953535746e-05;N_HET=1;N_HOMALT=0;N_HOMREF=10308;FREQ_HET=9.700261907071492e-05;FREQ_HOMALT=0.0;FREQ_HOMREF=0.9999029973809292;N_BI_GENOS=10309;Central_Han_AN=2128;Central_Han_AC=1;Central
chr5	46341212	.	A	<INS:ME:LINE1>	.	PASS	SVTYPE=INS:ME:LINE1;END=46341224;SVLEN=1728;AN=20618;AC=1;AF=4.850130953535746e-05;N_HET=1;N_HOMALT=0;N_HOMREF=10308;FREQ_HET=9.700261907071492e-05;FREQ_HOMALT=0.0;FREQ_HOMREF=0.9999029973809292;N_BI_GENOS=10309;East_Han_AN=6554;East_Han_AC=1;East_Han_AF=
chr5	46350694	.	G	<INS:ME:LINE1>	.	PASS	SVTYPE=INS:ME:LINE1;END=46350709;SVLEN=86;AN=20618;AC=1;AF=4.850130953535746e-05;N_HET=1;N_HOMALT=0;N_HOMREF=10308;FREQ_HET=9.700261907071492e-05;FREQ_HOMALT=0.0;FREQ_HOMREF=0.9999029973809292;N_BI_GENOS=10309;Central_Han_AN=2128;Central_Han_AC=1;Central_
chr5	46365542	.	T	<INS:ME:LINE1>	.	PASS	SVTYPE=INS:ME:LINE1;END=46365556;SVLEN=436;AN=20618;AC=7;AF=0.0003395091667475022;N_HET=7;N_HOMALT=0;N_HOMREF=10302;FREQ_HET=0.0006790183334950044;FREQ_HOMALT=0.0;FREQ_HOMREF=0.999320981666505;N_BI_GENOS=10309;Lingnan_Han_AN=1888;Lingnan_Han_AC=1;Lingnan_
chr5	46401340	.	T	<INS:ME:LINE1>	.	PASS	SVTYPE=INS:ME:LINE1;END=46401354;SVLEN=606;AN=20618;AC=2;AF=9.700261907071492e-05;N_HET=2;N_HOMALT=0;N_HOMREF=10307;FREQ_HET=0.00019400523814142983;FREQ_HOMALT=0.0;FREQ_HOMREF=0.9998059947618586;N_BI_GENOS=10309;Central_Han_AN=2128;Central_Han_AC=1;Centra
chr5	46416790	.	T	<INS:ME:LINE1>	.	PASS	SVTYPE=INS:ME:LINE1;END=46416807;SVLEN=1028;AN=20618;AC=1;AF=4.850130953535746e-05;N_HET=1;N_HOMALT=0;N_HOMREF=10308;FREQ_HET=9.700261907071492e-05;FREQ_HOMALT=0.0;FREQ_HOMREF=0.9999029973809292;N_BI_GENOS=10309;East_Han_AN=6554;East_Han_AC=1;East_Han_AF=
chr5	50147883	.	A	<INS:ME:LINE1>	.	PASS	SVTYPE=INS:ME:LINE1;END=50147899;SVLEN=1670;AN=20618;AC=1;AF=4.850130953535746e-05;N_HET=1;N_HOMALT=0;N_HOMREF=10308;FREQ_HET=9.700261907071492e-05;FREQ_HOMALT=0.0;FREQ_HOMREF=0.9999029973809292;N_BI_GENOS=10309;Northwest_Han_AN=1296;Northwest_Han_AC=1;No
chr5	50179379	.	A	<INS:ME:LINE1>	.	PASS	SVTYPE=INS:ME:LINE1;END=50179397;SVLEN=139;AN=20618;AC=2;AF=9.700261907071492e-05;N_HET=0;N_HOMALT=1;N_HOMREF=10308;FREQ_HET=0.0;FREQ_HOMALT=9.700261907071492e-05;FREQ_HOMREF=0.9999029973809292;N_BI_GENOS=10309;East_Han_AN=6554;East_Han_AC=2;East_Han_AF=0
chr5	50183247	.	T	<INS:ME:LINE1>	.	PASS	SVTYPE=INS:ME:LINE1;END=50183247;SVLEN=79;AN=20618;AC=8;AF=0.00038801047628285966;N_HET=8;N_HOMALT=0;N_HOMREF=10301;FREQ_HET=0.0007760209525657193;FREQ_HOMALT=0.0;FREQ_HOMREF=0.9992239790474343;N_BI_GENOS=10309;North_Han_AN=2272;North_Han_AC=1;North_Han_A
chr5	50192713	.	A	<INS:ME:LINE1>	.	PASS	SVTYPE=INS:ME:LINE1;END=50192723;SVLEN=579;AN=20618;AC=1;AF=4.850130953535746e-05;N_HET=1;N_HOMALT=0;N_HOMREF=10308;FREQ_HET=9.700261907071492e-05;FREQ_HOMALT=0.0;FREQ_HOMREF=0.9999029973809292;N_BI_GENOS=10309;South_Han_AN=1496;South_Han_AC=1;South_Han_A
chr5	50194993	.	C	<INS:ME:LINE1>	.	PASS	SVTYPE=INS:ME:LINE1;END=50195009;SVLEN=4056;AN=20618;AC=1;AF=4.850130953535746e-05;N_HET=1;N_HOMALT=0;N_HOMREF=10308;FREQ_HET=9.700261907071492e-05;FREQ_HOMALT=0.0;FREQ_HOMREF=0.9999029973809292;N_BI_GENOS=10309;South_Han_AN=1496;South_Han_AC=1;South_Han_
chr5	50245323	.	A	<INS:ME:LINE1>	.	PASS	SVTYPE=INS:ME:LINE1;END=50245339;SVLEN=1251;AN=20618;AC=1;AF=4.850130953535746e-05;N_HET=1;N_HOMALT=0;N_HOMREF=10308;FREQ_HET=9.700261907071492e-05;FREQ_HOMALT=0.0;FREQ_HOMREF=0.9999029973809292;N_BI_GENOS=10309;East_Han_AN=6554;East_Han_AC=1;East_Han_AF=
chr5	50313566	.	T	<INS:ME:LINE1>	.	PASS	SVTYPE=INS:ME:LINE1;END=50313577;SVLEN=969;AN=20618;AC=2;AF=9.700261907071492e-05;N_HET=2;N_HOMALT=0;N_HOMREF=10307;FREQ_HET=0.00019400523814142983;FREQ_HOMALT=0.0;FREQ_HOMREF=0.9998059947618586;N_BI_GENOS=10309;Lingnan_Han_AN=1888;Lingnan_Han_AC=1;Lingna
chr5	50343220	.	C	<INS:ME:LINE1>	.	PASS	SVTYPE=INS:ME:LINE1;END=50343236;SVLEN=930;AN=20618;AC=11;AF=0.0005335144404888932;N_HET=9;N_HOMALT=1;N_HOMREF=10299;FREQ_HET=0.0008730235716364342;FREQ_HOMALT=9.700261907071492e-05;FREQ_HOMREF=0.9990299738092928;N_BI_GENOS=10309;South_Han_AN=1496;South_Ha
chr5	50424293	.	A	<INS:ME:LINE1>	.	PASS	SVTYPE=INS:ME:LINE1;END=50424295;SVLEN=390;AN=20618;AC=1;AF=4.850130953535746e-05;N_HET=1;N_HOMALT=0;N_HOMREF=10308;FREQ_HET=9.700261907071492e-05;FREQ_HOMALT=0.0;FREQ_HOMREF=0.9999029973809292;N_BI_GENOS=10309;North_Han_AN=2272;North_Han_AC=1;North_Han_A
chr5	50556071	.	A	<INS:ME:LINE1>	.	PASS	SVTYPE=INS:ME:LINE1;END=50556082;SVLEN=512;AN=20618;AC=2;AF=9.700261907071492e-05;N_HET=2;N_HOMALT=0;N_HOMREF=10307;FREQ_HET=0.00019400523814142983;FREQ_HOMALT=0.0;FREQ_HOMREF=0.9998059947618586;N_BI_GENOS=10309;East_Han_AN=6554;East_Han_AC=2;East_Han_AF=
chr5	50783253	.	T	<INS:ME:LINE1>	.	PASS	SVTYPE=INS:ME:LINE1;END=50783261;SVLEN=733;AN=20618;AC=2;AF=9.700261907071492e-05;N_HET=2;N_HOMALT=0;N_HOMREF=10307;FREQ_HET=0.00019400523814142983;FREQ_HOMALT=0.0;FREQ_HOMREF=0.9998059947618586;N_BI_GENOS=10309;East_Han_AN=6554;East_Han_AC=1;East_Han_AF=
chr5	51009514	.	T	<INS:ME:LINE1>	.	PASS	SVTYPE=INS:ME:LINE1;END=51009532;SVLEN=1226;AN=20618;AC=1;AF=4.850130953535746e-05;N_HET=1;N_HOMALT=0;N_HOMREF=10308;FREQ_HET=9.700261907071492e-05;FREQ_HOMALT=0.0;FREQ_HOMREF=0.9999029973809292;N_BI_GENOS=10309;North_Han_AN=2272;North_Han_AC=1;North_Han_
chr5	51106994	.	A	<INS:ME:LINE1>	.	PASS	SVTYPE=INS:ME:LINE1;END=51107011;SVLEN=6000;AN=20618;AC=2;AF=9.700261907071492e-05;N_HET=2;N_HOMALT=0;N_HOMREF=10307;FREQ_HET=0.00019400523814142983;FREQ_HOMALT=0.0;FREQ_HOMREF=0.9998059947618586;N_BI_GENOS=10309;Lingnan_Han_AN=1888;Lingnan_Han_AC=2;Lingn
chr5	51196778	.	A	<INS:ME:LINE1>	.	PASS	SVTYPE=INS:ME:LINE1;END=51196794;SVLEN=1320;AN=20618;AC=1;AF=4.850130953535746e-05;N_HET=1;N_HOMALT=0;N_HOMREF=10308;FREQ_HET=9.700261907071492e-05;FREQ_HOMALT=0.0;FREQ_HOMREF=0.9999029973809292;N_BI_GENOS=10309;East_Han_AN=6554;East_Han_AC=1;East_Han_AF=
chr5	51209975	.	T	<INS:ME:LINE1>	.	PASS	SVTYPE=INS:ME:LINE1;END=51209975;SVLEN=738;AN=20618;AC=1;AF=4.850130953535746e-05;N_HET=1;N_HOMALT=0;N_HOMREF=10308;FREQ_HET=9.700261907071492e-05;FREQ_HOMALT=0.0;FREQ_HOMREF=0.9999029973809292;N_BI_GENOS=10309;Southeast_Han_AN=1908;Southeast_Han_AC=1;Sou
chr5	51354943	.	A	<INS:ME:LINE1>	.	PASS	SVTYPE=INS:ME:LINE1;END=51354963;SVLEN=235;AN=20618;AC=1;AF=4.850130953535746e-05;N_HET=1;N_HOMALT=0;N_HOMREF=10308;FREQ_HET=9.700261907071492e-05;FREQ_HOMALT=0.0;FREQ_HOMREF=0.9999029973809292;N_BI_GENOS=10309;East_Han_AN=6554;East_Han_AC=1;East_Han_AF=0
chr5	51452070	.	C	<INS:ME:LINE1>	.	PASS	SVTYPE=INS:ME:LINE1;END=51452086;SVLEN=5982;AN=20618;AC=1;AF=4.850130953535746e-05;N_HET=1;N_HOMALT=0;N_HOMREF=10308;FREQ_HET=9.700261907071492e-05;FREQ_HOMALT=0.0;FREQ_HOMREF=0.9999029973809292;N_BI_GENOS=10309;Tibetan_AN=414;Tibetan_AC=1;Tibetan_AF=0.00
chr5	51753935	.	T	<INS:ME:LINE1>	.	PASS	SVTYPE=INS:ME:LINE1;END=51753937;SVLEN=2913;AN=20618;AC=3;AF=0.00014550392860607236;N_HET=3;N_HOMALT=0;N_HOMREF=10306;FREQ_HET=0.0002910078572121447;FREQ_HOMALT=0.0;FREQ_HOMREF=0.9997089921427879;N_BI_GENOS=10309;East_Han_AN=6554;East_Han_AC=3;East_Han_AF
chr5	51850424	.	G	<INS:ME:LINE1>	.	PASS	SVTYPE=INS:ME:LINE1;END=51850437;SVLEN=5992;AN=20618;AC=1;AF=4.850130953535746e-05;N_HET=1;N_HOMALT=0;N_HOMREF=10308;FREQ_HET=9.700261907071492e-05;FREQ_HOMALT=0.0;FREQ_HOMREF=0.9999029973809292;N_BI_GENOS=10309;Central_Han_AN=2128;Central_Han_AC=1;Centra
chr5	52507016	.	C	<INS:ME:LINE1>	.	PASS	SVTYPE=INS:ME:LINE1;END=52507028;SVLEN=5991;AN=20618;AC=2;AF=9.700261907071492e-05;N_HET=2;N_HOMALT=0;N_HOMREF=10307;FREQ_HET=0.00019400523814142983;FREQ_HOMALT=0.0;FREQ_HOMREF=0.9998059947618586;N_BI_GENOS=10309;Tibetan_AN=414;Tibetan_AC=2;Tibetan_AF=0.0
chr5	52509540	.	A	<INS:ME:LINE1>	.	PASS	SVTYPE=INS:ME:LINE1;END=52509549;SVLEN=2005;AN=20618;AC=12;AF=0.0005820157144242894;N_HET=12;N_HOMALT=0;N_HOMREF=10297;FREQ_HET=0.0011640314288485789;FREQ_HOMALT=0.0;FREQ_HOMREF=0.9988359685711514;N_BI_GENOS=10309;North_Han_AN=2272;North_Han_AC=3;North_Ha
chr5	52836122	.	A	<INS:ME:LINE1>	.	PASS	SVTYPE=INS:ME:LINE1;END=52836139;SVLEN=5998;AN=20618;AC=71;AF=0.0034435929770103794;N_HET=69;N_HOMALT=1;N_HOMREF=10239;FREQ_HET=0.006693180715879329;FREQ_HOMALT=9.700261907071492e-05;FREQ_HOMREF=0.9932098166650499;N_BI_GENOS=10309;South_Han_AN=1496;South_
chr5	52843943	.	T	<INS:ME:LINE1>	.	PASS	SVTYPE=INS:ME:LINE1;END=52843958;SVLEN=5990;AN=20618;AC=1;AF=4.850130953535746e-05;N_HET=1;N_HOMALT=0;N_HOMREF=10308;FREQ_HET=9.700261907071492e-05;FREQ_HOMALT=0.0;FREQ_HOMREF=0.9999029973809292;N_BI_GENOS=10309;Central_Han_AN=2128;Central_Han_AC=1;Centra
chr5	52932737	.	A	<INS:ME:LINE1>	.	PASS	SVTYPE=INS:ME:LINE1;END=52932751;SVLEN=566;AN=20618;AC=3;AF=0.00014550392860607236;N_HET=3;N_HOMALT=0;N_HOMREF=10306;FREQ_HET=0.0002910078572121447;FREQ_HOMALT=0.0;FREQ_HOMREF=0.9997089921427879;N_BI_GENOS=10309;North_Han_AN=2272;North_Han_AC=1;North_Han_
chr5	53002105	.	T	<INS:ME:LINE1>	.	PASS	SVTYPE=INS:ME:LINE1;END=53002109;SVLEN=3467;AN=20618;AC=1;AF=4.850130953535746e-05;N_HET=1;N_HOMALT=0;N_HOMREF=10308;FREQ_HET=9.700261907071492e-05;FREQ_HOMALT=0.0;FREQ_HOMREF=0.9999029973809292;N_BI_GENOS=10309;Central_Han_AN=2128;Central_Han_AC=1;Centra
chr5	53296545	.	T	<INS:ME:LINE1>	.	PASS	SVTYPE=INS:ME:LINE1;END=53296561;SVLEN=6011;AN=20618;AC=3;AF=0.00014550392860607236;N_HET=1;N_HOMALT=1;N_HOMREF=10307;FREQ_HET=9.700261907071492e-05;FREQ_HOMALT=9.700261907071492e-05;FREQ_HOMREF=0.9998059947618586;N_BI_GENOS=10309;North_Han_AN=2272;North_
chr5	53299884	.	G	<INS:ME:LINE1>	.	PASS	SVTYPE=INS:ME:LINE1;END=53299902;SVLEN=1080;AN=20618;AC=4;AF=0.00019400523814142983;N_HET=4;N_HOMALT=0;N_HOMREF=10305;FREQ_HET=0.00038801047628285966;FREQ_HOMALT=0.0;FREQ_HOMREF=0.9996119895237171;N_BI_GENOS=10309;Northwest_Han_AN=1296;Northwest_Han_AC=3;
chr5	53334966	.	G	<INS:ME:LINE1>	.	PASS	SVTYPE=INS:ME:LINE1;END=53334981;SVLEN=117;AN=20618;AC=1;AF=4.850130953535746e-05;N_HET=1;N_HOMALT=0;N_HOMREF=10308;FREQ_HET=9.700261907071492e-05;FREQ_HOMALT=0.0;FREQ_HOMREF=0.9999029973809292;N_BI_GENOS=10309;East_Han_AN=6554;East_Han_AC=1;East_Han_AF=0
chr5	53372831	.	G	<INS:ME:LINE1>	.	PASS	SVTYPE=INS:ME:LINE1;END=53372831;SVLEN=242;AN=20618;AC=1;AF=4.850130953535746e-05;N_HET=1;N_HOMALT=0;N_HOMREF=10308;FREQ_HET=9.700261907071492e-05;FREQ_HOMALT=0.0;FREQ_HOMREF=0.9999029973809292;N_BI_GENOS=10309;East_Han_AN=6554;East_Han_AC=1;East_Han_AF=0
chr5	53373457	.	C	<INS:ME:LINE1>	.	PASS	SVTYPE=INS:ME:LINE1;END=53373457;SVLEN=493;AN=20618;AC=1;AF=4.850130953535746e-05;N_HET=1;N_HOMALT=0;N_HOMREF=10308;FREQ_HET=9.700261907071492e-05;FREQ_HOMALT=0.0;FREQ_HOMREF=0.9999029973809292;N_BI_GENOS=10309;East_Han_AN=6554;East_Han_AC=1;East_Han_AF=0
chr5	53974230	.	A	<INS:ME:LINE1>	.	PASS	SVTYPE=INS:ME:LINE1;END=53974241;SVLEN=6017;AN=20618;AC=1;AF=4.850130953535746e-05;N_HET=1;N_HOMALT=0;N_HOMREF=10308;FREQ_HET=9.700261907071492e-05;FREQ_HOMALT=0.0;FREQ_HOMREF=0.9999029973809292;N_BI_GENOS=10309;East_Han_AN=6554;East_Han_AC=1;East_Han_AF=
chr5	53978487	.	A	<INS:ME:LINE1>	.	PASS	SVTYPE=INS:ME:LINE1;END=53978500;SVLEN=5983;AN=20618;AC=2;AF=9.700261907071492e-05;N_HET=2;N_HOMALT=0;N_HOMREF=10307;FREQ_HET=0.00019400523814142983;FREQ_HOMALT=0.0;FREQ_HOMREF=0.9998059947618586;N_BI_GENOS=10309;East_Han_AN=6554;East_Han_AC=1;East_Han_AF
chr5	54384399	.	G	<INS:ME:LINE1>	.	PASS	SVTYPE=INS:ME:LINE1;END=54384413;SVLEN=334;AN=20618;AC=1;AF=4.850130953535746e-05;N_HET=1;N_HOMALT=0;N_HOMREF=10308;FREQ_HET=9.700261907071492e-05;FREQ_HOMALT=0.0;FREQ_HOMREF=0.9999029973809292;N_BI_GENOS=10309;East_Han_AN=6554;East_Han_AC=1;East_Han_AF=0
chr5	54490290	.	A	<INS:ME:LINE1>	.	PASS	SVTYPE=INS:ME:LINE1;END=54490306;SVLEN=1558;AN=20618;AC=2;AF=9.700261907071492e-05;N_HET=2;N_HOMALT=0;N_HOMREF=10307;FREQ_HET=0.00019400523814142983;FREQ_HOMALT=0.0;FREQ_HOMREF=0.9998059947618586;N_BI_GENOS=10309;Lingnan_Han_AN=1888;Lingnan_Han_AC=1;Lingn

chr5	55758887	.	A	<INS:ME:LINE1>	.	PASS	SVTYPE=INS:ME:LINE1;END=55758887;SVLEN=5971;AN=20618;AC=6;AF=0.0002910078572121447;N_HET=0;N_HOMALT=3;N_HOMREF=10306;FREQ_HET=0.0;FREQ_HOMALT=0.0002910078572121447;FREQ_HOMREF=0.9997089921427879;N_BI_GENOS=10309;East_Han_AN=6554;East_Han_AC=4;East_Han_AF=
chr5	55897911	.	G	<INS:ME:LINE1>	.	PASS	SVTYPE=INS:ME:LINE1;END=55897926;SVLEN=308;AN=20618;AC=2;AF=9.700261907071492e-05;N_HET=2;N_HOMALT=0;N_HOMREF=10307;FREQ_HET=0.00019400523814142983;FREQ_HOMALT=0.0;FREQ_HOMREF=0.9998059947618586;N_BI_GENOS=10309;North_Han_AN=2272;North_Han_AC=1;North_Han_
chr5	56706714	.	G	<INS:ME:LINE1>	.	PASS	SVTYPE=INS:ME:LINE1;END=56706714;SVLEN=457;AN=20618;AC=1;AF=4.850130953535746e-05;N_HET=1;N_HOMALT=0;N_HOMREF=10308;FREQ_HET=9.700261907071492e-05;FREQ_HOMALT=0.0;FREQ_HOMREF=0.9999029973809292;N_BI_GENOS=10309;Tibetan_AN=414;Tibetan_AC=1;Tibetan_AF=0.002
chr5	57433154	.	A	<INS:ME:LINE1>	.	PASS	SVTYPE=INS:ME:LINE1;END=57433169;SVLEN=437;AN=20618;AC=14;AF=0.0006790183334950044;N_HET=14;N_HOMALT=0;N_HOMREF=10295;FREQ_HET=0.0013580366669900088;FREQ_HOMALT=0.0;FREQ_HOMREF=0.99864196333301;N_BI_GENOS=10309;South_Han_AN=1496;South_Han_AC=2;South_Han_A
chr5	57511455	.	G	<INS:ME:LINE1>	.	PASS	SVTYPE=INS:ME:LINE1;END=57511470;SVLEN=1534;AN=20618;AC=9;AF=0.0004365117858182171;N_HET=9;N_HOMALT=0;N_HOMREF=10300;FREQ_HET=0.0008730235716364342;FREQ_HOMALT=0.0;FREQ_HOMREF=0.9991269764283636;N_BI_GENOS=10309;South_Han_AN=1496;South_Han_AC=2;South_Han_
chr5	57556961	.	C	<INS:ME:LINE1>	.	PASS	SVTYPE=INS:ME:LINE1;END=57556961;SVLEN=0;AN=20618;AC=1;AF=4.850130953535746e-05;N_HET=1;N_HOMALT=0;N_HOMREF=10308;FREQ_HET=9.700261907071492e-05;FREQ_HOMALT=0.0;FREQ_HOMREF=0.9999029973809292;N_BI_GENOS=10309;East_Han_AN=6554;East_Han_AC=1;East_Han_AF=0.0
chr5	57766518	.	A	<INS:ME:LINE1>	.	PASS	SVTYPE=INS:ME:LINE1;END=57766535;SVLEN=4269;AN=20618;AC=10;AF=0.00048501309535357455;N_HET=10;N_HOMALT=0;N_HOMREF=10299;FREQ_HET=0.0009700261907071491;FREQ_HOMALT=0.0;FREQ_HOMREF=0.9990299738092928;N_BI_GENOS=10309;South_Han_AN=1496;South_Han_AC=1;South_H
chr5	57983689	.	A	<INS:ME:LINE1>	.	PASS	SVTYPE=INS:ME:LINE1;END=57983689;SVLEN=1239;AN=20618;AC=61;AF=0.0029585798816568047;N_HET=61;N_HOMALT=0;N_HOMREF=10248;FREQ_HET=0.005917159763313609;FREQ_HOMALT=0.0;FREQ_HOMREF=0.9940828402366864;N_BI_GENOS=10309;Northwest_Han_AN=1296;Northwest_Han_AC=5;N
chr5	57983787	.	A	<INS:ME:LINE1>	.	PASS	SVTYPE=INS:ME:LINE1;END=57983787;SVLEN=1292;AN=20618;AC=520;AF=0.025220680958385876;N_HET=402;N_HOMALT=59;N_HOMREF=9848;FREQ_HET=0.03899505286642739;FREQ_HOMALT=0.0057231545251721794;FREQ_HOMREF=0.9552817926084004;N_BI_GENOS=10309;South_Han_AN=1496;South_
chr5	58259789	.	C	<INS:ME:LINE1>	.	PASS	SVTYPE=INS:ME:LINE1;END=58259789;SVLEN=0;AN=20618;AC=1;AF=4.850130953535746e-05;N_HET=1;N_HOMALT=0;N_HOMREF=10308;FREQ_HET=9.700261907071492e-05;FREQ_HOMALT=0.0;FREQ_HOMREF=0.9999029973809292;N_BI_GENOS=10309;South_Han_AN=1496;South_Han_AC=1;South_Han_AF=
chr5	58344130	.	G	<INS:ME:LINE1>	.	PASS	SVTYPE=INS:ME:LINE1;END=58344130;SVLEN=1691;AN=20618;AC=1;AF=4.850130953535746e-05;N_HET=1;N_HOMALT=0;N_HOMREF=10308;FREQ_HET=9.700261907071492e-05;FREQ_HOMALT=0.0;FREQ_HOMREF=0.9999029973809292;N_BI_GENOS=10309;East_Han_AN=6554;East_Han_AC=1;East_Han_AF=
chr5	58498797	.	T	<INS:ME:LINE1>	.	PASS	SVTYPE=INS:ME:LINE1;END=58498810;SVLEN=322;AN=20618;AC=2;AF=9.700261907071492e-05;N_HET=2;N_HOMALT=0;N_HOMREF=10307;FREQ_HET=0.00019400523814142983;FREQ_HOMALT=0.0;FREQ_HOMREF=0.9998059947618586;N_BI_GENOS=10309;South_Han_AN=1496;South_Han_AC=1;South_Han_
chr5	58626810	.	G	<INS:ME:LINE1>	.	PASS	SVTYPE=INS:ME:LINE1;END=58626831;SVLEN=6000;AN=20618;AC=1;AF=4.850130953535746e-05;N_HET=1;N_HOMALT=0;N_HOMREF=10308;FREQ_HET=9.700261907071492e-05;FREQ_HOMALT=0.0;FREQ_HOMREF=0.9999029973809292;N_BI_GENOS=10309;Southeast_Han_AN=1908;Southeast_Han_AC=1;So
chr5	58848218	.	A	<INS:ME:LINE1>	.	PASS	SVTYPE=INS:ME:LINE1;END=58848233;SVLEN=5983;AN=20618;AC=1;AF=4.850130953535746e-05;N_HET=1;N_HOMALT=0;N_HOMREF=10308;FREQ_HET=9.700261907071492e-05;FREQ_HOMALT=0.0;FREQ_HOMREF=0.9999029973809292;N_BI_GENOS=10309;North_Han_AN=2272;North_Han_AC=1;North_Han_
chr5	58862260	.	A	<INS:ME:LINE1>	.	PASS	SVTYPE=INS:ME:LINE1;END=58862260;SVLEN=650;AN=20618;AC=1;AF=4.850130953535746e-05;N_HET=1;N_HOMALT=0;N_HOMREF=10308;FREQ_HET=9.700261907071492e-05;FREQ_HOMALT=0.0;FREQ_HOMREF=0.9999029973809292;N_BI_GENOS=10309;Southeast_Han_AN=1908;Southeast_Han_AC=1;Sou
chr5	59027247	.	A	<INS:ME:LINE1>	.	PASS	SVTYPE=INS:ME:LINE1;END=59027262;SVLEN=5985;AN=20618;AC=1;AF=4.850130953535746e-05;N_HET=1;N_HOMALT=0;N_HOMREF=10308;FREQ_HET=9.700261907071492e-05;FREQ_HOMALT=0.0;FREQ_HOMREF=0.9999029973809292;N_BI_GENOS=10309;Southeast_Han_AN=1908;Southeast_Han_AC=1;So
chr5	59253952	.	A	<INS:ME:LINE1>	.	PASS	SVTYPE=INS:ME:LINE1;END=59253965;SVLEN=2269;AN=20618;AC=11;AF=0.000533514404888932;N_HET=11;N_HOMALT=0;N_HOMREF=10298;FREQ_HET=0.001067028809777864;FREQ_HOMALT=0.0;FREQ_HOMREF=0.9989329711902222;N_BI_GENOS=10309;North_Han_AN=2272;North_Han_AC=2;North_Han_
chr5	59457533	.	A	<INS:ME:LINE1>	.	PASS	SVTYPE=INS:ME:LINE1;END=59457547;SVLEN=6077;AN=20618;AC=1;AF=4.850130953535746e-05;N_HET=1;N_HOMALT=0;N_HOMREF=10308;FREQ_HET=9.700261907071492e-05;FREQ_HOMALT=0.0;FREQ_HOMREF=0.9999029973809292;N_BI_GENOS=10309;Northwest_Han_AN=1296;Northwest_Han_AC=1;No
chr5	59533266	.	G	<INS:ME:LINE1>	.	PASS	SVTYPE=INS:ME:LINE1;END=59533270;SVLEN=1014;AN=20618;AC=1;AF=4.850130953535746e-05;N_HET=1;N_HOMALT=0;N_HOMREF=10308;FREQ_HET=9.700261907071492e-05;FREQ_HOMALT=0.0;FREQ_HOMREF=0.9999029973809292;N_BI_GENOS=10309;Northwest_Han_AN=1296;Northwest_Han_AC=1;No
chr5	59572182	.	A	<INS:ME:LINE1>	.	PASS	SVTYPE=INS:ME:LINE1;END=59572199;SVLEN=867;AN=20618;AC=1;AF=4.850130953535746e-05;N_HET=1;N_HOMALT=0;N_HOMREF=10308;FREQ_HET=9.700261907071492e-05;FREQ_HOMALT=0.0;FREQ_HOMREF=0.9999029973809292;N_BI_GENOS=10309;East_Han_AN=6554;East_Han_AC=1;East_Han_AF=0
chr5	59743675	.	A	<INS:ME:LINE1>	.	PASS	SVTYPE=INS:ME:LINE1;END=59743688;SVLEN=1636;AN=20618;AC=24;AF=0.0011640314288485789;N_HET=24;N_HOMALT=0;N_HOMREF=10285;FREQ_HET=0.0023280628576971578;FREQ_HOMALT=0.0;FREQ_HOMREF=0.9976719371423028;N_BI_GENOS=10309;South_Han_AN=1496;South_Han_AC=4;South_Ha
chr5	59991210	.	T	<INS:ME:LINE1>	.	PASS	SVTYPE=INS:ME:LINE1;END=59991211;SVLEN=3488;AN=20618;AC=2;AF=9.700261907071492e-05;N_HET=2;N_HOMALT=0;N_HOMREF=10307;FREQ_HET=0.00019400523814142983;FREQ_HOMALT=0.0;FREQ_HOMREF=0.9998059947618586;N_BI_GENOS=10309;South_Han_AN=1496;South_Han_AC=1;South_Han
chr5	60029489	.	C	<INS:ME:LINE1>	.	PASS	SVTYPE=INS:ME:LINE1;END=60029495;SVLEN=379;AN=20618;AC=1;AF=4.850130953535746e-05;N_HET=1;N_HOMALT=0;N_HOMREF=10308;FREQ_HET=9.700261907071492e-05;FREQ_HOMALT=0.0;FREQ_HOMREF=0.9999029973809292;N_BI_GENOS=10309;Southeast_Han_AN=1908;Southeast_Han_AC=1;Sou
chr5	60219221	.	A	<INS:ME:LINE1>	.	PASS	SVTYPE=INS:ME:LINE1;END=60219236;SVLEN=1328;AN=20618;AC=1;AF=4.850130953535746e-05;N_HET=1;N_HOMALT=0;N_HOMREF=10308;FREQ_HET=9.700261907071492e-05;FREQ_HOMALT=0.0;FREQ_HOMREF=0.9999029973809292;N_BI_GENOS=10309;East_Han_AN=6554;East_Han_AC=1;East_Han_AF=
chr5	60263737	.	A	<INS:ME:LINE1>	.	PASS	SVTYPE=INS:ME:LINE1;END=60263754;SVLEN=5454;AN=20618;AC=1;AF=4.850130953535746e-05;N_HET=1;N_HOMALT=0;N_HOMREF=10308;FREQ_HET=9.700261907071492e-05;FREQ_HOMALT=0.0;FREQ_HOMREF=0.9999029973809292;N_BI_GENOS=10309;North_Han_AN=2272;North_Han_AC=1;North_Han_
chr5	60295123	.	A	<INS:ME:LINE1>	.	PASS	SVTYPE=INS:ME:LINE1;END=60295124;SVLEN=2980;AN=20618;AC=251;AF=0.012173828693374722;N_HET=239;N_HOMALT=6;N_HOMREF=10064;FREQ_HET=0.023183625957900862;FREQ_HOMALT=0.0005820157144242894;FREQ_HOMREF=0.9762343583276748;N_BI_GENOS=10309;South_Han_AN=1496;South
chr5	60354123	.	G	<INS:ME:LINE1>	.	PASS	SVTYPE=INS:ME:LINE1;END=60354133;SVLEN=440;AN=20618;AC=2;AF=9.700261907071492e-05;N_HET=2;N_HOMALT=0;N_HOMREF=10307;FREQ_HET=0.00019400523814142983;FREQ_HOMALT=0.0;FREQ_HOMREF=0.9998059947618586;N_BI_GENOS=10309;Central_Han_AN=2128;Central_Han_AC=1;Centra
chr5	60506381	.	A	<INS:ME:LINE1>	.	PASS	SVTYPE=INS:ME:LINE1;END=60506390;SVLEN=6069;AN=20618;AC=2;AF=9.700261907071492e-05;N_HET=2;N_HOMALT=0;N_HOMREF=10307;FREQ_HET=0.00019400523814142983;FREQ_HOMALT=0.0;FREQ_HOMREF=0.9998059947618586;N_BI_GENOS=10309;North_Han_AN=2272;North_Han_AC=2;North_Han
chr5	60520103	.	C	<INS:ME:LINE1>	.	PASS	SVTYPE=INS:ME:LINE1;END=60520115;SVLEN=1349;AN=20618;AC=130;AF=0.006305170239596469;N_HET=126;N_HOMALT=2;N_HOMREF=10181;FREQ_HET=0.012222330002910078;FREQ_HOMALT=0.00019400523814142983;FREQ_HOMREF=0.9875836647589485;N_BI_GENOS=10309;South_Han_AN=1496;Sout
chr5	60763888	.	A	<INS:ME:LINE1>	.	PASS	SVTYPE=INS:ME:LINE1;END=60763904;SVLEN=1218;AN=20618;AC=1;AF=4.850130953535746e-05;N_HET=1;N_HOMALT=0;N_HOMREF=10308;FREQ_HET=9.700261907071492e-05;FREQ_HOMALT=0.0;FREQ_HOMREF=0.9999029973809292;N_BI_GENOS=10309;Zhuang_AN=444;Zhuang_AC=1;Zhuang_AF=0.00225
chr5	60875403	.	A	<INS:ME:LINE1>	.	PASS	SVTYPE=INS:ME:LINE1;END=60875410;SVLEN=1441;AN=20618;AC=5;AF=0.00024250654767678728;N_HET=5;N_HOMALT=0;N_HOMREF=10304;FREQ_HET=0.00048501309535357455;FREQ_HOMALT=0.0;FREQ_HOMREF=0.9995149869046465;N_BI_GENOS=10309;South_Han_AN=1496;South_Han_AC=1;South_Ha
chr5	60948511	.	A	<INS:ME:LINE1>	.	PASS	SVTYPE=INS:ME:LINE1;END=60948511;SVLEN=2098;AN=20618;AC=1;AF=4.850130953535746e-05;N_HET=1;N_HOMALT=0;N_HOMREF=10308;FREQ_HET=9.700261907071492e-05;FREQ_HOMALT=0.0;FREQ_HOMREF=0.9999029973809292;N_BI_GENOS=10309;Southeast_Han_AN=1908;Southeast_Han_AC=1;So
chr5	61080697	.	A	<INS:ME:LINE1>	.	PASS	SVTYPE=INS:ME:LINE1;END=61080713;SVLEN=1270;AN=20618;AC=1;AF=4.850130953535746e-05;N_HET=1;N_HOMALT=0;N_HOMREF=10308;FREQ_HET=9.700261907071492e-05;FREQ_HOMALT=0.0;FREQ_HOMREF=0.9999029973809292;N_BI_GENOS=10309;Yi_AN=422;Yi_AC=1;Yi_AF=0.00236966824644549
chr5	61103492	.	A	<INS:ME:LINE1>	.	PASS	SVTYPE=INS:ME:LINE1;END=61103508;SVLEN=217;AN=20618;AC=2;AF=9.700261907071492e-05;N_HET=2;N_HOMALT=0;N_HOMREF=10307;FREQ_HET=0.00019400523814142983;FREQ_HOMALT=0.0;FREQ_HOMREF=0.9998059947618586;N_BI_GENOS=10309;South_Han_AN=1496;South_Han_AC=1;South_Han_
chr5	61249454	.	C	<INS:ME:LINE1>	.	PASS	SVTYPE=INS:ME:LINE1;END=61249470;SVLEN=393;AN=20618;AC=2;AF=9.700261907071492e-05;N_HET=2;N_HOMALT=0;N_HOMREF=10307;FREQ_HET=0.00019400523814142983;FREQ_HOMALT=0.0;FREQ_HOMREF=0.9998059947618586;N_BI_GENOS=10309;Lingnan_Han_AN=1888;Lingnan_Han_AC=1;Lingna
chr5	61351474	.	T	<INS:ME:LINE1>	.	PASS	SVTYPE=INS:ME:LINE1;END=61351485;SVLEN=6029;AN=20618;AC=1;AF=4.850130953535746e-05;N_HET=1;N_HOMALT=0;N_HOMREF=10308;FREQ_HET=9.700261907071492e-05;FREQ_HOMALT=0.0;FREQ_HOMREF=0.9999029973809292;N_BI_GENOS=10309;Mongolian_AN=454;Mongolian_AC=1;Mongolian_A
chr5	61691564	.	A	<INS:ME:LINE1>	.	PASS	SVTYPE=INS:ME:LINE1;END=61691580;SVLEN=654;AN=20618;AC=1;AF=4.850130953535746e-05;N_HET=1;N_HOMALT=0;N_HOMREF=10308;FREQ_HET=9.700261907071492e-05;FREQ_HOMALT=0.0;FREQ_HOMREF=0.9999029973809292;N_BI_GENOS=10309;Northwest_Han_AN=1296;Northwest_Han_AC=1;Nor
chr5	61749107	.	A	<INS:ME:LINE1>	.	PASS	SVTYPE=INS:ME:LINE1;END=61749123;SVLEN=751;AN=20618;AC=1;AF=4.850130953535746e-05;N_HET=1;N_HOMALT=0;N_HOMREF=10308;FREQ_HET=9.700261907071492e-05;FREQ_HOMALT=0.0;FREQ_HOMREF=0.9999029973809292;N_BI_GENOS=10309;North_Han_AN=2272;North_Han_AC=1;North_Han_A
chr5	61859350	.	A	<INS:ME:LINE1>	.	PASS	SVTYPE=INS:ME:LINE1;END=61859365;SVLEN=6063;AN=20618;AC=1;AF=4.850130953535746e-05;N_HET=1;N_HOMALT=0;N_HOMREF=10308;FREQ_HET=9.700261907071492e-05;FREQ_HOMALT=0.0;FREQ_HOMREF=0.9999029973809292;N_BI_GENOS=10309;East_Han_AN=6554;East_Han_AC=1;East_Han_AF=
chr5	61906637	.	A	<INS:ME:LINE1>	.	PASS	SVTYPE=INS:ME:LINE1;END=61906656;SVLEN=5992;AN=20618;AC=1;AF=4.850130953535746e-05;N_HET=1;N_HOMALT=0;N_HOMREF=10308;FREQ_HET=9.700261907071492e-05;FREQ_HOMALT=0.0;FREQ_HOMREF=0.9999029973809292;N_BI_GENOS=10309;Central_Han_AN=2128;Central_Han_AC=1;Centra
chr5	62125949	.	A	<INS:ME:LINE1>	.	PASS	SVTYPE=INS:ME:LINE1;END=62125965;SVLEN=1694;AN=20618;AC=1;AF=4.850130953535746e-05;N_HET=1;N_HOMALT=0;N_HOMREF=10308;FREQ_HET=9.700261907071492e-05;FREQ_HOMALT=0.0;FREQ_HOMREF=0.9999029973809292;N_BI_GENOS=10309;North_Han_AN=2272;North_Han_AC=1;North_Han_
chr5	62170579	.	T	<INS:ME:LINE1>	.	PASS	SVTYPE=INS:ME:LINE1;END=62170592;SVLEN=5993;AN=20618;AC=1;AF=4.850130953535746e-05;N_HET=1;N_HOMALT=0;N_HOMREF=10308;FREQ_HET=9.700261907071492e-05;FREQ_HOMALT=0.0;FREQ_HOMREF=0.9999029973809292;N_BI_GENOS=10309;Central_Han_AN=2128;Central_Han_AC=1;Centra
chr5	62779295	.	A	<INS:ME:LINE1>	.	PASS	SVTYPE=INS:ME:LINE1;END=62779311;SVLEN=928;AN=20618;AC=1;AF=4.850130953535746e-05;N_HET=1;N_HOMALT=0;N_HOMREF=10308;FREQ_HET=9.700261907071492e-05;FREQ_HOMALT=0.0;FREQ_HOMREF=0.9999029973809292;N_BI_GENOS=10309;East_Han_AN=6554;East_Han_AC=1;East_Han_AF=0
chr5	62847545	.	G	<INS:ME:LINE1>	.	PASS	SVTYPE=INS:ME:LINE1;END=62847566;SVLEN=1945;AN=20618;AC=2;AF=9.700261907071492e-05;N_HET=2;N_HOMALT=0;N_HOMREF=10307;FREQ_HET=0.00019400523814142983;FREQ_HOMALT=0.0;FREQ_HOMREF=0.9998059947618586;N_BI_GENOS=10309;East_Han_AN=6554;East_Han_AC=2;East_Han_AF
chr5	63022467	.	C	<INS:ME:LINE1>	.	PASS	SVTYPE=INS:ME:LINE1;END=63022486;SVLEN=2731;AN=20618;AC=2;AF=9.700261907071492e-05;N_HET=2;N_HOMALT=0;N_HOMREF=10307;FREQ_HET=0.00019400523814142983;FREQ_HOMALT=0.0;FREQ_HOMREF=0.9998059947618586;N_BI_GENOS=10309;East_Han_AN=6554;East_Han_AC=2;East_Han_AF
chr5	63169404	.	T	<INS:ME:LINE1>	.	PASS	SVTYPE=INS:ME:LINE1;END=63169421;SVLEN=232;AN=20618;AC=1;AF=4.850130953535746e-05;N_HET=1;N_HOMALT=0;N_HOMREF=10308;FREQ_HET=9.700261907071492e-05;FREQ_HOMALT=0.0;FREQ_HOMREF=0.9999029973809292;N_BI_GENOS=10309;East_Han_AN=6554;East_Han_AC=1;East_Han_AF=0
chr5	63462191	.	A	<INS:ME:LINE1>	.	PASS	SVTYPE=INS:ME:LINE1;END=63462207;SVLEN=1409;AN=20618;AC=1;AF=4.850130953535746e-05;N_HET=1;N_HOMALT=0;N_HOMREF=10308;FREQ_HET=9.700261907071492e-05;FREQ_HOMALT=0.0;FREQ_HOMREF=0.9999029973809292;N_BI_GENOS=10309;East_Han_AN=6554;East_Han_AC=1;East_Han_AF=
chr5	63495564	.	T	<INS:ME:LINE1>	.	PASS	SVTYPE=INS:ME:LINE1;END=63495575;SVLEN=5998;AN=20618;AC=2;AF=9.700261907071492e-05;N_HET=2;N_HOMALT=0;N_HOMREF=10307;FREQ_HET=0.00019400523814142983;FREQ_HOMALT=0.0;FREQ_HOMREF=0.9998059947618586;N_BI_GENOS=10309;East_Han_AN=6554;East_Han_AC=1;East_Han_AF
chr5	63671665	.	C	<INS:ME:LINE1>	.	PASS	SVTYPE=INS:ME:LINE1;END=63671665;SVLEN=1235;AN=20618;AC=2;AF=9.700261907071492e-05;N_HET=0;N_HOMALT=1;N_HOMREF=10308;FREQ_HET=0.0;FREQ_HOMALT=9.700261907071492e-05;FREQ_HOMREF=0.9999029973809292;N_BI_GENOS=10309;East_Han_AN=6554;East_Han_AC=2;East_Han_AF=
chr5	63709999	.	G	<INS:ME:LINE1>	.	PASS	SVTYPE=INS:ME:LINE1;END=63710008;SVLEN=1437;AN=20618;AC=1;AF=4.850130953535746e-05;N_HET=1;N_HOMALT=0;N_HOMREF=10308;FREQ_HET=9.700261907071492e-05;FREQ_HOMALT=0.0;FREQ_HOMREF=0.9999029973809292;N_BI_GENOS=10309;Southeast_Han_AN=1908;Southeast_Han_AC=1;So
chr5	63784748	.	T	<INS:ME:LINE1>	.	PASS	SVTYPE=INS:ME:LINE1;END=63784751;SVLEN=181;AN=20618;AC=1;AF=4.850130953535746e-05;N_HET=1;N_HOMALT=0;N_HOMREF=10308;FREQ_HET=9.700261907071492e-05;FREQ_HOMALT=0.0;FREQ_HOMREF=0.9999029973809292;N_BI_GENOS=10309;Miao_AN=482;Miao_AC=1;Miao_AF=0.002074688796
chr5	64280178	.	T	<INS:ME:LINE1>	.	PASS	SVTYPE=INS:ME:LINE1;END=64280197;SVLEN=6029;AN=20618;AC=1;AF=4.850130953535746e-05;N_HET=1;N_HOMALT=0;N_HOMREF=10308;FREQ_HET=9.700261907071492e-05;FREQ_HOMALT=0.0;FREQ_HOMREF=0.9999029973809292;N_BI_GENOS=10309;South_Han_AN=1496;South_Han_AC=1;South_Han_
chr5	64593702	.	C	<INS:ME:LINE1>	.	PASS	SVTYPE=INS:ME:LINE1;END=64593702;SVLEN=458;AN=20618;AC=1;AF=4.850130953535746e-05;N_HET=1;N_HOMALT=0;N_HOMREF=10308;FREQ_HET=9.700261907071492e-05;FREQ_HOMALT=0.0;FREQ_HOMREF=0.9999029973809292;N_BI_GENOS=10309;East_Han_AN=6554;East_Han_AC=1;East_Han_AF=0
chr5	64751674	.	A	<INS:ME:LINE1>	.	PASS	SVTYPE=INS:ME:LINE1;END=64751690;SVLEN=839;AN=20618;AC=1;AF=4.850130953535746e-05;N_HET=1;N_HOMALT=0;N_HOMREF=10308;FREQ_HET=9.700261907071492e-05;FREQ_HOMALT=0.0;FREQ_HOMREF=0.9999029973809292;N_BI_GENOS=10309;Central_Han_AN=2128;Central_Han_AC=1;Central
chr5	65385565	.	A	<INS:ME:LINE1>	.	PASS	SVTYPE=INS:ME:LINE1;END=65385575;SVLEN=6006;AN=20618;AC=1;AF=4.850130953535746e-05;N_HET=1;N_HOMALT=0;N_HOMREF=10308;FREQ_HET=9.700261907071492e-05;FREQ_HOMALT=0.0;FREQ_HOMREF=0.9999029973809292;N_BI_GENOS=10309;East_Han_AN=6554;East_Han_AC=1;East_Han_AF=
chr5	65833139	.	A	<INS:ME:LINE1>	.	PASS	SVTYPE=INS:ME:LINE1;END=65833155;SVLEN=768;AN=20618;AC=1;AF=4.850130953535746e-05;N_HET=1;N_HOMALT=0;N_HOMREF=10308;FREQ_HET=9.700261907071492e-05;FREQ_HOMALT=0.0;FREQ_HOMREF=0.9999029973809292;N_BI_GENOS=10309;Southeast_Han_AN=1908;Southeast_Han_AC=1;Sou
chr5	66353828	.	G	<INS:ME:LINE1>	.	PASS	SVTYPE=INS:ME:LINE1;END=66353842;SVLEN=217;AN=20618;AC=1;AF=4.850130953535746e-05;N_HET=1;N_HOMALT=0;N_HOMREF=10308;FREQ_HET=9.700261907071492e-05;FREQ_HOMALT=0.0;FREQ_HOMREF=0.9999029973809292;N_BI_GENOS=10309;East_Han_AN=6554;East_Han_AC=1;East_Han_AF=0
chr5	66456144	.	A	<INS:ME:LINE1>	.	PASS	SVTYPE=INS:ME:LINE1;END=66456158;SVLEN=5490;AN=20618;AC=4;AF=0.00019400523814142983;N_HET=4;N_HOMALT=0;N_HOMREF=10305;FREQ_HET=0.00038801047628285966;FREQ_HOMALT=0.0;FREQ_HOMREF=0.9996119895237171;N_BI_GENOS=10309;South_Han_AN=1496;South_Han_AC=2;South_Ha
chr5	66809298	.	C	<INS:ME:LINE1>	.	PASS	SVTYPE=INS:ME:LINE1;END=66809298;SVLEN=2382;AN=20618;AC=1;AF=4.850130953535746e-05;N_HET=1;N_HOMALT=0;N_HOMREF=10308;FREQ_HET=9.700261907071492e-05;FREQ_HOMALT=0.0;FREQ_HOMREF=0.9999029973809292;N_BI_GENOS=10309;East_Han_AN=6554;East_Han_AC=1;East_Han_AF=
chr5	66944658	.	T	<INS:ME:LINE1>	.	PASS	SVTYPE=INS:ME:LINE1;END=66944667;SVLEN=451;AN=20618;AC=1;AF=4.850130953535746e-05;N_HET=1;N_HOMALT=0;N_HOMREF=10308;FREQ_HET=9.700261907071492e-05;FREQ_HOMALT=0.0;FREQ_HOMREF=0.9999029973809292;N_BI_GENOS=10309;North_Han_AN=2272;North_Han_AC=1;North_Han_A
chr5	67146201	.	T	<INS:ME:LINE1>	.	PASS	SVTYPE=INS:ME:LINE1;END=67146204;SVLEN=107;AN=20618;AC=1;AF=4.850130953535746e-05;N_HET=1;N_HOMALT=0;N_HOMREF=10308;FREQ_HET=9.700261907071492e-05;FREQ_HOMALT=0.0;FREQ_HOMREF=0.9999029973809292;N_BI_GENOS=10309;Zhuang_AN=444;Zhuang_AC=1;Zhuang_AF=0.002252
chr5	67346078	.	A	<INS:ME:LINE1>	.	PASS	SVTYPE=INS:ME:LINE1;END=67346087;SVLEN=561;AN=20618;AC=1;AF=4.850130953535746e-05;N_HET=1;N_HOMALT=0;N_HOMREF=10308;FREQ_HET=9.700261907071492e-05;FREQ_HOMALT=0.0;FREQ_HOMREF=0.9999029973809292;N_BI_GENOS=10309;Southeast_Han_AN=1908;Southeast_Han_AC=1;Sou
chr5	67697274	.	G	<INS:ME:LINE1>	.	PASS	SVTYPE=INS:ME:LINE1;END=67697292;SVLEN=5930;AN=20618;AC=1;AF=4.850130953535746e-05;N_HET=1;N_HOMALT=0;N_HOMREF=10308;FREQ_HET=9.700261907071492e-05;FREQ_HOMALT=0.0;FREQ_HOMREF=0.9999029973809292;N_BI_GENOS=10309;Northwest_Han_AN=1296;Northwest_Han_AC=1;No
chr5	67735116	.	A	<INS:ME:LINE1>	.	PASS	SVTYPE=INS:ME:LINE1;END=67735116;SVLEN=1522;AN=20618;AC=1;AF=4.850130953535746e-05;N_HET=1;N_HOMALT=0;N_HOMREF=10308;FREQ_HET=9.700261907071492e-05;FREQ_HOMALT=0.0;FREQ_HOMREF=0.9999029973809292;N_BI_GENOS=10309;East_Han_AN=6554;East_Han_AC=1;East_Han_AF=
chr5	67826731	.	T	<INS:ME:LINE1>	.	PASS	SVTYPE=INS:ME:LINE1;END=67826731;SVLEN=1843;AN=20618;AC=1;AF=4.850130953535746e-05;N_HET=1;N_HOMALT=0;N_HOMREF=10308;FREQ_HET=9.700261907071492e-05;FREQ_HOMALT=0.0;FREQ_HOMREF=0.9999029973809292;N_BI_GENOS=10309;Zhuang_AN=444;Zhuang_AC=1;Zhuang_AF=0.00225
chr5	67870588	.	T	<INS:ME:LINE1>	.	PASS	SVTYPE=INS:ME:LINE1;END=67870603;SVLEN=712;AN=20618;AC=1;AF=4.850130953535746e-05;N_HET=1;N_HOMALT=0;N_HOMREF=10308;FREQ_HET=9.700261907071492e-05;FREQ_HOMALT=0.0;FREQ_HOMREF=0.9999029973809292;N_BI_GENOS=10309;Central_Han_AN=2128;Central_Han_AC=1;Central
chr5	67913597	.	T	<INS:ME:LINE1>	.	PASS	SVTYPE=INS:ME:LINE1;END=67913600;SVLEN=656;AN=20618;AC=1;AF=4.850130953535746e-05;N_HET=1;N_HOMALT=0;N_HOMREF=10308;FREQ_HET=9.700261907071492e-05;FREQ_HOMALT=0.0;FREQ_HOMREF=0.9999029973809292;N_BI_GENOS=10309;East_Han_AN=6554;East_Han_AC=1;East_Han_AF=0
chr5	67993078	.	A	<INS:ME:LINE1>	.	PASS	SVTYPE=INS:ME:LINE1;END=67993089;SVLEN=6031;AN=20618;AC=1;AF=4.850130953535746e-05;N_HET=1;N_HOMALT=0;N_HOMREF=10308;FREQ_HET=9.700261907071492e-05;FREQ_HOMALT=0.0;FREQ_HOMREF=0.9999029973809292;N_BI_GENOS=10309;North_Han_AN=2272;North_Han_AC=1;North_Han_
chr5	68007145	.	G	<INS:ME:LINE1>	.	PASS	SVTYPE=INS:ME:LINE1;END=68007154;SVLEN=6007;AN=20618;AC=1;AF=4.850130953535746e-05;N_HET=1;N_HOMALT=0;N_HOMREF=10308;FREQ_HET=9.700261907071492e-05;FREQ_HOMALT=0.0;FREQ_HOMREF=0.9999029973809292;N_BI_GENOS=10309;Central_Han_AN=2128;Central_Han_AC=1;Centra
chr5	68162227	.	A	<INS:ME:LINE1>	.	PASS	SVTYPE=INS:ME:LINE1;END=68162239;SVLEN=6041;AN=20618;AC=2;AF=9.700261907071492e-05;N_HET=2;N_HOMALT=0;N_HOMREF=10307;FREQ_HET=0.00019400523814142983;FREQ_HOMALT=0.0;FREQ_HOMREF=0.9998059947618586;N_BI_GENOS=10309;North_Han_AN=2272;North_Han_AC=1;North_Han
chr5	68516721	.	G	<INS:ME:LINE1>	.	PASS	SVTYPE=INS:ME:LINE1;END=68516736;SVLEN=908;AN=20618;AC=1;AF=4.850130953535746e-05;N_HET=1;N_HOMALT=0;N_HOMREF=10308;FREQ_HET=9.700261907071492e-05;FREQ_HOMALT=0.0;FREQ_HOMREF=0.9999029973809292;N_BI_GENOS=10309;Lingnan_Han_AN=1888;Lingnan_Han_AC=1;Lingnan
chr5	68597678	.	T	<INS:ME:LINE1>	.	PASS	SVTYPE=INS:ME:LINE1;END=68597682;SVLEN=260;AN=20618;AC=1;AF=4.850130953535746e-05;N_HET=1;N_HOMALT=0;N_HOMREF=10308;FREQ_HET=9.700261907071492e-05;FREQ_HOMALT=0.0;FREQ_HOMREF=0.9999029973809292;N_BI_GENOS=10309;East_Han_AN=6554;East_Han_AC=1;East_Han_AF=0
chr5	68829324	.	A	<INS:ME:LINE1>	.	PASS	SVTYPE=INS:ME:LINE1;END=68829336;SVLEN=5476;AN=20618;AC=9;AF=0.0004365117858182171;N_HET=9;N_HOMALT=0;N_HOMREF=10300;FREQ_HET=0.0008730235716364342;FREQ_HOMALT=0.0;FREQ_HOMREF=0.9991269764283636;N_BI_GENOS=10309;Northwest_Han_AN=1296;Northwest_Han_AC=1;No
chr5	68958138	.	A	<INS:ME:LINE1>	.	PASS	SVTYPE=INS:ME:LINE1;END=68958149;SVLEN=2892;AN=20618;AC=2;AF=9.700261907071492e-05;N_HET=2;N_HOMALT=0;N_HOMREF=10307;FREQ_HET=0.00019400523814142983;FREQ_HOMALT=0.0;FREQ_HOMREF=0.9998059947618586;N_BI_GENOS=10309;Lingnan_Han_AN=1888;Lingnan_Han_AC=2;Lingn
chr5	68967633	.	T	<INS:ME:LINE1>	.	PASS	SVTYPE=INS:ME:LINE1;END=68967648;SVLEN=566;AN=20618;AC=2339;AF=0.11344456300320109;N_HET=2021;N_HOMALT=159;N_HOMREF=8129;FREQ_HET=0.19604229314191482;FREQ_HOMALT=0.01542341643224367;FREQ_HOMREF=0.7885342904258414;N_BI_GENOS=10309;South_Han_AN=1496;South_H
chr5	71928668	.	G	<INS:ME:LINE1>	.	PASS	SVTYPE=INS:ME:LINE1;END=71928684;SVLEN=2559;AN=20618;AC=1;AF=4.850130953535746e-05;N_HET=1;N_HOMALT=0;N_HOMREF=10308;FREQ_HET=9.700261907071492e-05;FREQ_HOMALT=0.0;FREQ_HOMREF=0.9999029973809292;N_BI_GENOS=10309;North_Han_AN=2272;North_Han_AC=1;North_Han_
chr5	73128654	.	T	<INS:ME:LINE1>	.	PASS	SVTYPE=INS:ME:LINE1;END=73128669;SVLEN=6015;AN=20618;AC=3;AF=0.00014550392860607236;N_HET=1;N_HOMALT=1;N_HOMREF=10307;FREQ_HET=9.700261907071492e-05;FREQ_HOMALT=9.700261907071492e-05;FREQ_HOMREF=0.9998059947618586;N_BI_GENOS=10309;North_Han_AN=2272;North_
chr5	73321012	.	A	<INS:ME:LINE1>	.	PASS	SVTYPE=INS:ME:LINE1;END=73321026;SVLEN=5998;AN=20618;AC=1;AF=4.850130953535746e-05;N_HET=1;N_HOMALT=0;N_HOMREF=10308;FREQ_HET=9.700261907071492e-05;FREQ_HOMALT=0.0;FREQ_HOMREF=0.9999029973809292;N_BI_GENOS=10309;East_Han_AN=6554;East_Han_AC=1;East_Han_AF=
chr5	73595444	.	G	<INS:ME:LINE1>	.	PASS	SVTYPE=INS:ME:LINE1;END=73595459;SVLEN=6000;AN=20618;AC=2;AF=9.700261907071492e-05;N_HET=2;N_HOMALT=0;N_HOMREF=10307;FREQ_HET=0.00019400523814142983;FREQ_HOMALT=0.0;FREQ_HOMREF=0.9998059947618586;N_BI_GENOS=10309;Lingnan_Han_AN=1888;Lingnan_Han_AC=1;Lingn
chr5	73602911	.	T	<INS:ME:LINE1>	.	PASS	SVTYPE=INS:ME:LINE1;END=73602913;SVLEN=2309;AN=20618;AC=1;AF=4.850130953535746e-05;N_HET=1;N_HOMALT=0;N_HOMREF=10308;FREQ_HET=9.700261907071492e-05;FREQ_HOMALT=0.0;FREQ_HOMREF=0.9999029973809292;N_BI_GENOS=10309;East_Han_AN=6554;East_Han_AC=1;East_Han_AF=
chr5	73854024	.	T	<INS:ME:LINE1>	.	PASS	SVTYPE=INS:ME:LINE1;END=73854041;SVLEN=1482;AN=20618;AC=71;AF=0.0034435929770103794;N_HET=71;N_HOMALT=0;N_HOMREF=10238;FREQ_HET=0.006887185954020759;FREQ_HOMALT=0.0;FREQ_HOMREF=0.9931128140459793;N_BI_GENOS=10309;South_Han_AN=1496;South_Han_AC=7;South_Han
chr5	73979579	.	A	<INS:ME:LINE1>	.	PASS	SVTYPE=INS:ME:LINE1;END=73979585;SVLEN=760;AN=20618;AC=1;AF=4.850130953535746e-05;N_HET=1;N_HOMALT=0;N_HOMREF=10308;FREQ_HET=9.700261907071492e-05;FREQ_HOMALT=0.0;FREQ_HOMREF=0.9999029973809292;N_BI_GENOS=10309;Central_Han_AN=2128;Central_Han_AC=1;Central
chr5	73997118	.	A	<INS:ME:LINE1>	.	PASS	SVTYPE=INS:ME:LINE1;END=73997137;SVLEN=961;AN=20618;AC=12;AF=0.0005820157144242894;N_HET=12;N_HOMALT=0;N_HOMREF=10297;FREQ_HET=0.0011640314288485789;FREQ_HOMALT=0.0;FREQ_HOMREF=0.9988359685711514;N_BI_GENOS=10309;South_Han_AN=1496;South_Han_AC=1;South_Han
chr5	74072319	.	C	<INS:ME:LINE1>	.	PASS	SVTYPE=INS:ME:LINE1;END=74072334;SVLEN=6006;AN=20618;AC=1;AF=4.850130953535746e-05;N_HET=1;N_HOMALT=0;N_HOMREF=10308;FREQ_HET=9.700261907071492e-05;FREQ_HOMALT=0.0;FREQ_HOMREF=0.9999029973809292;N_BI_GENOS=10309;Tibetan_AN=414;Tibetan_AC=1;Tibetan_AF=0.00
chr5	74188919	.	A	<INS:ME:LINE1>	.	PASS	SVTYPE=INS:ME:LINE1;END=74188933;SVLEN=867;AN=20618;AC=1;AF=4.850130953535746e-05;N_HET=1;N_HOMALT=0;N_HOMREF=10308;FREQ_HET=9.700261907071492e-05;FREQ_HOMALT=0.0;FREQ_HOMREF=0.9999029973809292;N_BI_GENOS=10309;Southeast_Han_AN=1908;Southeast_Han_AC=1;Sou
chr5	74544645	.	T	<INS:ME:LINE1>	.	PASS	SVTYPE=INS:ME:LINE1;END=74544662;SVLEN=1971;AN=20618;AC=2;AF=9.700261907071492e-05;N_HET=2;N_HOMALT=0;N_HOMREF=10307;FREQ_HET=0.00019400523814142983;FREQ_HOMALT=0.0;FREQ_HOMREF=0.9998059947618586;N_BI_GENOS=10309;Southeast_Han_AN=1908;Southeast_Han_AC=1;S
chr5	74576186	.	A	<INS:ME:LINE1>	.	PASS	SVTYPE=INS:ME:LINE1;END=74576196;SVLEN=6009;AN=20618;AC=1;AF=4.850130953535746e-05;N_HET=1;N_HOMALT=0;N_HOMREF=10308;FREQ_HET=9.700261907071492e-05;FREQ_HOMALT=0.0;FREQ_HOMREF=0.9999029973809292;N_BI_GENOS=10309;South_Han_AN=1496;South_Han_AC=1;South_Han_
chr5	74770395	.	A	<INS:ME:LINE1>	.	PASS	SVTYPE=INS:ME:LINE1;END=74770410;SVLEN=188;AN=20618;AC=1;AF=4.850130953535746e-05;N_HET=1;N_HOMALT=0;N_HOMREF=10308;FREQ_HET=9.700261907071492e-05;FREQ_HOMALT=0.0;FREQ_HOMREF=0.9999029973809292;N_BI_GENOS=10309;East_Han_AN=6554;East_Han_AC=1;East_Han_AF=0
chr5	74805466	.	C	<INS:ME:LINE1>	.	PASS	SVTYPE=INS:ME:LINE1;END=74805482;SVLEN=6000;AN=20618;AC=4;AF=0.00019400523814142983;N_HET=4;N_HOMALT=0;N_HOMREF=10305;FREQ_HET=0.00038801047628285966;FREQ_HOMALT=0.0;FREQ_HOMREF=0.9996119895237171;N_BI_GENOS=10309;Northwest_Han_AN=1296;Northwest_Han_AC=1;
chr5	75846587	.	A	<INS:ME:LINE1>	.	PASS	SVTYPE=INS:ME:LINE1;END=75846603;SVLEN=112;AN=20618;AC=1;AF=4.850130953535746e-05;N_HET=1;N_HOMALT=0;N_HOMREF=10308;FREQ_HET=9.700261907071492e-05;FREQ_HOMALT=0.0;FREQ_HOMREF=0.9999029973809292;N_BI_GENOS=10309;Southeast_Han_AN=1908;Southeast_Han_AC=1;Sou
chr5	75930017	.	A	<INS:ME:LINE1>	.	PASS	SVTYPE=INS:ME:LINE1;END=75930030;SVLEN=528;AN=20618;AC=2;AF=9.700261907071492e-05;N_HET=2;N_HOMALT=0;N_HOMREF=10307;FREQ_HET=0.00019400523814142983;FREQ_HOMALT=0.0;FREQ_HOMREF=0.9998059947618586;N_BI_GENOS=10309;East_Han_AN=6554;East_Han_AC=2;East_Han_AF=
chr5	75994447	.	A	<INS:ME:LINE1>	.	PASS	SVTYPE=INS:ME:LINE1;END=75994460;SVLEN=948;AN=20618;AC=776;AF=0.037637016199437386;N_HET=714;N_HOMALT=31;N_HOMREF=9564;FREQ_HET=0.06925987001649045;FREQ_HOMALT=0.0030070811911921624;FREQ_HOMREF=0.9277330487923174;N_BI_GENOS=10309;South_Han_AN=1496;South_H
chr5	76501188	.	C	<INS:ME:LINE1>	.	PASS	SVTYPE=INS:ME:LINE1;END=76501205;SVLEN=5987;AN=20618;AC=1;AF=4.850130953535746e-05;N_HET=1;N_HOMALT=0;N_HOMREF=10308;FREQ_HET=9.700261907071492e-05;FREQ_HOMALT=0.0;FREQ_HOMREF=0.9999029973809292;N_BI_GENOS=10309;Central_Han_AN=2128;Central_Han_AC=1;Centra
chr5	76886444	.	C	<INS:ME:LINE1>	.	PASS	SVTYPE=INS:ME:LINE1;END=76886444;SVLEN=0;AN=20618;AC=13;AF=0.0006305170239596469;N_HET=13;N_HOMALT=0;N_HOMREF=10296;FREQ_HET=0.0012610340479192938;FREQ_HOMALT=0.0;FREQ_HOMREF=0.9987389659520807;N_BI_GENOS=10309;South_Han_AN=1496;South_Han_AC=2;South_Han_A
chr5	76914400	.	T	<INS:ME:LINE1>	.	PASS	SVTYPE=INS:ME:LINE1;END=76914400;SVLEN=0;AN=20618;AC=108;AF=0.005238141429818605;N_HET=70;N_HOMALT=19;N_HOMREF=10220;FREQ_HET=0.006790183334950043;FREQ_HOMALT=0.0018430497623435833;FREQ_HOMREF=0.9913667669027064;N_BI_GENOS=10309;South_Han_AN=1496;South_Ha
chr5	77044300	.	G	<INS:ME:LINE1>	.	PASS	SVTYPE=INS:ME:LINE1;END=77044300;SVLEN=0;AN=20618;AC=1;AF=4.850130953535746e-05;N_HET=1;N_HOMALT=0;N_HOMREF=10308;FREQ_HET=9.700261907071492e-05;FREQ_HOMALT=0.0;FREQ_HOMREF=0.9999029973809292;N_BI_GENOS=10309;East_Han_AN=6554;East_Han_AC=1;East_Han_AF=0.0

chr5	77197683	.	A	<INS:ME:LINE1>	.	PASS	SVTYPE=INS:ME:LINE1;END=77197706;SVLEN=3081;AN=20618;AC=6;AF=0.0002910078572121447;N_HET=6;N_HOMALT=0;N_HOMREF=10303;FREQ_HET=0.0005820157144242894;FREQ_HOMALT=0.0;FREQ_HOMREF=0.9994179842855757;N_BI_GENOS=10309;Lingnan_Han_AN=1888;Lingnan_Han_AC=2;Lingna
chr5	77254224	.	A	<INS:ME:LINE1>	.	PASS	SVTYPE=INS:ME:LINE1;END=77254241;SVLEN=214;AN=20618;AC=210;AF=0.010185275002425066;N_HET=196;N_HOMALT=7;N_HOMREF=10106;FREQ_HET=0.019012513337860124;FREQ_HOMALT=0.0006790183334950044;FREQ_HOMREF=0.9803084683286448;N_BI_GENOS=10309;South_Han_AN=1496;South_
chr5	77457092	.	G	<INS:ME:LINE1>	.	PASS	SVTYPE=INS:ME:LINE1;END=77457105;SVLEN=71;AN=20618;AC=3;AF=0.00014550392860607236;N_HET=3;N_HOMALT=0;N_HOMREF=10306;FREQ_HET=0.0002910078572121447;FREQ_HOMALT=0.0;FREQ_HOMREF=0.9997089921427879;N_BI_GENOS=10309;South_Han_AN=1496;South_Han_AC=1;South_Han_A
chr5	77591159	.	A	<INS:ME:LINE1>	.	PASS	SVTYPE=INS:ME:LINE1;END=77591162;SVLEN=262;AN=20618;AC=1;AF=4.850130953535746e-05;N_HET=1;N_HOMALT=0;N_HOMREF=10308;FREQ_HET=9.700261907071492e-05;FREQ_HOMALT=0.0;FREQ_HOMREF=0.9999029973809292;N_BI_GENOS=10309;East_Han_AN=6554;East_Han_AC=1;East_Han_AF=0
chr5	78052020	.	A	<INS:ME:LINE1>	.	PASS	SVTYPE=INS:ME:LINE1;END=78052036;SVLEN=1513;AN=20618;AC=19;AF=0.0009215248811717916;N_HET=19;N_HOMALT=0;N_HOMREF=10290;FREQ_HET=0.0018430497623435833;FREQ_HOMALT=0.0;FREQ_HOMREF=0.9981569502376564;N_BI_GENOS=10309;South_Han_AN=1496;South_Han_AC=1;South_Ha
chr5	78189443	.	G	<INS:ME:LINE1>	.	PASS	SVTYPE=INS:ME:LINE1;END=78189456;SVLEN=2815;AN=20618;AC=3;AF=0.00014550392860607236;N_HET=3;N_HOMALT=0;N_HOMREF=10306;FREQ_HET=0.0002910078572121447;FREQ_HOMALT=0.0;FREQ_HOMREF=0.9997089921427879;N_BI_GENOS=10309;Central_Han_AN=2128;Central_Han_AC=1;Centr
chr5	78352679	.	T	<INS:ME:LINE1>	.	PASS	SVTYPE=INS:ME:LINE1;END=78352695;SVLEN=1766;AN=20618;AC=1;AF=4.850130953535746e-05;N_HET=1;N_HOMALT=0;N_HOMREF=10308;FREQ_HET=9.700261907071492e-05;FREQ_HOMALT=0.0;FREQ_HOMREF=0.9999029973809292;N_BI_GENOS=10309;East_Han_AN=6554;East_Han_AC=1;East_Han_AF=
chr5	78661598	.	G	<INS:ME:LINE1>	.	PASS	SVTYPE=INS:ME:LINE1;END=78661609;SVLEN=6000;AN=20618;AC=19;AF=0.0009215248811717916;N_HET=19;N_HOMALT=0;N_HOMREF=10290;FREQ_HET=0.0018430497623435833;FREQ_HOMALT=0.0;FREQ_HOMREF=0.9981569502376564;N_BI_GENOS=10309;South_Han_AN=1496;South_Han_AC=4;South_Ha
chr5	78777318	.	A	<INS:ME:LINE1>	.	PASS	SVTYPE=INS:ME:LINE1;END=78777334;SVLEN=6017;AN=20618;AC=6;AF=0.0002910078572121447;N_HET=6;N_HOMALT=0;N_HOMREF=10303;FREQ_HET=0.0005820157144242894;FREQ_HOMALT=0.0;FREQ_HOMREF=0.9994179842855757;N_BI_GENOS=10309;Northwest_Han_AN=1296;Northwest_Han_AC=1;No
chr5	78824475	.	A	<INS:ME:LINE1>	.	PASS	SVTYPE=INS:ME:LINE1;END=78824491;SVLEN=1470;AN=20618;AC=4;AF=0.00019400523814142983;N_HET=4;N_HOMALT=0;N_HOMREF=10305;FREQ_HET=0.00038801047628285966;FREQ_HOMALT=0.0;FREQ_HOMREF=0.9996119895237171;N_BI_GENOS=10309;South_Han_AN=1496;South_Han_AC=1;South_Ha
chr5	78899268	.	C	<INS:ME:LINE1>	.	PASS	SVTYPE=INS:ME:LINE1;END=78899277;SVLEN=980;AN=20618;AC=1;AF=4.850130953535746e-05;N_HET=1;N_HOMALT=0;N_HOMREF=10308;FREQ_HET=9.700261907071492e-05;FREQ_HOMALT=0.0;FREQ_HOMREF=0.9999029973809292;N_BI_GENOS=10309;East_Han_AN=6554;East_Han_AC=1;East_Han_AF=0
chr5	78913100	.	T	<INS:ME:LINE1>	.	PASS	SVTYPE=INS:ME:LINE1;END=78913100;SVLEN=5971;AN=20618;AC=1;AF=4.850130953535746e-05;N_HET=1;N_HOMALT=0;N_HOMREF=10308;FREQ_HET=9.700261907071492e-05;FREQ_HOMALT=0.0;FREQ_HOMREF=0.9999029973809292;N_BI_GENOS=10309;North_Han_AN=2272;North_Han_AC=1;North_Han_
chr5	79953235	.	A	<INS:ME:LINE1>	.	PASS	SVTYPE=INS:ME:LINE1;END=79953251;SVLEN=1856;AN=20618;AC=2;AF=9.700261907071492e-05;N_HET=2;N_HOMALT=0;N_HOMREF=10307;FREQ_HET=0.00019400523814142983;FREQ_HOMALT=0.0;FREQ_HOMREF=0.9998059947618586;N_BI_GENOS=10309;North_Han_AN=2272;North_Han_AC=1;North_Han
chr5	80568889	.	A	<INS:ME:LINE1>	.	PASS	SVTYPE=INS:ME:LINE1;END=80568889;SVLEN=0;AN=20618;AC=3;AF=0.00014550392860607236;N_HET=1;N_HOMALT=1;N_HOMREF=10307;FREQ_HET=9.700261907071492e-05;FREQ_HOMALT=9.700261907071492e-05;FREQ_HOMREF=0.9998059947618586;N_BI_GENOS=10309;North_Han_AN=2272;North_Han
chr5	80964274	.	G	<INS:ME:LINE1>	.	PASS	SVTYPE=INS:ME:LINE1;END=80964286;SVLEN=6009;AN=20618;AC=1;AF=4.850130953535746e-05;N_HET=1;N_HOMALT=0;N_HOMREF=10308;FREQ_HET=9.700261907071492e-05;FREQ_HOMALT=0.0;FREQ_HOMREF=0.9999029973809292;N_BI_GENOS=10309;Central_Han_AN=2128;Central_Han_AC=1;Centra
chr5	81082712	.	A	<INS:ME:LINE1>	.	PASS	SVTYPE=INS:ME:LINE1;END=81082728;SVLEN=158;AN=20618;AC=1;AF=4.850130953535746e-05;N_HET=1;N_HOMALT=0;N_HOMREF=10308;FREQ_HET=9.700261907071492e-05;FREQ_HOMALT=0.0;FREQ_HOMREF=0.9999029973809292;N_BI_GENOS=10309;Lingnan_Han_AN=1888;Lingnan_Han_AC=1;Lingnan
chr5	81327043	.	G	<INS:ME:LINE1>	.	PASS	SVTYPE=INS:ME:LINE1;END=81327060;SVLEN=788;AN=20618;AC=1;AF=4.850130953535746e-05;N_HET=1;N_HOMALT=0;N_HOMREF=10308;FREQ_HET=9.700261907071492e-05;FREQ_HOMALT=0.0;FREQ_HOMREF=0.9999029973809292;N_BI_GENOS=10309;South_Han_AN=1496;South_Han_AC=1;South_Han_A
chr5	81729012	.	G	<INS:ME:LINE1>	.	PASS	SVTYPE=INS:ME:LINE1;END=81729020;SVLEN=6000;AN=20618;AC=1;AF=4.850130953535746e-05;N_HET=1;N_HOMALT=0;N_HOMREF=10308;FREQ_HET=9.700261907071492e-05;FREQ_HOMALT=0.0;FREQ_HOMREF=0.9999029973809292;N_BI_GENOS=10309;Manchu_AN=416;Manchu_AC=1;Manchu_AF=0.00240
chr5	81913103	.	C	<INS:ME:LINE1>	.	PASS	SVTYPE=INS:ME:LINE1;END=81913121;SVLEN=5999;AN=20618;AC=1;AF=4.850130953535746e-05;N_HET=1;N_HOMALT=0;N_HOMREF=10308;FREQ_HET=9.700261907071492e-05;FREQ_HOMALT=0.0;FREQ_HOMREF=0.9999029973809292;N_BI_GENOS=10309;North_Han_AN=2272;North_Han_AC=1;North_Han_
chr5	82112013	.	T	<INS:ME:LINE1>	.	PASS	SVTYPE=INS:ME:LINE1;END=82112026;SVLEN=6017;AN=20618;AC=1;AF=4.850130953535746e-05;N_HET=1;N_HOMALT=0;N_HOMREF=10308;FREQ_HET=9.700261907071492e-05;FREQ_HOMALT=0.0;FREQ_HOMREF=0.9999029973809292;N_BI_GENOS=10309;Central_Han_AN=2128;Central_Han_AC=1;Centra
chr5	82314821	.	C	<INS:ME:LINE1>	.	PASS	SVTYPE=INS:ME:LINE1;END=82314838;SVLEN=6006;AN=20618;AC=1;AF=4.850130953535746e-05;N_HET=1;N_HOMALT=0;N_HOMREF=10308;FREQ_HET=9.700261907071492e-05;FREQ_HOMALT=0.0;FREQ_HOMREF=0.9999029973809292;N_BI_GENOS=10309;Lingnan_Han_AN=1888;Lingnan_Han_AC=1;Lingna
chr5	82422277	.	G	<INS:ME:LINE1>	.	PASS	SVTYPE=INS:ME:LINE1;END=82422292;SVLEN=5998;AN=20618;AC=2;AF=9.700261907071492e-05;N_HET=2;N_HOMALT=0;N_HOMREF=10307;FREQ_HET=0.00019400523814142983;FREQ_HOMALT=0.0;FREQ_HOMREF=0.9998059947618586;N_BI_GENOS=10309;Central_Han_AN=2128;Central_Han_AC=1;Centr
chr5	82440009	.	A	<INS:ME:LINE1>	.	PASS	SVTYPE=INS:ME:LINE1;END=82440011;SVLEN=183;AN=20618;AC=1;AF=4.850130953535746e-05;N_HET=1;N_HOMALT=0;N_HOMREF=10308;FREQ_HET=9.700261907071492e-05;FREQ_HOMALT=0.0;FREQ_HOMREF=0.9999029973809292;N_BI_GENOS=10309;Manchu_AN=416;Manchu_AC=1;Manchu_AF=0.002403
chr5	82611013	.	G	<INS:ME:LINE1>	.	PASS	SVTYPE=INS:ME:LINE1;END=82611025;SVLEN=6008;AN=20618;AC=1;AF=4.850130953535746e-05;N_HET=1;N_HOMALT=0;N_HOMREF=10308;FREQ_HET=9.700261907071492e-05;FREQ_HOMALT=0.0;FREQ_HOMREF=0.9999029973809292;N_BI_GENOS=10309;South_Han_AN=1496;South_Han_AC=1;South_Han_
chr5	83060063	.	T	<INS:ME:LINE1>	.	PASS	SVTYPE=INS:ME:LINE1;END=83060063;SVLEN=1374;AN=20618;AC=162;AF=0.007857212144727907;N_HET=108;N_HOMALT=27;N_HOMREF=10174;FREQ_HET=0.01047628285963721;FREQ_HOMALT=0.0026190707149093026;FREQ_HOMREF=0.9869046464254535;N_BI_GENOS=10309;South_Han_AN=1496;South
chr5	83060161	.	A	<INS:ME:LINE1>	.	PASS	SVTYPE=INS:ME:LINE1;END=83060170;SVLEN=1377;AN=20618;AC=1;AF=4.850130953535746e-05;N_HET=1;N_HOMALT=0;N_HOMREF=10308;FREQ_HET=9.700261907071492e-05;FREQ_HOMALT=0.0;FREQ_HOMREF=0.9999029973809292;N_BI_GENOS=10309;East_Han_AN=6554;East_Han_AC=1;East_Han_AF=
chr5	83205501	.	T	<INS:ME:LINE1>	.	PASS	SVTYPE=INS:ME:LINE1;END=83205507;SVLEN=830;AN=20618;AC=1;AF=4.850130953535746e-05;N_HET=1;N_HOMALT=0;N_HOMREF=10308;FREQ_HET=9.700261907071492e-05;FREQ_HOMALT=0.0;FREQ_HOMREF=0.9999029973809292;N_BI_GENOS=10309;Central_Han_AN=2128;Central_Han_AC=1;Central
chr5	83293327	.	C	<INS:ME:LINE1>	.	PASS	SVTYPE=INS:ME:LINE1;END=83293343;SVLEN=682;AN=20618;AC=1;AF=4.850130953535746e-05;N_HET=1;N_HOMALT=0;N_HOMREF=10308;FREQ_HET=9.700261907071492e-05;FREQ_HOMALT=0.0;FREQ_HOMREF=0.9999029973809292;N_BI_GENOS=10309;Central_Han_AN=2128;Central_Han_AC=1;Central
chr5	83335863	.	A	<INS:ME:LINE1>	.	PASS	SVTYPE=INS:ME:LINE1;END=83335879;SVLEN=853;AN=20618;AC=2;AF=9.700261907071492e-05;N_HET=2;N_HOMALT=0;N_HOMREF=10307;FREQ_HET=0.00019400523814142983;FREQ_HOMALT=0.0;FREQ_HOMREF=0.9998059947618586;N_BI_GENOS=10309;South_Han_AN=1496;South_Han_AC=2;South_Han_
chr5	83515714	.	T	<INS:ME:LINE1>	.	PASS	SVTYPE=INS:ME:LINE1;END=83515732;SVLEN=2522;AN=20618;AC=1;AF=4.850130953535746e-05;N_HET=1;N_HOMALT=0;N_HOMREF=10308;FREQ_HET=9.700261907071492e-05;FREQ_HOMALT=0.0;FREQ_HOMREF=0.9999029973809292;N_BI_GENOS=10309;East_Han_AN=6554;East_Han_AC=1;East_Han_AF=
chr5	83598948	.	A	<INS:ME:LINE1>	.	PASS	SVTYPE=INS:ME:LINE1;END=83598966;SVLEN=6012;AN=20618;AC=2;AF=9.700261907071492e-05;N_HET=2;N_HOMALT=0;N_HOMREF=10307;FREQ_HET=0.00019400523814142983;FREQ_HOMALT=0.0;FREQ_HOMREF=0.9998059947618586;N_BI_GENOS=10309;East_Han_AN=6554;East_Han_AC=1;East_Han_AF
chr5	84036304	.	A	<INS:ME:LINE1>	.	PASS	SVTYPE=INS:ME:LINE1;END=84036319;SVLEN=963;AN=20618;AC=1;AF=4.850130953535746e-05;N_HET=1;N_HOMALT=0;N_HOMREF=10308;FREQ_HET=9.700261907071492e-05;FREQ_HOMALT=0.0;FREQ_HOMREF=0.9999029973809292;N_BI_GENOS=10309;Central_Han_AN=2128;Central_Han_AC=1;Central
chr5	84070586	.	T	<INS:ME:LINE1>	.	PASS	SVTYPE=INS:ME:LINE1;END=84070599;SVLEN=498;AN=20618;AC=1;AF=4.850130953535746e-05;N_HET=1;N_HOMALT=0;N_HOMREF=10308;FREQ_HET=9.700261907071492e-05;FREQ_HOMALT=0.0;FREQ_HOMREF=0.9999029973809292;N_BI_GENOS=10309;Lingnan_Han_AN=1888;Lingnan_Han_AC=1;Lingnan
chr5	84688286	.	C	<INS:ME:LINE1>	.	PASS	SVTYPE=INS:ME:LINE1;END=84688286;SVLEN=276;AN=20618;AC=1;AF=4.850130953535746e-05;N_HET=1;N_HOMALT=0;N_HOMREF=10308;FREQ_HET=9.700261907071492e-05;FREQ_HOMALT=0.0;FREQ_HOMREF=0.9999029973809292;N_BI_GENOS=10309;East_Han_AN=6554;East_Han_AC=1;East_Han_AF=0
chr5	84746854	.	G	<INS:ME:LINE1>	.	PASS	SVTYPE=INS:ME:LINE1;END=84746862;SVLEN=5485;AN=20618;AC=1;AF=4.850130953535746e-05;N_HET=1;N_HOMALT=0;N_HOMREF=10308;FREQ_HET=9.700261907071492e-05;FREQ_HOMALT=0.0;FREQ_HOMREF=0.9999029973809292;N_BI_GENOS=10309;Central_Han_AN=2128;Central_Han_AC=1;Centra
chr5	84867969	.	T	<INS:ME:LINE1>	.	PASS	SVTYPE=INS:ME:LINE1;END=84867983;SVLEN=2549;AN=20618;AC=19;AF=0.0009215248811717916;N_HET=19;N_HOMALT=0;N_HOMREF=10290;FREQ_HET=0.0018430497623435833;FREQ_HOMALT=0.0;FREQ_HOMREF=0.9981569502376564;N_BI_GENOS=10309;South_Han_AN=1496;South_Han_AC=3;South_Ha
chr5	84943797	.	C	<INS:ME:LINE1>	.	PASS	SVTYPE=INS:ME:LINE1;END=84943797;SVLEN=700;AN=20618;AC=473;AF=0.022941119410224076;N_HET=283;N_HOMALT=95;N_HOMREF=9931;FREQ_HET=0.027451741197012318;FREQ_HOMALT=0.009215248811717916;FREQ_HOMREF=0.9633330099912698;N_BI_GENOS=10309;South_Han_AN=1496;South_H
chr5	84943854	.	A	<INS:ME:LINE1>	.	PASS	SVTYPE=INS:ME:LINE1;END=84943854;SVLEN=721;AN=20618;AC=6414;AF=0.3110873993597827;N_HET=2508;N_HOMALT=1953;N_HOMREF=5848;FREQ_HET=0.243282568629353;FREQ_HOMALT=0.18944611504510622;FREQ_HOMREF=0.5672713163255408;N_BI_GENOS=10309;South_Han_AN=1496;South_Han
chr5	85183416	.	A	<INS:ME:LINE1>	.	PASS	SVTYPE=INS:ME:LINE1;END=85183433;SVLEN=351;AN=20618;AC=7;AF=0.0003395091667475022;N_HET=7;N_HOMALT=0;N_HOMREF=10302;FREQ_HET=0.0006790183334950044;FREQ_HOMALT=0.0;FREQ_HOMREF=0.999320981666505;N_BI_GENOS=10309;Lingnan_Han_AN=1888;Lingnan_Han_AC=1;Lingnan_
chr5	85248571	.	T	<INS:ME:LINE1>	.	PASS	SVTYPE=INS:ME:LINE1;END=85248571;SVLEN=15;AN=20618;AC=5;AF=0.00024250654767678728;N_HET=5;N_HOMALT=0;N_HOMREF=10304;FREQ_HET=0.00048501309535357455;FREQ_HOMALT=0.0;FREQ_HOMREF=0.9995149869046465;N_BI_GENOS=10309;South_Han_AN=1496;South_Han_AC=3;South_Han_
chr5	85298147	.	T	<INS:ME:LINE1>	.	PASS	SVTYPE=INS:ME:LINE1;END=85298164;SVLEN=4367;AN=20618;AC=1;AF=4.850130953535746e-05;N_HET=1;N_HOMALT=0;N_HOMREF=10308;FREQ_HET=9.700261907071492e-05;FREQ_HOMALT=0.0;FREQ_HOMREF=0.9999029973809292;N_BI_GENOS=10309;South_Han_AN=1496;South_Han_AC=1;South_Han_
chr5	85331743	.	G	<INS:ME:LINE1>	.	PASS	SVTYPE=INS:ME:LINE1;END=85331759;SVLEN=5999;AN=20618;AC=1;AF=4.850130953535746e-05;N_HET=1;N_HOMALT=0;N_HOMREF=10308;FREQ_HET=9.700261907071492e-05;FREQ_HOMALT=0.0;FREQ_HOMREF=0.9999029973809292;N_BI_GENOS=10309;Lingnan_Han_AN=1888;Lingnan_Han_AC=1;Lingna
chr5	85375581	.	T	<INS:ME:LINE1>	.	PASS	SVTYPE=INS:ME:LINE1;END=85375596;SVLEN=1065;AN=20618;AC=1;AF=4.850130953535746e-05;N_HET=1;N_HOMALT=0;N_HOMREF=10308;FREQ_HET=9.700261907071492e-05;FREQ_HOMALT=0.0;FREQ_HOMREF=0.9999029973809292;N_BI_GENOS=10309;Lingnan_Han_AN=1888;Lingnan_Han_AC=1;Lingna
chr5	85675042	.	A	<INS:ME:LINE1>	.	PASS	SVTYPE=INS:ME:LINE1;END=85675057;SVLEN=1723;AN=20618;AC=35;AF=0.0016975458337375108;N_HET=35;N_HOMALT=0;N_HOMREF=10274;FREQ_HET=0.0033950916674750217;FREQ_HOMALT=0.0;FREQ_HOMREF=0.996604908332525;N_BI_GENOS=10309;South_Han_AN=1496;South_Han_AC=7;South_Han
chr5	85748004	.	A	<INS:ME:LINE1>	.	PASS	SVTYPE=INS:ME:LINE1;END=85748018;SVLEN=887;AN=20618;AC=1;AF=4.850130953535746e-05;N_HET=1;N_HOMALT=0;N_HOMREF=10308;FREQ_HET=9.700261907071492e-05;FREQ_HOMALT=0.0;FREQ_HOMREF=0.9999029973809292;N_BI_GENOS=10309;East_Han_AN=6554;East_Han_AC=1;East_Han_AF=0
chr5	85952373	.	T	<INS:ME:LINE1>	.	PASS	SVTYPE=INS:ME:LINE1;END=85952391;SVLEN=1402;AN=20618;AC=3;AF=0.00014550392860607236;N_HET=3;N_HOMALT=0;N_HOMREF=10306;FREQ_HET=0.0002910078572121447;FREQ_HOMALT=0.0;FREQ_HOMREF=0.9997089921427879;N_BI_GENOS=10309;Northwest_Han_AN=1296;Northwest_Han_AC=1;N
chr5	85969226	.	T	<INS:ME:LINE1>	.	PASS	SVTYPE=INS:ME:LINE1;END=85969263;SVLEN=2569;AN=20618;AC=1;AF=4.850130953535746e-05;N_HET=1;N_HOMALT=0;N_HOMREF=10308;FREQ_HET=9.700261907071492e-05;FREQ_HOMALT=0.0;FREQ_HOMREF=0.9999029973809292;N_BI_GENOS=10309;East_Han_AN=6554;East_Han_AC=1;East_Han_AF=
chr5	86257914	.	A	<INS:ME:LINE1>	.	PASS	SVTYPE=INS:ME:LINE1;END=86257919;SVLEN=5127;AN=20618;AC=1;AF=4.850130953535746e-05;N_HET=1;N_HOMALT=0;N_HOMREF=10308;FREQ_HET=9.700261907071492e-05;FREQ_HOMALT=0.0;FREQ_HOMREF=0.9999029973809292;N_BI_GENOS=10309;Tibetan_AN=414;Tibetan_AC=1;Tibetan_AF=0.00
chr5	86334203	.	A	<INS:ME:LINE1>	.	PASS	SVTYPE=INS:ME:LINE1;END=86334203;SVLEN=2918;AN=20618;AC=1;AF=4.850130953535746e-05;N_HET=1;N_HOMALT=0;N_HOMREF=10308;FREQ_HET=9.700261907071492e-05;FREQ_HOMALT=0.0;FREQ_HOMREF=0.9999029973809292;N_BI_GENOS=10309;East_Han_AN=6554;East_Han_AC=1;East_Han_AF=
chr5	86340357	.	A	<INS:ME:LINE1>	.	PASS	SVTYPE=INS:ME:LINE1;END=86340357;SVLEN=835;AN=20618;AC=1;AF=4.850130953535746e-05;N_HET=1;N_HOMALT=0;N_HOMREF=10308;FREQ_HET=9.700261907071492e-05;FREQ_HOMALT=0.0;FREQ_HOMREF=0.9999029973809292;N_BI_GENOS=10309;East_Han_AN=6554;East_Han_AC=1;East_Han_AF=0
chr5	86340582	.	C	<INS:ME:LINE1>	.	PASS	SVTYPE=INS:ME:LINE1;END=86340582;SVLEN=1168;AN=20618;AC=1;AF=4.850130953535746e-05;N_HET=1;N_HOMALT=0;N_HOMREF=10308;FREQ_HET=9.700261907071492e-05;FREQ_HOMALT=0.0;FREQ_HOMREF=0.9999029973809292;N_BI_GENOS=10309;East_Han_AN=6554;East_Han_AC=1;East_Han_AF=
chr5	86414065	.	T	<INS:ME:LINE1>	.	PASS	SVTYPE=INS:ME:LINE1;END=86414067;SVLEN=564;AN=20618;AC=1;AF=4.850130953535746e-05;N_HET=1;N_HOMALT=0;N_HOMREF=10308;FREQ_HET=9.700261907071492e-05;FREQ_HOMALT=0.0;FREQ_HOMREF=0.9999029973809292;N_BI_GENOS=10309;Miao_AN=482;Miao_AC=1;Miao_AF=0.002074688796
chr5	86437891	.	A	<INS:ME:LINE1>	.	PASS	SVTYPE=INS:ME:LINE1;END=86437903;SVLEN=5998;AN=20618;AC=1;AF=4.850130953535746e-05;N_HET=1;N_HOMALT=0;N_HOMREF=10308;FREQ_HET=9.700261907071492e-05;FREQ_HOMALT=0.0;FREQ_HOMREF=0.9999029973809292;N_BI_GENOS=10309;Zhuang_AN=444;Zhuang_AC=1;Zhuang_AF=0.00225
chr5	86471887	.	A	<INS:ME:LINE1>	.	PASS	SVTYPE=INS:ME:LINE1;END=86471897;SVLEN=666;AN=20618;AC=1;AF=4.850130953535746e-05;N_HET=1;N_HOMALT=0;N_HOMREF=10308;FREQ_HET=9.700261907071492e-05;FREQ_HOMALT=0.0;FREQ_HOMREF=0.9999029973809292;N_BI_GENOS=10309;Yi_AN=422;Yi_AC=1;Yi_AF=0.002369668246445498
chr5	86601560	.	A	<INS:ME:LINE1>	.	PASS	SVTYPE=INS:ME:LINE1;END=86601560;SVLEN=1047;AN=20618;AC=1;AF=4.850130953535746e-05;N_HET=1;N_HOMALT=0;N_HOMREF=10308;FREQ_HET=9.700261907071492e-05;FREQ_HOMALT=0.0;FREQ_HOMREF=0.9999029973809292;N_BI_GENOS=10309;East_Han_AN=6554;East_Han_AC=1;East_Han_AF=
chr5	86895700	.	A	<INS:ME:LINE1>	.	PASS	SVTYPE=INS:ME:LINE1;END=86895714;SVLEN=1479;AN=20618;AC=5;AF=0.00024250654767678728;N_HET=5;N_HOMALT=0;N_HOMREF=10304;FREQ_HET=0.00048501309535357455;FREQ_HOMALT=0.0;FREQ_HOMREF=0.9995149869046465;N_BI_GENOS=10309;Lingnan_Han_AN=1888;Lingnan_Han_AC=1;Ling
chr5	86932383	.	A	<INS:ME:LINE1>	.	PASS	SVTYPE=INS:ME:LINE1;END=86932395;SVLEN=5998;AN=20618;AC=1;AF=4.850130953535746e-05;N_HET=1;N_HOMALT=0;N_HOMREF=10308;FREQ_HET=9.700261907071492e-05;FREQ_HOMALT=0.0;FREQ_HOMREF=0.9999029973809292;N_BI_GENOS=10309;North_Han_AN=2272;North_Han_AC=1;North_Han_
chr5	86943332	.	A	<INS:ME:LINE1>	.	PASS	SVTYPE=INS:ME:LINE1;END=86943336;SVLEN=120;AN=20618;AC=1;AF=4.850130953535746e-05;N_HET=1;N_HOMALT=0;N_HOMREF=10308;FREQ_HET=9.700261907071492e-05;FREQ_HOMALT=0.0;FREQ_HOMREF=0.9999029973809292;N_BI_GENOS=10309;Miao_AN=482;Miao_AC=1;Miao_AF=0.002074688796
chr5	87010733	.	A	<INS:ME:LINE1>	.	PASS	SVTYPE=INS:ME:LINE1;END=87010748;SVLEN=1033;AN=20618;AC=16;AF=0.0007760209525657193;N_HET=16;N_HOMALT=0;N_HOMREF=10293;FREQ_HET=0.0015520419051314386;FREQ_HOMALT=0.0;FREQ_HOMREF=0.9984479580948685;N_BI_GENOS=10309;South_Han_AN=1496;South_Han_AC=3;South_Ha
chr5	87027310	.	C	<INS:ME:LINE1>	.	PASS	SVTYPE=INS:ME:LINE1;END=87027310;SVLEN=694;AN=20618;AC=1;AF=4.850130953535746e-05;N_HET=1;N_HOMALT=0;N_HOMREF=10308;FREQ_HET=9.700261907071492e-05;FREQ_HOMALT=0.0;FREQ_HOMREF=0.9999029973809292;N_BI_GENOS=10309;East_Han_AN=6554;East_Han_AC=1;East_Han_AF=0
chr5	87372560	.	G	<INS:ME:LINE1>	.	PASS	SVTYPE=INS:ME:LINE1;END=87372560;SVLEN=3190;AN=20618;AC=2;AF=9.700261907071492e-05;N_HET=2;N_HOMALT=0;N_HOMREF=10307;FREQ_HET=0.00019400523814142983;FREQ_HOMALT=0.0;FREQ_HOMREF=0.9998059947618586;N_BI_GENOS=10309;East_Han_AN=6554;East_Han_AC=1;East_Han_AF
chr5	87634130	.	A	<INS:ME:LINE1>	.	PASS	SVTYPE=INS:ME:LINE1;END=87634141;SVLEN=6000;AN=20618;AC=4;AF=0.00019400523814142983;N_HET=4;N_HOMALT=0;N_HOMREF=10305;FREQ_HET=0.00038801047628285966;FREQ_HOMALT=0.0;FREQ_HOMREF=0.9996119895237171;N_BI_GENOS=10309;Lingnan_Han_AN=1888;Lingnan_Han_AC=2;Ling
chr5	87656856	.	A	<INS:ME:LINE1>	.	PASS	SVTYPE=INS:ME:LINE1;END=87656872;SVLEN=2031;AN=20618;AC=3;AF=0.00014550392860607236;N_HET=3;N_HOMALT=0;N_HOMREF=10306;FREQ_HET=0.0002910078572121447;FREQ_HOMALT=0.0;FREQ_HOMREF=0.9997089921427879;N_BI_GENOS=10309;East_Han_AN=6554;East_Han_AC=3;East_Han_AF
chr5	87775627	.	A	<INS:ME:LINE1>	.	PASS	SVTYPE=INS:ME:LINE1;END=87775645;SVLEN=991;AN=20618;AC=1;AF=4.850130953535746e-05;N_HET=1;N_HOMALT=0;N_HOMREF=10308;FREQ_HET=9.700261907071492e-05;FREQ_HOMALT=0.0;FREQ_HOMREF=0.9999029973809292;N_BI_GENOS=10309;North_Han_AN=2272;North_Han_AC=1;North_Han_A
chr5	87812031	.	A	<INS:ME:LINE1>	.	PASS	SVTYPE=INS:ME:LINE1;END=87812045;SVLEN=6000;AN=20618;AC=1;AF=4.850130953535746e-05;N_HET=1;N_HOMALT=0;N_HOMREF=10308;FREQ_HET=9.700261907071492e-05;FREQ_HOMALT=0.0;FREQ_HOMREF=0.9999029973809292;N_BI_GENOS=10309;South_Han_AN=1496;South_Han_AC=1;South_Han_
chr5	87901327	.	A	<INS:ME:LINE1>	.	PASS	SVTYPE=INS:ME:LINE1;END=87901340;SVLEN=3115;AN=20618;AC=1;AF=4.850130953535746e-05;N_HET=1;N_HOMALT=0;N_HOMREF=10308;FREQ_HET=9.700261907071492e-05;FREQ_HOMALT=0.0;FREQ_HOMREF=0.9999029973809292;N_BI_GENOS=10309;North_Han_AN=2272;North_Han_AC=1;North_Han_
chr5	87983343	.	T	<INS:ME:LINE1>	.	PASS	SVTYPE=INS:ME:LINE1;END=87983343;SVLEN=263;AN=20618;AC=9;AF=0.0004365117858182171;N_HET=7;N_HOMALT=1;N_HOMREF=10301;FREQ_HET=0.0006790183334950044;FREQ_HOMALT=9.700261907071492e-05;FREQ_HOMREF=0.9992239790474343;N_BI_GENOS=10309;North_Han_AN=2272;North_Ha
chr5	88074355	.	A	<INS:ME:LINE1>	.	PASS	SVTYPE=INS:ME:LINE1;END=88074371;SVLEN=5159;AN=20618;AC=1;AF=4.850130953535746e-05;N_HET=1;N_HOMALT=0;N_HOMREF=10308;FREQ_HET=9.700261907071492e-05;FREQ_HOMALT=0.0;FREQ_HOMREF=0.9999029973809292;N_BI_GENOS=10309;Central_Han_AN=2128;Central_Han_AC=1;Centra
chr5	88266503	.	A	<INS:ME:LINE1>	.	PASS	SVTYPE=INS:ME:LINE1;END=88266513;SVLEN=709;AN=20618;AC=26;AF=0.0012610340479192938;N_HET=24;N_HOMALT=1;N_HOMREF=10284;FREQ_HET=0.0023280628576971578;FREQ_HOMALT=9.700261907071492e-05;FREQ_HOMREF=0.9975749345232321;N_BI_GENOS=10309;South_Han_AN=1496;South_
chr5	88359943	.	A	<INS:ME:LINE1>	.	PASS	SVTYPE=INS:ME:LINE1;END=88359960;SVLEN=3633;AN=20618;AC=2;AF=9.700261907071492e-05;N_HET=2;N_HOMALT=0;N_HOMREF=10307;FREQ_HET=0.00019400523814142983;FREQ_HOMALT=0.0;FREQ_HOMREF=0.9998059947618586;N_BI_GENOS=10309;South_Han_AN=1496;South_Han_AC=1;South_Han
chr5	88429712	.	A	<INS:ME:LINE1>	.	PASS	SVTYPE=INS:ME:LINE1;END=88429727;SVLEN=5998;AN=20618;AC=1;AF=4.850130953535746e-05;N_HET=1;N_HOMALT=0;N_HOMREF=10308;FREQ_HET=9.700261907071492e-05;FREQ_HOMALT=0.0;FREQ_HOMREF=0.9999029973809292;N_BI_GENOS=10309;Northwest_Han_AN=1296;Northwest_Han_AC=1;No
chr5	88454206	.	G	<INS:ME:LINE1>	.	PASS	SVTYPE=INS:ME:LINE1;END=88454217;SVLEN=5999;AN=20618;AC=1;AF=4.850130953535746e-05;N_HET=1;N_HOMALT=0;N_HOMREF=10308;FREQ_HET=9.700261907071492e-05;FREQ_HOMALT=0.0;FREQ_HOMREF=0.9999029973809292;N_BI_GENOS=10309;Northwest_Han_AN=1296;Northwest_Han_AC=1;No
chr5	88554653	.	A	<INS:ME:LINE1>	.	PASS	SVTYPE=INS:ME:LINE1;END=88554662;SVLEN=6155;AN=20618;AC=1;AF=4.850130953535746e-05;N_HET=1;N_HOMALT=0;N_HOMREF=10308;FREQ_HET=9.700261907071492e-05;FREQ_HOMALT=0.0;FREQ_HOMREF=0.9999029973809292;N_BI_GENOS=10309;Hui_AN=444;Hui_AC=1;Hui_AF=0.00225225225225
chr5	88643918	.	C	<INS:ME:LINE1>	.	PASS	SVTYPE=INS:ME:LINE1;END=88643934;SVLEN=5981;AN=20618;AC=3;AF=0.00014550392860607236;N_HET=3;N_HOMALT=0;N_HOMREF=10306;FREQ_HET=0.0002910078572121447;FREQ_HOMALT=0.0;FREQ_HOMREF=0.9997089921427879;N_BI_GENOS=10309;North_Han_AN=2272;North_Han_AC=2;North_Han
chr5	89139076	.	A	<INS:ME:LINE1>	.	PASS	SVTYPE=INS:ME:LINE1;END=89139121;SVLEN=1228;AN=20618;AC=1;AF=4.850130953535746e-05;N_HET=1;N_HOMALT=0;N_HOMREF=10308;FREQ_HET=9.700261907071492e-05;FREQ_HOMALT=0.0;FREQ_HOMREF=0.9999029973809292;N_BI_GENOS=10309;East_Han_AN=6554;East_Han_AC=1;East_Han_AF=
chr5	89139139	.	G	<INS:ME:LINE1>	.	PASS	SVTYPE=INS:ME:LINE1;END=89139139;SVLEN=840;AN=20618;AC=1;AF=4.850130953535746e-05;N_HET=1;N_HOMALT=0;N_HOMREF=10308;FREQ_HET=9.700261907071492e-05;FREQ_HOMALT=0.0;FREQ_HOMREF=0.9999029973809292;N_BI_GENOS=10309;East_Han_AN=6554;East_Han_AC=1;East_Han_AF=0
chr5	89158520	.	A	<INS:ME:LINE1>	.	PASS	SVTYPE=INS:ME:LINE1;END=89158535;SVLEN=769;AN=20618;AC=1;AF=4.850130953535746e-05;N_HET=1;N_HOMALT=0;N_HOMREF=10308;FREQ_HET=9.700261907071492e-05;FREQ_HOMALT=0.0;FREQ_HOMREF=0.9999029973809292;N_BI_GENOS=10309;Southeast_Han_AN=1908;Southeast_Han_AC=1;Sou
chr5	89171781	.	G	<INS:ME:LINE1>	.	PASS	SVTYPE=INS:ME:LINE1;END=89171794;SVLEN=3438;AN=20618;AC=1;AF=4.850130953535746e-05;N_HET=1;N_HOMALT=0;N_HOMREF=10308;FREQ_HET=9.700261907071492e-05;FREQ_HOMALT=0.0;FREQ_HOMREF=0.9999029973809292;N_BI_GENOS=10309;North_Han_AN=2272;North_Han_AC=1;North_Han_
chr5	89341600	.	A	<INS:ME:LINE1>	.	PASS	SVTYPE=INS:ME:LINE1;END=89341614;SVLEN=891;AN=20618;AC=5;AF=0.00024250654767678728;N_HET=5;N_HOMALT=0;N_HOMREF=10304;FREQ_HET=0.00048501309535357455;FREQ_HOMALT=0.0;FREQ_HOMREF=0.9995149869046465;N_BI_GENOS=10309;Northwest_Han_AN=1296;Northwest_Han_AC=1;N
chr5	89349708	.	C	<INS:ME:LINE1>	.	PASS	SVTYPE=INS:ME:LINE1;END=89349725;SVLEN=947;AN=20618;AC=1;AF=4.850130953535746e-05;N_HET=1;N_HOMALT=0;N_HOMREF=10308;FREQ_HET=9.700261907071492e-05;FREQ_HOMALT=0.0;FREQ_HOMREF=0.9999029973809292;N_BI_GENOS=10309;North_Han_AN=2272;North_Han_AC=1;North_Han_A
chr5	89663420	.	A	<INS:ME:LINE1>	.	PASS	SVTYPE=INS:ME:LINE1;END=89663438;SVLEN=639;AN=20618;AC=1;AF=4.850130953535746e-05;N_HET=1;N_HOMALT=0;N_HOMREF=10308;FREQ_HET=9.700261907071492e-05;FREQ_HOMALT=0.0;FREQ_HOMREF=0.9999029973809292;N_BI_GENOS=10309;North_Han_AN=2272;North_Han_AC=1;North_Han_A
chr5	89745055	.	C	<INS:ME:LINE1>	.	PASS	SVTYPE=INS:ME:LINE1;END=89745127;SVLEN=4073;AN=20618;AC=1;AF=4.850130953535746e-05;N_HET=1;N_HOMALT=0;N_HOMREF=10308;FREQ_HET=9.700261907071492e-05;FREQ_HOMALT=0.0;FREQ_HOMREF=0.9999029973809292;N_BI_GENOS=10309;East_Han_AN=6554;East_Han_AC=1;East_Han_AF=
chr5	89897300	.	T	<INS:ME:LINE1>	.	PASS	SVTYPE=INS:ME:LINE1;END=89897300;SVLEN=205;AN=20618;AC=1;AF=4.850130953535746e-05;N_HET=1;N_HOMALT=0;N_HOMREF=10308;FREQ_HET=9.700261907071492e-05;FREQ_HOMALT=0.0;FREQ_HOMREF=0.9999029973809292;N_BI_GENOS=10309;East_Han_AN=6554;East_Han_AC=1;East_Han_AF=0
chr5	89999277	.	A	<INS:ME:LINE1>	.	PASS	SVTYPE=INS:ME:LINE1;END=89999278;SVLEN=1280;AN=20618;AC=3;AF=0.00014550392860607236;N_HET=3;N_HOMALT=0;N_HOMREF=10306;FREQ_HET=0.0002910078572121447;FREQ_HOMALT=0.0;FREQ_HOMREF=0.9997089921427879;N_BI_GENOS=10309;Lingnan_Han_AN=1888;Lingnan_Han_AC=3;Lingn
chr5	90046248	.	A	<INS:ME:LINE1>	.	PASS	SVTYPE=INS:ME:LINE1;END=90046264;SVLEN=6085;AN=20618;AC=4;AF=0.00019400523814142983;N_HET=4;N_HOMALT=0;N_HOMREF=10305;FREQ_HET=0.00038801047628285966;FREQ_HOMALT=0.0;FREQ_HOMREF=0.9996119895237171;N_BI_GENOS=10309;Lingnan_Han_AN=1888;Lingnan_Han_AC=1;Ling
chr5	90154951	.	G	<INS:ME:LINE1>	.	PASS	SVTYPE=INS:ME:LINE1;END=90154964;SVLEN=6000;AN=20618;AC=19143;AF=0.9284605684353477;N_HET=31;N_HOMALT=9556;N_HOMREF=722;FREQ_HET=0.0030070811911921624;FREQ_HOMALT=0.9269570278397516;FREQ_HOMREF=0.07003589096905616;N_BI_GENOS=10309;South_Han_AN=1496;South_
chr5	90155301	.	G	<INS:ME:LINE1>	.	PASS	SVTYPE=INS:ME:LINE1;END=90155301;SVLEN=177;AN=20618;AC=9;AF=0.0004365117858182171;N_HET=9;N_HOMALT=0;N_HOMREF=10300;FREQ_HET=0.0008730235716364342;FREQ_HOMALT=0.0;FREQ_HOMREF=0.9991269764283636;N_BI_GENOS=10309;South_Han_AN=1496;South_Han_AC=2;South_Han_A
chr5	90155490	.	T	<INS:ME:LINE1>	.	PASS	SVTYPE=INS:ME:LINE1;END=90155490;SVLEN=654;AN=20618;AC=26;AF=0.0012610340479192938;N_HET=26;N_HOMALT=0;N_HOMREF=10283;FREQ_HET=0.0025220680958385876;FREQ_HOMALT=0.0;FREQ_HOMREF=0.9974779319041615;N_BI_GENOS=10309;South_Han_AN=1496;South_Han_AC=5;South_Han
chr5	90598008	.	A	<INS:ME:LINE1>	.	PASS	SVTYPE=INS:ME:LINE1;END=90598017;SVLEN=5993;AN=20618;AC=1;AF=4.850130953535746e-05;N_HET=1;N_HOMALT=0;N_HOMREF=10308;FREQ_HET=9.700261907071492e-05;FREQ_HOMALT=0.0;FREQ_HOMREF=0.9999029973809292;N_BI_GENOS=10309;East_Han_AN=6554;East_Han_AC=1;East_Han_AF=
chr5	90605948	.	A	<INS:ME:LINE1>	.	PASS	SVTYPE=INS:ME:LINE1;END=90605948;SVLEN=2523;AN=20618;AC=2;AF=9.700261907071492e-05;N_HET=2;N_HOMALT=0;N_HOMREF=10307;FREQ_HET=0.00019400523814142983;FREQ_HOMALT=0.0;FREQ_HOMREF=0.9998059947618586;N_BI_GENOS=10309;East_Han_AN=6554;East_Han_AC=2;East_Han_AF
chr5	90640211	.	G	<INS:ME:LINE1>	.	PASS	SVTYPE=INS:ME:LINE1;END=90640225;SVLEN=2400;AN=20618;AC=1;AF=4.850130953535746e-05;N_HET=1;N_HOMALT=0;N_HOMREF=10308;FREQ_HET=9.700261907071492e-05;FREQ_HOMALT=0.0;FREQ_HOMREF=0.9999029973809292;N_BI_GENOS=10309;South_Han_AN=1496;South_Han_AC=1;South_Han_
chr5	90732002	.	T	<INS:ME:LINE1>	.	PASS	SVTYPE=INS:ME:LINE1;END=90732017;SVLEN=792;AN=20618;AC=1;AF=4.850130953535746e-05;N_HET=1;N_HOMALT=0;N_HOMREF=10308;FREQ_HET=9.700261907071492e-05;FREQ_HOMALT=0.0;FREQ_HOMREF=0.9999029973809292;N_BI_GENOS=10309;South_Han_AN=1496;South_Han_AC=1;South_Han_A
chr5	91168168	.	A	<INS:ME:LINE1>	.	PASS	SVTYPE=INS:ME:LINE1;END=91168174;SVLEN=469;AN=20618;AC=1;AF=4.850130953535746e-05;N_HET=1;N_HOMALT=0;N_HOMREF=10308;FREQ_HET=9.700261907071492e-05;FREQ_HOMALT=0.0;FREQ_HOMREF=0.9999029973809292;N_BI_GENOS=10309;South_Han_AN=1496;South_Han_AC=1;South_Han_A
chr5	91480358	.	A	<INS:ME:LINE1>	.	PASS	SVTYPE=INS:ME:LINE1;END=91480358;SVLEN=20;AN=20618;AC=1;AF=4.850130953535746e-05;N_HET=1;N_HOMALT=0;N_HOMREF=10308;FREQ_HET=9.700261907071492e-05;FREQ_HOMALT=0.0;FREQ_HOMREF=0.9999029973809292;N_BI_GENOS=10309;East_Han_AN=6554;East_Han_AC=1;East_Han_AF=0.
chr5	91589315	.	G	<INS:ME:LINE1>	.	PASS	SVTYPE=INS:ME:LINE1;END=91589333;SVLEN=6000;AN=20618;AC=4;AF=0.00019400523814142983;N_HET=4;N_HOMALT=0;N_HOMREF=10305;FREQ_HET=0.00038801047628285966;FREQ_HOMALT=0.0;FREQ_HOMREF=0.9996119895237171;N_BI_GENOS=10309;Northwest_Han_AN=1296;Northwest_Han_AC=1;
chr5	91591324	.	A	<INS:ME:LINE1>	.	PASS	SVTYPE=INS:ME:LINE1;END=91591339;SVLEN=6007;AN=20618;AC=1;AF=4.850130953535746e-05;N_HET=1;N_HOMALT=0;N_HOMREF=10308;FREQ_HET=9.700261907071492e-05;FREQ_HOMALT=0.0;FREQ_HOMREF=0.9999029973809292;N_BI_GENOS=10309;North_Han_AN=2272;North_Han_AC=1;North_Han_

chr5	91709274	.	A	<INS:ME:LINE1>	.	PASS	SVTYPE=INS:ME:LINE1;END=91709289;SVLEN=6006;AN=20618;AC=325;AF=0.015762925598991173;N_HET=303;N_HOMALT=11;N_HOMREF=9995;FREQ_HET=0.02939179357842662;FREQ_HOMALT=0.001067028809777864;FREQ_HOMREF=0.9695411776117955;N_BI_GENOS=10309;South_Han_AN=1496;South_H
chr5	91729048	.	C	<INS:ME:LINE1>	.	PASS	SVTYPE=INS:ME:LINE1;END=91729069;SVLEN=3382;AN=20618;AC=1;AF=4.850130953535746e-05;N_HET=1;N_HOMALT=0;N_HOMREF=10308;FREQ_HET=9.700261907071492e-05;FREQ_HOMALT=0.0;FREQ_HOMREF=0.9999029973809292;N_BI_GENOS=10309;North_Han_AN=2272;North_Han_AC=1;North_Han_
chr5	91760057	.	T	<INS:ME:LINE1>	.	PASS	SVTYPE=INS:ME:LINE1;END=91760073;SVLEN=5997;AN=20618;AC=33;AF=0.001600543214666796;N_HET=33;N_HOMALT=0;N_HOMREF=10276;FREQ_HET=0.003201086429333592;FREQ_HOMALT=0.0;FREQ_HOMREF=0.9967989135706664;N_BI_GENOS=10309;Northwest_Han_AN=1296;Northwest_Han_AC=1;No
chr5	91835337	.	G	<INS:ME:LINE1>	.	PASS	SVTYPE=INS:ME:LINE1;END=91835354;SVLEN=6000;AN=20618;AC=5;AF=0.0002425065476767872 8;N_HET=5;N_HOMALT=0;N_HOMREF=10304;FREQ_HET=0.0004850130953535 7455;FREQ_HOMALT=0.0;FREQ_HOMREF=0.9995149869046465;N_BI_GENOS=10309;South_Han_AN=1496;South_Han_AC=1;South_Ha
chr5	92169584	.	A	<INS:ME:LINE1>	.	PASS	SVTYPE=INS:ME:LINE1;END=92169599;SVLEN=1544;AN=20618;AC=2;AF=9.700261907071492e-05;N_HET=2;N_HOMALT=0;N_HOMREF=10307;FREQ_HET=0.00019400523814142983;FREQ_HOMALT=0.0;FREQ_HOMREF=0.9998059947618586;N_BI_GENOS=10309;Lingnan_Han_AN=1888;Lingnan_Han_AC=1;Lingn
chr5	92486308	.	A	<INS:ME:LINE1>	.	PASS	SVTYPE=INS:ME:LINE1;END=92486326;SVLEN=951;AN=20618;AC=1;AF=4.850130953535746e-05;N_HET=1;N_HOMALT=0;N_HOMREF=10308;FREQ_HET=9.700261907071492e-05;FREQ_HOMALT=0.0;FREQ_HOMREF=0.9999029973809292;N_BI_GENOS=10309;South_Han_AN=1496;South_Han_AC=1;South_Han_A
chr5	92741858	.	A	<INS:ME:LINE1>	.	PASS	SVTYPE=INS:ME:LINE1;END=92741874;SVLEN=1901;AN=20618;AC=1;AF=4.850130953535746e-05;N_HET=1;N_HOMALT=0;N_HOMREF=10308;FREQ_HET=9.700261907071492e-05;FREQ_HOMALT=0.0;FREQ_HOMREF=0.9999029973809292;N_BI_GENOS=10309;Northwest_Han_AN=1296;Northwest_Han_AC=1;No
chr5	92992821	.	A	<INS:ME:LINE1>	.	PASS	SVTYPE=INS:ME:LINE1;END=92992835;SVLEN=6026;AN=20618;AC=5;AF=0.00024250654767678728;N_HET=5;N_HOMALT=0;N_HOMREF=10304;FREQ_HET=0.00048501309535357455;FREQ_HOMALT=0.0;FREQ_HOMREF=0.9995149869046465;N_BI_GENOS=10309;Northwest_Han_AN=1296;Northwest_Han_AC=1;
chr5	93294321	.	G	<INS:ME:LINE1>	.	PASS	SVTYPE=INS:ME:LINE1;END=93294337;SVLEN=2808;AN=20618;AC=2;AF=9.700261907071492e-05;N_HET=2;N_HOMALT=0;N_HOMREF=10307;FREQ_HET=0.00019400523814142983;FREQ_HOMALT=0.0;FREQ_HOMREF=0.9998059947618586;N_BI_GENOS=10309;South_Han_AN=1496;South_Han_AC=1;South_Han
chr5	93662056	.	T	<INS:ME:LINE1>	.	PASS	SVTYPE=INS:ME:LINE1;END=93662061;SVLEN=693;AN=20618;AC=1;AF=4.850130953535746e-05;N_HET=1;N_HOMALT=0;N_HOMREF=10308;FREQ_HET=9.700261907071492e-05;FREQ_HOMALT=0.0;FREQ_HOMREF=0.9999029973809292;N_BI_GENOS=10309;East_Han_AN=6554;East_Han_AC=1;East_Han_AF=0
chr5	93785802	.	C	<INS:ME:LINE1>	.	PASS	SVTYPE=INS:ME:LINE1;END=93785816;SVLEN=923;AN=20618;AC=1;AF=4.850130953535746e-05;N_HET=1;N_HOMALT=0;N_HOMREF=10308;FREQ_HET=9.700261907071492e-05;FREQ_HOMALT=0.0;FREQ_HOMREF=0.9999029973809292;N_BI_GENOS=10309;East_Han_AN=6554;East_Han_AC=1;East_Han_AF=0
chr5	94246899	.	A	<INS:ME:LINE1>	.	PASS	SVTYPE=INS:ME:LINE1;END=94246899;SVLEN=5977;AN=20618;AC=1;AF=4.850130953535746e-05;N_HET=1;N_HOMALT=0;N_HOMREF=10308;FREQ_HET=9.700261907071492e-05;FREQ_HOMALT=0.0;FREQ_HOMREF=0.9999029973809292;N_BI_GENOS=10309;Southeast_Han_AN=1908;Southeast_Han_AC=1;So
chr5	94461008	.	G	<INS:ME:LINE1>	.	PASS	SVTYPE=INS:ME:LINE1;END=94461024;SVLEN=5999;AN=20618;AC=1;AF=4.850130953535746e-05;N_HET=1;N_HOMALT=0;N_HOMREF=10308;FREQ_HET=9.700261907071492e-05;FREQ_HOMALT=0.0;FREQ_HOMREF=0.9999029973809292;N_BI_GENOS=10309;Mongolian_AN=454;Mongolian_AC=1;Mongolian_A
chr5	94724995	.	C	<INS:ME:LINE1>	.	PASS	SVTYPE=INS:ME:LINE1;END=94725013;SVLEN=429;AN=20618;AC=1;AF=4.850130953535746e-05;N_HET=1;N_HOMALT=0;N_HOMREF=10308;FREQ_HET=9.700261907071492e-05;FREQ_HOMALT=0.0;FREQ_HOMREF=0.9999029973809292;N_BI_GENOS=10309;Northwest_Han_AN=1296;Northwest_Han_AC=1;Nor
chr5	94725159	.	C	<INS:ME:LINE1>	.	PASS	SVTYPE=INS:ME:LINE1;END=94725159;SVLEN=401;AN=20618;AC=1;AF=4.850130953535746e-05;N_HET=1;N_HOMALT=0;N_HOMREF=10308;FREQ_HET=9.700261907071492e-05;FREQ_HOMALT=0.0;FREQ_HOMREF=0.9999029973809292;N_BI_GENOS=10309;Northwest_Han_AN=1296;Northwest_Han_AC=1;Nor
chr5	94746512	.	G	<INS:ME:LINE1>	.	PASS	SVTYPE=INS:ME:LINE1;END=94746529;SVLEN=5988;AN=20618;AC=1;AF=4.850130953535746e-05;N_HET=1;N_HOMALT=0;N_HOMREF=10308;FREQ_HET=9.700261907071492e-05;FREQ_HOMALT=0.0;FREQ_HOMREF=0.9999029973809292;N_BI_GENOS=10309;Central_Han_AN=2128;Central_Han_AC=1;Centra
chr5	94913676	.	A	<INS:ME:LINE1>	.	PASS	SVTYPE=INS:ME:LINE1;END=94913688;SVLEN=195;AN=20618;AC=1;AF=4.850130953535746e-05;N_HET=1;N_HOMALT=0;N_HOMREF=10308;FREQ_HET=9.700261907071492e-05;FREQ_HOMALT=0.0;FREQ_HOMREF=0.9999029973809292;N_BI_GENOS=10309;North_Han_AN=2272;North_Han_AC=1;North_Han_A
chr5	95078569	.	A	<INS:ME:LINE1>	.	PASS	SVTYPE=INS:ME:LINE1;END=95078582;SVLEN=6010;AN=20618;AC=1;AF=4.850130953535746e-05;N_HET=1;N_HOMALT=0;N_HOMREF=10308;FREQ_HET=9.700261907071492e-05;FREQ_HOMALT=0.0;FREQ_HOMREF=0.9999029973809292;N_BI_GENOS=10309;Central_Han_AN=2128;Central_Han_AC=1;Centra
chr5	95558485	.	A	<INS:ME:LINE1>	.	PASS	SVTYPE=INS:ME:LINE1;END=95558501;SVLEN=5999;AN=20618;AC=2;AF=9.700261907071492e-05;N_HET=2;N_HOMALT=0;N_HOMREF=10307;FREQ_HET=0.00019400523814142983;FREQ_HOMALT=0.0;FREQ_HOMREF=0.9998059947618586;N_BI_GENOS=10309;East_Han_AN=6554;East_Han_AC=2;East_Han_AF
chr5	95729283	.	T	<INS:ME:LINE1>	.	PASS	SVTYPE=INS:ME:LINE1;END=95729297;SVLEN=6007;AN=20618;AC=1;AF=4.850130953535746e-05;N_HET=1;N_HOMALT=0;N_HOMREF=10308;FREQ_HET=9.700261907071492e-05;FREQ_HOMALT=0.0;FREQ_HOMREF=0.9999029973809292;N_BI_GENOS=10309;Lingnan_Han_AN=1888;Lingnan_Han_AC=1;Lingna
chr5	96622709	.	A	<INS:ME:LINE1>	.	PASS	SVTYPE=INS:ME:LINE1;END=96622725;SVLEN=1140;AN=20618;AC=1;AF=4.850130953535746e-05;N_HET=1;N_HOMALT=0;N_HOMREF=10308;FREQ_HET=9.700261907071492e-05;FREQ_HOMALT=0.0;FREQ_HOMREF=0.9999029973809292;N_BI_GENOS=10309;Central_Han_AN=2128;Central_Han_AC=1;Centra
chr5	96969028	.	A	<INS:ME:LINE1>	.	PASS	SVTYPE=INS:ME:LINE1;END=96969046;SVLEN=6000;AN=20618;AC=1;AF=4.850130953535746e-05;N_HET=1;N_HOMALT=0;N_HOMREF=10308;FREQ_HET=9.700261907071492e-05;FREQ_HOMALT=0.0;FREQ_HOMREF=0.9999029973809292;N_BI_GENOS=10309;East_Han_AN=6554;East_Han_AC=1;East_Han_AF=
chr5	97019726	.	G	<INS:ME:LINE1>	.	PASS	SVTYPE=INS:ME:LINE1;END=97019738;SVLEN=6006;AN=20618;AC=1;AF=4.850130953535746e-05;N_HET=1;N_HOMALT=0;N_HOMREF=10308;FREQ_HET=9.700261907071492e-05;FREQ_HOMALT=0.0;FREQ_HOMREF=0.9999029973809292;N_BI_GENOS=10309;Southeast_Han_AN=1908;Southeast_Han_AC=1;So
chr5	97231331	.	C	<INS:ME:LINE1>	.	PASS	SVTYPE=INS:ME:LINE1;END=97231341;SVLEN=2884;AN=20618;AC=12;AF=0.0005820157144242894;N_HET=12;N_HOMALT=0;N_HOMREF=10297;FREQ_HET=0.0011640314288485789;FREQ_HOMALT=0.0;FREQ_HOMREF=0.9988359685711514;N_BI_GENOS=10309;South_Han_AN=1496;South_Han_AC=2;South_Ha
chr5	97350731	.	A	<INS:ME:LINE1>	.	PASS	SVTYPE=INS:ME:LINE1;END=97350731;SVLEN=1039;AN=20618;AC=2;AF=9.700261907071492e-05;N_HET=0;N_HOMALT=1;N_HOMREF=10308;FREQ_HET=0.0;FREQ_HOMALT=9.700261907071492e-05;FREQ_HOMREF=0.9999029973809292;N_BI_GENOS=10309;East_Han_AN=6554;East_Han_AC=2;East_Han_AF=
chr5	97353017	.	T	<INS:ME:LINE1>	.	PASS	SVTYPE=INS:ME:LINE1;END=97353017;SVLEN=1112;AN=20618;AC=1;AF=4.850130953535746e-05;N_HET=1;N_HOMALT=0;N_HOMREF=10308;FREQ_HET=9.700261907071492e-05;FREQ_HOMALT=0.0;FREQ_HOMREF=0.9999029973809292;N_BI_GENOS=10309;East_Han_AN=6554;East_Han_AC=1;East_Han_AF=
chr5	97530702	.	A	<INS:ME:LINE1>	.	PASS	SVTYPE=INS:ME:LINE1;END=97530719;SVLEN=620;AN=20618;AC=3261;AF=0.15816277039480067;N_HET=2501;N_HOMALT=380;N_HOMREF=7428;FREQ_HET=0.24260355029585798;FREQ_HOMALT=0.03686099524687166;FREQ_HOMREF=0.7205354544572703;N_BI_GENOS=10309;South_Han_AN=1496;South_H
chr5	97552498	.	T	<INS:ME:LINE1>	.	PASS	SVTYPE=INS:ME:LINE1;END=97552513;SVLEN=2181;AN=20618;AC=2;AF=9.700261907071492e-05;N_HET=2;N_HOMALT=0;N_HOMREF=10307;FREQ_HET=0.00019400523814142983;FREQ_HOMALT=0.0;FREQ_HOMREF=0.9998059947618586;N_BI_GENOS=10309;East_Han_AN=6554;East_Han_AC=2;East_Han_AF
chr5	97561567	.	A	<INS:ME:LINE1>	.	PASS	SVTYPE=INS:ME:LINE1;END=97561581;SVLEN=202;AN=20618;AC=2;AF=9.700261907071492e-05;N_HET=2;N_HOMALT=0;N_HOMREF=10307;FREQ_HET=0.00019400523814142983;FREQ_HOMALT=0.0;FREQ_HOMREF=0.9998059947618586;N_BI_GENOS=10309;East_Han_AN=6554;East_Han_AC=1;East_Han_AF=
chr5	97766079	.	T	<INS:ME:LINE1>	.	PASS	SVTYPE=INS:ME:LINE1;END=97766093;SVLEN=493;AN=20618;AC=8;AF=0.0003880104762828596 6;N_HET=8;N_HOMALT=0;N_HOMREF=10301;FREQ_HET=0.0007760209525657193;FREQ_HOMALT=0.0;FREQ_HOMREF=0.9992239790474343;N_BI_GENOS=10309;South_Han_AN=1496;South_Han_AC=1;South_Han_
chr5	98001588	.	A	<INS:ME:LINE1>	.	PASS	SVTYPE=INS:ME:LINE1;END=98001604;SVLEN=781;AN=20618;AC=1;AF=4.850130953535746e-05;N_HET=1;N_HOMALT=0;N_HOMREF=10308;FREQ_HET=9.700261907071492e-05;FREQ_HOMALT=0.0;FREQ_HOMREF=0.9999029973809292;N_BI_GENOS=10309;East_Han_AN=6554;East_Han_AC=1;East_Han_AF=0
chr5	98046155	.	T	<INS:ME:LINE1>	.	PASS	SVTYPE=INS:ME:LINE1;END=98046169;SVLEN=2553;AN=20618;AC=1;AF=4.850130953535746e-05;N_HET=1;N_HOMALT=0;N_HOMREF=10308;FREQ_HET=9.700261907071492e-05;FREQ_HOMALT=0.0;FREQ_HOMREF=0.9999029973809292;N_BI_GENOS=10309;Manchu_AN=416;Manchu_AC=1;Manchu_AF=0.00240
chr5	98156373	.	A	<INS:ME:LINE1>	.	PASS	SVTYPE=INS:ME:LINE1;END=98156373;SVLEN=107;AN=20618;AC=1;AF=4.850130953535746e-05;N_HET=1;N_HOMALT=0;N_HOMREF=10308;FREQ_HET=9.700261907071492e-05;FREQ_HOMALT=0.0;FREQ_HOMREF=0.9999029973809292;N_BI_GENOS=10309;Southeast_Han_AN=1908;Southeast_Han_AC=1;Sou
chr5	99033987	.	A	<INS:ME:LINE1>	.	PASS	SVTYPE=INS:ME:LINE1;END=99034008;SVLEN=4239;AN=20618;AC=1;AF=4.850130953535746e-05;N_HET=1;N_HOMALT=0;N_HOMREF=10308;FREQ_HET=9.700261907071492e-05;FREQ_HOMALT=0.0;FREQ_HOMREF=0.9999029973809292;N_BI_GENOS=10309;Northwest_Han_AN=1296;Northwest_Han_AC=1;No
chr5	99189244	.	T	<INS:ME:LINE1>	.	PASS	SVTYPE=INS:ME:LINE1;END=99189259;SVLEN=707;AN=20618;AC=3;AF=0.00014550392860607236;N_HET=3;N_HOMALT=0;N_HOMREF=10306;FREQ_HET=0.0002910078572121447;FREQ_HOMALT=0.0;FREQ_HOMREF=0.9997089921427879;N_BI_GENOS=10309;East_Han_AN=6554;East_Han_AC=2;East_Han_AF=
chr5	99371194	.	A	<INS:ME:LINE1>	.	PASS	SVTYPE=INS:ME:LINE1;END=99371200;SVLEN=735;AN=20618;AC=2;AF=9.700261907071492e-05;N_HET=2;N_HOMALT=0;N_HOMREF=10307;FREQ_HET=0.00019400523814142983;FREQ_HOMALT=0.0;FREQ_HOMREF=0.9998059947618586;N_BI_GENOS=10309;Northwest_Han_AN=1296;Northwest_Han_AC=1;No
chr5	99399200	.	A	<INS:ME:LINE1>	.	PASS	SVTYPE=INS:ME:LINE1;END=99399218;SVLEN=317;AN=20618;AC=2;AF=9.700261907071492e-05;N_HET=2;N_HOMALT=0;N_HOMREF=10307;FREQ_HET=0.00019400523814142983;FREQ_HOMALT=0.0;FREQ_HOMREF=0.9998059947618586;N_BI_GENOS=10309;Northwest_Han_AN=1296;Northwest_Han_AC=1;No
chr5	99466125	.	T	<INS:ME:LINE1>	.	PASS	SVTYPE=INS:ME:LINE1;END=99466126;SVLEN=63;AN=20618;AC=3;AF=0.00014550392860607236;N_HET=3;N_HOMALT=0;N_HOMREF=10306;FREQ_HET=0.0002910078572121447;FREQ_HOMALT=0.0;FREQ_HOMREF=0.9997089921427879;N_BI_GENOS=10309;East_Han_AN=6554;East_Han_AC=2;East_Han_AF=0
chr5	99466377	.	A	<INS:ME:LINE1>	.	PASS	SVTYPE=INS:ME:LINE1;END=99466377;SVLEN=1668;AN=20618;AC=2;AF=9.700261907071492e-05;N_HET=0;N_HOMALT=1;N_HOMREF=10308;FREQ_HET=0.0;FREQ_HOMALT=9.700261907071492e-05;FREQ_HOMREF=0.9999029973809292;N_BI_GENOS=10309;Northwest_Han_AN=1296;Northwest_Han_AC=2;No
chr5	99588159	.	A	<INS:ME:LINE1>	.	PASS	SVTYPE=INS:ME:LINE1;END=99588162;SVLEN=655;AN=20618;AC=1;AF=4.850130953535746e-05;N_HET=1;N_HOMALT=0;N_HOMREF=10308;FREQ_HET=9.700261907071492e-05;FREQ_HOMALT=0.0;FREQ_HOMREF=0.9999029973809292;N_BI_GENOS=10309;East_Han_AN=6554;East_Han_AC=1;East_Han_AF=0
chr5	99588626	.	A	<INS:ME:LINE1>	.	PASS	SVTYPE=INS:ME:LINE1;END=99588637;SVLEN=858;AN=20618;AC=1;AF=4.850130953535746e-05;N_HET=1;N_HOMALT=0;N_HOMREF=10308;FREQ_HET=9.700261907071492e-05;FREQ_HOMALT=0.0;FREQ_HOMREF=0.9999029973809292;N_BI_GENOS=10309;North_Han_AN=2272;North_Han_AC=1;North_Han_A
chr5	99594521	.	G	<INS:ME:LINE1>	.	PASS	SVTYPE=INS:ME:LINE1;END=99594536;SVLEN=5215;AN=20618;AC=1;AF=4.850130953535746e-05;N_HET=1;N_HOMALT=0;N_HOMREF=10308;FREQ_HET=9.700261907071492e-05;FREQ_HOMALT=0.0;FREQ_HOMREF=0.9999029973809292;N_BI_GENOS=10309;East_Han_AN=6554;East_Han_AC=1;East_Han_AF=
chr5	99665647	.	A	<INS:ME:LINE1>	.	PASS	SVTYPE=INS:ME:LINE1;END=99665662;SVLEN=1586;AN=20618;AC=1;AF=4.850130953535746e-05;N_HET=1;N_HOMALT=0;N_HOMREF=10308;FREQ_HET=9.700261907071492e-05;FREQ_HOMALT=0.0;FREQ_HOMREF=0.9999029973809292;N_BI_GENOS=10309;Southeast_Han_AN=1908;Southeast_Han_AC=1;So
chr5	99832159	.	T	<INS:ME:LINE1>	.	PASS	SVTYPE=INS:ME:LINE1;END=99832173;SVLEN=2212;AN=20618;AC=1;AF=4.850130953535746e-05;N_HET=1;N_HOMALT=0;N_HOMREF=10308;FREQ_HET=9.700261907071492e-05;FREQ_HOMALT=0.0;FREQ_HOMREF=0.9999029973809292;N_BI_GENOS=10309;South_Han_AN=1496;South_Han_AC=1;South_Han_
chr5	100000413	.	A	<INS:ME:LINE1>	.	PASS	SVTYPE=INS:ME:LINE1;END=100000413;SVLEN=382;AN=20618;AC=1;AF=4.850130953535746e-05;N_HET=1;N_HOMALT=0;N_HOMREF=10308;FREQ_HET=9.700261907071492e-05;FREQ_HOMALT=0.0;FREQ_HOMREF=0.9999029973809292;N_BI_GENOS=10309;South_Han_AN=1496;South_Han_AC=1;South_Han_
chr5	100451495	.	T	<INS:ME:LINE1>	.	PASS	SVTYPE=INS:ME:LINE1;END=100451495;SVLEN=1571;AN=20618;AC=1;AF=4.850130953535746e-05;N_HET=1;N_HOMALT=0;N_HOMREF=10308;FREQ_HET=9.700261907071492e-05;FREQ_HOMALT=0.0;FREQ_HOMREF=0.9999029973809292;N_BI_GENOS=10309;East_Han_AN=6554;East_Han_AC=1;East_Han_AF
chr5	100451548	.	G	<INS:ME:LINE1>	.	PASS	SVTYPE=INS:ME:LINE1;END=100451548;SVLEN=478;AN=20618;AC=1;AF=4.850130953535746e-05;N_HET=1;N_HOMALT=0;N_HOMREF=10308;FREQ_HET=9.700261907071492e-05;FREQ_HOMALT=0.0;FREQ_HOMREF=0.9999029973809292;N_BI_GENOS=10309;East_Han_AN=6554;East_Han_AC=1;East_Han_AF=
chr5	100451841	.	C	<INS:ME:LINE1>	.	PASS	SVTYPE=INS:ME:LINE1;END=100451854;SVLEN=2107;AN=20618;AC=1;AF=4.850130953535746e-05;N_HET=1;N_HOMALT=0;N_HOMREF=10308;FREQ_HET=9.700261907071492e-05;FREQ_HOMALT=0.0;FREQ_HOMREF=0.9999029973809292;N_BI_GENOS=10309;East_Han_AN=6554;East_Han_AC=1;East_Han_AF
chr5	100529430	.	T	<INS:ME:LINE1>	.	PASS	SVTYPE=INS:ME:LINE1;END=100529501;SVLEN=1057;AN=20618;AC=1;AF=4.850130953535746e-05;N_HET=1;N_HOMALT=0;N_HOMREF=10308;FREQ_HET=9.700261907071492e-05;FREQ_HOMALT=0.0;FREQ_HOMREF=0.9999029973809292;N_BI_GENOS=10309;East_Han_AN=6554;East_Han_AC=1;East_Han_AF
chr5	100695550	.	A	<INS:ME:LINE1>	.	PASS	SVTYPE=INS:ME:LINE1;END=100695565;SVLEN=501;AN=20618;AC=1;AF=4.850130953535746e-05;N_HET=1;N_HOMALT=0;N_HOMREF=10308;FREQ_HET=9.700261907071492e-05;FREQ_HOMALT=0.0;FREQ_HOMREF=0.9999029973809292;N_BI_GENOS=10309;East_Han_AN=6554;East_Han_AC=1;East_Han_AF=
chr5	100886805	.	T	<INS:ME:LINE1>	.	PASS	SVTYPE=INS:ME:LINE1;END=100886818;SVLEN=1235;AN=20618;AC=1;AF=4.850130953535746e-05;N_HET=1;N_HOMALT=0;N_HOMREF=10308;FREQ_HET=9.700261907071492e-05;FREQ_HOMALT=0.0;FREQ_HOMREF=0.9999029973809292;N_BI_GENOS=10309;Yi_AN=422;Yi_AC=1;Yi_AF=0.0023696682464454
chr5	100895112	.	C	<INS:ME:LINE1>	.	PASS	SVTYPE=INS:ME:LINE1;END=100895129;SVLEN=1257;AN=20618;AC=3;AF=0.00014550392860607236;N_HET=3;N_HOMALT=0;N_HOMREF=10306;FREQ_HET=0.0002910078572121447;FREQ_HOMALT=0.0;FREQ_HOMREF=0.9997089921427879;N_BI_GENOS=10309;East_Han_AN=6554;East_Han_AC=1;East_Han_A
chr5	101052330	.	G	<INS:ME:LINE1>	.	PASS	SVTYPE=INS:ME:LINE1;END=101052345;SVLEN=6016;AN=20618;AC=1;AF=4.850130953535746e-05;N_HET=1;N_HOMALT=0;N_HOMREF=10308;FREQ_HET=9.700261907071492e-05;FREQ_HOMALT=0.0;FREQ_HOMREF=0.9999029973809292;N_BI_GENOS=10309;South_Han_AN=1496;South_Han_AC=1;South_Han
chr5	101190157	.	A	<INS:ME:LINE1>	.	PASS	SVTYPE=INS:ME:LINE1;END=101190173;SVLEN=434;AN=20618;AC=1;AF=4.850130953535746e-05;N_HET=1;N_HOMALT=0;N_HOMREF=10308;FREQ_HET=9.700261907071492e-05;FREQ_HOMALT=0.0;FREQ_HOMREF=0.9999029973809292;N_BI_GENOS=10309;East_Han_AN=6554;East_Han_AC=1;East_Han_AF=
chr5	101319416	.	A	<INS:ME:LINE1>	.	PASS	SVTYPE=INS:ME:LINE1;END=101319416;SVLEN=0;AN=20618;AC=7;AF=0.0003395091667475022;N_HET=7;N_HOMALT=0;N_HOMREF=10302;FREQ_HET=0.0006790183334950044;FREQ_HOMALT=0.0;FREQ_HOMREF=0.999320981666505;N_BI_GENOS=10309;East_Han_AN=6554;East_Han_AC=2;East_Han_AF=0.0
chr5	101409104	.	A	<INS:ME:LINE1>	.	PASS	SVTYPE=INS:ME:LINE1;END=101409104;SVLEN=666;AN=20618;AC=1;AF=4.850130953535746e-05;N_HET=1;N_HOMALT=0;N_HOMREF=10308;FREQ_HET=9.700261907071492e-05;FREQ_HOMALT=0.0;FREQ_HOMREF=0.9999029973809292;N_BI_GENOS=10309;East_Han_AN=6554;East_Han_AC=1;East_Han_AF=
chr5	101593215	.	A	<INS:ME:LINE1>	.	PASS	SVTYPE=INS:ME:LINE1;END=101593231;SVLEN=978;AN=20618;AC=1;AF=4.850130953535746e-05;N_HET=1;N_HOMALT=0;N_HOMREF=10308;FREQ_HET=9.700261907071492e-05;FREQ_HOMALT=0.0;FREQ_HOMREF=0.9999029973809292;N_BI_GENOS=10309;Lingnan_Han_AN=1888;Lingnan_Han_AC=1;Lingna
chr5	101871004	.	A	<INS:ME:LINE1>	.	PASS	SVTYPE=INS:ME:LINE1;END=101871017;SVLEN=6038;AN=20618;AC=1;AF=4.850130953535746e-05;N_HET=1;N_HOMALT=0;N_HOMREF=10308;FREQ_HET=9.700261907071492e-05;FREQ_HOMALT=0.0;FREQ_HOMREF=0.9999029973809292;N_BI_GENOS=10309;North_Han_AN=2272;North_Han_AC=1;North_Han
chr5	101900921	.	A	<INS:ME:LINE1>	.	PASS	SVTYPE=INS:ME:LINE1;END=101900921;SVLEN=2391;AN=20618;AC=1;AF=4.850130953535746e-05;N_HET=1;N_HOMALT=0;N_HOMREF=10308;FREQ_HET=9.700261907071492e-05;FREQ_HOMALT=0.0;FREQ_HOMREF=0.9999029973809292;N_BI_GENOS=10309;Tibetan_AN=414;Tibetan_AC=1;Tibetan_AF=0.0
chr5	101937201	.	T	<INS:ME:LINE1>	.	PASS	SVTYPE=INS:ME:LINE1;END=101937201;SVLEN=154;AN=20618;AC=1;AF=4.850130953535746e-05;N_HET=1;N_HOMALT=0;N_HOMREF=10308;FREQ_HET=9.700261907071492e-05;FREQ_HOMALT=0.0;FREQ_HOMREF=0.9999029973809292;N_BI_GENOS=10309;East_Han_AN=6554;East_Han_AC=1;East_Han_AF=
chr5	102036612	.	T	<INS:ME:LINE1>	.	PASS	SVTYPE=INS:ME:LINE1;END=102036619;SVLEN=199;AN=20618;AC=1;AF=4.850130953535746e-05;N_HET=1;N_HOMALT=0;N_HOMREF=10308;FREQ_HET=9.700261907071492e-05;FREQ_HOMALT=0.0;FREQ_HOMREF=0.9999029973809292;N_BI_GENOS=10309;Tibetan_AN=414;Tibetan_AC=1;Tibetan_AF=0.00
chr5	102041140	.	A	<INS:ME:LINE1>	.	PASS	SVTYPE=INS:ME:LINE1;END=102041156;SVLEN=825;AN=20618;AC=1;AF=4.850130953535746e-05;N_HET=1;N_HOMALT=0;N_HOMREF=10308;FREQ_HET=9.700261907071492e-05;FREQ_HOMALT=0.0;FREQ_HOMREF=0.9999029973809292;N_BI_GENOS=10309;Central_Han_AN=2128;Central_Han_AC=1;Centra
chr5	102081011	.	A	<INS:ME:LINE1>	.	PASS	SVTYPE=INS:ME:LINE1;END=102081022;SVLEN=6024;AN=20618;AC=2;AF=9.700261907071492e-05;N_HET=2;N_HOMALT=0;N_HOMREF=10307;FREQ_HET=0.00019400523814142983;FREQ_HOMALT=0.0;FREQ_HOMREF=0.9998059947618586;N_BI_GENOS=10309;North_Han_AN=2272;North_Han_AC=1;North_Ha
chr5	102418103	.	A	<INS:ME:LINE1>	.	PASS	SVTYPE=INS:ME:LINE1;END=102418122;SVLEN=5997;AN=20618;AC=52;AF=0.0025220680958385876;N_HET=50;N_HOMALT=1;N_HOMREF=10258;FREQ_HET=0.004850130953535745;FREQ_HOMALT=9.700261907071492e-05;FREQ_HOMREF=0.9950528664273935;N_BI_GENOS=10309;South_Han_AN=1496;South
chr5	102578053	.	G	<INS:ME:LINE1>	.	PASS	SVTYPE=INS:ME:LINE1;END=102578059;SVLEN=323;AN=20618;AC=8;AF=0.00038801047628285966;N_HET=2;N_HOMALT=3;N_HOMREF=10304;FREQ_HET=0.00019400523814142983;FREQ_HOMALT=0.0002910078572121447;FREQ_HOMREF=0.9995149869046465;N_BI_GENOS=10309;East_Han_AN=6554;East_H
chr5	102659848	.	A	<INS:ME:LINE1>	.	PASS	SVTYPE=INS:ME:LINE1;END=102659865;SVLEN=5998;AN=20618;AC=2;AF=9.700261907071492e-05;N_HET=2;N_HOMALT=0;N_HOMREF=10307;FREQ_HET=0.00019400523814142983;FREQ_HOMALT=0.0;FREQ_HOMREF=0.9998059947618586;N_BI_GENOS=10309;Central_Han_AN=2128;Central_Han_AC=1;Cent
chr5	103024897	.	A	<INS:ME:LINE1>	.	PASS	SVTYPE=INS:ME:LINE1;END=103024910;SVLEN=500;AN=20618;AC=5;AF=0.00024250654767678728;N_HET=3;N_HOMALT=1;N_HOMREF=10305;FREQ_HET=0.0002910078572121447;FREQ_HOMALT=9.700261907071492e-05;FREQ_HOMREF=0.9996119895237171;N_BI_GENOS=10309;South_Han_AN=1496;South_
chr5	103045054	.	T	<INS:ME:LINE1>	.	PASS	SVTYPE=INS:ME:LINE1;END=103045069;SVLEN=6001;AN=20618;AC=1;AF=4.850130953535746e-05;N_HET=1;N_HOMALT=0;N_HOMREF=10308;FREQ_HET=9.700261907071492e-05;FREQ_HOMALT=0.0;FREQ_HOMREF=0.9999029973809292;N_BI_GENOS=10309;Mongolian_AN=454;Mongolian_AC=1;Mongolian_
chr5	103357636	.	A	<INS:ME:LINE1>	.	PASS	SVTYPE=INS:ME:LINE1;END=103357648;SVLEN=442;AN=20618;AC=87;AF=0.004219613929576099;N_HET=83;N_HOMALT=2;N_HOMREF=10224;FREQ_HET=0.008051217382869338;FREQ_HOMALT=0.00019400523814142983;FREQ_HOMREF=0.9917547773789892;N_BI_GENOS=10309;South_Han_AN=1496;South_
chr5	103411920	.	A	<INS:ME:LINE1>	.	PASS	SVTYPE=INS:ME:LINE1;END=103411937;SVLEN=939;AN=20618;AC=284;AF=0.01377437190804151 8;N_HET=274;N_HOMALT=5;N_HOMREF=10030;FREQ_HET=0.026578717625375885;FREQ_HOMALT=0.00048501309535357455;FREQ_HOMREF=0.9729362692792706;N_BI_GENOS=10309;South_Han_AN=1496;Sout
chr5	103760110	.	A	<INS:ME:LINE1>	.	PASS	SVTYPE=INS:ME:LINE1;END=103760125;SVLEN=651;AN=20618;AC=1;AF=4.850130953535746e-05;N_HET=1;N_HOMALT=0;N_HOMREF=10308;FREQ_HET=9.700261907071492e-05;FREQ_HOMALT=0.0;FREQ_HOMREF=0.9999029973809292;N_BI_GENOS=10309;East_Han_AN=6554;East_Han_AC=1;East_Han_AF=
chr5	104542019	.	G	<INS:ME:LINE1>	.	PASS	SVTYPE=INS:ME:LINE1;END=104542037;SVLEN=6000;AN=20618;AC=57;AF=0.0027645746435153748;N_HET=57;N_HOMALT=0;N_HOMREF=10252;FREQ_HET=0.0055291492870307496;FREQ_HOMALT=0.0;FREQ_HOMREF=0.9944708507129693;N_BI_GENOS=10309;South_Han_AN=1496;South_Han_AC=3;South_H
chr5	104578743	.	A	<INS:ME:LINE1>	.	PASS	SVTYPE=INS:ME:LINE1;END=104578761;SVLEN=392;AN=20618;AC=3;AF=0.00014550392860607236;N_HET=3;N_HOMALT=0;N_HOMREF=10306;FREQ_HET=0.0002910078572121447;FREQ_HOMALT=0.0;FREQ_HOMREF=0.9997089921427879;N_BI_GENOS=10309;North_Han_AN=2272;North_Han_AC=1;North_Han
chr5	104650350	.	A	<INS:ME:LINE1>	.	PASS	SVTYPE=INS:ME:LINE1;END=104650367;SVLEN=649;AN=20618;AC=5;AF=0.00024250654767678728;N_HET=5;N_HOMALT=0;N_HOMREF=10304;FREQ_HET=0.00048501309535357455;FREQ_HOMALT=0.0;FREQ_HOMREF=0.9995149869046465;N_BI_GENOS=10309;East_Han_AN=6554;East_Han_AC=3;East_Han_A
chr5	104763939	.	A	<INS:ME:LINE1>	.	PASS	SVTYPE=INS:ME:LINE1;END=104763956;SVLEN=958;AN=20618;AC=4;AF=0.00019400523814142983;N_HET=4;N_HOMALT=0;N_HOMREF=10305;FREQ_HET=0.00038801047628285966;FREQ_HOMALT=0.0;FREQ_HOMREF=0.9996119895237171;N_BI_GENOS=10309;South_Han_AN=1496;South_Han_AC=2;South_Ha
chr5	104780598	.	A	<INS:ME:LINE1>	.	PASS	SVTYPE=INS:ME:LINE1;END=104780598;SVLEN=899;AN=20618;AC=1;AF=4.850130953535746e-05;N_HET=1;N_HOMALT=0;N_HOMREF=10308;FREQ_HET=9.700261907071492e-05;FREQ_HOMALT=0.0;FREQ_HOMREF=0.9999029973809292;N_BI_GENOS=10309;Hui_AN=444;Hui_AC=1;Hui_AF=0.0022522522525225
chr5	104880978	.	G	<INS:ME:LINE1>	.	PASS	SVTYPE=INS:ME:LINE1;END=104880992;SVLEN=1351;AN=20618;AC=1;AF=4.850130953535746e-05;N_HET=1;N_HOMALT=0;N_HOMREF=10308;FREQ_HET=9.700261907071492e-05;FREQ_HOMALT=0.0;FREQ_HOMREF=0.9999029973809292;N_BI_GENOS=10309;Mongolian_AN=454;Mongolian_AC=1;Mongolian_
chr5	106395858	.	G	<INS:ME:LINE1>	.	PASS	SVTYPE=INS:ME:LINE1;END=106395868;SVLEN=292;AN=20618;AC=1;AF=4.850130953535746e-05;N_HET=1;N_HOMALT=0;N_HOMREF=10308;FREQ_HET=9.700261907071492e-05;FREQ_HOMALT=0.0;FREQ_HOMREF=0.9999029973809292;N_BI_GENOS=10309;Lingnan_Han_AN=1888;Lingnan_Han_AC=1;Lingna
chr5	106422476	.	G	<INS:ME:LINE1>	.	PASS	SVTYPE=INS:ME:LINE1;END=106422485;SVLEN=5988;AN=20618;AC=1;AF=4.850130953535746e-05;N_HET=1;N_HOMALT=0;N_HOMREF=10308;FREQ_HET=9.700261907071492e-05;FREQ_HOMALT=0.0;FREQ_HOMREF=0.9999029973809292;N_BI_GENOS=10309;East_Han_AN=6554;East_Han_AC=1;East_Han_AF
chr5	106507646	.	G	<INS:ME:LINE1>	.	PASS	SVTYPE=INS:ME:LINE1;END=106507646;SVLEN=3982;AN=20618;AC=1;AF=4.850130953535746e-05;N_HET=1;N_HOMALT=0;N_HOMREF=10308;FREQ_HET=9.700261907071492e-05;FREQ_HOMALT=0.0;FREQ_HOMREF=0.9999029973809292;N_BI_GENOS=10309;Lingnan_Han_AN=1888;Lingnan_Han_AC=1;Lingn
chr5	106518345	.	A	<INS:ME:LINE1>	.	PASS	SVTYPE=INS:ME:LINE1;END=106518345;SVLEN=1350;AN=20618;AC=2;AF=9.700261907071492e-05;N_HET=2;N_HOMALT=0;N_HOMREF=10307;FREQ_HET=0.00019400523814142983;FREQ_HOMALT=0.0;FREQ_HOMREF=0.9998059947618586;N_BI_GENOS=10309;Lingnan_Han_AN=1888;Lingnan_Han_AC=1;Ling
chr5	106576509	.	G	<INS:ME:LINE1>	.	PASS	SVTYPE=INS:ME:LINE1;END=106576524;SVLEN=634;AN=20618;AC=9;AF=0.00043651178581821 71;N_HET=9;N_HOMALT=0;N_HOMREF=10300;FREQ_HET=0.0008730235716364342;FREQ_HOMALT=0.0;FREQ_HOMREF=0.9991269764283636;N_BI_GENOS=10309;Northwest_Han_AN=1296;Northwest_Han_AC=3;No
chr5	106599662	.	A	<INS:ME:LINE1>	.	PASS	SVTYPE=INS:ME:LINE1;END=106599672;SVLEN=5993;AN=20618;AC=3609;AF=0.17504122611310505;N_HET=2631;N_HOMALT=489;N_HOMREF=7189;FREQ_HET=0.2552138907750509;FREQ_HOMALT=0.04743428072557959;FREQ_HOMREF=0.6973518284993695;N_BI_GENOS=10309;South_Han_AN=1496;South_
chr5	106664690	.	C	<INS:ME:LINE1>	.	PASS	SVTYPE=INS:ME:LINE1;END=106664702;SVLEN=5998;AN=20618;AC=12;AF=0.0005820157144242894;N_HET=12;N_HOMALT=0;N_HOMREF=10297;FREQ_HET=0.0011640314288485789;FREQ_HOMALT=0.0;FREQ_HOMREF=0.9988359685711514;N_BI_GENOS=10309;South_Han_AN=1496;South_Han_AC=1;South_H
chr5	106845192	.	T	<INS:ME:LINE1>	.	PASS	SVTYPE=INS:ME:LINE1;END=106845192;SVLEN=10;AN=20618;AC=1;AF=4.850130953535746e-05;N_HET=1;N_HOMALT=0;N_HOMREF=10308;FREQ_HET=9.700261907071492e-05;FREQ_HOMALT=0.0;FREQ_HOMREF=0.9999029973809292;N_BI_GENOS=10309;South_Han_AN=1496;South_Han_AC=1;South_Han_A
chr5	106978802	.	T	<INS:ME:LINE1>	.	PASS	SVTYPE=INS:ME:LINE1;END=106978802;SVLEN=0;AN=20618;AC=2;AF=9.700261907071492e-05;N_HET=0;N_HOMALT=1;N_HOMREF=10308;FREQ_HET=0.0;FREQ_HOMALT=9.700261907071492e-05;FREQ_HOMREF=0.9999029973809292;N_BI_GENOS=10309;Yi_AN=422;Yi_AC=2;Yi_AF=0.004739336492890996;
chr5	107731298	.	A	<INS:ME:LINE1>	.	PASS	SVTYPE=INS:ME:LINE1;END=107731311;SVLEN=123;AN=20618;AC=1;AF=4.850130953535746e-05;N_HET=1;N_HOMALT=0;N_HOMREF=10308;FREQ_HET=9.700261907071492e-05;FREQ_HOMALT=0.0;FREQ_HOMREF=0.9999029973809292;N_BI_GENOS=10309;Central_Han_AN=2128;Central_Han_AC=1;Centra
chr5	107887767	.	C	<INS:ME:LINE1>	.	PASS	SVTYPE=INS:ME:LINE1;END=107887783;SVLEN=1771;AN=20618;AC=1;AF=4.850130953535746e-05;N_HET=1;N_HOMALT=0;N_HOMREF=10308;FREQ_HET=9.700261907071492e-05;FREQ_HOMALT=0.0;FREQ_HOMREF=0.9999029973809292;N_BI_GENOS=10309;East_Han_AN=6554;East_Han_AC=1;East_Han_AF
chr5	108337675	.	A	<INS:ME:LINE1>	.	PASS	SVTYPE=INS:ME:LINE1;END=108337685;SVLEN=729;AN=20618;AC=1;AF=4.850130953535746e-05;N_HET=1;N_HOMALT=0;N_HOMREF=10308;FREQ_HET=9.700261907071492e-05;FREQ_HOMALT=0.0;FREQ_HOMREF=0.9999029973809292;N_BI_GENOS=10309;East_Han_AN=6554;East_Han_AC=1;East_Han_AF=
chr5	108416063	.	A	<INS:ME:LINE1>	.	PASS	SVTYPE=INS:ME:LINE1;END=108416080;SVLEN=6042;AN=20618;AC=2;AF=9.700261907071492e-05;N_HET=2;N_HOMALT=0;N_HOMREF=10307;FREQ_HET=0.00019400523814142983;FREQ_HOMALT=0.0;FREQ_HOMREF=0.9998059947618586;N_BI_GENOS=10309;Zhuang_AN=444;Zhuang_AC=1;Zhuang_AF=0.002
chr5	108509126	.	G	<INS:ME:LINE1>	.	PASS	SVTYPE=INS:ME:LINE1;END=108509142;SVLEN=6052;AN=20618;AC=1;AF=4.850130953535746e-05;N_HET=1;N_HOMALT=0;N_HOMREF=10308;FREQ_HET=9.700261907071492e-05;FREQ_HOMALT=0.0;FREQ_HOMREF=0.9999029973809292;N_BI_GENOS=10309;East_Han_AN=6554;East_Han_AC=1;East_Han_AF
chr5	108767397	.	G	<INS:ME:LINE1>	.	PASS	SVTYPE=INS:ME:LINE1;END=108767397;SVLEN=5940;AN=20618;AC=1;AF=4.850130953535746e-05;N_HET=1;N_HOMALT=0;N_HOMREF=10308;FREQ_HET=9.700261907071492e-05;FREQ_HOMALT=0.0;FREQ_HOMREF=0.9999029973809292;N_BI_GENOS=10309;Central_Han_AN=2128;Central_Han_AC=1;Centr
chr5	109024016	.	G	<INS:ME:LINE1>	.	PASS	SVTYPE=INS:ME:LINE1;END=109024031;SVLEN=5999;AN=20618;AC=1;AF=4.850130953535746e-05;N_HET=1;N_HOMALT=0;N_HOMREF=10308;FREQ_HET=9.700261907071492e-05;FREQ_HOMALT=0.0;FREQ_HOMREF=0.9999029973809292;N_BI_GENOS=10309;Manchu_AN=416;Manchu_AC=1;Manchu_AF=0.0024
chr5	109084592	.	A	<INS:ME:LINE1>	.	PASS	SVTYPE=INS:ME:LINE1;END=109084607;SVLEN=1341;AN=20618;AC=3;AF=0.00014550392860607236;N_HET=3;N_HOMALT=0;N_HOMREF=10306;FREQ_HET=0.0002910078572121447;FREQ_HOMALT=0.0;FREQ_HOMREF=0.9997089921427879;N_BI_GENOS=10309;North_Han_AN=2272;North_Han_AC=1;North_Ha
chr5	109285365	.	A	<INS:ME:LINE1>	.	PASS	SVTYPE=INS:ME:LINE1;END=109285374;SVLEN=2855;AN=20618;AC=1;AF=4.850130953535746e-05;N_HET=1;N_HOMALT=0;N_HOMREF=10308;FREQ_HET=9.700261907071492e-05;FREQ_HOMALT=0.0;FREQ_HOMREF=0.9999029973809292;N_BI_GENOS=10309;East_Han_AN=6554;East_Han_AC=1;East_Han_AF
chr5	109469083	.	A	<INS:ME:LINE1>	.	PASS	SVTYPE=INS:ME:LINE1;END=109469083;SVLEN=0;AN=20618;AC=6;AF=0.0002910078572121447;N_HET=4;N_HOMALT=1;N_HOMREF=10304;FREQ_HET=0.00038801047628285966;FREQ_HOMALT=9.700261907071492e-05;FREQ_HOMREF=0.9995149869046465;N_BI_GENOS=10309;North_Han_AN=2272;North_Ha

chr5	109507513	.	T	<INS:ME:LINE1>	.	PASS	SVTYPE=INS:ME:LINE1;END=109507518;SVLEN=722;AN=20618;AC=1;AF=4.850130953535746e-05;N_HET=1;N_HOMALT=0;N_HOMREF=10308;FREQ_HET=9.700261907071492e-05;FREQ_HOMALT=0.0;FREQ_HOMREF=0.9999029973809292;N_BI_GENOS=10309;Southeast_Han_AN=1908;Southeast_Han_AC=1;So
chr5	109662214	.	A	<INS:ME:LINE1>	.	PASS	SVTYPE=INS:ME:LINE1;END=109662225;SVLEN=6005;AN=20618;AC=8;AF=0.00038801047628285966;N_HET=8;N_HOMALT=0;N_HOMREF=10301;FREQ_HET=0.0007760209525657193;FREQ_HOMALT=0.0;FREQ_HOMREF=0.9992239790474343;N_BI_GENOS=10309;Northwest_Han_AN=1296;Northwest_Han_AC=3;
chr5	109758219	.	T	<INS:ME:LINE1>	.	PASS	SVTYPE=INS:ME:LINE1;END=109758219;SVLEN=898;AN=20618;AC=1;AF=4.850130953535746e-05;N_HET=1;N_HOMALT=0;N_HOMREF=10308;FREQ_HET=9.700261907071492e-05;FREQ_HOMALT=0.0;FREQ_HOMREF=0.9999029973809292;N_BI_GENOS=10309;East_Han_AN=6554;East_Han_AC=1;East_Han_AF=
chr5	110131394	.	A	<INS:ME:LINE1>	.	PASS	SVTYPE=INS:ME:LINE1;END=110131408;SVLEN=437;AN=20618;AC=2;AF=9.700261907071492e-05;N_HET=2;N_HOMALT=0;N_HOMREF=10307;FREQ_HET=0.00019400523814142983;FREQ_HOMALT=0.0;FREQ_HOMREF=0.9998059947618586;N_BI_GENOS=10309;East_Han_AN=6554;East_Han_AC=2;East_Han_AF
chr5	110132002	.	C	<INS:ME:LINE1>	.	PASS	SVTYPE=INS:ME:LINE1;END=110132028;SVLEN=1094;AN=20618;AC=1;AF=4.850130953535746e-05;N_HET=1;N_HOMALT=0;N_HOMREF=10308;FREQ_HET=9.700261907071492e-05;FREQ_HOMALT=0.0;FREQ_HOMREF=0.9999029973809292;N_BI_GENOS=10309;North_Han_AN=2272;North_Han_AC=1;North_Han
chr5	110132436	.	T	<INS:ME:LINE1>	.	PASS	SVTYPE=INS:ME:LINE1;END=110132436;SVLEN=237;AN=20618;AC=1;AF=4.850130953535746e-05;N_HET=1;N_HOMALT=0;N_HOMREF=10308;FREQ_HET=9.700261907071492e-05;FREQ_HOMALT=0.0;FREQ_HOMREF=0.9999029973809292;N_BI_GENOS=10309;Lingnan_Han_AN=1888;Lingnan_Han_AC=1;Lingna
chr5	110144556	.	G	<INS:ME:LINE1>	.	PASS	SVTYPE=INS:ME:LINE1;END=110144568;SVLEN=5998;AN=20618;AC=12592;AF=0.6107284896692211;N_HET=900;N_HOMALT=5846;N_HOMREF=3563;FREQ_HET=0.08730235716364342;FREQ_HOMALT=0.5670773110873993;FREQ_HOMREF=0.34562033174895723;N_BI_GENOS=10309;South_Han_AN=1496;South
chr5	110231823	.	A	<INS:ME:LINE1>	.	PASS	SVTYPE=INS:ME:LINE1;END=110231841;SVLEN=941;AN=20618;AC=4987;AF=0.24187603065282762;N_HET=3255;N_HOMALT=866;N_HOMREF=6188;FREQ_HET=0.31574352507517706;FREQ_HOMALT=0.08400426811523912;FREQ_HOMREF=0.6002522068095839;N_BI_GENOS=10309;South_Han_AN=1496;South_
chr5	110299499	.	A	<INS:ME:LINE1>	.	PASS	SVTYPE=INS:ME:LINE1;END=110299510;SVLEN=931;AN=20618;AC=1;AF=4.850130953535746e-05;N_HET=1;N_HOMALT=0;N_HOMREF=10308;FREQ_HET=9.700261907071492e-05;FREQ_HOMALT=0.0;FREQ_HOMREF=0.9999029973809292;N_BI_GENOS=10309;East_Han_AN=6554;East_Han_AC=1;East_Han_AF=
chr5	110492144	.	T	<INS:ME:LINE1>	.	PASS	SVTYPE=INS:ME:LINE1;END=110492164;SVLEN=12;AN=20618;AC=1;AF=4.850130953535746e-05;N_HET=1;N_HOMALT=0;N_HOMREF=10308;FREQ_HET=9.700261907071492e-05;FREQ_HOMALT=0.0;FREQ_HOMREF=0.9999029973809292;N_BI_GENOS=10309;East_Han_AN=6554;East_Han_AC=1;East_Han_AF=0
chr5	111345184	.	T	<INS:ME:LINE1>	.	PASS	SVTYPE=INS:ME:LINE1;END=111345199;SVLEN=4758;AN=20618;AC=3;AF=0.00014550392860607236;N_HET=3;N_HOMALT=0;N_HOMREF=10306;FREQ_HET=0.0002910078572121447;FREQ_HOMALT=0.0;FREQ_HOMREF=0.9997089921427879;N_BI_GENOS=10309;Tibetan_AN=414;Tibetan_AC=3;Tibetan_AF=0.
chr5	111555341	.	G	<INS:ME:LINE1>	.	PASS	SVTYPE=INS:ME:LINE1;END=111555341;SVLEN=46;AN=20618;AC=1;AF=4.850130953535746e-05;N_HET=1;N_HOMALT=0;N_HOMREF=10308;FREQ_HET=9.700261907071492e-05;FREQ_HOMALT=0.0;FREQ_HOMREF=0.9999029973809292;N_BI_GENOS=10309;East_Han_AN=6554;East_Han_AC=1;East_Han_AF=0
chr5	111902064	.	G	<INS:ME:LINE1>	.	PASS	SVTYPE=INS:ME:LINE1;END=111902064;SVLEN=339;AN=20618;AC=1;AF=4.850130953535746e-05;N_HET=1;N_HOMALT=0;N_HOMREF=10308;FREQ_HET=9.700261907071492e-05;FREQ_HOMALT=0.0;FREQ_HOMREF=0.9999029973809292;N_BI_GENOS=10309;North_Han_AN=2272;North_Han_AC=1;North_Han_
chr5	112068758	.	G	<INS:ME:LINE1>	.	PASS	SVTYPE=INS:ME:LINE1;END=112068776;SVLEN=1441;AN=20618;AC=1;AF=4.850130953535746e-05;N_HET=1;N_HOMALT=0;N_HOMREF=10308;FREQ_HET=9.700261907071492e-05;FREQ_HOMALT=0.0;FREQ_HOMREF=0.9999029973809292;N_BI_GENOS=10309;North_Han_AN=2272;North_Han_AC=1;North_Han
chr5	112105781	.	C	<INS:ME:LINE1>	.	PASS	SVTYPE=INS:ME:LINE1;END=112105797;SVLEN=6012;AN=20618;AC=1;AF=4.850130953535746e-05;N_HET=1;N_HOMALT=0;N_HOMREF=10308;FREQ_HET=9.700261907071492e-05;FREQ_HOMALT=0.0;FREQ_HOMREF=0.9999029973809292;N_BI_GENOS=10309;North_Han_AN=2272;North_Han_AC=1;North_Han
chr5	112195732	.	A	<INS:ME:LINE1>	.	PASS	SVTYPE=INS:ME:LINE1;END=112195747;SVLEN=6026;AN=20618;AC=1;AF=4.850130953535746e-05;N_HET=1;N_HOMALT=0;N_HOMREF=10308;FREQ_HET=9.700261907071492e-05;FREQ_HOMALT=0.0;FREQ_HOMREF=0.9999029973809292;N_BI_GENOS=10309;East_Han_AN=6554;East_Han_AC=1;East_Han_AF
chr5	112241948	.	A	<INS:ME:LINE1>	.	PASS	SVTYPE=INS:ME:LINE1;END=112241948;SVLEN=3509;AN=20618;AC=1;AF=4.850130953535746e-05;N_HET=1;N_HOMALT=0;N_HOMREF=10308;FREQ_HET=9.700261907071492e-05;FREQ_HOMALT=0.0;FREQ_HOMREF=0.9999029973809292;N_BI_GENOS=10309;Hui_AN=444;Hui_AC=1;Hui_AF=0.0022522522522
chr5	112845590	.	A	<INS:ME:LINE1>	.	PASS	SVTYPE=INS:ME:LINE1;END=112845601;SVLEN=5998;AN=20618;AC=54;AF=0.0026190707149093026;N_HET=52;N_HOMALT=1;N_HOMREF=10256;FREQ_HET=0.005044136191677175;FREQ_HOMALT=9.700261907071492e-05;FREQ_HOMREF=0.9948588611892522;N_BI_GENOS=10309;South_Han_AN=1496;South
chr5	112933190	.	A	<INS:ME:LINE1>	.	PASS	SVTYPE=INS:ME:LINE1;END=112933190;SVLEN=0;AN=20618;AC=48;AF=0.0023280628576971578;N_HET=48;N_HOMALT=0;N_HOMREF=10261;FREQ_HET=0.0046561257153943155;FREQ_HOMALT=0.0;FREQ_HOMREF=0.9953438742846057;N_BI_GENOS=10309;South_Han_AN=1496;South_Han_AC=6;South_Han_
chr5	113367384	.	T	<INS:ME:LINE1>	.	PASS	SVTYPE=INS:ME:LINE1;END=113367384;SVLEN=5998;AN=20618;AC=1289;AF=0.06251818799107577;N_HET=1039;N_HOMALT=125;N_HOMREF=9145;FREQ_HET=0.1007857212144728;FREQ_HOMALT=0.012125327383839363;FREQ_HOMREF=0.8870889514016879;N_BI_GENOS=10309;South_Han_AN=1496;South
chr5	113398367	.	A	<INS:ME:LINE1>	.	PASS	SVTYPE=INS:ME:LINE1;END=113398383;SVLEN=6008;AN=20618;AC=4;AF=0.00019400523814142983;N_HET=4;N_HOMALT=0;N_HOMREF=10305;FREQ_HET=0.00038801047628285966;FREQ_HOMALT=0.0;FREQ_HOMREF=0.9996119895237171;N_BI_GENOS=10309;Northwest_Han_AN=1296;Northwest_Han_AC=2
chr5	113446954	.	T	<INS:ME:LINE1>	.	PASS	SVTYPE=INS:ME:LINE1;END=113446969;SVLEN=6018;AN=20618;AC=2;AF=9.700261907071492e-05;N_HET=0;N_HOMALT=1;N_HOMREF=10308;FREQ_HET=0.0;FREQ_HOMALT=9.700261907071492e-05;FREQ_HOMREF=0.9999029973809292;N_BI_GENOS=10309;Southeast_Han_AN=1908;Southeast_Han_AC=2;S
chr5	113507870	.	C	<INS:ME:LINE1>	.	PASS	SVTYPE=INS:ME:LINE1;END=113507885;SVLEN=5986;AN=20618;AC=3;AF=0.00014550392860607236;N_HET=3;N_HOMALT=0;N_HOMREF=10306;FREQ_HET=0.0002910078572121447;FREQ_HOMALT=0.0;FREQ_HOMREF=0.9997089921427879;N_BI_GENOS=10309;East_Han_AN=6554;East_Han_AC=3;East_Han_A
chr5	113703557	.	G	<INS:ME:LINE1>	.	PASS	SVTYPE=INS:ME:LINE1;END=113703557;SVLEN=1520;AN=20618;AC=1;AF=4.850130953535746e-05;N_HET=1;N_HOMALT=0;N_HOMREF=10308;FREQ_HET=9.700261907071492e-05;FREQ_HOMALT=0.0;FREQ_HOMREF=0.9999029973809292;N_BI_GENOS=10309;East_Han_AN=6554;East_Han_AC=1;East_Han_AF
chr5	113750206	.	A	<INS:ME:LINE1>	.	PASS	SVTYPE=INS:ME:LINE1;END=113750221;SVLEN=5997;AN=20618;AC=1;AF=4.850130953535746e-05;N_HET=1;N_HOMALT=0;N_HOMREF=10308;FREQ_HET=9.700261907071492e-05;FREQ_HOMALT=0.0;FREQ_HOMREF=0.9999029973809292;N_BI_GENOS=10309;South_Han_AN=1496;South_Han_AC=1;South_Han
chr5	113833250	.	T	<INS:ME:LINE1>	.	PASS	SVTYPE=INS:ME:LINE1;END=113833258;SVLEN=305;AN=20618;AC=1;AF=4.850130953535746e-05;N_HET=1;N_HOMALT=0;N_HOMREF=10308;FREQ_HET=9.700261907071492e-05;FREQ_HOMALT=0.0;FREQ_HOMREF=0.9999029973809292;N_BI_GENOS=10309;East_Han_AN=6554;East_Han_AC=1;East_Han_AF=
chr5	114114855	.	A	<INS:ME:LINE1>	.	PASS	SVTYPE=INS:ME:LINE1;END=114114861;SVLEN=1718;AN=20618;AC=2;AF=9.700261907071492e-05;N_HET=2;N_HOMALT=0;N_HOMREF=10307;FREQ_HET=0.00019400523814142983;FREQ_HOMALT=0.0;FREQ_HOMREF=0.9998059947618586;N_BI_GENOS=10309;North_Han_AN=2272;North_Han_AC=1;North_Ha
chr5	114215996	.	A	<INS:ME:LINE1>	.	PASS	SVTYPE=INS:ME:LINE1;END=114216012;SVLEN=837;AN=20618;AC=6;AF=0.0002910078572121447;N_HET=6;N_HOMALT=0;N_HOMREF=10303;FREQ_HET=0.0005820157144242894;FREQ_HOMALT=0.0;FREQ_HOMREF=0.9994179842855757;N_BI_GENOS=10309;North_Han_AN=2272;North_Han_AC=3;North_Han_
chr5	114257076	.	A	<INS:ME:LINE1>	.	PASS	SVTYPE=INS:ME:LINE1;END=114257076;SVLEN=1277;AN=20618;AC=1;AF=4.850130953535746e-05;N_HET=1;N_HOMALT=0;N_HOMREF=10308;FREQ_HET=9.700261907071492e-05;FREQ_HOMALT=0.0;FREQ_HOMREF=0.9999029973809292;N_BI_GENOS=10309;East_Han_AN=6554;East_Han_AC=1;East_Han_AF
chr5	114257358	.	T	<INS:ME:LINE1>	.	PASS	SVTYPE=INS:ME:LINE1;END=114257358;SVLEN=84;AN=20618;AC=1;AF=4.850130953535746e-05;N_HET=1;N_HOMALT=0;N_HOMREF=10308;FREQ_HET=9.700261907071492e-05;FREQ_HOMALT=0.0;FREQ_HOMREF=0.9999029973809292;N_BI_GENOS=10309;East_Han_AN=6554;East_Han_AC=1;East_Han_AF=0
chr5	114355639	.	G	<INS:ME:LINE1>	.	PASS	SVTYPE=INS:ME:LINE1;END=114355639;SVLEN=449;AN=20618;AC=1;AF=4.850130953535746e-05;N_HET=1;N_HOMALT=0;N_HOMREF=10308;FREQ_HET=9.700261907071492e-05;FREQ_HOMALT=0.0;FREQ_HOMREF=0.9999029973809292;N_BI_GENOS=10309;East_Han_AN=6554;East_Han_AC=1;East_Han_AF=
chr5	114589358	.	A	<INS:ME:LINE1>	.	PASS	SVTYPE=INS:ME:LINE1;END=114589373;SVLEN=1042;AN=20618;AC=1;AF=4.850130953535746e-05;N_HET=1;N_HOMALT=0;N_HOMREF=10308;FREQ_HET=9.700261907071492e-05;FREQ_HOMALT=0.0;FREQ_HOMREF=0.9999029973809292;N_BI_GENOS=10309;Central_Han_AN=2128;Central_Han_AC=1;Centr
chr5	114634414	.	G	<INS:ME:LINE1>	.	PASS	SVTYPE=INS:ME:LINE1;END=114634414;SVLEN=465;AN=20618;AC=1;AF=4.850130953535746e-05;N_HET=1;N_HOMALT=0;N_HOMREF=10308;FREQ_HET=9.700261907071492e-05;FREQ_HOMALT=0.0;FREQ_HOMREF=0.9999029973809292;N_BI_GENOS=10309;East_Han_AN=6554;East_Han_AC=1;East_Han_AF=
chr5	114678613	.	G	<INS:ME:LINE1>	.	PASS	SVTYPE=INS:ME:LINE1;END=114678628;SVLEN=1876;AN=20618;AC=1;AF=4.850130953535746e-05;N_HET=1;N_HOMALT=0;N_HOMREF=10308;FREQ_HET=9.700261907071492e-05;FREQ_HOMALT=0.0;FREQ_HOMREF=0.9999029973809292;N_BI_GENOS=10309;East_Han_AN=6554;East_Han_AC=1;East_Han_AF
chr5	114680627	.	T	<INS:ME:LINE1>	.	PASS	SVTYPE=INS:ME:LINE1;END=114680627;SVLEN=6000;AN=20618;AC=1;AF=4.850130953535746e-05;N_HET=1;N_HOMALT=0;N_HOMREF=10308;FREQ_HET=9.700261907071492e-05;FREQ_HOMALT=0.0;FREQ_HOMREF=0.9999029973809292;N_BI_GENOS=10309;Northwest_Han_AN=1296;Northwest_Han_AC=1;N
chr5	114750441	.	G	<INS:ME:LINE1>	.	PASS	SVTYPE=INS:ME:LINE1;END=114750458;SVLEN=1041;AN=20618;AC=2;AF=9.700261907071492e-05;N_HET=2;N_HOMALT=0;N_HOMREF=10307;FREQ_HET=0.00019400523814142983;FREQ_HOMALT=0.0;FREQ_HOMREF=0.9998059947618586;N_BI_GENOS=10309;Lingnan_Han_AN=1888;Lingnan_Han_AC=1;Ling
chr5	114834458	.	T	<INS:ME:LINE1>	.	PASS	SVTYPE=INS:ME:LINE1;END=114834458;SVLEN=827;AN=20618;AC=1;AF=4.850130953535746e-05;N_HET=1;N_HOMALT=0;N_HOMREF=10308;FREQ_HET=9.700261907071492e-05;FREQ_HOMALT=0.0;FREQ_HOMREF=0.9999029973809292;N_BI_GENOS=10309;East_Han_AN=6554;East_Han_AC=1;East_Han_AF=
chr5	114834545	.	A	<INS:ME:LINE1>	.	PASS	SVTYPE=INS:ME:LINE1;END=114834569;SVLEN=1150;AN=20618;AC=1;AF=4.850130953535746e-05;N_HET=1;N_HOMALT=0;N_HOMREF=10308;FREQ_HET=9.700261907071492e-05;FREQ_HOMALT=0.0;FREQ_HOMREF=0.9999029973809292;N_BI_GENOS=10309;East_Han_AN=6554;East_Han_AC=1;East_Han_AF
chr5	115146932	.	T	<INS:ME:LINE1>	.	PASS	SVTYPE=INS:ME:LINE1;END=115146948;SVLEN=5430;AN=20618;AC=5;AF=0.00024250654767678728;N_HET=5;N_HOMALT=0;N_HOMREF=10304;FREQ_HET=0.00048501309535357455;FREQ_HOMALT=0.0;FREQ_HOMREF=0.9995149869046465;N_BI_GENOS=10309;East_Han_AN=6554;East_Han_AC=3;East_Han_
chr5	115842046	.	G	<INS:ME:LINE1>	.	PASS	SVTYPE=INS:ME:LINE1;END=115842061;SVLEN=1179;AN=20618;AC=1;AF=4.850130953535746e-05;N_HET=1;N_HOMALT=0;N_HOMREF=10308;FREQ_HET=9.700261907071492e-05;FREQ_HOMALT=0.0;FREQ_HOMREF=0.9999029973809292;N_BI_GENOS=10309;Lingnan_Han_AN=1888;Lingnan_Han_AC=1;Lingn
chr5	116113365	.	G	<INS:ME:LINE1>	.	PASS	SVTYPE=INS:ME:LINE1;END=116113369;SVLEN=5995;AN=20618;AC=185;AF=0.00897274226404113;N_HET=167;N_HOMALT=9;N_HOMREF=10133;FREQ_HET=0.01619943738480939;FREQ_HOMALT=0.0008730235716364342;FREQ_HOMREF=0.9829275390435541;N_BI_GENOS=10309;South_Han_AN=1496;South_
chr5	117300266	.	T	<INS:ME:LINE1>	.	PASS	SVTYPE=INS:ME:LINE1;END=117300282;SVLEN=5993;AN=20618;AC=1;AF=4.850130953535746e-05;N_HET=1;N_HOMALT=0;N_HOMREF=10308;FREQ_HET=9.700261907071492e-05;FREQ_HOMALT=0.0;FREQ_HOMREF=0.9999029973809292;N_BI_GENOS=10309;Northwest_Han_AN=1296;Northwest_Han_AC=1;N
chr5	117389075	.	A	<INS:ME:LINE1>	.	PASS	SVTYPE=INS:ME:LINE1;END=117389090;SVLEN=6020;AN=20618;AC=1;AF=4.850130953535746e-05;N_HET=1;N_HOMALT=0;N_HOMREF=10308;FREQ_HET=9.700261907071492e-05;FREQ_HOMALT=0.0;FREQ_HOMREF=0.9999029973809292;N_BI_GENOS=10309;East_Han_AN=6554;East_Han_AC=1;East_Han_AF
chr5	117422873	.	T	<INS:ME:LINE1>	.	PASS	SVTYPE=INS:ME:LINE1;END=117422890;SVLEN=6007;AN=20618;AC=7;AF=0.0003395091667475022;N_HET=7;N_HOMALT=0;N_HOMREF=10302;FREQ_HET=0.0006790183334950044;FREQ_HOMALT=0.0;FREQ_HOMREF=0.999320981666505;N_BI_GENOS=10309;Lingnan_Han_AN=1888;Lingnan_Han_AC=1;Lingna
chr5	117445250	.	C	<INS:ME:LINE1>	.	PASS	SVTYPE=INS:ME:LINE1;END=117445264;SVLEN=292;AN=20618;AC=1;AF=4.850130953535746e-05;N_HET=1;N_HOMALT=0;N_HOMREF=10308;FREQ_HET=9.700261907071492e-05;FREQ_HOMALT=0.0;FREQ_HOMREF=0.9999029973809292;N_BI_GENOS=10309;East_Han_AN=6554;East_Han_AC=1;East_Han_AF=
chr5	117595699	.	T	<INS:ME:LINE1>	.	PASS	SVTYPE=INS:ME:LINE1;END=117595706;SVLEN=489;AN=20618;AC=1;AF=4.850130953535746e-05;N_HET=1;N_HOMALT=0;N_HOMREF=10308;FREQ_HET=9.700261907071492e-05;FREQ_HOMALT=0.0;FREQ_HOMREF=0.9999029973809292;N_BI_GENOS=10309;Southeast_Han_AN=1908;Southeast_Han_AC=1;So
chr5	117761348	.	A	<INS:ME:LINE1>	.	PASS	SVTYPE=INS:ME:LINE1;END=117761348;SVLEN=819;AN=20618;AC=1;AF=4.850130953535746e-05;N_HET=1;N_HOMALT=0;N_HOMREF=10308;FREQ_HET=9.700261907071492e-05;FREQ_HOMALT=0.0;FREQ_HOMREF=0.9999029973809292;N_BI_GENOS=10309;Lingnan_Han_AN=1888;Lingnan_Han_AC=1;Lingna
chr5	117775303	.	T	<INS:ME:LINE1>	.	PASS	SVTYPE=INS:ME:LINE1;END=117775303;SVLEN=255;AN=20618;AC=1;AF=4.850130953535746e-05;N_HET=1;N_HOMALT=0;N_HOMREF=10308;FREQ_HET=9.700261907071492e-05;FREQ_HOMALT=0.0;FREQ_HOMREF=0.9999029973809292;N_BI_GENOS=10309;East_Han_AN=6554;East_Han_AC=1;East_Han_AF=
chr5	117787215	.	T	<INS:ME:LINE1>	.	PASS	SVTYPE=INS:ME:LINE1;END=117787229;SVLEN=620;AN=20618;AC=1;AF=4.850130953535746e-05;N_HET=1;N_HOMALT=0;N_HOMREF=10308;FREQ_HET=9.700261907071492e-05;FREQ_HOMALT=0.0;FREQ_HOMREF=0.9999029973809292;N_BI_GENOS=10309;Southeast_Han_AN=1908;Southeast_Han_AC=1;So
chr5	117811725	.	T	<INS:ME:LINE1>	.	PASS	SVTYPE=INS:ME:LINE1;END=117811739;SVLEN=3250;AN=20618;AC=3;AF=0.00014550392860607236;N_HET=3;N_HOMALT=0;N_HOMREF=10306;FREQ_HET=0.0002910078572121447;FREQ_HOMALT=0.0;FREQ_HOMREF=0.9997089921427879;N_BI_GENOS=10309;North_Han_AN=2272;North_Han_AC=1;North_Ha
chr5	117862461	.	T	<INS:ME:LINE1>	.	PASS	SVTYPE=INS:ME:LINE1;END=117862475;SVLEN=6001;AN=20618;AC=2;AF=9.700261907071492e-05;N_HET=2;N_HOMALT=0;N_HOMREF=10307;FREQ_HET=0.00019400523814142983;FREQ_HOMALT=0.0;FREQ_HOMREF=0.9998059947618586;N_BI_GENOS=10309;Northwest_Han_AN=1296;Northwest_Han_AC=1;
chr5	117920921	.	T	<INS:ME:LINE1>	.	PASS	SVTYPE=INS:ME:LINE1;END=117920971;SVLEN=861;AN=20618;AC=1;AF=4.850130953535746e-05;N_HET=1;N_HOMALT=0;N_HOMREF=10308;FREQ_HET=9.700261907071492e-05;FREQ_HOMALT=0.0;FREQ_HOMREF=0.9999029973809292;N_BI_GENOS=10309;East_Han_AN=6554;East_Han_AC=1;East_Han_AF=
chr5	117951577	.	T	<INS:ME:LINE1>	.	PASS	SVTYPE=INS:ME:LINE1;END=117951594;SVLEN=599;AN=20618;AC=2;AF=9.700261907071492e-05;N_HET=2;N_HOMALT=0;N_HOMREF=10307;FREQ_HET=0.00019400523814142983;FREQ_HOMALT=0.0;FREQ_HOMREF=0.9998059947618586;N_BI_GENOS=10309;Lingnan_Han_AN=1888;Lingnan_Han_AC=1;Lingn
chr5	118062368	.	T	<INS:ME:LINE1>	.	PASS	SVTYPE=INS:ME:LINE1;END=118062368;SVLEN=123;AN=20618;AC=1;AF=4.850130953535746e-05;N_HET=1;N_HOMALT=0;N_HOMREF=10308;FREQ_HET=9.700261907071492e-05;FREQ_HOMALT=0.0;FREQ_HOMREF=0.9999029973809292;N_BI_GENOS=10309;East_Han_AN=6554;East_Han_AC=1;East_Han_AF=
chr5	118171663	.	A	<INS:ME:LINE1>	.	PASS	SVTYPE=INS:ME:LINE1;END=118171677;SVLEN=5991;AN=20618;AC=1;AF=4.850130953535746e-05;N_HET=1;N_HOMALT=0;N_HOMREF=10308;FREQ_HET=9.700261907071492e-05;FREQ_HOMALT=0.0;FREQ_HOMREF=0.9999029973809292;N_BI_GENOS=10309;North_Han_AN=2272;North_Han_AC=1;North_Han
chr5	118176887	.	A	<INS:ME:LINE1>	.	PASS	SVTYPE=INS:ME:LINE1;END=118176889;SVLEN=2632;AN=20618;AC=1;AF=4.850130953535746e-05;N_HET=1;N_HOMALT=0;N_HOMREF=10308;FREQ_HET=9.700261907071492e-05;FREQ_HOMALT=0.0;FREQ_HOMREF=0.9999029973809292;N_BI_GENOS=10309;Central_Han_AN=2128;Central_Han_AC=1;Centr
chr5	118232267	.	T	<INS:ME:LINE1>	.	PASS	SVTYPE=INS:ME:LINE1;END=118232287;SVLEN=739;AN=20618;AC=1;AF=4.850130953535746e-05;N_HET=1;N_HOMALT=0;N_HOMREF=10308;FREQ_HET=9.700261907071492e-05;FREQ_HOMALT=0.0;FREQ_HOMREF=0.9999029973809292;N_BI_GENOS=10309;South_Han_AN=1496;South_Han_AC=1;South_Han_
chr5	118345940	.	G	<INS:ME:LINE1>	.	PASS	SVTYPE=INS:ME:LINE1;END=118345955;SVLEN=1911;AN=20618;AC=2;AF=9.700261907071492e-05;N_HET=2;N_HOMALT=0;N_HOMREF=10307;FREQ_HET=0.00019400523814142983;FREQ_HOMALT=0.0;FREQ_HOMREF=0.9998059947618586;N_BI_GENOS=10309;North_Han_AN=2272;North_Han_AC=1;North_Ha
chr5	118454465	.	T	<INS:ME:LINE1>	.	PASS	SVTYPE=INS:ME:LINE1;END=118454465;SVLEN=193;AN=20618;AC=2;AF=9.700261907071492e-05;N_HET=2;N_HOMALT=0;N_HOMREF=10307;FREQ_HET=0.00019400523814142983;FREQ_HOMALT=0.0;FREQ_HOMREF=0.9998059947618586;N_BI_GENOS=10309;East_Han_AN=6554;East_Han_AC=2;East_Han_AF
chr5	118670873	.	C	<INS:ME:LINE1>	.	PASS	SVTYPE=INS:ME:LINE1;END=118670877;SVLEN=2209;AN=20618;AC=1;AF=4.850130953535746e-05;N_HET=1;N_HOMALT=0;N_HOMREF=10308;FREQ_HET=9.700261907071492e-05;FREQ_HOMALT=0.0;FREQ_HOMREF=0.9999029973809292;N_BI_GENOS=10309;East_Han_AN=6554;East_Han_AC=1;East_Han_AF
chr5	118914383	.	A	<INS:ME:LINE1>	.	PASS	SVTYPE=INS:ME:LINE1;END=118914393;SVLEN=1850;AN=20618;AC=1;AF=4.850130953535746e-05;N_HET=1;N_HOMALT=0;N_HOMREF=10308;FREQ_HET=9.700261907071492e-05;FREQ_HOMALT=0.0;FREQ_HOMREF=0.9999029973809292;N_BI_GENOS=10309;Tibetan_AN=414;Tibetan_AC=1;Tibetan_AF=0.0
chr5	119328372	.	T	<INS:ME:LINE1>	.	PASS	SVTYPE=INS:ME:LINE1;END=119328372;SVLEN=0;AN=20618;AC=1;AF=4.850130953535746e-05;N_HET=1;N_HOMALT=0;N_HOMREF=10308;FREQ_HET=9.700261907071492e-05;FREQ_HOMALT=0.0;FREQ_HOMREF=0.9999029973809292;N_BI_GENOS=10309;Southeast_Han_AN=1908;Southeast_Han_AC=1;Sout
chr5	119341138	.	G	<INS:ME:LINE1>	.	PASS	SVTYPE=INS:ME:LINE1;END=119341153;SVLEN=6005;AN=20618;AC=1;AF=4.850130953535746e-05;N_HET=1;N_HOMALT=0;N_HOMREF=10308;FREQ_HET=9.700261907071492e-05;FREQ_HOMALT=0.0;FREQ_HOMREF=0.9999029973809292;N_BI_GENOS=10309;North_Han_AN=2272;North_Han_AC=1;North_Han
chr5	120101338	.	T	<INS:ME:LINE1>	.	PASS	SVTYPE=INS:ME:LINE1;END=120101338;SVLEN=1589;AN=20618;AC=1;AF=4.850130953535746e-05;N_HET=1;N_HOMALT=0;N_HOMREF=10308;FREQ_HET=9.700261907071492e-05;FREQ_HOMALT=0.0;FREQ_HOMREF=0.9999029973809292;N_BI_GENOS=10309;East_Han_AN=6554;East_Han_AC=1;East_Han_AF
chr5	120122276	.	A	<INS:ME:LINE1>	.	PASS	SVTYPE=INS:ME:LINE1;END=120122286;SVLEN=765;AN=20618;AC=1;AF=4.850130953535746e-05;N_HET=1;N_HOMALT=0;N_HOMREF=10308;FREQ_HET=9.700261907071492e-05;FREQ_HOMALT=0.0;FREQ_HOMREF=0.9999029973809292;N_BI_GENOS=10309;Southeast_Han_AN=1908;Southeast_Han_AC=1;So
chr5	120184693	.	G	<INS:ME:LINE1>	.	PASS	SVTYPE=INS:ME:LINE1;END=120184708;SVLEN=3120;AN=20618;AC=1;AF=4.850130953535746e-05;N_HET=1;N_HOMALT=0;N_HOMREF=10308;FREQ_HET=9.700261907071492e-05;FREQ_HOMALT=0.0;FREQ_HOMREF=0.9999029973809292;N_BI_GENOS=10309;South_Han_AN=1496;South_Han_AC=1;South_Han
chr5	120230043	.	A	<INS:ME:LINE1>	.	PASS	SVTYPE=INS:ME:LINE1;END=120230059;SVLEN=870;AN=20618;AC=1;AF=4.850130953535746e-05;N_HET=1;N_HOMALT=0;N_HOMREF=10308;FREQ_HET=9.700261907071492e-05;FREQ_HOMALT=0.0;FREQ_HOMREF=0.9999029973809292;N_BI_GENOS=10309;Tibetan_AN=414;Tibetan_AC=1;Tibetan_AF=0.00
chr5	120516584	.	A	<INS:ME:LINE1>	.	PASS	SVTYPE=INS:ME:LINE1;END=120516601;SVLEN=6001;AN=20618;AC=4;AF=0.00019400523814142983;N_HET=4;N_HOMALT=0;N_HOMREF=10305;FREQ_HET=0.00038801047628285966;FREQ_HOMALT=0.0;FREQ_HOMREF=0.9996119895237171;N_BI_GENOS=10309;Northwest_Han_AN=1296;Northwest_Han_AC=1
chr5	120566780	.	A	<INS:ME:LINE1>	.	PASS	SVTYPE=INS:ME:LINE1;END=120566791;SVLEN=5994;AN=20618;AC=2;AF=9.700261907071492e-05;N_HET=2;N_HOMALT=0;N_HOMREF=10307;FREQ_HET=0.00019400523814142983;FREQ_HOMALT=0.0;FREQ_HOMREF=0.9998059947618586;N_BI_GENOS=10309;Lingnan_Han_AN=1888;Lingnan_Han_AC=1;Ling
chr5	120601512	.	G	<INS:ME:LINE1>	.	PASS	SVTYPE=INS:ME:LINE1;END=120601512;SVLEN=1072;AN=20618;AC=1;AF=4.850130953535746e-05;N_HET=1;N_HOMALT=0;N_HOMREF=10308;FREQ_HET=9.700261907071492e-05;FREQ_HOMALT=0.0;FREQ_HOMREF=0.9999029973809292;N_BI_GENOS=10309;East_Han_AN=6554;East_Han_AC=1;East_Han_AF
chr5	120637428	.	A	<INS:ME:LINE1>	.	PASS	SVTYPE=INS:ME:LINE1;END=120637442;SVLEN=6008;AN=20618;AC=1;AF=4.850130953535746e-05;N_HET=1;N_HOMALT=0;N_HOMREF=10308;FREQ_HET=9.700261907071492e-05;FREQ_HOMALT=0.0;FREQ_HOMREF=0.9999029973809292;N_BI_GENOS=10309;Northwest_Han_AN=1296;Northwest_Han_AC=1;N
chr5	120967914	.	A	<INS:ME:LINE1>	.	PASS	SVTYPE=INS:ME:LINE1;END=120967934;SVLEN=5997;AN=20618;AC=1;AF=4.850130953535746e-05;N_HET=1;N_HOMALT=0;N_HOMREF=10308;FREQ_HET=9.700261907071492e-05;FREQ_HOMALT=0.0;FREQ_HOMREF=0.9999029973809292;N_BI_GENOS=10309;Lingnan_Han_AN=1888;Lingnan_Han_AC=1;Lingn
chr5	121007773	.	A	<INS:ME:LINE1>	.	PASS	SVTYPE=INS:ME:LINE1;END=121007792;SVLEN=407;AN=20618;AC=1;AF=4.850130953535746e-05;N_HET=1;N_HOMALT=0;N_HOMREF=10308;FREQ_HET=9.700261907071492e-05;FREQ_HOMALT=0.0;FREQ_HOMREF=0.9999029973809292;N_BI_GENOS=10309;Southeast_Han_AN=1908;Southeast_Han_AC=1;So
chr5	121053589	.	T	<INS:ME:LINE1>	.	PASS	SVTYPE=INS:ME:LINE1;END=121053619;SVLEN=339;AN=20618;AC=1;AF=4.850130953535746e-05;N_HET=1;N_HOMALT=0;N_HOMREF=10308;FREQ_HET=9.700261907071492e-05;FREQ_HOMALT=0.0;FREQ_HOMREF=0.9999029973809292;N_BI_GENOS=10309;Northwest_Han_AN=1296;Northwest_Han_AC=1;No
chr5	121056355	.	A	<INS:ME:LINE1>	.	PASS	SVTYPE=INS:ME:LINE1;END=121056366;SVLEN=3554;AN=20618;AC=3;AF=0.00014550392860607236;N_HET=1;N_HOMALT=1;N_HOMREF=10307;FREQ_HET=9.700261907071492e-05;FREQ_HOMALT=9.700261907071492e-05;FREQ_HOMREF=0.9998059947618586;N_BI_GENOS=10309;Central_Han_AN=2128;Cen
chr5	121148024	.	C	<INS:ME:LINE1>	.	PASS	SVTYPE=INS:ME:LINE1;END=121148024;SVLEN=758;AN=20618;AC=2;AF=9.700261907071492e-05;N_HET=0;N_HOMALT=1;N_HOMREF=10308;FREQ_HET=0.0;FREQ_HOMALT=9.700261907071492e-05;FREQ_HOMREF=0.9999029973809292;N_BI_GENOS=10309;East_Han_AN=6554;East_Han_AC=2;East_Han_AF=
chr5	121182946	.	T	<INS:ME:LINE1>	.	PASS	SVTYPE=INS:ME:LINE1;END=121182965;SVLEN=5998;AN=20618;AC=6;AF=0.0002910078572121447;N_HET=6;N_HOMALT=0;N_HOMREF=10303;FREQ_HET=0.0005820157144242894;FREQ_HOMALT=0.0;FREQ_HOMREF=0.9994179842855757;N_BI_GENOS=10309;Northwest_Han_AN=1296;Northwest_Han_AC=1;N
chr5	121265280	.	A	<INS:ME:LINE1>	.	PASS	SVTYPE=INS:ME:LINE1;END=121265296;SVLEN=383;AN=20618;AC=1;AF=4.850130953535746e-05;N_HET=1;N_HOMALT=0;N_HOMREF=10308;FREQ_HET=9.700261907071492e-05;FREQ_HOMALT=0.0;FREQ_HOMREF=0.9999029973809292;N_BI_GENOS=10309;North_Han_AN=2272;North_Han_AC=1;North_Han_
chr5	121284331	.	A	<INS:ME:LINE1>	.	PASS	SVTYPE=INS:ME:LINE1;END=121284347;SVLEN=2129;AN=20618;AC=2;AF=9.700261907071492e-05;N_HET=2;N_HOMALT=0;N_HOMREF=10307;FREQ_HET=0.00019400523814142983;FREQ_HOMALT=0.0;FREQ_HOMREF=0.9998059947618586;N_BI_GENOS=10309;South_Han_AN=1496;South_Han_AC=1;South_Ha
chr5	121405600	.	A	<INS:ME:LINE1>	.	PASS	SVTYPE=INS:ME:LINE1;END=121405613;SVLEN=110;AN=20618;AC=5;AF=0.00024250654767678728;N_HET=5;N_HOMALT=0;N_HOMREF=10304;FREQ_HET=0.00048501309535357455;FREQ_HOMALT=0.0;FREQ_HOMREF=0.9995149869046465;N_BI_GENOS=10309;South_Han_AN=1496;South_Han_AC=2;South_Ha
chr5	121582283	.	A	<INS:ME:LINE1>	.	PASS	SVTYPE=INS:ME:LINE1;END=121582298;SVLEN=552;AN=20618;AC=1;AF=4.850130953535746e-05;N_HET=1;N_HOMALT=0;N_HOMREF=10308;FREQ_HET=9.700261907071492e-05;FREQ_HOMALT=0.0;FREQ_HOMREF=0.9999029973809292;N_BI_GENOS=10309;East_Han_AN=6554;East_Han_AC=1;East_Han_AF=
chr5	121591131	.	G	<INS:ME:LINE1>	.	PASS	SVTYPE=INS:ME:LINE1;END=121591157;SVLEN=2054;AN=20618;AC=1;AF=4.850130953535746e-05;N_HET=1;N_HOMALT=0;N_HOMREF=10308;FREQ_HET=9.700261907071492e-05;FREQ_HOMALT=0.0;FREQ_HOMREF=0.9999029973809292;N_BI_GENOS=10309;East_Han_AN=6554;East_Han_AC=1;East_Han_AF
chr5	121651254	.	C	<INS:ME:LINE1>	.	PASS	SVTYPE=INS:ME:LINE1;END=121651254;SVLEN=729;AN=20618;AC=1;AF=4.850130953535746e-05;N_HET=1;N_HOMALT=0;N_HOMREF=10308;FREQ_HET=9.700261907071492e-05;FREQ_HOMALT=0.0;FREQ_HOMREF=0.9999029973809292;N_BI_GENOS=10309;East_Han_AN=6554;East_Han_AC=1;East_Han_AF=
chr5	121651984	.	C	<INS:ME:LINE1>	.	PASS	SVTYPE=INS:ME:LINE1;END=121652012;SVLEN=81;AN=20618;AC=1;AF=4.850130953535746e-05;N_HET=1;N_HOMALT=0;N_HOMREF=10308;FREQ_HET=9.700261907071492e-05;FREQ_HOMALT=0.0;FREQ_HOMREF=0.9999029973809292;N_BI_GENOS=10309;East_Han_AN=6554;East_Han_AC=1;East_Han_AF=0
chr5	121783614	.	G	<INS:ME:LINE1>	.	PASS	SVTYPE=INS:ME:LINE1;END=121783614;SVLEN=2400;AN=20618;AC=1;AF=4.850130953535746e-05;N_HET=1;N_HOMALT=0;N_HOMREF=10308;FREQ_HET=9.700261907071492e-05;FREQ_HOMALT=0.0;FREQ_HOMREF=0.9999029973809292;N_BI_GENOS=10309;North_Han_AN=2272;North_Han_AC=1;North_Han
chr5	121862066	.	T	<INS:ME:LINE1>	.	PASS	SVTYPE=INS:ME:LINE1;END=121862083;SVLEN=938;AN=20618;AC=1;AF=4.850130953535746e-05;N_HET=1;N_HOMALT=0;N_HOMREF=10308;FREQ_HET=9.700261907071492e-05;FREQ_HOMALT=0.0;FREQ_HOMREF=0.9999029973809292;N_BI_GENOS=10309;Northwest_Han_AN=1296;Northwest_Han_AC=1;No
chr5	122180844	.	A	<INS:ME:LINE1>	.	PASS	SVTYPE=INS:ME:LINE1;END=122180860;SVLEN=1222;AN=20618;AC=2;AF=9.700261907071492e-05;N_HET=2;N_HOMALT=0;N_HOMREF=10307;FREQ_HET=0.00019400523814142983;FREQ_HOMALT=0.0;FREQ_HOMREF=0.9998059947618586;N_BI_GENOS=10309;East_Han_AN=6554;East_Han_AC=2;East_Han_A
chr5	122541427	.	G	<INS:ME:LINE1>	.	PASS	SVTYPE=INS:ME:LINE1;END=122541442;SVLEN=6000;AN=20618;AC=2;AF=9.700261907071492e-05;N_HET=2;N_HOMALT=0;N_HOMREF=10307;FREQ_HET=0.00019400523814142983;FREQ_HOMALT=0.0;FREQ_HOMREF=0.9998059947618586;N_BI_GENOS=10309;North_Han_AN=2272;North_Han_AC=1;North_Ha
chr5	122607690	.	A	<INS:ME:LINE1>	.	PASS	SVTYPE=INS:ME:LINE1;END=122607707;SVLEN=1371;AN=20618;AC=1;AF=4.850130953535746e-05;N_HET=1;N_HOMALT=0;N_HOMREF=10308;FREQ_HET=9.700261907071492e-05;FREQ_HOMALT=0.0;FREQ_HOMREF=0.9999029973809292;N_BI_GENOS=10309;South_Han_AN=1496;South_Han_AC=1;South_Han
chr5	122968975	.	T	<INS:ME:LINE1>	.	PASS	SVTYPE=INS:ME:LINE1;END=122968992;SVLEN=2687;AN=20618;AC=4;AF=0.00019400523814142983;N_HET=4;N_HOMALT=0;N_HOMREF=10305;FREQ_HET=0.00038801047628285966;FREQ_HOMALT=0.0;FREQ_HOMREF=0.9996119895237171;N_BI_GENOS=10309;Lingnan_Han_AN=1888;Lingnan_Han_AC=1;Lin
chr5	123077639	.	G	<INS:ME:LINE1>	.	PASS	SVTYPE=INS:ME:LINE1;END=123077649;SVLEN=5995;AN=20618;AC=3;AF=0.00014550392860607236;N_HET=1;N_HOMALT=1;N_HOMREF=10307;FREQ_HET=9.700261907071492e-05;FREQ_HOMALT=9.700261907071492e-05;FREQ_HOMREF=0.9998059947618586;N_BI_GENOS=10309;East_Han_AN=6554;East_H
chr5	123297919	.	A	<INS:ME:LINE1>	.	PASS	SVTYPE=INS:ME:LINE1;END=123297933;SVLEN=91;AN=20618;AC=1;AF=4.850130953535746e-05;N_HET=1;N_HOMALT=0;N_HOMREF=10308;FREQ_HET=9.700261907071492e-05;FREQ_HOMALT=0.0;FREQ_HOMREF=0.9999029973809292;N_BI_GENOS=10309;North_Han_AN=2272;North_Han_AC=1;North_Han_A
chr5	123314667	.	T	<INS:ME:LINE1>	.	PASS	SVTYPE=INS:ME:LINE1;END=123314667;SVLEN=310;AN=20618;AC=1;AF=4.850130953535746e-05;N_HET=1;N_HOMALT=0;N_HOMREF=10308;FREQ_HET=9.700261907071492e-05;FREQ_HOMALT=0.0;FREQ_HOMREF=0.9999029973809292;N_BI_GENOS=10309;East_Han_AN=6554;East_Han_AC=1;East_Han_AF=
chr5	123314768	.	A	<INS:ME:LINE1>	.	PASS	SVTYPE=INS:ME:LINE1;END=123314768;SVLEN=33;AN=20618;AC=1;AF=4.850130953535746e-05;N_HET=1;N_HOMALT=0;N_HOMREF=10308;FREQ_HET=9.700261907071492e-05;FREQ_HOMALT=0.0;FREQ_HOMREF=0.9999029973809292;N_BI_GENOS=10309;East_Han_AN=6554;East_Han_AC=1;East_Han_AF=0
chr5	123317132	.	C	<INS:ME:LINE1>	.	PASS	SVTYPE=INS:ME:LINE1;END=123317140;SVLEN=2949;AN=20618;AC=1;AF=4.850130953535746e-05;N_HET=1;N_HOMALT=0;N_HOMREF=10308;FREQ_HET=9.700261907071492e-05;FREQ_HOMALT=0.0;FREQ_HOMREF=0.9999029973809292;N_BI_GENOS=10309;East_Han_AN=6554;East_Han_AC=1;East_Han_AF
chr5	123685420	.	A	<INS:ME:LINE1>	.	PASS	SVTYPE=INS:ME:LINE1;END=123685420;SVLEN=797;AN=20618;AC=1;AF=4.850130953535746e-05;N_HET=1;N_HOMALT=0;N_HOMREF=10308;FREQ_HET=9.700261907071492e-05;FREQ_HOMALT=0.0;FREQ_HOMREF=0.9999029973809292;N_BI_GENOS=10309;East_Han_AN=6554;East_Han_AC=1;East_Han_AF=

chr5	123704953	.	C	<INS:ME:LINE1>	.	PASS	SVTYPE=INS:ME:LINE1;END=123704968;SVLEN=6004;AN=20618;AC=1;AF=4.850130953535746e-05;N_HET=1;N_HOMALT=0;N_HOMREF=10308;FREQ_HET=9.700261907071492e-05;FREQ_HOMALT=0.0;FREQ_HOMREF=0.9999029973809292;N_BI_GENOS=10309;Central_Han_AN=2128;Central_Han_AC=1;Centr
chr5	124222081	.	T	<INS:ME:LINE1>	.	PASS	SVTYPE=INS:ME:LINE1;END=124222097;SVLEN=682;AN=20618;AC=10;AF=0.00048501309535357455;N_HET=10;N_HOMALT=0;N_HOMREF=10299;FREQ_HET=0.0009700261907071491;FREQ_HOMALT=0.0;FREQ_HOMREF=0.9990299738092928;N_BI_GENOS=10309;Northwest_Han_AN=1296;Northwest_Han_AC=1
chr5	124248306	.	C	<INS:ME:LINE1>	.	PASS	SVTYPE=INS:ME:LINE1;END=124248306;SVLEN=915;AN=20618;AC=1;AF=4.850130953535746e-05;N_HET=1;N_HOMALT=0;N_HOMREF=10308;FREQ_HET=9.700261907071492e-05;FREQ_HOMALT=0.0;FREQ_HOMREF=0.9999029973809292;N_BI_GENOS=10309;East_Han_AN=6554;East_Han_AC=1;East_Han_AF=
chr5	124248848	.	T	<INS:ME:LINE1>	.	PASS	SVTYPE=INS:ME:LINE1;END=124248848;SVLEN=1232;AN=20618;AC=1;AF=4.850130953535746e-05;N_HET=1;N_HOMALT=0;N_HOMREF=10308;FREQ_HET=9.700261907071492e-05;FREQ_HOMALT=0.0;FREQ_HOMREF=0.9999029973809292;N_BI_GENOS=10309;East_Han_AN=6554;East_Han_AC=1;East_Han_AF
chr5	124375552	.	A	<INS:ME:LINE1>	.	PASS	SVTYPE=INS:ME:LINE1;END=124375566;SVLEN=6016;AN=20618;AC=2;AF=9.700261907071492e-05;N_HET=0;N_HOMALT=1;N_HOMREF=10308;FREQ_HET=0.0;FREQ_HOMALT=9.700261907071492e-05;FREQ_HOMREF=0.9999029973809292;N_BI_GENOS=10309;South_Han_AN=1496;South_Han_AC=2;South_Han
chr5	124412066	.	A	<INS:ME:LINE1>	.	PASS	SVTYPE=INS:ME:LINE1;END=124412082;SVLEN=6000;AN=20618;AC=1;AF=4.850130953535746e-05;N_HET=1;N_HOMALT=0;N_HOMREF=10308;FREQ_HET=9.700261907071492e-05;FREQ_HOMALT=0.0;FREQ_HOMREF=0.9999029973809292;N_BI_GENOS=10309;Lingnan_Han_AN=1888;Lingnan_Han_AC=1;Lingn
chr5	124567565	.	A	<INS:ME:LINE1>	.	PASS	SVTYPE=INS:ME:LINE1;END=124567578;SVLEN=5998;AN=20618;AC=1;AF=4.850130953535746e-05;N_HET=1;N_HOMALT=0;N_HOMREF=10308;FREQ_HET=9.700261907071492e-05;FREQ_HOMALT=0.0;FREQ_HOMREF=0.9999029973809292;N_BI_GENOS=10309;Lingnan_Han_AN=1888;Lingnan_Han_AC=1;Lingn
chr5	124819638	.	G	<INS:ME:LINE1>	.	PASS	SVTYPE=INS:ME:LINE1;END=124819656;SVLEN=4949;AN=20618;AC=1065;AF=0.05165389465515569;N_HET=967;N_HOMALT=49;N_HOMREF=9293;FREQ_HET=0.09380153264138132;FREQ_HOMALT=0.004753128334465031;FREQ_HOMREF=0.9014453390241537;N_BI_GENOS=10309;South_Han_AN=1496;South_
chr5	125704347	.	T	<INS:ME:LINE1>	.	PASS	SVTYPE=INS:ME:LINE1;END=125704352;SVLEN=792;AN=20618;AC=750;AF=0.03637598215151809;N_HET=642;N_HOMALT=54;N_HOMREF=9613;FREQ_HET=0.06227568144339897;FREQ_HOMALT=0.005238141429818605;FREQ_HOMREF=0.9324861771267824;N_BI_GENOS=10309;South_Han_AN=1496;South_Ha
chr5	125858672	.	T	<INS:ME:LINE1>	.	PASS	SVTYPE=INS:ME:LINE1;END=125858672;SVLEN=423;AN=20618;AC=1;AF=4.850130953535746e-05;N_HET=1;N_HOMALT=0;N_HOMREF=10308;FREQ_HET=9.700261907071492e-05;FREQ_HOMALT=0.0;FREQ_HOMREF=0.9999029973809292;N_BI_GENOS=10309;East_Han_AN=6554;East_Han_AC=1;East_Han_AF=
chr5	125905992	.	A	<INS:ME:LINE1>	.	PASS	SVTYPE=INS:ME:LINE1;END=125906006;SVLEN=830;AN=20618;AC=1;AF=4.850130953535746e-05;N_HET=1;N_HOMALT=0;N_HOMREF=10308;FREQ_HET=9.700261907071492e-05;FREQ_HOMALT=0.0;FREQ_HOMREF=0.9999029973809292;N_BI_GENOS=10309;Southeast_Han_AN=1908;Southeast_Han_AC=1;So
chr5	125964253	.	A	<INS:ME:LINE1>	.	PASS	SVTYPE=INS:ME:LINE1;END=125964267;SVLEN=6002;AN=20618;AC=1;AF=4.850130953535746e-05;N_HET=1;N_HOMALT=0;N_HOMREF=10308;FREQ_HET=9.700261907071492e-05;FREQ_HOMALT=0.0;FREQ_HOMREF=0.9999029973809292;N_BI_GENOS=10309;South_Han_AN=1496;South_Han_AC=1;South_Han
chr5	126131540	.	A	<INS:ME:LINE1>	.	PASS	SVTYPE=INS:ME:LINE1;END=126131553;SVLEN=5993;AN=20618;AC=343;AF=0.016635949170627606;N_HET=291;N_HOMALT=26;N_HOMREF=9992;FREQ_HET=0.028227762149578037;FREQ_HOMALT=0.0025220680958385876;FREQ_HOMREF=0.9692501697545833;N_BI_GENOS=10309;South_Han_AN=1496;Sout
chr5	126918717	.	G	<INS:ME:LINE1>	.	PASS	SVTYPE=INS:ME:LINE1;END=126918734;SVLEN=1008;AN=20618;AC=2;AF=9.700261907071492e-05;N_HET=2;N_HOMALT=0;N_HOMREF=10307;FREQ_HET=0.00019400523814142983;FREQ_HOMALT=0.0;FREQ_HOMREF=0.9998059947618586;N_BI_GENOS=10309;South_Han_AN=1496;South_Han_AC=1;South_Ha
chr5	126966641	.	A	<INS:ME:LINE1>	.	PASS	SVTYPE=INS:ME:LINE1;END=126966656;SVLEN=2825;AN=20618;AC=2;AF=9.700261907071492e-05;N_HET=2;N_HOMALT=0;N_HOMREF=10307;FREQ_HET=0.00019400523814142983;FREQ_HOMALT=0.0;FREQ_HOMREF=0.9998059947618586;N_BI_GENOS=10309;East_Han_AN=6554;East_Han_AC=2;East_Han_A
chr5	126979260	.	G	<INS:ME:LINE1>	.	PASS	SVTYPE=INS:ME:LINE1;END=126979276;SVLEN=4457;AN=20618;AC=1;AF=4.850130953535746e-05;N_HET=1;N_HOMALT=0;N_HOMREF=10308;FREQ_HET=9.700261907071492e-05;FREQ_HOMALT=0.0;FREQ_HOMREF=0.9999029973809292;N_BI_GENOS=10309;Northwest_Han_AN=1296;Northwest_Han_AC=1;N
chr5	126979405	.	A	<INS:ME:LINE1>	.	PASS	SVTYPE=INS:ME:LINE1;END=126979417;SVLEN=388;AN=20618;AC=1;AF=4.850130953535746e-05;N_HET=1;N_HOMALT=0;N_HOMREF=10308;FREQ_HET=9.700261907071492e-05;FREQ_HOMALT=0.0;FREQ_HOMREF=0.9999029973809292;N_BI_GENOS=10309;North_Han_AN=2272;North_Han_AC=1;North_Han_
chr5	127399339	.	G	<INS:ME:LINE1>	.	PASS	SVTYPE=INS:ME:LINE1;END=127399355;SVLEN=2358;AN=20618;AC=12;AF=0.0005820157144242894;N_HET=12;N_HOMALT=0;N_HOMREF=10297;FREQ_HET=0.0011640314288485789;FREQ_HOMALT=0.0;FREQ_HOMREF=0.9988359685711514;N_BI_GENOS=10309;South_Han_AN=1496;South_Han_AC=2;South_H
chr5	127421819	.	T	<INS:ME:LINE1>	.	PASS	SVTYPE=INS:ME:LINE1;END=127421824;SVLEN=1544;AN=20618;AC=4;AF=0.00019400523814142983;N_HET=4;N_HOMALT=0;N_HOMREF=10305;FREQ_HET=0.00038801047628285966;FREQ_HOMALT=0.0;FREQ_HOMREF=0.9996119895237171;N_BI_GENOS=10309;South_Han_AN=1496;South_Han_AC=1;South_H
chr5	127648391	.	G	<INS:ME:LINE1>	.	PASS	SVTYPE=INS:ME:LINE1;END=127648406;SVLEN=731;AN=20618;AC=1;AF=4.850130953535746e-05;N_HET=1;N_HOMALT=0;N_HOMREF=10308;FREQ_HET=9.700261907071492e-05;FREQ_HOMALT=0.0;FREQ_HOMREF=0.9999029973809292;N_BI_GENOS=10309;Lingnan_Han_AN=1888;Lingnan_Han_AC=1;Lingna
chr5	127872986	.	A	<INS:ME:LINE1>	.	PASS	SVTYPE=INS:ME:LINE1;END=127873003;SVLEN=6021;AN=20618;AC=1;AF=4.850130953535746e-05;N_HET=1;N_HOMALT=0;N_HOMREF=10308;FREQ_HET=9.700261907071492e-05;FREQ_HOMALT=0.0;FREQ_HOMREF=0.9999029973809292;N_BI_GENOS=10309;East_Han_AN=6554;East_Han_AC=1;East_Han_AF
chr5	127993874	.	G	<INS:ME:LINE1>	.	PASS	SVTYPE=INS:ME:LINE1;END=127993876;SVLEN=1516;AN=20618;AC=1;AF=4.850130953535746e-05;N_HET=1;N_HOMALT=0;N_HOMREF=10308;FREQ_HET=9.700261907071492e-05;FREQ_HOMALT=0.0;FREQ_HOMREF=0.9999029973809292;N_BI_GENOS=10309;Southeast_Han_AN=1908;Southeast_Han_AC=1;S
chr5	128307394	.	C	<INS:ME:LINE1>	.	PASS	SVTYPE=INS:ME:LINE1;END=128307401;SVLEN=459;AN=20618;AC=26;AF=0.001261034047919293 8;N_HET=26;N_HOMALT=0;N_HOMREF=10283;FREQ_HET=0.0025220680958385876;FREQ_HOMALT=0.0;FREQ_HOMREF=0.9974779319041615;N_BI_GENOS=10309;South_Han_AN=1496;South_Han_AC=1;South_Ha
chr5	128437651	.	A	<INS:ME:LINE1>	.	PASS	SVTYPE=INS:ME:LINE1;END=128437662;SVLEN=1006;AN=20618;AC=5;AF=0.00024250654767678728;N_HET=3;N_HOMALT=1;N_HOMREF=10305;FREQ_HET=0.0002910078572121447;FREQ_HOMALT=9.700261907071492e-05;FREQ_HOMREF=0.9996119895237171;N_BI_GENOS=10309;South_Han_AN=1496;South
chr5	128467826	.	A	<INS:ME:LINE1>	.	PASS	SVTYPE=INS:ME:LINE1;END=128467828;SVLEN=924;AN=20618;AC=1;AF=4.850130953535746e-05;N_HET=1;N_HOMALT=0;N_HOMREF=10308;FREQ_HET=9.700261907071492e-05;FREQ_HOMALT=0.0;FREQ_HOMREF=0.9999029973809292;N_BI_GENOS=10309;East_Han_AN=6554;East_Han_AC=1;East_Han_AF=
chr5	128835596	.	C	<INS:ME:LINE1>	.	PASS	SVTYPE=INS:ME:LINE1;END=128835601;SVLEN=424;AN=20618;AC=2;AF=9.700261907071492e-05;N_HET=2;N_HOMALT=0;N_HOMREF=10307;FREQ_HET=0.00019400523814142983;FREQ_HOMALT=0.0;FREQ_HOMREF=0.9998059947618586;N_BI_GENOS=10309;East_Han_AN=6554;East_Han_AC=1;East_Han_AF
chr5	128879424	.	T	<INS:ME:LINE1>	.	PASS	SVTYPE=INS:ME:LINE1;END=128879428;SVLEN=243;AN=20618;AC=3;AF=0.0001455039286060 7236;N_HET=3;N_HOMALT=0;N_HOMREF=10306;FREQ_HET=0.0002910078572121447;FREQ_HOMALT=0.0;FREQ_HOMREF=0.9997089921427879;N_BI_GENOS=10309;South_Han_AN=1496;South_Han_AC=1;South_Han
chr5	129156923	.	A	<INS:ME:LINE1>	.	PASS	SVTYPE=INS:ME:LINE1;END=129156923;SVLEN=148;AN=20618;AC=1;AF=4.850130953535746e-05;N_HET=1;N_HOMALT=0;N_HOMREF=10308;FREQ_HET=9.700261907071492e-05;FREQ_HOMALT=0.0;FREQ_HOMREF=0.9999029973809292;N_BI_GENOS=10309;Northwest_Han_AN=1296;Northwest_Han_AC=1;No
chr5	129274511	.	G	<INS:ME:LINE1>	.	PASS	SVTYPE=INS:ME:LINE1;END=129274528;SVLEN=2106;AN=20618;AC=1;AF=4.850130953535746e-05;N_HET=1;N_HOMALT=0;N_HOMREF=10308;FREQ_HET=9.700261907071492e-05;FREQ_HOMALT=0.0;FREQ_HOMREF=0.9999029973809292;N_BI_GENOS=10309;Northwest_Han_AN=1296;Northwest_Han_AC=1;N
chr5	129767022	.	A	<INS:ME:LINE1>	.	PASS	SVTYPE=INS:ME:LINE1;END=129767041;SVLEN=1401;AN=20618;AC=104;AF=0.005044136191677175;N_HET=102;N_HOMALT=1;N_HOMREF=10206;FREQ_HET=0.009894267145212922;FREQ_HOMALT=9.700261907071492e-05;FREQ_HOMREF=0.9900087302357163;N_BI_GENOS=10309;South_Han_AN=1496;Sout
chr5	129820418	.	T	<INS:ME:LINE1>	.	PASS	SVTYPE=INS:ME:LINE1;END=129820431;SVLEN=6009;AN=20618;AC=2;AF=9.700261907071492e-05;N_HET=2;N_HOMALT=0;N_HOMREF=10307;FREQ_HET=0.00019400523814142983;FREQ_HOMALT=0.0;FREQ_HOMREF=0.9998059947618586;N_BI_GENOS=10309;East_Han_AN=6554;East_Han_AC=1;East_Han_A
chr5	129940612	.	T	<INS:ME:LINE1>	.	PASS	SVTYPE=INS:ME:LINE1;END=129940620;SVLEN=342;AN=20618;AC=2;AF=9.700261907071492e-05;N_HET=2;N_HOMALT=0;N_HOMREF=10307;FREQ_HET=0.00019400523814142983;FREQ_HOMALT=0.0;FREQ_HOMREF=0.9998059947618586;N_BI_GENOS=10309;East_Han_AN=6554;East_Han_AC=1;East_Han_AF
chr5	129997380	.	A	<INS:ME:LINE1>	.	PASS	SVTYPE=INS:ME:LINE1;END=129997397;SVLEN=364;AN=20618;AC=1;AF=4.850130953535746e-05;N_HET=1;N_HOMALT=0;N_HOMREF=10308;FREQ_HET=9.700261907071492e-05;FREQ_HOMALT=0.0;FREQ_HOMREF=0.9999029973809292;N_BI_GENOS=10309;Central_Han_AN=2128;Central_Han_AC=1;Centra
chr5	130296639	.	C	<INS:ME:LINE1>	.	PASS	SVTYPE=INS:ME:LINE1;END=130296653;SVLEN=2179;AN=20618;AC=2;AF=9.700261907071492e-05;N_HET=2;N_HOMALT=0;N_HOMREF=10307;FREQ_HET=0.00019400523814142983;FREQ_HOMALT=0.0;FREQ_HOMREF=0.9998059947618586;N_BI_GENOS=10309;Central_Han_AN=2128;Central_Han_AC=1;Cent
chr5	130502963	.	A	<INS:ME:LINE1>	.	PASS	SVTYPE=INS:ME:LINE1;END=130502981;SVLEN=2549;AN=20618;AC=1;AF=4.850130953535746e-05;N_HET=1;N_HOMALT=0;N_HOMREF=10308;FREQ_HET=9.700261907071492e-05;FREQ_HOMALT=0.0;FREQ_HOMREF=0.9999029973809292;N_BI_GENOS=10309;Lingnan_Han_AN=1888;Lingnan_Han_AC=1;Lingn
chr5	130507182	.	C	<INS:ME:LINE1>	.	PASS	SVTYPE=INS:ME:LINE1;END=130507197;SVLEN=6005;AN=20618;AC=1;AF=4.850130953535746e-05;N_HET=1;N_HOMALT=0;N_HOMREF=10308;FREQ_HET=9.700261907071492e-05;FREQ_HOMALT=0.0;FREQ_HOMREF=0.9999029973809292;N_BI_GENOS=10309;North_Han_AN=2272;North_Han_AC=1;North_Han
chr5	130561744	.	A	<INS:ME:LINE1>	.	PASS	SVTYPE=INS:ME:LINE1;END=130561744;SVLEN=911;AN=20618;AC=1;AF=4.850130953535746e-05;N_HET=1;N_HOMALT=0;N_HOMREF=10308;FREQ_HET=9.700261907071492e-05;FREQ_HOMALT=0.0;FREQ_HOMREF=0.9999029973809292;N_BI_GENOS=10309;East_Han_AN=6554;East_Han_AC=1;East_Han_AF=
chr5	130912632	.	A	<INS:ME:LINE1>	.	PASS	SVTYPE=INS:ME:LINE1;END=130912648;SVLEN=6032;AN=20618;AC=1;AF=4.850130953535746e-05;N_HET=1;N_HOMALT=0;N_HOMREF=10308;FREQ_HET=9.700261907071492e-05;FREQ_HOMALT=0.0;FREQ_HOMREF=0.9999029973809292;N_BI_GENOS=10309;Northwest_Han_AN=1296;Northwest_Han_AC=1;N
chr5	131862672	.	T	<INS:ME:LINE1>	.	PASS	SVTYPE=INS:ME:LINE1;END=131862688;SVLEN=1399;AN=20618;AC=3;AF=0.0001455039286060 7236;N_HET=3;N_HOMALT=0;N_HOMREF=10306;FREQ_HET=0.0002910078572121447;FREQ_HOMALT=0.0;FREQ_HOMREF=0.9997089921427879;N_BI_GENOS=10309;East_Han_AN=6554;East_Han_AC=1;East_Han_A
chr5	131968733	.	T	<INS:ME:LINE1>	.	PASS	SVTYPE=INS:ME:LINE1;END=131968746;SVLEN=783;AN=20618;AC=1;AF=4.850130953535746e-05;N_HET=1;N_HOMALT=0;N_HOMREF=10308;FREQ_HET=9.700261907071492e-05;FREQ_HOMALT=0.0;FREQ_HOMREF=0.9999029973809292;N_BI_GENOS=10309;Central_Han_AN=2128;Central_Han_AC=1;Centra
chr5	132102368	.	G	<INS:ME:LINE1>	.	PASS	SVTYPE=INS:ME:LINE1;END=132102383;SVLEN=853;AN=20618;AC=2;AF=9.700261907071492e-05;N_HET=2;N_HOMALT=0;N_HOMREF=10307;FREQ_HET=0.00019400523814142983;FREQ_HOMALT=0.0;FREQ_HOMREF=0.9998059947618586;N_BI_GENOS=10309;Northwest_Han_AN=1296;Northwest_Han_AC=1;N
chr5	132111831	.	A	<INS:ME:LINE1>	.	PASS	SVTYPE=INS:ME:LINE1;END=132111848;SVLEN=1246;AN=20618;AC=2;AF=9.700261907071492e-05;N_HET=2;N_HOMALT=0;N_HOMREF=10307;FREQ_HET=0.00019400523814142983;FREQ_HOMALT=0.0;FREQ_HOMREF=0.9998059947618586;N_BI_GENOS=10309;Lingnan_Han_AN=1888;Lingnan_Han_AC=2;Ling
chr5	132143354	.	A	<INS:ME:LINE1>	.	PASS	SVTYPE=INS:ME:LINE1;END=132143365;SVLEN=728;AN=20618;AC=260;AF=0.012610340479192938;N_HET=204;N_HOMALT=28;N_HOMREF=10077;FREQ_HET=0.019788534290425843;FREQ_HOMALT=0.0027160733339800175;FREQ_HOMREF=0.9774953923755941;N_BI_GENOS=10309;South_Han_AN=1496;Sout
chr5	132385304	.	T	<INS:ME:LINE1>	.	PASS	SVTYPE=INS:ME:LINE1;END=132385321;SVLEN=3323;AN=20618;AC=1;AF=4.850130953535746e-05;N_HET=1;N_HOMALT=0;N_HOMREF=10308;FREQ_HET=9.700261907071492e-05;FREQ_HOMALT=0.0;FREQ_HOMREF=0.9999029973809292;N_BI_GENOS=10309;North_Han_AN=2272;North_Han_AC=1;North_Han
chr5	132643292	.	C	<INS:ME:LINE1>	.	PASS	SVTYPE=INS:ME:LINE1;END=132643306;SVLEN=984;AN=20618;AC=2;AF=9.700261907071492e-05;N_HET=2;N_HOMALT=0;N_HOMREF=10307;FREQ_HET=0.00019400523814142983;FREQ_HOMALT=0.0;FREQ_HOMREF=0.9998059947618586;N_BI_GENOS=10309;East_Han_AN=6554;East_Han_AC=1;East_Han_AF
chr5	132971735	.	T	<INS:ME:LINE1>	.	PASS	SVTYPE=INS:ME:LINE1;END=132971751;SVLEN=213;AN=20618;AC=1;AF=4.850130953535746e-05;N_HET=1;N_HOMALT=0;N_HOMREF=10308;FREQ_HET=9.700261907071492e-05;FREQ_HOMALT=0.0;FREQ_HOMREF=0.9999029973809292;N_BI_GENOS=10309;East_Han_AN=6554;East_Han_AC=1;East_Han_AF=
chr5	133401279	.	A	<INS:ME:LINE1>	.	PASS	SVTYPE=INS:ME:LINE1;END=133401296;SVLEN=5999;AN=20618;AC=8;AF=0.00038801047628285966;N_HET=8;N_HOMALT=0;N_HOMREF=10301;FREQ_HET=0.0007760209525657193;FREQ_HOMALT=0.0;FREQ_HOMREF=0.9992239790474343;N_BI_GENOS=10309;South_Han_AN=1496;South_Han_AC=2;South_Ha
chr5	133469180	.	A	<INS:ME:LINE1>	.	PASS	SVTYPE=INS:ME:LINE1;END=133469190;SVLEN=189;AN=20618;AC=1;AF=4.850130953535746e-05;N_HET=1;N_HOMALT=0;N_HOMREF=10308;FREQ_HET=9.700261907071492e-05;FREQ_HOMALT=0.0;FREQ_HOMREF=0.9999029973809292;N_BI_GENOS=10309;East_Han_AN=6554;East_Han_AC=1;East_Han_AF=
chr5	133570324	.	A	<INS:ME:LINE1>	.	PASS	SVTYPE=INS:ME:LINE1;END=133570337;SVLEN=5998;AN=20618;AC=2;AF=9.700261907071492e-05;N_HET=2;N_HOMALT=0;N_HOMREF=10307;FREQ_HET=0.00019400523814142983;FREQ_HOMALT=0.0;FREQ_HOMREF=0.9998059947618586;N_BI_GENOS=10309;Hui_AN=444;Hui_AC=2;Hui_AF=0.004504504504
chr5	133598394	.	A	<INS:ME:LINE1>	.	PASS	SVTYPE=INS:ME:LINE1;END=133598407;SVLEN=3593;AN=20618;AC=1362;AF=0.06605878358715685;N_HET=1184;N_HOMALT=89;N_HOMREF=9036;FREQ_HET=0.11485110097972645;FREQ_HOMALT=0.008633233097293627;FREQ_HOMREF=0.8765156659229799;N_BI_GENOS=10309;South_Han_AN=1496;South
chr5	133667925	.	A	<INS:ME:LINE1>	.	PASS	SVTYPE=INS:ME:LINE1;END=133667942;SVLEN=6050;AN=20618;AC=1;AF=4.850130953535746e-05;N_HET=1;N_HOMALT=0;N_HOMREF=10308;FREQ_HET=9.700261907071492e-05;FREQ_HOMALT=0.0;FREQ_HOMREF=0.9999029973809292;N_BI_GENOS=10309;Zhuang_AN=444;Zhuang_AC=1;Zhuang_AF=0.0022
chr5	133768879	.	A	<INS:ME:LINE1>	.	PASS	SVTYPE=INS:ME:LINE1;END=133768896;SVLEN=6024;AN=20618;AC=1;AF=4.850130953535746e-05;N_HET=1;N_HOMALT=0;N_HOMREF=10308;FREQ_HET=9.700261907071492e-05;FREQ_HOMALT=0.0;FREQ_HOMREF=0.9999029973809292;N_BI_GENOS=10309;North_Han_AN=2272;North_Han_AC=1;North_Han
chr5	133777630	.	G	<INS:ME:LINE1>	.	PASS	SVTYPE=INS:ME:LINE1;END=133777641;SVLEN=6043;AN=20618;AC=2;AF=9.700261907071492e-05;N_HET=2;N_HOMALT=0;N_HOMREF=10307;FREQ_HET=0.00019400523814142983;FREQ_HOMALT=0.0;FREQ_HOMREF=0.9998059947618586;N_BI_GENOS=10309;Central_Han_AN=2128;Central_Han_AC=1;Cent
chr5	134232590	.	A	<INS:ME:LINE1>	.	PASS	SVTYPE=INS:ME:LINE1;END=134232607;SVLEN=6006;AN=20618;AC=2;AF=9.700261907071492e-05;N_HET=2;N_HOMALT=0;N_HOMREF=10307;FREQ_HET=0.00019400523814142983;FREQ_HOMALT=0.0;FREQ_HOMREF=0.9998059947618586;N_BI_GENOS=10309;Southeast_Han_AN=1908;Southeast_Han_AC=2;
chr5	135207836	.	G	<INS:ME:LINE1>	.	PASS	SVTYPE=INS:ME:LINE1;END=135207854;SVLEN=1924;AN=20618;AC=2;AF=9.700261907071492e-05;N_HET=2;N_HOMALT=0;N_HOMREF=10307;FREQ_HET=0.00019400523814142983;FREQ_HOMALT=0.0;FREQ_HOMREF=0.9998059947618586;N_BI_GENOS=10309;Central_Han_AN=2128;Central_Han_AC=2;Cent
chr5	135371906	.	A	<INS:ME:LINE1>	.	PASS	SVTYPE=INS:ME:LINE1;END=135371923;SVLEN=5993;AN=20618;AC=157;AF=0.007614705597051121;N_HET=149;N_HOMALT=4;N_HOMREF=10156;FREQ_HET=0.014453390241536522;FREQ_HOMALT=0.00038801047628285966;FREQ_HOMREF=0.9851585992821806;N_BI_GENOS=10309;South_Han_AN=1496;Sou
chr5	135377363	.	C	<INS:ME:LINE1>	.	PASS	SVTYPE=INS:ME:LINE1;END=135377372;SVLEN=5989;AN=20618;AC=1;AF=4.850130953535746e-05;N_HET=1;N_HOMALT=0;N_HOMREF=10308;FREQ_HET=9.700261907071492e-05;FREQ_HOMALT=0.0;FREQ_HOMREF=0.9999029973809292;N_BI_GENOS=10309;South_Han_AN=1496;South_Han_AC=1;South_Han
chr5	135757234	.	A	<INS:ME:LINE1>	.	PASS	SVTYPE=INS:ME:LINE1;END=135757234;SVLEN=5959;AN=20618;AC=1;AF=4.850130953535746e-05;N_HET=1;N_HOMALT=0;N_HOMREF=10308;FREQ_HET=9.700261907071492e-05;FREQ_HOMALT=0.0;FREQ_HOMREF=0.9999029973809292;N_BI_GENOS=10309;Tibetan_AN=414;Tibetan_AC=1;Tibetan_AF=0.0
chr5	135971143	.	T	<INS:ME:LINE1>	.	PASS	SVTYPE=INS:ME:LINE1;END=135971155;SVLEN=6008;AN=20618;AC=2;AF=9.700261907071492e-05;N_HET=2;N_HOMALT=0;N_HOMREF=10307;FREQ_HET=0.00019400523814142983;FREQ_HOMALT=0.0;FREQ_HOMREF=0.9998059947618586;N_BI_GENOS=10309;East_Han_AN=6554;East_Han_AC=2;East_Han_A
chr5	136313698	.	G	<INS:ME:LINE1>	.	PASS	SVTYPE=INS:ME:LINE1;END=136313713;SVLEN=3011;AN=20618;AC=1;AF=4.850130953535746e-05;N_HET=1;N_HOMALT=0;N_HOMREF=10308;FREQ_HET=9.700261907071492e-05;FREQ_HOMALT=0.0;FREQ_HOMREF=0.9999029973809292;N_BI_GENOS=10309;Northwest_Han_AN=1296;Northwest_Han_AC=1;N
chr5	136322959	.	C	<INS:ME:LINE1>	.	PASS	SVTYPE=INS:ME:LINE1;END=136322976;SVLEN=6071;AN=20618;AC=3;AF=0.0001455039286060 7236;N_HET=3;N_HOMALT=0;N_HOMREF=10306;FREQ_HET=0.0002910078572121447;FREQ_HOMALT=0.0;FREQ_HOMREF=0.9997089921427879;N_BI_GENOS=10309;East_Han_AN=6554;East_Han_AC=2;East_Han_A
chr5	137054350	.	A	<INS:ME:LINE1>	.	PASS	SVTYPE=INS:ME:LINE1;END=137054350;SVLEN=219;AN=20618;AC=2;AF=9.700261907071492e-05;N_HET=2;N_HOMALT=0;N_HOMREF=10307;FREQ_HET=0.00019400523814142983;FREQ_HOMALT=0.0;FREQ_HOMREF=0.9998059947618586;N_BI_GENOS=10309;South_Han_AN=1496;South_Han_AC=1;South_Han
chr5	137679085	.	G	<INS:ME:LINE1>	.	PASS	SVTYPE=INS:ME:LINE1;END=137679101;SVLEN=5998;AN=20618;AC=11253;AF=0.5457852362013774;N_HET=5005;N_HOMALT=3124;N_HOMREF=2180;FREQ_HET=0.4854981084489281;FREQ_HOMALT=0.3030361819769134;FREQ_HOMREF=0.21146570957415 85;N_BI_GENOS=10309;South_Han_AN=1496;South_
chr5	137794457	.	C	<INS:ME:LINE1>	.	PASS	SVTYPE=INS:ME:LINE1;END=137794470;SVLEN=519;AN=20618;AC=5447;AF=0.2641866330390920 7;N_HET=3381;N_HOMALT=1033;N_HOMREF=5895;FREQ_HET=0.3279658550780871;FREQ_HOMALT=0.1002037055000485;FREQ_HOMREF=0.5718304394218644;N_BI_GENOS=10309;South_Han_AN=1496;South_H
chr5	137865711	.	A	<INS:ME:LINE1>	.	PASS	SVTYPE=INS:ME:LINE1;END=137865725;SVLEN=6014;AN=20618;AC=1;AF=4.850130953535746e-05;N_HET=1;N_HOMALT=0;N_HOMREF=10308;FREQ_HET=9.700261907071492e-05;FREQ_HOMALT=0.0;FREQ_HOMREF=0.9999029973809292;N_BI_GENOS=10309;East_Han_AN=6554;East_Han_AC=1;East_Han_AF
chr5	139945076	.	T	<INS:ME:LINE1>	.	PASS	SVTYPE=INS:ME:LINE1;END=139945092;SVLEN=415;AN=20618;AC=2;AF=9.700261907071492e-05;N_HET=2;N_HOMALT=0;N_HOMREF=10307;FREQ_HET=0.00019400523814142983;FREQ_HOMALT=0.0;FREQ_HOMREF=0.9998059947618586;N_BI_GENOS=10309;South_Han_AN=1496;South_Han_AC=2;South_Han
chr5	140834083	.	A	<INS:ME:LINE1>	.	PASS	SVTYPE=INS:ME:LINE1;END=140834094;SVLEN=6003;AN=20618;AC=2;AF=9.700261907071492e-05;N_HET=2;N_HOMALT=0;N_HOMREF=10307;FREQ_HET=0.00019400523814142983;FREQ_HOMALT=0.0;FREQ_HOMREF=0.9998059947618586;N_BI_GENOS=10309;Northwest_Han_AN=1296;Northwest_Han_AC=1;
chr5	141202804	.	C	<INS:ME:LINE1>	.	PASS	SVTYPE=INS:ME:LINE1;END=141202820;SVLEN=6000;AN=20618;AC=1;AF=4.850130953535746e-05;N_HET=1;N_HOMALT=0;N_HOMREF=10308;FREQ_HET=9.700261907071492e-05;FREQ_HOMALT=0.0;FREQ_HOMREF=0.9999029973809292;N_BI_GENOS=10309;Tibetan_AN=414;Tibetan_AC=1;Tibetan_AF=0.0
chr5	141760074	.	A	<INS:ME:LINE1>	.	PASS	SVTYPE=INS:ME:LINE1;END=141760090;SVLEN=566;AN=20618;AC=1;AF=4.850130953535746e-05;N_HET=1;N_HOMALT=0;N_HOMREF=10308;FREQ_HET=9.700261907071492e-05;FREQ_HOMALT=0.0;FREQ_HOMREF=0.9999029973809292;N_BI_GENOS=10309;East_Han_AN=6554;East_Han_AC=1;East_Han_AF=
chr5	142759915	.	A	<INS:ME:LINE1>	.	PASS	SVTYPE=INS:ME:LINE1;END=142759929;SVLEN=5997;AN=20618;AC=3;AF=0.0001455039286060 7236;N_HET=3;N_HOMALT=0;N_HOMREF=10306;FREQ_HET=0.0002910078572121447;FREQ_HOMALT=0.0;FREQ_HOMREF=0.9997089921427879;N_BI_GENOS=10309;East_Han_AN=6554;East_Han_AC=2;East_Han_A
chr5	142820294	.	G	<INS:ME:LINE1>	.	PASS	SVTYPE=INS:ME:LINE1;END=142820301;SVLEN=2659;AN=20618;AC=1;AF=4.850130953535746e-05;N_HET=1;N_HOMALT=0;N_HOMREF=10308;FREQ_HET=9.700261907071492e-05;FREQ_HOMALT=0.0;FREQ_HOMREF=0.9999029973809292;N_BI_GENOS=10309;East_Han_AN=6554;East_Han_AC=1;East_Han_AF
chr5	143306522	.	C	<INS:ME:LINE1>	.	PASS	SVTYPE=INS:ME:LINE1;END=143306538;SVLEN=732;AN=20618;AC=1;AF=4.850130953535746e-05;N_HET=1;N_HOMALT=0;N_HOMREF=10308;FREQ_HET=9.700261907071492e-05;FREQ_HOMALT=0.0;FREQ_HOMREF=0.9999029973809292;N_BI_GENOS=10309;Lingnan_Han_AN=1888;Lingnan_Han_AC=1;Lingna
chr5	143308977	.	G	<INS:ME:LINE1>	.	PASS	SVTYPE=INS:ME:LINE1;END=143308991;SVLEN=5998;AN=20618;AC=11;AF=0.0005335144048888 32;N_HET=11;N_HOMALT=0;N_HOMREF=10298;FREQ_HET=0.0010670288097777864;FREQ_HOMALT=0.0;FREQ_HOMREF=0.9989329711902222;N_BI_GENOS=10309;South_Han_AN=1496;South_Han_AC=1;South_Han
chr5	143656961	.	C	<INS:ME:LINE1>	.	PASS	SVTYPE=INS:ME:LINE1;END=143656961;SVLEN=1477;AN=20618;AC=1;AF=4.850130953535746e-05;N_HET=1;N_HOMALT=0;N_HOMREF=10308;FREQ_HET=9.700261907071492e-05;FREQ_HOMALT=0.0;FREQ_HOMREF=0.9999029973809292;N_BI_GENOS=10309;North_Han_AN=2272;North_Han_AC=1;North_Han
chr5	143806088	.	A	<INS:ME:LINE1>	.	PASS	SVTYPE=INS:ME:LINE1;END=143806102;SVLEN=5988;AN=20618;AC=1;AF=4.850130953535746e-05;N_HET=1;N_HOMALT=0;N_HOMREF=10308;FREQ_HET=9.700261907071492e-05;FREQ_HOMALT=0.0;FREQ_HOMREF=0.9999029973809292;N_BI_GENOS=10309;East_Han_AN=6554;East_Han_AC=1;East_Han_AF
chr5	143944818	.	A	<INS:ME:LINE1>	.	PASS	SVTYPE=INS:ME:LINE1;END=143944829;SVLEN=1371;AN=20618;AC=1;AF=4.850130953535746e-05;N_HET=1;N_HOMALT=0;N_HOMREF=10308;FREQ_HET=9.700261907071492e-05;FREQ_HOMALT=0.0;FREQ_HOMREF=0.9999029973809292;N_BI_GENOS=10309;East_Han_AN=6554;East_Han_AC=1;East_Han_AF
chr5	143944975	.	C	<INS:ME:LINE1>	.	PASS	SVTYPE=INS:ME:LINE1;END=143944975;SVLEN=1138;AN=20618;AC=1;AF=4.850130953535746e-05;N_HET=1;N_HOMALT=0;N_HOMREF=10308;FREQ_HET=9.700261907071492e-05;FREQ_HOMALT=0.0;FREQ_HOMREF=0.9999029973809292;N_BI_GENOS=10309;East_Han_AN=6554;East_Han_AC=1;East_Han_AF
chr5	143974435	.	G	<INS:ME:LINE1>	.	PASS	SVTYPE=INS:ME:LINE1;END=143974451;SVLEN=5997;AN=20618;AC=1;AF=4.850130953535746e-05;N_HET=1;N_HOMALT=0;N_HOMREF=10308;FREQ_HET=9.700261907071492e-05;FREQ_HOMALT=0.0;FREQ_HOMREF=0.9999029973809292;N_BI_GENOS=10309;Central_Han_AN=2128;Central_Han_AC=1;Centr
chr5	144034310	.	A	<INS:ME:LINE1>	.	PASS	SVTYPE=INS:ME:LINE1;END=144034324;SVLEN=5998;AN=20618;AC=794;AF=0.0385100397710738 2;N_HET=730;N_HOMALT=32;N_HOMREF=9547;FREQ_HET=0.07081191192162188;FREQ_HOMALT=0.0031040838102628773;FREQ_HOMREF=0.9260840042681152;N_BI_GENOS=10309;South_Han_AN=1496;South_
chr5	144062804	.	T	<INS:ME:LINE1>	.	PASS	SVTYPE=INS:ME:LINE1;END=144062804;SVLEN=232;AN=20618;AC=2;AF=9.700261907071492e-05;N_HET=2;N_HOMALT=0;N_HOMREF=10307;FREQ_HET=0.00019400523814142983;FREQ_HOMALT=0.0;FREQ_HOMREF=0.9998059947618586;N_BI_GENOS=10309;Northwest_Han_AN=1296;Northwest_Han_AC=1;N
chr5	144073215	.	G	<INS:ME:LINE1>	.	PASS	SVTYPE=INS:ME:LINE1;END=144073233;SVLEN=2565;AN=20618;AC=1;AF=4.850130953535746e-05;N_HET=1;N_HOMALT=0;N_HOMREF=10308;FREQ_HET=9.700261907071492e-05;FREQ_HOMALT=0.0;FREQ_HOMREF=0.9999029973809292;N_BI_GENOS=10309;Central_Han_AN=2128;Central_Han_AC=1;Centr
chr5	144102083	.	T	<INS:ME:LINE1>	.	PASS	SVTYPE=INS:ME:LINE1;END=144102098;SVLEN=5997;AN=20618;AC=1;AF=4.850130953535746e-05;N_HET=1;N_HOMALT=0;N_HOMREF=10308;FREQ_HET=9.700261907071492e-05;FREQ_HOMALT=0.0;FREQ_HOMREF=0.9999029973809292;N_BI_GENOS=10309;North_Han_AN=2272;North_Han_AC=1;North_Han
chr5	144214272	.	T	<INS:ME:LINE1>	.	PASS	SVTYPE=INS:ME:LINE1;END=144214289;SVLEN=381;AN=20618;AC=1;AF=4.850130953535746e-05;N_HET=1;N_HOMALT=0;N_HOMREF=10308;FREQ_HET=9.700261907071492e-05;FREQ_HOMALT=0.0;FREQ_HOMREF=0.9999029973809292;N_BI_GENOS=10309;East_Han_AN=6554;East_Han_AC=1;East_Han_AF=
chr5	144450280	.	A	<INS:ME:LINE1>	.	PASS	SVTYPE=INS:ME:LINE1;END=144450294;SVLEN=748;AN=20618;AC=5;AF=0.00024250654767678728;N_HET=3;N_HOMALT=1;N_HOMREF=10305;FREQ_HET=0.0002910078572121447;FREQ_HOMALT=9.700261907071492e-05;FREQ_HOMREF=0.9996119895237171;N_BI_GENOS=10309;Lingnan_Han_AN=1888;Ling
chr5	144494289	.	T	<INS:ME:LINE1>	.	PASS	SVTYPE=INS:ME:LINE1;END=144494289;SVLEN=433;AN=20618;AC=2;AF=9.700261907071492e-05;N_HET=0;N_HOMALT=1;N_HOMREF=10308;FREQ_HET=0.0;FREQ_HOMALT=9.700261907071492e-05;FREQ_HOMREF=0.9999029973809292;N_BI_GENOS=10309;East_Han_AN=6554;East_Han_AC=2;East_Han_AF=
chr5	144508754	.	T	<INS:ME:LINE1>	.	PASS	SVTYPE=INS:ME:LINE1;END=144508791;SVLEN=203;AN=20618;AC=1;AF=4.850130953535746e-05;N_HET=1;N_HOMALT=0;N_HOMREF=10308;FREQ_HET=9.700261907071492e-05;FREQ_HOMALT=0.0;FREQ_HOMREF=0.9999029973809292;N_BI_GENOS=10309;Lingnan_Han_AN=1888;Lingnan_Han_AC=1;Lingna
chr5	144853892	.	T	<INS:ME:LINE1>	.	PASS	SVTYPE=INS:ME:LINE1;END=144853897;SVLEN=792;AN=20618;AC=2;AF=9.700261907071492e-05;N_HET=2;N_HOMALT=0;N_HOMREF=10307;FREQ_HET=0.00019400523814142983;FREQ_HOMALT=0.0;FREQ_HOMREF=0.9998059947618586;N_BI_GENOS=10309;North_Han_AN=2272;North_Han_AC=2;North_Han
chr5	144854006	.	A	<INS:ME:LINE1>	.	PASS	SVTYPE=INS:ME:LINE1;END=144854021;SVLEN=1949;AN=20618;AC=10;AF=0.00048501309535357455;N_HET=10;N_HOMALT=0;N_HOMREF=10299;FREQ_HET=0.0009700261907071491;FREQ_HOMALT=0.0;FREQ_HOMREF=0.9990299738092928;N_BI_GENOS=10309;North_Han_AN=2272;North_Han_AC=1;North_
chr5	144902867	.	C	<INS:ME:LINE1>	.	PASS	SVTYPE=INS:ME:LINE1;END=144902886;SVLEN=1602;AN=20618;AC=2;AF=9.700261907071492e-05;N_HET=2;N_HOMALT=0;N_HOMREF=10307;FREQ_HET=0.00019400523814142983;FREQ_HOMALT=0.0;FREQ_HOMREF=0.9998059947618586;N_BI_GENOS=10309;Lingnan_Han_AN=1888;Lingnan_Han_AC=2;Ling
chr5	144951431	.	T	<INS:ME:LINE1>	.	PASS	SVTYPE=INS:ME:LINE1;END=144951450;SVLEN=849;AN=20618;AC=4378;AF=0.21233873314579493;N_HET=3150;N_HOMALT=614;N_HOMREF=6545;FREQ_HET=0.30555825007275195;FREQ_HOMALT=0.05955960810941895;FREQ_HOMREF=0.6348821418178291;N_BI_GENOS=10309;South_Han_AN=1496;South_
chr5	144990528	.	A	<INS:ME:LINE1>	.	PASS	SVTYPE=INS:ME:LINE1;END=144990528;SVLEN=716;AN=20618;AC=1;AF=4.850130953535746e-05;N_HET=1;N_HOMALT=0;N_HOMREF=10308;FREQ_HET=9.700261907071492e-05;FREQ_HOMALT=0.0;FREQ_HOMREF=0.9999029973809292;N_BI_GENOS=10309;East_Han_AN=6554;East_Han_AC=1;East_Han_AF=
chr5	145001948	.	A	<INS:ME:LINE1>	.	PASS	SVTYPE=INS:ME:LINE1;END=145001960;SVLEN=6011;AN=20618;AC=1;AF=4.850130953535746e-05;N_HET=1;N_HOMALT=0;N_HOMREF=10308;FREQ_HET=9.700261907071492e-05;FREQ_HOMALT=0.0;FREQ_HOMREF=0.9999029973809292;N_BI_GENOS=10309;Central_Han_AN=2128;Central_Han_AC=1;Centr
chr5	145169148	.	A	<INS:ME:LINE1>	.	PASS	SVTYPE=INS:ME:LINE1;END=145169169;SVLEN=897;AN=20618;AC=1;AF=4.850130953535746e-05;N_HET=1;N_HOMALT=0;N_HOMREF=10308;FREQ_HET=9.700261907071492e-05;FREQ_HOMALT=0.0;FREQ_HOMREF=0.9999029973809292;N_BI_GENOS=10309;North_Han_AN=2272;North_Han_AC=1;North_Han_
chr5	145202792	.	A	<INS:ME:LINE1>	.	PASS	SVTYPE=INS:ME:LINE1;END=145202809;SVLEN=1677;AN=20618;AC=1;AF=4.850130953535746e-05;N_HET=1;N_HOMALT=0;N_HOMREF=10308;FREQ_HET=9.700261907071492e-05;FREQ_HOMALT=0.0;FREQ_HOMREF=0.9999029973809292;N_BI_GENOS=10309;Central_Han_AN=2128;Central_Han_AC=1;Centr
chr5	145344213	.	T	<INS:ME:LINE1>	.	PASS	SVTYPE=INS:ME:LINE1;END=145344213;SVLEN=201;AN=20618;AC=1;AF=4.850130953535746e-05;N_HET=1;N_HOMALT=0;N_HOMREF=10308;FREQ_HET=9.700261907071492e-05;FREQ_HOMALT=0.0;FREQ_HOMREF=0.9999029973809292;N_BI_GENOS=10309;East_Han_AN=6554;East_Han_AC=1;East_Han_AF=
chr5	145447002	.	C	<INS:ME:LINE1>	.	PASS	SVTYPE=INS:ME:LINE1;END=145447050;SVLEN=5434;AN=20618;AC=1;AF=4.850130953535746e-05;N_HET=1;N_HOMALT=0;N_HOMREF=10308;FREQ_HET=9.700261907071492e-05;FREQ_HOMALT=0.0;FREQ_HOMREF=0.9999029973809292;N_BI_GENOS=10309;East_Han_AN=6554;East_Han_AC=1;East_Han_AF

chr5	145479892	.	G	<INS:ME:LINE1>	.	PASS	SVTYPE=INS:ME:LINE1;END=145479900;SVLEN=506;AN=20618;AC=4;AF=0.00019400523814142983;N_HET=2;N_HOMALT=1;N_HOMREF=10306;FREQ_HET=0.00019400523814142983;FREQ_HOMALT=9.700261907071492e-05;FREQ_HOMREF=0.9997089921427879;N_BI_GENOS=10309;Lingnan_Han_AN=1888;Lin
chr5	145517332	.	T	<INS:ME:LINE1>	.	PASS	SVTYPE=INS:ME:LINE1;END=145517350;SVLEN=778;AN=20618;AC=1;AF=4.850130953535746e-05;N_HET=1;N_HOMALT=0;N_HOMREF=10308;FREQ_HET=9.700261907071492e-05;FREQ_HOMALT=0.0;FREQ_HOMREF=0.9999029973809292;N_BI_GENOS=10309;East_Han_AN=6554;East_Han_AC=1;East_Han_AF=
chr5	145701672	.	A	<INS:ME:LINE1>	.	PASS	SVTYPE=INS:ME:LINE1;END=145701686;SVLEN=586;AN=20618;AC=1;AF=4.850130953535746e-05;N_HET=1;N_HOMALT=0;N_HOMREF=10308;FREQ_HET=9.700261907071492e-05;FREQ_HOMALT=0.0;FREQ_HOMREF=0.9999029973809292;N_BI_GENOS=10309;East_Han_AN=6554;East_Han_AC=1;East_Han_AF=
chr5	146172108	.	A	<INS:ME:LINE1>	.	PASS	SVTYPE=INS:ME:LINE1;END=146172124;SVLEN=6026;AN=20618;AC=1;AF=4.850130953535746e-05;N_HET=1;N_HOMALT=0;N_HOMREF=10308;FREQ_HET=9.700261907071492e-05;FREQ_HOMALT=0.0;FREQ_HOMREF=0.9999029973809292;N_BI_GENOS=10309;East_Han_AN=6554;East_Han_AC=1;East_Han_AF
chr5	146286068	.	T	<INS:ME:LINE1>	.	PASS	SVTYPE=INS:ME:LINE1;END=146286068;SVLEN=159;AN=20618;AC=1;AF=4.850130953535746e-05;N_HET=1;N_HOMALT=0;N_HOMREF=10308;FREQ_HET=9.700261907071492e-05;FREQ_HOMALT=0.0;FREQ_HOMREF=0.9999029973809292;N_BI_GENOS=10309;East_Han_AN=6554;East_Han_AC=1;East_Han_AF=
chr5	146974318	.	A	<INS:ME:LINE1>	.	PASS	SVTYPE=INS:ME:LINE1;END=146974329;SVLEN=3522;AN=20618;AC=1;AF=4.850130953535746e-05;N_HET=1;N_HOMALT=0;N_HOMREF=10308;FREQ_HET=9.700261907071492e-05;FREQ_HOMALT=0.0;FREQ_HOMREF=0.9999029973809292;N_BI_GENOS=10309;Manchu_AN=416;Manchu_AC=1;Manchu_AF=0.0024
chr5	147267889	.	G	<INS:ME:LINE1>	.	PASS	SVTYPE=INS:ME:LINE1;END=147267907;SVLEN=1215;AN=20618;AC=1;AF=4.850130953535746e-05;N_HET=1;N_HOMALT=0;N_HOMREF=10308;FREQ_HET=9.700261907071492e-05;FREQ_HOMALT=0.0;FREQ_HOMREF=0.9999029973809292;N_BI_GENOS=10309;Mongolian_AN=454;Mongolian_AC=1;Mongolian_
chr5	147330159	.	A	<INS:ME:LINE1>	.	PASS	SVTYPE=INS:ME:LINE1;END=147330173;SVLEN=5998;AN=20618;AC=1;AF=4.850130953535746e-05;N_HET=1;N_HOMALT=0;N_HOMREF=10308;FREQ_HET=9.700261907071492e-05;FREQ_HOMALT=0.0;FREQ_HOMREF=0.9999029973809292;N_BI_GENOS=10309;Lingnan_Han_AN=1888;Lingnan_Han_AC=1;Lingn
chr5	147778862	.	A	<INS:ME:LINE1>	.	PASS	SVTYPE=INS:ME:LINE1;END=147778875;SVLEN=6000;AN=20618;AC=1;AF=4.850130953535746e-05;N_HET=1;N_HOMALT=0;N_HOMREF=10308;FREQ_HET=9.700261907071492e-05;FREQ_HOMALT=0.0;FREQ_HOMREF=0.9999029973809292;N_BI_GENOS=10309;Central_Han_AN=2128;Central_Han_AC=1;Centr
chr5	147811379	.	A	<INS:ME:LINE1>	.	PASS	SVTYPE=INS:ME:LINE1;END=147811392;SVLEN=1178;AN=20618;AC=55;AF=0.00266757202444466;N_HET=49;N_HOMALT=3;N_HOMREF=10257;FREQ_HET=0.004753128334465031;FREQ_HOMALT=0.0002910078572121447;FREQ_HOMREF=0.9949558638083228;N_BI_GENOS=10309;South_Han_AN=1496;South_H
chr5	147868866	.	C	<INS:ME:LINE1>	.	PASS	SVTYPE=INS:ME:LINE1;END=147868866;SVLEN=0;AN=20618;AC=2;AF=9.700261907071492e-05;N_HET=0;N_HOMALT=1;N_HOMREF=10308;FREQ_HET=0.0;FREQ_HOMALT=9.700261907071492e-05;FREQ_HOMREF=0.9999029973809292;N_BI_GENOS=10309;Northwest_Han_AN=1296;Northwest_Han_AC=2;Nort
chr5	147872877	.	G	<INS:ME:LINE1>	.	PASS	SVTYPE=INS:ME:LINE1;END=147872877;SVLEN=0;AN=20618;AC=2;AF=9.700261907071492e-05;N_HET=2;N_HOMALT=0;N_HOMREF=10307;FREQ_HET=0.00019400523814142983;FREQ_HOMALT=0.0;FREQ_HOMREF=0.9998059947618586;N_BI_GENOS=10309;East_Han_AN=6554;East_Han_AC=2;East_Han_AF=0
chr5	147883849	.	T	<INS:ME:LINE1>	.	PASS	SVTYPE=INS:ME:LINE1;END=147883862;SVLEN=32;AN=20618;AC=138;AF=0.006693180715879329;N_HET=104;N_HOMALT=17;N_HOMREF=10188;FREQ_HET=0.01008827238335435;FREQ_HOMALT=0.0016490445242021534;FREQ_HOMREF=0.9882626830924435;N_BI_GENOS=10309;South_Han_AN=1496;South_
chr5	148040279	.	G	<INS:ME:LINE1>	.	PASS	SVTYPE=INS:ME:LINE1;END=148040296;SVLEN=5993;AN=20618;AC=1;AF=4.850130953535746e-05;N_HET=1;N_HOMALT=0;N_HOMREF=10308;FREQ_HET=9.700261907071492e-05;FREQ_HOMALT=0.0;FREQ_HOMREF=0.9999029973809292;N_BI_GENOS=10309;Central_Han_AN=2128;Central_Han_AC=1;Centr
chr5	148089120	.	C	<INS:ME:LINE1>	.	PASS	SVTYPE=INS:ME:LINE1;END=148089134;SVLEN=581;AN=20618;AC=1;AF=4.850130953535746e-05;N_HET=1;N_HOMALT=0;N_HOMREF=10308;FREQ_HET=9.700261907071492e-05;FREQ_HOMALT=0.0;FREQ_HOMREF=0.9999029973809292;N_BI_GENOS=10309;Southeast_Han_AN=1908;Southeast_Han_AC=1;So
chr5	148202752	.	A	<INS:ME:LINE1>	.	PASS	SVTYPE=INS:ME:LINE1;END=148202762;SVLEN=3026;AN=20618;AC=3;AF=0.00014550392860607236;N_HET=3;N_HOMALT=0;N_HOMREF=10306;FREQ_HET=0.0002910078572121447;FREQ_HOMALT=0.0;FREQ_HOMREF=0.9997089921427879;N_BI_GENOS=10309;East_Han_AN=6554;East_Han_AC=2;East_Han_A
chr5	148289466	.	A	<INS:ME:LINE1>	.	PASS	SVTYPE=INS:ME:LINE1;END=148289482;SVLEN=6009;AN=20618;AC=2;AF=9.700261907071492e-05;N_HET=2;N_HOMALT=0;N_HOMREF=10307;FREQ_HET=0.00019400523814142983;FREQ_HOMALT=0.0;FREQ_HOMREF=0.9998059947618586;N_BI_GENOS=10309;Lingnan_Han_AN=1888;Lingnan_Han_AC=2;Ling
chr5	148462031	.	T	<INS:ME:LINE1>	.	PASS	SVTYPE=INS:ME:LINE1;END=148462032;SVLEN=974;AN=20618;AC=1;AF=4.850130953535746e-05;N_HET=1;N_HOMALT=0;N_HOMREF=10308;FREQ_HET=9.700261907071492e-05;FREQ_HOMALT=0.0;FREQ_HOMREF=0.9999029973809292;N_BI_GENOS=10309;South_Han_AN=1496;South_Han_AC=1;South_Han_
chr5	148711190	.	G	<INS:ME:LINE1>	.	PASS	SVTYPE=INS:ME:LINE1;END=148711205;SVLEN=1606;AN=20618;AC=1;AF=4.850130953535746e-05;N_HET=1;N_HOMALT=0;N_HOMREF=10308;FREQ_HET=9.700261907071492e-05;FREQ_HOMALT=0.0;FREQ_HOMREF=0.9999029973809292;N_BI_GENOS=10309;Lingnan_Han_AN=1888;Lingnan_Han_AC=1;Lingn
chr5	148734681	.	C	<INS:ME:LINE1>	.	PASS	SVTYPE=INS:ME:LINE1;END=148734698;SVLEN=5997;AN=20618;AC=1;AF=4.850130953535746e-05;N_HET=1;N_HOMALT=0;N_HOMREF=10308;FREQ_HET=9.700261907071492e-05;FREQ_HOMALT=0.0;FREQ_HOMREF=0.9999029973809292;N_BI_GENOS=10309;Northwest_Han_AN=1296;Northwest_Han_AC=1;N
chr5	148859042	.	A	<INS:ME:LINE1>	.	PASS	SVTYPE=INS:ME:LINE1;END=148859056;SVLEN=5986;AN=20618;AC=1;AF=4.850130953535746e-05;N_HET=1;N_HOMALT=0;N_HOMREF=10308;FREQ_HET=9.700261907071492e-05;FREQ_HOMALT=0.0;FREQ_HOMREF=0.9999029973809292;N_BI_GENOS=10309;Lingnan_Han_AN=1888;Lingnan_Han_AC=1;Lingn
chr5	149013549	.	A	<INS:ME:LINE1>	.	PASS	SVTYPE=INS:ME:LINE1;END=149013564;SVLEN=5993;AN=20618;AC=3;AF=0.00014550392860607236;N_HET=3;N_HOMALT=0;N_HOMREF=10306;FREQ_HET=0.0002910078572121447;FREQ_HOMALT=0.0;FREQ_HOMREF=0.9997089921427879;N_BI_GENOS=10309;North_Han_AN=2272;North_Han_AC=1;North_Ha
chr5	150846872	.	A	<INS:ME:LINE1>	.	PASS	SVTYPE=INS:ME:LINE1;END=150846872;SVLEN=5996;AN=20618;AC=1;AF=4.850130953535746e-05;N_HET=1;N_HOMALT=0;N_HOMREF=10308;FREQ_HET=9.700261907071492e-05;FREQ_HOMALT=0.0;FREQ_HOMREF=0.9999029973809292;N_BI_GENOS=10309;North_Han_AN=2272;North_Han_AC=1;North_Han
chr5	150888473	.	A	<INS:ME:LINE1>	.	PASS	SVTYPE=INS:ME:LINE1;END=150888489;SVLEN=6025;AN=20618;AC=1;AF=4.850130953535746e-05;N_HET=1;N_HOMALT=0;N_HOMREF=10308;FREQ_HET=9.700261907071492e-05;FREQ_HOMALT=0.0;FREQ_HOMREF=0.9999029973809292;N_BI_GENOS=10309;Southeast_Han_AN=1908;Southeast_Han_AC=1;S
chr5	150918597	.	C	<INS:ME:LINE1>	.	PASS	SVTYPE=INS:ME:LINE1;END=150918610;SVLEN=1841;AN=20618;AC=2;AF=9.700261907071492e-05;N_HET=2;N_HOMALT=0;N_HOMREF=10307;FREQ_HET=0.00019400523814142983;FREQ_HOMALT=0.0;FREQ_HOMREF=0.9998059947618586;N_BI_GENOS=10309;Lingnan_Han_AN=1888;Lingnan_Han_AC=1;Ling
chr5	151504698	.	A	<INS:ME:LINE1>	.	PASS	SVTYPE=INS:ME:LINE1;END=151504715;SVLEN=789;AN=20618;AC=1;AF=4.850130953535746e-05;N_HET=1;N_HOMALT=0;N_HOMREF=10308;FREQ_HET=9.700261907071492e-05;FREQ_HOMALT=0.0;FREQ_HOMREF=0.9999029973809292;N_BI_GENOS=10309;East_Han_AN=6554;East_Han_AC=1;East_Han_AF=
chr5	151958210	.	T	<INS:ME:LINE1>	.	PASS	SVTYPE=INS:ME:LINE1;END=151958220;SVLEN=206;AN=20618;AC=1;AF=4.850130953535746e-05;N_HET=1;N_HOMALT=0;N_HOMREF=10308;FREQ_HET=9.700261907071492e-05;FREQ_HOMALT=0.0;FREQ_HOMREF=0.9999029973809292;N_BI_GENOS=10309;Central_Han_AN=2128;Central_Han_AC=1;Centra
chr5	152085067	.	G	<INS:ME:LINE1>	.	PASS	SVTYPE=INS:ME:LINE1;END=152085079;SVLEN=6000;AN=20618;AC=2;AF=9.700261907071492e-05;N_HET=2;N_HOMALT=0;N_HOMREF=10307;FREQ_HET=0.00019400523814142983;FREQ_HOMALT=0.0;FREQ_HOMREF=0.9998059947618586;N_BI_GENOS=10309;Southeast_Han_AN=1908;Southeast_Han_AC=2;
chr5	152257413	.	A	<INS:ME:LINE1>	.	PASS	SVTYPE=INS:ME:LINE1;END=152257430;SVLEN=939;AN=20618;AC=5;AF=0.00024250654767678728;N_HET=5;N_HOMALT=0;N_HOMREF=10304;FREQ_HET=0.00048501309535357465;FREQ_HOMALT=0.0;FREQ_HOMREF=0.9995149869046465;N_BI_GENOS=10309;South_Han_AN=1496;South_Han_AC=2;South_Ha
chr5	153224371	.	T	<INS:ME:LINE1>	.	PASS	SVTYPE=INS:ME:LINE1;END=153224387;SVLEN=414;AN=20618;AC=1;AF=4.850130953535746e-05;N_HET=1;N_HOMALT=0;N_HOMREF=10308;FREQ_HET=9.700261907071492e-05;FREQ_HOMALT=0.0;FREQ_HOMREF=0.9999029973809292;N_BI_GENOS=10309;East_Han_AN=6554;East_Han_AC=1;East_Han_AF=
chr5	153262362	.	A	<INS:ME:LINE1>	.	PASS	SVTYPE=INS:ME:LINE1;END=153262376;SVLEN=415;AN=20618;AC=1;AF=4.850130953535746e-05;N_HET=1;N_HOMALT=0;N_HOMREF=10308;FREQ_HET=9.700261907071492e-05;FREQ_HOMALT=0.0;FREQ_HOMREF=0.9999029973809292;N_BI_GENOS=10309;East_Han_AN=6554;East_Han_AC=1;East_Han_AF=
chr5	153423786	.	A	<INS:ME:LINE1>	.	PASS	SVTYPE=INS:ME:LINE1;END=153423803;SVLEN=6023;AN=20618;AC=4;AF=0.00019400523814142983;N_HET=4;N_HOMALT=0;N_HOMREF=10305;FREQ_HET=0.00038801047628285966;FREQ_HOMALT=0.0;FREQ_HOMREF=0.9996119895237171;N_BI_GENOS=10309;Northwest_Han_AN=1296;Northwest_Han_AC=1
chr5	155093490	.	A	<INS:ME:LINE1>	.	PASS	SVTYPE=INS:ME:LINE1;END=155093507;SVLEN=4298;AN=20618;AC=1;AF=4.850130953535746e-05;N_HET=1;N_HOMALT=0;N_HOMREF=10308;FREQ_HET=9.700261907071492e-05;FREQ_HOMALT=0.0;FREQ_HOMREF=0.9999029973809292;N_BI_GENOS=10309;Southeast_Han_AN=1908;Southeast_Han_AC=1;S
chr5	155369907	.	A	<INS:ME:LINE1>	.	PASS	SVTYPE=INS:ME:LINE1;END=155369922;SVLEN=5247;AN=20618;AC=1;AF=4.850130953535746e-05;N_HET=1;N_HOMALT=0;N_HOMREF=10308;FREQ_HET=9.700261907071492e-05;FREQ_HOMALT=0.0;FREQ_HOMREF=0.9999029973809292;N_BI_GENOS=10309;Lingnan_Han_AN=1888;Lingnan_Han_AC=1;Lingn
chr5	155413211	.	T	<INS:ME:LINE1>	.	PASS	SVTYPE=INS:ME:LINE1;END=155413227;SVLEN=443;AN=20618;AC=1;AF=4.850130953535746e-05;N_HET=1;N_HOMALT=0;N_HOMREF=10308;FREQ_HET=9.700261907071492e-05;FREQ_HOMALT=0.0;FREQ_HOMREF=0.9999029973809292;N_BI_GENOS=10309;Southeast_Han_AN=1908;Southeast_Han_AC=1;So
chr5	155494372	.	A	<INS:ME:LINE1>	.	PASS	SVTYPE=INS:ME:LINE1;END=155494377;SVLEN=74;AN=20618;AC=7223;AF=0.3503249587738869;N_HET=3831;N_HOMALT=1696;N_HOMREF=4782;FREQ_HET=0.37161703365990884;FREQ_HOMALT=0.16451644194393247;FREQ_HOMREF=0.4638665243961587;N_BI_GENOS=10309;South_Han_AN=1496;South_H
chr5	155539046	.	G	<INS:ME:LINE1>	.	PASS	SVTYPE=INS:ME:LINE1;END=155539062;SVLEN=6002;AN=20618;AC=8;AF=0.00038801047628285966;N_HET=8;N_HOMALT=0;N_HOMREF=10301;FREQ_HET=0.0007760209525657193;FREQ_HOMALT=0.0;FREQ_HOMREF=0.9992239790474343;N_BI_GENOS=10309;South_Han_AN=1496;South_Han_AC=1;South_Ha
chr5	155652156	.	T	<INS:ME:LINE1>	.	PASS	SVTYPE=INS:ME:LINE1;END=155652166;SVLEN=414;AN=20618;AC=1;AF=4.850130953535746e-05;N_HET=1;N_HOMALT=0;N_HOMREF=10308;FREQ_HET=9.700261907071492e-05;FREQ_HOMALT=0.0;FREQ_HOMREF=0.9999029973809292;N_BI_GENOS=10309;Central_Han_AN=2128;Central_Han_AC=1;Centra
chr5	155782551	.	T	<INS:ME:LINE1>	.	PASS	SVTYPE=INS:ME:LINE1;END=155782569;SVLEN=770;AN=20618;AC=2;AF=9.700261907071492e-05;N_HET=2;N_HOMALT=0;N_HOMREF=10307;FREQ_HET=0.00019400523814142983;FREQ_HOMALT=0.0;FREQ_HOMREF=0.9998059947618586;N_BI_GENOS=10309;South_Han_AN=1496;South_Han_AC=1;South_Han
chr5	155788141	.	T	<INS:ME:LINE1>	.	PASS	SVTYPE=INS:ME:LINE1;END=155788148;SVLEN=747;AN=20618;AC=2;AF=9.700261907071492e-05;N_HET=2;N_HOMALT=0;N_HOMREF=10307;FREQ_HET=0.00019400523814142983;FREQ_HOMALT=0.0;FREQ_HOMREF=0.9998059947618586;N_BI_GENOS=10309;East_Han_AN=6554;East_Han_AC=1;East_Han_AF
chr5	155822284	.	A	<INS:ME:LINE1>	.	PASS	SVTYPE=INS:ME:LINE1;END=155822300;SVLEN=923;AN=20618;AC=2;AF=9.700261907071492e-05;N_HET=2;N_HOMALT=0;N_HOMREF=10307;FREQ_HET=0.00019400523814142983;FREQ_HOMALT=0.0;FREQ_HOMREF=0.9998059947618586;N_BI_GENOS=10309;Northwest_Han_AN=1296;Northwest_Han_AC=1;N
chr5	155848901	.	A	<INS:ME:LINE1>	.	PASS	SVTYPE=INS:ME:LINE1;END=155848917;SVLEN=6007;AN=20618;AC=1;AF=4.850130953535746e-05;N_HET=1;N_HOMALT=0;N_HOMREF=10308;FREQ_HET=9.700261907071492e-05;FREQ_HOMALT=0.0;FREQ_HOMREF=0.9999029973809292;N_BI_GENOS=10309;North_Han_AN=2272;North_Han_AC=1;North_Han
chr5	155906898	.	A	<INS:ME:LINE1>	.	PASS	SVTYPE=INS:ME:LINE1;END=155906898;SVLEN=2236;AN=20618;AC=1508;AF=0.07313997477931904;N_HET=1108;N_HOMALT=200;N_HOMREF=9001;FREQ_HET=0.10747890193035212;FREQ_HOMALT=0.01940052381414298;FREQ_HOMREF=0.8731205742555049;N_BI_GENOS=10309;South_Han_AN=1496;South
chr5	155907080	.	G	<INS:ME:LINE1>	.	PASS	SVTYPE=INS:ME:LINE1;END=155907080;SVLEN=2257;AN=20618;AC=14;AF=0.0006790183334950044;N_HET=14;N_HOMALT=0;N_HOMREF=10295;FREQ_HET=0.001358036666900088;FREQ_HOMALT=0.0;FREQ_HOMREF=0.9986419633333301;N_BI_GENOS=10309;South_Han_AN=1496;South_Han_AC=1;South_Han
chr5	155966596	.	T	<INS:ME:LINE1>	.	PASS	SVTYPE=INS:ME:LINE1;END=155966596;SVLEN=66;AN=20618;AC=1;AF=4.850130953535746e-05;N_HET=1;N_HOMALT=0;N_HOMREF=10308;FREQ_HET=9.700261907071492e-05;FREQ_HOMALT=0.0;FREQ_HOMREF=0.9999029973809292;N_BI_GENOS=10309;Central_Han_AN=2128;Central_Han_AC=1;Central
chr5	156128403	.	G	<INS:ME:LINE1>	.	PASS	SVTYPE=INS:ME:LINE1;END=156128419;SVLEN=1791;AN=20618;AC=1;AF=4.850130953535746e-05;N_HET=1;N_HOMALT=0;N_HOMREF=10308;FREQ_HET=9.700261907071492e-05;FREQ_HOMALT=0.0;FREQ_HOMREF=0.9999029973809292;N_BI_GENOS=10309;Yi_AN=422;Yi_AC=1;Yi_AF=0.0023696682464454
chr5	156158421	.	G	<INS:ME:LINE1>	.	PASS	SVTYPE=INS:ME:LINE1;END=156158438;SVLEN=72;AN=20618;AC=1;AF=4.850130953535746e-05;N_HET=1;N_HOMALT=0;N_HOMREF=10308;FREQ_HET=9.700261907071492e-05;FREQ_HOMALT=0.0;FREQ_HOMREF=0.9999029973809292;N_BI_GENOS=10309;Mongolian_AN=454;Mongolian_AC=1;Mongolian_AF
chr5	156192854	.	A	<INS:ME:LINE1>	.	PASS	SVTYPE=INS:ME:LINE1;END=156192868;SVLEN=1353;AN=20618;AC=9674;AF=0.469201668445048;N_HET=2784;N_HOMALT=3445;N_HOMREF=4080;FREQ_HET=0.27005529149287033;FREQ_HOMALT=0.33417402269861285;FREQ_HOMREF=0.3957706858085168;N_BI_GENOS=10309;South_Han_AN=1496;South_
chr5	156457211	.	A	<INS:ME:LINE1>	.	PASS	SVTYPE=INS:ME:LINE1;END=156457223;SVLEN=6009;AN=20618;AC=3;AF=0.00014550392860607236;N_HET=3;N_HOMALT=0;N_HOMREF=10306;FREQ_HET=0.0002910078572121447;FREQ_HOMALT=0.0;FREQ_HOMREF=0.9997089921427879;N_BI_GENOS=10309;North_Han_AN=2272;North_Han_AC=1;North_Ha
chr5	156524796	.	G	<INS:ME:LINE1>	.	PASS	SVTYPE=INS:ME:LINE1;END=156524812;SVLEN=6005;AN=20618;AC=1;AF=4.850130953535746e-05;N_HET=1;N_HOMALT=0;N_HOMREF=10308;FREQ_HET=9.700261907071492e-05;FREQ_HOMALT=0.0;FREQ_HOMREF=0.9999029973809292;N_BI_GENOS=10309;South_Han_AN=1496;South_Han_AC=1;South_Han
chr5	156527510	.	A	<INS:ME:LINE1>	.	PASS	SVTYPE=INS:ME:LINE1;END=156527523;SVLEN=1413;AN=20618;AC=2;AF=9.700261907071492e-05;N_HET=2;N_HOMALT=0;N_HOMREF=10307;FREQ_HET=0.00019400523814142983;FREQ_HOMALT=0.0;FREQ_HOMREF=0.9998059947618586;N_BI_GENOS=10309;Northwest_Han_AN=1296;Northwest_Han_AC=1;
chr5	156629844	.	G	<INS:ME:LINE1>	.	PASS	SVTYPE=INS:ME:LINE1;END=156629879;SVLEN=762;AN=20618;AC=1;AF=4.850130953535746e-05;N_HET=1;N_HOMALT=0;N_HOMREF=10308;FREQ_HET=9.700261907071492e-05;FREQ_HOMALT=0.0;FREQ_HOMREF=0.9999029973809292;N_BI_GENOS=10309;Southeast_Han_AN=1908;Southeast_Han_AC=1;So
chr5	156644658	.	A	<INS:ME:LINE1>	.	PASS	SVTYPE=INS:ME:LINE1;END=156644672;SVLEN=6032;AN=20618;AC=1;AF=4.850130953535746e-05;N_HET=1;N_HOMALT=0;N_HOMREF=10308;FREQ_HET=9.700261907071492e-05;FREQ_HOMALT=0.0;FREQ_HOMREF=0.9999029973809292;N_BI_GENOS=10309;Lingnan_Han_AN=1888;Lingnan_Han_AC=1;Lingn
chr5	157009494	.	A	<INS:ME:LINE1>	.	PASS	SVTYPE=INS:ME:LINE1;END=157009496;SVLEN=707;AN=20618;AC=1;AF=4.850130953535746e-05;N_HET=1;N_HOMALT=0;N_HOMREF=10308;FREQ_HET=9.700261907071492e-05;FREQ_HOMALT=0.0;FREQ_HOMREF=0.9999029973809292;N_BI_GENOS=10309;South_Han_AN=1496;South_Han_AC=1;South_Han_
chr5	157090041	.	T	<INS:ME:LINE1>	.	PASS	SVTYPE=INS:ME:LINE1;END=157090041;SVLEN=1740;AN=20618;AC=1;AF=4.850130953535746e-05;N_HET=1;N_HOMALT=0;N_HOMREF=10308;FREQ_HET=9.700261907071492e-05;FREQ_HOMALT=0.0;FREQ_HOMREF=0.9999029973809292;N_BI_GENOS=10309;East_Han_AN=6554;East_Han_AC=1;East_Han_AF
chr5	157172221	.	T	<INS:ME:LINE1>	.	PASS	SVTYPE=INS:ME:LINE1;END=157172237;SVLEN=1808;AN=20618;AC=8;AF=0.00038801047628285966;N_HET=8;N_HOMALT=0;N_HOMREF=10301;FREQ_HET=0.0007760209525657193;FREQ_HOMALT=0.0;FREQ_HOMREF=0.9992239790474343;N_BI_GENOS=10309;South_Han_AN=1496;South_Han_AC=1;South_Ha
chr5	157990064	.	C	<INS:ME:LINE1>	.	PASS	SVTYPE=INS:ME:LINE1;END=157990077;SVLEN=6014;AN=20618;AC=2;AF=9.700261907071492e-05;N_HET=2;N_HOMALT=0;N_HOMREF=10307;FREQ_HET=0.00019400523814142983;FREQ_HOMALT=0.0;FREQ_HOMREF=0.9998059947618586;N_BI_GENOS=10309;North_Han_AN=2272;North_Han_AC=1;North_Ha
chr5	158440304	.	A	<INS:ME:LINE1>	.	PASS	SVTYPE=INS:ME:LINE1;END=158440305;SVLEN=4865;AN=20618;AC=8;AF=0.00038801047628285966;N_HET=6;N_HOMALT=1;N_HOMREF=10302;FREQ_HET=0.0005820157144242894;FREQ_HOMALT=9.700261907071492e-05;FREQ_HOMREF=0.999320981666505;N_BI_GENOS=10309;Central_Han_AN=2128;Cent
chr5	158770614	.	G	<INS:ME:LINE1>	.	PASS	SVTYPE=INS:ME:LINE1;END=158770614;SVLEN=512;AN=20618;AC=1;AF=4.850130953535746e-05;N_HET=1;N_HOMALT=0;N_HOMREF=10308;FREQ_HET=9.700261907071492e-05;FREQ_HOMALT=0.0;FREQ_HOMREF=0.9999029973809292;N_BI_GENOS=10309;East_Han_AN=6554;East_Han_AC=1;East_Han_AF=
chr5	158965641	.	A	<INS:ME:LINE1>	.	PASS	SVTYPE=INS:ME:LINE1;END=158965654;SVLEN=6006;AN=20618;AC=2;AF=9.700261907071492e-05;N_HET=2;N_HOMALT=0;N_HOMREF=10307;FREQ_HET=0.00019400523814142983;FREQ_HOMALT=0.0;FREQ_HOMREF=0.9998059947618586;N_BI_GENOS=10309;Lingnan_Han_AN=1888;Lingnan_Han_AC=2;Ling
chr5	159171597	.	C	<INS:ME:LINE1>	.	PASS	SVTYPE=INS:ME:LINE1;END=159171616;SVLEN=1023;AN=20618;AC=1;AF=4.850130953535746e-05;N_HET=1;N_HOMALT=0;N_HOMREF=10308;FREQ_HET=9.700261907071492e-05;FREQ_HOMALT=0.0;FREQ_HOMREF=0.9999029973809292;N_BI_GENOS=10309;Northwest_Han_AN=1296;Northwest_Han_AC=1;N
chr5	159366385	.	G	<INS:ME:LINE1>	.	PASS	SVTYPE=INS:ME:LINE1;END=159366401;SVLEN=6006;AN=20618;AC=1;AF=4.850130953535746e-05;N_HET=1;N_HOMALT=0;N_HOMREF=10308;FREQ_HET=9.700261907071492e-05;FREQ_HOMALT=0.0;FREQ_HOMREF=0.9999029973809292;N_BI_GENOS=10309;Central_Han_AN=2128;Central_Han_AC=1;Centr
chr5	159924192	.	C	<INS:ME:LINE1>	.	PASS	SVTYPE=INS:ME:LINE1;END=159924192;SVLEN=3809;AN=20618;AC=23;AF=0.0011155301193132214;N_HET=23;N_HOMALT=0;N_HOMREF=10286;FREQ_HET=0.002231060238626443;FREQ_HOMALT=0.0;FREQ_HOMREF=0.9977689397613736;N_BI_GENOS=10309;South_Han_AN=1496;South_Han_AC=2;South_Ha
chr5	160522787	.	A	<INS:ME:LINE1>	.	PASS	SVTYPE=INS:ME:LINE1;END=160522787;SVLEN=0;AN=20618;AC=2;AF=9.700261907071492e-05;N_HET=0;N_HOMALT=1;N_HOMREF=10308;FREQ_HET=0.0;FREQ_HOMALT=9.700261907071492e-05;FREQ_HOMREF=0.9999029973809292;N_BI_GENOS=10309;Central_Han_AN=2128;Central_Han_AC=2;Central_
chr5	160843195	.	A	<INS:ME:LINE1>	.	PASS	SVTYPE=INS:ME:LINE1;END=160843195;SVLEN=2119;AN=20618;AC=1;AF=4.850130953535746e-05;N_HET=1;N_HOMALT=0;N_HOMREF=10308;FREQ_HET=9.700261907071492e-05;FREQ_HOMALT=0.0;FREQ_HOMREF=0.9999029973809292;N_BI_GENOS=10309;South_Han_AN=1496;South_Han_AC=1;South_Han
chr5	160858640	.	T	<INS:ME:LINE1>	.	PASS	SVTYPE=INS:ME:LINE1;END=160858646;SVLEN=233;AN=20618;AC=1;AF=4.850130953535746e-05;N_HET=1;N_HOMALT=0;N_HOMREF=10308;FREQ_HET=9.700261907071492e-05;FREQ_HOMALT=0.0;FREQ_HOMREF=0.9999029973809292;N_BI_GENOS=10309;Tibetan_AN=414;Tibetan_AC=1;Tibetan_AF=0.00
chr5	160988982	.	T	<INS:ME:LINE1>	.	PASS	SVTYPE=INS:ME:LINE1;END=160988996;SVLEN=5997;AN=20618;AC=1;AF=4.850130953535746e-05;N_HET=1;N_HOMALT=0;N_HOMREF=10308;FREQ_HET=9.700261907071492e-05;FREQ_HOMALT=0.0;FREQ_HOMREF=0.9999029973809292;N_BI_GENOS=10309;Northwest_Han_AN=1296;Northwest_Han_AC=1;N
chr5	161008657	.	T	<INS:ME:LINE1>	.	PASS	SVTYPE=INS:ME:LINE1;END=161008673;SVLEN=455;AN=20618;AC=1108;AF=0.05373945096517606;N_HET=1042;N_HOMALT=33;N_HOMREF=9234;FREQ_HET=0.10107672907168494;FREQ_HOMALT=0.003201086429333592;FREQ_HOMREF=0.8957221844989814;N_BI_GENOS=10309;South_Han_AN=1496;South_Han
chr5	161382524	.	T	<INS:ME:LINE1>	.	PASS	SVTYPE=INS:ME:LINE1;END=161382543;SVLEN=842;AN=20618;AC=2;AF=9.700261907071492e-05;N_HET=2;N_HOMALT=0;N_HOMREF=10307;FREQ_HET=0.00019400523814142983;FREQ_HOMALT=0.0;FREQ_HOMREF=0.9998059947618586;N_BI_GENOS=10309;South_Han_AN=1496;South_Han_AC=1;South_Han
chr5	161527077	.	A	<INS:ME:LINE1>	.	PASS	SVTYPE=INS:ME:LINE1;END=161527090;SVLEN=5997;AN=20618;AC=3;AF=0.00014550392860607236;N_HET=1;N_HOMALT=1;N_HOMREF=10307;FREQ_HET=9.700261907071492e-05;FREQ_HOMALT=9.700261907071492e-05;FREQ_HOMREF=0.9998059947618586;N_BI_GENOS=10309;East_Han_AN=6554;East_H
chr5	161569759	.	C	<INS:ME:LINE1>	.	PASS	SVTYPE=INS:ME:LINE1;END=161569776;SVLEN=1877;AN=20618;AC=2;AF=9.700261907071492e-05;N_HET=2;N_HOMALT=0;N_HOMREF=10307;FREQ_HET=0.00019400523814142983;FREQ_HOMALT=0.0;FREQ_HOMREF=0.9998059947618586;N_BI_GENOS=10309;Lingnan_Han_AN=1888;Lingnan_Han_AC=2;Ling
chr5	161682079	.	G	<INS:ME:LINE1>	.	PASS	SVTYPE=INS:ME:LINE1;END=161682086;SVLEN=4153;AN=20618;AC=3;AF=0.00014550392860607236;N_HET=3;N_HOMALT=0;N_HOMREF=10306;FREQ_HET=0.0002910078572121447;FREQ_HOMALT=0.0;FREQ_HOMREF=0.9997089921427879;N_BI_GENOS=10309;South_Han_AN=1496;South_Han_AC=1;South_Ha
chr5	161885500	.	A	<INS:ME:LINE1>	.	PASS	SVTYPE=INS:ME:LINE1;END=161885505;SVLEN=3732;AN=20618;AC=1;AF=4.850130953535746e-05;N_HET=1;N_HOMALT=0;N_HOMREF=10308;FREQ_HET=9.700261907071492e-05;FREQ_HOMALT=0.0;FREQ_HOMREF=0.9999029973809292;N_BI_GENOS=10309;Southeast_Han_AN=1908;Southeast_Han_AC=1;S
chr5	162746694	.	A	<INS:ME:LINE1>	.	PASS	SVTYPE=INS:ME:LINE1;END=162746713;SVLEN=2901;AN=20618;AC=5;AF=0.00024250654767678728;N_HET=5;N_HOMALT=0;N_HOMREF=10304;FREQ_HET=0.00048501309535357455;FREQ_HOMALT=0.0;FREQ_HOMREF=0.9995149869046465;N_BI_GENOS=10309;South_Han_AN=1496;South_Han_AC=1;South_H
chr5	162765739	.	A	<INS:ME:LINE1>	.	PASS	SVTYPE=INS:ME:LINE1;END=162765756;SVLEN=974;AN=20618;AC=2;AF=9.700261907071492e-05;N_HET=2;N_HOMALT=0;N_HOMREF=10307;FREQ_HET=0.00019400523814142983;FREQ_HOMALT=0.0;FREQ_HOMREF=0.9998059947618586;N_BI_GENOS=10309;Central_Han_AN=2128;Central_Han_AC=1;Centr
chr5	162897710	.	A	<INS:ME:LINE1>	.	PASS	SVTYPE=INS:ME:LINE1;END=162897724;SVLEN=785;AN=20618;AC=1;AF=4.850130953535746e-05;N_HET=1;N_HOMALT=0;N_HOMREF=10308;FREQ_HET=9.700261907071492e-05;FREQ_HOMALT=0.0;FREQ_HOMREF=0.9999029973809292;N_BI_GENOS=10309;East_Han_AN=6554;East_Han_AC=1;East_Han_AF=
chr5	162947410	.	G	<INS:ME:LINE1>	.	PASS	SVTYPE=INS:ME:LINE1;END=162947423;SVLEN=6001;AN=20618;AC=2;AF=9.700261907071492e-05;N_HET=2;N_HOMALT=0;N_HOMREF=10307;FREQ_HET=0.00019400523814142983;FREQ_HOMALT=0.0;FREQ_HOMREF=0.9998059947618586;N_BI_GENOS=10309;Lingnan_Han_AN=1888;Lingnan_Han_AC=2;Ling
chr5	163062316	.	T	<INS:ME:LINE1>	.	PASS	SVTYPE=INS:ME:LINE1;END=163062317;SVLEN=2870;AN=20618;AC=1;AF=4.850130953535746e-05;N_HET=1;N_HOMALT=0;N_HOMREF=10308;FREQ_HET=9.700261907071492e-05;FREQ_HOMALT=0.0;FREQ_HOMREF=0.9999029973809292;N_BI_GENOS=10309;Central_Han_AN=2128;Central_Han_AC=1;Centr
chr5	163283359	.	C	<INS:ME:LINE1>	.	PASS	SVTYPE=INS:ME:LINE1;END=163283373;SVLEN=373;AN=20618;AC=1;AF=4.850130953535746e-05;N_HET=1;N_HOMALT=0;N_HOMREF=10308;FREQ_HET=9.700261907071492e-05;FREQ_HOMALT=0.0;FREQ_HOMREF=0.9999029973809292;N_BI_GENOS=10309;Northwest_Han_AN=1296;Northwest_Han_AC=1;No
chr5	163347563	.	A	<INS:ME:LINE1>	.	PASS	SVTYPE=INS:ME:LINE1;END=163347581;SVLEN=706;AN=20618;AC=2;AF=9.700261907071492e-05;N_HET=2;N_HOMALT=0;N_HOMREF=10307;FREQ_HET=0.00019400523814142983;FREQ_HOMALT=0.0;FREQ_HOMREF=0.9998059947618586;N_BI_GENOS=10309;Northwest_Han_AN=1296;Northwest_Han_AC=1;N
chr5	163403240	.	A	<INS:ME:LINE1>	.	PASS	SVTYPE=INS:ME:LINE1;END=163403250;SVLEN=500;AN=20618;AC=1;AF=4.850130953535746e-05;N_HET=1;N_HOMALT=0;N_HOMREF=10308;FREQ_HET=9.700261907071492e-05;FREQ_HOMALT=0.0;FREQ_HOMREF=0.9999029973809292;N_BI_GENOS=10309;North_Han_AN=2272;North_Han_AC=1;North_Han_
chr5	163596784	.	T	<INS:ME:LINE1>	.	PASS	SVTYPE=INS:ME:LINE1;END=163596784;SVLEN=5964;AN=20618;AC=1;AF=4.850130953535746e-05;N_HET=1;N_HOMALT=0;N_HOMREF=10308;FREQ_HET=9.700261907071492e-05;FREQ_HOMALT=0.0;FREQ_HOMREF=0.9999029973809292;N_BI_GENOS=10309;Hui_AN=444;Hui_AC=1;Hui_AF=0.0022522522522
chr5	163711677	.	A	<INS:ME:LINE1>	.	PASS	SVTYPE=INS:ME:LINE1;END=163711690;SVLEN=5998;AN=20618;AC=1;AF=4.850130953535746e-05;N_HET=1;N_HOMALT=0;N_HOMREF=10308;FREQ_HET=9.700261907071492e-05;FREQ_HOMALT=0.0;FREQ_HOMREF=0.9999029973809292;N_BI_GENOS=10309;South_Han_AN=1496;South_Han_AC=1;South_Han
chr5	164204679	.	T	<INS:ME:LINE1>	.	PASS	SVTYPE=INS:ME:LINE1;END=164204697;SVLEN=390;AN=20618;AC=1;AF=4.850130953535746e-05;N_HET=1;N_HOMALT=0;N_HOMREF=10308;FREQ_HET=9.700261907071492e-05;FREQ_HOMALT=0.0;FREQ_HOMREF=0.9999029973809292;N_BI_GENOS=10309;East_Han_AN=6554;East_Han_AC=1;East_Han_AF=
chr5	164207557	.	C	<INS:ME:LINE1>	.	PASS	SVTYPE=INS:ME:LINE1;END=164207571;SVLEN=266;AN=20618;AC=2;AF=9.700261907071492e-05;N_HET=2;N_HOMALT=0;N_HOMREF=10307;FREQ_HET=0.00019400523814142983;FREQ_HOMALT=0.0;FREQ_HOMREF=0.9998059947618586;N_BI_GENOS=10309;Central_Han_AN=2128;Central_Han_AC=2;Centr
chr5	164412763	.	A	<INS:ME:LINE1>	.	PASS	SVTYPE=INS:ME:LINE1;END=164412766;SVLEN=963;AN=20618;AC=2;AF=9.700261907071492e-05;N_HET=2;N_HOMALT=0;N_HOMREF=10307;FREQ_HET=0.00019400523814142983;FREQ_HOMALT=0.0;FREQ_HOMREF=0.9998059947618586;N_BI_GENOS=10309;Central_Han_AN=2128;Central_Han_AC=2;Centr
chr5	164553304	.	T	<INS:ME:LINE1>	.	PASS	SVTYPE=INS:ME:LINE1;END=164553317;SVLEN=1019;AN=20618;AC=46;AF=0.002231060238626443;N_HET=46;N_HOMALT=0;N_HOMREF=10263;FREQ_HET=0.004462120477252886;FREQ_HOMALT=0.0;FREQ_HOMREF=0.9955378795227471;N_BI_GENOS=10309;South_Han_AN=1496;South_Han_AC=5;South_Han
chr5	164750920	.	G	<INS:ME:LINE1>	.	PASS	SVTYPE=INS:ME:LINE1;END=164750939;SVLEN=1138;AN=20618;AC=1;AF=4.850130953535746e-05;N_HET=1;N_HOMALT=0;N_HOMREF=10308;FREQ_HET=9.700261907071492e-05;FREQ_HOMALT=0.0;FREQ_HOMREF=0.9999029973809292;N_BI_GENOS=10309;South_Han_AN=1496;South_Han_AC=1;South_Han
chr5	164859316	.	A	<INS:ME:LINE1>	.	PASS	SVTYPE=INS:ME:LINE1;END=164859330;SVLEN=2238;AN=20618;AC=1;AF=4.850130953535746e-05;N_HET=1;N_HOMALT=0;N_HOMREF=10308;FREQ_HET=9.700261907071492e-05;FREQ_HOMALT=0.0;FREQ_HOMREF=0.9999029973809292;N_BI_GENOS=10309;North_Han_AN=2272;North_Han_AC=1;North_Han
chr5	164965502	.	A	<INS:ME:LINE1>	.	PASS	SVTYPE=INS:ME:LINE1;END=164965518;SVLEN=6038;AN=20618;AC=1;AF=4.850130953535746e-05;N_HET=1;N_HOMALT=0;N_HOMREF=10308;FREQ_HET=9.700261907071492e-05;FREQ_HOMALT=0.0;FREQ_HOMREF=0.9999029973809292;N_BI_GENOS=10309;Tibetan_AN=414;Tibetan_AC=1;Tibetan_AF=0.0
chr5	165049693	.	C	<INS:ME:LINE1>	.	PASS	SVTYPE=INS:ME:LINE1;END=165049701;SVLEN=36;AN=20618;AC=1;AF=4.850130953535746e-05;N_HET=1;N_HOMALT=0;N_HOMREF=10308;FREQ_HET=9.700261907071492e-05;FREQ_HOMALT=0.0;FREQ_HOMREF=0.9999029973809292;N_BI_GENOS=10309;Central_Han_AN=2128;Central_Han_AC=1;Central
chr5	165063570	.	G	<INS:ME:LINE1>	.	PASS	SVTYPE=INS:ME:LINE1;END=165063586;SVLEN=1330;AN=20618;AC=1;AF=4.850130953535746e-05;N_HET=1;N_HOMALT=0;N_HOMREF=10308;FREQ_HET=9.700261907071492e-05;FREQ_HOMALT=0.0;FREQ_HOMREF=0.9999029973809292;N_BI_GENOS=10309;South_Han_AN=1496;South_Han_AC=1;South_Han
chr5	165068137	.	C	<INS:ME:LINE1>	.	PASS	SVTYPE=INS:ME:LINE1;END=165068154;SVLEN=6000;AN=20618;AC=104;AF=0.005044136191677175;N_HET=88;N_HOMALT=8;N_HOMREF=10213;FREQ_HET=0.008536230478222911;FREQ_HOMALT=0.0007760209525657193;FREQ_HOMREF=0.9906877485692114;N_BI_GENOS=10309;South_Han_AN=1496;South
chr5	165111426	.	G	<INS:ME:LINE1>	.	PASS	SVTYPE=INS:ME:LINE1;END=165111436;SVLEN=5998;AN=20618;AC=1;AF=4.850130953535746e-05;N_HET=1;N_HOMALT=0;N_HOMREF=10308;FREQ_HET=9.700261907071492e-05;FREQ_HOMALT=0.0;FREQ_HOMREF=0.9999029973809292;N_BI_GENOS=10309;East_Han_AN=6554;East_Han_AC=1;East_Han_AF
chr5	165365767	.	T	<INS:ME:LINE1>	.	PASS	SVTYPE=INS:ME:LINE1;END=165365781;SVLEN=4044;AN=20618;AC=1;AF=4.850130953535746e-05;N_HET=1;N_HOMALT=0;N_HOMREF=10308;FREQ_HET=9.700261907071492e-05;FREQ_HOMALT=0.0;FREQ_HOMREF=0.9999029973809292;N_BI_GENOS=10309;Tibetan_AN=414;Tibetan_AC=1;Tibetan_AF=0.0
chr5	165497745	.	A	<INS:ME:LINE1>	.	PASS	SVTYPE=INS:ME:LINE1;END=165497762;SVLEN=1439;AN=20618;AC=2;AF=9.700261907071492e-05;N_HET=2;N_HOMALT=0;N_HOMREF=10307;FREQ_HET=0.00019400523814142983;FREQ_HOMALT=0.0;FREQ_HOMREF=0.9998059947618586;N_BI_GENOS=10309;East_Han_AN=6554;East_Han_AC=1;East_Han_A

chr5	165763336	.	G	<INS:ME:LINE1>	.	PASS	SVTYPE=INS:ME:LINE1;END=165763336;SVLEN=0;AN=20618;AC=6;AF=0.0002910078572121447;N_HET=4;N_HOMALT=1;N_HOMREF=10304;FREQ_HET=0.00038801047628285966;FREQ_HOMALT=9.700261907071492e-05;FREQ_HOMREF=0.9995149869046465;N_BI_GENOS=10309;Northwest_Han_AN=1296;Nort
chr5	165903621	.	C	<INS:ME:LINE1>	.	PASS	SVTYPE=INS:ME:LINE1;END=165903621;SVLEN=6;AN=20618;AC=1;AF=4.850130953535746e-05;N_HET=1;N_HOMALT=0;N_HOMREF=10308;FREQ_HET=9.700261907071492e-05;FREQ_HOMALT=0.0;FREQ_HOMREF=0.9999029973809292;N_BI_GENOS=10309;Southeast_Han_AN=1908;Southeast_Han_AC=1;Sout
chr5	165957166	.	A	<INS:ME:LINE1>	.	PASS	SVTYPE=INS:ME:LINE1;END=165957182;SVLEN=439;AN=20618;AC=2;AF=9.700261907071492e-05;N_HET=2;N_HOMALT=0;N_HOMREF=10307;FREQ_HET=0.00019400523814142983;FREQ_HOMALT=0.0;FREQ_HOMREF=0.9998059947618586;N_BI_GENOS=10309;Central_Han_AN=2128;Central_Han_AC=1;Centr
chr5	166222092	.	A	<INS:ME:LINE1>	.	PASS	SVTYPE=INS:ME:LINE1;END=166222108;SVLEN=457;AN=20618;AC=1;AF=4.850130953535746e-05;N_HET=1;N_HOMALT=0;N_HOMREF=10308;FREQ_HET=9.700261907071492e-05;FREQ_HOMALT=0.0;FREQ_HOMREF=0.9999029973809292;N_BI_GENOS=10309;East_Han_AN=6554;East_Han_AC=1;East_Han_AF=
chr5	166462377	.	T	<INS:ME:LINE1>	.	PASS	SVTYPE=INS:ME:LINE1;END=166462395;SVLEN=1176;AN=20618;AC=2;AF=9.700261907071492e-05;N_HET=2;N_HOMALT=0;N_HOMREF=10307;FREQ_HET=0.00019400523814142983;FREQ_HOMALT=0.0;FREQ_HOMREF=0.9998059947618586;N_BI_GENOS=10309;Tibetan_AN=414;Tibetan_AC=2;Tibetan_AF=0.
chr5	167018989	.	A	<INS:ME:LINE1>	.	PASS	SVTYPE=INS:ME:LINE1;END=167019002;SVLEN=2199;AN=20618;AC=1;AF=4.850130953535746e-05;N_HET=1;N_HOMALT=0;N_HOMREF=10308;FREQ_HET=9.700261907071492e-05;FREQ_HOMALT=0.0;FREQ_HOMREF=0.9999029973809292;N_BI_GENOS=10309;East_Han_AN=6554;East_Han_AC=1;East_Han_AF
chr5	167225384	.	T	<INS:ME:LINE1>	.	PASS	SVTYPE=INS:ME:LINE1;END=167225384;SVLEN=382;AN=20618;AC=1;AF=4.850130953535746e-05;N_HET=1;N_HOMALT=0;N_HOMREF=10308;FREQ_HET=9.700261907071492e-05;FREQ_HOMALT=0.0;FREQ_HOMREF=0.9999029973809292;N_BI_GENOS=10309;East_Han_AN=6554;East_Han_AC=1;East_Han_AF=
chr5	167885982	.	C	<INS:ME:LINE1>	.	PASS	SVTYPE=INS:ME:LINE1;END=167885996;SVLEN=266;AN=20618;AC=1;AF=4.850130953535746e-05;N_HET=1;N_HOMALT=0;N_HOMREF=10308;FREQ_HET=9.700261907071492e-05;FREQ_HOMALT=0.0;FREQ_HOMREF=0.9999029973809292;N_BI_GENOS=10309;South_Han_AN=1496;South_Han_AC=1;South_Han_
chr5	168059682	.	T	<INS:ME:LINE1>	.	PASS	SVTYPE=INS:ME:LINE1;END=168059697;SVLEN=6000;AN=20618;AC=6;AF=0.0002910078572121447;N_HET=6;N_HOMALT=0;N_HOMREF=10303;FREQ_HET=0.0005820157144242894;FREQ_HOMALT=0.0;FREQ_HOMREF=0.9994179842855757;N_BI_GENOS=10309;Lingnan_Han_AN=1888;Lingnan_Han_AC=2;Lingn
chr5	168129334	.	A	<INS:ME:LINE1>	.	PASS	SVTYPE=INS:ME:LINE1;END=168129350;SVLEN=5999;AN=20618;AC=1;AF=4.850130953535746e-05;N_HET=1;N_HOMALT=0;N_HOMREF=10308;FREQ_HET=9.700261907071492e-05;FREQ_HOMALT=0.0;FREQ_HOMREF=0.9999029973809292;N_BI_GENOS=10309;East_Han_AN=6554;East_Han_AC=1;East_Han_AF
chr5	168732314	.	A	<INS:ME:LINE1>	.	PASS	SVTYPE=INS:ME:LINE1;END=168732314;SVLEN=1047;AN=20618;AC=1;AF=4.850130953535746e-05;N_HET=1;N_HOMALT=0;N_HOMREF=10308;FREQ_HET=9.700261907071492e-05;FREQ_HOMALT=0.0;FREQ_HOMREF=0.9999029973809292;N_BI_GENOS=10309;East_Han_AN=6554;East_Han_AC=1;East_Han_AF
chr5	169231986	.	C	<INS:ME:LINE1>	.	PASS	SVTYPE=INS:ME:LINE1;END=169231996;SVLEN=357;AN=20618;AC=5;AF=0.00024250654767678728;N_HET=5;N_HOMALT=0;N_HOMREF=10304;FREQ_HET=0.00048501309535357455;FREQ_HOMALT=0.0;FREQ_HOMREF=0.9995149869046465;N_BI_GENOS=10309;Northwest_Han_AN=1296;Northwest_Han_AC=1;
chr5	169235134	.	T	<INS:ME:LINE1>	.	PASS	SVTYPE=INS:ME:LINE1;END=169235135;SVLEN=3434;AN=20618;AC=1;AF=4.850130953535746e-05;N_HET=1;N_HOMALT=0;N_HOMREF=10308;FREQ_HET=9.700261907071492e-05;FREQ_HOMALT=0.0;FREQ_HOMREF=0.9999029973809292;N_BI_GENOS=10309;Central_Han_AN=2128;Central_Han_AC=1;Centr
chr5	169405605	.	G	<INS:ME:LINE1>	.	PASS	SVTYPE=INS:ME:LINE1;END=169405620;SVLEN=5998;AN=20618;AC=1;AF=4.850130953535746e-05;N_HET=1;N_HOMALT=0;N_HOMREF=10308;FREQ_HET=9.700261907071492e-05;FREQ_HOMALT=0.0;FREQ_HOMREF=0.9999029973809292;N_BI_GENOS=10309;Northwest_Han_AN=1296;Northwest_Han_AC=1;N
chr5	169474535	.	A	<INS:ME:LINE1>	.	PASS	SVTYPE=INS:ME:LINE1;END=169474551;SVLEN=6031;AN=20618;AC=1;AF=4.850130953535746e-05;N_HET=1;N_HOMALT=0;N_HOMREF=10308;FREQ_HET=9.700261907071492e-05;FREQ_HOMALT=0.0;FREQ_HOMREF=0.9999029973809292;N_BI_GENOS=10309;Southeast_Han_AN=1908;Southeast_Han_AC=1;S
chr5	169575746	.	A	<INS:ME:LINE1>	.	PASS	SVTYPE=INS:ME:LINE1;END=169575746;SVLEN=92;AN=20618;AC=1;AF=4.850130953535746e-05;N_HET=1;N_HOMALT=0;N_HOMREF=10308;FREQ_HET=9.700261907071492e-05;FREQ_HOMALT=0.0;FREQ_HOMREF=0.9999029973809292;N_BI_GENOS=10309;Mongolian_AN=454;Mongolian_AC=1;Mongolian_AF
chr5	169696275	.	T	<INS:ME:LINE1>	.	PASS	SVTYPE=INS:ME:LINE1;END=169696292;SVLEN=1404;AN=20618;AC=1;AF=4.850130953535746e-05;N_HET=1;N_HOMALT=0;N_HOMREF=10308;FREQ_HET=9.700261907071492e-05;FREQ_HOMALT=0.0;FREQ_HOMREF=0.9999029973809292;N_BI_GENOS=10309;East_Han_AN=6554;East_Han_AC=1;East_Han_AF
chr5	169739714	.	A	<INS:ME:LINE1>	.	PASS	SVTYPE=INS:ME:LINE1;END=169739733;SVLEN=933;AN=20618;AC=3;AF=0.00014550392860607236;N_HET=3;N_HOMALT=0;N_HOMREF=10306;FREQ_HET=0.0002910078572121447;FREQ_HOMALT=0.0;FREQ_HOMREF=0.9997089921427879;N_BI_GENOS=10309;Lingnan_Han_AN=1888;Lingnan_Han_AC=1;Lingn
chr5	169826208	.	A	<INS:ME:LINE1>	.	PASS	SVTYPE=INS:ME:LINE1;END=169826222;SVLEN=1144;AN=20618;AC=3;AF=0.00014550392860607236;N_HET=3;N_HOMALT=0;N_HOMREF=10306;FREQ_HET=0.0002910078572121447;FREQ_HOMALT=0.0;FREQ_HOMREF=0.9997089921427879;N_BI_GENOS=10309;South_Han_AN=1496;South_Han_AC=1;South_Ha
chr5	169949688	.	A	<INS:ME:LINE1>	.	PASS	SVTYPE=INS:ME:LINE1;END=169949688;SVLEN=0;AN=20618;AC=1;AF=4.850130953535746e-05;N_HET=1;N_HOMALT=0;N_HOMREF=10308;FREQ_HET=9.700261907071492e-05;FREQ_HOMALT=0.0;FREQ_HOMREF=0.9999029973809292;N_BI_GENOS=10309;Northwest_Han_AN=1296;Northwest_Han_AC=1;Nort
chr5	170209203	.	G	<INS:ME:LINE1>	.	PASS	SVTYPE=INS:ME:LINE1;END=170209218;SVLEN=6068;AN=20618;AC=2;AF=9.700261907071492e-05;N_HET=2;N_HOMALT=0;N_HOMREF=10307;FREQ_HET=0.00019400523814142983;FREQ_HOMALT=0.0;FREQ_HOMREF=0.9998059947618586;N_BI_GENOS=10309;Central_Han_AN=2128;Central_Han_AC=1;Cent
chr5	170457263	.	A	<INS:ME:LINE1>	.	PASS	SVTYPE=INS:ME:LINE1;END=170457276;SVLEN=6025;AN=20618;AC=1;AF=4.850130953535746e-05;N_HET=1;N_HOMALT=0;N_HOMREF=10308;FREQ_HET=9.700261907071492e-05;FREQ_HOMALT=0.0;FREQ_HOMREF=0.9999029973809292;N_BI_GENOS=10309;Yi_AN=422;Yi_AC=1;Yi_AF=0.0023696682464454
chr5	170816153	.	T	<INS:ME:LINE1>	.	PASS	SVTYPE=INS:ME:LINE1;END=170816153;SVLEN=824;AN=20618;AC=1;AF=4.850130953535746e-05;N_HET=1;N_HOMALT=0;N_HOMREF=10308;FREQ_HET=9.700261907071492e-05;FREQ_HOMALT=0.0;FREQ_HOMREF=0.9999029973809292;N_BI_GENOS=10309;East_Han_AN=6554;East_Han_AC=1;East_Han_AF=
chr5	170990398	.	T	<INS:ME:LINE1>	.	PASS	SVTYPE=INS:ME:LINE1;END=170990415;SVLEN=3030;AN=20618;AC=1;AF=4.850130953535746e-05;N_HET=1;N_HOMALT=0;N_HOMREF=10308;FREQ_HET=9.700261907071492e-05;FREQ_HOMALT=0.0;FREQ_HOMREF=0.9999029973809292;N_BI_GENOS=10309;East_Han_AN=6554;East_Han_AC=1;East_Han_AF
chr5	171012121	.	A	<INS:ME:LINE1>	.	PASS	SVTYPE=INS:ME:LINE1;END=171012136;SVLEN=2046;AN=20618;AC=4;AF=0.00019400523814142983;N_HET=4;N_HOMALT=0;N_HOMREF=10305;FREQ_HET=0.00038801047628285966;FREQ_HOMALT=0.0;FREQ_HOMREF=0.9996119895237171;N_BI_GENOS=10309;East_Han_AN=6554;East_Han_AC=3;East_Han_
chr5	171150819	.	A	<INS:ME:LINE1>	.	PASS	SVTYPE=INS:ME:LINE1;END=171150836;SVLEN=6007;AN=20618;AC=3;AF=0.00014550392860607236;N_HET=3;N_HOMALT=0;N_HOMREF=10306;FREQ_HET=0.0002910078572121447;FREQ_HOMALT=0.0;FREQ_HOMREF=0.9997089921427879;N_BI_GENOS=10309;East_Han_AN=6554;East_Han_AC=3;East_Han_A
chr5	171266942	.	T	<INS:ME:LINE1>	.	PASS	SVTYPE=INS:ME:LINE1;END=171266946;SVLEN=4258;AN=20618;AC=3;AF=0.00014550392860607236;N_HET=1;N_HOMALT=1;N_HOMREF=10307;FREQ_HET=9.700261907071492e-05;FREQ_HOMALT=9.700261907071492e-05;FREQ_HOMREF=0.9998059947618586;N_BI_GENOS=10309;North_Han_AN=2272;North
chr5	171820045	.	A	<INS:ME:LINE1>	.	PASS	SVTYPE=INS:ME:LINE1;END=171820060;SVLEN=5998;AN=20618;AC=5;AF=0.00024250654767678728;N_HET=5;N_HOMALT=0;N_HOMREF=10304;FREQ_HET=0.00048501309535357455;FREQ_HOMALT=0.0;FREQ_HOMREF=0.9995149869046465;N_BI_GENOS=10309;South_Han_AN=1496;South_Han_AC=2;South_H
chr5	172225651	.	A	<INS:ME:LINE1>	.	PASS	SVTYPE=INS:ME:LINE1;END=172225667;SVLEN=1045;AN=20618;AC=1;AF=4.850130953535746e-05;N_HET=1;N_HOMALT=0;N_HOMREF=10308;FREQ_HET=9.700261907071492e-05;FREQ_HOMALT=0.0;FREQ_HOMREF=0.9999029973809292;N_BI_GENOS=10309;East_Han_AN=6554;East_Han_AC=1;East_Han_AF
chr5	173218944	.	A	<INS:ME:LINE1>	.	PASS	SVTYPE=INS:ME:LINE1;END=173218957;SVLEN=90;AN=20618;AC=477;AF=0.023135124648365507;N_HET=389;N_HOMALT=44;N_HOMREF=9876;FREQ_HET=0.0377340188185081;FREQ_HOMALT=0.004268115239111456;FREQ_HOMREF=0.9579978659423805;N_BI_GENOS=10309;South_Han_AN=1496;South_Han
chr5	173315662	.	T	<INS:ME:LINE1>	.	PASS	SVTYPE=INS:ME:LINE1;END=173315676;SVLEN=6002;AN=20618;AC=1;AF=4.850130953535746e-05;N_HET=1;N_HOMALT=0;N_HOMREF=10308;FREQ_HET=9.700261907071492e-05;FREQ_HOMALT=0.0;FREQ_HOMREF=0.9999029973809292;N_BI_GENOS=10309;Southeast_Han_AN=1908;Southeast_Han_AC=1;S
chr5	173695188	.	A	<INS:ME:LINE1>	.	PASS	SVTYPE=INS:ME:LINE1;END=173695205;SVLEN=1984;AN=20618;AC=1;AF=4.850130953535746e-05;N_HET=1;N_HOMALT=0;N_HOMREF=10308;FREQ_HET=9.700261907071492e-05;FREQ_HOMALT=0.0;FREQ_HOMREF=0.9999029973809292;N_BI_GENOS=10309;Southeast_Han_AN=1908;Southeast_Han_AC=1;S
chr5	174945734	.	A	<INS:ME:LINE1>	.	PASS	SVTYPE=INS:ME:LINE1;END=174945734;SVLEN=6010;AN=20618;AC=1;AF=4.850130953535746e-05;N_HET=1;N_HOMALT=0;N_HOMREF=10308;FREQ_HET=9.700261907071492e-05;FREQ_HOMALT=0.0;FREQ_HOMREF=0.9999029973809292;N_BI_GENOS=10309;Central_Han_AN=2128;Central_Han_AC=1;Centr
chr5	175111880	.	T	<INS:ME:LINE1>	.	PASS	SVTYPE=INS:ME:LINE1;END=175111880;SVLEN=111;AN=20618;AC=1;AF=4.850130953535746e-05;N_HET=1;N_HOMALT=0;N_HOMREF=10308;FREQ_HET=9.700261907071492e-05;FREQ_HOMALT=0.0;FREQ_HOMREF=0.9999029973809292;N_BI_GENOS=10309;Zhuang_AN=444;Zhuang_AC=1;Zhuang_AF=0.00225
chr5	175333803	.	A	<INS:ME:LINE1>	.	PASS	SVTYPE=INS:ME:LINE1;END=175333803;SVLEN=0;AN=20618;AC=9;AF=0.0004365117858182171;N_HET=9;N_HOMALT=0;N_HOMREF=10300;FREQ_HET=0.0008730235716364342;FREQ_HOMALT=0.0;FREQ_HOMREF=0.9991269764283636;N_BI_GENOS=10309;Lingnan_Han_AN=1888;Lingnan_Han_AC=2;Lingnan_
chr5	175497097	.	A	<INS:ME:LINE1>	.	PASS	SVTYPE=INS:ME:LINE1;END=175497109;SVLEN=5985;AN=20618;AC=1;AF=4.850130953535746e-05;N_HET=1;N_HOMALT=0;N_HOMREF=10308;FREQ_HET=9.700261907071492e-05;FREQ_HOMALT=0.0;FREQ_HOMREF=0.9999029973809292;N_BI_GENOS=10309;North_Han_AN=2272;North_Han_AC=1;North_Han
chr5	176963507	.	G	<INS:ME:LINE1>	.	PASS	SVTYPE=INS:ME:LINE1;END=176963521;SVLEN=2359;AN=20618;AC=1;AF=4.850130953535746e-05;N_HET=1;N_HOMALT=0;N_HOMREF=10308;FREQ_HET=9.700261907071492e-05;FREQ_HOMALT=0.0;FREQ_HOMREF=0.9999029973809292;N_BI_GENOS=10309;South_Han_AN=1496;South_Han_AC=1;South_Han
chr5	179942185	.	G	<INS:ME:LINE1>	.	PASS	SVTYPE=INS:ME:LINE1;END=179942185;SVLEN=0;AN=20618;AC=609;AF=0.02953729750703269;N_HET=205;N_HOMALT=202;N_HOMREF=9902;FREQ_HET=0.019885536909496557;FREQ_HOMALT=0.019594529052284412;FREQ_HOMREF=0.960519934038219;N_BI_GENOS=10309;South_Han_AN=1496;South_Han
chr5	180201789	.	C	<INS:ME:LINE1>	.	PASS	SVTYPE=INS:ME:LINE1;END=180201792;SVLEN=861;AN=20618;AC=2;AF=9.700261907071492e-05;N_HET=2;N_HOMALT=0;N_HOMREF=10307;FREQ_HET=0.00019400523814142983;FREQ_HOMALT=0.0;FREQ_HOMREF=0.9998059947618586;N_BI_GENOS=10309;East_Han_AN=6554;East_Han_AC=2;East_Han_AF
chr5	180503263	.	T	<INS:ME:LINE1>	.	PASS	SVTYPE=INS:ME:LINE1;END=180503279;SVLEN=5993;AN=20618;AC=1;AF=4.850130953535746e-05;N_HET=1;N_HOMALT=0;N_HOMREF=10308;FREQ_HET=9.700261907071492e-05;FREQ_HOMALT=0.0;FREQ_HOMREF=0.9999029973809292;N_BI_GENOS=10309;Central_Han_AN=2128;Central_Han_AC=1;Centr
chr5	180878438	.	A	<INS:ME:LINE1>	.	PASS	SVTYPE=INS:ME:LINE1;END=180878453;SVLEN=213;AN=20618;AC=3;AF=0.00014550392860607236;N_HET=1;N_HOMALT=1;N_HOMREF=10307;FREQ_HET=9.700261907071492e-05;FREQ_HOMALT=9.700261907071492e-05;FREQ_HOMREF=0.9998059947618586;N_BI_GENOS=10309;East_Han_AN=6554;East_Ha
chr5	180917722	.	A	<INS:ME:LINE1>	.	PASS	SVTYPE=INS:ME:LINE1;END=180917736;SVLEN=1494;AN=20618;AC=2;AF=9.700261907071492e-05;N_HET=2;N_HOMALT=0;N_HOMREF=10307;FREQ_HET=0.00019400523814142983;FREQ_HOMALT=0.0;FREQ_HOMREF=0.9998059947618586;N_BI_GENOS=10309;Central_Han_AN=2128;Central_Han_AC=1;Cent
chr6	565074	.	A	<INS:ME:LINE1>	.	PASS	SVTYPE=INS:ME:LINE1;END=565088;SVLEN=967;AN=20618;AC=71;AF=0.0034435929770103794;N_HET=69;N_HOMALT=1;N_HOMREF=10239;FREQ_HET=0.006693180715879329;FREQ_HOMALT=9.700261907071492e-05;FREQ_HOMREF=0.9932098166650499;N_BI_GENOS=10309;South_Han_AN=1496;South_Han
chr6	686308	.	A	<INS:ME:LINE1>	.	PASS	SVTYPE=INS:ME:LINE1;END=686327;SVLEN=308;AN=20618;AC=2;AF=9.700261907071492e-05;N_HET=2;N_HOMALT=0;N_HOMREF=10307;FREQ_HET=0.00019400523814142983;FREQ_HOMALT=0.0;FREQ_HOMREF=0.9998059947618586;N_BI_GENOS=10309;East_Han_AN=6554;East_Han_AC=1;East_Han_AF=0.
chr6	1215198	.	T	<INS:ME:LINE1>	.	PASS	SVTYPE=INS:ME:LINE1;END=1215213;SVLEN=886;AN=20618;AC=8;AF=0.00038801047628285966;N_HET=8;N_HOMALT=0;N_HOMREF=10301;FREQ_HET=0.0007760209525657193;FREQ_HOMALT=0.0;FREQ_HOMREF=0.9992239790474343;N_BI_GENOS=10309;South_Han_AN=1496;South_Han_AC=1;South_Han_A
chr6	1375557	.	A	<INS:ME:LINE1>	.	PASS	SVTYPE=INS:ME:LINE1;END=1375572;SVLEN=303;AN=20618;AC=4;AF=0.00019400523814142983;N_HET=4;N_HOMALT=0;N_HOMREF=10305;FREQ_HET=0.00038801047628285966;FREQ_HOMALT=0.0;FREQ_HOMREF=0.9996119895237171;N_BI_GENOS=10309;North_Han_AN=2272;North_Han_AC=1;North_Han_
chr6	1950390	.	A	<INS:ME:LINE1>	.	PASS	SVTYPE=INS:ME:LINE1;END=1950405;SVLEN=5999;AN=20618;AC=1;AF=4.850130953535746e-05;N_HET=1;N_HOMALT=0;N_HOMREF=10308;FREQ_HET=9.700261907071492e-05;FREQ_HOMALT=0.0;FREQ_HOMREF=0.9999029973809292;N_BI_GENOS=10309;East_Han_AN=6554;East_Han_AC=1;East_Han_AF=0
chr6	1988866	.	A	<INS:ME:LINE1>	.	PASS	SVTYPE=INS:ME:LINE1;END=1988899;SVLEN=687;AN=20618;AC=3;AF=0.00014550392860607236;N_HET=3;N_HOMALT=0;N_HOMREF=10306;FREQ_HET=0.0002910078572121447;FREQ_HOMALT=0.0;FREQ_HOMREF=0.9997089921427879;N_BI_GENOS=10309;South_Han_AN=1496;South_Han_AC=1;South_Han_A
chr6	1994317	.	A	<INS:ME:LINE1>	.	PASS	SVTYPE=INS:ME:LINE1;END=1994317;SVLEN=70;AN=20618;AC=1;AF=4.850130953535746e-05;N_HET=1;N_HOMALT=0;N_HOMREF=10308;FREQ_HET=9.700261907071492e-05;FREQ_HOMALT=0.0;FREQ_HOMREF=0.9999029973809292;N_BI_GENOS=10309;Mongolian_AN=454;Mongolian_AC=1;Mongolian_AF=0
chr6	2082208	.	A	<INS:ME:LINE1>	.	PASS	SVTYPE=INS:ME:LINE1;END=2082208;SVLEN=6008;AN=20618;AC=1;AF=4.850130953535746e-05;N_HET=1;N_HOMALT=0;N_HOMREF=10308;FREQ_HET=9.700261907071492e-05;FREQ_HOMALT=0.0;FREQ_HOMREF=0.9999029973809292;N_BI_GENOS=10309;East_Han_AN=6554;East_Han_AC=1;East_Han_AF=0
chr6	2135548	.	G	<INS:ME:LINE1>	.	PASS	SVTYPE=INS:ME:LINE1;END=2135556;SVLEN=129;AN=20618;AC=2;AF=9.700261907071492e-05;N_HET=2;N_HOMALT=0;N_HOMREF=10307;FREQ_HET=0.00019400523814142983;FREQ_HOMALT=0.0;FREQ_HOMREF=0.9998059947618586;N_BI_GENOS=10309;Northwest_Han_AN=1296;Northwest_Han_AC=1;Nor
chr6	2513703	.	A	<INS:ME:LINE1>	.	PASS	SVTYPE=INS:ME:LINE1;END=2513703;SVLEN=0;AN=20618;AC=51;AF=0.0024735667863032304;N_HET=51;N_HOMALT=0;N_HOMREF=10258;FREQ_HET=0.004947133572606461;FREQ_HOMALT=0.0;FREQ_HOMREF=0.9950528664273935;N_BI_GENOS=10309;South_Han_AN=1496;South_Han_AC=4;South_Han_AF=
chr6	3301204	.	G	<INS:ME:LINE1>	.	PASS	SVTYPE=INS:ME:LINE1;END=3301220;SVLEN=6004;AN=20618;AC=1;AF=4.850130953535746e-05;N_HET=1;N_HOMALT=0;N_HOMREF=10308;FREQ_HET=9.700261907071492e-05;FREQ_HOMALT=0.0;FREQ_HOMREF=0.9999029973809292;N_BI_GENOS=10309;Tibetan_AN=414;Tibetan_AC=1;Tibetan_AF=0.002
chr6	3578353	.	T	<INS:ME:LINE1>	.	PASS	SVTYPE=INS:ME:LINE1;END=3578363;SVLEN=79;AN=20618;AC=1;AF=4.850130953535746e-05;N_HET=1;N_HOMALT=0;N_HOMREF=10308;FREQ_HET=9.700261907071492e-05;FREQ_HOMALT=0.0;FREQ_HOMREF=0.9999029973809292;N_BI_GENOS=10309;South_Han_AN=1496;South_Han_AC=1;South_Han_AF=
chr6	3615485	.	A	<INS:ME:LINE1>	.	PASS	SVTYPE=INS:ME:LINE1;END=3615503;SVLEN=251;AN=20618;AC=2;AF=9.700261907071492e-05;N_HET=2;N_HOMALT=0;N_HOMREF=10307;FREQ_HET=0.00019400523814142983;FREQ_HOMALT=0.0;FREQ_HOMREF=0.9998059947618586;N_BI_GENOS=10309;North_Han_AN=2272;North_Han_AC=1;North_Han_A
chr6	4143922	.	A	<INS:ME:LINE1>	.	PASS	SVTYPE=INS:ME:LINE1;END=4143938;SVLEN=5989;AN=20618;AC=1;AF=4.850130953535746e-05;N_HET=1;N_HOMALT=0;N_HOMREF=10308;FREQ_HET=9.700261907071492e-05;FREQ_HOMALT=0.0;FREQ_HOMREF=0.9999029973809292;N_BI_GENOS=10309;Hui_AN=444;Hui_AC=1;Hui_AF=0.002252252252252
chr6	4430494	.	A	<INS:ME:LINE1>	.	PASS	SVTYPE=INS:ME:LINE1;END=4430494;SVLEN=15;AN=20618;AC=1;AF=4.850130953535746e-05;N_HET=1;N_HOMALT=0;N_HOMREF=10308;FREQ_HET=9.700261907071492e-05;FREQ_HOMALT=0.0;FREQ_HOMREF=0.9999029973809292;N_BI_GENOS=10309;Lingnan_Han_AN=1888;Lingnan_Han_AC=1;Lingnan_H
chr6	5366933	.	G	<INS:ME:LINE1>	.	PASS	SVTYPE=INS:ME:LINE1;END=5366948;SVLEN=1144;AN=20618;AC=2;AF=9.700261907071492e-05;N_HET=2;N_HOMALT=0;N_HOMREF=10307;FREQ_HET=0.00019400523814142983;FREQ_HOMALT=0.0;FREQ_HOMREF=0.9998059947618586;N_BI_GENOS=10309;North_Han_AN=2272;North_Han_AC=1;North_Han_
chr6	5965291	.	T	<INS:ME:LINE1>	.	PASS	SVTYPE=INS:ME:LINE1;END=5965309;SVLEN=471;AN=20618;AC=1;AF=4.850130953535746e-05;N_HET=1;N_HOMALT=0;N_HOMREF=10308;FREQ_HET=9.700261907071492e-05;FREQ_HOMALT=0.0;FREQ_HOMREF=0.9999029973809292;N_BI_GENOS=10309;East_Han_AN=6554;East_Han_AC=1;East_Han_AF=0.
chr6	6264777	.	T	<INS:ME:LINE1>	.	PASS	SVTYPE=INS:ME:LINE1;END=6264786;SVLEN=5998;AN=20618;AC=3;AF=0.00014550392860607236;N_HET=3;N_HOMALT=0;N_HOMREF=10306;FREQ_HET=0.0002910078572121447;FREQ_HOMALT=0.0;FREQ_HOMREF=0.9997089921427879;N_BI_GENOS=10309;North_Han_AN=2272;North_Han_AC=2;North_Han_
chr6	6349670	.	A	<INS:ME:LINE1>	.	PASS	SVTYPE=INS:ME:LINE1;END=6349681;SVLEN=543;AN=20618;AC=1;AF=4.850130953535746e-05;N_HET=1;N_HOMALT=0;N_HOMREF=10308;FREQ_HET=9.700261907071492e-05;FREQ_HOMALT=0.0;FREQ_HOMREF=0.9999029973809292;N_BI_GENOS=10309;East_Han_AN=6554;East_Han_AC=1;East_Han_AF=0.
chr6	6369673	.	A	<INS:ME:LINE1>	.	PASS	SVTYPE=INS:ME:LINE1;END=6369673;SVLEN=5977;AN=20618;AC=1;AF=4.850130953535746e-05;N_HET=1;N_HOMALT=0;N_HOMREF=10308;FREQ_HET=9.700261907071492e-05;FREQ_HOMALT=0.0;FREQ_HOMREF=0.9999029973809292;N_BI_GENOS=10309;Southeast_Han_AN=1908;Southeast_Han_AC=1;Sou
chr6	6460020	.	A	<INS:ME:LINE1>	.	PASS	SVTYPE=INS:ME:LINE1;END=6460032;SVLEN=1079;AN=20618;AC=1;AF=4.850130953535746e-05;N_HET=1;N_HOMALT=0;N_HOMREF=10308;FREQ_HET=9.700261907071492e-05;FREQ_HOMALT=0.0;FREQ_HOMREF=0.9999029973809292;N_BI_GENOS=10309;North_Han_AN=2272;North_Han_AC=1;North_Han_A
chr6	6528663	.	T	<INS:ME:LINE1>	.	PASS	SVTYPE=INS:ME:LINE1;END=6528679;SVLEN=1506;AN=20618;AC=7;AF=0.0003395091667475022;N_HET=7;N_HOMALT=0;N_HOMREF=10302;FREQ_HET=0.0006790183334950044;FREQ_HOMALT=0.0;FREQ_HOMREF=0.999320981666505;N_BI_GENOS=10309;South_Han_AN=1496;South_Han_AC=1;South_Han_AF
chr6	6596988	.	T	<INS:ME:LINE1>	.	PASS	SVTYPE=INS:ME:LINE1;END=6597001;SVLEN=95;AN=20618;AC=1;AF=4.850130953535746e-05;N_HET=1;N_HOMALT=0;N_HOMREF=10308;FREQ_HET=9.700261907071492e-05;FREQ_HOMALT=0.0;FREQ_HOMREF=0.9999029973809292;N_BI_GENOS=10309;Mongolian_AN=454;Mongolian_AC=1;Mongolian_AF=0
chr6	6854686	.	T	<INS:ME:LINE1>	.	PASS	SVTYPE=INS:ME:LINE1;END=6854686;SVLEN=5958;AN=20618;AC=2;AF=9.700261907071492e-05;N_HET=2;N_HOMALT=0;N_HOMREF=10307;FREQ_HET=0.00019400523814142983;FREQ_HOMALT=0.0;FREQ_HOMREF=0.9998059947618586;N_BI_GENOS=10309;North_Han_AN=2272;North_Han_AC=1;North_Han_
chr6	7826740	.	A	<INS:ME:LINE1>	.	PASS	SVTYPE=INS:ME:LINE1;END=7826756;SVLEN=752;AN=20618;AC=3;AF=0.00014550392860607236;N_HET=3;N_HOMALT=0;N_HOMREF=10306;FREQ_HET=0.0002910078572121447;FREQ_HOMALT=0.0;FREQ_HOMREF=0.9997089921427879;N_BI_GENOS=10309;South_Han_AN=1496;South_Han_AC=1;South_Han_A
chr6	8242365	.	A	<INS:ME:LINE1>	.	PASS	SVTYPE=INS:ME:LINE1;END=8242397;SVLEN=6000;AN=20618;AC=9;AF=0.0004365117858182171;N_HET=9;N_HOMALT=0;N_HOMREF=10300;FREQ_HET=0.0008730235716364342;FREQ_HOMALT=0.0;FREQ_HOMREF=0.9991269764283636;N_BI_GENOS=10309;Northwest_Han_AN=1296;Northwest_Han_AC=2;Nor
chr6	8361962	.	A	<INS:ME:LINE1>	.	PASS	SVTYPE=INS:ME:LINE1;END=8361962;SVLEN=0;AN=20618;AC=1;AF=4.850130953535746e-05;N_HET=1;N_HOMALT=0;N_HOMREF=10308;FREQ_HET=9.700261907071492e-05;FREQ_HOMALT=0.0;FREQ_HOMREF=0.9999029973809292;N_BI_GENOS=10309;Lingnan_Han_AN=1888;Lingnan_Han_AC=1;Lingnan_Ha
chr6	8373444	.	T	<INS:ME:LINE1>	.	PASS	SVTYPE=INS:ME:LINE1;END=8373444;SVLEN=42;AN=20618;AC=1;AF=4.850130953535746e-05;N_HET=1;N_HOMALT=0;N_HOMREF=10308;FREQ_HET=9.700261907071492e-05;FREQ_HOMALT=0.0;FREQ_HOMREF=0.9999029973809292;N_BI_GENOS=10309;South_Han_AN=1496;South_Han_AC=1;South_Han_AF=
chr6	8524737	.	A	<INS:ME:LINE1>	.	PASS	SVTYPE=INS:ME:LINE1;END=8524752;SVLEN=3902;AN=20618;AC=1;AF=4.850130953535746e-05;N_HET=1;N_HOMALT=0;N_HOMREF=10308;FREQ_HET=9.700261907071492e-05;FREQ_HOMALT=0.0;FREQ_HOMREF=0.9999029973809292;N_BI_GENOS=10309;South_Han_AN=1496;South_Han_AC=1;South_Han_A
chr6	8610827	.	T	<INS:ME:LINE1>	.	PASS	SVTYPE=INS:ME:LINE1;END=8610841;SVLEN=1382;AN=20618;AC=21;AF=0.0010185275002425065;N_HET=21;N_HOMALT=0;N_HOMREF=10288;FREQ_HET=0.002037055000485013;FREQ_HOMALT=0.0;FREQ_HOMREF=0.997962944999515;N_BI_GENOS=10309;South_Han_AN=1496;South_Han_AC=4;South_Han_A
chr6	8887870	.	A	<INS:ME:LINE1>	.	PASS	SVTYPE=INS:ME:LINE1;END=8887886;SVLEN=5999;AN=20618;AC=1;AF=4.850130953535746e-05;N_HET=1;N_HOMALT=0;N_HOMREF=10308;FREQ_HET=9.700261907071492e-05;FREQ_HOMALT=0.0;FREQ_HOMREF=0.9999029973809292;N_BI_GENOS=10309;East_Han_AN=6554;East_Han_AC=1;East_Han_AF=0
chr6	8903129	.	A	<INS:ME:LINE1>	.	PASS	SVTYPE=INS:ME:LINE1;END=8903146;SVLEN=877;AN=20618;AC=2;AF=9.700261907071492e-05;N_HET=2;N_HOMALT=0;N_HOMREF=10307;FREQ_HET=0.00019400523814142983;FREQ_HOMALT=0.0;FREQ_HOMREF=0.9998059947618586;N_BI_GENOS=10309;Northwest_Han_AN=1296;Northwest_Han_AC=1;Nor
chr6	9141117	.	G	<INS:ME:LINE1>	.	PASS	SVTYPE=INS:ME:LINE1;END=9141132;SVLEN=655;AN=20618;AC=1;AF=4.850130953535746e-05;N_HET=1;N_HOMALT=0;N_HOMREF=10308;FREQ_HET=9.700261907071492e-05;FREQ_HOMALT=0.0;FREQ_HOMREF=0.9999029973809292;N_BI_GENOS=10309;Northwest_Han_AN=1296;Northwest_Han_AC=1;Nort
chr6	9152227	.	G	<INS:ME:LINE1>	.	PASS	SVTYPE=INS:ME:LINE1;END=9152227;SVLEN=0;AN=20618;AC=22;AF=0.001067028809777864;N_HET=20;N_HOMALT=1;N_HOMREF=10288;FREQ_HET=0.0019400523814142982;FREQ_HOMALT=9.700261907071492e-05;FREQ_HOMREF=0.997962944999515;N_BI_GENOS=10309;South_Han_AN=1496;South_Han_A
chr6	9250996	.	G	<INS:ME:LINE1>	.	PASS	SVTYPE=INS:ME:LINE1;END=9251012;SVLEN=1100;AN=20618;AC=1;AF=4.850130953535746e-05;N_HET=1;N_HOMALT=0;N_HOMREF=10308;FREQ_HET=9.700261907071492e-05;FREQ_HOMALT=0.0;FREQ_HOMREF=0.9999029973809292;N_BI_GENOS=10309;Miao_AN=482;Miao_AC=1;Miao_AF=0.002074688796
chr6	9328516	.	G	<INS:ME:LINE1>	.	PASS	SVTYPE=INS:ME:LINE1;END=9328529;SVLEN=694;AN=20618;AC=11;AF=0.000533514404888932;N_HET=11;N_HOMALT=0;N_HOMREF=10298;FREQ_HET=0.001067028809777864;FREQ_HOMALT=0.0;FREQ_HOMREF=0.9989329711902222;N_BI_GENOS=10309;South_Han_AN=1496;South_Han_AC=1;South_Han_AF
chr6	9373653	.	A	<INS:ME:LINE1>	.	PASS	SVTYPE=INS:ME:LINE1;END=9373662;SVLEN=6028;AN=20618;AC=2;AF=9.700261907071492e-05;N_HET=2;N_HOMALT=0;N_HOMREF=10307;FREQ_HET=0.00019400523814142983;FREQ_HOMALT=0.0;FREQ_HOMREF=0.9998059947618586;N_BI_GENOS=10309;East_Han_AN=6554;East_Han_AC=2;East_Han_AF=
chr6	9432697	.	A	<INS:ME:LINE1>	.	PASS	SVTYPE=INS:ME:LINE1;END=9432714;SVLEN=6083;AN=20618;AC=1;AF=4.850130953535746e-05;N_HET=1;N_HOMALT=0;N_HOMREF=10308;FREQ_HET=9.700261907071492e-05;FREQ_HOMALT=0.0;FREQ_HOMREF=0.9999029973809292;N_BI_GENOS=10309;Lingnan_Han_AN=1888;Lingnan_Han_AC=1;Lingnan
chr6	9609320	.	G	<INS:ME:LINE1>	.	PASS	SVTYPE=INS:ME:LINE1;END=9609335;SVLEN=107;AN=20618;AC=1;AF=4.850130953535746e-05;N_HET=1;N_HOMALT=0;N_HOMREF=10308;FREQ_HET=9.700261907071492e-05;FREQ_HOMALT=0.0;FREQ_HOMREF=0.9999029973809292;N_BI_GENOS=10309;North_Han_AN=2272;North_Han_AC=1;North_Han_AF
chr6	9702204	.	A	<INS:ME:LINE1>	.	PASS	SVTYPE=INS:ME:LINE1;END=9702219;SVLEN=1723;AN=20618;AC=1;AF=4.850130953535746e-05;N_HET=1;N_HOMALT=0;N_HOMREF=10308;FREQ_HET=9.700261907071492e-05;FREQ_HOMALT=0.0;FREQ_HOMREF=0.9999029973809292;N_BI_GENOS=10309;Lingnan_Han_AN=1888;Lingnan_Han_AC=1;Lingnan
chr6	9708925	.	A	<INS:ME:LINE1>	.	PASS	SVTYPE=INS:ME:LINE1;END=9708940;SVLEN=1523;AN=20618;AC=1;AF=4.850130953535746e-05;N_HET=1;N_HOMALT=0;N_HOMREF=10308;FREQ_HET=9.700261907071492e-05;FREQ_HOMALT=0.0;FREQ_HOMREF=0.9999029973809292;N_BI_GENOS=10309;Northwest_Han_AN=1296;Northwest_Han_AC=1;Nor
chr6	9835298	.	T	<INS:ME:LINE1>	.	PASS	SVTYPE=INS:ME:LINE1;END=9835313;SVLEN=5999;AN=20618;AC=1;AF=4.850130953535746e-05;N_HET=1;N_HOMALT=0;N_HOMREF=10308;FREQ_HET=9.700261907071492e-05;FREQ_HOMALT=0.0;FREQ_HOMREF=0.9999029973809292;N_BI_GENOS=10309;East_Han_AN=6554;East_Han_AC=1;East_Han_AF=0
chr6	9915797	.	T	<INS:ME:LINE1>	.	PASS	SVTYPE=INS:ME:LINE1;END=9915808;SVLEN=311;AN=20618;AC=1;AF=4.850130953535746e-05;N_HET=1;N_HOMALT=0;N_HOMREF=10308;FREQ_HET=9.700261907071492e-05;FREQ_HOMALT=0.0;FREQ_HOMREF=0.9999029973809292;N_BI_GENOS=10309;Central_Han_AN=2128;Central_Han_AC=1;Central_
chr6	10103767	.	T	<INS:ME:LINE1>	.	PASS	SVTYPE=INS:ME:LINE1;END=10103784;SVLEN=780;AN=20618;AC=1;AF=4.850130953535746e-05;N_HET=1;N_HOMALT=0;N_HOMREF=10308;FREQ_HET=9.700261907071492e-05;FREQ_HOMALT=0.0;FREQ_HOMREF=0.9999029973809292;N_BI_GENOS=10309;East_Han_AN=6554;East_Han_AC=1;East_Han_AF=0
chr6	10949721	.	T	<INS:ME:LINE1>	.	PASS	SVTYPE=INS:ME:LINE1;END=10949737;SVLEN=5988;AN=20618;AC=1;AF=4.850130953535746e-05;N_HET=1;N_HOMALT=0;N_HOMREF=10308;FREQ_HET=9.700261907071492e-05;FREQ_HOMALT=0.0;FREQ_HOMREF=0.9999029973809292;N_BI_GENOS=10309;North_Han_AN=2272;North_Han_AC=1;North_Han_
chr6	11445790	.	C	<INS:ME:LINE1>	.	PASS	SVTYPE=INS:ME:LINE1;END=11445805;SVLEN=135;AN=20618;AC=1;AF=4.850130953535746e-05;N_HET=1;N_HOMALT=0;N_HOMREF=10308;FREQ_HET=9.700261907071492e-05;FREQ_HOMALT=0.0;FREQ_HOMREF=0.9999029973809292;N_BI_GENOS=10309;Tibetan_AN=414;Tibetan_AC=1;Tibetan_AF=0.002
chr6	12154828	.	A	<INS:ME:LINE1>	.	PASS	SVTYPE=INS:ME:LINE1;END=12154844;SVLEN=462;AN=20618;AC=2;AF=9.700261907071492e-05;N_HET=2;N_HOMALT=0;N_HOMREF=10307;FREQ_HET=0.00019400523814142983;FREQ_HOMALT=0.0;FREQ_HOMREF=0.9998059947618586;N_BI_GENOS=10309;North_Han_AN=2272;North_Han_AC=2;North_Han_
chr6	12268559	.	G	<INS:ME:LINE1>	.	PASS	SVTYPE=INS:ME:LINE1;END=12268603;SVLEN=1400;AN=20618;AC=1;AF=4.850130953535746e-05;N_HET=1;N_HOMALT=0;N_HOMREF=10308;FREQ_HET=9.700261907071492e-05;FREQ_HOMALT=0.0;FREQ_HOMREF=0.9999029973809292;N_BI_GENOS=10309;East_Han_AN=6554;East_Han_AC=1;East_Han_AF=
chr6	12657223	.	A	<INS:ME:LINE1>	.	PASS	SVTYPE=INS:ME:LINE1;END=12657239;SVLEN=955;AN=20618;AC=1;AF=4.850130953535746e-05;N_HET=1;N_HOMALT=0;N_HOMREF=10308;FREQ_HET=9.700261907071492e-05;FREQ_HOMALT=0.0;FREQ_HOMREF=0.9999029973809292;N_BI_GENOS=10309;North_Han_AN=2272;North_Han_AC=1;North_Han_A
chr6	13190783	.	A	<INS:ME:LINE1>	.	PASS	SVTYPE=INS:ME:LINE1;END=13190801;SVLEN=5999;AN=20618;AC=4118;AF=0.199728392666602;N_HET=2996;N_HOMALT=561;N_HOMREF=6752;FREQ_HET=0.2906198467358619;FREQ_HOMALT=0.054418469298671064;FREQ_HOMREF=0.6549616839654671;N_BI_GENOS=10309;South_Han_AN=1496;South_Ha
chr6	13190912	.	C	<INS:ME:LINE1>	.	PASS	SVTYPE=INS:ME:LINE1;END=13190930;SVLEN=4588;AN=20618;AC=2;AF=9.700261907071492e-05;N_HET=2;N_HOMALT=0;N_HOMREF=10307;FREQ_HET=0.00019400523814142983;FREQ_HOMALT=0.0;FREQ_HOMREF=0.9998059947618586;N_BI_GENOS=10309;East_Han_AN=6554;East_Han_AC=2;East_Han_AF
chr6	13191214	.	G	<INS:ME:LINE1>	.	PASS	SVTYPE=INS:ME:LINE1;END=13191214;SVLEN=0;AN=20618;AC=17;AF=0.0008245222621010767;N_HET=17;N_HOMALT=0;N_HOMREF=10292;FREQ_HET=0.0016490445242021534;FREQ_HOMALT=0.0;FREQ_HOMREF=0.9983509554757979;N_BI_GENOS=10309;South_Han_AN=1496;South_Han_AC=1;South_Han_A
chr6	13199788	.	A	<INS:ME:LINE1>	.	PASS	SVTYPE=INS:ME:LINE1;END=13199802;SVLEN=5998;AN=20618;AC=1;AF=4.850130953535746e-05;N_HET=1;N_HOMALT=0;N_HOMREF=10308;FREQ_HET=9.700261907071492e-05;FREQ_HOMALT=0.0;FREQ_HOMREF=0.9999029973809292;N_BI_GENOS=10309;Lingnan_Han_AN=1888;Lingnan_Han_AC=1;Lingna
chr6	13502682	.	T	<INS:ME:LINE1>	.	PASS	SVTYPE=INS:ME:LINE1;END=13502682;SVLEN=5971;AN=20618;AC=68;AF=0.0032980890484043067;N_HET=30;N_HOMALT=19;N_HOMREF=10260;FREQ_HET=0.0029100785721214474;FREQ_HOMALT=0.0018430497623435833;FREQ_HOMREF=0.9952468716655349;N_BI_GENOS=10309;South_Han_AN=1496;Sout

chr6	13502803	.	A	<INS:ME:LINE1>	.	PASS	SVTYPE=INS:ME:LINE1;END=13502817;SVLEN=5997;AN=20618;AC=14234;AF=0.690367639926278;N_HET=2756;N_HOMALT=5739;N_HOMREF=1814;FREQ_HET=0.2673392181588903;FREQ_HOMALT=0.5566980308468329;FREQ_HOMREF=0.17596275099427686;N_BI_GENOS=10309;South_Han_AN=1496;South_H
chr6	14412145	.	A	<INS:ME:LINE1>	.	PASS	SVTYPE=INS:ME:LINE1;END=14412161;SVLEN=5998;AN=20618;AC=9;AF=0.0004365117858182171;N_HET=9;N_HOMALT=0;N_HOMREF=10300;FREQ_HET=0.0008730235716364342;FREQ_HOMALT=0.0;FREQ_HOMREF=0.9991269764283636;N_BI_GENOS=10309;East_Han_AN=6554;East_Han_AC=7;East_Han_AF=
chr6	14591858	.	G	<INS:ME:LINE1>	.	PASS	SVTYPE=INS:ME:LINE1;END=14591873;SVLEN=5996;AN=20618;AC=28;AF=0.0013580366669900088;N_HET=26;N_HOMALT=1;N_HOMREF=10282;FREQ_HET=0.0025220680958385876;FREQ_HOMALT=9.700261907071492e-05;FREQ_HOMREF=0.9973809292850907;N_BI_GENOS=10309;South_Han_AN=1496;South
chr6	16417453	.	C	<INS:ME:LINE1>	.	PASS	SVTYPE=INS:ME:LINE1;END=16417453;SVLEN=110;AN=20618;AC=861;AF=0.04175962750994277;N_HET=799;N_HOMALT=31;N_HOMREF=9479;FREQ_HET=0.07750509263750122;FREQ_HOMALT=0.0030070811911921624;FREQ_HOMREF=0.9194878261713066;N_BI_GENOS=10309;South_Han_AN=1496;South_Ha
chr6	16752893	.	A	<INS:ME:LINE1>	.	PASS	SVTYPE=INS:ME:LINE1;END=16752897;SVLEN=751;AN=20618;AC=1;AF=4.850130953535746e-05;N_HET=1;N_HOMALT=0;N_HOMREF=10308;FREQ_HET=9.700261907071492e-05;FREQ_HOMALT=0.0;FREQ_HOMREF=0.9999029973809292;N_BI_GENOS=10309;Central_Han_AN=2128;Central_Han_AC=1;Central
chr6	16918790	.	T	<INS:ME:LINE1>	.	PASS	SVTYPE=INS:ME:LINE1;END=16918794;SVLEN=1343;AN=20618;AC=1;AF=4.850130953535746e-05;N_HET=1;N_HOMALT=0;N_HOMREF=10308;FREQ_HET=9.700261907071492e-05;FREQ_HOMALT=0.0;FREQ_HOMREF=0.9999029973809292;N_BI_GENOS=10309;North_Han_AN=2272;North_Han_AC=1;North_Han_
chr6	16950725	.	C	<INS:ME:LINE1>	.	PASS	SVTYPE=INS:ME:LINE1;END=16950725;SVLEN=599;AN=20618;AC=2393;AF=0.11606363371811039;N_HET=949;N_HOMALT=722;N_HOMREF=8638;FREQ_HET=0.09205548549810845;FREQ_HOMALT=0.07003589096905616;FREQ_HOMREF=0.8379086235328354;N_BI_GENOS=10309;South_Han_AN=1496;South_Ha
chr6	16951435	.	T	<INS:ME:LINE1>	.	PASS	SVTYPE=INS:ME:LINE1;END=16951435;SVLEN=63;AN=20618;AC=2;AF=9.700261907071492e-05;N_HET=2;N_HOMALT=0;N_HOMREF=10307;FREQ_HET=0.00019400523814142983;FREQ_HOMALT=0.0;FREQ_HOMREF=0.9998059947618586;N_BI_GENOS=10309;East_Han_AN=6554;East_Han_AC=1;East_Han_AF=0
chr6	17460194	.	G	<INS:ME:LINE1>	.	PASS	SVTYPE=INS:ME:LINE1;END=17460194;SVLEN=5987;AN=20618;AC=1;AF=4.850130953535746e-05;N_HET=1;N_HOMALT=0;N_HOMREF=10308;FREQ_HET=9.700261907071492e-05;FREQ_HOMALT=0.0;FREQ_HOMREF=0.9999029973809292;N_BI_GENOS=10309;East_Han_AN=6554;East_Han_AC=1;East_Han_AF=
chr6	17843463	.	A	<INS:ME:LINE1>	.	PASS	SVTYPE=INS:ME:LINE1;END=17843476;SVLEN=3364;AN=20618;AC=1;AF=4.850130953535746e-05;N_HET=1;N_HOMALT=0;N_HOMREF=10308;FREQ_HET=9.700261907071492e-05;FREQ_HOMALT=0.0;FREQ_HOMREF=0.9999029973809292;N_BI_GENOS=10309;Central_Han_AN=2128;Central_Han_AC=1;Centra
chr6	17904467	.	A	<INS:ME:LINE1>	.	PASS	SVTYPE=INS:ME:LINE1;END=17904482;SVLEN=6017;AN=20618;AC=1;AF=4.850130953535746e-05;N_HET=1;N_HOMALT=0;N_HOMREF=10308;FREQ_HET=9.700261907071492e-05;FREQ_HOMALT=0.0;FREQ_HOMREF=0.9999029973809292;N_BI_GENOS=10309;Central_Han_AN=2128;Central_Han_AC=1;Centra
chr6	18265439	.	A	<INS:ME:LINE1>	.	PASS	SVTYPE=INS:ME:LINE1;END=18265452;SVLEN=5979;AN=20618;AC=6;AF=0.0002910078572121447;N_HET=6;N_HOMALT=0;N_HOMREF=10303;FREQ_HET=0.0005820157144242894;FREQ_HOMALT=0.0;FREQ_HOMREF=0.9994179842855757;N_BI_GENOS=10309;South_Han_AN=1496;South_Han_AC=1;South_Han_
chr6	18338203	.	G	<INS:ME:LINE1>	.	PASS	SVTYPE=INS:ME:LINE1;END=18338221;SVLEN=363;AN=20618;AC=2;AF=9.700261907071492e-05;N_HET=2;N_HOMALT=0;N_HOMREF=10307;FREQ_HET=0.00019400523814142983;FREQ_HOMALT=0.0;FREQ_HOMREF=0.9998059947618586;N_BI_GENOS=10309;Miao_AN=482;Miao_AC=2;Miao_AF=0.00414937759
chr6	18481271	.	A	<INS:ME:LINE1>	.	PASS	SVTYPE=INS:ME:LINE1;END=18481285;SVLEN=453;AN=20618;AC=1;AF=4.850130953535746e-05;N_HET=1;N_HOMALT=0;N_HOMREF=10308;FREQ_HET=9.700261907071492e-05;FREQ_HOMALT=0.0;FREQ_HOMREF=0.9999029973809292;N_BI_GENOS=10309;North_Han_AN=2272;North_Han_AC=1;North_Han_A
chr6	18571533	.	A	<INS:ME:LINE1>	.	PASS	SVTYPE=INS:ME:LINE1;END=18571533;SVLEN=5990;AN=20618;AC=1;AF=4.850130953535746e-05;N_HET=1;N_HOMALT=0;N_HOMREF=10308;FREQ_HET=9.700261907071492e-05;FREQ_HOMALT=0.0;FREQ_HOMREF=0.9999029973809292;N_BI_GENOS=10309;Yi_AN=422;Yi_AC=1;Yi_AF=0.00236966824644549
chr6	18700762	.	A	<INS:ME:LINE1>	.	PASS	SVTYPE=INS:ME:LINE1;END=18700764;SVLEN=767;AN=20618;AC=4;AF=0.00019400523814142983;N_HET=4;N_HOMALT=0;N_HOMREF=10305;FREQ_HET=0.0003880104762828596 6;FREQ_HOMALT=0.0;FREQ_HOMREF=0.9996119895237171;N_BI_GENOS=10309;South_Han_AN=1496;South_Han_AC=1;South_Han
chr6	18757083	.	T	<INS:ME:LINE1>	.	PASS	SVTYPE=INS:ME:LINE1;END=18757083;SVLEN=0;AN=20618;AC=2;AF=9.700261907071492e-05;N_HET=2;N_HOMALT=0;N_HOMREF=10307;FREQ_HET=0.00019400523814142983;FREQ_HOMALT=0.0;FREQ_HOMREF=0.9998059947618586;N_BI_GENOS=10309;East_Han_AN=6554;East_Han_AC=1;East_Han_AF=0.
chr6	18799644	.	T	<INS:ME:LINE1>	.	PASS	SVTYPE=INS:ME:LINE1;END=18799661;SVLEN=5998;AN=20618;AC=5;AF=0.00024250654767678728;N_HET=5;N_HOMALT=0;N_HOMREF=10304;FREQ_HET=0.00048501309535357455;FREQ_HOMALT=0.0;FREQ_HOMREF=0.9995149869046465;N_BI_GENOS=10309;South_Han_AN=1496;South_Han_AC=2;South_Ha
chr6	18893706	.	A	<INS:ME:LINE1>	.	PASS	SVTYPE=INS:ME:LINE1;END=18893716;SVLEN=717;AN=20618;AC=5;AF=0.00024250654767678728;N_HET=5;N_HOMALT=0;N_HOMREF=10304;FREQ_HET=0.00048501309535357455;FREQ_HOMALT=0.0;FREQ_HOMREF=0.9995149869046465;N_BI_GENOS=10309;North_Han_AN=2272;North_Han_AC=1;North_Han
chr6	19056528	.	C	<INS:ME:LINE1>	.	PASS	SVTYPE=INS:ME:LINE1;END=19056528;SVLEN=1018;AN=20618;AC=1;AF=4.850130953535746e-05;N_HET=1;N_HOMALT=0;N_HOMREF=10308;FREQ_HET=9.700261907071492e-05;FREQ_HOMALT=0.0;FREQ_HOMREF=0.9999029973809292;N_BI_GENOS=10309;East_Han_AN=6554;East_Han_AC=1;East_Han_AF=
chr6	19215702	.	T	<INS:ME:LINE1>	.	PASS	SVTYPE=INS:ME:LINE1;END=19215717;SVLEN=6053;AN=20618;AC=1;AF=4.850130953535746e-05;N_HET=1;N_HOMALT=0;N_HOMREF=10308;FREQ_HET=9.700261907071492e-05;FREQ_HOMALT=0.0;FREQ_HOMREF=0.9999029973809292;N_BI_GENOS=10309;North_Han_AN=2272;North_Han_AC=1;North_Han_
chr6	19257980	.	A	<INS:ME:LINE1>	.	PASS	SVTYPE=INS:ME:LINE1;END=19257995;SVLEN=328;AN=20618;AC=39;AF=0.0018915510718789407;N_HET=39;N_HOMALT=0;N_HOMREF=10270;FREQ_HET=0.0037831021437578815;FREQ_HOMALT=0.0;FREQ_HOMREF=0.9962168978562421;N_BI_GENOS=10309;South_Han_AN=1496;South_Han_AC=1;South_Han
chr6	19648437	.	A	<INS:ME:LINE1>	.	PASS	SVTYPE=INS:ME:LINE1;END=19648447;SVLEN=1275;AN=20618;AC=1;AF=4.850130953535746e-05;N_HET=1;N_HOMALT=0;N_HOMREF=10308;FREQ_HET=9.700261907071492e-05;FREQ_HOMALT=0.0;FREQ_HOMREF=0.9999029973809292;N_BI_GENOS=10309;East_Han_AN=6554;East_Han_AC=1;East_Han_AF=
chr6	19792883	.	A	<INS:ME:LINE1>	.	PASS	SVTYPE=INS:ME:LINE1;END=19792898;SVLEN=5998;AN=20618;AC=16;AF=0.0007760209525657193;N_HET=16;N_HOMALT=0;N_HOMREF=10293;FREQ_HET=0.0015520419051314386;FREQ_HOMALT=0.0;FREQ_HOMREF=0.9984479580948685;N_BI_GENOS=10309;Northwest_Han_AN=1296;Northwest_Han_AC=6;
chr6	20158900	.	A	<INS:ME:LINE1>	.	PASS	SVTYPE=INS:ME:LINE1;END=20158914;SVLEN=5998;AN=20618;AC=1;AF=4.850130953535746e-05;N_HET=1;N_HOMALT=0;N_HOMREF=10308;FREQ_HET=9.700261907071492e-05;FREQ_HOMALT=0.0;FREQ_HOMREF=0.9999029973809292;N_BI_GENOS=10309;South_Han_AN=1496;South_Han_AC=1;South_Han_
chr6	20200424	.	C	<INS:ME:LINE1>	.	PASS	SVTYPE=INS:ME:LINE1;END=20200437;SVLEN=5998;AN=20618;AC=5;AF=0.00024250654767678728;N_HET=5;N_HOMALT=0;N_HOMREF=10304;FREQ_HET=0.00048501309535357455;FREQ_HOMALT=0.0;FREQ_HOMREF=0.9995149869046465;N_BI_GENOS=10309;East_Han_AN=6554;East_Han_AC=5;East_Han_A
chr6	20455549	.	A	<INS:ME:LINE1>	.	PASS	SVTYPE=INS:ME:LINE1;END=20455563;SVLEN=1217;AN=20618;AC=1;AF=4.850130953535746e-05;N_HET=1;N_HOMALT=0;N_HOMREF=10308;FREQ_HET=9.700261907071492e-05;FREQ_HOMALT=0.0;FREQ_HOMREF=0.9999029973809292;N_BI_GENOS=10309;East_Han_AN=6554;East_Han_AC=1;East_Han_AF=
chr6	21884775	.	T	<INS:ME:LINE1>	.	PASS	SVTYPE=INS:ME:LINE1;END=21884791;SVLEN=6008;AN=20618;AC=7;AF=0.0003395091667475022;N_HET=7;N_HOMALT=0;N_HOMREF=10302;FREQ_HET=0.0006790183334950044;FREQ_HOMALT=0.0;FREQ_HOMREF=0.999320981666505;N_BI_GENOS=10309;South_Han_AN=1496;South_Han_AC=1;South_Han_A
chr6	22098066	.	T	<INS:ME:LINE1>	.	PASS	SVTYPE=INS:ME:LINE1;END=22098066;SVLEN=368;AN=20618;AC=1;AF=4.850130953535746e-05;N_HET=1;N_HOMALT=0;N_HOMREF=10308;FREQ_HET=9.700261907071492e-05;FREQ_HOMALT=0.0;FREQ_HOMREF=0.9999029973809292;N_BI_GENOS=10309;Lingnan_Han_AN=1888;Lingnan_Han_AC=1;Lingnan
chr6	22287274	.	G	<INS:ME:LINE1>	.	PASS	SVTYPE=INS:ME:LINE1;END=22287287;SVLEN=1419;AN=20618;AC=1;AF=4.850130953535746e-05;N_HET=1;N_HOMALT=0;N_HOMREF=10308;FREQ_HET=9.700261907071492e-05;FREQ_HOMALT=0.0;FREQ_HOMREF=0.9999029973809292;N_BI_GENOS=10309;Lingnan_Han_AN=1888;Lingnan_Han_AC=1;Lingna
chr6	22299931	.	A	<INS:ME:LINE1>	.	PASS	SVTYPE=INS:ME:LINE1;END=22299931;SVLEN=108;AN=20618;AC=2;AF=9.700261907071492e-05;N_HET=0;N_HOMALT=1;N_HOMREF=10308;FREQ_HET=0.0;FREQ_HOMALT=9.700261907071492e-05;FREQ_HOMREF=0.9999029973809292;N_BI_GENOS=10309;Northwest_Han_AN=1296;Northwest_Han_AC=2;Nor
chr6	22841219	.	A	<INS:ME:LINE1>	.	PASS	SVTYPE=INS:ME:LINE1;END=22841244;SVLEN=1883;AN=20618;AC=1;AF=4.850130953535746e-05;N_HET=1;N_HOMALT=0;N_HOMREF=10308;FREQ_HET=9.700261907071492e-05;FREQ_HOMALT=0.0;FREQ_HOMREF=0.9999029973809292;N_BI_GENOS=10309;East_Han_AN=6554;East_Han_AC=1;East_Han_AF=
chr6	22951520	.	T	<INS:ME:LINE1>	.	PASS	SVTYPE=INS:ME:LINE1;END=22951533;SVLEN=209;AN=20618;AC=2;AF=9.700261907071492e-05;N_HET=0;N_HOMALT=1;N_HOMREF=10308;FREQ_HET=0.0;FREQ_HOMALT=9.700261907071492e-05;FREQ_HOMREF=0.9999029973809292;N_BI_GENOS=10309;East_Han_AN=6554;East_Han_AC=2;East_Han_AF=0
chr6	22990964	.	T	<INS:ME:LINE1>	.	PASS	SVTYPE=INS:ME:LINE1;END=22990989;SVLEN=3445;AN=20618;AC=1;AF=4.850130953535746e-05;N_HET=1;N_HOMALT=0;N_HOMREF=10308;FREQ_HET=9.700261907071492e-05;FREQ_HOMALT=0.0;FREQ_HOMREF=0.9999029973809292;N_BI_GENOS=10309;East_Han_AN=6554;East_Han_AC=1;East_Han_AF=
chr6	23175749	.	A	<INS:ME:LINE1>	.	PASS	SVTYPE=INS:ME:LINE1;END=23175749;SVLEN=3425;AN=20618;AC=1;AF=4.850130953535746e-05;N_HET=1;N_HOMALT=0;N_HOMREF=10308;FREQ_HET=9.700261907071492e-05;FREQ_HOMALT=0.0;FREQ_HOMREF=0.9999029973809292;N_BI_GENOS=10309;East_Han_AN=6554;East_Han_AC=1;East_Han_AF=
chr6	23186694	.	A	<INS:ME:LINE1>	.	PASS	SVTYPE=INS:ME:LINE1;END=23186712;SVLEN=6000;AN=20618;AC=1;AF=4.850130953535746e-05;N_HET=1;N_HOMALT=0;N_HOMREF=10308;FREQ_HET=9.700261907071492e-05;FREQ_HOMALT=0.0;FREQ_HOMREF=0.9999029973809292;N_BI_GENOS=10309;Northwest_Han_AN=1296;Northwest_Han_AC=1;No
chr6	23414296	.	G	<INS:ME:LINE1>	.	PASS	SVTYPE=INS:ME:LINE1;END=23414313;SVLEN=412;AN=20618;AC=1;AF=4.850130953535746e-05;N_HET=1;N_HOMALT=0;N_HOMREF=10308;FREQ_HET=9.700261907071492e-05;FREQ_HOMALT=0.0;FREQ_HOMREF=0.9999029973809292;N_BI_GENOS=10309;Yi_AN=422;Yi_AC=1;Yi_AF=0.002369668246445498
chr6	23539276	.	T	<INS:ME:LINE1>	.	PASS	SVTYPE=INS:ME:LINE1;END=23539285;SVLEN=1482;AN=20618;AC=1;AF=4.850130953535746e-05;N_HET=1;N_HOMALT=0;N_HOMREF=10308;FREQ_HET=9.700261907071492e-05;FREQ_HOMALT=0.0;FREQ_HOMREF=0.9999029973809292;N_BI_GENOS=10309;Lingnan_Han_AN=1888;Lingnan_Han_AC=1;Lingna
chr6	23642064	.	A	<INS:ME:LINE1>	.	PASS	SVTYPE=INS:ME:LINE1;END=23642079;SVLEN=644;AN=20618;AC=1;AF=4.850130953535746e-05;N_HET=1;N_HOMALT=0;N_HOMREF=10308;FREQ_HET=9.700261907071492e-05;FREQ_HOMALT=0.0;FREQ_HOMREF=0.9999029973809292;N_BI_GENOS=10309;Mongolian_AN=454;Mongolian_AC=1;Mongolian_AF
chr6	23755647	.	A	<INS:ME:LINE1>	.	PASS	SVTYPE=INS:ME:LINE1;END=23755685;SVLEN=739;AN=20618;AC=3;AF=0.0001455039286060 7236;N_HET=1;N_HOMALT=1;N_HOMREF=10307;FREQ_HET=9.700261907071492e-05;FREQ_HOMALT=9.700261907071492e-05;FREQ_HOMREF=0.9998059947618586;N_BI_GENOS=10309;East_Han_AN=6554;East_Han
chr6	23810067	.	A	<INS:ME:LINE1>	.	PASS	SVTYPE=INS:ME:LINE1;END=23810085;SVLEN=466;AN=20618;AC=1;AF=4.850130953535746e-05;N_HET=1;N_HOMALT=0;N_HOMREF=10308;FREQ_HET=9.700261907071492e-05;FREQ_HOMALT=0.0;FREQ_HOMREF=0.9999029973809292;N_BI_GENOS=10309;East_Han_AN=6554;East_Han_AC=1;East_Han_AF=0
chr6	23838819	.	A	<INS:ME:LINE1>	.	PASS	SVTYPE=INS:ME:LINE1;END=23838834;SVLEN=2030;AN=20618;AC=1;AF=4.850130953535746e-05;N_HET=1;N_HOMALT=0;N_HOMREF=10308;FREQ_HET=9.700261907071492e-05;FREQ_HOMALT=0.0;FREQ_HOMREF=0.9999029973809292;N_BI_GENOS=10309;South_Han_AN=1496;South_Han_AC=1;South_Han_
chr6	24120398	.	T	<INS:ME:LINE1>	.	PASS	SVTYPE=INS:ME:LINE1;END=24120398;SVLEN=6000;AN=20618;AC=1;AF=4.850130953535746e-05;N_HET=1;N_HOMALT=0;N_HOMREF=10308;FREQ_HET=9.700261907071492e-05;FREQ_HOMALT=0.0;FREQ_HOMREF=0.9999029973809292;N_BI_GENOS=10309;Lingnan_Han_AN=1888;Lingnan_Han_AC=1;Lingna
chr6	24189558	.	A	<INS:ME:LINE1>	.	PASS	SVTYPE=INS:ME:LINE1;END=24189573;SVLEN=6031;AN=20618;AC=1;AF=4.850130953535746e-05;N_HET=1;N_HOMALT=0;N_HOMREF=10308;FREQ_HET=9.700261907071492e-05;FREQ_HOMALT=0.0;FREQ_HOMREF=0.9999029973809292;N_BI_GENOS=10309;Northwest_Han_AN=1296;Northwest_Han_AC=1;No
chr6	24190579	.	A	<INS:ME:LINE1>	.	PASS	SVTYPE=INS:ME:LINE1;END=24190592;SVLEN=1143;AN=20618;AC=2;AF=9.700261907071492e-05;N_HET=2;N_HOMALT=0;N_HOMREF=10307;FREQ_HET=0.00019400523814142983;FREQ_HOMALT=0.0;FREQ_HOMREF=0.9998059947618586;N_BI_GENOS=10309;North_Han_AN=2272;North_Han_AC=1;North_Han
chr6	24209490	.	A	<INS:ME:LINE1>	.	PASS	SVTYPE=INS:ME:LINE1;END=24209497;SVLEN=367;AN=20618;AC=1;AF=4.850130953535746e-05;N_HET=1;N_HOMALT=0;N_HOMREF=10308;FREQ_HET=9.700261907071492e-05;FREQ_HOMALT=0.0;FREQ_HOMREF=0.9999029973809292;N_BI_GENOS=10309;East_Han_AN=6554;East_Han_AC=1;East_Han_AF=0
chr6	25085530	.	G	<INS:ME:LINE1>	.	PASS	SVTYPE=INS:ME:LINE1;END=25085544;SVLEN=6005;AN=20618;AC=4;AF=0.00019400523814142983;N_HET=4;N_HOMALT=0;N_HOMREF=10305;FREQ_HET=0.0003880104762828596 6;FREQ_HOMALT=0.0;FREQ_HOMREF=0.9996119895237171;N_BI_GENOS=10309;North_Han_AN=2272;North_Han_AC=1;North_Ha
chr6	25104291	.	A	<INS:ME:LINE1>	.	PASS	SVTYPE=INS:ME:LINE1;END=25104307;SVLEN=5998;AN=20618;AC=1;AF=4.850130953535746e-05;N_HET=1;N_HOMALT=0;N_HOMREF=10308;FREQ_HET=9.700261907071492e-05;FREQ_HOMALT=0.0;FREQ_HOMREF=0.9999029973809292;N_BI_GENOS=10309;Zhuang_AN=444;Zhuang_AC=1;Zhuang_AF=0.00225
chr6	25266027	.	A	<INS:ME:LINE1>	.	PASS	SVTYPE=INS:ME:LINE1;END=25266043;SVLEN=6038;AN=20618;AC=1;AF=4.850130953535746e-05;N_HET=1;N_HOMALT=0;N_HOMREF=10308;FREQ_HET=9.700261907071492e-05;FREQ_HOMALT=0.0;FREQ_HOMREF=0.9999029973809292;N_BI_GENOS=10309;East_Han_AN=6554;East_Han_AC=1;East_Han_AF=
chr6	25524772	.	A	<INS:ME:LINE1>	.	PASS	SVTYPE=INS:ME:LINE1;END=25524782;SVLEN=1003;AN=20618;AC=1;AF=4.850130953535746e-05;N_HET=1;N_HOMALT=0;N_HOMREF=10308;FREQ_HET=9.700261907071492e-05;FREQ_HOMALT=0.0;FREQ_HOMREF=0.9999029973809292;N_BI_GENOS=10309;North_Han_AN=2272;North_Han_AC=1;North_Han_
chr6	25645316	.	T	<INS:ME:LINE1>	.	PASS	SVTYPE=INS:ME:LINE1;END=25645328;SVLEN=5993;AN=20618;AC=1;AF=4.850130953535746e-05;N_HET=1;N_HOMALT=0;N_HOMREF=10308;FREQ_HET=9.700261907071492e-05;FREQ_HOMALT=0.0;FREQ_HOMREF=0.9999029973809292;N_BI_GENOS=10309;North_Han_AN=2272;North_Han_AC=1;North_Han_
chr6	25942385	.	A	<INS:ME:LINE1>	.	PASS	SVTYPE=INS:ME:LINE1;END=25942400;SVLEN=115;AN=20618;AC=1;AF=4.850130953535746e-05;N_HET=1;N_HOMALT=0;N_HOMREF=10308;FREQ_HET=9.700261907071492e-05;FREQ_HOMALT=0.0;FREQ_HOMREF=0.9999029973809292;N_BI_GENOS=10309;Northwest_Han_AN=1296;Northwest_Han_AC=1;Nor
chr6	26845899	.	A	<INS:ME:LINE1>	.	PASS	SVTYPE=INS:ME:LINE1;END=26845913;SVLEN=5989;AN=20618;AC=1;AF=4.850130953535746e-05;N_HET=1;N_HOMALT=0;N_HOMREF=10308;FREQ_HET=9.700261907071492e-05;FREQ_HOMALT=0.0;FREQ_HOMREF=0.9999029973809292;N_BI_GENOS=10309;Hui_AN=444;Hui_AC=1;Hui_AF=0.00225225225225
chr6	27049892	.	A	<INS:ME:LINE1>	.	PASS	SVTYPE=INS:ME:LINE1;END=27049909;SVLEN=506;AN=20618;AC=2;AF=9.700261907071492e-05;N_HET=2;N_HOMALT=0;N_HOMREF=10307;FREQ_HET=0.00019400523814142983;FREQ_HOMALT=0.0;FREQ_HOMREF=0.9998059947618586;N_BI_GENOS=10309;East_Han_AN=6554;East_Han_AC=2;East_Han_AF=
chr6	27845203	.	G	<INS:ME:LINE1>	.	PASS	SVTYPE=INS:ME:LINE1;END=27845203;SVLEN=20;AN=20618;AC=1;AF=4.850130953535746e-05;N_HET=1;N_HOMALT=0;N_HOMREF=10308;FREQ_HET=9.700261907071492e-05;FREQ_HOMALT=0.0;FREQ_HOMREF=0.9999029973809292;N_BI_GENOS=10309;South_Han_AN=1496;South_Han_AC=1;South_Han_AF
chr6	27952272	.	A	<INS:ME:LINE1>	.	PASS	SVTYPE=INS:ME:LINE1;END=27952279;SVLEN=430;AN=20618;AC=2;AF=9.700261907071492e-05;N_HET=2;N_HOMALT=0;N_HOMREF=10307;FREQ_HET=0.00019400523814142983;FREQ_HOMALT=0.0;FREQ_HOMREF=0.9998059947618586;N_BI_GENOS=10309;East_Han_AN=6554;East_Han_AC=1;East_Han_AF=
chr6	27988267	.	T	<INS:ME:LINE1>	.	PASS	SVTYPE=INS:ME:LINE1;END=27988267;SVLEN=6000;AN=20618;AC=16;AF=0.0007760209525657193;N_HET=8;N_HOMALT=4;N_HOMREF=10297;FREQ_HET=0.0007760209525657193;FREQ_HOMALT=0.0003880104762828596 6;FREQ_HOMREF=0.9988359685711514;N_BI_GENOS=10309;South_Han_AN=1496;South
chr6	28033834	.	T	<INS:ME:LINE1>	.	PASS	SVTYPE=INS:ME:LINE1;END=28033851;SVLEN=718;AN=20618;AC=1;AF=4.850130953535746e-05;N_HET=1;N_HOMALT=0;N_HOMREF=10308;FREQ_HET=9.700261907071492e-05;FREQ_HOMALT=0.0;FREQ_HOMREF=0.9999029973809292;N_BI_GENOS=10309;Northwest_Han_AN=1296;Northwest_Han_AC=1;Nor
chr6	33760994	.	G	<INS:ME:LINE1>	.	PASS	SVTYPE=INS:ME:LINE1;END=33761009;SVLEN=472;AN=20618;AC=22;AF=0.001067028809777864;N_HET=22;N_HOMALT=0;N_HOMREF=10287;FREQ_HET=0.002134057619555728;FREQ_HOMALT=0.0;FREQ_HOMREF=0.9978659423804442;N_BI_GENOS=10309;South_Han_AN=1496;South_Han_AC=2;South_Han_A
chr6	35702387	.	A	<INS:ME:LINE1>	.	PASS	SVTYPE=INS:ME:LINE1;END=35702404;SVLEN=88;AN=20618;AC=1;AF=4.850130953535746e-05;N_HET=1;N_HOMALT=0;N_HOMREF=10308;FREQ_HET=9.700261907071492e-05;FREQ_HOMALT=0.0;FREQ_HOMREF=0.9999029973809292;N_BI_GENOS=10309;Northwest_Han_AN=1296;Northwest_Han_AC=1;Nort
chr6	36174540	.	G	<INS:ME:LINE1>	.	PASS	SVTYPE=INS:ME:LINE1;END=36174540;SVLEN=67;AN=20618;AC=1;AF=4.850130953535746e-05;N_HET=1;N_HOMALT=0;N_HOMREF=10308;FREQ_HET=9.700261907071492e-05;FREQ_HOMALT=0.0;FREQ_HOMREF=0.9999029973809292;N_BI_GENOS=10309;Yi_AN=422;Yi_AC=1;Yi_AF=0.002369668246445498;
chr6	37953928	.	A	<INS:ME:LINE1>	.	PASS	SVTYPE=INS:ME:LINE1;END=37953946;SVLEN=1022;AN=20618;AC=1;AF=4.850130953535746e-05;N_HET=1;N_HOMALT=0;N_HOMREF=10308;FREQ_HET=9.700261907071492e-05;FREQ_HOMALT=0.0;FREQ_HOMREF=0.9999029973809292;N_BI_GENOS=10309;East_Han_AN=6554;East_Han_AC=1;East_Han_AF=
chr6	38034821	.	A	<INS:ME:LINE1>	.	PASS	SVTYPE=INS:ME:LINE1;END=38034834;SVLEN=759;AN=20618;AC=2;AF=9.700261907071492e-05;N_HET=2;N_HOMALT=0;N_HOMREF=10307;FREQ_HET=0.00019400523814142983;FREQ_HOMALT=0.0;FREQ_HOMREF=0.9998059947618586;N_BI_GENOS=10309;East_Han_AN=6554;East_Han_AC=2;East_Han_AF=
chr6	38491820	.	A	<INS:ME:LINE1>	.	PASS	SVTYPE=INS:ME:LINE1;END=38491835;SVLEN=5986;AN=20618;AC=1;AF=4.850130953535746e-05;N_HET=1;N_HOMALT=0;N_HOMREF=10308;FREQ_HET=9.700261907071492e-05;FREQ_HOMALT=0.0;FREQ_HOMREF=0.9999029973809292;N_BI_GENOS=10309;Lingnan_Han_AN=1888;Lingnan_Han_AC=1;Lingna
chr6	38568722	.	T	<INS:ME:LINE1>	.	PASS	SVTYPE=INS:ME:LINE1;END=38568736;SVLEN=334;AN=20618;AC=1;AF=4.850130953535746e-05;N_HET=1;N_HOMALT=0;N_HOMREF=10308;FREQ_HET=9.700261907071492e-05;FREQ_HOMALT=0.0;FREQ_HOMREF=0.9999029973809292;N_BI_GENOS=10309;North_Han_AN=2272;North_Han_AC=1;North_Han_A
chr6	38627932	.	A	<INS:ME:LINE1>	.	PASS	SVTYPE=INS:ME:LINE1;END=38627946;SVLEN=6070;AN=20618;AC=1;AF=4.850130953535746e-05;N_HET=1;N_HOMALT=0;N_HOMREF=10308;FREQ_HET=9.700261907071492e-05;FREQ_HOMALT=0.0;FREQ_HOMREF=0.9999029973809292;N_BI_GENOS=10309;East_Han_AN=6554;East_Han_AC=1;East_Han_AF=
chr6	38732675	.	C	<INS:ME:LINE1>	.	PASS	SVTYPE=INS:ME:LINE1;END=38732694;SVLEN=5980;AN=20618;AC=1;AF=4.850130953535746e-05;N_HET=1;N_HOMALT=0;N_HOMREF=10308;FREQ_HET=9.700261907071492e-05;FREQ_HOMALT=0.0;FREQ_HOMREF=0.9999029973809292;N_BI_GENOS=10309;Zhuang_AN=444;Zhuang_AC=1;Zhuang_AF=0.00225
chr6	38733614	.	A	<INS:ME:LINE1>	.	PASS	SVTYPE=INS:ME:LINE1;END=38733627;SVLEN=6000;AN=20618;AC=1;AF=4.850130953535746e-05;N_HET=1;N_HOMALT=0;N_HOMREF=10308;FREQ_HET=9.700261907071492e-05;FREQ_HOMALT=0.0;FREQ_HOMREF=0.9999029973809292;N_BI_GENOS=10309;Central_Han_AN=2128;Central_Han_AC=1;Centra
chr6	38735133	.	G	<INS:ME:LINE1>	.	PASS	SVTYPE=INS:ME:LINE1;END=38735148;SVLEN=5998;AN=20618;AC=5;AF=0.00024250654767678728;N_HET=5;N_HOMALT=0;N_HOMREF=10304;FREQ_HET=0.00048501309535357455;FREQ_HOMALT=0.0;FREQ_HOMREF=0.9995149869046465;N_BI_GENOS=10309;East_Han_AN=6554;East_Han_AC=3;East_Han_A
chr6	38812366	.	T	<INS:ME:LINE1>	.	PASS	SVTYPE=INS:ME:LINE1;END=38812379;SVLEN=5998;AN=20618;AC=1;AF=4.850130953535746e-05;N_HET=1;N_HOMALT=0;N_HOMREF=10308;FREQ_HET=9.700261907071492e-05;FREQ_HOMALT=0.0;FREQ_HOMREF=0.9999029973809292;N_BI_GENOS=10309;Northwest_Han_AN=1296;Northwest_Han_AC=1;No
chr6	39485320	.	A	<INS:ME:LINE1>	.	PASS	SVTYPE=INS:ME:LINE1;END=39485320;SVLEN=5983;AN=20618;AC=2;AF=9.700261907071492e-05;N_HET=2;N_HOMALT=0;N_HOMREF=10307;FREQ_HET=0.00019400523814142983;FREQ_HOMALT=0.0;FREQ_HOMREF=0.9998059947618586;N_BI_GENOS=10309;Lingnan_Han_AN=1888;Lingnan_Han_AC=1;Lingn
chr6	40297349	.	A	<INS:ME:LINE1>	.	PASS	SVTYPE=INS:ME:LINE1;END=40297359;SVLEN=2825;AN=20618;AC=1;AF=4.850130953535746e-05;N_HET=1;N_HOMALT=0;N_HOMREF=10308;FREQ_HET=9.700261907071492e-05;FREQ_HOMALT=0.0;FREQ_HOMREF=0.9999029973809292;N_BI_GENOS=10309;Northwest_Han_AN=1296;Northwest_Han_AC=1;No
chr6	40483561	.	T	<INS:ME:LINE1>	.	PASS	SVTYPE=INS:ME:LINE1;END=40483580;SVLEN=75;AN=20618;AC=2;AF=9.700261907071492e-05;N_HET=2;N_HOMALT=0;N_HOMREF=10307;FREQ_HET=0.00019400523814142983;FREQ_HOMALT=0.0;FREQ_HOMREF=0.9998059947618586;N_BI_GENOS=10309;Central_Han_AN=2128;Central_Han_AC=1;Central
chr6	40689293	.	A	<INS:ME:LINE1>	.	PASS	SVTYPE=INS:ME:LINE1;END=40689308;SVLEN=1401;AN=20618;AC=1;AF=4.850130953535746e-05;N_HET=1;N_HOMALT=0;N_HOMREF=10308;FREQ_HET=9.700261907071492e-05;FREQ_HOMALT=0.0;FREQ_HOMREF=0.9999029973809292;N_BI_GENOS=10309;North_Han_AN=2272;North_Han_AC=1;North_Han_
chr6	40772497	.	A	<INS:ME:LINE1>	.	PASS	SVTYPE=INS:ME:LINE1;END=40772510;SVLEN=150;AN=20618;AC=19;AF=0.0009215248811717916;N_HET=19;N_HOMALT=0;N_HOMREF=10290;FREQ_HET=0.0018430497623435833;FREQ_HOMALT=0.0;FREQ_HOMREF=0.9981569502376564;N_BI_GENOS=10309;South_Han_AN=1496;South_Han_AC=3;South_Han
chr6	41154865	.	G	<INS:ME:LINE1>	.	PASS	SVTYPE=INS:ME:LINE1;END=41154885;SVLEN=818;AN=20618;AC=1;AF=4.850130953535746e-05;N_HET=1;N_HOMALT=0;N_HOMREF=10308;FREQ_HET=9.700261907071492e-05;FREQ_HOMALT=0.0;FREQ_HOMREF=0.9999029973809292;N_BI_GENOS=10309;East_Han_AN=6554;East_Han_AC=1;East_Han_AF=0
chr6	41175446	.	A	<INS:ME:LINE1>	.	PASS	SVTYPE=INS:ME:LINE1;END=41175461;SVLEN=6024;AN=20618;AC=1;AF=4.850130953535746e-05;N_HET=1;N_HOMALT=0;N_HOMREF=10308;FREQ_HET=9.700261907071492e-05;FREQ_HOMALT=0.0;FREQ_HOMREF=0.9999029973809292;N_BI_GENOS=10309;Lingnan_Han_AN=1888;Lingnan_Han_AC=1;Lingna
chr6	42061468	.	T	<INS:ME:LINE1>	.	PASS	SVTYPE=INS:ME:LINE1;END=42061482;SVLEN=296;AN=20618;AC=3;AF=0.0001455039286060 7236;N_HET=3;N_HOMALT=0;N_HOMREF=10306;FREQ_HET=0.0002910078572121447;FREQ_HOMALT=0.0;FREQ_HOMREF=0.9997089921427879;N_BI_GENOS=10309;East_Han_AN=6554;East_Han_AC=3;East_Han_AF=
chr6	42245944	.	T	<INS:ME:LINE1>	.	PASS	SVTYPE=INS:ME:LINE1;END=42245961;SVLEN=6039;AN=20618;AC=1;AF=4.850130953535746e-05;N_HET=1;N_HOMALT=0;N_HOMREF=10308;FREQ_HET=9.700261907071492e-05;FREQ_HOMALT=0.0;FREQ_HOMREF=0.9999029973809292;N_BI_GENOS=10309;Central_Han_AN=2128;Central_Han_AC=1;Centra
chr6	42346021	.	A	<INS:ME:LINE1>	.	PASS	SVTYPE=INS:ME:LINE1;END=42346037;SVLEN=747;AN=20618;AC=1;AF=4.850130953535746e-05;N_HET=1;N_HOMALT=0;N_HOMREF=10308;FREQ_HET=9.700261907071492e-05;FREQ_HOMALT=0.0;FREQ_HOMREF=0.9999029973809292;N_BI_GENOS=10309;Miao_AN=482;Miao_AC=1;Miao_AF=0.002074688796
chr6	43689459	.	G	<INS:ME:LINE1>	.	PASS	SVTYPE=INS:ME:LINE1;END=43689459;SVLEN=768;AN=20618;AC=1;AF=4.850130953535746e-05;N_HET=1;N_HOMALT=0;N_HOMREF=10308;FREQ_HET=9.700261907071492e-05;FREQ_HOMALT=0.0;FREQ_HOMREF=0.9999029973809292;N_BI_GENOS=10309;East_Han_AN=6554;East_Han_AC=1;East_Han_AF=0
chr6	44789868	.	A	<INS:ME:LINE1>	.	PASS	SVTYPE=INS:ME:LINE1;END=44789881;SVLEN=471;AN=20618;AC=1;AF=4.850130953535746e-05;N_HET=1;N_HOMALT=0;N_HOMREF=10308;FREQ_HET=9.700261907071492e-05;FREQ_HOMALT=0.0;FREQ_HOMREF=0.9999029973809292;N_BI_GENOS=10309;South_Han_AN=1496;South_Han_AC=1;South_Han_A
chr6	44939829	.	C	<INS:ME:LINE1>	.	PASS	SVTYPE=INS:ME:LINE1;END=44939829;SVLEN=183;AN=20618;AC=1;AF=4.850130953535746e-05;N_HET=1;N_HOMALT=0;N_HOMREF=10308;FREQ_HET=9.700261907071492e-05;FREQ_HOMALT=0.0;FREQ_HOMREF=0.9999029973809292;N_BI_GENOS=10309;East_Han_AN=6554;East_Han_AC=1;East_Han_AF=0
chr6	44953273	.	C	<INS:ME:LINE1>	.	PASS	SVTYPE=INS:ME:LINE1;END=44953280;SVLEN=1024;AN=20618;AC=2;AF=9.700261907071492e-05;N_HET=2;N_HOMALT=0;N_HOMREF=10307;FREQ_HET=0.00019400523814142983;FREQ_HOMALT=0.0;FREQ_HOMREF=0.9998059947618586;N_BI_GENOS=10309;East_Han_AN=6554;East_Han_AC=1;East_Han_AF
chr6	45087131	.	A	<INS:ME:LINE1>	.	PASS	SVTYPE=INS:ME:LINE1;END=45087147;SVLEN=527;AN=20618;AC=2;AF=9.700261907071492e-05;N_HET=2;N_HOMALT=0;N_HOMREF=10307;FREQ_HET=0.00019400523814142983;FREQ_HOMALT=0.0;FREQ_HOMREF=0.9998059947618586;N_BI_GENOS=10309;South_Han_AN=1496;South_Han_AC=1;South_Han_
chr6	45288383	.	T	<INS:ME:LINE1>	.	PASS	SVTYPE=INS:ME:LINE1;END=45288385;SVLEN=810;AN=20618;AC=1;AF=4.850130953535746e-05;N_HET=1;N_HOMALT=0;N_HOMREF=10308;FREQ_HET=9.700261907071492e-05;FREQ_HOMALT=0.0;FREQ_HOMREF=0.9999029973809292;N_BI_GENOS=10309;Southeast_Han_AN=1908;Southeast_Han_AC=1;Sou
chr6	45345200	.	T	<INS:ME:LINE1>	.	PASS	SVTYPE=INS:ME:LINE1;END=45345216;SVLEN=556;AN=20618;AC=13;AF=0.0006305170239596469;N_HET=13;N_HOMALT=0;N_HOMREF=10296;FREQ_HET=0.0012610340479192938;FREQ_HOMALT=0.0;FREQ_HOMREF=0.9987389659520807;N_BI_GENOS=10309;South_Han_AN=1496;South_Han_AC=3;South_Han
chr6	45431400	.	A	<INS:ME:LINE1>	.	PASS	SVTYPE=INS:ME:LINE1;END=45431414;SVLEN=6000;AN=20618;AC=1;AF=4.850130953535746e-05;N_HET=1;N_HOMALT=0;N_HOMREF=10308;FREQ_HET=9.700261907071492e-05;FREQ_HOMALT=0.0;FREQ_HOMREF=0.9999029973809292;N_BI_GENOS=10309;South_Han_AN=1496;South_Han_AC=1;South_Han_
chr6	45884959	.	A	<INS:ME:LINE1>	.	PASS	SVTYPE=INS:ME:LINE1;END=45884975;SVLEN=16;AN=20618;AC=1583;AF=0.07677757299447086;N_HET=71;N_HOMALT=756;N_HOMREF=9482;FREQ_HET=0.006887185954020759;FREQ_HOMALT=0.07333398001746047;FREQ_HOMREF=0.9197788340285188;N_BI_GENOS=10309;South_Han_AN=1496;South_Han
chr6	45899318	.	T	<INS:ME:LINE1>	.	PASS	SVTYPE=INS:ME:LINE1;END=45899331;SVLEN=5640;AN=20618;AC=4;AF=0.00019400523814142983;N_HET=4;N_HOMALT=0;N_HOMREF=10305;FREQ_HET=0.0003880104762828596 6;FREQ_HOMALT=0.0;FREQ_HOMREF=0.9996119895237171;N_BI_GENOS=10309;South_Han_AN=1496;South_Han_AC=2;South_Ha
chr6	45956583	.	G	<INS:ME:LINE1>	.	PASS	SVTYPE=INS:ME:LINE1;END=45956597;SVLEN=677;AN=20618;AC=509;AF=0.02468716655349 6943;N_HET=469;N_HOMALT=20;N_HOMREF=9820;FREQ_HET=0.04549422834416529;FREQ_HOMALT=0.0019400523814142982;FREQ_HOMREF=0.9525657192744205;N_BI_GENOS=10309;South_Han_AN=1496;South_H
chr6	46342557	.	A	<INS:ME:LINE1>	.	PASS	SVTYPE=INS:ME:LINE1;END=46342569;SVLEN=1124;AN=20618;AC=12307;AF=0.5969056164516442;N_HET=4197;N_HOMALT=4055;N_HOMREF=2057;FREQ_HET=0.40711999223979045;FREQ_HOMALT=0.39334562033174897;FREQ_HOMREF=0.19953438742846058;N_BI_GENOS=10309;South_Han_AN=1496;Sout
chr6	46380630	.	A	<INS:ME:LINE1>	.	PASS	SVTYPE=INS:ME:LINE1;END=46380646;SVLEN=1819;AN=20618;AC=10;AF=0.00048501309535357455;N_HET=10;N_HOMALT=0;N_HOMREF=10299;FREQ_HET=0.0009700261907071491;FREQ_HOMALT=0.0;FREQ_HOMREF=0.9990299738092928;N_BI_GENOS=10309;South_Han_AN=1496;South_Han_AC=2;South_H
chr6	46461161	.	T	<INS:ME:LINE1>	.	PASS	SVTYPE=INS:ME:LINE1;END=46461176;SVLEN=1415;AN=20618;AC=1;AF=4.850130953535746e-05;N_HET=1;N_HOMALT=0;N_HOMREF=10308;FREQ_HET=9.700261907071492e-05;FREQ_HOMALT=0.0;FREQ_HOMREF=0.9999029973809292;N_BI_GENOS=10309;East_Han_AN=6554;East_Han_AC=1;East_Han_AF=
chr6	46513868	.	A	<INS:ME:LINE1>	.	PASS	SVTYPE=INS:ME:LINE1;END=46513882;SVLEN=1930;AN=20618;AC=1;AF=4.850130953535746e-05;N_HET=1;N_HOMALT=0;N_HOMREF=10308;FREQ_HET=9.700261907071492e-05;FREQ_HOMALT=0.0;FREQ_HOMREF=0.9999029973809292;N_BI_GENOS=10309;East_Han_AN=6554;East_Han_AC=1;East_Han_AF=
chr6	46580013	.	C	<INS:ME:LINE1>	.	PASS	SVTYPE=INS:ME:LINE1;END=46580013;SVLEN=320;AN=20618;AC=1;AF=4.850130953535746e-05;N_HET=1;N_HOMALT=0;N_HOMREF=10308;FREQ_HET=9.700261907071492e-05;FREQ_HOMALT=0.0;FREQ_HOMREF=0.9999029973809292;N_BI_GENOS=10309;East_Han_AN=6554;East_Han_AC=1;East_Han_AF=0

chr6	46731552	.	G	<INS:ME:LINE1>	.	PASS	SVTYPE=INS:ME:LINE1;END=46731566;SVLEN=6073;AN=20618;AC=2;AF=9.700261907071492e-05;N_HET=2;N_HOMALT=0;N_HOMREF=10307;FREQ_HET=0.0001940052381414298 3;FREQ_HOMALT=0.0;FREQ_HOMREF=0.9998059947618586;N_BI_GENOS=10309;North_Han_AN=2272;North_Han_AC=2;North_Han
chr6	46953831	.	A	<INS:ME:LINE1>	.	PASS	SVTYPE=INS:ME:LINE1;END=46953831;SVLEN=5993;AN=20618;AC=1;AF=4.850130953535746e-05;N_HET=1;N_HOMALT=0;N_HOMREF=10308;FREQ_HET=9.700261907071492e-05;FREQ_HOMALT=0.0;FREQ_HOMREF=0.9999029973809292;N_BI_GENOS=10309;Central_Han_AN=2128;Central_Han_AC=1;Centra
chr6	47072920	.	G	<INS:ME:LINE1>	.	PASS	SVTYPE=INS:ME:LINE1;END=47072967;SVLEN=876;AN=20618;AC=1;AF=4.850130953535746e-05;N_HET=1;N_HOMALT=0;N_HOMREF=10308;FREQ_HET=9.700261907071492e-05;FREQ_HOMALT=0.0;FREQ_HOMREF=0.9999029973809292;N_BI_GENOS=10309;North_Han_AN=2272;North_Han_AC=1;North_Han_A
chr6	47101880	.	G	<INS:ME:LINE1>	.	PASS	SVTYPE=INS:ME:LINE1;END=47101880;SVLEN=0;AN=20618;AC=2;AF=9.700261907071492e-05;N_HET=0;N_HOMALT=1;N_HOMREF=10308;FREQ_HET=0.0;FREQ_HOMALT=9.700261907071492e-05;FREQ_HOMREF=0.9999029973809292;N_BI_GENOS=10309;Southeast_Han_AN=1908;Southeast_Han_AC=2;South
chr6	47800792	.	T	<INS:ME:LINE1>	.	PASS	SVTYPE=INS:ME:LINE1;END=47800805;SVLEN=6007;AN=20618;AC=3;AF=0.00014550392860607236;N_HET=3;N_HOMALT=0;N_HOMREF=10306;FREQ_HET=0.0002910078572121447;FREQ_HOMALT=0.0;FREQ_HOMREF=0.9997089921427879;N_BI_GENOS=10309;East_Han_AN=6554;East_Han_AC=2;East_Han_AF
chr6	47815088	.	G	<INS:ME:LINE1>	.	PASS	SVTYPE=INS:ME:LINE1;END=47815103;SVLEN=6006;AN=20618;AC=11;AF=0.0005335144048889 32;N_HET=11;N_HOMALT=0;N_HOMREF=10298;FREQ_HET=0.0010670288097777864;FREQ_HOMALT=0.0;FREQ_HOMREF=0.9989329711902222;N_BI_GENOS=10309;East_Han_AN=6554;East_Han_AC=5;East_Han_AF=
chr6	47823263	.	A	<INS:ME:LINE1>	.	PASS	SVTYPE=INS:ME:LINE1;END=47823279;SVLEN=1186;AN=20618;AC=7;AF=0.0003395091667475022;N_HET=7;N_HOMALT=0;N_HOMREF=10302;FREQ_HET=0.0006790183334950044;FREQ_HOMALT=0.0;FREQ_HOMREF=0.999320981666505;N_BI_GENOS=10309;South_Han_AN=1496;South_Han_AC=1;South_Han_A
chr6	47834965	.	T	<INS:ME:LINE1>	.	PASS	SVTYPE=INS:ME:LINE1;END=47834985;SVLEN=2755;AN=20618;AC=3;AF=0.00014550392860607236;N_HET=3;N_HOMALT=0;N_HOMREF=10306;FREQ_HET=0.0002910078572121447;FREQ_HOMALT=0.0;FREQ_HOMREF=0.9997089921427879;N_BI_GENOS=10309;East_Han_AN=6554;East_Han_AC=3;East_Han_AF
chr6	47858786	.	A	<INS:ME:LINE1>	.	PASS	SVTYPE=INS:ME:LINE1;END=47858797;SVLEN=922;AN=20618;AC=1;AF=4.850130953535746e-05;N_HET=1;N_HOMALT=0;N_HOMREF=10308;FREQ_HET=9.700261907071492e-05;FREQ_HOMALT=0.0;FREQ_HOMREF=0.9999029973809292;N_BI_GENOS=10309;North_Han_AN=2272;North_Han_AC=1;North_Han_A
chr6	47873054	.	A	<INS:ME:LINE1>	.	PASS	SVTYPE=INS:ME:LINE1;END=47873068;SVLEN=208;AN=20618;AC=1;AF=4.850130953535746e-05;N_HET=1;N_HOMALT=0;N_HOMREF=10308;FREQ_HET=9.700261907071492e-05;FREQ_HOMALT=0.0;FREQ_HOMREF=0.9999029973809292;N_BI_GENOS=10309;South_Han_AN=1496;South_Han_AC=1;South_Han_A
chr6	47922489	.	T	<INS:ME:LINE1>	.	PASS	SVTYPE=INS:ME:LINE1;END=47922501;SVLEN=158;AN=20618;AC=1;AF=4.850130953535746e-05;N_HET=1;N_HOMALT=0;N_HOMREF=10308;FREQ_HET=9.700261907071492e-05;FREQ_HOMALT=0.0;FREQ_HOMREF=0.9999029973809292;N_BI_GENOS=10309;Southeast_Han_AN=1908;Southeast_Han_AC=1;Sou
chr6	48027029	.	T	<INS:ME:LINE1>	.	PASS	SVTYPE=INS:ME:LINE1;END=48027046;SVLEN=5997;AN=20618;AC=1;AF=4.850130953535746e-05;N_HET=1;N_HOMALT=0;N_HOMREF=10308;FREQ_HET=9.700261907071492e-05;FREQ_HOMALT=0.0;FREQ_HOMREF=0.9999029973809292;N_BI_GENOS=10309;Tibetan_AN=414;Tibetan_AC=1;Tibetan_AF=0.00
chr6	48077208	.	A	<INS:ME:LINE1>	.	PASS	SVTYPE=INS:ME:LINE1;END=48077226;SVLEN=6000;AN=20618;AC=1;AF=4.850130953535746e-05;N_HET=1;N_HOMALT=0;N_HOMREF=10308;FREQ_HET=9.700261907071492e-05;FREQ_HOMALT=0.0;FREQ_HOMREF=0.9999029973809292;N_BI_GENOS=10309;East_Han_AN=6554;East_Han_AC=1;East_Han_AF=
chr6	48098344	.	T	<INS:ME:LINE1>	.	PASS	SVTYPE=INS:ME:LINE1;END=48098344;SVLEN=0;AN=20618;AC=1;AF=4.850130953535746e-05;N_HET=1;N_HOMALT=0;N_HOMREF=10308;FREQ_HET=9.700261907071492e-05;FREQ_HOMALT=0.0;FREQ_HOMREF=0.9999029973809292;N_BI_GENOS=10309;Southeast_Han_AN=1908;Southeast_Han_AC=1;South
chr6	48188290	.	A	<INS:ME:LINE1>	.	PASS	SVTYPE=INS:ME:LINE1;END=48188306;SVLEN=1024;AN=20618;AC=1;AF=4.850130953535746e-05;N_HET=1;N_HOMALT=0;N_HOMREF=10308;FREQ_HET=9.700261907071492e-05;FREQ_HOMALT=0.0;FREQ_HOMREF=0.9999029973809292;N_BI_GENOS=10309;East_Han_AN=6554;East_Han_AC=1;East_Han_AF=
chr6	48205631	.	A	<INS:ME:LINE1>	.	PASS	SVTYPE=INS:ME:LINE1;END=48205634;SVLEN=95;AN=20618;AC=6;AF=0.0002910078572121447;N_HET=4;N_HOMALT=1;N_HOMREF=10304;FREQ_HET=0.0003880104762828596 6;FREQ_HOMALT=9.700261907071492e-05;FREQ_HOMREF=0.999514986904646 5;N_BI_GENOS=10309;South_Han_AN=1496;South_Ha
chr6	48471941	.	A	<INS:ME:LINE1>	.	PASS	SVTYPE=INS:ME:LINE1;END=48471957;SVLEN=708;AN=20618;AC=1;AF=4.850130953535746e-05;N_HET=1;N_HOMALT=0;N_HOMREF=10308;FREQ_HET=9.700261907071492e-05;FREQ_HOMALT=0.0;FREQ_HOMREF=0.9999029973809292;N_BI_GENOS=10309;Mongolian_AN=454;Mongolian_AC=1;Mongolian_AF
chr6	48644266	.	T	<INS:ME:LINE1>	.	PASS	SVTYPE=INS:ME:LINE1;END=48644288;SVLEN=74;AN=20618;AC=1;AF=4.850130953535746e-05;N_HET=1;N_HOMALT=0;N_HOMREF=10308;FREQ_HET=9.700261907071492e-05;FREQ_HOMALT=0.0;FREQ_HOMREF=0.9999029973809292;N_BI_GENOS=10309;Tibetan_AN=414;Tibetan_AC=1;Tibetan_AF=0.0024
chr6	48715490	.	A	<INS:ME:LINE1>	.	PASS	SVTYPE=INS:ME:LINE1;END=48715512;SVLEN=953;AN=20618;AC=1;AF=4.850130953535746e-05;N_HET=1;N_HOMALT=0;N_HOMREF=10308;FREQ_HET=9.700261907071492e-05;FREQ_HOMALT=0.0;FREQ_HOMREF=0.9999029973809292;N_BI_GENOS=10309;Tibetan_AN=414;Tibetan_AC=1;Tibetan_AF=0.002
chr6	48840931	.	G	<INS:ME:LINE1>	.	PASS	SVTYPE=INS:ME:LINE1;END=48840974;SVLEN=4212;AN=20618;AC=1;AF=4.850130953535746e-05;N_HET=1;N_HOMALT=0;N_HOMREF=10308;FREQ_HET=9.700261907071492e-05;FREQ_HOMALT=0.0;FREQ_HOMREF=0.9999029973809292;N_BI_GENOS=10309;East_Han_AN=6554;East_Han_AC=1;East_Han_AF=
chr6	48976216	.	A	<INS:ME:LINE1>	.	PASS	SVTYPE=INS:ME:LINE1;END=48976234;SVLEN=739;AN=20618;AC=1;AF=4.850130953535746e-05;N_HET=1;N_HOMALT=0;N_HOMREF=10308;FREQ_HET=9.700261907071492e-05;FREQ_HOMALT=0.0;FREQ_HOMREF=0.9999029973809292;N_BI_GENOS=10309;North_Han_AN=2272;North_Han_AC=1;North_Han_A
chr6	49299162	.	A	<INS:ME:LINE1>	.	PASS	SVTYPE=INS:ME:LINE1;END=49299179;SVLEN=2513;AN=20618;AC=2;AF=9.700261907071492e-05;N_HET=2;N_HOMALT=0;N_HOMREF=10307;FREQ_HET=0.0001940052381414298 3;FREQ_HOMALT=0.0;FREQ_HOMREF=0.9998059947618586;N_BI_GENOS=10309;Hui_AN=444;Hui_AC=1;Hui_AF=0.0022522522522
chr6	49532353	.	A	<INS:ME:LINE1>	.	PASS	SVTYPE=INS:ME:LINE1;END=49532367;SVLEN=467;AN=20618;AC=7;AF=0.0003395091667475022;N_HET=5;N_HOMALT=1;N_HOMREF=10303;FREQ_HET=0.0004850130953535757;FREQ_HOMALT=9.700261907071492e-05;FREQ_HOMREF=0.9994179842855757;N_BI_GENOS=10309;Lingnan_Han_AN=1888;Lingn
chr6	49666382	.	A	<INS:ME:LINE1>	.	PASS	SVTYPE=INS:ME:LINE1;END=49666400;SVLEN=1790;AN=20618;AC=9;AF=0.0004365117858182171;N_HET=9;N_HOMALT=0;N_HOMREF=10300;FREQ_HET=0.0008730235716364342;FREQ_HOMALT=0.0;FREQ_HOMREF=0.9991269764283636;N_BI_GENOS=10309;Lingnan_Han_AN=1888;Lingnan_Han_AC=1;Lingna
chr6	49852152	.	A	<INS:ME:LINE1>	.	PASS	SVTYPE=INS:ME:LINE1;END=49852167;SVLEN=6007;AN=20618;AC=1;AF=4.850130953535746e-05;N_HET=1;N_HOMALT=0;N_HOMREF=10308;FREQ_HET=9.700261907071492e-05;FREQ_HOMALT=0.0;FREQ_HOMREF=0.9999029973809292;N_BI_GENOS=10309;East_Han_AN=6554;East_Han_AC=1;East_Han_AF=
chr6	49871129	.	A	<INS:ME:LINE1>	.	PASS	SVTYPE=INS:ME:LINE1;END=49871139;SVLEN=5990;AN=20618;AC=2;AF=9.700261907071492e-05;N_HET=0;N_HOMALT=1;N_HOMREF=10308;FREQ_HET=0.0;FREQ_HOMALT=9.700261907071492e-05;FREQ_HOMREF=0.9999029973809292;N_BI_GENOS=10309;Hui_AN=444;Hui_AC=2;Hui_AF=0.00450450450450
chr6	50182791	.	A	<INS:ME:LINE1>	.	PASS	SVTYPE=INS:ME:LINE1;END=50182803;SVLEN=1697;AN=20618;AC=12;AF=0.0005820157144242894;N_HET=10;N_HOMALT=1;N_HOMREF=10298;FREQ_HET=0.0009700261907071491;FREQ_HOMALT=9.700261907071492e-05;FREQ_HOMREF=0.9989329711902222;N_BI_GENOS=10309;South_Han_AN=1496;South
chr6	50216887	.	T	<INS:ME:LINE1>	.	PASS	SVTYPE=INS:ME:LINE1;END=50216901;SVLEN=1555;AN=20618;AC=1;AF=4.850130953535746e-05;N_HET=1;N_HOMALT=0;N_HOMREF=10308;FREQ_HET=9.700261907071492e-05;FREQ_HOMALT=0.0;FREQ_HOMREF=0.9999029973809292;N_BI_GENOS=10309;East_Han_AN=6554;East_Han_AC=1;East_Han_AF=
chr6	50551485	.	T	<INS:ME:LINE1>	.	PASS	SVTYPE=INS:ME:LINE1;END=50551487;SVLEN=783;AN=20618;AC=1;AF=4.850130953535746e-05;N_HET=1;N_HOMALT=0;N_HOMREF=10308;FREQ_HET=9.700261907071492e-05;FREQ_HOMALT=0.0;FREQ_HOMREF=0.9999029973809292;N_BI_GENOS=10309;East_Han_AN=6554;East_Han_AC=1;East_Han_AF=0
chr6	51155694	.	A	<INS:ME:LINE1>	.	PASS	SVTYPE=INS:ME:LINE1;END=51155709;SVLEN=5999;AN=20618;AC=3;AF=0.00014550392860607236;N_HET=3;N_HOMALT=0;N_HOMREF=10306;FREQ_HET=0.0002910078572121447;FREQ_HOMALT=0.0;FREQ_HOMREF=0.9997089921427879;N_BI_GENOS=10309;Lingnan_Han_AN=1888;Lingnan_Han_AC=1;Lingn
chr6	51283398	.	A	<INS:ME:LINE1>	.	PASS	SVTYPE=INS:ME:LINE1;END=51283414;SVLEN=954;AN=20618;AC=2;AF=9.700261907071492e-05;N_HET=2;N_HOMALT=0;N_HOMREF=10307;FREQ_HET=0.0001940052381414298 3;FREQ_HOMALT=0.0;FREQ_HOMREF=0.9998059947618586;N_BI_GENOS=10309;Southeast_Han_AN=1908;Southeast_Han_AC=2;So
chr6	51373934	.	A	<INS:ME:LINE1>	.	PASS	SVTYPE=INS:ME:LINE1;END=51373939;SVLEN=967;AN=20618;AC=1;AF=4.850130953535746e-05;N_HET=1;N_HOMALT=0;N_HOMREF=10308;FREQ_HET=9.700261907071492e-05;FREQ_HOMALT=0.0;FREQ_HOMREF=0.9999029973809292;N_BI_GENOS=10309;Central_Han_AN=2128;Central_Han_AC=1;Central
chr6	51507082	.	A	<INS:ME:LINE1>	.	PASS	SVTYPE=INS:ME:LINE1;END=51507082;SVLEN=583;AN=20618;AC=1;AF=4.850130953535746e-05;N_HET=1;N_HOMALT=0;N_HOMREF=10308;FREQ_HET=9.700261907071492e-05;FREQ_HOMALT=0.0;FREQ_HOMREF=0.9999029973809292;N_BI_GENOS=10309;East_Han_AN=6554;East_Han_AC=1;East_Han_AF=0
chr6	51561556	.	G	<INS:ME:LINE1>	.	PASS	SVTYPE=INS:ME:LINE1;END=51561573;SVLEN=719;AN=20618;AC=2;AF=9.700261907071492e-05;N_HET=2;N_HOMALT=0;N_HOMREF=10307;FREQ_HET=0.0001940052381414298 3;FREQ_HOMALT=0.0;FREQ_HOMREF=0.9998059947618586;N_BI_GENOS=10309;Lingnan_Han_AN=1888;Lingnan_Han_AC=1;Lingna
chr6	51595675	.	A	<INS:ME:LINE1>	.	PASS	SVTYPE=INS:ME:LINE1;END=51595689;SVLEN=623;AN=20618;AC=18;AF=0.0008730235716364342;N_HET=16;N_HOMALT=1;N_HOMREF=10292;FREQ_HET=0.0015520419051314386;FREQ_HOMALT=9.700261907071492e-05;FREQ_HOMREF=0.9983509554757979;N_BI_GENOS=10309;South_Han_AN=1496;South_
chr6	51647164	.	A	<INS:ME:LINE1>	.	PASS	SVTYPE=INS:ME:LINE1;END=51647178;SVLEN=335;AN=20618;AC=2;AF=9.700261907071492e-05;N_HET=0;N_HOMALT=1;N_HOMREF=10308;FREQ_HET=0.0;FREQ_HOMALT=9.700261907071492e-05;FREQ_HOMREF=0.9999029973809292;N_BI_GENOS=10309;Central_Han_AN=2128;Central_Han_AC=2;Central
chr6	51766615	.	T	<INS:ME:LINE1>	.	PASS	SVTYPE=INS:ME:LINE1;END=51766615;SVLEN=27;AN=20618;AC=1;AF=4.850130953535746e-05;N_HET=1;N_HOMALT=0;N_HOMREF=10308;FREQ_HET=9.700261907071492e-05;FREQ_HOMALT=0.0;FREQ_HOMREF=0.9999029973809292;N_BI_GENOS=10309;East_Han_AN=6554;East_Han_AC=1;East_Han_AF=0.
chr6	51958366	.	A	<INS:ME:LINE1>	.	PASS	SVTYPE=INS:ME:LINE1;END=51958366;SVLEN=666;AN=20618;AC=1;AF=4.850130953535746e-05;N_HET=1;N_HOMALT=0;N_HOMREF=10308;FREQ_HET=9.700261907071492e-05;FREQ_HOMALT=0.0;FREQ_HOMREF=0.9999029973809292;N_BI_GENOS=10309;North_Han_AN=2272;North_Han_AC=1;North_Han_A
chr6	52051880	.	G	<INS:ME:LINE1>	.	PASS	SVTYPE=INS:ME:LINE1;END=52051897;SVLEN=5993;AN=20618;AC=7;AF=0.0003395091667475022;N_HET=7;N_HOMALT=0;N_HOMREF=10302;FREQ_HET=0.0006790183334950044;FREQ_HOMALT=0.0;FREQ_HOMREF=0.999320981666505;N_BI_GENOS=10309;South_Han_AN=1496;South_Han_AC=1;South_Han_A
chr6	52595063	.	G	<INS:ME:LINE1>	.	PASS	SVTYPE=INS:ME:LINE1;END=52595079;SVLEN=998;AN=20618;AC=3;AF=0.00014550392860607236;N_HET=3;N_HOMALT=0;N_HOMREF=10306;FREQ_HET=0.0002910078572121447;FREQ_HOMALT=0.0;FREQ_HOMREF=0.9997089921427879;N_BI_GENOS=10309;North_Han_AN=2272;North_Han_AC=1;North_Han_
chr6	52672330	.	A	<INS:ME:LINE1>	.	PASS	SVTYPE=INS:ME:LINE1;END=52672330;SVLEN=11;AN=20618;AC=1;AF=4.850130953535746e-05;N_HET=1;N_HOMALT=0;N_HOMREF=10308;FREQ_HET=9.700261907071492e-05;FREQ_HOMALT=0.0;FREQ_HOMREF=0.9999029973809292;N_BI_GENOS=10309;South_Han_AN=1496;South_Han_AC=1;South_Han_AF
chr6	53016762	.	C	<INS:ME:LINE1>	.	PASS	SVTYPE=INS:ME:LINE1;END=53016776;SVLEN=1162;AN=20618;AC=4;AF=0.00019400523814142983;N_HET=4;N_HOMALT=0;N_HOMREF=10305;FREQ_HET=0.0003880104762828596 6;FREQ_HOMALT=0.0;FREQ_HOMREF=0.9996119895237171;N_BI_GENOS=10309;Lingnan_Han_AN=1888;Lingnan_Han_AC=1;Ling
chr6	53020797	.	C	<INS:ME:LINE1>	.	PASS	SVTYPE=INS:ME:LINE1;END=53020812;SVLEN=344;AN=20618;AC=1;AF=4.850130953535746e-05;N_HET=1;N_HOMALT=0;N_HOMREF=10308;FREQ_HET=9.700261907071492e-05;FREQ_HOMALT=0.0;FREQ_HOMREF=0.9999029973809292;N_BI_GENOS=10309;Southeast_Han_AN=1908;Southeast_Han_AC=1;Sou
chr6	53091663	.	G	<INS:ME:LINE1>	.	PASS	SVTYPE=INS:ME:LINE1;END=53091682;SVLEN=2586;AN=20618;AC=1;AF=4.850130953535746e-05;N_HET=1;N_HOMALT=0;N_HOMREF=10308;FREQ_HET=9.700261907071492e-05;FREQ_HOMALT=0.0;FREQ_HOMREF=0.9999029973809292;N_BI_GENOS=10309;East_Han_AN=6554;East_Han_AC=1;East_Han_AF=
chr6	53329393	.	A	<INS:ME:LINE1>	.	PASS	SVTYPE=INS:ME:LINE1;END=53329393;SVLEN=5983;AN=20618;AC=1;AF=4.850130953535746e-05;N_HET=1;N_HOMALT=0;N_HOMREF=10308;FREQ_HET=9.700261907071492e-05;FREQ_HOMALT=0.0;FREQ_HOMREF=0.9999029973809292;N_BI_GENOS=10309;Yi_AN=422;Yi_AC=1;Yi_AF=0.00236966824644549
chr6	53486369	.	T	<INS:ME:LINE1>	.	PASS	SVTYPE=INS:ME:LINE1;END=53486385;SVLEN=6060;AN=20618;AC=1;AF=4.850130953535746e-05;N_HET=1;N_HOMALT=0;N_HOMREF=10308;FREQ_HET=9.700261907071492e-05;FREQ_HOMALT=0.0;FREQ_HOMREF=0.9999029973809292;N_BI_GENOS=10309;North_Han_AN=2272;North_Han_AC=1;North_Han_
chr6	53630724	.	A	<INS:ME:LINE1>	.	PASS	SVTYPE=INS:ME:LINE1;END=53630737;SVLEN=3053;AN=20618;AC=1;AF=4.850130953535746e-05;N_HET=1;N_HOMALT=0;N_HOMREF=10308;FREQ_HET=9.700261907071492e-05;FREQ_HOMALT=0.0;FREQ_HOMREF=0.9999029973809292;N_BI_GENOS=10309;Mongolian_AN=454;Mongolian_AC=1;Mongolian_A
chr6	53688131	.	A	<INS:ME:LINE1>	.	PASS	SVTYPE=INS:ME:LINE1;END=53688134;SVLEN=5838;AN=20618;AC=13;AF=0.0006305170239596469;N_HET=13;N_HOMALT=0;N_HOMREF=10296;FREQ_HET=0.0012610340479192938;FREQ_HOMALT=0.0;FREQ_HOMREF=0.9987389659520807;N_BI_GENOS=10309;South_Han_AN=1496;South_Han_AC=1;South_Ha
chr6	53783960	.	T	<INS:ME:LINE1>	.	PASS	SVTYPE=INS:ME:LINE1;END=53783960;SVLEN=0;AN=20618;AC=1;AF=4.850130953535746e-05;N_HET=1;N_HOMALT=0;N_HOMREF=10308;FREQ_HET=9.700261907071492e-05;FREQ_HOMALT=0.0;FREQ_HOMREF=0.9999029973809292;N_BI_GENOS=10309;East_Han_AN=6554;East_Han_AC=1;East_Han_AF=0.0
chr6	54344583	.	A	<INS:ME:LINE1>	.	PASS	SVTYPE=INS:ME:LINE1;END=54344594;SVLEN=1570;AN=20618;AC=1;AF=4.850130953535746e-05;N_HET=1;N_HOMALT=0;N_HOMREF=10308;FREQ_HET=9.700261907071492e-05;FREQ_HOMALT=0.0;FREQ_HOMREF=0.9999029973809292;N_BI_GENOS=10309;North_Han_AN=2272;North_Han_AC=1;North_Han_
chr6	54375373	.	G	<INS:ME:LINE1>	.	PASS	SVTYPE=INS:ME:LINE1;END=54375390;SVLEN=1206;AN=20618;AC=1;AF=4.850130953535746e-05;N_HET=1;N_HOMALT=0;N_HOMREF=10308;FREQ_HET=9.700261907071492e-05;FREQ_HOMALT=0.0;FREQ_HOMREF=0.9999029973809292;N_BI_GENOS=10309;Tibetan_AN=414;Tibetan_AC=1;Tibetan_AF=0.00
chr6	55206000	.	A	<INS:ME:LINE1>	.	PASS	SVTYPE=INS:ME:LINE1;END=55206018;SVLEN=5999;AN=20618;AC=1;AF=4.850130953535746e-05;N_HET=1;N_HOMALT=0;N_HOMREF=10308;FREQ_HET=9.700261907071492e-05;FREQ_HOMALT=0.0;FREQ_HOMREF=0.9999029973809292;N_BI_GENOS=10309;Hui_AN=444;Hui_AC=1;Hui_AF=0.00225225225225
chr6	55714953	.	T	<INS:ME:LINE1>	.	PASS	SVTYPE=INS:ME:LINE1;END=55714969;SVLEN=396;AN=20618;AC=1;AF=4.850130953535746e-05;N_HET=1;N_HOMALT=0;N_HOMREF=10308;FREQ_HET=9.700261907071492e-05;FREQ_HOMALT=0.0;FREQ_HOMREF=0.9999029973809292;N_BI_GENOS=10309;East_Han_AN=6554;East_Han_AC=1;East_Han_AF=0
chr6	55790227	.	T	<INS:ME:LINE1>	.	PASS	SVTYPE=INS:ME:LINE1;END=55790241;SVLEN=872;AN=20618;AC=1;AF=4.850130953535746e-05;N_HET=1;N_HOMALT=0;N_HOMREF=10308;FREQ_HET=9.700261907071492e-05;FREQ_HOMALT=0.0;FREQ_HOMREF=0.9999029973809292;N_BI_GENOS=10309;Central_Han_AN=2128;Central_Han_AC=1;Central
chr6	56159159	.	A	<INS:ME:LINE1>	.	PASS	SVTYPE=INS:ME:LINE1;END=56159173;SVLEN=6028;AN=20618;AC=1;AF=4.850130953535746e-05;N_HET=1;N_HOMALT=0;N_HOMREF=10308;FREQ_HET=9.700261907071492e-05;FREQ_HOMALT=0.0;FREQ_HOMREF=0.9999029973809292;N_BI_GENOS=10309;South_Han_AN=1496;South_Han_AC=1;South_Han_
chr6	56470835	.	A	<INS:ME:LINE1>	.	PASS	SVTYPE=INS:ME:LINE1;END=56470852;SVLEN=1007;AN=20618;AC=1;AF=4.850130953535746e-05;N_HET=1;N_HOMALT=0;N_HOMREF=10308;FREQ_HET=9.700261907071492e-05;FREQ_HOMALT=0.0;FREQ_HOMREF=0.9999029973809292;N_BI_GENOS=10309;East_Han_AN=6554;East_Han_AC=1;East_Han_AF=
chr6	56606156	.	C	<INS:ME:LINE1>	.	PASS	SVTYPE=INS:ME:LINE1;END=56606171;SVLEN=256;AN=20618;AC=1;AF=4.850130953535746e-05;N_HET=1;N_HOMALT=0;N_HOMREF=10308;FREQ_HET=9.700261907071492e-05;FREQ_HOMALT=0.0;FREQ_HOMREF=0.9999029973809292;N_BI_GENOS=10309;Hui_AN=444;Hui_AC=1;Hui_AF=0.00225225225225 2
chr6	56623966	.	C	<INS:ME:LINE1>	.	PASS	SVTYPE=INS:ME:LINE1;END=56623984;SVLEN=52;AN=20618;AC=2;AF=9.700261907071492e-05;N_HET=2;N_HOMALT=0;N_HOMREF=10307;FREQ_HET=0.0001940052381414298 3;FREQ_HOMALT=0.0;FREQ_HOMREF=0.9998059947618586;N_BI_GENOS=10309;North_Han_AN=2272;North_Han_AC=1;North_Han
chr6	56741144	.	A	<INS:ME:LINE1>	.	PASS	SVTYPE=INS:ME:LINE1;END=56741159;SVLEN=6021;AN=20618;AC=2;AF=9.700261907071492e-05;N_HET=2;N_HOMALT=0;N_HOMREF=10307;FREQ_HET=0.0001940052381414298 3;FREQ_HOMALT=0.0;FREQ_HOMREF=0.9998059947618586;N_BI_GENOS=10309;South_Han_AN=1496;South_Han_AC=1;South_Han
chr6	56864497	.	G	<INS:ME:LINE1>	.	PASS	SVTYPE=INS:ME:LINE1;END=56864517;SVLEN=1004;AN=20618;AC=426;AF=0.020661557862062276;N_HET=412;N_HOMALT=7;N_HOMREF=9890;FREQ_HET=0.03996507905713454;FREQ_HOMALT=0.0006790183334950044;FREQ_HOMREF=0.9593559026093704;N_BI_GENOS=10309;South_Han_AN=1496;South_H
chr6	56930627	.	A	<INS:ME:LINE1>	.	PASS	SVTYPE=INS:ME:LINE1;END=56930641;SVLEN=5997;AN=20618;AC=1;AF=4.850130953535746e-05;N_HET=1;N_HOMALT=0;N_HOMREF=10308;FREQ_HET=9.700261907071492e-05;FREQ_HOMALT=0.0;FREQ_HOMREF=0.9999029973809292;N_BI_GENOS=10309;North_Han_AN=2272;North_Han_AC=1;North_Han_
chr6	57192580	.	T	<INS:ME:LINE1>	.	PASS	SVTYPE=INS:ME:LINE1;END=57192597;SVLEN=256;AN=20618;AC=1;AF=4.850130953535746e-05;N_HET=1;N_HOMALT=0;N_HOMREF=10308;FREQ_HET=9.700261907071492e-05;FREQ_HOMALT=0.0;FREQ_HOMREF=0.9999029973809292;N_BI_GENOS=10309;North_Han_AN=2272;North_Han_AC=1;North_Han_A
chr6	57423037	.	T	<INS:ME:LINE1>	.	PASS	SVTYPE=INS:ME:LINE1;END=57423045;SVLEN=5995;AN=20618;AC=13;AF=0.0006305170239596469;N_HET=13;N_HOMALT=0;N_HOMREF=10296;FREQ_HET=0.0012610340479192938;FREQ_HOMALT=0.0;FREQ_HOMREF=0.9987389659520807;N_BI_GENOS=10309;South_Han_AN=1496;South_Han_AC=1;South_Ha
chr6	57729517	.	T	<INS:ME:LINE1>	.	PASS	SVTYPE=INS:ME:LINE1;END=57729518;SVLEN=328;AN=20618;AC=1;AF=4.850130953535746e-05;N_HET=1;N_HOMALT=0;N_HOMREF=10308;FREQ_HET=9.700261907071492e-05;FREQ_HOMALT=0.0;FREQ_HOMREF=0.9999029973809292;N_BI_GENOS=10309;East_Han_AN=6554;East_Han_AC=1;East_Han_AF=0
chr6	57977252	.	G	<INS:ME:LINE1>	.	PASS	SVTYPE=INS:ME:LINE1;END=57977259;SVLEN=1013;AN=20618;AC=2;AF=9.700261907071492e-05;N_HET=2;N_HOMALT=0;N_HOMREF=10307;FREQ_HET=0.0001940052381414298 3;FREQ_HOMALT=0.0;FREQ_HOMREF=0.9998059947618586;N_BI_GENOS=10309;Tibetan_AN=414;Tibetan_AC=2;Tibetan_AF=0.0
chr6	58029178	.	A	<INS:ME:LINE1>	.	PASS	SVTYPE=INS:ME:LINE1;END=58029193;SVLEN=6000;AN=20618;AC=2;AF=9.700261907071492e-05;N_HET=2;N_HOMALT=0;N_HOMREF=10307;FREQ_HET=0.0001940052381414298 3;FREQ_HOMALT=0.0;FREQ_HOMREF=0.9998059947618586;N_BI_GENOS=10309;East_Han_AN=6554;East_Han_AC=2;East_Han_AF
chr6	58051569	.	T	<INS:ME:LINE1>	.	PASS	SVTYPE=INS:ME:LINE1;END=58051603;SVLEN=38;AN=20618;AC=2;AF=9.700261907071492e-05;N_HET=0;N_HOMALT=1;N_HOMREF=10308;FREQ_HET=0.0;FREQ_HOMALT=9.700261907071492e-05;FREQ_HOMREF=0.9999029973809292;N_BI_GENOS=10309;East_Han_AN=6554;East_Han_AC=2;East_Han_AF=0.
chr6	58060407	.	A	<INS:ME:LINE1>	.	PASS	SVTYPE=INS:ME:LINE1;END=58060427;SVLEN=481;AN=20618;AC=14;AF=0.0006790183334950044;N_HET=12;N_HOMALT=1;N_HOMREF=10296;FREQ_HET=0.0011640314288485789;FREQ_HOMALT=9.700261907071492e-05;FREQ_HOMREF=0.9987389659520807;N_BI_GENOS=10309;Northwest_Han_AN=1296;No
chr6	58155488	.	T	<INS:ME:LINE1>	.	PASS	SVTYPE=INS:ME:LINE1;END=58155500;SVLEN=266;AN=20618;AC=817;AF=0.03962556989038704;N_HET=261;N_HOMALT=278;N_HOMREF=9770;FREQ_HET=0.02531768357745659;FREQ_HOMALT=0.026966728101658746;FREQ_HOMREF=0.9477155883208846;N_BI_GENOS=10309;South_Han_AN=1496;South_Ha
chr6	58220448	.	G	<INS:ME:LINE1>	.	PASS	SVTYPE=INS:ME:LINE1;END=58220462;SVLEN=122;AN=20618;AC=18;AF=0.0008730235716364342;N_HET=18;N_HOMALT=0;N_HOMREF=10291;FREQ_HET=0.0017460471432728683;FREQ_HOMALT=0.0;FREQ_HOMREF=0.9982539528567271;N_BI_GENOS=10309;South_Han_AN=1496;South_Han_AC=3;South_Han
chr6	60574079	.	T	<INS:ME:LINE1>	.	PASS	SVTYPE=INS:ME:LINE1;END=60574095;SVLEN=4731;AN=20618;AC=2;AF=9.700261907071492e-05;N_HET=2;N_HOMALT=0;N_HOMREF=10307;FREQ_HET=0.0001940052381414298 3;FREQ_HOMALT=0.0;FREQ_HOMREF=0.9998059947618586;N_BI_GENOS=10309;Mongolian_AN=454;Mongolian_AC=2;Mongolian_
chr6	60717711	.	A	<INS:ME:LINE1>	.	PASS	SVTYPE=INS:ME:LINE1;END=60717732;SVLEN=911;AN=20618;AC=1;AF=4.850130953535746e-05;N_HET=1;N_HOMALT=0;N_HOMREF=10308;FREQ_HET=9.700261907071492e-05;FREQ_HOMALT=0.0;FREQ_HOMREF=0.9999029973809292;N_BI_GENOS=10309;East_Han_AN=6554;East_Han_AC=1;East_Han_AF=0
chr6	60771427	.	A	<INS:ME:LINE1>	.	PASS	SVTYPE=INS:ME:LINE1;END=60771442;SVLEN=1855;AN=20618;AC=2;AF=9.700261907071492e-05;N_HET=2;N_HOMALT=0;N_HOMREF=10307;FREQ_HET=0.0001940052381414298 3;FREQ_HOMALT=0.0;FREQ_HOMREF=0.9998059947618586;N_BI_GENOS=10309;Central_Han_AN=2128;Central_Han_AC=1;Centr
chr6	61066545	.	G	<INS:ME:LINE1>	.	PASS	SVTYPE=INS:ME:LINE1;END=61066545;SVLEN=973;AN=20618;AC=1;AF=4.850130953535746e-05;N_HET=1;N_HOMALT=0;N_HOMREF=10308;FREQ_HET=9.700261907071492e-05;FREQ_HOMALT=0.0;FREQ_HOMREF=0.9999029973809292;N_BI_GENOS=10309;North_Han_AN=2272;North_Han_AC=1;North_Han_A
chr6	61319233	.	T	<INS:ME:LINE1>	.	PASS	SVTYPE=INS:ME:LINE1;END=61319237;SVLEN=88;AN=20618;AC=2;AF=9.700261907071492e-05;N_HET=2;N_HOMALT=0;N_HOMREF=10307;FREQ_HET=0.0001940052381414298 3;FREQ_HOMALT=0.0;FREQ_HOMREF=0.9998059947618586;N_BI_GENOS=10309;Southeast_Han_AN=1908;Southeast_Han_AC=2;Sou
chr6	61382799	.	G	<INS:ME:LINE1>	.	PASS	SVTYPE=INS:ME:LINE1;END=61382799;SVLEN=0;AN=20618;AC=4;AF=0.00019400523814142983;N_HET=4;N_HOMALT=0;N_HOMREF=10305;FREQ_HET=0.0003880104762828596 6;FREQ_HOMALT=0.0;FREQ_HOMREF=0.9996119895237171;N_BI_GENOS=10309;South_Han_AN=1496;South_Han_AC=1;South_Han_A
chr6	61551300	.	T	<INS:ME:LINE1>	.	PASS	SVTYPE=INS:ME:LINE1;END=61551304;SVLEN=1187;AN=20618;AC=1;AF=4.850130953535746e-05;N_HET=1;N_HOMALT=0;N_HOMREF=10308;FREQ_HET=9.700261907071492e-05;FREQ_HOMALT=0.0;FREQ_HOMREF=0.9999029973809292;N_BI_GENOS=10309;East_Han_AN=6554;East_Han_AC=1;East_Han_AF=
chr6	61633271	.	G	<INS:ME:LINE1>	.	PASS	SVTYPE=INS:ME:LINE1;END=61633281;SVLEN=889;AN=20618;AC=1;AF=4.850130953535746e-05;N_HET=1;N_HOMALT=0;N_HOMREF=10308;FREQ_HET=9.700261907071492e-05;FREQ_HOMALT=0.0;FREQ_HOMREF=0.9999029973809292;N_BI_GENOS=10309;Southeast_Han_AN=1908;Southeast_Han_AC=1;Sou
chr6	61646577	.	A	<INS:ME:LINE1>	.	PASS	SVTYPE=INS:ME:LINE1;END=61646577;SVLEN=2735;AN=20618;AC=1;AF=4.850130953535746e-05;N_HET=1;N_HOMALT=0;N_HOMREF=10308;FREQ_HET=9.700261907071492e-05;FREQ_HOMALT=0.0;FREQ_HOMREF=0.9999029973809292;N_BI_GENOS=10309;South_Han_AN=1496;South_Han_AC=1;South_Han_
chr6	61706482	.	A	<INS:ME:LINE1>	.	PASS	SVTYPE=INS:ME:LINE1;END=61706494;SVLEN=938;AN=20618;AC=1;AF=4.850130953535746e-05;N_HET=1;N_HOMALT=0;N_HOMREF=10308;FREQ_HET=9.700261907071492e-05;FREQ_HOMALT=0.0;FREQ_HOMREF=0.9999029973809292;N_BI_GENOS=10309;South_Han_AN=1496;South_Han_AC=1;South_Han_A
chr6	61741810	.	A	<INS:ME:LINE1>	.	PASS	SVTYPE=INS:ME:LINE1;END=61741828;SVLEN=2105;AN=20618;AC=1;AF=4.850130953535746e-05;N_HET=1;N_HOMALT=0;N_HOMREF=10308;FREQ_HET=9.700261907071492e-05;FREQ_HOMALT=0.0;FREQ_HOMREF=0.9999029973809292;N_BI_GENOS=10309;East_Han_AN=6554;East_Han_AC=1;East_Han_AF=
chr6	61833603	.	A	<INS:ME:LINE1>	.	PASS	SVTYPE=INS:ME:LINE1;END=61833603;SVLEN=1921;AN=20618;AC=1;AF=4.850130953535746e-05;N_HET=1;N_HOMALT=0;N_HOMREF=10308;FREQ_HET=9.700261907071492e-05;FREQ_HOMALT=0.0;FREQ_HOMREF=0.9999029973809292;N_BI_GENOS=10309;East_Han_AN=6554;East_Han_AC=1;East_Han_AF=
chr6	61866602	.	A	<INS:ME:LINE1>	.	PASS	SVTYPE=INS:ME:LINE1;END=61866607;SVLEN=385;AN=20618;AC=4;AF=0.00019400523814142983;N_HET=4;N_HOMALT=0;N_HOMREF=10305;FREQ_HET=0.0003880104762828596 6;FREQ_HOMALT=0.0;FREQ_HOMREF=0.9996119895237171;N_BI_GENOS=10309;North_Han_AN=2272;North_Han_AC=2;North_Han
chr6	61902865	.	T	<INS:ME:LINE1>	.	PASS	SVTYPE=INS:ME:LINE1;END=61902880;SVLEN=1144;AN=20618;AC=30;AF=0.0014550392860607237;N_HET=30;N_HOMALT=0;N_HOMREF=10279;FREQ_HET=0.0029100785721214474;FREQ_HOMALT=0.0;FREQ_HOMREF=0.9970899214278786;N_BI_GENOS=10309;South_Han_AN=1496;South_Han_AC=2;South_Ha
chr6	61965909	.	A	<INS:ME:LINE1>	.	PASS	SVTYPE=INS:ME:LINE1;END=61965928;SVLEN=1149;AN=20618;AC=2;AF=9.700261907071492e-05;N_HET=2;N_HOMALT=0;N_HOMREF=10307;FREQ_HET=0.0001940052381414298 3;FREQ_HOMALT=0.0;FREQ_HOMREF=0.9998059947618586;N_BI_GENOS=10309;East_Han_AN=6554;East_Han_AC=2;East_Han_AF
chr6	61979863	.	T	<INS:ME:LINE1>	.	PASS	SVTYPE=INS:ME:LINE1;END=61979881;SVLEN=1540;AN=20618;AC=1;AF=4.850130953535746e-05;N_HET=1;N_HOMALT=0;N_HOMREF=10308;FREQ_HET=9.700261907071492e-05;FREQ_HOMALT=0.0;FREQ_HOMREF=0.9999029973809292;N_BI_GENOS=10309;East_Han_AN=6554;East_Han_AC=1;East_Han_AF=
chr6	61983239	.	T	<INS:ME:LINE1>	.	PASS	SVTYPE=INS:ME:LINE1;END=61983239;SVLEN=0;AN=20618;AC=3;AF=0.00014550392860607236;N_HET=1;N_HOMALT=1;N_HOMREF=10307;FREQ_HET=9.700261907071492e-05;FREQ_HOMALT=9.700261907071492e-05;FREQ_HOMREF=0.9998059947618586;N_BI_GENOS=10309;South_Han_AN=1496;South_Han
chr6	62173551	.	C	<INS:ME:LINE1>	.	PASS	SVTYPE=INS:ME:LINE1;END=62173551;SVLEN=425;AN=20618;AC=1;AF=4.850130953535746e-05;N_HET=1;N_HOMALT=0;N_HOMREF=10308;FREQ_HET=9.700261907071492e-05;FREQ_HOMALT=0.0;FREQ_HOMREF=0.9999029973809292;N_BI_GENOS=10309;South_Han_AN=1496;South_Han_AC=1;South_Han_A
chr6	62284241	.	T	<INS:ME:LINE1>	.	PASS	SVTYPE=INS:ME:LINE1;END=62284242;SVLEN=3307;AN=20618;AC=1;AF=4.850130953535746e-05;N_HET=1;N_HOMALT=0;N_HOMREF=10308;FREQ_HET=9.700261907071492e-05;FREQ_HOMALT=0.0;FREQ_HOMREF=0.9999029973809292;N_BI_GENOS=10309;Lingnan_Han_AN=1888;Lingnan_Han_AC=1;Lingna
chr6	62484607	.	G	<INS:ME:LINE1>	.	PASS	SVTYPE=INS:ME:LINE1;END=62484608;SVLEN=3623;AN=20618;AC=49;AF=0.002376564167232515 4;N_HET=45;N_HOMALT=2;N_HOMREF=10262;FREQ_HET=0.004365117858182171;FREQ_HOMALT=0.00019400523814142983;FREQ_HOMREF=0.9954408769036764;N_BI_GENOS=10309;South_Han_AN=1496;South
chr6	62587072	.	C	<INS:ME:LINE1>	.	PASS	SVTYPE=INS:ME:LINE1;END=62587072;SVLEN=0;AN=20618;AC=349;AF=0.01692695702783975;N_HET=3;N_HOMALT=173;N_HOMREF=10133;FREQ_HET=0.0002910078572121447;FREQ_HOMALT=0.016781453099233668;FREQ_HOMREF=0.9829275390435541;N_BI_GENOS=10309;South_Han_AN=1496;South_Han_
chr6	62658290	.	T	<INS:ME:LINE1>	.	PASS	SVTYPE=INS:ME:LINE1;END=62658302;SVLEN=6000;AN=20618;AC=2980;AF=0.1445339024153652;N_HET=2344;N_HOMALT=318;N_HOMREF=7647;FREQ_HET=0.22737413910175575;FREQ_HOMALT=0.030846832864487 34;FREQ_HOMREF=0.741779028033757;N_BI_GENOS=10309;South_Han_AN=1496;South_Ha
chr6	62834591	.	T	<INS:ME:LINE1>	.	PASS	SVTYPE=INS:ME:LINE1;END=62834591;SVLEN=334;AN=20618;AC=1;AF=4.850130953535746e-05;N_HET=1;N_HOMALT=0;N_HOMREF=10308;FREQ_HET=9.700261907071492e-05;FREQ_HOMALT=0.0;FREQ_HOMREF=0.9999029973809292;N_BI_GENOS=10309;East_Han_AN=6554;East_Han_AC=1;East_Han_AF=0
chr6	63131508	.	A	<INS:ME:LINE1>	.	PASS	SVTYPE=INS:ME:LINE1;END=63131508;SVLEN=1619;AN=20618;AC=1;AF=4.850130953535746e-05;N_HET=1;N_HOMALT=0;N_HOMREF=10308;FREQ_HET=9.700261907071492e-05;FREQ_HOMALT=0.0;FREQ_HOMREF=0.9999029973809292;N_BI_GENOS=10309;Central_Han_AN=2128;Central_Han_AC=1;Centra
chr6	63855460	.	T	<INS:ME:LINE1>	.	PASS	SVTYPE=INS:ME:LINE1;END=63855470;SVLEN=664;AN=20618;AC=1;AF=4.850130953535746e-05;N_HET=1;N_HOMALT=0;N_HOMREF=10308;FREQ_HET=9.700261907071492e-05;FREQ_HOMALT=0.0;FREQ_HOMREF=0.9999029973809292;N_BI_GENOS=10309;Southeast_Han_AN=1908;Southeast_Han_AC=1;Sou
chr6	63929258	.	A	<INS:ME:LINE1>	.	PASS	SVTYPE=INS:ME:LINE1;END=63929258;SVLEN=4467;AN=20618;AC=1;AF=4.850130953535746e-05;N_HET=1;N_HOMALT=0;N_HOMREF=10308;FREQ_HET=9.700261907071492e-05;FREQ_HOMALT=0.0;FREQ_HOMREF=0.9999029973809292;N_BI_GENOS=10309;East_Han_AN=6554;East_Han_AC=1;East_Han_AF=

chr6	63964545	.	A	<INS:ME:LINE1>	.	PASS	SVTYPE=INS:ME:LINE1;END=63964560;SVLEN=504;AN=20618;AC=1;AF=4.850130953535746e-05;N_HET=1;N_HOMALT=0;N_HOMREF=10308;FREQ_HET=9.700261907071492e-05;FREQ_HOMALT=0.0;FREQ_HOMREF=0.9999029973809292;N_BI_GENOS=10309;North_Han_AN=2272;North_Han_AC=1;North_Han_A
chr6	64012496	.	A	<INS:ME:LINE1>	.	PASS	SVTYPE=INS:ME:LINE1;END=64012511;SVLEN=666;AN=20618;AC=1;AF=4.850130953535746e-05;N_HET=1;N_HOMALT=0;N_HOMREF=10308;FREQ_HET=9.700261907071492e-05;FREQ_HOMALT=0.0;FREQ_HOMREF=0.9999029973809292;N_BI_GENOS=10309;South_Han_AN=1496;South_Han_AC=1;South_Han_A
chr6	64021071	.	A	<INS:ME:LINE1>	.	PASS	SVTYPE=INS:ME:LINE1;END=64021082;SVLEN=5996;AN=20618;AC=1;AF=4.850130953535746e-05;N_HET=1;N_HOMALT=0;N_HOMREF=10308;FREQ_HET=9.700261907071492e-05;FREQ_HOMALT=0.0;FREQ_HOMREF=0.9999029973809292;N_BI_GENOS=10309;Lingnan_Han_AN=1888;Lingnan_Han_AC=1;Lingna
chr6	64112031	.	A	<INS:ME:LINE1>	.	PASS	SVTYPE=INS:ME:LINE1;END=64112048;SVLEN=887;AN=20618;AC=3;AF=0.0001455039286060 7236;N_HET=3;N_HOMALT=0;N_HOMREF=10306;FREQ_HET=0.00029100785721 21447;FREQ_HOMALT=0.0;FREQ_HOMREF=0.9997089921427879;N_BI_GENOS=10309;South_Han_AN=1496;South_Han_AC=1;South_Han_
chr6	64329219	.	A	<INS:ME:LINE1>	.	PASS	SVTYPE=INS:ME:LINE1;END=64329230;SVLEN=6000;AN=20618;AC=1;AF=4.850130953535746e-05;N_HET=1;N_HOMALT=0;N_HOMREF=10308;FREQ_HET=9.700261907071492e-05;FREQ_HOMALT=0.0;FREQ_HOMREF=0.9999029973809292;N_BI_GENOS=10309;Southeast_Han_AN=1908;Southeast_Han_AC=1;So
chr6	64494895	.	A	<INS:ME:LINE1>	.	PASS	SVTYPE=INS:ME:LINE1;END=64494901;SVLEN=428;AN=20618;AC=1;AF=4.850130953535746e-05;N_HET=1;N_HOMALT=0;N_HOMREF=10308;FREQ_HET=9.700261907071492e-05;FREQ_HOMALT=0.0;FREQ_HOMREF=0.9999029973809292;N_BI_GENOS=10309;East_Han_AN=6554;East_Han_AC=1;East_Han_AF=0
chr6	64513107	.	G	<INS:ME:LINE1>	.	PASS	SVTYPE=INS:ME:LINE1;END=64513107;SVLEN=1956;AN=20618;AC=10;AF=0.00048501309535357455;N_HET=10;N_HOMALT=0;N_HOMREF=10299;FREQ_HET=0.0009700261907071491;FREQ_HOMALT=0.0;FREQ_HOMREF=0.9990299738092928;N_BI_GENOS=10309;Northwest_Han_AN=1296;Northwest_Han_AC=2
chr6	64608222	.	A	<INS:ME:LINE1>	.	PASS	SVTYPE=INS:ME:LINE1;END=64608238;SVLEN=2681;AN=20618;AC=1;AF=4.850130953535746e-05;N_HET=1;N_HOMALT=0;N_HOMREF=10308;FREQ_HET=9.700261907071492e-05;FREQ_HOMALT=0.0;FREQ_HOMREF=0.9999029973809292;N_BI_GENOS=10309;Southeast_Han_AN=1908;Southeast_Han_AC=1;So
chr6	64617570	.	A	<INS:ME:LINE1>	.	PASS	SVTYPE=INS:ME:LINE1;END=64617580;SVLEN=1895;AN=20618;AC=1;AF=4.850130953535746e-05;N_HET=1;N_HOMALT=0;N_HOMREF=10308;FREQ_HET=9.700261907071492e-05;FREQ_HOMALT=0.0;FREQ_HOMREF=0.9999029973809292;N_BI_GENOS=10309;North_Han_AN=2272;North_Han_AC=1;North_Han_
chr6	64692351	.	A	<INS:ME:LINE1>	.	PASS	SVTYPE=INS:ME:LINE1;END=64692351;SVLEN=95;AN=20618;AC=1;AF=4.850130953535746e-05;N_HET=1;N_HOMALT=0;N_HOMREF=10308;FREQ_HET=9.700261907071492e-05;FREQ_HOMALT=0.0;FREQ_HOMREF=0.9999029973809292;N_BI_GENOS=10309;East_Han_AN=6554;East_Han_AC=1;East_Han_AF=0.
chr6	64982944	.	A	<INS:ME:LINE1>	.	PASS	SVTYPE=INS:ME:LINE1;END=64982965;SVLEN=1328;AN=20618;AC=1;AF=4.850130953535746e-05;N_HET=1;N_HOMALT=0;N_HOMREF=10308;FREQ_HET=9.700261907071492e-05;FREQ_HOMALT=0.0;FREQ_HOMREF=0.9999029973809292;N_BI_GENOS=10309;East_Han_AN=6554;East_Han_AC=1;East_Han_AF=
chr6	65129629	.	C	<INS:ME:LINE1>	.	PASS	SVTYPE=INS:ME:LINE1;END=65129629;SVLEN=750;AN=20618;AC=1;AF=4.850130953535746e-05;N_HET=1;N_HOMALT=0;N_HOMREF=10308;FREQ_HET=9.700261907071492e-05;FREQ_HOMALT=0.0;FREQ_HOMREF=0.9999029973809292;N_BI_GENOS=10309;East_Han_AN=6554;East_Han_AC=1;East_Han_AF=0
chr6	65248032	.	A	<INS:ME:LINE1>	.	PASS	SVTYPE=INS:ME:LINE1;END=65248049;SVLEN=5993;AN=20618;AC=5;AF=0.00024250654767678728;N_HET=5;N_HOMALT=0;N_HOMREF=10304;FREQ_HET=0.00048501309535357455;FREQ_HOMALT=0.0;FREQ_HOMREF=0.9995149869046465;N_BI_GENOS=10309;East_Han_AN=6554;East_Han_AC=3;East_Han_A
chr6	65294420	.	G	<INS:ME:LINE1>	.	PASS	SVTYPE=INS:ME:LINE1;END=65294436;SVLEN=2701;AN=20618;AC=1;AF=4.850130953535746e-05;N_HET=1;N_HOMALT=0;N_HOMREF=10308;FREQ_HET=9.700261907071492e-05;FREQ_HOMALT=0.0;FREQ_HOMREF=0.9999029973809292;N_BI_GENOS=10309;Tibetan_AN=414;Tibetan_AC=1;Tibetan_AF=0.00
chr6	65516574	.	T	<INS:ME:LINE1>	.	PASS	SVTYPE=INS:ME:LINE1;END=65516579;SVLEN=412;AN=20618;AC=1;AF=4.850130953535746e-05;N_HET=1;N_HOMALT=0;N_HOMREF=10308;FREQ_HET=9.700261907071492e-05;FREQ_HOMALT=0.0;FREQ_HOMREF=0.9999029973809292;N_BI_GENOS=10309;East_Han_AN=6554;East_Han_AC=1;East_Han_AF=0
chr6	65694793	.	A	<INS:ME:LINE1>	.	PASS	SVTYPE=INS:ME:LINE1;END=65694793;SVLEN=680;AN=20618;AC=12;AF=0.0005820157144242894;N_HET=10;N_HOMALT=1;N_HOMREF=10298;FREQ_HET=0.0009700261907071491;FREQ_HOMALT=9.700261907071492e-05;FREQ_HOMREF=0.9989329711902222;N_BI_GENOS=10309;Lingnan_Han_AN=1888;Ling
chr6	65723911	.	A	<INS:ME:LINE1>	.	PASS	SVTYPE=INS:ME:LINE1;END=65723923;SVLEN=787;AN=20618;AC=1;AF=4.850130953535746e-05;N_HET=1;N_HOMALT=0;N_HOMREF=10308;FREQ_HET=9.700261907071492e-05;FREQ_HOMALT=0.0;FREQ_HOMREF=0.9999029973809292;N_BI_GENOS=10309;East_Han_AN=6554;East_Han_AC=1;East_Han_AF=0
chr6	66036454	.	T	<INS:ME:LINE1>	.	PASS	SVTYPE=INS:ME:LINE1;END=66036467;SVLEN=915;AN=20618;AC=2;AF=9.700261907071492e-05;N_HET=2;N_HOMALT=0;N_HOMREF=10307;FREQ_HET=0.00019400523814142983;FREQ_HOMALT=0.0;FREQ_HOMREF=0.9998059947618586;N_BI_GENOS=10309;East_Han_AN=6554;East_Han_AC=1;East_Han_AF=
chr6	66448636	.	A	<INS:ME:LINE1>	.	PASS	SVTYPE=INS:ME:LINE1;END=66448679;SVLEN=936;AN=20618;AC=2;AF=9.700261907071492e-05;N_HET=2;N_HOMALT=0;N_HOMREF=10307;FREQ_HET=0.00019400523814142983;FREQ_HOMALT=0.0;FREQ_HOMREF=0.9998059947618586;N_BI_GENOS=10309;East_Han_AN=6554;East_Han_AC=2;East_Han_AF=
chr6	66511972	.	A	<INS:ME:LINE1>	.	PASS	SVTYPE=INS:ME:LINE1;END=66511980;SVLEN=1538;AN=20618;AC=1;AF=4.850130953535746e-05;N_HET=1;N_HOMALT=0;N_HOMREF=10308;FREQ_HET=9.700261907071492e-05;FREQ_HOMALT=0.0;FREQ_HOMREF=0.9999029973809292;N_BI_GENOS=10309;South_Han_AN=1496;South_Han_AC=1;South_Han_
chr6	66663822	.	A	<INS:ME:LINE1>	.	PASS	SVTYPE=INS:ME:LINE1;END=66663828;SVLEN=5979;AN=20618;AC=4;AF=0.00019400523814142983;N_HET=2;N_HOMALT=1;N_HOMREF=10306;FREQ_HET=0.00019400523814142983;FREQ_HOMALT=9.700261907071492e-05;FREQ_HOMREF=0.9997089921427879;N_BI_GENOS=10309;East_Han_AN=6554;East_H
chr6	66682199	.	G	<INS:ME:LINE1>	.	PASS	SVTYPE=INS:ME:LINE1;END=66682215;SVLEN=1970;AN=20618;AC=1;AF=4.850130953535746e-05;N_HET=1;N_HOMALT=0;N_HOMREF=10308;FREQ_HET=9.700261907071492e-05;FREQ_HOMALT=0.0;FREQ_HOMREF=0.9999029973809292;N_BI_GENOS=10309;Lingnan_Han_AN=1888;Lingnan_Han_AC=1;Lingna
chr6	66718575	.	A	<INS:ME:LINE1>	.	PASS	SVTYPE=INS:ME:LINE1;END=66718586;SVLEN=152;AN=20618;AC=1;AF=4.850130953535746e-05;N_HET=1;N_HOMALT=0;N_HOMREF=10308;FREQ_HET=9.700261907071492e-05;FREQ_HOMALT=0.0;FREQ_HOMREF=0.9999029973809292;N_BI_GENOS=10309;Manchu_AN=416;Manchu_AC=1;Manchu_AF=0.002403
chr6	66835893	.	T	<INS:ME:LINE1>	.	PASS	SVTYPE=INS:ME:LINE1;END=66835901;SVLEN=1467;AN=20618;AC=2;AF=9.700261907071492e-05;N_HET=2;N_HOMALT=0;N_HOMREF=10307;FREQ_HET=0.00019400523814142983;FREQ_HOMALT=0.0;FREQ_HOMREF=0.9998059947618586;N_BI_GENOS=10309;East_Han_AN=6554;East_Han_AC=1;East_Han_AF
chr6	66912858	.	A	<INS:ME:LINE1>	.	PASS	SVTYPE=INS:ME:LINE1;END=66912867;SVLEN=598;AN=20618;AC=1;AF=4.850130953535746e-05;N_HET=1;N_HOMALT=0;N_HOMREF=10308;FREQ_HET=9.700261907071492e-05;FREQ_HOMALT=0.0;FREQ_HOMREF=0.9999029973809292;N_BI_GENOS=10309;Northwest_Han_AN=1296;Northwest_Han_AC=1;Nor
chr6	67003805	.	A	<INS:ME:LINE1>	.	PASS	SVTYPE=INS:ME:LINE1;END=67003814;SVLEN=118;AN=20618;AC=1;AF=4.850130953535746e-05;N_HET=1;N_HOMALT=0;N_HOMREF=10308;FREQ_HET=9.700261907071492e-05;FREQ_HOMALT=0.0;FREQ_HOMREF=0.9999029973809292;N_BI_GENOS=10309;Lingnan_Han_AN=1888;Lingnan_Han_AC=1;Lingnan
chr6	67150550	.	A	<INS:ME:LINE1>	.	PASS	SVTYPE=INS:ME:LINE1;END=67150566;SVLEN=4344;AN=20618;AC=1;AF=4.850130953535746e-05;N_HET=1;N_HOMALT=0;N_HOMREF=10308;FREQ_HET=9.700261907071492e-05;FREQ_HOMALT=0.0;FREQ_HOMREF=0.9999029973809292;N_BI_GENOS=10309;Southeast_Han_AN=1908;Southeast_Han_AC=1;So
chr6	67169801	.	C	<INS:ME:LINE1>	.	PASS	SVTYPE=INS:ME:LINE1;END=67169801;SVLEN=453;AN=20618;AC=1;AF=4.850130953535746e-05;N_HET=1;N_HOMALT=0;N_HOMREF=10308;FREQ_HET=9.700261907071492e-05;FREQ_HOMALT=0.0;FREQ_HOMREF=0.9999029973809292;N_BI_GENOS=10309;East_Han_AN=6554;East_Han_AC=1;East_Han_AF=0
chr6	67194429	.	T	<INS:ME:LINE1>	.	PASS	SVTYPE=INS:ME:LINE1;END=67194444;SVLEN=956;AN=20618;AC=1;AF=4.850130953535746e-05;N_HET=1;N_HOMALT=0;N_HOMREF=10308;FREQ_HET=9.700261907071492e-05;FREQ_HOMALT=0.0;FREQ_HOMREF=0.9999029973809292;N_BI_GENOS=10309;Central_Han_AN=2128;Central_Han_AC=1;Central
chr6	67213495	.	G	<INS:ME:LINE1>	.	PASS	SVTYPE=INS:ME:LINE1;END=67213513;SVLEN=924;AN=20618;AC=1;AF=4.850130953535746e-05;N_HET=1;N_HOMALT=0;N_HOMREF=10308;FREQ_HET=9.700261907071492e-05;FREQ_HOMALT=0.0;FREQ_HOMREF=0.9999029973809292;N_BI_GENOS=10309;East_Han_AN=6554;East_Han_AC=1;East_Han_AF=0
chr6	67245610	.	T	<INS:ME:LINE1>	.	PASS	SVTYPE=INS:ME:LINE1;END=67245610;SVLEN=1364;AN=20618;AC=1;AF=4.850130953535746e-05;N_HET=1;N_HOMALT=0;N_HOMREF=10308;FREQ_HET=9.700261907071492e-05;FREQ_HOMALT=0.0;FREQ_HOMREF=0.9999029973809292;N_BI_GENOS=10309;Central_Han_AN=2128;Central_Han_AC=1;Centra
chr6	67420279	.	A	<INS:ME:LINE1>	.	PASS	SVTYPE=INS:ME:LINE1;END=67420296;SVLEN=1244;AN=20618;AC=1;AF=4.850130953535746e-05;N_HET=1;N_HOMALT=0;N_HOMREF=10308;FREQ_HET=9.700261907071492e-05;FREQ_HOMALT=0.0;FREQ_HOMREF=0.9999029973809292;N_BI_GENOS=10309;Southeast_Han_AN=1908;Southeast_Han_AC=1;So
chr6	67422456	.	T	<INS:ME:LINE1>	.	PASS	SVTYPE=INS:ME:LINE1;END=67422472;SVLEN=969;AN=20618;AC=4;AF=0.00019400523814142983;N_HET=4;N_HOMALT=0;N_HOMREF=10305;FREQ_HET=0.00038801047628285966;FREQ_HOMALT=0.0;FREQ_HOMREF=0.9996119895237171;N_BI_GENOS=10309;Northwest_Han_AN=1296;Northwest_Han_AC=2;N
chr6	67434709	.	A	<INS:ME:LINE1>	.	PASS	SVTYPE=INS:ME:LINE1;END=67434718;SVLEN=6056;AN=20618;AC=1;AF=4.850130953535746e-05;N_HET=1;N_HOMALT=0;N_HOMREF=10308;FREQ_HET=9.700261907071492e-05;FREQ_HOMALT=0.0;FREQ_HOMREF=0.9999029973809292;N_BI_GENOS=10309;Northwest_Han_AN=1296;Northwest_Han_AC=1;No
chr6	67435961	.	A	<INS:ME:LINE1>	.	PASS	SVTYPE=INS:ME:LINE1;END=67435977;SVLEN=315;AN=20618;AC=3;AF=0.00014550392860607236;N_HET=3;N_HOMALT=0;N_HOMREF=10306;FREQ_HET=0.0002910078572121447;FREQ_HOMALT=0.0;FREQ_HOMREF=0.9997089921427879;N_BI_GENOS=10309;Lingnan_Han_AN=1888;Lingnan_Han_AC=1;Lingna
chr6	67569576	.	A	<INS:ME:LINE1>	.	PASS	SVTYPE=INS:ME:LINE1;END=67569590;SVLEN=6020;AN=20618;AC=2;AF=9.700261907071492e-05;N_HET=0;N_HOMALT=1;N_HOMREF=10308;FREQ_HET=0.0;FREQ_HOMALT=9.700261907071492e-05;FREQ_HOMREF=0.9999029973809292;N_BI_GENOS=10309;Southeast_Han_AN=1908;Southeast_Han_AC=2;So
chr6	67836712	.	A	<INS:ME:LINE1>	.	PASS	SVTYPE=INS:ME:LINE1;END=67836719;SVLEN=436;AN=20618;AC=3;AF=0.00014550392860607236;N_HET=3;N_HOMALT=0;N_HOMREF=10306;FREQ_HET=0.0002910078572121447;FREQ_HOMALT=0.0;FREQ_HOMREF=0.9997089921427879;N_BI_GENOS=10309;Lingnan_Han_AN=1888;Lingnan_Han_AC=1;Lingna
chr6	68414900	.	A	<INS:ME:LINE1>	.	PASS	SVTYPE=INS:ME:LINE1;END=68414916;SVLEN=1275;AN=20618;AC=17;AF=0.0008245222621010767;N_HET=17;N_HOMALT=0;N_HOMREF=10292;FREQ_HET=0.0016490445242021534;FREQ_HOMALT=0.0;FREQ_HOMREF=0.9983509554757979;N_BI_GENOS=10309;Northwest_Han_AN=1296;Northwest_Han_AC=2;
chr6	68429609	.	A	<INS:ME:LINE1>	.	PASS	SVTYPE=INS:ME:LINE1;END=68429629;SVLEN=1513;AN=20618;AC=1;AF=4.850130953535746e-05;N_HET=1;N_HOMALT=0;N_HOMREF=10308;FREQ_HET=9.700261907071492e-05;FREQ_HOMALT=0.0;FREQ_HOMREF=0.9999029973809292;N_BI_GENOS=10309;Southeast_Han_AN=1908;Southeast_Han_AC=1;So
chr6	68488836	.	T	<INS:ME:LINE1>	.	PASS	SVTYPE=INS:ME:LINE1;END=68488853;SVLEN=2292;AN=20618;AC=4;AF=0.00019400523814142983;N_HET=4;N_HOMALT=0;N_HOMREF=10305;FREQ_HET=0.00038801047628285966;FREQ_HOMALT=0.0;FREQ_HOMREF=0.9996119895237171;N_BI_GENOS=10309;South_Han_AN=1496;South_Han_AC=1;South_Ha
chr6	68568267	.	T	<INS:ME:LINE1>	.	PASS	SVTYPE=INS:ME:LINE1;END=68568267;SVLEN=400;AN=20618;AC=1;AF=4.850130953535746e-05;N_HET=1;N_HOMALT=0;N_HOMREF=10308;FREQ_HET=9.700261907071492e-05;FREQ_HOMALT=0.0;FREQ_HOMREF=0.9999029973809292;N_BI_GENOS=10309;East_Han_AN=6554;East_Han_AC=1;East_Han_AF=0
chr6	68735672	.	G	<INS:ME:LINE1>	.	PASS	SVTYPE=INS:ME:LINE1;END=68735686;SVLEN=6007;AN=20618;AC=1;AF=4.850130953535746e-05;N_HET=1;N_HOMALT=0;N_HOMREF=10308;FREQ_HET=9.700261907071492e-05;FREQ_HOMALT=0.0;FREQ_HOMREF=0.9999029973809292;N_BI_GENOS=10309;Northwest_Han_AN=1296;Northwest_Han_AC=1;No
chr6	68828964	.	A	<INS:ME:LINE1>	.	PASS	SVTYPE=INS:ME:LINE1;END=68828974;SVLEN=536;AN=20618;AC=5;AF=0.00024250654767678728;N_HET=3;N_HOMALT=1;N_HOMREF=10305;FREQ_HET=0.0002910078572121447;FREQ_HOMALT=9.700261907071492e-05;FREQ_HOMREF=0.9996119895237171;N_BI_GENOS=10309;East_Han_AN=6554;East_Han
chr6	68940903	.	A	<INS:ME:LINE1>	.	PASS	SVTYPE=INS:ME:LINE1;END=68940920;SVLEN=428;AN=20618;AC=11;AF=0.0005335144044888932;N_HET=11;N_HOMALT=0;N_HOMREF=10298;FREQ_HET=0.001067028809777864;FREQ_HOMALT=0.0;FREQ_HOMREF=0.9989329711902222;N_BI_GENOS=10309;South_Han_AN=1496;South_Han_AC=1;South_Han_A
chr6	69028254	.	T	<INS:ME:LINE1>	.	PASS	SVTYPE=INS:ME:LINE1;END=69028270;SVLEN=321;AN=20618;AC=1;AF=4.850130953535746e-05;N_HET=1;N_HOMALT=0;N_HOMREF=10308;FREQ_HET=9.700261907071492e-05;FREQ_HOMALT=0.0;FREQ_HOMREF=0.9999029973809292;N_BI_GENOS=10309;Northwest_Han_AN=1296;Northwest_Han_AC=1;Nor
chr6	69610016	.	A	<INS:ME:LINE1>	.	PASS	SVTYPE=INS:ME:LINE1;END=69610034;SVLEN=1469;AN=20618;AC=1;AF=4.850130953535746e-05;N_HET=1;N_HOMALT=0;N_HOMREF=10308;FREQ_HET=9.700261907071492e-05;FREQ_HOMALT=0.0;FREQ_HOMREF=0.9999029973809292;N_BI_GENOS=10309;East_Han_AN=6554;East_Han_AC=1;East_Han_AF=
chr6	69992023	.	A	<INS:ME:LINE1>	.	PASS	SVTYPE=INS:ME:LINE1;END=69992026;SVLEN=1082;AN=20618;AC=1;AF=4.850130953535746e-05;N_HET=1;N_HOMALT=0;N_HOMREF=10308;FREQ_HET=9.700261907071492e-05;FREQ_HOMALT=0.0;FREQ_HOMREF=0.9999029973809292;N_BI_GENOS=10309;East_Han_AN=6554;East_Han_AC=1;East_Han_AF=
chr6	69992389	.	T	<INS:ME:LINE1>	.	PASS	SVTYPE=INS:ME:LINE1;END=69992389;SVLEN=79;AN=20618;AC=1;AF=4.850130953535746e-05;N_HET=1;N_HOMALT=0;N_HOMREF=10308;FREQ_HET=9.700261907071492e-05;FREQ_HOMALT=0.0;FREQ_HOMREF=0.9999029973809292;N_BI_GENOS=10309;East_Han_AN=6554;East_Han_AC=1;East_Han_AF=0.
chr6	70736251	.	A	<INS:ME:LINE1>	.	PASS	SVTYPE=INS:ME:LINE1;END=70736268;SVLEN=6004;AN=20618;AC=1;AF=4.850130953535746e-05;N_HET=1;N_HOMALT=0;N_HOMREF=10308;FREQ_HET=9.700261907071492e-05;FREQ_HOMALT=0.0;FREQ_HOMREF=0.9999029973809292;N_BI_GENOS=10309;Manchu_AN=416;Manchu_AC=1;Manchu_AF=0.00240
chr6	71290715	.	T	<INS:ME:LINE1>	.	PASS	SVTYPE=INS:ME:LINE1;END=71290728;SVLEN=1535;AN=20618;AC=2;AF=9.700261907071492e-05;N_HET=2;N_HOMALT=0;N_HOMREF=10307;FREQ_HET=0.00019400523814142983;FREQ_HOMALT=0.0;FREQ_HOMREF=0.9998059947618586;N_BI_GENOS=10309;Northwest_Han_AN=1296;Northwest_Han_AC=1;N
chr6	71329041	.	C	<INS:ME:LINE1>	.	PASS	SVTYPE=INS:ME:LINE1;END=71329055;SVLEN=5997;AN=20618;AC=768;AF=0.037249005723154524;N_HET=702;N_HOMALT=33;N_HOMREF=9574;FREQ_HET=0.06809583858764187;FREQ_HOMALT=0.0032010864293335 92;FREQ_HOMREF=0.9287030749830245;N_BI_GENOS=10309;South_Han_AN=1496;South_H
chr6	71534236	.	A	<INS:ME:LINE1>	.	PASS	SVTYPE=INS:ME:LINE1;END=71534252;SVLEN=908;AN=20618;AC=1;AF=4.850130953535746e-05;N_HET=1;N_HOMALT=0;N_HOMREF=10308;FREQ_HET=9.700261907071492e-05;FREQ_HOMALT=0.0;FREQ_HOMREF=0.9999029973809292;N_BI_GENOS=10309;East_Han_AN=6554;East_Han_AC=1;East_Han_AF=0
chr6	71580632	.	A	<INS:ME:LINE1>	.	PASS	SVTYPE=INS:ME:LINE1;END=71580650;SVLEN=398;AN=20618;AC=1;AF=4.850130953535746e-05;N_HET=1;N_HOMALT=0;N_HOMREF=10308;FREQ_HET=9.700261907071492e-05;FREQ_HOMALT=0.0;FREQ_HOMREF=0.9999029973809292;N_BI_GENOS=10309;Central_Han_AN=2128;Central_Han_AC=1;Central
chr6	71947360	.	C	<INS:ME:LINE1>	.	PASS	SVTYPE=INS:ME:LINE1;END=71947372;SVLEN=6015;AN=20618;AC=1;AF=4.850130953535746e-05;N_HET=1;N_HOMALT=0;N_HOMREF=10308;FREQ_HET=9.700261907071492e-05;FREQ_HOMALT=0.0;FREQ_HOMREF=0.9999029973809292;N_BI_GENOS=10309;Lingnan_Han_AN=1888;Lingnan_Han_AC=1;Lingna
chr6	72093704	.	T	<INS:ME:LINE1>	.	PASS	SVTYPE=INS:ME:LINE1;END=72093716;SVLEN=5999;AN=20618;AC=1;AF=4.850130953535746e-05;N_HET=1;N_HOMALT=0;N_HOMREF=10308;FREQ_HET=9.700261907071492e-05;FREQ_HOMALT=0.0;FREQ_HOMREF=0.9999029973809292;N_BI_GENOS=10309;Southeast_Han_AN=1908;Southeast_Han_AC=1;So
chr6	72398920	.	A	<INS:ME:LINE1>	.	PASS	SVTYPE=INS:ME:LINE1;END=72398930;SVLEN=335;AN=20618;AC=1;AF=4.850130953535746e-05;N_HET=1;N_HOMALT=0;N_HOMREF=10308;FREQ_HET=9.700261907071492e-05;FREQ_HOMALT=0.0;FREQ_HOMREF=0.9999029973809292;N_BI_GENOS=10309;East_Han_AN=6554;East_Han_AC=1;East_Han_AF=0
chr6	72779095	.	A	<INS:ME:LINE1>	.	PASS	SVTYPE=INS:ME:LINE1;END=72779111;SVLEN=1377;AN=20618;AC=1;AF=4.850130953535746e-05;N_HET=1;N_HOMALT=0;N_HOMREF=10308;FREQ_HET=9.700261907071492e-05;FREQ_HOMALT=0.0;FREQ_HOMREF=0.9999029973809292;N_BI_GENOS=10309;Mongolian_AN=454;Mongolian_AC=1;Mongolian_A
chr6	72853930	.	A	<INS:ME:LINE1>	.	PASS	SVTYPE=INS:ME:LINE1;END=72853930;SVLEN=1230;AN=20618;AC=1;AF=4.850130953535746e-05;N_HET=1;N_HOMALT=0;N_HOMREF=10308;FREQ_HET=9.700261907071492e-05;FREQ_HOMALT=0.0;FREQ_HOMREF=0.9999029973809292;N_BI_GENOS=10309;Central_Han_AN=2128;Central_Han_AC=1;Centra
chr6	72911376	.	A	<INS:ME:LINE1>	.	PASS	SVTYPE=INS:ME:LINE1;END=72911387;SVLEN=1094;AN=20618;AC=1;AF=4.850130953535746e-05;N_HET=1;N_HOMALT=0;N_HOMREF=10308;FREQ_HET=9.700261907071492e-05;FREQ_HOMALT=0.0;FREQ_HOMREF=0.9999029973809292;N_BI_GENOS=10309;South_Han_AN=1496;South_Han_AC=1;South_Han_
chr6	72931167	.	A	<INS:ME:LINE1>	.	PASS	SVTYPE=INS:ME:LINE1;END=72931181;SVLEN=5999;AN=20618;AC=8;AF=0.00038801047628285966;N_HET=8;N_HOMALT=0;N_HOMREF=10301;FREQ_HET=0.0007760209525657193;FREQ_HOMALT=0.0;FREQ_HOMREF=0.9992239790474343;N_BI_GENOS=10309;South_Han_AN=1496;South_Han_AC=1;South_Han
chr6	73053141	.	G	<INS:ME:LINE1>	.	PASS	SVTYPE=INS:ME:LINE1;END=73053183;SVLEN=4000;AN=20618;AC=1;AF=4.850130953535746e-05;N_HET=1;N_HOMALT=0;N_HOMREF=10308;FREQ_HET=9.700261907071492e-05;FREQ_HOMALT=0.0;FREQ_HOMREF=0.9999029973809292;N_BI_GENOS=10309;East_Han_AN=6554;East_Han_AC=1;East_Han_AF=
chr6	73069274	.	T	<INS:ME:LINE1>	.	PASS	SVTYPE=INS:ME:LINE1;END=73069291;SVLEN=1550;AN=20618;AC=1;AF=4.850130953535746e-05;N_HET=1;N_HOMALT=0;N_HOMREF=10308;FREQ_HET=9.700261907071492e-05;FREQ_HOMALT=0.0;FREQ_HOMREF=0.9999029973809292;N_BI_GENOS=10309;East_Han_AN=6554;East_Han_AC=1;East_Han_AF=
chr6	73237056	.	A	<INS:ME:LINE1>	.	PASS	SVTYPE=INS:ME:LINE1;END=73237071;SVLEN=186;AN=20618;AC=1;AF=4.850130953535746e-05;N_HET=1;N_HOMALT=0;N_HOMREF=10308;FREQ_HET=9.700261907071492e-05;FREQ_HOMALT=0.0;FREQ_HOMREF=0.9999029973809292;N_BI_GENOS=10309;Yi_AN=422;Yi_AC=1;Yi_AF=0.002369668246445498
chr6	73485339	.	C	<INS:ME:LINE1>	.	PASS	SVTYPE=INS:ME:LINE1;END=73485352;SVLEN=69;AN=20618;AC=17;AF=0.0008245222621010767;N_HET=15;N_HOMALT=1;N_HOMREF=10293;FREQ_HET=0.0014550392860607237;FREQ_HOMALT=9.700261907071492e-05;FREQ_HOMREF=0.9984479580948685;N_BI_GENOS=10309;South_Han_AN=1496;South_H
chr6	73515758	.	A	<INS:ME:LINE1>	.	PASS	SVTYPE=INS:ME:LINE1;END=73515773;SVLEN=5998;AN=20618;AC=1;AF=4.850130953535746e-05;N_HET=1;N_HOMALT=0;N_HOMREF=10308;FREQ_HET=9.700261907071492e-05;FREQ_HOMALT=0.0;FREQ_HOMREF=0.9999029973809292;N_BI_GENOS=10309;Mongolian_AN=454;Mongolian_AC=1;Mongolian_A
chr6	73698507	.	A	<INS:ME:LINE1>	.	PASS	SVTYPE=INS:ME:LINE1;END=73698520;SVLEN=5987;AN=20618;AC=1;AF=4.850130953535746e-05;N_HET=1;N_HOMALT=0;N_HOMREF=10308;FREQ_HET=9.700261907071492e-05;FREQ_HOMALT=0.0;FREQ_HOMREF=0.9999029973809292;N_BI_GENOS=10309;North_Han_AN=2272;North_Han_AC=1;North_Han_
chr6	73818062	.	A	<INS:ME:LINE1>	.	PASS	SVTYPE=INS:ME:LINE1;END=73818078;SVLEN=628;AN=20618;AC=148;AF=0.007178193811232903;N_HET=142;N_HOMALT=3;N_HOMREF=10164;FREQ_HET=0.013774371908041518;FREQ_HOMALT=0.0002910078572121447;FREQ_HOMREF=0.9859346202347463;N_BI_GENOS=10309;South_Han_AN=1496;South_
chr6	73951906	.	A	<INS:ME:LINE1>	.	PASS	SVTYPE=INS:ME:LINE1;END=73951925;SVLEN=1795;AN=20618;AC=1;AF=4.850130953535746e-05;N_HET=1;N_HOMALT=0;N_HOMREF=10308;FREQ_HET=9.700261907071492e-05;FREQ_HOMALT=0.0;FREQ_HOMREF=0.9999029973809292;N_BI_GENOS=10309;Lingnan_Han_AN=1888;Lingnan_Han_AC=1;Lingna
chr6	74158741	.	A	<INS:ME:LINE1>	.	PASS	SVTYPE=INS:ME:LINE1;END=74158750;SVLEN=331;AN=20618;AC=1;AF=4.850130953535746e-05;N_HET=1;N_HOMALT=0;N_HOMREF=10308;FREQ_HET=9.700261907071492e-05;FREQ_HOMALT=0.0;FREQ_HOMREF=0.9999029973809292;N_BI_GENOS=10309;East_Han_AN=6554;East_Han_AC=1;East_Han_AF=0
chr6	74159774	.	T	<INS:ME:LINE1>	.	PASS	SVTYPE=INS:ME:LINE1;END=74159774;SVLEN=164;AN=20618;AC=1;AF=4.850130953535746e-05;N_HET=1;N_HOMALT=0;N_HOMREF=10308;FREQ_HET=9.700261907071492e-05;FREQ_HOMALT=0.0;FREQ_HOMREF=0.9999029973809292;N_BI_GENOS=10309;East_Han_AN=6554;East_Han_AC=1;East_Han_AF=0
chr6	74186915	.	G	<INS:ME:LINE1>	.	PASS	SVTYPE=INS:ME:LINE1;END=74186928;SVLEN=5997;AN=20618;AC=295;AF=0.01430788631293045;N_HET=283;N_HOMALT=6;N_HOMREF=10020;FREQ_HET=0.027451741197012318;FREQ_HOMALT=0.0005820157144242894;FREQ_HOMREF=0.9719662430885634;N_BI_GENOS=10309;South_Han_AN=1496;South_
chr6	74211018	.	C	<INS:ME:LINE1>	.	PASS	SVTYPE=INS:ME:LINE1;END=74211031;SVLEN=5997;AN=20618;AC=7;AF=0.0003395091667475022;N_HET=7;N_HOMALT=0;N_HOMREF=10302;FREQ_HET=0.0006790183334950044;FREQ_HOMALT=0.0;FREQ_HOMREF=0.999320981666505;N_BI_GENOS=10309;South_Han_AN=1496;South_Han_AC=1;South_Han_A
chr6	74225859	.	A	<INS:ME:LINE1>	.	PASS	SVTYPE=INS:ME:LINE1;END=74225874;SVLEN=3355;AN=20618;AC=1238;AF=0.06004462120477253;N_HET=1108;N_HOMALT=65;N_HOMREF=9136;FREQ_HET=0.10747890193035212;FREQ_HOMALT=0.006305170239596469;FREQ_HOMREF=0.8862159278300514;N_BI_GENOS=10309;South_Han_AN=1496;South_
chr6	74408911	.	C	<INS:ME:LINE1>	.	PASS	SVTYPE=INS:ME:LINE1;END=74408928;SVLEN=2883;AN=20618;AC=2;AF=9.700261907071492e-05;N_HET=2;N_HOMALT=0;N_HOMREF=10307;FREQ_HET=0.00019400523814142983;FREQ_HOMALT=0.0;FREQ_HOMREF=0.9998059947618586;N_BI_GENOS=10309;Central_Han_AN=2128;Central_Han_AC=2;Centr
chr6	74445092	.	A	<INS:ME:LINE1>	.	PASS	SVTYPE=INS:ME:LINE1;END=74445108;SVLEN=891;AN=20618;AC=1;AF=4.850130953535746e-05;N_HET=1;N_HOMALT=0;N_HOMREF=10308;FREQ_HET=9.700261907071492e-05;FREQ_HOMALT=0.0;FREQ_HOMREF=0.9999029973809292;N_BI_GENOS=10309;Tibetan_AN=414;Tibetan_AC=1;Tibetan_AF=0.002
chr6	74594634	.	T	<INS:ME:LINE1>	.	PASS	SVTYPE=INS:ME:LINE1;END=74594635;SVLEN=61;AN=20618;AC=1;AF=4.850130953535746e-05;N_HET=1;N_HOMALT=0;N_HOMREF=10308;FREQ_HET=9.700261907071492e-05;FREQ_HOMALT=0.0;FREQ_HOMREF=0.9999029973809292;N_BI_GENOS=10309;Southeast_Han_AN=1908;Southeast_Han_AC=1;Sout
chr6	74656029	.	A	<INS:ME:LINE1>	.	PASS	SVTYPE=INS:ME:LINE1;END=74656046;SVLEN=492;AN=20618;AC=1;AF=4.850130953535746e-05;N_HET=1;N_HOMALT=0;N_HOMREF=10308;FREQ_HET=9.700261907071492e-05;FREQ_HOMALT=0.0;FREQ_HOMREF=0.9999029973809292;N_BI_GENOS=10309;North_Han_AN=2272;North_Han_AC=1;North_Han_A
chr6	75063270	.	G	<INS:ME:LINE1>	.	PASS	SVTYPE=INS:ME:LINE1;END=75063280;SVLEN=1950;AN=20618;AC=1;AF=4.850130953535746e-05;N_HET=1;N_HOMALT=0;N_HOMREF=10308;FREQ_HET=9.700261907071492e-05;FREQ_HOMALT=0.0;FREQ_HOMREF=0.9999029973809292;N_BI_GENOS=10309;East_Han_AN=6554;East_Han_AC=1;East_Han_AF=
chr6	75190186	.	A	<INS:ME:LINE1>	.	PASS	SVTYPE=INS:ME:LINE1;END=75190202;SVLEN=546;AN=20618;AC=1;AF=4.850130953535746e-05;N_HET=1;N_HOMALT=0;N_HOMREF=10308;FREQ_HET=9.700261907071492e-05;FREQ_HOMALT=0.0;FREQ_HOMREF=0.9999029973809292;N_BI_GENOS=10309;East_Han_AN=6554;East_Han_AC=1;East_Han_AF=0
chr6	75283190	.	C	<INS:ME:LINE1>	.	PASS	SVTYPE=INS:ME:LINE1;END=75283204;SVLEN=3109;AN=20618;AC=2;AF=9.700261907071492e-05;N_HET=2;N_HOMALT=0;N_HOMREF=10307;FREQ_HET=0.00019400523814142983;FREQ_HOMALT=0.0;FREQ_HOMREF=0.9998059947618586;N_BI_GENOS=10309;East_Han_AN=6554;East_Han_AC=2;East_Han_AF
chr6	75898459	.	T	<INS:ME:LINE1>	.	PASS	SVTYPE=INS:ME:LINE1;END=75898464;SVLEN=1026;AN=20618;AC=1;AF=4.850130953535746e-05;N_HET=1;N_HOMALT=0;N_HOMREF=10308;FREQ_HET=9.700261907071492e-05;FREQ_HOMALT=0.0;FREQ_HOMREF=0.9999029973809292;N_BI_GENOS=10309;East_Han_AN=6554;East_Han_AC=1;East_Han_AF=
chr6	75927807	.	A	<INS:ME:LINE1>	.	PASS	SVTYPE=INS:ME:LINE1;END=75927824;SVLEN=4719;AN=20618;AC=2644;AF=0.12823746241148512;N_HET=2124;N_HOMALT=260;N_HOMREF=7925;FREQ_HET=0.20603356290619848;FREQ_HOMALT=0.025220680958385876;FREQ_HOMREF=0.7687457561354156;N_BI_GENOS=10309;South_Han_AN=1496;South
chr6	75970256	.	A	<INS:ME:LINE1>	.	PASS	SVTYPE=INS:ME:LINE1;END=75970270;SVLEN=6029;AN=20618;AC=2;AF=9.700261907071492e-05;N_HET=2;N_HOMALT=0;N_HOMREF=10307;FREQ_HET=0.00019400523814142983;FREQ_HOMALT=0.0;FREQ_HOMREF=0.9998059947618586;N_BI_GENOS=10309;Lingnan_Han_AN=1888;Lingnan_Han_AC=1;Lingn
chr6	76046316	.	A	<INS:ME:LINE1>	.	PASS	SVTYPE=INS:ME:LINE1;END=76046330;SVLEN=5999;AN=20618;AC=4;AF=0.00019400523814142983;N_HET=4;N_HOMALT=0;N_HOMREF=10305;FREQ_HET=0.00038801047628285966;FREQ_HOMALT=0.0;FREQ_HOMREF=0.9996119895237171;N_BI_GENOS=10309;South_Han_AN=1496;South_Han_AC=1;South_Ha
chr6	76087139	.	T	<INS:ME:LINE1>	.	PASS	SVTYPE=INS:ME:LINE1;END=76087154;SVLEN=1124;AN=20618;AC=2;AF=9.700261907071492e-05;N_HET=2;N_HOMALT=0;N_HOMREF=10307;FREQ_HET=0.00019400523814142983;FREQ_HOMALT=0.0;FREQ_HOMREF=0.9998059947618586;N_BI_GENOS=10309;Northwest_Han_AN=1296;Northwest_Han_AC=1;N
chr6	76254113	.	G	<INS:ME:LINE1>	.	PASS	SVTYPE=INS:ME:LINE1;END=76254147;SVLEN=1247;AN=20618;AC=1;AF=4.850130953535746e-05;N_HET=1;N_HOMALT=0;N_HOMREF=10308;FREQ_HET=9.700261907071492e-05;FREQ_HOMALT=0.0;FREQ_HOMREF=0.9999029973809292;N_BI_GENOS=10309;East_Han_AN=6554;East_Han_AC=1;East_Han_AF=
chr6	76274994	.	C	<INS:ME:LINE1>	.	PASS	SVTYPE=INS:ME:LINE1;END=76275011;SVLEN=6004;AN=20618;AC=1;AF=4.850130953535746e-05;N_HET=1;N_HOMALT=0;N_HOMREF=10308;FREQ_HET=9.700261907071492e-05;FREQ_HOMALT=0.0;FREQ_HOMREF=0.9999029973809292;N_BI_GENOS=10309;Mongolian_AN=454;Mongolian_AC=1;Mongolian_A
chr6	76279948	.	A	<INS:ME:LINE1>	.	PASS	SVTYPE=INS:ME:LINE1;END=76279958;SVLEN=1560;AN=20618;AC=4;AF=0.00019400523814142983;N_HET=2;N_HOMALT=1;N_HOMREF=10306;FREQ_HET=0.00019400523814142983;FREQ_HOMALT=9.700261907071492e-05;FREQ_HOMREF=0.9997089921427879;N_BI_GENOS=10309;Northwest_Han_AN=1296;N
chr6	76280204	.	C	<INS:ME:LINE1>	.	PASS	SVTYPE=INS:ME:LINE1;END=76280204;SVLEN=238;AN=20618;AC=1;AF=4.850130953535746e-05;N_HET=1;N_HOMALT=0;N_HOMREF=10308;FREQ_HET=9.700261907071492e-05;FREQ_HOMALT=0.0;FREQ_HOMREF=0.9999029973809292;N_BI_GENOS=10309;East_Han_AN=6554;East_Han_AC=1;East_Han_AF=0
chr6	76280423	.	A	<INS:ME:LINE1>	.	PASS	SVTYPE=INS:ME:LINE1;END=76280461;SVLEN=676;AN=20618;AC=1;AF=4.850130953535746e-05;N_HET=1;N_HOMALT=0;N_HOMREF=10308;FREQ_HET=9.700261907071492e-05;FREQ_HOMALT=0.0;FREQ_HOMREF=0.9999029973809292;N_BI_GENOS=10309;East_Han_AN=6554;East_Han_AC=1;East_Han_AF=0
chr6	76280808	.	T	<INS:ME:LINE1>	.	PASS	SVTYPE=INS:ME:LINE1;END=76280818;SVLEN=1017;AN=20618;AC=1;AF=4.850130953535746e-05;N_HET=1;N_HOMALT=0;N_HOMREF=10308;FREQ_HET=9.700261907071492e-05;FREQ_HOMALT=0.0;FREQ_HOMREF=0.9999029973809292;N_BI_GENOS=10309;East_Han_AN=6554;East_Han_AC=1;East_Han_AF=
chr6	76304109	.	A	<INS:ME:LINE1>	.	PASS	SVTYPE=INS:ME:LINE1;END=76304154;SVLEN=459;AN=20618;AC=1;AF=4.850130953535746e-05;N_HET=1;N_HOMALT=0;N_HOMREF=10308;FREQ_HET=9.700261907071492e-05;FREQ_HOMALT=0.0;FREQ_HOMREF=0.9999029973809292;N_BI_GENOS=10309;East_Han_AN=6554;East_Han_AC=1;East_Han_AF=0
chr6	76512854	.	T	<INS:ME:LINE1>	.	PASS	SVTYPE=INS:ME:LINE1;END=76512868;SVLEN=6011;AN=20618;AC=2;AF=9.700261907071492e-05;N_HET=2;N_HOMALT=0;N_HOMREF=10307;FREQ_HET=0.00019400523814142983;FREQ_HOMALT=0.0;FREQ_HOMREF=0.9998059947618586;N_BI_GENOS=10309;South_Han_AN=1496;South_Han_AC=1;South_Han
chr6	76670210	.	C	<INS:ME:LINE1>	.	PASS	SVTYPE=INS:ME:LINE1;END=76670210;SVLEN=1962;AN=20618;AC=488;AF=0.023668639053254437;N_HET=446;N_HOMALT=21;N_HOMREF=9842;FREQ_HET=0.043263168105538846;FREQ_HOMALT=0.0020370550004850 13;FREQ_HOMREF=0.9546997768939761;N_BI_GENOS=10309;South_Han_AN=1496;South_Han
chr6	76769641	.	A	<INS:ME:LINE1>	.	PASS	SVTYPE=INS:ME:LINE1;END=76769658;SVLEN=1624;AN=20618;AC=2;AF=9.700261907071492e-05;N_HET=2;N_HOMALT=0;N_HOMREF=10307;FREQ_HET=0.00019400523814142983;FREQ_HOMALT=0.0;FREQ_HOMREF=0.9998059947618586;N_BI_GENOS=10309;Manchu_AN=416;Manchu_AC=1;Manchu_AF=0.0024
chr6	76826006	.	C	<INS:ME:LINE1>	.	PASS	SVTYPE=INS:ME:LINE1;END=76826014;SVLEN=1220;AN=20618;AC=1;AF=4.850130953535746e-05;N_HET=1;N_HOMALT=0;N_HOMREF=10308;FREQ_HET=9.700261907071492e-05;FREQ_HOMALT=0.0;FREQ_HOMREF=0.9999029973809292;N_BI_GENOS=10309;East_Han_AN=6554;East_Han_AC=1;East_Han_AF=

chr6	76826204	.	G	<INS:ME:LINE1>	.	PASS	SVTYPE=INS:ME:LINE1;END=76826204;SVLEN=80;AN=20618;AC=1;AF=4.850130953535746e-05;N_HET=1;N_HOMALT=0;N_HOMREF=10308;FREQ_HET=9.700261907071492e-05;FREQ_HOMALT=0.0;FREQ_HOMREF=0.9999029973809292;N_BI_GENOS=10309;East_Han_AN=6554;East_Han_AC=1;East_Han_AF=0.
chr6	76955773	.	A	<INS:ME:LINE1>	.	PASS	SVTYPE=INS:ME:LINE1;END=76955773;SVLEN=612;AN=20618;AC=2;AF=9.700261907071492e-05;N_HET=2;N_HOMALT=0;N_HOMREF=10307;FREQ_HET=0.0001940052381414298З;FREQ_HOMALT=0.0;FREQ_HOMREF=0.9998059947618586;N_BI_GENOS=10309;South_Han_AN=1496;South_Han_AC=1;South_Han_
chr6	76969256	.	G	<INS:ME:LINE1>	.	PASS	SVTYPE=INS:ME:LINE1;END=76969256;SVLEN=440;AN=20618;AC=1;AF=4.850130953535746e-05;N_HET=1;N_HOMALT=0;N_HOMREF=10308;FREQ_HET=9.700261907071492e-05;FREQ_HOMALT=0.0;FREQ_HOMREF=0.9999029973809292;N_BI_GENOS=10309;East_Han_AN=6554;East_Han_AC=1;East_Han_AF=0
chr6	76996031	.	A	<INS:ME:LINE1>	.	PASS	SVTYPE=INS:ME:LINE1;END=76996046;SVLEN=356;AN=20618;AC=1;AF=4.850130953535746e-05;N_HET=1;N_HOMALT=0;N_HOMREF=10308;FREQ_HET=9.700261907071492e-05;FREQ_HOMALT=0.0;FREQ_HOMREF=0.9999029973809292;N_BI_GENOS=10309;Central_Han_AN=2128;Central_Han_AC=1;Central
chr6	77270924	.	A	<INS:ME:LINE1>	.	PASS	SVTYPE=INS:ME:LINE1;END=77270932;SVLEN=779;AN=20618;AC=8888;AF=0.43107963915025704;N_HET=4848;N_HOMALT=2020;N_HOMREF=3441;FREQ_HET=0.47026869725482559;FREQ_HOMALT=0.19594529052284412;FREQ_HOMREF=0.33378601222233;N_BI_GENOS=10309;South_Han_AN=1496;South_Han
chr6	77545293	.	G	<INS:ME:LINE1>	.	PASS	SVTYPE=INS:ME:LINE1;END=77545311;SVLEN=5991;AN=20618;AC=150;AF=0.0072751964303036185;N_HET=142;N_HOMALT=4;N_HOMREF=10163;FREQ_HET=0.013774371908041518;FREQ_HOMALT=0.00038801047628285966;FREQ_HOMREF=0.9858376176156757;N_BI_GENOS=10309;South_Han_AN=1496;Sou
chr6	77771232	.	A	<INS:ME:LINE1>	.	PASS	SVTYPE=INS:ME:LINE1;END=77771275;SVLEN=809;AN=20618;AC=1;AF=4.850130953535746e-05;N_HET=1;N_HOMALT=0;N_HOMREF=10308;FREQ_HET=9.700261907071492e-05;FREQ_HOMALT=0.0;FREQ_HOMREF=0.9999029973809292;N_BI_GENOS=10309;East_Han_AN=6554;East_Han_AC=1;East_Han_AF=0
chr6	77772876	.	G	<INS:ME:LINE1>	.	PASS	SVTYPE=INS:ME:LINE1;END=77772894;SVLEN=1272;AN=20618;AC=1;AF=4.850130953535746e-05;N_HET=1;N_HOMALT=0;N_HOMREF=10308;FREQ_HET=9.700261907071492e-05;FREQ_HOMALT=0.0;FREQ_HOMREF=0.9999029973809292;N_BI_GENOS=10309;North_Han_AN=2272;North_Han_AC=1;North_Han_
chr6	78039674	.	A	<INS:ME:LINE1>	.	PASS	SVTYPE=INS:ME:LINE1;END=78039690;SVLEN=1095;AN=20618;AC=790;AF=0.03831603453293239;N_HET=692;N_HOMALT=49;N_HOMREF=9568;FREQ_HET=0.06712581239693471;FREQ_HOMALT=0.004753128334465031;FREQ_HOMREF=0.9281210592686002;N_BI_GENOS=10309;South_Han_AN=1496;South_Ha
chr6	78048824	.	A	<INS:ME:LINE1>	.	PASS	SVTYPE=INS:ME:LINE1;END=78048824;SVLEN=33;AN=20618;AC=1;AF=4.850130953535746e-05;N_HET=1;N_HOMALT=0;N_HOMREF=10308;FREQ_HET=9.700261907071492e-05;FREQ_HOMALT=0.0;FREQ_HOMREF=0.9999029973809292;N_BI_GENOS=10309;East_Han_AN=6554;East_Han_AC=1;East_Han_AF=0.
chr6	78179401	.	T	<INS:ME:LINE1>	.	PASS	SVTYPE=INS:ME:LINE1;END=78179417;SVLEN=5999;AN=20618;AC=1;AF=4.850130953535746e-05;N_HET=1;N_HOMALT=0;N_HOMREF=10308;FREQ_HET=9.700261907071492e-05;FREQ_HOMALT=0.0;FREQ_HOMREF=0.9999029973809292;N_BI_GENOS=10309;Southeast_Han_AN=1908;Southeast_Han_AC=1;So
chr6	78235894	.	A	<INS:ME:LINE1>	.	PASS	SVTYPE=INS:ME:LINE1;END=78235898;SVLEN=527;AN=20618;AC=1;AF=4.850130953535746e-05;N_HET=1;N_HOMALT=0;N_HOMREF=10308;FREQ_HET=9.700261907071492e-05;FREQ_HOMALT=0.0;FREQ_HOMREF=0.9999029973809292;N_BI_GENOS=10309;Southeast_Han_AN=1908;Southeast_Han_AC=1;Sou
chr6	78406841	.	A	<INS:ME:LINE1>	.	PASS	SVTYPE=INS:ME:LINE1;END=78406847;SVLEN=512;AN=20618;AC=1;AF=4.850130953535746e-05;N_HET=1;N_HOMALT=0;N_HOMREF=10308;FREQ_HET=9.700261907071492e-05;FREQ_HOMALT=0.0;FREQ_HOMREF=0.9999029973809292;N_BI_GENOS=10309;Central_Han_AN=2128;Central_Han_AC=1;Central
chr6	78533744	.	T	<INS:ME:LINE1>	.	PASS	SVTYPE=INS:ME:LINE1;END=78533759;SVLEN=754;AN=20618;AC=1;AF=4.850130953535746e-05;N_HET=1;N_HOMALT=0;N_HOMREF=10308;FREQ_HET=9.700261907071492e-05;FREQ_HOMALT=0.0;FREQ_HOMREF=0.9999029973809292;N_BI_GENOS=10309;Zhuang_AN=444;Zhuang_AC=1;Zhuang_AF=0.002252
chr6	78558197	.	T	<INS:ME:LINE1>	.	PASS	SVTYPE=INS:ME:LINE1;END=78558197;SVLEN=104;AN=20618;AC=1;AF=4.850130953535746e-05;N_HET=1;N_HOMALT=0;N_HOMREF=10308;FREQ_HET=9.700261907071492e-05;FREQ_HOMALT=0.0;FREQ_HOMREF=0.9999029973809292;N_BI_GENOS=10309;East_Han_AN=6554;East_Han_AC=1;East_Han_AF=0
chr6	78812601	.	A	<INS:ME:LINE1>	.	PASS	SVTYPE=INS:ME:LINE1;END=78812617;SVLEN=133;AN=20618;AC=1;AF=4.850130953535746e-05;N_HET=1;N_HOMALT=0;N_HOMREF=10308;FREQ_HET=9.700261907071492e-05;FREQ_HOMALT=0.0;FREQ_HOMREF=0.9999029973809292;N_BI_GENOS=10309;South_Han_AN=1496;South_Han_AC=1;South_Han_A
chr6	79000593	.	C	<INS:ME:LINE1>	.	PASS	SVTYPE=INS:ME:LINE1;END=79000614;SVLEN=5997;AN=20618;AC=11;AF=0.000533514404888932;N_HET=11;N_HOMALT=0;N_HOMREF=10298;FREQ_HET=0.001067028809777864;FREQ_HOMALT=0.0;FREQ_HOMREF=0.9989329711902222;N_BI_GENOS=10309;North_Han_AN=2272;North_Han_AC=2;North_Han_
chr6	79169968	.	G	<INS:ME:LINE1>	.	PASS	SVTYPE=INS:ME:LINE1;END=79169985;SVLEN=914;AN=20618;AC=1;AF=4.850130953535746e-05;N_HET=1;N_HOMALT=0;N_HOMREF=10308;FREQ_HET=9.700261907071492e-05;FREQ_HOMALT=0.0;FREQ_HOMREF=0.9999029973809292;N_BI_GENOS=10309;Manchu_AN=416;Manchu_AC=1;Manchu_AF=0.002403
chr6	79866376	.	A	<INS:ME:LINE1>	.	PASS	SVTYPE=INS:ME:LINE1;END=79866390;SVLEN=433;AN=20618;AC=1;AF=4.850130953535746e-05;N_HET=1;N_HOMALT=0;N_HOMREF=10308;FREQ_HET=9.700261907071492e-05;FREQ_HOMALT=0.0;FREQ_HOMREF=0.9999029973809292;N_BI_GENOS=10309;Southeast_Han_AN=1908;Southeast_Han_AC=1;Sou
chr6	79868360	.	T	<INS:ME:LINE1>	.	PASS	SVTYPE=INS:ME:LINE1;END=79868372;SVLEN=938;AN=20618;AC=1;AF=4.850130953535746e-05;N_HET=1;N_HOMALT=0;N_HOMREF=10308;FREQ_HET=9.700261907071492e-05;FREQ_HOMALT=0.0;FREQ_HOMREF=0.9999029973809292;N_BI_GENOS=10309;Southeast_Han_AN=1908;Southeast_Han_AC=1;Sou
chr6	79906435	.	A	<INS:ME:LINE1>	.	PASS	SVTYPE=INS:ME:LINE1;END=79906451;SVLEN=2037;AN=20618;AC=5;AF=0.00024250654767678728;N_HET=5;N_HOMALT=0;N_HOMREF=10304;FREQ_HET=0.00048501309535357455;FREQ_HOMALT=0.0;FREQ_HOMREF=0.9995149869046465;N_BI_GENOS=10309;East_Han_AN=6554;East_Han_AC=1;East_Han_A
chr6	80156562	.	T	<INS:ME:LINE1>	.	PASS	SVTYPE=INS:ME:LINE1;END=80156578;SVLEN=6006;AN=20618;AC=2;AF=9.700261907071492e-05;N_HET=2;N_HOMALT=0;N_HOMREF=10307;FREQ_HET=0.00019400523814142983;FREQ_HOMALT=0.0;FREQ_HOMREF=0.9998059947618586;N_BI_GENOS=10309;North_Han_AN=2272;North_Han_AC=1;North_Han
chr6	80380762	.	C	<INS:ME:LINE1>	.	PASS	SVTYPE=INS:ME:LINE1;END=80380762;SVLEN=562;AN=20618;AC=1;AF=4.850130953535746e-05;N_HET=1;N_HOMALT=0;N_HOMREF=10308;FREQ_HET=9.700261907071492e-05;FREQ_HOMALT=0.0;FREQ_HOMREF=0.9999029973809292;N_BI_GENOS=10309;East_Han_AN=6554;East_Han_AC=1;East_Han_AF=0
chr6	80638562	.	A	<INS:ME:LINE1>	.	PASS	SVTYPE=INS:ME:LINE1;END=80638577;SVLEN=829;AN=20618;AC=1;AF=4.850130953535746e-05;N_HET=1;N_HOMALT=0;N_HOMREF=10308;FREQ_HET=9.700261907071492e-05;FREQ_HOMALT=0.0;FREQ_HOMREF=0.9999029973809292;N_BI_GENOS=10309;North_Han_AN=2272;North_Han_AC=1;North_Han_A
chr6	80810756	.	A	<INS:ME:LINE1>	.	PASS	SVTYPE=INS:ME:LINE1;END=80810773;SVLEN=5998;AN=20618;AC=1;AF=4.850130953535746e-05;N_HET=1;N_HOMALT=0;N_HOMREF=10308;FREQ_HET=9.700261907071492e-05;FREQ_HOMALT=0.0;FREQ_HOMREF=0.9999029973809292;N_BI_GENOS=10309;Central_Han_AN=2128;Central_Han_AC=1;Centra
chr6	80984307	.	G	<INS:ME:LINE1>	.	PASS	SVTYPE=INS:ME:LINE1;END=80984307;SVLEN=1367;AN=20618;AC=2;AF=9.700261907071492e-05;N_HET=2;N_HOMALT=0;N_HOMREF=10307;FREQ_HET=0.00019400523814142983;FREQ_HOMALT=0.0;FREQ_HOMREF=0.9998059947618586;N_BI_GENOS=10309;North_Han_AN=2272;North_Han_AC=1;North_Han
chr6	81100073	.	C	<INS:ME:LINE1>	.	PASS	SVTYPE=INS:ME:LINE1;END=81100088;SVLEN=2292;AN=20618;AC=1;AF=4.850130953535746e-05;N_HET=1;N_HOMALT=0;N_HOMREF=10308;FREQ_HET=9.700261907071492e-05;FREQ_HOMALT=0.0;FREQ_HOMREF=0.9999029973809292;N_BI_GENOS=10309;Lingnan_Han_AN=1888;Lingnan_Han_AC=1;Lingna
chr6	81142625	.	A	<INS:ME:LINE1>	.	PASS	SVTYPE=INS:ME:LINE1;END=81142665;SVLEN=1744;AN=20618;AC=1;AF=4.850130953535746e-05;N_HET=1;N_HOMALT=0;N_HOMREF=10308;FREQ_HET=9.700261907071492e-05;FREQ_HOMALT=0.0;FREQ_HOMREF=0.9999029973809292;N_BI_GENOS=10309;East_Han_AN=6554;East_Han_AC=1;East_Han_AF=
chr6	81295870	.	A	<INS:ME:LINE1>	.	PASS	SVTYPE=INS:ME:LINE1;END=81295883;SVLEN=1602;AN=20618;AC=2;AF=9.700261907071492e-05;N_HET=2;N_HOMALT=0;N_HOMREF=10307;FREQ_HET=0.00019400523814142983;FREQ_HOMALT=0.0;FREQ_HOMREF=0.9998059947618586;N_BI_GENOS=10309;East_Han_AN=6554;East_Han_AC=1;East_Han_AF
chr6	81633714	.	T	<INS:ME:LINE1>	.	PASS	SVTYPE=INS:ME:LINE1;END=81633730;SVLEN=5998;AN=20618;AC=1;AF=4.850130953535746e-05;N_HET=1;N_HOMALT=0;N_HOMREF=10308;FREQ_HET=9.700261907071492e-05;FREQ_HOMALT=0.0;FREQ_HOMREF=0.9999029973809292;N_BI_GENOS=10309;South_Han_AN=1496;South_Han_AC=1;South_Han_
chr6	81653271	.	T	<INS:ME:LINE1>	.	PASS	SVTYPE=INS:ME:LINE1;END=81653287;SVLEN=351;AN=20618;AC=1;AF=4.850130953535746e-05;N_HET=1;N_HOMALT=0;N_HOMREF=10308;FREQ_HET=9.700261907071492e-05;FREQ_HOMALT=0.0;FREQ_HOMREF=0.9999029973809292;N_BI_GENOS=10309;East_Han_AN=6554;East_Han_AC=1;East_Han_AF=0
chr6	81695325	.	T	<INS:ME:LINE1>	.	PASS	SVTYPE=INS:ME:LINE1;END=81695326;SVLEN=990;AN=20618;AC=8;AF=0.00038801047628285966;N_HET=6;N_HOMALT=1;N_HOMREF=10302;FREQ_HET=0.0005820157144242894;FREQ_HOMALT=9.700261907071492e-05;FREQ_HOMREF=0.9993209816666505;N_BI_GENOS=10309;North_Han_AN=2272;North_Ha
chr6	81766893	.	A	<INS:ME:LINE1>	.	PASS	SVTYPE=INS:ME:LINE1;END=81766908;SVLEN=887;AN=20618;AC=2;AF=9.700261907071492e-05;N_HET=2;N_HOMALT=0;N_HOMREF=10307;FREQ_HET=0.00019400523814142983;FREQ_HOMALT=0.0;FREQ_HOMREF=0.9998059947618586;N_BI_GENOS=10309;East_Han_AN=6554;East_Han_AC=2;East_Han_AF=
chr6	81989306	.	A	<INS:ME:LINE1>	.	PASS	SVTYPE=INS:ME:LINE1;END=81989319;SVLEN=1010;AN=20618;AC=1;AF=4.850130953535746e-05;N_HET=1;N_HOMALT=0;N_HOMREF=10308;FREQ_HET=9.700261907071492e-05;FREQ_HOMALT=0.0;FREQ_HOMREF=0.9999029973809292;N_BI_GENOS=10309;Mongolian_AN=454;Mongolian_AC=1;Mongolian_A
chr6	81993617	.	C	<INS:ME:LINE1>	.	PASS	SVTYPE=INS:ME:LINE1;END=81993618;SVLEN=903;AN=20618;AC=1;AF=4.850130953535746e-05;N_HET=1;N_HOMALT=0;N_HOMREF=10308;FREQ_HET=9.700261907071492e-05;FREQ_HOMALT=0.0;FREQ_HOMREF=0.9999029973809292;N_BI_GENOS=10309;Central_Han_AN=2128;Central_Han_AC=1;Central
chr6	82753200	.	A	<INS:ME:LINE1>	.	PASS	SVTYPE=INS:ME:LINE1;END=82753216;SVLEN=954;AN=20618;AC=1;AF=4.850130953535746e-05;N_HET=1;N_HOMALT=0;N_HOMREF=10308;FREQ_HET=9.700261907071492e-05;FREQ_HOMALT=0.0;FREQ_HOMREF=0.9999029973809292;N_BI_GENOS=10309;Central_Han_AN=2128;Central_Han_AC=1;Central
chr6	82881596	.	A	<INS:ME:LINE1>	.	PASS	SVTYPE=INS:ME:LINE1;END=82881607;SVLEN=5998;AN=20618;AC=2;AF=9.700261907071492e-05;N_HET=2;N_HOMALT=0;N_HOMREF=10307;FREQ_HET=0.00019400523814142983;FREQ_HOMALT=0.0;FREQ_HOMREF=0.9998059947618586;N_BI_GENOS=10309;Lingnan_Han_AN=1888;Lingnan_Han_AC=1;Lingn
chr6	83035564	.	A	<INS:ME:LINE1>	.	PASS	SVTYPE=INS:ME:LINE1;END=83035579;SVLEN=5998;AN=20618;AC=183;AF=0.008875739644970414;N_HET=169;N_HOMALT=7;N_HOMREF=10133;FREQ_HET=0.01639344262295082;FREQ_HOMALT=0.0006790183334950044;FREQ_HOMREF=0.9829275390435541;N_BI_GENOS=10309;South_Han_AN=1496;South_
chr6	83169028	.	A	<INS:ME:LINE1>	.	PASS	SVTYPE=INS:ME:LINE1;END=83169044;SVLEN=1338;AN=20618;AC=1;AF=4.850130953535746e-05;N_HET=1;N_HOMALT=0;N_HOMREF=10308;FREQ_HET=9.700261907071492e-05;FREQ_HOMALT=0.0;FREQ_HOMREF=0.9999029973809292;N_BI_GENOS=10309;East_Han_AN=6554;East_Han_AC=1;East_Han_AF=
chr6	83413027	.	A	<INS:ME:LINE1>	.	PASS	SVTYPE=INS:ME:LINE1;END=83413043;SVLEN=425;AN=20618;AC=1;AF=4.850130953535746e-05;N_HET=1;N_HOMALT=0;N_HOMREF=10308;FREQ_HET=9.700261907071492e-05;FREQ_HOMALT=0.0;FREQ_HOMREF=0.9999029973809292;N_BI_GENOS=10309;Southeast_Han_AN=1908;Southeast_Han_AC=1;Sou
chr6	83416380	.	A	<INS:ME:LINE1>	.	PASS	SVTYPE=INS:ME:LINE1;END=83416397;SVLEN=1641;AN=20618;AC=110;AF=0.005335144048889З2;N_HET=102;N_HOMALT=4;N_HOMREF=10203;FREQ_HET=0.009894267145212922;FREQ_HOMALT=0.00038801047628285966;FREQ_HOMREF=0.9897177223785042;N_BI_GENOS=10309;South_Han_AN=1496;South
chr6	84094024	.	C	<INS:ME:LINE1>	.	PASS	SVTYPE=INS:ME:LINE1;END=84094040;SVLEN=6000;AN=20618;AC=3;AF=0.00014550392860607236;N_HET=3;N_HOMALT=0;N_HOMREF=10306;FREQ_HET=0.0002910078572121447;FREQ_HOMALT=0.0;FREQ_HOMREF=0.9997089921427879;N_BI_GENOS=10309;Central_Han_AN=2128;Central_Han_AC=3;Centr
chr6	84312082	.	T	<INS:ME:LINE1>	.	PASS	SVTYPE=INS:ME:LINE1;END=84312093;SVLEN=2047;AN=20618;AC=1;AF=4.850130953535746e-05;N_HET=1;N_HOMALT=0;N_HOMREF=10308;FREQ_HET=9.700261907071492e-05;FREQ_HOMALT=0.0;FREQ_HOMREF=0.9999029973809292;N_BI_GENOS=10309;North_Han_AN=2272;North_Han_AC=1;North_Han_
chr6	84359506	.	A	<INS:ME:LINE1>	.	PASS	SVTYPE=INS:ME:LINE1;END=84359521;SVLEN=5997;AN=20618;AC=1;AF=4.850130953535746e-05;N_HET=1;N_HOMALT=0;N_HOMREF=10308;FREQ_HET=9.700261907071492e-05;FREQ_HOMALT=0.0;FREQ_HOMREF=0.9999029973809292;N_BI_GENOS=10309;Tibetan_AN=414;Tibetan_AC=1;Tibetan_AF=0.00
chr6	84375754	.	T	<INS:ME:LINE1>	.	PASS	SVTYPE=INS:ME:LINE1;END=84375754;SVLEN=322;AN=20618;AC=1;AF=4.850130953535746e-05;N_HET=1;N_HOMALT=0;N_HOMREF=10308;FREQ_HET=9.700261907071492e-05;FREQ_HOMALT=0.0;FREQ_HOMREF=0.9999029973809292;N_BI_GENOS=10309;East_Han_AN=6554;East_Han_AC=1;East_Han_AF=0
chr6	84392386	.	A	<INS:ME:LINE1>	.	PASS	SVTYPE=INS:ME:LINE1;END=84392402;SVLEN=743;AN=20618;AC=13;AF=0.0006305170239596469;N_HET=13;N_HOMALT=0;N_HOMREF=10296;FREQ_HET=0.0012610340479192938;FREQ_HOMALT=0.0;FREQ_HOMREF=0.9987389659520807;N_BI_GENOS=10309;Northwest_Han_AN=1296;Northwest_Han_AC=2;N
chr6	84450436	.	A	<INS:ME:LINE1>	.	PASS	SVTYPE=INS:ME:LINE1;END=84450450;SVLEN=5999;AN=20618;AC=1;AF=4.850130953535746e-05;N_HET=1;N_HOMALT=0;N_HOMREF=10308;FREQ_HET=9.700261907071492e-05;FREQ_HOMALT=0.0;FREQ_HOMREF=0.9999029973809292;N_BI_GENOS=10309;Mongolian_AN=454;Mongolian_AC=1;Mongolian_A
chr6	84495475	.	A	<INS:ME:LINE1>	.	PASS	SVTYPE=INS:ME:LINE1;END=84495492;SVLEN=197;AN=20618;AC=11;AF=0.000533514404888932;N_HET=11;N_HOMALT=0;N_HOMREF=10298;FREQ_HET=0.001067028809777864;FREQ_HOMALT=0.0;FREQ_HOMREF=0.9989329711902222;N_BI_GENOS=10309;Lingnan_Han_AN=1888;Lingnan_Han_AC=1;Lingnan
chr6	84558480	.	T	<INS:ME:LINE1>	.	PASS	SVTYPE=INS:ME:LINE1;END=84558496;SVLEN=2694;AN=20618;AC=12;AF=0.0005820157144242894;N_HET=12;N_HOMALT=0;N_HOMREF=10297;FREQ_HET=0.0011640314288485789;FREQ_HOMALT=0.0;FREQ_HOMREF=0.9988359685711514;N_BI_GENOS=10309;Lingnan_Han_AN=1888;Lingnan_Han_AC=1;Ling
chr6	84765114	.	A	<INS:ME:LINE1>	.	PASS	SVTYPE=INS:ME:LINE1;END=84765132;SVLEN=451;AN=20618;AC=1;AF=4.850130953535746e-05;N_HET=1;N_HOMALT=0;N_HOMREF=10308;FREQ_HET=9.700261907071492e-05;FREQ_HOMALT=0.0;FREQ_HOMREF=0.9999029973809292;N_BI_GENOS=10309;Southeast_Han_AN=1908;Southeast_Han_AC=1;Sou
chr6	84781947	.	A	<INS:ME:LINE1>	.	PASS	SVTYPE=INS:ME:LINE1;END=84781965;SVLEN=993;AN=20618;AC=4;AF=0.00019400523814142983;N_HET=4;N_HOMALT=0;N_HOMREF=10305;FREQ_HET=0.00038801047628285966;FREQ_HOMALT=0.0;FREQ_HOMREF=0.9996119895237171;N_BI_GENOS=10309;Northwest_Han_AN=1296;Northwest_Han_AC=1;N
chr6	84876026	.	A	<INS:ME:LINE1>	.	PASS	SVTYPE=INS:ME:LINE1;END=84876041;SVLEN=6000;AN=20618;AC=8;AF=0.00038801047628285966;N_HET=8;N_HOMALT=0;N_HOMREF=10301;FREQ_HET=0.0007760209525657193;FREQ_HOMALT=0.0;FREQ_HOMREF=0.9992239790474343;N_BI_GENOS=10309;Lingnan_Han_AN=1888;Lingnan_Han_AC=1;Lingn
chr6	85024029	.	A	<INS:ME:LINE1>	.	PASS	SVTYPE=INS:ME:LINE1;END=85024041;SVLEN=258;AN=20618;AC=2;AF=9.700261907071492e-05;N_HET=2;N_HOMALT=0;N_HOMREF=10307;FREQ_HET=0.00019400523814142983;FREQ_HOMALT=0.0;FREQ_HOMREF=0.9998059947618586;N_BI_GENOS=10309;Northwest_Han_AN=1296;Northwest_Han_AC=1;No
chr6	85221684	.	C	<INS:ME:LINE1>	.	PASS	SVTYPE=INS:ME:LINE1;END=85221702;SVLEN=5988;AN=20618;AC=9;AF=0.0004365117858182171;N_HET=1;N_HOMALT=4;N_HOMREF=10304;FREQ_HET=9.700261907071492e-05;FREQ_HOMALT=0.00038801047628285966;FREQ_HOMREF=0.9995149869046465;N_BI_GENOS=10309;East_Han_AN=6554;East_Ha
chr6	85238009	.	C	<INS:ME:LINE1>	.	PASS	SVTYPE=INS:ME:LINE1;END=85238026;SVLEN=2145;AN=20618;AC=5960;AF=0.2890678048307304;N_HET=4122;N_HOMALT=919;N_HOMREF=5268;FREQ_HET=0.39984479580948684;FREQ_HOMALT=0.089145406925987;FREQ_HOMREF=0.5110097972645261;N_BI_GENOS=10309;South_Han_AN=1496;South_Han
chr6	85431567	.	G	<INS:ME:LINE1>	.	PASS	SVTYPE=INS:ME:LINE1;END=85431582;SVLEN=6006;AN=20618;AC=1;AF=4.850130953535746e-05;N_HET=1;N_HOMALT=0;N_HOMREF=10308;FREQ_HET=9.700261907071492e-05;FREQ_HOMALT=0.0;FREQ_HOMREF=0.9999029973809292;N_BI_GENOS=10309;Yi_AN=422;Yi_AC=1;Yi_AF=0.002369668246445 49
chr6	85492421	.	T	<INS:ME:LINE1>	.	PASS	SVTYPE=INS:ME:LINE1;END=85492421;SVLEN=624;AN=20618;AC=1;AF=4.850130953535746e-05;N_HET=1;N_HOMALT=0;N_HOMREF=10308;FREQ_HET=9.700261907071492e-05;FREQ_HOMALT=0.0;FREQ_HOMREF=0.9999029973809292;N_BI_GENOS=10309;North_Han_AN=2272;North_Han_AC=1;North_Han_A
chr6	86077749	.	A	<INS:ME:LINE1>	.	PASS	SVTYPE=INS:ME:LINE1;END=86077765;SVLEN=541;AN=20618;AC=3;AF=0.00014550392860607236;N_HET=3;N_HOMALT=0;N_HOMREF=10306;FREQ_HET=0.0002910078572121447;FREQ_HOMALT=0.0;FREQ_HOMREF=0.9997089921427879;N_BI_GENOS=10309;South_Han_AN=1496;South_Han_AC=1;South_Han_
chr6	86448417	.	A	<INS:ME:LINE1>	.	PASS	SVTYPE=INS:ME:LINE1;END=86448419;SVLEN=142;AN=20618;AC=1;AF=4.850130953535746e-05;N_HET=1;N_HOMALT=0;N_HOMREF=10308;FREQ_HET=9.700261907071492e-05;FREQ_HOMALT=0.0;FREQ_HOMREF=0.9999029973809292;N_BI_GENOS=10309;North_Han_AN=2272;North_Han_AC=1;North_Han_A
chr6	86556768	.	G	<INS:ME:LINE1>	.	PASS	SVTYPE=INS:ME:LINE1;END=86556786;SVLEN=599;AN=20618;AC=1;AF=4.850130953535746e-05;N_HET=1;N_HOMALT=0;N_HOMREF=10308;FREQ_HET=9.700261907071492e-05;FREQ_HOMALT=0.0;FREQ_HOMREF=0.9999029973809292;N_BI_GENOS=10309;South_Han_AN=1496;South_Han_AC=1;South_Han_A
chr6	86576447	.	A	<INS:ME:LINE1>	.	PASS	SVTYPE=INS:ME:LINE1;END=86576462;SVLEN=217;AN=20618;AC=2;AF=9.700261907071492e-05;N_HET=2;N_HOMALT=0;N_HOMREF=10307;FREQ_HET=0.00019400523814142983;FREQ_HOMALT=0.0;FREQ_HOMREF=0.9998059947618586;N_BI_GENOS=10309;East_Han_AN=6554;East_Han_AC=2;East_Han_AF=
chr6	86619011	.	A	<INS:ME:LINE1>	.	PASS	SVTYPE=INS:ME:LINE1;END=86619011;SVLEN=320;AN=20618;AC=8;AF=0.00038801047628285966;N_HET=8;N_HOMALT=0;N_HOMREF=10301;FREQ_HET=0.0007760209525657193;FREQ_HOMALT=0.0;FREQ_HOMREF=0.9992239790474343;N_BI_GENOS=10309;Northwest_Han_AN=1296;Northwest_Han_AC=1;No
chr6	86644082	.	G	<INS:ME:LINE1>	.	PASS	SVTYPE=INS:ME:LINE1;END=86644082;SVLEN=0;AN=20618;AC=1;AF=4.850130953535746e-05;N_HET=1;N_HOMALT=0;N_HOMREF=10308;FREQ_HET=9.700261907071492e-05;FREQ_HOMALT=0.0;FREQ_HOMREF=0.9999029973809292;N_BI_GENOS=10309;Central_Han_AN=2128;Central_Han_AC=1;Central_H
chr6	86961043	.	A	<INS:ME:LINE1>	.	PASS	SVTYPE=INS:ME:LINE1;END=86961060;SVLEN=981;AN=20618;AC=1;AF=4.850130953535746e-05;N_HET=1;N_HOMALT=0;N_HOMREF=10308;FREQ_HET=9.700261907071492e-05;FREQ_HOMALT=0.0;FREQ_HOMREF=0.9999029973809292;N_BI_GENOS=10309;East_Han_AN=6554;East_Han_AC=1;East_Han_AF=0
chr6	87020418	.	G	<INS:ME:LINE1>	.	PASS	SVTYPE=INS:ME:LINE1;END=87020433;SVLEN=6007;AN=20618;AC=1;AF=4.850130953535746e-05;N_HET=1;N_HOMALT=0;N_HOMREF=10308;FREQ_HET=9.700261907071492e-05;FREQ_HOMALT=0.0;FREQ_HOMREF=0.9999029973809292;N_BI_GENOS=10309;Manchu_AN=416;Manchu_AC=1;Manchu_AF=0.00240
chr6	87032388	.	A	<INS:ME:LINE1>	.	PASS	SVTYPE=INS:ME:LINE1;END=87032390;SVLEN=257;AN=20618;AC=1;AF=4.850130953535746e-05;N_HET=1;N_HOMALT=0;N_HOMREF=10308;FREQ_HET=9.700261907071492e-05;FREQ_HOMALT=0.0;FREQ_HOMREF=0.9999029973809292;N_BI_GENOS=10309;South_Han_AN=1496;South_Han_AC=1;South_Han_A
chr6	87041114	.	C	<INS:ME:LINE1>	.	PASS	SVTYPE=INS:ME:LINE1;END=87041125;SVLEN=1489;AN=20618;AC=1;AF=4.850130953535746e-05;N_HET=1;N_HOMALT=0;N_HOMREF=10308;FREQ_HET=9.700261907071492e-05;FREQ_HOMALT=0.0;FREQ_HOMREF=0.9999029973809292;N_BI_GENOS=10309;Lingnan_Han_AN=1888;Lingnan_Han_AC=1;Lingna
chr6	87331716	.	A	<INS:ME:LINE1>	.	PASS	SVTYPE=INS:ME:LINE1;END=87331731;SVLEN=6015;AN=20618;AC=1;AF=4.850130953535746e-05;N_HET=1;N_HOMALT=0;N_HOMREF=10308;FREQ_HET=9.700261907071492e-05;FREQ_HOMALT=0.0;FREQ_HOMREF=0.9999029973809292;N_BI_GENOS=10309;Central_Han_AN=2128;Central_Han_AC=1;Centra
chr6	88505323	.	T	<INS:ME:LINE1>	.	PASS	SVTYPE=INS:ME:LINE1;END=88505338;SVLEN=3430;AN=20618;AC=1;AF=4.850130953535746e-05;N_HET=1;N_HOMALT=0;N_HOMREF=10308;FREQ_HET=9.700261907071492e-05;FREQ_HOMALT=0.0;FREQ_HOMREF=0.9999029973809292;N_BI_GENOS=10309;Southeast_Han_AN=1908;Southeast_Han_AC=1;So
chr6	88720960	.	A	<INS:ME:LINE1>	.	PASS	SVTYPE=INS:ME:LINE1;END=88720975;SVLEN=6081;AN=20618;AC=1;AF=4.850130953535746e-05;N_HET=1;N_HOMALT=0;N_HOMREF=10308;FREQ_HET=9.700261907071492e-05;FREQ_HOMALT=0.0;FREQ_HOMREF=0.9999029973809292;N_BI_GENOS=10309;East_Han_AN=6554;East_Han_AC=1;East_Han_AF=
chr6	88917430	.	C	<INS:ME:LINE1>	.	PASS	SVTYPE=INS:ME:LINE1;END=88917430;SVLEN=6000;AN=20618;AC=1;AF=4.850130953535746e-05;N_HET=1;N_HOMALT=0;N_HOMREF=10308;FREQ_HET=9.700261907071492e-05;FREQ_HOMALT=0.0;FREQ_HOMREF=0.9999029973809292;N_BI_GENOS=10309;South_Han_AN=1496;South_Han_AC=1;South_Han_
chr6	89268544	.	A	<INS:ME:LINE1>	.	PASS	SVTYPE=INS:ME:LINE1;END=89268562;SVLEN=1364;AN=20618;AC=1;AF=4.850130953535746e-05;N_HET=1;N_HOMALT=0;N_HOMREF=10308;FREQ_HET=9.700261907071492e-05;FREQ_HOMALT=0.0;FREQ_HOMREF=0.9999029973809292;N_BI_GENOS=10309;Lingnan_Han_AN=1888;Lingnan_Han_AC=1;Lingna
chr6	89468946	.	T	<INS:ME:LINE1>	.	PASS	SVTYPE=INS:ME:LINE1;END=89468946;SVLEN=5969;AN=20618;AC=1;AF=4.850130953535746e-05;N_HET=1;N_HOMALT=0;N_HOMREF=10308;FREQ_HET=9.700261907071492e-05;FREQ_HOMALT=0.0;FREQ_HOMREF=0.9999029973809292;N_BI_GENOS=10309;North_Han_AN=2272;North_Han_AC=1;North_Han_
chr6	90010888	.	A	<INS:ME:LINE1>	.	PASS	SVTYPE=INS:ME:LINE1;END=90010905;SVLEN=6008;AN=20618;AC=4;AF=0.00019400523814142983;N_HET=4;N_HOMALT=0;N_HOMREF=10305;FREQ_HET=0.00038801047628285966;FREQ_HOMALT=0.0;FREQ_HOMREF=0.9996119895237171;N_BI_GENOS=10309;Northwest_Han_AN=1296;Northwest_Han_AC=1;
chr6	90103656	.	T	<INS:ME:LINE1>	.	PASS	SVTYPE=INS:ME:LINE1;END=90103673;SVLEN=162;AN=20618;AC=1;AF=4.850130953535746e-05;N_HET=1;N_HOMALT=0;N_HOMREF=10308;FREQ_HET=9.700261907071492e-05;FREQ_HOMALT=0.0;FREQ_HOMREF=0.9999029973809292;N_BI_GENOS=10309;North_Han_AN=2272;North_Han_AC=1;North_Han_A
chr6	90124956	.	A	<INS:ME:LINE1>	.	PASS	SVTYPE=INS:ME:LINE1;END=90124971;SVLEN=6002;AN=20618;AC=1;AF=4.850130953535746e-05;N_HET=1;N_HOMALT=0;N_HOMREF=10308;FREQ_HET=9.700261907071492e-05;FREQ_HOMALT=0.0;FREQ_HOMREF=0.9999029973809292;N_BI_GENOS=10309;Central_Han_AN=2128;Central_Han_AC=1;Centra
chr6	90294620	.	A	<INS:ME:LINE1>	.	PASS	SVTYPE=INS:ME:LINE1;END=90294636;SVLEN=872;AN=20618;AC=1;AF=4.850130953535746e-05;N_HET=1;N_HOMALT=0;N_HOMREF=10308;FREQ_HET=9.700261907071492e-05;FREQ_HOMALT=0.0;FREQ_HOMREF=0.9999029973809292;N_BI_GENOS=10309;North_Han_AN=2272;North_Han_AC=1;North_Han_A
chr6	90303468	.	A	<INS:ME:LINE1>	.	PASS	SVTYPE=INS:ME:LINE1;END=90303485;SVLEN=6000;AN=20618;AC=2;AF=9.700261907071492e-05;N_HET=0;N_HOMALT=1;N_HOMREF=10308;FREQ_HET=0.0;FREQ_HOMALT=9.700261907071492e-05;FREQ_HOMREF=0.9999029973809292;N_BI_GENOS=10309;South_Han_AN=1496;South_Han_AC=2;South_Han_
chr6	90494372	.	A	<INS:ME:LINE1>	.	PASS	SVTYPE=INS:ME:LINE1;END=90494389;SVLEN=573;AN=20618;AC=6;AF=0.0002910078572121447;N_HET=6;N_HOMALT=0;N_HOMREF=10303;FREQ_HET=0.0005820157144242894;FREQ_HOMALT=0.0;FREQ_HOMREF=0.9994179842855757;N_BI_GENOS=10309;South_Han_AN=1496;South_Han_AC=1;South_Han_A
chr6	90565440	.	G	<INS:ME:LINE1>	.	PASS	SVTYPE=INS:ME:LINE1;END=90565454;SVLEN=6019;AN=20618;AC=1;AF=4.850130953535746e-05;N_HET=1;N_HOMALT=0;N_HOMREF=10308;FREQ_HET=9.700261907071492e-05;FREQ_HOMALT=0.0;FREQ_HOMREF=0.9999029973809292;N_BI_GENOS=10309;Southeast_Han_AN=1908;Southeast_Han_AC=1;So
chr6	90927085	.	A	<INS:ME:LINE1>	.	PASS	SVTYPE=INS:ME:LINE1;END=90927098;SVLEN=155;AN=20618;AC=94;AF=0.004559123096323601;N_HET=92;N_HOMALT=1;N_HOMREF=10216;FREQ_HET=0.008924240954505771;FREQ_HOMALT=9.700261907071492e-05;FREQ_HOMREF=0.9909787564264235;N_BI_GENOS=10309;South_Han_AN=1496;South_Ha
chr6	90959789	.	T	<INS:ME:LINE1>	.	PASS	SVTYPE=INS:ME:LINE1;END=90959803;SVLEN=969;AN=20618;AC=7131;AF=0.345862838296634;N_HET=3231;N_HOMALT=1950;N_HOMREF=5128;FREQ_HET=0.31341546221747985;FREQ_HOMALT=0.18915510718789408;FREQ_HOMREF=0.4974294305946261;N_BI_GENOS=10309;South_Han_AN=1496;South_Ha
chr6	91038507	.	G	<INS:ME:LINE1>	.	PASS	SVTYPE=INS:ME:LINE1;END=91038521;SVLEN=6034;AN=20618;AC=12;AF=0.0005820157144242894;N_HET=10;N_HOMALT=1;N_HOMREF=10298;FREQ_HET=0.0009700261907071491;FREQ_HOMALT=9.700261907071492e-05;FREQ_HOMREF=0.9989329711902222;N_BI_GENOS=10309;South_Han_AN=1496;South
chr6	91222506	.	G	<INS:ME:LINE1>	.	PASS	SVTYPE=INS:ME:LINE1;END=91222525;SVLEN=389;AN=20618;AC=2;AF=9.700261907071492e-05;N_HET=2;N_HOMALT=0;N_HOMREF=10307;FREQ_HET=0.00019400523814142983;FREQ_HOMALT=0.0;FREQ_HOMREF=0.9998059947618586;N_BI_GENOS=10309;East_Han_AN=6554;East_Han_AC=2;East_Han_AF=
chr6	91247506	.	A	<INS:ME:LINE1>	.	PASS	SVTYPE=INS:ME:LINE1;END=91247514;SVLEN=798;AN=20618;AC=1;AF=4.850130953535746e-05;N_HET=1;N_HOMALT=0;N_HOMREF=10308;FREQ_HET=9.700261907071492e-05;FREQ_HOMALT=0.0;FREQ_HOMREF=0.9999029973809292;N_BI_GENOS=10309;North_Han_AN=2272;North_Han_AC=1;North_Han_A
chr6	91488604	.	A	<INS:ME:LINE1>	.	PASS	SVTYPE=INS:ME:LINE1;END=91488619;SVLEN=1610;AN=20618;AC=1;AF=4.850130953535746e-05;N_HET=1;N_HOMALT=0;N_HOMREF=10308;FREQ_HET=9.700261907071492e-05;FREQ_HOMALT=0.0;FREQ_HOMREF=0.9999029973809292;N_BI_GENOS=10309;Lingnan_Han_AN=1888;Lingnan_Han_AC=1;Lingna
chr6	91562940	.	T	<INS:ME:LINE1>	.	PASS	SVTYPE=INS:ME:LINE1;END=91562957;SVLEN=1093;AN=20618;AC=97;AF=0.004704627024929673;N_HET=75;N_HOMALT=11;N_HOMREF=10223;FREQ_HET=0.007275196430303618 5;FREQ_HOMALT=0.001067028809777864;FREQ_HOMREF=0.9916577747599186;N_BI_GENOS=10309;South_Han_AN=1496;South_
chr6	91626403	.	T	<INS:ME:LINE1>	.	PASS	SVTYPE=INS:ME:LINE1;END=91626404;SVLEN=3347;AN=20618;AC=1;AF=4.850130953535746e-05;N_HET=1;N_HOMALT=0;N_HOMREF=10308;FREQ_HET=9.700261907071492e-05;FREQ_HOMALT=0.0;FREQ_HOMREF=0.9999029973809292;N_BI_GENOS=10309;North_Han_AN=2272;North_Han_AC=1;North_Han_
chr6	91720862	.	A	<INS:ME:LINE1>	.	PASS	SVTYPE=INS:ME:LINE1;END=91720877;SVLEN=960;AN=20618;AC=2;AF=9.700261907071492e-05;N_HET=2;N_HOMALT=0;N_HOMREF=10307;FREQ_HET=0.00019400523814142983;FREQ_HOMALT=0.0;FREQ_HOMREF=0.9998059947618586;N_BI_GENOS=10309;North_Han_AN=2272;North_Han_AC=1;North_Han_
chr6	91763576	.	G	<INS:ME:LINE1>	.	PASS	SVTYPE=INS:ME:LINE1;END=91763586;SVLEN=174;AN=20618;AC=6;AF=0.0002910078572121447;N_HET=6;N_HOMALT=0;N_HOMREF=10303;FREQ_HET=0.0005820157144242894;FREQ_HOMALT=0.0;FREQ_HOMREF=0.9994179842855757;N_BI_GENOS=10309;Lingnan_Han_AN=1888;Lingnan_Han_AC=1;Lingnan
chr6	91841093	.	T	<INS:ME:LINE1>	.	PASS	SVTYPE=INS:ME:LINE1;END=91841093;SVLEN=0;AN=20618;AC=1;AF=4.850130953535746e-05;N_HET=1;N_HOMALT=0;N_HOMREF=10308;FREQ_HET=9.700261907071492e-05;FREQ_HOMALT=0.0;FREQ_HOMREF=0.9999029973809292;N_BI_GENOS=10309;Lingnan_Han_AN=1888;Lingnan_Han_AC=1;Lingnan_H
chr6	92001639	.	G	<INS:ME:LINE1>	.	PASS	SVTYPE=INS:ME:LINE1;END=92001655;SVLEN=582;AN=20618;AC=2;AF=9.700261907071492e-05;N_HET=2;N_HOMALT=0;N_HOMREF=10307;FREQ_HET=0.00019400523814142983;FREQ_HOMALT=0.0;FREQ_HOMREF=0.9998059947618586;N_BI_GENOS=10309;Central_Han_AN=2128;Central_Han_AC=2;Centra
chr6	92226755	.	T	<INS:ME:LINE1>	.	PASS	SVTYPE=INS:ME:LINE1;END=92226770;SVLEN=2041;AN=20618;AC=23;AF=0.0011155301193132214;N_HET=23;N_HOMALT=0;N_HOMREF=10286;FREQ_HET=0.0022310602386264443;FREQ_HOMALT=0.0;FREQ_HOMREF=0.9977689397613736;N_BI_GENOS=10309;South_Han_AN=1496;South_Han_AC=1;South_Han
chr6	92249699	.	A	<INS:ME:LINE1>	.	PASS	SVTYPE=INS:ME:LINE1;END=92249711;SVLEN=106;AN=20618;AC=1;AF=4.850130953535746e-05;N_HET=1;N_HOMALT=0;N_HOMREF=10308;FREQ_HET=9.700261907071492e-05;FREQ_HOMALT=0.0;FREQ_HOMREF=0.9999029973809292;N_BI_GENOS=10309;South_Han_AN=1496;South_Han_AC=1;South_Han_A
chr6	92296221	.	T	<INS:ME:LINE1>	.	PASS	SVTYPE=INS:ME:LINE1;END=92296222;SVLEN=1052;AN=20618;AC=1;AF=4.850130953535746e-05;N_HET=1;N_HOMALT=0;N_HOMREF=10308;FREQ_HET=9.700261907071492e-05;FREQ_HOMALT=0.0;FREQ_HOMREF=0.9999029973809292;N_BI_GENOS=10309;East_Han_AN=6554;East_Han_AC=1;East_Han_AF=
chr6	92397576	.	G	<INS:ME:LINE1>	.	PASS	SVTYPE=INS:ME:LINE1;END=92397592;SVLEN=2015;AN=20618;AC=1;AF=4.850130953535746e-05;N_HET=1;N_HOMALT=0;N_HOMREF=10308;FREQ_HET=9.700261907071492e-05;FREQ_HOMALT=0.0;FREQ_HOMREF=0.9999029973809292;N_BI_GENOS=10309;South_Han_AN=1496;South_Han_AC=1;South_Han_

chr6	92729123 .	C	<INS:ME:LINE1>	.	PASS	SVTYPE=INS:ME:LINE1;END=92729135;SVLEN=6000;AN=20618;AC=1;AF=4.850130953535746e-05;N_HET=1;N_HOMALT=0;N_HOMREF=10308;FREQ_HET=9.700261907071492e-05;FREQ_HOMALT=0.0;FREQ_HOMREF=0.9999029973809292;N_BI_GENOS=10309;Central_Han_AN=2128;Central_Han_AC=1;Centra
chr6	92758232 .	A	<INS:ME:LINE1>	.	PASS	SVTYPE=INS:ME:LINE1;END=92758246;SVLEN=5986;AN=20618;AC=3;AF=0.00014550392860607236;N_HET=3;N_HOMALT=0;N_HOMREF=10306;FREQ_HET=0.0002910078572121447;FREQ_HOMALT=0.0;FREQ_HOMREF=0.9997089921427879;N_BI_GENOS=10309;South_Han_AN=1496;South_Han_AC=1;South_Han
chr6	93205480 .	C	<INS:ME:LINE1>	.	PASS	SVTYPE=INS:ME:LINE1;END=93205480;SVLEN=771;AN=20618;AC=1;AF=4.850130953535746e-05;N_HET=1;N_HOMALT=0;N_HOMREF=10308;FREQ_HET=9.700261907071492e-05;FREQ_HOMALT=0.0;FREQ_HOMREF=0.9999029973809292;N_BI_GENOS=10309;East_Han_AN=6554;East_Han_AC=1;East_Han_AF=0
chr6	93657230 .	G	<INS:ME:LINE1>	.	PASS	SVTYPE=INS:ME:LINE1;END=93657230;SVLEN=53;AN=20618;AC=1;AF=4.850130953535746e-05;N_HET=1;N_HOMALT=0;N_HOMREF=10308;FREQ_HET=9.700261907071492e-05;FREQ_HOMALT=0.0;FREQ_HOMREF=0.9999029973809292;N_BI_GENOS=10309;East_Han_AN=6554;East_Han_AC=1;East_Han_AF=0.
chr6	93657386 .	G	<INS:ME:LINE1>	.	PASS	SVTYPE=INS:ME:LINE1;END=93657399;SVLEN=6009;AN=20618;AC=1;AF=4.850130953535746e-05;N_HET=1;N_HOMALT=0;N_HOMREF=10308;FREQ_HET=9.700261907071492e-05;FREQ_HOMALT=0.0;FREQ_HOMREF=0.9999029973809292;N_BI_GENOS=10309;South_Han_AN=1496;South_Han_AC=1;South_Han_
chr6	93851768 .	T	<INS:ME:LINE1>	.	PASS	SVTYPE=INS:ME:LINE1;END=93851777;SVLEN=1092;AN=20618;AC=3;AF=0.00014550392860607236;N_HET=1;N_HOMALT=1;N_HOMREF=10307;FREQ_HET=9.700261907071492e-05;FREQ_HOMALT=9.700261907071492e-05;FREQ_HOMREF=0.9998059947618586;N_BI_GENOS=10309;East_Han_AN=6554;East_Ha
chr6	94000273 .	C	<INS:ME:LINE1>	.	PASS	SVTYPE=INS:ME:LINE1;END=94000281;SVLEN=61;AN=20618;AC=2;AF=9.700261907071492e-05;N_HET=2;N_HOMALT=0;N_HOMREF=10307;FREQ_HET=0.00019400523814142983;FREQ_HOMALT=0.0;FREQ_HOMREF=0.9998059947618586;N_BI_GENOS=10309;Lingnan_Han_AN=1888;Lingnan_Han_AC=1;Lingnan
chr6	94383563 .	T	<INS:ME:LINE1>	.	PASS	SVTYPE=INS:ME:LINE1;END=94383577;SVLEN=6000;AN=20618;AC=1;AF=4.850130953535746e-05;N_HET=1;N_HOMALT=0;N_HOMREF=10308;FREQ_HET=9.700261907071492e-05;FREQ_HOMALT=0.0;FREQ_HOMREF=0.9999029973809292;N_BI_GENOS=10309;North_Han_AN=2272;North_Han_AC=1;North_Han_
chr6	94408467 .	C	<INS:ME:LINE1>	.	PASS	SVTYPE=INS:ME:LINE1;END=94408476;SVLEN=1104;AN=20618;AC=1;AF=4.850130953535746e-05;N_HET=1;N_HOMALT=0;N_HOMREF=10308;FREQ_HET=9.700261907071492e-05;FREQ_HOMALT=0.0;FREQ_HOMREF=0.9999029973809292;N_BI_GENOS=10309;East_Han_AN=6554;East_Han_AC=1;East_Han_AF=
chr6	94457264 .	A	<INS:ME:LINE1>	.	PASS	SVTYPE=INS:ME:LINE1;END=94457264;SVLEN=10;AN=20618;AC=1;AF=4.850130953535746e-05;N_HET=1;N_HOMALT=0;N_HOMREF=10308;FREQ_HET=9.700261907071492e-05;FREQ_HOMALT=0.0;FREQ_HOMREF=0.9999029973809292;N_BI_GENOS=10309;Southeast_Han_AN=1908;Southeast_Han_AC=1;Sout
chr6	94595774 .	T	<INS:ME:LINE1>	.	PASS	SVTYPE=INS:ME:LINE1;END=94595774;SVLEN=0;AN=20618;AC=1;AF=4.850130953535746e-05;N_HET=1;N_HOMALT=0;N_HOMREF=10308;FREQ_HET=9.700261907071492e-05;FREQ_HOMALT=0.0;FREQ_HOMREF=0.9999029973809292;N_BI_GENOS=10309;East_Han_AN=6554;East_Han_AC=1;East_Han_AF=0.0
chr6	94613400 .	A	<INS:ME:LINE1>	.	PASS	SVTYPE=INS:ME:LINE1;END=94613400;SVLEN=710;AN=20618;AC=1;AF=4.850130953535746e-05;N_HET=1;N_HOMALT=0;N_HOMREF=10308;FREQ_HET=9.700261907071492e-05;FREQ_HOMALT=0.0;FREQ_HOMREF=0.9999029973809292;N_BI_GENOS=10309;East_Han_AN=6554;East_Han_AC=1;East_Han_AF=0
chr6	94619704 .	A	<INS:ME:LINE1>	.	PASS	SVTYPE=INS:ME:LINE1;END=94619704;SVLEN=439;AN=20618;AC=1;AF=4.850130953535746e-05;N_HET=1;N_HOMALT=0;N_HOMREF=10308;FREQ_HET=9.700261907071492e-05;FREQ_HOMALT=0.0;FREQ_HOMREF=0.9999029973809292;N_BI_GENOS=10309;East_Han_AN=6554;East_Han_AC=1;East_Han_AF=0
chr6	95016089 .	A	<INS:ME:LINE1>	.	PASS	SVTYPE=INS:ME:LINE1;END=95016102;SVLEN=2243;AN=20618;AC=1;AF=4.850130953535746e-05;N_HET=1;N_HOMALT=0;N_HOMREF=10308;FREQ_HET=9.700261907071492e-05;FREQ_HOMALT=0.0;FREQ_HOMREF=0.9999029973809292;N_BI_GENOS=10309;Northwest_Han_AN=1296;Northwest_Han_AC=1;No
chr6	95125841 .	A	<INS:ME:LINE1>	.	PASS	SVTYPE=INS:ME:LINE1;END=95125846;SVLEN=97;AN=20618;AC=4;AF=0.00019400523814142983;N_HET=4;N_HOMALT=0;N_HOMREF=10305;FREQ_HET=0.00038801047628285966;FREQ_HOMALT=0.0;FREQ_HOMREF=0.9996119895237171;N_BI_GENOS=10309;South_Han_AN=1496;South_Han_AC=1;South_Han_
chr6	95148714 .	C	<INS:ME:LINE1>	.	PASS	SVTYPE=INS:ME:LINE1;END=95148714;SVLEN=149;AN=20618;AC=4;AF=0.00019400523814142983;N_HET=4;N_HOMALT=0;N_HOMREF=10305;FREQ_HET=0.00038801047628285966;FREQ_HOMALT=0.0;FREQ_HOMREF=0.9996119895237171;N_BI_GENOS=10309;Northwest_Han_AN=1296;Northwest_Han_AC=1;N
chr6	95229002 .	C	<INS:ME:LINE1>	.	PASS	SVTYPE=INS:ME:LINE1;END=95229006;SVLEN=48;AN=20618;AC=1;AF=4.850130953535746e-05;N_HET=1;N_HOMALT=0;N_HOMREF=10308;FREQ_HET=9.700261907071492e-05;FREQ_HOMALT=0.0;FREQ_HOMREF=0.9999029973809292;N_BI_GENOS=10309;East_Han_AN=6554;East_Han_AC=1;East_Han_AF=0.
chr6	95265560 .	T	<INS:ME:LINE1>	.	PASS	SVTYPE=INS:ME:LINE1;END=95265561;SVLEN=387;AN=20618;AC=2;AF=9.700261907071492e-05;N_HET=2;N_HOMALT=0;N_HOMREF=10307;FREQ_HET=0.00019400523814142983;FREQ_HOMALT=0.0;FREQ_HOMREF=0.9998059947618586;N_BI_GENOS=10309;Lingnan_Han_AN=1888;Lingnan_Han_AC=1;Lingna
chr6	95272986 .	T	<INS:ME:LINE1>	.	PASS	SVTYPE=INS:ME:LINE1;END=95273001;SVLEN=820;AN=20618;AC=5;AF=0.00024250654767678728;N_HET=5;N_HOMALT=0;N_HOMREF=10304;FREQ_HET=0.00048501309535357455;FREQ_HOMALT=0.0;FREQ_HOMREF=0.9995149869046465;N_BI_GENOS=10309;Northwest_Han_AN=1296;Northwest_Han_AC=1;N
chr6	95767236 .	A	<INS:ME:LINE1>	.	PASS	SVTYPE=INS:ME:LINE1;END=95767246;SVLEN=1404;AN=20618;AC=5;AF=0.00024250654767678728;N_HET=5;N_HOMALT=0;N_HOMREF=10304;FREQ_HET=0.00048501309535357455;FREQ_HOMALT=0.0;FREQ_HOMREF=0.9995149869046465;N_BI_GENOS=10309;Lingnan_Han_AN=1888;Lingnan_Han_AC=1;Ling
chr6	95977077 .	T	<INS:ME:LINE1>	.	PASS	SVTYPE=INS:ME:LINE1;END=95977090;SVLEN=6064;AN=20618;AC=2;AF=9.700261907071492e-05;N_HET=2;N_HOMALT=0;N_HOMREF=10307;FREQ_HET=0.00019400523814142983;FREQ_HOMALT=0.0;FREQ_HOMREF=0.9998059947618586;N_BI_GENOS=10309;East_Han_AN=6554;East_Han_AC=2;East_Han_AF
chr6	96296371 .	A	<INS:ME:LINE1>	.	PASS	SVTYPE=INS:ME:LINE1;END=96296371;SVLEN=957;AN=20618;AC=1;AF=4.850130953535746e-05;N_HET=1;N_HOMALT=0;N_HOMREF=10308;FREQ_HET=9.700261907071492e-05;FREQ_HOMALT=0.0;FREQ_HOMREF=0.9999029973809292;N_BI_GENOS=10309;East_Han_AN=6554;East_Han_AC=1;East_Han_AF=0
chr6	96418927 .	C	<INS:ME:LINE1>	.	PASS	SVTYPE=INS:ME:LINE1;END=96418927;SVLEN=12;AN=20618;AC=1;AF=4.850130953535746e-05;N_HET=1;N_HOMALT=0;N_HOMREF=10308;FREQ_HET=9.700261907071492e-05;FREQ_HOMALT=0.0;FREQ_HOMREF=0.9999029973809292;N_BI_GENOS=10309;South_Han_AN=1496;South_Han_AC=1;South_Han_AF
chr6	96453241 .	A	<INS:ME:LINE1>	.	PASS	SVTYPE=INS:ME:LINE1;END=96453257;SVLEN=6005;AN=20618;AC=2;AF=9.700261907071492e-05;N_HET=2;N_HOMALT=0;N_HOMREF=10307;FREQ_HET=0.00019400523814142983;FREQ_HOMALT=0.0;FREQ_HOMREF=0.9998059947618586;N_BI_GENOS=10309;East_Han_AN=6554;East_Han_AC=1;East_Han_AF
chr6	96706917 .	C	<INS:ME:LINE1>	.	PASS	SVTYPE=INS:ME:LINE1;END=96706933;SVLEN=6000;AN=20618;AC=2;AF=9.700261907071492e-05;N_HET=2;N_HOMALT=0;N_HOMREF=10307;FREQ_HET=0.00019400523814142983;FREQ_HOMALT=0.0;FREQ_HOMREF=0.9998059947618586;N_BI_GENOS=10309;Lingnan_Han_AN=1888;Lingnan_Han_AC=1;Lingn
chr6	96927485 .	T	<INS:ME:LINE1>	.	PASS	SVTYPE=INS:ME:LINE1;END=96927504;SVLEN=223;AN=20618;AC=2;AF=9.700261907071492e-05;N_HET=2;N_HOMALT=0;N_HOMREF=10307;FREQ_HET=0.00019400523814142983;FREQ_HOMALT=0.0;FREQ_HOMREF=0.9998059947618586;N_BI_GENOS=10309;South_Han_AN=1496;South_Han_AC=1;South_Han_
chr6	97003345 .	T	<INS:ME:LINE1>	.	PASS	SVTYPE=INS:ME:LINE1;END=97003367;SVLEN=809;AN=20618;AC=1;AF=4.850130953535746e-05;N_HET=1;N_HOMALT=0;N_HOMREF=10308;FREQ_HET=9.700261907071492e-05;FREQ_HOMALT=0.0;FREQ_HOMREF=0.9999029973809292;N_BI_GENOS=10309;East_Han_AN=6554;East_Han_AC=1;East_Han_AF=0
chr6	97199701 .	T	<INS:ME:LINE1>	.	PASS	SVTYPE=INS:ME:LINE1;END=97199716;SVLEN=5997;AN=20618;AC=2;AF=9.700261907071492e-05;N_HET=2;N_HOMALT=0;N_HOMREF=10307;FREQ_HET=0.00019400523814142983;FREQ_HOMALT=0.0;FREQ_HOMREF=0.9998059947618586;N_BI_GENOS=10309;Northwest_Han_AN=1296;Northwest_Han_AC=2;N
chr6	97434622 .	G	<INS:ME:LINE1>	.	PASS	SVTYPE=INS:ME:LINE1;END=97434638;SVLEN=804;AN=20618;AC=53;AF=0.002570569405373945;N_HET=43;N_HOMALT=5;N_HOMREF=10261;FREQ_HET=0.004171112620040741;FREQ_HOMALT=0.0004850130953535357455;FREQ_HOMREF=0.9953438742846057;N_BI_GENOS=10309;South_Han_AN=1496;South_H
chr6	97464757 .	T	<INS:ME:LINE1>	.	PASS	SVTYPE=INS:ME:LINE1;END=97464773;SVLEN=467;AN=20618;AC=1;AF=4.850130953535746e-05;N_HET=1;N_HOMALT=0;N_HOMREF=10308;FREQ_HET=9.700261907071492e-05;FREQ_HOMALT=0.0;FREQ_HOMREF=0.9999029973809292;N_BI_GENOS=10309;East_Han_AN=6554;East_Han_AC=1;East_Han_AF=0
chr6	97648321 .	A	<INS:ME:LINE1>	.	PASS	SVTYPE=INS:ME:LINE1;END=97648337;SVLEN=838;AN=20618;AC=6;AF=0.0002910078572121447;N_HET=4;N_HOMALT=1;N_HOMREF=10304;FREQ_HET=0.00038801047628285966;FREQ_HOMALT=9.700261907071492e-05;FREQ_HOMREF=0.9995149869046465;N_BI_GENOS=10309;Lingnan_Han_AN=1888;Lingn
chr6	97650504 .	T	<INS:ME:LINE1>	.	PASS	SVTYPE=INS:ME:LINE1;END=97650523;SVLEN=965;AN=20618;AC=1;AF=4.850130953535746e-05;N_HET=1;N_HOMALT=0;N_HOMREF=10308;FREQ_HET=9.700261907071492e-05;FREQ_HOMALT=0.0;FREQ_HOMREF=0.9999029973809292;N_BI_GENOS=10309;Tibetan_AN=414;Tibetan_AC=1;Tibetan_AF=0.002
chr6	97721006 .	A	<INS:ME:LINE1>	.	PASS	SVTYPE=INS:ME:LINE1;END=97721020;SVLEN=1462;AN=20618;AC=2;AF=9.700261907071492e-05;N_HET=2;N_HOMALT=0;N_HOMREF=10307;FREQ_HET=0.00019400523814142983;FREQ_HOMALT=0.0;FREQ_HOMREF=0.9998059947618586;N_BI_GENOS=10309;South_Han_AN=1496;South_Han_AC=1;South_Han
chr6	97996246 .	A	<INS:ME:LINE1>	.	PASS	SVTYPE=INS:ME:LINE1;END=97996249;SVLEN=78;AN=20618;AC=1;AF=4.850130953535746e-05;N_HET=1;N_HOMALT=0;N_HOMREF=10308;FREQ_HET=9.700261907071492e-05;FREQ_HOMALT=0.0;FREQ_HOMREF=0.9999029973809292;N_BI_GENOS=10309;Zhuang_AN=444;Zhuang_AC=1;Zhuang_AF=0.0022522
chr6	98100400 .	T	<INS:ME:LINE1>	.	PASS	SVTYPE=INS:ME:LINE1;END=98100400;SVLEN=74;AN=20618;AC=1;AF=4.850130953535746e-05;N_HET=1;N_HOMALT=0;N_HOMREF=10308;FREQ_HET=9.700261907071492e-05;FREQ_HOMALT=0.0;FREQ_HOMREF=0.9999029973809292;N_BI_GENOS=10309;East_Han_AN=6554;East_Han_AC=1;East_Han_AF=0.
chr6	98105495 .	C	<INS:ME:LINE1>	.	PASS	SVTYPE=INS:ME:LINE1;END=98105505;SVLEN=5994;AN=20618;AC=1;AF=4.850130953535746e-05;N_HET=1;N_HOMALT=0;N_HOMREF=10308;FREQ_HET=9.700261907071492e-05;FREQ_HOMALT=0.0;FREQ_HOMREF=0.9999029973809292;N_BI_GENOS=10309;East_Han_AN=6554;East_Han_AC=1;East_Han_AF=
chr6	98125916 .	T	<INS:ME:LINE1>	.	PASS	SVTYPE=INS:ME:LINE1;END=98125920;SVLEN=940;AN=20618;AC=1;AF=4.850130953535746e-05;N_HET=1;N_HOMALT=0;N_HOMREF=10308;FREQ_HET=9.700261907071492e-05;FREQ_HOMALT=0.0;FREQ_HOMREF=0.9999029973809292;N_BI_GENOS=10309;Central_Han_AN=2128;Central_Han_AC=1;Central
chr6	98204247 .	C	<INS:ME:LINE1>	.	PASS	SVTYPE=INS:ME:LINE1;END=98204257;SVLEN=5997;AN=20618;AC=1457;AF=0.07066640799301581;N_HET=1287;N_HOMALT=85;N_HOMREF=8937;FREQ_HET=0.12484237074401008;FREQ_HOMALT=0.008245222621010767;FREQ_HOMREF=0.8669124066349791;N_BI_GENOS=10309;South_Han_AN=1496;South_
chr6	98355311 .	T	<INS:ME:LINE1>	.	PASS	SVTYPE=INS:ME:LINE1;END=98355319;SVLEN=6000;AN=20618;AC=1;AF=4.850130953535746e-05;N_HET=1;N_HOMALT=0;N_HOMREF=10308;FREQ_HET=9.700261907071492e-05;FREQ_HOMALT=0.0;FREQ_HOMREF=0.9999029973809292;N_BI_GENOS=10309;East_Han_AN=6554;East_Han_AC=1;East_Han_AF=
chr6	99091253 .	G	<INS:ME:LINE1>	.	PASS	SVTYPE=INS:ME:LINE1;END=99091266;SVLEN=6000;AN=20618;AC=1;AF=4.850130953535746e-05;N_HET=1;N_HOMALT=0;N_HOMREF=10308;FREQ_HET=9.700261907071492e-05;FREQ_HOMALT=0.0;FREQ_HOMREF=0.9999029973809292;N_BI_GENOS=10309;Southeast_Han_AN=1908;Southeast_Han_AC=1;So
chr6	99271503 .	C	<INS:ME:LINE1>	.	PASS	SVTYPE=INS:ME:LINE1;END=99271521;SVLEN=663;AN=20618;AC=4;AF=0.00019400523814142983;N_HET=4;N_HOMALT=0;N_HOMREF=10305;FREQ_HET=0.00038801047628285966;FREQ_HOMALT=0.0;FREQ_HOMREF=0.9996119895237171;N_BI_GENOS=10309;North_Han_AN=2272;North_Han_AC=1;North_Han
chr6	99746029 .	A	<INS:ME:LINE1>	.	PASS	SVTYPE=INS:ME:LINE1;END=99746029;SVLEN=1872;AN=20618;AC=1;AF=4.850130953535746e-05;N_HET=1;N_HOMALT=0;N_HOMREF=10308;FREQ_HET=9.700261907071492e-05;FREQ_HOMALT=0.0;FREQ_HOMREF=0.9999029973809292;N_BI_GENOS=10309;Miao_AN=482;Miao_AC=1;Miao_AF=0.00207468879
chr6	99847135 .	T	<INS:ME:LINE1>	.	PASS	SVTYPE=INS:ME:LINE1;END=99847147;SVLEN=6000;AN=20618;AC=1;AF=4.850130953535746e-05;N_HET=1;N_HOMALT=0;N_HOMREF=10308;FREQ_HET=9.700261907071492e-05;FREQ_HOMALT=0.0;FREQ_HOMREF=0.9999029973809292;N_BI_GENOS=10309;East_Han_AN=6554;East_Han_AC=1;East_Han_AF=
chr6	100003808 .	T	<INS:ME:LINE1>	.	PASS	SVTYPE=INS:ME:LINE1;END=100003822;SVLEN=1595;AN=20618;AC=1;AF=4.850130953535746e-05;N_HET=1;N_HOMALT=0;N_HOMREF=10308;FREQ_HET=9.700261907071492e-05;FREQ_HOMALT=0.0;FREQ_HOMREF=0.9999029973809292;N_BI_GENOS=10309;Southeast_Han_AN=1908;Southeast_Han_AC=1;S
chr6	100177063 .	A	<INS:ME:LINE1>	.	PASS	SVTYPE=INS:ME:LINE1;END=100177075;SVLEN=6003;AN=20618;AC=3;AF=0.00014550392860607236;N_HET=3;N_HOMALT=0;N_HOMREF=10306;FREQ_HET=0.0002910078572121447;FREQ_HOMALT=0.0;FREQ_HOMREF=0.9997089921427879;N_BI_GENOS=10309;East_Han_AN=6554;East_Han_AC=2;East_Han_A
chr6	100445961 .	G	<INS:ME:LINE1>	.	PASS	SVTYPE=INS:ME:LINE1;END=100445975;SVLEN=1413;AN=20618;AC=1;AF=4.850130953535746e-05;N_HET=1;N_HOMALT=0;N_HOMREF=10308;FREQ_HET=9.700261907071492e-05;FREQ_HOMALT=0.0;FREQ_HOMREF=0.9999029973809292;N_BI_GENOS=10309;East_Han_AN=6554;East_Han_AC=1;East_Han_AF
chr6	100746160 .	A	<INS:ME:LINE1>	.	PASS	SVTYPE=INS:ME:LINE1;END=100746160;SVLEN=33;AN=20618;AC=635;AF=0.030798331554951985;N_HET=117;N_HOMALT=259;N_HOMREF=9933;FREQ_HET=0.011349306431273645;FREQ_HOMALT=0.025123678339315163;FREQ_HOMREF=0.9635270152294112;N_BI_GENOS=10309;South_Han_AN=1496;South_
chr6	100983884 .	T	<INS:ME:LINE1>	.	PASS	SVTYPE=INS:ME:LINE1;END=100983899;SVLEN=5994;AN=20618;AC=1;AF=4.850130953535746e-05;N_HET=1;N_HOMALT=0;N_HOMREF=10308;FREQ_HET=9.700261907071492e-05;FREQ_HOMALT=0.0;FREQ_HOMREF=0.9999029973809292;N_BI_GENOS=10309;Manchu_AN=416;Manchu_AC=1;Manchu_AF=0.0024
chr6	101006580 .	A	<INS:ME:LINE1>	.	PASS	SVTYPE=INS:ME:LINE1;END=101006591;SVLEN=2299;AN=20618;AC=31;AF=0.0015035405955960812;N_HET=31;N_HOMALT=0;N_HOMREF=10278;FREQ_HET=0.0030070811911921624;FREQ_HOMALT=0.0;FREQ_HOMREF=0.9969929188088078;N_BI_GENOS=10309;South_Han_AN=1496;South_Han_AC=5;South_H
chr6	101353147 .	A	<INS:ME:LINE1>	.	PASS	SVTYPE=INS:ME:LINE1;END=101353160;SVLEN=785;AN=20618;AC=3;AF=0.00014550392860607236;N_HET=3;N_HOMALT=0;N_HOMREF=10306;FREQ_HET=0.0002910078572121447;FREQ_HOMALT=0.0;FREQ_HOMREF=0.9997089921427879;N_BI_GENOS=10309;North_Han_AN=2272;North_Han_AC=3;North_Han
chr6	101368876 .	A	<INS:ME:LINE1>	.	PASS	SVTYPE=INS:ME:LINE1;END=101368893;SVLEN=1644;AN=20618;AC=1;AF=4.850130953535746e-05;N_HET=1;N_HOMALT=0;N_HOMREF=10308;FREQ_HET=9.700261907071492e-05;FREQ_HOMALT=0.0;FREQ_HOMREF=0.9999029973809292;N_BI_GENOS=10309;Southeast_Han_AN=1908;Southeast_Han_AC=1;S
chr6	101530830 .	G	<INS:ME:LINE1>	.	PASS	SVTYPE=INS:ME:LINE1;END=101530848;SVLEN=6064;AN=20618;AC=1;AF=4.850130953535746e-05;N_HET=1;N_HOMALT=0;N_HOMREF=10308;FREQ_HET=9.700261907071492e-05;FREQ_HOMALT=0.0;FREQ_HOMREF=0.9999029973809292;N_BI_GENOS=10309;East_Han_AN=6554;East_Han_AC=1;East_Han_AF
chr6	101577024 .	G	<INS:ME:LINE1>	.	PASS	SVTYPE=INS:ME:LINE1;END=101577024;SVLEN=977;AN=20618;AC=1;AF=4.850130953535746e-05;N_HET=1;N_HOMALT=0;N_HOMREF=10308;FREQ_HET=9.700261907071492e-05;FREQ_HOMALT=0.0;FREQ_HOMREF=0.9999029973809292;N_BI_GENOS=10309;East_Han_AN=6554;East_Han_AC=1;East_Han_AF=
chr6	101835883 .	G	<INS:ME:LINE1>	.	PASS	SVTYPE=INS:ME:LINE1;END=101835896;SVLEN=1085;AN=20618;AC=5337;AF=0.2588514889902027;N_HET=3163;N_HOMALT=1087;N_HOMREF=6059;FREQ_HET=0.3068192841206713;FREQ_HOMALT=0.1054418469298671;FREQ_HOMREF=0.5877388689494616;N_BI_GENOS=10309;South_Han_AN=1496;South_H
chr6	101837601 .	T	<INS:ME:LINE1>	.	PASS	SVTYPE=INS:ME:LINE1;END=101837612;SVLEN=75;AN=20618;AC=1;AF=4.850130953535746e-05;N_HET=1;N_HOMALT=0;N_HOMREF=10308;FREQ_HET=9.700261907071492e-05;FREQ_HOMALT=0.0;FREQ_HOMREF=0.9999029973809292;N_BI_GENOS=10309;East_Han_AN=6554;East_Han_AC=1;East_Han_AF=0
chr6	101877402 .	T	<INS:ME:LINE1>	.	PASS	SVTYPE=INS:ME:LINE1;END=101877402;SVLEN=545;AN=20618;AC=1;AF=4.850130953535746e-05;N_HET=1;N_HOMALT=0;N_HOMREF=10308;FREQ_HET=9.700261907071492e-05;FREQ_HOMALT=0.0;FREQ_HOMREF=0.9999029973809292;N_BI_GENOS=10309;South_Han_AN=1496;South_Han_AC=1;South_Han_
chr6	101907606 .	A	<INS:ME:LINE1>	.	PASS	SVTYPE=INS:ME:LINE1;END=101907622;SVLEN=2235;AN=20618;AC=1;AF=4.850130953535746e-05;N_HET=1;N_HOMALT=0;N_HOMREF=10308;FREQ_HET=9.700261907071492e-05;FREQ_HOMALT=0.0;FREQ_HOMREF=0.9999029973809292;N_BI_GENOS=10309;Central_Han_AN=2128;Central_Han_AC=1;Centr
chr6	101915113 .	A	<INS:ME:LINE1>	.	PASS	SVTYPE=INS:ME:LINE1;END=101915115;SVLEN=354;AN=20618;AC=1;AF=4.850130953535746e-05;N_HET=1;N_HOMALT=0;N_HOMREF=10308;FREQ_HET=9.700261907071492e-05;FREQ_HOMALT=0.0;FREQ_HOMREF=0.9999029973809292;N_BI_GENOS=10309;Mongolian_AN=454;Mongolian_AC=1;Mongolian_A
chr6	102215516 .	A	<INS:ME:LINE1>	.	PASS	SVTYPE=INS:ME:LINE1;END=102215524;SVLEN=6004;AN=20618;AC=1;AF=4.850130953535746e-05;N_HET=1;N_HOMALT=0;N_HOMREF=10308;FREQ_HET=9.700261907071492e-05;FREQ_HOMALT=0.0;FREQ_HOMREF=0.9999029973809292;N_BI_GENOS=10309;South_Han_AN=1496;South_Han_AC=1;South_Han
chr6	102220374 .	C	<INS:ME:LINE1>	.	PASS	SVTYPE=INS:ME:LINE1;END=102220386;SVLEN=740;AN=20618;AC=4;AF=0.00019400523814142983;N_HET=4;N_HOMALT=0;N_HOMREF=10305;FREQ_HET=0.00038801047628285966;FREQ_HOMALT=0.0;FREQ_HOMREF=0.9996119895237171;N_BI_GENOS=10309;South_Han_AN=1496;South_Han_AC=1;South_Ha
chr6	102289126 .	T	<INS:ME:LINE1>	.	PASS	SVTYPE=INS:ME:LINE1;END=102289126;SVLEN=1274;AN=20618;AC=16;AF=0.0007760209525657193;N_HET=16;N_HOMALT=0;N_HOMREF=10293;FREQ_HET=0.0015520419051314386;FREQ_HOMALT=0.0;FREQ_HOMREF=0.9984479580948685;N_BI_GENOS=10309;South_Han_AN=1496;South_Han_AC=2;South_H
chr6	102304206 .	A	<INS:ME:LINE1>	.	PASS	SVTYPE=INS:ME:LINE1;END=102304206;SVLEN=173;AN=20618;AC=1;AF=4.850130953535746e-05;N_HET=1;N_HOMALT=0;N_HOMREF=10308;FREQ_HET=9.700261907071492e-05;FREQ_HOMALT=0.0;FREQ_HOMREF=0.9999029973809292;N_BI_GENOS=10309;East_Han_AN=6554;East_Han_AC=1;East_Han_AF=
chr6	102397595 .	C	<INS:ME:LINE1>	.	PASS	SVTYPE=INS:ME:LINE1;END=102397595;SVLEN=205;AN=20618;AC=2;AF=9.700261907071492e-05;N_HET=2;N_HOMALT=0;N_HOMREF=10307;FREQ_HET=0.00019400523814142983;FREQ_HOMALT=0.0;FREQ_HOMREF=0.9998059947618586;N_BI_GENOS=10309;East_Han_AN=6554;East_Han_AC=1;East_Han_AF
chr6	102397801 .	T	<INS:ME:LINE1>	.	PASS	SVTYPE=INS:ME:LINE1;END=102397801;SVLEN=256;AN=20618;AC=143;AF=0.006935687263556 1164;N_HET=143;N_HOMALT=0;N_HOMREF=10166;FREQ_HET=0.013871374527112233;FREQ_HOMALT=0.0;FREQ_HOMREF=0.9861286254728878;N_BI_GENOS=10309;South_Han_AN=1496;South_Han_AC=24;South_
chr6	102398210 .	G	<INS:ME:LINE1>	.	PASS	SVTYPE=INS:ME:LINE1;END=102398219;SVLEN=6000;AN=20618;AC=1558;AF=0.07556504025608692;N_HET=748;N_HOMALT=405;N_HOMREF=9156;FREQ_HET=0.07255795906489475;FREQ_HOMALT=0.039286060723639535;FREQ_HOMREF=0.888155980 2114657;N_BI_GENOS=10309;South_Han_AN=1496;South
chr6	102421819 .	C	<INS:ME:LINE1>	.	PASS	SVTYPE=INS:ME:LINE1;END=102421819;SVLEN=537;AN=20618;AC=1;AF=4.850130953535746e-05;N_HET=1;N_HOMALT=0;N_HOMREF=10308;FREQ_HET=9.700261907071492e-05;FREQ_HOMALT=0.0;FREQ_HOMREF=0.9999029973809292;N_BI_GENOS=10309;East_Han_AN=6554;East_Han_AC=1;East_Han_AF=
chr6	102427758 .	A	<INS:ME:LINE1>	.	PASS	SVTYPE=INS:ME:LINE1;END=102427759;SVLEN=515;AN=20618;AC=5;AF=0.00024250654767678728;N_HET=5;N_HOMALT=0;N_HOMREF=10304;FREQ_HET=0.00048501309535357455;FREQ_HOMALT=0.0;FREQ_HOMREF=0.9995149869046465;N_BI_GENOS=10309;South_Han_AN=1496;South_Han_AC=1;South_Ha
chr6	102444553 .	A	<INS:ME:LINE1>	.	PASS	SVTYPE=INS:ME:LINE1;END=102444568;SVLEN=565;AN=20618;AC=1;AF=4.850130953535746e-05;N_HET=1;N_HOMALT=0;N_HOMREF=10308;FREQ_HET=9.700261907071492e-05;FREQ_HOMALT=0.0;FREQ_HOMREF=0.9999029973809292;N_BI_GENOS=10309;Southeast_Han_AN=1908;Southeast_Han_AC=1;So
chr6	102791211 .	A	<INS:ME:LINE1>	.	PASS	SVTYPE=INS:ME:LINE1;END=102791220;SVLEN=789;AN=20618;AC=1;AF=4.850130953535746e-05;N_HET=1;N_HOMALT=0;N_HOMREF=10308;FREQ_HET=9.700261907071492e-05;FREQ_HOMALT=0.0;FREQ_HOMREF=0.9999029973809292;N_BI_GENOS=10309;Southeast_Han_AN=1908;Southeast_Han_AC=1;So
chr6	102865858 .	G	<INS:ME:LINE1>	.	PASS	SVTYPE=INS:ME:LINE1;END=102865858;SVLEN=674;AN=20618;AC=1;AF=4.850130953535746e-05;N_HET=1;N_HOMALT=0;N_HOMREF=10308;FREQ_HET=9.700261907071492e-05;FREQ_HOMALT=0.0;FREQ_HOMREF=0.9999029973809292;N_BI_GENOS=10309;East_Han_AN=6554;East_Han_AC=1;East_Han_AF=
chr6	103056694 .	T	<INS:ME:LINE1>	.	PASS	SVTYPE=INS:ME:LINE1;END=103056694;SVLEN=1048;AN=20618;AC=1;AF=4.850130953535746e-05;N_HET=1;N_HOMALT=0;N_HOMREF=10308;FREQ_HET=9.700261907071492e-05;FREQ_HOMALT=0.0;FREQ_HOMREF=0.9999029973809292;N_BI_GENOS=10309;East_Han_AN=6554;East_Han_AC=1;East_Han_AF
chr6	103205858 .	T	<INS:ME:LINE1>	.	PASS	SVTYPE=INS:ME:LINE1;END=103205877;SVLEN=5046;AN=20618;AC=1;AF=4.850130953535746e-05;N_HET=1;N_HOMALT=0;N_HOMREF=10308;FREQ_HET=9.700261907071492e-05;FREQ_HOMALT=0.0;FREQ_HOMREF=0.9999029973809292;N_BI_GENOS=10309;Southeast_Han_AN=1908;Southeast_Han_AC=1;S
chr6	103210788 .	A	<INS:ME:LINE1>	.	PASS	SVTYPE=INS:ME:LINE1;END=103210804;SVLEN=6007;AN=20618;AC=3;AF=0.00014550392860607236;N_HET=1;N_HOMALT=1;N_HOMREF=10307;FREQ_HET=9.700261907071492e-05;FREQ_HOMALT=9.700261907071492e-05;FREQ_HOMREF=0.9998059947618586;N_BI_GENOS=10309;South_Han_AN=1496;South
chr6	103333244 .	G	<INS:ME:LINE1>	.	PASS	SVTYPE=INS:ME:LINE1;END=103333262;SVLEN=1707;AN=20618;AC=1;AF=4.850130953535746e-05;N_HET=1;N_HOMALT=0;N_HOMREF=10308;FREQ_HET=9.700261907071492e-05;FREQ_HOMALT=0.0;FREQ_HOMREF=0.9999029973809292;N_BI_GENOS=10309;Southeast_Han_AN=1908;Southeast_Han_AC=1;S
chr6	103421213 .	A	<INS:ME:LINE1>	.	PASS	SVTYPE=INS:ME:LINE1;END=103421225;SVLEN=1530;AN=20618;AC=3;AF=0.00014550392860607236;N_HET=3;N_HOMALT=0;N_HOMREF=10306;FREQ_HET=0.0002910078572121447;FREQ_HOMALT=0.0;FREQ_HOMREF=0.9997089921427879;N_BI_GENOS=10309;Lingnan_Han_AN=1888;Lingnan_Han_AC=1;Ling
chr6	103698935 .	T	<INS:ME:LINE1>	.	PASS	SVTYPE=INS:ME:LINE1;END=103698951;SVLEN=6004;AN=20618;AC=1;AF=4.850130953535746e-05;N_HET=1;N_HOMALT=0;N_HOMREF=10308;FREQ_HET=9.700261907071492e-05;FREQ_HOMALT=0.0;FREQ_HOMREF=0.9999029973809292;N_BI_GENOS=10309;Southeast_Han_AN=1908;Southeast_Han_AC=1;S
chr6	104015753 .	A	<INS:ME:LINE1>	.	PASS	SVTYPE=INS:ME:LINE1;END=104015768;SVLEN=1028;AN=20618;AC=2;AF=9.700261907071492e-05;N_HET=2;N_HOMALT=0;N_HOMREF=10307;FREQ_HET=0.00019400523814142983;FREQ_HOMALT=0.0;FREQ_HOMREF=0.9998059947618586;N_BI_GENOS=10309;East_Han_AN=6554;East_Han_AC=1;East_Han_A
chr6	104248447 .	A	<INS:ME:LINE1>	.	PASS	SVTYPE=INS:ME:LINE1;END=104248462;SVLEN=695;AN=20618;AC=30;AF=0.0014550392860607237;N_HET=28;N_HOMALT=1;N_HOMREF=10280;FREQ_HET=0.0027160733339800175;FREQ_HOMALT=9.700261907071492e-05;FREQ_HOMREF=0.9971869240469493;N_BI_GENOS=10309;South_Han_AN=1496;South
chr6	104386789 .	G	<INS:ME:LINE1>	.	PASS	SVTYPE=INS:ME:LINE1;END=104386805;SVLEN=924;AN=20618;AC=1;AF=4.850130953535746e-05;N_HET=1;N_HOMALT=0;N_HOMREF=10308;FREQ_HET=9.700261907071492e-05;FREQ_HOMALT=0.0;FREQ_HOMREF=0.9999029973809292;N_BI_GENOS=10309;East_Han_AN=6554;East_Han_AC=1;East_Han_AF=
chr6	104792462 .	A	<INS:ME:LINE1>	.	PASS	SVTYPE=INS:ME:LINE1;END=104792476;SVLEN=5998;AN=20618;AC=2;AF=9.700261907071492e-05;N_HET=2;N_HOMALT=0;N_HOMREF=10307;FREQ_HET=0.00019400523814142983;FREQ_HOMALT=0.0;FREQ_HOMREF=0.9998059947618586;N_BI_GENOS=10309;Lingnan_Han_AN=1888;Lingnan_Han_AC=1;Ling
chr6	104823558 .	G	<INS:ME:LINE1>	.	PASS	SVTYPE=INS:ME:LINE1;END=104823580;SVLEN=5997;AN=20618;AC=56;AF=0.0027160733339800175;N_HET=56;N_HOMALT=0;N_HOMREF=10253;FREQ_HET=0.005432146667960035;FREQ_HOMALT=0.0;FREQ_HOMREF=0.99456785333204;N_BI_GENOS=10309;South_Han_AN=1496;South_Han_AC=7;South_Han_
chr6	105279377 .	A	<INS:ME:LINE1>	.	PASS	SVTYPE=INS:ME:LINE1;END=105279391;SVLEN=1549;AN=20618;AC=2;AF=9.700261907071492e-05;N_HET=2;N_HOMALT=0;N_HOMREF=10307;FREQ_HET=0.00019400523814142983;FREQ_HOMALT=0.0;FREQ_HOMREF=0.9998059947618586;N_BI_GENOS=10309;East_Han_AN=6554;East_Han_AC=1;East_Han_A
chr6	105506990 .	A	<INS:ME:LINE1>	.	PASS	SVTYPE=INS:ME:LINE1;END=105507005;SVLEN=6001;AN=20618;AC=2;AF=9.700261907071492e-05;N_HET=2;N_HOMALT=0;N_HOMREF=10307;FREQ_HET=0.00019400523814142983;FREQ_HOMALT=0.0;FREQ_HOMREF=0.9998059947618586;N_BI_GENOS=10309;South_Han_AN=1496;South_Han_AC=2;South_Ha
chr6	105654544 .	C	<INS:ME:LINE1>	.	PASS	SVTYPE=INS:ME:LINE1;END=105654557;SVLEN=6000;AN=20618;AC=2;AF=9.700261907071492e-05;N_HET=2;N_HOMALT=0;N_HOMREF=10307;FREQ_HET=0.00019400523814142983;FREQ_HOMALT=0.0;FREQ_HOMREF=0.9998059947618586;N_BI_GENOS=10309;East_Han_AN=6554;East_Han_AC=2;East_Han_A
chr6	105958426 .	A	<INS:ME:LINE1>	.	PASS	SVTYPE=INS:ME:LINE1;END=105958438;SVLEN=205;AN=20618;AC=3;AF=0.00014550392860607236;N_HET=3;N_HOMALT=0;N_HOMREF=10306;FREQ_HET=0.0002910078572121447;FREQ_HOMALT=0.0;FREQ_HOMREF=0.9997089921427879;N_BI_GENOS=10309;Central_Han_AN=2128;Central_Han_AC=1;Centr
chr6	106217120 .	A	<INS:ME:LINE1>	.	PASS	SVTYPE=INS:ME:LINE1;END=106217135;SVLEN=6008;AN=20618;AC=1;AF=4.850130953535746e-05;N_HET=1;N_HOMALT=0;N_HOMREF=10308;FREQ_HET=9.700261907071492e-05;FREQ_HOMALT=0.0;FREQ_HOMREF=0.9999029973809292;N_BI_GENOS=10309;East_Han_AN=6554;East_Han_AC=1;East_Han_AF
chr6	106413990 .	A	<INS:ME:LINE1>	.	PASS	SVTYPE=INS:ME:LINE1;END=106413998;SVLEN=1049;AN=20618;AC=1;AF=4.850130953535746e-05;N_HET=1;N_HOMALT=0;N_HOMREF=10308;FREQ_HET=9.700261907071492e-05;FREQ_HOMALT=0.0;FREQ_HOMREF=0.9999029973809292;N_BI_GENOS=10309;East_Han_AN=6554;East_Han_AC=1;East_Han_AF
chr6	106428394 .	T	<INS:ME:LINE1>	.	PASS	SVTYPE=INS:ME:LINE1;END=106428394;SVLEN=1885;AN=20618;AC=1;AF=4.850130953535746e-05;N_HET=1;N_HOMALT=0;N_HOMREF=10308;FREQ_HET=9.700261907071492e-05;FREQ_HOMALT=0.0;FREQ_HOMREF=0.9999029973809292;N_BI_GENOS=10309;Central_Han_AN=2128;Central_Han_AC=1;Centr
chr6	107486927 .	A	<INS:ME:LINE1>	.	PASS	SVTYPE=INS:ME:LINE1;END=107486941;SVLEN=358;AN=20618;AC=1;AF=4.850130953535746e-05;N_HET=1;N_HOMALT=0;N_HOMREF=10308;FREQ_HET=9.700261907071492e-05;FREQ_HOMALT=0.0;FREQ_HOMREF=0.9999029973809292;N_BI_GENOS=10309;Yi_AN=422;Yi_AC=1;Yi_AF=0.002369668246445 49
chr6	108465709 .	T	<INS:ME:LINE1>	.	PASS	SVTYPE=INS:ME:LINE1;END=108465725;SVLEN=990;AN=20618;AC=1;AF=4.850130953535746e-05;N_HET=1;N_HOMALT=0;N_HOMREF=10308;FREQ_HET=9.700261907071492e-05;FREQ_HOMALT=0.0;FREQ_HOMREF=0.9999029973809292;N_BI_GENOS=10309;South_Han_AN=1496;South_Han_AC=1;South_Han_
chr6	109571614 .	A	<INS:ME:LINE1>	.	PASS	SVTYPE=INS:ME:LINE1;END=109571631;SVLEN=247;AN=20618;AC=1;AF=4.850130953535746e-05;N_HET=1;N_HOMALT=0;N_HOMREF=10308;FREQ_HET=9.700261907071492e-05;FREQ_HOMALT=0.0;FREQ_HOMREF=0.9999029973809292;N_BI_GENOS=10309;Central_Han_AN=2128;Central_Han_AC=1;Centra
chr6	109590116 .	T	<INS:ME:LINE1>	.	PASS	SVTYPE=INS:ME:LINE1;END=109590129;SVLEN=1332;AN=20618;AC=1;AF=4.850130953535746e-05;N_HET=1;N_HOMALT=0;N_HOMREF=10308;FREQ_HET=9.700261907071492e-05;FREQ_HOMALT=0.0;FREQ_HOMREF=0.9999029973809292;N_BI_GENOS=10309;East_Han_AN=6554;East_Han_AC=1;East_Han_AF
chr6	109639024 .	C	<INS:ME:LINE1>	.	PASS	SVTYPE=INS:ME:LINE1;END=109639036;SVLEN=6015;AN=20618;AC=1;AF=4.850130953535746e-05;N_HET=1;N_HOMALT=0;N_HOMREF=10308;FREQ_HET=9.700261907071492e-05;FREQ_HOMALT=0.0;FREQ_HOMREF=0.9999029973809292;N_BI_GENOS=10309;East_Han_AN=6554;East_Han_AC=1;East_Han_AF
chr6	109848164 .	A	<INS:ME:LINE1>	.	PASS	SVTYPE=INS:ME:LINE1;END=109848164;SVLEN=6004;AN=20618;AC=1;AF=4.850130953535746e-05;N_HET=1;N_HOMALT=0;N_HOMREF=10308;FREQ_HET=9.700261907071492e-05;FREQ_HOMALT=0.0;FREQ_HOMREF=0.9999029973809292;N_BI_GENOS=10309;North_Han_AN=2272;North_Han_AC=1;North_Han
chr6	109870103 .	C	<INS:ME:LINE1>	.	PASS	SVTYPE=INS:ME:LINE1;END=109870112;SVLEN=5998;AN=20618;AC=1;AF=4.850130953535746e-05;N_HET=1;N_HOMALT=0;N_HOMREF=10308;FREQ_HET=9.700261907071492e-05;FREQ_HOMALT=0.0;FREQ_HOMREF=0.9999029973809292;N_BI_GENOS=10309;Miao_AN=482;Miao_AC=1;Miao_AF=0.0020746887
chr6	109918321 .	A	<INS:ME:LINE1>	.	PASS	SVTYPE=INS:ME:LINE1;END=109918334;SVLEN=2529;AN=20618;AC=1;AF=4.850130953535746e-05;N_HET=1;N_HOMALT=0;N_HOMREF=10308;FREQ_HET=9.700261907071492e-05;FREQ_HOMALT=0.0;FREQ_HOMREF=0.9999029973809292;N_BI_GENOS=10309;Central_Han_AN=2128;Central_Han_AC=1;Centr

chr6	110188362	.	A	<INS:ME:LINE1>	.	PASS	SVTYPE=INS:ME:LINE1;END=110188374;SVLEN=5991;AN=20618;AC=2;AF=9.700261907071492e-05;N_HET=2;N_HOMALT=0;N_HOMREF=10307;FREQ_HET=0.00019400523814142983;FREQ_HOMALT=0.0;FREQ_HOMREF=0.9998059947618586;N_BI_GENOS=10309;South_Han_AN=1496;South_Han_AC=1;South_Ha
chr6	110283018	.	A	<INS:ME:LINE1>	.	PASS	SVTYPE=INS:ME:LINE1;END=110283033;SVLEN=512;AN=20618;AC=1;AF=4.850130953535746e-05;N_HET=1;N_HOMALT=0;N_HOMREF=10308;FREQ_HET=9.700261907071492e-05;FREQ_HOMALT=0.0;FREQ_HOMREF=0.9999029973809292;N_BI_GENOS=10309;Tibetan_AN=414;Tibetan_AC=1;Tibetan_AF=0.00
chr6	110292369	.	A	<INS:ME:LINE1>	.	PASS	SVTYPE=INS:ME:LINE1;END=110292384;SVLEN=2837;AN=20618;AC=1;AF=4.850130953535746e-05;N_HET=1;N_HOMALT=0;N_HOMREF=10308;FREQ_HET=9.700261907071492e-05;FREQ_HOMALT=0.0;FREQ_HOMREF=0.9999029973809292;N_BI_GENOS=10309;North_Han_AN=2272;North_Han_AC=1;North_Han
chr6	110658862	.	A	<INS:ME:LINE1>	.	PASS	SVTYPE=INS:ME:LINE1;END=110658879;SVLEN=167;AN=20618;AC=4;AF=0.00019400523814142983;N_HET=4;N_HOMALT=0;N_HOMREF=10305;FREQ_HET=0.0003880104762828596 6;FREQ_HOMALT=0.0;FREQ_HOMREF=0.9996119895237171;N_BI_GENOS=10309;North_Han_AN=2272;North_Han_AC=1;North_Ha
chr6	110956995	.	A	<INS:ME:LINE1>	.	PASS	SVTYPE=INS:ME:LINE1;END=110956995;SVLEN=0;AN=20618;AC=10;AF=0.0004850130953535 7455;N_HET=0;N_HOMALT=5;N_HOMREF=10304;FREQ_HET=0.0;FREQ_HOMALT=0.00048501309535357455;FREQ_HOMREF=0.9995149869046465;N_BI_GENOS=10309;South_Han_AN=1496;South_Han_AC=6;South_Han
chr6	111948265	.	A	<INS:ME:LINE1>	.	PASS	SVTYPE=INS:ME:LINE1;END=111948281;SVLEN=1691;AN=20618;AC=1;AF=4.850130953535746e-05;N_HET=1;N_HOMALT=0;N_HOMREF=10308;FREQ_HET=9.700261907071492e-05;FREQ_HOMALT=0.0;FREQ_HOMREF=0.9999029973809292;N_BI_GENOS=10309;Central_Han_AN=2128;Central_Han_AC=1;Centr
chr6	111983960	.	A	<INS:ME:LINE1>	.	PASS	SVTYPE=INS:ME:LINE1;END=111983970;SVLEN=6008;AN=20618;AC=1;AF=4.850130953535746e-05;N_HET=1;N_HOMALT=0;N_HOMREF=10308;FREQ_HET=9.700261907071492e-05;FREQ_HOMALT=0.0;FREQ_HOMREF=0.9999029973809292;N_BI_GENOS=10309;Southeast_Han_AN=1908;Southeast_Han_AC=1;S
chr6	112363951	.	A	<INS:ME:LINE1>	.	PASS	SVTYPE=INS:ME:LINE1;END=112363968;SVLEN=5997;AN=20618;AC=4;AF=0.00019400523814142983;N_HET=4;N_HOMALT=0;N_HOMREF=10305;FREQ_HET=0.00038801047628285966;FREQ_HOMALT=0.0;FREQ_HOMREF=0.9996119895237171;N_BI_GENOS=10309;Northwest_Han_AN=1296;Northwest_Han_AC=2
chr6	112661694	.	T	<INS:ME:LINE1>	.	PASS	SVTYPE=INS:ME:LINE1;END=112661694;SVLEN=5783;AN=20618;AC=1;AF=4.850130953535746e-05;N_HET=1;N_HOMALT=0;N_HOMREF=10308;FREQ_HET=9.700261907071492e-05;FREQ_HOMALT=0.0;FREQ_HOMREF=0.9999029973809292;N_BI_GENOS=10309;Southeast_Han_AN=1908;Southeast_Han_AC=1;S
chr6	112680007	.	A	<INS:ME:LINE1>	.	PASS	SVTYPE=INS:ME:LINE1;END=112680018;SVLEN=2113;AN=20618;AC=1;AF=4.850130953535746e-05;N_HET=1;N_HOMALT=0;N_HOMREF=10308;FREQ_HET=9.700261907071492e-05;FREQ_HOMALT=0.0;FREQ_HOMREF=0.9999029973809292;N_BI_GENOS=10309;East_Han_AN=6554;East_Han_AC=1;East_Han_AF
chr6	113364873	.	A	<INS:ME:LINE1>	.	PASS	SVTYPE=INS:ME:LINE1;END=113364879;SVLEN=984;AN=20618;AC=18;AF=0.0008730235716364342;N_HET=16;N_HOMALT=1;N_HOMREF=10292;FREQ_HET=0.0015520419051314386;FREQ_HOMALT=9.700261907071492e-05;FREQ_HOMREF=0.9983509554757979;N_BI_GENOS=10309;South_Han_AN=1496;South
chr6	113412208	.	G	<INS:ME:LINE1>	.	PASS	SVTYPE=INS:ME:LINE1;END=113412221;SVLEN=5998;AN=20618;AC=3;AF=0.00014550392860607236;N_HET=3;N_HOMALT=0;N_HOMREF=10306;FREQ_HET=0.0002910078572121447;FREQ_HOMALT=0.0;FREQ_HOMREF=0.9997089921427879;N_BI_GENOS=10309;South_Han_AN=1496;South_Han_AC=1;South_Ha
chr6	113538099	.	A	<INS:ME:LINE1>	.	PASS	SVTYPE=INS:ME:LINE1;END=113538116;SVLEN=726;AN=20618;AC=1;AF=4.850130953535746e-05;N_HET=1;N_HOMALT=0;N_HOMREF=10308;FREQ_HET=9.700261907071492e-05;FREQ_HOMALT=0.0;FREQ_HOMREF=0.9999029973809292;N_BI_GENOS=10309;South_Han_AN=1496;South_Han_AC=1;South_Han_
chr6	113649296	.	T	<INS:ME:LINE1>	.	PASS	SVTYPE=INS:ME:LINE1;END=113649309;SVLEN=2112;AN=20618;AC=2;AF=9.700261907071492e-05;N_HET=2;N_HOMALT=0;N_HOMREF=10307;FREQ_HET=0.00019400523814142983;FREQ_HOMALT=0.0;FREQ_HOMREF=0.9998059947618586;N_BI_GENOS=10309;East_Han_AN=6554;East_Han_AC=1;East_Han_A
chr6	113694478	.	T	<INS:ME:LINE1>	.	PASS	SVTYPE=INS:ME:LINE1;END=113694490;SVLEN=1556;AN=20618;AC=1;AF=4.850130953535746e-05;N_HET=1;N_HOMALT=0;N_HOMREF=10308;FREQ_HET=9.700261907071492e-05;FREQ_HOMALT=0.0;FREQ_HOMREF=0.9999029973809292;N_BI_GENOS=10309;Northwest_Han_AN=1296;Northwest_Han_AC=1;N
chr6	113770014	.	T	<INS:ME:LINE1>	.	PASS	SVTYPE=INS:ME:LINE1;END=113770014;SVLEN=2113;AN=20618;AC=1868;AF=0.09060044621204773;N_HET=1756;N_HOMALT=56;N_HOMREF=8497;FREQ_HET=0.1703365990881754;FREQ_HOMALT=0.005432146667960035;FREQ_HOMREF=0.8242312542438646;N_BI_GENOS=10309;South_Han_AN=1496;South_
chr6	113909379	.	A	<INS:ME:LINE1>	.	PASS	SVTYPE=INS:ME:LINE1;END=113909392;SVLEN=1203;AN=20618;AC=1;AF=4.850130953535746e-05;N_HET=1;N_HOMALT=0;N_HOMREF=10308;FREQ_HET=9.700261907071492e-05;FREQ_HOMALT=0.0;FREQ_HOMREF=0.9999029973809292;N_BI_GENOS=10309;Zhuang_AN=444;Zhuang_AC=1;Zhuang_AF=0.0022
chr6	114206662	.	A	<INS:ME:LINE1>	.	PASS	SVTYPE=INS:ME:LINE1;END=114206676;SVLEN=4517;AN=20618;AC=1;AF=4.850130953535746e-05;N_HET=1;N_HOMALT=0;N_HOMREF=10308;FREQ_HET=9.700261907071492e-05;FREQ_HOMALT=0.0;FREQ_HOMREF=0.9999029973809292;N_BI_GENOS=10309;East_Han_AN=6554;East_Han_AC=1;East_Han_AF
chr6	114693243	.	G	<INS:ME:LINE1>	.	PASS	SVTYPE=INS:ME:LINE1;END=114693252;SVLEN=6000;AN=20618;AC=478;AF=0.023183625957900862;N_HET=454;N_HOMALT=12;N_HOMREF=9843;FREQ_HET=0.04403918905810457;FREQ_HOMALT=0.0011640314288485789;FREQ_HOMREF=0.9547967795130469;N_BI_GENOS=10309;South_Han_AN=1496;South
chr6	114777543	.	G	<INS:ME:LINE1>	.	PASS	SVTYPE=INS:ME:LINE1;END=114777556;SVLEN=5989;AN=20618;AC=1;AF=4.850130953535746e-05;N_HET=1;N_HOMALT=0;N_HOMREF=10308;FREQ_HET=9.700261907071492e-05;FREQ_HOMALT=0.0;FREQ_HOMREF=0.9999029973809292;N_BI_GENOS=10309;Lingnan_Han_AN=1888;Lingnan_Han_AC=1;Lingn
chr6	114785591	.	C	<INS:ME:LINE1>	.	PASS	SVTYPE=INS:ME:LINE1;END=114785607;SVLEN=464;AN=20618;AC=1;AF=4.850130953535746e-05;N_HET=1;N_HOMALT=0;N_HOMREF=10308;FREQ_HET=9.700261907071492e-05;FREQ_HOMALT=0.0;FREQ_HOMREF=0.9999029973809292;N_BI_GENOS=10309;Mongolian_AN=454;Mongolian_AC=1;Mongolian_A
chr6	114789171	.	A	<INS:ME:LINE1>	.	PASS	SVTYPE=INS:ME:LINE1;END=114789180;SVLEN=310;AN=20618;AC=12;AF=0.0005820157144242894;N_HET=12;N_HOMALT=0;N_HOMREF=10297;FREQ_HET=0.0011640314288485789;FREQ_HOMALT=0.0;FREQ_HOMREF=0.9988359685711514;N_BI_GENOS=10309;South_Han_AN=1496;South_Han_AC=1;South_Ha
chr6	114963061	.	A	<INS:ME:LINE1>	.	PASS	SVTYPE=INS:ME:LINE1;END=114963072;SVLEN=654;AN=20618;AC=1;AF=4.850130953535746e-05;N_HET=1;N_HOMALT=0;N_HOMREF=10308;FREQ_HET=9.700261907071492e-05;FREQ_HOMALT=0.0;FREQ_HOMREF=0.9999029973809292;N_BI_GENOS=10309;North_Han_AN=2272;North_Han_AC=1;North_Han_
chr6	115029558	.	G	<INS:ME:LINE1>	.	PASS	SVTYPE=INS:ME:LINE1;END=115029574;SVLEN=1592;AN=20618;AC=7;AF=0.0003395091667475022;N_HET=7;N_HOMALT=0;N_HOMREF=10302;FREQ_HET=0.0006790183334950044;FREQ_HOMALT=0.0;FREQ_HOMREF=0.999320981666505;N_BI_GENOS=10309;Northwest_Han_AN=1296;Northwest_Han_AC=1;No
chr6	115258112	.	T	<INS:ME:LINE1>	.	PASS	SVTYPE=INS:ME:LINE1;END=115258148;SVLEN=1213;AN=20618;AC=1;AF=4.850130953535746e-05;N_HET=1;N_HOMALT=0;N_HOMREF=10308;FREQ_HET=9.700261907071492e-05;FREQ_HOMALT=0.0;FREQ_HOMREF=0.9999029973809292;N_BI_GENOS=10309;East_Han_AN=6554;East_Han_AC=1;East_Han_AF
chr6	115583443	.	T	<INS:ME:LINE1>	.	PASS	SVTYPE=INS:ME:LINE1;END=115583443;SVLEN=679;AN=20618;AC=2;AF=9.700261907071492e-05;N_HET=2;N_HOMALT=0;N_HOMREF=10307;FREQ_HET=0.00019400523814142983;FREQ_HOMALT=0.0;FREQ_HOMREF=0.9998059947618586;N_BI_GENOS=10309;East_Han_AN=6554;East_Han_AC=2;East_Han_AF
chr6	115885315	.	A	<INS:ME:LINE1>	.	PASS	SVTYPE=INS:ME:LINE1;END=115885329;SVLEN=1139;AN=20618;AC=2;AF=9.700261907071492e-05;N_HET=2;N_HOMALT=0;N_HOMREF=10307;FREQ_HET=0.00019400523814142983;FREQ_HOMALT=0.0;FREQ_HOMREF=0.9998059947618586;N_BI_GENOS=10309;Central_Han_AN=2128;Central_Han_AC=1;Cent
chr6	116196309	.	T	<INS:ME:LINE1>	.	PASS	SVTYPE=INS:ME:LINE1;END=116196329;SVLEN=353;AN=20618;AC=2;AF=9.700261907071492e-05;N_HET=2;N_HOMALT=0;N_HOMREF=10307;FREQ_HET=0.00019400523814142983;FREQ_HOMALT=0.0;FREQ_HOMREF=0.9998059947618586;N_BI_GENOS=10309;Central_Han_AN=2128;Central_Han_AC=1;Centr
chr6	116212600	.	A	<INS:ME:LINE1>	.	PASS	SVTYPE=INS:ME:LINE1;END=116212615;SVLEN=762;AN=20618;AC=1;AF=4.850130953535746e-05;N_HET=1;N_HOMALT=0;N_HOMREF=10308;FREQ_HET=9.700261907071492e-05;FREQ_HOMALT=0.0;FREQ_HOMREF=0.9999029973809292;N_BI_GENOS=10309;Lingnan_Han_AN=1888;Lingnan_Han_AC=1;Lingna
chr6	116976055	.	T	<INS:ME:LINE1>	.	PASS	SVTYPE=INS:ME:LINE1;END=116976070;SVLEN=465;AN=20618;AC=18;AF=0.0008730235716364342;N_HET=12;N_HOMALT=3;N_HOMREF=10294;FREQ_HET=0.0011640314288485789;FREQ_HOMALT=0.0002910078572121447;FREQ_HOMREF=0.9985449607139393;N_BI_GENOS=10309;Northwest_Han_AN=1296;N
chr6	116977153	.	C	<INS:ME:LINE1>	.	PASS	SVTYPE=INS:ME:LINE1;END=116977166;SVLEN=1741;AN=20618;AC=2;AF=9.700261907071492e-05;N_HET=2;N_HOMALT=0;N_HOMREF=10307;FREQ_HET=0.00019400523814142983;FREQ_HOMALT=0.0;FREQ_HOMREF=0.9998059947618586;N_BI_GENOS=10309;Lingnan_Han_AN=1888;Lingnan_Han_AC=1;Ling
chr6	117074320	.	A	<INS:ME:LINE1>	.	PASS	SVTYPE=INS:ME:LINE1;END=117074337;SVLEN=5998;AN=20618;AC=606;AF=0.02939179357842662;N_HET=554;N_HOMALT=26;N_HOMREF=9729;FREQ_HET=0.05373945096517606;FREQ_HOMALT=0.0025220680958385876;FREQ_HOMREF=0.9437384809389854;N_BI_GENOS=10309;South_Han_AN=1496;South_
chr6	117234545	.	A	<INS:ME:LINE1>	.	PASS	SVTYPE=INS:ME:LINE1;END=117234547;SVLEN=1099;AN=20618;AC=2;AF=9.700261907071492e-05;N_HET=0;N_HOMALT=1;N_HOMREF=10308;FREQ_HET=0.0;FREQ_HOMALT=9.700261907071492e-05;FREQ_HOMREF=0.9999029973809292;N_BI_GENOS=10309;Southeast_Han_AN=1908;Southeast_Han_AC=2;S
chr6	117425786	.	A	<INS:ME:LINE1>	.	PASS	SVTYPE=INS:ME:LINE1;END=117425797;SVLEN=2056;AN=20618;AC=1;AF=4.850130953535746e-05;N_HET=1;N_HOMALT=0;N_HOMREF=10308;FREQ_HET=9.700261907071492e-05;FREQ_HOMALT=0.0;FREQ_HOMREF=0.9999029973809292;N_BI_GENOS=10309;North_Han_AN=2272;North_Han_AC=1;North_Han
chr6	117834459	.	A	<INS:ME:LINE1>	.	PASS	SVTYPE=INS:ME:LINE1;END=117834472;SVLEN=379;AN=20618;AC=1;AF=4.850130953535746e-05;N_HET=1;N_HOMALT=0;N_HOMREF=10308;FREQ_HET=9.700261907071492e-05;FREQ_HOMALT=0.0;FREQ_HOMREF=0.9999029973809292;N_BI_GENOS=10309;South_Han_AN=1496;South_Han_AC=1;South_Han_
chr6	118040462	.	G	<INS:ME:LINE1>	.	PASS	SVTYPE=INS:ME:LINE1;END=118040475;SVLEN=229;AN=20618;AC=3;AF=0.00014550392860607236;N_HET=3;N_HOMALT=0;N_HOMREF=10306;FREQ_HET=0.0002910078572121447;FREQ_HOMALT=0.0;FREQ_HOMREF=0.9997089921427879;N_BI_GENOS=10309;Lingnan_Han_AN=1888;Lingnan_Han_AC=1;Lingn
chr6	118123423	.	G	<INS:ME:LINE1>	.	PASS	SVTYPE=INS:ME:LINE1;END=118123423;SVLEN=544;AN=20618;AC=1;AF=4.850130953535746e-05;N_HET=1;N_HOMALT=0;N_HOMREF=10308;FREQ_HET=9.700261907071492e-05;FREQ_HOMALT=0.0;FREQ_HOMREF=0.9999029973809292;N_BI_GENOS=10309;South_Han_AN=1496;South_Han_AC=1;South_Han_
chr6	118181474	.	A	<INS:ME:LINE1>	.	PASS	SVTYPE=INS:ME:LINE1;END=118181474;SVLEN=912;AN=20618;AC=3;AF=0.00014550392860607236;N_HET=3;N_HOMALT=0;N_HOMREF=10306;FREQ_HET=0.0002910078572121447;FREQ_HOMALT=0.0;FREQ_HOMREF=0.9997089921427879;N_BI_GENOS=10309;Central_Han_AN=2128;Central_Han_AC=2;Centr
chr6	118259565	.	T	<INS:ME:LINE1>	.	PASS	SVTYPE=INS:ME:LINE1;END=118259581;SVLEN=6010;AN=20618;AC=1;AF=4.850130953535746e-05;N_HET=1;N_HOMALT=0;N_HOMREF=10308;FREQ_HET=9.700261907071492e-05;FREQ_HOMALT=0.0;FREQ_HOMREF=0.9999029973809292;N_BI_GENOS=10309;East_Han_AN=6554;East_Han_AC=1;East_Han_AF
chr6	118324362	.	G	<INS:ME:LINE1>	.	PASS	SVTYPE=INS:ME:LINE1;END=118324376;SVLEN=1527;AN=20618;AC=1;AF=4.850130953535746e-05;N_HET=1;N_HOMALT=0;N_HOMREF=10308;FREQ_HET=9.700261907071492e-05;FREQ_HOMALT=0.0;FREQ_HOMREF=0.9999029973809292;N_BI_GENOS=10309;East_Han_AN=6554;East_Han_AC=1;East_Han_AF
chr6	118448374	.	A	<INS:ME:LINE1>	.	PASS	SVTYPE=INS:ME:LINE1;END=118448392;SVLEN=3399;AN=20618;AC=1;AF=4.850130953535746e-05;N_HET=1;N_HOMALT=0;N_HOMREF=10308;FREQ_HET=9.700261907071492e-05;FREQ_HOMALT=0.0;FREQ_HOMREF=0.9999029973809292;N_BI_GENOS=10309;Zhuang_AN=444;Zhuang_AC=1;Zhuang_AF=0.0022
chr6	118558626	.	A	<INS:ME:LINE1>	.	PASS	SVTYPE=INS:ME:LINE1;END=118558626;SVLEN=0;AN=20618;AC=1;AF=4.850130953535746e-05;N_HET=1;N_HOMALT=0;N_HOMREF=10308;FREQ_HET=9.700261907071492e-05;FREQ_HOMALT=0.0;FREQ_HOMREF=0.9999029973809292;N_BI_GENOS=10309;East_Han_AN=6554;East_Han_AC=1;East_Han_AF=0.
chr6	119341246	.	C	<INS:ME:LINE1>	.	PASS	SVTYPE=INS:ME:LINE1;END=119341262;SVLEN=1491;AN=20618;AC=1;AF=4.850130953535746e-05;N_HET=1;N_HOMALT=0;N_HOMREF=10308;FREQ_HET=9.700261907071492e-05;FREQ_HOMALT=0.0;FREQ_HOMREF=0.9999029973809292;N_BI_GENOS=10309;East_Han_AN=6554;East_Han_AC=1;East_Han_AF
chr6	119677531	.	T	<INS:ME:LINE1>	.	PASS	SVTYPE=INS:ME:LINE1;END=119677537;SVLEN=4385;AN=20618;AC=1;AF=4.850130953535746e-05;N_HET=1;N_HOMALT=0;N_HOMREF=10308;FREQ_HET=9.700261907071492e-05;FREQ_HOMALT=0.0;FREQ_HOMREF=0.9999029973809292;N_BI_GENOS=10309;Northwest_Han_AN=1296;Northwest_Han_AC=1;N
chr6	119790553	.	G	<INS:ME:LINE1>	.	PASS	SVTYPE=INS:ME:LINE1;END=119790568;SVLEN=5999;AN=20618;AC=1;AF=4.850130953535746e-05;N_HET=1;N_HOMALT=0;N_HOMREF=10308;FREQ_HET=9.700261907071492e-05;FREQ_HOMALT=0.0;FREQ_HOMREF=0.9999029973809292;N_BI_GENOS=10309;Lingnan_Han_AN=1888;Lingnan_Han_AC=1;Lingn
chr6	119795952	.	C	<INS:ME:LINE1>	.	PASS	SVTYPE=INS:ME:LINE1;END=119795952;SVLEN=1385;AN=20618;AC=1;AF=4.850130953535746e-05;N_HET=1;N_HOMALT=0;N_HOMREF=10308;FREQ_HET=9.700261907071492e-05;FREQ_HOMALT=0.0;FREQ_HOMREF=0.9999029973809292;N_BI_GENOS=10309;East_Han_AN=6554;East_Han_AC=1;East_Han_AF
chr6	120022456	.	T	<INS:ME:LINE1>	.	PASS	SVTYPE=INS:ME:LINE1;END=120022471;SVLEN=6006;AN=20618;AC=2;AF=9.700261907071492e-05;N_HET=2;N_HOMALT=0;N_HOMREF=10307;FREQ_HET=0.00019400523814142983;FREQ_HOMALT=0.0;FREQ_HOMREF=0.9998059947618586;N_BI_GENOS=10309;Lingnan_Han_AN=1888;Lingnan_Han_AC=2;Ling
chr6	120107273	.	A	<INS:ME:LINE1>	.	PASS	SVTYPE=INS:ME:LINE1;END=120107287;SVLEN=684;AN=20618;AC=1;AF=4.850130953535746e-05;N_HET=1;N_HOMALT=0;N_HOMREF=10308;FREQ_HET=9.700261907071492e-05;FREQ_HOMALT=0.0;FREQ_HOMREF=0.9999029973809292;N_BI_GENOS=10309;Mongolian_AN=454;Mongolian_AC=1;Mongolian_A
chr6	120283378	.	G	<INS:ME:LINE1>	.	PASS	SVTYPE=INS:ME:LINE1;END=120283394;SVLEN=2415;AN=20618;AC=432;AF=0.02095256571927442;N_HET=418;N_HOMALT=7;N_HOMREF=9884;FREQ_HET=0.04054709477155883;FREQ_HOMALT=0.0006790183334950044;FREQ_HOMREF=0.9587738868949461;N_BI_GENOS=10309;South_Han_AN=1496;South_H
chr6	120466308	.	C	<INS:ME:LINE1>	.	PASS	SVTYPE=INS:ME:LINE1;END=120466308;SVLEN=0;AN=20618;AC=1;AF=4.850130953535746e-05;N_HET=1;N_HOMALT=0;N_HOMREF=10308;FREQ_HET=9.700261907071492e-05;FREQ_HOMALT=0.0;FREQ_HOMREF=0.9999029973809292;N_BI_GENOS=10309;Southeast_Han_AN=1908;Southeast_Han_AC=1;Sout
chr6	120486187	.	A	<INS:ME:LINE1>	.	PASS	SVTYPE=INS:ME:LINE1;END=120486204;SVLEN=883;AN=20618;AC=13;AF=0.0006305170239596469;N_HET=11;N_HOMALT=1;N_HOMREF=10297;FREQ_HET=0.001067028809777864;FREQ_HOMALT=9.700261907071492e-05;FREQ_HOMREF=0.9988359685711514;N_BI_GENOS=10309;South_Han_AN=1496;South_
chr6	120947529	.	A	<INS:ME:LINE1>	.	PASS	SVTYPE=INS:ME:LINE1;END=120947543;SVLEN=934;AN=20618;AC=1;AF=4.850130953535746e-05;N_HET=1;N_HOMALT=0;N_HOMREF=10308;FREQ_HET=9.700261907071492e-05;FREQ_HOMALT=0.0;FREQ_HOMREF=0.9999029973809292;N_BI_GENOS=10309;Manchu_AN=416;Manchu_AC=1;Manchu_AF=0.00240
chr6	120974622	.	T	<INS:ME:LINE1>	.	PASS	SVTYPE=INS:ME:LINE1;END=120974632;SVLEN=5999;AN=20618;AC=1;AF=4.850130953535746e-05;N_HET=1;N_HOMALT=0;N_HOMREF=10308;FREQ_HET=9.700261907071492e-05;FREQ_HOMALT=0.0;FREQ_HOMREF=0.9999029973809292;N_BI_GENOS=10309;East_Han_AN=6554;East_Han_AC=1;East_Han_AF
chr6	121107259	.	A	<INS:ME:LINE1>	.	PASS	SVTYPE=INS:ME:LINE1;END=121107275;SVLEN=1023;AN=20618;AC=1;AF=4.850130953535746e-05;N_HET=1;N_HOMALT=0;N_HOMREF=10308;FREQ_HET=9.700261907071492e-05;FREQ_HOMALT=0.0;FREQ_HOMREF=0.9999029973809292;N_BI_GENOS=10309;North_Han_AN=2272;North_Han_AC=1;North_Han
chr6	121331734	.	G	<INS:ME:LINE1>	.	PASS	SVTYPE=INS:ME:LINE1;END=121331750;SVLEN=5998;AN=20618;AC=1;AF=4.850130953535746e-05;N_HET=1;N_HOMALT=0;N_HOMREF=10308;FREQ_HET=9.700261907071492e-05;FREQ_HOMALT=0.0;FREQ_HOMREF=0.9999029973809292;N_BI_GENOS=10309;East_Han_AN=6554;East_Han_AC=1;East_Han_AF
chr6	121343177	.	C	<INS:ME:LINE1>	.	PASS	SVTYPE=INS:ME:LINE1;END=121343177;SVLEN=75;AN=20618;AC=1;AF=4.850130953535746e-05;N_HET=1;N_HOMALT=0;N_HOMREF=10308;FREQ_HET=9.700261907071492e-05;FREQ_HOMALT=0.0;FREQ_HOMREF=0.9999029973809292;N_BI_GENOS=10309;North_Han_AN=2272;North_Han_AC=1;North_Han_A
chr6	121350277	.	A	<INS:ME:LINE1>	.	PASS	SVTYPE=INS:ME:LINE1;END=121350292;SVLEN=4098;AN=20618;AC=1;AF=4.850130953535746e-05;N_HET=1;N_HOMALT=0;N_HOMREF=10308;FREQ_HET=9.700261907071492e-05;FREQ_HOMALT=0.0;FREQ_HOMREF=0.9999029973809292;N_BI_GENOS=10309;East_Han_AN=6554;East_Han_AC=1;East_Han_AF
chr6	121850721	.	G	<INS:ME:LINE1>	.	PASS	SVTYPE=INS:ME:LINE1;END=121850736;SVLEN=6007;AN=20618;AC=9;AF=0.0004365117858182171;N_HET=9;N_HOMALT=0;N_HOMREF=10300;FREQ_HET=0.0008730235716364342;FREQ_HOMALT=0.0;FREQ_HOMREF=0.9991269764283636;N_BI_GENOS=10309;South_Han_AN=1496;South_Han_AC=1;South_Han
chr6	121907670	.	T	<INS:ME:LINE1>	.	PASS	SVTYPE=INS:ME:LINE1;END=121907670;SVLEN=262;AN=20618;AC=1;AF=4.850130953535746e-05;N_HET=1;N_HOMALT=0;N_HOMREF=10308;FREQ_HET=9.700261907071492e-05;FREQ_HOMALT=0.0;FREQ_HOMREF=0.9999029973809292;N_BI_GENOS=10309;East_Han_AN=6554;East_Han_AC=1;East_Han_AF=
chr6	121961151	.	G	<INS:ME:LINE1>	.	PASS	SVTYPE=INS:ME:LINE1;END=121961167;SVLEN=124;AN=20618;AC=1;AF=4.850130953535746e-05;N_HET=1;N_HOMALT=0;N_HOMREF=10308;FREQ_HET=9.700261907071492e-05;FREQ_HOMALT=0.0;FREQ_HOMREF=0.9999029973809292;N_BI_GENOS=10309;Lingnan_Han_AN=1888;Lingnan_Han_AC=1;Lingna
chr6	122211504	.	A	<INS:ME:LINE1>	.	PASS	SVTYPE=INS:ME:LINE1;END=122211518;SVLEN=676;AN=20618;AC=1;AF=4.850130953535746e-05;N_HET=1;N_HOMALT=0;N_HOMREF=10308;FREQ_HET=9.700261907071492e-05;FREQ_HOMALT=0.0;FREQ_HOMREF=0.9999029973809292;N_BI_GENOS=10309;South_Han_AN=1496;South_Han_AC=1;South_Han_
chr6	122512754	.	A	<INS:ME:LINE1>	.	PASS	SVTYPE=INS:ME:LINE1;END=122512770;SVLEN=6000;AN=20618;AC=1;AF=4.850130953535746e-05;N_HET=1;N_HOMALT=0;N_HOMREF=10308;FREQ_HET=9.700261907071492e-05;FREQ_HOMALT=0.0;FREQ_HOMREF=0.9999029973809292;N_BI_GENOS=10309;Miao_AN=482;Miao_AC=1;Miao_AF=0.0020746887
chr6	122585863	.	T	<INS:ME:LINE1>	.	PASS	SVTYPE=INS:ME:LINE1;END=122585879;SVLEN=521;AN=20618;AC=1;AF=4.850130953535746e-05;N_HET=1;N_HOMALT=0;N_HOMREF=10308;FREQ_HET=9.700261907071492e-05;FREQ_HOMALT=0.0;FREQ_HOMREF=0.9999029973809292;N_BI_GENOS=10309;Central_Han_AN=2128;Central_Han_AC=1;Centra
chr6	122709500	.	A	<INS:ME:LINE1>	.	PASS	SVTYPE=INS:ME:LINE1;END=122709514;SVLEN=5997;AN=20618;AC=3;AF=0.00014550392860607236;N_HET=3;N_HOMALT=0;N_HOMREF=10306;FREQ_HET=0.0002910078572121447;FREQ_HOMALT=0.0;FREQ_HOMREF=0.9997089921427879;N_BI_GENOS=10309;South_Han_AN=1496;South_Han_AC=1;South_Ha
chr6	122905712	.	A	<INS:ME:LINE1>	.	PASS	SVTYPE=INS:ME:LINE1;END=122905729;SVLEN=5998;AN=20618;AC=2;AF=9.700261907071492e-05;N_HET=2;N_HOMALT=0;N_HOMREF=10307;FREQ_HET=0.00019400523814142983;FREQ_HOMALT=0.0;FREQ_HOMREF=0.9998059947618586;N_BI_GENOS=10309;South_Han_AN=1496;South_Han_AC=1;South_Ha
chr6	122975196	.	T	<INS:ME:LINE1>	.	PASS	SVTYPE=INS:ME:LINE1;END=122975196;SVLEN=657;AN=20618;AC=9;AF=0.0004365117858182171;N_HET=9;N_HOMALT=0;N_HOMREF=10300;FREQ_HET=0.0008730235716364342;FREQ_HOMALT=0.0;FREQ_HOMREF=0.9991269764283636;N_BI_GENOS=10309;Lingnan_Han_AN=1888;Lingnan_Han_AC=4;Lingna
chr6	122982848	.	T	<INS:ME:LINE1>	.	PASS	SVTYPE=INS:ME:LINE1;END=122982862;SVLEN=797;AN=20618;AC=1;AF=4.850130953535746e-05;N_HET=1;N_HOMALT=0;N_HOMREF=10308;FREQ_HET=9.700261907071492e-05;FREQ_HOMALT=0.0;FREQ_HOMREF=0.9999029973809292;N_BI_GENOS=10309;East_Han_AN=6554;East_Han_AC=1;East_Han_AF=
chr6	123105505	.	G	<INS:ME:LINE1>	.	PASS	SVTYPE=INS:ME:LINE1;END=123105524;SVLEN=1102;AN=20618;AC=1;AF=4.850130953535746e-05;N_HET=1;N_HOMALT=0;N_HOMREF=10308;FREQ_HET=9.700261907071492e-05;FREQ_HOMALT=0.0;FREQ_HOMREF=0.9999029973809292;N_BI_GENOS=10309;Manchu_AN=416;Manchu_AC=1;Manchu_AF=0.0024
chr6	123326482	.	T	<INS:ME:LINE1>	.	PASS	SVTYPE=INS:ME:LINE1;END=123326498;SVLEN=725;AN=20618;AC=1250;AF=0.06062663691919682;N_HET=1140;N_HOMALT=55;N_HOMREF=9114;FREQ_HET=0.110582985740615;FREQ_HOMALT=0.00533514404888932;FREQ_HOMREF=0.8840818702104957;N_BI_GENOS=10309;South_Han_AN=1496;South_Han
chr6	123340569	.	T	<INS:ME:LINE1>	.	PASS	SVTYPE=INS:ME:LINE1;END=123340583;SVLEN=1162;AN=20618;AC=2;AF=9.700261907071492e-05;N_HET=2;N_HOMALT=0;N_HOMREF=10307;FREQ_HET=0.00019400523814142983;FREQ_HOMALT=0.0;FREQ_HOMREF=0.9998059947618586;N_BI_GENOS=10309;East_Han_AN=6554;East_Han_AC=2;East_Han_A
chr6	123343647	.	C	<INS:ME:LINE1>	.	PASS	SVTYPE=INS:ME:LINE1;END=123343647;SVLEN=210;AN=20618;AC=1;AF=4.850130953535746e-05;N_HET=1;N_HOMALT=0;N_HOMREF=10308;FREQ_HET=9.700261907071492e-05;FREQ_HOMALT=0.0;FREQ_HOMREF=0.9999029973809292;N_BI_GENOS=10309;East_Han_AN=6554;East_Han_AC=1;East_Han_AF=
chr6	123532792	.	G	<INS:ME:LINE1>	.	PASS	SVTYPE=INS:ME:LINE1;END=123532801;SVLEN=5999;AN=20618;AC=411;AF=0.019934038219031915;N_HET=393;N_HOMALT=9;N_HOMREF=9907;FREQ_HET=0.03812202929479096;FREQ_HOMALT=0.0008730235716364342;FREQ_HOMREF=0.9610049471335727;N_BI_GENOS=10309;South_Han_AN=1496;South_
chr6	123563238	.	A	<INS:ME:LINE1>	.	PASS	SVTYPE=INS:ME:LINE1;END=123563253;SVLEN=697;AN=20618;AC=1;AF=4.850130953535746e-05;N_HET=1;N_HOMALT=0;N_HOMREF=10308;FREQ_HET=9.700261907071492e-05;FREQ_HOMALT=0.0;FREQ_HOMREF=0.9999029973809292;N_BI_GENOS=10309;East_Han_AN=6554;East_Han_AC=1;East_Han_AF=
chr6	123635885	.	T	<INS:ME:LINE1>	.	PASS	SVTYPE=INS:ME:LINE1;END=123635903;SVLEN=3012;AN=20618;AC=1;AF=4.850130953535746e-05;N_HET=1;N_HOMALT=0;N_HOMREF=10308;FREQ_HET=9.700261907071492e-05;FREQ_HOMALT=0.0;FREQ_HOMREF=0.9999029973809292;N_BI_GENOS=10309;Southeast_Han_AN=1908;Southeast_Han_AC=1;S
chr6	123889036	.	G	<INS:ME:LINE1>	.	PASS	SVTYPE=INS:ME:LINE1;END=123889051;SVLEN=1499;AN=20618;AC=2;AF=9.700261907071492e-05;N_HET=2;N_HOMALT=0;N_HOMREF=10307;FREQ_HET=0.00019400523814142983;FREQ_HOMALT=0.0;FREQ_HOMREF=0.9998059947618586;N_BI_GENOS=10309;East_Han_AN=6554;East_Han_AC=1;East_Han_A
chr6	123944694	.	T	<INS:ME:LINE1>	.	PASS	SVTYPE=INS:ME:LINE1;END=123944704;SVLEN=409;AN=20618;AC=1;AF=4.850130953535746e-05;N_HET=1;N_HOMALT=0;N_HOMREF=10308;FREQ_HET=9.700261907071492e-05;FREQ_HOMALT=0.0;FREQ_HOMREF=0.9999029973809292;N_BI_GENOS=10309;East_Han_AN=6554;East_Han_AC=1;East_Han_AF=
chr6	123998480	.	G	<INS:ME:LINE1>	.	PASS	SVTYPE=INS:ME:LINE1;END=123998488;SVLEN=1793;AN=20618;AC=1;AF=4.850130953535746e-05;N_HET=1;N_HOMALT=0;N_HOMREF=10308;FREQ_HET=9.700261907071492e-05;FREQ_HOMALT=0.0;FREQ_HOMREF=0.9999029973809292;N_BI_GENOS=10309;Central_Han_AN=2128;Central_Han_AC=1;Centr
chr6	124206731	.	T	<INS:ME:LINE1>	.	PASS	SVTYPE=INS:ME:LINE1;END=124206734;SVLEN=399;AN=20618;AC=1;AF=4.850130953535746e-05;N_HET=1;N_HOMALT=0;N_HOMREF=10308;FREQ_HET=9.700261907071492e-05;FREQ_HOMALT=0.0;FREQ_HOMREF=0.9999029973809292;N_BI_GENOS=10309;Miao_AN=482;Miao_AC=1;Miao_AF=0.00207468879
chr6	124289641	.	T	<INS:ME:LINE1>	.	PASS	SVTYPE=INS:ME:LINE1;END=124289649;SVLEN=344;AN=20618;AC=1;AF=4.850130953535746e-05;N_HET=1;N_HOMALT=0;N_HOMREF=10308;FREQ_HET=9.700261907071492e-05;FREQ_HOMALT=0.0;FREQ_HOMREF=0.9999029973809292;N_BI_GENOS=10309;South_Han_AN=1496;South_Han_AC=1;South_Han_
chr6	124385863	.	A	<INS:ME:LINE1>	.	PASS	SVTYPE=INS:ME:LINE1;END=124385876;SVLEN=65;AN=20618;AC=1;AF=4.850130953535746e-05;N_HET=1;N_HOMALT=0;N_HOMREF=10308;FREQ_HET=9.700261907071492e-05;FREQ_HOMALT=0.0;FREQ_HOMREF=0.9999029973809292;N_BI_GENOS=10309;East_Han_AN=6554;East_Han_AC=1;East_Han_AF=0
chr6	124651932	.	T	<INS:ME:LINE1>	.	PASS	SVTYPE=INS:ME:LINE1;END=124651932;SVLEN=8;AN=20618;AC=3;AF=0.00014550392860607236;N_HET=3;N_HOMALT=0;N_HOMREF=10306;FREQ_HET=0.0002910078572121447;FREQ_HOMALT=0.0;FREQ_HOMREF=0.9997089921427879;N_BI_GENOS=10309;North_Han_AN=2272;North_Han_AC=2;North_Han_A
chr6	124755874	.	A	<INS:ME:LINE1>	.	PASS	SVTYPE=INS:ME:LINE1;END=124755883;SVLEN=1549;AN=20618;AC=1;AF=4.850130953535746e-05;N_HET=1;N_HOMALT=0;N_HOMREF=10308;FREQ_HET=9.700261907071492e-05;FREQ_HOMALT=0.0;FREQ_HOMREF=0.9999029973809292;N_BI_GENOS=10309;East_Han_AN=6554;East_Han_AC=1;East_Han_AF
chr6	124929536	.	A	<INS:ME:LINE1>	.	PASS	SVTYPE=INS:ME:LINE1;END=124929547;SVLEN=6004;AN=20618;AC=1;AF=4.850130953535746e-05;N_HET=1;N_HOMALT=0;N_HOMREF=10308;FREQ_HET=9.700261907071492e-05;FREQ_HOMALT=0.0;FREQ_HOMREF=0.9999029973809292;N_BI_GENOS=10309;East_Han_AN=6554;East_Han_AC=1;East_Han_AF
chr6	124986188	.	C	<INS:ME:LINE1>	.	PASS	SVTYPE=INS:ME:LINE1;END=124986206;SVLEN=879;AN=20618;AC=1;AF=4.850130953535746e-05;N_HET=1;N_HOMALT=0;N_HOMREF=10308;FREQ_HET=9.700261907071492e-05;FREQ_HOMALT=0.0;FREQ_HOMREF=0.9999029973809292;N_BI_GENOS=10309;North_Han_AN=2272;North_Han_AC=1;North_Han_
chr6	125761623	.	G	<INS:ME:LINE1>	.	PASS	SVTYPE=INS:ME:LINE1;END=125761635;SVLEN=122;AN=20618;AC=2;AF=9.700261907071492e-05;N_HET=2;N_HOMALT=0;N_HOMREF=10307;FREQ_HET=0.00019400523814142983;FREQ_HOMALT=0.0;FREQ_HOMREF=0.9998059947618586;N_BI_GENOS=10309;South_Han_AN=1496;South_Han_AC=1;South_Han
chr6	125824278	.	A	<INS:ME:LINE1>	.	PASS	SVTYPE=INS:ME:LINE1;END=125824293;SVLEN=1881;AN=20618;AC=1;AF=4.850130953535746e-05;N_HET=1;N_HOMALT=0;N_HOMREF=10308;FREQ_HET=9.700261907071492e-05;FREQ_HOMALT=0.0;FREQ_HOMREF=0.9999029973809292;N_BI_GENOS=10309;East_Han_AN=6554;East_Han_AC=1;East_Han_AF
chr6	125898969	.	T	<INS:ME:LINE1>	.	PASS	SVTYPE=INS:ME:LINE1;END=125898969;SVLEN=99;AN=20618;AC=1;AF=4.850130953535746e-05;N_HET=1;N_HOMALT=0;N_HOMREF=10308;FREQ_HET=9.700261907071492e-05;FREQ_HOMALT=0.0;FREQ_HOMREF=0.9999029973809292;N_BI_GENOS=10309;East_Han_AN=6554;East_Han_AC=1;East_Han_AF=0
chr6	126046214	.	A	<INS:ME:LINE1>	.	PASS	SVTYPE=INS:ME:LINE1;END=126046218;SVLEN=780;AN=20618;AC=1;AF=4.850130953535746e-05;N_HET=1;N_HOMALT=0;N_HOMREF=10308;FREQ_HET=9.700261907071492e-05;FREQ_HOMALT=0.0;FREQ_HOMREF=0.9999029973809292;N_BI_GENOS=10309;North_Han_AN=2272;North_Han_AC=1;North_Han_
chr6	126302452	.	A	<INS:ME:LINE1>	.	PASS	SVTYPE=INS:ME:LINE1;END=126302465;SVLEN=298;AN=20618;AC=5;AF=0.00024250654767678728;N_HET=5;N_HOMALT=0;N_HOMREF=10304;FREQ_HET=0.00048501309535357455;FREQ_HOMALT=0.0;FREQ_HOMREF=0.9995149869046465;N_BI_GENOS=10309;East_Han_AN=6554;East_Han_AC=1;East_Han_A
chr6	126477128	.	T	<INS:ME:LINE1>	.	PASS	SVTYPE=INS:ME:LINE1;END=126477143;SVLEN=5998;AN=20618;AC=1;AF=4.850130953535746e-05;N_HET=1;N_HOMALT=0;N_HOMREF=10308;FREQ_HET=9.700261907071492e-05;FREQ_HOMALT=0.0;FREQ_HOMREF=0.9999029973809292;N_BI_GENOS=10309;South_Han_AN=1496;South_Han_AC=1;South_Han
chr6	126964293	.	A	<INS:ME:LINE1>	.	PASS	SVTYPE=INS:ME:LINE1;END=126964310;SVLEN=940;AN=20618;AC=2;AF=9.700261907071492e-05;N_HET=0;N_HOMALT=1;N_HOMREF=10308;FREQ_HET=0.0;FREQ_HOMALT=9.700261907071492e-05;FREQ_HOMREF=0.9999029973809292;N_BI_GENOS=10309;South_Han_AN=1496;South_Han_AC=2;South_Han_
chr6	128701909	.	C	<INS:ME:LINE1>	.	PASS	SVTYPE=INS:ME:LINE1;END=128701927;SVLEN=5991;AN=20618;AC=1;AF=4.850130953535746e-05;N_HET=1;N_HOMALT=0;N_HOMREF=10308;FREQ_HET=9.700261907071492e-05;FREQ_HOMALT=0.0;FREQ_HOMREF=0.9999029973809292;N_BI_GENOS=10309;Central_Han_AN=2128;Central_Han_AC=1;Centr
chr6	128771082	.	G	<INS:ME:LINE1>	.	PASS	SVTYPE=INS:ME:LINE1;END=128771088;SVLEN=746;AN=20618;AC=1;AF=4.850130953535746e-05;N_HET=1;N_HOMALT=0;N_HOMREF=10308;FREQ_HET=9.700261907071492e-05;FREQ_HOMALT=0.0;FREQ_HOMREF=0.9999029973809292;N_BI_GENOS=10309;East_Han_AN=6554;East_Han_AC=1;East_Han_AF=
chr6	128870249	.	T	<INS:ME:LINE1>	.	PASS	SVTYPE=INS:ME:LINE1;END=128870260;SVLEN=200;AN=20618;AC=2;AF=9.700261907071492e-05;N_HET=2;N_HOMALT=0;N_HOMREF=10307;FREQ_HET=0.00019400523814142983;FREQ_HOMALT=0.0;FREQ_HOMREF=0.9998059947618586;N_BI_GENOS=10309;East_Han_AN=6554;East_Han_AC=2;East_Han_AF
chr6	129428067	.	T	<INS:ME:LINE1>	.	PASS	SVTYPE=INS:ME:LINE1;END=129428067;SVLEN=287;AN=20618;AC=1;AF=4.850130953535746e-05;N_HET=1;N_HOMALT=0;N_HOMREF=10308;FREQ_HET=9.700261907071492e-05;FREQ_HOMALT=0.0;FREQ_HOMREF=0.9999029973809292;N_BI_GENOS=10309;East_Han_AN=6554;East_Han_AC=1;East_Han_AF=
chr6	129660167	.	G	<INS:ME:LINE1>	.	PASS	SVTYPE=INS:ME:LINE1;END=129660184;SVLEN=495;AN=20618;AC=2;AF=9.700261907071492e-05;N_HET=2;N_HOMALT=0;N_HOMREF=10307;FREQ_HET=0.00019400523814142983;FREQ_HOMALT=0.0;FREQ_HOMREF=0.9998059947618586;N_BI_GENOS=10309;Central_Han_AN=2128;Central_Han_AC=1;Centr

chr6	129741741	.	A	<INS:ME:LINE1>	.	PASS	SVTYPE=INS:ME:LINE1;END=129741755;SVLEN=5997;AN=20618;AC=56;AF=0.0027160733339800175;N_HET=54;N_HOMALT=1;N_HOMREF=10254;FREQ_HET=0.005238141429818605;FREQ_HOMALT=9.700261907071492e-05;FREQ_HOMREF=0.9946648559511106;N_BI_GENOS=10309;South_Han_AN=1496;South
chr6	129786295	.	A	<INS:ME:LINE1>	.	PASS	SVTYPE=INS:ME:LINE1;END=129786303;SVLEN=290;AN=20618;AC=26;AF=0.0012610340479192938;N_HET=24;N_HOMALT=1;N_HOMREF=10284;FREQ_HET=0.0023280628576971578;FREQ_HOMALT=9.700261907071492e-05;FREQ_HOMREF=0.9975749345232321;N_BI_GENOS=10309;South_Han_AN=1496;South
chr6	130227994	.	A	<INS:ME:LINE1>	.	PASS	SVTYPE=INS:ME:LINE1;END=130228007;SVLEN=6058;AN=20618;AC=1;AF=4.850130953535746e-05;N_HET=1;N_HOMALT=0;N_HOMREF=10308;FREQ_HET=9.700261907071492e-05;FREQ_HOMALT=0.0;FREQ_HOMREF=0.9999029973809292;N_BI_GENOS=10309;South_Han_AN=1496;South_Han_AC=1;South_Han
chr6	130252718	.	T	<INS:ME:LINE1>	.	PASS	SVTYPE=INS:ME:LINE1;END=130252718;SVLEN=655;AN=20618;AC=1;AF=4.850130953535746e-05;N_HET=1;N_HOMALT=0;N_HOMREF=10308;FREQ_HET=9.700261907071492e-05;FREQ_HOMALT=0.0;FREQ_HOMREF=0.9999029973809292;N_BI_GENOS=10309;East_Han_AN=6554;East_Han_AC=1;East_Han_AF=
chr6	130437678	.	G	<INS:ME:LINE1>	.	PASS	SVTYPE=INS:ME:LINE1;END=130437688;SVLEN=6000;AN=20618;AC=1;AF=4.850130953535746e-05;N_HET=1;N_HOMALT=0;N_HOMREF=10308;FREQ_HET=9.700261907071492e-05;FREQ_HOMALT=0.0;FREQ_HOMREF=0.9999029973809292;N_BI_GENOS=10309;East_Han_AN=6554;East_Han_AC=1;East_Han_AF
chr6	130516185	.	T	<INS:ME:LINE1>	.	PASS	SVTYPE=INS:ME:LINE1;END=130516194;SVLEN=6002;AN=20618;AC=1;AF=4.850130953535746e-05;N_HET=1;N_HOMALT=0;N_HOMREF=10308;FREQ_HET=9.700261907071492e-05;FREQ_HOMALT=0.0;FREQ_HOMREF=0.9999029973809292;N_BI_GENOS=10309;Northwest_Han_AN=1296;Northwest_Han_AC=1;N
chr6	130578954	.	C	<INS:ME:LINE1>	.	PASS	SVTYPE=INS:ME:LINE1;END=130578969;SVLEN=5982;AN=20618;AC=2;AF=9.700261907071492e-05;N_HET=2;N_HOMALT=0;N_HOMREF=10307;FREQ_HET=0.00019400523814142983;FREQ_HOMALT=0.0;FREQ_HOMREF=0.9998059947618586;N_BI_GENOS=10309;Central_Han_AN=2128;Central_Han_AC=1;Cent
chr6	130645474	.	A	<INS:ME:LINE1>	.	PASS	SVTYPE=INS:ME:LINE1;END=130645474;SVLEN=0;AN=20618;AC=3;AF=0.00014550392860607236;N_HET=3;N_HOMALT=0;N_HOMREF=10306;FREQ_HET=0.0002910078572121447;FREQ_HOMALT=0.0;FREQ_HOMREF=0.9997089921427879;N_BI_GENOS=10309;Lingnan_Han_AN=1888;Lingnan_Han_AC=1;Lingnan
chr6	130701100	.	G	<INS:ME:LINE1>	.	PASS	SVTYPE=INS:ME:LINE1;END=130701110;SVLEN=2234;AN=20618;AC=1;AF=4.850130953535746e-05;N_HET=1;N_HOMALT=0;N_HOMREF=10308;FREQ_HET=9.700261907071492e-05;FREQ_HOMALT=0.0;FREQ_HOMREF=0.9999029973809292;N_BI_GENOS=10309;Miao_AN=482;Miao_AC=1;Miao_AF=0.0020746887
chr6	130719679	.	G	<INS:ME:LINE1>	.	PASS	SVTYPE=INS:ME:LINE1;END=130719695;SVLEN=515;AN=20618;AC=1;AF=4.850130953535746e-05;N_HET=1;N_HOMALT=0;N_HOMREF=10308;FREQ_HET=9.700261907071492e-05;FREQ_HOMALT=0.0;FREQ_HOMREF=0.9999029973809292;N_BI_GENOS=10309;Southeast_Han_AN=1908;Southeast_Han_AC=1;So
chr6	131102330	.	A	<INS:ME:LINE1>	.	PASS	SVTYPE=INS:ME:LINE1;END=131102332;SVLEN=85;AN=20618;AC=22;AF=0.001067028809777864;N_HET=20;N_HOMALT=1;N_HOMREF=10288;FREQ_HET=0.0019400523814142982;FREQ_HOMALT=9.700261907071492e-05;FREQ_HOMREF=0.997962944999515;N_BI_GENOS=10309;South_Han_AN=1496;South_Ha
chr6	131168440	.	A	<INS:ME:LINE1>	.	PASS	SVTYPE=INS:ME:LINE1;END=131168458;SVLEN=6020;AN=20618;AC=1;AF=4.850130953535746e-05;N_HET=1;N_HOMALT=0;N_HOMREF=10308;FREQ_HET=9.700261907071492e-05;FREQ_HOMALT=0.0;FREQ_HOMREF=0.9999029973809292;N_BI_GENOS=10309;Northwest_Han_AN=1296;Northwest_Han_AC=1;N
chr6	131297207	.	C	<INS:ME:LINE1>	.	PASS	SVTYPE=INS:ME:LINE1;END=131297207;SVLEN=0;AN=20618;AC=1;AF=4.850130953535746e-05;N_HET=1;N_HOMALT=0;N_HOMREF=10308;FREQ_HET=9.700261907071492e-05;FREQ_HOMALT=0.0;FREQ_HOMREF=0.9999029973809292;N_BI_GENOS=10309;South_Han_AN=1496;South_Han_AC=1;South_Han_AF
chr6	131401032	.	T	<INS:ME:LINE1>	.	PASS	SVTYPE=INS:ME:LINE1;END=131401045;SVLEN=470;AN=20618;AC=1;AF=4.850130953535746e-05;N_HET=1;N_HOMALT=0;N_HOMREF=10308;FREQ_HET=9.700261907071492e-05;FREQ_HOMALT=0.0;FREQ_HOMREF=0.9999029973809292;N_BI_GENOS=10309;Central_Han_AN=2128;Central_Han_AC=1;Centra
chr6	131504556	.	T	<INS:ME:LINE1>	.	PASS	SVTYPE=INS:ME:LINE1;END=131504556;SVLEN=0;AN=20618;AC=4;AF=0.00019400523814142983;N_HET=2;N_HOMALT=1;N_HOMREF=10306;FREQ_HET=0.00019400523814142983;FREQ_HOMALT=9.700261907071492e-05;FREQ_HOMREF=0.9997089921427879;N_BI_GENOS=10309;Northwest_Han_AN=1296;Nor
chr6	131903492	.	C	<INS:ME:LINE1>	.	PASS	SVTYPE=INS:ME:LINE1;END=131903492;SVLEN=0;AN=20618;AC=8;AF=0.0003880104762828966;N_HET=4;N_HOMALT=2;N_HOMREF=10303;FREQ_HET=0.0003880104762828966;FREQ_HOMALT=0.00019400523814142983;FREQ_HOMREF=0.9994179842855757;N_BI_GENOS=10309;South_Han_AN=1496;South_
chr6	131906115	.	A	<INS:ME:LINE1>	.	PASS	SVTYPE=INS:ME:LINE1;END=131906115;SVLEN=0;AN=20618;AC=1;AF=4.850130953535746e-05;N_HET=1;N_HOMALT=0;N_HOMREF=10308;FREQ_HET=9.700261907071492e-05;FREQ_HOMALT=0.0;FREQ_HOMREF=0.9999029973809292;N_BI_GENOS=10309;Northwest_Han_AN=1296;Northwest_Han_AC=1;Nort
chr6	131907179	.	T	<INS:ME:LINE1>	.	PASS	SVTYPE=INS:ME:LINE1;END=131907179;SVLEN=0;AN=20618;AC=2;AF=9.700261907071492e-05;N_HET=0;N_HOMALT=1;N_HOMREF=10308;FREQ_HET=0.0;FREQ_HOMALT=9.700261907071492e-05;FREQ_HOMREF=0.9999029973809292;N_BI_GENOS=10309;Northwest_Han_AN=1296;Northwest_Han_AC=2;Nort
chr6	131907245	.	C	<INS:ME:LINE1>	.	PASS	SVTYPE=INS:ME:LINE1;END=131907245;SVLEN=0;AN=20618;AC=1;AF=4.850130953535746e-05;N_HET=1;N_HOMALT=0;N_HOMREF=10308;FREQ_HET=9.700261907071492e-05;FREQ_HOMALT=0.0;FREQ_HOMREF=0.9999029973809292;N_BI_GENOS=10309;East_Han_AN=6554;East_Han_AC=1;East_Han_AF=0.
chr6	132038852	.	G	<INS:ME:LINE1>	.	PASS	SVTYPE=INS:ME:LINE1;END=132038862;SVLEN=6020;AN=20618;AC=1;AF=4.850130953535746e-05;N_HET=1;N_HOMALT=0;N_HOMREF=10308;FREQ_HET=9.700261907071492e-05;FREQ_HOMALT=0.0;FREQ_HOMREF=0.9999029973809292;N_BI_GENOS=10309;East_Han_AN=6554;East_Han_AC=1;East_Han_AF
chr6	132622296	.	C	<INS:ME:LINE1>	.	PASS	SVTYPE=INS:ME:LINE1;END=132622297;SVLEN=67;AN=20618;AC=1;AF=4.850130953535746e-05;N_HET=1;N_HOMALT=0;N_HOMREF=10308;FREQ_HET=9.700261907071492e-05;FREQ_HOMALT=0.0;FREQ_HOMREF=0.9999029973809292;N_BI_GENOS=10309;North_Han_AN=2272;North_Han_AC=1;North_Han_A
chr6	132774910	.	A	<INS:ME:LINE1>	.	PASS	SVTYPE=INS:ME:LINE1;END=132774910;SVLEN=100;AN=20618;AC=2;AF=9.700261907071492e-05;N_HET=2;N_HOMALT=0;N_HOMREF=10307;FREQ_HET=0.00019400523814142983;FREQ_HOMALT=0.0;FREQ_HOMREF=0.9998059947618586;N_BI_GENOS=10309;East_Han_AN=6554;East_Han_AC=1;East_Han_AF
chr6	133055638	.	A	<INS:ME:LINE1>	.	PASS	SVTYPE=INS:ME:LINE1;END=133055652;SVLEN=1086;AN=20618;AC=1;AF=4.850130953535746e-05;N_HET=1;N_HOMALT=0;N_HOMREF=10308;FREQ_HET=9.700261907071492e-05;FREQ_HOMALT=0.0;FREQ_HOMREF=0.9999029973809292;N_BI_GENOS=10309;Manchu_AN=416;Manchu_AC=1;Manchu_AF=0.0024
chr6	133309716	.	T	<INS:ME:LINE1>	.	PASS	SVTYPE=INS:ME:LINE1;END=133309734;SVLEN=1243;AN=20618;AC=1;AF=4.850130953535746e-05;N_HET=1;N_HOMALT=0;N_HOMREF=10308;FREQ_HET=9.700261907071492e-05;FREQ_HOMALT=0.0;FREQ_HOMREF=0.9999029973809292;N_BI_GENOS=10309;East_Han_AN=6554;East_Han_AC=1;East_Han_AF
chr6	133410627	.	G	<INS:ME:LINE1>	.	PASS	SVTYPE=INS:ME:LINE1;END=133410643;SVLEN=266;AN=20618;AC=1;AF=4.850130953535746e-05;N_HET=1;N_HOMALT=0;N_HOMREF=10308;FREQ_HET=9.700261907071492e-05;FREQ_HOMALT=0.0;FREQ_HOMREF=0.9999029973809292;N_BI_GENOS=10309;East_Han_AN=6554;East_Han_AC=1;East_Han_AF=
chr6	133716661	.	A	<INS:ME:LINE1>	.	PASS	SVTYPE=INS:ME:LINE1;END=133716678;SVLEN=5997;AN=20618;AC=36;AF=0.0017460471432728683;N_HET=28;N_HOMALT=4;N_HOMREF=10277;FREQ_HET=0.0027160733339800175;FREQ_HOMALT=0.0003880104762828966;FREQ_HOMREF=0.9968959161897372;N_BI_GENOS=10309;South_Han_AN=1496;Sou
chr6	133733901	.	C	<INS:ME:LINE1>	.	PASS	SVTYPE=INS:ME:LINE1;END=133733901;SVLEN=1443;AN=20618;AC=3;AF=0.00014550392860607236;N_HET=1;N_HOMALT=1;N_HOMREF=10307;FREQ_HET=9.700261907071492e-05;FREQ_HOMALT=9.700261907071492e-05;FREQ_HOMREF=0.9998059947618586;N_BI_GENOS=10309;East_Han_AN=6554;East_H
chr6	133833656	.	A	<INS:ME:LINE1>	.	PASS	SVTYPE=INS:ME:LINE1;END=133833670;SVLEN=3809;AN=20618;AC=1;AF=4.850130953535746e-05;N_HET=1;N_HOMALT=0;N_HOMREF=10308;FREQ_HET=9.700261907071492e-05;FREQ_HOMALT=0.0;FREQ_HOMREF=0.9999029973809292;N_BI_GENOS=10309;North_Han_AN=2272;North_Han_AC=1;North_Han
chr6	134549653	.	C	<INS:ME:LINE1>	.	PASS	SVTYPE=INS:ME:LINE1;END=134549670;SVLEN=6005;AN=20618;AC=1;AF=4.850130953535746e-05;N_HET=1;N_HOMALT=0;N_HOMREF=10308;FREQ_HET=9.700261907071492e-05;FREQ_HOMALT=0.0;FREQ_HOMREF=0.9999029973809292;N_BI_GENOS=10309;Central_Han_AN=2128;Central_Han_AC=1;Centr
chr6	134899746	.	G	<INS:ME:LINE1>	.	PASS	SVTYPE=INS:ME:LINE1;END=134899759;SVLEN=5994;AN=20618;AC=1;AF=4.850130953535746e-05;N_HET=1;N_HOMALT=0;N_HOMREF=10308;FREQ_HET=9.700261907071492e-05;FREQ_HOMALT=0.0;FREQ_HOMREF=0.9999029973809292;N_BI_GENOS=10309;East_Han_AN=6554;East_Han_AC=1;East_Han_AF
chr6	135162788	.	T	<INS:ME:LINE1>	.	PASS	SVTYPE=INS:ME:LINE1;END=135162804;SVLEN=1510;AN=20618;AC=8;AF=0.0003880104762828966;N_HET=8;N_HOMALT=0;N_HOMREF=10301;FREQ_HET=0.0007760209525657193;FREQ_HOMALT=0.0;FREQ_HOMREF=0.9992239790474343;N_BI_GENOS=10309;Northwest_Han_AN=1296;Northwest_Han_AC=1;
chr6	136510418	.	G	<INS:ME:LINE1>	.	PASS	SVTYPE=INS:ME:LINE1;END=136510434;SVLEN=413;AN=20618;AC=1;AF=4.850130953535746e-05;N_HET=1;N_HOMALT=0;N_HOMREF=10308;FREQ_HET=9.700261907071492e-05;FREQ_HOMALT=0.0;FREQ_HOMREF=0.9999029973809292;N_BI_GENOS=10309;East_Han_AN=6554;East_Han_AC=1;East_Han_AF=
chr6	137452300	.	A	<INS:ME:LINE1>	.	PASS	SVTYPE=INS:ME:LINE1;END=137452305;SVLEN=318;AN=20618;AC=2;AF=9.700261907071492e-05;N_HET=0;N_HOMALT=1;N_HOMREF=10308;FREQ_HET=0.0;FREQ_HOMALT=9.700261907071492e-05;FREQ_HOMREF=0.9999029973809292;N_BI_GENOS=10309;East_Han_AN=6554;East_Han_AC=2;East_Han_AF=
chr6	138628172	.	A	<INS:ME:LINE1>	.	PASS	SVTYPE=INS:ME:LINE1;END=138628172;SVLEN=0;AN=20618;AC=18;AF=0.0008730235716364342;N_HET=10;N_HOMALT=4;N_HOMREF=10295;FREQ_HET=0.0009700261907071491;FREQ_HOMALT=0.0003880104762828966;FREQ_HOMREF=0.99864196333301;N_BI_GENOS=10309;Northwest_Han_AN=1296;Nort
chr6	139109830	.	A	<INS:ME:LINE1>	.	PASS	SVTYPE=INS:ME:LINE1;END=139109845;SVLEN=277;AN=20618;AC=3;AF=0.00014550392860607236;N_HET=1;N_HOMALT=1;N_HOMREF=10307;FREQ_HET=9.700261907071492e-05;FREQ_HOMALT=9.700261907071492e-05;FREQ_HOMREF=0.9998059947618586;N_BI_GENOS=10309;Lingnan_Han_AN=1888;Ling
chr6	139116446	.	T	<INS:ME:LINE1>	.	PASS	SVTYPE=INS:ME:LINE1;END=139116461;SVLEN=6005;AN=20618;AC=2;AF=9.700261907071492e-05;N_HET=2;N_HOMALT=0;N_HOMREF=10307;FREQ_HET=0.00019400523814142983;FREQ_HOMALT=0.0;FREQ_HOMREF=0.9998059947618586;N_BI_GENOS=10309;Central_Han_AN=2128;Central_Han_AC=1;Cent
chr6	139218303	.	A	<INS:ME:LINE1>	.	PASS	SVTYPE=INS:ME:LINE1;END=139218315;SVLEN=6000;AN=20618;AC=1;AF=4.850130953535746e-05;N_HET=1;N_HOMALT=0;N_HOMREF=10308;FREQ_HET=9.700261907071492e-05;FREQ_HOMALT=0.0;FREQ_HOMREF=0.9999029973809292;N_BI_GENOS=10309;Central_Han_AN=2128;Central_Han_AC=1;Centr
chr6	139520561	.	T	<INS:ME:LINE1>	.	PASS	SVTYPE=INS:ME:LINE1;END=139520582;SVLEN=1603;AN=20618;AC=3;AF=0.00014550392860607236;N_HET=3;N_HOMALT=0;N_HOMREF=10306;FREQ_HET=0.0002910078572121447;FREQ_HOMALT=0.0;FREQ_HOMREF=0.9997089921427879;N_BI_GENOS=10309;South_Han_AN=1496;South_Han_AC=1;South_Ha
chr6	139897078	.	T	<INS:ME:LINE1>	.	PASS	SVTYPE=INS:ME:LINE1;END=139897082;SVLEN=385;AN=20618;AC=1;AF=4.850130953535746e-05;N_HET=1;N_HOMALT=0;N_HOMREF=10308;FREQ_HET=9.700261907071492e-05;FREQ_HOMALT=0.0;FREQ_HOMREF=0.9999029973809292;N_BI_GENOS=10309;South_Han_AN=1496;South_Han_AC=1;South_Han_
chr6	140006130	.	G	<INS:ME:LINE1>	.	PASS	SVTYPE=INS:ME:LINE1;END=140006144;SVLEN=5998;AN=20618;AC=3;AF=0.00014550392860607236;N_HET=3;N_HOMALT=0;N_HOMREF=10306;FREQ_HET=0.0002910078572121447;FREQ_HOMALT=0.0;FREQ_HOMREF=0.9997089921427879;N_BI_GENOS=10309;South_Han_AN=1496;South_Han_AC=2;South_Ha
chr6	140023556	.	A	<INS:ME:LINE1>	.	PASS	SVTYPE=INS:ME:LINE1;END=140023569;SVLEN=5999;AN=20618;AC=1;AF=4.850130953535746e-05;N_HET=1;N_HOMALT=0;N_HOMREF=10308;FREQ_HET=9.700261907071492e-05;FREQ_HOMALT=0.0;FREQ_HOMREF=0.9999029973809292;N_BI_GENOS=10309;Central_Han_AN=2128;Central_Han_AC=1;Centr
chr6	140131231	.	A	<INS:ME:LINE1>	.	PASS	SVTYPE=INS:ME:LINE1;END=140131242;SVLEN=3436;AN=20618;AC=1;AF=4.850130953535746e-05;N_HET=1;N_HOMALT=0;N_HOMREF=10308;FREQ_HET=9.700261907071492e-05;FREQ_HOMALT=0.0;FREQ_HOMREF=0.9999029973809292;N_BI_GENOS=10309;Tibetan_AN=414;Tibetan_AC=1;Tibetan_AF=0.0
chr6	140135467	.	G	<INS:ME:LINE1>	.	PASS	SVTYPE=INS:ME:LINE1;END=140135467;SVLEN=239;AN=20618;AC=1;AF=4.850130953535746e-05;N_HET=1;N_HOMALT=0;N_HOMREF=10308;FREQ_HET=9.700261907071492e-05;FREQ_HOMALT=0.0;FREQ_HOMREF=0.9999029973809292;N_BI_GENOS=10309;East_Han_AN=6554;East_Han_AC=1;East_Han_AF=
chr6	140424905	.	T	<INS:ME:LINE1>	.	PASS	SVTYPE=INS:ME:LINE1;END=140424918;SVLEN=5997;AN=20618;AC=2;AF=9.700261907071492e-05;N_HET=2;N_HOMALT=0;N_HOMREF=10307;FREQ_HET=0.00019400523814142983;FREQ_HOMALT=0.0;FREQ_HOMREF=0.9998059947618586;N_BI_GENOS=10309;East_Han_AN=6554;East_Han_AC=1;East_Han_A
chr6	140725204	.	T	<INS:ME:LINE1>	.	PASS	SVTYPE=INS:ME:LINE1;END=140725204;SVLEN=1386;AN=20618;AC=1;AF=4.850130953535746e-05;N_HET=1;N_HOMALT=0;N_HOMREF=10308;FREQ_HET=9.700261907071492e-05;FREQ_HOMALT=0.0;FREQ_HOMREF=0.9999029973809292;N_BI_GENOS=10309;East_Han_AN=6554;East_Han_AC=1;East_Han_AF
chr6	141129443	.	A	<INS:ME:LINE1>	.	PASS	SVTYPE=INS:ME:LINE1;END=141129443;SVLEN=321;AN=20618;AC=1;AF=4.850130953535746e-05;N_HET=1;N_HOMALT=0;N_HOMREF=10308;FREQ_HET=9.700261907071492e-05;FREQ_HOMALT=0.0;FREQ_HOMREF=0.9999029973809292;N_BI_GENOS=10309;Miao_AN=482;Miao_AC=1;Miao_AF=0.00207468879
chr6	141758099	.	A	<INS:ME:LINE1>	.	PASS	SVTYPE=INS:ME:LINE1;END=141758118;SVLEN=827;AN=20618;AC=13;AF=0.0006305170239596469;N_HET=11;N_HOMALT=1;N_HOMREF=10297;FREQ_HET=0.001067028809777864;FREQ_HOMALT=9.700261907071492e-05;FREQ_HOMREF=0.9988359685711514;N_BI_GENOS=10309;Northwest_Han_AN=1296;No
chr6	142016068	.	T	<INS:ME:LINE1>	.	PASS	SVTYPE=INS:ME:LINE1;END=142016068;SVLEN=0;AN=20618;AC=1;AF=4.850130953535746e-05;N_HET=1;N_HOMALT=0;N_HOMREF=10308;FREQ_HET=9.700261907071492e-05;FREQ_HOMALT=0.0;FREQ_HOMREF=0.9999029973809292;N_BI_GENOS=10309;Southeast_Han_AN=1908;Southeast_Han_AC=1;Sout
chr6	142128963	.	G	<INS:ME:LINE1>	.	PASS	SVTYPE=INS:ME:LINE1;END=142128973;SVLEN=5988;AN=20618;AC=3241;AF=0.15719274420409352;N_HET=2603;N_HOMALT=319;N_HOMREF=7387;FREQ_HET=0.2524978174410709;FREQ_HOMALT=0.030943835483558058;FREQ_HOMREF=0.716558347075371;N_BI_GENOS=10309;South_Han_AN=1496;South_
chr6	142314099	.	A	<INS:ME:LINE1>	.	PASS	SVTYPE=INS:ME:LINE1;END=142314118;SVLEN=780;AN=20618;AC=1;AF=4.850130953535746e-05;N_HET=1;N_HOMALT=0;N_HOMREF=10308;FREQ_HET=9.700261907071492e-05;FREQ_HOMALT=0.0;FREQ_HOMREF=0.9999029973809292;N_BI_GENOS=10309;Miao_AN=482;Miao_AC=1;Miao_AF=0.00207468879
chr6	142389978	.	A	<INS:ME:LINE1>	.	PASS	SVTYPE=INS:ME:LINE1;END=142389994;SVLEN=783;AN=20618;AC=1;AF=4.850130953535746e-05;N_HET=1;N_HOMALT=0;N_HOMREF=10308;FREQ_HET=9.700261907071492e-05;FREQ_HOMALT=0.0;FREQ_HOMREF=0.9999029973809292;N_BI_GENOS=10309;East_Han_AN=6554;East_Han_AC=1;East_Han_AF=
chr6	142826276	.	C	<INS:ME:LINE1>	.	PASS	SVTYPE=INS:ME:LINE1;END=142826277;SVLEN=5998;AN=20618;AC=1;AF=4.850130953535746e-05;N_HET=1;N_HOMALT=0;N_HOMREF=10308;FREQ_HET=9.700261907071492e-05;FREQ_HOMALT=0.0;FREQ_HOMREF=0.9999029973809292;N_BI_GENOS=10309;Central_Han_AN=2128;Central_Han_AC=1;Centr
chr6	143037803	.	T	<INS:ME:LINE1>	.	PASS	SVTYPE=INS:ME:LINE1;END=143037805;SVLEN=1162;AN=20618;AC=1;AF=4.850130953535746e-05;N_HET=1;N_HOMALT=0;N_HOMREF=10308;FREQ_HET=9.700261907071492e-05;FREQ_HOMALT=0.0;FREQ_HOMREF=0.9999029973809292;N_BI_GENOS=10309;East_Han_AN=6554;East_Han_AC=1;East_Han_AF
chr6	143251349	.	C	<INS:ME:LINE1>	.	PASS	SVTYPE=INS:ME:LINE1;END=143251363;SVLEN=3725;AN=20618;AC=1;AF=4.850130953535746e-05;N_HET=1;N_HOMALT=0;N_HOMREF=10308;FREQ_HET=9.700261907071492e-05;FREQ_HOMALT=0.0;FREQ_HOMREF=0.9999029973809292;N_BI_GENOS=10309;Lingnan_Han_AN=1888;Lingnan_Han_AC=1;Lingn
chr6	143478469	.	A	<INS:ME:LINE1>	.	PASS	SVTYPE=INS:ME:LINE1;END=143478479;SVLEN=1276;AN=20618;AC=3;AF=0.00014550392860607236;N_HET=3;N_HOMALT=0;N_HOMREF=10306;FREQ_HET=0.0002910078572121447;FREQ_HOMALT=0.0;FREQ_HOMREF=0.9997089921427879;N_BI_GENOS=10309;Lingnan_Han_AN=1888;Lingnan_Han_AC=1;Ling
chr6	143709056	.	A	<INS:ME:LINE1>	.	PASS	SVTYPE=INS:ME:LINE1;END=143709070;SVLEN=5998;AN=20618;AC=1;AF=4.850130953535746e-05;N_HET=1;N_HOMALT=0;N_HOMREF=10308;FREQ_HET=9.700261907071492e-05;FREQ_HOMALT=0.0;FREQ_HOMREF=0.9999029973809292;N_BI_GENOS=10309;Central_Han_AN=2128;Central_Han_AC=1;Centr
chr6	143951821	.	G	<INS:ME:LINE1>	.	PASS	SVTYPE=INS:ME:LINE1;END=143951834;SVLEN=706;AN=20618;AC=1;AF=4.850130953535746e-05;N_HET=1;N_HOMALT=0;N_HOMREF=10308;FREQ_HET=9.700261907071492e-05;FREQ_HOMALT=0.0;FREQ_HOMREF=0.9999029973809292;N_BI_GENOS=10309;North_Han_AN=2272;North_Han_AC=1;North_Han_
chr6	143962498	.	A	<INS:ME:LINE1>	.	PASS	SVTYPE=INS:ME:LINE1;END=143962515;SVLEN=3655;AN=20618;AC=3;AF=0.00014550392860607236;N_HET=3;N_HOMALT=0;N_HOMREF=10306;FREQ_HET=0.0002910078572121447;FREQ_HOMALT=0.0;FREQ_HOMREF=0.9997089921427879;N_BI_GENOS=10309;Northwest_Han_AN=1296;Northwest_Han_AC=1;
chr6	144305370	.	T	<INS:ME:LINE1>	.	PASS	SVTYPE=INS:ME:LINE1;END=144305388;SVLEN=3584;AN=20618;AC=1;AF=4.850130953535746e-05;N_HET=1;N_HOMALT=0;N_HOMREF=10308;FREQ_HET=9.700261907071492e-05;FREQ_HOMALT=0.0;FREQ_HOMREF=0.9999029973809292;N_BI_GENOS=10309;Central_Han_AN=2128;Central_Han_AC=1;Centr
chr6	144415456	.	A	<INS:ME:LINE1>	.	PASS	SVTYPE=INS:ME:LINE1;END=144415459;SVLEN=1363;AN=20618;AC=2;AF=9.700261907071492e-05;N_HET=2;N_HOMALT=0;N_HOMREF=10307;FREQ_HET=0.00019400523814142983;FREQ_HOMALT=0.0;FREQ_HOMREF=0.9998059947618586;N_BI_GENOS=10309;Lingnan_Han_AN=1888;Lingnan_Han_AC=2;Ling
chr6	144741990	.	A	<INS:ME:LINE1>	.	PASS	SVTYPE=INS:ME:LINE1;END=144742006;SVLEN=5963;AN=20618;AC=5;AF=0.00024250654767678728;N_HET=5;N_HOMALT=0;N_HOMREF=10304;FREQ_HET=0.0004850130953535455;FREQ_HOMALT=0.0;FREQ_HOMREF=0.9995149869046465;N_BI_GENOS=10309;Lingnan_Han_AN=1888;Lingnan_Han_AC=1;Lin
chr6	144968531	.	A	<INS:ME:LINE1>	.	PASS	SVTYPE=INS:ME:LINE1;END=144968547;SVLEN=4032;AN=20618;AC=7;AF=0.0003395091667475022;N_HET=5;N_HOMALT=1;N_HOMREF=10303;FREQ_HET=0.0004850130953535455;FREQ_HOMALT=9.700261907071492e-05;FREQ_HOMREF=0.9994179842855757;N_BI_GENOS=10309;South_Han_AN=1496;South
chr6	145078515	.	G	<INS:ME:LINE1>	.	PASS	SVTYPE=INS:ME:LINE1;END=145078531;SVLEN=5986;AN=20618;AC=1;AF=4.850130953535746e-05;N_HET=1;N_HOMALT=0;N_HOMREF=10308;FREQ_HET=9.700261907071492e-05;FREQ_HOMALT=0.0;FREQ_HOMREF=0.9999029973809292;N_BI_GENOS=10309;Central_Han_AN=2128;Central_Han_AC=1;Centr
chr6	145103434	.	T	<INS:ME:LINE1>	.	PASS	SVTYPE=INS:ME:LINE1;END=145103434;SVLEN=0;AN=20618;AC=2;AF=9.700261907071492e-05;N_HET=2;N_HOMALT=0;N_HOMREF=10307;FREQ_HET=0.00019400523814142983;FREQ_HOMALT=0.0;FREQ_HOMREF=0.9998059947618586;N_BI_GENOS=10309;South_Han_AN=1496;South_Han_AC=1;South_Han_A
chr6	145179791	.	A	<INS:ME:LINE1>	.	PASS	SVTYPE=INS:ME:LINE1;END=145179804;SVLEN=1572;AN=20618;AC=7732;AF=0.3750121253273838;N_HET=2656;N_HOMALT=2538;N_HOMREF=5115;FREQ_HET=0.2576389562518188;FREQ_HOMALT=0.24619264720147443;FREQ_HOMREF=0.49616839654670675;N_BI_GENOS=10309;South_Han_AN=1496;South
chr6	145686617	.	A	<INS:ME:LINE1>	.	PASS	SVTYPE=INS:ME:LINE1;END=145686630;SVLEN=1624;AN=20618;AC=1;AF=4.850130953535746e-05;N_HET=1;N_HOMALT=0;N_HOMREF=10308;FREQ_HET=9.700261907071492e-05;FREQ_HOMALT=0.0;FREQ_HOMREF=0.9999029973809292;N_BI_GENOS=10309;Southeast_Han_AN=1908;Southeast_Han_AC=1;S
chr6	146025123	.	T	<INS:ME:LINE1>	.	PASS	SVTYPE=INS:ME:LINE1;END=146025128;SVLEN=3955;AN=20618;AC=2;AF=9.700261907071492e-05;N_HET=2;N_HOMALT=0;N_HOMREF=10307;FREQ_HET=0.00019400523814142983;FREQ_HOMALT=0.0;FREQ_HOMREF=0.9998059947618586;N_BI_GENOS=10309;Central_Han_AN=2128;Central_Han_AC=1;Cent
chr6	146131317	.	A	<INS:ME:LINE1>	.	PASS	SVTYPE=INS:ME:LINE1;END=146131347;SVLEN=923;AN=20618;AC=3;AF=0.00014550392860607236;N_HET=3;N_HOMALT=0;N_HOMREF=10306;FREQ_HET=0.0002910078572121447;FREQ_HOMALT=0.0;FREQ_HOMREF=0.9997089921427879;N_BI_GENOS=10309;East_Han_AN=6554;East_Han_AC=2;East_Han_AF
chr6	146672253	.	A	<INS:ME:LINE1>	.	PASS	SVTYPE=INS:ME:LINE1;END=146672273;SVLEN=832;AN=20618;AC=2;AF=9.700261907071492e-05;N_HET=2;N_HOMALT=0;N_HOMREF=10307;FREQ_HET=0.00019400523814142983;FREQ_HOMALT=0.0;FREQ_HOMREF=0.9998059947618586;N_BI_GENOS=10309;East_Han_AN=6554;East_Han_AC=2;East_Han_AF
chr6	146708321	.	C	<INS:ME:LINE1>	.	PASS	SVTYPE=INS:ME:LINE1;END=146708333;SVLEN=1332;AN=20618;AC=6;AF=0.0002910078572121447;N_HET=6;N_HOMALT=0;N_HOMREF=10303;FREQ_HET=0.0005820157144242894;FREQ_HOMALT=0.0;FREQ_HOMREF=0.9994179842855757;N_BI_GENOS=10309;South_Han_AN=1496;South_Han_AC=1;South_Han
chr6	146964017	.	T	<INS:ME:LINE1>	.	PASS	SVTYPE=INS:ME:LINE1;END=146964033;SVLEN=6019;AN=20618;AC=1;AF=4.850130953535746e-05;N_HET=1;N_HOMALT=0;N_HOMREF=10308;FREQ_HET=9.700261907071492e-05;FREQ_HOMALT=0.0;FREQ_HOMREF=0.9999029973809292;N_BI_GENOS=10309;Central_Han_AN=2128;Central_Han_AC=1;Centr
chr6	147452962	.	A	<INS:ME:LINE1>	.	PASS	SVTYPE=INS:ME:LINE1;END=147452977;SVLEN=6000;AN=20618;AC=71;AF=0.0034435929770103794;N_HET=71;N_HOMALT=0;N_HOMREF=10238;FREQ_HET=0.006887185954020759;FREQ_HOMALT=0.0;FREQ_HOMREF=0.9931128140459793;N_BI_GENOS=10309;South_Han_AN=1496;South_Han_AC=9;South_Ha
chr6	147842433	.	A	<INS:ME:LINE1>	.	PASS	SVTYPE=INS:ME:LINE1;END=147842451;SVLEN=6000;AN=20618;AC=45;AF=0.0021825589290910856;N_HET=43;N_HOMALT=1;N_HOMREF=10265;FREQ_HET=0.004171111262004741;FREQ_HOMALT=9.700261907071492e-05;FREQ_HOMREF=0.9957318847608886;N_BI_GENOS=10309;South_Han_AN=1496;South
chr6	147853566	.	A	<INS:ME:LINE1>	.	PASS	SVTYPE=INS:ME:LINE1;END=147853581;SVLEN=221;AN=20618;AC=1;AF=4.850130953535746e-05;N_HET=1;N_HOMALT=0;N_HOMREF=10308;FREQ_HET=9.700261907071492e-05;FREQ_HOMALT=0.0;FREQ_HOMREF=0.9999029973809292;N_BI_GENOS=10309;Manchu_AN=416;Manchu_AC=1;Manchu_AF=0.00240
chr6	147874652	.	A	<INS:ME:LINE1>	.	PASS	SVTYPE=INS:ME:LINE1;END=147874672;SVLEN=2590;AN=20618;AC=9285;AF=0.45033465903579395;N_HET=4983;N_HOMALT=2151;N_HOMREF=3175;FREQ_HET=0.4833640508293724;FREQ_HOMALT=0.20865263362110778;FREQ_HOMREF=0.30798331554951985;N_BI_GENOS=10309;South_Han_AN=1496;Sout
chr6	147949812	.	C	<INS:ME:LINE1>	.	PASS	SVTYPE=INS:ME:LINE1;END=147949827;SVLEN=5979;AN=20618;AC=1;AF=4.850130953535746e-05;N_HET=1;N_HOMALT=0;N_HOMREF=10308;FREQ_HET=9.700261907071492e-05;FREQ_HOMALT=0.0;FREQ_HOMREF=0.9999029973809292;N_BI_GENOS=10309;East_Han_AN=6554;East_Han_AC=1;East_Han_AF
chr6	148843368	.	T	<INS:ME:LINE1>	.	PASS	SVTYPE=INS:ME:LINE1;END=148843386;SVLEN=6006;AN=20618;AC=1;AF=4.850130953535746e-05;N_HET=1;N_HOMALT=0;N_HOMREF=10308;FREQ_HET=9.700261907071492e-05;FREQ_HOMALT=0.0;FREQ_HOMREF=0.9999029973809292;N_BI_GENOS=10309;East_Han_AN=6554;East_Han_AC=1;East_Han_AF
chr6	149026973	.	G	<INS:ME:LINE1>	.	PASS	SVTYPE=INS:ME:LINE1;END=149026982;SVLEN=6022;AN=20618;AC=1;AF=4.850130953535746e-05;N_HET=1;N_HOMALT=0;N_HOMREF=10308;FREQ_HET=9.700261907071492e-05;FREQ_HOMALT=0.0;FREQ_HOMREF=0.9999029973809292;N_BI_GENOS=10309;South_Han_AN=1496;South_Han_AC=1;South_Han
chr6	149174820	.	A	<INS:ME:LINE1>	.	PASS	SVTYPE=INS:ME:LINE1;END=149174832;SVLEN=453;AN=20618;AC=4;AF=0.00019400523814142983;N_HET=4;N_HOMALT=0;N_HOMREF=10305;FREQ_HET=0.0003880104762828966;FREQ_HOMALT=0.0;FREQ_HOMREF=0.9996119895237171;N_BI_GENOS=10309;East_Han_AN=6554;East_Han_AC=1;East_Han_A
chr6	149641724	.	A	<INS:ME:LINE1>	.	PASS	SVTYPE=INS:ME:LINE1;END=149641739;SVLEN=6000;AN=20618;AC=1;AF=4.850130953535746e-05;N_HET=1;N_HOMALT=0;N_HOMREF=10308;FREQ_HET=9.700261907071492e-05;FREQ_HOMALT=0.0;FREQ_HOMREF=0.9999029973809292;N_BI_GENOS=10309;Southeast_Han_AN=1908;Southeast_Han_AC=1;S
chr6	149993232	.	T	<INS:ME:LINE1>	.	PASS	SVTYPE=INS:ME:LINE1;END=149993238;SVLEN=1162;AN=20618;AC=1;AF=4.850130953535746e-05;N_HET=1;N_HOMALT=0;N_HOMREF=10308;FREQ_HET=9.700261907071492e-05;FREQ_HOMALT=0.0;FREQ_HOMREF=0.9999029973809292;N_BI_GENOS=10309;East_Han_AN=6554;East_Han_AC=1;East_Han_AF
chr6	150043401	.	G	<INS:ME:LINE1>	.	PASS	SVTYPE=INS:ME:LINE1;END=150043416;SVLEN=5946;AN=20618;AC=1;AF=4.850130953535746e-05;N_HET=1;N_HOMALT=0;N_HOMREF=10308;FREQ_HET=9.700261907071492e-05;FREQ_HOMALT=0.0;FREQ_HOMREF=0.9999029973809292;N_BI_GENOS=10309;East_Han_AN=6554;East_Han_AC=1;East_Han_AF
chr6	150361734	.	A	<INS:ME:LINE1>	.	PASS	SVTYPE=INS:ME:LINE1;END=150361734;SVLEN=462;AN=20618;AC=1;AF=4.850130953535746e-05;N_HET=1;N_HOMALT=0;N_HOMREF=10308;FREQ_HET=9.700261907071492e-05;FREQ_HOMALT=0.0;FREQ_HOMREF=0.9999029973809292;N_BI_GENOS=10309;North_Han_AN=2272;North_Han_AC=1;North_Han_
chr6	150535355	.	G	<INS:ME:LINE1>	.	PASS	SVTYPE=INS:ME:LINE1;END=150535362;SVLEN=88;AN=20618;AC=2;AF=9.700261907071492e-05;N_HET=2;N_HOMALT=0;N_HOMREF=10307;FREQ_HET=0.00019400523814142983;FREQ_HOMALT=0.0;FREQ_HOMREF=0.9998059947618586;N_BI_GENOS=10309;Northwest_Han_AN=1296;Northwest_Han_AC=1;No
chr6	150652103	.	T	<INS:ME:LINE1>	.	PASS	SVTYPE=INS:ME:LINE1;END=150652107;SVLEN=3174;AN=20618;AC=1;AF=4.850130953535746e-05;N_HET=1;N_HOMALT=0;N_HOMREF=10308;FREQ_HET=9.700261907071492e-05;FREQ_HOMALT=0.0;FREQ_HOMREF=0.9999029973809292;N_BI_GENOS=10309;East_Han_AN=6554;East_Han_AC=1;East_Han_AF
chr6	150761657	.	A	<INS:ME:LINE1>	.	PASS	SVTYPE=INS:ME:LINE1;END=150761672;SVLEN=6000;AN=20618;AC=6;AF=0.0002910078572121447;N_HET=6;N_HOMALT=0;N_HOMREF=10303;FREQ_HET=0.0005820157144242894;FREQ_HOMALT=0.0;FREQ_HOMREF=0.9994179842855757;N_BI_GENOS=10309;North_Han_AN=2272;North_Han_AC=1;North_Han
chr6	151038028	.	A	<INS:ME:LINE1>	.	PASS	SVTYPE=INS:ME:LINE1;END=151038044;SVLEN=710;AN=20618;AC=1;AF=4.850130953535746e-05;N_HET=1;N_HOMALT=0;N_HOMREF=10308;FREQ_HET=9.700261907071492e-05;FREQ_HOMALT=0.0;FREQ_HOMREF=0.9999029973809292;N_BI_GENOS=10309;Southeast_Han_AN=1908;Southeast_Han_AC=1;So
chr6	151205734	.	A	<INS:ME:LINE1>	.	PASS	SVTYPE=INS:ME:LINE1;END=151205752;SVLEN=1496;AN=20618;AC=4;AF=0.00019400523814142983;N_HET=4;N_HOMALT=0;N_HOMREF=10305;FREQ_HET=0.0003880104762828966;FREQ_HOMALT=0.0;FREQ_HOMREF=0.9996119895237171;N_BI_GENOS=10309;South_Han_AN=1496;South_Han_AC=1;South_H
chr6	151278053	.	T	<INS:ME:LINE1>	.	PASS	SVTYPE=INS:ME:LINE1;END=151278068;SVLEN=3296;AN=20618;AC=4;AF=0.00019400523814142983;N_HET=4;N_HOMALT=0;N_HOMREF=10305;FREQ_HET=0.0003880104762828966;FREQ_HOMALT=0.0;FREQ_HOMREF=0.9996119895237171;N_BI_GENOS=10309;Lingnan_Han_AN=1888;Lingnan_Han_AC=1;Lin
chr6	151776343	.	C	<INS:ME:LINE1>	.	PASS	SVTYPE=INS:ME:LINE1;END=151776354;SVLEN=6000;AN=20618;AC=1;AF=4.850130953535746e-05;N_HET=1;N_HOMALT=0;N_HOMREF=10308;FREQ_HET=9.700261907071492e-05;FREQ_HOMALT=0.0;FREQ_HOMREF=0.9999029973809292;N_BI_GENOS=10309;East_Han_AN=6554;East_Han_AC=1;East_Han_AF
chr6	151895756	.	G	<INS:ME:LINE1>	.	PASS	SVTYPE=INS:ME:LINE1;END=151895769;SVLEN=6069;AN=20618;AC=1;AF=4.850130953535746e-05;N_HET=1;N_HOMALT=0;N_HOMREF=10308;FREQ_HET=9.700261907071492e-05;FREQ_HOMALT=0.0;FREQ_HOMREF=0.9999029973809292;N_BI_GENOS=10309;Central_Han_AN=2128;Central_Han_AC=1;Centr
chr6	151943685	.	A	<INS:ME:LINE1>	.	PASS	SVTYPE=INS:ME:LINE1;END=151943699;SVLEN=5997;AN=20618;AC=15;AF=0.0007275196430303619;N_HET=13;N_HOMALT=1;N_HOMREF=10295;FREQ_HET=0.0012610340479192938;FREQ_HOMALT=9.700261907071492e-05;FREQ_HOMREF=0.99864196333301;N_BI_GENOS=10309;Northwest_Han_AN=1296;No
chr6	152015841	.	G	<INS:ME:LINE1>	.	PASS	SVTYPE=INS:ME:LINE1;END=152015849;SVLEN=278;AN=20618;AC=1;AF=4.850130953535746e-05;N_HET=1;N_HOMALT=0;N_HOMREF=10308;FREQ_HET=9.700261907071492e-05;FREQ_HOMALT=0.0;FREQ_HOMREF=0.9999029973809292;N_BI_GENOS=10309;South_Han_AN=1496;South_Han_AC=1;South_Han_
chr6	152161476	.	T	<INS:ME:LINE1>	.	PASS	SVTYPE=INS:ME:LINE1;END=152161492;SVLEN=5999;AN=20618;AC=1;AF=4.850130953535746e-05;N_HET=1;N_HOMALT=0;N_HOMREF=10308;FREQ_HET=9.700261907071492e-05;FREQ_HOMALT=0.0;FREQ_HOMREF=0.9999029973809292;N_BI_GENOS=10309;Hui_AN=444;Hui_AC=1;Hui_AF=0.0022522522522
chr6	152213475	.	A	<INS:ME:LINE1>	.	PASS	SVTYPE=INS:ME:LINE1;END=152213486;SVLEN=5995;AN=20618;AC=1;AF=4.850130953535746e-05;N_HET=1;N_HOMALT=0;N_HOMREF=10308;FREQ_HET=9.700261907071492e-05;FREQ_HOMALT=0.0;FREQ_HOMREF=0.9999029973809292;N_BI_GENOS=10309;East_Han_AN=6554;East_Han_AC=1;East_Han_AF
chr6	152375879	.	C	<INS:ME:LINE1>	.	PASS	SVTYPE=INS:ME:LINE1;END=152375895;SVLEN=6009;AN=20618;AC=1;AF=4.850130953535746e-05;N_HET=1;N_HOMALT=0;N_HOMREF=10308;FREQ_HET=9.700261907071492e-05;FREQ_HOMALT=0.0;FREQ_HOMREF=0.9999029973809292;N_BI_GENOS=10309;East_Han_AN=6554;East_Han_AC=1;East_Han_AF

chr6	152380363	.	G	<INS:ME:LINE1>	.	PASS	SVTYPE=INS:ME:LINE1;END=152380380;SVLEN=942;AN=20618;AC=4;AF=0.000194005238141429833;N_HET=4;N_HOMALT=0;N_HOMREF=10305;FREQ_HET=0.000388010476282859666;FREQ_HOMALT=0.0;FREQ_HOMREF=0.9996119895237171;N_BI_GENOS=10309;Tibetan_AN=414;Tibetan_AC=4;Tibetan_AF=0.
chr6	152508877	.	A	<INS:ME:LINE1>	.	PASS	SVTYPE=INS:ME:LINE1;END=152508896;SVLEN=516;AN=20618;AC=1;AF=4.850130953535746e-05;N_HET=1;N_HOMALT=0;N_HOMREF=10308;FREQ_HET=9.700261907071492e-05;FREQ_HOMALT=0.0;FREQ_HOMREF=0.9999029973809292;N_BI_GENOS=10309;North_Han_AN=2272;North_Han_AC=1;North_Han_
chr6	153040847	.	T	<INS:ME:LINE1>	.	PASS	SVTYPE=INS:ME:LINE1;END=153040859;SVLEN=2335;AN=20618;AC=1;AF=4.850130953535746e-05;N_HET=1;N_HOMALT=0;N_HOMREF=10308;FREQ_HET=9.700261907071492e-05;FREQ_HOMALT=0.0;FREQ_HOMREF=0.9999029973809292;N_BI_GENOS=10309;North_Han_AN=2272;North_Han_AC=1;North_Han
chr6	153059938	.	A	<INS:ME:LINE1>	.	PASS	SVTYPE=INS:ME:LINE1;END=153059953;SVLEN=777;AN=20618;AC=1;AF=4.850130953535746e-05;N_HET=1;N_HOMALT=0;N_HOMREF=10308;FREQ_HET=9.700261907071492e-05;FREQ_HOMALT=0.0;FREQ_HOMREF=0.9999029973809292;N_BI_GENOS=10309;South_Han_AN=1496;South_Han_AC=1;South_Han_
chr6	153235547	.	T	<INS:ME:LINE1>	.	PASS	SVTYPE=INS:ME:LINE1;END=153235547;SVLEN=1311;AN=20618;AC=1;AF=4.850130953535746e-05;N_HET=1;N_HOMALT=0;N_HOMREF=10308;FREQ_HET=9.700261907071492e-05;FREQ_HOMALT=0.0;FREQ_HOMREF=0.9999029973809292;N_BI_GENOS=10309;Northwest_Han_AN=1296;Northwest_Han_AC=1;N
chr6	153286273	.	C	<INS:ME:LINE1>	.	PASS	SVTYPE=INS:ME:LINE1;END=153286286;SVLEN=2389;AN=20618;AC=4;AF=0.00019400523814142983;N_HET=4;N_HOMALT=0;N_HOMREF=10305;FREQ_HET=0.000388010476282859666;FREQ_HOMALT=0.0;FREQ_HOMREF=0.9996119895237171;N_BI_GENOS=10309;Lingnan_Han_AN=1888;Lingnan_Han_AC=3;Lin
chr6	153623036	.	A	<INS:ME:LINE1>	.	PASS	SVTYPE=INS:ME:LINE1;END=153623053;SVLEN=6029;AN=20618;AC=2;AF=9.700261907071492e-05;N_HET=2;N_HOMALT=0;N_HOMREF=10307;FREQ_HET=0.00019400523814142983;FREQ_HOMALT=0.0;FREQ_HOMREF=0.9998059947618586;N_BI_GENOS=10309;Northwest_Han_AN=1296;Northwest_Han_AC=1;
chr6	153884495	.	A	<INS:ME:LINE1>	.	PASS	SVTYPE=INS:ME:LINE1;END=153884510;SVLEN=1792;AN=20618;AC=1;AF=4.850130953535746e-05;N_HET=1;N_HOMALT=0;N_HOMREF=10308;FREQ_HET=9.700261907071492e-05;FREQ_HOMALT=0.0;FREQ_HOMREF=0.9999029973809292;N_BI_GENOS=10309;East_Han_AN=6554;East_Han_AC=1;East_Han_AF
chr6	153992956	.	C	<INS:ME:LINE1>	.	PASS	SVTYPE=INS:ME:LINE1;END=153992974;SVLEN=1645;AN=20618;AC=1;AF=4.850130953535746e-05;N_HET=1;N_HOMALT=0;N_HOMREF=10308;FREQ_HET=9.700261907071492e-05;FREQ_HOMALT=0.0;FREQ_HOMREF=0.9999029973809292;N_BI_GENOS=10309;Southeast_Han_AN=1908;Southeast_Han_AC=1;S
chr6	154064146	.	T	<INS:ME:LINE1>	.	PASS	SVTYPE=INS:ME:LINE1;END=154064146;SVLEN=2718;AN=20618;AC=1;AF=4.850130953535746e-05;N_HET=1;N_HOMALT=0;N_HOMREF=10308;FREQ_HET=9.700261907071492e-05;FREQ_HOMALT=0.0;FREQ_HOMREF=0.9999029973809292;N_BI_GENOS=10309;Southeast_Han_AN=1908;Southeast_Han_AC=1;S
chr6	154096562	.	A	<INS:ME:LINE1>	.	PASS	SVTYPE=INS:ME:LINE1;END=154096578;SVLEN=1114;AN=20618;AC=1;AF=4.850130953535746e-05;N_HET=1;N_HOMALT=0;N_HOMREF=10308;FREQ_HET=9.700261907071492e-05;FREQ_HOMALT=0.0;FREQ_HOMREF=0.9999029973809292;N_BI_GENOS=10309;North_Han_AN=2272;North_Han_AC=1;North_Han
chr6	154182534	.	C	<INS:ME:LINE1>	.	PASS	SVTYPE=INS:ME:LINE1;END=154182545;SVLEN=5999;AN=20618;AC=114;AF=0.0055291492870307496;N_HET=112;N_HOMALT=1;N_HOMREF=10196;FREQ_HET=0.01086429333592007;FREQ_HOMALT=9.700261907071492e-05;FREQ_HOMREF=0.9890387040450093;N_BI_GENOS=10309;South_Han_AN=1496;Sout
chr6	154228376	.	A	<INS:ME:LINE1>	.	PASS	SVTYPE=INS:ME:LINE1;END=154228387;SVLEN=5998;AN=20618;AC=4;AF=0.00019400523814142983;N_HET=4;N_HOMALT=0;N_HOMREF=10305;FREQ_HET=0.000388010476282859666;FREQ_HOMALT=0.0;FREQ_HOMREF=0.9996119895237171;N_BI_GENOS=10309;East_Han_AN=6554;East_Han_AC=3;East_Han_
chr6	154251054	.	T	<INS:ME:LINE1>	.	PASS	SVTYPE=INS:ME:LINE1;END=154251069;SVLEN=6066;AN=20618;AC=1;AF=4.850130953535746e-05;N_HET=1;N_HOMALT=0;N_HOMREF=10308;FREQ_HET=9.700261907071492e-05;FREQ_HOMALT=0.0;FREQ_HOMREF=0.9999029973809292;N_BI_GENOS=10309;East_Han_AN=6554;East_Han_AC=1;East_Han_AF
chr6	154394618	.	A	<INS:ME:LINE1>	.	PASS	SVTYPE=INS:ME:LINE1;END=154394620;SVLEN=75;AN=20618;AC=1;AF=4.850130953535746e-05;N_HET=1;N_HOMALT=0;N_HOMREF=10308;FREQ_HET=9.700261907071492e-05;FREQ_HOMALT=0.0;FREQ_HOMREF=0.9999029973809292;N_BI_GENOS=10309;Lingnan_Han_AN=1888;Lingnan_Han_AC=1;Lingnan
chr6	154445632	.	A	<INS:ME:LINE1>	.	PASS	SVTYPE=INS:ME:LINE1;END=154445647;SVLEN=5998;AN=20618;AC=6;AF=0.00029100785721214447;N_HET=6;N_HOMALT=0;N_HOMREF=10303;FREQ_HET=0.0005820157144242894;FREQ_HOMALT=0.0;FREQ_HOMREF=0.9994179842855757;N_BI_GENOS=10309;Northwest_Han_AN=1296;Northwest_Han_AC=2;N
chr6	154595235	.	G	<INS:ME:LINE1>	.	PASS	SVTYPE=INS:ME:LINE1;END=154595245;SVLEN=6000;AN=20618;AC=3;AF=0.00014550392860607236;N_HET=1;N_HOMALT=1;N_HOMREF=10307;FREQ_HET=9.700261907071492e-05;FREQ_HOMALT=9.700261907071492e-05;FREQ_HOMREF=0.9998059947618586;N_BI_GENOS=10309;North_Han_AN=2272;North
chr6	155142232	.	A	<INS:ME:LINE1>	.	PASS	SVTYPE=INS:ME:LINE1;END=155142232;SVLEN=0;AN=20618;AC=1;AF=4.850130953535746e-05;N_HET=1;N_HOMALT=0;N_HOMREF=10308;FREQ_HET=9.700261907071492e-05;FREQ_HOMALT=0.0;FREQ_HOMREF=0.9999029973809292;N_BI_GENOS=10309;North_Han_AN=2272;North_Han_AC=1;North_Han_AF
chr6	156120818	.	A	<INS:ME:LINE1>	.	PASS	SVTYPE=INS:ME:LINE1;END=156120835;SVLEN=1251;AN=20618;AC=4;AF=0.00019400523814142983;N_HET=4;N_HOMALT=0;N_HOMREF=10305;FREQ_HET=0.000388010476282859666;FREQ_HOMALT=0.0;FREQ_HOMREF=0.9996119895237171;N_BI_GENOS=10309;Northwest_Han_AN=1296;Northwest_Han_AC=1
chr6	156130430	.	T	<INS:ME:LINE1>	.	PASS	SVTYPE=INS:ME:LINE1;END=156130447;SVLEN=849;AN=20618;AC=1;AF=4.850130953535746e-05;N_HET=1;N_HOMALT=0;N_HOMREF=10308;FREQ_HET=9.700261907071492e-05;FREQ_HOMALT=0.0;FREQ_HOMREF=0.9999029973809292;N_BI_GENOS=10309;Central_Han_AN=2128;Central_Han_AC=1;Centra
chr6	156296523	.	A	<INS:ME:LINE1>	.	PASS	SVTYPE=INS:ME:LINE1;END=156296537;SVLEN=5995;AN=20618;AC=3;AF=0.00014550392860607236;N_HET=3;N_HOMALT=0;N_HOMREF=10306;FREQ_HET=0.00029100785721214447;FREQ_HOMALT=0.0;FREQ_HOMREF=0.9997089921427879;N_BI_GENOS=10309;East_Han_AN=6554;East_Han_AC=3;East_Han_A
chr6	156344758	.	A	<INS:ME:LINE1>	.	PASS	SVTYPE=INS:ME:LINE1;END=156344773;SVLEN=5999;AN=20618;AC=3;AF=0.00014550392860607236;N_HET=3;N_HOMALT=0;N_HOMREF=10306;FREQ_HET=0.00029100785721214447;FREQ_HOMALT=0.0;FREQ_HOMREF=0.9997089921427879;N_BI_GENOS=10309;East_Han_AN=6554;East_Han_AC=3;East_Han_A
chr6	156442498	.	C	<INS:ME:LINE1>	.	PASS	SVTYPE=INS:ME:LINE1;END=156442514;SVLEN=6006;AN=20618;AC=1;AF=4.850130953535746e-05;N_HET=1;N_HOMALT=0;N_HOMREF=10308;FREQ_HET=9.700261907071492e-05;FREQ_HOMALT=0.0;FREQ_HOMREF=0.9999029973809292;N_BI_GENOS=10309;North_Han_AN=2272;North_Han_AC=1;North_Han
chr6	156740572	.	A	<INS:ME:LINE1>	.	PASS	SVTYPE=INS:ME:LINE1;END=156740590;SVLEN=5998;AN=20618;AC=1;AF=4.850130953535746e-05;N_HET=1;N_HOMALT=0;N_HOMREF=10308;FREQ_HET=9.700261907071492e-05;FREQ_HOMALT=0.0;FREQ_HOMREF=0.9999029973809292;N_BI_GENOS=10309;Southeast_Han_AN=1908;Southeast_Han_AC=1;S
chr6	156864144	.	A	<INS:ME:LINE1>	.	PASS	SVTYPE=INS:ME:LINE1;END=156864161;SVLEN=605;AN=20618;AC=1;AF=4.850130953535746e-05;N_HET=1;N_HOMALT=0;N_HOMREF=10308;FREQ_HET=9.700261907071492e-05;FREQ_HOMALT=0.0;FREQ_HOMREF=0.9999029973809292;N_BI_GENOS=10309;East_Han_AN=6554;East_Han_AC=1;East_Han_AF=
chr6	157130643	.	A	<INS:ME:LINE1>	.	PASS	SVTYPE=INS:ME:LINE1;END=157130654;SVLEN=146;AN=20618;AC=1;AF=4.850130953535746e-05;N_HET=1;N_HOMALT=0;N_HOMREF=10308;FREQ_HET=9.700261907071492e-05;FREQ_HOMALT=0.0;FREQ_HOMREF=0.9999029973809292;N_BI_GENOS=10309;Manchu_AN=416;Manchu_AC=1;Manchu_AF=0.00240
chr6	157547704	.	C	<INS:ME:LINE1>	.	PASS	SVTYPE=INS:ME:LINE1;END=157547704;SVLEN=5998;AN=20618;AC=3034;AF=0.1471529731302745;N_HET=1158;N_HOMALT=938;N_HOMREF=8213;FREQ_HET=0.11232903288388786;FREQ_HOMALT=0.09098845668833058;FREQ_HOMREF=0.7966825104277816;N_BI_GENOS=10309;South_Han_AN=1496;South_
chr6	157655917	.	T	<INS:ME:LINE1>	.	PASS	SVTYPE=INS:ME:LINE1;END=157655917;SVLEN=1555;AN=20618;AC=1;AF=4.850130953535746e-05;N_HET=1;N_HOMALT=0;N_HOMREF=10308;FREQ_HET=9.700261907071492e-05;FREQ_HOMALT=0.0;FREQ_HOMREF=0.9999029973809292;N_BI_GENOS=10309;South_Han_AN=1496;South_Han_AC=1;South_Han
chr6	157869076	.	C	<INS:ME:LINE1>	.	PASS	SVTYPE=INS:ME:LINE1;END=157869088;SVLEN=6004;AN=20618;AC=5;AF=0.00024250654767678728;N_HET=5;N_HOMALT=0;N_HOMREF=10304;FREQ_HET=0.00048501309535357455;FREQ_HOMALT=0.0;FREQ_HOMREF=0.9995149869046465;N_BI_GENOS=10309;Lingnan_Han_AN=1888;Lingnan_Han_AC=1;Lin
chr6	157905382	.	A	<INS:ME:LINE1>	.	PASS	SVTYPE=INS:ME:LINE1;END=157905387;SVLEN=461;AN=20618;AC=4;AF=0.00019400523814142983;N_HET=4;N_HOMALT=0;N_HOMREF=10305;FREQ_HET=0.000388010476282859666;FREQ_HOMALT=0.0;FREQ_HOMREF=0.9996119895237171;N_BI_GENOS=10309;East_Han_AN=6554;East_Han_AC=1;East_Han_A
chr6	159122658	.	T	<INS:ME:LINE1>	.	PASS	SVTYPE=INS:ME:LINE1;END=159122669;SVLEN=665;AN=20618;AC=1;AF=4.850130953535746e-05;N_HET=1;N_HOMALT=0;N_HOMREF=10308;FREQ_HET=9.700261907071492e-05;FREQ_HOMALT=0.0;FREQ_HOMREF=0.9999029973809292;N_BI_GENOS=10309;Lingnan_Han_AN=1888;Lingnan_Han_AC=1;Lingna
chr6	159153526	.	G	<INS:ME:LINE1>	.	PASS	SVTYPE=INS:ME:LINE1;END=159153526;SVLEN=6010;AN=20618;AC=1;AF=4.850130953535746e-05;N_HET=1;N_HOMALT=0;N_HOMREF=10308;FREQ_HET=9.700261907071492e-05;FREQ_HOMALT=0.0;FREQ_HOMREF=0.9999029973809292;N_BI_GENOS=10309;Lingnan_Han_AN=1888;Lingnan_Han_AC=1;Lingn
chr6	159416667	.	A	<INS:ME:LINE1>	.	PASS	SVTYPE=INS:ME:LINE1;END=159416684;SVLEN=5991;AN=20618;AC=9;AF=0.0004365117858182171;N_HET=9;N_HOMALT=0;N_HOMREF=10300;FREQ_HET=0.0008730235716364342;FREQ_HOMALT=0.0;FREQ_HOMREF=0.9991269764283636;N_BI_GENOS=10309;South_Han_AN=1496;South_Han_AC=1;South_Han
chr6	160074606	.	A	<INS:ME:LINE1>	.	PASS	SVTYPE=INS:ME:LINE1;END=160074614;SVLEN=288;AN=20618;AC=1;AF=4.850130953535746e-05;N_HET=1;N_HOMALT=0;N_HOMREF=10308;FREQ_HET=9.700261907071492e-05;FREQ_HOMALT=0.0;FREQ_HOMREF=0.9999029973809292;N_BI_GENOS=10309;East_Han_AN=6554;East_Han_AC=1;East_Han_AF=
chr6	160480668	.	A	<INS:ME:LINE1>	.	PASS	SVTYPE=INS:ME:LINE1;END=160480686;SVLEN=667;AN=20618;AC=33;AF=0.001600543214666796;N_HET=33;N_HOMALT=0;N_HOMREF=10276;FREQ_HET=0.003201086429333592;FREQ_HOMALT=0.0;FREQ_HOMREF=0.9967989135706664;N_BI_GENOS=10309;South_Han_AN=1496;South_Han_AC=5;South_Han_
chr6	160649326	.	T	<INS:ME:LINE1>	.	PASS	SVTYPE=INS:ME:LINE1;END=160649326;SVLEN=660;AN=20618;AC=1;AF=4.850130953535746e-05;N_HET=1;N_HOMALT=0;N_HOMREF=10308;FREQ_HET=9.700261907071492e-05;FREQ_HOMALT=0.0;FREQ_HOMREF=0.9999029973809292;N_BI_GENOS=10309;Lingnan_Han_AN=1888;Lingnan_Han_AC=1;Lingna
chr6	160833642	.	A	<INS:ME:LINE1>	.	PASS	SVTYPE=INS:ME:LINE1;END=160833655;SVLEN=5984;AN=20618;AC=1;AF=4.850130953535746e-05;N_HET=1;N_HOMALT=0;N_HOMREF=10308;FREQ_HET=9.700261907071492e-05;FREQ_HOMALT=0.0;FREQ_HOMREF=0.9999029973809292;N_BI_GENOS=10309;East_Han_AN=6554;East_Han_AC=1;East_Han_AF
chr6	161116061	.	A	<INS:ME:LINE1>	.	PASS	SVTYPE=INS:ME:LINE1;END=161116066;SVLEN=341;AN=20618;AC=2;AF=9.700261907071492e-05;N_HET=2;N_HOMALT=0;N_HOMREF=10307;FREQ_HET=0.00019400523814142983;FREQ_HOMALT=0.0;FREQ_HOMREF=0.9998059947618586;N_BI_GENOS=10309;East_Han_AN=6554;East_Han_AC=1;East_Han_AF
chr6	161233450	.	T	<INS:ME:LINE1>	.	PASS	SVTYPE=INS:ME:LINE1;END=161233463;SVLEN=5214;AN=20618;AC=1;AF=4.850130953535746e-05;N_HET=1;N_HOMALT=0;N_HOMREF=10308;FREQ_HET=9.700261907071492e-05;FREQ_HOMALT=0.0;FREQ_HOMREF=0.9999029973809292;N_BI_GENOS=10309;Mongolian_AN=454;Mongolian_AC=1;Mongolian_
chr6	161451021	.	C	<INS:ME:LINE1>	.	PASS	SVTYPE=INS:ME:LINE1;END=161451021;SVLEN=5999;AN=20618;AC=1;AF=4.850130953535746e-05;N_HET=1;N_HOMALT=0;N_HOMREF=10308;FREQ_HET=9.700261907071492e-05;FREQ_HOMALT=0.0;FREQ_HOMREF=0.9999029973809292;N_BI_GENOS=10309;Northwest_Han_AN=1296;Northwest_Han_AC=1;N
chr6	161577965	.	A	<INS:ME:LINE1>	.	PASS	SVTYPE=INS:ME:LINE1;END=161577972;SVLEN=2408;AN=20618;AC=2;AF=9.700261907071492e-05;N_HET=2;N_HOMALT=0;N_HOMREF=10307;FREQ_HET=0.00019400523814142983;FREQ_HOMALT=0.0;FREQ_HOMREF=0.9998059947618586;N_BI_GENOS=10309;Central_Han_AN=2128;Central_Han_AC=2;Cent
chr6	161627222	.	A	<INS:ME:LINE1>	.	PASS	SVTYPE=INS:ME:LINE1;END=161627236;SVLEN=858;AN=20618;AC=1;AF=4.850130953535746e-05;N_HET=1;N_HOMALT=0;N_HOMREF=10308;FREQ_HET=9.700261907071492e-05;FREQ_HOMALT=0.0;FREQ_HOMREF=0.9999029973809292;N_BI_GENOS=10309;Southeast_Han_AN=1908;Southeast_Han_AC=1;So
chr6	161701430	.	A	<INS:ME:LINE1>	.	PASS	SVTYPE=INS:ME:LINE1;END=161701430;SVLEN=1878;AN=20618;AC=1;AF=4.850130953535746e-05;N_HET=1;N_HOMALT=0;N_HOMREF=10308;FREQ_HET=9.700261907071492e-05;FREQ_HOMALT=0.0;FREQ_HOMREF=0.9999029973809292;N_BI_GENOS=10309;Northwest_Han_AN=1296;Northwest_Han_AC=1;N
chr6	161760224	.	G	<INS:ME:LINE1>	.	PASS	SVTYPE=INS:ME:LINE1;END=161760236;SVLEN=3896;AN=20618;AC=5217;AF=0.25303133184595983;N_HET=1151;N_HOMALT=2033;N_HOMREF=7125;FREQ_HET=0.11165001455039286;FREQ_HOMALT=0.1972063245707634;FREQ_HOMREF=0.6911436608788437;N_BI_GENOS=10309;South_Han_AN=1496;South
chr6	161865092	.	G	<INS:ME:LINE1>	.	PASS	SVTYPE=INS:ME:LINE1;END=161865107;SVLEN=6013;AN=20618;AC=1;AF=4.850130953535746e-05;N_HET=1;N_HOMALT=0;N_HOMREF=10308;FREQ_HET=9.700261907071492e-05;FREQ_HOMALT=0.0;FREQ_HOMREF=0.9999029973809292;N_BI_GENOS=10309;East_Han_AN=6554;East_Han_AC=1;East_Han_AF
chr6	161913248	.	A	<INS:ME:LINE1>	.	PASS	SVTYPE=INS:ME:LINE1;END=161913262;SVLEN=1838;AN=20618;AC=1;AF=4.850130953535746e-05;N_HET=1;N_HOMALT=0;N_HOMREF=10308;FREQ_HET=9.700261907071492e-05;FREQ_HOMALT=0.0;FREQ_HOMREF=0.9999029973809292;N_BI_GENOS=10309;Southeast_Han_AN=1908;Southeast_Han_AC=1;S
chr6	162364597	.	C	<INS:ME:LINE1>	.	PASS	SVTYPE=INS:ME:LINE1;END=162364597;SVLEN=586;AN=20618;AC=1;AF=4.850130953535746e-05;N_HET=1;N_HOMALT=0;N_HOMREF=10308;FREQ_HET=9.700261907071492e-05;FREQ_HOMALT=0.0;FREQ_HOMREF=0.9999029973809292;N_BI_GENOS=10309;Central_Han_AN=2128;Central_Han_AC=1;Centra
chr6	162642793	.	A	<INS:ME:LINE1>	.	PASS	SVTYPE=INS:ME:LINE1;END=162642793;SVLEN=421;AN=20618;AC=2;AF=9.700261907071492e-05;N_HET=2;N_HOMALT=0;N_HOMREF=10307;FREQ_HET=0.00019400523814142983;FREQ_HOMALT=0.0;FREQ_HOMREF=0.9998059947618586;N_BI_GENOS=10309;Central_Han_AN=2128;Central_Han_AC=1;Centr
chr6	162722584	.	A	<INS:ME:LINE1>	.	PASS	SVTYPE=INS:ME:LINE1;END=162722595;SVLEN=1540;AN=20618;AC=1;AF=4.850130953535746e-05;N_HET=1;N_HOMALT=0;N_HOMREF=10308;FREQ_HET=9.700261907071492e-05;FREQ_HOMALT=0.0;FREQ_HOMREF=0.9999029973809292;N_BI_GENOS=10309;South_Han_AN=1496;South_Han_AC=1;South_Han
chr6	162795603	.	A	<INS:ME:LINE1>	.	PASS	SVTYPE=INS:ME:LINE1;END=162795618;SVLEN=64;AN=20618;AC=1;AF=4.850130953535746e-05;N_HET=1;N_HOMALT=0;N_HOMREF=10308;FREQ_HET=9.700261907071492e-05;FREQ_HOMALT=0.0;FREQ_HOMREF=0.9999029973809292;N_BI_GENOS=10309;South_Han_AN=1496;South_Han_AC=1;South_Han_A
chr6	162818057	.	A	<INS:ME:LINE1>	.	PASS	SVTYPE=INS:ME:LINE1;END=162818066;SVLEN=5871;AN=20618;AC=1;AF=4.850130953535746e-05;N_HET=1;N_HOMALT=0;N_HOMREF=10308;FREQ_HET=9.700261907071492e-05;FREQ_HOMALT=0.0;FREQ_HOMREF=0.9999029973809292;N_BI_GENOS=10309;Southeast_Han_AN=1908;Southeast_Han_AC=1;S
chr6	162884584	.	A	<INS:ME:LINE1>	.	PASS	SVTYPE=INS:ME:LINE1;END=162884602;SVLEN=279;AN=20618;AC=1;AF=4.850130953535746e-05;N_HET=1;N_HOMALT=0;N_HOMREF=10308;FREQ_HET=9.700261907071492e-05;FREQ_HOMALT=0.0;FREQ_HOMREF=0.9999029973809292;N_BI_GENOS=10309;Central_Han_AN=2128;Central_Han_AC=1;Centra
chr6	162929358	.	A	<INS:ME:LINE1>	.	PASS	SVTYPE=INS:ME:LINE1;END=162929375;SVLEN=1529;AN=20618;AC=2;AF=9.700261907071492e-05;N_HET=2;N_HOMALT=0;N_HOMREF=10307;FREQ_HET=0.00019400523814142983;FREQ_HOMALT=0.0;FREQ_HOMREF=0.9998059947618586;N_BI_GENOS=10309;Northwest_Han_AN=1296;Northwest_Han_AC=1;
chr6	163210270	.	A	<INS:ME:LINE1>	.	PASS	SVTYPE=INS:ME:LINE1;END=163210285;SVLEN=6059;AN=20618;AC=2;AF=9.700261907071492e-05;N_HET=2;N_HOMALT=0;N_HOMREF=10307;FREQ_HET=0.00019400523814142983;FREQ_HOMALT=0.0;FREQ_HOMREF=0.9998059947618586;N_BI_GENOS=10309;East_Han_AN=6554;East_Han_AC=2;East_Han_A
chr6	163597969	.	G	<INS:ME:LINE1>	.	PASS	SVTYPE=INS:ME:LINE1;END=163597982;SVLEN=6005;AN=20618;AC=1;AF=4.850130953535746e-05;N_HET=1;N_HOMALT=0;N_HOMREF=10308;FREQ_HET=9.700261907071492e-05;FREQ_HOMALT=0.0;FREQ_HOMREF=0.9999029973809292;N_BI_GENOS=10309;Lingnan_Han_AN=1888;Lingnan_Han_AC=1;Lingn
chr6	164451967	.	T	<INS:ME:LINE1>	.	PASS	SVTYPE=INS:ME:LINE1;END=164451983;SVLEN=502;AN=20618;AC=2;AF=9.700261907071492e-05;N_HET=2;N_HOMALT=0;N_HOMREF=10307;FREQ_HET=0.00019400523814142983;FREQ_HOMALT=0.0;FREQ_HOMREF=0.9998059947618586;N_BI_GENOS=10309;Northwest_Han_AN=1296;Northwest_Han_AC=1;N
chr6	164675235	.	G	<INS:ME:LINE1>	.	PASS	SVTYPE=INS:ME:LINE1;END=164675243;SVLEN=857;AN=20618;AC=1;AF=4.850130953535746e-05;N_HET=1;N_HOMALT=0;N_HOMREF=10308;FREQ_HET=9.700261907071492e-05;FREQ_HOMALT=0.0;FREQ_HOMREF=0.9999029973809292;N_BI_GENOS=10309;Yi_AN=422;Yi_AC=1;Yi_AF=0.00236966824644549
chr6	164792470	.	A	<INS:ME:LINE1>	.	PASS	SVTYPE=INS:ME:LINE1;END=164792470;SVLEN=0;AN=20618;AC=1;AF=4.850130953535746e-05;N_HET=1;N_HOMALT=0;N_HOMREF=10308;FREQ_HET=9.700261907071492e-05;FREQ_HOMALT=0.0;FREQ_HOMREF=0.9999029973809292;N_BI_GENOS=10309;Central_Han_AN=2128;Central_Han_AC=1;Central_
chr6	167305742	.	C	<INS:ME:LINE1>	.	PASS	SVTYPE=INS:ME:LINE1;END=167305757;SVLEN=214;AN=20618;AC=38;AF=0.0018430497623435833;N_HET=34;N_HOMALT=2;N_HOMREF=10273;FREQ_HET=0.0032980890484043067;FREQ_HOMALT=0.00019400523814142983;FREQ_HOMREF=0.9965079057134543;N_BI_GENOS=10309;South_Han_AN=1496;Sout
chr6	167905801	.	G	<INS:ME:LINE1>	.	PASS	SVTYPE=INS:ME:LINE1;END=167905802;SVLEN=3365;AN=20618;AC=1;AF=4.850130953535746e-05;N_HET=1;N_HOMALT=0;N_HOMREF=10308;FREQ_HET=9.700261907071492e-05;FREQ_HOMALT=0.0;FREQ_HOMREF=0.9999029973809292;N_BI_GENOS=10309;Yi_AN=422;Yi_AC=1;Yi_AF=0.0023696682464454
chr6	168227031	.	A	<INS:ME:LINE1>	.	PASS	SVTYPE=INS:ME:LINE1;END=168227047;SVLEN=5990;AN=20618;AC=1;AF=4.850130953535746e-05;N_HET=1;N_HOMALT=0;N_HOMREF=10308;FREQ_HET=9.700261907071492e-05;FREQ_HOMALT=0.0;FREQ_HOMREF=0.9999029973809292;N_BI_GENOS=10309;East_Han_AN=6554;East_Han_AC=1;East_Han_AF
chr6	169527654	.	A	<INS:ME:LINE1>	.	PASS	SVTYPE=INS:ME:LINE1;END=169527668;SVLEN=3086;AN=20618;AC=1;AF=4.850130953535746e-05;N_HET=1;N_HOMALT=0;N_HOMREF=10308;FREQ_HET=9.700261907071492e-05;FREQ_HOMALT=0.0;FREQ_HOMREF=0.9999029973809292;N_BI_GENOS=10309;East_Han_AN=6554;East_Han_AC=1;East_Han_AF
chr6	169605108	.	A	<INS:ME:LINE1>	.	PASS	SVTYPE=INS:ME:LINE1;END=169605124;SVLEN=1988;AN=20618;AC=3;AF=0.00014550392860607236;N_HET=1;N_HOMALT=1;N_HOMREF=10307;FREQ_HET=9.700261907071492e-05;FREQ_HOMALT=9.700261907071492e-05;FREQ_HOMREF=0.9998059947618586;N_BI_GENOS=10309;East_Han_AN=6554;East_H
chr7	2364611	.	T	<INS:ME:LINE1>	.	PASS	SVTYPE=INS:ME:LINE1;END=2364615;SVLEN=4190;AN=20618;AC=3;AF=0.00014550392860607236;N_HET=3;N_HOMALT=0;N_HOMREF=10306;FREQ_HET=0.00029100785721214447;FREQ_HOMALT=0.0;FREQ_HOMREF=0.9997089921427879;N_BI_GENOS=10309;South_Han_AN=1496;South_Han_AC=1;South_Han_
chr7	3224384	.	A	<INS:ME:LINE1>	.	PASS	SVTYPE=INS:ME:LINE1;END=3224400;SVLEN=1229;AN=20618;AC=1;AF=4.850130953535746e-05;N_HET=1;N_HOMALT=0;N_HOMREF=10308;FREQ_HET=9.700261907071492e-05;FREQ_HOMALT=0.0;FREQ_HOMREF=0.9999029973809292;N_BI_GENOS=10309;Southeast_Han_AN=1908;Southeast_Han_AC=1;Sou
chr7	3325466	.	A	<INS:ME:LINE1>	.	PASS	SVTYPE=INS:ME:LINE1;END=3325474;SVLEN=2156;AN=20618;AC=1;AF=4.850130953535746e-05;N_HET=1;N_HOMALT=0;N_HOMREF=10308;FREQ_HET=9.700261907071492e-05;FREQ_HOMALT=0.0;FREQ_HOMREF=0.9999029973809292;N_BI_GENOS=10309;North_Han_AN=2272;North_Han_AC=1;North_Han_A
chr7	3353231	.	T	<INS:ME:LINE1>	.	PASS	SVTYPE=INS:ME:LINE1;END=3353246;SVLEN=638;AN=20618;AC=1;AF=4.850130953535746e-05;N_HET=1;N_HOMALT=0;N_HOMREF=10308;FREQ_HET=9.700261907071492e-05;FREQ_HOMALT=0.0;FREQ_HOMREF=0.9999029973809292;N_BI_GENOS=10309;Southeast_Han_AN=1908;Southeast_Han_AC=1;Sout
chr7	3379573	.	G	<INS:ME:LINE1>	.	PASS	SVTYPE=INS:ME:LINE1;END=3379584;SVLEN=336;AN=20618;AC=25;AF=0.0012125327383839363;N_HET=25;N_HOMALT=0;N_HOMREF=10284;FREQ_HET=0.0024250654767678727;FREQ_HOMALT=0.0;FREQ_HOMREF=0.9975749345232321;N_BI_GENOS=10309;South_Han_AN=1496;South_Han_AC=2;South_Han_
chr7	3470408	.	A	<INS:ME:LINE1>	.	PASS	SVTYPE=INS:ME:LINE1;END=3470425;SVLEN=3078;AN=20618;AC=1;AF=4.850130953535746e-05;N_HET=1;N_HOMALT=0;N_HOMREF=10308;FREQ_HET=9.700261907071492e-05;FREQ_HOMALT=0.0;FREQ_HOMREF=0.9999029973809292;N_BI_GENOS=10309;South_Han_AN=1496;South_Han_AC=1;South_Han_A
chr7	3550796	.	A	<INS:ME:LINE1>	.	PASS	SVTYPE=INS:ME:LINE1;END=3550812;SVLEN=5997;AN=20618;AC=1;AF=4.850130953535746e-05;N_HET=1;N_HOMALT=0;N_HOMREF=10308;FREQ_HET=9.700261907071492e-05;FREQ_HOMALT=0.0;FREQ_HOMREF=0.9999029973809292;N_BI_GENOS=10309;East_Han_AN=6554;East_Han_AC=1;East_Han_AF=0
chr7	3828634	.	A	<INS:ME:LINE1>	.	PASS	SVTYPE=INS:ME:LINE1;END=3828646;SVLEN=5998;AN=20618;AC=1;AF=4.850130953535746e-05;N_HET=1;N_HOMALT=0;N_HOMREF=10308;FREQ_HET=9.700261907071492e-05;FREQ_HOMALT=0.0;FREQ_HOMREF=0.9999029973809292;N_BI_GENOS=10309;North_Han_AN=2272;North_Han_AC=1;North_Han_A
chr7	4286980	.	A	<INS:ME:LINE1>	.	PASS	SVTYPE=INS:ME:LINE1;END=4286993;SVLEN=5997;AN=20618;AC=1;AF=4.850130953535746e-05;N_HET=1;N_HOMALT=0;N_HOMREF=10308;FREQ_HET=9.700261907071492e-05;FREQ_HOMALT=0.0;FREQ_HOMREF=0.9999029973809292;N_BI_GENOS=10309;East_Han_AN=6554;East_Han_AC=1;East_Han_AF=0
chr7	4297759	.	A	<INS:ME:LINE1>	.	PASS	SVTYPE=INS:ME:LINE1;END=4297774;SVLEN=944;AN=20618;AC=2;AF=9.700261907071492e-05;N_HET=2;N_HOMALT=0;N_HOMREF=10307;FREQ_HET=0.00019400523814142983;FREQ_HOMALT=0.0;FREQ_HOMREF=0.9998059947618586;N_BI_GENOS=10309;Mongolian_AN=454;Mongolian_AC=2;Mongolian_AF
chr7	4958695	.	A	<INS:ME:LINE1>	.	PASS	SVTYPE=INS:ME:LINE1;END=4958709;SVLEN=713;AN=20618;AC=1;AF=4.850130953535746e-05;N_HET=1;N_HOMALT=0;N_HOMREF=10308;FREQ_HET=9.700261907071492e-05;FREQ_HOMALT=0.0;FREQ_HOMREF=0.9999029973809292;N_BI_GENOS=10309;South_Han_AN=1496;South_Han_AC=1;South_Han_AF
chr7	5270373	.	A	<INS:ME:LINE1>	.	PASS	SVTYPE=INS:ME:LINE1;END=5270390;SVLEN=254;AN=20618;AC=4;AF=0.00019400523814142983;N_HET=4;N_HOMALT=0;N_HOMREF=10305;FREQ_HET=0.000388010476282859666;FREQ_HOMALT=0.0;FREQ_HOMREF=0.9996119895237171;N_BI_GENOS=10309;South_Han_AN=1496;South_Han_AC=1;South_Han_
chr7	6883515	.	C	<INS:ME:LINE1>	.	PASS	SVTYPE=INS:ME:LINE1;END=6883515;SVLEN=0;AN=20618;AC=1;AF=4.850130953535746e-05;N_HET=1;N_HOMALT=0;N_HOMREF=10308;FREQ_HET=9.700261907071492e-05;FREQ_HOMALT=0.0;FREQ_HOMREF=0.9999029973809292;N_BI_GENOS=10309;East_Han_AN=6554;East_Han_AC=1;East_Han_AF=0.00
chr7	7099511	.	C	<INS:ME:LINE1>	.	PASS	SVTYPE=INS:ME:LINE1;END=7099524;SVLEN=1115;AN=20618;AC=1;AF=4.850130953535746e-05;N_HET=1;N_HOMALT=0;N_HOMREF=10308;FREQ_HET=9.700261907071492e-05;FREQ_HOMALT=0.0;FREQ_HOMREF=0.9999029973809292;N_BI_GENOS=10309;East_Han_AN=6554;East_Han_AC=1;East_Han_AF=0
chr7	7734413	.	A	<INS:ME:LINE1>	.	PASS	SVTYPE=INS:ME:LINE1;END=7734428;SVLEN=460;AN=20618;AC=1;AF=4.850130953535746e-05;N_HET=1;N_HOMALT=0;N_HOMREF=10308;FREQ_HET=9.700261907071492e-05;FREQ_HOMALT=0.0;FREQ_HOMREF=0.9999029973809292;N_BI_GENOS=10309;North_Han_AN=2272;North_Han_AC=1;North_Han_AF
chr7	7834652	.	A	<INS:ME:LINE1>	.	PASS	SVTYPE=INS:ME:LINE1;END=7834663;SVLEN=74;AN=20618;AC=64;AF=0.0031040838102628773;N_HET=64;N_HOMALT=0;N_HOMREF=10245;FREQ_HET=0.006208167620525755;FREQ_HOMALT=0.0;FREQ_HOMREF=0.9937918323794742;N_BI_GENOS=10309;South_Han_AN=1496;South_Han_AC=6;South_Han_AF
chr7	7979396	.	C	<INS:ME:LINE1>	.	PASS	SVTYPE=INS:ME:LINE1;END=7979396;SVLEN=6000;AN=20618;AC=6;AF=0.00029100785721214447;N_HET=6;N_HOMALT=0;N_HOMREF=10303;FREQ_HET=0.0005820157144242894;FREQ_HOMALT=0.0;FREQ_HOMREF=0.9994179842855757;N_BI_GENOS=10309;Northwest_Han_AN=1296;Northwest_Han_AC=3;Nor
chr7	8181761	.	A	<INS:ME:LINE1>	.	PASS	SVTYPE=INS:ME:LINE1;END=8181778;SVLEN=3887;AN=20618;AC=2;AF=9.700261907071492e-05;N_HET=2;N_HOMALT=0;N_HOMREF=10307;FREQ_HET=0.00019400523814142983;FREQ_HOMALT=0.0;FREQ_HOMREF=0.9998059947618586;N_BI_GENOS=10309;East_Han_AN=6554;East_Han_AC=1;East_Han_AF=
chr7	8555647	.	C	<INS:ME:LINE1>	.	PASS	SVTYPE=INS:ME:LINE1;END=8555665;SVLEN=6005;AN=20618;AC=1;AF=4.850130953535746e-05;N_HET=1;N_HOMALT=0;N_HOMREF=10308;FREQ_HET=9.700261907071492e-05;FREQ_HOMALT=0.0;FREQ_HOMREF=0.9999029973809292;N_BI_GENOS=10309;Hui_AN=444;Hui_AC=1;Hui_AF=0.002252252252252
chr7	8597152	.	T	<INS:ME:LINE1>	.	PASS	SVTYPE=INS:ME:LINE1;END=8597166;SVLEN=6005;AN=20618;AC=1;AF=4.850130953535746e-05;N_HET=1;N_HOMALT=0;N_HOMREF=10308;FREQ_HET=9.700261907071492e-05;FREQ_HOMALT=0.0;FREQ_HOMREF=0.9999029973809292;N_BI_GENOS=10309;Southeast_Han_AN=1908;Southeast_Han_AC=1;Sou
chr7	8839243	.	G	<INS:ME:LINE1>	.	PASS	SVTYPE=INS:ME:LINE1;END=8839258;SVLEN=1164;AN=20618;AC=1;AF=4.850130953535746e-05;N_HET=1;N_HOMALT=0;N_HOMREF=10308;FREQ_HET=9.700261907071492e-05;FREQ_HOMALT=0.0;FREQ_HOMREF=0.9999029973809292;N_BI_GENOS=10309;East_Han_AN=6554;East_Han_AC=1;East_Han_AF=0
chr7	8847074	.	A	<INS:ME:LINE1>	.	PASS	SVTYPE=INS:ME:LINE1;END=8847085;SVLEN=5998;AN=20618;AC=5717;AF=0.27728198661363856;N_HET=2829;N_HOMALT=1444;N_HOMREF=6036;FREQ_HET=0.2744204093510525;FREQ_HOMALT=0.14007178193811232;FREQ_HOMREF=0.5855078087108352;N_BI_GENOS=10309;South_Han_AN=1496;South_H
chr7	8898955	.	A	<INS:ME:LINE1>	.	PASS	SVTYPE=INS:ME:LINE1;END=8898955;SVLEN=655;AN=20618;AC=1;AF=4.850130953535746e-05;N_HET=1;N_HOMALT=0;N_HOMREF=10308;FREQ_HET=9.700261907071492e-05;FREQ_HOMALT=0.0;FREQ_HOMREF=0.9999029973809292;N_BI_GENOS=10309;East_Han_AN=6554;East_Han_AC=1;East_Han_AF=0.
chr7	9321987	.	C	<INS:ME:LINE1>	.	PASS	SVTYPE=INS:ME:LINE1;END=9322009;SVLEN=552;AN=20618;AC=2;AF=9.700261907071492e-05;N_HET=0;N_HOMALT=1;N_HOMREF=10308;FREQ_HET=0.0;FREQ_HOMALT=9.700261907071492e-05;FREQ_HOMREF=0.9999029973809292;N_BI_GENOS=10309;North_Han_AN=2272;North_Han_AC=2;North_Han_AF
chr7	9489469	.	T	<INS:ME:LINE1>	.	PASS	SVTYPE=INS:ME:LINE1;END=9489469;SVLEN=73;AN=20618;AC=1;AF=4.850130953535746e-05;N_HET=1;N_HOMALT=0;N_HOMREF=10308;FREQ_HET=9.700261907071492e-05;FREQ_HOMALT=0.0;FREQ_HOMREF=0.9999029973809292;N_BI_GENOS=10309;East_Han_AN=6554;East_Han_AC=1;East_Han_AF=0.0
chr7	9591866	.	G	<INS:ME:LINE1>	.	PASS	SVTYPE=INS:ME:LINE1;END=9591866;SVLEN=811;AN=20618;AC=1;AF=4.850130953535746e-05;N_HET=1;N_HOMALT=0;N_HOMREF=10308;FREQ_HET=9.700261907071492e-05;FREQ_HOMALT=0.0;FREQ_HOMREF=0.9999029973809292;N_BI_GENOS=10309;East_Han_AN=6554;East_Han_AC=1;East_Han_AF=0.
chr7	9611342	.	A	<INS:ME:LINE1>	.	PASS	SVTYPE=INS:ME:LINE1;END=9611357;SVLEN=891;AN=20618;AC=3;AF=0.00014550392860607236;N_HET=1;N_HOMALT=1;N_HOMREF=10307;FREQ_HET=9.700261907071492e-05;FREQ_HOMALT=9.700261907071492e-05;FREQ_HOMREF=0.9998059947618586;N_BI_GENOS=10309;Central_Han_AN=2128;Centra
chr7	9654539	.	A	<INS:ME:LINE1>	.	PASS	SVTYPE=INS:ME:LINE1;END=9654557;SVLEN=1692;AN=20618;AC=6;AF=0.00029100785721214447;N_HET=6;N_HOMALT=0;N_HOMREF=10303;FREQ_HET=0.0005820157144242894;FREQ_HOMALT=0.0;FREQ_HOMREF=0.9994179842855757;N_BI_GENOS=10309;South_Han_AN=1496;South_Han_AC=1;South_Han_A
chr7	9736461	.	A	<INS:ME:LINE1>	.	PASS	SVTYPE=INS:ME:LINE1;END=9736461;SVLEN=1898;AN=20618;AC=1;AF=4.850130953535746e-05;N_HET=1;N_HOMALT=0;N_HOMREF=10308;FREQ_HET=9.700261907071492e-05;FREQ_HOMALT=0.0;FREQ_HOMREF=0.9999029973809292;N_BI_GENOS=10309;North_Han_AN=2272;North_Han_AC=1;North_Han_A
chr7	9858298	.	T	<INS:ME:LINE1>	.	PASS	SVTYPE=INS:ME:LINE1;END=9858312;SVLEN=5999;AN=20618;AC=1;AF=4.850130953535746e-05;N_HET=1;N_HOMALT=0;N_HOMREF=10308;FREQ_HET=9.700261907071492e-05;FREQ_HOMALT=0.0;FREQ_HOMREF=0.9999029973809292;N_BI_GENOS=10309;North_Han_AN=2272;North_Han_AC=1;North_Han_A
chr7	10225403	.	T	<INS:ME:LINE1>	.	PASS	SVTYPE=INS:ME:LINE1;END=10225420;SVLEN=955;AN=20618;AC=19;AF=0.0009215248811717916;N_HET=19;N_HOMALT=0;N_HOMREF=10290;FREQ_HET=0.0018430497623435833;FREQ_HOMALT=0.0;FREQ_HOMREF=0.9981569502376564;N_BI_GENOS=10309;South_Han_AN=1496;South_Han_AC=7;South_Han
chr7	10287824	.	A	<INS:ME:LINE1>	.	PASS	SVTYPE=INS:ME:LINE1;END=10287849;SVLEN=605;AN=20618;AC=2;AF=9.700261907071492e-05;N_HET=2;N_HOMALT=0;N_HOMREF=10307;FREQ_HET=0.00019400523814142983;FREQ_HOMALT=0.0;FREQ_HOMREF=0.9998059947618586;N_BI_GENOS=10309;Lingnan_Han_AN=1888;Lingnan_Han_AC=1;Lingna
chr7	10296208	.	A	<INS:ME:LINE1>	.	PASS	SVTYPE=INS:ME:LINE1;END=10296224;SVLEN=1304;AN=20618;AC=2;AF=9.700261907071492e-05;N_HET=2;N_HOMALT=0;N_HOMREF=10307;FREQ_HET=0.00019400523814142983;FREQ_HOMALT=0.0;FREQ_HOMREF=0.9998059947618586;N_BI_GENOS=10309;East_Han_AN=6554;East_Han_AC=1;East_Han_AF

chr7	10447208	.	C	<INS:ME:LINE1>	.	PASS	SVTYPE=INS:ME:LINE1;END=10447245;SVLEN=1740;AN=20618;AC=1;AF=4.850130953535746e-05;N_HET=1;N_HOMALT=0;N_HOMREF=10308;FREQ_HET=9.700261907071492e-05;FREQ_HOMALT=0.0;FREQ_HOMREF=0.9999029973809292;N_BI_GENOS=10309;East_Han_AN=6554;East_Han_AC=1;East_Han_AF=
chr7	10447516	.	C	<INS:ME:LINE1>	.	PASS	SVTYPE=INS:ME:LINE1;END=10447516;SVLEN=770;AN=20618;AC=1;AF=4.850130953535746e-05;N_HET=1;N_HOMALT=0;N_HOMREF=10308;FREQ_HET=9.700261907071492e-05;FREQ_HOMALT=0.0;FREQ_HOMREF=0.9999029973809292;N_BI_GENOS=10309;East_Han_AN=6554;East_Han_AC=1;East_Han_AF=0
chr7	10448507	.	A	<INS:ME:LINE1>	.	PASS	SVTYPE=INS:ME:LINE1;END=10448507;SVLEN=570;AN=20618;AC=1;AF=4.850130953535746e-05;N_HET=1;N_HOMALT=0;N_HOMREF=10308;FREQ_HET=9.700261907071492e-05;FREQ_HOMALT=0.0;FREQ_HOMREF=0.9999029973809292;N_BI_GENOS=10309;East_Han_AN=6554;East_Han_AC=1;East_Han_AF=0
chr7	10491073	.	T	<INS:ME:LINE1>	.	PASS	SVTYPE=INS:ME:LINE1;END=10491090;SVLEN=1076;AN=20618;AC=1;AF=4.850130953535746e-05;N_HET=1;N_HOMALT=0;N_HOMREF=10308;FREQ_HET=9.700261907071492e-05;FREQ_HOMALT=0.0;FREQ_HOMREF=0.9999029973809292;N_BI_GENOS=10309;Southeast_Han_AN=1908;Southeast_Han_AC=1;So
chr7	10674519	.	A	<INS:ME:LINE1>	.	PASS	SVTYPE=INS:ME:LINE1;END=10674519;SVLEN=864;AN=20618;AC=2;AF=9.700261907071492e-05;N_HET=2;N_HOMALT=0;N_HOMREF=10307;FREQ_HET=0.00019400523814142983;FREQ_HOMALT=0.0;FREQ_HOMREF=0.9998059947618586;N_BI_GENOS=10309;Northwest_Han_AN=1296;Northwest_Han_AC=2;No
chr7	10679377	.	A	<INS:ME:LINE1>	.	PASS	SVTYPE=INS:ME:LINE1;END=10679380;SVLEN=226;AN=20618;AC=1;AF=4.850130953535746e-05;N_HET=1;N_HOMALT=0;N_HOMREF=10308;FREQ_HET=9.700261907071492e-05;FREQ_HOMALT=0.0;FREQ_HOMREF=0.9999029973809292;N_BI_GENOS=10309;North_Han_AN=2272;North_Han_AC=1;North_Han_A
chr7	10715663	.	T	<INS:ME:LINE1>	.	PASS	SVTYPE=INS:ME:LINE1;END=10715678;SVLEN=827;AN=20618;AC=1;AF=4.850130953535746e-05;N_HET=1;N_HOMALT=0;N_HOMREF=10308;FREQ_HET=9.700261907071492e-05;FREQ_HOMALT=0.0;FREQ_HOMREF=0.9999029973809292;N_BI_GENOS=10309;East_Han_AN=6554;East_Han_AC=1;East_Han_AF=0
chr7	10727171	.	A	<INS:ME:LINE1>	.	PASS	SVTYPE=INS:ME:LINE1;END=10727186;SVLEN=6000;AN=20618;AC=1;AF=4.850130953535746e-05;N_HET=1;N_HOMALT=0;N_HOMREF=10308;FREQ_HET=9.700261907071492e-05;FREQ_HOMALT=0.0;FREQ_HOMREF=0.9999029973809292;N_BI_GENOS=10309;East_Han_AN=6554;East_Han_AC=1;East_Han_AF=
chr7	10872878	.	A	<INS:ME:LINE1>	.	PASS	SVTYPE=INS:ME:LINE1;END=10872896;SVLEN=471;AN=20618;AC=1;AF=4.850130953535746e-05;N_HET=1;N_HOMALT=0;N_HOMREF=10308;FREQ_HET=9.700261907071492e-05;FREQ_HOMALT=0.0;FREQ_HOMREF=0.9999029973809292;N_BI_GENOS=10309;East_Han_AN=6554;East_Han_AC=1;East_Han_AF=0
chr7	11155168	.	G	<INS:ME:LINE1>	.	PASS	SVTYPE=INS:ME:LINE1;END=11155168;SVLEN=9;AN=20618;AC=1;AF=4.850130953535746e-05;N_HET=1;N_HOMALT=0;N_HOMREF=10308;FREQ_HET=9.700261907071492e-05;FREQ_HOMALT=0.0;FREQ_HOMREF=0.9999029973809292;N_BI_GENOS=10309;Southeast_Han_AN=1908;Southeast_Han_AC=1;South
chr7	11266704	.	T	<INS:ME:LINE1>	.	PASS	SVTYPE=INS:ME:LINE1;END=11266704;SVLEN=715;AN=20618;AC=1;AF=4.850130953535746e-05;N_HET=1;N_HOMALT=0;N_HOMREF=10308;FREQ_HET=9.700261907071492e-05;FREQ_HOMALT=0.0;FREQ_HOMREF=0.9999029973809292;N_BI_GENOS=10309;East_Han_AN=6554;East_Han_AC=1;East_Han_AF=0
chr7	11429544	.	A	<INS:ME:LINE1>	.	PASS	SVTYPE=INS:ME:LINE1;END=11429559;SVLEN=1009;AN=20618;AC=1;AF=4.850130953535746e-05;N_HET=1;N_HOMALT=0;N_HOMREF=10308;FREQ_HET=9.700261907071492e-05;FREQ_HOMALT=0.0;FREQ_HOMREF=0.9999029973809292;N_BI_GENOS=10309;Southeast_Han_AN=1908;Southeast_Han_AC=1;So
chr7	11731562	.	C	<INS:ME:LINE1>	.	PASS	SVTYPE=INS:ME:LINE1;END=11731578;SVLEN=5274;AN=20618;AC=1;AF=4.850130953535746e-05;N_HET=1;N_HOMALT=0;N_HOMREF=10308;FREQ_HET=9.700261907071492e-05;FREQ_HOMALT=0.0;FREQ_HOMREF=0.9999029973809292;N_BI_GENOS=10309;Central_Han_AN=2128;Central_Han_AC=1;Centra
chr7	11876827	.	A	<INS:ME:LINE1>	.	PASS	SVTYPE=INS:ME:LINE1;END=11876844;SVLEN=566;AN=20618;AC=1;AF=4.850130953535746e-05;N_HET=1;N_HOMALT=0;N_HOMREF=10308;FREQ_HET=9.700261907071492e-05;FREQ_HOMALT=0.0;FREQ_HOMREF=0.9999029973809292;N_BI_GENOS=10309;Manchu_AN=416;Manchu_AC=1;Manchu_AF=0.002403
chr7	11901215	.	T	<INS:ME:LINE1>	.	PASS	SVTYPE=INS:ME:LINE1;END=11901229;SVLEN=938;AN=20618;AC=1;AF=4.850130953535746e-05;N_HET=1;N_HOMALT=0;N_HOMREF=10308;FREQ_HET=9.700261907071492e-05;FREQ_HOMALT=0.0;FREQ_HOMREF=0.9999029973809292;N_BI_GENOS=10309;Hui_AN=444;Hui_AC=1;Hui_AF=0.002252252252252
chr7	12543172	.	A	<INS:ME:LINE1>	.	PASS	SVTYPE=INS:ME:LINE1;END=12543188;SVLEN=6085;AN=20618;AC=1;AF=4.850130953535746e-05;N_HET=1;N_HOMALT=0;N_HOMREF=10308;FREQ_HET=9.700261907071492e-05;FREQ_HOMALT=0.0;FREQ_HOMREF=0.9999029973809292;N_BI_GENOS=10309;Lingnan_Han_AN=1888;Lingnan_Han_AC=1;Lingna
chr7	12685527	.	A	<INS:ME:LINE1>	.	PASS	SVTYPE=INS:ME:LINE1;END=12685535;SVLEN=965;AN=20618;AC=8;AF=0.00038801047628285966;N_HET=6;N_HOMALT=1;N_HOMREF=10302;FREQ_HET=0.0005820157144242894;FREQ_HOMALT=9.700261907071492e-05;FREQ_HOMREF=0.999320981666505;N_BI_GENOS=10309;Northwest_Han_AN=1296;Nort
chr7	13206778	.	T	<INS:ME:LINE1>	.	PASS	SVTYPE=INS:ME:LINE1;END=13206778;SVLEN=2474;AN=20618;AC=1;AF=4.850130953535746e-05;N_HET=1;N_HOMALT=0;N_HOMREF=10308;FREQ_HET=9.700261907071492e-05;FREQ_HOMALT=0.0;FREQ_HOMREF=0.9999029973809292;N_BI_GENOS=10309;East_Han_AN=6554;East_Han_AC=1;East_Han_AF=
chr7	13298637	.	G	<INS:ME:LINE1>	.	PASS	SVTYPE=INS:ME:LINE1;END=13298637;SVLEN=1718;AN=20618;AC=1;AF=4.850130953535746e-05;N_HET=1;N_HOMALT=0;N_HOMREF=10308;FREQ_HET=9.700261907071492e-05;FREQ_HOMALT=0.0;FREQ_HOMREF=0.9999029973809292;N_BI_GENOS=10309;East_Han_AN=6554;East_Han_AC=1;East_Han_AF=
chr7	13343572	.	C	<INS:ME:LINE1>	.	PASS	SVTYPE=INS:ME:LINE1;END=13343585;SVLEN=2449;AN=20618;AC=6;AF=0.0002910078572121447;N_HET=6;N_HOMALT=0;N_HOMREF=10303;FREQ_HET=0.0005820157144242894;FREQ_HOMALT=0.0;FREQ_HOMREF=0.9994179842855757;N_BI_GENOS=10309;North_Han_AN=2272;North_Han_AC=1;North_Han_
chr7	14193981	.	G	<INS:ME:LINE1>	.	PASS	SVTYPE=INS:ME:LINE1;END=14193997;SVLEN=5998;AN=20618;AC=1;AF=4.850130953535746e-05;N_HET=1;N_HOMALT=0;N_HOMREF=10308;FREQ_HET=9.700261907071492e-05;FREQ_HOMALT=0.0;FREQ_HOMREF=0.9999029973809292;N_BI_GENOS=10309;North_Han_AN=2272;North_Han_AC=1;North_Han_
chr7	14334654	.	G	<INS:ME:LINE1>	.	PASS	SVTYPE=INS:ME:LINE1;END=14334669;SVLEN=5946;AN=20618;AC=1;AF=4.850130953535746e-05;N_HET=1;N_HOMALT=0;N_HOMREF=10308;FREQ_HET=9.700261907071492e-05;FREQ_HOMALT=0.0;FREQ_HOMREF=0.9999029973809292;N_BI_GENOS=10309;Lingnan_Han_AN=1888;Lingnan_Han_AC=1;Lingna
chr7	15148989	.	C	<INS:ME:LINE1>	.	PASS	SVTYPE=INS:ME:LINE1;END=15148989;SVLEN=302;AN=20618;AC=1;AF=4.850130953535746e-05;N_HET=1;N_HOMALT=0;N_HOMREF=10308;FREQ_HET=9.700261907071492e-05;FREQ_HOMALT=0.0;FREQ_HOMREF=0.9999029973809292;N_BI_GENOS=10309;East_Han_AN=6554;East_Han_AC=1;East_Han_AF=0
chr7	15306041	.	A	<INS:ME:LINE1>	.	PASS	SVTYPE=INS:ME:LINE1;END=15306057;SVLEN=667;AN=20618;AC=6;AF=0.0002910078572121447;N_HET=4;N_HOMALT=1;N_HOMREF=10304;FREQ_HET=0.00038801047628285966;FREQ_HOMALT=9.700261907071492e-05;FREQ_HOMREF=0.9995149869046465;N_BI_GENOS=10309;South_Han_AN=1496;South_H
chr7	15345123	.	T	<INS:ME:LINE1>	.	PASS	SVTYPE=INS:ME:LINE1;END=15345136;SVLEN=6015;AN=20618;AC=1;AF=4.850130953535746e-05;N_HET=1;N_HOMALT=0;N_HOMREF=10308;FREQ_HET=9.700261907071492e-05;FREQ_HOMALT=0.0;FREQ_HOMREF=0.9999029973809292;N_BI_GENOS=10309;Yi_AN=422;Yi_AC=1;Yi_AF=0.00236966824644549
chr7	15445812	.	T	<INS:ME:LINE1>	.	PASS	SVTYPE=INS:ME:LINE1;END=15445814;SVLEN=1004;AN=20618;AC=2;AF=9.700261907071492e-05;N_HET=2;N_HOMALT=0;N_HOMREF=10307;FREQ_HET=0.00019400523814142983;FREQ_HOMALT=0.0;FREQ_HOMREF=0.9998059947618586;N_BI_GENOS=10309;Northwest_Han_AN=1296;Northwest_Han_AC=1;N
chr7	15456286	.	A	<INS:ME:LINE1>	.	PASS	SVTYPE=INS:ME:LINE1;END=15456298;SVLEN=241;AN=20618;AC=1;AF=4.850130953535746e-05;N_HET=1;N_HOMALT=0;N_HOMREF=10308;FREQ_HET=9.700261907071492e-05;FREQ_HOMALT=0.0;FREQ_HOMREF=0.9999029973809292;N_BI_GENOS=10309;East_Han_AN=6554;East_Han_AC=1;East_Han_AF=0
chr7	15483146	.	A	<INS:ME:LINE1>	.	PASS	SVTYPE=INS:ME:LINE1;END=15483162;SVLEN=617;AN=20618;AC=1;AF=4.850130953535746e-05;N_HET=1;N_HOMALT=0;N_HOMREF=10308;FREQ_HET=9.700261907071492e-05;FREQ_HOMALT=0.0;FREQ_HOMREF=0.9999029973809292;N_BI_GENOS=10309;East_Han_AN=6554;East_Han_AC=1;East_Han_AF=0
chr7	15725845	.	A	<INS:ME:LINE1>	.	PASS	SVTYPE=INS:ME:LINE1;END=15725859;SVLEN=270;AN=20618;AC=1;AF=4.850130953535746e-05;N_HET=1;N_HOMALT=0;N_HOMREF=10308;FREQ_HET=9.700261907071492e-05;FREQ_HOMALT=0.0;FREQ_HOMREF=0.9999029973809292;N_BI_GENOS=10309;Southeast_Han_AN=1908;Southeast_Han_AC=1;Sou
chr7	16098591	.	A	<INS:ME:LINE1>	.	PASS	SVTYPE=INS:ME:LINE1;END=16098607;SVLEN=899;AN=20618;AC=2;AF=9.700261907071492e-05;N_HET=2;N_HOMALT=0;N_HOMREF=10307;FREQ_HET=0.00019400523814142983;FREQ_HOMALT=0.0;FREQ_HOMREF=0.9998059947618586;N_BI_GENOS=10309;South_Han_AN=1496;South_Han_AC=2;South_Han_
chr7	16260999	.	T	<INS:ME:LINE1>	.	PASS	SVTYPE=INS:ME:LINE1;END=16260999;SVLEN=4570;AN=20618;AC=1;AF=4.850130953535746e-05;N_HET=1;N_HOMALT=0;N_HOMREF=10308;FREQ_HET=9.700261907071492e-05;FREQ_HOMALT=0.0;FREQ_HOMREF=0.9999029973809292;N_BI_GENOS=10309;Lingnan_Han_AN=1888;Lingnan_Han_AC=1;Lingna
chr7	16284516	.	C	<INS:ME:LINE1>	.	PASS	SVTYPE=INS:ME:LINE1;END=16284534;SVLEN=539;AN=20618;AC=1;AF=4.850130953535746e-05;N_HET=1;N_HOMALT=0;N_HOMREF=10308;FREQ_HET=9.700261907071492e-05;FREQ_HOMALT=0.0;FREQ_HOMREF=0.9999029973809292;N_BI_GENOS=10309;Central_Han_AN=2128;Central_Han_AC=1;Central
chr7	16364319	.	T	<INS:ME:LINE1>	.	PASS	SVTYPE=INS:ME:LINE1;END=16364336;SVLEN=5998;AN=20618;AC=1;AF=4.850130953535746e-05;N_HET=1;N_HOMALT=0;N_HOMREF=10308;FREQ_HET=9.700261907071492e-05;FREQ_HOMALT=0.0;FREQ_HOMREF=0.9999029973809292;N_BI_GENOS=10309;East_Han_AN=6554;East_Han_AC=1;East_Han_AF=
chr7	16432760	.	T	<INS:ME:LINE1>	.	PASS	SVTYPE=INS:ME:LINE1;END=16432773;SVLEN=5998;AN=20618;AC=1;AF=4.850130953535746e-05;N_HET=1;N_HOMALT=0;N_HOMREF=10308;FREQ_HET=9.700261907071492e-05;FREQ_HOMALT=0.0;FREQ_HOMREF=0.9999029973809292;N_BI_GENOS=10309;Lingnan_Han_AN=1888;Lingnan_Han_AC=1;Lingna
chr7	16503573	.	A	<INS:ME:LINE1>	.	PASS	SVTYPE=INS:ME:LINE1;END=16503588;SVLEN=2152;AN=20618;AC=1;AF=4.850130953535746e-05;N_HET=1;N_HOMALT=0;N_HOMREF=10308;FREQ_HET=9.700261907071492e-05;FREQ_HOMALT=0.0;FREQ_HOMREF=0.9999029973809292;N_BI_GENOS=10309;Central_Han_AN=2128;Central_Han_AC=1;Centra
chr7	17013826	.	A	<INS:ME:LINE1>	.	PASS	SVTYPE=INS:ME:LINE1;END=17013826;SVLEN=110;AN=20618;AC=1;AF=4.850130953535746e-05;N_HET=1;N_HOMALT=0;N_HOMREF=10308;FREQ_HET=9.700261907071492e-05;FREQ_HOMALT=0.0;FREQ_HOMREF=0.9999029973809292;N_BI_GENOS=10309;Northwest_Han_AN=1296;Northwest_Han_AC=1;Nor
chr7	17014324	.	A	<INS:ME:LINE1>	.	PASS	SVTYPE=INS:ME:LINE1;END=17014334;SVLEN=6000;AN=20618;AC=2;AF=9.700261907071492e-05;N_HET=2;N_HOMALT=0;N_HOMREF=10307;FREQ_HET=0.00019400523814142983;FREQ_HOMALT=0.0;FREQ_HOMREF=0.9998059947618586;N_BI_GENOS=10309;East_Han_AN=6554;East_Han_AC=2;East_Han_AF
chr7	17055076	.	T	<INS:ME:LINE1>	.	PASS	SVTYPE=INS:ME:LINE1;END=17055090;SVLEN=225;AN=20618;AC=5700;AF=0.2764574643515375;N_HET=3358;N_HOMALT=1171;N_HOMREF=5780;FREQ_HET=0.32573479483946066;FREQ_HOMALT=0.11359006693180716;FREQ_HOMREF=0.5606751382287322;N_BI_GENOS=10309;South_Han_AN=1496;South_H
chr7	17089145	.	G	<INS:ME:LINE1>	.	PASS	SVTYPE=INS:ME:LINE1;END=17089145;SVLEN=0;AN=20618;AC=1;AF=4.850130953535746e-05;N_HET=1;N_HOMALT=0;N_HOMREF=10308;FREQ_HET=9.700261907071492e-05;FREQ_HOMALT=0.0;FREQ_HOMREF=0.9999029973809292;N_BI_GENOS=10309;East_Han_AN=6554;East_Han_AC=1;East_Han_AF=0.0
chr7	17120290	.	A	<INS:ME:LINE1>	.	PASS	SVTYPE=INS:ME:LINE1;END=17120306;SVLEN=2497;AN=20618;AC=5;AF=0.00024250654767678728;N_HET=5;N_HOMALT=0;N_HOMREF=10304;FREQ_HET=0.00048501309535357455;FREQ_HOMALT=0.0;FREQ_HOMREF=0.9995149869046465;N_BI_GENOS=10309;North_Han_AN=2272;North_Han_AC=1;North_Ha
chr7	17133013	.	A	<INS:ME:LINE1>	.	PASS	SVTYPE=INS:ME:LINE1;END=17133027;SVLEN=6055;AN=20618;AC=1;AF=4.850130953535746e-05;N_HET=1;N_HOMALT=0;N_HOMREF=10308;FREQ_HET=9.700261907071492e-05;FREQ_HOMALT=0.0;FREQ_HOMREF=0.9999029973809292;N_BI_GENOS=10309;Central_Han_AN=2128;Central_Han_AC=1;Centra
chr7	17173521	.	A	<INS:ME:LINE1>	.	PASS	SVTYPE=INS:ME:LINE1;END=17173524;SVLEN=1597;AN=20618;AC=2;AF=9.700261907071492e-05;N_HET=2;N_HOMALT=0;N_HOMREF=10307;FREQ_HET=0.00019400523814142983;FREQ_HOMALT=0.0;FREQ_HOMREF=0.9998059947618586;N_BI_GENOS=10309;South_Han_AN=1496;South_Han_AC=1;South_Han
chr7	17189027	.	A	<INS:ME:LINE1>	.	PASS	SVTYPE=INS:ME:LINE1;END=17189040;SVLEN=1812;AN=20618;AC=1;AF=4.850130953535746e-05;N_HET=1;N_HOMALT=0;N_HOMREF=10308;FREQ_HET=9.700261907071492e-05;FREQ_HOMALT=0.0;FREQ_HOMREF=0.9999029973809292;N_BI_GENOS=10309;South_Han_AN=1496;South_Han_AC=1;South_Han_
chr7	17324801	.	A	<INS:ME:LINE1>	.	PASS	SVTYPE=INS:ME:LINE1;END=17324835;SVLEN=1248;AN=20618;AC=40;AF=0.0019400523814142982;N_HET=38;N_HOMALT=1;N_HOMREF=10270;FREQ_HET=0.0036860995246871665;FREQ_HOMALT=9.700261907071492e-05;FREQ_HOMREF=0.9962168978562421;N_BI_GENOS=10309;South_Han_AN=1496;South
chr7	18022953	.	T	<INS:ME:LINE1>	.	PASS	SVTYPE=INS:ME:LINE1;END=18022966;SVLEN=1608;AN=20618;AC=1;AF=4.850130953535746e-05;N_HET=1;N_HOMALT=0;N_HOMREF=10308;FREQ_HET=9.700261907071492e-05;FREQ_HOMALT=0.0;FREQ_HOMREF=0.9999029973809292;N_BI_GENOS=10309;North_Han_AN=2272;North_Han_AC=1;North_Han_
chr7	18475156	.	C	<INS:ME:LINE1>	.	PASS	SVTYPE=INS:ME:LINE1;END=18475172;SVLEN=2172;AN=20618;AC=1;AF=4.850130953535746e-05;N_HET=1;N_HOMALT=0;N_HOMREF=10308;FREQ_HET=9.700261907071492e-05;FREQ_HOMALT=0.0;FREQ_HOMREF=0.9999029973809292;N_BI_GENOS=10309;Miao_AN=482;Miao_AC=1;Miao_AF=0.00207468879
chr7	18951095	.	T	<INS:ME:LINE1>	.	PASS	SVTYPE=INS:ME:LINE1;END=18951107;SVLEN=329;AN=20618;AC=1;AF=4.850130953535746e-05;N_HET=1;N_HOMALT=0;N_HOMREF=10308;FREQ_HET=9.700261907071492e-05;FREQ_HOMALT=0.0;FREQ_HOMREF=0.9999029973809292;N_BI_GENOS=10309;Central_Han_AN=2128;Central_Han_AC=1;Central
chr7	18963380	.	A	<INS:ME:LINE1>	.	PASS	SVTYPE=INS:ME:LINE1;END=18963381;SVLEN=653;AN=20618;AC=1;AF=4.850130953535746e-05;N_HET=1;N_HOMALT=0;N_HOMREF=10308;FREQ_HET=9.700261907071492e-05;FREQ_HOMALT=0.0;FREQ_HOMREF=0.9999029973809292;N_BI_GENOS=10309;East_Han_AN=6554;East_Han_AC=1;East_Han_AF=0
chr7	19538725	.	T	<INS:ME:LINE1>	.	PASS	SVTYPE=INS:ME:LINE1;END=19538725;SVLEN=130;AN=20618;AC=1;AF=4.850130953535746e-05;N_HET=1;N_HOMALT=0;N_HOMREF=10308;FREQ_HET=9.700261907071492e-05;FREQ_HOMALT=0.0;FREQ_HOMREF=0.9999029973809292;N_BI_GENOS=10309;Northwest_Han_AN=1296;Northwest_Han_AC=1;Nor
chr7	19625699	.	A	<INS:ME:LINE1>	.	PASS	SVTYPE=INS:ME:LINE1;END=19625711;SVLEN=5994;AN=20618;AC=1;AF=4.850130953535746e-05;N_HET=1;N_HOMALT=0;N_HOMREF=10308;FREQ_HET=9.700261907071492e-05;FREQ_HOMALT=0.0;FREQ_HOMREF=0.9999029973809292;N_BI_GENOS=10309;Zhuang_AN=444;Zhuang_AC=1;Zhuang_AF=0.00225
chr7	20130698	.	A	<INS:ME:LINE1>	.	PASS	SVTYPE=INS:ME:LINE1;END=20130717;SVLEN=1504;AN=20618;AC=775;AF=0.03758851488990203;N_HET=695;N_HOMALT=40;N_HOMREF=9574;FREQ_HET=0.06741682025414686;FREQ_HOMALT=0.0038801047628285964;FREQ_HOMREF=0.9287030749830245;N_BI_GENOS=10309;South_Han_AN=1496;South_H
chr7	20175507	.	A	<INS:ME:LINE1>	.	PASS	SVTYPE=INS:ME:LINE1;END=20175525;SVLEN=6008;AN=20618;AC=1;AF=4.850130953535746e-05;N_HET=1;N_HOMALT=0;N_HOMREF=10308;FREQ_HET=9.700261907071492e-05;FREQ_HOMALT=0.0;FREQ_HOMREF=0.9999029973809292;N_BI_GENOS=10309;East_Han_AN=6554;East_Han_AC=1;East_Han_AF=
chr7	20344400	.	G	<INS:ME:LINE1>	.	PASS	SVTYPE=INS:ME:LINE1;END=20344413;SVLEN=6148;AN=20618;AC=1;AF=4.850130953535746e-05;N_HET=1;N_HOMALT=0;N_HOMREF=10308;FREQ_HET=9.700261907071492e-05;FREQ_HOMALT=0.0;FREQ_HOMREF=0.9999029973809292;N_BI_GENOS=10309;Lingnan_Han_AN=1888;Lingnan_Han_AC=1;Lingna
chr7	20518072	.	A	<INS:ME:LINE1>	.	PASS	SVTYPE=INS:ME:LINE1;END=20518090;SVLEN=6000;AN=20618;AC=2;AF=9.700261907071492e-05;N_HET=2;N_HOMALT=0;N_HOMREF=10307;FREQ_HET=0.00019400523814142983;FREQ_HOMALT=0.0;FREQ_HOMREF=0.9998059947618586;N_BI_GENOS=10309;Lingnan_Han_AN=1888;Lingnan_Han_AC=2;Lingn
chr7	21115247	.	T	<INS:ME:LINE1>	.	PASS	SVTYPE=INS:ME:LINE1;END=21115261;SVLEN=107;AN=20618;AC=1;AF=4.850130953535746e-05;N_HET=1;N_HOMALT=0;N_HOMREF=10308;FREQ_HET=9.700261907071492e-05;FREQ_HOMALT=0.0;FREQ_HOMREF=0.9999029973809292;N_BI_GENOS=10309;South_Han_AN=1496;South_Han_AC=1;South_Han_A
chr7	21117607	.	G	<INS:ME:LINE1>	.	PASS	SVTYPE=INS:ME:LINE1;END=21117617;SVLEN=591;AN=20618;AC=1;AF=4.850130953535746e-05;N_HET=1;N_HOMALT=0;N_HOMREF=10308;FREQ_HET=9.700261907071492e-05;FREQ_HOMALT=0.0;FREQ_HOMREF=0.9999029973809292;N_BI_GENOS=10309;North_Han_AN=2272;North_Han_AC=1;North_Han_A
chr7	21354530	.	A	<INS:ME:LINE1>	.	PASS	SVTYPE=INS:ME:LINE1;END=21354546;SVLEN=766;AN=20618;AC=4;AF=0.00019400523814142983;N_HET=2;N_HOMALT=1;N_HOMREF=10306;FREQ_HET=0.00019400523814142983;FREQ_HOMALT=9.700261907071492e-05;FREQ_HOMREF=0.9997089921427879;N_BI_GENOS=10309;Northwest_Han_AN=1296;No
chr7	21568540	.	G	<INS:ME:LINE1>	.	PASS	SVTYPE=INS:ME:LINE1;END=21568567;SVLEN=1737;AN=20618;AC=1;AF=4.850130953535746e-05;N_HET=1;N_HOMALT=0;N_HOMREF=10308;FREQ_HET=9.700261907071492e-05;FREQ_HOMALT=0.0;FREQ_HOMREF=0.9999029973809292;N_BI_GENOS=10309;North_Han_AN=2272;North_Han_AC=1;North_Han_
chr7	21765160	.	A	<INS:ME:LINE1>	.	PASS	SVTYPE=INS:ME:LINE1;END=21765174;SVLEN=5996;AN=20618;AC=7;AF=0.0003395091667475022;N_HET=7;N_HOMALT=0;N_HOMREF=10302;FREQ_HET=0.0006790183334950044;FREQ_HOMALT=0.0;FREQ_HOMREF=0.999320981666505;N_BI_GENOS=10309;South_Han_AN=1496;South_Han_AC=1;South_Han_A
chr7	22222659	.	A	<INS:ME:LINE1>	.	PASS	SVTYPE=INS:ME:LINE1;END=22222661;SVLEN=259;AN=20618;AC=4;AF=0.00019400523814142983;N_HET=4;N_HOMALT=0;N_HOMREF=10305;FREQ_HET=0.00038801047628285966;FREQ_HOMALT=0.0;FREQ_HOMREF=0.9996119895237171;N_BI_GENOS=10309;East_Han_AN=6554;East_Han_AC=2;East_Han_AF
chr7	22256397	.	C	<INS:ME:LINE1>	.	PASS	SVTYPE=INS:ME:LINE1;END=22256398;SVLEN=335;AN=20618;AC=13;AF=0.0006305170239596469;N_HET=13;N_HOMALT=0;N_HOMREF=10296;FREQ_HET=0.0012610340479192938;FREQ_HOMALT=0.0;FREQ_HOMREF=0.9987389659520807;N_BI_GENOS=10309;East_Han_AN=6554;East_Han_AC=9;East_Han_AF
chr7	22813873	.	T	<INS:ME:LINE1>	.	PASS	SVTYPE=INS:ME:LINE1;END=22813873;SVLEN=0;AN=20618;AC=3;AF=0.00014550392860607236;N_HET=3;N_HOMALT=0;N_HOMREF=10306;FREQ_HET=0.0002910078572121447;FREQ_HOMALT=0.0;FREQ_HOMREF=0.9997089921427879;N_BI_GENOS=10309;East_Han_AN=6554;East_Han_AC=1;East_Han_AF=0.
chr7	23785756	.	C	<INS:ME:LINE1>	.	PASS	SVTYPE=INS:ME:LINE1;END=23785771;SVLEN=5989;AN=20618;AC=3;AF=0.00014550392860607236;N_HET=3;N_HOMALT=0;N_HOMREF=10306;FREQ_HET=0.0002910078572121447;FREQ_HOMALT=0.0;FREQ_HOMREF=0.9997089921427879;N_BI_GENOS=10309;South_Han_AN=1496;South_Han_AC=2;South_Han
chr7	23882651	.	A	<INS:ME:LINE1>	.	PASS	SVTYPE=INS:ME:LINE1;END=23882668;SVLEN=937;AN=20618;AC=3;AF=0.00014550392860607236;N_HET=3;N_HOMALT=0;N_HOMREF=10306;FREQ_HET=0.0002910078572121447;FREQ_HOMALT=0.0;FREQ_HOMREF=0.9997089921427879;N_BI_GENOS=10309;South_Han_AN=1496;South_Han_AC=1;South_Han_
chr7	24039324	.	G	<INS:ME:LINE1>	.	PASS	SVTYPE=INS:ME:LINE1;END=24039338;SVLEN=5998;AN=20618;AC=1;AF=4.850130953535746e-05;N_HET=1;N_HOMALT=0;N_HOMREF=10308;FREQ_HET=9.700261907071492e-05;FREQ_HOMALT=0.0;FREQ_HOMREF=0.9999029973809292;N_BI_GENOS=10309;South_Han_AN=1496;South_Han_AC=1;South_Han_
chr7	24047273	.	T	<INS:ME:LINE1>	.	PASS	SVTYPE=INS:ME:LINE1;END=24047288;SVLEN=6003;AN=20618;AC=1;AF=4.850130953535746e-05;N_HET=1;N_HOMALT=0;N_HOMREF=10308;FREQ_HET=9.700261907071492e-05;FREQ_HOMALT=0.0;FREQ_HOMREF=0.9999029973809292;N_BI_GENOS=10309;East_Han_AN=6554;East_Han_AC=1;East_Han_AF=
chr7	24110592	.	T	<INS:ME:LINE1>	.	PASS	SVTYPE=INS:ME:LINE1;END=24110596;SVLEN=6055;AN=20618;AC=2;AF=9.700261907071492e-05;N_HET=2;N_HOMALT=0;N_HOMREF=10307;FREQ_HET=0.00019400523814142983;FREQ_HOMALT=0.0;FREQ_HOMREF=0.9998059947618586;N_BI_GENOS=10309;South_Han_AN=1496;South_Han_AC=1;South_Han
chr7	24238126	.	A	<INS:ME:LINE1>	.	PASS	SVTYPE=INS:ME:LINE1;END=24238142;SVLEN=6005;AN=20618;AC=3;AF=0.00014550392860607236;N_HET=3;N_HOMALT=0;N_HOMREF=10306;FREQ_HET=0.0002910078572121447;FREQ_HOMALT=0.0;FREQ_HOMREF=0.9997089921427879;N_BI_GENOS=10309;Hui_AN=444;Hui_AC=2;Hui_AF=0.0045045045045
chr7	24289306	.	A	<INS:ME:LINE1>	.	PASS	SVTYPE=INS:ME:LINE1;END=24289318;SVLEN=5999;AN=20618;AC=1;AF=4.850130953535746e-05;N_HET=1;N_HOMALT=0;N_HOMREF=10308;FREQ_HET=9.700261907071492e-05;FREQ_HOMALT=0.0;FREQ_HOMREF=0.9999029973809292;N_BI_GENOS=10309;South_Han_AN=1496;South_Han_AC=1;South_Han_
chr7	24397405	.	G	<INS:ME:LINE1>	.	PASS	SVTYPE=INS:ME:LINE1;END=24397405;SVLEN=0;AN=20618;AC=4;AF=0.00019400523814142983;N_HET=0;N_HOMALT=2;N_HOMREF=10307;FREQ_HET=0.0;FREQ_HOMALT=0.00019400523814142983;FREQ_HOMREF=0.9998059947618586;N_BI_GENOS=10309;East_Han_AN=6554;East_Han_AC=2;East_Han_AF=0
chr7	24872837	.	C	<INS:ME:LINE1>	.	PASS	SVTYPE=INS:ME:LINE1;END=24872853;SVLEN=637;AN=20618;AC=5;AF=0.00024250654767678728;N_HET=3;N_HOMALT=1;N_HOMREF=10305;FREQ_HET=0.0002910078572121447;FREQ_HOMALT=9.700261907071492e-05;FREQ_HOMREF=0.9996119895237171;N_BI_GENOS=10309;South_Han_AN=1496;South_H
chr7	25389073	.	G	<INS:ME:LINE1>	.	PASS	SVTYPE=INS:ME:LINE1;END=25389085;SVLEN=5999;AN=20618;AC=2;AF=9.700261907071492e-05;N_HET=2;N_HOMALT=0;N_HOMREF=10307;FREQ_HET=0.00019400523814142983;FREQ_HOMALT=0.0;FREQ_HOMREF=0.9998059947618586;N_BI_GENOS=10309;North_Han_AN=2272;North_Han_AC=1;North_Han
chr7	25390574	.	A	<INS:ME:LINE1>	.	PASS	SVTYPE=INS:ME:LINE1;END=25390590;SVLEN=221;AN=20618;AC=1;AF=4.850130953535746e-05;N_HET=1;N_HOMALT=0;N_HOMREF=10308;FREQ_HET=9.700261907071492e-05;FREQ_HOMALT=0.0;FREQ_HOMREF=0.9999029973809292;N_BI_GENOS=10309;East_Han_AN=6554;East_Han_AC=1;East_Han_AF=0
chr7	25407806	.	T	<INS:ME:LINE1>	.	PASS	SVTYPE=INS:ME:LINE1;END=25407822;SVLEN=5172;AN=20618;AC=1;AF=4.850130953535746e-05;N_HET=1;N_HOMALT=0;N_HOMREF=10308;FREQ_HET=9.700261907071492e-05;FREQ_HOMALT=0.0;FREQ_HOMREF=0.9999029973809292;N_BI_GENOS=10309;South_Han_AN=1496;South_Han_AC=1;South_Han_
chr7	25505252	.	G	<INS:ME:LINE1>	.	PASS	SVTYPE=INS:ME:LINE1;END=25505264;SVLEN=6014;AN=20618;AC=9;AF=0.0004365117858182171;N_HET=7;N_HOMALT=1;N_HOMREF=10301;FREQ_HET=0.0006790183334950044;FREQ_HOMALT=9.700261907071492e-05;FREQ_HOMREF=0.9992239790474343;N_BI_GENOS=10309;Lingnan_Han_AN=1888;Lingn
chr7	26118807	.	A	<INS:ME:LINE1>	.	PASS	SVTYPE=INS:ME:LINE1;END=26118823;SVLEN=355;AN=20618;AC=27;AF=0.0013095353574546513;N_HET=27;N_HOMALT=0;N_HOMREF=10282;FREQ_HET=0.0026190707149093026;FREQ_HOMALT=0.0;FREQ_HOMREF=0.9973809292850907;N_BI_GENOS=10309;South_Han_AN=1496;South_Han_AC=3;South_Han
chr7	27667811	.	T	<INS:ME:LINE1>	.	PASS	SVTYPE=INS:ME:LINE1;END=27667827;SVLEN=5998;AN=20618;AC=2;AF=9.700261907071492e-05;N_HET=2;N_HOMALT=0;N_HOMREF=10307;FREQ_HET=0.00019400523814142983;FREQ_HOMALT=0.0;FREQ_HOMREF=0.9998059947618586;N_BI_GENOS=10309;Lingnan_Han_AN=1888;Lingnan_Han_AC=1;Lingn
chr7	27810403	.	A	<INS:ME:LINE1>	.	PASS	SVTYPE=INS:ME:LINE1;END=27810419;SVLEN=6066;AN=20618;AC=1;AF=4.850130953535746e-05;N_HET=1;N_HOMALT=0;N_HOMREF=10308;FREQ_HET=9.700261907071492e-05;FREQ_HOMALT=0.0;FREQ_HOMREF=0.9999029973809292;N_BI_GENOS=10309;Southeast_Han_AN=1908;Southeast_Han_AC=1;So
chr7	28432596	.	G	<INS:ME:LINE1>	.	PASS	SVTYPE=INS:ME:LINE1;END=28432601;SVLEN=646;AN=20618;AC=1;AF=4.850130953535746e-05;N_HET=1;N_HOMALT=0;N_HOMREF=10308;FREQ_HET=9.700261907071492e-05;FREQ_HOMALT=0.0;FREQ_HOMREF=0.9999029973809292;N_BI_GENOS=10309;Central_Han_AN=2128;Central_Han_AC=1;Central
chr7	28502386	.	A	<INS:ME:LINE1>	.	PASS	SVTYPE=INS:ME:LINE1;END=28502399;SVLEN=6017;AN=20618;AC=1;AF=4.850130953535746e-05;N_HET=1;N_HOMALT=0;N_HOMREF=10308;FREQ_HET=9.700261907071492e-05;FREQ_HOMALT=0.0;FREQ_HOMREF=0.9999029973809292;N_BI_GENOS=10309;Yi_AN=422;Yi_AC=1;Yi_AF=0.00236966824644549
chr7	29069499	.	A	<INS:ME:LINE1>	.	PASS	SVTYPE=INS:ME:LINE1;END=29069514;SVLEN=6042;AN=20618;AC=1;AF=4.850130953535746e-05;N_HET=1;N_HOMALT=0;N_HOMREF=10308;FREQ_HET=9.700261907071492e-05;FREQ_HOMALT=0.0;FREQ_HOMREF=0.9999029973809292;N_BI_GENOS=10309;East_Han_AN=6554;East_Han_AC=1;East_Han_AF=
chr7	29075758	.	A	<INS:ME:LINE1>	.	PASS	SVTYPE=INS:ME:LINE1;END=29075797;SVLEN=5062;AN=20618;AC=1;AF=4.850130953535746e-05;N_HET=1;N_HOMALT=0;N_HOMREF=10308;FREQ_HET=9.700261907071492e-05;FREQ_HOMALT=0.0;FREQ_HOMREF=0.9999029973809292;N_BI_GENOS=10309;East_Han_AN=6554;East_Han_AC=1;East_Han_AF=
chr7	29757153	.	G	<INS:ME:LINE1>	.	PASS	SVTYPE=INS:ME:LINE1;END=29757166;SVLEN=5998;AN=20618;AC=1;AF=4.850130953535746e-05;N_HET=1;N_HOMALT=0;N_HOMREF=10308;FREQ_HET=9.700261907071492e-05;FREQ_HOMALT=0.0;FREQ_HOMREF=0.9999029973809292;N_BI_GENOS=10309;Central_Han_AN=2128;Central_Han_AC=1;Centra
chr7	29868129	.	A	<INS:ME:LINE1>	.	PASS	SVTYPE=INS:ME:LINE1;END=29868147;SVLEN=4438;AN=20618;AC=1;AF=4.850130953535746e-05;N_HET=1;N_HOMALT=0;N_HOMREF=10308;FREQ_HET=9.700261907071492e-05;FREQ_HOMALT=0.0;FREQ_HOMREF=0.9999029973809292;N_BI_GENOS=10309;South_Han_AN=1496;South_Han_AC=1;South_Han_
chr7	30009096	.	A	<INS:ME:LINE1>	.	PASS	SVTYPE=INS:ME:LINE1;END=30009115;SVLEN=712;AN=20618;AC=1;AF=4.850130953535746e-05;N_HET=1;N_HOMALT=0;N_HOMREF=10308;FREQ_HET=9.700261907071492e-05;FREQ_HOMALT=0.0;FREQ_HOMREF=0.9999029973809292;N_BI_GENOS=10309;Northwest_Han_AN=1296;Northwest_Han_AC=1;Nor
chr7	30111921	.	T	<INS:ME:LINE1>	.	PASS	SVTYPE=INS:ME:LINE1;END=30111921;SVLEN=1;AN=20618;AC=1;AF=4.850130953535746e-05;N_HET=1;N_HOMALT=0;N_HOMREF=10308;FREQ_HET=9.700261907071492e-05;FREQ_HOMALT=0.0;FREQ_HOMREF=0.9999029973809292;N_BI_GENOS=10309;East_Han_AN=6554;East_Han_AC=1;East_Han_AF=0.0
chr7	30887064	.	A	<INS:ME:LINE1>	.	PASS	SVTYPE=INS:ME:LINE1;END=30887067;SVLEN=1284;AN=20618;AC=1;AF=4.850130953535746e-05;N_HET=1;N_HOMALT=0;N_HOMREF=10308;FREQ_HET=9.700261907071492e-05;FREQ_HOMALT=0.0;FREQ_HOMREF=0.9999029973809292;N_BI_GENOS=10309;Manchu_AN=416;Manchu_AC=1;Manchu_AF=0.00240
chr7	31055615	.	A	<INS:ME:LINE1>	.	PASS	SVTYPE=INS:ME:LINE1;END=31055631;SVLEN=6028;AN=20618;AC=1;AF=4.850130953535746e-05;N_HET=1;N_HOMALT=0;N_HOMREF=10308;FREQ_HET=9.700261907071492e-05;FREQ_HOMALT=0.0;FREQ_HOMREF=0.9999029973809292;N_BI_GENOS=10309;Northwest_Han_AN=1296;Northwest_Han_AC=1;No
chr7	31172598	.	A	<INS:ME:LINE1>	.	PASS	SVTYPE=INS:ME:LINE1;END=31172612;SVLEN=567;AN=20618;AC=1;AF=4.850130953535746e-05;N_HET=1;N_HOMALT=0;N_HOMREF=10308;FREQ_HET=9.700261907071492e-05;FREQ_HOMALT=0.0;FREQ_HOMREF=0.9999029973809292;N_BI_GENOS=10309;East_Han_AN=6554;East_Han_AC=1;East_Han_AF=0
chr7	31218154	.	A	<INS:ME:LINE1>	.	PASS	SVTYPE=INS:ME:LINE1;END=31218171;SVLEN=1038;AN=20618;AC=1;AF=4.850130953535746e-05;N_HET=1;N_HOMALT=0;N_HOMREF=10308;FREQ_HET=9.700261907071492e-05;FREQ_HOMALT=0.0;FREQ_HOMREF=0.9999029973809292;N_BI_GENOS=10309;North_Han_AN=2272;North_Han_AC=1;North_Han_
chr7	31240536	.	G	<INS:ME:LINE1>	.	PASS	SVTYPE=INS:ME:LINE1;END=31240546;SVLEN=982;AN=20618;AC=1;AF=4.850130953535746e-05;N_HET=1;N_HOMALT=0;N_HOMREF=10308;FREQ_HET=9.700261907071492e-05;FREQ_HOMALT=0.0;FREQ_HOMREF=0.9999029973809292;N_BI_GENOS=10309;East_Han_AN=6554;East_Han_AC=1;East_Han_AF=0
chr7	31679909	.	T	<INS:ME:LINE1>	.	PASS	SVTYPE=INS:ME:LINE1;END=31679921;SVLEN=97;AN=20618;AC=2;AF=9.700261907071492e-05;N_HET=2;N_HOMALT=0;N_HOMREF=10307;FREQ_HET=0.00019400523814142983;FREQ_HOMALT=0.0;FREQ_HOMREF=0.9998059947618586;N_BI_GENOS=10309;East_Han_AN=6554;East_Han_AC=2;East_Han_AF=0
chr7	32151292	.	G	<INS:ME:LINE1>	.	PASS	SVTYPE=INS:ME:LINE1;END=32151309;SVLEN=186;AN=20618;AC=1;AF=4.850130953535746e-05;N_HET=1;N_HOMALT=0;N_HOMREF=10308;FREQ_HET=9.700261907071492e-05;FREQ_HOMALT=0.0;FREQ_HOMREF=0.9999029973809292;N_BI_GENOS=10309;Northwest_Han_AN=1296;Northwest_Han_AC=1;Nor
chr7	32165414	.	A	<INS:ME:LINE1>	.	PASS	SVTYPE=INS:ME:LINE1;END=32165428;SVLEN=1823;AN=20618;AC=1;AF=4.850130953535746e-05;N_HET=1;N_HOMALT=0;N_HOMREF=10308;FREQ_HET=9.700261907071492e-05;FREQ_HOMALT=0.0;FREQ_HOMREF=0.9999029973809292;N_BI_GENOS=10309;South_Han_AN=1496;South_Han_AC=1;South_Han_
chr7	32204045	.	A	<INS:ME:LINE1>	.	PASS	SVTYPE=INS:ME:LINE1;END=32204061;SVLEN=6001;AN=20618;AC=1;AF=4.850130953535746e-05;N_HET=1;N_HOMALT=0;N_HOMREF=10308;FREQ_HET=9.700261907071492e-05;FREQ_HOMALT=0.0;FREQ_HOMREF=0.9999029973809292;N_BI_GENOS=10309;Lingnan_Han_AN=1888;Lingnan_Han_AC=1;Lingna
chr7	32232324	.	C	<INS:ME:LINE1>	.	PASS	SVTYPE=INS:ME:LINE1;END=32232341;SVLEN=6016;AN=20618;AC=1;AF=4.850130953535746e-05;N_HET=1;N_HOMALT=0;N_HOMREF=10308;FREQ_HET=9.700261907071492e-05;FREQ_HOMALT=0.0;FREQ_HOMREF=0.9999029973809292;N_BI_GENOS=10309;North_Han_AN=2272;North_Han_AC=1;North_Han_

chr7	33194501 .	G	<INS:ME:LINE1>	.	PASS	SVTYPE=INS:ME:LINE1;END=33194516;SVLEN=1332;AN=20618;AC=1;AF=4.850130953535746e-05;N_HET=1;N_HOMALT=0;N_HOMREF=10308;FREQ_HET=9.700261907071492e-05;FREQ_HOMALT=0.0;FREQ_HOMREF=0.9999029973809292;N_BI_GENOS=10309;North_Han_AN=2272;North_Han_AC=1;North_Han_
chr7	33442423 .	A	<INS:ME:LINE1>	.	PASS	SVTYPE=INS:ME:LINE1;END=33442439;SVLEN=5993;AN=20618;AC=15;AF=0.0007275196430303619;N_HET=13;N_HOMALT=1;N_HOMREF=10295;FREQ_HET=0.0012610340479192938;FREQ_HOMALT=9.700261907071492e-05;FREQ_HOMREF=0.998641963333301;N_BI_GENOS=10309;South_Han_AN=1496;South_H
chr7	33521747 .	T	<INS:ME:LINE1>	.	PASS	SVTYPE=INS:ME:LINE1;END=33521758;SVLEN=1049;AN=20618;AC=1;AF=4.850130953535746e-05;N_HET=1;N_HOMALT=0;N_HOMREF=10308;FREQ_HET=9.700261907071492e-05;FREQ_HOMALT=0.0;FREQ_HOMREF=0.9999029973809292;N_BI_GENOS=10309;East_Han_AN=6554;East_Han_AC=1;East_Han_AF=
chr7	33550379 .	A	<INS:ME:LINE1>	.	PASS	SVTYPE=INS:ME:LINE1;END=33550393;SVLEN=5985;AN=20618;AC=1;AF=4.850130953535746e-05;N_HET=1;N_HOMALT=0;N_HOMREF=10308;FREQ_HET=9.700261907071492e-05;FREQ_HOMALT=0.0;FREQ_HOMREF=0.9999029973809292;N_BI_GENOS=10309;East_Han_AN=6554;East_Han_AC=1;East_Han_AF=
chr7	33918735 .	G	<INS:ME:LINE1>	.	PASS	SVTYPE=INS:ME:LINE1;END=33918752;SVLEN=91;AN=20618;AC=1;AF=4.850130953535746e-05;N_HET=1;N_HOMALT=0;N_HOMREF=10308;FREQ_HET=9.700261907071492e-05;FREQ_HOMALT=0.0;FREQ_HOMREF=0.9999029973809292;N_BI_GENOS=10309;East_Han_AN=6554;East_Han_AC=1;East_Han_AF=0.
chr7	34268995 .	C	<INS:ME:LINE1>	.	PASS	SVTYPE=INS:ME:LINE1;END=34269006;SVLEN=6024;AN=20618;AC=1;AF=4.850130953535746e-05;N_HET=1;N_HOMALT=0;N_HOMREF=10308;FREQ_HET=9.700261907071492e-05;FREQ_HOMALT=0.0;FREQ_HOMREF=0.9999029973809292;N_BI_GENOS=10309;North_Han_AN=2272;North_Han_AC=1;North_Han_
chr7	34663068 .	G	<INS:ME:LINE1>	.	PASS	SVTYPE=INS:ME:LINE1;END=34663068;SVLEN=0;AN=20618;AC=6;AF=0.0002910078572121447;N_HET=2;N_HOMALT=2;N_HOMREF=10305;FREQ_HET=0.0001940052381414298;FREQ_HOMALT=0.0001940052381414298;FREQ_HOMREF=0.9996119895237171;N_BI_GENOS=10309;East_Han_AN=6554;East_Han_
chr7	35248655 .	G	<INS:ME:LINE1>	.	PASS	SVTYPE=INS:ME:LINE1;END=35248670;SVLEN=584;AN=20618;AC=1;AF=4.850130953535746e-05;N_HET=1;N_HOMALT=0;N_HOMREF=10308;FREQ_HET=9.700261907071492e-05;FREQ_HOMALT=0.0;FREQ_HOMREF=0.9999029973809292;N_BI_GENOS=10309;Lingnan_Han_AN=1888;Lingnan_Han_AC=1;Lingnan
chr7	35390242 .	T	<INS:ME:LINE1>	.	PASS	SVTYPE=INS:ME:LINE1;END=35390242;SVLEN=93;AN=20618;AC=1;AF=4.850130953535746e-05;N_HET=1;N_HOMALT=0;N_HOMREF=10308;FREQ_HET=9.700261907071492e-05;FREQ_HOMALT=0.0;FREQ_HOMREF=0.9999029973809292;N_BI_GENOS=10309;Southeast_Han_AN=1908;Southeast_Han_AC=1;Sout
chr7	35596740 .	A	<INS:ME:LINE1>	.	PASS	SVTYPE=INS:ME:LINE1;END=35596757;SVLEN=5995;AN=20618;AC=8;AF=0.0003880104762828596;N_HET=6;N_HOMALT=1;N_HOMREF=10302;FREQ_HET=0.0005820157144242894;FREQ_HOMALT=9.700261907071492e-05;FREQ_HOMREF=0.999320981666505;N_BI_GENOS=10309;East_Han_AN=6554;East_Han
chr7	36155273 .	T	<INS:ME:LINE1>	.	PASS	SVTYPE=INS:ME:LINE1;END=36155288;SVLEN=6000;AN=20618;AC=1;AF=4.850130953535746e-05;N_HET=1;N_HOMALT=0;N_HOMREF=10308;FREQ_HET=9.700261907071492e-05;FREQ_HOMALT=0.0;FREQ_HOMREF=0.9999029973809292;N_BI_GENOS=10309;Northwest_Han_AN=1296;Northwest_Han_AC=1;No
chr7	36334608 .	A	<INS:ME:LINE1>	.	PASS	SVTYPE=INS:ME:LINE1;END=36334625;SVLEN=6027;AN=20618;AC=1;AF=4.850130953535746e-05;N_HET=1;N_HOMALT=0;N_HOMREF=10308;FREQ_HET=9.700261907071492e-05;FREQ_HOMALT=0.0;FREQ_HOMREF=0.9999029973809292;N_BI_GENOS=10309;Southeast_Han_AN=1908;Southeast_Han_AC=1;So
chr7	36807305 .	A	<INS:ME:LINE1>	.	PASS	SVTYPE=INS:ME:LINE1;END=36807307;SVLEN=1739;AN=20618;AC=97;AF=0.004704627024929673;N_HET=97;N_HOMALT=0;N_HOMREF=10212;FREQ_HET=0.009409254049859346;FREQ_HOMALT=0.0;FREQ_HOMREF=0.9905907459501406;N_BI_GENOS=10309;South_Han_AN=1496;South_Han_AC=2;South_Han_
chr7	37036328 .	A	<INS:ME:LINE1>	.	PASS	SVTYPE=INS:ME:LINE1;END=37036343;SVLEN=5999;AN=20618;AC=1;AF=4.850130953535746e-05;N_HET=1;N_HOMALT=0;N_HOMREF=10308;FREQ_HET=9.700261907071492e-05;FREQ_HOMALT=0.0;FREQ_HOMREF=0.9999029973809292;N_BI_GENOS=10309;South_Han_AN=1496;South_Han_AC=1;South_Han_
chr7	37491881 .	A	<INS:ME:LINE1>	.	PASS	SVTYPE=INS:ME:LINE1;END=37491884;SVLEN=646;AN=20618;AC=1;AF=4.850130953535746e-05;N_HET=1;N_HOMALT=0;N_HOMREF=10308;FREQ_HET=9.700261907071492e-05;FREQ_HOMALT=0.0;FREQ_HOMREF=0.9999029973809292;N_BI_GENOS=10309;Northwest_Han_AN=1296;Northwest_Han_AC=1;Nor
chr7	37625767 .	A	<INS:ME:LINE1>	.	PASS	SVTYPE=INS:ME:LINE1;END=37625780;SVLEN=3774;AN=20618;AC=1;AF=4.850130953535746e-05;N_HET=1;N_HOMALT=0;N_HOMREF=10308;FREQ_HET=9.700261907071492e-05;FREQ_HOMALT=0.0;FREQ_HOMREF=0.9999029973809292;N_BI_GENOS=10309;East_Han_AN=6554;East_Han_AC=1;East_Han_AF=
chr7	37721165 .	C	<INS:ME:LINE1>	.	PASS	SVTYPE=INS:ME:LINE1;END=37721182;SVLEN=1211;AN=20618;AC=63;AF=0.0030555825007275196;N_HET=61;N_HOMALT=1;N_HOMREF=10247;FREQ_HET=0.005917159763313609;FREQ_HOMALT=9.700261907071492e-05;FREQ_HOMREF=0.9939858376176157;N_BI_GENOS=10309;South_Han_AN=1496;South_
chr7	37910182 .	C	<INS:ME:LINE1>	.	PASS	SVTYPE=INS:ME:LINE1;END=37910194;SVLEN=1037;AN=20618;AC=5;AF=0.0002425065476767878;N_HET=5;N_HOMALT=0;N_HOMREF=10304;FREQ_HET=0.0004850130953535746;FREQ_HOMALT=0.0;FREQ_HOMREF=0.9995149869046465;N_BI_GENOS=10309;East_Han_AN=6554;East_Han_AC=5;East_Han_A
chr7	37931528 .	A	<INS:ME:LINE1>	.	PASS	SVTYPE=INS:ME:LINE1;END=37931528;SVLEN=1266;AN=20618;AC=1;AF=4.850130953535746e-05;N_HET=1;N_HOMALT=0;N_HOMREF=10308;FREQ_HET=9.700261907071492e-05;FREQ_HOMALT=0.0;FREQ_HOMREF=0.9999029973809292;N_BI_GENOS=10309;Northwest_Han_AN=1296;Northwest_Han_AC=1;No
chr7	38451979 .	T	<INS:ME:LINE1>	.	PASS	SVTYPE=INS:ME:LINE1;END=38451996;SVLEN=941;AN=20618;AC=1;AF=4.850130953535746e-05;N_HET=1;N_HOMALT=0;N_HOMREF=10308;FREQ_HET=9.700261907071492e-05;FREQ_HOMALT=0.0;FREQ_HOMREF=0.9999029973809292;N_BI_GENOS=10309;East_Han_AN=6554;East_Han_AC=1;East_Han_AF=0
chr7	38511814 .	C	<INS:ME:LINE1>	.	PASS	SVTYPE=INS:ME:LINE1;END=38511820;SVLEN=6142;AN=20618;AC=1;AF=4.850130953535746e-05;N_HET=1;N_HOMALT=0;N_HOMREF=10308;FREQ_HET=9.700261907071492e-05;FREQ_HOMALT=0.0;FREQ_HOMREF=0.9999029973809292;N_BI_GENOS=10309;North_Han_AN=2272;North_Han_AC=1;North_Han_
chr7	38779377 .	A	<INS:ME:LINE1>	.	PASS	SVTYPE=INS:ME:LINE1;END=38779393;SVLEN=696;AN=20618;AC=1;AF=4.850130953535746e-05;N_HET=1;N_HOMALT=0;N_HOMREF=10308;FREQ_HET=9.700261907071492e-05;FREQ_HOMALT=0.0;FREQ_HOMREF=0.9999029973809292;N_BI_GENOS=10309;Southeast_Han_AN=1908;Southeast_Han_AC=1;Sou
chr7	39065245 .	A	<INS:ME:LINE1>	.	PASS	SVTYPE=INS:ME:LINE1;END=39065259;SVLEN=1922;AN=20618;AC=1;AF=4.850130953535746e-05;N_HET=1;N_HOMALT=0;N_HOMREF=10308;FREQ_HET=9.700261907071492e-05;FREQ_HOMALT=0.0;FREQ_HOMREF=0.9999029973809292;N_BI_GENOS=10309;Mongolian_AN=454;Mongolian_AC=1;Mongolian_A
chr7	39414191 .	A	<INS:ME:LINE1>	.	PASS	SVTYPE=INS:ME:LINE1;END=39414195;SVLEN=200;AN=20618;AC=1;AF=4.850130953535746e-05;N_HET=1;N_HOMALT=0;N_HOMREF=10308;FREQ_HET=9.700261907071492e-05;FREQ_HOMALT=0.0;FREQ_HOMREF=0.9999029973809292;N_BI_GENOS=10309;North_Han_AN=2272;North_Han_AC=1;North_Han_A
chr7	40313204 .	A	<INS:ME:LINE1>	.	PASS	SVTYPE=INS:ME:LINE1;END=40313221;SVLEN=6005;AN=20618;AC=1;AF=4.850130953535746e-05;N_HET=1;N_HOMALT=0;N_HOMREF=10308;FREQ_HET=9.700261907071492e-05;FREQ_HOMALT=0.0;FREQ_HOMREF=0.9999029973809292;N_BI_GENOS=10309;North_Han_AN=2272;North_Han_AC=1;North_Han_
chr7	40500185 .	T	<INS:ME:LINE1>	.	PASS	SVTYPE=INS:ME:LINE1;END=40500199;SVLEN=6008;AN=20618;AC=1;AF=4.850130953535746e-05;N_HET=1;N_HOMALT=0;N_HOMREF=10308;FREQ_HET=9.700261907071492e-05;FREQ_HOMALT=0.0;FREQ_HOMREF=0.9999029973809292;N_BI_GENOS=10309;North_Han_AN=2272;North_Han_AC=1;North_Han_
chr7	40503705 .	A	<INS:ME:LINE1>	.	PASS	SVTYPE=INS:ME:LINE1;END=40503705;SVLEN=323;AN=20618;AC=2;AF=9.700261907071492e-05;N_HET=2;N_HOMALT=0;N_HOMREF=10307;FREQ_HET=0.0001940052381414298;FREQ_HOMALT=0.0;FREQ_HOMREF=0.9998059947618586;N_BI_GENOS=10309;East_Han_AN=6554;East_Han_AC=2;East_Han_AF=
chr7	40633138 .	A	<INS:ME:LINE1>	.	PASS	SVTYPE=INS:ME:LINE1;END=40633153;SVLEN=984;AN=20618;AC=5;AF=0.0002425065476767878;N_HET=5;N_HOMALT=0;N_HOMREF=10304;FREQ_HET=0.0004850130953535746;FREQ_HOMALT=0.0;FREQ_HOMREF=0.9995149869046465;N_BI_GENOS=10309;South_Han_AN=1496;South_Han_AC=1;South_Han
chr7	40866803 .	A	<INS:ME:LINE1>	.	PASS	SVTYPE=INS:ME:LINE1;END=40866810;SVLEN=535;AN=20618;AC=1;AF=4.850130953535746e-05;N_HET=1;N_HOMALT=0;N_HOMREF=10308;FREQ_HET=9.700261907071492e-05;FREQ_HOMALT=0.0;FREQ_HOMREF=0.9999029973809292;N_BI_GENOS=10309;Central_Han_AN=2128;Central_Han_AC=1;Central
chr7	40958754 .	G	<INS:ME:LINE1>	.	PASS	SVTYPE=INS:ME:LINE1;END=40958771;SVLEN=3330;AN=20618;AC=1;AF=4.850130953535746e-05;N_HET=1;N_HOMALT=0;N_HOMREF=10308;FREQ_HET=9.700261907071492e-05;FREQ_HOMALT=0.0;FREQ_HOMREF=0.9999029973809292;N_BI_GENOS=10309;Southeast_Han_AN=1908;Southeast_Han_AC=1;So
chr7	41047071 .	A	<INS:ME:LINE1>	.	PASS	SVTYPE=INS:ME:LINE1;END=41047071;SVLEN=1085;AN=20618;AC=1;AF=4.850130953535746e-05;N_HET=1;N_HOMALT=0;N_HOMREF=10308;FREQ_HET=9.700261907071492e-05;FREQ_HOMALT=0.0;FREQ_HOMREF=0.9999029973809292;N_BI_GENOS=10309;Central_Han_AN=2128;Central_Han_AC=1;Centra
chr7	41103439 .	A	<INS:ME:LINE1>	.	PASS	SVTYPE=INS:ME:LINE1;END=41103442;SVLEN=144;AN=20618;AC=1;AF=4.850130953535746e-05;N_HET=1;N_HOMALT=0;N_HOMREF=10308;FREQ_HET=9.700261907071492e-05;FREQ_HOMALT=0.0;FREQ_HOMREF=0.9999029973809292;N_BI_GENOS=10309;East_Han_AN=6554;East_Han_AC=1;East_Han_AF=0
chr7	41209299 .	A	<INS:ME:LINE1>	.	PASS	SVTYPE=INS:ME:LINE1;END=41209299;SVLEN=987;AN=20618;AC=1;AF=4.850130953535746e-05;N_HET=1;N_HOMALT=0;N_HOMREF=10308;FREQ_HET=9.700261907071492e-05;FREQ_HOMALT=0.0;FREQ_HOMREF=0.9999029973809292;N_BI_GENOS=10309;Central_Han_AN=2128;Central_Han_AC=1;Central
chr7	41271187 .	T	<INS:ME:LINE1>	.	PASS	SVTYPE=INS:ME:LINE1;END=41271202;SVLEN=377;AN=20618;AC=247;AF=0.01197982345523329;N_HET=241;N_HOMALT=3;N_HOMREF=10065;FREQ_HET=0.023377631196042293;FREQ_HOMALT=0.0002910078572121447;FREQ_HOMREF=0.9763313609467456;N_BI_GENOS=10309;South_Han_AN=1496;South_H
chr7	41871848 .	A	<INS:ME:LINE1>	.	PASS	SVTYPE=INS:ME:LINE1;END=41871864;SVLEN=922;AN=20618;AC=1;AF=4.850130953535746e-05;N_HET=1;N_HOMALT=0;N_HOMREF=10308;FREQ_HET=9.700261907071492e-05;FREQ_HOMALT=0.0;FREQ_HOMREF=0.9999029973809292;N_BI_GENOS=10309;East_Han_AN=6554;East_Han_AC=1;East_Han_AF=0
chr7	41983440 .	T	<INS:ME:LINE1>	.	PASS	SVTYPE=INS:ME:LINE1;END=41983456;SVLEN=1263;AN=20618;AC=1;AF=4.850130953535746e-05;N_HET=1;N_HOMALT=0;N_HOMREF=10308;FREQ_HET=9.700261907071492e-05;FREQ_HOMALT=0.0;FREQ_HOMREF=0.9999029973809292;N_BI_GENOS=10309;East_Han_AN=6554;East_Han_AC=1;East_Han_AF=
chr7	42162398 .	A	<INS:ME:LINE1>	.	PASS	SVTYPE=INS:ME:LINE1;END=42162416;SVLEN=1449;AN=20618;AC=4;AF=0.0001940052381414298;N_HET=4;N_HOMALT=0;N_HOMREF=10305;FREQ_HET=0.0003880104762828596;FREQ_HOMALT=0.0;FREQ_HOMREF=0.9996119895237171;N_BI_GENOS=10309;Central_Han_AN=2128;Central_Han_AC=1;Cent
chr7	42490264 .	T	<INS:ME:LINE1>	.	PASS	SVTYPE=INS:ME:LINE1;END=42490276;SVLEN=5994;AN=20618;AC=144;AF=0.006984188573091473;N_HET=142;N_HOMALT=1;N_HOMREF=10166;FREQ_HET=0.013774371908041518;FREQ_HOMALT=9.700261907071492e-05;FREQ_HOMREF=0.9861286254728878;N_BI_GENOS=10309;South_Han_AN=1496;South
chr7	42612182 .	A	<INS:ME:LINE1>	.	PASS	SVTYPE=INS:ME:LINE1;END=42612197;SVLEN=6107;AN=20618;AC=1;AF=4.850130953535746e-05;N_HET=1;N_HOMALT=0;N_HOMREF=10308;FREQ_HET=9.700261907071492e-05;FREQ_HOMALT=0.0;FREQ_HOMREF=0.9999029973809292;N_BI_GENOS=10309;East_Han_AN=6554;East_Han_AC=1;East_Han_AF=
chr7	42876143 .	C	<INS:ME:LINE1>	.	PASS	SVTYPE=INS:ME:LINE1;END=42876143;SVLEN=0;AN=20618;AC=2;AF=9.700261907071492e-05;N_HET=0;N_HOMALT=1;N_HOMREF=10308;FREQ_HET=0.0;FREQ_HOMALT=9.700261907071492e-05;FREQ_HOMREF=0.9999029973809292;N_BI_GENOS=10309;Zhuang_AN=444;Zhuang_AC=2;Zhuang_AF=0.00450450
chr7	43015714 .	A	<INS:ME:LINE1>	.	PASS	SVTYPE=INS:ME:LINE1;END=43015714;SVLEN=11;AN=20618;AC=1;AF=4.850130953535746e-05;N_HET=1;N_HOMALT=0;N_HOMREF=10308;FREQ_HET=9.700261907071492e-05;FREQ_HOMALT=0.0;FREQ_HOMREF=0.9999029973809292;N_BI_GENOS=10309;Lingnan_Han_AN=1888;Lingnan_Han_AC=1;Lingnan_
chr7	43042640 .	A	<INS:ME:LINE1>	.	PASS	SVTYPE=INS:ME:LINE1;END=43042656;SVLEN=5977;AN=20618;AC=3;AF=0.0001455039286060 7236;N_HET=3;N_HOMALT=0;N_HOMREF=10306;FREQ_HET=0.0002910078572121447;FREQ_HOMALT=0.0;FREQ_HOMREF=0.9997089921427879;N_BI_GENOS=10309;East_Han_AN=6554;East_Han_AC=3;East_Han_AF
chr7	43187567 .	C	<INS:ME:LINE1>	.	PASS	SVTYPE=INS:ME:LINE1;END=43187584;SVLEN=6000;AN=20618;AC=81;AF=0.003928606072363954;N_HET=79;N_HOMALT=1;N_HOMREF=10229;FREQ_HET=0.007663206906586477;FREQ_HOMALT=9.700261907071492e-05;FREQ_HOMREF=0.9922397904743429;N_BI_GENOS=10309;Northwest_Han_AN=1296;Nor
chr7	44315225 .	T	<INS:ME:LINE1>	.	PASS	SVTYPE=INS:ME:LINE1;END=44315240;SVLEN=6012;AN=20618;AC=2;AF=9.700261907071492e-05;N_HET=2;N_HOMALT=0;N_HOMREF=10307;FREQ_HET=0.0001940052381414298;FREQ_HOMALT=0.0;FREQ_HOMREF=0.9998059947618586;N_BI_GENOS=10309;North_Han_AN=2272;North_Han_AC=1;North_Han
chr7	44414063 .	A	<INS:ME:LINE1>	.	PASS	SVTYPE=INS:ME:LINE1;END=44414082;SVLEN=251;AN=20618;AC=1;AF=4.850130953535746e-05;N_HET=1;N_HOMALT=0;N_HOMREF=10308;FREQ_HET=9.700261907071492e-05;FREQ_HOMALT=0.0;FREQ_HOMREF=0.9999029973809292;N_BI_GENOS=10309;Central_Han_AN=2128;Central_Han_AC=1;Central
chr7	44430145 .	A	<INS:ME:LINE1>	.	PASS	SVTYPE=INS:ME:LINE1;END=44430161;SVLEN=5994;AN=20618;AC=1;AF=4.850130953535746e-05;N_HET=1;N_HOMALT=0;N_HOMREF=10308;FREQ_HET=9.700261907071492e-05;FREQ_HOMALT=0.0;FREQ_HOMREF=0.9999029973809292;N_BI_GENOS=10309;Central_Han_AN=2128;Central_Han_AC=1;Centra
chr7	44840474 .	A	<INS:ME:LINE1>	.	PASS	SVTYPE=INS:ME:LINE1;END=44840484;SVLEN=6003;AN=20618;AC=1;AF=4.850130953535746e-05;N_HET=1;N_HOMALT=0;N_HOMREF=10308;FREQ_HET=9.700261907071492e-05;FREQ_HOMALT=0.0;FREQ_HOMREF=0.9999029973809292;N_BI_GENOS=10309;East_Han_AN=6554;East_Han_AC=1;East_Han_AF=
chr7	44846034 .	A	<INS:ME:LINE1>	.	PASS	SVTYPE=INS:ME:LINE1;END=44846034;SVLEN=0;AN=20618;AC=1;AF=4.850130953535746e-05;N_HET=1;N_HOMALT=0;N_HOMREF=10308;FREQ_HET=9.700261907071492e-05;FREQ_HOMALT=0.0;FREQ_HOMREF=0.9999029973809292;N_BI_GENOS=10309;Northwest_Han_AN=1296;Northwest_Han_AC=1;North
chr7	45418236 .	A	<INS:ME:LINE1>	.	PASS	SVTYPE=INS:ME:LINE1;END=45418247;SVLEN=6000;AN=20618;AC=1;AF=4.850130953535746e-05;N_HET=1;N_HOMALT=0;N_HOMREF=10308;FREQ_HET=9.700261907071492e-05;FREQ_HOMALT=0.0;FREQ_HOMREF=0.9999029973809292;N_BI_GENOS=10309;Central_Han_AN=2128;Central_Han_AC=1;Centra
chr7	45701163 .	G	<INS:ME:LINE1>	.	PASS	SVTYPE=INS:ME:LINE1;END=45701174;SVLEN=6000;AN=20618;AC=1;AF=4.850130953535746e-05;N_HET=1;N_HOMALT=0;N_HOMREF=10308;FREQ_HET=9.700261907071492e-05;FREQ_HOMALT=0.0;FREQ_HOMREF=0.9999029973809292;N_BI_GENOS=10309;South_Han_AN=1496;South_Han_AC=1;South_Han_
chr7	45748338 .	C	<INS:ME:LINE1>	.	PASS	SVTYPE=INS:ME:LINE1;END=45748350;SVLEN=471;AN=20618;AC=13;AF=0.0006305170239596469;N_HET=13;N_HOMALT=0;N_HOMREF=10296;FREQ_HET=0.0012610340479192938;FREQ_HOMALT=0.0;FREQ_HOMREF=0.9987389659520807;N_BI_GENOS=10309;Northwest_Han_AN=1296;Northwest_Han_AC=1;N
chr7	46034946 .	A	<INS:ME:LINE1>	.	PASS	SVTYPE=INS:ME:LINE1;END=46034959;SVLEN=6000;AN=20618;AC=3;AF=0.0001455039286060 7236;N_HET=3;N_HOMALT=0;N_HOMREF=10306;FREQ_HET=0.0002910078572121447;FREQ_HOMALT=0.0;FREQ_HOMREF=0.9997089921427879;N_BI_GENOS=10309;Northwest_Han_AN=1296;Northwest_Han_AC=1;N
chr7	46287406 .	G	<INS:ME:LINE1>	.	PASS	SVTYPE=INS:ME:LINE1;END=46287417;SVLEN=311;AN=20618;AC=2;AF=9.700261907071492e-05;N_HET=2;N_HOMALT=0;N_HOMREF=10307;FREQ_HET=0.0001940052381414298;FREQ_HOMALT=0.0;FREQ_HOMREF=0.9998059947618586;N_BI_GENOS=10309;South_Han_AN=1496;South_Han_AC=1;South_Han_
chr7	46304532 .	T	<INS:ME:LINE1>	.	PASS	SVTYPE=INS:ME:LINE1;END=46304532;SVLEN=50;AN=20618;AC=1;AF=4.850130953535746e-05;N_HET=1;N_HOMALT=0;N_HOMREF=10308;FREQ_HET=9.700261907071492e-05;FREQ_HOMALT=0.0;FREQ_HOMREF=0.9999029973809292;N_BI_GENOS=10309;East_Han_AN=6554;East_Han_AC=1;East_Han_AF=0.
chr7	46310737 .	T	<INS:ME:LINE1>	.	PASS	SVTYPE=INS:ME:LINE1;END=46310750;SVLEN=520;AN=20618;AC=1323;AF=0.06416723251527791;N_HET=1219;N_HOMALT=52;N_HOMREF=9038;FREQ_HET=0.11824619264720147;FREQ_HOMALT=0.005044136191677175;FREQ_HOMREF=0.8767096711611213;N_BI_GENOS=10309;South_Han_AN=1496;South_H
chr7	46343500 .	A	<INS:ME:LINE1>	.	PASS	SVTYPE=INS:ME:LINE1;END=46343512;SVLEN=683;AN=20618;AC=4;AF=0.0001940052381414298;N_HET=4;N_HOMALT=0;N_HOMREF=10305;FREQ_HET=0.0003880104762828596;FREQ_HOMALT=0.0;FREQ_HOMREF=0.9996119895237171;N_BI_GENOS=10309;Northwest_Han_AN=1296;Northwest_Han_AC=1;N
chr7	46348253 .	T	<INS:ME:LINE1>	.	PASS	SVTYPE=INS:ME:LINE1;END=46348253;SVLEN=1926;AN=20618;AC=1;AF=4.850130953535746e-05;N_HET=1;N_HOMALT=0;N_HOMREF=10308;FREQ_HET=9.700261907071492e-05;FREQ_HOMALT=0.0;FREQ_HOMREF=0.9999029973809292;N_BI_GENOS=10309;North_Han_AN=2272;North_Han_AC=1;North_Han_
chr7	46348461 .	T	<INS:ME:LINE1>	.	PASS	SVTYPE=INS:ME:LINE1;END=46348474;SVLEN=894;AN=20618;AC=2;AF=9.700261907071492e-05;N_HET=2;N_HOMALT=0;N_HOMREF=10307;FREQ_HET=0.0001940052381414298;FREQ_HOMALT=0.0;FREQ_HOMREF=0.9998059947618586;N_BI_GENOS=10309;South_Han_AN=1496;South_Han_AC=1;South_Han_
chr7	46358593 .	A	<INS:ME:LINE1>	.	PASS	SVTYPE=INS:ME:LINE1;END=46358607;SVLEN=5981;AN=20618;AC=1;AF=4.850130953535746e-05;N_HET=1;N_HOMALT=0;N_HOMREF=10308;FREQ_HET=9.700261907071492e-05;FREQ_HOMALT=0.0;FREQ_HOMREF=0.9999029973809292;N_BI_GENOS=10309;North_Han_AN=2272;North_Han_AC=1;North_Han_
chr7	46408773 .	A	<INS:ME:LINE1>	.	PASS	SVTYPE=INS:ME:LINE1;END=46408773;SVLEN=980;AN=20618;AC=1;AF=4.850130953535746e-05;N_HET=1;N_HOMALT=0;N_HOMREF=10308;FREQ_HET=9.700261907071492e-05;FREQ_HOMALT=0.0;FREQ_HOMREF=0.9999029973809292;N_BI_GENOS=10309;Northwest_Han_AN=1296;Northwest_Han_AC=1;Nor
chr7	46644477 .	T	<INS:ME:LINE1>	.	PASS	SVTYPE=INS:ME:LINE1;END=46644477;SVLEN=225;AN=20618;AC=1;AF=4.850130953535746e-05;N_HET=1;N_HOMALT=0;N_HOMREF=10308;FREQ_HET=9.700261907071492e-05;FREQ_HOMALT=0.0;FREQ_HOMREF=0.9999029973809292;N_BI_GENOS=10309;Tibetan_AN=414;Tibetan_AC=1;Tibetan_AF=0.002
chr7	46684144 .	A	<INS:ME:LINE1>	.	PASS	SVTYPE=INS:ME:LINE1;END=46684157;SVLEN=5993;AN=20618;AC=1;AF=4.850130953535746e-05;N_HET=1;N_HOMALT=0;N_HOMREF=10308;FREQ_HET=9.700261907071492e-05;FREQ_HOMALT=0.0;FREQ_HOMREF=0.9999029973809292;N_BI_GENOS=10309;Southeast_Han_AN=1908;Southeast_Han_AC=1;So
chr7	46741802 .	A	<INS:ME:LINE1>	.	PASS	SVTYPE=INS:ME:LINE1;END=46741812;SVLEN=213;AN=20618;AC=1;AF=4.850130953535746e-05;N_HET=1;N_HOMALT=0;N_HOMREF=10308;FREQ_HET=9.700261907071492e-05;FREQ_HOMALT=0.0;FREQ_HOMREF=0.9999029973809292;N_BI_GENOS=10309;Hui_AN=444;Hui_AC=1;Hui_AF=0.002252252252252
chr7	46785002 .	A	<INS:ME:LINE1>	.	PASS	SVTYPE=INS:ME:LINE1;END=46785012;SVLEN=93;AN=20618;AC=1;AF=4.850130953535746e-05;N_HET=1;N_HOMALT=0;N_HOMREF=10308;FREQ_HET=9.700261907071492e-05;FREQ_HOMALT=0.0;FREQ_HOMREF=0.9999029973809292;N_BI_GENOS=10309;East_Han_AN=6554;East_Han_AC=1;East_Han_AF=0.
chr7	46998665 .	A	<INS:ME:LINE1>	.	PASS	SVTYPE=INS:ME:LINE1;END=46998676;SVLEN=567;AN=20618;AC=102;AF=0.004947133572606461;N_HET=102;N_HOMALT=0;N_HOMREF=10207;FREQ_HET=0.009894267145212922;FREQ_HOMALT=0.0;FREQ_HOMREF=0.9901057328547871;N_BI_GENOS=10309;South_Han_AN=1496;South_Han_AC=8;South_Han
chr7	47002393 .	C	<INS:ME:LINE1>	.	PASS	SVTYPE=INS:ME:LINE1;END=47002407;SVLEN=847;AN=20618;AC=1;AF=4.850130953535746e-05;N_HET=1;N_HOMALT=0;N_HOMREF=10308;FREQ_HET=9.700261907071492e-05;FREQ_HOMALT=0.0;FREQ_HOMREF=0.9999029973809292;N_BI_GENOS=10309;Central_Han_AN=2128;Central_Han_AC=1;Central
chr7	47116170 .	A	<INS:ME:LINE1>	.	PASS	SVTYPE=INS:ME:LINE1;END=47116189;SVLEN=5999;AN=20618;AC=1;AF=4.850130953535746e-05;N_HET=1;N_HOMALT=0;N_HOMREF=10308;FREQ_HET=9.700261907071492e-05;FREQ_HOMALT=0.0;FREQ_HOMREF=0.9999029973809292;N_BI_GENOS=10309;East_Han_AN=6554;East_Han_AC=1;East_Han_AF=
chr7	47136950 .	T	<INS:ME:LINE1>	.	PASS	SVTYPE=INS:ME:LINE1;END=47136966;SVLEN=6005;AN=20618;AC=1;AF=4.850130953535746e-05;N_HET=1;N_HOMALT=0;N_HOMREF=10308;FREQ_HET=9.700261907071492e-05;FREQ_HOMALT=0.0;FREQ_HOMREF=0.9999029973809292;N_BI_GENOS=10309;Southeast_Han_AN=1908;Southeast_Han_AC=1;So
chr7	47185746 .	G	<INS:ME:LINE1>	.	PASS	SVTYPE=INS:ME:LINE1;END=47185755;SVLEN=5998;AN=20618;AC=2;AF=9.700261907071492e-05;N_HET=2;N_HOMALT=0;N_HOMREF=10307;FREQ_HET=0.0001940052381414298;FREQ_HOMALT=0.0;FREQ_HOMREF=0.9998059947618586;N_BI_GENOS=10309;South_Han_AN=1496;South_Han_AC=1;South_Han
chr7	47825227 .	A	<INS:ME:LINE1>	.	PASS	SVTYPE=INS:ME:LINE1;END=47825240;SVLEN=5998;AN=20618;AC=1;AF=4.850130953535746e-05;N_HET=1;N_HOMALT=0;N_HOMREF=10308;FREQ_HET=9.700261907071492e-05;FREQ_HOMALT=0.0;FREQ_HOMREF=0.9999029973809292;N_BI_GENOS=10309;Central_Han_AN=2128;Central_Han_AC=1;Centra
chr7	48534047 .	G	<INS:ME:LINE1>	.	PASS	SVTYPE=INS:ME:LINE1;END=48534058;SVLEN=6048;AN=20618;AC=1;AF=4.850130953535746e-05;N_HET=1;N_HOMALT=0;N_HOMREF=10308;FREQ_HET=9.700261907071492e-05;FREQ_HOMALT=0.0;FREQ_HOMREF=0.9999029973809292;N_BI_GENOS=10309;South_Han_AN=1496;South_Han_AC=1;South_Han_
chr7	48736732 .	T	<INS:ME:LINE1>	.	PASS	SVTYPE=INS:ME:LINE1;END=48736732;SVLEN=0;AN=20618;AC=9;AF=0.0004365117858182171;N_HET=7;N_HOMALT=1;N_HOMREF=10301;FREQ_HET=0.0006790183334950044;FREQ_HOMALT=9.700261907071492e-05;FREQ_HOMREF=0.9992239790474343;N_BI_GENOS=10309;Northwest_Han_AN=1296;Northw
chr7	49148781 .	T	<INS:ME:LINE1>	.	PASS	SVTYPE=INS:ME:LINE1;END=49148781;SVLEN=908;AN=20618;AC=2;AF=9.700261907071492e-05;N_HET=0;N_HOMALT=1;N_HOMREF=10308;FREQ_HET=0.0;FREQ_HOMALT=9.700261907071492e-05;FREQ_HOMREF=0.9999029973809292;N_BI_GENOS=10309;East_Han_AN=6554;East_Han_AC=2;East_Han_AF=0
chr7	49149217 .	T	<INS:ME:LINE1>	.	PASS	SVTYPE=INS:ME:LINE1;END=49149217;SVLEN=685;AN=20618;AC=1;AF=4.850130953535746e-05;N_HET=1;N_HOMALT=0;N_HOMREF=10308;FREQ_HET=9.700261907071492e-05;FREQ_HOMALT=0.0;FREQ_HOMREF=0.9999029973809292;N_BI_GENOS=10309;East_Han_AN=6554;East_Han_AC=1;East_Han_AF=0
chr7	49200396 .	A	<INS:ME:LINE1>	.	PASS	SVTYPE=INS:ME:LINE1;END=49200412;SVLEN=5998;AN=20618;AC=1;AF=4.850130953535746e-05;N_HET=1;N_HOMALT=0;N_HOMREF=10308;FREQ_HET=9.700261907071492e-05;FREQ_HOMALT=0.0;FREQ_HOMREF=0.9999029973809292;N_BI_GENOS=10309;Central_Han_AN=2128;Central_Han_AC=1;Centra
chr7	49476370 .	G	<INS:ME:LINE1>	.	PASS	SVTYPE=INS:ME:LINE1;END=49476370;SVLEN=187;AN=20618;AC=1;AF=4.850130953535746e-05;N_HET=1;N_HOMALT=0;N_HOMREF=10308;FREQ_HET=9.700261907071492e-05;FREQ_HOMALT=0.0;FREQ_HOMREF=0.9999029973809292;N_BI_GENOS=10309;East_Han_AN=6554;East_Han_AC=1;East_Han_AF=0
chr7	49477084 .	G	<INS:ME:LINE1>	.	PASS	SVTYPE=INS:ME:LINE1;END=49477084;SVLEN=802;AN=20618;AC=1;AF=4.850130953535746e-05;N_HET=1;N_HOMALT=0;N_HOMREF=10308;FREQ_HET=9.700261907071492e-05;FREQ_HOMALT=0.0;FREQ_HOMREF=0.9999029973809292;N_BI_GENOS=10309;East_Han_AN=6554;East_Han_AC=1;East_Han_AF=0
chr7	49557494 .	C	<INS:ME:LINE1>	.	PASS	SVTYPE=INS:ME:LINE1;END=49557494;SVLEN=589;AN=20618;AC=1;AF=4.850130953535746e-05;N_HET=1;N_HOMALT=0;N_HOMREF=10308;FREQ_HET=9.700261907071492e-05;FREQ_HOMALT=0.0;FREQ_HOMREF=0.9999029973809292;N_BI_GENOS=10309;East_Han_AN=6554;East_Han_AC=1;East_Han_AF=0
chr7	49740724 .	A	<INS:ME:LINE1>	.	PASS	SVTYPE=INS:ME:LINE1;END=49740742;SVLEN=1548;AN=20618;AC=1;AF=4.850130953535746e-05;N_HET=1;N_HOMALT=0;N_HOMREF=10308;FREQ_HET=9.700261907071492e-05;FREQ_HOMALT=0.0;FREQ_HOMREF=0.9999029973809292;N_BI_GENOS=10309;Central_Han_AN=2128;Central_Han_AC=1;Centra
chr7	49977627 .	A	<INS:ME:LINE1>	.	PASS	SVTYPE=INS:ME:LINE1;END=49977667;SVLEN=4119;AN=20618;AC=5;AF=0.0002425065476767878;N_HET=5;N_HOMALT=0;N_HOMREF=10304;FREQ_HET=0.0004850130953535746;FREQ_HOMALT=0.0;FREQ_HOMREF=0.9995149869046465;N_BI_GENOS=10309;North_Han_AN=2272;North_Han_AC=1;North_Ha
chr7	50051262 .	T	<INS:ME:LINE1>	.	PASS	SVTYPE=INS:ME:LINE1;END=50051262;SVLEN=623;AN=20618;AC=1;AF=4.850130953535746e-05;N_HET=1;N_HOMALT=0;N_HOMREF=10308;FREQ_HET=9.700261907071492e-05;FREQ_HOMALT=0.0;FREQ_HOMREF=0.9999029973809292;N_BI_GENOS=10309;East_Han_AN=6554;East_Han_AC=1;East_Han_AF=0
chr7	50051765 .	A	<INS:ME:LINE1>	.	PASS	SVTYPE=INS:ME:LINE1;END=50051765;SVLEN=823;AN=20618;AC=2;AF=9.700261907071492e-05;N_HET=0;N_HOMALT=1;N_HOMREF=10308;FREQ_HET=0.0;FREQ_HOMALT=9.700261907071492e-05;FREQ_HOMREF=0.9999029973809292;N_BI_GENOS=10309;East_Han_AN=6554;East_Han_AC=2;East_Han_AF=0
chr7	50059071 .	A	<INS:ME:LINE1>	.	PASS	SVTYPE=INS:ME:LINE1;END=50059086;SVLEN=985;AN=20618;AC=1;AF=4.850130953535746e-05;N_HET=1;N_HOMALT=0;N_HOMREF=10308;FREQ_HET=9.700261907071492e-05;FREQ_HOMALT=0.0;FREQ_HOMREF=0.9999029973809292;N_BI_GENOS=10309;Manchu_AN=416;Manchu_AC=1;Manchu_AF=0.002403
chr7	50080856 .	T	<INS:ME:LINE1>	.	PASS	SVTYPE=INS:ME:LINE1;END=50080872;SVLEN=1212;AN=20618;AC=6;AF=0.0002910078572121447;N_HET=6;N_HOMALT=0;N_HOMREF=10303;FREQ_HET=0.0005820157144242894;FREQ_HOMALT=0.0;FREQ_HOMREF=0.9994179842855757;N_BI_GENOS=10309;South_Han_AN=1496;South_Han_AC=2;South_Han_
chr7	50770775 .	G	<INS:ME:LINE1>	.	PASS	SVTYPE=INS:ME:LINE1;END=50770790;SVLEN=5998;AN=20618;AC=1;AF=4.850130953535746e-05;N_HET=1;N_HOMALT=0;N_HOMREF=10308;FREQ_HET=9.700261907071492e-05;FREQ_HOMALT=0.0;FREQ_HOMREF=0.9999029973809292;N_BI_GENOS=10309;North_Han_AN=2272;North_Han_AC=1;North_Han_
chr7	51526615 .	A	<INS:ME:LINE1>	.	PASS	SVTYPE=INS:ME:LINE1;END=51526615;SVLEN=907;AN=20618;AC=5;AF=0.0002425065476767878;N_HET=1;N_HOMALT=2;N_HOMREF=10306;FREQ_HET=9.700261907071492e-05;FREQ_HOMALT=0.0001940052381414298;FREQ_HOMREF=0.9997089921427879;N_BI_GENOS=10309;East_Han_AN=6554;East_Ha
chr7	51680937 .	A	<INS:ME:LINE1>	.	PASS	SVTYPE=INS:ME:LINE1;END=51680945;SVLEN=2729;AN=20618;AC=1;AF=4.850130953535746e-05;N_HET=1;N_HOMALT=0;N_HOMREF=10308;FREQ_HET=9.700261907071492e-05;FREQ_HOMALT=0.0;FREQ_HOMREF=0.9999029973809292;N_BI_GENOS=10309;South_Han_AN=1496;South_Han_AC=1;South_Han_
chr7	51858465 .	C	<INS:ME:LINE1>	.	PASS	SVTYPE=INS:ME:LINE1;END=51858465;SVLEN=349;AN=20618;AC=1;AF=4.850130953535746e-05;N_HET=1;N_HOMALT=0;N_HOMREF=10308;FREQ_HET=9.700261907071492e-05;FREQ_HOMALT=0.0;FREQ_HOMREF=0.9999029973809292;N_BI_GENOS=10309;South_Han_AN=1496;South_Han_AC=1;South_Han_A
chr7	52087607 .	A	<INS:ME:LINE1>	.	PASS	SVTYPE=INS:ME:LINE1;END=52087624;SVLEN=639;AN=20618;AC=3;AF=0.0001455039286060 7236;N_HET=3;N_HOMALT=0;N_HOMREF=10306;FREQ_HET=0.0002910078572121447;FREQ_HOMALT=0.0;FREQ_HOMREF=0.9997089921427879;N_BI_GENOS=10309;Northwest_Han_AN=1296;Northwest_Han_AC=1;No
chr7	52172626 .	T	<INS:ME:LINE1>	.	PASS	SVTYPE=INS:ME:LINE1;END=52172641;SVLEN=1932;AN=20618;AC=80;AF=0.0038801047628285964;N_HET=78;N_HOMALT=1;N_HOMREF=10230;FREQ_HET=0.007566204287515763;FREQ_HOMALT=9.700261907071492e-05;FREQ_HOMREF=0.9923367930934135;N_BI_GENOS=10309;South_Han_AN=1496;South_
chr7	52483860 .	T	<INS:ME:LINE1>	.	PASS	SVTYPE=INS:ME:LINE1;END=52483876;SVLEN=2350;AN=20618;AC=1;AF=4.850130953535746e-05;N_HET=1;N_HOMALT=0;N_HOMREF=10308;FREQ_HET=9.700261907071492e-05;FREQ_HOMALT=0.0;FREQ_HOMREF=0.9999029973809292;N_BI_GENOS=10309;East_Han_AN=6554;East_Han_AC=1;East_Han_AF=
chr7	52499833 .	A	<INS:ME:LINE1>	.	PASS	SVTYPE=INS:ME:LINE1;END=52499849;SVLEN=987;AN=20618;AC=1;AF=4.850130953535746e-05;N_HET=1;N_HOMALT=0;N_HOMREF=10308;FREQ_HET=9.700261907071492e-05;FREQ_HOMALT=0.0;FREQ_HOMREF=0.9999029973809292;N_BI_GENOS=10309;Southeast_Han_AN=1908;Southeast_Han_AC=1;Sou
chr7	52578260 .	T	<INS:ME:LINE1>	.	PASS	SVTYPE=INS:ME:LINE1;END=52578276;SVLEN=241;AN=20618;AC=1;AF=4.850130953535746e-05;N_HET=1;N_HOMALT=0;N_HOMREF=10308;FREQ_HET=9.700261907071492e-05;FREQ_HOMALT=0.0;FREQ_HOMREF=0.9999029973809292;N_BI_GENOS=10309;Northwest_Han_AN=1296;Northwest_Han_AC=1;Nor
chr7	52598914 .	A	<INS:ME:LINE1>	.	PASS	SVTYPE=INS:ME:LINE1;END=52598931;SVLEN=6011;AN=20618;AC=1;AF=4.850130953535746e-05;N_HET=1;N_HOMALT=0;N_HOMREF=10308;FREQ_HET=9.700261907071492e-05;FREQ_HOMALT=0.0;FREQ_HOMREF=0.9999029973809292;N_BI_GENOS=10309;Lingnan_Han_AN=1888;Lingnan_Han_AC=1;Lingna
chr7	52721765 .	A	<INS:ME:LINE1>	.	PASS	SVTYPE=INS:ME:LINE1;END=52721778;SVLEN=267;AN=20618;AC=7915;AF=0.3838878649723543;N_HET=975;N_HOMALT=3470;N_HOMREF=5864;FREQ_HET=0.09457755359394704;FREQ_HOMALT=0.33659908817538076;FREQ_HOMREF=0.5688233582306722;N_BI_GENOS=10309;South_Han_AN=1496;South_Ha
chr7	52869601 .	T	<INS:ME:LINE1>	.	PASS	SVTYPE=INS:ME:LINE1;END=52869614;SVLEN=1659;AN=20618;AC=1;AF=4.850130953535746e-05;N_HET=1;N_HOMALT=0;N_HOMREF=10308;FREQ_HET=9.700261907071492e-05;FREQ_HOMALT=0.0;FREQ_HOMREF=0.9999029973809292;N_BI_GENOS=10309;East_Han_AN=6554;East_Han_AC=1;East_Han_AF=

chr7	53171538	.	T	<INS:ME:LINE1>	.	PASS	SVTYPE=INS:ME:LINE1;END=53171550;SVLEN=1215;AN=20618;AC=1;AF=4.850130953535746e-05;N_HET=1;N_HOMALT=0;N_HOMREF=10308;FREQ_HET=9.700261907071492e-05;FREQ_HOMALT=0.0;FREQ_HOMREF=0.9999029973809292;N_BI_GENOS=10309;North_Han_AN=2272;North_Han_AC=1;North_Han_
chr7	53173994	.	A	<INS:ME:LINE1>	.	PASS	SVTYPE=INS:ME:LINE1;END=53174012;SVLEN=1817;AN=20618;AC=2;AF=9.700261907071492e-05;N_HET=2;N_HOMALT=0;N_HOMREF=10307;FREQ_HET=0.00019400523814142983;FREQ_HOMALT=0.0;FREQ_HOMREF=0.9998059947618586;N_BI_GENOS=10309;Hui_AN=444;Hui_AC=2;Hui_AF=0.0045045045045
chr7	53307847	.	A	<INS:ME:LINE1>	.	PASS	SVTYPE=INS:ME:LINE1;END=53307863;SVLEN=764;AN=20618;AC=8;AF=0.0003880104762828966;N_HET=8;N_HOMALT=0;N_HOMREF=10301;FREQ_HET=0.0007760209525657193;FREQ_HOMALT=0.0;FREQ_HOMREF=0.9992239790474343;N_BI_GENOS=10309;South_Han_AN=1496;South_Han_AC=1;South_Han_
chr7	53383880	.	A	<INS:ME:LINE1>	.	PASS	SVTYPE=INS:ME:LINE1;END=53383893;SVLEN=472;AN=20618;AC=1;AF=4.850130953535746e-05;N_HET=1;N_HOMALT=0;N_HOMREF=10308;FREQ_HET=9.700261907071492e-05;FREQ_HOMALT=0.0;FREQ_HOMREF=0.9999029973809292;N_BI_GENOS=10309;East_Han_AN=6554;East_Han_AC=1;East_Han_AF=0
chr7	53393011	.	A	<INS:ME:LINE1>	.	PASS	SVTYPE=INS:ME:LINE1;END=53393028;SVLEN=5997;AN=20618;AC=1;AF=4.850130953535746e-05;N_HET=1;N_HOMALT=0;N_HOMREF=10308;FREQ_HET=9.700261907071492e-05;FREQ_HOMALT=0.0;FREQ_HOMREF=0.9999029973809292;N_BI_GENOS=10309;North_Han_AN=2272;North_Han_AC=1;North_Han_
chr7	53584808	.	T	<INS:ME:LINE1>	.	PASS	SVTYPE=INS:ME:LINE1;END=53584823;SVLEN=6000;AN=20618;AC=2065;AF=0.10015520419051314;N_HET=1685;N_HOMALT=190;N_HOMREF=8434;FREQ_HET=0.16344941313415462;FREQ_HOMALT=0.01843049762343583;FREQ_HOMREF=0.8181200892424095;N_BI_GENOS=10309;South_Han_AN=1496;South_
chr7	53644127	.	G	<INS:ME:LINE1>	.	PASS	SVTYPE=INS:ME:LINE1;END=53644138;SVLEN=216;AN=20618;AC=1;AF=4.850130953535746e-05;N_HET=1;N_HOMALT=0;N_HOMREF=10308;FREQ_HET=9.700261907071492e-05;FREQ_HOMALT=0.0;FREQ_HOMREF=0.9999029973809292;N_BI_GENOS=10309;Lingnan_Han_AN=1888;Lingnan_Han_AC=1;Lingnan
chr7	53678215	.	A	<INS:ME:LINE1>	.	PASS	SVTYPE=INS:ME:LINE1;END=53678215;SVLEN=359;AN=20618;AC=1;AF=4.850130953535746e-05;N_HET=1;N_HOMALT=0;N_HOMREF=10308;FREQ_HET=9.700261907071492e-05;FREQ_HOMALT=0.0;FREQ_HOMREF=0.9999029973809292;N_BI_GENOS=10309;East_Han_AN=6554;East_Han_AC=1;East_Han_AF=0
chr7	53678951	.	A	<INS:ME:LINE1>	.	PASS	SVTYPE=INS:ME:LINE1;END=53678993;SVLEN=1637;AN=20618;AC=1;AF=4.850130953535746e-05;N_HET=1;N_HOMALT=0;N_HOMREF=10308;FREQ_HET=9.700261907071492e-05;FREQ_HOMALT=0.0;FREQ_HOMREF=0.9999029973809292;N_BI_GENOS=10309;East_Han_AN=6554;East_Han_AC=1;East_Han_AF=
chr7	53680000	.	T	<INS:ME:LINE1>	.	PASS	SVTYPE=INS:ME:LINE1;END=53680000;SVLEN=69;AN=20618;AC=1;AF=4.850130953535746e-05;N_HET=1;N_HOMALT=0;N_HOMREF=10308;FREQ_HET=9.700261907071492e-05;FREQ_HOMALT=0.0;FREQ_HOMREF=0.9999029973809292;N_BI_GENOS=10309;East_Han_AN=6554;East_Han_AC=1;East_Han_AF=0.
chr7	53784034	.	A	<INS:ME:LINE1>	.	PASS	SVTYPE=INS:ME:LINE1;END=53784050;SVLEN=5998;AN=20618;AC=1;AF=4.850130953535746e-05;N_HET=1;N_HOMALT=0;N_HOMREF=10308;FREQ_HET=9.700261907071492e-05;FREQ_HOMALT=0.0;FREQ_HOMREF=0.9999029973809292;N_BI_GENOS=10309;Northwest_Han_AN=1296;Northwest_Han_AC=1;No
chr7	53828081	.	A	<INS:ME:LINE1>	.	PASS	SVTYPE=INS:ME:LINE1;END=53828081;SVLEN=1311;AN=20618;AC=1;AF=4.850130953535746e-05;N_HET=1;N_HOMALT=0;N_HOMREF=10308;FREQ_HET=9.700261907071492e-05;FREQ_HOMALT=0.0;FREQ_HOMREF=0.9999029973809292;N_BI_GENOS=10309;East_Han_AN=6554;East_Han_AC=1;East_Han_AF=
chr7	54002210	.	C	<INS:ME:LINE1>	.	PASS	SVTYPE=INS:ME:LINE1;END=54002227;SVLEN=5983;AN=20618;AC=1;AF=4.850130953535746e-05;N_HET=1;N_HOMALT=0;N_HOMREF=10308;FREQ_HET=9.700261907071492e-05;FREQ_HOMALT=0.0;FREQ_HOMREF=0.9999029973809292;N_BI_GENOS=10309;North_Han_AN=2272;North_Han_AC=1;North_Han_
chr7	54170418	.	T	<INS:ME:LINE1>	.	PASS	SVTYPE=INS:ME:LINE1;END=54170431;SVLEN=535;AN=20618;AC=1;AF=4.850130953535746e-05;N_HET=1;N_HOMALT=0;N_HOMREF=10308;FREQ_HET=9.700261907071492e-05;FREQ_HOMALT=0.0;FREQ_HOMREF=0.9999029973809292;N_BI_GENOS=10309;Southeast_Han_AN=1908;Southeast_Han_AC=1;Sou
chr7	54729532	.	T	<INS:ME:LINE1>	.	PASS	SVTYPE=INS:ME:LINE1;END=54729548;SVLEN=309;AN=20618;AC=1;AF=4.850130953535746e-05;N_HET=1;N_HOMALT=0;N_HOMREF=10308;FREQ_HET=9.700261907071492e-05;FREQ_HOMALT=0.0;FREQ_HOMREF=0.9999029973809292;N_BI_GENOS=10309;East_Han_AN=6554;East_Han_AC=1;East_Han_AF=0
chr7	55058483	.	A	<INS:ME:LINE1>	.	PASS	SVTYPE=INS:ME:LINE1;END=55058496;SVLEN=5997;AN=20618;AC=1;AF=4.850130953535746e-05;N_HET=1;N_HOMALT=0;N_HOMREF=10308;FREQ_HET=9.700261907071492e-05;FREQ_HOMALT=0.0;FREQ_HOMREF=0.9999029973809292;N_BI_GENOS=10309;Central_Han_AN=2128;Central_Han_AC=1;Centra
chr7	55250292	.	T	<INS:ME:LINE1>	.	PASS	SVTYPE=INS:ME:LINE1;END=55250307;SVLEN=1219;AN=20618;AC=1;AF=4.850130953535746e-05;N_HET=1;N_HOMALT=0;N_HOMREF=10308;FREQ_HET=9.700261907071492e-05;FREQ_HOMALT=0.0;FREQ_HOMREF=0.9999029973809292;N_BI_GENOS=10309;Central_Han_AN=2128;Central_Han_AC=1;Centra
chr7	55663224	.	A	<INS:ME:LINE1>	.	PASS	SVTYPE=INS:ME:LINE1;END=55663239;SVLEN=6015;AN=20618;AC=3;AF=0.00014550392860607236;N_HET=1;N_HOMALT=1;N_HOMREF=10307;FREQ_HET=9.700261907071492e-05;FREQ_HOMALT=9.700261907071492e-05;FREQ_HOMREF=0.9998059947618586;N_BI_GENOS=10309;East_Han_AN=6554;East_Ha
chr7	55734533	.	A	<INS:ME:LINE1>	.	PASS	SVTYPE=INS:ME:LINE1;END=55734548;SVLEN=5997;AN=20618;AC=2;AF=9.700261907071492e-05;N_HET=2;N_HOMALT=0;N_HOMREF=10307;FREQ_HET=0.00019400523814142983;FREQ_HOMALT=0.0;FREQ_HOMREF=0.9998059947618586;N_BI_GENOS=10309;North_Han_AN=2272;North_Han_AC=1;North_Han
chr7	55993764	.	T	<INS:ME:LINE1>	.	PASS	SVTYPE=INS:ME:LINE1;END=55993781;SVLEN=140;AN=20618;AC=1;AF=4.850130953535746e-05;N_HET=1;N_HOMALT=0;N_HOMREF=10308;FREQ_HET=9.700261907071492e-05;FREQ_HOMALT=0.0;FREQ_HOMREF=0.9999029973809292;N_BI_GENOS=10309;Northwest_Han_AN=1296;Northwest_Han_AC=1;Nor
chr7	56148415	.	A	<INS:ME:LINE1>	.	PASS	SVTYPE=INS:ME:LINE1;END=56148431;SVLEN=5989;AN=20618;AC=1;AF=4.850130953535746e-05;N_HET=1;N_HOMALT=0;N_HOMREF=10308;FREQ_HET=9.700261907071492e-05;FREQ_HOMALT=0.0;FREQ_HOMREF=0.9999029973809292;N_BI_GENOS=10309;East_Han_AN=6554;East_Han_AC=1;East_Han_AF=
chr7	56189932	.	G	<INS:ME:LINE1>	.	PASS	SVTYPE=INS:ME:LINE1;END=56189943;SVLEN=6053;AN=20618;AC=1;AF=4.850130953535746e-05;N_HET=1;N_HOMALT=0;N_HOMREF=10308;FREQ_HET=9.700261907071492e-05;FREQ_HOMALT=0.0;FREQ_HOMREF=0.9999029973809292;N_BI_GENOS=10309;East_Han_AN=6554;East_Han_AC=1;East_Han_AF=
chr7	56647383	.	T	<INS:ME:LINE1>	.	PASS	SVTYPE=INS:ME:LINE1;END=56647383;SVLEN=839;AN=20618;AC=3;AF=0.00014550392860607236;N_HET=3;N_HOMALT=0;N_HOMREF=10306;FREQ_HET=0.0002910078572121447;FREQ_HOMALT=0.0;FREQ_HOMREF=0.9997089921427879;N_BI_GENOS=10309;North_Han_AN=2272;North_Han_AC=1;North_Han_
chr7	56707554	.	G	<INS:ME:LINE1>	.	PASS	SVTYPE=INS:ME:LINE1;END=56707568;SVLEN=3528;AN=20618;AC=1;AF=4.850130953535746e-05;N_HET=1;N_HOMALT=0;N_HOMREF=10308;FREQ_HET=9.700261907071492e-05;FREQ_HOMALT=0.0;FREQ_HOMREF=0.9999029973809292;N_BI_GENOS=10309;North_Han_AN=2272;North_Han_AC=1;North_Han_
chr7	57094296	.	T	<INS:ME:LINE1>	.	PASS	SVTYPE=INS:ME:LINE1;END=57094296;SVLEN=323;AN=20618;AC=1;AF=4.850130953535746e-05;N_HET=1;N_HOMALT=0;N_HOMREF=10308;FREQ_HET=9.700261907071492e-05;FREQ_HOMALT=0.0;FREQ_HOMREF=0.9999029973809292;N_BI_GENOS=10309;Tibetan_AN=414;Tibetan_AC=1;Tibetan_AF=0.002
chr7	57480818	.	A	<INS:ME:LINE1>	.	PASS	SVTYPE=INS:ME:LINE1;END=57480833;SVLEN=857;AN=20618;AC=1;AF=4.850130953535746e-05;N_HET=1;N_HOMALT=0;N_HOMREF=10308;FREQ_HET=9.700261907071492e-05;FREQ_HOMALT=0.0;FREQ_HOMREF=0.9999029973809292;N_BI_GENOS=10309;East_Han_AN=6554;East_Han_AC=1;East_Han_AF=0
chr7	57589870	.	A	<INS:ME:LINE1>	.	PASS	SVTYPE=INS:ME:LINE1;END=57589879;SVLEN=828;AN=20618;AC=3;AF=0.00014550392860607236;N_HET=3;N_HOMALT=0;N_HOMREF=10306;FREQ_HET=0.0002910078572121447;FREQ_HOMALT=0.0;FREQ_HOMREF=0.9997089921427879;N_BI_GENOS=10309;North_Han_AN=2272;North_Han_AC=2;North_Han_
chr7	57702761	.	A	<INS:ME:LINE1>	.	PASS	SVTYPE=INS:ME:LINE1;END=57702774;SVLEN=260;AN=20618;AC=3;AF=0.00014550392860607236;N_HET=3;N_HOMALT=0;N_HOMREF=10306;FREQ_HET=0.0002910078572121447;FREQ_HOMALT=0.0;FREQ_HOMREF=0.9997089921427879;N_BI_GENOS=10309;East_Han_AN=6554;East_Han_AC=2;East_Han_AF=
chr7	57702967	.	A	<INS:ME:LINE1>	.	PASS	SVTYPE=INS:ME:LINE1;END=57702981;SVLEN=1529;AN=20618;AC=12;AF=0.0005820157144242894;N_HET=10;N_HOMALT=1;N_HOMREF=10298;FREQ_HET=0.0009700261907071491;FREQ_HOMALT=9.700261907071492e-05;FREQ_HOMREF=0.9989329711902222;N_BI_GENOS=10309;South_Han_AN=1496;South
chr7	57734605	.	A	<INS:ME:LINE1>	.	PASS	SVTYPE=INS:ME:LINE1;END=57734627;SVLEN=988;AN=20618;AC=2;AF=9.700261907071492e-05;N_HET=2;N_HOMALT=0;N_HOMREF=10307;FREQ_HET=0.00019400523814142983;FREQ_HOMALT=0.0;FREQ_HOMREF=0.9998059947618586;N_BI_GENOS=10309;East_Han_AN=6554;East_Han_AC=2;East_Han_AF=
chr7	57741174	.	A	<INS:ME:LINE1>	.	PASS	SVTYPE=INS:ME:LINE1;END=57741189;SVLEN=3660;AN=20618;AC=2;AF=9.700261907071492e-05;N_HET=2;N_HOMALT=0;N_HOMREF=10307;FREQ_HET=0.00019400523814142983;FREQ_HOMALT=0.0;FREQ_HOMREF=0.9998059947618586;N_BI_GENOS=10309;Lingnan_Han_AN=1888;Lingnan_Han_AC=2;Lingn
chr7	57791409	.	A	<INS:ME:LINE1>	.	PASS	SVTYPE=INS:ME:LINE1;END=57791422;SVLEN=711;AN=20618;AC=1;AF=4.850130953535746e-05;N_HET=1;N_HOMALT=0;N_HOMREF=10308;FREQ_HET=9.700261907071492e-05;FREQ_HOMALT=0.0;FREQ_HOMREF=0.9999029973809292;N_BI_GENOS=10309;Northwest_Han_AN=1296;Northwest_Han_AC=1;Nor
chr7	57863677	.	A	<INS:ME:LINE1>	.	PASS	SVTYPE=INS:ME:LINE1;END=57863687;SVLEN=667;AN=20618;AC=3;AF=0.00014550392860607236;N_HET=3;N_HOMALT=0;N_HOMREF=10306;FREQ_HET=0.0002910078572121447;FREQ_HOMALT=0.0;FREQ_HOMREF=0.9997089921427879;N_BI_GENOS=10309;Lingnan_Han_AN=1888;Lingnan_Han_AC=3;Lingna
chr7	57925654	.	T	<INS:ME:LINE1>	.	PASS	SVTYPE=INS:ME:LINE1;END=57925659;SVLEN=3125;AN=20618;AC=8;AF=0.0003880104762828966;N_HET=8;N_HOMALT=0;N_HOMREF=10301;FREQ_HET=0.0007760209525657193;FREQ_HOMALT=0.0;FREQ_HOMREF=0.9992239790474343;N_BI_GENOS=10309;South_Han_AN=1496;South_Han_AC=1;South_Han
chr7	57948451	.	C	<INS:ME:LINE1>	.	PASS	SVTYPE=INS:ME:LINE1;END=57948468;SVLEN=371;AN=20618;AC=21;AF=0.0010185275002425065;N_HET=19;N_HOMALT=1;N_HOMREF=10289;FREQ_HET=0.0018430497623435833;FREQ_HOMALT=9.700261907071492e-05;FREQ_HOMREF=0.9980599476185857;N_BI_GENOS=10309;South_Han_AN=1496;South_
chr7	57954589	.	G	<INS:ME:LINE1>	.	PASS	SVTYPE=INS:ME:LINE1;END=57954590;SVLEN=2036;AN=20618;AC=1;AF=4.850130953535746e-05;N_HET=1;N_HOMALT=0;N_HOMREF=10308;FREQ_HET=9.700261907071492e-05;FREQ_HOMALT=0.0;FREQ_HOMREF=0.9999029973809292;N_BI_GENOS=10309;North_Han_AN=2272;North_Han_AC=1;North_Han_
chr7	57978434	.	T	<INS:ME:LINE1>	.	PASS	SVTYPE=INS:ME:LINE1;END=57978452;SVLEN=77;AN=20618;AC=1;AF=4.850130953535746e-05;N_HET=1;N_HOMALT=0;N_HOMREF=10308;FREQ_HET=9.700261907071492e-05;FREQ_HOMALT=0.0;FREQ_HOMREF=0.9999029973809292;N_BI_GENOS=10309;North_Han_AN=2272;North_Han_AC=1;North_Han_AF
chr7	57991522	.	A	<INS:ME:LINE1>	.	PASS	SVTYPE=INS:ME:LINE1;END=57991522;SVLEN=758;AN=20618;AC=2;AF=9.700261907071492e-05;N_HET=2;N_HOMALT=0;N_HOMREF=10307;FREQ_HET=0.00019400523814142983;FREQ_HOMALT=0.0;FREQ_HOMREF=0.9998059947618586;N_BI_GENOS=10309;Lingnan_Han_AN=1888;Lingnan_Han_AC=1;Lingna
chr7	58013847	.	A	<INS:ME:LINE1>	.	PASS	SVTYPE=INS:ME:LINE1;END=58013862;SVLEN=1090;AN=20618;AC=1;AF=4.850130953535746e-05;N_HET=1;N_HOMALT=0;N_HOMREF=10308;FREQ_HET=9.700261907071492e-05;FREQ_HOMALT=0.0;FREQ_HOMREF=0.9999029973809292;N_BI_GENOS=10309;East_Han_AN=6554;East_Han_AC=1;East_Han_AF=
chr7	60958353	.	C	<INS:ME:LINE1>	.	PASS	SVTYPE=INS:ME:LINE1;END=60958353;SVLEN=0;AN=20618;AC=1;AF=4.850130953535746e-05;N_HET=1;N_HOMALT=0;N_HOMREF=10308;FREQ_HET=9.700261907071492e-05;FREQ_HOMALT=0.0;FREQ_HOMREF=0.9999029973809292;N_BI_GENOS=10309;North_Han_AN=2272;North_Han_AC=1;North_Han_AF=
chr7	60958813	.	G	<INS:ME:LINE1>	.	PASS	SVTYPE=INS:ME:LINE1;END=60958830;SVLEN=850;AN=20618;AC=6;AF=0.0002910078572121447;N_HET=6;N_HOMALT=0;N_HOMREF=10303;FREQ_HET=0.0005820157144242894;FREQ_HOMALT=0.0;FREQ_HOMREF=0.9994179842855757;N_BI_GENOS=10309;South_Han_AN=1496;South_Han_AC=1;South_Han_A
chr7	60968501	.	A	<INS:ME:LINE1>	.	PASS	SVTYPE=INS:ME:LINE1;END=60968501;SVLEN=0;AN=20618;AC=1;AF=4.850130953535746e-05;N_HET=1;N_HOMALT=0;N_HOMREF=10308;FREQ_HET=9.700261907071492e-05;FREQ_HOMALT=0.0;FREQ_HOMREF=0.9999029973809292;N_BI_GENOS=10309;North_Han_AN=2272;North_Han_AC=1;North_Han_AF=
chr7	60968615	.	G	<INS:ME:LINE1>	.	PASS	SVTYPE=INS:ME:LINE1;END=60968615;SVLEN=0;AN=20618;AC=1;AF=4.850130953535746e-05;N_HET=1;N_HOMALT=0;N_HOMREF=10308;FREQ_HET=9.700261907071492e-05;FREQ_HOMALT=0.0;FREQ_HOMREF=0.9999029973809292;N_BI_GENOS=10309;South_Han_AN=1496;South_Han_AC=1;South_Han_AF=
chr7	60968803	.	A	<INS:ME:LINE1>	.	PASS	SVTYPE=INS:ME:LINE1;END=60968803;SVLEN=0;AN=20618;AC=3;AF=0.00014550392860607236;N_HET=3;N_HOMALT=0;N_HOMREF=10306;FREQ_HET=0.0002910078572121447;FREQ_HOMALT=0.0;FREQ_HOMREF=0.9997089921427879;N_BI_GENOS=10309;Northwest_Han_AN=1296;Northwest_Han_AC=1;Nort
chr7	60982218	.	G	<INS:ME:LINE1>	.	PASS	SVTYPE=INS:ME:LINE1;END=60982233;SVLEN=840;AN=20618;AC=15;AF=0.0007275196430303619;N_HET=15;N_HOMALT=0;N_HOMREF=10294;FREQ_HET=0.0014550392860607237;FREQ_HOMALT=0.0;FREQ_HOMREF=0.9985449607139393;N_BI_GENOS=10309;South_Han_AN=1496;South_Han_AC=2;South_Han
chr7	60996282	.	G	<INS:ME:LINE1>	.	PASS	SVTYPE=INS:ME:LINE1;END=60996282;SVLEN=137;AN=20618;AC=1;AF=4.850130953535746e-05;N_HET=1;N_HOMALT=0;N_HOMREF=10308;FREQ_HET=9.700261907071492e-05;FREQ_HOMALT=0.0;FREQ_HOMREF=0.9999029973809292;N_BI_GENOS=10309;Central_Han_AN=2128;Central_Han_AC=1;Central
chr7	61061391	.	A	<INS:ME:LINE1>	.	PASS	SVTYPE=INS:ME:LINE1;END=61061391;SVLEN=5908;AN=20618;AC=1;AF=4.850130953535746e-05;N_HET=1;N_HOMALT=0;N_HOMREF=10308;FREQ_HET=9.700261907071492e-05;FREQ_HOMALT=0.0;FREQ_HOMREF=0.9999029973809292;N_BI_GENOS=10309;Northwest_Han_AN=1296;Northwest_Han_AC=1;No
chr7	61096461	.	A	<INS:ME:LINE1>	.	PASS	SVTYPE=INS:ME:LINE1;END=61096469;SVLEN=1397;AN=20618;AC=1;AF=4.850130953535746e-05;N_HET=1;N_HOMALT=0;N_HOMREF=10308;FREQ_HET=9.700261907071492e-05;FREQ_HOMALT=0.0;FREQ_HOMREF=0.9999029973809292;N_BI_GENOS=10309;South_Han_AN=1496;South_Han_AC=1;South_Han_
chr7	61922167	.	G	<INS:ME:LINE1>	.	PASS	SVTYPE=INS:ME:LINE1;END=61922181;SVLEN=867;AN=20618;AC=4;AF=0.00019400523814142983;N_HET=4;N_HOMALT=0;N_HOMREF=10305;FREQ_HET=0.0003880104762828966;FREQ_HOMALT=0.0;FREQ_HOMREF=0.9996119895237171;N_BI_GENOS=10309;South_Han_AN=1496;South_Han_AC=1;South_Han
chr7	62334056	.	C	<INS:ME:LINE1>	.	PASS	SVTYPE=INS:ME:LINE1;END=62334070;SVLEN=5998;AN=20618;AC=5296;AF=0.2568629352992531;N_HET=3208;N_HOMALT=1044;N_HOMREF=6057;FREQ_HET=0.31118440197885344;FREQ_HOMALT=0.10127073430982636;FREQ_HOMREF=0.5875448637113202;N_BI_GENOS=10309;South_Han_AN=1496;South_Han_
chr7	62334121	.	C	<INS:ME:LINE1>	.	PASS	SVTYPE=INS:ME:LINE1;END=62334121;SVLEN=5998;AN=20618;AC=16;AF=0.0007760209525657193;N_HET=16;N_HOMALT=0;N_HOMREF=10293;FREQ_HET=0.0015520419051314386;FREQ_HOMALT=0.0;FREQ_HOMREF=0.9984479580948685;N_BI_GENOS=10309;South_Han_AN=1496;South_Han_AC=1;South_Ha
chr7	62381696	.	A	<INS:ME:LINE1>	.	PASS	SVTYPE=INS:ME:LINE1;END=62381696;SVLEN=0;AN=20618;AC=1;AF=4.850130953535746e-05;N_HET=1;N_HOMALT=0;N_HOMREF=10308;FREQ_HET=9.700261907071492e-05;FREQ_HOMALT=0.0;FREQ_HOMREF=0.9999029973809292;N_BI_GENOS=10309;East_Han_AN=6554;East_Han_AC=1;East_Han_AF=0.0
chr7	62382957	.	G	<INS:ME:LINE1>	.	PASS	SVTYPE=INS:ME:LINE1;END=62382957;SVLEN=0;AN=20618;AC=2;AF=9.700261907071492e-05;N_HET=2;N_HOMALT=0;N_HOMREF=10307;FREQ_HET=0.00019400523814142983;FREQ_HOMALT=0.0;FREQ_HOMREF=0.9998059947618586;N_BI_GENOS=10309;Lingnan_Han_AN=1888;Lingnan_Han_AC=1;Lingnan_
chr7	62401148	.	A	<INS:ME:LINE1>	.	PASS	SVTYPE=INS:ME:LINE1;END=62401150;SVLEN=2325;AN=20618;AC=1;AF=4.850130953535746e-05;N_HET=1;N_HOMALT=0;N_HOMREF=10308;FREQ_HET=9.700261907071492e-05;FREQ_HOMALT=0.0;FREQ_HOMREF=0.9999029973809292;N_BI_GENOS=10309;Southeast_Han_AN=1908;Southeast_Han_AC=1;So
chr7	62420759	.	A	<INS:ME:LINE1>	.	PASS	SVTYPE=INS:ME:LINE1;END=62420759;SVLEN=0;AN=20618;AC=1;AF=4.850130953535746e-05;N_HET=1;N_HOMALT=0;N_HOMREF=10308;FREQ_HET=9.700261907071492e-05;FREQ_HOMALT=0.0;FREQ_HOMREF=0.9999029973809292;N_BI_GENOS=10309;Lingnan_Han_AN=1888;Lingnan_Han_AC=1;Lingnan_H
chr7	62436984	.	T	<INS:ME:LINE1>	.	PASS	SVTYPE=INS:ME:LINE1;END=62436986;SVLEN=378;AN=20618;AC=1;AF=4.850130953535746e-05;N_HET=1;N_HOMALT=0;N_HOMREF=10308;FREQ_HET=9.700261907071492e-05;FREQ_HOMALT=0.0;FREQ_HOMREF=0.9999029973809292;N_BI_GENOS=10309;Central_Han_AN=2128;Central_Han_AC=1;Central
chr7	62564649	.	A	<INS:ME:LINE1>	.	PASS	SVTYPE=INS:ME:LINE1;END=62564666;SVLEN=217;AN=20618;AC=1;AF=4.850130953535746e-05;N_HET=1;N_HOMALT=0;N_HOMREF=10308;FREQ_HET=9.700261907071492e-05;FREQ_HOMALT=0.0;FREQ_HOMREF=0.9999029973809292;N_BI_GENOS=10309;Southeast_Han_AN=1908;Southeast_Han_AC=1;Sou
chr7	62572533	.	C	<INS:ME:LINE1>	.	PASS	SVTYPE=INS:ME:LINE1;END=62572533;SVLEN=427;AN=20618;AC=1;AF=4.850130953535746e-05;N_HET=1;N_HOMALT=0;N_HOMREF=10308;FREQ_HET=9.700261907071492e-05;FREQ_HOMALT=0.0;FREQ_HOMREF=0.9999029973809292;N_BI_GENOS=10309;Central_Han_AN=2128;Central_Han_AC=1;Central
chr7	62609245	.	T	<INS:ME:LINE1>	.	PASS	SVTYPE=INS:ME:LINE1;END=62609250;SVLEN=303;AN=20618;AC=2;AF=9.700261907071492e-05;N_HET=2;N_HOMALT=0;N_HOMREF=10307;FREQ_HET=0.00019400523814142983;FREQ_HOMALT=0.0;FREQ_HOMREF=0.9998059947618586;N_BI_GENOS=10309;Southeast_Han_AN=1908;Southeast_Han_AC=2;So
chr7	62664168	.	C	<INS:ME:LINE1>	.	PASS	SVTYPE=INS:ME:LINE1;END=62664173;SVLEN=840;AN=20618;AC=1;AF=4.850130953535746e-05;N_HET=1;N_HOMALT=0;N_HOMREF=10308;FREQ_HET=9.700261907071492e-05;FREQ_HOMALT=0.0;FREQ_HOMREF=0.9999029973809292;N_BI_GENOS=10309;Lingnan_Han_AN=1888;Lingnan_Han_AC=1;Lingnan
chr7	62868749	.	C	<INS:ME:LINE1>	.	PASS	SVTYPE=INS:ME:LINE1;END=62868763;SVLEN=378;AN=20618;AC=12;AF=0.0005820157144242894;N_HET=12;N_HOMALT=0;N_HOMREF=10297;FREQ_HET=0.0011640314288485789;FREQ_HOMALT=0.0;FREQ_HOMREF=0.9988359685711514;N_BI_GENOS=10309;South_Han_AN=1496;South_Han_AC=3;South_Han
chr7	62875152	.	A	<INS:ME:LINE1>	.	PASS	SVTYPE=INS:ME:LINE1;END=62875162;SVLEN=1010;AN=20618;AC=1;AF=4.850130953535746e-05;N_HET=1;N_HOMALT=0;N_HOMREF=10308;FREQ_HET=9.700261907071492e-05;FREQ_HOMALT=0.0;FREQ_HOMREF=0.9999029973809292;N_BI_GENOS=10309;South_Han_AN=1496;South_Han_AC=1;South_Han_
chr7	62896365	.	G	<INS:ME:LINE1>	.	PASS	SVTYPE=INS:ME:LINE1;END=62896380;SVLEN=1130;AN=20618;AC=1;AF=4.850130953535746e-05;N_HET=1;N_HOMALT=0;N_HOMREF=10308;FREQ_HET=9.700261907071492e-05;FREQ_HOMALT=0.0;FREQ_HOMREF=0.9999029973809292;N_BI_GENOS=10309;Central_Han_AN=2128;Central_Han_AC=1;Centra
chr7	62912942	.	T	<INS:ME:LINE1>	.	PASS	SVTYPE=INS:ME:LINE1;END=62912960;SVLEN=1956;AN=20618;AC=1;AF=4.850130953535746e-05;N_HET=1;N_HOMALT=0;N_HOMREF=10308;FREQ_HET=9.700261907071492e-05;FREQ_HOMALT=0.0;FREQ_HOMREF=0.9999029973809292;N_BI_GENOS=10309;East_Han_AN=6554;East_Han_AC=1;East_Han_AF=
chr7	63032551	.	A	<INS:ME:LINE1>	.	PASS	SVTYPE=INS:ME:LINE1;END=63032569;SVLEN=1630;AN=20618;AC=5973;AF=0.2896983218546901;N_HET=3997;N_HOMALT=988;N_HOMREF=5324;FREQ_HET=0.3877194684256475;FREQ_HOMALT=0.09583858764186633;FREQ_HOMREF=0.5164419439324862;N_BI_GENOS=10309;South_Han_AN=1496;South_Ha
chr7	63088167	.	A	<INS:ME:LINE1>	.	PASS	SVTYPE=INS:ME:LINE1;END=63088178;SVLEN=2479;AN=20618;AC=2;AF=9.700261907071492e-05;N_HET=2;N_HOMALT=0;N_HOMREF=10307;FREQ_HET=0.00019400523814142983;FREQ_HOMALT=0.0;FREQ_HOMREF=0.9998059947618586;N_BI_GENOS=10309;East_Han_AN=6554;East_Han_AC=1;East_Han_AF
chr7	63146484	.	C	<INS:ME:LINE1>	.	PASS	SVTYPE=INS:ME:LINE1;END=63146485;SVLEN=834;AN=20618;AC=1;AF=4.850130953535746e-05;N_HET=1;N_HOMALT=0;N_HOMREF=10308;FREQ_HET=9.700261907071492e-05;FREQ_HOMALT=0.0;FREQ_HOMREF=0.9999029973809292;N_BI_GENOS=10309;Southeast_Han_AN=1908;Southeast_Han_AC=1;Sou
chr7	65095224	.	T	<INS:ME:LINE1>	.	PASS	SVTYPE=INS:ME:LINE1;END=65095239;SVLEN=5991;AN=20618;AC=1;AF=4.850130953535746e-05;N_HET=1;N_HOMALT=0;N_HOMREF=10308;FREQ_HET=9.700261907071492e-05;FREQ_HOMALT=0.0;FREQ_HOMREF=0.9999029973809292;N_BI_GENOS=10309;North_Han_AN=2272;North_Han_AC=1;North_Han_
chr7	65449727	.	A	<INS:ME:LINE1>	.	PASS	SVTYPE=INS:ME:LINE1;END=65449741;SVLEN=4660;AN=20618;AC=1;AF=4.850130953535746e-05;N_HET=1;N_HOMALT=0;N_HOMREF=10308;FREQ_HET=9.700261907071492e-05;FREQ_HOMALT=0.0;FREQ_HOMREF=0.9999029973809292;N_BI_GENOS=10309;Manchu_AN=416;Manchu_AC=1;Manchu_AF=0.00240
chr7	65611917	.	A	<INS:ME:LINE1>	.	PASS	SVTYPE=INS:ME:LINE1;END=65611921;SVLEN=170;AN=20618;AC=1;AF=4.850130953535746e-05;N_HET=1;N_HOMALT=0;N_HOMREF=10308;FREQ_HET=9.700261907071492e-05;FREQ_HOMALT=0.0;FREQ_HOMREF=0.9999029973809292;N_BI_GENOS=10309;Central_Han_AN=2128;Central_Han_AC=1;Central
chr7	65626114	.	A	<INS:ME:LINE1>	.	PASS	SVTYPE=INS:ME:LINE1;END=65626131;SVLEN=6000;AN=20618;AC=1;AF=4.850130953535746e-05;N_HET=1;N_HOMALT=0;N_HOMREF=10308;FREQ_HET=9.700261907071492e-05;FREQ_HOMALT=0.0;FREQ_HOMREF=0.9999029973809292;N_BI_GENOS=10309;East_Han_AN=6554;East_Han_AC=1;East_Han_AF=
chr7	66115590	.	T	<INS:ME:LINE1>	.	PASS	SVTYPE=INS:ME:LINE1;END=66115605;SVLEN=425;AN=20618;AC=1;AF=4.850130953535746e-05;N_HET=1;N_HOMALT=0;N_HOMREF=10308;FREQ_HET=9.700261907071492e-05;FREQ_HOMALT=0.0;FREQ_HOMREF=0.9999029973809292;N_BI_GENOS=10309;Yi_AN=422;Yi_AC=1;Yi_AF=0.002369668246445498
chr7	66247743	.	A	<INS:ME:LINE1>	.	PASS	SVTYPE=INS:ME:LINE1;END=66247761;SVLEN=184;AN=20618;AC=1;AF=4.850130953535746e-05;N_HET=1;N_HOMALT=0;N_HOMREF=10308;FREQ_HET=9.700261907071492e-05;FREQ_HOMALT=0.0;FREQ_HOMREF=0.9999029973809292;N_BI_GENOS=10309;North_Han_AN=2272;North_Han_AC=1;North_Han_A
chr7	67101992	.	T	<INS:ME:LINE1>	.	PASS	SVTYPE=INS:ME:LINE1;END=67102006;SVLEN=313;AN=20618;AC=2;AF=9.700261907071492e-05;N_HET=2;N_HOMALT=0;N_HOMREF=10307;FREQ_HET=0.00019400523814142983;FREQ_HOMALT=0.0;FREQ_HOMREF=0.9998059947618586;N_BI_GENOS=10309;South_Han_AN=1496;South_Han_AC=1;South_Han_
chr7	67126184	.	A	<INS:ME:LINE1>	.	PASS	SVTYPE=INS:ME:LINE1;END=67126199;SVLEN=5997;AN=20618;AC=416;AF=0.0201765447667087;N_HET=334;N_HOMALT=41;N_HOMREF=9934;FREQ_HET=0.03239887476961878;FREQ_HOMALT=0.003977107381899311;FREQ_HOMREF=0.963624017848482;N_BI_GENOS=10309;South_Han_AN=1496;South_Han_
chr7	67870912	.	G	<INS:ME:LINE1>	.	PASS	SVTYPE=INS:ME:LINE1;END=67870926;SVLEN=974;AN=20618;AC=4;AF=0.00019400523814142983;N_HET=4;N_HOMALT=0;N_HOMREF=10305;FREQ_HET=0.0003880104762828966;FREQ_HOMALT=0.0;FREQ_HOMREF=0.9996119895237171;N_BI_GENOS=10309;South_Han_AN=1496;South_Han_AC=1;South_Han
chr7	68308812	.	A	<INS:ME:LINE1>	.	PASS	SVTYPE=INS:ME:LINE1;END=68308830;SVLEN=765;AN=20618;AC=1;AF=4.850130953535746e-05;N_HET=1;N_HOMALT=0;N_HOMREF=10308;FREQ_HET=9.700261907071492e-05;FREQ_HOMALT=0.0;FREQ_HOMREF=0.9999029973809292;N_BI_GENOS=10309;Lingnan_Han_AN=1888;Lingnan_Han_AC=1;Lingnan
chr7	68337586	.	G	<INS:ME:LINE1>	.	PASS	SVTYPE=INS:ME:LINE1;END=68337602;SVLEN=532;AN=20618;AC=1;AF=4.850130953535746e-05;N_HET=1;N_HOMALT=0;N_HOMREF=10308;FREQ_HET=9.700261907071492e-05;FREQ_HOMALT=0.0;FREQ_HOMREF=0.9999029973809292;N_BI_GENOS=10309;Southeast_Han_AN=1908;Southeast_Han_AC=1;Sou
chr7	68484374	.	T	<INS:ME:LINE1>	.	PASS	SVTYPE=INS:ME:LINE1;END=68484382;SVLEN=5999;AN=20618;AC=1;AF=4.850130953535746e-05;N_HET=1;N_HOMALT=0;N_HOMREF=10308;FREQ_HET=9.700261907071492e-05;FREQ_HOMALT=0.0;FREQ_HOMREF=0.9999029973809292;N_BI_GENOS=10309;North_Han_AN=2272;North_Han_AC=1;North_Han_
chr7	68499001	.	A	<INS:ME:LINE1>	.	PASS	SVTYPE=INS:ME:LINE1;END=68499010;SVLEN=716;AN=20618;AC=2;AF=9.700261907071492e-05;N_HET=2;N_HOMALT=0;N_HOMREF=10307;FREQ_HET=0.00019400523814142983;FREQ_HOMALT=0.0;FREQ_HOMREF=0.9998059947618586;N_BI_GENOS=10309;East_Han_AN=6554;East_Han_AC=2;East_Han_AF=
chr7	68875898	.	T	<INS:ME:LINE1>	.	PASS	SVTYPE=INS:ME:LINE1;END=68875913;SVLEN=729;AN=20618;AC=1;AF=4.850130953535746e-05;N_HET=1;N_HOMALT=0;N_HOMREF=10308;FREQ_HET=9.700261907071492e-05;FREQ_HOMALT=0.0;FREQ_HOMREF=0.9999029973809292;N_BI_GENOS=10309;Tibetan_AN=414;Tibetan_AC=1;Tibetan_AF=0.002
chr7	69614367	.	T	<INS:ME:LINE1>	.	PASS	SVTYPE=INS:ME:LINE1;END=69614367;SVLEN=0;AN=20618;AC=1;AF=4.850130953535746e-05;N_HET=1;N_HOMALT=0;N_HOMREF=10308;FREQ_HET=9.700261907071492e-05;FREQ_HOMALT=0.0;FREQ_HOMREF=0.9999029973809292;N_BI_GENOS=10309;South_Han_AN=1496;South_Han_AC=1;South_Han_AF=
chr7	70013009	.	T	<INS:ME:LINE1>	.	PASS	SVTYPE=INS:ME:LINE1;END=70013023;SVLEN=2041;AN=20618;AC=2;AF=9.700261907071492e-05;N_HET=2;N_HOMALT=0;N_HOMREF=10307;FREQ_HET=0.00019400523814142983;FREQ_HOMALT=0.0;FREQ_HOMREF=0.9998059947618586;N_BI_GENOS=10309;Central_Han_AN=2128;Central_Han_AC=2;Centr
chr7	70060393	.	G	<INS:ME:LINE1>	.	PASS	SVTYPE=INS:ME:LINE1;END=70060404;SVLEN=1457;AN=20618;AC=43;AF=0.0020855563100203706;N_HET=41;N_HOMALT=1;N_HOMREF=10267;FREQ_HET=0.003977107381899311;FREQ_HOMALT=9.700261907071492e-05;FREQ_HOMREF=0.99592588999903;N_BI_GENOS=10309;South_Han_AN=1496;South_Ha
chr7	70152518	.	A	<INS:ME:LINE1>	.	PASS	SVTYPE=INS:ME:LINE1;END=70152534;SVLEN=795;AN=20618;AC=1;AF=4.850130953535746e-05;N_HET=1;N_HOMALT=0;N_HOMREF=10308;FREQ_HET=9.700261907071492e-05;FREQ_HOMALT=0.0;FREQ_HOMREF=0.9999029973809292;N_BI_GENOS=10309;North_Han_AN=2272;North_Han_AC=1;North_Han_A
chr7	70217389	.	A	<INS:ME:LINE1>	.	PASS	SVTYPE=INS:ME:LINE1;END=70217389;SVLEN=3433;AN=20618;AC=1;AF=4.850130953535746e-05;N_HET=1;N_HOMALT=0;N_HOMREF=10308;FREQ_HET=9.700261907071492e-05;FREQ_HOMALT=0.0;FREQ_HOMREF=0.9999029973809292;N_BI_GENOS=10309;Central_Han_AN=2128;Central_Han_AC=1;Centra
chr7	70723689	.	G	<INS:ME:LINE1>	.	PASS	SVTYPE=INS:ME:LINE1;END=70723703;SVLEN=330;AN=20618;AC=13320;AF=0.6460374430109613;N_HET=4386;N_HOMALT=4467;N_HOMREF=1456;FREQ_HET=0.4254534872441556;FREQ_HOMALT=0.4333106993888835;FREQ_HOMREF=0.14123581336696092;N_BI_GENOS=10309;South_Han_AN=1496;South_H
chr7	70724443	.	T	<INS:ME:LINE1>	.	PASS	SVTYPE=INS:ME:LINE1;END=70724463;SVLEN=724;AN=20618;AC=6;AF=0.0002910078572121447;N_HET=6;N_HOMALT=0;N_HOMREF=10303;FREQ_HET=0.0005820157144242894;FREQ_HOMALT=0.0;FREQ_HOMREF=0.9994179842855757;N_BI_GENOS=10309;East_Han_AN=6554;East_Han_AC=1;East_Han_AF=0
chr7	70739018	.	T	<INS:ME:LINE1>	.	PASS	SVTYPE=INS:ME:LINE1;END=70739018;SVLEN=695;AN=20618;AC=1;AF=4.850130953535746e-05;N_HET=1;N_HOMALT=0;N_HOMREF=10308;FREQ_HET=9.700261907071492e-05;FREQ_HOMALT=0.0;FREQ_HOMREF=0.9999029973809292;N_BI_GENOS=10309;Lingnan_Han_AN=1888;Lingnan_Han_AC=1;Lingnan
chr7	71319096	.	T	<INS:ME:LINE1>	.	PASS	SVTYPE=INS:ME:LINE1;END=71319096;SVLEN=0;AN=20618;AC=3;AF=0.00014550392860607236;N_HET=1;N_HOMALT=1;N_HOMREF=10307;FREQ_HET=9.700261907071492e-05;FREQ_HOMALT=9.700261907071492e-05;FREQ_HOMREF=0.9998059947618586;N_BI_GENOS=10309;North_Han_AN=2272;North_Han
chr7	71928256	.	T	<INS:ME:LINE1>	.	PASS	SVTYPE=INS:ME:LINE1;END=71928259;SVLEN=1231;AN=20618;AC=1;AF=4.850130953535746e-05;N_HET=1;N_HOMALT=0;N_HOMREF=10308;FREQ_HET=9.700261907071492e-05;FREQ_HOMALT=0.0;FREQ_HOMREF=0.9999029973809292;N_BI_GENOS=10309;East_Han_AN=6554;East_Han_AC=1;East_Han_AF=
chr7	72000369	.	A	<INS:ME:LINE1>	.	PASS	SVTYPE=INS:ME:LINE1;END=72000385;SVLEN=6000;AN=20618;AC=241;AF=0.011688815598021146;N_HET=227;N_HOMALT=7;N_HOMREF=10075;FREQ_HET=0.022019594529052285;FREQ_HOMALT=0.0006790183334950044;FREQ_HOMREF=0.9773013871374527;N_BI_GENOS=10309;South_Han_AN=1496;South
chr7	72372383	.	A	<INS:ME:LINE1>	.	PASS	SVTYPE=INS:ME:LINE1;END=72372392;SVLEN=1726;AN=20618;AC=13;AF=0.0006305170239596469;N_HET=13;N_HOMALT=0;N_HOMREF=10296;FREQ_HET=0.0012610340479192938;FREQ_HOMALT=0.0;FREQ_HOMREF=0.9987389659520807;N_BI_GENOS=10309;South_Han_AN=1496;South_Han_AC=3;South_Ha
chr7	72485446	.	T	<INS:ME:LINE1>	.	PASS	SVTYPE=INS:ME:LINE1;END=72485459;SVLEN=281;AN=20618;AC=10;AF=0.00048501309535357455;N_HET=10;N_HOMALT=0;N_HOMREF=10299;FREQ_HET=0.0009700261907071491;FREQ_HOMALT=0.0;FREQ_HOMREF=0.9990299738092928;N_BI_GENOS=10309;South_Han_AN=1496;South_Han_AC=3;South_Ha
chr7	72508222	.	G	<INS:ME:LINE1>	.	PASS	SVTYPE=INS:ME:LINE1;END=72508234;SVLEN=6000;AN=20618;AC=2;AF=9.700261907071492e-05;N_HET=2;N_HOMALT=0;N_HOMREF=10307;FREQ_HET=0.00019400523814142983;FREQ_HOMALT=0.0;FREQ_HOMREF=0.9998059947618586;N_BI_GENOS=10309;Lingnan_Han_AN=1888;Lingnan_Han_AC=2;Lingn
chr7	72707765	.	A	<INS:ME:LINE1>	.	PASS	SVTYPE=INS:ME:LINE1;END=72707781;SVLEN=6006;AN=20618;AC=1;AF=4.850130953535746e-05;N_HET=1;N_HOMALT=0;N_HOMREF=10308;FREQ_HET=9.700261907071492e-05;FREQ_HOMALT=0.0;FREQ_HOMREF=0.9999029973809292;N_BI_GENOS=10309;Southeast_Han_AN=1908;Southeast_Han_AC=1;So

chr7	76087922	.	A	<INS:ME:LINE1>	.	PASS	SVTYPE=INS:ME:LINE1;END=76087936;SVLEN=6000;AN=20618;AC=3;AF=0.00014550392860607236;N_HET=3;N_HOMALT=0;N_HOMREF=10306;FREQ_HET=0.0002910078572121447;FREQ_HOMALT=0.0;FREQ_HOMREF=0.9997089921427879;N_BI_GENOS=10309;East_Han_AN=6554;East_Han_AC=1;East_Han_AF
chr7	76856029	.	C	<INS:ME:LINE1>	.	PASS	SVTYPE=INS:ME:LINE1;END=76856047;SVLEN=6003;AN=20618;AC=2;AF=9.700261907071492e-05;N_HET=2;N_HOMALT=0;N_HOMREF=10307;FREQ_HET=0.0001940052381414983;FREQ_HOMALT=0.0;FREQ_HOMREF=0.9998059947618586;N_BI_GENOS=10309;East_Han_AN=6554;East_Han_AC=2;East_Han_AF
chr7	77591606	.	T	<INS:ME:LINE1>	.	PASS	SVTYPE=INS:ME:LINE1;END=77591625;SVLEN=71;AN=20618;AC=1;AF=4.850130953535746e-05;N_HET=1;N_HOMALT=0;N_HOMREF=10308;FREQ_HET=9.700261907071492e-05;FREQ_HOMALT=0.0;FREQ_HOMREF=0.9999029973809292;N_BI_GENOS=10309;North_Han_AN=2272;North_Han_AC=1;North_Han_AF
chr7	78363591	.	T	<INS:ME:LINE1>	.	PASS	SVTYPE=INS:ME:LINE1;END=78363597;SVLEN=558;AN=20618;AC=1;AF=4.850130953535746e-05;N_HET=1;N_HOMALT=0;N_HOMREF=10308;FREQ_HET=9.700261907071492e-05;FREQ_HOMALT=0.0;FREQ_HOMREF=0.9999029973809292;N_BI_GENOS=10309;Southeast_Han_AN=1908;Southeast_Han_AC=1;Sou
chr7	78408309	.	A	<INS:ME:LINE1>	.	PASS	SVTYPE=INS:ME:LINE1;END=78408316;SVLEN=1689;AN=20618;AC=1;AF=4.850130953535746e-05;N_HET=1;N_HOMALT=0;N_HOMREF=10308;FREQ_HET=9.700261907071492e-05;FREQ_HOMALT=0.0;FREQ_HOMREF=0.9999029973809292;N_BI_GENOS=10309;East_Han_AN=6554;East_Han_AC=1;East_Han_AF=
chr7	78485937	.	A	<INS:ME:LINE1>	.	PASS	SVTYPE=INS:ME:LINE1;END=78485953;SVLEN=121;AN=20618;AC=1;AF=4.850130953535746e-05;N_HET=1;N_HOMALT=0;N_HOMREF=10308;FREQ_HET=9.700261907071492e-05;FREQ_HOMALT=0.0;FREQ_HOMREF=0.9999029973809292;N_BI_GENOS=10309;Lingnan_Han_AN=1888;Lingnan_Han_AC=1;Lingnan
chr7	79276570	.	G	<INS:ME:LINE1>	.	PASS	SVTYPE=INS:ME:LINE1;END=79276585;SVLEN=5997;AN=20618;AC=21;AF=0.0010185275002425065;N_HET=21;N_HOMALT=0;N_HOMREF=10288;FREQ_HET=0.002037055000485013;FREQ_HOMALT=0.0;FREQ_HOMREF=0.997962944999515;N_BI_GENOS=10309;South_Han_AN=1496;South_Han_AC=3;South_Han_
chr7	79302163	.	A	<INS:ME:LINE1>	.	PASS	SVTYPE=INS:ME:LINE1;END=79302176;SVLEN=990;AN=20618;AC=1;AF=4.850130953535746e-05;N_HET=1;N_HOMALT=0;N_HOMREF=10308;FREQ_HET=9.700261907071492e-05;FREQ_HOMALT=0.0;FREQ_HOMREF=0.9999029973809292;N_BI_GENOS=10309;Northwest_Han_AN=1296;Northwest_Han_AC=1;Nor
chr7	79433869	.	G	<INS:ME:LINE1>	.	PASS	SVTYPE=INS:ME:LINE1;END=79433885;SVLEN=6058;AN=20618;AC=1;AF=4.850130953535746e-05;N_HET=1;N_HOMALT=0;N_HOMREF=10308;FREQ_HET=9.700261907071492e-05;FREQ_HOMALT=0.0;FREQ_HOMREF=0.9999029973809292;N_BI_GENOS=10309;Northwest_Han_AN=1296;Northwest_Han_AC=1;No
chr7	79689922	.	A	<INS:ME:LINE1>	.	PASS	SVTYPE=INS:ME:LINE1;END=79689922;SVLEN=0;AN=20618;AC=12;AF=0.0005820157144242894;N_HET=10;N_HOMALT=1;N_HOMREF=10298;FREQ_HET=0.0009700261907071491;FREQ_HOMALT=9.700261907071492e-05;FREQ_HOMREF=0.9989329711902222;N_BI_GENOS=10309;Northwest_Han_AN=1296;Nort
chr7	79820351	.	A	<INS:ME:LINE1>	.	PASS	SVTYPE=INS:ME:LINE1;END=79820370;SVLEN=804;AN=20618;AC=1;AF=4.850130953535746e-05;N_HET=1;N_HOMALT=0;N_HOMREF=10308;FREQ_HET=9.700261907071492e-05;FREQ_HOMALT=0.0;FREQ_HOMREF=0.9999029973809292;N_BI_GENOS=10309;Miao_AN=482;Miao_AC=1;Miao_AF=0.002074688796
chr7	79904914	.	G	<INS:ME:LINE1>	.	PASS	SVTYPE=INS:ME:LINE1;END=79904929;SVLEN=5999;AN=20618;AC=1;AF=4.850130953535746e-05;N_HET=1;N_HOMALT=0;N_HOMREF=10308;FREQ_HET=9.700261907071492e-05;FREQ_HOMALT=0.0;FREQ_HOMREF=0.9999029973809292;N_BI_GENOS=10309;East_Han_AN=6554;East_Han_AC=1;East_Han_AF=
chr7	80248653	.	G	<INS:ME:LINE1>	.	PASS	SVTYPE=INS:ME:LINE1;END=80248667;SVLEN=988;AN=20618;AC=20;AF=0.0009700261907071491;N_HET=18;N_HOMALT=1;N_HOMREF=10290;FREQ_HET=0.0017460471432728683;FREQ_HOMALT=9.700261907071492e-05;FREQ_HOMREF=0.9981569502376564;N_BI_GENOS=10309;Northwest_Han_AN=1296;No
chr7	80559113	.	G	<INS:ME:LINE1>	.	PASS	SVTYPE=INS:ME:LINE1;END=80559138;SVLEN=1204;AN=20618;AC=1;AF=4.850130953535746e-05;N_HET=1;N_HOMALT=0;N_HOMREF=10308;FREQ_HET=9.700261907071492e-05;FREQ_HOMALT=0.0;FREQ_HOMREF=0.9999029973809292;N_BI_GENOS=10309;East_Han_AN=6554;East_Han_AC=1;East_Han_AF=
chr7	81164872	.	T	<INS:ME:LINE1>	.	PASS	SVTYPE=INS:ME:LINE1;END=81164875;SVLEN=164;AN=20618;AC=1;AF=4.850130953535746e-05;N_HET=1;N_HOMALT=0;N_HOMREF=10308;FREQ_HET=9.700261907071492e-05;FREQ_HOMALT=0.0;FREQ_HOMREF=0.9999029973809292;N_BI_GENOS=10309;Central_Han_AN=2128;Central_Han_AC=1;Central
chr7	81324627	.	A	<INS:ME:LINE1>	.	PASS	SVTYPE=INS:ME:LINE1;END=81324638;SVLEN=5996;AN=20618;AC=1;AF=4.850130953535746e-05;N_HET=1;N_HOMALT=0;N_HOMREF=10308;FREQ_HET=9.700261907071492e-05;FREQ_HOMALT=0.0;FREQ_HOMREF=0.9999029973809292;N_BI_GENOS=10309;East_Han_AN=6554;East_Han_AC=1;East_Han_AF=
chr7	81363626	.	G	<INS:ME:LINE1>	.	PASS	SVTYPE=INS:ME:LINE1;END=81363626;SVLEN=1189;AN=20618;AC=2;AF=9.700261907071492e-05;N_HET=2;N_HOMALT=0;N_HOMREF=10307;FREQ_HET=0.0001940052381414983;FREQ_HOMALT=0.0;FREQ_HOMREF=0.9998059947618586;N_BI_GENOS=10309;Central_Han_AN=2128;Central_Han_AC=2;Centr
chr7	81610335	.	T	<INS:ME:LINE1>	.	PASS	SVTYPE=INS:ME:LINE1;END=81610339;SVLEN=74;AN=20618;AC=8;AF=0.0003880104762828596;N_HET=8;N_HOMALT=0;N_HOMREF=10301;FREQ_HET=0.0007760209525657193;FREQ_HOMALT=0.0;FREQ_HOMREF=0.9992239790474343;N_BI_GENOS=10309;Northwest_Han_AN=1296;Northwest_Han_AC=4;Nor
chr7	81755301	.	A	<INS:ME:LINE1>	.	PASS	SVTYPE=INS:ME:LINE1;END=81755313;SVLEN=130;AN=20618;AC=14;AF=0.0006790183334950044;N_HET=14;N_HOMALT=0;N_HOMREF=10295;FREQ_HET=0.0013580366669900088;FREQ_HOMALT=0.0;FREQ_HOMREF=0.99864196333301;N_BI_GENOS=10309;Northwest_Han_AN=1296;Northwest_Han_AC=1;Nor
chr7	81874733	.	C	<INS:ME:LINE1>	.	PASS	SVTYPE=INS:ME:LINE1;END=81874740;SVLEN=131;AN=20618;AC=2;AF=9.700261907071492e-05;N_HET=2;N_HOMALT=0;N_HOMREF=10307;FREQ_HET=0.0001940052381414983;FREQ_HOMALT=0.0;FREQ_HOMREF=0.9998059947618586;N_BI_GENOS=10309;Lingnan_Han_AN=1888;Lingnan_Han_AC=1;Lingna
chr7	82030362	.	G	<INS:ME:LINE1>	.	PASS	SVTYPE=INS:ME:LINE1;END=82030375;SVLEN=1615;AN=20618;AC=1;AF=4.850130953535746e-05;N_HET=1;N_HOMALT=0;N_HOMREF=10308;FREQ_HET=9.700261907071492e-05;FREQ_HOMALT=0.0;FREQ_HOMREF=0.9999029973809292;N_BI_GENOS=10309;East_Han_AN=6554;East_Han_AC=1;East_Han_AF=
chr7	82044712	.	G	<INS:ME:LINE1>	.	PASS	SVTYPE=INS:ME:LINE1;END=82044727;SVLEN=6000;AN=20618;AC=2;AF=9.700261907071492e-05;N_HET=2;N_HOMALT=0;N_HOMREF=10307;FREQ_HET=0.0001940052381414983;FREQ_HOMALT=0.0;FREQ_HOMREF=0.9998059947618586;N_BI_GENOS=10309;East_Han_AN=6554;East_Han_AC=2;East_Han_AF
chr7	82169444	.	T	<INS:ME:LINE1>	.	PASS	SVTYPE=INS:ME:LINE1;END=82169464;SVLEN=967;AN=20618;AC=5;AF=0.00024250654767678728;N_HET=5;N_HOMALT=0;N_HOMREF=10304;FREQ_HET=0.00048501309535357455;FREQ_HOMALT=0.0;FREQ_HOMREF=0.9995149869046465;N_BI_GENOS=10309;South_Han_AN=1496;South_Han_AC=2;South_Han
chr7	82300854	.	A	<INS:ME:LINE1>	.	PASS	SVTYPE=INS:ME:LINE1;END=82300869;SVLEN=2246;AN=20618;AC=1;AF=4.850130953535746e-05;N_HET=1;N_HOMALT=0;N_HOMREF=10308;FREQ_HET=9.700261907071492e-05;FREQ_HOMALT=0.0;FREQ_HOMREF=0.9999029973809292;N_BI_GENOS=10309;North_Han_AN=2272;North_Han_AC=1;North_Han_
chr7	82373568	.	A	<INS:ME:LINE1>	.	PASS	SVTYPE=INS:ME:LINE1;END=82373570;SVLEN=106;AN=20618;AC=1;AF=4.850130953535746e-05;N_HET=1;N_HOMALT=0;N_HOMREF=10308;FREQ_HET=9.700261907071492e-05;FREQ_HOMALT=0.0;FREQ_HOMREF=0.9999029973809292;N_BI_GENOS=10309;North_Han_AN=2272;North_Han_AC=1;North_Han_A
chr7	83274138	.	G	<INS:ME:LINE1>	.	PASS	SVTYPE=INS:ME:LINE1;END=83274138;SVLEN=15;AN=20618;AC=1;AF=4.850130953535746e-05;N_HET=1;N_HOMALT=0;N_HOMREF=10308;FREQ_HET=9.700261907071492e-05;FREQ_HOMALT=0.0;FREQ_HOMREF=0.9999029973809292;N_BI_GENOS=10309;East_Han_AN=6554;East_Han_AC=1;East_Han_AF=0.
chr7	83460626	.	G	<INS:ME:LINE1>	.	PASS	SVTYPE=INS:ME:LINE1;END=83460626;SVLEN=0;AN=20618;AC=17;AF=0.0008245222621010767;N_HET=1;N_HOMALT=8;N_HOMREF=10300;FREQ_HET=9.700261907071492e-05;FREQ_HOMALT=0.0007760209525657193;FREQ_HOMREF=0.9991269764283636;N_BI_GENOS=10309;Lingnan_Han_AN=1888;Lingnan
chr7	84068624	.	T	<INS:ME:LINE1>	.	PASS	SVTYPE=INS:ME:LINE1;END=84068624;SVLEN=1994;AN=20618;AC=3;AF=0.00014550392860607236;N_HET=3;N_HOMALT=0;N_HOMREF=10306;FREQ_HET=0.0002910078572121447;FREQ_HOMALT=0.0;FREQ_HOMREF=0.9997089921427879;N_BI_GENOS=10309;South_Han_AN=1496;South_Han_AC=1;South_Han
chr7	84333448	.	A	<INS:ME:LINE1>	.	PASS	SVTYPE=INS:ME:LINE1;END=84333462;SVLEN=705;AN=20618;AC=1;AF=4.850130953535746e-05;N_HET=1;N_HOMALT=0;N_HOMREF=10308;FREQ_HET=9.700261907071492e-05;FREQ_HOMALT=0.0;FREQ_HOMREF=0.9999029973809292;N_BI_GENOS=10309;North_Han_AN=2272;North_Han_AC=1;North_Han_A
chr7	84604741	.	A	<INS:ME:LINE1>	.	PASS	SVTYPE=INS:ME:LINE1;END=84604756;SVLEN=1223;AN=20618;AC=35;AF=0.0016975458337375108;N_HET=29;N_HOMALT=3;N_HOMREF=10277;FREQ_HET=0.0028130759530507325;FREQ_HOMALT=0.0002910078572121447;FREQ_HOMREF=0.9968959161897377;N_BI_GENOS=10309;South_Han_AN=1496;South
chr7	85272407	.	T	<INS:ME:LINE1>	.	PASS	SVTYPE=INS:ME:LINE1;END=85272409;SVLEN=208;AN=20618;AC=5356;AF=0.2597730138713745;N_HET=2182;N_HOMALT=1587;N_HOMREF=6540;FREQ_HET=0.21165971481229992;FREQ_HOMALT=0.15394315646522455;FREQ_HOMREF=0.6343971287224756;N_BI_GENOS=10309;South_Han_AN=1496;South_H
chr7	85281807	.	A	<INS:ME:LINE1>	.	PASS	SVTYPE=INS:ME:LINE1;END=85281821;SVLEN=6000;AN=20618;AC=1;AF=4.850130953535746e-05;N_HET=1;N_HOMALT=0;N_HOMREF=10308;FREQ_HET=9.700261907071492e-05;FREQ_HOMALT=0.0;FREQ_HOMREF=0.9999029973809292;N_BI_GENOS=10309;Northwest_Han_AN=1296;Northwest_Han_AC=1;No
chr7	85384486	.	A	<INS:ME:LINE1>	.	PASS	SVTYPE=INS:ME:LINE1;END=85384486;SVLEN=8;AN=20618;AC=1;AF=4.850130953535746e-05;N_HET=1;N_HOMALT=0;N_HOMREF=10308;FREQ_HET=9.700261907071492e-05;FREQ_HOMALT=0.0;FREQ_HOMREF=0.9999029973809292;N_BI_GENOS=10309;Southeast_Han_AN=1908;Southeast_Han_AC=1;South
chr7	85415420	.	A	<INS:ME:LINE1>	.	PASS	SVTYPE=INS:ME:LINE1;END=85415435;SVLEN=1666;AN=20618;AC=2899;AF=0.14060529634300126;N_HET=2099;N_HOMALT=400;N_HOMREF=7810;FREQ_HET=0.2036084974294306;FREQ_HOMALT=0.03880104762828596;FREQ_HOMREF=0.7575904549422834;N_BI_GENOS=10309;South_Han_AN=1496;South_H
chr7	86399002	.	T	<INS:ME:LINE1>	.	PASS	SVTYPE=INS:ME:LINE1;END=86399017;SVLEN=511;AN=20618;AC=1;AF=4.850130953535746e-05;N_HET=1;N_HOMALT=0;N_HOMREF=10308;FREQ_HET=9.700261907071492e-05;FREQ_HOMALT=0.0;FREQ_HOMREF=0.9999029973809292;N_BI_GENOS=10309;East_Han_AN=6554;East_Han_AC=1;East_Han_AF=0
chr7	86655539	.	A	<INS:ME:LINE1>	.	PASS	SVTYPE=INS:ME:LINE1;END=86655557;SVLEN=58;AN=20618;AC=1;AF=4.850130953535746e-05;N_HET=1;N_HOMALT=0;N_HOMREF=10308;FREQ_HET=9.700261907071492e-05;FREQ_HOMALT=0.0;FREQ_HOMREF=0.9999029973809292;N_BI_GENOS=10309;North_Han_AN=2272;North_Han_AC=1;North_Han_AF
chr7	87011914	.	A	<INS:ME:LINE1>	.	PASS	SVTYPE=INS:ME:LINE1;END=87011922;SVLEN=2109;AN=20618;AC=1;AF=4.850130953535746e-05;N_HET=1;N_HOMALT=0;N_HOMREF=10308;FREQ_HET=9.700261907071492e-05;FREQ_HOMALT=0.0;FREQ_HOMREF=0.9999029973809292;N_BI_GENOS=10309;North_Han_AN=2272;North_Han_AC=1;North_Han_
chr7	87085032	.	T	<INS:ME:LINE1>	.	PASS	SVTYPE=INS:ME:LINE1;END=87085047;SVLEN=6017;AN=20618;AC=1;AF=4.850130953535746e-05;N_HET=1;N_HOMALT=0;N_HOMREF=10308;FREQ_HET=9.700261907071492e-05;FREQ_HOMALT=0.0;FREQ_HOMREF=0.9999029973809292;N_BI_GENOS=10309;Central_Han_AN=2128;Central_Han_AC=1;Centra
chr7	87642116	.	A	<INS:ME:LINE1>	.	PASS	SVTYPE=INS:ME:LINE1;END=87642129;SVLEN=1108;AN=20618;AC=1;AF=4.850130953535746e-05;N_HET=1;N_HOMALT=0;N_HOMREF=10308;FREQ_HET=9.700261907071492e-05;FREQ_HOMALT=0.0;FREQ_HOMREF=0.9999029973809292;N_BI_GENOS=10309;Tibetan_AN=414;Tibetan_AC=1;Tibetan_AF=0.00
chr7	87848651	.	T	<INS:ME:LINE1>	.	PASS	SVTYPE=INS:ME:LINE1;END=87848655;SVLEN=774;AN=20618;AC=1;AF=4.850130953535746e-05;N_HET=1;N_HOMALT=0;N_HOMREF=10308;FREQ_HET=9.700261907071492e-05;FREQ_HOMALT=0.0;FREQ_HOMREF=0.9999029973809292;N_BI_GENOS=10309;Central_Han_AN=2128;Central_Han_AC=1;Central
chr7	88097449	.	A	<INS:ME:LINE1>	.	PASS	SVTYPE=INS:ME:LINE1;END=88097475;SVLEN=891;AN=20618;AC=1;AF=4.850130953535746e-05;N_HET=1;N_HOMALT=0;N_HOMREF=10308;FREQ_HET=9.700261907071492e-05;FREQ_HOMALT=0.0;FREQ_HOMREF=0.9999029973809292;N_BI_GENOS=10309;East_Han_AN=6554;East_Han_AC=1;East_Han_AF=0
chr7	88139470	.	G	<INS:ME:LINE1>	.	PASS	SVTYPE=INS:ME:LINE1;END=88139485;SVLEN=6000;AN=20618;AC=3;AF=0.00014550392860607236;N_HET=3;N_HOMALT=0;N_HOMREF=10306;FREQ_HET=0.0002910078572121447;FREQ_HOMALT=0.0;FREQ_HOMREF=0.9997089921427879;N_BI_GENOS=10309;Lingnan_Han_AN=1888;Lingnan_Han_AC=1;Lingn
chr7	88478762	.	C	<INS:ME:LINE1>	.	PASS	SVTYPE=INS:ME:LINE1;END=88478772;SVLEN=1321;AN=20618;AC=1;AF=4.850130953535746e-05;N_HET=1;N_HOMALT=0;N_HOMREF=10308;FREQ_HET=9.700261907071492e-05;FREQ_HOMALT=0.0;FREQ_HOMREF=0.9999029973809292;N_BI_GENOS=10309;Southeast_Han_AN=1908;Southeast_Han_AC=1;So
chr7	88479502	.	A	<INS:ME:LINE1>	.	PASS	SVTYPE=INS:ME:LINE1;END=88479523;SVLEN=817;AN=20618;AC=2;AF=9.700261907071492e-05;N_HET=2;N_HOMALT=0;N_HOMREF=10307;FREQ_HET=0.0001940052381414983;FREQ_HOMALT=0.0;FREQ_HOMREF=0.9998059947618586;N_BI_GENOS=10309;North_Han_AN=2272;North_Han_AC=2;North_Han_
chr7	88698566	.	A	<INS:ME:LINE1>	.	PASS	SVTYPE=INS:ME:LINE1;END=88698578;SVLEN=447;AN=20618;AC=16;AF=0.0007760209525657193;N_HET=14;N_HOMALT=1;N_HOMREF=10294;FREQ_HET=0.0013580366669900088;FREQ_HOMALT=9.700261907071492e-05;FREQ_HOMREF=0.9985449607139393;N_BI_GENOS=10309;South_Han_AN=1496;South_
chr7	88714903	.	T	<INS:ME:LINE1>	.	PASS	SVTYPE=INS:ME:LINE1;END=88714918;SVLEN=303;AN=20618;AC=1;AF=4.850130953535746e-05;N_HET=1;N_HOMALT=0;N_HOMREF=10308;FREQ_HET=9.700261907071492e-05;FREQ_HOMALT=0.0;FREQ_HOMREF=0.9999029973809292;N_BI_GENOS=10309;East_Han_AN=6554;East_Han_AC=1;East_Han_AF=0
chr7	88936467	.	T	<INS:ME:LINE1>	.	PASS	SVTYPE=INS:ME:LINE1;END=88936481;SVLEN=409;AN=20618;AC=2;AF=9.700261907071492e-05;N_HET=2;N_HOMALT=0;N_HOMREF=10307;FREQ_HET=0.0001940052381414983;FREQ_HOMALT=0.0;FREQ_HOMREF=0.9998059947618586;N_BI_GENOS=10309;Northwest_Han_AN=1296;Northwest_Han_AC=1;No
chr7	89102847	.	A	<INS:ME:LINE1>	.	PASS	SVTYPE=INS:ME:LINE1;END=89102847;SVLEN=224;AN=20618;AC=1;AF=4.850130953535746e-05;N_HET=1;N_HOMALT=0;N_HOMREF=10308;FREQ_HET=9.700261907071492e-05;FREQ_HOMALT=0.0;FREQ_HOMREF=0.9999029973809292;N_BI_GENOS=10309;East_Han_AN=6554;East_Han_AC=1;East_Han_AF=0
chr7	89226462	.	A	<INS:ME:LINE1>	.	PASS	SVTYPE=INS:ME:LINE1;END=89226476;SVLEN=1215;AN=20618;AC=1;AF=4.850130953535746e-05;N_HET=1;N_HOMALT=0;N_HOMREF=10308;FREQ_HET=9.700261907071492e-05;FREQ_HOMALT=0.0;FREQ_HOMREF=0.9999029973809292;N_BI_GENOS=10309;East_Han_AN=6554;East_Han_AC=1;East_Han_AF=
chr7	89550753	.	A	<INS:ME:LINE1>	.	PASS	SVTYPE=INS:ME:LINE1;END=89550753;SVLEN=1694;AN=20618;AC=2;AF=9.700261907071492e-05;N_HET=2;N_HOMALT=0;N_HOMREF=10307;FREQ_HET=0.0001940052381414983;FREQ_HOMALT=0.0;FREQ_HOMREF=0.9998059947618586;N_BI_GENOS=10309;East_Han_AN=6554;East_Han_AC=1;East_Han_AF
chr7	89775934	.	A	<INS:ME:LINE1>	.	PASS	SVTYPE=INS:ME:LINE1;END=89775940;SVLEN=1072;AN=20618;AC=1;AF=4.850130953535746e-05;N_HET=1;N_HOMALT=0;N_HOMREF=10308;FREQ_HET=9.700261907071492e-05;FREQ_HOMALT=0.0;FREQ_HOMREF=0.9999029973809292;N_BI_GENOS=10309;East_Han_AN=6554;East_Han_AC=1;East_Han_AF=
chr7	89801820	.	G	<INS:ME:LINE1>	.	PASS	SVTYPE=INS:ME:LINE1;END=89801820;SVLEN=72;AN=20618;AC=1;AF=4.850130953535746e-05;N_HET=1;N_HOMALT=0;N_HOMREF=10308;FREQ_HET=9.700261907071492e-05;FREQ_HOMALT=0.0;FREQ_HOMREF=0.9999029973809292;N_BI_GENOS=10309;Lingnan_Han_AN=1888;Lingnan_Han_AC=1;Lingnan_
chr7	89890593	.	T	<INS:ME:LINE1>	.	PASS	SVTYPE=INS:ME:LINE1;END=89890593;SVLEN=4451;AN=20618;AC=1;AF=4.850130953535746e-05;N_HET=1;N_HOMALT=0;N_HOMREF=10308;FREQ_HET=9.700261907071492e-05;FREQ_HOMALT=0.0;FREQ_HOMREF=0.9999029973809292;N_BI_GENOS=10309;North_Han_AN=2272;North_Han_AC=1;North_Han_
chr7	89932768	.	A	<INS:ME:LINE1>	.	PASS	SVTYPE=INS:ME:LINE1;END=89932777;SVLEN=293;AN=20618;AC=3;AF=0.00014550392860607236;N_HET=3;N_HOMALT=0;N_HOMREF=10306;FREQ_HET=0.0002910078572121447;FREQ_HOMALT=0.0;FREQ_HOMREF=0.9997089921427879;N_BI_GENOS=10309;Lingnan_Han_AN=1888;Lingnan_Han_AC=1;Lingna
chr7	90046339	.	G	<INS:ME:LINE1>	.	PASS	SVTYPE=INS:ME:LINE1;END=90046339;SVLEN=2494;AN=20618;AC=1;AF=4.850130953535746e-05;N_HET=1;N_HOMALT=0;N_HOMREF=10308;FREQ_HET=9.700261907071492e-05;FREQ_HOMALT=0.0;FREQ_HOMREF=0.9999029973809292;N_BI_GENOS=10309;East_Han_AN=6554;East_Han_AC=1;East_Han_AF=
chr7	90281353	.	A	<INS:ME:LINE1>	.	PASS	SVTYPE=INS:ME:LINE1;END=90281364;SVLEN=1042;AN=20618;AC=1;AF=4.850130953535746e-05;N_HET=1;N_HOMALT=0;N_HOMREF=10308;FREQ_HET=9.700261907071492e-05;FREQ_HOMALT=0.0;FREQ_HOMREF=0.9999029973809292;N_BI_GENOS=10309;Southeast_Han_AN=1908;Southeast_Han_AC=1;So
chr7	90315095	.	A	<INS:ME:LINE1>	.	PASS	SVTYPE=INS:ME:LINE1;END=90315110;SVLEN=5998;AN=20618;AC=409;AF=0.019837035599961198;N_HET=289;N_HOMALT=60;N_HOMREF=9960;FREQ_HET=0.02803375691143661;FREQ_HOMALT=0.005820157144242895;FREQ_HOMREF=0.9661460859443205;N_BI_GENOS=10309;South_Han_AN=1496;South_H
chr7	90336591	.	T	<INS:ME:LINE1>	.	PASS	SVTYPE=INS:ME:LINE1;END=90336602;SVLEN=6016;AN=20618;AC=1;AF=4.850130953535746e-05;N_HET=1;N_HOMALT=0;N_HOMREF=10308;FREQ_HET=9.700261907071492e-05;FREQ_HOMALT=0.0;FREQ_HOMREF=0.9999029973809292;N_BI_GENOS=10309;Central_Han_AN=2128;Central_Han_AC=1;Centra
chr7	90393140	.	A	<INS:ME:LINE1>	.	PASS	SVTYPE=INS:ME:LINE1;END=90393144;SVLEN=1078;AN=20618;AC=2;AF=9.700261907071492e-05;N_HET=2;N_HOMALT=0;N_HOMREF=10307;FREQ_HET=0.0001940052381414983;FREQ_HOMALT=0.0;FREQ_HOMREF=0.9998059947618586;N_BI_GENOS=10309;Central_Han_AN=2128;Central_Han_AC=1;Centr
chr7	90702592	.	T	<INS:ME:LINE1>	.	PASS	SVTYPE=INS:ME:LINE1;END=90702607;SVLEN=5998;AN=20618;AC=2590;AF=0.12561839169657582;N_HET=2010;N_HOMALT=290;N_HOMREF=8009;FREQ_HET=0.19497526433213697;FREQ_HOMALT=0.028130759530507324;FREQ_HOMREF=0.7768939761373557;N_BI_GENOS=10309;South_Han_AN=1496;South
chr7	90716449	.	A	<INS:ME:LINE1>	.	PASS	SVTYPE=INS:ME:LINE1;END=90716464;SVLEN=5999;AN=20618;AC=1;AF=4.850130953535746e-05;N_HET=1;N_HOMALT=0;N_HOMREF=10308;FREQ_HET=9.700261907071492e-05;FREQ_HOMALT=0.0;FREQ_HOMREF=0.9999029973809292;N_BI_GENOS=10309;North_Han_AN=2272;North_Han_AC=1;North_Han_
chr7	90836356	.	T	<INS:ME:LINE1>	.	PASS	SVTYPE=INS:ME:LINE1;END=90836372;SVLEN=1752;AN=20618;AC=1;AF=4.850130953535746e-05;N_HET=1;N_HOMALT=0;N_HOMREF=10308;FREQ_HET=9.700261907071492e-05;FREQ_HOMALT=0.0;FREQ_HOMREF=0.9999029973809292;N_BI_GENOS=10309;East_Han_AN=6554;East_Han_AC=1;East_Han_AF=
chr7	91141228	.	A	<INS:ME:LINE1>	.	PASS	SVTYPE=INS:ME:LINE1;END=91141245;SVLEN=1336;AN=20618;AC=3;AF=0.00014550392860607236;N_HET=3;N_HOMALT=0;N_HOMREF=10306;FREQ_HET=0.0002910078572121447;FREQ_HOMALT=0.0;FREQ_HOMREF=0.9997089921427879;N_BI_GENOS=10309;Tibetan_AN=414;Tibetan_AC=3;Tibetan_AF=0.0
chr7	91167519	.	C	<INS:ME:LINE1>	.	PASS	SVTYPE=INS:ME:LINE1;END=91167519;SVLEN=76;AN=20618;AC=1;AF=4.850130953535746e-05;N_HET=1;N_HOMALT=0;N_HOMREF=10308;FREQ_HET=9.700261907071492e-05;FREQ_HOMALT=0.0;FREQ_HOMREF=0.9999029973809292;N_BI_GENOS=10309;Southeast_Han_AN=1908;Southeast_Han_AC=1;Sout
chr7	91813296	.	A	<INS:ME:LINE1>	.	PASS	SVTYPE=INS:ME:LINE1;END=91813308;SVLEN=6068;AN=20618;AC=1;AF=4.850130953535746e-05;N_HET=1;N_HOMALT=0;N_HOMREF=10308;FREQ_HET=9.700261907071492e-05;FREQ_HOMALT=0.0;FREQ_HOMREF=0.9999029973809292;N_BI_GENOS=10309;South_Han_AN=1496;South_Han_AC=1;South_Han_
chr7	91865776	.	C	<INS:ME:LINE1>	.	PASS	SVTYPE=INS:ME:LINE1;END=91865776;SVLEN=188;AN=20618;AC=1;AF=4.850130953535746e-05;N_HET=1;N_HOMALT=0;N_HOMREF=10308;FREQ_HET=9.700261907071492e-05;FREQ_HOMALT=0.0;FREQ_HOMREF=0.9999029973809292;N_BI_GENOS=10309;Central_Han_AN=2128;Central_Han_AC=1;Central
chr7	91938825	.	C	<INS:ME:LINE1>	.	PASS	SVTYPE=INS:ME:LINE1;END=91938841;SVLEN=6073;AN=20618;AC=1;AF=4.850130953535746e-05;N_HET=1;N_HOMALT=0;N_HOMREF=10308;FREQ_HET=9.700261907071492e-05;FREQ_HOMALT=0.0;FREQ_HOMREF=0.9999029973809292;N_BI_GENOS=10309;Central_Han_AN=2128;Central_Han_AC=1;Centra
chr7	92705990	.	T	<INS:ME:LINE1>	.	PASS	SVTYPE=INS:ME:LINE1;END=92706005;SVLEN=6004;AN=20618;AC=1;AF=4.850130953535746e-05;N_HET=1;N_HOMALT=0;N_HOMREF=10308;FREQ_HET=9.700261907071492e-05;FREQ_HOMALT=0.0;FREQ_HOMREF=0.9999029973809292;N_BI_GENOS=10309;East_Han_AN=6554;East_Han_AC=1;East_Han_AF=
chr7	92847429	.	C	<INS:ME:LINE1>	.	PASS	SVTYPE=INS:ME:LINE1;END=92847440;SVLEN=6014;AN=20618;AC=1;AF=4.850130953535746e-05;N_HET=1;N_HOMALT=0;N_HOMREF=10308;FREQ_HET=9.700261907071492e-05;FREQ_HOMALT=0.0;FREQ_HOMREF=0.9999029973809292;N_BI_GENOS=10309;East_Han_AN=6554;East_Han_AC=1;East_Han_AF=
chr7	93115508	.	A	<INS:ME:LINE1>	.	PASS	SVTYPE=INS:ME:LINE1;END=93115524;SVLEN=6000;AN=20618;AC=1;AF=4.850130953535746e-05;N_HET=1;N_HOMALT=0;N_HOMREF=10308;FREQ_HET=9.700261907071492e-05;FREQ_HOMALT=0.0;FREQ_HOMREF=0.9999029973809292;N_BI_GENOS=10309;East_Han_AN=6554;East_Han_AC=1;East_Han_AF=
chr7	93193767	.	A	<INS:ME:LINE1>	.	PASS	SVTYPE=INS:ME:LINE1;END=93193767;SVLEN=0;AN=20618;AC=1;AF=4.850130953535746e-05;N_HET=1;N_HOMALT=0;N_HOMREF=10308;FREQ_HET=9.700261907071492e-05;FREQ_HOMALT=0.0;FREQ_HOMREF=0.9999029973809292;N_BI_GENOS=10309;Southeast_Han_AN=1908;Southeast_Han_AC=1;South
chr7	93265036	.	A	<INS:ME:LINE1>	.	PASS	SVTYPE=INS:ME:LINE1;END=93265039;SVLEN=1050;AN=20618;AC=2;AF=9.700261907071492e-05;N_HET=2;N_HOMALT=0;N_HOMREF=10307;FREQ_HET=0.0001940052381414983;FREQ_HOMALT=0.0;FREQ_HOMREF=0.9998059947618586;N_BI_GENOS=10309;Lingnan_Han_AN=1888;Lingnan_Han_AC=1;Lingn
chr7	93414961	.	A	<INS:ME:LINE1>	.	PASS	SVTYPE=INS:ME:LINE1;END=93414980;SVLEN=306;AN=20618;AC=1;AF=4.850130953535746e-05;N_HET=1;N_HOMALT=0;N_HOMREF=10308;FREQ_HET=9.700261907071492e-05;FREQ_HOMALT=0.0;FREQ_HOMREF=0.9999029973809292;N_BI_GENOS=10309;Southeast_Han_AN=1908;Southeast_Han_AC=1;Sou
chr7	93517298	.	G	<INS:ME:LINE1>	.	PASS	SVTYPE=INS:ME:LINE1;END=93517315;SVLEN=105;AN=20618;AC=5603;AF=0.2717528373266078;N_HET=3723;N_HOMALT=940;N_HOMREF=5646;FREQ_HET=0.3611407508002716;FREQ_HOMALT=0.09118246192647202;FREQ_HOMREF=0.5476767872732564;N_BI_GENOS=10309;South_Han_AN=1496;South_Han
chr7	93517527	.	A	<INS:ME:LINE1>	.	PASS	SVTYPE=INS:ME:LINE1;END=93517544;SVLEN=425;AN=20618;AC=2;AF=9.700261907071492e-05;N_HET=2;N_HOMALT=0;N_HOMREF=10307;FREQ_HET=0.0001940052381414983;FREQ_HOMALT=0.0;FREQ_HOMREF=0.9998059947618586;N_BI_GENOS=10309;Manchu_AN=416;Manchu_AC=2;Manchu_AF=0.00480
chr7	93636361	.	G	<INS:ME:LINE1>	.	PASS	SVTYPE=INS:ME:LINE1;END=93636375;SVLEN=6036;AN=20618;AC=1;AF=4.850130953535746e-05;N_HET=1;N_HOMALT=0;N_HOMREF=10308;FREQ_HET=9.700261907071492e-05;FREQ_HOMALT=0.0;FREQ_HOMREF=0.9999029973809292;N_BI_GENOS=10309;Hui_AN=444;Hui_AC=1;Hui_AF=0.00225225225225
chr7	93711872	.	A	<INS:ME:LINE1>	.	PASS	SVTYPE=INS:ME:LINE1;END=93711874;SVLEN=498;AN=20618;AC=6;AF=0.0002910078572121447;N_HET=6;N_HOMALT=0;N_HOMREF=10303;FREQ_HET=0.0005820157144242894;FREQ_HOMALT=0.0;FREQ_HOMREF=0.9994179842855757;N_BI_GENOS=10309;Northwest_Han_AN=1296;Northwest_Han_AC=1;Nor
chr7	94017307	.	A	<INS:ME:LINE1>	.	PASS	SVTYPE=INS:ME:LINE1;END=94017321;SVLEN=1951;AN=20618;AC=3;AF=0.00014550392860607236;N_HET=3;N_HOMALT=0;N_HOMREF=10306;FREQ_HET=0.0002910078572121447;FREQ_HOMALT=0.0;FREQ_HOMREF=0.9997089921427879;N_BI_GENOS=10309;Yi_AN=422;Yi_AC=3;Yi_AF=0.0071090047393364
chr7	94923736	.	G	<INS:ME:LINE1>	.	PASS	SVTYPE=INS:ME:LINE1;END=94923748;SVLEN=5391;AN=20618;AC=1;AF=4.850130953535746e-05;N_HET=1;N_HOMALT=0;N_HOMREF=10308;FREQ_HET=9.700261907071492e-05;FREQ_HOMALT=0.0;FREQ_HOMREF=0.9999029973809292;N_BI_GENOS=10309;South_Han_AN=1496;South_Han_AC=1;South_Han_
chr7	95037300	.	T	<INS:ME:LINE1>	.	PASS	SVTYPE=INS:ME:LINE1;END=95037300;SVLEN=478;AN=20618;AC=1;AF=4.850130953535746e-05;N_HET=1;N_HOMALT=0;N_HOMREF=10308;FREQ_HET=9.700261907071492e-05;FREQ_HOMALT=0.0;FREQ_HOMREF=0.9999029973809292;N_BI_GENOS=10309;Lingnan_Han_AN=1888;Lingnan_Han_AC=1;Lingnan
chr7	95301083	.	A	<INS:ME:LINE1>	.	PASS	SVTYPE=INS:ME:LINE1;END=95301083;SVLEN=113;AN=20618;AC=1;AF=4.850130953535746e-05;N_HET=1;N_HOMALT=0;N_HOMREF=10308;FREQ_HET=9.700261907071492e-05;FREQ_HOMALT=0.0;FREQ_HOMREF=0.9999029973809292;N_BI_GENOS=10309;East_Han_AN=6554;East_Han_AC=1;East_Han_AF=0
chr7	95402101	.	A	<INS:ME:LINE1>	.	PASS	SVTYPE=INS:ME:LINE1;END=95402115;SVLEN=3087;AN=20618;AC=2;AF=9.700261907071492e-05;N_HET=2;N_HOMALT=0;N_HOMREF=10307;FREQ_HET=0.0001940052381414983;FREQ_HOMALT=0.0;FREQ_HOMREF=0.9998059947618586;N_BI_GENOS=10309;Yi_AN=422;Yi_AC=2;Yi_AF=0.0047393364928909
chr7	95610051	.	A	<INS:ME:LINE1>	.	PASS	SVTYPE=INS:ME:LINE1;END=95610067;SVLEN=5998;AN=20618;AC=1;AF=4.850130953535746e-05;N_HET=1;N_HOMALT=0;N_HOMREF=10308;FREQ_HET=9.700261907071492e-05;FREQ_HOMALT=0.0;FREQ_HOMREF=0.9999029973809292;N_BI_GENOS=10309;South_Han_AN=1496;South_Han_AC=1;South_Han_AF=
chr7	95676766	.	A	<INS:ME:LINE1>	.	PASS	SVTYPE=INS:ME:LINE1;END=95676781;SVLEN=6012;AN=20618;AC=1;AF=4.850130953535746e-05;N_HET=1;N_HOMALT=0;N_HOMREF=10308;FREQ_HET=9.700261907071492e-05;FREQ_HOMALT=0.0;FREQ_HOMREF=0.9999029973809292;N_BI_GENOS=10309;Hui_AN=444;Hui_AC=1;Hui_AF=0.00225225225225
chr7	95702090	.	C	<INS:ME:LINE1>	.	PASS	SVTYPE=INS:ME:LINE1;END=95702090;SVLEN=1172;AN=20618;AC=1;AF=4.850130953535746e-05;N_HET=1;N_HOMALT=0;N_HOMREF=10308;FREQ_HET=9.700261907071492e-05;FREQ_HOMALT=0.0;FREQ_HOMREF=0.9999029973809292;N_BI_GENOS=10309;East_Han_AN=6554;East_Han_AC=1;East_Han_AF=
chr7	95769668	.	T	<INS:ME:LINE1>	.	PASS	SVTYPE=INS:ME:LINE1;END=95769681;SVLEN=6031;AN=20618;AC=1;AF=4.850130953535746e-05;N_HET=1;N_HOMALT=0;N_HOMREF=10308;FREQ_HET=9.700261907071492e-05;FREQ_HOMALT=0.0;FREQ_HOMREF=0.9999029973809292;N_BI_GENOS=10309;Lingnan_Han_AN=1888;Lingnan_Han_AC=1;Lingna
chr7	95849181	.	A	<INS:ME:LINE1>	.	PASS	SVTYPE=INS:ME:LINE1;END=95849184;SVLEN=1980;AN=20618;AC=2;AF=9.700261907071492e-05;N_HET=2;N_HOMALT=0;N_HOMREF=10307;FREQ_HET=0.0001940052381414983;FREQ_HOMALT=0.0;FREQ_HOMREF=0.9998059947618586;N_BI_GENOS=10309;South_Han_AN=1496;South_Han_AC=1;South_Han
chr7	96016595	.	T	<INS:ME:LINE1>	.	PASS	SVTYPE=INS:ME:LINE1;END=96016609;SVLEN=515;AN=20618;AC=3;AF=0.00014550392860607236;N_HET=3;N_HOMALT=0;N_HOMREF=10306;FREQ_HET=0.0002910078572121447;FREQ_HOMALT=0.0;FREQ_HOMREF=0.9997089921427879;N_BI_GENOS=10309;South_Han_AN=1496;South_Han_AC=1;South_Han_
chr7	96208979	.	G	<INS:ME:LINE1>	.	PASS	SVTYPE=INS:ME:LINE1;END=96208994;SVLEN=5991;AN=20618;AC=5;AF=0.00024250654767678728;N_HET=5;N_HOMALT=0;N_HOMREF=10304;FREQ_HET=0.00048501309535357455;FREQ_HOMALT=0.0;FREQ_HOMREF=0.9995149869046465;N_BI_GENOS=10309;North_Han_AN=2272;North_Han_AC=1;North_Ha
chr7	96432059	.	G	<INS:ME:LINE1>	.	PASS	SVTYPE=INS:ME:LINE1;END=96432068;SVLEN=125;AN=20618;AC=4;AF=0.0001940052381414983;N_HET=4;N_HOMALT=0;N_HOMREF=10305;FREQ_HET=0.00038801047628285966;FREQ_HOMALT=0.0;FREQ_HOMREF=0.9996119895237171;N_BI_GENOS=10309;Tibetan_AN=414;Tibetan_AC=4;Tibetan_AF=0.0
chr7	96480771	.	A	<INS:ME:LINE1>	.	PASS	SVTYPE=INS:ME:LINE1;END=96480786;SVLEN=1040;AN=20618;AC=1;AF=4.850130953535746e-05;N_HET=1;N_HOMALT=0;N_HOMREF=10308;FREQ_HET=9.700261907071492e-05;FREQ_HOMALT=0.0;FREQ_HOMREF=0.9999029973809292;N_BI_GENOS=10309;Lingnan_Han_AN=1888;Lingnan_Han_AC=1;Lingna
chr7	96490583	.	A	<INS:ME:LINE1>	.	PASS	SVTYPE=INS:ME:LINE1;END=96490583;SVLEN=411;AN=20618;AC=1;AF=4.850130953535746e-05;N_HET=1;N_HOMALT=0;N_HOMREF=10308;FREQ_HET=9.700261907071492e-05;FREQ_HOMALT=0.0;FREQ_HOMREF=0.9999029973809292;N_BI_GENOS=10309;South_Han_AN=1496;South_Han_AC=1;South_Han_A
chr7	96679462	.	C	<INS:ME:LINE1>	.	PASS	SVTYPE=INS:ME:LINE1;END=96679474;SVLEN=937;AN=20618;AC=1;AF=4.850130953535746e-05;N_HET=1;N_HOMALT=0;N_HOMREF=10308;FREQ_HET=9.700261907071492e-05;FREQ_HOMALT=0.0;FREQ_HOMREF=0.9999029973809292;N_BI_GENOS=10309;East_Han_AN=6554;East_Han_AC=1;East_Han_AF=0
chr7	96827932	.	C	<INS:ME:LINE1>	.	PASS	SVTYPE=INS:ME:LINE1;END=96827944;SVLEN=1367;AN=20618;AC=93;AF=0.004510621786788243;N_HET=91;N_HOMALT=1;N_HOMREF=10217;FREQ_HET=0.008827238335435058;FREQ_HOMALT=9.700261907071492e-05;FREQ_HOMREF=0.9910757590454943;N_BI_GENOS=10309;South_Han_AN=1496;South_H
chr7	96891339	.	A	<INS:ME:LINE1>	.	PASS	SVTYPE=INS:ME:LINE1;END=96891348;SVLEN=4285;AN=20618;AC=2;AF=9.700261907071492e-05;N_HET=2;N_HOMALT=0;N_HOMREF=10307;FREQ_HET=0.0001940052381414983;FREQ_HOMALT=0.0;FREQ_HOMREF=0.9998059947618586;N_BI_GENOS=10309;South_Han_AN=1496;South_Han_AC=1;South_Han
chr7	96934028	.	A	<INS:ME:LINE1>	.	PASS	SVTYPE=INS:ME:LINE1;END=96934044;SVLEN=5501;AN=20618;AC=1;AF=4.850130953535746e-05;N_HET=1;N_HOMALT=0;N_HOMREF=10308;FREQ_HET=9.700261907071492e-05;FREQ_HOMALT=0.0;FREQ_HOMREF=0.9999029973809292;N_BI_GENOS=10309;Northwest_Han_AN=1296;Northwest_Han_AC=1;No

chr7	97109024	.	A	<INS:ME:LINE1>	.	PASS	SVTYPE=INS:ME:LINE1;END=97109040;SVLEN=5999;AN=20618;AC=1;AF=4.850130953535746e-05;N_HET=1;N_HOMALT=0;N_HOMREF=10308;FREQ_HET=9.700261907071492e-05;FREQ_HOMALT=0.0;FREQ_HOMREF=0.9999029973809292;N_BI_GENOS=10309;Southeast_Han_AN=1908;Southeast_Han_AC=1;So
chr7	97193646	.	A	<INS:ME:LINE1>	.	PASS	SVTYPE=INS:ME:LINE1;END=97193660;SVLEN=6022;AN=20618;AC=1;AF=4.850130953535746e-05;N_HET=1;N_HOMALT=0;N_HOMREF=10308;FREQ_HET=9.700261907071492e-05;FREQ_HOMALT=0.0;FREQ_HOMREF=0.9999029973809292;N_BI_GENOS=10309;East_Han_AN=6554;East_Han_AC=1;East_Han_AF=
chr7	97420336	.	A	<INS:ME:LINE1>	.	PASS	SVTYPE=INS:ME:LINE1;END=97420352;SVLEN=5915;AN=20618;AC=124;AF=0.006014162382384325;N_HET=120;N_HOMALT=2;N_HOMREF=10187;FREQ_HET=0.01164031428848579;FREQ_HOMALT=0.00019400523814142983;FREQ_HOMREF=0.9881656804733728;N_BI_GENOS=10309;South_Han_AN=1496;South
chr7	97745310	.	A	<INS:ME:LINE1>	.	PASS	SVTYPE=INS:ME:LINE1;END=97745325;SVLEN=233;AN=20618;AC=171;AF=0.008293723930546124;N_HET=163;N_HOMALT=4;N_HOMREF=10142;FREQ_HET=0.015811426908526532;FREQ_HOMALT=0.00038801047628285966;FREQ_HOMREF=0.9838005626151906;N_BI_GENOS=10309;South_Han_AN=1496;South
chr7	97773322	.	T	<INS:ME:LINE1>	.	PASS	SVTYPE=INS:ME:LINE1;END=97773327;SVLEN=734;AN=20618;AC=2;AF=9.700261907071492e-05;N_HET=2;N_HOMALT=0;N_HOMREF=10307;FREQ_HET=0.00019400523814142983;FREQ_HOMALT=0.0;FREQ_HOMREF=0.9998059947618586;N_BI_GENOS=10309;Lingnan_Han_AN=1888;Lingnan_Han_AC=1;Lingna
chr7	97774295	.	T	<INS:ME:LINE1>	.	PASS	SVTYPE=INS:ME:LINE1;END=97774295;SVLEN=2880;AN=20618;AC=3;AF=0.00014550392860607236;N_HET=3;N_HOMALT=0;N_HOMREF=10306;FREQ_HET=0.0002910078572121447;FREQ_HOMALT=0.0;FREQ_HOMREF=0.9997089921427879;N_BI_GENOS=10309;East_Han_AN=6554;East_Han_AC=2;East_Han_AF
chr7	98568474	.	C	<INS:ME:LINE1>	.	PASS	SVTYPE=INS:ME:LINE1;END=98568474;SVLEN=8;AN=20618;AC=1;AF=4.850130953535746e-05;N_HET=1;N_HOMALT=0;N_HOMREF=10308;FREQ_HET=9.700261907071492e-05;FREQ_HOMALT=0.0;FREQ_HOMREF=0.9999029973809292;N_BI_GENOS=10309;East_Han_AN=6554;East_Han_AC=1;East_Han_AF=0.0
chr7	98903311	.	T	<INS:ME:LINE1>	.	PASS	SVTYPE=INS:ME:LINE1;END=98903330;SVLEN=6004;AN=20618;AC=1;AF=4.850130953535746e-05;N_HET=1;N_HOMALT=0;N_HOMREF=10308;FREQ_HET=9.700261907071492e-05;FREQ_HOMALT=0.0;FREQ_HOMREF=0.9999029973809292;N_BI_GENOS=10309;North_Han_AN=2272;North_Han_AC=1;North_Han_
chr7	99021575	.	G	<INS:ME:LINE1>	.	PASS	SVTYPE=INS:ME:LINE1;END=99021594;SVLEN=603;AN=20618;AC=10;AF=0.00048501309535357455;N_HET=10;N_HOMALT=0;N_HOMREF=10299;FREQ_HET=0.0009700261907071491;FREQ_HOMALT=0.0;FREQ_HOMREF=0.9990299738092928;N_BI_GENOS=10309;South_Han_AN=1496;South_Han_AC=1;South_Ha
chr7	100899439	.	G	<INS:ME:LINE1>	.	PASS	SVTYPE=INS:ME:LINE1;END=100899452;SVLEN=126;AN=20618;AC=1;AF=4.850130953535746e-05;N_HET=1;N_HOMALT=0;N_HOMREF=10308;FREQ_HET=9.700261907071492e-05;FREQ_HOMALT=0.0;FREQ_HOMREF=0.9999029973809292;N_BI_GENOS=10309;Southeast_Han_AN=1908;Southeast_Han_AC=1;So
chr7	101197185	.	T	<INS:ME:LINE1>	.	PASS	SVTYPE=INS:ME:LINE1;END=101197185;SVLEN=12;AN=20618;AC=1;AF=4.850130953535746e-05;N_HET=1;N_HOMALT=0;N_HOMREF=10308;FREQ_HET=9.700261907071492e-05;FREQ_HOMALT=0.0;FREQ_HOMREF=0.9999029973809292;N_BI_GENOS=10309;Mongolian_AN=454;Mongolian_AC=1;Mongolian_AF
chr7	101653635	.	A	<INS:ME:LINE1>	.	PASS	SVTYPE=INS:ME:LINE1;END=101653650;SVLEN=178;AN=20618;AC=10;AF=0.00048501309535357455;N_HET=10;N_HOMALT=0;N_HOMREF=10299;FREQ_HET=0.0009700261907071491;FREQ_HOMALT=0.0;FREQ_HOMREF=0.9990299738092928;N_BI_GENOS=10309;East_Han_AN=6554;East_Han_AC=7;East_Han_
chr7	102842225	.	C	<INS:ME:LINE1>	.	PASS	SVTYPE=INS:ME:LINE1;END=102842225;SVLEN=6015;AN=20618;AC=1;AF=4.850130953535746e-05;N_HET=1;N_HOMALT=0;N_HOMREF=10308;FREQ_HET=9.700261907071492e-05;FREQ_HOMALT=0.0;FREQ_HOMREF=0.9999029973809292;N_BI_GENOS=10309;South_Han_AN=1496;South_Han_AC=1;South_Han
chr7	102947491	.	C	<INS:ME:LINE1>	.	PASS	SVTYPE=INS:ME:LINE1;END=102947506;SVLEN=6000;AN=20618;AC=1;AF=4.850130953535746e-05;N_HET=1;N_HOMALT=0;N_HOMREF=10308;FREQ_HET=9.700261907071492e-05;FREQ_HOMALT=0.0;FREQ_HOMREF=0.9999029973809292;N_BI_GENOS=10309;Southeast_Han_AN=1908;Southeast_Han_AC=1;S
chr7	103396332	.	A	<INS:ME:LINE1>	.	PASS	SVTYPE=INS:ME:LINE1;END=103396345;SVLEN=6059;AN=20618;AC=1;AF=4.850130953535746e-05;N_HET=1;N_HOMALT=0;N_HOMREF=10308;FREQ_HET=9.700261907071492e-05;FREQ_HOMALT=0.0;FREQ_HOMREF=0.9999029973809292;N_BI_GENOS=10309;Lingnan_Han_AN=1888;Lingnan_Han_AC=1;Lingn
chr7	103577700	.	G	<INS:ME:LINE1>	.	PASS	SVTYPE=INS:ME:LINE1;END=103577713;SVLEN=1812;AN=20618;AC=10;AF=0.00048501309535357455;N_HET=10;N_HOMALT=0;N_HOMREF=10299;FREQ_HET=0.0009700261907071491;FREQ_HOMALT=0.0;FREQ_HOMREF=0.9990299738092928;N_BI_GENOS=10309;South_Han_AN=1496;South_Han_AC=2;South_
chr7	103644138	.	A	<INS:ME:LINE1>	.	PASS	SVTYPE=INS:ME:LINE1;END=103644138;SVLEN=1766;AN=20618;AC=1;AF=4.850130953535746e-05;N_HET=1;N_HOMALT=0;N_HOMREF=10308;FREQ_HET=9.700261907071492e-05;FREQ_HOMALT=0.0;FREQ_HOMREF=0.9999029973809292;N_BI_GENOS=10309;East_Han_AN=6554;East_Han_AC=1;East_Han_AF
chr7	103646925	.	T	<INS:ME:LINE1>	.	PASS	SVTYPE=INS:ME:LINE1;END=103646925;SVLEN=544;AN=20618;AC=1;AF=4.850130953535746e-05;N_HET=1;N_HOMALT=0;N_HOMREF=10308;FREQ_HET=9.700261907071492e-05;FREQ_HOMALT=0.0;FREQ_HOMREF=0.9999029973809292;N_BI_GENOS=10309;East_Han_AN=6554;East_Han_AC=1;East_Han_AF=
chr7	103652460	.	A	<INS:ME:LINE1>	.	PASS	SVTYPE=INS:ME:LINE1;END=103652474;SVLEN=3066;AN=20618;AC=1;AF=4.850130953535746e-05;N_HET=1;N_HOMALT=0;N_HOMREF=10308;FREQ_HET=9.700261907071492e-05;FREQ_HOMALT=0.0;FREQ_HOMREF=0.9999029973809292;N_BI_GENOS=10309;East_Han_AN=6554;East_Han_AC=1;East_Han_AF
chr7	103731638	.	A	<INS:ME:LINE1>	.	PASS	SVTYPE=INS:ME:LINE1;END=103731639;SVLEN=1715;AN=20618;AC=12;AF=0.0005820157144242894;N_HET=12;N_HOMALT=0;N_HOMREF=10297;FREQ_HET=0.0011640314288485789;FREQ_HOMALT=0.0;FREQ_HOMREF=0.9988359685711514;N_BI_GENOS=10309;South_Han_AN=1496;South_Han_AC=2;South_H
chr7	104154067	.	T	<INS:ME:LINE1>	.	PASS	SVTYPE=INS:ME:LINE1;END=104154067;SVLEN=1430;AN=20618;AC=4;AF=0.00019400523814142983;N_HET=4;N_HOMALT=0;N_HOMREF=10305;FREQ_HET=0.00038801047628285966;FREQ_HOMALT=0.0;FREQ_HOMREF=0.9996119892537171;N_BI_GENOS=10309;South_Han_AN=1496;South_Han_AC=4;South_H
chr7	104259702	.	C	<INS:ME:LINE1>	.	PASS	SVTYPE=INS:ME:LINE1;END=104259711;SVLEN=6000;AN=20618;AC=1;AF=4.850130953535746e-05;N_HET=1;N_HOMALT=0;N_HOMREF=10308;FREQ_HET=9.700261907071492e-05;FREQ_HOMALT=0.0;FREQ_HOMREF=0.9999029973809292;N_BI_GENOS=10309;Central_Han_AN=2128;Central_Han_AC=1;Centr
chr7	104433842	.	A	<INS:ME:LINE1>	.	PASS	SVTYPE=INS:ME:LINE1;END=104433858;SVLEN=163;AN=20618;AC=1;AF=4.850130953535746e-05;N_HET=1;N_HOMALT=0;N_HOMREF=10308;FREQ_HET=9.700261907071492e-05;FREQ_HOMALT=0.0;FREQ_HOMREF=0.9999029973809292;N_BI_GENOS=10309;South_Han_AN=1496;South_Han_AC=1;South_Han_
chr7	104524206	.	C	<INS:ME:LINE1>	.	PASS	SVTYPE=INS:ME:LINE1;END=104524221;SVLEN=5945;AN=20618;AC=3;AF=0.00014550392860607236;N_HET=3;N_HOMALT=0;N_HOMREF=10306;FREQ_HET=0.0002910078572121447;FREQ_HOMALT=0.0;FREQ_HOMREF=0.9997089921427879;N_BI_GENOS=10309;East_Han_AN=6554;East_Han_AC=2;East_Han_A
chr7	104619117	.	T	<INS:ME:LINE1>	.	PASS	SVTYPE=INS:ME:LINE1;END=104619119;SVLEN=53;AN=20618;AC=1;AF=4.850130953535746e-05;N_HET=1;N_HOMALT=0;N_HOMREF=10308;FREQ_HET=9.700261907071492e-05;FREQ_HOMALT=0.0;FREQ_HOMREF=0.9999029973809292;N_BI_GENOS=10309;Zhuang_AN=444;Zhuang_AC=1;Zhuang_AF=0.002252
chr7	104741386	.	A	<INS:ME:LINE1>	.	PASS	SVTYPE=INS:ME:LINE1;END=104741399;SVLEN=884;AN=20618;AC=9;AF=0.0004365117858182171;N_HET=9;N_HOMALT=0;N_HOMREF=10300;FREQ_HET=0.0008730235716364342;FREQ_HOMALT=0.0;FREQ_HOMREF=0.9991269764283636;N_BI_GENOS=10309;South_Han_AN=1496;South_Han_AC=1;South_Han_
chr7	104771659	.	A	<INS:ME:LINE1>	.	PASS	SVTYPE=INS:ME:LINE1;END=104771677;SVLEN=5942;AN=20618;AC=601;AF=0.02914928703074983;N_HET=475;N_HOMALT=63;N_HOMREF=9771;FREQ_HET=0.04607624405858958;FREQ_HOMALT=0.006111165001455039;FREQ_HOMREF=0.9478125909399554;N_BI_GENOS=10309;South_Han_AN=1496;South_H
chr7	105188385	.	G	<INS:ME:LINE1>	.	PASS	SVTYPE=INS:ME:LINE1;END=105188393;SVLEN=5988;AN=20618;AC=10;AF=0.00048501309535357455;N_HET=10;N_HOMALT=0;N_HOMREF=10299;FREQ_HET=0.0009700261907071491;FREQ_HOMALT=0.0;FREQ_HOMREF=0.9990299738092928;N_BI_GENOS=10309;South_Han_AN=1496;South_Han_AC=3;South_
chr7	106327449	.	T	<INS:ME:LINE1>	.	PASS	SVTYPE=INS:ME:LINE1;END=106327449;SVLEN=0;AN=20618;AC=66;AF=0.003201086429333592;N_HET=50;N_HOMALT=8;N_HOMREF=10251;FREQ_HET=0.004850130953535745;FREQ_HOMALT=0.0007760209525657193;FREQ_HOMREF=0.9943738480938985;N_BI_GENOS=10309;South_Han_AN=1496;South_Han
chr7	106405675	.	A	<INS:ME:LINE1>	.	PASS	SVTYPE=INS:ME:LINE1;END=106405680;SVLEN=426;AN=20618;AC=1;AF=4.850130953535746e-05;N_HET=1;N_HOMALT=0;N_HOMREF=10308;FREQ_HET=9.700261907071492e-05;FREQ_HOMALT=0.0;FREQ_HOMREF=0.9999029973809292;N_BI_GENOS=10309;Hui_AN=444;Hui_AC=1;Hui_AF=0.00225225225225
chr7	107081898	.	T	<INS:ME:LINE1>	.	PASS	SVTYPE=INS:ME:LINE1;END=107081912;SVLEN=6000;AN=20618;AC=2;AF=9.700261907071492e-05;N_HET=2;N_HOMALT=0;N_HOMREF=10307;FREQ_HET=0.00019400523814142983;FREQ_HOMALT=0.0;FREQ_HOMREF=0.9998059947618586;N_BI_GENOS=10309;South_Han_AN=1496;South_Han_AC=2;South_Ha
chr7	107086476	.	C	<INS:ME:LINE1>	.	PASS	SVTYPE=INS:ME:LINE1;END=107086496;SVLEN=6002;AN=20618;AC=2;AF=9.700261907071492e-05;N_HET=2;N_HOMALT=0;N_HOMREF=10307;FREQ_HET=0.00019400523814142983;FREQ_HOMALT=0.0;FREQ_HOMREF=0.9998059947618586;N_BI_GENOS=10309;East_Han_AN=6554;East_Han_AC=2;East_Han_A
chr7	107374708	.	A	<INS:ME:LINE1>	.	PASS	SVTYPE=INS:ME:LINE1;END=107374727;SVLEN=5999;AN=20618;AC=2;AF=9.700261907071492e-05;N_HET=2;N_HOMALT=0;N_HOMREF=10307;FREQ_HET=0.00019400523814142983;FREQ_HOMALT=0.0;FREQ_HOMREF=0.9998059947618586;N_BI_GENOS=10309;East_Han_AN=6554;East_Han_AC=2;East_Han_A
chr7	107541613	.	A	<INS:ME:LINE1>	.	PASS	SVTYPE=INS:ME:LINE1;END=107541628;SVLEN=149;AN=20618;AC=1;AF=4.850130953535746e-05;N_HET=1;N_HOMALT=0;N_HOMREF=10308;FREQ_HET=9.700261907071492e-05;FREQ_HOMALT=0.0;FREQ_HOMREF=0.9999029973809292;N_BI_GENOS=10309;East_Han_AN=6554;East_Han_AC=1;East_Han_AF=
chr7	107555366	.	G	<INS:ME:LINE1>	.	PASS	SVTYPE=INS:ME:LINE1;END=107555379;SVLEN=6000;AN=20618;AC=1;AF=4.850130953535746e-05;N_HET=1;N_HOMALT=0;N_HOMREF=10308;FREQ_HET=9.700261907071492e-05;FREQ_HOMALT=0.0;FREQ_HOMREF=0.9999029973809292;N_BI_GENOS=10309;Northwest_Han_AN=1296;Northwest_Han_AC=1;N
chr7	108188804	.	T	<INS:ME:LINE1>	.	PASS	SVTYPE=INS:ME:LINE1;END=108188819;SVLEN=5998;AN=20618;AC=19823;AF=0.9614414589193908;N_HET=625;N_HOMALT=9599;N_HOMREF=85;FREQ_HET=0.06062663691919682;FREQ_HOMALT=0.9311281404597924;FREQ_HOMREF=0.008245222621010767;N_BI_GENOS=10309;South_Han_AN=1496;South_
chr7	108287157	.	G	<INS:ME:LINE1>	.	PASS	SVTYPE=INS:ME:LINE1;END=108287157;SVLEN=283;AN=20618;AC=1;AF=4.850130953535746e-05;N_HET=1;N_HOMALT=0;N_HOMREF=10308;FREQ_HET=9.700261907071492e-05;FREQ_HOMALT=0.0;FREQ_HOMREF=0.9999029973809292;N_BI_GENOS=10309;East_Han_AN=6554;East_Han_AC=1;East_Han_AF=
chr7	108287810	.	A	<INS:ME:LINE1>	.	PASS	SVTYPE=INS:ME:LINE1;END=108287810;SVLEN=586;AN=20618;AC=1;AF=4.850130953535746e-05;N_HET=1;N_HOMALT=0;N_HOMREF=10308;FREQ_HET=9.700261907071492e-05;FREQ_HOMALT=0.0;FREQ_HOMREF=0.9999029973809292;N_BI_GENOS=10309;East_Han_AN=6554;East_Han_AC=1;East_Han_AF=
chr7	108665673	.	T	<INS:ME:LINE1>	.	PASS	SVTYPE=INS:ME:LINE1;END=108665690;SVLEN=803;AN=20618;AC=1;AF=4.850130953535746e-05;N_HET=1;N_HOMALT=0;N_HOMREF=10308;FREQ_HET=9.700261907071492e-05;FREQ_HOMALT=0.0;FREQ_HOMREF=0.9999029973809292;N_BI_GENOS=10309;Central_Han_AN=2128;Central_Han_AC=1;Centra
chr7	108685216	.	A	<INS:ME:LINE1>	.	PASS	SVTYPE=INS:ME:LINE1;END=108685234;SVLEN=1082;AN=20618;AC=712;AF=0.03453293238917451;N_HET=514;N_HOMALT=99;N_HOMREF=9696;FREQ_HET=0.049859346202347464;FREQ_HOMALT=0.009603259288000775;FREQ_HOMREF=0.9405373945096518;N_BI_GENOS=10309;South_Han_AN=1496;South_
chr7	108959611	.	A	<INS:ME:LINE1>	.	PASS	SVTYPE=INS:ME:LINE1;END=108959627;SVLEN=1691;AN=20618;AC=1;AF=4.850130953535746e-05;N_HET=1;N_HOMALT=0;N_HOMREF=10308;FREQ_HET=9.700261907071492e-05;FREQ_HOMALT=0.0;FREQ_HOMREF=0.9999029973809292;N_BI_GENOS=10309;East_Han_AN=6554;East_Han_AC=1;East_Han_AF
chr7	109036512	.	T	<INS:ME:LINE1>	.	PASS	SVTYPE=INS:ME:LINE1;END=109036529;SVLEN=330;AN=20618;AC=1;AF=4.850130953535746e-05;N_HET=1;N_HOMALT=0;N_HOMREF=10308;FREQ_HET=9.700261907071492e-05;FREQ_HOMALT=0.0;FREQ_HOMREF=0.9999029973809292;N_BI_GENOS=10309;East_Han_AN=6554;East_Han_AC=1;East_Han_AF=
chr7	109116534	.	T	<INS:ME:LINE1>	.	PASS	SVTYPE=INS:ME:LINE1;END=109116549;SVLEN=5976;AN=20618;AC=1;AF=4.850130953535746e-05;N_HET=1;N_HOMALT=0;N_HOMREF=10308;FREQ_HET=9.700261907071492e-05;FREQ_HOMALT=0.0;FREQ_HOMREF=0.9999029973809292;N_BI_GENOS=10309;Lingnan_Han_AN=1888;Lingnan_Han_AC=1;Lingn
chr7	109345809	.	A	<INS:ME:LINE1>	.	PASS	SVTYPE=INS:ME:LINE1;END=109345819;SVLEN=1092;AN=20618;AC=1;AF=4.850130953535746e-05;N_HET=1;N_HOMALT=0;N_HOMREF=10308;FREQ_HET=9.700261907071492e-05;FREQ_HOMALT=0.0;FREQ_HOMREF=0.9999029973809292;N_BI_GENOS=10309;Manchu_AN=416;Manchu_AC=1;Manchu_AF=0.0024
chr7	109452391	.	T	<INS:ME:LINE1>	.	PASS	SVTYPE=INS:ME:LINE1;END=109452406;SVLEN=1422;AN=20618;AC=1;AF=4.850130953535746e-05;N_HET=1;N_HOMALT=0;N_HOMREF=10308;FREQ_HET=9.700261907071492e-05;FREQ_HOMALT=0.0;FREQ_HOMREF=0.9999029973809292;N_BI_GENOS=10309;East_Han_AN=6554;East_Han_AC=1;East_Han_AF
chr7	109597514	.	A	<INS:ME:LINE1>	.	PASS	SVTYPE=INS:ME:LINE1;END=109597514;SVLEN=0;AN=20618;AC=1;AF=4.850130953535746e-05;N_HET=1;N_HOMALT=0;N_HOMREF=10308;FREQ_HET=9.700261907071492e-05;FREQ_HOMALT=0.0;FREQ_HOMREF=0.9999029973809292;N_BI_GENOS=10309;Southeast_Han_AN=1908;Southeast_Han_AC=1;Sout
chr7	109615232	.	G	<INS:ME:LINE1>	.	PASS	SVTYPE=INS:ME:LINE1;END=109615247;SVLEN=1898;AN=20618;AC=1;AF=4.850130953535746e-05;N_HET=1;N_HOMALT=0;N_HOMREF=10308;FREQ_HET=9.700261907071492e-05;FREQ_HOMALT=0.0;FREQ_HOMREF=0.9999029973809292;N_BI_GENOS=10309;Central_Han_AN=2128;Central_Han_AC=1;Centr
chr7	109756206	.	C	<INS:ME:LINE1>	.	PASS	SVTYPE=INS:ME:LINE1;END=109756222;SVLEN=1475;AN=20618;AC=1;AF=4.850130953535746e-05;N_HET=1;N_HOMALT=0;N_HOMREF=10308;FREQ_HET=9.700261907071492e-05;FREQ_HOMALT=0.0;FREQ_HOMREF=0.9999029973809292;N_BI_GENOS=10309;East_Han_AN=6554;East_Han_AC=1;East_Han_AF
chr7	109955101	.	C	<INS:ME:LINE1>	.	PASS	SVTYPE=INS:ME:LINE1;END=109955113;SVLEN=1373;AN=20618;AC=3;AF=0.00014550392860607236;N_HET=3;N_HOMALT=0;N_HOMREF=10306;FREQ_HET=0.0002910078572121447;FREQ_HOMALT=0.0;FREQ_HOMREF=0.9997089921427879;N_BI_GENOS=10309;South_Han_AN=1496;South_Han_AC=1;South_Ha
chr7	110100455	.	A	<INS:ME:LINE1>	.	PASS	SVTYPE=INS:ME:LINE1;END=110100488;SVLEN=1088;AN=20618;AC=1;AF=4.850130953535746e-05;N_HET=1;N_HOMALT=0;N_HOMREF=10308;FREQ_HET=9.700261907071492e-05;FREQ_HOMALT=0.0;FREQ_HOMREF=0.9999029973809292;N_BI_GENOS=10309;East_Han_AN=6554;East_Han_AC=1;East_Han_AF
chr7	110132206	.	T	<INS:ME:LINE1>	.	PASS	SVTYPE=INS:ME:LINE1;END=110132206;SVLEN=385;AN=20618;AC=1;AF=4.850130953535746e-05;N_HET=1;N_HOMALT=0;N_HOMREF=10308;FREQ_HET=9.700261907071492e-05;FREQ_HOMALT=0.0;FREQ_HOMREF=0.9999029973809292;N_BI_GENOS=10309;East_Han_AN=6554;East_Han_AC=1;East_Han_AF=
chr7	110132392	.	A	<INS:ME:LINE1>	.	PASS	SVTYPE=INS:ME:LINE1;END=110132392;SVLEN=714;AN=20618;AC=2;AF=9.700261907071492e-05;N_HET=2;N_HOMALT=0;N_HOMREF=10307;FREQ_HET=0.00019400523814142983;FREQ_HOMALT=0.0;FREQ_HOMREF=0.9998059947618586;N_BI_GENOS=10309;East_Han_AN=6554;East_Han_AC=2;East_Han_AF
chr7	110187085	.	A	<INS:ME:LINE1>	.	PASS	SVTYPE=INS:ME:LINE1;END=110187101;SVLEN=840;AN=20618;AC=1;AF=4.850130953535746e-05;N_HET=1;N_HOMALT=0;N_HOMREF=10308;FREQ_HET=9.700261907071492e-05;FREQ_HOMALT=0.0;FREQ_HOMREF=0.9999029973809292;N_BI_GENOS=10309;East_Han_AN=6554;East_Han_AC=1;East_Han_AF=
chr7	110229096	.	A	<INS:ME:LINE1>	.	PASS	SVTYPE=INS:ME:LINE1;END=110229103;SVLEN=3289;AN=20618;AC=4;AF=0.00019400523814142983;N_HET=2;N_HOMALT=1;N_HOMREF=10306;FREQ_HET=0.00019400523814142983;FREQ_HOMALT=9.700261907071492e-05;FREQ_HOMREF=0.9997089921427879;N_BI_GENOS=10309;East_Han_AN=6554;East_
chr7	110507046	.	A	<INS:ME:LINE1>	.	PASS	SVTYPE=INS:ME:LINE1;END=110507065;SVLEN=5998;AN=20618;AC=1;AF=4.850130953535746e-05;N_HET=1;N_HOMALT=0;N_HOMREF=10308;FREQ_HET=9.700261907071492e-05;FREQ_HOMALT=0.0;FREQ_HOMREF=0.9999029973809292;N_BI_GENOS=10309;East_Han_AN=6554;East_Han_AC=1;East_Han_AF
chr7	110778727	.	A	<INS:ME:LINE1>	.	PASS	SVTYPE=INS:ME:LINE1;END=110778741;SVLEN=919;AN=20618;AC=1;AF=4.850130953535746e-05;N_HET=1;N_HOMALT=0;N_HOMREF=10308;FREQ_HET=9.700261907071492e-05;FREQ_HOMALT=0.0;FREQ_HOMREF=0.9999029973809292;N_BI_GENOS=10309;East_Han_AN=6554;East_Han_AC=1;East_Han_AF=
chr7	110844693	.	T	<INS:ME:LINE1>	.	PASS	SVTYPE=INS:ME:LINE1;END=110844694;SVLEN=58;AN=20618;AC=13242;AF=0.6422543408672035;N_HET=1152;N_HOMALT=6045;N_HOMREF=3112;FREQ_HET=0.11174701716946357;FREQ_HOMALT=0.5863808322824716;FREQ_HOMREF=0.3018721505480648;N_BI_GENOS=10309;South_Han_AN=1496;South_H
chr7	110888168	.	A	<INS:ME:LINE1>	.	PASS	SVTYPE=INS:ME:LINE1;END=110888184;SVLEN=2580;AN=20618;AC=3;AF=0.00014550392860607236;N_HET=3;N_HOMALT=0;N_HOMREF=10306;FREQ_HET=0.0002910078572121447;FREQ_HOMALT=0.0;FREQ_HOMREF=0.9997089921427879;N_BI_GENOS=10309;East_Han_AN=6554;East_Han_AC=2;East_Han_A
chr7	110900140	.	A	<INS:ME:LINE1>	.	PASS	SVTYPE=INS:ME:LINE1;END=110900160;SVLEN=5998;AN=20618;AC=1;AF=4.850130953535746e-05;N_HET=1;N_HOMALT=0;N_HOMREF=10308;FREQ_HET=9.700261907071492e-05;FREQ_HOMALT=0.0;FREQ_HOMREF=0.9999029973809292;N_BI_GENOS=10309;Central_Han_AN=2128;Central_Han_AC=1;Centr
chr7	111295195	.	T	<INS:ME:LINE1>	.	PASS	SVTYPE=INS:ME:LINE1;END=111295213;SVLEN=1857;AN=20618;AC=1;AF=4.850130953535746e-05;N_HET=1;N_HOMALT=0;N_HOMREF=10308;FREQ_HET=9.700261907071492e-05;FREQ_HOMALT=0.0;FREQ_HOMREF=0.9999029973809292;N_BI_GENOS=10309;Mongolian_AN=454;Mongolian_AC=1;Mongolian_
chr7	111364336	.	A	<INS:ME:LINE1>	.	PASS	SVTYPE=INS:ME:LINE1;END=111364352;SVLEN=3685;AN=20618;AC=1;AF=4.850130953535746e-05;N_HET=1;N_HOMALT=0;N_HOMREF=10308;FREQ_HET=9.700261907071492e-05;FREQ_HOMALT=0.0;FREQ_HOMREF=0.9999029973809292;N_BI_GENOS=10309;South_Han_AN=1496;South_Han_AC=1;South_Han
chr7	111409949	.	G	<INS:ME:LINE1>	.	PASS	SVTYPE=INS:ME:LINE1;END=111409949;SVLEN=695;AN=20618;AC=1;AF=4.850130953535746e-05;N_HET=1;N_HOMALT=0;N_HOMREF=10308;FREQ_HET=9.700261907071492e-05;FREQ_HOMALT=0.0;FREQ_HOMREF=0.9999029973809292;N_BI_GENOS=10309;South_Han_AN=1496;South_Han_AC=1;South_Han_
chr7	111517135	.	A	<INS:ME:LINE1>	.	PASS	SVTYPE=INS:ME:LINE1;END=111517135;SVLEN=0;AN=20618;AC=1;AF=4.850130953535746e-05;N_HET=1;N_HOMALT=0;N_HOMREF=10308;FREQ_HET=9.700261907071492e-05;FREQ_HOMALT=0.0;FREQ_HOMREF=0.9999029973809292;N_BI_GENOS=10309;Yi_AN=422;Yi_AC=1;Yi_AF=0.002369668246445498;
chr7	111592851	.	G	<INS:ME:LINE1>	.	PASS	SVTYPE=INS:ME:LINE1;END=111592879;SVLEN=4297;AN=20618;AC=2;AF=9.700261907071492e-05;N_HET=2;N_HOMALT=0;N_HOMREF=10307;FREQ_HET=0.00019400523814142983;FREQ_HOMALT=0.0;FREQ_HOMREF=0.9998059947618586;N_BI_GENOS=10309;East_Han_AN=6554;East_Han_AC=1;East_Han_A
chr7	111675027	.	A	<INS:ME:LINE1>	.	PASS	SVTYPE=INS:ME:LINE1;END=111675040;SVLEN=720;AN=20618;AC=1;AF=4.850130953535746e-05;N_HET=1;N_HOMALT=0;N_HOMREF=10308;FREQ_HET=9.700261907071492e-05;FREQ_HOMALT=0.0;FREQ_HOMREF=0.9999029973809292;N_BI_GENOS=10309;Tibetan_AN=414;Tibetan_AC=1;Tibetan_AF=0.00
chr7	111792684	.	C	<INS:ME:LINE1>	.	PASS	SVTYPE=INS:ME:LINE1;END=111792697;SVLEN=6042;AN=20618;AC=1;AF=4.850130953535746e-05;N_HET=1;N_HOMALT=0;N_HOMREF=10308;FREQ_HET=9.700261907071492e-05;FREQ_HOMALT=0.0;FREQ_HOMREF=0.9999029973809292;N_BI_GENOS=10309;Northwest_Han_AN=1296;Northwest_Han_AC=1;N
chr7	111954711	.	A	<INS:ME:LINE1>	.	PASS	SVTYPE=INS:ME:LINE1;END=111954726;SVLEN=3795;AN=20618;AC=1;AF=4.850130953535746e-05;N_HET=1;N_HOMALT=0;N_HOMREF=10308;FREQ_HET=9.700261907071492e-05;FREQ_HOMALT=0.0;FREQ_HOMREF=0.9999029973809292;N_BI_GENOS=10309;Southeast_Han_AN=1908;Southeast_Han_AC=1;S
chr7	112123254	.	A	<INS:ME:LINE1>	.	PASS	SVTYPE=INS:ME:LINE1;END=112123270;SVLEN=5999;AN=20618;AC=1;AF=4.850130953535746e-05;N_HET=1;N_HOMALT=0;N_HOMREF=10308;FREQ_HET=9.700261907071492e-05;FREQ_HOMALT=0.0;FREQ_HOMREF=0.9999029973809292;N_BI_GENOS=10309;East_Han_AN=6554;East_Han_AC=1;East_Han_AF
chr7	112377143	.	A	<INS:ME:LINE1>	.	PASS	SVTYPE=INS:ME:LINE1;END=112377143;SVLEN=5995;AN=20618;AC=1;AF=4.850130953535746e-05;N_HET=1;N_HOMALT=0;N_HOMREF=10308;FREQ_HET=9.700261907071492e-05;FREQ_HOMALT=0.0;FREQ_HOMREF=0.9999029973809292;N_BI_GENOS=10309;East_Han_AN=6554;East_Han_AC=1;East_Han_AF
chr7	112493279	.	A	<INS:ME:LINE1>	.	PASS	SVTYPE=INS:ME:LINE1;END=112493291;SVLEN=1783;AN=20618;AC=1;AF=4.850130953535746e-05;N_HET=1;N_HOMALT=0;N_HOMREF=10308;FREQ_HET=9.700261907071492e-05;FREQ_HOMALT=0.0;FREQ_HOMREF=0.9999029973809292;N_BI_GENOS=10309;North_Han_AN=2272;North_Han_AC=1;North_Han
chr7	112593699	.	T	<INS:ME:LINE1>	.	PASS	SVTYPE=INS:ME:LINE1;END=112593714;SVLEN=4261;AN=20618;AC=1;AF=4.850130953535746e-05;N_HET=1;N_HOMALT=0;N_HOMREF=10308;FREQ_HET=9.700261907071492e-05;FREQ_HOMALT=0.0;FREQ_HOMREF=0.9999029973809292;N_BI_GENOS=10309;East_Han_AN=6554;East_Han_AC=1;East_Han_AF
chr7	112874027	.	T	<INS:ME:LINE1>	.	PASS	SVTYPE=INS:ME:LINE1;END=112874027;SVLEN=385;AN=20618;AC=1;AF=4.850130953535746e-05;N_HET=1;N_HOMALT=0;N_HOMREF=10308;FREQ_HET=9.700261907071492e-05;FREQ_HOMALT=0.0;FREQ_HOMREF=0.9999029973809292;N_BI_GENOS=10309;Northwest_Han_AN=1296;Northwest_Han_AC=1;No
chr7	113149555	.	A	<INS:ME:LINE1>	.	PASS	SVTYPE=INS:ME:LINE1;END=113149568;SVLEN=825;AN=20618;AC=21;AF=0.0010185275002425065;N_HET=21;N_HOMALT=0;N_HOMREF=10288;FREQ_HET=0.002037055000485013;FREQ_HOMALT=0.0;FREQ_HOMREF=0.997962944999515;N_BI_GENOS=10309;South_Han_AN=1496;South_Han_AC=1;South_Han_
chr7	113197653	.	A	<INS:ME:LINE1>	.	PASS	SVTYPE=INS:ME:LINE1;END=113197669;SVLEN=1032;AN=20618;AC=1;AF=4.850130953535746e-05;N_HET=1;N_HOMALT=0;N_HOMREF=10308;FREQ_HET=9.700261907071492e-05;FREQ_HOMALT=0.0;FREQ_HOMREF=0.9999029973809292;N_BI_GENOS=10309;East_Han_AN=6554;East_Han_AC=1;East_Han_AF
chr7	113237416	.	C	<INS:ME:LINE1>	.	PASS	SVTYPE=INS:ME:LINE1;END=113237416;SVLEN=0;AN=20618;AC=2;AF=9.700261907071492e-05;N_HET=2;N_HOMALT=0;N_HOMREF=10307;FREQ_HET=0.00019400523814142983;FREQ_HOMALT=0.0;FREQ_HOMREF=0.9998059947618586;N_BI_GENOS=10309;South_Han_AN=1496;South_Han_AC=2;South_Han_A
chr7	113309760	.	T	<INS:ME:LINE1>	.	PASS	SVTYPE=INS:ME:LINE1;END=113309776;SVLEN=5999;AN=20618;AC=1;AF=4.850130953535746e-05;N_HET=1;N_HOMALT=0;N_HOMREF=10308;FREQ_HET=9.700261907071492e-05;FREQ_HOMALT=0.0;FREQ_HOMREF=0.9999029973809292;N_BI_GENOS=10309;Lingnan_Han_AN=1888;Lingnan_Han_AC=1;Lingn
chr7	113388062	.	T	<INS:ME:LINE1>	.	PASS	SVTYPE=INS:ME:LINE1;END=113388076;SVLEN=730;AN=20618;AC=2;AF=9.700261907071492e-05;N_HET=2;N_HOMALT=0;N_HOMREF=10307;FREQ_HET=0.00019400523814142983;FREQ_HOMALT=0.0;FREQ_HOMREF=0.9998059947618586;N_BI_GENOS=10309;Northwest_Han_AN=1296;Northwest_Han_AC=1;N
chr7	113613418	.	A	<INS:ME:LINE1>	.	PASS	SVTYPE=INS:ME:LINE1;END=113613429;SVLEN=954;AN=20618;AC=1;AF=4.850130953535746e-05;N_HET=1;N_HOMALT=0;N_HOMREF=10308;FREQ_HET=9.700261907071492e-05;FREQ_HOMALT=0.0;FREQ_HOMREF=0.9999029973809292;N_BI_GENOS=10309;Northwest_Han_AN=1296;Northwest_Han_AC=1;No
chr7	113653137	.	A	<INS:ME:LINE1>	.	PASS	SVTYPE=INS:ME:LINE1;END=113653146;SVLEN=714;AN=20618;AC=1;AF=4.850130953535746e-05;N_HET=1;N_HOMALT=0;N_HOMREF=10308;FREQ_HET=9.700261907071492e-05;FREQ_HOMALT=0.0;FREQ_HOMREF=0.9999029973809292;N_BI_GENOS=10309;Lingnan_Han_AN=1888;Lingnan_Han_AC=1;Lingna
chr7	113799340	.	G	<INS:ME:LINE1>	.	PASS	SVTYPE=INS:ME:LINE1;END=113799354;SVLEN=6008;AN=20618;AC=1;AF=4.850130953535746e-05;N_HET=1;N_HOMALT=0;N_HOMREF=10308;FREQ_HET=9.700261907071492e-05;FREQ_HOMALT=0.0;FREQ_HOMREF=0.9999029973809292;N_BI_GENOS=10309;Southeast_Han_AN=1908;Southeast_Han_AC=1;S
chr7	113850580	.	A	<INS:ME:LINE1>	.	PASS	SVTYPE=INS:ME:LINE1;END=113850598;SVLEN=103;AN=20618;AC=2;AF=9.700261907071492e-05;N_HET=2;N_HOMALT=0;N_HOMREF=10307;FREQ_HET=0.00019400523814142983;FREQ_HOMALT=0.0;FREQ_HOMREF=0.9998059947618586;N_BI_GENOS=10309;Miao_AN=482;Miao_AC=2;Miao_AF=0.0041493775
chr7	113854833	.	A	<INS:ME:LINE1>	.	PASS	SVTYPE=INS:ME:LINE1;END=113854833;SVLEN=396;AN=20618;AC=1;AF=4.850130953535746e-05;N_HET=1;N_HOMALT=0;N_HOMREF=10308;FREQ_HET=9.700261907071492e-05;FREQ_HOMALT=0.0;FREQ_HOMREF=0.9999029973809292;N_BI_GENOS=10309;Hui_AN=444;Hui_AC=1;Hui_AF=0.00225225225225
chr7	113871164	.	A	<INS:ME:LINE1>	.	PASS	SVTYPE=INS:ME:LINE1;END=113871180;SVLEN=479;AN=20618;AC=9;AF=0.0004365117858182171;N_HET=1;N_HOMALT=4;N_HOMREF=10304;FREQ_HET=9.700261907071492e-05;FREQ_HOMALT=0.00038801047628285966;FREQ_HOMREF=0.9995149869046465;N_BI_GENOS=10309;North_Han_AN=2272;North_
chr7	113896570	.	T	<INS:ME:LINE1>	.	PASS	SVTYPE=INS:ME:LINE1;END=113896570;SVLEN=1028;AN=20618;AC=1;AF=4.850130953535746e-05;N_HET=1;N_HOMALT=0;N_HOMREF=10308;FREQ_HET=9.700261907071492e-05;FREQ_HOMALT=0.0;FREQ_HOMREF=0.9999029973809292;N_BI_GENOS=10309;East_Han_AN=6554;East_Han_AC=1;East_Han_AF
chr7	113905558	.	T	<INS:ME:LINE1>	.	PASS	SVTYPE=INS:ME:LINE1;END=113905576;SVLEN=508;AN=20618;AC=1;AF=4.850130953535746e-05;N_HET=1;N_HOMALT=0;N_HOMREF=10308;FREQ_HET=9.700261907071492e-05;FREQ_HOMALT=0.0;FREQ_HOMREF=0.9999029973809292;N_BI_GENOS=10309;Lingnan_Han_AN=1888;Lingnan_Han_AC=1;Lingna
chr7	113909244	.	A	<INS:ME:LINE1>	.	PASS	SVTYPE=INS:ME:LINE1;END=113909244;SVLEN=5961;AN=20618;AC=1;AF=4.850130953535746e-05;N_HET=1;N_HOMALT=0;N_HOMREF=10308;FREQ_HET=9.700261907071492e-05;FREQ_HOMALT=0.0;FREQ_HOMREF=0.9999029973809292;N_BI_GENOS=10309;East_Han_AN=6554;East_Han_AC=1;East_Han_AF
chr7	113974295	.	A	<INS:ME:LINE1>	.	PASS	SVTYPE=INS:ME:LINE1;END=113974308;SVLEN=870;AN=20618;AC=2;AF=9.700261907071492e-05;N_HET=2;N_HOMALT=0;N_HOMREF=10307;FREQ_HET=0.00019400523814142983;FREQ_HOMALT=0.0;FREQ_HOMREF=0.9998059947618586;N_BI_GENOS=10309;Miao_AN=482;Miao_AC=2;Miao_AF=0.0041493775
chr7	114433710	.	T	<INS:ME:LINE1>	.	PASS	SVTYPE=INS:ME:LINE1;END=114433710;SVLEN=5987;AN=20618;AC=1;AF=4.850130953535746e-05;N_HET=1;N_HOMALT=0;N_HOMREF=10308;FREQ_HET=9.700261907071492e-05;FREQ_HOMALT=0.0;FREQ_HOMREF=0.9999029973809292;N_BI_GENOS=10309;Tibetan_AN=414;Tibetan_AC=1;Tibetan_AF=0.0
chr7	114498747	.	A	<INS:ME:LINE1>	.	PASS	SVTYPE=INS:ME:LINE1;END=114498758;SVLEN=1542;AN=20618;AC=1;AF=4.850130953535746e-05;N_HET=1;N_HOMALT=0;N_HOMREF=10308;FREQ_HET=9.700261907071492e-05;FREQ_HOMALT=0.0;FREQ_HOMREF=0.9999029973809292;N_BI_GENOS=10309;Northwest_Han_AN=1296;Northwest_Han_AC=1;N
chr7	114545795	.	C	<INS:ME:LINE1>	.	PASS	SVTYPE=INS:ME:LINE1;END=114545810;SVLEN=5975;AN=20618;AC=1;AF=4.850130953535746e-05;N_HET=1;N_HOMALT=0;N_HOMREF=10308;FREQ_HET=9.700261907071492e-05;FREQ_HOMALT=0.0;FREQ_HOMREF=0.9999029973809292;N_BI_GENOS=10309;Central_Han_AN=2128;Central_Han_AC=1;Centr
chr7	115129776	.	G	<INS:ME:LINE1>	.	PASS	SVTYPE=INS:ME:LINE1;END=115129792;SVLEN=1307;AN=20618;AC=4;AF=0.00019400523814142983;N_HET=4;N_HOMALT=0;N_HOMREF=10305;FREQ_HET=0.00038801047628285966;FREQ_HOMALT=0.0;FREQ_HOMREF=0.9996119892537171;N_BI_GENOS=10309;North_Han_AN=2272;North_Han_AC=1;North_H
chr7	115355248	.	T	<INS:ME:LINE1>	.	PASS	SVTYPE=INS:ME:LINE1;END=115355261;SVLEN=1710;AN=20618;AC=1;AF=4.850130953535746e-05;N_HET=1;N_HOMALT=0;N_HOMREF=10308;FREQ_HET=9.700261907071492e-05;FREQ_HOMALT=0.0;FREQ_HOMREF=0.9999029973809292;N_BI_GENOS=10309;East_Han_AN=6554;East_Han_AC=1;East_Han_AF
chr7	115356791	.	T	<INS:ME:LINE1>	.	PASS	SVTYPE=INS:ME:LINE1;END=115356791;SVLEN=1837;AN=20618;AC=1;AF=4.850130953535746e-05;N_HET=1;N_HOMALT=0;N_HOMREF=10308;FREQ_HET=9.700261907071492e-05;FREQ_HOMALT=0.0;FREQ_HOMREF=0.9999029973809292;N_BI_GENOS=10309;Northwest_Han_AN=1296;Northwest_Han_AC=1;N
chr7	115411493	.	G	<INS:ME:LINE1>	.	PASS	SVTYPE=INS:ME:LINE1;END=115411506;SVLEN=1834;AN=20618;AC=1;AF=4.850130953535746e-05;N_HET=1;N_HOMALT=0;N_HOMREF=10308;FREQ_HET=9.700261907071492e-05;FREQ_HOMALT=0.0;FREQ_HOMREF=0.9999029973809292;N_BI_GENOS=10309;Northwest_Han_AN=1296;Northwest_Han_AC=1;N
chr7	115461035	.	T	<INS:ME:LINE1>	.	PASS	SVTYPE=INS:ME:LINE1;END=115461044;SVLEN=1242;AN=20618;AC=1;AF=4.850130953535746e-05;N_HET=1;N_HOMALT=0;N_HOMREF=10308;FREQ_HET=9.700261907071492e-05;FREQ_HOMALT=0.0;FREQ_HOMREF=0.9999029973809292;N_BI_GENOS=10309;East_Han_AN=6554;East_Han_AC=1;East_Han_AF
chr7	115548083	.	T	<INS:ME:LINE1>	.	PASS	SVTYPE=INS:ME:LINE1;END=115548099;SVLEN=383;AN=20618;AC=1;AF=4.850130953535746e-05;N_HET=1;N_HOMALT=0;N_HOMREF=10308;FREQ_HET=9.700261907071492e-05;FREQ_HOMALT=0.0;FREQ_HOMREF=0.9999029973809292;N_BI_GENOS=10309;Yi_AN=422;Yi_AC=1;Yi_AF=0.00236966824644549

chr7	115581661	.	A	<INS:ME:LINE1>	.	PASS	SVTYPE=INS:ME:LINE1;END=115581670;SVLEN=1794;AN=20618;AC=1;AF=4.850130953535746e-05;N_HET=1;N_HOMALT=0;N_HOMREF=10308;FREQ_HET=9.700261907071492e-05;FREQ_HOMALT=0.0;FREQ_HOMREF=0.9999029973809292;N_BI_GENOS=10309;Mongolian_AN=454;Mongolian_AC=1;Mongolian_
chr7	115638229	.	T	<INS:ME:LINE1>	.	PASS	SVTYPE=INS:ME:LINE1;END=115638242;SVLEN=3799;AN=20618;AC=2;AF=9.700261907071492e-05;N_HET=0;N_HOMALT=1;N_HOMREF=10308;FREQ_HET=0.0;FREQ_HOMALT=9.700261907071492e-05;FREQ_HOMREF=0.9999029973809292;N_BI_GENOS=10309;Miao_AN=482;Miao_AC=2;Miao_AF=0.0041493775
chr7	115904400	.	A	<INS:ME:LINE1>	.	PASS	SVTYPE=INS:ME:LINE1;END=115904413;SVLEN=1457;AN=20618;AC=12;AF=0.0005820157144242894;N_HET=12;N_HOMALT=0;N_HOMREF=10297;FREQ_HET=0.0011640314288485789;FREQ_HOMALT=0.0;FREQ_HOMREF=0.9988359685711514;N_BI_GENOS=10309;Northwest_Han_AN=1296;Northwest_Han_AC=1
chr7	115945313	.	G	<INS:ME:LINE1>	.	PASS	SVTYPE=INS:ME:LINE1;END=115945324;SVLEN=505;AN=20618;AC=7;AF=0.0003395091667475022;N_HET=7;N_HOMALT=0;N_HOMREF=10302;FREQ_HET=0.0006790183334950044;FREQ_HOMALT=0.0;FREQ_HOMREF=0.999320981666505;N_BI_GENOS=10309;Northwest_Han_AN=1296;Northwest_Han_AC=1;Nor
chr7	116567935	.	C	<INS:ME:LINE1>	.	PASS	SVTYPE=INS:ME:LINE1;END=116567948;SVLEN=608;AN=20618;AC=8;AF=0.0003880104762828596;N_HET=8;N_HOMALT=0;N_HOMREF=10301;FREQ_HET=0.0007760209525657193;FREQ_HOMALT=0.0;FREQ_HOMREF=0.9992239790474343;N_BI_GENOS=10309;Lingnan_Han_AN=1888;Lingnan_Han_AC=1;Lingn
chr7	116630265	.	G	<INS:ME:LINE1>	.	PASS	SVTYPE=INS:ME:LINE1;END=116630285;SVLEN=6005;AN=20618;AC=2;AF=9.700261907071492e-05;N_HET=2;N_HOMALT=0;N_HOMREF=10307;FREQ_HET=0.00019400523814142983;FREQ_HOMALT=0.0;FREQ_HOMREF=0.9998059947618586;N_BI_GENOS=10309;Central_Han_AN=2128;Central_Han_AC=1;Cent
chr7	116677511	.	A	<INS:ME:LINE1>	.	PASS	SVTYPE=INS:ME:LINE1;END=116677525;SVLEN=6014;AN=20618;AC=1;AF=4.850130953535746e-05;N_HET=1;N_HOMALT=0;N_HOMREF=10308;FREQ_HET=9.700261907071492e-05;FREQ_HOMALT=0.0;FREQ_HOMREF=0.9999029973809292;N_BI_GENOS=10309;Central_Han_AN=2128;Central_Han_AC=1;Centr
chr7	116770673	.	T	<INS:ME:LINE1>	.	PASS	SVTYPE=INS:ME:LINE1;END=116770686;SVLEN=59;AN=20618;AC=1;AF=4.850130953535746e-05;N_HET=1;N_HOMALT=0;N_HOMREF=10308;FREQ_HET=9.700261907071492e-05;FREQ_HOMALT=0.0;FREQ_HOMREF=0.9999029973809292;N_BI_GENOS=10309;East_Han_AN=6554;East_Han_AC=1;East_Han_AF=0
chr7	117425268	.	T	<INS:ME:LINE1>	.	PASS	SVTYPE=INS:ME:LINE1;END=117425283;SVLEN=741;AN=20618;AC=2;AF=9.700261907071492e-05;N_HET=0;N_HOMALT=1;N_HOMREF=10308;FREQ_HET=0.0;FREQ_HOMALT=9.700261907071492e-05;FREQ_HOMREF=0.9999029973809292;N_BI_GENOS=10309;East_Han_AN=6554;East_Han_AC=2;East_Han_AF=
chr7	117430181	.	C	<INS:ME:LINE1>	.	PASS	SVTYPE=INS:ME:LINE1;END=117430194;SVLEN=604;AN=20618;AC=3;AF=0.00014550392860607236;N_HET=1;N_HOMALT=1;N_HOMREF=10307;FREQ_HET=9.700261907071492e-05;FREQ_HOMALT=9.700261907071492e-05;FREQ_HOMREF=0.9998059947618586;N_BI_GENOS=10309;Northwest_Han_AN=1296;No
chr7	117444932	.	T	<INS:ME:LINE1>	.	PASS	SVTYPE=INS:ME:LINE1;END=117444948;SVLEN=2626;AN=20618;AC=6;AF=0.0002910078572121447;N_HET=4;N_HOMALT=1;N_HOMREF=10304;FREQ_HET=0.0003880104762828596 6;FREQ_HOMALT=9.700261907071492e-05;FREQ_HOMREF=0.9995149869046465;N_BI_GENOS=10309;South_Han_AN=1496;South
chr7	117497671	.	G	<INS:ME:LINE1>	.	PASS	SVTYPE=INS:ME:LINE1;END=117497687;SVLEN=918;AN=20618;AC=1;AF=4.850130953535746e-05;N_HET=1;N_HOMALT=0;N_HOMREF=10308;FREQ_HET=9.700261907071492e-05;FREQ_HOMALT=0.0;FREQ_HOMREF=0.9999029973809292;N_BI_GENOS=10309;East_Han_AN=6554;East_Han_AC=1;East_Han_AF=
chr7	117569627	.	G	<INS:ME:LINE1>	.	PASS	SVTYPE=INS:ME:LINE1;END=117569636;SVLEN=1597;AN=20618;AC=1;AF=4.850130953535746e-05;N_HET=1;N_HOMALT=0;N_HOMREF=10308;FREQ_HET=9.700261907071492e-05;FREQ_HOMALT=0.0;FREQ_HOMREF=0.9999029973809292;N_BI_GENOS=10309;Northwest_Han_AN=1296;Northwest_Han_AC=1;N
chr7	117800872	.	T	<INS:ME:LINE1>	.	PASS	SVTYPE=INS:ME:LINE1;END=117800888;SVLEN=6000;AN=20618;AC=1;AF=4.850130953535746e-05;N_HET=1;N_HOMALT=0;N_HOMREF=10308;FREQ_HET=9.700261907071492e-05;FREQ_HOMALT=0.0;FREQ_HOMREF=0.9999029973809292;N_BI_GENOS=10309;North_Han_AN=2272;North_Han_AC=1;North_Han
chr7	117809252	.	T	<INS:ME:LINE1>	.	PASS	SVTYPE=INS:ME:LINE1;END=117809267;SVLEN=5998;AN=20618;AC=27;AF=0.0013095353574546513;N_HET=27;N_HOMALT=0;N_HOMREF=10282;FREQ_HET=0.0026190707149093026;FREQ_HOMALT=0.0;FREQ_HOMREF=0.9973809292850907;N_BI_GENOS=10309;South_Han_AN=1496;South_Han_AC=2;South_H
chr7	118351779	.	G	<INS:ME:LINE1>	.	PASS	SVTYPE=INS:ME:LINE1;END=118351798;SVLEN=266;AN=20618;AC=1;AF=4.850130953535746e-05;N_HET=1;N_HOMALT=0;N_HOMREF=10308;FREQ_HET=9.700261907071492e-05;FREQ_HOMALT=0.0;FREQ_HOMREF=0.9999029973809292;N_BI_GENOS=10309;East_Han_AN=6554;East_Han_AC=1;East_Han_AF=
chr7	118662478	.	G	<INS:ME:LINE1>	.	PASS	SVTYPE=INS:ME:LINE1;END=118662494;SVLEN=486;AN=20618;AC=1;AF=4.850130953535746e-05;N_HET=1;N_HOMALT=0;N_HOMREF=10308;FREQ_HET=9.700261907071492e-05;FREQ_HOMALT=0.0;FREQ_HOMREF=0.9999029973809292;N_BI_GENOS=10309;Lingnan_Han_AN=1888;Lingnan_Han_AC=1;Lingna
chr7	118691695	.	A	<INS:ME:LINE1>	.	PASS	SVTYPE=INS:ME:LINE1;END=118691708;SVLEN=6000;AN=20618;AC=1;AF=4.850130953535746e-05;N_HET=1;N_HOMALT=0;N_HOMREF=10308;FREQ_HET=9.700261907071492e-05;FREQ_HOMALT=0.0;FREQ_HOMREF=0.9999029973809292;N_BI_GENOS=10309;Central_Han_AN=2128;Central_Han_AC=1;Centr
chr7	118863242	.	A	<INS:ME:LINE1>	.	PASS	SVTYPE=INS:ME:LINE1;END=118863253;SVLEN=68;AN=20618;AC=5;AF=0.00024250654767678728;N_HET=5;N_HOMALT=0;N_HOMREF=10304;FREQ_HET=0.00048501309535357455;FREQ_HOMALT=0.0;FREQ_HOMREF=0.9995149869046465;N_BI_GENOS=10309;Lingnan_Han_AN=1888;Lingnan_Han_AC=1;Lingn
chr7	118935366	.	T	<INS:ME:LINE1>	.	PASS	SVTYPE=INS:ME:LINE1;END=118935381;SVLEN=536;AN=20618;AC=2;AF=9.700261907071492e-05;N_HET=2;N_HOMALT=0;N_HOMREF=10307;FREQ_HET=0.00019400523814142983;FREQ_HOMALT=0.0;FREQ_HOMREF=0.9998059947618586;N_BI_GENOS=10309;South_Han_AN=1496;South_Han_AC=1;South_Han
chr7	119353428	.	T	<INS:ME:LINE1>	.	PASS	SVTYPE=INS:ME:LINE1;END=119353442;SVLEN=2210;AN=20618;AC=6;AF=0.0002910078572121447;N_HET=4;N_HOMALT=1;N_HOMREF=10304;FREQ_HET=0.0003880104762828596 6;FREQ_HOMALT=9.700261907071492e-05;FREQ_HOMREF=0.9995149869046465;N_BI_GENOS=10309;South_Han_AN=1496;South
chr7	119419804	.	G	<INS:ME:LINE1>	.	PASS	SVTYPE=INS:ME:LINE1;END=119419814;SVLEN=5995;AN=20618;AC=1;AF=4.850130953535746e-05;N_HET=1;N_HOMALT=0;N_HOMREF=10308;FREQ_HET=9.700261907071492e-05;FREQ_HOMALT=0.0;FREQ_HOMREF=0.9999029973809292;N_BI_GENOS=10309;East_Han_AN=6554;East_Han_AC=1;East_Han_AF
chr7	119449327	.	A	<INS:ME:LINE1>	.	PASS	SVTYPE=INS:ME:LINE1;END=119449344;SVLEN=667;AN=20618;AC=3;AF=0.00014550392860607236;N_HET=3;N_HOMALT=0;N_HOMREF=10306;FREQ_HET=0.0002910078572121447;FREQ_HOMALT=0.0;FREQ_HOMREF=0.9997089921427879;N_BI_GENOS=10309;South_Han_AN=1496;South_Han_AC=1;South_Han
chr7	119603903	.	A	<INS:ME:LINE1>	.	PASS	SVTYPE=INS:ME:LINE1;END=119603903;SVLEN=413;AN=20618;AC=1;AF=4.850130953535746e-05;N_HET=1;N_HOMALT=0;N_HOMREF=10308;FREQ_HET=9.700261907071492e-05;FREQ_HOMALT=0.0;FREQ_HOMREF=0.9999029973809292;N_BI_GENOS=10309;East_Han_AN=6554;East_Han_AC=1;East_Han_AF=
chr7	119806506	.	C	<INS:ME:LINE1>	.	PASS	SVTYPE=INS:ME:LINE1;END=119806507;SVLEN=46;AN=20618;AC=289;AF=0.014016878455718305;N_HET=237;N_HOMALT=26;N_HOMREF=10046;FREQ_HET=0.022989620719759435;FREQ_HOMALT=0.0025220680958385876;FREQ_HOMREF=0.9744883111844019;N_BI_GENOS=10309;South_Han_AN=1496;South
chr7	119987953	.	A	<INS:ME:LINE1>	.	PASS	SVTYPE=INS:ME:LINE1;END=119987994;SVLEN=1440;AN=20618;AC=1;AF=4.850130953535746e-05;N_HET=1;N_HOMALT=0;N_HOMREF=10308;FREQ_HET=9.700261907071492e-05;FREQ_HOMALT=0.0;FREQ_HOMREF=0.9999029973809292;N_BI_GENOS=10309;East_Han_AN=6554;East_Han_AC=1;East_Han_AF
chr7	119988307	.	C	<INS:ME:LINE1>	.	PASS	SVTYPE=INS:ME:LINE1;END=119988335;SVLEN=1168;AN=20618;AC=1;AF=4.850130953535746e-05;N_HET=1;N_HOMALT=0;N_HOMREF=10308;FREQ_HET=9.700261907071492e-05;FREQ_HOMALT=0.0;FREQ_HOMREF=0.9999029973809292;N_BI_GENOS=10309;East_Han_AN=6554;East_Han_AC=1;East_Han_AF
chr7	120338356	.	T	<INS:ME:LINE1>	.	PASS	SVTYPE=INS:ME:LINE1;END=120338358;SVLEN=487;AN=20618;AC=10;AF=0.0004850130953535357455;N_HET=10;N_HOMALT=0;N_HOMREF=10299;FREQ_HET=0.0009700261907071491;FREQ_HOMALT=0.0;FREQ_HOMREF=0.9990299738092928;N_BI_GENOS=10309;South_Han_AN=1496;South_Han_AC=1;South_H
chr7	120633039	.	T	<INS:ME:LINE1>	.	PASS	SVTYPE=INS:ME:LINE1;END=120633039;SVLEN=1461;AN=20618;AC=1;AF=4.850130953535746e-05;N_HET=1;N_HOMALT=0;N_HOMREF=10308;FREQ_HET=9.700261907071492e-05;FREQ_HOMALT=0.0;FREQ_HOMREF=0.9999029973809292;N_BI_GENOS=10309;Lingnan_Han_AN=1888;Lingnan_Han_AC=1;Lingn
chr7	120646486	.	T	<INS:ME:LINE1>	.	PASS	SVTYPE=INS:ME:LINE1;END=120646486;SVLEN=77;AN=20618;AC=1;AF=4.850130953535746e-05;N_HET=1;N_HOMALT=0;N_HOMREF=10308;FREQ_HET=9.700261907071492e-05;FREQ_HOMALT=0.0;FREQ_HOMREF=0.9999029973809292;N_BI_GENOS=10309;East_Han_AN=6554;East_Han_AC=1;East_Han_AF=0
chr7	120704455	.	A	<INS:ME:LINE1>	.	PASS	SVTYPE=INS:ME:LINE1;END=120704470;SVLEN=229;AN=20618;AC=9;AF=0.0004365117858182171;N_HET=7;N_HOMALT=1;N_HOMREF=10301;FREQ_HET=0.0006790183334950044;FREQ_HOMALT=9.700261907071492e-05;FREQ_HOMREF=0.9992239790474343;N_BI_GENOS=10309;South_Han_AN=1496;South_H
chr7	120744826	.	A	<INS:ME:LINE1>	.	PASS	SVTYPE=INS:ME:LINE1;END=120744844;SVLEN=887;AN=20618;AC=1;AF=4.850130953535746e-05;N_HET=1;N_HOMALT=0;N_HOMREF=10308;FREQ_HET=9.700261907071492e-05;FREQ_HOMALT=0.0;FREQ_HOMREF=0.9999029973809292;N_BI_GENOS=10309;Lingnan_Han_AN=1888;Lingnan_Han_AC=1;Lingna
chr7	121124122	.	T	<INS:ME:LINE1>	.	PASS	SVTYPE=INS:ME:LINE1;END=121124123;SVLEN=86;AN=20618;AC=3;AF=0.00014550392860607236;N_HET=3;N_HOMALT=0;N_HOMREF=10306;FREQ_HET=0.0002910078572121447;FREQ_HOMALT=0.0;FREQ_HOMREF=0.9997089921427879;N_BI_GENOS=10309;North_Han_AN=2272;North_Han_AC=2;North_Han_
chr7	121652294	.	T	<INS:ME:LINE1>	.	PASS	SVTYPE=INS:ME:LINE1;END=121652349;SVLEN=2582;AN=20618;AC=1;AF=4.850130953535746e-05;N_HET=1;N_HOMALT=0;N_HOMREF=10308;FREQ_HET=9.700261907071492e-05;FREQ_HOMALT=0.0;FREQ_HOMREF=0.9999029973809292;N_BI_GENOS=10309;East_Han_AN=6554;East_Han_AC=1;East_Han_AF
chr7	122452122	.	G	<INS:ME:LINE1>	.	PASS	SVTYPE=INS:ME:LINE1;END=122452135;SVLEN=5985;AN=20618;AC=1;AF=4.850130953535746e-05;N_HET=1;N_HOMALT=0;N_HOMREF=10308;FREQ_HET=9.700261907071492e-05;FREQ_HOMALT=0.0;FREQ_HOMREF=0.9999029973809292;N_BI_GENOS=10309;Central_Han_AN=2128;Central_Han_AC=1;Centr
chr7	122484490	.	A	<INS:ME:LINE1>	.	PASS	SVTYPE=INS:ME:LINE1;END=122484504;SVLEN=517;AN=20618;AC=578;AF=0.02803375691143661;N_HET=536;N_HOMALT=21;N_HOMREF=9752;FREQ_HET=0.05199340382190319;FREQ_HOMALT=0.002037055000485013;FREQ_HOMREF=0.9459695411776118;N_BI_GENOS=10309;South_Han_AN=1496;South_Ha
chr7	122600766	.	A	<INS:ME:LINE1>	.	PASS	SVTYPE=INS:ME:LINE1;END=122600779;SVLEN=1900;AN=20618;AC=4;AF=0.00019400523814142983;N_HET=4;N_HOMALT=0;N_HOMREF=10305;FREQ_HET=0.0003880104762828596 6;FREQ_HOMALT=0.0;FREQ_HOMREF=0.9996119895237171;N_BI_GENOS=10309;South_Han_AN=1496;South_Han_AC=3;South_H
chr7	122632563	.	A	<INS:ME:LINE1>	.	PASS	SVTYPE=INS:ME:LINE1;END=122632563;SVLEN=646;AN=20618;AC=2;AF=9.700261907071492e-05;N_HET=2;N_HOMALT=0;N_HOMREF=10307;FREQ_HET=0.00019400523814142983;FREQ_HOMALT=0.0;FREQ_HOMREF=0.9998059947618586;N_BI_GENOS=10309;East_Han_AN=6554;East_Han_AC=2;East_Han_AF
chr7	122796605	.	T	<INS:ME:LINE1>	.	PASS	SVTYPE=INS:ME:LINE1;END=122796610;SVLEN=1390;AN=20618;AC=1;AF=4.850130953535746e-05;N_HET=1;N_HOMALT=0;N_HOMREF=10308;FREQ_HET=9.700261907071492e-05;FREQ_HOMALT=0.0;FREQ_HOMREF=0.9999029973809292;N_BI_GENOS=10309;East_Han_AN=6554;East_Han_AC=1;East_Han_AF
chr7	123111992	.	A	<INS:ME:LINE1>	.	PASS	SVTYPE=INS:ME:LINE1;END=123112010;SVLEN=296;AN=20618;AC=1;AF=4.850130953535746e-05;N_HET=1;N_HOMALT=0;N_HOMREF=10308;FREQ_HET=9.700261907071492e-05;FREQ_HOMALT=0.0;FREQ_HOMREF=0.9999029973809292;N_BI_GENOS=10309;Miao_AN=482;Miao_AC=1;Miao_AF=0.00207468879
chr7	123343073	.	A	<INS:ME:LINE1>	.	PASS	SVTYPE=INS:ME:LINE1;END=123343088;SVLEN=2317;AN=20618;AC=29;AF=0.0014065379765253662;N_HET=23;N_HOMALT=3;N_HOMREF=10283;FREQ_HET=0.002231060238626443;FREQ_HOMALT=0.0002910078572121447;FREQ_HOMREF=0.9974779319041615;N_BI_GENOS=10309;South_Han_AN=1496;South
chr7	123379802	.	T	<INS:ME:LINE1>	.	PASS	SVTYPE=INS:ME:LINE1;END=123379816;SVLEN=1070;AN=20618;AC=1;AF=4.850130953535746e-05;N_HET=1;N_HOMALT=0;N_HOMREF=10308;FREQ_HET=9.700261907071492e-05;FREQ_HOMALT=0.0;FREQ_HOMREF=0.9999029973809292;N_BI_GENOS=10309;Tibetan_AN=414;Tibetan_AC=1;Tibetan_AF=0.0
chr7	123392598	.	A	<INS:ME:LINE1>	.	PASS	SVTYPE=INS:ME:LINE1;END=123392620;SVLEN=5998;AN=20618;AC=3;AF=0.00014550392860607236;N_HET=3;N_HOMALT=0;N_HOMREF=10306;FREQ_HET=0.0002910078572121447;FREQ_HOMALT=0.0;FREQ_HOMREF=0.9997089921427879;N_BI_GENOS=10309;Lingnan_Han_AN=1888;Lingnan_Han_AC=3;Ling
chr7	123473571	.	T	<INS:ME:LINE1>	.	PASS	SVTYPE=INS:ME:LINE1;END=123473579;SVLEN=589;AN=20618;AC=2;AF=9.700261907071492e-05;N_HET=2;N_HOMALT=0;N_HOMREF=10307;FREQ_HET=0.00019400523814142983;FREQ_HOMALT=0.0;FREQ_HOMREF=0.9998059947618586;N_BI_GENOS=10309;Northwest_Han_AN=1296;Northwest_Han_AC=1;N
chr7	124107888	.	A	<INS:ME:LINE1>	.	PASS	SVTYPE=INS:ME:LINE1;END=124107902;SVLEN=154;AN=20618;AC=1;AF=4.850130953535746e-05;N_HET=1;N_HOMALT=0;N_HOMREF=10308;FREQ_HET=9.700261907071492e-05;FREQ_HOMALT=0.0;FREQ_HOMREF=0.9999029973809292;N_BI_GENOS=10309;Southeast_Han_AN=1908;Southeast_Han_AC=1;So
chr7	124165537	.	T	<INS:ME:LINE1>	.	PASS	SVTYPE=INS:ME:LINE1;END=124165554;SVLEN=2838;AN=20618;AC=1;AF=4.850130953535746e-05;N_HET=1;N_HOMALT=0;N_HOMREF=10308;FREQ_HET=9.700261907071492e-05;FREQ_HOMALT=0.0;FREQ_HOMREF=0.9999029973809292;N_BI_GENOS=10309;East_Han_AN=6554;East_Han_AC=1;East_Han_AF
chr7	124212697	.	A	<INS:ME:LINE1>	.	PASS	SVTYPE=INS:ME:LINE1;END=124212708;SVLEN=86;AN=20618;AC=2;AF=9.700261907071492e-05;N_HET=0;N_HOMALT=1;N_HOMREF=10308;FREQ_HET=0.0;FREQ_HOMALT=9.700261907071492e-05;FREQ_HOMREF=0.9999029973809292;N_BI_GENOS=10309;North_Han_AN=2272;North_Han_AC=2;North_Han_A
chr7	124225414	.	A	<INS:ME:LINE1>	.	PASS	SVTYPE=INS:ME:LINE1;END=124225432;SVLEN=1555;AN=20618;AC=1;AF=4.850130953535746e-05;N_HET=1;N_HOMALT=0;N_HOMREF=10308;FREQ_HET=9.700261907071492e-05;FREQ_HOMALT=0.0;FREQ_HOMREF=0.9999029973809292;N_BI_GENOS=10309;Northwest_Han_AN=1296;Northwest_Han_AC=1;N
chr7	124227537	.	A	<INS:ME:LINE1>	.	PASS	SVTYPE=INS:ME:LINE1;END=124227550;SVLEN=5999;AN=20618;AC=1;AF=4.850130953535746e-05;N_HET=1;N_HOMALT=0;N_HOMREF=10308;FREQ_HET=9.700261907071492e-05;FREQ_HOMALT=0.0;FREQ_HOMREF=0.9999029973809292;N_BI_GENOS=10309;Central_Han_AN=2128;Central_Han_AC=1;Centr
chr7	124336526	.	A	<INS:ME:LINE1>	.	PASS	SVTYPE=INS:ME:LINE1;END=124336541;SVLEN=102;AN=20618;AC=1;AF=4.850130953535746e-05;N_HET=1;N_HOMALT=0;N_HOMREF=10308;FREQ_HET=9.700261907071492e-05;FREQ_HOMALT=0.0;FREQ_HOMREF=0.9999029973809292;N_BI_GENOS=10309;Southeast_Han_AN=1908;Southeast_Han_AC=1;So
chr7	124345644	.	T	<INS:ME:LINE1>	.	PASS	SVTYPE=INS:ME:LINE1;END=124345645;SVLEN=215;AN=20618;AC=61;AF=0.0029585798816568047;N_HET=53;N_HOMALT=4;N_HOMREF=10252;FREQ_HET=0.005141138810747 89;FREQ_HOMALT=0.0003880104762828596 6;FREQ_HOMREF=0.9944708507129693;N_BI_GENOS=10309;South_Han_AN=1496;South_
chr7	124543184	.	C	<INS:ME:LINE1>	.	PASS	SVTYPE=INS:ME:LINE1;END=124543199;SVLEN=1662;AN=20618;AC=1;AF=4.850130953535746e-05;N_HET=1;N_HOMALT=0;N_HOMREF=10308;FREQ_HET=9.700261907071492e-05;FREQ_HOMALT=0.0;FREQ_HOMREF=0.9999029973809292;N_BI_GENOS=10309;Central_Han_AN=2128;Central_Han_AC=1;Centr
chr7	124574349	.	C	<INS:ME:LINE1>	.	PASS	SVTYPE=INS:ME:LINE1;END=124574364;SVLEN=5997;AN=20618;AC=1;AF=4.850130953535746e-05;N_HET=1;N_HOMALT=0;N_HOMREF=10308;FREQ_HET=9.700261907071492e-05;FREQ_HOMALT=0.0;FREQ_HOMREF=0.9999029973809292;N_BI_GENOS=10309;North_Han_AN=2272;North_Han_AC=1;North_Han
chr7	124811263	.	T	<INS:ME:LINE1>	.	PASS	SVTYPE=INS:ME:LINE1;END=124811280;SVLEN=3045;AN=20618;AC=14;AF=0.0006790183334950044;N_HET=14;N_HOMALT=0;N_HOMREF=10295;FREQ_HET=0.0013580366669900088;FREQ_HOMALT=0.0;FREQ_HOMREF=0.99864196333301;N_BI_GENOS=10309;South_Han_AN=1496;South_Han_AC=2;South_Han
chr7	125376282	.	G	<INS:ME:LINE1>	.	PASS	SVTYPE=INS:ME:LINE1;END=125376303;SVLEN=4562;AN=20618;AC=1;AF=4.850130953535746e-05;N_HET=1;N_HOMALT=0;N_HOMREF=10308;FREQ_HET=9.700261907071492e-05;FREQ_HOMALT=0.0;FREQ_HOMREF=0.9999029973809292;N_BI_GENOS=10309;Southeast_Han_AN=1908;Southeast_Han_AC=1;S
chr7	125774389	.	G	<INS:ME:LINE1>	.	PASS	SVTYPE=INS:ME:LINE1;END=125774404;SVLEN=851;AN=20618;AC=1;AF=4.850130953535746e-05;N_HET=1;N_HOMALT=0;N_HOMREF=10308;FREQ_HET=9.700261907071492e-05;FREQ_HOMALT=0.0;FREQ_HOMREF=0.9999029973809292;N_BI_GENOS=10309;Lingnan_Han_AN=1888;Lingnan_Han_AC=1;Lingna
chr7	126233815	.	C	<INS:ME:LINE1>	.	PASS	SVTYPE=INS:ME:LINE1;END=126233815;SVLEN=7;AN=20618;AC=1;AF=4.850130953535746e-05;N_HET=1;N_HOMALT=0;N_HOMREF=10308;FREQ_HET=9.700261907071492e-05;FREQ_HOMALT=0.0;FREQ_HOMREF=0.9999029973809292;N_BI_GENOS=10309;East_Han_AN=6554;East_Han_AC=1;East_Han_AF=0.
chr7	126536217	.	T	<INS:ME:LINE1>	.	PASS	SVTYPE=INS:ME:LINE1;END=126536232;SVLEN=3545;AN=20618;AC=1;AF=4.850130953535746e-05;N_HET=1;N_HOMALT=0;N_HOMREF=10308;FREQ_HET=9.700261907071492e-05;FREQ_HOMALT=0.0;FREQ_HOMREF=0.9999029973809292;N_BI_GENOS=10309;North_Han_AN=2272;North_Han_AC=1;North_Han
chr7	126677490	.	T	<INS:ME:LINE1>	.	PASS	SVTYPE=INS:ME:LINE1;END=126677490;SVLEN=474;AN=20618;AC=1;AF=4.850130953535746e-05;N_HET=1;N_HOMALT=0;N_HOMREF=10308;FREQ_HET=9.700261907071492e-05;FREQ_HOMALT=0.0;FREQ_HOMREF=0.9999029973809292;N_BI_GENOS=10309;East_Han_AN=6554;East_Han_AC=1;East_Han_AF=
chr7	126781422	.	A	<INS:ME:LINE1>	.	PASS	SVTYPE=INS:ME:LINE1;END=126781431;SVLEN=1126;AN=20618;AC=1;AF=4.850130953535746e-05;N_HET=1;N_HOMALT=0;N_HOMREF=10308;FREQ_HET=9.700261907071492e-05;FREQ_HOMALT=0.0;FREQ_HOMREF=0.9999029973809292;N_BI_GENOS=10309;Hui_AN=444;Hui_AC=1;Hui_AF=0.0022522522522
chr7	127084339	.	A	<INS:ME:LINE1>	.	PASS	SVTYPE=INS:ME:LINE1;END=127084356;SVLEN=2430;AN=20618;AC=3;AF=0.00014550392860607236;N_HET=3;N_HOMALT=0;N_HOMREF=10306;FREQ_HET=0.0002910078572121447;FREQ_HOMALT=0.0;FREQ_HOMREF=0.9997089921427879;N_BI_GENOS=10309;Northwest_Han_AN=1296;Northwest_Han_AC=1;
chr7	127086489	.	G	<INS:ME:LINE1>	.	PASS	SVTYPE=INS:ME:LINE1;END=127086503;SVLEN=5998;AN=20618;AC=8;AF=0.0003880104762828596 6;N_HET=8;N_HOMALT=0;N_HOMREF=10301;FREQ_HET=0.0007760209525657193;FREQ_HOMALT=0.0;FREQ_HOMREF=0.9992239790474343;N_BI_GENOS=10309;South_Han_AN=1496;South_Han_AC=2;South_Ha
chr7	127110616	.	T	<INS:ME:LINE1>	.	PASS	SVTYPE=INS:ME:LINE1;END=127110632;SVLEN=894;AN=20618;AC=4;AF=0.00019400523814142983;N_HET=4;N_HOMALT=0;N_HOMREF=10305;FREQ_HET=0.0003880104762828596 6;FREQ_HOMALT=0.0;FREQ_HOMREF=0.9996119895237171;N_BI_GENOS=10309;South_Han_AN=1496;South_Han_AC=1;South_Ha
chr7	127292771	.	A	<INS:ME:LINE1>	.	PASS	SVTYPE=INS:ME:LINE1;END=127292787;SVLEN=6027;AN=20618;AC=4;AF=0.00019400523814142983;N_HET=4;N_HOMALT=0;N_HOMREF=10305;FREQ_HET=0.0003880104762828596 6;FREQ_HOMALT=0.0;FREQ_HOMREF=0.9996119895237171;N_BI_GENOS=10309;North_Han_AN=2272;North_Han_AC=1;North_H
chr7	127328046	.	A	<INS:ME:LINE1>	.	PASS	SVTYPE=INS:ME:LINE1;END=127328052;SVLEN=684;AN=20618;AC=1;AF=4.850130953535746e-05;N_HET=1;N_HOMALT=0;N_HOMREF=10308;FREQ_HET=9.700261907071492e-05;FREQ_HOMALT=0.0;FREQ_HOMREF=0.9999029973809292;N_BI_GENOS=10309;East_Han_AN=6554;East_Han_AC=1;East_Han_AF=
chr7	127394276	.	G	<INS:ME:LINE1>	.	PASS	SVTYPE=INS:ME:LINE1;END=127394292;SVLEN=5947;AN=20618;AC=1;AF=4.850130953535746e-05;N_HET=1;N_HOMALT=0;N_HOMREF=10308;FREQ_HET=9.700261907071492e-05;FREQ_HOMALT=0.0;FREQ_HOMREF=0.9999029973809292;N_BI_GENOS=10309;Northwest_Han_AN=1296;Northwest_Han_AC=1;N
chr7	127482003	.	A	<INS:ME:LINE1>	.	PASS	SVTYPE=INS:ME:LINE1;END=127482011;SVLEN=6000;AN=20618;AC=4;AF=0.00019400523814142983;N_HET=4;N_HOMALT=0;N_HOMREF=10305;FREQ_HET=0.0003880104762828596 6;FREQ_HOMALT=0.0;FREQ_HOMREF=0.9996119895237171;N_BI_GENOS=10309;South_Han_AN=1496;South_Han_AC=1;South_H
chr7	127867366	.	A	<INS:ME:LINE1>	.	PASS	SVTYPE=INS:ME:LINE1;END=127867382;SVLEN=5801;AN=20618;AC=2;AF=9.700261907071492e-05;N_HET=2;N_HOMALT=0;N_HOMREF=10307;FREQ_HET=0.00019400523814142983;FREQ_HOMALT=0.0;FREQ_HOMREF=0.9998059947618586;N_BI_GENOS=10309;Lingnan_Han_AN=1888;Lingnan_Han_AC=2;Ling
chr7	127921561	.	T	<INS:ME:LINE1>	.	PASS	SVTYPE=INS:ME:LINE1;END=127921575;SVLEN=197;AN=20618;AC=4;AF=0.00019400523814142983;N_HET=4;N_HOMALT=0;N_HOMREF=10305;FREQ_HET=0.0003880104762828596 6;FREQ_HOMALT=0.0;FREQ_HOMREF=0.9996119895237171;N_BI_GENOS=10309;Lingnan_Han_AN=1888;Lingnan_Han_AC=2;Ling
chr7	128220397	.	T	<INS:ME:LINE1>	.	PASS	SVTYPE=INS:ME:LINE1;END=128220415;SVLEN=195;AN=20618;AC=1;AF=4.850130953535746e-05;N_HET=1;N_HOMALT=0;N_HOMREF=10308;FREQ_HET=9.700261907071492e-05;FREQ_HOMALT=0.0;FREQ_HOMREF=0.9999029973809292;N_BI_GENOS=10309;Lingnan_Han_AN=1888;Lingnan_Han_AC=1;Lingna
chr7	128290374	.	T	<INS:ME:LINE1>	.	PASS	SVTYPE=INS:ME:LINE1;END=128290387;SVLEN=1989;AN=20618;AC=1;AF=4.850130953535746e-05;N_HET=1;N_HOMALT=0;N_HOMREF=10308;FREQ_HET=9.700261907071492e-05;FREQ_HOMALT=0.0;FREQ_HOMREF=0.9999029973809292;N_BI_GENOS=10309;East_Han_AN=6554;East_Han_AC=1;East_Han_AF
chr7	128290813	.	C	<INS:ME:LINE1>	.	PASS	SVTYPE=INS:ME:LINE1;END=128290817;SVLEN=3436;AN=20618;AC=3;AF=0.00014550392860607236;N_HET=3;N_HOMALT=0;N_HOMREF=10306;FREQ_HET=0.0002910078572121447;FREQ_HOMALT=0.0;FREQ_HOMREF=0.9997089921427879;N_BI_GENOS=10309;North_Han_AN=2272;North_Han_AC=1;North_Ha
chr7	129762279	.	A	<INS:ME:LINE1>	.	PASS	SVTYPE=INS:ME:LINE1;END=129762298;SVLEN=284;AN=20618;AC=1;AF=4.850130953535746e-05;N_HET=1;N_HOMALT=0;N_HOMREF=10308;FREQ_HET=9.700261907071492e-05;FREQ_HOMALT=0.0;FREQ_HOMREF=0.9999029973809292;N_BI_GENOS=10309;Southeast_Han_AN=1908;Southeast_Han_AC=1;So
chr7	129861222	.	A	<INS:ME:LINE1>	.	PASS	SVTYPE=INS:ME:LINE1;END=129861222;SVLEN=1158;AN=20618;AC=2;AF=9.700261907071492e-05;N_HET=2;N_HOMALT=0;N_HOMREF=10307;FREQ_HET=0.00019400523814142983;FREQ_HOMALT=0.0;FREQ_HOMREF=0.9998059947618586;N_BI_GENOS=10309;East_Han_AN=6554;East_Han_AC=2;East_Han_A
chr7	130403614	.	A	<INS:ME:LINE1>	.	PASS	SVTYPE=INS:ME:LINE1;END=130403627;SVLEN=5993;AN=20618;AC=7;AF=0.0003395091667475022;N_HET=7;N_HOMALT=0;N_HOMREF=10302;FREQ_HET=0.0006790183334950044;FREQ_HOMALT=0.0;FREQ_HOMREF=0.999320981666505;N_BI_GENOS=10309;South_Han_AN=1496;South_Han_AC=1;South_Han_
chr7	130628442	.	A	<INS:ME:LINE1>	.	PASS	SVTYPE=INS:ME:LINE1;END=130628453;SVLEN=1016;AN=20618;AC=14;AF=0.0006790183334950044;N_HET=14;N_HOMALT=0;N_HOMREF=10295;FREQ_HET=0.0013580366669900088;FREQ_HOMALT=0.0;FREQ_HOMREF=0.99864196333301;N_BI_GENOS=10309;Lingnan_Han_AN=1888;Lingnan_Han_AC=1;Lingn
chr7	130861016	.	G	<INS:ME:LINE1>	.	PASS	SVTYPE=INS:ME:LINE1;END=130861030;SVLEN=6003;AN=20618;AC=1;AF=4.850130953535746e-05;N_HET=1;N_HOMALT=0;N_HOMREF=10308;FREQ_HET=9.700261907071492e-05;FREQ_HOMALT=0.0;FREQ_HOMREF=0.9999029973809292;N_BI_GENOS=10309;East_Han_AN=6554;East_Han_AC=1;East_Han_AF
chr7	130875789	.	A	<INS:ME:LINE1>	.	PASS	SVTYPE=INS:ME:LINE1;END=130875802;SVLEN=54;AN=20618;AC=2;AF=9.700261907071492e-05;N_HET=2;N_HOMALT=0;N_HOMREF=10307;FREQ_HET=0.00019400523814142983;FREQ_HOMALT=0.0;FREQ_HOMREF=0.9998059947618586;N_BI_GENOS=10309;North_Han_AN=2272;North_Han_AC=1;North_Han_
chr7	131182661	.	A	<INS:ME:LINE1>	.	PASS	SVTYPE=INS:ME:LINE1;END=131182673;SVLEN=6062;AN=20618;AC=1;AF=4.850130953535746e-05;N_HET=1;N_HOMALT=0;N_HOMREF=10308;FREQ_HET=9.700261907071492e-05;FREQ_HOMALT=0.0;FREQ_HOMREF=0.9999029973809292;N_BI_GENOS=10309;Mongolian_AN=454;Mongolian_AC=1;Mongolian_
chr7	131524379	.	A	<INS:ME:LINE1>	.	PASS	SVTYPE=INS:ME:LINE1;END=131524379;SVLEN=0;AN=20618;AC=3;AF=0.00014550392860607236;N_HET=1;N_HOMALT=1;N_HOMREF=10307;FREQ_HET=9.700261907071492e-05;FREQ_HOMALT=9.700261907071492e-05;FREQ_HOMREF=0.9998059947618586;N_BI_GENOS=10309;East_Han_AN=6554;East_Han_
chr7	131707759	.	T	<INS:ME:LINE1>	.	PASS	SVTYPE=INS:ME:LINE1;END=131707772;SVLEN=957;AN=20618;AC=1;AF=4.850130953535746e-05;N_HET=1;N_HOMALT=0;N_HOMREF=10308;FREQ_HET=9.700261907071492e-05;FREQ_HOMALT=0.0;FREQ_HOMREF=0.9999029973809292;N_BI_GENOS=10309;Southeast_Han_AN=1908;Southeast_Han_AC=1;So
chr7	132059733	.	A	<INS:ME:LINE1>	.	PASS	SVTYPE=INS:ME:LINE1;END=132059733;SVLEN=1130;AN=20618;AC=1;AF=4.850130953535746e-05;N_HET=1;N_HOMALT=0;N_HOMREF=10308;FREQ_HET=9.700261907071492e-05;FREQ_HOMALT=0.0;FREQ_HOMREF=0.9999029973809292;N_BI_GENOS=10309;Northwest_Han_AN=1296;Northwest_Han_AC=1;N
[truncated: 11,639,294 more chars]
